# *De novo* Assembly and Characterization of *Cajanus scarabaeoides* (L.) Thouars Transcriptome by Paired-End Sequencing

**DOI:** 10.3389/fmolb.2017.00048

**Published:** 2017-07-12

**Authors:** Deepti Nigam, Swati Saxena, G. Ramakrishna, Archana Singh, N. K. Singh, Kishor Gaikwad

**Affiliations:** ^1^Indian Council of Agricultural Research-National Research Centre on Plant Biotechnology New Delhi, India; ^2^Division of Biochemistry, Indian Council of Agricultural Research-Indian Agricultural Research Institute New Delhi, India

**Keywords:** *Cajanus scarabaeoides* (L.) Thouars, cultivated wild relatives (CWR), *de novo* assembly, illumina sequencing, SSR's

## Abstract

Pigeonpea [*Cajanus cajan* (L.) Millsp.] is a heat and drought resilient legume crop grown mostly in Asia and Africa. Pigeonpea is affected by various biotic (diseases and insect pests) and abiotic stresses (salinity and water logging) which limit the yield potential of this crop. However, resistance to all these constraints is not readily available in the cultivated genotypes and some of the wild relatives have been found to withstand these resistances. Thus, the utilization of crop wild relatives (CWR) in pigeonpea breeding has been effective in conferring resistance, quality and breeding efficiency traits to this crop. Bud and leaf tissue of *Cajanus scarabaeoides*, a wild relative of pigeon pea were used for transcriptome profiling. Approximately 30 million clean reads filtered from raw reads by removal of adaptors, ambiguous reads and low-quality reads (3.02 gigabase pairs) were generated by Illumina paired-end RNA-seq technology. All of these clean reads were pooled and assembled *de novo* into 1,17,007 transcripts using the Trinity. Finally, a total of 98,664 unigenes were derived with mean length of 396 bp and N50 values of 1393. The assembly produced significant mapping results (73.68%) in BLASTN searches of the *Glycine max* CDS sequence database (Ensembl). Further, uniprot database of Viridiplantae was used for unigene annotation; 81,799 of 98,664 (82.90%) unigenes were finally annotated with gene descriptions or conserved protein domains. Further, a total of 23,475 SSRs were identified in 27,321 unigenes. This data will provide useful information for mining of functionally important genes and SSR markers for pigeonpea improvement.

## Introduction

To keep pace with the growing human population and dietary requirements, food production must double in the next 25 years (McCouch et al., 2013). The intensifying food demand, climate change, soil degradation, water and land shortages are putting more pressure on the productivity of the current crop-production system (Kastner et al., 2012). In order to achieve gain in food availability, concentrated efforts to intensify food production and making it sustainable are required (Foley et al., 2011). Answers to meet these challenges lies in utilization of novel genetic diversity for production of crop varieties comprising attributes such as heat, cold and drought tolerance, disease and pest resistance (Esquinas-Alcázar, 2005). Domestication and concentration of human population has resulted in severe loss of crop genetic diversity (Olsenand and Gross, 2008). Interestingly, the wild relatives of the cultivated plant species known as Crop wild relatives (CWR) possess much broader spectrum of genetic variations in comparison to the cultivated species (Guarino and Lobell, 2011). The wild relatives have evolved in nature and show adaptation to extreme environmental changes (heat, cold, drought, salinity) and have the capability to withstand damage by insect pest and diseases (Dempewolf et al., 2014). However their utilization is hampered by limited availability of genomic resources.

*Cajanus scarabaeoides* (L.) Thouars. is most widely distributed wild species of pigeonpea, belongs to the secondary gene pool and is native to tropical and temperate regions of India, Sri Lanka, Australia, Africa, China etc. (Saxena, 2008). It is a creeper climber, the stem and seeds are very nutritive and can be used for feed and food purposes respectively and it is known to exhibit antibiosis as well as mechanical resistance to pod borers (Van der Maesen, 1985). It is highly resistant to *Helicoverpa armigera* (pod borer) due to presence of short non-glandular trichomes on the pods (Sharma et al., 2009). The flower initiation is known to be very early in *C. scarabaeoides* as compared to popular pigeonpea cultivars. This trait can be utilized in breeding programs to reduce harvest time to avoid drastic effects of changing climate. The somatic chromosome features of *C. scarabaeoides* are similar to the cultivated pigeonpea (Hari D Upadhyaya, 2006). It has many desirable attributes and is cross-compatible with cultivated pigeonpea, and interspecific gene transfer is possible through conventional hybridization. The phenomenon of heterosis has been effectively used for genetic improvement in pigeonpea as it shows considerable extent of natural outcrossing. A number of cytoplasmic male sterile (CMS) systems are available in pigeonpea but CMS lines derived from *C. scarabaeoides* (A2 cytoplasm) has been widely utilized to develop promising commercial hybrids (Choudhary and Singh, 2015). Unfortunately, very narrow genomic information is available for *C. scarabaeoides*.

Keeping in mind the decisive importance of this species, we performed Illumina paired-end sequencing to generate the bud and leaf transcriptome of *C. scarabaeoides*. This is the primary report of transcriptome sequencing and *de novo* assembly of *C. scarabaeoides* transcriptome. The transcriptome database will provide a valuable resource for gene discovery and marker development linked with superior traits.

## Materials and methods

### Plant material and RNA isolation

Seeds of *C. scarabaeoides* (Accession N0- ICPW87) were obtained from the International Crops Research Institute for Semi-Arid Tropics (ICRISAT), Patancheru, India. The seeds were sown in pots containing 1 kg of soil with 34°C temperature at day and 25°C at night with relative humidity approximately 68% and a photoperiod of 16/8 h. The plants were maintained in the experimental green house at NRCPB, New Delhi, India. Buds and leaves of were used as plant material in the present study. Fresh leaves and buds were harvested and immediately frozen in liquid nitrogen and stored in −80°C until further use. Total RNA from the buds and leaves was extracted using Spectrum Plant Total RNA Kit (Sigma) following the manufacturer's protocol. RNA was quality was checked by Agilent 2100 Bioanalyzer RNA Nanochip (Agilent Technologies) and quantity was checked by Nanodrop spectrophotometer (Thermo scientific). Total RNA at concentration of ≥500 ng/ul, OD 260/280 = 1.8~2.2, RNA Integrity Number (RIN) ≥ 7 were used for RNA library preparation.

### Library preparation and illumina sequencing

A Truseq RNA Sample Prep Kit (Illumina) was employed in poly-A based mRNA purification and cDNA library construction was carried out according to the manufacturer's instructions. 1 ug of total RNA sample of bud and leaf tissue were used for poly-A mRNA selection using streptavidin-coated magnetic beads. Two rounds of enrichment for poly-A mRNA was carried followed by mRNA fragmentation. cDNA was synthesized from enriched and fragmented RNA using reverse transcriptase (Super Script II). Double stranded Illumina libraries were obtained by 15 cycles of PCR amplification using Illumina PCR primers. The size and purity of the libraries were checked by Agilent 2100 Bioanalyzer. The cDNA libraries of average fragment length 360 bp, approximately were constructed. Finally, both the libraries were sequenced using paired end protocol on the Illumina MiSeq system. The raw sequence data generated in present study have been deposited at NCBI in the Short Read Archive (SRA) database under the accession number SRA548291 (experiment accession numbers SRX2661106 and SRX2661107 for bud and leaf tissue, respectively).

### *De novo* transcriptome assembly

The base quality of the raw paired end reads from bud (17,388,560) and leaf (13,146,615) respectively of 100 bp were assessed by FastQC v0.11.2 (Andrews, 2010). Raw sequenced reads were processed using Trimmomatic software version 0.36 (Bolger et al., 2014) for trimming and quality filtering. In this step, raw reads containing adapter sequence, reads with poly-N (≥10%) and low quality (sQ ≤ 5) were removed to obtain high quality *de novo* transcriptome sequence data. Simultaneously, Q20, Q30, GC-content and sequence duplication level of the reads were calculated and the downstream analyses was performed on the filtered clean data.

The high quality filtered reads for each library were then *de novo* assembled using Trinity software package version v2.4.0 (Grabherr et al., 2011), all parameters set to default. The assembly validation was performed using Bowtie2 aligner where the filtered reads were mapped back to the assembled transcripts. Further, unigenes were retrieved with the aid of CD-HIT-EST software (http://weizhongli-lab.org/cd-hit/) that clustered the transcripts with identity parameter of 95%. Unigenes were compared with the *Glycine max* coding sequence as well as uniprot databases of Viridiplantae by using blastx with a typical cut-off *E*-value of 1e^−5^ and similarity level > = 90% to search for homologs. Further, to calculate the abundance estimation for each unigene, reads from each sample were then mapped as separately onto the assembled transcripts i.e. unigenes and the read count for each unigene was obtained. The expression quantity of each gene (fragments per kilobase of exon model per million mapped fragments, FPKM) was estimated using RSEM (Li and Dewey, 2011) provided within the Trinity package. Potential simple sequence repeats (SSRs) were detected using MISA (MIcroSAtellite, http://pgrc.ipk-gatersleben.de/misa/) (Thiel et al., 2003). In present study, repeats of one to six nucleotides in the core motif were considered. Mononucleotide repeats ≥ 8 bases, dinucleotides ≥ 10 bases (five repeats) and trinucleotides and tetranucleotides ≥ 12 bases (four and three repeats respectively), pentanucleotide ≥ 15 bases (3 repeats) and hexanucleotides ≥ 18 bases (3 repeats).

## Results

### Data quality assessment

Next generation sequencing technology has been extensively applied to characterize the transcriptome profiles in multiple non-model plants. Equal quantity of total RNA extracted from the bud and leaf tissue of *C. scarabaeoides* was used to construct cDNA library which was then sequenced on Illumina MiSeq platform. Initially, the base quality of the sequenced reads was evaluated. A total of 30,535,175 raw paired-ends read of ~145 bp were obtained. The raw data has been deposited to NCBI with accession number SRA548291. Clean reads were filtered from raw reads by removal of adaptors, ambiguous reads and low-quality reads. After quality checking and pre-processing approximately 30,531,601 clean reads (7.69 GB) were retained Table 1. The GC content was 45.10% and the Q20 and Q30 were 94.243 and 90.12% respectively.

**Table 1:** Pre and post assembly statistics.

**PRE-ASSEMBLY STATISTIC**				
**Tissues**	**Total raw reads**	**Clean reads (after trimming)**	**Base paires (Mbp)**	**Mean length**
Bud	17,388,560	17,386,773	1,576.86	145
Leaf	13,146,615	13,144,828	1,443.43	144
	**Trinity based *de novo* assembly**			
	Total trinity transcripts:	117,007		
	Percent GC	41.48		
	Contig N10	3,403		
	Contig N20	2,621		
	Contig N30	2,127		
	Contig N40	1,758		
	Contig N50	1,422		
	Median contig length	487		
	Average contig	850.42		
	Total assembled bases	99,504,824		

### *De novo* assembly and validation

All the clean reads were *de novo* assembled by Trinity program (Grabherr et al., 2011), which generated a total of 1,17,007 transcripts with an average length of 487 bp and an N50 of 1,422 bp (Table 1). These transcripts then fed to Cluster Database at High Identity with Tolerance-EST i.e., CD-HIT-EST software (Nakasugi et al., 2013) with percent identity 95% to remove redundancy at sequence level producing non-redundant' (nr) representative sequences as output. In this way, number of assembled transcripts got reduced from a total of 1,17,007 to 98,664 transcripts, and defined as unigenes with a mean length of 396 bp and N50 of 1,393. Among the unigenes, the shortest and longest unigenes are 201 and 13,448 bp, respectively. Moreover, 26,876 unigenes were within the 200–400 bp, and 9,444 unigenes were within the 400–1,000 bp. In the meanwhile, we observed that part of unigenes (62,344) were over 1000 bp, which were very helpful to further annotation and functional analysis of unigenes. Assessment of transcriptome assembly was performed with the transcripts (obtained from Trinity assembler). First of all, Bowtie2 was used to align the reads to the constructed transcriptome from Trinity and then we counted the number of proper pairs, improper or orphan read alignments to capture the read alignment statistics. In our case, **93.75%** of the mapped fragments were found mapped as proper pairs (yielding concordant alignments 1 or more times to the reconstructed transcriptome) suggesting our proper assembly. The remaining unassembled reads likely correspond to lowly expressed transcripts with unsatisfactory coverage, or are of low quality or aberrant reads. A better *de novo* assembly was obtained in current sequencing data and the results are shown in Supplementary Material, File 1.

Again at this level, the quality assessment of transcriptome assembly was done through the examination of the full-length or nearly full-length transcripts numbers. Therefore we performed blastx analysis of the assembled transcripts i.e. unigenes against *Glycine max* coding sequence (CDS) and uniprot database of whole Viridiplantae with the stringent *E*-value of 1e^−5^ and percent identity ≥ 90% resulted in 72,703 (73.68%) and 81,799 (82.90%) uniquely mapped unigenes (Table 2; Supplementary Material, Files 2, 3).

**Table 2:** Unigenes mapping results with *Glycine max* coding sequence (Ensembl).

***Mapping***	***Unigenes***
Number of unigenes (n)	98664
Number of uniquely mapped unigenes to Glycine max CDS (n)	72703
Total number of CDS in Glycine max (n)	88647
Total number of unique mapped CDS in Glycine max (n)	72382
Total length of CDS in Glycine max (bp)	113030349
Total length of mapped CDS in Glycine max (bp)	91164324
Total length of unigenes/transcripts (bp)	50469865
Total length of mapped unigenes/transcripts(bp)	31499627
Total length of overlapping sequences (bp)	29345789
Percentage of mapped CDS number	73.19 %

Further, a total of 23,475 SSR loci were identified in 27,321 unigene sequences of which 6128 sequences contained more than one SSR (Supplementary Material, File 4). For identification of different types of SSRs, only the perfect repeats were used. Among them, mononucleotide was the most abundant repeat unit i.e. 12613, followed by trinucleotide which was 4402, 3027 dinucleotide, 1,139 tetranucleotide, 1,356 hexanucleotide and 938 pentanucleotide.

## Conclusion

This is the first report of a transcriptome database *Cajanus scarabaeoides* which is a closest wild relative to *C. cajan*, and one of the easiest wild species that can crossed with pigeonpea cultivars. Next-generation RNA sequencing was performed on mRNAs obtained from bud and leaf tissue. Unigenes and SSR makers identified in present study can be used be used for improvement of cultivated pigeon pea with specific traits, however extended deep researches are required to confirm these findings. Incorporation of different facts at transcriptome, metabolome and proteome approaches would lead to comprehensive research programs focus on engineering *C. scarabaeoides*.

## Author contributions

KG and NS conceived this study. KG designed the experimental plan. SS and GR participated in sample collection, RNA sequencing. DN analyzed the sequence data. DN and SS wrote the manuscript. All authors have read and approved the final manuscript.

### Conflict of interest statement

The authors declare that the research was conducted in the absence of any commercial or financial relationships that could be construed as a potential conflict of interest.
