# Supplementary material for: De novo Assembly and Characterization of Cajanus scarabaeoides (L.) Thouars Transcriptome by Paired-End Sequencing
Source: Front Mol Biosci. 2017 Jul 12;4:48. doi: 10.3389/fmolb.2017.00048 (PMC5506216; doi:10.3389/fmolb.2017.00048)
Supplement: Supplementary file 2 [file DataSheet2.ZIP › Supplementary_file_1_sheet2.doc]

Query_id	Subject_id	Percent_Identity	alignment length	gap open	q_start	q_end	q_start	q_end	E-value	bit score

TRINITY_DN15690_c0_g2_i1	Glyma.08G087600.2	100	467	9	153	8039	3382	11250	0	11762

TRINITY_DN15690_c0_g2_i1	Glyma.08G087600.1	100	467	9	153	8039	3382	11250	0	11762

TRINITY_DN15690_c0_g2_i1	Glyma.05G133100.1	96.95121951	451	8	153	8039	3421	11289	0	11851

TRINITY_DN15690_c0_g2_i1	Glyma.05G133100.2	99.0920882	448	8	153	7937	3421	11187	0	11679

TRINITY_DN15641_c0_g1_i1	Glyma.18G275200.1	96.16519174	491	3	150	7719	15327	7755	0	11212

TRINITY_DN12868_c0_g1_i1	Glyma.17G175000.1	99.17127072	643	81	569	7621	7086	1	0	8246

TRINITY_DN12868_c0_g1_i1	Glyma.17G175000.2	99.18032787	641	82	569	7621	7080	1	0	8218

TRINITY_DN12868_c0_g1_i1	Glyma.05G096000.1	99.16666667	682	90	569	7621	7041	1	0	7873

TRINITY_DN12868_c0_g1_i1	Glyma.05G096000.2	99.16666667	680	91	569	7621	7035	1	0	7845

TRINITY_DN16280_c0_g1_i2	Glyma.15G007100.1	97.87234043	357	2	161	7237	7077	1	0	11081

TRINITY_DN16280_c0_g1_i2	Glyma.13G366100.1	97.87234043	352	0	161	7237	7077	1	0	11119

TRINITY_DN15641_c0_g1_i1	Glyma.08G252600.1	100	421	8	150	7125	15096	8118	0	10491

TRINITY_DN15894_c0_g1_i1	Glyma.04G104900.1	98.97959184	374	4	441	7223	6783	1	0	10432

TRINITY_DN15894_c0_g1_i1	Glyma.06G105900.3	100	368	2	441	7223	6783	1	0	10477

TRINITY_DN15894_c0_g1_i1	Glyma.06G105900.1	98.6970684	368	2	441	7223	6783	1	0	10477

TRINITY_DN15894_c0_g1_i1	Glyma.06G105900.2	100	368	2	441	7223	6783	1	0	10477

TRINITY_DN16374_c1_g1_i4	Glyma.07G069400.1	100	767	72	36	6551	142	6681	0	6986

TRINITY_DN16397_c0_g1_i2	Glyma.04G236900.1	100	373	10	571	7152	6576	1	0	10017

TRINITY_DN16374_c1_g1_i4	Glyma.09G257900.1	100	581	56	36	6545	145	6663	0	8141

TRINITY_DN16374_c1_g1_i3	Glyma.09G257900.1	100	476	32	36	6542	145	6669	0	8887

TRINITY_DN16374_c1_g1_i3	Glyma.18G234700.1	100	497	24	36	6542	145	6669	0	8970

TRINITY_DN16403_c1_g1_i1	Glyma.05G181600.1	100	748	10	323	6784	6456	1	0	7685

TRINITY_DN16403_c1_g1_i2	Glyma.05G181600.1	100	697	10	344	6805	6456	1	0	7967

TRINITY_DN16403_c1_g1_i1	Glyma.08G139300.1	100	737	9	323	6784	6456	1	0	7757

TRINITY_DN16403_c1_g1_i2	Glyma.08G139300.1	100	687	9	344	6805	6456	1	0	8034

TRINITY_DN16403_c1_g1_i2	Glyma.11G237800.1	100	366	2	344	6796	6660	202	0	9862

TRINITY_DN16403_c1_g1_i1	Glyma.11G237800.1	100	314	2	323	6775	6660	202	0	10150

TRINITY_DN16374_c1_g1_i4	Glyma.18G234700.1	100	586	35	36	6427	145	6554	0	8187

TRINITY_DN16397_c0_g1_i2	Glyma.06G127400.1	100	374	0	638	7073	6515	80	0	9814

TRINITY_DN16403_c1_g1_i2	Glyma.18G019600.1	100	353	2	344	6741	6405	2	0	9832

TRINITY_DN16403_c1_g1_i1	Glyma.18G019600.1	100	301	2	323	6720	6405	2	0	10120

TRINITY_DN16374_c1_g1_i6	Glyma.07G069400.1	100	554	42	6	6345	345	6681	0	8120

TRINITY_DN16374_c1_g1_i6	Glyma.09G257900.1	100	738	63	12	6339	348	6663	0	6900

TRINITY_DN16374_c1_g1_i7	Glyma.09G257900.1	100	633	39	12	6336	348	6669	0	7644

TRINITY_DN16374_c1_g1_i7	Glyma.18G234700.1	100	655	32	9	6336	345	6669	0	7705

TRINITY_DN16393_c0_g1_i4	Glyma.06G098700.1	96.42857143	757	62	197	6462	10917	4658	0	6663

TRINITY_DN16393_c0_g1_i4	Glyma.04G096900.2	96.42857143	769	70	197	6462	10956	4706	0	6571

TRINITY_DN16393_c0_g1_i3	Glyma.14G183000.1	96.42857143	796	66	197	6471	10959	4706	0	6532

TRINITY_DN16393_c0_g1_i3	Glyma.14G183000.2	96.42857143	796	66	197	6471	10959	4706	0	6532

TRINITY_DN16393_c0_g1_i3	Glyma.02G216000.1	96.42857143	801	66	197	6471	10950	4697	0	6538

TRINITY_DN16393_c0_g1_i3	Glyma.02G216000.2	96.42857143	801	66	197	6471	10950	4697	0	6538

TRINITY_DN16393_c0_g1_i3	Glyma.06G098700.1	96.42857143	445	30	197	6471	10917	4658	0	8693

TRINITY_DN16393_c0_g1_i3	Glyma.04G096900.2	96.42857143	468	27	197	6471	10956	4706	0	8586

TRINITY_DN16393_c0_g1_i4	Glyma.14G183000.1	96.42857143	546	25	197	6462	10959	4706	0	8292

TRINITY_DN16393_c0_g1_i4	Glyma.14G183000.2	97.74647887	546	25	197	6462	10959	4706	0	8292

TRINITY_DN16393_c0_g1_i4	Glyma.02G216000.1	96.42857143	565	25	197	6462	10950	4697	0	8198

TRINITY_DN16393_c0_g1_i4	Glyma.02G216000.2	96.42857143	565	25	197	6462	10950	4697	0	8198

TRINITY_DN15903_c0_g1_i1	Glyma.08G173500.1	99.24242424	414	5	579	6840	6538	277	0	9226

TRINITY_DN15903_c0_g1_i2	Glyma.08G173500.1	96.875	414	5	413	6674	6538	277	0	9226

TRINITY_DN15903_c0_g1_i3	Glyma.08G173500.1	97.35099338	414	5	674	6935	6538	277	0	9226

TRINITY_DN15903_c0_g1_i1	Glyma.15G253800.1	98.97959184	420	3	579	6840	6538	277	0	9204

TRINITY_DN15903_c0_g1_i1	Glyma.15G253800.2	96.875	420	3	579	6840	6538	277	0	9204

TRINITY_DN15903_c0_g1_i2	Glyma.15G253800.1	95.89041096	420	3	413	6674	6538	277	0	9204

TRINITY_DN15903_c0_g1_i2	Glyma.15G253800.2	100	420	3	413	6674	6538	277	0	9204

TRINITY_DN15903_c0_g1_i3	Glyma.15G253800.1	98.85496183	420	3	674	6935	6538	277	0	9204

TRINITY_DN15903_c0_g1_i3	Glyma.15G253800.2	98.85550787	420	3	674	6935	6538	277	0	9204

TRINITY_DN16374_c1_g1_i6	Glyma.18G234700.1	100	744	43	9	6221	345	6554	0	6922

TRINITY_DN16393_c0_g1_i1	Glyma.06G098700.1	96.42857143	828	63	1	6115	10769	4658	0	5999

TRINITY_DN16393_c0_g1_i1	Glyma.04G096900.2	96.42857143	834	70	1	6115	10808	4706	0	5941

TRINITY_DN16393_c0_g1_i2	Glyma.14G183000.1	96.42857143	725	56	1	6124	10811	4706	0	6722

TRINITY_DN16393_c0_g1_i2	Glyma.14G183000.2	96.42857143	725	56	1	6124	10811	4706	0	6722

TRINITY_DN16393_c0_g1_i2	Glyma.02G216000.1	96.42857143	734	56	1	6124	10802	4697	0	6706

TRINITY_DN16393_c0_g1_i2	Glyma.02G216000.2	96.42857143	734	56	1	6124	10802	4697	0	6706

TRINITY_DN16393_c0_g1_i2	Glyma.06G098700.1	96.42857143	516	31	1	6124	10769	4658	0	8030

TRINITY_DN16393_c0_g1_i2	Glyma.04G096900.2	96.42857143	533	27	1	6124	10808	4706	0	7954

TRINITY_DN16374_c1_g1_i3	Glyma.07G069400.1	100	821	65	36	6093	142	6220	0	5932

TRINITY_DN12050_c0_g2_i3	Glyma.16G093800.38	96.57534247	1038	139	351	6380	1	5958	0	3823

TRINITY_DN12050_c0_g2_i3	Glyma.16G093800.1	96.57534247	1038	139	351	6380	1	5958	0	3823

TRINITY_DN12050_c0_g2_i3	Glyma.16G093800.36	96.58634538	1033	144	351	6380	1	5949	0	3792

TRINITY_DN12050_c0_g2_i3	Glyma.16G093800.15	96.59090909	1033	144	351	6380	1	5949	0	3792

TRINITY_DN12050_c0_g2_i2	Glyma.16G093800.38	99.72677596	1019	139	336	6365	1	5958	0	3928

TRINITY_DN12050_c0_g2_i2	Glyma.16G093800.1	99.7706422	1019	139	336	6365	1	5958	0	3928

TRINITY_DN12050_c0_g2_i2	Glyma.16G093800.36	99.78540773	1014	144	336	6365	1	5949	0	3897

TRINITY_DN12050_c0_g2_i2	Glyma.16G093800.15	99.77426637	1014	144	336	6365	1	5949	0	3897

TRINITY_DN16393_c0_g1_i1	Glyma.14G183000.1	97.73755656	471	17	1	6115	10811	4706	0	8480

TRINITY_DN16393_c0_g1_i1	Glyma.14G183000.2	96.42857143	471	17	1	6115	10811	4706	0	8480

TRINITY_DN16393_c0_g1_i1	Glyma.02G216000.1	96.42857143	494	18	1	6115	10802	4697	0	8364

TRINITY_DN16393_c0_g1_i1	Glyma.02G216000.2	96.42857143	494	18	1	6115	10802	4697	0	8364

TRINITY_DN12050_c0_g2_i3	Glyma.16G093800.37	96.57142857	1039	137	351	6380	1	5955	0	3831

TRINITY_DN12050_c0_g2_i3	Glyma.16G093800.16	96.58119658	1039	137	351	6380	1	5955	0	3831

TRINITY_DN12050_c0_g2_i3	Glyma.16G093800.35	96.59090909	1034	142	351	6380	1	5946	0	3801

TRINITY_DN12050_c0_g2_i3	Glyma.16G093800.14	96.58536585	1034	142	351	6380	1	5946	0	3801

TRINITY_DN12050_c0_g2_i2	Glyma.16G093800.37	99.78118162	1020	137	336	6365	1	5955	0	3936

TRINITY_DN12050_c0_g2_i2	Glyma.16G093800.16	99.74811083	1020	137	336	6365	1	5955	0	3936

TRINITY_DN12050_c0_g2_i2	Glyma.16G093800.35	99.76359338	1015	142	336	6365	1	5946	0	3906

TRINITY_DN12050_c0_g2_i2	Glyma.16G093800.14	99.72144847	1015	142	336	6365	1	5946	0	3906

TRINITY_DN16393_c0_g1_i4	Glyma.04G096900.1	96.42857143	764	70	406	6462	10747	4706	0	6213

TRINITY_DN12050_c0_g2_i3	Glyma.03G081300.5	96.58119658	1057	142	351	6380	1	5928	0	3690

TRINITY_DN12050_c0_g2_i3	Glyma.03G081300.6	96.57701711	1057	142	351	6380	1	5928	0	3690

TRINITY_DN12050_c0_g2_i3	Glyma.03G081300.4	96.57142857	1057	142	351	6380	1	5928	0	3690

TRINITY_DN12050_c0_g2_i3	Glyma.03G081300.3	96.59090909	1057	142	351	6380	1	5928	0	3690

TRINITY_DN12050_c0_g2_i3	Glyma.03G081300.1	96.57142857	1057	142	351	6380	1	5928	0	3690

TRINITY_DN12050_c0_g2_i2	Glyma.03G081300.5	99.73958333	1039	142	336	6365	1	5928	0	3790

TRINITY_DN12050_c0_g2_i2	Glyma.03G081300.6	99.78902954	1039	142	336	6365	1	5928	0	3790

TRINITY_DN12050_c0_g2_i2	Glyma.03G081300.4	99.7706422	1039	142	336	6365	1	5928	0	3790

TRINITY_DN12050_c0_g2_i2	Glyma.03G081300.3	99.72677596	1039	142	336	6365	1	5928	0	3790

TRINITY_DN12050_c0_g2_i2	Glyma.03G081300.1	99.74811083	1039	142	336	6365	1	5928	0	3790

TRINITY_DN12050_c0_g2_i3	Glyma.16G093800.33	98.48484848	1038	134	351	6380	1	5943	0	3892

TRINITY_DN12050_c0_g2_i3	Glyma.16G093800.34	98.48484848	1038	134	351	6380	1	5943	0	3892

TRINITY_DN12050_c0_g2_i3	Glyma.16G093800.7	98.48484848	1038	134	351	6380	1	5943	0	3892

TRINITY_DN12050_c0_g2_i3	Glyma.16G093800.28	96.58119658	1033	139	351	6380	1	5934	0	3862

TRINITY_DN12050_c0_g2_i3	Glyma.16G093800.29	96.58536585	1033	139	351	6380	1	5934	0	3862

TRINITY_DN12050_c0_g2_i3	Glyma.16G093800.13	96.57534247	1033	139	351	6380	1	5934	0	3862

TRINITY_DN12050_c0_g2_i2	Glyma.16G093800.33	99.74937343	1019	134	336	6365	1	5943	0	3997

TRINITY_DN12050_c0_g2_i2	Glyma.16G093800.34	99.79757085	1019	134	336	6365	1	5943	0	3997

TRINITY_DN12050_c0_g2_i2	Glyma.16G093800.7	99.71910112	1019	134	336	6365	1	5943	0	3997

TRINITY_DN12050_c0_g2_i2	Glyma.16G093800.28	99.72144847	1014	139	336	6365	1	5934	0	3967

TRINITY_DN12050_c0_g2_i2	Glyma.16G093800.29	99.72677596	1014	139	336	6365	1	5934	0	3967

TRINITY_DN12050_c0_g2_i2	Glyma.16G093800.13	99.78540773	1014	139	336	6365	1	5934	0	3967

TRINITY_DN12050_c0_g2_i3	Glyma.16G093800.31	98.47328244	1039	134	351	6380	1	5940	0	3879

TRINITY_DN12050_c0_g2_i3	Glyma.16G093800.5	96.58119658	1039	134	351	6380	1	5940	0	3879

TRINITY_DN12050_c0_g2_i3	Glyma.16G093800.25	96.57534247	1034	139	351	6380	1	5931	0	3847

TRINITY_DN12050_c0_g2_i3	Glyma.16G093800.18	96.57142857	1034	139	351	6380	1	5931	0	3847

TRINITY_DN12050_c0_g2_i2	Glyma.16G093800.31	99.73190349	1020	134	336	6365	1	5940	0	3984

TRINITY_DN12050_c0_g2_i2	Glyma.16G093800.5	99.78795589	1020	134	336	6365	1	5940	0	3984

TRINITY_DN12050_c0_g2_i2	Glyma.16G093800.25	99.75062344	1015	139	336	6365	1	5931	0	3952

TRINITY_DN12050_c0_g2_i2	Glyma.16G093800.18	99.2481203	1015	139	336	6365	1	5931	0	3952

TRINITY_DN16393_c0_g1_i1	Glyma.04G096900.1	96.42857143	832	70	62	6115	10747	4706	0	5840

TRINITY_DN16393_c0_g1_i3	Glyma.14G183000.3	96.42857143	780	66	406	6471	10750	4706	0	6235

TRINITY_DN12050_c0_g2_i3	Glyma.16G093800.32	98.48484848	1039	132	351	6380	1	5940	0	3901

TRINITY_DN12050_c0_g2_i3	Glyma.16G093800.41	98.48484848	1039	132	351	6380	1	5940	0	3901

TRINITY_DN12050_c0_g2_i3	Glyma.16G093800.6	98.48484848	1039	132	351	6380	1	5940	0	3901

TRINITY_DN12050_c0_g2_i3	Glyma.16G093800.39	98.47328244	1039	132	351	6380	1	5940	0	3901

TRINITY_DN12050_c0_g2_i3	Glyma.16G093800.2	98.47328244	1039	132	351	6380	1	5940	0	3901

TRINITY_DN12050_c0_g2_i3	Glyma.16G093800.26	96.57258065	1034	137	351	6380	1	5931	0	3869

TRINITY_DN12050_c0_g2_i3	Glyma.16G093800.27	96.57142857	1034	137	351	6380	1	5931	0	3869

TRINITY_DN12050_c0_g2_i3	Glyma.16G093800.12	96.58119658	1034	137	351	6380	1	5931	0	3869

TRINITY_DN12050_c0_g2_i2	Glyma.16G093800.32	99.73614776	1020	132	336	6365	1	5940	0	4006

TRINITY_DN12050_c0_g2_i2	Glyma.16G093800.41	99.76635514	1020	132	336	6365	1	5940	0	4006

TRINITY_DN12050_c0_g2_i2	Glyma.16G093800.6	99.76744186	1020	132	336	6365	1	5940	0	4006

TRINITY_DN12050_c0_g2_i2	Glyma.16G093800.39	99.27007299	1020	132	336	6365	1	5940	0	4006

TRINITY_DN12050_c0_g2_i2	Glyma.16G093800.2	99.74587039	1020	132	336	6365	1	5940	0	4006

TRINITY_DN12050_c0_g2_i2	Glyma.16G093800.26	99.76580796	1015	137	336	6365	1	5931	0	3975

TRINITY_DN12050_c0_g2_i2	Glyma.16G093800.27	99.80314961	1015	137	336	6365	1	5931	0	3975

TRINITY_DN12050_c0_g2_i2	Glyma.16G093800.12	99.72677596	1015	137	336	6365	1	5931	0	3975

TRINITY_DN12050_c0_g2_i3	Glyma.16G093800.30	98.48484848	1040	132	351	6380	1	5937	0	3886

TRINITY_DN12050_c0_g2_i3	Glyma.16G093800.4	98.46938776	1040	132	351	6380	1	5937	0	3886

TRINITY_DN12050_c0_g2_i3	Glyma.16G093800.24	96.58119658	1035	137	351	6380	1	5928	0	3856

TRINITY_DN12050_c0_g2_i3	Glyma.16G093800.17	96.57534247	1035	137	351	6380	1	5928	0	3856

TRINITY_DN12050_c0_g2_i2	Glyma.16G093800.30	99.75186104	1021	132	336	6365	1	5937	0	3991

TRINITY_DN12050_c0_g2_i2	Glyma.16G093800.4	99.74747475	1021	132	336	6365	1	5937	0	3991

TRINITY_DN12050_c0_g2_i2	Glyma.16G093800.24	99.71830986	1016	137	336	6365	1	5928	0	3962

TRINITY_DN12050_c0_g2_i2	Glyma.16G093800.17	99.77578475	1016	137	336	6365	1	5928	0	3962

TRINITY_DN16393_c0_g1_i2	Glyma.14G183000.3	96.42857143	720	56	62	6124	10750	4706	0	6637

TRINITY_DN16393_c0_g1_i3	Glyma.04G096900.1	96.42857143	463	27	406	6471	10747	4706	0	8227

TRINITY_DN16393_c0_g1_i2	Glyma.04G096900.1	96.42857143	531	27	62	6124	10747	4706	0	7853

TRINITY_DN12050_c0_g2_i2	Glyma.08G352600.1	100	553	39	336	6365	1	6024	0	7557

TRINITY_DN12050_c0_g2_i2	Glyma.08G352600.4	100	548	44	336	6365	1	6018	0	7546

TRINITY_DN12050_c0_g2_i2	Glyma.08G352600.2	100	548	44	336	6365	1	6018	0	7546

TRINITY_DN12050_c0_g2_i3	Glyma.08G352600.1	98.48484848	540	39	351	6380	1	6024	0	7629

TRINITY_DN12050_c0_g2_i3	Glyma.08G352600.4	98.48484848	535	44	351	6380	1	6018	0	7618

TRINITY_DN12050_c0_g2_i3	Glyma.08G352600.2	98.48101266	535	44	351	6380	1	6018	0	7618

TRINITY_DN16393_c0_g1_i4	Glyma.14G183000.3	96.42857143	530	25	406	6462	10750	4706	0	7995

TRINITY_DN16393_c0_g1_i1	Glyma.14G183000.3	96.42857143	466	17	62	6115	10750	4706	0	8395

TRINITY_DN16397_c0_g1_i2	Glyma.19G065600.1	100	680	16	658	6695	6102	68	0	7286

TRINITY_DN16374_c1_g1_i3	Glyma.07G069400.2	100	800	65	36	5921	142	6048	0	5731

TRINITY_DN12050_c0_g2_i2	Glyma.18G169600.1	100	546	35	336	6255	1	5908	0	7387

TRINITY_DN12050_c0_g2_i2	Glyma.18G169600.2	100	546	35	336	6255	1	5908	0	7387

TRINITY_DN12050_c0_g2_i2	Glyma.18G169600.3	99.72826087	546	35	336	6255	1	5908	0	7387

TRINITY_DN12050_c0_g2_i2	Glyma.18G169600.4	99.79919679	546	35	336	6255	1	5908	0	7387

TRINITY_DN12050_c0_g2_i2	Glyma.18G169600.5	99.7716895	546	35	336	6255	1	5908	0	7387

TRINITY_DN12050_c0_g2_i3	Glyma.18G169600.1	98.47908745	535	35	351	6270	1	5908	0	7448

TRINITY_DN12050_c0_g2_i3	Glyma.18G169600.2	98.48484848	535	35	351	6270	1	5908	0	7448

TRINITY_DN12050_c0_g2_i3	Glyma.18G169600.3	98.47715736	535	35	351	6270	1	5908	0	7448

TRINITY_DN12050_c0_g2_i3	Glyma.18G169600.4	98.47764034	535	35	351	6270	1	5908	0	7448

TRINITY_DN12050_c0_g2_i3	Glyma.18G169600.5	98.48484848	535	35	351	6270	1	5908	0	7448

TRINITY_DN16374_c1_g1_i4	Glyma.07G069400.2	100	706	61	36	5912	142	6048	0	6305

TRINITY_DN16397_c0_g1_i2	Glyma.19G065600.4	100	656	14	658	6592	5958	27	0	7239

TRINITY_DN16374_c1_g1_i7	Glyma.07G069400.1	100	608	35	6	5887	345	6220	0	7068

TRINITY_DN15176_c0_g1_i1	Glyma.07G211600.1	96.90265487	492	26	1	5841	58	5937	0	7725

TRINITY_DN15176_c0_g1_i1	Glyma.07G211600.2	96.26168224	492	26	1	5841	58	5937	0	7725

TRINITY_DN15176_c0_g1_i1	Glyma.07G211600.3	98	492	26	1	5841	58	5937	0	7725

TRINITY_DN15176_c0_g1_i1	Glyma.07G211600.4	95.7537155	492	26	1	5841	58	5937	0	7725

TRINITY_DN15176_c0_g1_i2	Glyma.07G211600.1	100	492	26	1	5841	58	5937	0	7725

TRINITY_DN15176_c0_g1_i2	Glyma.07G211600.2	100	492	26	1	5841	58	5937	0	7725

TRINITY_DN15176_c0_g1_i2	Glyma.07G211600.3	100	492	26	1	5841	58	5937	0	7725

TRINITY_DN15176_c0_g1_i2	Glyma.07G211600.4	95.59748428	492	26	1	5841	58	5937	0	7725

TRINITY_DN15176_c0_g1_i1	Glyma.02G133400.1	97.84482759	469	16	1	5841	58	5934	0	7834

TRINITY_DN15176_c0_g1_i1	Glyma.02G133400.2	99.07407407	469	16	1	5841	58	5934	0	7834

TRINITY_DN15176_c0_g1_i2	Glyma.02G133400.1	100	469	16	1	5841	58	5934	0	7834

TRINITY_DN15176_c0_g1_i2	Glyma.02G133400.2	100	469	16	1	5841	58	5934	0	7834

TRINITY_DN16271_c0_g1_i1	Glyma.18G300200.1	100	327	2	27	5903	5877	1	0	9009

TRINITY_DN16271_c0_g1_i1	Glyma.08G361500.1	98.63013699	326	2	27	5903	5877	1	0	9014

TRINITY_DN16299_c0_g1_i3	Glyma.12G102400.1	98.1595092	824	45	242	6047	7185	1353	0	5810

TRINITY_DN16299_c0_g1_i4	Glyma.12G102400.1	100	872	49	285	6083	7178	1353	0	5509

TRINITY_DN16299_c0_g1_i2	Glyma.12G102400.1	95.45454545	579	20	242	6062	7185	1353	0	7332

TRINITY_DN16299_c0_g1_i5	Glyma.12G102400.1	97.71309771	627	24	285	6098	7178	1353	0	7031

TRINITY_DN13156_c0_g1_i1	Glyma.04G073900.1	100	443	25	91	5900	11	5826	0	7982

TRINITY_DN13156_c0_g1_i1	Glyma.04G073900.3	100	443	25	91	5900	2	5817	0	7982

TRINITY_DN13156_c0_g1_i1	Glyma.04G073900.2	100	443	25	91	5900	2	5817	0	7982

TRINITY_DN16299_c0_g1_i3	Glyma.06G301900.1	100	822	38	242	6047	7167	1347	0	5899

TRINITY_DN16299_c0_g1_i3	Glyma.06G301900.2	98.1595092	822	39	242	6047	7164	1347	0	5880

TRINITY_DN16299_c0_g1_i2	Glyma.06G301900.1	98.61111111	564	21	242	6062	7167	1347	0	7448

TRINITY_DN16299_c0_g1_i2	Glyma.06G301900.2	97.11538462	564	22	242	6062	7164	1347	0	7428

TRINITY_DN16299_c0_g1_i5	Glyma.12G196800.1	96.98857736	665	21	278	6104	7128	1329	0	6813

TRINITY_DN16299_c0_g1_i4	Glyma.06G301900.1	100	874	40	285	6083	7160	1347	0	5587

TRINITY_DN16299_c0_g1_i4	Glyma.06G301900.2	100	874	41	285	6083	7157	1347	0	5568

TRINITY_DN16299_c0_g1_i5	Glyma.13G305000.1	95	674	24	278	6098	7158	1353	0	6807

TRINITY_DN16299_c0_g1_i5	Glyma.06G301900.1	100	616	23	285	6098	7160	1347	0	7136

TRINITY_DN16299_c0_g1_i5	Glyma.06G301900.2	98.8317757	616	24	285	6098	7157	1347	0	7116

TRINITY_DN16299_c0_g1_i2	Glyma.12G196800.1	97.84313725	729	23	249	6068	7121	1329	0	6434

TRINITY_DN16299_c0_g1_i2	Glyma.13G305000.1	95.45454545	734	26	249	6062	7151	1353	0	6451

TRINITY_DN16299_c0_g1_i4	Glyma.12G196800.1	95.85253456	357	6	278	6089	7128	1329	0	8623

TRINITY_DN16299_c0_g1_i3	Glyma.12G196800.1	100	421	8	249	6053	7121	1329	0	8244

TRINITY_DN16299_c0_g1_i4	Glyma.13G305000.1	100	377	4	278	6083	7158	1353	0	8589

TRINITY_DN16299_c0_g1_i3	Glyma.13G305000.1	96.51162791	437	6	249	6047	7151	1353	0	8233

TRINITY_DN11273_c0_g1_i1	Glyma.07G132300.1	96.1038961	442	5	1016	6802	6126	337	0	8202

TRINITY_DN11273_c0_g1_i2	Glyma.07G132300.1	100	442	5	1031	6817	6126	337	0	8202

TRINITY_DN11273_c0_g1_i1	Glyma.18G181200.1	100	456	4	1016	6802	6123	337	0	8117

TRINITY_DN11273_c0_g1_i1	Glyma.18G181200.2	100	456	4	1016	6802	6123	337	0	8117

TRINITY_DN11273_c0_g1_i1	Glyma.18G181200.3	98.5915493	456	4	1016	6802	6123	337	0	8117

TRINITY_DN11273_c0_g1_i2	Glyma.18G181200.1	100	456	4	1031	6817	6123	337	0	8117

TRINITY_DN11273_c0_g1_i2	Glyma.18G181200.2	98.26589595	456	4	1031	6817	6123	337	0	8117

TRINITY_DN11273_c0_g1_i2	Glyma.18G181200.3	95.80838323	456	4	1031	6817	6123	337	0	8117

TRINITY_DN16374_c1_g1_i7	Glyma.07G069400.2	100	587	35	6	5715	345	6048	0	6866

TRINITY_DN16374_c1_g1_i6	Glyma.07G069400.2	100	493	31	6	5706	345	6048	0	7439

TRINITY_DN12050_c0_g2_i2	Glyma.08G352600.3	99.78494624	522	41	680	6365	288	5967	0	7083

TRINITY_DN12050_c0_g2_i3	Glyma.08G352600.3	98.48484848	522	41	695	6380	288	5967	0	7083

TRINITY_DN11572_c0_g3_i1	Glyma.04G192300.1	96	338	12	257	5965	1	5694	0	8384

TRINITY_DN13464_c0_g1_i1	Glyma.10G295100.1	100	355	2	293	6007	1	5721	0	8560

TRINITY_DN13464_c0_g1_i1	Glyma.10G295100.2	98.75120077	355	2	293	6007	1	5721	0	8560

TRINITY_DN13464_c0_g1_i1	Glyma.20G244900.1	100	353	1	293	6007	1	5718	0	8584

TRINITY_DN16397_c0_g1_i6	Glyma.04G236900.1	98.14814815	453	4	571	6282	6576	871	0	8000

TRINITY_DN16397_c0_g1_i5	Glyma.04G236900.1	98.1595092	413	4	571	6282	6576	871	0	8222

TRINITY_DN16369_c0_g1_i2	Glyma.11G107500.1	100	609	41	1	5648	17	5619	0	6399

TRINITY_DN16316_c0_g1_i2	Glyma.07G252100.1	100	483	29	1	5624	2477	8100	0	7234

TRINITY_DN16316_c0_g1_i2	Glyma.17G022300.3	100	483	38	1	5624	2486	8097	0	7179

TRINITY_DN16316_c0_g1_i2	Glyma.17G022300.1	100	483	38	1	5624	2486	8097	0	7179

TRINITY_DN16316_c0_g1_i2	Glyma.17G022300.2	100	483	38	1	5624	2426	8037	0	7179

TRINITY_DN10368_c0_g2_i1	Glyma.14G024600.1	100	524	22	164	5789	6435	798	0	7276

TRINITY_DN10368_c0_g2_i1	Glyma.14G024600.2	100	522	25	164	5789	6429	798	0	7249

TRINITY_DN10368_c0_g2_i1	Glyma.02G290200.1	97.5	519	13	164	5789	6444	798	0	7361

TRINITY_DN16316_c0_g1_i1	Glyma.07G252100.1	95.8041958	485	25	1	5606	2477	8100	0	7306

TRINITY_DN16316_c0_g1_i1	Glyma.17G022300.3	95.81589958	485	30	1	5606	2486	8097	0	7251

TRINITY_DN16316_c0_g1_i1	Glyma.17G022300.1	100	485	30	1	5606	2486	8097	0	7251

TRINITY_DN16316_c0_g1_i1	Glyma.17G022300.2	95.81673307	485	30	1	5606	2426	8037	0	7251

TRINITY_DN16397_c0_g1_i6	Glyma.06G127400.1	98.16513761	471	0	638	6282	6515	871	0	7816

TRINITY_DN16397_c0_g1_i5	Glyma.06G127400.1	98.16513761	426	0	638	6282	6515	871	0	8065

TRINITY_DN16369_c0_g1_i5	Glyma.11G107500.1	100	473	29	1	5615	17	5619	0	7361

TRINITY_DN16397_c0_g1_i5	Glyma.19G065600.1	98.1595092	536	10	658	6282	6102	481	0	7354

TRINITY_DN16397_c0_g1_i5	Glyma.19G065600.4	98.15100154	536	10	658	6282	5958	337	0	7354

TRINITY_DN16397_c0_g1_i6	Glyma.19G065600.1	98.14814815	518	10	658	6282	6102	481	0	7454

TRINITY_DN16397_c0_g1_i6	Glyma.19G065600.4	98.16513761	518	10	658	6282	5958	337	0	7454

TRINITY_DN5829_c0_g1_i1	Glyma.05G241400.1	99.14529915	384	12	581	6190	5613	1	0	8074

TRINITY_DN5829_c0_g1_i1	Glyma.08G048400.1	98.41269841	352	7	581	6190	5613	1	0	8285

TRINITY_DN16369_c0_g1_i2	Glyma.11G107500.2	100	604	41	1	5583	17	5554	0	6307

TRINITY_DN14718_c0_g2_i1	Glyma.04G156200.1	96.12403101	388	9	561	6137	5568	1	0	8024

TRINITY_DN14718_c0_g2_i1	Glyma.06G223500.1	96.04519774	394	13	583	6137	5567	1	0	7877

TRINITY_DN16369_c0_g1_i5	Glyma.11G107500.2	100	468	29	1	5550	17	5554	0	7269

TRINITY_DN16259_c0_g2_i1	Glyma.01G094200.1	99.56521739	512	58	348	5872	1	5469	0	6529

TRINITY_DN16259_c0_g2_i3	Glyma.01G094200.1	95.19480519	463	54	348	5875	1	5469	0	6818

TRINITY_DN16259_c0_g2_i2	Glyma.01G094200.1	96.19883041	461	56	348	5869	1	5469	0	6791

TRINITY_DN16259_c0_g2_i1	Glyma.08G340500.1	98.84393064	497	42	348	5864	1	5514	0	6927

TRINITY_DN16259_c0_g2_i1	Glyma.08G340500.2	98.84393064	497	46	348	5864	1	5508	0	6889

TRINITY_DN16259_c0_g2_i3	Glyma.08G340500.1	100	448	42	348	5867	1	5514	0	7219

TRINITY_DN16259_c0_g2_i3	Glyma.08G340500.2	98.79518072	448	46	348	5867	1	5508	0	7180

TRINITY_DN16259_c0_g2_i2	Glyma.08G340500.1	99.14529915	446	43	348	5861	1	5514	0	7191

TRINITY_DN16259_c0_g2_i2	Glyma.08G340500.2	96.19834711	446	47	348	5861	1	5508	0	7153

TRINITY_DN5307_c0_g1_i1	Glyma.19G010200.1	100	433	18	884	6419	6526	1021	0	7409

TRINITY_DN15096_c0_g1_i1	Glyma.16G024600.6	96.61016949	346	23	1	5509	237	5766	0	7938

TRINITY_DN15096_c0_g1_i1	Glyma.16G024600.1	99.52606635	346	23	1	5509	237	5766	0	7938

TRINITY_DN15096_c0_g1_i2	Glyma.16G024600.6	98.24561404	346	23	1	5509	237	5766	0	7938

TRINITY_DN15096_c0_g1_i2	Glyma.16G024600.1	98.93617021	346	23	1	5509	237	5766	0	7938

TRINITY_DN15096_c0_g1_i1	Glyma.16G024600.4	95.06398537	346	23	1	5509	3	5532	0	7938

TRINITY_DN15096_c0_g1_i1	Glyma.16G024600.5	100	346	23	1	5509	3	5532	0	7938

TRINITY_DN15096_c0_g1_i2	Glyma.16G024600.4	98.01980198	346	23	1	5509	3	5532	0	7938

TRINITY_DN15096_c0_g1_i2	Glyma.16G024600.5	99.53271028	346	23	1	5509	3	5532	0	7938

TRINITY_DN15096_c0_g1_i1	Glyma.16G024600.2	97.09302326	344	25	1	5509	3	5514	0	7832

TRINITY_DN15096_c0_g1_i1	Glyma.16G024600.3	97.09480122	344	25	1	5509	3	5514	0	7832

TRINITY_DN15096_c0_g1_i2	Glyma.16G024600.2	99.52606635	344	25	1	5509	3	5514	0	7832

TRINITY_DN15096_c0_g1_i2	Glyma.16G024600.3	99.52830189	344	25	1	5509	3	5514	0	7832

TRINITY_DN5307_c0_g1_i1	Glyma.13G170000.1	95.57522124	466	19	884	6419	6607	1099	0	7345

TRINITY_DN5307_c0_g1_i1	Glyma.13G170000.2	100	466	19	884	6419	6076	568	0	7345

TRINITY_DN15096_c0_g1_i1	Glyma.07G055900.2	99.52606635	354	23	1	5511	3	5516	0	7914

TRINITY_DN15096_c0_g1_i1	Glyma.07G055900.3	99.52830189	354	23	1	5511	3	5516	0	7914

TRINITY_DN15096_c0_g1_i1	Glyma.07G055900.4	98.80952381	354	23	1	5511	3	5516	0	7914

TRINITY_DN15096_c0_g1_i1	Glyma.07G055900.1	97.09302326	354	23	1	5511	3	5516	0	7914

TRINITY_DN15096_c0_g1_i2	Glyma.07G055900.2	98.64253394	354	23	1	5511	3	5516	0	7914

TRINITY_DN15096_c0_g1_i2	Glyma.07G055900.3	95.6043956	354	23	1	5511	3	5516	0	7914

TRINITY_DN15096_c0_g1_i2	Glyma.07G055900.4	95.6043956	354	23	1	5511	3	5516	0	7914

TRINITY_DN15096_c0_g1_i2	Glyma.07G055900.1	97.2972973	354	23	1	5511	3	5516	0	7914

TRINITY_DN16369_c0_g1_i5	Glyma.06G003600.1	100	698	40	181	5615	194	5688	0	5738

TRINITY_DN16369_c0_g1_i5	Glyma.06G003600.3	95.5801105	698	40	181	5615	194	5688	0	5738

TRINITY_DN15562_c1_g1_i3	Glyma.08G148300.1	100	280	6	225	5722	1	5508	0	8556

TRINITY_DN15562_c1_g1_i3	Glyma.15G212800.1	100	269	8	225	5722	1	5508	0	8617

TRINITY_DN16369_c0_g1_i2	Glyma.06G003600.1	100	573	23	181	5648	194	5688	0	6665

TRINITY_DN16369_c0_g1_i2	Glyma.06G003600.3	100	573	23	181	5648	194	5688	0	6665

TRINITY_DN13547_c0_g1_i2	Glyma.07G045600.1	98.04878049	395	17	356	5827	1	5478	0	7690

TRINITY_DN10609_c0_g1_i1	Glyma.10G030000.1	100	342	2	751	6237	10830	5335	0	8196

TRINITY_DN10609_c0_g1_i1	Glyma.10G030000.3	95.83333333	342	2	751	6237	10479	4984	0	8196

TRINITY_DN10609_c0_g1_i1	Glyma.10G030000.2	100	342	2	751	6237	10479	4984	0	8196

TRINITY_DN10609_c0_g1_i1	Glyma.02G144200.1	100	321	5	751	6237	10818	5326	0	8294

TRINITY_DN11572_c0_g1_i1	Glyma.04G192300.1	97.36842105	320	13	257	5707	1	5434	0	7995

TRINITY_DN11572_c0_g2_i1	Glyma.04G192300.1	98.23529412	320	13	257	5706	1	5434	0	8000

TRINITY_DN13187_c0_g1_i1	Glyma.13G114800.5	96.80851064	350	23	572	6007	1	5439	0	7841

TRINITY_DN13187_c0_g1_i1	Glyma.13G114800.1	96.80851064	350	23	572	6007	1	5439	0	7841

TRINITY_DN13187_c0_g1_i1	Glyma.13G114800.4	96.80365297	350	23	572	6007	1	5439	0	7841

TRINITY_DN13187_c0_g1_i1	Glyma.13G114800.3	96.79487179	350	23	572	6007	1	5439	0	7841

TRINITY_DN13187_c0_g1_i1	Glyma.13G114800.2	96.80851064	350	23	572	6007	1	5439	0	7841

TRINITY_DN12906_c0_g1_i1	Glyma.08G336400.1	98.6013986	282	5	287	5728	5454	1	0	8432

TRINITY_DN16297_c0_g1_i2	Glyma.20G149200.2	95.92274678	488	25	431	5826	1	5435	0	6892

TRINITY_DN16297_c0_g1_i2	Glyma.20G149200.3	97.43589744	488	25	431	5826	1	5435	0	6892

TRINITY_DN16297_c0_g1_i2	Glyma.20G149200.4	98.41269841	488	25	431	5826	1	5435	0	6892

TRINITY_DN16297_c0_g1_i2	Glyma.20G149200.1	99.14529915	488	25	431	5826	1	5435	0	6892

TRINITY_DN12906_c0_g1_i1	Glyma.18G071500.1	100	266	4	287	5728	5448	1	0	8516

TRINITY_DN16297_c0_g1_i2	Glyma.10G245400.2	100	506	35	431	5826	1	5414	0	6735

TRINITY_DN16297_c0_g1_i2	Glyma.10G245400.3	100	506	35	431	5826	1	5414	0	6735

TRINITY_DN16297_c0_g1_i2	Glyma.10G245400.7	100	506	35	431	5826	1	5414	0	6735

TRINITY_DN16297_c0_g1_i2	Glyma.10G245400.6	95.72649573	506	35	431	5826	1	5414	0	6735

TRINITY_DN16297_c0_g1_i2	Glyma.10G245400.1	97.42813918	506	35	431	5826	1	5414	0	6735

TRINITY_DN16297_c0_g1_i2	Glyma.10G245400.5	96.20689655	506	35	431	5826	1	5414	0	6735

TRINITY_DN16297_c0_g1_i2	Glyma.10G245400.4	100	506	35	431	5826	1	5414	0	6735

TRINITY_DN12868_c0_g1_i1	Glyma.05G096000.3	99.18032787	554	72	2284	7621	5353	1	0	5710

TRINITY_DN16369_c0_g1_i5	Glyma.04G004000.1	100	657	38	236	5615	252	5676	0	5943

TRINITY_DN16369_c0_g1_i2	Glyma.04G004000.1	100	528	16	236	5648	252	5676	0	6905

TRINITY_DN15657_c0_g1_i1	Glyma.18G232900.3	99.57983193	877	79	349	5703	1426	6801	0	4412

TRINITY_DN15657_c0_g1_i1	Glyma.18G232900.1	99.57983193	877	79	349	5703	1426	6801	0	4412

TRINITY_DN15657_c0_g1_i1	Glyma.18G232900.2	99.57983193	877	79	349	5703	1366	6741	0	4412

TRINITY_DN16271_c0_g1_i1	Glyma.08G361500.2	97.66081871	312	2	511	5903	5393	1	0	8198

TRINITY_DN13533_c0_g1_i1	Glyma.04G013900.1	97.76119403	294	3	1	5372	734	6108	0	8266

TRINITY_DN12050_c0_g2_i3	Glyma.16G093800.23	96.57794677	901	99	351	5623	1	5234	0	3574

TRINITY_DN12050_c0_g2_i2	Glyma.16G093800.23	98.47715736	882	99	336	5608	1	5234	0	3679

TRINITY_DN13516_c0_g1_i1	Glyma.17G044000.1	100	297	11	278	5593	1	5349	0	7986

TRINITY_DN13516_c0_g1_i1	Glyma.13G115800.1	95.01312336	295	19	278	5593	1	5349	0	7997

TRINITY_DN12050_c0_g2_i3	Glyma.16G093800.11	96.59090909	900	96	351	5623	1	5222	0	3635

TRINITY_DN12050_c0_g2_i2	Glyma.16G093800.11	98.48229342	881	96	336	5608	1	5222	0	3740

TRINITY_DN12050_c0_g2_i3	Glyma.16G093800.10	96.58792651	901	96	351	5623	1	5219	0	3622

TRINITY_DN12050_c0_g2_i2	Glyma.16G093800.10	98.48484848	882	96	336	5608	1	5219	0	3727

TRINITY_DN12050_c0_g2_i3	Glyma.16G093800.42	99.25373134	901	94	351	5623	1	5219	0	3644

TRINITY_DN12050_c0_g2_i3	Glyma.16G093800.9	96.59090909	901	94	351	5623	1	5219	0	3644

TRINITY_DN12050_c0_g2_i3	Glyma.16G093800.3	96.59090909	901	94	351	5623	1	5219	0	3644

TRINITY_DN12050_c0_g2_i3	Glyma.16G093800.40	96.58385093	901	94	351	5623	1	5219	0	3644

TRINITY_DN12050_c0_g2_i2	Glyma.16G093800.42	99.26470588	882	94	336	5608	1	5219	0	3749

TRINITY_DN12050_c0_g2_i2	Glyma.16G093800.9	99.26470588	882	94	336	5608	1	5219	0	3749

TRINITY_DN12050_c0_g2_i2	Glyma.16G093800.3	99.26470588	882	94	336	5608	1	5219	0	3749

TRINITY_DN12050_c0_g2_i2	Glyma.16G093800.40	99.74093264	882	94	336	5608	1	5219	0	3749

TRINITY_DN12050_c0_g2_i3	Glyma.16G093800.8	99.25925926	902	94	351	5623	1	5216	0	3629

TRINITY_DN12050_c0_g2_i2	Glyma.16G093800.8	98.48484848	883	94	336	5608	1	5216	0	3735

TRINITY_DN16369_c0_g1_i2	Glyma.12G032500.2	100	552	26	327	5648	9	5286	0	6364

TRINITY_DN16369_c0_g1_i2	Glyma.12G032500.1	100	552	26	327	5648	9	5286	0	6364

TRINITY_DN11391_c0_g1_i1	Glyma.03G252300.1	100	311	1	1	5296	2601	7893	0	8037

TRINITY_DN11391_c0_g1_i1	Glyma.19G249900.1	96.46302251	304	1	1	5296	2601	7893	0	8076

TRINITY_DN4960_c0_g1_i1	Glyma.04G096100.1	96.3800905	343	7	1	5282	4481	9771	0	7768

TRINITY_DN4960_c0_g1_i1	Glyma.04G096100.4	96.34146341	343	7	1	5282	4481	9771	0	7768

TRINITY_DN4960_c0_g1_i1	Glyma.04G096100.2	96.36363636	343	8	1	5282	4481	9768	0	7749

TRINITY_DN4960_c0_g1_i1	Glyma.04G096100.3	96.36363636	343	8	1	5282	4481	9768	0	7749

TRINITY_DN4960_c0_g2_i1	Glyma.04G096100.1	96.37426901	343	8	1	5279	4481	9771	0	7749

TRINITY_DN4960_c0_g2_i1	Glyma.04G096100.4	96.34146341	343	8	1	5279	4481	9771	0	7749

TRINITY_DN4960_c0_g1_i1	Glyma.06G097800.1	96.36363636	330	8	1	5282	4454	9744	0	7840

TRINITY_DN16369_c0_g1_i5	Glyma.12G032500.2	100	411	11	327	5615	9	5286	0	7363

TRINITY_DN16369_c0_g1_i5	Glyma.12G032500.1	100	411	11	327	5615	9	5286	0	7363

TRINITY_DN4960_c0_g2_i1	Glyma.04G096100.2	96.36363636	343	7	1	5279	4481	9768	0	7762

TRINITY_DN4960_c0_g2_i1	Glyma.04G096100.3	96.37681159	343	7	1	5279	4481	9768	0	7762

TRINITY_DN4960_c0_g2_i1	Glyma.06G097800.1	96.35036496	330	7	1	5279	4454	9744	0	7854

TRINITY_DN15894_c0_g1_i1	Glyma.06G105900.4	98.97959184	302	0	441	5723	5283	1	0	8083

TRINITY_DN14663_c0_g1_i1	Glyma.07G216600.1	100	388	14	253	5511	1	5268	0	7465

TRINITY_DN14663_c0_g1_i12	Glyma.07G216600.1	97.76119403	388	14	253	5511	1	5268	0	7465

TRINITY_DN14663_c0_g1_i3	Glyma.07G216600.1	95.63106796	388	14	253	5511	1	5268	0	7465

TRINITY_DN14663_c0_g1_i6	Glyma.07G216600.1	99.11660777	388	14	253	5511	1	5268	0	7465

TRINITY_DN14663_c0_g1_i1	Glyma.20G013500.1	100	379	13	253	5511	1	5265	0	7496

TRINITY_DN14663_c0_g1_i12	Glyma.20G013500.1	100	379	13	253	5511	1	5265	0	7496

TRINITY_DN14663_c0_g1_i3	Glyma.20G013500.1	100	379	13	253	5511	1	5265	0	7496

TRINITY_DN14663_c0_g1_i6	Glyma.20G013500.1	99.11816578	379	13	253	5511	1	5265	0	7496

TRINITY_DN13155_c0_g1_i1	Glyma.10G207500.1	100	302	9	468	5650	6081	899	0	7810

TRINITY_DN13155_c0_g1_i1	Glyma.20G183400.1	100	290	7	468	5650	6081	899	0	7899

TRINITY_DN16118_c0_g1_i1	Glyma.08G226700.1	95.93495935	611	41	1	5158	42	5181	0	5703

TRINITY_DN16118_c0_g1_i1	Glyma.07G000400.1	95.93495935	608	32	1	5158	42	5187	0	5770

TRINITY_DN16118_c0_g1_i2	Glyma.08G226700.1	99.23664122	323	11	1	5152	42	5181	0	7448

TRINITY_DN16118_c0_g1_i2	Glyma.07G000400.1	97.05882353	306	9	1	5152	42	5187	0	7581

TRINITY_DN5756_c0_g1_i1	Glyma.03G136000.1	97.36842105	286	2	3	5156	328	5481	0	7923

TRINITY_DN15433_c0_g2_i1	Glyma.07G028800.3	100	423	24	63	5147	1	5127	0	6643

TRINITY_DN15433_c0_g2_i1	Glyma.07G028800.1	96.6442953	423	24	63	5147	1	5127	0	6643

TRINITY_DN15433_c0_g2_i1	Glyma.07G028800.4	98.1981982	423	24	63	5147	1	5127	0	6643

TRINITY_DN15433_c0_g2_i2	Glyma.07G028800.3	98.55072464	423	24	238	5322	1	5127	0	6643

TRINITY_DN15433_c0_g2_i2	Glyma.07G028800.1	98.55072464	423	24	238	5322	1	5127	0	6643

TRINITY_DN15433_c0_g2_i2	Glyma.07G028800.4	98.55072464	423	24	238	5322	1	5127	0	6643

TRINITY_DN15433_c0_g2_i1	Glyma.07G028800.2	98.55072464	421	25	63	5147	1	5121	0	6615

TRINITY_DN15433_c0_g2_i2	Glyma.07G028800.2	96.72727273	421	25	238	5322	1	5121	0	6615

TRINITY_DN2765_c0_g1_i1	Glyma.19G186900.1	96.2962963	259	0	70	5202	1	5133	0	8045

TRINITY_DN16395_c0_g3_i2	Glyma.14G058300.1	98.39034205	350	1	424	5542	1	5098	0	7378

TRINITY_DN16395_c0_g3_i2	Glyma.02G258200.1	98.38709677	339	0	424	5542	1	5119	0	7576

TRINITY_DN16395_c0_g3_i1	Glyma.14G058300.1	98.38709677	213	1	424	5542	1	5098	0	8137

TRINITY_DN16395_c0_g3_i1	Glyma.02G258200.1	98.4	205	0	424	5542	1	5119	0	8318

TRINITY_DN16395_c0_g3_i1	Glyma.18G026000.1	96.1038961	437	1	424	5538	1	5097	0	6909

TRINITY_DN16395_c0_g3_i2	Glyma.18G026000.1	98.40546697	321	1	424	5538	1	5097	0	7552

TRINITY_DN16395_c0_g3_i1	Glyma.11G231100.1	98.38709677	435	1	424	5534	1	5093	0	6913

TRINITY_DN16395_c0_g3_i2	Glyma.11G231100.1	96.11111111	319	1	424	5534	1	5093	0	7555

TRINITY_DN11671_c0_g1_i1	Glyma.03G091100.1	96	529	9	62	5149	1	5103	0	6340

TRINITY_DN14159_c0_g1_i1	Glyma.07G035600.1	95.2	562	24	180	5228	16308	11263	0	5969

TRINITY_DN16369_c0_g1_i1	Glyma.11G107500.2	100	502	35	1	5038	17	5018	0	5947

TRINITY_DN16369_c0_g1_i1	Glyma.11G107500.1	100	502	35	1	5038	17	5018	0	5947

TRINITY_DN15657_c0_g1_i3	Glyma.18G232900.3	96.11111111	598	56	1	4998	1780	6801	0	5485

TRINITY_DN15657_c0_g1_i3	Glyma.18G232900.1	96.11111111	598	56	1	4998	1780	6801	0	5485

TRINITY_DN15657_c0_g1_i3	Glyma.18G232900.2	97.24137931	598	56	1	4998	1720	6741	0	5485

TRINITY_DN13533_c0_g1_i1	Glyma.06G013800.1	100	293	4	1	5043	734	5778	0	7659

TRINITY_DN13411_c0_g1_i1	Glyma.04G096600.1	95.69892473	336	10	679	5701	7536	2503	0	7341

TRINITY_DN13411_c0_g1_i1	Glyma.06G098300.1	96.66666667	341	15	679	5701	7917	2899	0	7239

TRINITY_DN13411_c0_g1_i1	Glyma.06G098300.4	96.32768362	341	15	679	5701	7899	2881	0	7239

TRINITY_DN13411_c0_g1_i1	Glyma.06G098300.2	97.95918367	341	15	679	5701	7839	2821	0	7239

TRINITY_DN13411_c0_g1_i1	Glyma.06G098300.3	97.2972973	338	16	679	5701	7884	2881	0	7158

TRINITY_DN16369_c0_g1_i3	Glyma.11G107500.2	100	362	25	1	5005	17	5018	0	6907

TRINITY_DN16369_c0_g1_i3	Glyma.11G107500.1	100	362	25	1	5005	17	5018	0	6907

TRINITY_DN15226_c0_g1_i5	Glyma.15G120300.1	97.19626168	412	18	133	5145	1	5007	0	6793

TRINITY_DN15226_c0_g1_i2	Glyma.15G120300.1	97.06601467	403	12	181	5193	1	5007	0	6876

TRINITY_DN15226_c0_g1_i4	Glyma.15G120300.1	95.23809524	403	12	123	5135	1	5007	0	6876

TRINITY_DN15226_c0_g1_i5	Glyma.09G014600.1	96.77419355	396	17	133	5145	1	4998	0	6855

TRINITY_DN15226_c0_g1_i2	Glyma.09G014600.1	95.61128527	375	15	181	5193	1	4998	0	6983

TRINITY_DN15226_c0_g1_i4	Glyma.09G014600.1	100	375	15	123	5135	1	4998	0	6983

TRINITY_DN16395_c0_g3_i1	Glyma.02G228400.1	98.38709677	924	19	429	5438	3	5012	0	4023

TRINITY_DN16395_c0_g3_i1	Glyma.01G179300.1	96.12903226	940	16	429	5438	3	5012	0	3956

TRINITY_DN16395_c0_g3_i1	Glyma.11G062900.1	98.38709677	934	16	429	5438	3	5012	0	3989

TRINITY_DN14663_c0_g1_i10	Glyma.07G216600.1	95.45454545	367	14	253	5256	1	5013	0	7110

TRINITY_DN14663_c0_g1_i11	Glyma.07G216600.1	97.89473684	367	14	253	5256	1	5013	0	7110

TRINITY_DN14663_c0_g1_i2	Glyma.07G216600.1	95.87628866	367	14	253	5256	1	5013	0	7110

TRINITY_DN14663_c0_g1_i4	Glyma.07G216600.1	95.78544061	367	14	253	5256	1	5013	0	7110

TRINITY_DN14663_c0_g1_i10	Glyma.20G013500.1	99.77426637	360	13	253	5256	1	5010	0	7131

TRINITY_DN14663_c0_g1_i11	Glyma.20G013500.1	97.08029197	360	13	253	5256	1	5010	0	7131

TRINITY_DN14663_c0_g1_i2	Glyma.20G013500.1	95.87628866	360	13	253	5256	1	5010	0	7131

TRINITY_DN14663_c0_g1_i4	Glyma.20G013500.1	100	360	13	253	5256	1	5010	0	7131

TRINITY_DN16395_c0_g3_i1	Glyma.14G195400.1	98.38709677	925	17	429	5425	3	4999	0	4004

TRINITY_DN16395_c0_g3_i1	Glyma.02G228400.2	98.41269841	915	19	429	5379	3	4953	0	3964

TRINITY_DN14718_c0_g2_i1	Glyma.06G223500.2	96.11063094	350	8	583	5533	4982	29	0	7123

TRINITY_DN13156_c0_g1_i1	Glyma.06G075000.1	100	371	14	964	5900	839	5772	0	6842

TRINITY_DN13156_c0_g1_i1	Glyma.06G075000.2	99.49238579	371	14	964	5900	830	5763	0	6842

TRINITY_DN15595_c0_g1_i1	Glyma.03G121000.1	100	296	16	723	5634	4948	1	0	7221

TRINITY_DN3656_c0_g1_i1	Glyma.02G195600.1	97.69307924	338	16	2	4907	1659	6561	0	6968

TRINITY_DN15595_c0_g1_i1	Glyma.19G125600.1	98.52398524	312	14	733	5634	4899	1	0	7060

TRINITY_DN15595_c0_g1_i1	Glyma.19G125600.2	96.85314685	312	15	733	5634	4887	1	0	6983

TRINITY_DN3656_c0_g1_i1	Glyma.10G082100.1	100	340	17	2	4907	1659	6525	0	6868

TRINITY_DN15992_c0_g1_i1	Glyma.04G180100.2	96.06299213	439	22	476	5380	4851	1	0	6266

TRINITY_DN15992_c0_g1_i1	Glyma.04G180100.3	96.06299213	439	22	476	5380	4851	1	0	6266

TRINITY_DN15992_c0_g1_i1	Glyma.04G180100.4	96.06299213	439	22	476	5380	4851	1	0	6266

TRINITY_DN15992_c0_g1_i1	Glyma.04G180100.5	95.94594595	439	22	476	5380	4851	1	0	6266

TRINITY_DN15992_c0_g1_i1	Glyma.04G180100.1	100	439	22	476	5380	4851	1	0	6266

TRINITY_DN15992_c0_g1_i2	Glyma.04G180100.2	95.52238806	439	22	344	5248	4851	1	0	6266

TRINITY_DN15992_c0_g1_i2	Glyma.04G180100.3	95.52529183	439	22	344	5248	4851	1	0	6266

TRINITY_DN15992_c0_g1_i2	Glyma.04G180100.4	96.15384615	439	22	344	5248	4851	1	0	6266

TRINITY_DN15992_c0_g1_i2	Glyma.04G180100.5	96.15384615	439	22	344	5248	4851	1	0	6266

TRINITY_DN15992_c0_g1_i2	Glyma.04G180100.1	97.18309859	439	22	344	5248	4851	1	0	6266

TRINITY_DN15992_c0_g1_i3	Glyma.04G180100.2	100	439	22	277	5181	4851	1	0	6266

TRINITY_DN15992_c0_g1_i3	Glyma.04G180100.3	100	439	22	277	5181	4851	1	0	6266

TRINITY_DN15992_c0_g1_i3	Glyma.04G180100.4	100	439	22	277	5181	4851	1	0	6266

TRINITY_DN15992_c0_g1_i3	Glyma.04G180100.5	100	439	22	277	5181	4851	1	0	6266

TRINITY_DN15992_c0_g1_i3	Glyma.04G180100.1	96.7032967	439	22	277	5181	4851	1	0	6266

TRINITY_DN15992_c0_g1_i1	Glyma.06G184600.1	95.65217391	437	24	476	5380	4842	1	0	6218

TRINITY_DN15992_c0_g1_i2	Glyma.06G184600.1	96.2962963	437	24	344	5248	4842	1	0	6218

TRINITY_DN15992_c0_g1_i3	Glyma.06G184600.1	96.52406417	437	24	277	5181	4842	1	0	6218

TRINITY_DN16369_c0_g1_i3	Glyma.06G003600.1	100	713	44	181	5002	194	5084	0	4503

TRINITY_DN16369_c0_g1_i3	Glyma.06G003600.3	100	713	44	181	5002	194	5084	0	4503

TRINITY_DN16369_c0_g1_i1	Glyma.06G003600.1	100	588	28	181	5035	194	5084	0	5432

TRINITY_DN16369_c0_g1_i1	Glyma.06G003600.3	100	588	28	181	5035	194	5084	0	5432

TRINITY_DN14663_c0_g1_i1	Glyma.07G216600.2	100	358	14	253	5111	1	4868	0	6892

TRINITY_DN14663_c0_g1_i10	Glyma.07G216600.2	99.66555184	358	14	253	5111	1	4868	0	6892

TRINITY_DN14663_c0_g1_i11	Glyma.07G216600.2	95.37037037	358	14	253	5111	1	4868	0	6892

TRINITY_DN14663_c0_g1_i12	Glyma.07G216600.2	97.61904762	358	14	253	5111	1	4868	0	6892

TRINITY_DN14663_c0_g1_i2	Glyma.07G216600.2	100	358	14	253	5111	1	4868	0	6892

TRINITY_DN14663_c0_g1_i3	Glyma.07G216600.2	98.61111111	358	14	253	5111	1	4868	0	6892

TRINITY_DN14663_c0_g1_i4	Glyma.07G216600.2	99.11504425	358	14	253	5111	1	4868	0	6892

TRINITY_DN14663_c0_g1_i6	Glyma.07G216600.2	99.11894273	358	14	253	5111	1	4868	0	6892

TRINITY_DN10114_c0_g1_i1	Glyma.15G092400.3	95.18072289	322	3	96	4955	1	4863	0	7143

TRINITY_DN10114_c0_g1_i1	Glyma.15G092400.1	100	322	3	96	4955	1	4863	0	7143

TRINITY_DN10114_c0_g1_i1	Glyma.15G092400.4	97.07792208	322	3	96	4955	1	4863	0	7143

TRINITY_DN10114_c0_g1_i1	Glyma.15G092400.5	100	322	3	96	4955	1	4863	0	7143

TRINITY_DN10114_c0_g1_i1	Glyma.15G092400.2	99.46236559	322	3	96	4955	1	4863	0	7143

TRINITY_DN13595_c0_g1_i1	Glyma.03G128300.2	100	225	5	440	5293	4866	4	0	7675

TRINITY_DN10114_c0_g1_i1	Glyma.13G220000.1	100	325	5	101	4955	6	4863	0	7107

TRINITY_DN10114_c0_g1_i1	Glyma.13G220000.2	98.07692308	325	5	101	4955	6	4863	0	7107

TRINITY_DN9975_c0_g1_i1	Glyma.09G123500.1	100	278	3	489	5333	4848	1	0	7360

TRINITY_DN13595_c0_g1_i1	Glyma.03G128300.1	100	220	5	461	5293	4845	4	0	7664

TRINITY_DN15316_c0_g2_i1	Glyma.05G031000.1	95.91836735	413	16	1112	5916	4878	59	0	6294

TRINITY_DN15316_c0_g2_i1	Glyma.05G031000.2	100	410	18	1112	5916	4875	59	0	6292

TRINITY_DN15316_c0_g2_i2	Glyma.05G031000.1	97.6744186	408	23	1112	5898	4878	59	0	6205

TRINITY_DN15316_c0_g2_i2	Glyma.05G031000.2	97.6744186	405	25	1112	5898	4875	59	0	6204

TRINITY_DN16369_c0_g1_i3	Glyma.04G004000.1	100	672	39	236	5002	252	5072	0	4710

TRINITY_DN16369_c0_g1_i1	Glyma.04G004000.1	100	543	21	236	5035	252	5072	0	5670

TRINITY_DN9980_c0_g1_i1	Glyma.04G123500.1	96.15384615	300	6	3	4805	412	5209	0	7153

TRINITY_DN12696_c0_g1_i1	Glyma.06G117700.2	98.88888889	401	33	1245	6005	1348	6120	0	6172

TRINITY_DN12696_c0_g1_i1	Glyma.06G117700.1	98.88888889	401	33	1245	6005	1348	6120	0	6172

TRINITY_DN9980_c0_g1_i1	Glyma.09G215000.1	98.63013699	300	3	3	4805	412	5212	0	7184

TRINITY_DN9980_c0_g1_i1	Glyma.09G215000.2	98.63013699	300	3	3	4805	265	5065	0	7184

TRINITY_DN16397_c0_g1_i2	Glyma.14G162300.1	100	568	12	2168	6965	4985	191	0	5638

TRINITY_DN12696_c0_g1_i1	Glyma.04G245400.1	98.9010989	403	29	1245	6005	1480	6252	0	6172

TRINITY_DN12050_c0_g2_i3	Glyma.03G081300.2	96.58119658	818	95	351	5087	1	4674	0	3155

TRINITY_DN12050_c0_g2_i2	Glyma.03G081300.2	98.48484848	800	95	336	5072	1	4674	0	3254

TRINITY_DN15316_c0_g2_i1	Glyma.17G095800.2	100	400	18	1113	5878	4877	106	0	6292

TRINITY_DN15316_c0_g2_i1	Glyma.17G095800.1	99.49494949	400	18	1113	5878	4877	106	0	6292

TRINITY_DN15316_c0_g2_i2	Glyma.17G095800.2	96.11650485	396	21	1113	5860	4877	106	0	6198

TRINITY_DN15316_c0_g2_i2	Glyma.17G095800.1	96.11650485	396	21	1113	5860	4877	106	0	6198

TRINITY_DN11572_c0_g3_i1	Glyma.06G173500.1	99.34640523	310	0	1178	5970	31	4823	0	7134

TRINITY_DN10905_c0_g3_i2	Glyma.15G268800.5	97.26027397	301	2	335	5113	5847	1063	0	7131

TRINITY_DN10905_c0_g3_i2	Glyma.15G268800.4	97.61904762	301	2	335	5113	5847	1063	0	7131

TRINITY_DN10905_c0_g3_i2	Glyma.15G268800.3	97.61904762	301	2	335	5113	5847	1063	0	7131

TRINITY_DN10905_c0_g3_i2	Glyma.15G268800.1	97.61904762	301	2	335	5113	5847	1063	0	7131

TRINITY_DN10905_c0_g3_i2	Glyma.15G268800.2	95.41284404	301	2	335	5113	5847	1063	0	7131

TRINITY_DN10905_c0_g3_i2	Glyma.15G268800.6	95.33678756	301	2	335	5113	5847	1063	0	7131

TRINITY_DN10905_c0_g3_i2	Glyma.08G157400.1	96.20253165	315	1	335	5113	5844	1063	0	7066

TRINITY_DN16369_c0_g1_i5	Glyma.06G003600.2	95.58652729	579	26	882	5615	1	4770	0	5247

TRINITY_DN16369_c0_g1_i2	Glyma.06G003600.2	100	454	9	882	5648	1	4770	0	6174

TRINITY_DN15597_c0_g1_i1	Glyma.19G012300.1	100	462	23	377	5131	1	4725	0	5829

TRINITY_DN15597_c0_g1_i1	Glyma.19G012300.7	96.61835749	462	23	377	5131	1	4725	0	5829

TRINITY_DN15597_c0_g1_i1	Glyma.19G012300.4	98.07692308	462	23	377	5131	1	4725	0	5829

TRINITY_DN15597_c0_g1_i1	Glyma.19G012300.8	96.2962963	462	23	377	5131	1	4725	0	5829

TRINITY_DN15597_c0_g1_i1	Glyma.19G012300.9	98.87218045	462	23	377	5131	1	4725	0	5829

TRINITY_DN15597_c0_g1_i2	Glyma.19G012300.1	100	462	23	377	5131	1	4725	0	5829

TRINITY_DN15597_c0_g1_i2	Glyma.19G012300.7	100	462	23	377	5131	1	4725	0	5829

TRINITY_DN15597_c0_g1_i2	Glyma.19G012300.4	100	462	23	377	5131	1	4725	0	5829

TRINITY_DN15597_c0_g1_i2	Glyma.19G012300.8	100	462	23	377	5131	1	4725	0	5829

TRINITY_DN15597_c0_g1_i2	Glyma.19G012300.9	97.08029197	462	23	377	5131	1	4725	0	5829

TRINITY_DN15597_c0_g1_i3	Glyma.19G012300.1	95.83333333	462	23	377	5131	1	4725	0	5829

TRINITY_DN15597_c0_g1_i3	Glyma.19G012300.7	95.83333333	462	23	377	5131	1	4725	0	5829

TRINITY_DN15597_c0_g1_i3	Glyma.19G012300.4	98.11320755	462	23	377	5131	1	4725	0	5829

TRINITY_DN15597_c0_g1_i3	Glyma.19G012300.8	98.36956522	462	23	377	5131	1	4725	0	5829

TRINITY_DN15597_c0_g1_i3	Glyma.19G012300.9	98.36065574	462	23	377	5131	1	4725	0	5829

TRINITY_DN15597_c0_g1_i4	Glyma.19G012300.1	98.93617021	462	23	377	5131	1	4725	0	5829

TRINITY_DN15597_c0_g1_i4	Glyma.19G012300.7	96.2962963	462	23	377	5131	1	4725	0	5829

TRINITY_DN15597_c0_g1_i4	Glyma.19G012300.4	96.2962963	462	23	377	5131	1	4725	0	5829

TRINITY_DN15597_c0_g1_i4	Glyma.19G012300.8	97.2972973	462	23	377	5131	1	4725	0	5829

TRINITY_DN15597_c0_g1_i4	Glyma.19G012300.9	100	462	23	377	5131	1	4725	0	5829

TRINITY_DN15597_c0_g1_i1	Glyma.13G070500.1	97.98270893	463	17	377	5131	1	4743	0	5962

TRINITY_DN15597_c0_g1_i2	Glyma.13G070500.1	98.42519685	463	17	377	5131	1	4743	0	5962

TRINITY_DN15597_c0_g1_i3	Glyma.13G070500.1	96.37681159	463	17	377	5131	1	4743	0	5962

TRINITY_DN15597_c0_g1_i4	Glyma.13G070500.1	98.93617021	463	17	377	5131	1	4743	0	5962

TRINITY_DN10217_c0_g2_i2	Glyma.01G128700.1	97.16312057	483	38	728	5443	5085	373	0	5493

TRINITY_DN10217_c0_g2_i2	Glyma.01G128700.2	97.14285714	483	38	728	5443	5085	373	0	5493

TRINITY_DN13516_c0_g1_i1	Glyma.17G044000.2	99.33774834	242	7	847	5593	36	4788	0	7365

TRINITY_DN10217_c0_g2_i2	Glyma.03G041600.1	97.14285714	473	46	728	5443	5067	373	0	5531

TRINITY_DN10217_c0_g2_i2	Glyma.03G041600.2	97.15909091	473	46	728	5443	4872	178	0	5531

TRINITY_DN2765_c0_g1_i1	Glyma.19G186900.2	98.7804878	243	0	451	5202	1	4752	0	7430

TRINITY_DN8107_c0_g2_i1	Glyma.08G187500.1	98.6381323	328	28	169	4888	125	4847	0	6652

TRINITY_DN13595_c0_g1_i1	Glyma.19G130800.1	100	220	4	565	5293	4746	4	0	7439

TRINITY_DN16060_c0_g1_i1	Glyma.05G039400.4	95.88235294	412	28	679	5363	4715	1	0	5921

TRINITY_DN16060_c0_g1_i1	Glyma.05G039400.5	95.88235294	412	28	679	5363	4715	1	0	5921

TRINITY_DN16060_c0_g1_i1	Glyma.05G039400.1	96.66666667	412	28	679	5363	4715	1	0	5921

TRINITY_DN16060_c0_g1_i1	Glyma.05G039400.2	98.39285714	412	28	679	5363	4715	1	0	5921

TRINITY_DN16060_c0_g1_i1	Glyma.05G039400.3	98.39572193	412	28	679	5363	4715	1	0	5921

TRINITY_DN16060_c0_g1_i1	Glyma.05G039400.6	98.39572193	412	28	679	5363	4715	1	0	5921

TRINITY_DN16060_c0_g1_i2	Glyma.05G039400.4	100	412	28	665	5349	4715	1	0	5921

TRINITY_DN16060_c0_g1_i2	Glyma.05G039400.5	96.55172414	412	28	665	5349	4715	1	0	5921

TRINITY_DN16060_c0_g1_i2	Glyma.05G039400.1	98.7012987	412	28	665	5349	4715	1	0	5921

TRINITY_DN16060_c0_g1_i2	Glyma.05G039400.2	98.7012987	412	28	665	5349	4715	1	0	5921

TRINITY_DN16060_c0_g1_i2	Glyma.05G039400.3	98.7012987	412	28	665	5349	4715	1	0	5921

TRINITY_DN16060_c0_g1_i2	Glyma.05G039400.6	95.45454545	412	28	665	5349	4715	1	0	5921

TRINITY_DN16060_c0_g1_i3	Glyma.05G039400.4	99.26470588	412	28	758	5442	4715	1	0	5921

TRINITY_DN16060_c0_g1_i3	Glyma.05G039400.5	100	412	28	758	5442	4715	1	0	5921

TRINITY_DN16060_c0_g1_i3	Glyma.05G039400.1	95.61403509	412	28	758	5442	4715	1	0	5921

TRINITY_DN16060_c0_g1_i3	Glyma.05G039400.2	95.97315436	412	28	758	5442	4715	1	0	5921

TRINITY_DN16060_c0_g1_i3	Glyma.05G039400.3	95.7957958	412	28	758	5442	4715	1	0	5921

TRINITY_DN16060_c0_g1_i3	Glyma.05G039400.6	95.7957958	412	28	758	5442	4715	1	0	5921

TRINITY_DN13105_c1_g1_i1	Glyma.11G081200.2	99.48186528	228	2	165	4906	1	4742	0	7483

TRINITY_DN13105_c1_g1_i1	Glyma.11G081200.1	99.48186528	228	2	165	4906	1	4742	0	7483

TRINITY_DN13105_c1_g1_i1	Glyma.11G081200.3	97.4025974	228	2	165	4906	1	4742	0	7483

TRINITY_DN13105_c1_g1_i2	Glyma.11G081200.2	97.38372093	228	3	165	4903	1	4740	0	7472

TRINITY_DN13105_c1_g1_i2	Glyma.11G081200.1	97.4025974	228	3	165	4903	1	4740	0	7472

TRINITY_DN13105_c1_g1_i2	Glyma.11G081200.3	97.39292365	228	3	165	4903	1	4740	0	7472

TRINITY_DN13595_c0_g1_i1	Glyma.19G130800.2	98.46153846	222	1	565	5293	4731	4	0	7496

TRINITY_DN16369_c0_g1_i1	Glyma.12G032500.2	100	446	20	327	5038	9	4685	0	5904

TRINITY_DN16369_c0_g1_i1	Glyma.12G032500.1	100	446	20	327	5038	9	4685	0	5904

TRINITY_DN13105_c1_g1_i2	Glyma.01G162200.2	97.4025974	231	0	165	4885	1	4721	0	7439

TRINITY_DN13105_c1_g1_i2	Glyma.01G162200.1	97.3880597	231	0	165	4885	1	4721	0	7439

TRINITY_DN13105_c1_g1_i1	Glyma.01G162200.2	97.37609329	230	0	165	4885	1	4721	0	7444

TRINITY_DN13105_c1_g1_i1	Glyma.01G162200.1	97.39776952	230	0	165	4885	1	4721	0	7444

TRINITY_DN15226_c0_g1_i2	Glyma.17G005500.1	100	787	69	194	4865	5	4664	0	3714

TRINITY_DN15226_c0_g1_i4	Glyma.17G005500.1	100	787	69	136	4807	5	4664	0	3714

TRINITY_DN15226_c0_g1_i5	Glyma.17G005500.1	99.28825623	765	67	146	4817	5	4664	0	3847

TRINITY_DN15226_c0_g1_i5	Glyma.07G268200.1	97.19626168	764	57	146	4817	5	4664	0	3886

TRINITY_DN12696_c0_g1_i1	Glyma.04G245400.2	98.89807163	394	28	1332	6005	1460	6141	0	6102

TRINITY_DN12696_c0_g1_i1	Glyma.04G245400.3	98.88888889	394	28	1332	6005	1460	6141	0	6102

TRINITY_DN14527_c0_g1_i2	Glyma.19G095700.1	98.7012987	338	18	77	4751	4849	160	0	6481

TRINITY_DN15562_c1_g1_i3	Glyma.15G212800.2	96.07843137	219	1	1017	5722	1	4707	0	7472

TRINITY_DN16264_c3_g2_i2	Glyma.07G209300.1	100	412	17	149	4824	8378	3703	0	6043

TRINITY_DN16264_c3_g2_i2	Glyma.02G135700.1	100	401	12	148	4824	7194	2500	0	6244

TRINITY_DN14527_c0_g1_i2	Glyma.16G053500.1	98.7012987	346	14	77	4751	4849	160	0	6503

TRINITY_DN15226_c0_g1_i2	Glyma.07G268200.1	97.18309859	783	57	209	4865	20	4664	0	3753

TRINITY_DN15226_c0_g1_i4	Glyma.07G268200.1	98.27586207	783	57	151	4807	20	4664	0	3753

TRINITY_DN9975_c0_g1_i1	Glyma.09G123500.2	100	261	2	489	5172	4686	3	0	7171

TRINITY_DN11105_c0_g1_i1	Glyma.11G002600.1	95.23809524	222	0	1	4685	2723	7407	0	7422

TRINITY_DN11105_c0_g1_i1	Glyma.01G241300.1	100	216	0	1	4685	2723	7407	0	7456

TRINITY_DN11105_c0_g1_i1	Glyma.01G241300.2	100	216	0	1	4685	1907	6591	0	7456

TRINITY_DN16389_c1_g1_i2	Glyma.10G249300.2	95.16129032	464	66	1566	6113	4614	1	0	4748

TRINITY_DN16389_c1_g1_i2	Glyma.10G249300.1	95.18072289	464	66	1566	6113	4614	1	0	4748

TRINITY_DN16369_c0_g1_i3	Glyma.12G032500.2	100	301	7	327	5005	9	4685	0	6905

TRINITY_DN16369_c0_g1_i3	Glyma.12G032500.1	100	301	7	327	5005	9	4685	0	6905

TRINITY_DN16156_c0_g1_i3	Glyma.18G221200.1	100	560	16	270	4941	5350	685	0	5376

TRINITY_DN16156_c0_g1_i3	Glyma.03G022900.1	100	555	22	271	4941	5349	688	0	5371

TRINITY_DN16156_c0_g1_i1	Glyma.18G221200.1	100	491	14	270	4941	5350	685	0	5770

TRINITY_DN16156_c0_g1_i1	Glyma.03G022900.1	99.31972789	493	17	271	4941	5349	688	0	5736

TRINITY_DN16156_c0_g1_i1	Glyma.09G268400.1	98	385	8	271	4941	5355	682	0	6445

TRINITY_DN16156_c0_g1_i3	Glyma.09G268400.1	99.58847737	285	8	271	4941	5355	682	0	6999

TRINITY_DN15789_c0_g1_i1	Glyma.17G249900.1	96.46464646	441	22	1	4646	710	5352	0	5884

TRINITY_DN16389_c1_g1_i2	Glyma.10G249300.4	95.18072289	464	65	1566	6113	4596	1	0	4831

TRINITY_DN16389_c1_g1_i2	Glyma.10G249300.3	95.18072289	464	65	1566	6113	4596	1	0	4831

TRINITY_DN15597_c0_g1_i1	Glyma.19G012300.2	100	453	22	491	5131	73	4686	0	5686

TRINITY_DN15597_c0_g1_i1	Glyma.19G012300.5	97.56097561	453	22	491	5131	73	4686	0	5686

TRINITY_DN15597_c0_g1_i1	Glyma.19G012300.3	96.27071823	453	22	491	5131	73	4686	0	5686

TRINITY_DN15597_c0_g1_i1	Glyma.19G012300.6	97.08737864	453	22	491	5131	73	4686	0	5686

TRINITY_DN15597_c0_g1_i2	Glyma.19G012300.2	97.43589744	453	22	491	5131	73	4686	0	5686

TRINITY_DN15597_c0_g1_i2	Glyma.19G012300.5	96.875	453	22	491	5131	73	4686	0	5686

TRINITY_DN15597_c0_g1_i2	Glyma.19G012300.3	100	453	22	491	5131	73	4686	0	5686

TRINITY_DN15597_c0_g1_i2	Glyma.19G012300.6	100	453	22	491	5131	73	4686	0	5686

TRINITY_DN15597_c0_g1_i3	Glyma.19G012300.2	99.41860465	453	22	491	5131	73	4686	0	5686

TRINITY_DN15597_c0_g1_i3	Glyma.19G012300.5	95.87155963	453	22	491	5131	73	4686	0	5686

TRINITY_DN15597_c0_g1_i3	Glyma.19G012300.3	100	453	22	491	5131	73	4686	0	5686

TRINITY_DN15597_c0_g1_i3	Glyma.19G012300.6	100	453	22	491	5131	73	4686	0	5686

TRINITY_DN15597_c0_g1_i4	Glyma.19G012300.2	95.89041096	453	22	491	5131	73	4686	0	5686

TRINITY_DN15597_c0_g1_i4	Glyma.19G012300.5	97.46835443	453	22	491	5131	73	4686	0	5686

TRINITY_DN15597_c0_g1_i4	Glyma.19G012300.3	95.3271028	453	22	491	5131	73	4686	0	5686

TRINITY_DN15597_c0_g1_i4	Glyma.19G012300.6	95.41284404	453	22	491	5131	73	4686	0	5686

TRINITY_DN10030_c0_g2_i1	Glyma.01G166400.1	99.45054945	219	6	3	4628	7012	11631	0	7247

TRINITY_DN10030_c0_g2_i1	Glyma.11G077000.1	96.44268775	224	2	1	4628	7010	11631	0	7267

TRINITY_DN16118_c0_g1_i1	Glyma.08G226700.2	95.94017094	566	39	568	5158	447	5019	0	4916

TRINITY_DN16118_c0_g1_i1	Glyma.07G000400.2	95.93495935	560	32	568	5158	447	5025	0	4988

TRINITY_DN11422_c0_g1_i1	Glyma.06G178200.1	100	380	9	340	4941	55	4668	0	6327

TRINITY_DN11422_c0_g1_i1	Glyma.06G178200.2	100	380	9	340	4941	55	4668	0	6327

TRINITY_DN11422_c0_g1_i1	Glyma.04G187600.3	98.98648649	374	10	340	4941	55	4656	0	6283

TRINITY_DN11422_c0_g1_i1	Glyma.04G187600.1	98	374	10	340	4941	55	4656	0	6283

TRINITY_DN15270_c0_g1_i1	Glyma.02G281000.1	98.36065574	265	8	29	4632	694	5300	0	6931

TRINITY_DN15270_c0_g1_i1	Glyma.14G033600.3	100	269	4	29	4632	688	5294	0	6931

TRINITY_DN15270_c0_g1_i1	Glyma.14G033600.1	96.09375	269	4	29	4632	688	5294	0	6931

TRINITY_DN15270_c0_g1_i1	Glyma.14G033600.2	95.55555556	269	4	29	4632	688	5294	0	6931

TRINITY_DN13400_c0_g1_i1	Glyma.19G208400.1	95.23809524	407	19	1	4570	460	5044	0	5827

TRINITY_DN13400_c0_g1_i3	Glyma.19G208400.1	99.2481203	407	19	1	4570	460	5044	0	5827

TRINITY_DN13533_c0_g1_i1	Glyma.06G013800.2	98.57142857	270	3	1	4605	734	5341	0	6983

TRINITY_DN13400_c0_g1_i1	Glyma.03G211200.1	99.2481203	410	25	1	4568	454	5012	0	5662

TRINITY_DN13400_c0_g1_i3	Glyma.03G211200.1	100	410	25	1	4568	454	5012	0	5662

TRINITY_DN16118_c0_g1_i2	Glyma.08G226700.2	95.07389163	290	11	568	5152	447	5019	0	6584

TRINITY_DN16118_c0_g1_i2	Glyma.07G000400.2	100	274	9	568	5152	447	5025	0	6711

TRINITY_DN12946_c0_g1_i3	Glyma.13G156500.1	98.96907216	329	15	261	4841	4914	325	0	6473

TRINITY_DN12946_c0_g1_i3	Glyma.13G156500.2	98.96907216	328	16	261	4841	4908	325	0	6440

TRINITY_DN15231_c1_g4_i2	Glyma.03G133700.1	100	295	7	704	5293	4599	1	0	6789

TRINITY_DN15231_c1_g4_i3	Glyma.03G133700.1	97.49216301	252	2	704	5296	4599	1	0	7059

TRINITY_DN12946_c0_g1_i3	Glyma.17G104100.1	100	323	18	261	4841	4911	328	0	6501

TRINITY_DN15231_c1_g4_i2	Glyma.19G136000.1	99.27536232	301	6	704	5293	4596	1	0	6759

TRINITY_DN12946_c0_g1_i2	Glyma.13G156500.1	98.96907216	329	15	261	4836	4914	325	0	6497

TRINITY_DN12946_c0_g1_i2	Glyma.13G156500.2	98.96907216	328	16	261	4836	4908	325	0	6464

TRINITY_DN15231_c1_g4_i3	Glyma.19G136000.1	97.5	262	1	704	5296	4596	1	0	7016

TRINITY_DN16395_c0_g3_i2	Glyma.14G058300.2	98.41269841	319	0	424	5018	1	4595	0	6719

TRINITY_DN16395_c0_g3_i1	Glyma.14G058300.2	98.39572193	195	0	424	5018	1	4595	0	7406

TRINITY_DN12946_c0_g1_i2	Glyma.17G104100.1	98.96907216	323	18	261	4836	4911	328	0	6525

TRINITY_DN11189_c0_g1_i1	Glyma.09G285300.1	97.6344086	274	27	97	4647	1	4572	0	6556

TRINITY_DN11189_c0_g1_i1	Glyma.20G000700.1	95.23809524	247	22	97	4647	1	4566	0	6667

TRINITY_DN16131_c0_g4_i1	Glyma.13G107400.1	100	209	0	218	4810	1	4593	0	7324

TRINITY_DN16131_c0_g4_i1	Glyma.17G051900.1	96.96969697	208	0	218	4810	1	4593	0	7330

TRINITY_DN9980_c0_g1_i1	Glyma.04G123500.2	96.15384615	286	5	220	4805	3	4582	0	6835

TRINITY_DN10502_c0_g1_i1	Glyma.07G233100.1	98.87640449	280	13	245	4808	8111	12677	0	6685

TRINITY_DN10502_c0_g1_i1	Glyma.07G233100.2	96.47058824	280	13	245	4808	8108	12674	0	6685

TRINITY_DN12789_c0_g1_i1	Glyma.19G037200.1	98.88888889	234	1	220	4788	6393	1819	0	7114

TRINITY_DN12789_c0_g1_i1	Glyma.19G037200.3	97.4025974	234	1	220	4788	4767	193	0	7114

TRINITY_DN12789_c0_g1_i1	Glyma.11G061700.1	95.72649573	233	1	220	4788	6393	1819	0	7119

TRINITY_DN12789_c0_g1_i1	Glyma.11G061700.3	98.61111111	233	1	220	4788	5400	826	0	7119

TRINITY_DN12789_c0_g1_i1	Glyma.11G061700.4	95	233	1	220	4788	5400	826	0	7119

TRINITY_DN12789_c0_g1_i1	Glyma.11G061700.2	98.14814815	233	1	220	4788	5400	826	0	7119

TRINITY_DN16320_c0_g3_i1	Glyma.13G127500.1	96.42857143	401	14	753	5312	4554	1	0	6006

TRINITY_DN11716_c0_g1_i1	Glyma.05G167200.2	100	237	2	531	5103	6453	1881	0	7121

TRINITY_DN11716_c0_g1_i1	Glyma.05G167200.1	99.40828402	237	2	531	5103	6453	1881	0	7121

TRINITY_DN11716_c0_g1_i2	Glyma.05G167200.2	97.1291866	237	2	608	5180	6453	1881	0	7121

TRINITY_DN11716_c0_g1_i2	Glyma.05G167200.1	95.65217391	237	2	608	5180	6453	1881	0	7121

TRINITY_DN11716_c0_g1_i3	Glyma.05G167200.2	96.882494	237	2	516	5088	6453	1881	0	7121

TRINITY_DN11716_c0_g1_i3	Glyma.05G167200.1	100	237	2	516	5088	6453	1881	0	7121

TRINITY_DN11716_c0_g1_i1	Glyma.08G125500.2	99.40828402	240	0	531	5103	6456	1884	0	7116

TRINITY_DN11716_c0_g1_i1	Glyma.08G125500.3	99.41176471	240	0	531	5103	6456	1884	0	7116

TRINITY_DN11716_c0_g1_i1	Glyma.08G125500.1	97.85714286	240	0	531	5103	6456	1884	0	7116

TRINITY_DN11716_c0_g1_i2	Glyma.08G125500.2	95.65217391	240	0	608	5180	6456	1884	0	7116

TRINITY_DN11716_c0_g1_i2	Glyma.08G125500.3	97.52066116	240	0	608	5180	6456	1884	0	7116

TRINITY_DN11716_c0_g1_i2	Glyma.08G125500.1	97.05882353	240	0	608	5180	6456	1884	0	7116

TRINITY_DN11716_c0_g1_i3	Glyma.08G125500.2	100	240	0	516	5088	6456	1884	0	7116

TRINITY_DN11716_c0_g1_i3	Glyma.08G125500.3	97.79411765	240	0	516	5088	6456	1884	0	7116

TRINITY_DN11716_c0_g1_i3	Glyma.08G125500.1	99.74587039	240	0	516	5088	6456	1884	0	7116

TRINITY_DN16320_c0_g3_i1	Glyma.19G169000.1	96.42857143	420	48	754	5285	4613	85	0	5701

TRINITY_DN16320_c0_g3_i2	Glyma.13G127500.1	96.42857143	281	9	753	5300	4554	1	0	6737

TRINITY_DN16320_c0_g3_i2	Glyma.19G169000.1	96.42857143	562	50	754	5273	4613	85	0	4870

TRINITY_DN16117_c1_g1_i1	Glyma.07G121900.5	97.10144928	328	6	473	5021	4549	7	0	6523

TRINITY_DN16117_c1_g1_i1	Glyma.07G121900.1	97.10144928	328	6	473	5021	4549	7	0	6523

TRINITY_DN16117_c1_g1_i1	Glyma.07G121900.3	96.80851064	328	6	473	5021	4549	7	0	6523

TRINITY_DN16117_c1_g1_i1	Glyma.07G121900.4	96.80851064	328	6	473	5021	4549	7	0	6523

TRINITY_DN16117_c1_g1_i1	Glyma.07G121900.2	98.45360825	328	6	473	5021	4549	7	0	6523

TRINITY_DN16117_c1_g1_i3	Glyma.07G121900.5	100	328	6	473	5021	4549	7	0	6523

TRINITY_DN16117_c1_g1_i3	Glyma.07G121900.1	96.63212435	328	6	473	5021	4549	7	0	6523

TRINITY_DN16117_c1_g1_i3	Glyma.07G121900.3	100	328	6	473	5021	4549	7	0	6523

TRINITY_DN16117_c1_g1_i3	Glyma.07G121900.4	100	328	6	473	5021	4549	7	0	6523

TRINITY_DN16117_c1_g1_i3	Glyma.07G121900.2	98.7012987	328	6	473	5021	4549	7	0	6523

TRINITY_DN16117_c1_g1_i1	Glyma.03G101000.3	96.55172414	362	4	478	5021	4553	7	0	6349

TRINITY_DN16117_c1_g1_i1	Glyma.03G101000.2	96.33300297	362	4	478	5021	4553	7	0	6349

TRINITY_DN16117_c1_g1_i3	Glyma.03G101000.3	100	362	4	478	5021	4553	7	0	6349

TRINITY_DN16117_c1_g1_i3	Glyma.03G101000.2	95.08196721	362	4	478	5021	4553	7	0	6349

TRINITY_DN16118_c0_g1_i1	Glyma.15G000300.3	95.37572254	448	9	620	5158	487	5022	0	5803

TRINITY_DN16118_c0_g1_i1	Glyma.15G000300.1	95.37572254	448	9	620	5158	487	5022	0	5803

TRINITY_DN16118_c0_g1_i1	Glyma.15G000300.2	95.37572254	448	9	620	5158	487	5022	0	5803

TRINITY_DN9975_c0_g1_i1	Glyma.09G123500.3	100	256	3	795	5333	4542	1	0	6916

TRINITY_DN16374_c1_g1_i3	Glyma.16G035100.1	100	592	47	35	4512	144	4660	0	4566

TRINITY_DN16374_c1_g1_i4	Glyma.16G035100.1	100	592	47	35	4512	144	4660	0	4566

TRINITY_DN11572_c0_g1_i1	Glyma.06G173500.1	97.36842105	289	2	1178	5707	31	4558	0	6752

TRINITY_DN16264_c3_g2_i1	Glyma.02G135700.1	96.72131148	385	12	294	4796	7020	2500	0	6011

TRINITY_DN11572_c0_g2_i1	Glyma.06G173500.1	96.77419355	289	1	1178	5706	31	4558	0	6756

TRINITY_DN16264_c3_g2_i1	Glyma.07G209300.1	98.07692308	394	16	295	4796	8201	3703	0	5834

TRINITY_DN16312_c0_g1_i2	Glyma.19G210700.1	100	423	42	145	4625	32	4515	0	5507

TRINITY_DN16312_c0_g1_i1	Glyma.19G210700.1	100	422	42	145	4625	32	4515	0	5513

TRINITY_DN10217_c0_g2_i3	Glyma.01G128700.1	95.65217391	478	36	110	4585	4842	373	0	5092

TRINITY_DN10217_c0_g2_i3	Glyma.01G128700.2	95.65217391	478	36	110	4585	4842	373	0	5092

TRINITY_DN16312_c0_g1_i4	Glyma.19G210700.1	100	614	52	145	4605	32	4504	0	4327

TRINITY_DN10217_c0_g2_i3	Glyma.03G041600.1	97.14285714	469	45	110	4585	4824	373	0	5125

TRINITY_DN10217_c0_g2_i3	Glyma.03G041600.2	97.14566929	469	45	110	4585	4629	178	0	5125

TRINITY_DN16312_c0_g1_i3	Glyma.19G210700.1	100	440	42	145	4614	32	4504	0	5393

TRINITY_DN15595_c0_g1_i1	Glyma.19G125600.5	100	286	7	1140	5634	4507	1	0	6650

TRINITY_DN15595_c0_g1_i1	Glyma.19G125600.4	100	286	7	1140	5634	4507	1	0	6650

TRINITY_DN15595_c0_g1_i1	Glyma.19G125600.3	95.72649573	286	8	1140	5634	4495	1	0	6573

TRINITY_DN11934_c0_g1_i1	Glyma.09G234400.1	100	261	3	474	4965	6143	1649	0	6824

TRINITY_DN11934_c0_g1_i1	Glyma.09G234400.2	99.36708861	261	3	474	4965	6143	1649	0	6824

TRINITY_DN11934_c0_g1_i1	Glyma.09G234400.3	100	261	3	474	4965	6143	1649	0	6824

TRINITY_DN11934_c0_g1_i1	Glyma.09G234400.4	100	261	3	474	4965	6143	1649	0	6824

TRINITY_DN11934_c0_g1_i1	Glyma.12G002300.3	100	248	3	474	4965	6143	1649	0	6896

TRINITY_DN11934_c0_g1_i1	Glyma.12G002300.2	96.35036496	248	3	474	4965	6143	1649	0	6896

TRINITY_DN11934_c0_g1_i1	Glyma.12G002300.1	96.35036496	248	3	474	4965	6143	1649	0	6896

TRINITY_DN8774_c0_g1_i1	Glyma.20G227200.1	100	261	20	327	4793	1	4458	0	6490

TRINITY_DN8774_c0_g1_i1	Glyma.20G227200.2	100	261	20	327	4793	1	4458	0	6490

TRINITY_DN8774_c0_g1_i2	Glyma.20G227200.1	100	261	20	327	4793	1	4458	0	6490

TRINITY_DN8774_c0_g1_i2	Glyma.20G227200.2	100	261	20	327	4793	1	4458	0	6490

TRINITY_DN8774_c0_g1_i3	Glyma.20G227200.1	95.36784741	261	20	327	4793	1	4458	0	6490

TRINITY_DN8774_c0_g1_i3	Glyma.20G227200.2	96.38554217	261	20	327	4793	1	4458	0	6490

TRINITY_DN9338_c0_g1_i1	Glyma.13G355000.2	95.31772575	686	47	240	4683	4464	9	0	4041

TRINITY_DN9338_c0_g1_i1	Glyma.13G355000.1	98.13084112	686	47	240	4683	4464	9	0	4041

TRINITY_DN16131_c0_g2_i2	Glyma.13G128700.1	97.26651481	409	6	1	4467	76	4551	0	5930

TRINITY_DN16131_c0_g2_i2	Glyma.19G170700.1	97.265625	321	4	1	4469	76	4548	0	6423

TRINITY_DN16131_c0_g2_i2	Glyma.10G041700.1	100	410	10	1	4461	76	4545	0	5891

TRINITY_DN16131_c0_g2_i2	Glyma.03G169500.2	99.2	307	8	1	4461	76	4539	0	6457

TRINITY_DN16131_c0_g2_i2	Glyma.03G169500.1	99.2	307	8	1	4461	76	4539	0	6457

TRINITY_DN16299_c0_g1_i1	Glyma.12G102400.1	99.2248062	502	20	225	4664	5804	1353	0	5208

TRINITY_DN15709_c0_g1_i1	Glyma.15G276000.1	95.58823529	338	31	136	4566	19	4455	0	5962

TRINITY_DN15657_c0_g1_i2	Glyma.03G009200.3	99.06542056	377	29	1	4454	1027	5444	0	5794

TRINITY_DN15657_c0_g1_i2	Glyma.03G009200.1	96.875	377	29	1	4454	1027	5444	0	5794

TRINITY_DN15657_c0_g1_i2	Glyma.03G009200.2	96.875	377	29	1	4454	1027	5444	0	5794

TRINITY_DN15034_c0_g1_i1	Glyma.13G297100.1	96.93094629	739	27	392	4834	5226	775	0	3947

TRINITY_DN15657_c0_g1_i2	Glyma.07G070700.1	96.55172414	387	29	1	4454	1027	5429	0	5674

TRINITY_DN15657_c0_g1_i2	Glyma.07G070700.3	97.14285714	387	29	1	4454	181	4583	0	5674

TRINITY_DN15657_c0_g1_i2	Glyma.07G070700.2	96.66666667	387	29	1	4454	181	4583	0	5674

TRINITY_DN3229_c0_g2_i1	Glyma.08G045900.1	96.51162791	256	2	82	4536	4461	1	0	6782

TRINITY_DN9002_c0_g1_i1	Glyma.07G108700.1	99.00990099	214	5	1	4452	2761	7218	0	6986

TRINITY_DN9002_c0_g1_i1	Glyma.07G108700.2	99.00990099	214	5	1	4452	2758	7215	0	6986

TRINITY_DN3229_c0_g2_i1	Glyma.05G238800.1	96.49122807	265	4	82	4536	4458	1	0	6734

TRINITY_DN9002_c0_g1_i1	Glyma.03G118400.1	100	241	3	1	4452	2851	7305	0	6839

TRINITY_DN9338_c0_g1_i1	Glyma.08G201300.1	100	293	8	240	4688	4449	1	0	6505

TRINITY_DN16299_c0_g1_i1	Glyma.06G301900.1	98.48484848	493	21	225	4664	5786	1347	0	5291

TRINITY_DN16299_c0_g1_i1	Glyma.06G301900.2	96.5034965	493	22	225	4664	5783	1347	0	5271

TRINITY_DN9338_c0_g1_i1	Glyma.07G015800.1	96.07843137	276	7	240	4688	4449	1	0	6610

TRINITY_DN13155_c0_g1_i1	Glyma.10G207500.2	100	253	5	1200	5650	5349	899	0	6763

TRINITY_DN16299_c0_g1_i1	Glyma.12G196800.1	99.23664122	603	22	224	4670	5748	1329	0	4608

TRINITY_DN13155_c0_g1_i1	Glyma.20G183400.2	100	246	2	1200	5650	5349	899	0	6824

TRINITY_DN16299_c0_g1_i1	Glyma.13G305000.1	97.6744186	606	25	224	4664	5778	1353	0	4636

TRINITY_DN16403_c1_g1_i4	Glyma.11G237800.1	100	467	11	344	4777	6660	2221	0	5496

TRINITY_DN16403_c1_g1_i3	Glyma.11G237800.1	100	395	11	323	4756	6660	2221	0	5895

TRINITY_DN16403_c1_g1_i4	Glyma.18G019600.1	100	471	9	344	4777	6405	1966	0	5485

TRINITY_DN16403_c1_g1_i3	Glyma.18G019600.1	100	400	9	323	4756	6405	1966	0	5879

TRINITY_DN8212_c0_g1_i1	Glyma.01G219800.1	100	329	11	1	4435	1057	5491	0	6279

TRINITY_DN15433_c0_g2_i1	Glyma.08G214000.1	98.55072464	371	20	766	5147	5	4434	0	5694

TRINITY_DN15433_c0_g2_i2	Glyma.08G214000.1	98.4984985	371	20	941	5322	5	4434	0	5694

TRINITY_DN8212_c0_g1_i1	Glyma.11G023700.1	100	316	6	1	4435	1057	5488	0	6353

TRINITY_DN15634_c2_g1_i2	Glyma.20G187400.1	96.92307692	453	16	237	4658	4425	1	0	5509

TRINITY_DN12946_c0_g1_i3	Glyma.13G156500.5	98.96907216	317	11	421	4841	4751	325	0	6270

TRINITY_DN16403_c1_g1_i3	Glyma.05G181600.1	100	333	0	323	4756	6456	2023	0	6344

TRINITY_DN16403_c1_g1_i4	Glyma.05G181600.1	100	269	0	344	4777	6456	2023	0	6698

TRINITY_DN16403_c1_g1_i3	Glyma.08G139300.1	100	328	0	323	4754	6456	2025	0	6368

TRINITY_DN12946_c0_g1_i2	Glyma.13G156500.5	98.96907216	317	11	421	4836	4751	325	0	6292

TRINITY_DN16403_c1_g1_i4	Glyma.08G139300.1	100	264	0	344	4775	6456	2025	0	6722

TRINITY_DN16389_c1_g1_i2	Glyma.20G144400.1	95.16129032	429	55	1806	6113	4380	1	0	4748

TRINITY_DN16117_c1_g1_i1	Glyma.03G101000.1	98.2300885	354	4	613	5021	4418	7	0	6144

TRINITY_DN16117_c1_g1_i3	Glyma.03G101000.1	100	354	4	613	5021	4418	7	0	6144

TRINITY_DN15078_c0_g1_i1	Glyma.09G267100.1	100	257	5	28	4429	4473	8886	0	6650

TRINITY_DN15078_c0_g1_i1	Glyma.18G223300.1	95.18072289	250	5	28	4429	4482	8895	0	6689

TRINITY_DN16352_c0_g2_i3	Glyma.08G194600.1	100	400	15	427	4807	4396	1	0	5727

TRINITY_DN8107_c0_g2_i1	Glyma.05G168600.1	98.21428571	593	38	533	4897	501	4868	0	4440

TRINITY_DN15078_c0_g1_i1	Glyma.03G021400.1	99.46524064	661	22	28	4397	4341	8728	0	4237

TRINITY_DN8107_c0_g2_i1	Glyma.05G168600.5	98.21428571	593	37	533	4897	501	4865	0	4455

TRINITY_DN16271_c0_g1_i1	Glyma.18G300200.2	97.66081871	247	1	27	4410	5784	1398	0	6713

TRINITY_DN16118_c0_g1_i2	Glyma.15G000300.3	95.07389163	625	18	779	5152	664	5022	0	4418

TRINITY_DN16118_c0_g1_i2	Glyma.15G000300.1	100	625	18	779	5152	664	5022	0	4418

TRINITY_DN16118_c0_g1_i2	Glyma.15G000300.2	97.6969697	625	18	779	5152	664	5022	0	4418

TRINITY_DN16352_c0_g2_i3	Glyma.07G011600.1	100	443	7	423	4784	4393	15	0	5511

TRINITY_DN16264_c3_g2_i3	Glyma.02G135700.1	100	371	12	594	4947	6871	2500	0	5814

TRINITY_DN10035_c0_g2_i1	Glyma.02G012900.1	98.16513761	334	7	1	4366	750	5124	0	6115

TRINITY_DN8107_c0_g2_i1	Glyma.05G168600.4	98.21428571	594	29	533	4897	501	4850	0	4518

TRINITY_DN16264_c3_g2_i3	Glyma.07G209300.1	98.62068966	379	16	594	4947	8053	3703	0	5644

TRINITY_DN8107_c0_g2_i1	Glyma.05G168600.2	98.21428571	594	28	533	4897	501	4847	0	4532

TRINITY_DN14527_c0_g1_i1	Glyma.19G095700.1	100	310	11	484	4824	4497	160	0	6102

TRINITY_DN14527_c0_g1_i1	Glyma.16G053500.1	95.12195122	318	9	485	4824	4502	160	0	6095

TRINITY_DN16131_c0_g2_i2	Glyma.13G128700.2	100	401	6	124	4467	1	4353	0	5747

TRINITY_DN38460_c0_g1_i1	Glyma.08G316100.1	100	452	14	145	4489	3301	7630	0	5356

TRINITY_DN38460_c0_g1_i1	Glyma.08G316100.3	100	452	14	145	4489	2206	6535	0	5356

TRINITY_DN16131_c0_g2_i2	Glyma.10G041700.3	100	402	10	124	4461	1	4347	0	5709

TRINITY_DN16131_c0_g2_i2	Glyma.10G041700.2	95.83333333	402	10	124	4461	1	4347	0	5709

TRINITY_DN16320_c0_g3_i2	Glyma.03G167800.1	97.73584906	518	35	754	5064	4619	285	0	4826

TRINITY_DN16320_c0_g3_i1	Glyma.03G167800.1	96.42857143	375	31	754	5076	4619	285	0	5696

TRINITY_DN11759_c0_g1_i2	Glyma.08G101500.1	99.17355372	421	10	282	4595	4348	4	0	5491

TRINITY_DN11759_c0_g1_i2	Glyma.05G145000.1	96.40718563	421	14	282	4595	4339	1	0	5485

TRINITY_DN16427_c0_g3_i6	Glyma.11G157400.1	100	237	2	456	4790	4914	580	0	6682

TRINITY_DN16252_c0_g1_i3	Glyma.08G293600.1	97.82608696	460	39	72	4375	13	4291	0	4939

TRINITY_DN15187_c0_g1_i3	Glyma.08G152400.1	100	329	12	540	4847	4329	1	0	6013

TRINITY_DN15231_c1_g4_i2	Glyma.03G133700.2	100	280	7	704	5023	4329	1	0	6373

TRINITY_DN15231_c1_g4_i3	Glyma.03G133700.2	97.5	237	2	704	5026	4329	1	0	6643

TRINITY_DN16252_c0_g1_i3	Glyma.08G293600.10	97.82608696	460	37	72	4375	13	4288	0	4953

TRINITY_DN16153_c0_g1_i1	Glyma.04G062400.1	96.59863946	224	12	326	4649	4315	1	0	6641

TRINITY_DN15231_c1_g4_i3	Glyma.10G106500.1	97.5	534	43	971	5271	4305	26	0	4582

TRINITY_DN16153_c0_g1_i1	Glyma.06G063400.4	97.04968944	226	10	326	4649	4309	1	0	6625

TRINITY_DN16153_c0_g1_i1	Glyma.06G063400.3	100	226	10	326	4649	4309	1	0	6625

TRINITY_DN16153_c0_g1_i1	Glyma.06G063400.1	100	226	10	326	4649	4309	1	0	6625

TRINITY_DN16153_c0_g1_i1	Glyma.04G062400.2	100	225	9	326	4649	4312	1	0	6650

TRINITY_DN16153_c0_g1_i1	Glyma.04G062400.3	96.59863946	225	9	326	4649	4312	1	0	6650

TRINITY_DN16374_c1_g1_i6	Glyma.16G035100.1	100	375	11	1	4306	343	4660	0	5753

TRINITY_DN16374_c1_g1_i7	Glyma.16G035100.1	100	375	11	1	4306	343	4660	0	5753

TRINITY_DN15231_c1_g4_i2	Glyma.10G106500.1	100	488	37	971	5268	4305	26	0	4852

TRINITY_DN16151_c0_g2_i5	Glyma.02G026700.2	95.4954955	424	35	253	4509	1	4293	0	5092

TRINITY_DN16151_c0_g2_i5	Glyma.02G026700.1	100	424	35	253	4509	1	4293	0	5092

TRINITY_DN16430_c0_g2_i6	Glyma.11G033000.1	98.14814815	393	25	392	4678	1	4272	0	5421

TRINITY_DN16151_c0_g2_i7	Glyma.02G026700.2	98.4375	233	7	318	4604	1	4293	0	6466

TRINITY_DN16151_c0_g2_i7	Glyma.02G026700.1	95.94594595	233	7	318	4604	1	4293	0	6466

TRINITY_DN16430_c0_g2_i6	Glyma.01G209200.1	98.14814815	400	17	392	4678	1	4275	0	5513

TRINITY_DN16297_c0_g1_i1	Glyma.10G245400.2	97.34042553	394	17	431	4686	1	4277	0	5435

TRINITY_DN16297_c0_g1_i1	Glyma.10G245400.3	97.34219269	394	17	431	4686	1	4277	0	5435

TRINITY_DN16297_c0_g1_i1	Glyma.10G245400.7	96.98275862	394	17	431	4686	1	4277	0	5435

TRINITY_DN16297_c0_g1_i1	Glyma.10G245400.6	97.33059548	394	17	431	4686	1	4277	0	5435

TRINITY_DN16297_c0_g1_i1	Glyma.10G245400.1	98.30508475	394	17	431	4686	1	4277	0	5435

TRINITY_DN16297_c0_g1_i1	Glyma.10G245400.5	99.21259843	394	17	431	4686	1	4277	0	5435

TRINITY_DN16297_c0_g1_i1	Glyma.10G245400.4	99.21875	394	17	431	4686	1	4277	0	5435

TRINITY_DN16297_c0_g1_i1	Glyma.20G149200.2	96.65738162	384	15	431	4688	1	4276	0	5498

TRINITY_DN16297_c0_g1_i1	Glyma.20G149200.3	97.33727811	384	15	431	4688	1	4276	0	5498

TRINITY_DN16297_c0_g1_i1	Glyma.20G149200.4	97.33840304	384	15	431	4688	1	4276	0	5498

TRINITY_DN16297_c0_g1_i1	Glyma.20G149200.1	97.33840304	384	15	431	4688	1	4276	0	5498

TRINITY_DN16397_c0_g1_i4	Glyma.19G065600.1	100	329	5	1	4281	5067	793	0	6023

TRINITY_DN16397_c0_g1_i4	Glyma.19G065600.4	100	329	5	1	4281	4923	649	0	6023

TRINITY_DN13464_c0_g1_i1	Glyma.10G295100.3	98.75311721	274	2	293	4569	1	4283	0	6353

TRINITY_DN16397_c0_g1_i3	Glyma.19G065600.1	100	480	10	166	4443	5067	793	0	5177

TRINITY_DN16397_c0_g1_i3	Glyma.19G065600.4	100	480	10	166	4443	4923	649	0	5177

TRINITY_DN16397_c0_g1_i4	Glyma.06G127400.1	100	389	2	1	4281	5460	1183	0	5731

TRINITY_DN16397_c0_g1_i4	Glyma.04G236900.1	100	379	2	1	4281	5460	1183	0	5786

TRINITY_DN15562_c1_g1_i3	Glyma.08G148300.2	96.07843137	214	6	225	4493	1	4279	0	6652

TRINITY_DN16397_c0_g1_i3	Glyma.06G127400.1	100	235	0	166	4443	5460	1183	0	6599

TRINITY_DN16397_c0_g1_i3	Glyma.04G236900.1	100	215	0	166	4443	5460	1183	0	6709

TRINITY_DN15372_c0_g1_i2	Glyma.10G049100.5	96.3060686	345	5	1	4269	402	4670	0	5895

TRINITY_DN15372_c0_g1_i2	Glyma.10G049100.4	99.00990099	345	5	1	4269	402	4670	0	5895

TRINITY_DN15372_c0_g1_i2	Glyma.10G049100.1	100	345	5	1	4269	402	4670	0	5895

TRINITY_DN15372_c0_g1_i2	Glyma.10G049100.2	97.81021898	345	5	1	4269	402	4670	0	5895

TRINITY_DN15372_c0_g1_i2	Glyma.10G049100.3	98.07692308	345	5	1	4269	402	4670	0	5895

TRINITY_DN15372_c0_g1_i2	Glyma.13G136900.1	100	350	5	1	4269	402	4673	0	5908

TRINITY_DN15372_c0_g1_i2	Glyma.13G136900.2	100	350	5	1	4269	402	4673	0	5908

TRINITY_DN8107_c0_g2_i1	Glyma.08G127200.1	98.21428571	572	25	635	4899	603	4849	0	4503

TRINITY_DN16427_c0_g3_i6	Glyma.13G195200.1	100	585	17	463	4723	4259	1	0	4494

TRINITY_DN16151_c0_g2_i3	Glyma.02G026700.2	95.53314121	429	34	34	4248	46	4293	0	5000

TRINITY_DN16151_c0_g2_i3	Glyma.02G026700.1	95.53314121	429	34	34	4248	46	4293	0	5000

TRINITY_DN11422_c0_g1_i1	Glyma.06G178200.3	96.03960396	357	9	340	4586	55	4313	0	5799

TRINITY_DN16406_c0_g1_i5	Glyma.07G125200.1	97.43589744	260	0	1	4258	345	4602	0	6423

TRINITY_DN16406_c0_g1_i4	Glyma.07G125200.1	99.58158996	249	0	1	4258	345	4602	0	6484

TRINITY_DN16406_c0_g1_i3	Glyma.07G125200.1	98.50746269	241	0	1	4258	345	4602	0	6529

TRINITY_DN14023_c0_g2_i2	Glyma.05G011900.1	95.215311	240	3	169	4422	1	4257	0	6501

TRINITY_DN14023_c0_g2_i2	Glyma.17G120100.1	95.215311	256	1	169	4422	1	4257	0	6423

TRINITY_DN15808_c0_g1_i4	Glyma.04G163600.2	98.50746269	408	28	129	4358	1	4201	0	5086

TRINITY_DN15808_c0_g1_i4	Glyma.04G163600.1	96.62162162	408	28	129	4358	1	4201	0	5086

TRINITY_DN15808_c0_g1_i4	Glyma.04G163600.3	100	408	28	129	4358	1	4201	0	5086

TRINITY_DN16023_c0_g1_i3	Glyma.18G063400.3	98.18181818	389	12	370	4596	4293	40	0	5526

TRINITY_DN15226_c0_g1_i2	Glyma.07G268200.3	96.73913043	720	57	658	4865	4	4199	0	3273

TRINITY_DN15226_c0_g1_i4	Glyma.07G268200.3	97.4137931	720	57	600	4807	4	4199	0	3273

TRINITY_DN15226_c0_g1_i5	Glyma.07G268200.3	96.41693811	720	57	610	4817	4	4199	0	3273

TRINITY_DN16151_c0_g2_i1	Glyma.02G026700.3	99.23664122	423	34	253	4440	1	4221	0	4983

TRINITY_DN15808_c0_g1_i6	Glyma.04G163600.2	98.63013699	409	23	129	4340	1	4201	0	5164

TRINITY_DN15808_c0_g1_i6	Glyma.04G163600.1	100	409	23	129	4340	1	4201	0	5164

TRINITY_DN15808_c0_g1_i6	Glyma.04G163600.3	100	409	23	129	4340	1	4201	0	5164

TRINITY_DN16433_c0_g2_i2	Glyma.11G221100.1	95.07042254	334	7	145	4374	1	4233	0	5925

TRINITY_DN16151_c0_g2_i4	Glyma.02G026700.3	100	232	6	318	4535	1	4221	0	6357

TRINITY_DN16283_c0_g2_i3	Glyma.19G171200.1	96.57657658	451	19	264	4459	4335	122	0	5079

TRINITY_DN16283_c0_g2_i4	Glyma.19G171200.1	95.86206897	339	21	264	4459	4335	122	0	5699

TRINITY_DN16312_c0_g1_i1	Glyma.10G019000.2	100	702	30	437	4625	300	4506	0	3609

TRINITY_DN16312_c0_g1_i1	Glyma.10G019000.1	100	702	30	437	4625	300	4506	0	3609

TRINITY_DN16312_c0_g1_i2	Glyma.10G019000.2	100	701	30	437	4625	300	4506	0	3615

TRINITY_DN16312_c0_g1_i2	Glyma.10G019000.1	100	701	30	437	4625	300	4506	0	3615

TRINITY_DN16312_c0_g1_i3	Glyma.10G019000.2	100	687	30	437	4625	300	4506	0	3692

TRINITY_DN16312_c0_g1_i3	Glyma.10G019000.1	100	687	30	437	4625	300	4506	0	3692

TRINITY_DN16283_c0_g2_i3	Glyma.03G170000.1	95.91836735	454	19	264	4459	4323	122	0	5007

TRINITY_DN16283_c0_g2_i4	Glyma.03G170000.1	97.33333333	346	18	264	4459	4323	122	0	5605

TRINITY_DN16312_c0_g1_i4	Glyma.10G019000.2	100	505	31	437	4616	300	4506	0	4663

TRINITY_DN16312_c0_g1_i4	Glyma.10G019000.1	100	505	31	437	4616	300	4506	0	4663

TRINITY_DN15372_c0_g1_i1	Glyma.10G049100.5	95.79831933	343	5	1	4208	402	4609	0	5794

TRINITY_DN15372_c0_g1_i1	Glyma.10G049100.4	99.31972789	343	5	1	4208	402	4609	0	5794

TRINITY_DN15372_c0_g1_i1	Glyma.10G049100.1	97.95918367	343	5	1	4208	402	4609	0	5794

TRINITY_DN15372_c0_g1_i1	Glyma.10G049100.2	95.35211268	343	5	1	4208	402	4609	0	5794

TRINITY_DN15372_c0_g1_i1	Glyma.10G049100.3	96.93251534	343	5	1	4208	402	4609	0	5794

TRINITY_DN15372_c0_g1_i1	Glyma.13G136900.1	97.6744186	346	5	1	4208	402	4612	0	5818

TRINITY_DN15372_c0_g1_i1	Glyma.13G136900.2	99.04534606	346	5	1	4208	402	4612	0	5818

TRINITY_DN8774_c0_g1_i1	Glyma.20G227200.3	100	241	20	610	4793	188	4362	0	6078

TRINITY_DN8774_c0_g1_i2	Glyma.20G227200.3	96.27403846	241	20	610	4793	188	4362	0	6078

TRINITY_DN8774_c0_g1_i3	Glyma.20G227200.3	96.38554217	241	20	610	4793	188	4362	0	6078

TRINITY_DN14159_c0_g1_i1	Glyma.16G004800.1	100	462	27	180	4363	16236	12065	0	4913

TRINITY_DN14159_c0_g1_i1	Glyma.16G004800.3	95.04950495	462	27	180	4363	16221	12050	0	4913

TRINITY_DN14159_c0_g1_i1	Glyma.16G004800.2	100	462	27	180	4363	15939	11768	0	4913

TRINITY_DN5704_c0_g1_i1	Glyma.07G037400.2	98.14814815	273	23	44	4210	4167	1	0	5851

TRINITY_DN5704_c0_g1_i1	Glyma.07G037400.1	95.55555556	273	23	44	4210	4167	1	0	5851

TRINITY_DN5704_c0_g2_i1	Glyma.07G037400.2	96.46017699	273	23	580	4746	4167	1	0	5851

TRINITY_DN5704_c0_g2_i1	Glyma.07G037400.1	98.14814815	273	23	580	4746	4167	1	0	5851

TRINITY_DN5704_c0_g1_i1	Glyma.16G006800.1	98.14814815	260	18	44	4210	4164	1	0	5903

TRINITY_DN5704_c0_g2_i1	Glyma.16G006800.1	100	260	18	580	4746	4164	1	0	5903

TRINITY_DN16283_c0_g2_i4	Glyma.13G130000.1	100	406	11	264	4459	4287	122	0	5306

TRINITY_DN16283_c0_g2_i3	Glyma.13G130000.1	98.60627178	299	10	264	4459	4287	122	0	5899

TRINITY_DN16283_c0_g2_i4	Glyma.10G042600.1	100	423	6	264	4457	4311	124	0	5352

TRINITY_DN16283_c0_g2_i3	Glyma.10G042600.1	95.91078067	313	5	264	4457	4311	124	0	5962

TRINITY_DN2769_c0_g1_i1	Glyma.20G150800.1	100	247	5	132	4319	4980	800	0	6253

TRINITY_DN2769_c0_g1_i1	Glyma.10G243400.1	95.35353535	246	7	132	4319	4983	803	0	6259

TRINITY_DN2769_c0_g1_i1	Glyma.10G243400.2	96.47749511	246	9	132	4319	4974	803	0	6202

TRINITY_DN16169_c0_g1_i3	Glyma.10G292400.3	100	424	19	513	4676	4155	1	0	4983

TRINITY_DN16169_c0_g1_i3	Glyma.10G292400.1	97.76536313	424	19	513	4676	4155	1	0	4983

TRINITY_DN16447_c1_g1_i6	Glyma.08G162300.1	99.00990099	413	32	934	5055	1995	6164	0	4926

TRINITY_DN16447_c1_g1_i7	Glyma.08G162300.1	97.9020979	413	33	1168	5286	1995	6164	0	4905

TRINITY_DN16447_c1_g1_i6	Glyma.08G162300.2	99.13043478	412	33	934	5055	1995	6161	0	4911

TRINITY_DN16447_c1_g1_i7	Glyma.08G162300.2	97.2826087	412	34	1168	5286	1995	6161	0	4892

TRINITY_DN16430_c0_g2_i5	Glyma.11G033000.1	98.57142857	383	23	46	4206	118	4272	0	5302

TRINITY_DN2769_c0_g2_i1	Glyma.20G150800.1	99.14529915	248	2	132	4306	4980	800	0	6309

TRINITY_DN2769_c0_g2_i1	Glyma.10G243400.1	98.21428571	247	2	132	4306	4983	803	0	6314

TRINITY_DN2769_c0_g2_i1	Glyma.10G243400.2	99.49811794	247	4	132	4306	4974	803	0	6255

TRINITY_DN16169_c0_g1_i3	Glyma.20G242400.1	99.06542056	421	18	513	4676	4137	1	0	5027

TRINITY_DN16169_c0_g1_i3	Glyma.20G242400.2	100	421	18	513	4676	4137	1	0	5027

TRINITY_DN16447_c1_g1_i7	Glyma.15G265300.1	97.46192893	407	26	1168	5286	1992	6152	0	4992

TRINITY_DN16447_c1_g1_i7	Glyma.15G265300.2	99.7716895	407	26	1168	5286	1914	6074	0	4992

TRINITY_DN16447_c1_g1_i7	Glyma.15G265300.3	96.19883041	407	26	1168	5286	1068	5228	0	4992

TRINITY_DN16447_c1_g1_i6	Glyma.15G265300.1	100	406	25	934	5055	1992	6152	0	5016

TRINITY_DN16447_c1_g1_i6	Glyma.15G265300.2	95.54317549	406	25	934	5055	1914	6074	0	5016

TRINITY_DN16447_c1_g1_i6	Glyma.15G265300.3	97.10144928	406	25	934	5055	1068	5228	0	5016

TRINITY_DN16369_c0_g1_i3	Glyma.06G003600.2	100	594	31	882	5002	1	4166	0	4012

TRINITY_DN16128_c0_g1_i2	Glyma.15G043500.1	97.82608696	341	18	521	4675	4161	1	0	5602

TRINITY_DN16128_c0_g1_i3	Glyma.15G043500.1	97.82608696	341	18	849	5003	4161	1	0	5602

TRINITY_DN16447_c1_g1_i7	Glyma.15G265300.4	100	407	26	1170	5286	14	4172	0	4988

TRINITY_DN16447_c1_g1_i6	Glyma.15G265300.4	96.47058824	406	25	936	5055	14	4172	0	5012

TRINITY_DN7170_c0_g1_i1	Glyma.03G247300.1	96.22641509	257	13	97	4249	1281	5445	0	6091

TRINITY_DN7170_c0_g2_i1	Glyma.03G247300.1	100	257	13	97	4249	1281	5445	0	6091

TRINITY_DN7170_c0_g1_i1	Glyma.19G245100.1	97.91666667	248	15	97	4249	1281	5421	0	5986

TRINITY_DN7170_c0_g2_i1	Glyma.19G245100.1	95.52845528	248	15	97	4249	1281	5421	0	5986

TRINITY_DN16369_c0_g1_i1	Glyma.06G003600.2	100	469	14	882	5035	1	4166	0	4940

TRINITY_DN16430_c0_g2_i6	Glyma.11G032900.1	98.74476987	377	18	392	4549	1	4143	0	5337

TRINITY_DN16430_c0_g2_i6	Glyma.11G032900.2	98.14814815	377	18	392	4549	1	4143	0	5337

TRINITY_DN16430_c0_g2_i5	Glyma.01G209200.1	100	391	12	46	4206	118	4275	0	5387

TRINITY_DN16305_c0_g1_i1	Glyma.20G019300.1	100	179	3	63	4229	1	4167	0	6671

TRINITY_DN16169_c0_g1_i2	Glyma.10G292400.3	95.625	424	19	513	4654	4155	23	0	4942

TRINITY_DN16169_c0_g1_i2	Glyma.10G292400.1	95.62563581	424	19	513	4654	4155	23	0	4942

TRINITY_DN10905_c0_g2_i1	Glyma.08G157400.1	100	258	1	1	4165	5230	1063	0	6248

TRINITY_DN10905_c0_g2_i1	Glyma.15G268800.5	100	245	1	1	4165	5230	1063	0	6320

TRINITY_DN10905_c0_g2_i1	Glyma.15G268800.4	100	245	1	1	4165	5230	1063	0	6320

TRINITY_DN10905_c0_g2_i1	Glyma.15G268800.3	100	245	1	1	4165	5230	1063	0	6320

TRINITY_DN10905_c0_g2_i1	Glyma.15G268800.1	100	245	1	1	4165	5230	1063	0	6320

TRINITY_DN10905_c0_g2_i1	Glyma.15G268800.2	100	245	1	1	4165	5230	1063	0	6320

TRINITY_DN10905_c0_g2_i1	Glyma.15G268800.6	100	245	1	1	4165	5230	1063	0	6320

TRINITY_DN10085_c0_g1_i1	Glyma.05G013400.1	100	272	4	150	4300	1	4160	0	6083

TRINITY_DN10085_c0_g1_i1	Glyma.17G121600.1	100	257	6	150	4300	1	4160	0	6167

TRINITY_DN10864_c0_g1_i2	Glyma.13G057300.1	97.70114943	281	7	977	5136	4856	724	0	5939

TRINITY_DN10864_c0_g1_i3	Glyma.13G057300.1	99.35483871	281	7	887	5046	4856	724	0	5939

TRINITY_DN10905_c0_g1_i1	Glyma.08G157400.1	96.58703072	257	1	6	4162	5222	1063	0	6239

TRINITY_DN10905_c0_g1_i1	Glyma.15G268800.5	97.59036145	244	1	6	4162	5222	1063	0	6311

TRINITY_DN10905_c0_g1_i1	Glyma.15G268800.4	95.35232384	244	1	6	4162	5222	1063	0	6311

TRINITY_DN10905_c0_g1_i1	Glyma.15G268800.3	99.31192661	244	1	6	4162	5222	1063	0	6311

TRINITY_DN10905_c0_g1_i1	Glyma.15G268800.1	98.7654321	244	1	6	4162	5222	1063	0	6311

TRINITY_DN10905_c0_g1_i1	Glyma.15G268800.2	98.7654321	244	1	6	4162	5222	1063	0	6311

TRINITY_DN10905_c0_g1_i1	Glyma.15G268800.6	96.58634538	244	1	6	4162	5222	1063	0	6311

TRINITY_DN16330_c0_g1_i4	Glyma.10G097800.1	95.18072289	329	9	243	4394	1	4155	0	5786

TRINITY_DN16330_c0_g1_i1	Glyma.03G137000.1	95.16908213	483	10	195	4343	1	4155	0	4926

TRINITY_DN16330_c0_g1_i1	Glyma.19G139300.1	95.18072289	476	11	195	4343	1	4152	0	4944

TRINITY_DN16320_c0_g3_i1	Glyma.13G127500.2	96.42857143	370	14	1169	5312	4138	1	0	5409

TRINITY_DN16169_c0_g1_i2	Glyma.20G242400.1	99.00990099	421	18	513	4654	4137	23	0	4987

TRINITY_DN16169_c0_g1_i2	Glyma.20G242400.2	96.42857143	421	18	513	4654	4137	23	0	4987

TRINITY_DN16330_c0_g1_i4	Glyma.03G137000.1	95.16129032	417	2	243	4394	1	4155	0	5343

TRINITY_DN16330_c0_g1_i1	Glyma.10G097800.1	95.18518519	279	2	195	4343	1	4155	0	6089

TRINITY_DN16406_c0_g1_i5	Glyma.01G006600.1	97.43589744	256	0	1	4154	345	4498	0	6253

TRINITY_DN16406_c0_g1_i4	Glyma.01G006600.1	97.42857143	245	0	1	4154	345	4498	0	6314

TRINITY_DN16406_c0_g1_i3	Glyma.01G006600.1	98.63013699	241	0	1	4154	345	4498	0	6336

TRINITY_DN38460_c0_g1_i1	Glyma.08G316100.2	100	436	14	145	4291	3301	7432	0	5079

TRINITY_DN16330_c0_g1_i4	Glyma.19G139300.1	95.16129032	413	0	243	4394	1	4152	0	5380

TRINITY_DN16320_c0_g3_i2	Glyma.13G127500.2	96.42857143	250	9	1169	5300	4138	1	0	6141

TRINITY_DN16352_c0_g2_i3	Glyma.08G190700.1	100	611	38	427	4523	4381	246	0	3912

TRINITY_DN16156_c0_g1_i2	Glyma.09G268400.1	100	400	6	271	4411	5355	1215	0	5398

TRINITY_DN16156_c0_g1_i4	Glyma.09G268400.1	96.92307692	300	6	271	4411	5355	1215	0	5952

TRINITY_DN16156_c0_g1_i4	Glyma.18G221200.1	96.92307692	433	7	270	4411	5350	1218	0	5180

TRINITY_DN16156_c0_g1_i4	Glyma.03G022900.1	96.92307692	425	12	271	4411	5349	1221	0	5193

TRINITY_DN13694_c0_g1_i2	Glyma.07G103000.3	96.96969697	270	1	102	4244	1	4131	0	6078

TRINITY_DN13694_c0_g1_i2	Glyma.07G103000.1	96.96969697	270	1	102	4244	1	4131	0	6078

TRINITY_DN13694_c0_g1_i2	Glyma.07G103000.2	98.50746269	270	1	102	4244	1	4131	0	6078

TRINITY_DN16156_c0_g1_i2	Glyma.18G221200.1	98	364	5	270	4411	5350	1218	0	5574

TRINITY_DN16156_c0_g1_i2	Glyma.03G022900.1	100	363	7	271	4411	5349	1221	0	5559

TRINITY_DN12789_c0_g1_i1	Glyma.11G061700.5	96.71361502	207	1	220	4353	4140	1	0	6460

TRINITY_DN2553_c0_g1_i1	Glyma.09G282200.1	98.83040936	281	5	249	4381	14283	10154	0	5989

TRINITY_DN2553_c0_g1_i1	Glyma.09G282200.2	100	281	5	249	4381	13740	9611	0	5989

TRINITY_DN2553_c0_g1_i1	Glyma.09G282200.3	99.35483871	280	7	249	4381	14277	10154	0	5956

TRINITY_DN15657_c0_g1_i2	Glyma.09G259400.1	98.24561404	579	33	349	4463	1426	5517	0	4043

TRINITY_DN15657_c0_g1_i2	Glyma.09G259400.2	97.86856128	579	33	349	4463	1426	5517	0	4043

TRINITY_DN15657_c0_g1_i2	Glyma.09G259400.3	97.87234043	579	33	349	4463	1426	5517	0	4043

TRINITY_DN13968_c0_g3_i2	Glyma.07G192500.1	97.16024341	469	41	223	4314	1	4080	0	4427

TRINITY_DN2553_c0_g1_i1	Glyma.20G003400.1	99.25373134	293	4	249	4381	14277	10151	0	5971

TRINITY_DN16252_c0_g1_i1	Glyma.08G293600.1	98.47715736	441	27	72	4180	13	4114	0	4844

TRINITY_DN13968_c0_g3_i3	Glyma.07G192500.1	96.40718563	547	49	223	4308	1	4080	0	3978

TRINITY_DN13694_c0_g1_i1	Glyma.07G103000.3	95.8677686	270	0	102	4232	1	4131	0	6133

TRINITY_DN13694_c0_g1_i1	Glyma.07G103000.1	96.82539683	270	0	102	4232	1	4131	0	6133

TRINITY_DN13694_c0_g1_i1	Glyma.07G103000.2	95.09202454	270	0	102	4232	1	4131	0	6133

TRINITY_DN15657_c0_g1_i2	Glyma.18G232900.3	98.71794872	585	27	349	4460	1426	5533	0	4128

TRINITY_DN15657_c0_g1_i2	Glyma.18G232900.1	98.24561404	585	27	349	4460	1426	5533	0	4128

TRINITY_DN15657_c0_g1_i2	Glyma.18G232900.2	97.5308642	585	27	349	4460	1366	5473	0	4128

TRINITY_DN13968_c0_g3_i2	Glyma.07G192500.3	98.30508475	471	39	223	4314	1	4077	0	4429

TRINITY_DN16252_c0_g1_i1	Glyma.08G293600.10	96.64031621	441	25	72	4180	13	4111	0	4859

TRINITY_DN13968_c0_g3_i3	Glyma.07G192500.3	96.40718563	549	47	223	4308	1	4077	0	3980

TRINITY_DN13968_c0_g3_i4	Glyma.07G192500.1	96.07843137	395	31	223	4317	1	4080	0	4944

TRINITY_DN13968_c0_g3_i1	Glyma.07G192500.1	100	473	36	223	4311	1	4080	0	4495

TRINITY_DN14144_c0_g1_i1	Glyma.19G204200.2	95.38461538	289	8	156	4256	1	4122	0	5864

TRINITY_DN13968_c0_g3_i4	Glyma.07G192500.3	96.07843137	397	29	223	4317	1	4077	0	4948

TRINITY_DN13968_c0_g3_i1	Glyma.07G192500.3	100	475	34	223	4311	1	4077	0	4499

TRINITY_DN13968_c0_g3_i2	Glyma.13G183900.5	100	474	34	223	4314	1	4071	0	4484

TRINITY_DN13968_c0_g3_i3	Glyma.13G183900.5	96.40635798	547	43	223	4308	1	4071	0	4052

TRINITY_DN13968_c0_g3_i4	Glyma.13G183900.5	96.07843137	389	30	223	4317	1	4071	0	5009

TRINITY_DN16397_c0_g1_i5	Glyma.14G162300.1	98.16513761	378	6	2168	6282	4985	874	0	5463

TRINITY_DN16397_c0_g1_i6	Glyma.14G162300.1	98.14814815	345	6	2168	6282	4985	874	0	5646

TRINITY_DN13968_c0_g3_i1	Glyma.13G183900.5	100	462	35	223	4311	1	4071	0	4577

TRINITY_DN13968_c0_g3_i2	Glyma.07G192500.2	100	466	35	223	4314	1	4041	0	4457

TRINITY_DN13968_c0_g3_i3	Glyma.07G192500.2	96.40718563	544	43	223	4308	1	4041	0	4008

TRINITY_DN15627_c0_g1_i1	Glyma.01G184500.1	95.74468085	530	29	5	4099	805	4869	0	4311

TRINITY_DN15627_c0_g1_i1	Glyma.17G176300.1	95.74468085	239	3	1	4099	765	4869	0	6218

TRINITY_DN15627_c0_g1_i1	Glyma.05G090500.1	100	235	3	1	4099	765	4869	0	6240

TRINITY_DN10114_c0_g1_i1	Glyma.15G092400.6	97.31800766	269	2	96	4196	1	4101	0	6050

TRINITY_DN13968_c0_g3_i4	Glyma.07G192500.2	96.07843137	392	25	223	4317	1	4041	0	4975

TRINITY_DN13968_c0_g3_i1	Glyma.07G192500.2	99.35064935	470	30	223	4311	1	4041	0	4527

TRINITY_DN14724_c1_g1_i1	Glyma.19G121300.1	96.93251534	353	38	377	4447	4029	1	0	5059

TRINITY_DN16428_c1_g1_i3	Glyma.09G278500.1	96.72131148	182	19	510	4573	4122	47	0	6264

TRINITY_DN5829_c0_g1_i1	Glyma.05G241400.2	96.875	272	8	2114	6190	4083	1	0	5962

TRINITY_DN12896_c0_g1_i1	Glyma.15G122400.1	95.83333333	279	34	226	4256	1	4067	0	5533

TRINITY_DN10732_c0_g1_i2	Glyma.13G340300.1	100	240	6	226	4308	4077	1	0	6161

TRINITY_DN16406_c0_g1_i5	Glyma.07G125200.2	97.86585366	249	0	1	4082	345	4426	0	6159

TRINITY_DN16406_c0_g1_i3	Glyma.07G125200.2	100	230	0	1	4082	345	4426	0	6264

TRINITY_DN16406_c0_g1_i4	Glyma.07G125200.2	97.42990654	230	0	1	4082	345	4426	0	6264

TRINITY_DN14144_c0_g1_i1	Glyma.19G204200.1	95.38461538	287	8	156	4214	1	4080	0	5797

TRINITY_DN14144_c0_g1_i1	Glyma.03G206600.1	100	261	10	156	4214	1	4080	0	5941

TRINITY_DN14877_c0_g1_i1	Glyma.19G016300.1	98.27586207	256	8	300	4361	4206	133	0	5962

TRINITY_DN14877_c0_g1_i1	Glyma.19G016300.2	100	256	7	300	4361	4203	133	0	5975

TRINITY_DN15562_c1_g1_i1	Glyma.08G148300.1	98.51851852	199	1	587	4661	1433	5508	0	6418

TRINITY_DN15562_c1_g1_i1	Glyma.15G212800.1	97.95918367	189	1	587	4661	1433	5508	0	6473

TRINITY_DN15562_c1_g1_i1	Glyma.15G212800.2	96	189	1	587	4661	632	4707	0	6473

TRINITY_DN14084_c0_g1_i1	Glyma.11G096400.1	99.45945946	363	2	446	4519	4074	1	0	5502

TRINITY_DN14084_c0_g1_i1	Glyma.12G022500.2	99.45945946	354	2	446	4519	4074	1	0	5552

TRINITY_DN14084_c0_g1_i1	Glyma.12G022500.1	99.45945946	354	2	446	4519	4074	1	0	5552

TRINITY_DN16428_c1_g1_i3	Glyma.18G210300.1	96.27192982	218	13	510	4573	4098	47	0	6131

TRINITY_DN13968_c0_g3_i2	Glyma.13G183900.1	95.38461538	469	34	223	4261	1	4018	0	4414

TRINITY_DN13968_c0_g3_i3	Glyma.13G183900.1	96.40718563	540	43	223	4255	1	4018	0	3993

TRINITY_DN13968_c0_g3_i4	Glyma.13G183900.1	96.07843137	384	30	223	4264	1	4018	0	4939

TRINITY_DN13968_c0_g3_i1	Glyma.13G183900.1	97.64705882	455	35	223	4258	1	4018	0	4518

TRINITY_DN16212_c0_g1_i2	Glyma.03G183600.1	98.61751152	317	15	269	4300	4029	1	0	5459

TRINITY_DN16212_c0_g1_i3	Glyma.03G183600.1	95.09803922	259	13	269	4300	4029	1	0	5792

TRINITY_DN16441_c4_g2_i4	Glyma.13G235300.1	95	297	10	601	4640	5268	1229	0	5738

TRINITY_DN16441_c4_g2_i4	Glyma.13G235300.3	96.58119658	297	10	601	4640	4488	449	0	5738

TRINITY_DN16441_c4_g2_i4	Glyma.13G235300.2	98.8372093	297	10	601	4640	4488	449	0	5738

TRINITY_DN16430_c0_g2_i5	Glyma.11G032900.1	100	367	13	46	4077	118	4143	0	5219

TRINITY_DN16430_c0_g2_i5	Glyma.11G032900.2	98.80668258	367	13	46	4077	118	4143	0	5219

TRINITY_DN16212_c0_g1_i2	Glyma.19G184300.1	98.61591696	313	19	269	4300	4020	1	0	5480

TRINITY_DN16212_c0_g1_i3	Glyma.19G184300.1	98.15384615	258	17	269	4300	4020	1	0	5795

TRINITY_DN13547_c0_g1_i2	Glyma.16G013500.1	98.05194805	302	16	356	4366	1	4026	0	5553

TRINITY_DN13547_c0_g1_i2	Glyma.16G013500.2	98.03921569	302	16	356	4366	1	4026	0	5553

TRINITY_DN13547_c0_g1_i2	Glyma.16G013500.3	98.04964539	302	16	356	4366	1	4026	0	5553

TRINITY_DN15235_c0_g1_i4	Glyma.10G227400.6	100	314	4	4	4032	412	4440	0	5679

TRINITY_DN15235_c0_g1_i4	Glyma.10G227400.4	100	314	4	4	4032	412	4440	0	5679

TRINITY_DN15235_c0_g1_i4	Glyma.10G227400.3	100	314	4	4	4032	412	4440	0	5679

TRINITY_DN15235_c0_g1_i4	Glyma.10G227400.1	95.91836735	314	4	4	4032	412	4440	0	5679

TRINITY_DN15235_c0_g1_i4	Glyma.10G227400.2	100	314	4	4	4032	412	4440	0	5679

TRINITY_DN15235_c0_g1_i4	Glyma.10G227400.5	98.8372093	314	4	4	4032	412	4440	0	5679

TRINITY_DN16266_c0_g2_i2	Glyma.02G094800.1	96.25	231	11	99	4115	181	4209	0	6061

TRINITY_DN15235_c0_g1_i2	Glyma.10G227400.6	96.21212121	315	4	4	4031	412	4439	0	5672

TRINITY_DN15235_c0_g1_i2	Glyma.10G227400.4	100	315	4	4	4031	412	4439	0	5672

TRINITY_DN15235_c0_g1_i2	Glyma.10G227400.3	95.67567568	315	4	4	4031	412	4439	0	5672

TRINITY_DN15235_c0_g1_i2	Glyma.10G227400.1	95.67567568	315	4	4	4031	412	4439	0	5672

TRINITY_DN15235_c0_g1_i2	Glyma.10G227400.2	96	315	4	4	4031	412	4439	0	5672

TRINITY_DN15235_c0_g1_i2	Glyma.10G227400.5	96.86028257	315	4	4	4031	412	4439	0	5672

TRINITY_DN16266_c0_g2_i2	Glyma.18G286600.1	100	233	9	99	4115	181	4209	0	6061

TRINITY_DN16297_c0_g2_i1	Glyma.11G006800.1	97.01492537	364	20	308	4318	1	4008	0	5171

TRINITY_DN16297_c0_g2_i1	Glyma.11G006800.2	100	364	20	308	4318	1	4008	0	5171

TRINITY_DN16422_c0_g2_i6	Glyma.01G238200.1	97.56097561	407	32	343	4351	1	3960	0	4665

TRINITY_DN16422_c0_g2_i7	Glyma.01G238200.1	98.63013699	407	32	344	4352	1	3960	0	4665

TRINITY_DN16422_c0_g2_i4	Glyma.01G238200.1	99.54954955	391	35	344	4351	1	3960	0	4748

TRINITY_DN16422_c0_g2_i5	Glyma.01G238200.1	96.03960396	391	35	343	4350	1	3960	0	4748

TRINITY_DN16422_c0_g2_i4	Glyma.01G238200.2	98.42519685	389	36	344	4351	1	3945	0	4662

TRINITY_DN16422_c0_g2_i5	Glyma.01G238200.2	100	389	36	343	4350	1	3945	0	4662

TRINITY_DN16422_c0_g2_i1	Glyma.01G238200.1	99.38271605	406	31	344	4351	1	3960	0	4676

TRINITY_DN16422_c0_g2_i2	Glyma.01G238200.1	98.22695035	406	31	343	4350	1	3960	0	4676

TRINITY_DN16266_c0_g2_i2	Glyma.20G239800.1	97.5308642	405	5	99	4115	181	4200	0	5138

TRINITY_DN16422_c0_g2_i6	Glyma.01G238200.2	100	410	40	343	4351	1	3945	0	4586

TRINITY_DN16422_c0_g2_i7	Glyma.01G238200.2	98.63013699	410	40	344	4352	1	3945	0	4586

TRINITY_DN16297_c0_g2_i1	Glyma.01G236400.1	97.90026247	353	5	308	4318	1	4017	0	5380

TRINITY_DN16285_c1_g1_i2	Glyma.05G178900.4	99.26470588	299	9	633	4646	1	4017	0	5698

TRINITY_DN16285_c1_g1_i2	Glyma.05G178900.1	99.44134078	299	9	633	4646	1	4017	0	5698

TRINITY_DN16285_c1_g1_i2	Glyma.05G178900.2	100	299	9	633	4646	1	4017	0	5698

TRINITY_DN16422_c0_g2_i1	Glyma.01G238200.2	99.38271605	409	39	344	4351	1	3945	0	4595

TRINITY_DN16422_c0_g2_i2	Glyma.01G238200.2	98.22695035	409	39	343	4350	1	3945	0	4595

TRINITY_DN14563_c0_g1_i5	Glyma.18G218900.1	100	342	33	349	4338	3999	19	0	5083

TRINITY_DN16285_c1_g1_i1	Glyma.11G239800.2	97.34513274	500	13	633	4646	1	4011	0	4567

TRINITY_DN16285_c1_g1_i2	Glyma.11G239800.2	97.92060491	412	13	633	4646	1	4011	0	5055

TRINITY_DN16285_c1_g1_i1	Glyma.05G178900.4	97.5	230	5	633	4646	1	4017	0	6102

TRINITY_DN16285_c1_g1_i1	Glyma.05G178900.1	95.0310559	230	5	633	4646	1	4017	0	6102

TRINITY_DN16285_c1_g1_i1	Glyma.05G178900.2	95.6043956	230	5	633	4646	1	4017	0	6102

TRINITY_DN16285_c1_g1_i1	Glyma.08G136400.2	100	218	5	633	4646	1	4017	0	6168

TRINITY_DN16285_c1_g1_i1	Glyma.08G136400.3	97.70114943	218	5	633	4646	1	4017	0	6168

TRINITY_DN16285_c1_g1_i1	Glyma.08G136400.1	96	218	5	633	4646	1	4017	0	6168

TRINITY_DN16285_c1_g1_i2	Glyma.08G136400.2	100	287	4	633	4646	1	4017	0	5797

TRINITY_DN16285_c1_g1_i2	Glyma.08G136400.3	95.23809524	287	4	633	4646	1	4017	0	5797

TRINITY_DN16285_c1_g1_i2	Glyma.08G136400.1	100	287	4	633	4646	1	4017	0	5797

TRINITY_DN16266_c0_g2_i2	Glyma.10G290800.1	98.98989899	413	5	99	4110	181	4195	0	5084

TRINITY_DN15235_c0_g1_i4	Glyma.16G172600.1	100	737	25	34	4032	448	4446	0	3158

TRINITY_DN15235_c0_g1_i4	Glyma.16G172400.1	95.89041096	732	25	34	4032	517	4512	0	3166

TRINITY_DN15235_c0_g1_i4	Glyma.16G172400.4	95.89041096	732	25	34	4032	475	4470	0	3166

TRINITY_DN15235_c0_g1_i4	Glyma.16G172400.3	95.89041096	732	25	34	4032	448	4443	0	3166

TRINITY_DN15235_c0_g1_i2	Glyma.16G172600.1	98.36065574	738	25	34	4031	448	4445	0	3151

TRINITY_DN15235_c0_g1_i2	Glyma.16G172400.1	96.52605459	733	25	34	4031	517	4511	0	3158

TRINITY_DN15235_c0_g1_i2	Glyma.16G172400.4	96.52509653	733	25	34	4031	475	4469	0	3158

TRINITY_DN15235_c0_g1_i2	Glyma.16G172400.3	95.51495017	733	25	34	4031	448	4442	0	3158

TRINITY_DN11189_c0_g1_i1	Glyma.20G000700.2	100	196	12	669	4647	3	3990	0	5987

TRINITY_DN6467_c0_g1_i1	Glyma.02G174100.1	100	273	8	1	4000	2127	6126	0	5797

TRINITY_DN6467_c0_g2_i1	Glyma.02G174100.1	95.85253456	273	8	1	4000	2127	6126	0	5797

TRINITY_DN6467_c0_g1_i1	Glyma.02G174100.3	100	273	8	1	4000	2124	6123	0	5797

TRINITY_DN6467_c0_g2_i1	Glyma.02G174100.3	98.64864865	273	8	1	4000	2124	6123	0	5797

TRINITY_DN6467_c0_g1_i1	Glyma.02G174100.2	100	273	8	1	4000	2055	6054	0	5797

TRINITY_DN6467_c0_g2_i1	Glyma.02G174100.2	97.98319328	273	8	1	4000	2055	6054	0	5797

TRINITY_DN6467_c0_g1_i1	Glyma.09G112200.1	96.15384615	284	7	2	4000	2152	6150	0	5734

TRINITY_DN6467_c0_g1_i1	Glyma.09G112200.5	98.14814815	284	7	2	4000	2152	6150	0	5734

TRINITY_DN6467_c0_g2_i1	Glyma.09G112200.1	97.63593381	284	7	2	4000	2152	6150	0	5734

TRINITY_DN6467_c0_g2_i1	Glyma.09G112200.5	97.63779528	284	7	2	4000	2152	6150	0	5734

TRINITY_DN6467_c0_g1_i1	Glyma.09G112200.4	96.15384615	284	7	2	4000	2140	6138	0	5734

TRINITY_DN6467_c0_g2_i1	Glyma.09G112200.4	97.63513514	284	7	2	4000	2140	6138	0	5734

TRINITY_DN6467_c0_g1_i1	Glyma.09G112200.3	98.7012987	284	7	2	4000	2131	6129	0	5734

TRINITY_DN6467_c0_g2_i1	Glyma.09G112200.3	99.21259843	284	7	2	4000	2131	6129	0	5734

TRINITY_DN6467_c0_g1_i1	Glyma.09G112200.2	100	284	7	2	4000	2119	6117	0	5734

TRINITY_DN6467_c0_g2_i1	Glyma.09G112200.2	96.55172414	284	7	2	4000	2119	6117	0	5734

TRINITY_DN15235_c0_g1_i4	Glyma.10G227300.5	95.89041096	597	6	34	4030	454	4450	0	4030

TRINITY_DN15235_c0_g1_i4	Glyma.10G227300.4	95.89041096	597	6	34	4030	454	4450	0	4030

TRINITY_DN15235_c0_g1_i4	Glyma.10G227300.3	95.89041096	597	6	34	4030	454	4450	0	4030

TRINITY_DN15235_c0_g1_i4	Glyma.10G227300.1	95.89041096	597	6	34	4030	454	4450	0	4030

TRINITY_DN15235_c0_g1_i4	Glyma.10G227300.2	95.89041096	597	6	34	4030	454	4450	0	4030

TRINITY_DN15235_c0_g1_i2	Glyma.10G227300.5	96.33333333	596	6	34	4030	454	4450	0	4036

TRINITY_DN15235_c0_g1_i2	Glyma.10G227300.4	100	596	6	34	4030	454	4450	0	4036

TRINITY_DN15235_c0_g1_i2	Glyma.10G227300.3	95.36231884	596	6	34	4030	454	4450	0	4036

TRINITY_DN15235_c0_g1_i2	Glyma.10G227300.1	95.78313253	596	6	34	4030	454	4450	0	4036

TRINITY_DN15235_c0_g1_i2	Glyma.10G227300.2	96.48241206	596	6	34	4030	454	4450	0	4036

TRINITY_DN5346_c0_g1_i1	Glyma.02G129800.1	97.5308642	404	14	450	4425	6	3990	0	4985

TRINITY_DN5346_c0_g1_i1	Glyma.02G129800.2	98.71794872	404	14	450	4425	6	3990	0	4985

TRINITY_DN13187_c0_g1_i1	Glyma.17G045200.2	96.79487179	251	21	572	4538	1	3975	0	5733

TRINITY_DN16286_c0_g1_i3	Glyma.16G182500.1	97.05400982	247	1	301	4276	4014	36	0	5960

TRINITY_DN13547_c0_g1_i2	Glyma.07G045600.3	98.03921569	289	16	1868	5827	1	3960	0	5513

TRINITY_DN16369_c0_g1_i5	Glyma.06G003600.4	95.5801105	476	23	1689	5615	1	3960	0	4340

TRINITY_DN16369_c0_g1_i2	Glyma.06G003600.4	100	351	6	1689	5648	1	3960	0	5269

TRINITY_DN13898_c0_g1_i1	Glyma.10G289800.1	100	322	18	170	4123	3945	1	0	5304

TRINITY_DN16430_c0_g2_i6	Glyma.01G209300.1	99.44134078	342	22	392	4344	1	3917	0	5073

TRINITY_DN5748_c0_g1_i1	Glyma.02G305200.1	99.38650307	281	4	82	4035	1	3939	0	5581

TRINITY_DN15078_c0_g1_i1	Glyma.18G223300.2	100	215	5	28	3974	4482	8440	0	6043

TRINITY_DN17688_c0_g1_i1	Glyma.02G158100.1	97.88732394	267	5	1	3954	451	4395	0	5731

TRINITY_DN17688_c0_g1_i1	Glyma.02G158100.2	95.65217391	267	5	1	3954	451	4395	0	5731

TRINITY_DN5748_c0_g1_i1	Glyma.14G008200.1	100	282	4	82	4034	1	3947	0	5666

TRINITY_DN16285_c1_g1_i1	Glyma.11G239800.1	100	493	13	698	4646	69	4014	0	4486

TRINITY_DN16285_c1_g1_i2	Glyma.11G239800.1	95.57522124	405	13	698	4646	69	4014	0	4974

TRINITY_DN16023_c0_g1_i3	Glyma.18G063400.1	98.18181818	345	3	370	4315	4452	501	0	5349

TRINITY_DN13895_c0_g1_i1	Glyma.13G145100.3	96.34551495	334	21	550	4449	3942	1	0	5070

TRINITY_DN13895_c0_g1_i1	Glyma.13G145100.1	95.34050179	334	21	550	4449	3942	1	0	5070

TRINITY_DN13895_c0_g1_i1	Glyma.13G145100.2	95.04132231	334	21	550	4449	3942	1	0	5070

TRINITY_DN14064_c0_g1_i3	Glyma.13G209800.1	97.8021978	210	1	317	4262	3967	19	0	6109

TRINITY_DN14064_c0_g1_i3	Glyma.15G102800.1	100	205	1	317	4262	3949	1	0	6137

TRINITY_DN12783_c0_g1_i1	Glyma.06G202500.1	97.94520548	184	0	300	4246	6267	2321	0	6270

TRINITY_DN12783_c0_g1_i1	Glyma.06G202500.2	97.94520548	184	0	300	4246	6006	2060	0	6270

TRINITY_DN16286_c0_g1_i2	Glyma.16G182500.2	97.47706422	247	1	301	4243	3981	36	0	5899

TRINITY_DN8701_c0_g1_i1	Glyma.17G015200.1	100	285	4	325	4261	4002	60	0	5664

TRINITY_DN8701_c0_g1_i1	Glyma.07G259000.1	96.80851064	275	6	325	4261	3999	60	0	5699

TRINITY_DN5160_c0_g1_i2	Glyma.02G219200.1	97.60609358	253	4	217	4155	1087	5016	0	5814

TRINITY_DN5160_c0_g1_i2	Glyma.02G219200.2	100	253	4	217	4155	676	4605	0	5814

TRINITY_DN5160_c0_g1_i2	Glyma.02G219200.3	98.86363636	253	5	217	4155	1087	5013	0	5795

TRINITY_DN15657_c0_g1_i1	Glyma.03G009200.3	100	270	13	1	3937	1027	4930	0	5550

TRINITY_DN15657_c0_g1_i1	Glyma.03G009200.1	100	270	13	1	3937	1027	4930	0	5550

TRINITY_DN15657_c0_g1_i1	Glyma.03G009200.2	100	270	13	1	3937	1027	4930	0	5550

TRINITY_DN15657_c0_g1_i1	Glyma.07G070700.1	99.57983193	276	16	1	3937	1027	4915	0	5432

TRINITY_DN15657_c0_g1_i1	Glyma.07G070700.3	99.45945946	276	16	1	3937	181	4069	0	5432

TRINITY_DN15657_c0_g1_i1	Glyma.07G070700.2	95.1417004	276	16	1	3937	181	4069	0	5432

TRINITY_DN3079_c0_g2_i1	Glyma.05G001600.1	100	356	20	17	3932	7	3912	0	5007

TRINITY_DN3079_c0_g1_i1	Glyma.05G001600.1	97.32620321	356	19	17	3931	7	3912	0	5011

TRINITY_DN9338_c0_g1_i1	Glyma.08G201300.2	98.14814815	269	5	766	4688	3920	1	0	5690

TRINITY_DN6116_c0_g1_i1	Glyma.11G256800.1	97.11286089	235	9	362	4285	5856	1939	0	5873

TRINITY_DN6116_c0_g1_i1	Glyma.11G256800.6	96.07843137	235	9	362	4285	5853	1936	0	5873

TRINITY_DN6116_c0_g1_i1	Glyma.11G256800.2	97.11191336	235	9	362	4285	5853	1936	0	5873

TRINITY_DN6116_c0_g1_i1	Glyma.11G256800.5	96.07843137	235	9	362	4285	5850	1933	0	5873

TRINITY_DN6116_c0_g1_i1	Glyma.18G000100.1	96.07843137	229	3	362	4285	5856	1936	0	5925

TRINITY_DN12456_c0_g1_i1	Glyma.14G036800.1	95.29780564	229	1	143	4063	5408	1485	0	5958

TRINITY_DN15602_c0_g1_i1	Glyma.03G226400.1	97.14285714	210	6	401	4321	1	3915	0	6006

TRINITY_DN15980_c0_g2_i2	Glyma.10G182300.1	97.72727273	297	19	110	4021	1	3882	0	5264

TRINITY_DN15634_c2_g1_i2	Glyma.20G187400.2	95.12195122	389	4	237	4151	3915	1	0	5053

TRINITY_DN15090_c0_g1_i2	Glyma.03G032900.1	95.625	274	3	309	4210	4644	743	0	5622

TRINITY_DN13895_c0_g1_i1	Glyma.10G058300.1	100	352	22	580	4449	3885	1	0	4883

TRINITY_DN13895_c0_g1_i1	Glyma.10G058300.3	100	352	22	580	4449	3885	1	0	4883

TRINITY_DN13895_c0_g1_i1	Glyma.10G058300.2	100	352	22	580	4449	3885	1	0	4883

TRINITY_DN16286_c0_g1_i2	Glyma.10G234500.1	98.4375	534	18	301	4181	4119	230	0	4089

TRINITY_DN15867_c0_g3_i2	Glyma.17G052600.1	100	333	10	189	4058	1	3882	0	5136

TRINITY_DN15867_c0_g3_i2	Glyma.13G106700.16	97.36842105	338	7	189	4058	1	3888	0	5158

TRINITY_DN15867_c0_g3_i2	Glyma.13G106700.11	98.046875	338	7	189	4058	1	3888	0	5158

TRINITY_DN15867_c0_g3_i2	Glyma.13G106700.12	96.7806841	338	7	189	4058	1	3888	0	5158

TRINITY_DN15867_c0_g3_i2	Glyma.13G106700.13	99.2481203	338	7	189	4058	1	3888	0	5158

TRINITY_DN15867_c0_g3_i2	Glyma.13G106700.14	96.72131148	338	7	189	4058	1	3888	0	5158

TRINITY_DN15867_c0_g3_i2	Glyma.13G106700.7	100	338	7	189	4058	1	3888	0	5158

TRINITY_DN15867_c0_g3_i2	Glyma.13G106700.1	100	338	7	189	4058	1	3888	0	5158

TRINITY_DN15867_c0_g3_i2	Glyma.13G106700.2	100	338	7	189	4058	1	3888	0	5158

TRINITY_DN15867_c0_g3_i2	Glyma.13G106700.3	100	338	7	189	4058	1	3888	0	5158

TRINITY_DN15867_c0_g3_i2	Glyma.13G106700.4	100	338	7	189	4058	1	3888	0	5158

TRINITY_DN15867_c0_g3_i2	Glyma.13G106700.17	98.36065574	338	7	189	4058	1	3888	0	5158

TRINITY_DN15784_c0_g1_i2	Glyma.14G181800.3	99.45355191	303	40	1	3828	34	3879	0	4820

TRINITY_DN15784_c0_g1_i2	Glyma.14G181800.2	99.45355191	303	40	1	3828	34	3879	0	4820

TRINITY_DN15784_c0_g1_i2	Glyma.14G181800.1	96.0199005	303	40	1	3828	34	3879	0	4820

TRINITY_DN15784_c0_g1_i5	Glyma.14G181800.3	100	303	40	1	3828	34	3879	0	4820

TRINITY_DN15784_c0_g1_i5	Glyma.14G181800.2	99.72826087	303	40	1	3828	34	3879	0	4820

TRINITY_DN15784_c0_g1_i5	Glyma.14G181800.1	95.83333333	303	40	1	3828	34	3879	0	4820

TRINITY_DN15784_c0_g1_i8	Glyma.14G181800.3	96.71052632	303	40	1	3828	34	3879	0	4820

TRINITY_DN15784_c0_g1_i8	Glyma.14G181800.2	98.11320755	303	40	1	3828	34	3879	0	4820

TRINITY_DN15784_c0_g1_i8	Glyma.14G181800.1	95.23809524	303	40	1	3828	34	3879	0	4820

TRINITY_DN15226_c0_g1_i1	Glyma.15G120300.1	97.32142857	401	17	1	3873	1147	5007	0	4710

TRINITY_DN15226_c0_g1_i1	Glyma.09G014600.1	97.32142857	392	23	1	3873	1147	4998	0	4700

TRINITY_DN16023_c0_g1_i3	Glyma.18G063400.2	98.18181818	340	3	370	4249	3948	63	0	5254

TRINITY_DN15931_c0_g2_i2	Glyma.13G151000.1	98.86363636	312	17	2038	5876	2102	5982	0	5168

TRINITY_DN15931_c0_g2_i2	Glyma.13G151000.2	98.86363636	312	17	2038	5876	2102	5982	0	5168

TRINITY_DN6214_c0_g1_i1	Glyma.19G200400.1	97.22222222	260	14	5	3869	3	3870	0	5550

TRINITY_DN14844_c0_g1_i2	Glyma.08G279800.1	98.50746269	270	9	484	4344	3873	1	0	5513

TRINITY_DN6214_c0_g1_i1	Glyma.03G202700.1	99.06542056	256	15	5	3869	9	3876	0	5583

TRINITY_DN15531_c0_g1_i2	Glyma.06G017700.1	100	230	9	64	3933	1	3876	0	5812

TRINITY_DN15531_c0_g1_i2	Glyma.04G017300.1	100	213	3	64	3933	1	3876	0	5939

TRINITY_DN16406_c0_g1_i5	Glyma.01G006600.2	97.86585366	241	0	284	4154	1	3871	0	5814

TRINITY_DN16406_c0_g1_i4	Glyma.01G006600.2	97.4317817	230	0	284	4154	1	3871	0	5875

TRINITY_DN16406_c0_g1_i3	Glyma.01G006600.2	97.22222222	226	0	284	4154	1	3871	0	5897

TRINITY_DN16153_c0_g1_i1	Glyma.06G063400.2	97	174	4	786	4649	3861	1	0	6141

TRINITY_DN6028_c0_g1_i1	Glyma.14G152500.1	95.65217391	206	0	573	4436	6408	2545	0	5995

TRINITY_DN6028_c0_g1_i1	Glyma.14G152500.4	95.65217391	206	0	573	4436	6408	2545	0	5995

TRINITY_DN6028_c0_g1_i1	Glyma.14G152500.2	95.66395664	206	0	573	4436	6408	2545	0	5995

TRINITY_DN6028_c0_g1_i1	Glyma.14G152500.3	95.65217391	206	0	573	4436	6408	2545	0	5995

TRINITY_DN6028_c0_g1_i1	Glyma.13G030700.1	98.97959184	206	0	573	4436	6408	2545	0	5995

TRINITY_DN6028_c0_g1_i1	Glyma.13G030700.2	97.33333333	206	0	573	4436	6408	2545	0	5995

TRINITY_DN8107_c0_g2_i1	Glyma.08G127200.2	100	527	23	635	4492	603	4439	0	4013

TRINITY_DN13474_c0_g2_i4	Glyma.07G042100.1	100	333	36	97	3900	97	3921	0	4662

TRINITY_DN13474_c0_g2_i1	Glyma.07G042100.1	97.88235294	328	41	97	3882	97	3921	0	4573

TRINITY_DN15187_c0_g1_i2	Glyma.08G152400.1	99.06976744	266	5	139	3985	3859	1	0	5576

TRINITY_DN16297_c0_g1_i2	Glyma.11G006800.1	100	751	46	1019	4837	382	4212	0	2593

TRINITY_DN16297_c0_g1_i2	Glyma.11G006800.2	97.46835443	751	46	1019	4837	382	4212	0	2593

TRINITY_DN15264_c0_g1_i5	Glyma.10G137600.3	100	364	11	154	3999	1	3849	0	5027

TRINITY_DN15264_c0_g1_i5	Glyma.10G137600.2	100	364	11	154	3999	1	3849	0	5027

TRINITY_DN15264_c0_g1_i5	Glyma.10G137600.1	96.66666667	364	11	154	3999	1	3849	0	5027

TRINITY_DN15264_c0_g1_i5	Glyma.02G008000.1	97.6744186	364	7	154	3999	1	3849	0	5038

TRINITY_DN15264_c0_g1_i8	Glyma.10G137600.3	95.55353902	286	7	154	3999	1	3849	0	5481

TRINITY_DN15264_c0_g1_i8	Glyma.10G137600.2	95.20547945	286	7	154	3999	1	3849	0	5481

TRINITY_DN15264_c0_g1_i8	Glyma.10G137600.1	95.20547945	286	7	154	3999	1	3849	0	5481

TRINITY_DN6168_c0_g1_i1	Glyma.10G164800.1	95.221843	285	18	109	3921	22	3867	0	5254

TRINITY_DN15264_c0_g1_i8	Glyma.02G008000.1	97.14285714	282	5	154	3999	1	3849	0	5504

TRINITY_DN16297_c0_g1_i2	Glyma.01G236400.1	95.46142209	734	46	1019	4837	394	4221	0	2712

TRINITY_DN15264_c0_g1_i6	Glyma.03G224100.1	100	401	9	201	4034	7	3852	0	4771

TRINITY_DN15264_c0_g1_i3	Glyma.03G224100.1	97.5308642	298	6	201	4034	7	3852	0	5352

TRINITY_DN8923_c0_g1_i1	Glyma.17G046900.1	96.72131148	320	9	71	3897	1	3842	0	5192

TRINITY_DN13968_c0_g3_i2	Glyma.13G183900.4	95.38461538	430	27	499	4314	211	4023	0	4335

TRINITY_DN16185_c3_g2_i2	Glyma.13G342400.1	97.63157895	229	12	1	3840	7	3816	0	5585

TRINITY_DN16185_c3_g2_i2	Glyma.13G342400.6	98.98167006	229	12	1	3840	7	3816	0	5585

TRINITY_DN16185_c3_g2_i2	Glyma.13G342400.4	98.9821883	229	12	1	3840	7	3816	0	5585

TRINITY_DN13968_c0_g3_i3	Glyma.13G183900.4	96.40718563	503	36	499	4308	211	4023	0	3903

TRINITY_DN9107_c0_g1_i1	Glyma.11G057500.1	97.93814433	341	20	162	3967	38	3867	0	4896

TRINITY_DN13968_c0_g3_i4	Glyma.13G183900.4	96.07843137	345	23	499	4317	211	4023	0	4859

TRINITY_DN16185_c3_g2_i2	Glyma.13G342400.5	97.83549784	229	11	1	3840	7	3813	0	5598

TRINITY_DN16185_c3_g2_i2	Glyma.13G342400.2	100	229	11	1	3840	7	3813	0	5598

TRINITY_DN16185_c3_g2_i2	Glyma.13G342400.3	98.57142857	229	11	1	3840	7	3813	0	5598

TRINITY_DN13968_c0_g3_i1	Glyma.13G183900.4	98	418	28	499	4311	211	4023	0	4427

TRINITY_DN8923_c0_g1_i1	Glyma.13G112900.1	96.73913043	341	5	71	3897	1	3836	0	5092

TRINITY_DN9107_c0_g1_i1	Glyma.01G184700.1	99.26470588	315	19	162	3967	38	3858	0	5003

TRINITY_DN16156_c0_g1_i3	Glyma.01G144200.1	97.9757085	451	7	271	4108	5427	1599	0	4519

TRINITY_DN16156_c0_g1_i3	Glyma.01G144200.3	97.97752809	451	7	271	4108	5427	1599	0	4519

TRINITY_DN16156_c0_g1_i3	Glyma.01G144200.2	98.57142857	451	7	271	4108	4272	444	0	4519

TRINITY_DN16156_c0_g1_i4	Glyma.01G144200.1	96.92307692	423	7	271	4108	5427	1599	0	4674

TRINITY_DN16156_c0_g1_i4	Glyma.01G144200.3	97.56097561	423	7	271	4108	5427	1599	0	4674

TRINITY_DN16156_c0_g1_i4	Glyma.01G144200.2	96.92307692	423	7	271	4108	4272	444	0	4674

TRINITY_DN16156_c0_g1_i1	Glyma.01G144200.1	95.57522124	383	5	271	4108	5427	1599	0	4907

TRINITY_DN16156_c0_g1_i1	Glyma.01G144200.3	96.42857143	383	5	271	4108	5427	1599	0	4907

TRINITY_DN16156_c0_g1_i1	Glyma.01G144200.2	97.07317073	383	5	271	4108	4272	444	0	4907

TRINITY_DN16156_c0_g1_i2	Glyma.01G144200.1	99.34640523	355	5	271	4108	5427	1599	0	5062

TRINITY_DN16156_c0_g1_i2	Glyma.01G144200.3	99.76359338	355	5	271	4108	5427	1599	0	5062

TRINITY_DN16156_c0_g1_i2	Glyma.01G144200.2	96.01593625	355	5	271	4108	4272	444	0	5062

TRINITY_DN15515_c0_g1_i1	Glyma.03G146500.1	96.2406015	303	13	214	4029	1	3825	0	5227

TRINITY_DN15515_c0_g1_i2	Glyma.03G146500.1	97.22222222	303	13	241	4056	1	3825	0	5227

TRINITY_DN16185_c3_g2_i2	Glyma.15G031900.1	96.2962963	232	9	8	3840	20	3819	0	5568

TRINITY_DN16185_c3_g2_i2	Glyma.15G031900.2	98.9821883	232	9	8	3840	17	3816	0	5568

TRINITY_DN326_c0_g1_i2	Glyma.03G253000.1	100	138	1	134	3964	1	3834	0	6296

TRINITY_DN326_c0_g1_i2	Glyma.03G253000.3	100	138	1	134	3964	1	3834	0	6296

TRINITY_DN326_c0_g1_i2	Glyma.19G250600.1	97.36842105	134	1	134	3964	1	3834	0	6318

TRINITY_DN15515_c0_g1_i1	Glyma.19G149800.1	100	295	11	214	4029	1	3825	0	5293

TRINITY_DN15515_c0_g1_i2	Glyma.19G149800.1	97.22222222	295	11	241	4056	1	3825	0	5293

TRINITY_DN14084_c0_g1_i1	Glyma.12G022500.3	99.45945946	332	2	691	4519	3829	1	0	5221

TRINITY_DN6168_c0_g1_i1	Glyma.20G227000.1	95.221843	270	12	109	3921	22	3825	0	5343

TRINITY_DN16441_c4_g2_i4	Glyma.13G175200.1	97.5	600	47	847	4640	5022	1253	0	3217

TRINITY_DN16212_c0_g1_i3	Glyma.13G142100.1	98.33887043	484	7	270	4083	4025	212	0	4318

TRINITY_DN16212_c0_g1_i2	Glyma.13G142100.1	98.27089337	424	3	270	4083	4025	212	0	4673

TRINITY_DN16212_c0_g1_i3	Glyma.10G055000.1	99.01960784	455	6	270	4080	4031	220	0	4479

TRINITY_DN16212_c0_g1_i3	Glyma.10G055000.2	97.10144928	455	6	270	4080	3977	166	0	4479

TRINITY_DN6116_c0_g1_i1	Glyma.11G256800.4	98.77300613	226	9	362	4173	5757	1952	0	5716

TRINITY_DN6116_c0_g1_i1	Glyma.11G256800.3	97.11286089	226	9	362	4173	5754	1949	0	5716

TRINITY_DN16212_c0_g1_i2	Glyma.10G055000.1	97.17514124	388	4	270	4080	4031	220	0	4861

TRINITY_DN16212_c0_g1_i2	Glyma.10G055000.2	100	388	4	270	4080	3977	166	0	4861

TRINITY_DN9338_c0_g1_i1	Glyma.15G019200.1	95.40229885	591	29	896	4683	3805	9	0	3502

TRINITY_DN16309_c0_g1_i2	Glyma.05G060300.1	98.21428571	462	33	2991	6754	2897	6684	0	4004

TRINITY_DN16309_c0_g1_i2	Glyma.05G060300.2	98.21428571	462	33	2991	6754	2897	6684	0	4004

TRINITY_DN16309_c0_g1_i2	Glyma.05G060300.3	98.20143885	462	33	2991	6754	2897	6684	0	4004

TRINITY_DN16309_c0_g1_i3	Glyma.05G060300.1	97.5	462	33	2925	6688	2897	6684	0	4004

TRINITY_DN16309_c0_g1_i3	Glyma.05G060300.2	97.5	462	33	2925	6688	2897	6684	0	4004

TRINITY_DN16309_c0_g1_i3	Glyma.05G060300.3	97.5	462	33	2925	6688	2897	6684	0	4004

TRINITY_DN16441_c4_g2_i4	Glyma.07G201200.1	95.04950495	619	35	847	4640	5016	1253	0	3206

TRINITY_DN16441_c4_g2_i4	Glyma.07G201200.2	95.62043796	619	35	847	4640	5013	1250	0	3206

TRINITY_DN16252_c0_g1_i3	Glyma.08G293600.7	95.12195122	382	36	592	4375	1	3760	0	4429

TRINITY_DN14654_c0_g1_i1	Glyma.11G089400.2	96.55172414	503	18	277	4078	3784	1	0	4063

TRINITY_DN14654_c0_g1_i1	Glyma.11G089400.1	99.13294798	503	18	277	4078	3784	1	0	4063

TRINITY_DN14654_c0_g1_i1	Glyma.11G089400.3	97.47474747	503	18	277	4078	3784	1	0	4063

TRINITY_DN14654_c0_g1_i1	Glyma.01G155300.1	96.55172414	481	19	277	4078	3784	1	0	4185

TRINITY_DN16397_c0_g1_i3	Glyma.14G162300.1	97.98657718	434	10	641	4443	4985	1186	0	4554

TRINITY_DN16252_c0_g1_i3	Glyma.08G293600.6	97.82608696	382	34	592	4375	1	3757	0	4442

TRINITY_DN12896_c0_g1_i1	Glyma.15G122400.2	95.84352078	245	29	226	3981	1	3792	0	5247

TRINITY_DN16397_c0_g1_i4	Glyma.14G162300.1	100	304	2	481	4281	4980	1186	0	5297

TRINITY_DN6121_c0_g1_i1	Glyma.08G069400.1	99.47368421	257	2	510	4307	3798	1	0	5579

TRINITY_DN15782_c0_g1_i4	Glyma.04G087300.1	99.21259843	247	7	5	3800	568	4344	0	5507

TRINITY_DN15782_c0_g1_i4	Glyma.06G089000.2	100	228	7	5	3800	571	4344	0	5594

TRINITY_DN15782_c0_g1_i4	Glyma.06G089000.1	96.29005059	228	7	5	3800	571	4344	0	5594

TRINITY_DN334_c0_g1_i1	Glyma.13G158400.1	100	306	7	1	3778	219	4008	0	5227

TRINITY_DN16114_c0_g1_i3	Glyma.11G009500.1	98.64864865	229	25	136	3891	1	3774	0	5419

TRINITY_DN17153_c0_g1_i1	Glyma.20G218400.2	97.36842105	267	9	393	4169	3777	1	0	5385

TRINITY_DN17153_c0_g1_i1	Glyma.20G218400.1	95.57522124	267	9	393	4169	3777	1	0	5385

TRINITY_DN17153_c0_g1_i1	Glyma.20G218400.3	98.36065574	267	9	393	4169	3777	1	0	5385

TRINITY_DN14654_c0_g1_i1	Glyma.16G111200.3	99.57446809	314	14	256	4034	3816	44	0	5123

TRINITY_DN14654_c0_g1_i1	Glyma.16G111200.2	96.83257919	314	14	256	4034	3816	44	0	5123

TRINITY_DN14654_c0_g1_i1	Glyma.16G111200.1	98.96373057	314	14	256	4034	3816	44	0	5123

TRINITY_DN17153_c0_g1_i1	Glyma.10G171900.2	100	278	8	393	4169	3777	1	0	5336

TRINITY_DN17153_c0_g1_i1	Glyma.10G171900.1	100	278	8	393	4169	3777	1	0	5336

TRINITY_DN9262_c0_g1_i1	Glyma.05G212000.1	100	258	16	1	3746	616	4394	0	5258

TRINITY_DN14654_c0_g1_i1	Glyma.09G194900.1	98.82352941	292	13	256	4034	3810	44	0	5217

TRINITY_DN8107_c0_g2_i1	Glyma.05G168600.3	98.18181818	529	26	533	4308	501	4255	0	3818

TRINITY_DN16349_c0_g2_i1	Glyma.10G019300.1	98.73417722	258	18	68	3829	1	3744	0	5169

TRINITY_DN11030_c0_g2_i1	Glyma.13G280700.1	98.7854251	206	5	206	3985	1	3777	0	5786

TRINITY_DN15782_c0_g1_i2	Glyma.04G087300.1	99.0521327	242	5	5	3784	568	4344	0	5609

TRINITY_DN15782_c0_g1_i2	Glyma.06G089000.2	97.52475248	223	5	5	3784	571	4344	0	5696

TRINITY_DN15782_c0_g1_i2	Glyma.06G089000.1	99.0521327	223	5	5	3784	571	4344	0	5696

TRINITY_DN16349_c0_g2_i1	Glyma.02G155100.1	98.73417722	243	29	68	3829	1	3732	0	5208

TRINITY_DN11030_c0_g2_i1	Glyma.12G220900.1	100	196	8	206	3985	1	3750	0	5701

TRINITY_DN16430_c0_g2_i6	Glyma.11G032900.3	99.44134078	334	18	392	4158	1	3750	0	4863

TRINITY_DN8774_c0_g1_i1	Glyma.20G227200.4	97.6635514	209	17	327	4086	1	3748	0	5498

TRINITY_DN8774_c0_g1_i2	Glyma.20G227200.4	97.40932642	209	17	327	4086	1	3748	0	5498

TRINITY_DN8774_c0_g1_i3	Glyma.20G227200.4	96.34369287	209	17	327	4086	1	3748	0	5498

TRINITY_DN16114_c0_g1_i3	Glyma.01G233300.1	100	234	17	136	3891	1	3762	0	5457

TRINITY_DN11523_c0_g1_i2	Glyma.16G011100.4	98.82352941	297	45	688	4383	3753	1	0	4673

TRINITY_DN11523_c0_g1_i2	Glyma.16G011100.1	97.43589744	297	45	688	4383	3753	1	0	4673

TRINITY_DN16349_c0_g2_i1	Glyma.10G019400.1	98.72881356	423	52	68	3829	1	3681	0	3958

TRINITY_DN16430_c0_g2_i5	Glyma.01G209300.1	98.14814815	335	9	111	3872	159	3917	0	4972

TRINITY_DN16428_c1_g1_i3	Glyma.18G210300.3	100	184	2	510	4280	3777	22	0	5847

TRINITY_DN16428_c1_g1_i3	Glyma.18G210300.2	100	184	2	510	4280	3756	1	0	5847

TRINITY_DN15657_c0_g1_i4	Glyma.03G009200.3	97.6744186	552	44	1	3749	1729	5444	0	3441

TRINITY_DN15657_c0_g1_i4	Glyma.03G009200.1	95.74468085	552	44	1	3749	1729	5444	0	3441

TRINITY_DN15657_c0_g1_i4	Glyma.03G009200.2	96.96969697	552	44	1	3749	1729	5444	0	3441

TRINITY_DN8943_c0_g1_i1	Glyma.10G140500.1	100	451	17	741	4474	3769	9	0	4205

TRINITY_DN11523_c0_g1_i2	Glyma.07G042700.1	95.5801105	300	36	688	4383	3753	1	0	4745

TRINITY_DN8943_c0_g1_i1	Glyma.20G089300.1	100	435	15	741	4474	3769	9	0	4305

TRINITY_DN15264_c0_g1_i8	Glyma.03G224100.1	100	559	13	244	3999	103	3852	0	3712

TRINITY_DN15657_c0_g1_i4	Glyma.07G070700.1	98.7012987	556	47	1	3749	1729	5429	0	3367

TRINITY_DN15657_c0_g1_i4	Glyma.07G070700.3	97.72727273	556	47	1	3749	883	4583	0	3367

TRINITY_DN15657_c0_g1_i4	Glyma.07G070700.2	100	556	47	1	3749	883	4583	0	3367

TRINITY_DN15264_c0_g1_i3	Glyma.10G137600.3	95.04950495	504	8	285	4034	91	3849	0	4047

TRINITY_DN15264_c0_g1_i3	Glyma.10G137600.2	100	504	8	285	4034	91	3849	0	4047

TRINITY_DN15264_c0_g1_i3	Glyma.10G137600.1	100	504	8	285	4034	91	3849	0	4047

TRINITY_DN15264_c0_g1_i5	Glyma.03G224100.1	100	456	10	244	3999	103	3852	0	4294

TRINITY_DN15264_c0_g1_i3	Glyma.02G008000.1	96.2962963	503	6	285	4034	91	3849	0	4063

TRINITY_DN15657_c0_g1_i4	Glyma.09G259400.1	96.71532847	301	5	1	3758	1780	5517	0	5099

TRINITY_DN15657_c0_g1_i4	Glyma.09G259400.2	98.77300613	301	5	1	3758	1780	5517	0	5099

TRINITY_DN15657_c0_g1_i4	Glyma.09G259400.3	96.76258993	301	5	1	3758	1780	5517	0	5099

TRINITY_DN15264_c0_g1_i6	Glyma.10G137600.3	100	426	4	285	4034	91	3849	0	4501

TRINITY_DN15264_c0_g1_i6	Glyma.10G137600.2	100	426	4	285	4034	91	3849	0	4501

TRINITY_DN15264_c0_g1_i6	Glyma.10G137600.1	97.14285714	426	4	285	4034	91	3849	0	4501

TRINITY_DN15264_c0_g1_i6	Glyma.02G008000.1	100	421	4	285	4034	91	3849	0	4529

TRINITY_DN15657_c0_g1_i4	Glyma.18G232900.3	100	306	4	1	3755	1780	5533	0	5199

TRINITY_DN15657_c0_g1_i4	Glyma.18G232900.1	99.1011236	306	4	1	3755	1780	5533	0	5199

TRINITY_DN15657_c0_g1_i4	Glyma.18G232900.2	96.91011236	306	4	1	3755	1720	5473	0	5199

TRINITY_DN15832_c0_g2_i1	Glyma.19G021500.1	100	249	4	264	4013	3753	1	0	5509

TRINITY_DN16428_c1_g1_i2	Glyma.09G278500.1	100	167	19	993	4717	3783	47	0	5722

TRINITY_DN15832_c0_g2_i1	Glyma.13G063700.1	98.24120603	242	2	264	4013	3750	1	0	5563

TRINITY_DN16283_c0_g2_i1	Glyma.19G171200.1	95	298	9	1	3746	3858	122	0	5186

TRINITY_DN16283_c0_g2_i2	Glyma.19G171200.1	95.08196721	239	9	1	3746	3858	122	0	5513

TRINITY_DN16283_c0_g2_i2	Glyma.13G130000.1	99.27007299	455	25	1	3746	3810	122	0	4017

TRINITY_DN16283_c0_g2_i1	Glyma.13G130000.1	100	400	25	1	3746	3810	122	0	4322

TRINITY_DN16283_c0_g2_i1	Glyma.03G170000.1	95	299	8	1	3746	3846	122	0	5114

TRINITY_DN16283_c0_g2_i2	Glyma.03G170000.1	95.08196721	243	9	1	3746	3846	122	0	5424

TRINITY_DN16283_c0_g2_i2	Glyma.10G042600.1	99.27007299	472	19	1	3744	3834	124	0	4063

TRINITY_DN16283_c0_g2_i1	Glyma.10G042600.1	98.63013699	418	19	1	3744	3834	124	0	4362

TRINITY_DN14989_c0_g1_i1	Glyma.13G222000.1	95.94272076	433	6	84	3797	37	3777	0	4313

TRINITY_DN8943_c0_g1_i1	Glyma.03G226200.1	100	213	2	741	4482	3742	1	0	5720

TRINITY_DN8943_c0_g1_i1	Glyma.19G223200.1	100	210	2	741	4482	3742	1	0	5736

TRINITY_DN13347_c0_g1_i2	Glyma.09G206300.1	95.73459716	205	6	371	4102	1	3738	0	5696

TRINITY_DN13347_c0_g1_i2	Glyma.01G016500.2	95.73459716	197	5	371	4102	1	3741	0	5758

TRINITY_DN13347_c0_g1_i2	Glyma.01G016500.1	95.73459716	197	5	371	4102	1	3741	0	5758

TRINITY_DN15226_c0_g1_i2	Glyma.07G268200.2	95.34313725	636	54	1164	4865	855	4544	0	2826

TRINITY_DN15226_c0_g1_i4	Glyma.07G268200.2	97.4137931	636	54	1106	4807	855	4544	0	2826

TRINITY_DN15226_c0_g1_i5	Glyma.07G268200.2	96.41693811	636	54	1116	4817	855	4544	0	2826

TRINITY_DN15235_c0_g1_i4	Glyma.16G172400.2	100	668	23	310	4032	1	3720	0	3022

TRINITY_DN15235_c0_g1_i2	Glyma.16G172400.2	98.92473118	669	23	310	4031	1	3719	0	3014

TRINITY_DN9235_c0_g1_i1	Glyma.08G111400.1	95.22058824	273	12	1	3714	4636	8358	0	5193

TRINITY_DN15531_c0_g1_i2	Glyma.04G017300.2	99	205	4	64	3789	1	3733	0	5712

TRINITY_DN16428_c1_g1_i2	Glyma.18G210300.1	100	200	13	993	4717	3759	47	0	5605

TRINITY_DN15562_c1_g1_i1	Glyma.15G212800.4	100	170	1	931	4661	1	3732	0	5943

TRINITY_DN15562_c1_g1_i1	Glyma.15G212800.3	99.13793103	170	1	931	4661	1	3732	0	5943

TRINITY_DN15562_c1_g1_i3	Glyma.15G212800.4	97.72727273	170	1	1992	5722	1	3732	0	5943

TRINITY_DN15562_c1_g1_i3	Glyma.15G212800.3	100	170	1	1992	5722	1	3732	0	5943

TRINITY_DN2580_c0_g1_i1	Glyma.13G062800.1	99.01960784	195	6	200	3922	3729	1	0	5757

TRINITY_DN2580_c0_g1_i1	Glyma.19G022800.1	99.01960784	210	3	200	3922	3729	1	0	5685

TRINITY_DN643_c0_g1_i1	Glyma.06G145900.5	96.66666667	198	0	506	4230	3810	86	0	5782

TRINITY_DN643_c0_g1_i1	Glyma.06G145900.4	96.66666667	198	0	506	4230	3810	86	0	5782

TRINITY_DN643_c0_g1_i1	Glyma.06G145900.2	100	198	0	506	4230	3810	86	0	5782

TRINITY_DN643_c0_g1_i1	Glyma.06G145900.1	100	198	0	506	4230	3810	86	0	5782

TRINITY_DN643_c0_g1_i1	Glyma.06G145900.3	95.94095941	198	0	506	4230	3810	86	0	5782

TRINITY_DN643_c0_g1_i1	Glyma.04G220000.1	97.05882353	195	0	506	4230	3810	86	0	5799

TRINITY_DN643_c0_g1_i1	Glyma.04G220000.2	97.6744186	195	0	506	4230	3810	86	0	5799

TRINITY_DN643_c0_g1_i1	Glyma.04G220000.3	100	195	0	506	4230	3810	86	0	5799

TRINITY_DN15235_c0_g1_i4	Glyma.20G165000.1	100	286	2	310	4032	1	3723	0	5280

TRINITY_DN15235_c0_g1_i4	Glyma.20G165000.2	100	286	2	310	4032	1	3723	0	5280

TRINITY_DN15235_c0_g1_i2	Glyma.20G165000.1	98.02631579	287	2	310	4031	1	3722	0	5273

TRINITY_DN15235_c0_g1_i2	Glyma.20G165000.2	96.53333333	287	2	310	4031	1	3722	0	5273

TRINITY_DN8358_c0_g1_i1	Glyma.02G212700.1	95.65217391	262	8	187	3894	1	3705	0	5210

TRINITY_DN8358_c0_g1_i1	Glyma.02G212700.4	100	262	8	187	3894	1	3705	0	5210

TRINITY_DN8358_c0_g1_i1	Glyma.02G212700.3	100	262	10	187	3894	1	3702	0	5192

TRINITY_DN15484_c0_g1_i5	Glyma.15G261100.1	100	425	39	411	4100	3699	1	0	4163

TRINITY_DN8358_c0_g1_i1	Glyma.14G180600.2	96.59090909	252	8	187	3894	1	3702	0	5280

TRINITY_DN8358_c0_g1_i1	Glyma.14G180600.1	96.59090909	252	8	187	3894	1	3702	0	5280

TRINITY_DN6178_c0_g1_i1	Glyma.05G131500.2	99.47961839	266	12	1	3701	3308	7008	0	5184

TRINITY_DN6178_c0_g1_i1	Glyma.05G131500.1	100	266	12	1	3701	3308	7008	0	5184

TRINITY_DN14571_c0_g2_i2	Glyma.18G245000.1	96	324	25	437	4135	3672	1	0	4684

TRINITY_DN14571_c0_g2_i1	Glyma.18G245000.1	100	321	25	437	4114	3672	1	0	4599

TRINITY_DN14844_c0_g1_i2	Glyma.02G041400.1	100	489	21	484	4170	3843	139	0	3951

TRINITY_DN14844_c0_g1_i2	Glyma.02G041400.6	100	487	22	484	4170	3840	139	0	3941

TRINITY_DN11523_c0_g1_i1	Glyma.16G011100.4	95	295	45	399	4029	3688	1	0	4564

TRINITY_DN11523_c0_g1_i1	Glyma.16G011100.1	100	295	45	399	4029	3688	1	0	4564

TRINITY_DN14908_c0_g1_i2	Glyma.12G088600.5	95.90163934	247	13	780	4439	3693	1	0	5072

TRINITY_DN14908_c0_g1_i2	Glyma.12G088600.4	96.96969697	247	13	780	4439	3693	1	0	5072

TRINITY_DN14908_c0_g1_i2	Glyma.12G088600.1	96.96969697	247	13	780	4439	3693	1	0	5072

TRINITY_DN14908_c0_g1_i2	Glyma.12G088600.2	95.91836735	247	13	780	4439	3693	1	0	5072

TRINITY_DN14908_c0_g1_i2	Glyma.12G088600.3	95.90163934	247	13	780	4439	3693	1	0	5072

TRINITY_DN6168_c0_g1_i1	Glyma.10G164800.2	95.221843	273	18	109	3777	22	3723	0	5055

TRINITY_DN14908_c0_g1_i2	Glyma.11G183800.4	95.91836735	282	17	780	4439	3693	1	0	4900

TRINITY_DN14908_c0_g1_i2	Glyma.11G183800.2	95.91527988	282	17	780	4439	3693	1	0	4900

TRINITY_DN14908_c0_g1_i2	Glyma.11G183800.3	95.91194969	282	17	780	4439	3693	1	0	4900

TRINITY_DN14908_c0_g1_i2	Glyma.11G183800.1	95.91836735	282	17	780	4439	3693	1	0	4900

TRINITY_DN15515_c0_g1_i1	Glyma.19G149800.3	100	282	7	343	4029	10	3711	0	5166

TRINITY_DN15515_c0_g1_i2	Glyma.19G149800.3	96.07142857	282	7	370	4056	10	3711	0	5166

TRINITY_DN11523_c0_g1_i1	Glyma.07G042700.1	100	297	36	399	4029	3688	1	0	4641

TRINITY_DN15270_c0_g1_i1	Glyma.02G281000.2	100	214	2	29	3727	694	4390	0	5622

TRINITY_DN16345_c0_g1_i5	Glyma.09G182600.1	98.4496124	189	4	440	4138	3717	34	0	5686

TRINITY_DN16345_c0_g1_i5	Glyma.07G093700.1	98.4375	186	5	440	4138	3729	40	0	5742

TRINITY_DN16245_c0_g1_i5	Glyma.07G007700.1	98.21428571	262	4	364	4059	3696	1	0	5352

TRINITY_DN16245_c0_g1_i5	Glyma.07G007700.4	95	262	4	364	4059	3696	1	0	5352

TRINITY_DN16245_c0_g1_i2	Glyma.07G007700.1	95.94594595	260	7	364	4038	3696	1	0	5227

TRINITY_DN16245_c0_g1_i2	Glyma.07G007700.4	98.07692308	260	7	364	4038	3696	1	0	5227

TRINITY_DN16245_c0_g1_i5	Glyma.07G007700.2	100	260	7	364	4059	3675	1	0	5227

TRINITY_DN16245_c0_g1_i5	Glyma.07G007700.3	96.82539683	260	7	364	4059	3675	1	0	5227

TRINITY_DN16245_c0_g1_i5	Glyma.08G190400.1	98.11320755	262	2	364	4059	3696	1	0	5363

TRINITY_DN16245_c0_g1_i5	Glyma.08G190400.2	100	259	4	364	4059	3675	1	0	5243

TRINITY_DN16245_c0_g1_i2	Glyma.08G190400.1	96.58500371	258	6	364	4038	3696	1	0	5249

TRINITY_DN16252_c0_g1_i1	Glyma.08G293600.9	95.67307692	391	26	72	3747	13	3675	0	4349

TRINITY_DN16252_c0_g1_i3	Glyma.08G293600.9	100	391	26	72	3747	13	3675	0	4349

TRINITY_DN12926_c0_g1_i1	Glyma.13G124600.3	96.20253165	250	14	17	3685	1	3678	0	5204

TRINITY_DN12926_c0_g1_i1	Glyma.13G124600.1	95.96412556	250	14	17	3685	1	3678	0	5204

TRINITY_DN16043_c0_g2_i3	Glyma.08G293900.1	96.93877551	196	9	28	3708	1	3684	0	5620

TRINITY_DN16252_c0_g1_i1	Glyma.08G293600.8	99.31034483	391	24	72	3747	13	3672	0	4364

TRINITY_DN16252_c0_g1_i3	Glyma.08G293600.8	96.34146341	391	24	72	3747	13	3672	0	4364

TRINITY_DN16245_c0_g1_i4	Glyma.15G025800.4	99.18367347	379	23	285	3953	3675	1	0	4494

TRINITY_DN16245_c0_g1_i4	Glyma.15G025800.3	99.18478261	379	23	285	3953	3675	1	0	4494

TRINITY_DN16245_c0_g1_i4	Glyma.15G025800.1	97.68339768	379	23	285	3953	3675	1	0	4494

TRINITY_DN16245_c0_g1_i4	Glyma.13G348200.2	98.7012987	386	23	285	3953	3675	1	0	4477

TRINITY_DN16245_c0_g1_i4	Glyma.13G348200.1	96.07843137	386	23	285	3953	3675	1	0	4477

TRINITY_DN16297_c0_g2_i1	Glyma.10G245400.2	96.84210526	690	26	695	4368	610	4277	0	2813

TRINITY_DN16297_c0_g2_i1	Glyma.10G245400.3	97.8805395	690	26	695	4368	610	4277	0	2813

TRINITY_DN16297_c0_g2_i1	Glyma.10G245400.7	96.10778443	690	26	695	4368	610	4277	0	2813

TRINITY_DN16297_c0_g2_i1	Glyma.10G245400.6	96.33699634	690	26	695	4368	610	4277	0	2813

TRINITY_DN16297_c0_g2_i1	Glyma.10G245400.1	100	690	26	695	4368	610	4277	0	2813

TRINITY_DN16297_c0_g2_i1	Glyma.10G245400.5	96.96969697	690	26	695	4368	610	4277	0	2813

TRINITY_DN16297_c0_g2_i1	Glyma.10G245400.4	98.59550562	690	26	695	4368	610	4277	0	2813

TRINITY_DN16266_c0_g2_i2	Glyma.10G290800.2	100	389	5	432	4110	1	3682	0	4602

TRINITY_DN16297_c0_g2_i1	Glyma.20G149200.2	98.14814815	691	13	695	4370	607	4276	0	2867

TRINITY_DN16297_c0_g2_i1	Glyma.20G149200.3	97.6744186	691	13	695	4370	607	4276	0	2867

TRINITY_DN16297_c0_g2_i1	Glyma.20G149200.4	96.73913043	691	13	695	4370	607	4276	0	2867

TRINITY_DN16297_c0_g2_i1	Glyma.20G149200.1	96.16161616	691	13	695	4370	607	4276	0	2867

TRINITY_DN14844_c0_g1_i2	Glyma.01G023600.1	96.3963964	476	22	484	4140	3843	168	0	3973

TRINITY_DN14844_c0_g1_i2	Glyma.01G023600.2	96.16564417	476	22	484	4140	3843	168	0	3973

TRINITY_DN16245_c0_g1_i5	Glyma.15G025800.4	100	467	31	394	4059	3642	1	0	3884

TRINITY_DN16245_c0_g1_i5	Glyma.15G025800.3	99.33333333	467	31	394	4059	3642	1	0	3884

TRINITY_DN16245_c0_g1_i5	Glyma.15G025800.1	98.31932773	467	31	394	4059	3642	1	0	3884

TRINITY_DN16245_c0_g1_i5	Glyma.13G348200.2	98.87640449	459	33	395	4059	3641	1	0	3927

TRINITY_DN16245_c0_g1_i5	Glyma.13G348200.1	100	459	33	395	4059	3641	1	0	3927

TRINITY_DN15187_c0_g1_i4	Glyma.08G152400.1	100	258	5	95	3760	3678	1	0	5286

TRINITY_DN3229_c0_g1_i1	Glyma.05G238800.1	96.51162791	220	4	4	3677	3677	1	0	5541

TRINITY_DN16441_c1_g1_i1	Glyma.14G016800.1	97.59450172	315	8	88	3762	1	3663	0	4942

TRINITY_DN16245_c0_g1_i2	Glyma.07G007700.2	96.42857143	261	4	364	4038	3675	1	0	5319

TRINITY_DN16245_c0_g1_i2	Glyma.07G007700.3	99.51923077	261	4	364	4038	3675	1	0	5319

TRINITY_DN3229_c0_g1_i1	Glyma.08G045900.1	96.51162791	205	1	4	3677	3677	1	0	5635

TRINITY_DN16245_c0_g1_i2	Glyma.08G190400.2	95.93495935	259	2	364	4038	3675	1	0	5341

TRINITY_DN12946_c0_g1_i3	Glyma.13G156500.3	100	286	15	261	3915	4875	1212	0	5001

TRINITY_DN14659_c2_g1_i2	Glyma.20G190900.1	97.12230216	517	16	332	3997	3657	1	0	3770

TRINITY_DN14659_c2_g1_i4	Glyma.11G113000.1	99.21259843	573	13	493	4146	3666	1	0	3452

TRINITY_DN14659_c2_g1_i2	Glyma.10G199300.1	96.82539683	521	10	332	3997	3657	1	0	3759

TRINITY_DN16245_c0_g1_i4	Glyma.07G007700.1	97.2972973	341	15	309	3953	3669	1	0	4698

TRINITY_DN16245_c0_g1_i4	Glyma.07G007700.4	95.32467532	341	15	309	3953	3669	1	0	4698

TRINITY_DN14659_c2_g1_i3	Glyma.20G190900.1	97.19626168	413	12	493	4158	3657	1	0	4368

TRINITY_DN16245_c0_g1_i4	Glyma.08G190400.1	98.37398374	335	14	309	3953	3669	1	0	4743

TRINITY_DN14659_c2_g1_i4	Glyma.12G038900.4	97.6744186	573	10	493	4146	3666	1	0	3474

TRINITY_DN14659_c2_g1_i4	Glyma.12G038900.1	95.87231701	573	10	493	4146	3666	1	0	3474

TRINITY_DN14659_c2_g1_i4	Glyma.12G038900.2	96.15384615	573	10	493	4146	3666	1	0	3474

TRINITY_DN14659_c2_g1_i3	Glyma.10G199300.1	100	416	4	493	4158	3657	1	0	4362

TRINITY_DN12946_c0_g1_i2	Glyma.13G156500.3	96.69117647	286	15	261	3910	4875	1212	0	5025

TRINITY_DN14659_c2_g1_i1	Glyma.11G113000.1	95.84352078	448	9	332	3985	3666	1	0	4178

TRINITY_DN14659_c2_g1_i3	Glyma.11G113000.1	100	335	4	493	4158	3666	1	0	4881

TRINITY_DN14659_c2_g1_i3	Glyma.12G038900.4	100	329	5	493	4158	3666	1	0	4915

TRINITY_DN14659_c2_g1_i3	Glyma.12G038900.1	100	329	5	493	4158	3666	1	0	4915

TRINITY_DN14659_c2_g1_i3	Glyma.12G038900.2	100	329	5	493	4158	3666	1	0	4915

TRINITY_DN14659_c2_g1_i1	Glyma.12G038900.4	99.68454259	451	5	332	3985	3666	1	0	4183

TRINITY_DN14659_c2_g1_i1	Glyma.12G038900.1	97.89156627	451	5	332	3985	3666	1	0	4183

TRINITY_DN14659_c2_g1_i1	Glyma.12G038900.2	95.84352078	451	5	332	3985	3666	1	0	4183

TRINITY_DN14659_c2_g1_i2	Glyma.11G113000.1	97.44897959	210	0	332	3997	3666	1	0	5607

TRINITY_DN14659_c2_g1_i2	Glyma.12G038900.4	100	207	0	332	3997	3666	1	0	5624

TRINITY_DN14659_c2_g1_i2	Glyma.12G038900.1	100	207	0	332	3997	3666	1	0	5624

TRINITY_DN14659_c2_g1_i2	Glyma.12G038900.2	96.82539683	207	0	332	3997	3666	1	0	5624

TRINITY_DN16441_c1_g1_i1	Glyma.02G296800.1	97.56097561	309	8	103	3762	8	3657	0	4939

TRINITY_DN16441_c1_g1_i1	Glyma.02G296800.4	98.18181818	309	9	103	3762	8	3648	0	4879

TRINITY_DN16441_c1_g1_i1	Glyma.02G296800.2	97.07112971	307	9	103	3762	8	3648	0	4891

TRINITY_DN16441_c1_g1_i1	Glyma.02G296800.5	97.07112971	307	10	103	3762	8	3651	0	4911

TRINITY_DN16441_c1_g1_i1	Glyma.02G296800.3	97.14285714	305	11	103	3762	8	3642	0	4863

TRINITY_DN16245_c0_g5_i2	Glyma.17G123800.1	100	289	12	257	3910	3654	1	0	5070

TRINITY_DN12896_c0_g1_i1	Glyma.09G016800.1	95.83333333	249	36	621	4256	447	4073	0	5000

TRINITY_DN16245_c0_g1_i2	Glyma.15G025800.4	100	465	26	394	4038	3642	1	0	3969

TRINITY_DN16245_c0_g1_i2	Glyma.15G025800.3	96.55172414	465	26	394	4038	3642	1	0	3969

TRINITY_DN16245_c0_g1_i2	Glyma.15G025800.1	95.87628866	465	26	394	4038	3642	1	0	3969

TRINITY_DN14659_c2_g1_i1	Glyma.20G190900.1	98.21428571	363	7	332	3985	3657	1	0	4689

TRINITY_DN14659_c2_g1_i1	Glyma.10G199300.1	95.05376344	363	9	332	3985	3657	1	0	4689

TRINITY_DN13105_c1_g1_i1	Glyma.09G278900.1	97.39776952	256	6	165	3821	1	3657	0	5302

TRINITY_DN13105_c1_g1_i2	Glyma.09G278900.1	97.38562092	256	6	165	3821	1	3657	0	5302

TRINITY_DN14654_c0_g1_i3	Glyma.16G111200.3	100	234	6	256	3914	3816	165	0	5404

TRINITY_DN14654_c0_g1_i3	Glyma.16G111200.2	100	234	6	256	3914	3816	165	0	5404

TRINITY_DN14654_c0_g1_i3	Glyma.16G111200.1	99.19354839	234	6	256	3914	3816	165	0	5404

TRINITY_DN14654_c0_g1_i3	Glyma.09G194900.1	97.84946237	213	7	256	3914	3810	165	0	5481

TRINITY_DN15484_c0_g1_i5	Glyma.08G165900.1	100	395	35	477	4100	3636	1	0	4205

TRINITY_DN15484_c0_g1_i5	Glyma.08G165900.2	99.27536232	395	35	477	4100	3636	1	0	4205

TRINITY_DN16438_c0_g1_i5	Glyma.02G101800.1	96.7032967	313	23	134	3778	1	3633	0	4776

TRINITY_DN16245_c0_g5_i1	Glyma.17G123800.1	98.13953488	260	6	257	3910	3654	1	0	5264

TRINITY_DN14659_c2_g1_i4	Glyma.20G190900.1	98.14323607	259	3	493	4146	3657	1	0	5288

TRINITY_DN14659_c2_g1_i4	Glyma.10G199300.1	100	258	3	493	4146	3657	1	0	5293

TRINITY_DN15467_c0_g1_i1	Glyma.17G002000.1	97.24310777	196	0	176	3832	1	3657	0	5668

TRINITY_DN15467_c0_g1_i1	Glyma.07G272400.1	99.45054945	192	1	176	3832	1	3654	0	5670

TRINITY_DN16245_c0_g1_i2	Glyma.13G348200.2	95.40636042	464	24	395	4038	3641	1	0	4006

TRINITY_DN16245_c0_g1_i2	Glyma.13G348200.1	96.92307692	464	24	395	4038	3641	1	0	4006

TRINITY_DN16430_c0_g2_i5	Glyma.11G032900.3	98.14814815	324	13	46	3686	118	3750	0	4745

TRINITY_DN16245_c0_g1_i4	Glyma.07G007700.2	97.79005525	344	7	309	3953	3648	1	0	4778

TRINITY_DN16245_c0_g1_i4	Glyma.07G007700.3	99.42363112	344	7	309	3953	3648	1	0	4778

TRINITY_DN16245_c0_g1_i4	Glyma.08G190400.2	98.93048128	339	6	309	3953	3648	1	0	4817

TRINITY_DN16352_c0_g2_i3	Glyma.08G194600.2	100	297	9	427	4063	3643	1	0	4977

TRINITY_DN16297_c0_g1_i1	Glyma.11G006800.1	98.30508475	715	41	1019	4636	382	4008	0	2479

TRINITY_DN16297_c0_g1_i1	Glyma.11G006800.2	99.86648865	715	41	1019	4636	382	4008	0	2479

TRINITY_DN16297_c0_g1_i1	Glyma.01G236400.1	98.30508475	686	43	1019	4636	394	4017	0	2621

TRINITY_DN10930_c0_g1_i1	Glyma.10G291300.1	95.73459716	224	0	527	4171	3645	1	0	5491

TRINITY_DN10930_c0_g1_i1	Glyma.20G240500.1	96	214	0	527	4171	3645	1	0	5546

TRINITY_DN16428_c1_g1_i2	Glyma.18G210300.6	100	195	13	1081	4717	3671	47	0	5470

TRINITY_DN16428_c1_g1_i3	Glyma.18G210300.6	99.0228013	195	13	937	4573	3671	47	0	5470

TRINITY_DN10732_c0_g1_i2	Glyma.15G034000.1	100	222	3	643	4274	3639	2	0	5384

TRINITY_DN14654_c0_g1_i3	Glyma.01G155300.1	99.19354839	535	21	277	3912	3784	167	0	3568

TRINITY_DN14023_c0_g2_i2	Glyma.05G011900.2	97.84946237	214	4	169	3793	1	3639	0	5433

TRINITY_DN14654_c0_g1_i3	Glyma.11G089400.2	99.38650307	552	18	277	3910	3784	169	0	3482

TRINITY_DN14654_c0_g1_i3	Glyma.11G089400.1	98.7654321	552	18	277	3910	3784	169	0	3482

TRINITY_DN14654_c0_g1_i3	Glyma.11G089400.3	98.7654321	552	18	277	3910	3784	169	0	3482

TRINITY_DN13501_c0_g2_i1	Glyma.06G068900.1	97.82608696	273	2	111	3749	22	3657	0	5188

TRINITY_DN16285_c1_g1_i2	Glyma.05G178900.3	95	278	9	633	4263	1	3634	0	5107

TRINITY_DN8943_c0_g1_i1	Glyma.10G140500.4	100	437	17	871	4474	3639	9	0	4043

TRINITY_DN16285_c1_g1_i1	Glyma.05G178900.3	96.36871508	209	5	633	4263	1	3634	0	5511

TRINITY_DN5058_c0_g1_i1	Glyma.09G153100.1	100	203	3	1	3634	732	4350	0	5489

TRINITY_DN5058_c0_g1_i1	Glyma.16G203900.1	100	197	5	1	3634	726	4344	0	5522

TRINITY_DN5058_c0_g1_i1	Glyma.16G203900.3	97.36842105	197	5	1	3634	180	3798	0	5522

TRINITY_DN5058_c0_g1_i1	Glyma.16G203900.2	98.57142857	197	5	1	3634	147	3765	0	5522

TRINITY_DN15634_c2_g1_i2	Glyma.10G203000.1	98.91304348	358	6	237	3863	3627	1	0	4682

TRINITY_DN15634_c2_g1_i2	Glyma.10G203000.2	98.39228296	351	15	237	3863	3606	1	0	4584

TRINITY_DN13952_c0_g1_i2	Glyma.08G040500.1	100	192	5	362	3982	3627	1	0	5585

TRINITY_DN10912_c0_g1_i2	Glyma.06G061600.1	96.07843137	323	27	161	3748	3618	1	0	4610

TRINITY_DN11523_c0_g1_i1	Glyma.16G011100.3	97.95918367	291	45	483	4029	3604	1	0	4431

TRINITY_DN11523_c0_g1_i1	Glyma.16G011100.2	95.07323569	291	45	483	4029	3604	1	0	4431

TRINITY_DN11523_c0_g1_i2	Glyma.16G011100.3	97.05882353	291	45	837	4383	3604	1	0	4431

TRINITY_DN11523_c0_g1_i2	Glyma.16G011100.2	96.37188209	291	45	837	4383	3604	1	0	4431

TRINITY_DN13952_c0_g1_i3	Glyma.08G040500.1	100	192	5	351	3969	3625	1	0	5581

TRINITY_DN10912_c0_g1_i2	Glyma.04G061000.1	97.63313609	326	22	161	3748	3618	1	0	4604

TRINITY_DN16374_c1_g1_i2	Glyma.09G257900.1	100	443	49	1	3574	3084	6663	0	3675

TRINITY_DN13501_c0_g2_i1	Glyma.04G067300.3	100	278	4	132	3749	1	3615	0	5110

TRINITY_DN13501_c0_g2_i1	Glyma.04G067300.2	100	278	4	132	3749	1	3615	0	5110

TRINITY_DN13501_c0_g2_i1	Glyma.04G067300.1	100	278	4	132	3749	1	3615	0	5110

TRINITY_DN16252_c0_g1_i1	Glyma.08G293600.12	100	383	24	72	3675	13	3600	0	4276

TRINITY_DN16252_c0_g1_i3	Glyma.08G293600.12	100	383	24	72	3675	13	3600	0	4276

TRINITY_DN11523_c0_g1_i1	Glyma.07G042700.2	100	294	36	483	4029	3604	1	0	4503

TRINITY_DN11523_c0_g1_i2	Glyma.07G042700.2	100	294	36	837	4383	3604	1	0	4503

TRINITY_DN13501_c0_g2_i1	Glyma.06G068900.3	97.82608696	271	2	132	3749	1	3615	0	5160

TRINITY_DN13501_c0_g2_i1	Glyma.06G068900.2	100	271	2	132	3749	1	3615	0	5160

TRINITY_DN8358_c0_g1_i1	Glyma.02G212700.5	96.91119691	256	8	292	3894	1	3600	0	5049

TRINITY_DN16199_c0_g2_i1	Glyma.09G262000.1	95.47038328	234	13	315	3901	2	3609	0	5120

TRINITY_DN13101_c0_g1_i3	Glyma.14G112200.1	99.26470588	235	4	88	3701	1	3603	0	5267

TRINITY_DN13101_c0_g1_i3	Glyma.17G215700.1	99.2481203	214	7	88	3701	1	3597	0	5345

TRINITY_DN15515_c0_g1_i1	Glyma.03G146500.2	100	282	8	431	4029	2	3612	0	4996

TRINITY_DN15515_c0_g1_i2	Glyma.03G146500.2	96.07142857	282	8	458	4056	2	3612	0	4996

TRINITY_DN16199_c0_g2_i1	Glyma.18G230100.1	96.76258993	238	11	315	3901	2	3612	0	5162

TRINITY_DN15657_c0_g1_i1	Glyma.09G259400.1	100	572	38	349	3937	1426	4996	0	3077

TRINITY_DN15657_c0_g1_i1	Glyma.09G259400.2	100	572	38	349	3937	1426	4996	0	3077

TRINITY_DN15657_c0_g1_i1	Glyma.09G259400.3	99.01960784	572	38	349	3937	1426	4996	0	3077

TRINITY_DN15627_c0_g1_i1	Glyma.01G184500.2	100	452	27	497	4099	1	3573	0	3845

TRINITY_DN15914_c0_g2_i1	Glyma.02G271900.1	96.3963964	210	1	269	3874	3612	1	0	5469

TRINITY_DN16345_c0_g1_i5	Glyma.08G044700.1	98.4375	466	16	446	4047	3699	104	0	3932

TRINITY_DN16252_c0_g1_i1	Glyma.08G293600.7	95.43973941	363	24	592	4180	1	3583	0	4335

TRINITY_DN6339_c0_g1_i1	Glyma.12G052800.1	99.16666667	217	7	180	3782	1	3606	0	5404

TRINITY_DN6339_c0_g1_i1	Glyma.12G052800.2	99.16666667	217	8	180	3782	1	3603	0	5385

TRINITY_DN6339_c0_g2_i1	Glyma.12G052800.1	97.64705882	217	8	180	3779	1	3606	0	5385

TRINITY_DN16345_c0_g1_i5	Glyma.05G237500.1	98.4375	453	10	446	4047	3705	104	0	4076

TRINITY_DN16252_c0_g1_i1	Glyma.08G293600.6	100	363	22	592	4180	1	3580	0	4348

TRINITY_DN16374_c1_g1_i5	Glyma.09G257900.1	100	338	25	1	3571	3084	6669	0	4420

TRINITY_DN6339_c0_g1_i1	Glyma.11G128300.1	99.16666667	223	4	180	3782	1	3591	0	5297

TRINITY_DN6339_c0_g1_i1	Glyma.11G128300.2	99.16666667	223	5	180	3782	1	3588	0	5276

TRINITY_DN6339_c0_g2_i1	Glyma.11G128300.1	97.64705882	223	5	180	3779	1	3591	0	5276

TRINITY_DN15914_c0_g2_i1	Glyma.14G044900.1	97.52808989	210	1	274	3874	3607	1	0	5459

TRINITY_DN15914_c0_g2_i1	Glyma.14G044900.2	96.15384615	210	1	274	3874	3607	1	0	5459

TRINITY_DN16374_c1_g1_i2	Glyma.07G069400.1	100	209	29	1	3580	3114	6681	0	5077

TRINITY_DN6339_c0_g2_i1	Glyma.12G052800.2	99.16666667	217	7	180	3779	1	3603	0	5398

TRINITY_DN14844_c0_g1_i2	Glyma.02G041400.3	100	471	23	484	4064	3594	1	0	3832

TRINITY_DN14844_c0_g1_i2	Glyma.02G041400.2	96	469	24	484	4064	3591	1	0	3825

TRINITY_DN6339_c0_g2_i1	Glyma.11G128300.2	98	223	4	180	3779	1	3588	0	5291

TRINITY_DN14563_c0_g1_i6	Glyma.18G218900.1	98.64864865	272	27	349	3917	3999	428	0	4771

TRINITY_DN15484_c0_g1_i5	Glyma.08G165900.3	96.82539683	380	33	539	4100	3574	1	0	4185

TRINITY_DN15484_c0_g1_i5	Glyma.08G165900.4	99.02912621	380	33	539	4100	3574	1	0	4185

TRINITY_DN15531_c0_g1_i3	Glyma.06G017700.1	98.7012987	214	7	64	3650	1	3593	0	5389

TRINITY_DN12822_c0_g1_i2	Glyma.18G009000.1	100	605	70	357	3926	3471	1	0	2335

TRINITY_DN12822_c0_g1_i2	Glyma.11G248200.1	100	581	75	357	3926	3462	1	0	2410

TRINITY_DN12822_c0_g1_i2	Glyma.11G248200.2	100	581	75	357	3926	3462	1	0	2410

TRINITY_DN12896_c0_g2_i1	Glyma.08G337200.1	95.83333333	248	13	1	3579	452	4039	0	5138

TRINITY_DN15531_c0_g1_i3	Glyma.04G017300.2	100	200	3	64	3650	1	3593	0	5489

TRINITY_DN15531_c0_g1_i3	Glyma.04G017300.1	98.7012987	200	3	64	3650	1	3593	0	5489

TRINITY_DN12822_c0_g1_i3	Glyma.18G009000.1	100	561	69	357	3923	3471	1	0	2593

TRINITY_DN12822_c0_g1_i3	Glyma.11G248200.1	100	543	74	357	3923	3462	1	0	2634

TRINITY_DN12822_c0_g1_i3	Glyma.11G248200.2	100	543	74	357	3923	3462	1	0	2634

TRINITY_DN12896_c0_g2_i1	Glyma.08G337200.2	95.83333333	248	12	1	3579	452	4036	0	5153

TRINITY_DN16374_c1_g1_i5	Glyma.18G234700.1	100	353	19	1	3571	3099	6669	0	4440

TRINITY_DN16309_c0_g1_i1	Glyma.11G033600.2	98.94736842	284	33	2997	6496	2835	6408	0	4416

TRINITY_DN16309_c0_g1_i1	Glyma.11G033600.1	98.94366197	284	33	2997	6496	2835	6408	0	4416

TRINITY_DN16309_c0_g1_i1	Glyma.11G033600.3	98.94459103	284	33	2997	6496	2835	6408	0	4416

TRINITY_DN16309_c0_g1_i1	Glyma.11G033600.4	98.94736842	284	33	2997	6496	2835	6408	0	4416

TRINITY_DN16309_c0_g1_i4	Glyma.11G033600.2	100	284	33	3063	6562	2835	6408	0	4416

TRINITY_DN16309_c0_g1_i4	Glyma.11G033600.1	95.45454545	284	33	3063	6562	2835	6408	0	4416

TRINITY_DN16309_c0_g1_i4	Glyma.11G033600.3	99.3006993	284	33	3063	6562	2835	6408	0	4416

TRINITY_DN16309_c0_g1_i4	Glyma.11G033600.4	99.31034483	284	33	3063	6562	2835	6408	0	4416

TRINITY_DN11843_c0_g1_i1	Glyma.03G220700.1	100	216	1	135	3720	1	3583	0	5406

TRINITY_DN11843_c0_g1_i1	Glyma.19G217500.1	100	202	1	135	3720	1	3583	0	5483

TRINITY_DN16039_c0_g1_i5	Glyma.09G066000.1	99.78118162	228	3	124	3708	1	3579	0	5319

TRINITY_DN16039_c0_g1_i5	Glyma.15G172100.1	99.78070175	218	1	124	3708	1	3582	0	5393

TRINITY_DN12822_c0_g1_i3	Glyma.08G102200.1	100	399	33	357	3923	3528	1	0	3947

TRINITY_DN12822_c0_g1_i3	Glyma.08G102200.2	100	399	33	357	3923	3528	1	0	3947

TRINITY_DN12822_c0_g1_i3	Glyma.08G102200.3	100	399	33	357	3923	3528	1	0	3947

TRINITY_DN12822_c0_g1_i3	Glyma.08G102200.4	100	399	33	357	3923	3528	1	0	3947

TRINITY_DN15226_c0_g1_i1	Glyma.17G005500.1	100	503	62	1	3545	1138	4664	0	3234

TRINITY_DN15226_c0_g1_i1	Glyma.07G268200.1	97.32142857	504	55	1	3545	1138	4664	0	3240

TRINITY_DN15226_c0_g1_i1	Glyma.07G268200.2	97.32142857	504	55	1	3545	1018	4544	0	3240

TRINITY_DN15226_c0_g1_i1	Glyma.07G268200.3	97.32142857	504	55	1	3545	673	4199	0	3240

TRINITY_DN16169_c0_g1_i1	Glyma.20G242400.1	97	386	16	1179	4751	3540	1	0	4157

TRINITY_DN16169_c0_g1_i1	Glyma.20G242400.2	98.30508475	386	16	1179	4751	3540	1	0	4157

TRINITY_DN12822_c0_g1_i2	Glyma.08G102200.1	100	363	28	357	3926	3528	1	0	4189

TRINITY_DN12822_c0_g1_i2	Glyma.08G102200.2	100	363	28	357	3926	3528	1	0	4189

TRINITY_DN12822_c0_g1_i2	Glyma.08G102200.3	100	363	28	357	3926	3528	1	0	4189

TRINITY_DN12822_c0_g1_i2	Glyma.08G102200.4	100	363	28	357	3926	3528	1	0	4189

TRINITY_DN16438_c0_g1_i8	Glyma.02G101800.1	95.98765432	311	14	132	3699	63	3633	0	4741

TRINITY_DN9051_c1_g2_i1	Glyma.18G297900.1	97.11934156	220	8	4	3581	6404	2836	0	5297

TRINITY_DN16169_c0_g1_i1	Glyma.10G292400.3	96.15384615	385	16	1179	4751	3543	1	0	4205

TRINITY_DN16169_c0_g1_i1	Glyma.10G292400.1	96.0443038	385	16	1179	4751	3543	1	0	4205

TRINITY_DN16398_c1_g1_i1	Glyma.18G280500.3	100	262	14	255	3806	1	3576	0	4975

TRINITY_DN16398_c1_g1_i1	Glyma.18G280500.1	100	262	14	255	3806	1	3576	0	4975

TRINITY_DN16398_c1_g1_i1	Glyma.18G280500.2	100	262	14	255	3806	1	3576	0	4975

TRINITY_DN16168_c0_g1_i2	Glyma.16G219100.2	100	507	24	620	4182	3540	5	0	3441

TRINITY_DN16168_c0_g1_i2	Glyma.16G219100.3	97.10144928	507	24	620	4182	3540	5	0	3441

TRINITY_DN16168_c0_g1_i2	Glyma.16G219100.1	97.10144928	507	24	620	4182	3540	5	0	3441

TRINITY_DN16168_c0_g1_i6	Glyma.16G219100.2	97.22814499	488	24	524	4086	3540	5	0	3546

TRINITY_DN16168_c0_g1_i6	Glyma.16G219100.3	100	488	24	524	4086	3540	5	0	3546

TRINITY_DN16168_c0_g1_i6	Glyma.16G219100.1	98.30508475	488	24	524	4086	3540	5	0	3546

TRINITY_DN16286_c0_g1_i2	Glyma.16G182500.1	97.47706422	216	0	667	4243	3612	36	0	5409

TRINITY_DN16286_c0_g1_i3	Glyma.16G182500.2	98.8372093	216	0	700	4276	3612	36	0	5409

TRINITY_DN16309_c0_g1_i1	Glyma.01G208600.1	98.94736842	276	36	2999	6490	2842	6405	0	4455

TRINITY_DN16309_c0_g1_i4	Glyma.01G208600.1	100	276	36	3065	6556	2842	6405	0	4455

TRINITY_DN16398_c1_g1_i1	Glyma.08G256300.1	100	250	8	255	3806	1	3576	0	5064

TRINITY_DN14956_c0_g1_i1	Glyma.06G147600.1	98.11320755	466	33	293	3838	3555	1	0	3703

TRINITY_DN13895_c0_g1_i2	Glyma.13G145100.3	100	298	17	346	3874	3568	1	0	4608

TRINITY_DN13895_c0_g1_i2	Glyma.13G145100.1	99.55357143	298	17	346	3874	3568	1	0	4608

TRINITY_DN13895_c0_g1_i2	Glyma.13G145100.2	97.01492537	298	17	346	3874	3568	1	0	4608

TRINITY_DN16168_c0_g1_i2	Glyma.09G169500.1	95.08196721	482	29	620	4182	3516	5	0	3458

TRINITY_DN16168_c0_g1_i6	Glyma.09G169500.1	96.2962963	463	29	524	4086	3516	5	0	3563

TRINITY_DN13956_c0_g1_i2	Glyma.09G196100.1	99.46236559	269	14	83	3633	1	3572	0	4948

TRINITY_DN15334_c0_g1_i1	Glyma.04G204800.1	99.46808511	199	9	384	3953	3543	1	0	5304

TRINITY_DN15334_c0_g1_i1	Glyma.06G160800.1	99.46808511	191	2	384	3953	3567	1	0	5515

TRINITY_DN16245_c0_g5_i2	Glyma.05G015400.1	100	276	7	347	3910	3561	1	0	5011

TRINITY_DN13956_c0_g1_i2	Glyma.16G112900.1	99.46236559	269	12	83	3633	1	3563	0	4990

TRINITY_DN10035_c0_g2_i1	Glyma.02G012900.2	98.16513761	259	7	1	3550	750	4308	0	5024

TRINITY_DN16245_c0_g5_i1	Glyma.05G015400.1	96.2406015	241	3	347	3910	3561	1	0	5227

TRINITY_DN13895_c0_g1_i2	Glyma.10G058300.1	97.59036145	316	18	346	3874	3541	1	0	4479

TRINITY_DN13895_c0_g1_i2	Glyma.10G058300.3	97.59036145	316	18	346	3874	3541	1	0	4479

TRINITY_DN13895_c0_g1_i2	Glyma.10G058300.2	95.09803922	316	18	346	3874	3541	1	0	4479

TRINITY_DN14023_c0_g2_i1	Glyma.17G120100.2	100	219	1	169	3727	1	3562	0	5345

TRINITY_DN14023_c0_g2_i1	Glyma.05G011900.1	95.8041958	212	3	169	3726	1	3561	0	5371

TRINITY_DN14023_c0_g2_i1	Glyma.17G120100.1	100	219	1	169	3726	1	3561	0	5343

TRINITY_DN14023_c0_g2_i2	Glyma.17G120100.2	97.85002622	219	1	169	3726	1	3561	0	5343

TRINITY_DN14023_c0_g2_i1	Glyma.05G011900.2	95.81151832	212	3	169	3725	1	3560	0	5369

TRINITY_DN10864_c0_g1_i1	Glyma.13G057300.1	98.52941176	238	3	1567	5123	4253	724	0	5075

TRINITY_DN15748_c0_g1_i2	Glyma.08G283700.1	97.36842105	202	1	343	3898	4380	828	0	5428

TRINITY_DN16158_c0_g1_i5	Glyma.20G001300.1	100	194	6	347	3887	3531	1	0	5234

TRINITY_DN16158_c0_g1_i5	Glyma.20G001300.2	100	194	6	347	3887	3531	1	0	5234

TRINITY_DN16158_c0_g1_i5	Glyma.09G284800.1	96.15384615	190	9	347	3887	3513	1	0	5140

TRINITY_DN15748_c0_g1_i2	Glyma.18G142500.1	95.75471698	191	1	344	3898	4379	828	0	5487

TRINITY_DN15748_c0_g1_i2	Glyma.18G142500.2	100	191	1	344	3898	4379	828	0	5487

TRINITY_DN16329_c1_g1_i3	Glyma.12G170100.1	98.33333333	279	1	515	4066	3549	1	0	4994

TRINITY_DN16329_c1_g1_i3	Glyma.08G188000.1	98.33333333	259	1	515	4066	3549	1	0	5105

TRINITY_DN326_c0_g1_i2	Glyma.03G253000.4	97.95918367	122	1	416	3964	1	3552	0	5864

TRINITY_DN13662_c0_g1_i1	Glyma.11G094300.1	100	224	2	345	3893	3549	1	0	5302

TRINITY_DN13662_c0_g1_i1	Glyma.12G020700.1	100	227	0	345	3893	3549	1	0	5297

TRINITY_DN277_c0_g1_i1	Glyma.07G249800.1	100	235	6	375	3911	3546	1	0	5166

TRINITY_DN277_c0_g1_i1	Glyma.07G249800.2	100	235	6	375	3911	3546	1	0	5166

TRINITY_DN277_c0_g1_i1	Glyma.07G249800.3	100	235	6	375	3911	3546	1	0	5166

TRINITY_DN277_c0_g1_i1	Glyma.17G024600.2	98.85057471	230	5	375	3911	3525	1	0	5070

TRINITY_DN277_c0_g1_i1	Glyma.17G024600.1	95.19230769	230	5	375	3911	3525	1	0	5070

TRINITY_DN16114_c0_g1_i3	Glyma.11G009500.2	97.93103448	215	25	136	3649	1	3532	0	5049

TRINITY_DN15815_c0_g2_i2	Glyma.12G147000.1	97.6744186	323	18	466	3984	3543	1	0	4566

TRINITY_DN15815_c0_g2_i2	Glyma.12G147000.3	100	321	21	466	3984	3540	1	0	4556

TRINITY_DN16168_c0_g1_i5	Glyma.16G219100.2	100	256	7	620	4159	3540	1	0	5053

TRINITY_DN16168_c0_g1_i5	Glyma.16G219100.3	96.05055292	256	7	620	4159	3540	1	0	5053

TRINITY_DN16168_c0_g1_i5	Glyma.16G219100.1	97.82082324	256	7	620	4159	3540	1	0	5053

TRINITY_DN16168_c0_g1_i8	Glyma.16G219100.2	96.42857143	237	7	524	4063	3540	1	0	5158

TRINITY_DN16168_c0_g1_i8	Glyma.16G219100.3	96.42857143	237	7	524	4063	3540	1	0	5158

TRINITY_DN16168_c0_g1_i8	Glyma.16G219100.1	96.42857143	237	7	524	4063	3540	1	0	5158

TRINITY_DN16158_c0_g1_i5	Glyma.20G102900.1	100	428	18	347	3887	3513	1	0	3943

TRINITY_DN16158_c0_g1_i5	Glyma.20G102900.2	98.84169884	428	18	347	3887	3513	1	0	3943

TRINITY_DN16308_c1_g3_i2	Glyma.13G071900.1	100	658	42	132	3654	1	3520	0	2608

TRINITY_DN16016_c0_g1_i1	Glyma.10G221500.1	100	191	7	408	3945	3534	6	0	5350

TRINITY_DN16016_c0_g1_i1	Glyma.10G221500.3	95.85253456	191	7	408	3945	3534	6	0	5350

TRINITY_DN16016_c0_g1_i1	Glyma.10G221500.2	97.36842105	191	7	408	3945	3534	6	0	5350

TRINITY_DN16016_c0_g1_i3	Glyma.10G221500.1	97.83549784	191	7	408	3945	3534	6	0	5350

TRINITY_DN16016_c0_g1_i3	Glyma.10G221500.3	100	191	7	408	3945	3534	6	0	5350

TRINITY_DN16016_c0_g1_i3	Glyma.10G221500.2	98.57142857	191	7	408	3945	3534	6	0	5350

TRINITY_DN16016_c0_g1_i1	Glyma.20G170000.1	96.42857143	191	6	408	3940	3537	11	0	5328

TRINITY_DN16016_c0_g1_i3	Glyma.20G170000.1	97.63157895	191	6	408	3940	3537	11	0	5328

TRINITY_DN16168_c0_g1_i5	Glyma.09G169500.1	100	232	8	620	4159	3516	1	0	5097

TRINITY_DN16168_c0_g1_i8	Glyma.09G169500.1	95.78947368	213	8	524	4063	3516	1	0	5203

TRINITY_DN12822_c0_g1_i1	Glyma.18G009000.1	100	336	29	361	3879	3471	1	0	4128

TRINITY_DN12822_c0_g1_i1	Glyma.11G248200.1	100	314	31	361	3879	3462	1	0	4191

TRINITY_DN12822_c0_g1_i1	Glyma.11G248200.2	100	314	31	361	3879	3462	1	0	4191

TRINITY_DN14844_c0_g1_i2	Glyma.18G146600.1	97.87234043	248	6	484	4002	3537	1	0	5042

TRINITY_DN12822_c0_g1_i4	Glyma.18G009000.1	100	292	28	361	3876	3471	1	0	4385

TRINITY_DN12822_c0_g1_i4	Glyma.11G248200.1	100	276	30	361	3876	3462	1	0	4416

TRINITY_DN12822_c0_g1_i4	Glyma.11G248200.2	100	276	30	361	3876	3462	1	0	4416

TRINITY_DN16158_c0_g1_i7	Glyma.20G001300.1	100	198	5	72	3590	3531	1	0	5313

TRINITY_DN16158_c0_g1_i7	Glyma.20G001300.2	100	198	5	72	3590	3531	1	0	5313

TRINITY_DN16158_c0_g1_i4	Glyma.20G001300.1	100	194	5	347	3865	3531	1	0	5336

TRINITY_DN16158_c0_g1_i4	Glyma.20G001300.2	100	194	5	347	3865	3531	1	0	5336

TRINITY_DN16158_c0_g1_i7	Glyma.09G284800.1	99.27007299	192	6	72	3590	3513	1	0	5230

TRINITY_DN16158_c0_g1_i4	Glyma.09G284800.1	100	190	6	347	3865	3513	1	0	5241

TRINITY_DN16245_c0_g5_i1	Glyma.06G208900.1	98.96907216	506	19	381	3901	3533	13	0	3578

TRINITY_DN16245_c0_g5_i1	Glyma.06G208900.4	98.96907216	506	19	381	3901	3533	13	0	3578

TRINITY_DN16308_c1_g3_i2	Glyma.20G050700.1	100	639	44	146	3654	9	3502	0	2599

TRINITY_DN14575_c0_g1_i1	Glyma.14G135900.1	100	303	0	3263	6793	5457	1927	0	4843

TRINITY_DN14575_c0_g1_i1	Glyma.14G135900.2	100	303	0	3263	6793	5433	1903	0	4843

TRINITY_DN8943_c0_g1_i1	Glyma.03G226200.2	100	191	5	953	4482	3522	1	0	5398

TRINITY_DN16245_c0_g5_i2	Glyma.06G208900.1	98.7654321	455	16	381	3901	3533	13	0	3882

TRINITY_DN16245_c0_g5_i2	Glyma.06G208900.4	99.15254237	455	16	381	3901	3533	13	0	3882

TRINITY_DN16245_c0_g5_i1	Glyma.04G144900.1	95.9446093	515	15	381	3898	3530	13	0	3555

TRINITY_DN16245_c0_g5_i2	Glyma.04G144900.1	96.66666667	466	15	381	3898	3530	13	0	3827

TRINITY_DN15815_c0_g2_i2	Glyma.06G254500.1	98.8372093	331	5	466	3984	3525	1	0	4626

TRINITY_DN16158_c0_g1_i4	Glyma.20G102900.1	98.80952381	430	11	347	3865	3513	1	0	4034

TRINITY_DN16158_c0_g1_i4	Glyma.20G102900.2	98.80952381	430	11	347	3865	3513	1	0	4034

TRINITY_DN16158_c0_g1_i7	Glyma.20G102900.1	96.72801636	428	11	72	3590	3513	1	0	4045

TRINITY_DN16158_c0_g1_i7	Glyma.20G102900.2	97.02970297	428	11	72	3590	3513	1	0	4045

TRINITY_DN14287_c0_g1_i1	Glyma.15G108000.1	100	259	21	385	3863	3501	2	0	4638

TRINITY_DN15328_c0_g4_i1	Glyma.16G025800.1	98.27586207	597	68	1	3450	529	3986	0	2318

TRINITY_DN15328_c0_g4_i1	Glyma.16G025800.2	98.27586207	596	69	1	3450	529	3983	0	2303

TRINITY_DN16286_c0_g1_i3	Glyma.10G234500.1	100	472	12	700	4214	3744	230	0	3799

TRINITY_DN6168_c0_g1_i1	Glyma.20G227000.2	97.91666667	252	12	109	3616	22	3520	0	4879

TRINITY_DN15914_c0_g2_i1	Glyma.14G044900.3	96.15384615	207	1	274	3785	3577	60	0	5312

TRINITY_DN5301_c0_g1_i1	Glyma.04G232700.1	99.00990099	239	2	248	3761	7179	3666	0	5155

TRINITY_DN5301_c0_g1_i1	Glyma.04G232700.2	100	239	2	248	3761	5541	2028	0	5155

TRINITY_DN5301_c0_g1_i1	Glyma.06G132300.1	95.6284153	256	0	248	3761	7182	3669	0	5072

TRINITY_DN15634_c2_g1_i3	Glyma.20G187400.1	98.82352941	348	16	1272	4769	3501	1	0	4385

TRINITY_DN12946_c0_g1_i3	Glyma.13G156500.6	97.61904762	274	11	421	3915	4712	1212	0	4798

TRINITY_DN12946_c0_g1_i3	Glyma.13G156500.4	98.9010989	274	11	421	3915	4712	1212	0	4798

TRINITY_DN10648_c0_g1_i2	Glyma.10G141400.1	100	333	26	133	3608	3576	86	0	4294

TRINITY_DN15515_c0_g1_i1	Glyma.19G149800.2	100	270	7	537	4029	222	3729	0	4874

TRINITY_DN15515_c0_g1_i2	Glyma.19G149800.2	96.07142857	270	7	564	4056	222	3729	0	4874

TRINITY_DN12946_c0_g1_i2	Glyma.13G156500.6	100	274	11	421	3910	4712	1212	0	4820

TRINITY_DN12946_c0_g1_i2	Glyma.13G156500.4	98.93048128	274	11	421	3910	4712	1212	0	4820

TRINITY_DN15328_c0_g4_i1	Glyma.07G056700.3	98.38709677	597	60	1	3450	529	3968	0	2412

TRINITY_DN15328_c0_g4_i1	Glyma.07G056700.1	98.38709677	597	60	1	3450	529	3968	0	2412

TRINITY_DN15328_c0_g4_i1	Glyma.07G056700.2	98.27586207	596	61	1	3450	529	3965	0	2398

TRINITY_DN10648_c0_g1_i1	Glyma.10G141400.1	97.36842105	264	16	133	3602	3576	86	0	4715

TRINITY_DN16245_c0_g3_i4	Glyma.04G166100.1	97.54098361	220	1	375	3877	3500	1	0	5230

TRINITY_DN16245_c0_g3_i4	Glyma.08G268900.1	98.71794872	504	23	301	3792	3569	86	0	3494

TRINITY_DN16345_c0_g1_i5	Glyma.07G093700.2	98.4375	169	5	642	4138	3527	40	0	5463

TRINITY_DN14956_c0_g1_i1	Glyma.04G218300.1	98.11320755	442	24	293	3764	3564	84	0	3766

TRINITY_DN14803_c0_g1_i1	Glyma.16G060500.1	97.08431836	250	8	270	3752	3495	1	0	4992

TRINITY_DN15784_c0_g1_i2	Glyma.02G214300.1	96.49122807	255	25	1	3462	34	3492	0	4617

TRINITY_DN15784_c0_g1_i5	Glyma.02G214300.1	95.83333333	255	25	1	3462	34	3492	0	4617

TRINITY_DN15784_c0_g1_i8	Glyma.02G214300.1	98.27833572	255	25	1	3462	34	3492	0	4617

TRINITY_DN14803_c0_g1_i1	Glyma.19G086600.1	99.31972789	233	10	270	3752	3486	1	0	5072

TRINITY_DN15242_c0_g1_i1	Glyma.20G086700.1	99.31506849	377	25	221	3680	3687	228	0	4036

TRINITY_DN14817_c0_g1_i1	Glyma.17G071400.2	97.31343284	369	24	937	4395	1	3471	0	4156

TRINITY_DN14817_c0_g1_i1	Glyma.17G071400.1	97.31343284	369	24	937	4395	1	3471	0	4156

TRINITY_DN11068_c0_g1_i1	Glyma.06G083500.1	99.76798144	336	18	560	4002	3567	107	0	4170

TRINITY_DN11068_c0_g1_i2	Glyma.06G083500.1	98.18181818	336	18	470	3912	3567	107	0	4170

TRINITY_DN15242_c0_g1_i1	Glyma.20G086700.2	100	377	25	221	3678	3606	149	0	4032

TRINITY_DN16374_c1_g1_i2	Glyma.18G234700.1	100	442	30	1	3456	3099	6554	0	3657

TRINITY_DN14654_c0_g1_i6	Glyma.01G155300.1	98.8372093	509	21	522	3994	3621	167	0	3411

TRINITY_DN11068_c0_g1_i1	Glyma.04G081800.1	96.35722679	332	19	560	4002	3534	107	0	4023

TRINITY_DN11068_c0_g1_i2	Glyma.04G081800.1	100	332	19	470	3912	3534	107	0	4023

TRINITY_DN15242_c0_g1_i2	Glyma.20G086700.1	100	226	14	221	3683	3687	228	0	4957

TRINITY_DN14817_c0_g1_i2	Glyma.17G071400.2	99.82517483	234	5	937	4407	1	3471	0	5048

TRINITY_DN14817_c0_g1_i2	Glyma.17G071400.1	100	234	5	937	4407	1	3471	0	5048

TRINITY_DN14654_c0_g1_i6	Glyma.11G089400.2	100	521	18	522	3992	3621	169	0	3352

TRINITY_DN14654_c0_g1_i6	Glyma.11G089400.1	100	521	18	522	3992	3621	169	0	3352

TRINITY_DN14654_c0_g1_i6	Glyma.11G089400.3	95.36423841	521	18	522	3992	3621	169	0	3352

TRINITY_DN15242_c0_g1_i2	Glyma.20G086700.2	100	226	14	221	3681	3606	149	0	4953

TRINITY_DN14654_c0_g1_i6	Glyma.16G111200.3	95.53571429	224	5	522	3996	3633	165	0	5127

TRINITY_DN14654_c0_g1_i6	Glyma.16G111200.2	100	224	5	522	3996	3633	165	0	5127

TRINITY_DN14654_c0_g1_i6	Glyma.16G111200.1	100	224	5	522	3996	3633	165	0	5127

TRINITY_DN15923_c0_g1_i1	Glyma.11G040200.1	95.85253456	165	5	1	3470	341	3807	0	5408

TRINITY_DN13931_c0_g1_i1	Glyma.19G189100.5	98.63013699	206	2	912	4385	3474	1	0	5264

TRINITY_DN13931_c0_g1_i1	Glyma.19G189100.3	98.75	206	2	912	4385	3474	1	0	5264

TRINITY_DN13931_c0_g1_i1	Glyma.19G189100.1	96.11650485	206	2	912	4385	3474	1	0	5264

TRINITY_DN13931_c0_g1_i1	Glyma.19G189100.2	96.11650485	206	2	912	4385	3474	1	0	5264

TRINITY_DN13931_c0_g1_i1	Glyma.19G189100.4	100	206	2	912	4385	3474	1	0	5264

TRINITY_DN14654_c0_g1_i6	Glyma.09G194900.1	95.53571429	203	6	523	3996	3626	165	0	5203

TRINITY_DN14817_c0_g1_i1	Glyma.02G206500.1	98.93617021	385	15	937	4395	1	3465	0	4128

TRINITY_DN14817_c0_g1_i1	Glyma.02G206500.2	95.94594595	385	15	937	4395	1	3465	0	4128

TRINITY_DN14817_c0_g1_i2	Glyma.02G206500.1	95.94594595	236	3	937	4407	1	3465	0	5064

TRINITY_DN14817_c0_g1_i2	Glyma.02G206500.2	98.29059829	236	3	937	4407	1	3465	0	5064

TRINITY_DN15923_c0_g1_i1	Glyma.01G202600.1	96.25668449	164	0	1	3470	332	3801	0	5500

TRINITY_DN12099_c0_g1_i1	Glyma.06G073200.1	100	327	28	196	3612	1	3432	0	4019

TRINITY_DN5846_c0_g1_i1	Glyma.12G024600.1	100	280	7	1	3460	2436	5901	0	4789

TRINITY_DN15867_c0_g3_i2	Glyma.17G052600.2	100	273	10	615	4058	1	3456	0	4682

TRINITY_DN13433_c0_g1_i1	Glyma.10G024500.1	100	372	10	2	3454	3449	1	0	4157

TRINITY_DN16335_c0_g2_i1	Glyma.09G273900.1	97.90794979	299	2	700	4161	3894	430	0	4723

TRINITY_DN14659_c2_g1_i4	Glyma.12G038900.3	99.21259843	531	11	493	3940	3465	7	0	3319

TRINITY_DN14659_c2_g1_i4	Glyma.12G038900.5	99.21259843	531	11	493	3940	3465	7	0	3319

TRINITY_DN16335_c0_g2_i1	Glyma.18G213100.5	97.91666667	321	1	700	4161	3891	436	0	4577

TRINITY_DN16335_c0_g2_i1	Glyma.18G213100.1	97.90026247	321	1	700	4161	3891	436	0	4577

TRINITY_DN16335_c0_g2_i1	Glyma.18G213100.2	97.91666667	321	1	700	4161	3891	436	0	4577

TRINITY_DN16335_c0_g2_i1	Glyma.18G213100.3	97.91666667	321	1	700	4161	3891	436	0	4577

TRINITY_DN16335_c0_g2_i1	Glyma.18G213100.4	97.90575916	321	1	700	4161	3891	436	0	4577

TRINITY_DN14659_c2_g1_i1	Glyma.12G038900.3	96.1038961	409	6	332	3779	3465	7	0	4028

TRINITY_DN14659_c2_g1_i1	Glyma.12G038900.5	98.50746269	409	6	332	3779	3465	7	0	4028

TRINITY_DN14659_c2_g1_i3	Glyma.12G038900.3	100	316	5	493	3950	3465	8	0	4602

TRINITY_DN14659_c2_g1_i3	Glyma.12G038900.5	97.19626168	316	5	493	3950	3465	8	0	4602

TRINITY_DN15861_c0_g2_i1	Glyma.13G229200.1	100	259	7	424	3873	10	3459	0	4837

TRINITY_DN15861_c0_g2_i2	Glyma.13G229200.1	100	259	7	552	4001	10	3459	0	4837

TRINITY_DN13595_c0_g1_i1	Glyma.03G128300.3	100	146	0	440	3898	3459	1	0	5579

TRINITY_DN9338_c0_g1_i1	Glyma.15G019200.3	95.40229885	535	21	896	4344	3442	3	0	3247

TRINITY_DN14659_c2_g1_i2	Glyma.12G038900.3	97.60956175	194	0	332	3789	3465	8	0	5312

TRINITY_DN14659_c2_g1_i2	Glyma.12G038900.5	100	194	0	332	3789	3465	8	0	5312

TRINITY_DN16245_c0_g3_i4	Glyma.16G099700.1	96	492	25	399	3841	3473	31	0	3478

TRINITY_DN15242_c0_g1_i2	Glyma.20G202100.2	100	481	45	221	3637	3423	1	0	3264

TRINITY_DN15242_c0_g1_i2	Glyma.20G202100.1	100	481	45	221	3637	3423	1	0	3264

TRINITY_DN8232_c0_g1_i1	Glyma.08G196100.1	96.9924812	346	16	1	3433	240	3684	0	4268

TRINITY_DN16430_c0_g2_i6	Glyma.11G033000.2	99.44134078	307	20	392	3827	1	3423	0	4372

TRINITY_DN15242_c0_g1_i2	Glyma.10G188600.1	96.82539683	497	39	221	3637	3426	1	0	3227

TRINITY_DN7713_c0_g1_i1	Glyma.13G370300.1	99.26470588	207	2	199	3647	956	4407	0	5208

TRINITY_DN8232_c0_g1_i1	Glyma.07G012400.1	100	355	15	1	3433	246	3687	0	4231

TRINITY_DN5423_c0_g1_i1	Glyma.15G219200.1	100	215	8	627	4072	3762	341	0	4985

TRINITY_DN15242_c0_g1_i1	Glyma.20G202100.2	97.10144928	374	32	221	3634	3423	1	0	3914

TRINITY_DN15242_c0_g1_i1	Glyma.20G202100.1	95.67567568	374	32	221	3634	3423	1	0	3914

TRINITY_DN14391_c0_g1_i1	Glyma.09G174000.1	95.50561798	172	15	569	3992	3909	465	0	5240

TRINITY_DN14391_c0_g1_i2	Glyma.09G174000.1	95.50561798	172	15	483	3906	3909	465	0	5240

TRINITY_DN15242_c0_g1_i1	Glyma.10G188600.1	100	365	31	221	3634	3426	1	0	4006

TRINITY_DN5423_c0_g1_i1	Glyma.15G219200.2	97.61904762	216	7	627	4072	3759	341	0	4992

TRINITY_DN14391_c0_g1_i1	Glyma.07G103400.1	95.50561798	178	11	569	3992	3894	459	0	5182

TRINITY_DN14391_c0_g1_i2	Glyma.07G103400.1	95.50561798	178	11	483	3906	3894	459	0	5182

TRINITY_DN8107_c0_g2_i1	Glyma.05G168600.6	99.15966387	489	26	533	3968	501	3915	0	3411

TRINITY_DN8107_c0_g2_i1	Glyma.05G168600.7	100	489	26	533	3968	501	3915	0	3411

TRINITY_DN10533_c0_g1_i1	Glyma.17G002500.1	97.95918367	277	15	72	3489	282	3711	0	4578

TRINITY_DN10533_c0_g1_i1	Glyma.07G271700.1	97.95081967	259	18	81	3489	300	3723	0	4580

TRINITY_DN15242_c0_g1_i1	Glyma.10G008700.1	95	374	24	221	3634	3420	1	0	3995

TRINITY_DN15242_c0_g1_i1	Glyma.20G086700.3	95	370	25	221	3634	3414	1	0	3989

TRINITY_DN9537_c0_g1_i1	Glyma.04G191900.1	96.95431472	281	9	31	3466	393	3804	0	4623

TRINITY_DN16245_c0_g2_i1	Glyma.05G006800.1	96.66666667	316	20	318	3741	3585	162	0	4440

TRINITY_DN16245_c0_g2_i1	Glyma.19G007400.1	96.7032967	312	24	318	3741	3570	162	0	4364

TRINITY_DN9262_c0_g1_i2	Glyma.05G212000.1	96.69421488	232	16	1	3396	616	4044	0	4756

TRINITY_DN15642_c0_g1_i3	Glyma.11G010400.1	98.50746269	219	3	218	3652	1	3432	0	5099

TRINITY_DN15642_c0_g1_i4	Glyma.11G010400.1	96	219	3	292	3726	1	3432	0	5099

TRINITY_DN2629_c0_g1_i1	Glyma.08G205400.1	96	267	12	263	3661	3426	1	0	4573

TRINITY_DN15642_c0_g1_i3	Glyma.01G232500.2	100	223	1	218	3652	1	3432	0	5088

TRINITY_DN15642_c0_g1_i3	Glyma.01G232500.1	100	223	1	218	3652	1	3432	0	5088

TRINITY_DN15642_c0_g1_i4	Glyma.01G232500.2	99.72451791	223	1	292	3726	1	3432	0	5088

TRINITY_DN15642_c0_g1_i4	Glyma.01G232500.1	100	223	1	292	3726	1	3432	0	5088

TRINITY_DN16345_c0_g1_i2	Glyma.09G182600.1	95.3271028	180	3	440	3874	3717	293	0	5282

TRINITY_DN16345_c0_g1_i2	Glyma.07G093700.1	95.3271028	176	2	440	3874	3729	299	0	5343

TRINITY_DN16345_c0_g1_i2	Glyma.08G044700.1	95.33333333	439	16	446	3870	3699	281	0	3755

TRINITY_DN16345_c0_g1_i2	Glyma.08G044700.6	95.3307393	439	16	446	3869	3420	3	0	3753

TRINITY_DN16345_c0_g1_i5	Glyma.08G044700.6	98.46153846	439	16	446	3869	3420	3	0	3753

TRINITY_DN16438_c0_g1_i6	Glyma.02G101800.1	95.31680441	294	19	134	3552	1	3417	0	4518

TRINITY_DN326_c0_g1_i2	Glyma.03G253000.2	97.96747967	118	1	134	3563	1	3433	0	5666

TRINITY_DN15242_c0_g1_i2	Glyma.20G086700.3	98.35526316	219	14	221	3637	3414	1	0	4911

TRINITY_DN16428_c1_g1_i2	Glyma.18G210300.3	100	166	2	993	4424	3438	22	0	5321

TRINITY_DN16428_c1_g1_i2	Glyma.18G210300.2	100	166	2	993	4424	3417	1	0	5321

TRINITY_DN16345_c0_g1_i2	Glyma.05G237500.1	95.3271028	425	10	446	3870	3705	281	0	3904

TRINITY_DN15242_c0_g1_i2	Glyma.10G008700.1	95.39641944	230	9	221	3637	3420	1	0	4911

TRINITY_DN5160_c0_g1_i2	Glyma.02G219200.4	100	216	4	217	3644	1087	4505	0	5075

TRINITY_DN16428_c1_g1_i1	Glyma.18G210300.1	100	172	2	510	3937	4098	686	0	5280

TRINITY_DN16428_c1_g1_i1	Glyma.18G210300.3	100	172	2	510	3937	3777	365	0	5280

TRINITY_DN16428_c1_g1_i1	Glyma.18G210300.2	100	172	2	510	3937	3756	344	0	5280

TRINITY_DN16428_c1_g1_i1	Glyma.09G278500.1	100	155	2	510	3937	4122	710	0	5374

TRINITY_DN11898_c0_g2_i1	Glyma.04G091700.1	98.75	302	18	134	3536	3414	1	0	4427

TRINITY_DN14287_c0_g1_i1	Glyma.13G204700.2	100	247	19	385	3762	3450	52	0	4529

TRINITY_DN14287_c0_g1_i1	Glyma.13G204700.1	100	247	19	385	3762	3450	52	0	4529

TRINITY_DN39762_c0_g1_i1	Glyma.18G174200.6	99.10714286	343	28	1	3374	20	3423	0	4015

TRINITY_DN39762_c0_g1_i1	Glyma.18G174200.5	99.11504425	343	28	1	3374	20	3423	0	4015

TRINITY_DN39762_c0_g1_i1	Glyma.18G174200.2	99.12280702	343	28	1	3374	20	3423	0	4015

TRINITY_DN39762_c0_g1_i1	Glyma.18G174200.3	99.10714286	343	28	1	3374	20	3423	0	4015

TRINITY_DN39762_c0_g1_i1	Glyma.18G174200.4	99.11894273	343	28	1	3374	20	3423	0	4015

TRINITY_DN39762_c0_g1_i1	Glyma.18G174200.1	99.12280702	343	28	1	3374	20	3423	0	4015

TRINITY_DN15994_c0_g1_i2	Glyma.15G006100.1	100	246	16	455	3862	3393	1	0	4700

TRINITY_DN15994_c0_g1_i2	Glyma.15G006100.2	100	246	16	455	3862	3393	1	0	4700

TRINITY_DN14656_c0_g1_i1	Glyma.20G107300.1	100	216	27	734	4141	3375	4	0	4732

TRINITY_DN14656_c0_g1_i1	Glyma.20G107300.3	97.36842105	216	27	734	4141	3375	4	0	4732

TRINITY_DN14656_c0_g1_i1	Glyma.10G282000.1	97.36842105	244	16	734	4138	3387	1	0	4676

TRINITY_DN14656_c0_g1_i1	Glyma.20G107300.2	100	215	27	734	4138	3369	1	0	4732

TRINITY_DN6086_c0_g2_i1	Glyma.03G131000.1	97.27891156	380	32	221	3602	96	3483	0	3825

TRINITY_DN15433_c0_g2_i3	Glyma.07G028800.3	100	277	15	63	3445	1	3407	0	4525

TRINITY_DN15433_c0_g2_i3	Glyma.07G028800.1	100	277	15	63	3445	1	3407	0	4525

TRINITY_DN15433_c0_g2_i3	Glyma.07G028800.4	96.72131148	277	15	63	3445	1	3407	0	4525

TRINITY_DN15433_c0_g2_i4	Glyma.07G028800.3	98.30508475	277	15	238	3620	1	3407	0	4525

TRINITY_DN15433_c0_g2_i4	Glyma.07G028800.1	98.1512605	277	15	238	3620	1	3407	0	4525

TRINITY_DN15433_c0_g2_i4	Glyma.07G028800.4	96.08695652	277	15	238	3620	1	3407	0	4525

TRINITY_DN15433_c0_g2_i3	Glyma.07G028800.2	96.07843137	275	16	63	3445	1	3401	0	4497

TRINITY_DN15433_c0_g2_i4	Glyma.07G028800.2	100	275	16	238	3620	1	3401	0	4497

TRINITY_DN15994_c0_g1_i2	Glyma.07G028000.1	96.20938628	258	13	455	3862	3393	1	0	4700

TRINITY_DN15994_c0_g1_i2	Glyma.08G214600.5	97.17138103	251	7	455	3862	3402	1	0	4798

TRINITY_DN15994_c0_g1_i2	Glyma.08G214600.7	96.77419355	251	7	455	3862	3402	1	0	4798

TRINITY_DN15994_c0_g1_i2	Glyma.08G214600.6	100	251	7	455	3862	3402	1	0	4798

TRINITY_DN15994_c0_g1_i2	Glyma.08G214600.4	98.04270463	251	7	455	3862	3402	1	0	4798

TRINITY_DN15994_c0_g1_i2	Glyma.08G214600.2	99.23664122	251	7	455	3862	3402	1	0	4798

TRINITY_DN15994_c0_g1_i2	Glyma.08G214600.1	96.32516704	251	7	455	3862	3402	1	0	4798

TRINITY_DN15994_c0_g1_i2	Glyma.08G214600.3	96.2962963	251	7	455	3862	3402	1	0	4798

TRINITY_DN14929_c0_g1_i2	Glyma.13G056200.1	99.43181818	379	20	250	3643	3423	33	0	3938

TRINITY_DN39762_c0_g1_i1	Glyma.08G356500.3	99.11504425	327	33	6	3374	22	3417	0	4108

TRINITY_DN39762_c0_g1_i1	Glyma.08G356500.2	99.10714286	327	33	6	3374	22	3417	0	4108

TRINITY_DN39762_c0_g1_i1	Glyma.08G356500.1	99.1202346	327	33	6	3374	22	3417	0	4108

TRINITY_DN14929_c0_g1_i2	Glyma.19G030400.1	99.42196532	367	26	250	3643	3396	12	0	3988

TRINITY_DN15994_c0_g1_i3	Glyma.15G006100.1	97.65166341	349	20	455	3850	3393	1	0	4174

TRINITY_DN15994_c0_g1_i3	Glyma.15G006100.2	97.31343284	349	20	455	3850	3393	1	0	4174

TRINITY_DN15994_c0_g1_i2	Glyma.13G367300.1	97.16981132	235	5	455	3862	3399	1	0	4933

TRINITY_DN15340_c0_g1_i1	Glyma.17G112500.1	98.42519685	162	3	527	3928	3405	1	0	5337

TRINITY_DN6086_c0_g2_i1	Glyma.19G133000.1	97.27626459	387	37	221	3602	96	3459	0	3720

TRINITY_DN15994_c0_g1_i3	Glyma.13G367300.1	96.20253165	328	7	455	3850	3399	1	0	4362

TRINITY_DN10648_c0_g1_i2	Glyma.10G141400.4	100	316	19	133	3510	3393	1	0	4274

TRINITY_DN10648_c0_g1_i2	Glyma.10G141400.3	100	316	19	133	3510	3393	1	0	4274

TRINITY_DN10648_c0_g1_i2	Glyma.10G141400.2	95.56650246	316	19	133	3510	3393	1	0	4274

TRINITY_DN5748_c0_g1_i1	Glyma.14G008200.2	99.38650307	242	3	82	3486	1	3396	0	4889

TRINITY_DN15994_c0_g1_i3	Glyma.08G214600.5	97.76536313	170	2	455	3850	3402	1	0	5269

TRINITY_DN15994_c0_g1_i3	Glyma.08G214600.7	99.07407407	170	2	455	3850	3402	1	0	5269

TRINITY_DN15994_c0_g1_i3	Glyma.08G214600.6	100	170	2	455	3850	3402	1	0	5269

TRINITY_DN15994_c0_g1_i3	Glyma.08G214600.4	100	170	2	455	3850	3402	1	0	5269

TRINITY_DN15994_c0_g1_i3	Glyma.08G214600.2	99.08256881	170	2	455	3850	3402	1	0	5269

TRINITY_DN15994_c0_g1_i3	Glyma.08G214600.1	100	170	2	455	3850	3402	1	0	5269

TRINITY_DN15994_c0_g1_i3	Glyma.08G214600.3	100	170	2	455	3850	3402	1	0	5269

TRINITY_DN15994_c0_g1_i3	Glyma.07G028000.1	97.78534923	170	8	455	3850	3393	1	0	5210

TRINITY_DN15340_c0_g1_i1	Glyma.13G158800.1	99.27007299	160	4	527	3928	3402	1	0	5363

TRINITY_DN16352_c0_g2_i3	Glyma.08G190700.2	100	532	36	1166	4523	3642	246	0	2996

TRINITY_DN16151_c0_g2_i3	Glyma.08G292900.1	100	443	39	872	4236	830	4179	0	3230

TRINITY_DN16151_c0_g2_i3	Glyma.08G292900.5	100	443	39	872	4236	830	4179	0	3230

TRINITY_DN16151_c0_g2_i5	Glyma.08G292900.1	96.77419355	443	39	1133	4497	830	4179	0	3230

TRINITY_DN16151_c0_g2_i5	Glyma.08G292900.5	97.77777778	443	39	1133	4497	830	4179	0	3230

TRINITY_DN16151_c0_g2_i3	Glyma.08G292900.6	96.42857143	443	40	872	4236	830	4176	0	3212

TRINITY_DN16151_c0_g2_i3	Glyma.08G292900.4	95.75757576	443	40	872	4236	830	4176	0	3212

TRINITY_DN16151_c0_g2_i3	Glyma.08G292900.2	95.41284404	443	40	872	4236	830	4176	0	3212

TRINITY_DN16151_c0_g2_i5	Glyma.08G292900.6	98.13084112	443	40	1133	4497	830	4176	0	3212

TRINITY_DN16151_c0_g2_i5	Glyma.08G292900.4	100	443	40	1133	4497	830	4176	0	3212

TRINITY_DN16151_c0_g2_i5	Glyma.08G292900.2	100	443	40	1133	4497	830	4176	0	3212

TRINITY_DN14991_c0_g2_i1	Glyma.14G163000.1	100	266	10	407	3781	1	3393	0	4554

TRINITY_DN15340_c0_g1_i1	Glyma.13G158800.2	97.22222222	160	3	527	3928	3399	1	0	5376

TRINITY_DN14991_c0_g2_i1	Glyma.13G082100.4	100	279	13	407	3781	1	3390	0	4495

TRINITY_DN15602_c0_g1_i1	Glyma.19G223400.1	98.4496124	155	5	401	3800	1	3393	0	5374

TRINITY_DN15642_c0_g1_i3	Glyma.12G017600.1	100	445	18	264	3652	50	3426	0	3605

TRINITY_DN15642_c0_g1_i3	Glyma.12G017600.2	100	445	18	264	3652	50	3426	0	3605

TRINITY_DN15642_c0_g1_i4	Glyma.12G017600.1	97.19101124	445	18	338	3726	50	3426	0	3605

TRINITY_DN15642_c0_g1_i4	Glyma.12G017600.2	97.61904762	445	18	338	3726	50	3426	0	3605

TRINITY_DN16151_c0_g2_i3	Glyma.18G130400.3	98.85057471	425	36	872	4236	797	4137	0	3328

TRINITY_DN16151_c0_g2_i3	Glyma.18G130400.1	98.14814815	425	36	872	4236	797	4137	0	3328

TRINITY_DN16151_c0_g2_i5	Glyma.18G130400.3	99.86684421	425	36	1133	4497	797	4137	0	3328

TRINITY_DN16151_c0_g2_i5	Glyma.18G130400.1	98.7012987	425	36	1133	4497	797	4137	0	3328

TRINITY_DN10648_c0_g1_i1	Glyma.10G141400.4	99.73544974	247	9	133	3504	3393	1	0	4695

TRINITY_DN10648_c0_g1_i1	Glyma.10G141400.3	100	247	9	133	3504	3393	1	0	4695

TRINITY_DN10648_c0_g1_i1	Glyma.10G141400.2	100	247	9	133	3504	3393	1	0	4695

TRINITY_DN14991_c0_g2_i1	Glyma.13G082100.3	100	279	13	407	3777	1	3386	0	4488

TRINITY_DN14991_c0_g2_i1	Glyma.13G082100.1	100	279	13	407	3777	1	3386	0	4488

TRINITY_DN14064_c0_g1_i3	Glyma.13G209800.2	97.8021978	176	1	317	3710	3397	1	0	5278

TRINITY_DN12701_c0_g1_i1	Glyma.05G220300.1	100	175	7	1	3390	163	3543	0	5199

TRINITY_DN12701_c0_g1_i1	Glyma.08G026700.1	100	158	6	1	3390	163	3543	0	5293

TRINITY_DN10648_c0_g1_i2	Glyma.19G224200.2	98.61111111	415	23	146	3510	3380	1	0	3723

TRINITY_DN10648_c0_g1_i2	Glyma.19G224200.1	100	415	23	146	3510	3380	1	0	3723

TRINITY_DN7067_c0_g1_i1	Glyma.01G168100.1	96.20938628	306	5	66	3449	1	3384	0	4477

TRINITY_DN2743_c0_g1_i1	Glyma.12G083200.1	98	181	10	366	3731	3387	1	0	5083

TRINITY_DN16168_c0_g1_i3	Glyma.16G219100.2	100	473	24	241	3615	3352	5	0	3282

TRINITY_DN16168_c0_g1_i3	Glyma.16G219100.3	100	473	24	241	3615	3352	5	0	3282

TRINITY_DN16168_c0_g1_i3	Glyma.16G219100.1	99.57446809	473	24	241	3615	3352	5	0	3282

TRINITY_DN16151_c0_g2_i2	Glyma.08G292900.1	97.82608696	437	45	1133	4479	830	4179	0	3314

TRINITY_DN16151_c0_g2_i2	Glyma.08G292900.5	97.83163265	437	45	1133	4479	830	4179	0	3314

TRINITY_DN16151_c0_g2_i6	Glyma.08G292900.1	95	437	45	872	4218	830	4179	0	3314

TRINITY_DN16151_c0_g2_i6	Glyma.08G292900.5	95.37750385	437	45	872	4218	830	4179	0	3314

TRINITY_DN16151_c0_g2_i2	Glyma.08G292900.6	97.83393502	437	46	1133	4479	830	4176	0	3295

TRINITY_DN16151_c0_g2_i2	Glyma.08G292900.4	99.02912621	437	46	1133	4479	830	4176	0	3295

TRINITY_DN16151_c0_g2_i2	Glyma.08G292900.2	100	437	46	1133	4479	830	4176	0	3295

TRINITY_DN16151_c0_g2_i6	Glyma.08G292900.6	97.64705882	437	46	872	4218	830	4176	0	3295

TRINITY_DN16151_c0_g2_i6	Glyma.08G292900.4	98.6407767	437	46	872	4218	830	4176	0	3295

TRINITY_DN16151_c0_g2_i6	Glyma.08G292900.2	96	437	46	872	4218	830	4176	0	3295

TRINITY_DN3079_c0_g2_i1	Glyma.19G001500.1	100	257	14	554	3932	566	3933	0	4634

TRINITY_DN10648_c0_g1_i1	Glyma.19G224200.2	96.8503937	464	23	146	3504	3380	1	0	3446

TRINITY_DN10648_c0_g1_i1	Glyma.19G224200.1	100	464	23	146	3504	3380	1	0	3446

TRINITY_DN3079_c0_g1_i1	Glyma.19G001500.1	95.11400651	257	13	554	3931	566	3933	0	4639

TRINITY_DN16168_c0_g1_i3	Glyma.09G169500.1	96.01873536	450	29	241	3615	3328	5	0	3288

TRINITY_DN16158_c0_g1_i5	Glyma.10G286300.1	100	377	14	347	3728	3531	172	0	3971

TRINITY_DN7067_c0_g1_i1	Glyma.11G075200.1	100	284	9	76	3449	14	3396	0	4593

TRINITY_DN16043_c0_g2_i2	Glyma.08G293900.1	98.57549858	166	7	28	3402	1	3378	0	5232

TRINITY_DN15484_c0_g1_i2	Glyma.08G165900.3	99.68652038	331	31	4013	7363	3363	1	0	4078

TRINITY_DN15484_c0_g1_i2	Glyma.08G165900.4	96.66666667	331	31	4013	7363	3363	1	0	4078

TRINITY_DN15484_c0_g1_i2	Glyma.08G165900.1	96.66666667	331	31	4013	7363	3363	1	0	4078

TRINITY_DN15484_c0_g1_i2	Glyma.08G165900.2	95.66878981	331	31	4013	7363	3363	1	0	4078

TRINITY_DN15934_c0_g1_i1	Glyma.10G212600.1	96.17547807	306	13	418	3784	1	3380	0	4440

TRINITY_DN15934_c0_g1_i1	Glyma.10G212600.2	100	306	14	418	3784	1	3377	0	4421

TRINITY_DN15934_c0_g1_i1	Glyma.20G178500.1	95.02702703	296	9	418	3784	1	3380	0	4495

TRINITY_DN15934_c0_g1_i1	Glyma.20G178500.2	98.94366197	296	10	418	3784	1	3377	0	4477

TRINITY_DN16151_c0_g2_i2	Glyma.18G130400.3	100	425	32	1133	4479	797	4137	0	3411

TRINITY_DN16151_c0_g2_i2	Glyma.18G130400.1	100	425	32	1133	4479	797	4137	0	3411

TRINITY_DN16151_c0_g2_i6	Glyma.18G130400.3	98	425	32	872	4218	797	4137	0	3411

TRINITY_DN16151_c0_g2_i6	Glyma.18G130400.1	96.38554217	425	32	872	4218	797	4137	0	3411

TRINITY_DN15484_c0_g1_i2	Glyma.15G261100.1	100	340	35	4013	7363	3360	1	0	4030

TRINITY_DN10648_c0_g1_i2	Glyma.20G090000.1	100	305	6	133	3510	3372	1	0	4477

TRINITY_DN16335_c0_g2_i1	Glyma.16G220100.4	97.9020979	499	21	700	4068	3519	157	0	3297

TRINITY_DN16335_c0_g2_i1	Glyma.16G220100.2	97.89473684	499	21	700	4068	3519	157	0	3297

TRINITY_DN15755_c0_g1_i2	Glyma.11G034100.1	96.89265537	244	15	1	3352	3	3378	0	4684

TRINITY_DN15755_c0_g1_i2	Glyma.11G034100.2	100	244	15	1	3352	3	3378	0	4684

TRINITY_DN15755_c0_g1_i2	Glyma.11G034100.3	95.54317549	244	15	1	3352	3	3378	0	4684

TRINITY_DN15755_c0_g1_i4	Glyma.11G034100.1	100	244	15	1	3352	3	3378	0	4684

TRINITY_DN15755_c0_g1_i4	Glyma.11G034100.2	98.38709677	244	15	1	3352	3	3378	0	4684

TRINITY_DN15755_c0_g1_i4	Glyma.11G034100.3	96.62921348	244	15	1	3352	3	3378	0	4684

TRINITY_DN2743_c0_g1_i1	Glyma.11G191100.1	100	196	8	366	3731	3375	1	0	5044

TRINITY_DN16335_c0_g2_i1	Glyma.16G220100.5	97.91666667	507	16	700	4068	3522	157	0	3304

TRINITY_DN16335_c0_g2_i1	Glyma.16G220100.3	97.91666667	507	16	700	4068	3522	157	0	3304

TRINITY_DN16335_c0_g2_i1	Glyma.16G220100.1	97.91666667	507	16	700	4068	3522	157	0	3304

TRINITY_DN11189_c0_g1_i2	Glyma.09G285300.1	95.65217391	183	16	1059	4411	1211	4572	0	4970

TRINITY_DN8701_c0_g1_i1	Glyma.17G015200.2	97.69736842	215	2	325	3697	3450	75	0	5024

TRINITY_DN16245_c0_g5_i2	Glyma.01G092900.1	97.89719626	795	76	422	3751	3513	184	0	1258

TRINITY_DN11189_c0_g1_i2	Glyma.20G000700.1	100	162	10	1059	4411	1214	4566	0	5062

TRINITY_DN11189_c0_g1_i2	Glyma.20G000700.2	95.77464789	162	10	1059	4411	638	3990	0	5062

TRINITY_DN16245_c0_g5_i1	Glyma.01G092900.1	98.5915493	797	75	422	3751	3513	184	0	1258

TRINITY_DN10648_c0_g1_i1	Glyma.20G090000.1	97.36842105	236	2	133	3504	3372	1	0	4909

TRINITY_DN16064_c0_g2_i1	Glyma.15G011900.1	98.85714286	526	36	15	3370	32	3363	0	2963

TRINITY_DN16064_c0_g2_i3	Glyma.15G011900.1	96.55172414	526	36	15	3370	32	3363	0	2963

TRINITY_DN11759_c0_g1_i2	Glyma.05G145000.2	96.42857143	357	13	1252	4595	3368	1	0	4054

TRINITY_DN16064_c0_g2_i1	Glyma.15G012000.1	100	527	35	15	3370	32	3366	0	2987

TRINITY_DN16064_c0_g2_i3	Glyma.15G012000.1	100	527	35	15	3370	32	3366	0	2987

TRINITY_DN6182_c0_g1_i1	Glyma.07G054500.1	97.81420765	240	8	1	3364	4242	7602	0	4796

TRINITY_DN8789_c0_g1_i1	Glyma.17G255500.1	100	209	1	813	4173	5724	2358	0	5022

TRINITY_DN15687_c0_g1_i1	Glyma.13G197000.1	97.84946237	250	3	210	3569	1	3366	0	4793

TRINITY_DN15070_c0_g1_i2	Glyma.09G256700.1	95.72649573	170	0	442	3807	3366	1	0	5275

TRINITY_DN15070_c0_g1_i2	Glyma.18G235800.1	97.19298246	145	0	442	3807	3366	1	0	5413

TRINITY_DN13164_c0_g1_i3	Glyma.19G113200.1	97.51243781	643	11	110	3466	1	3357	0	2549

TRINITY_DN16369_c0_g1_i3	Glyma.06G003600.4	100	491	28	1689	5002	1	3356	0	3107

TRINITY_DN16158_c0_g1_i4	Glyma.10G286300.1	98.80952381	378	7	347	3706	3531	172	0	4067

TRINITY_DN16158_c0_g1_i7	Glyma.10G286300.1	99.52191235	376	7	72	3431	3531	172	0	4078

TRINITY_DN14575_c0_g1_i1	Glyma.17G197200.4	100	279	4	3263	6624	5532	2174	0	4621

TRINITY_DN14575_c0_g1_i1	Glyma.17G197200.5	97.87234043	279	4	3263	6624	5532	2174	0	4621

TRINITY_DN14575_c0_g1_i1	Glyma.17G197200.1	100	279	4	3263	6624	5532	2174	0	4621

TRINITY_DN14575_c0_g1_i1	Glyma.17G197200.2	97.11538462	279	4	3263	6624	5508	2150	0	4621

TRINITY_DN14575_c0_g1_i1	Glyma.17G197200.3	100	279	4	3263	6624	5508	2150	0	4621

TRINITY_DN11500_c0_g1_i1	Glyma.07G151400.1	98.95833333	272	6	121	3483	1	3357	0	4654

TRINITY_DN11500_c0_g1_i2	Glyma.07G151400.1	99.02912621	272	7	121	3472	1	3357	0	4582

TRINITY_DN16369_c0_g1_i1	Glyma.06G003600.4	100	366	11	1689	5035	1	3356	0	4034

TRINITY_DN11500_c0_g1_i1	Glyma.18G202700.1	99.17184265	245	4	121	3483	1	3348	0	4756

TRINITY_DN11500_c0_g1_i2	Glyma.18G202700.1	99.02912621	245	5	121	3472	1	3348	0	4686

TRINITY_DN16040_c0_g1_i4	Glyma.14G044500.1	96	231	2	356	3718	3357	1	0	4892

TRINITY_DN16040_c0_g1_i4	Glyma.14G044500.2	95.47511312	231	3	356	3718	3354	1	0	4872

TRINITY_DN16040_c0_g1_i3	Glyma.14G044500.1	96.59863946	199	2	610	3972	3357	1	0	5070

TRINITY_DN16040_c0_g1_i3	Glyma.14G044500.2	96.24664879	199	3	610	3972	3354	1	0	5049

TRINITY_DN16040_c0_g1_i1	Glyma.14G044500.1	97.36842105	181	2	356	3718	3357	1	0	5169

TRINITY_DN16040_c0_g1_i1	Glyma.14G044500.2	95.40636042	181	3	356	3718	3354	1	0	5149

TRINITY_DN16040_c0_g1_i2	Glyma.14G044500.1	99.82142857	149	2	610	3972	3357	1	0	5347

TRINITY_DN16040_c0_g1_i2	Glyma.14G044500.2	99.82269504	149	3	610	3972	3354	1	0	5326

TRINITY_DN16040_c0_g1_i4	Glyma.02G271500.1	97.36842105	236	2	357	3718	3356	1	0	4863

TRINITY_DN16040_c0_g1_i4	Glyma.02G271500.2	97.36842105	236	3	357	3718	3353	1	0	4843

TRINITY_DN16040_c0_g1_i3	Glyma.02G271500.1	97.43589744	205	2	611	3972	3356	1	0	5035

TRINITY_DN16040_c0_g1_i3	Glyma.02G271500.2	97.43589744	205	3	611	3972	3353	1	0	5014

TRINITY_DN16040_c0_g1_i1	Glyma.02G271500.1	97.72727273	186	2	357	3718	3356	1	0	5140

TRINITY_DN16040_c0_g1_i1	Glyma.02G271500.2	100	186	3	357	3718	3353	1	0	5120

TRINITY_DN16040_c0_g1_i2	Glyma.02G271500.1	97.52650177	155	2	611	3972	3356	1	0	5312

TRINITY_DN16040_c0_g1_i2	Glyma.02G271500.2	98.18181818	155	3	611	3972	3353	1	0	5291

TRINITY_DN8789_c0_g1_i1	Glyma.17G255500.2	95.65217391	209	0	813	4173	5718	2358	0	5049

TRINITY_DN8789_c0_g1_i1	Glyma.14G216900.6	97.43589744	206	0	813	4173	5685	2325	0	5066

TRINITY_DN8789_c0_g1_i1	Glyma.14G216900.4	96.29909366	206	0	813	4173	5685	2325	0	5066

TRINITY_DN8789_c0_g1_i1	Glyma.14G216900.1	100	206	0	813	4173	5685	2325	0	5066

TRINITY_DN8789_c0_g1_i1	Glyma.14G216900.3	100	206	0	813	4173	5685	2325	0	5066

TRINITY_DN12607_c0_g1_i1	Glyma.13G094500.3	97.05882353	306	15	192	3527	1	3333	0	4180

TRINITY_DN12607_c0_g1_i1	Glyma.13G094500.4	97.05882353	306	15	192	3527	1	3333	0	4180

TRINITY_DN12607_c0_g1_i1	Glyma.13G094500.2	97.05882353	306	15	192	3527	1	3333	0	4180

TRINITY_DN12607_c0_g1_i1	Glyma.13G094500.1	97.04142012	306	15	192	3527	1	3333	0	4180

TRINITY_DN12607_c0_g1_i1	Glyma.13G094500.5	97.04918033	306	15	192	3527	1	3333	0	4180

TRINITY_DN15311_c0_g1_i1	Glyma.18G298700.1	98.73417722	254	5	87	3443	1	3357	0	4759

TRINITY_DN13016_c0_g1_i1	Glyma.09G226400.2	100	185	12	571	3912	3357	1	0	5044

TRINITY_DN14904_c0_g2_i1	Glyma.02G086100.1	96.2962963	309	18	223	3562	1	3343	0	4265

TRINITY_DN14565_c0_g1_i1	Glyma.03G207600.1	95.77464789	292	13	351	3697	3347	1	0	4431

TRINITY_DN13187_c0_g1_i1	Glyma.17G045200.1	96.82539683	210	11	572	3914	1	3352	0	4891

TRINITY_DN16168_c0_g1_i1	Glyma.16G219100.2	95.70707071	222	7	241	3592	3352	1	0	4894

TRINITY_DN16168_c0_g1_i1	Glyma.16G219100.3	95.70815451	222	7	241	3592	3352	1	0	4894

TRINITY_DN16168_c0_g1_i1	Glyma.16G219100.1	96.51972158	222	7	241	3592	3352	1	0	4894

TRINITY_DN16212_c0_g1_i3	Glyma.13G142100.3	99.2	408	7	270	3622	3353	1	0	3888

TRINITY_DN16438_c0_g1_i1	Glyma.02G101800.1	100	297	9	132	3474	63	3417	0	4462

TRINITY_DN16212_c0_g1_i2	Glyma.13G142100.3	95.45454545	348	3	270	3622	3353	1	0	4242

TRINITY_DN12254_c0_g1_i2	Glyma.16G063300.1	100	204	18	485	3835	1	3327	0	4859

TRINITY_DN12254_c0_g1_i2	Glyma.19G082100.3	100	199	3	485	3835	1	3354	0	5060

TRINITY_DN12254_c0_g1_i2	Glyma.19G082100.1	100	199	3	485	3835	1	3354	0	5060

TRINITY_DN16262_c0_g1_i2	Glyma.08G156400.1	98.7012987	396	27	537	3860	1	3339	0	3709

TRINITY_DN15070_c0_g1_i2	Glyma.09G256700.2	97.59036145	170	1	442	3794	3348	1	0	5217

TRINITY_DN15070_c0_g1_i2	Glyma.18G235800.2	97.19350074	145	1	442	3794	3348	1	0	5356

TRINITY_DN16064_c0_g2_i1	Glyma.07G033500.1	97.77777778	249	8	17	3365	34	3361	0	4636

TRINITY_DN16064_c0_g2_i3	Glyma.07G033500.1	96.22641509	249	8	17	3365	34	3361	0	4636

TRINITY_DN35799_c0_g1_i1	Glyma.10G055100.1	100	237	1	697	4045	4504	1153	0	4857

TRINITY_DN35799_c0_g1_i1	Glyma.10G055100.2	98.56850716	237	1	697	4045	3589	238	0	4857

TRINITY_DN16168_c0_g1_i1	Glyma.09G169500.1	96.875	200	8	241	3592	3328	1	0	4927

TRINITY_DN8232_c0_g1_i1	Glyma.07G012400.2	100	343	13	1	3334	246	3588	0	4126

TRINITY_DN16262_c0_g1_i2	Glyma.05G191200.1	98.00796813	411	25	537	3860	1	3339	0	3681

TRINITY_DN35799_c0_g1_i1	Glyma.13G142200.1	100	238	0	697	4045	4501	1153	0	4867

TRINITY_DN35799_c0_g1_i1	Glyma.13G142200.2	100	238	0	697	4045	3901	553	0	4867

TRINITY_DN3079_c0_g2_i1	Glyma.19G001500.2	100	249	13	596	3932	1	3327	0	4608

TRINITY_DN10035_c0_g2_i1	Glyma.10G013500.2	100	234	12	1	3330	747	4091	0	4761

TRINITY_DN10035_c0_g2_i1	Glyma.10G013500.1	97.82945736	234	12	1	3330	747	4091	0	4761

TRINITY_DN10035_c0_g2_i1	Glyma.10G013500.4	98.92473118	234	12	1	3330	348	3692	0	4761

TRINITY_DN10035_c0_g2_i1	Glyma.10G013500.3	98.16513761	234	12	1	3330	195	3539	0	4761

TRINITY_DN3079_c0_g1_i1	Glyma.19G001500.2	98.74055416	249	12	596	3931	1	3327	0	4612

TRINITY_DN13164_c0_g1_i1	Glyma.08G114900.1	96.56357388	222	2	171	3515	1	3345	0	4937

TRINITY_DN16262_c0_g1_i5	Glyma.04G213400.1	100	418	29	439	3777	1	3300	0	3531

TRINITY_DN16262_c0_g1_i5	Glyma.04G213400.2	100	416	31	439	3777	1	3297	0	3524

TRINITY_DN16349_c0_g2_i1	Glyma.02G155000.1	98.73015873	366	23	494	3829	10	3321	0	3879

TRINITY_DN16262_c0_g1_i5	Glyma.06G152800.1	100	410	31	439	3777	1	3288	0	3509

TRINITY_DN15320_c0_g1_i1	Glyma.15G045100.1	100	263	9	290	3627	3486	147	0	4654

TRINITY_DN16428_c1_g1_i2	Glyma.18G210300.5	100	161	2	1081	4424	3350	22	0	5186

TRINITY_DN16428_c1_g1_i3	Glyma.18G210300.5	95.45454545	161	2	937	4280	3350	22	0	5186

TRINITY_DN16428_c1_g1_i2	Glyma.18G210300.4	100	161	2	1081	4424	3329	1	0	5186

TRINITY_DN16428_c1_g1_i3	Glyma.18G210300.4	95.93023256	161	2	937	4280	3329	1	0	5186

TRINITY_DN12896_c0_g2_i1	Glyma.18G072400.1	95.83858764	255	6	244	3581	12	3351	0	4710

TRINITY_DN13604_c0_g1_i1	Glyma.11G218000.2	96.2962963	201	2	147	3488	1	3342	0	5048

TRINITY_DN16368_c0_g2_i2	Glyma.03G156600.1	100	587	19	1	3332	961	4292	0	2791

TRINITY_DN16262_c0_g1_i8	Glyma.05G191200.1	98.68073879	299	6	439	3774	1	3339	0	4457

TRINITY_DN13604_c0_g1_i1	Glyma.18G039000.1	96.2962963	196	0	147	3488	1	3342	0	5086

TRINITY_DN16262_c0_g1_i8	Glyma.06G152800.1	95.83333333	331	30	439	3774	1	3288	0	3962

TRINITY_DN16262_c0_g1_i8	Glyma.04G213400.1	98.68073879	345	24	439	3774	1	3300	0	3973

TRINITY_DN16262_c0_g1_i8	Glyma.04G213400.2	96.42857143	343	26	439	3774	1	3297	0	3964

TRINITY_DN16262_c0_g1_i8	Glyma.08G156400.1	98.67986799	299	1	439	3774	1	3339	0	4490

TRINITY_DN16262_c0_g1_i5	Glyma.05G191200.1	97.05882353	219	0	439	3777	1	3339	0	4953

TRINITY_DN16262_c0_g1_i5	Glyma.08G156400.1	100	204	0	439	3777	1	3339	0	5036

TRINITY_DN14092_c0_g1_i1	Glyma.10G243000.1	100	300	12	122	3448	1	3324	0	4340

TRINITY_DN15755_c0_g1_i2	Glyma.01G208100.1	96.46017699	237	12	31	3352	18	3348	0	4713

TRINITY_DN15755_c0_g1_i4	Glyma.01G208100.1	97.37609329	237	12	31	3352	18	3348	0	4713

TRINITY_DN13164_c0_g1_i3	Glyma.16G039900.1	97.52066116	623	19	140	3466	22	3345	0	2573

TRINITY_DN13164_c0_g1_i3	Glyma.16G039900.2	97.51552795	623	19	140	3466	22	3345	0	2573

TRINITY_DN13595_c0_g1_i1	Glyma.19G130800.3	100	139	1	565	3898	3333	1	0	5380

TRINITY_DN7067_c0_g1_i1	Glyma.02G050300.1	100	716	42	144	3444	73	3388	0	1871

TRINITY_DN12563_c0_g1_i3	Glyma.08G027800.2	99.3006993	546	46	269	3558	60	3340	0	2560

TRINITY_DN12563_c0_g1_i3	Glyma.08G027800.1	100	546	46	269	3558	60	3340	0	2560

TRINITY_DN15994_c0_g1_i3	Glyma.13G158800.1	96.55172414	796	70	539	3815	3324	37	0	1147

TRINITY_DN16262_c0_g1_i2	Glyma.06G152800.1	96.27659574	233	19	537	3860	1	3288	0	4571

TRINITY_DN16430_c0_g2_i5	Glyma.11G033000.2	98.14814815	297	18	46	3355	118	3423	0	4253

TRINITY_DN14961_c3_g1_i1	Glyma.06G204400.1	100	296	8	844	4152	1	3315	0	4311

TRINITY_DN9714_c0_g2_i1	Glyma.07G122900.1	100	261	6	332	3644	6483	3165	0	4556

TRINITY_DN15994_c0_g1_i3	Glyma.13G158800.2	100	796	69	539	3815	3321	37	0	1151

TRINITY_DN9714_c0_g2_i1	Glyma.07G122900.2	100	259	7	332	3644	6459	3165	0	4423

TRINITY_DN16262_c0_g1_i2	Glyma.04G213400.1	96.27329193	246	14	537	3860	1	3300	0	4610

TRINITY_DN16262_c0_g1_i2	Glyma.04G213400.2	98.80952381	244	16	537	3860	1	3297	0	4601

TRINITY_DN14989_c0_g1_i1	Glyma.13G222000.2	95.94594595	377	4	502	3797	26	3348	0	3862

TRINITY_DN38330_c0_g1_i1	Glyma.10G118100.1	98.31932773	280	16	1	3316	708	3999	0	4329

TRINITY_DN38330_c0_g1_i1	Glyma.10G118100.3	98.31932773	280	16	1	3316	36	3327	0	4329

TRINITY_DN14386_c0_g1_i1	Glyma.10G232300.1	100	264	21	364	3633	1	3309	0	4229

TRINITY_DN14386_c0_g1_i1	Glyma.10G232300.2	100	264	21	364	3633	1	3309	0	4229

TRINITY_DN12196_c0_g1_i1	Glyma.12G205800.1	100	150	0	185	3508	1	3324	0	5308

TRINITY_DN12196_c0_g1_i1	Glyma.12G205800.2	100	150	0	185	3508	1	3324	0	5308

TRINITY_DN16309_c0_g1_i5	Glyma.05G060300.1	99.30555556	382	25	12	3287	3151	6455	0	3611

TRINITY_DN16309_c0_g1_i5	Glyma.05G060300.2	99.31506849	382	25	12	3287	3151	6455	0	3611

TRINITY_DN16309_c0_g1_i5	Glyma.05G060300.3	99.3006993	382	25	12	3287	3151	6455	0	3611

TRINITY_DN16245_c0_g1_i4	Glyma.07G007700.7	96.07843137	287	13	658	3953	3320	1	0	4364

TRINITY_DN16245_c0_g1_i4	Glyma.07G007700.8	96.07843137	287	13	658	3953	3320	1	0	4364

TRINITY_DN16245_c0_g1_i5	Glyma.07G007700.7	96.82539683	221	4	740	4059	3320	1	0	4885

TRINITY_DN16245_c0_g1_i5	Glyma.07G007700.8	99.12280702	221	4	740	4059	3320	1	0	4885

TRINITY_DN16245_c0_g1_i2	Glyma.07G007700.7	100	219	7	740	4038	3320	1	0	4759

TRINITY_DN16245_c0_g1_i2	Glyma.07G007700.8	95.40636042	219	7	740	4038	3320	1	0	4759

TRINITY_DN16245_c0_g1_i5	Glyma.07G007700.5	99.12280702	219	7	740	4059	3299	1	0	4759

TRINITY_DN16245_c0_g1_i5	Glyma.07G007700.6	95.66666667	219	7	740	4059	3299	1	0	4759

TRINITY_DN14386_c0_g1_i1	Glyma.20G161800.2	100	257	25	364	3633	1	3297	0	4224

TRINITY_DN14386_c0_g1_i1	Glyma.20G161800.1	100	257	25	364	3633	1	3297	0	4224

TRINITY_DN14386_c0_g1_i1	Glyma.20G161800.3	100	257	25	364	3633	1	3297	0	4224

TRINITY_DN14837_c0_g4_i1	Glyma.15G230600.2	97.5308642	165	2	198	3512	1	3315	0	5197

TRINITY_DN14837_c0_g4_i1	Glyma.15G230600.1	95.22091975	165	2	198	3512	1	3315	0	5197

TRINITY_DN14837_c0_g4_i1	Glyma.13G192900.1	97.56097561	165	4	198	3512	1	3309	0	5158

TRINITY_DN14961_c3_g1_i1	Glyma.06G204400.2	100	296	7	844	4152	1	3303	0	4366

TRINITY_DN8872_c0_g1_i1	Glyma.09G035500.1	98.63013699	381	17	66	3364	112	3414	0	3842

TRINITY_DN8872_c0_g1_i2	Glyma.09G035500.1	95.53450609	381	17	66	3364	112	3414	0	3842

TRINITY_DN15754_c0_g1_i3	Glyma.08G072100.1	100	419	42	304	3597	3240	1	0	3203

TRINITY_DN15754_c0_g1_i3	Glyma.05G117000.1	98.13084112	410	27	304	3597	3267	1	0	3426

TRINITY_DN12563_c0_g1_i2	Glyma.01G002100.1	100	298	18	268	3546	4	3313	0	4239

TRINITY_DN14961_c3_g1_i1	Glyma.04G161000.4	100	280	3	844	4152	1	3306	0	4506

TRINITY_DN14961_c3_g1_i1	Glyma.04G161000.3	100	280	3	844	4152	1	3306	0	4506

TRINITY_DN14961_c3_g1_i1	Glyma.04G161000.1	100	280	3	844	4152	1	3306	0	4506

TRINITY_DN14961_c3_g1_i1	Glyma.04G161000.2	100	280	3	844	4152	1	3306	0	4506

TRINITY_DN12563_c0_g1_i3	Glyma.01G002100.1	98.80095923	268	11	268	3558	4	3313	0	4482

TRINITY_DN5197_c0_g2_i1	Glyma.06G239100.1	98.15602837	333	53	3	3261	1750	5005	0	3578

TRINITY_DN13689_c0_g1_i2	Glyma.06G139800.1	100	253	32	258	3520	1	3299	0	4325

TRINITY_DN5197_c0_g2_i1	Glyma.18G007300.1	100	342	45	3	3261	1747	4999	0	3520

TRINITY_DN5197_c0_g2_i1	Glyma.18G007300.2	96.34146341	342	45	3	3261	499	3751	0	3520

TRINITY_DN13633_c0_g1_i1	Glyma.07G215200.1	100	175	19	293	3582	3375	83	0	4903

TRINITY_DN13633_c0_g1_i2	Glyma.07G215200.1	95.84569733	175	19	360	3649	3375	83	0	4903

TRINITY_DN13633_c0_g1_i1	Glyma.07G215200.2	100	175	22	293	3582	3372	83	0	4885

TRINITY_DN13633_c0_g1_i2	Glyma.07G215200.2	98.68421053	175	22	360	3649	3372	83	0	4885

TRINITY_DN8872_c0_g1_i1	Glyma.15G140000.1	100	389	11	68	3364	150	3450	0	3827

TRINITY_DN8872_c0_g1_i2	Glyma.15G140000.1	98.34254144	389	11	68	3364	150	3450	0	3827

TRINITY_DN11821_c0_g1_i2	Glyma.13G322000.1	98.57142857	234	8	821	4122	3642	347	0	4686

TRINITY_DN15755_c0_g1_i1	Glyma.11G034100.1	95.14563107	237	12	202	3487	75	3378	0	4628

TRINITY_DN15755_c0_g1_i1	Glyma.11G034100.2	96.90346084	237	12	202	3487	75	3378	0	4628

TRINITY_DN15755_c0_g1_i1	Glyma.11G034100.3	100	237	12	202	3487	75	3378	0	4628

TRINITY_DN15755_c0_g1_i3	Glyma.11G034100.1	95.67723343	237	12	202	3487	75	3378	0	4628

TRINITY_DN15755_c0_g1_i3	Glyma.11G034100.2	95.67723343	237	12	202	3487	75	3378	0	4628

TRINITY_DN15755_c0_g1_i3	Glyma.11G034100.3	97.6744186	237	12	202	3487	75	3378	0	4628

TRINITY_DN15754_c0_g1_i3	Glyma.15G053600.1	98.13084112	358	19	304	3597	3267	1	0	3781

TRINITY_DN15122_c0_g2_i1	Glyma.18G035800.1	96.56862745	427	9	565	3865	4420	1126	0	3637

TRINITY_DN15549_c0_g1_i2	Glyma.08G055400.1	95.53571429	238	19	391	3662	3276	1	0	4373

TRINITY_DN9262_c0_g1_i1	Glyma.05G212000.2	97.97979798	230	16	1	3266	616	3914	0	4527

TRINITY_DN9262_c0_g1_i2	Glyma.05G212000.2	98.61111111	230	16	1	3266	616	3914	0	4527

TRINITY_DN13164_c0_g1_i3	Glyma.08G114900.1	97.50957854	299	35	185	3466	70	3345	0	4122

TRINITY_DN15549_c0_g1_i2	Glyma.05G247000.4	95.53571429	239	15	391	3662	3270	1	0	4362

TRINITY_DN15549_c0_g1_i2	Glyma.05G247000.1	97.64705882	239	15	391	3662	3270	1	0	4362

TRINITY_DN15549_c0_g1_i2	Glyma.08G055400.3	100	239	17	391	3662	3273	1	0	4381

TRINITY_DN8358_c0_g1_i1	Glyma.02G212700.2	96.35761589	235	8	187	3474	1	3285	0	4584

TRINITY_DN13633_c0_g1_i1	Glyma.02G103800.1	98.86363636	182	8	293	3580	3375	82	0	4937

TRINITY_DN13633_c0_g1_i2	Glyma.02G103800.1	99.05660377	182	8	360	3647	3375	82	0	4937

TRINITY_DN12563_c0_g1_i3	Glyma.05G221300.1	96.96969697	558	43	269	3558	60	3298	0	2521

TRINITY_DN8943_c0_g1_i1	Glyma.10G140500.3	100	393	13	1193	4474	3297	9	0	3707

TRINITY_DN16245_c0_g1_i4	Glyma.07G007700.5	96.07843137	290	5	658	3953	3299	1	0	4444

TRINITY_DN16245_c0_g1_i4	Glyma.07G007700.6	96.07843137	290	5	658	3953	3299	1	0	4444

TRINITY_DN15755_c0_g1_i1	Glyma.01G208100.1	98.79518072	230	12	202	3487	54	3348	0	4686

TRINITY_DN15755_c0_g1_i3	Glyma.01G208100.1	95.67567568	230	12	202	3487	54	3348	0	4686

TRINITY_DN16245_c0_g1_i2	Glyma.07G007700.5	99.27007299	220	4	740	4038	3299	1	0	4852

TRINITY_DN16245_c0_g1_i2	Glyma.07G007700.6	95.23809524	220	4	740	4038	3299	1	0	4852

TRINITY_DN8943_c0_g1_i1	Glyma.20G089300.4	100	379	13	1195	4474	3295	9	0	3781

TRINITY_DN15754_c0_g1_i3	Glyma.08G178900.1	98.13084112	377	24	304	3597	3246	1	0	3618

TRINITY_DN16314_c1_g2_i3	Glyma.10G223500.1	98.7854251	323	13	447	3730	3293	1	0	4156

TRINITY_DN16314_c1_g2_i2	Glyma.10G223500.1	100	380	19	447	3712	3293	1	0	3735

TRINITY_DN16314_c1_g2_i1	Glyma.10G223500.1	100	314	6	447	3736	3293	1	0	4266

TRINITY_DN16314_c1_g2_i4	Glyma.10G223500.1	98.79518072	371	12	447	3718	3293	1	0	3845

TRINITY_DN39762_c0_g1_i1	Glyma.08G356500.4	99.11504425	320	33	6	3259	22	3302	0	3934

TRINITY_DN16314_c1_g1_i3	Glyma.05G187300.1	100	208	1	697	3993	3294	1	0	4916

TRINITY_DN16314_c1_g1_i3	Glyma.08G145600.1	100	198	1	697	3993	3294	1	0	4972

TRINITY_DN14596_c0_g1_i1	Glyma.20G174900.5	99.06976744	255	31	34	3294	1	3268	0	4266

TRINITY_DN14596_c0_g1_i1	Glyma.20G174900.1	100	255	31	34	3294	1	3268	0	4266

TRINITY_DN14596_c0_g1_i1	Glyma.20G174900.4	100	255	31	34	3294	1	3268	0	4266

TRINITY_DN14718_c0_g2_i2	Glyma.06G223500.1	96.08695652	194	5	2949	6228	3289	1	0	4863

TRINITY_DN14718_c0_g2_i3	Glyma.06G223500.1	96.07843137	194	5	751	4030	3289	1	0	4863

TRINITY_DN16064_c0_g2_i1	Glyma.13G361900.3	98.85496183	511	30	89	3370	100	3363	0	2970

TRINITY_DN16064_c0_g2_i1	Glyma.13G361900.2	98.85550787	511	30	89	3370	100	3363	0	2970

TRINITY_DN16064_c0_g2_i1	Glyma.13G361900.1	98.85714286	511	30	89	3370	100	3363	0	2970

TRINITY_DN16064_c0_g2_i3	Glyma.13G361900.3	96.07843137	511	30	89	3370	100	3363	0	2970

TRINITY_DN16064_c0_g2_i3	Glyma.13G361900.2	96.07843137	511	30	89	3370	100	3363	0	2970

TRINITY_DN16064_c0_g2_i3	Glyma.13G361900.1	96.07843137	511	30	89	3370	100	3363	0	2970

TRINITY_DN15122_c0_g2_i2	Glyma.18G035800.1	98.10725552	245	0	565	3859	4420	1126	0	4728

TRINITY_DN8789_c0_g1_i1	Glyma.14G216900.5	97.12041885	202	0	879	4173	5619	2325	0	4966

TRINITY_DN8789_c0_g1_i1	Glyma.14G216900.2	99.05660377	202	0	879	4173	5619	2325	0	4966

TRINITY_DN16314_c1_g2_i3	Glyma.10G223500.3	98.79032258	323	12	447	3730	3287	1	0	4183

TRINITY_DN12563_c0_g1_i3	Glyma.07G130400.1	95.3125	279	6	269	3558	50	3331	0	4434

TRINITY_DN12563_c0_g1_i3	Glyma.07G130400.3	98.80136986	279	6	269	3558	41	3322	0	4434

TRINITY_DN12563_c0_g1_i2	Glyma.05G221300.1	100	548	41	269	3546	60	3298	0	2599

TRINITY_DN16314_c1_g2_i2	Glyma.10G223500.3	100	381	15	447	3712	3287	1	0	3757

TRINITY_DN11623_c0_g1_i1	Glyma.12G030400.1	96.23655914	284	25	547	3804	365	3631	0	4113

TRINITY_DN13689_c0_g1_i2	Glyma.06G139800.12	97.5	254	21	258	3520	1	3281	0	4403

TRINITY_DN13689_c0_g1_i2	Glyma.06G139800.13	98.30508475	254	21	258	3520	1	3281	0	4403

TRINITY_DN16314_c1_g2_i1	Glyma.10G223500.3	100	314	5	447	3736	3287	1	0	4294

TRINITY_DN14596_c0_g1_i1	Glyma.10G217200.1	99.06976744	245	30	34	3294	1	3262	0	4327

TRINITY_DN16314_c1_g2_i4	Glyma.10G223500.3	98.79518072	372	8	447	3718	3287	1	0	3868

TRINITY_DN12563_c0_g1_i2	Glyma.05G221300.3	100	543	47	269	3546	60	3271	0	2486

TRINITY_DN12563_c0_g1_i2	Glyma.07G130400.1	100	312	8	269	3546	50	3331	0	4241

TRINITY_DN12563_c0_g1_i2	Glyma.07G130400.3	100	312	8	269	3546	41	3322	0	4241

TRINITY_DN13547_c0_g1_i2	Glyma.07G045600.4	98.03921569	245	12	2546	5827	1	3273	0	4578

TRINITY_DN16233_c0_g2_i3	Glyma.11G166100.1	100	323	17	237	3494	3285	1	0	4102

TRINITY_DN16233_c0_g2_i2	Glyma.11G166100.1	96.3060686	252	17	407	3664	3285	1	0	4495

TRINITY_DN15847_c1_g2_i1	Glyma.02G280400.1	100	211	11	127	3391	14	3296	0	4756

TRINITY_DN13689_c0_g1_i2	Glyma.06G139800.11	96.15384615	254	20	258	3520	1	3272	0	4444

TRINITY_DN13689_c0_g1_i2	Glyma.06G139800.10	100	252	21	258	3520	1	3263	0	4397

TRINITY_DN15847_c1_g2_i1	Glyma.14G034200.1	98.42931937	211	11	123	3391	13	3293	0	4780

TRINITY_DN15847_c1_g2_i3	Glyma.02G280400.1	99.14529915	160	3	127	3400	14	3296	0	5118

TRINITY_DN15122_c0_g2_i1	Glyma.18G035700.1	98.4	456	29	598	3862	4390	1129	0	3295

TRINITY_DN7742_c0_g1_i1	Glyma.17G026600.1	95.03546099	191	0	124	3405	1	3282	0	5003

TRINITY_DN16043_c0_g2_i2	Glyma.08G293900.2	96.37681159	161	7	28	3300	1	3276	0	5072

TRINITY_DN16043_c0_g2_i3	Glyma.08G293900.2	95.30201342	161	7	28	3300	1	3276	0	5072

TRINITY_DN12196_c0_g1_i1	Glyma.13G295100.1	96.18320611	145	0	185	3466	1	3282	0	5258

TRINITY_DN15122_c0_g2_i1	Glyma.11G221900.1	97.85714286	473	28	598	3862	4390	1129	0	3212

TRINITY_DN15320_c0_g1_i1	Glyma.13G328600.1	95.03546099	268	4	290	3568	3501	223	0	4549

TRINITY_DN15320_c0_g1_i1	Glyma.13G328600.2	100	268	4	290	3568	3501	223	0	4549

TRINITY_DN15847_c1_g2_i3	Glyma.14G034200.1	96.2962963	167	2	123	3400	13	3293	0	5114

TRINITY_DN15549_c0_g1_i6	Glyma.08G055400.1	100	238	13	391	3626	3276	1	0	4429

TRINITY_DN15847_c1_g2_i1	Glyma.14G034200.2	95.55555556	211	11	127	3391	14	3290	0	4772

TRINITY_DN14718_c0_g2_i2	Glyma.04G156200.1	96.12403101	190	3	2949	6228	3271	1	0	4946

TRINITY_DN14718_c0_g2_i3	Glyma.04G156200.1	96.07843137	190	3	751	4030	3271	1	0	4946

TRINITY_DN326_c0_g1_i1	Glyma.03G253000.1	99.08536585	113	1	733	4009	555	3834	0	5411

TRINITY_DN326_c0_g1_i1	Glyma.03G253000.3	97.34513274	113	1	733	4009	555	3834	0	5411

TRINITY_DN326_c0_g1_i1	Glyma.19G250600.1	95.55555556	113	1	733	4009	555	3834	0	5411

TRINITY_DN326_c0_g1_i1	Glyma.03G253000.4	98.36065574	113	1	733	4009	273	3552	0	5411

TRINITY_DN2648_c0_g2_i1	Glyma.15G099000.1	100	335	52	233	3429	212	3444	0	3373

TRINITY_DN14596_c0_g1_i2	Glyma.20G174900.5	98.95833333	257	24	34	3277	1	3268	0	4335

TRINITY_DN14596_c0_g1_i2	Glyma.20G174900.1	98.95833333	257	24	34	3277	1	3268	0	4335

TRINITY_DN14596_c0_g1_i2	Glyma.20G174900.4	98.95833333	257	24	34	3277	1	3268	0	4335

TRINITY_DN8088_c0_g2_i1	Glyma.16G000200.1	97.89473684	210	5	94	3363	1	3276	0	4815

TRINITY_DN15122_c0_g2_i1	Glyma.02G250000.2	95.06726457	253	10	593	3865	4392	1123	0	4567

TRINITY_DN15122_c0_g2_i1	Glyma.02G250000.1	100	253	10	593	3865	4392	1123	0	4567

TRINITY_DN16389_c1_g1_i1	Glyma.10G249300.2	95.1754386	337	42	1566	4764	4614	1380	0	3410

TRINITY_DN16389_c1_g1_i1	Glyma.10G249300.1	95.16129032	337	42	1566	4764	4614	1380	0	3410

TRINITY_DN16389_c1_g1_i1	Glyma.10G249300.4	98.4	337	42	1566	4764	4596	1362	0	3410

TRINITY_DN16389_c1_g1_i1	Glyma.10G249300.3	95.16129032	337	42	1566	4764	4596	1362	0	3410

TRINITY_DN11578_c0_g1_i2	Glyma.19G216000.1	98.79518072	240	13	50	3315	568	3822	0	4508

TRINITY_DN15549_c0_g1_i6	Glyma.08G055400.3	97.64705882	239	11	391	3626	3273	1	0	4436

TRINITY_DN15549_c0_g1_i6	Glyma.05G247000.4	100	239	15	391	3626	3270	1	0	4418

TRINITY_DN15549_c0_g1_i6	Glyma.05G247000.1	100	239	15	391	3626	3270	1	0	4418

TRINITY_DN15847_c1_g2_i3	Glyma.14G034200.2	98	167	2	127	3400	14	3290	0	5107

TRINITY_DN15122_c0_g2_i2	Glyma.18G035700.1	98.63013699	512	25	598	3856	4390	1129	0	3013

TRINITY_DN15755_c0_g1_i1	Glyma.01G208100.2	98.79518072	228	10	227	3487	1	3273	0	4671

TRINITY_DN15755_c0_g1_i2	Glyma.01G208100.2	95.67567568	228	10	92	3352	1	3273	0	4671

TRINITY_DN15755_c0_g1_i3	Glyma.01G208100.2	95.67669173	228	10	227	3487	1	3273	0	4671

TRINITY_DN15755_c0_g1_i4	Glyma.01G208100.2	99.21259843	228	10	92	3352	1	3273	0	4671

TRINITY_DN16314_c1_g2_i4	Glyma.02G080900.1	98.79518072	220	15	446	3718	3237	1	0	4560

TRINITY_DN8088_c0_g2_i1	Glyma.16G000200.2	97.87234043	211	3	94	3363	1	3273	0	4822

TRINITY_DN15832_c0_g2_i1	Glyma.13G063700.2	98.67109635	209	2	264	3537	3276	3	0	4867

TRINITY_DN14654_c0_g2_i1	Glyma.05G208100.1	100	204	12	279	3524	3261	1	0	4628

TRINITY_DN8088_c0_g2_i1	Glyma.12G163700.1	97.87985866	201	2	94	3363	1	3273	0	4878

TRINITY_DN16314_c1_g2_i2	Glyma.02G080900.1	100	199	19	446	3712	3237	1	0	4638

TRINITY_DN15122_c0_g2_i2	Glyma.02G250000.2	100	417	10	593	3859	4392	1123	0	3653

TRINITY_DN15122_c0_g2_i2	Glyma.02G250000.1	97.12837838	417	10	593	3859	4392	1123	0	3653

TRINITY_DN14596_c0_g1_i2	Glyma.10G217200.1	98.95833333	247	23	34	3277	1	3262	0	4396

TRINITY_DN12563_c0_g1_i3	Glyma.05G221300.2	98.80668258	551	45	297	3558	2	3208	0	2483

TRINITY_DN15122_c0_g2_i2	Glyma.14G066600.3	100	413	11	593	3858	4392	1124	0	3674

TRINITY_DN15122_c0_g2_i2	Glyma.14G066600.2	100	413	11	593	3858	4392	1124	0	3674

TRINITY_DN15122_c0_g2_i2	Glyma.14G066600.1	95.78313253	413	11	593	3858	4392	1124	0	3674

TRINITY_DN15122_c0_g2_i1	Glyma.14G066600.3	98.73417722	257	5	593	3864	4392	1124	0	4577

TRINITY_DN15122_c0_g2_i1	Glyma.14G066600.2	100	257	5	593	3864	4392	1124	0	4577

TRINITY_DN15122_c0_g2_i1	Glyma.14G066600.1	97.13193117	257	5	593	3864	4392	1124	0	4577

TRINITY_DN17153_c0_g1_i1	Glyma.20G218400.4	100	229	6	900	4169	3270	1	0	4726

TRINITY_DN17153_c0_g1_i1	Glyma.20G218400.5	99.3006993	229	6	900	4169	3270	1	0	4726

TRINITY_DN15122_c0_g2_i2	Glyma.11G221900.1	98.63013699	531	19	598	3856	4390	1129	0	2940

TRINITY_DN15847_c1_g2_i3	Glyma.18G119000.1	97.64705882	354	14	135	3394	28	3284	0	3895

TRINITY_DN8701_c0_g1_i1	Glyma.07G259000.2	99.54751131	196	3	325	3594	3273	1	0	4939

TRINITY_DN16069_c0_g1_i2	Glyma.04G213100.1	100	196	2	2628	5897	2587	5856	0	4942

TRINITY_DN15847_c1_g2_i3	Glyma.08G302000.1	100	351	12	136	3394	38	3293	0	3943

TRINITY_DN12794_c0_g1_i1	Glyma.09G068600.1	98.1512605	170	2	426	3687	3268	1	0	5055

TRINITY_DN12794_c0_g1_i1	Glyma.15G176400.1	97.32937685	191	1	424	3687	3267	1	0	4955

TRINITY_DN13689_c0_g1_i2	Glyma.06G139800.5	100	255	9	258	3520	1	3254	0	4521

TRINITY_DN13689_c0_g1_i2	Glyma.06G139800.7	96.70886076	255	9	258	3520	1	3254	0	4521

TRINITY_DN13689_c0_g1_i2	Glyma.06G139800.9	98.7012987	255	9	258	3520	1	3254	0	4521

TRINITY_DN13689_c0_g1_i2	Glyma.06G139800.4	96.15384615	253	10	258	3520	1	3245	0	4475

TRINITY_DN16026_c0_g2_i3	Glyma.11G112700.1	98.26589595	253	16	316	3573	1	3228	0	4333

TRINITY_DN16026_c0_g2_i9	Glyma.11G112700.1	99.33333333	253	16	316	3573	1	3228	0	4333

TRINITY_DN14877_c0_g1_i3	Glyma.19G016300.1	95.95744681	192	8	1196	4443	3392	133	0	4813

TRINITY_DN13016_c0_g1_i1	Glyma.09G226400.1	100	182	12	665	3912	3263	1	0	4887

TRINITY_DN12563_c0_g1_i2	Glyma.05G221300.2	100	541	43	297	3546	2	3208	0	2560

TRINITY_DN16026_c0_g2_i1	Glyma.11G112700.1	97.10144928	375	39	316	3560	1	3221	0	3539

TRINITY_DN15593_c0_g2_i1	Glyma.06G025400.2	97.61904762	531	46	165	3371	1	3243	0	2593

TRINITY_DN15593_c0_g2_i1	Glyma.06G025400.3	100	531	46	165	3371	1	3243	0	2593

TRINITY_DN15593_c0_g2_i1	Glyma.06G025400.1	100	531	46	165	3371	1	3243	0	2593

TRINITY_DN15593_c0_g2_i1	Glyma.06G025400.4	100	531	46	165	3371	1	3243	0	2593

TRINITY_DN13689_c0_g1_i2	Glyma.04G225000.1	100	265	2	258	3520	1	3257	0	4519

TRINITY_DN16309_c0_g1_i1	Glyma.11G033600.5	98.21428571	257	32	3318	6496	3038	6288	0	3982

TRINITY_DN16309_c0_g1_i1	Glyma.11G033600.7	98.21428571	257	32	3318	6496	3038	6288	0	3982

TRINITY_DN16309_c0_g1_i1	Glyma.11G033600.6	98	257	32	3318	6496	3038	6288	0	3982

TRINITY_DN16309_c0_g1_i4	Glyma.11G033600.5	99.30555556	257	32	3384	6562	3038	6288	0	3982

TRINITY_DN16309_c0_g1_i4	Glyma.11G033600.7	99.29824561	257	32	3384	6562	3038	6288	0	3982

TRINITY_DN16309_c0_g1_i4	Glyma.11G033600.6	99.31506849	257	32	3384	6562	3038	6288	0	3982

TRINITY_DN14877_c0_g1_i3	Glyma.19G016300.2	98.66666667	192	7	1196	4443	3389	133	0	4826

TRINITY_DN15073_c0_g1_i2	Glyma.14G132500.1	95.5654102	458	25	266	3507	3242	1	0	3229

TRINITY_DN16151_c0_g2_i3	Glyma.08G292900.3	96.875	409	40	872	4094	830	4034	0	3138

TRINITY_DN16151_c0_g2_i5	Glyma.08G292900.3	95.18072289	409	40	1133	4355	830	4034	0	3138

TRINITY_DN5160_c0_g1_i2	Glyma.02G219200.5	97.31182796	209	4	217	3478	1087	4339	0	4807

TRINITY_DN14654_c0_g2_i1	Glyma.08G014800.2	96.46393211	204	8	279	3528	3249	3	0	4719

TRINITY_DN14654_c0_g2_i1	Glyma.08G014800.1	96.46393211	204	8	279	3528	3249	3	0	4719

TRINITY_DN16254_c0_g1_i2	Glyma.07G004300.1	96.21621622	714	91	397	3594	3207	4	0	1303

TRINITY_DN15847_c1_g2_i1	Glyma.18G119000.1	100	313	5	135	3385	28	3284	0	4209

TRINITY_DN15073_c0_g1_i3	Glyma.04G074300.10	100	221	6	368	3616	3258	1	0	4734

TRINITY_DN15073_c0_g1_i3	Glyma.04G074300.11	95.18900344	221	6	368	3616	3258	1	0	4734

TRINITY_DN15073_c0_g1_i3	Glyma.04G074300.3	98.13664596	221	6	368	3616	3258	1	0	4734

TRINITY_DN15073_c0_g1_i3	Glyma.04G074300.4	100	221	6	368	3616	3258	1	0	4734

TRINITY_DN15073_c0_g1_i3	Glyma.06G075500.2	97.08737864	213	4	368	3616	3258	1	0	4778

TRINITY_DN15073_c0_g1_i3	Glyma.06G075500.6	97.71428571	213	4	368	3616	3258	1	0	4778

TRINITY_DN15073_c0_g1_i3	Glyma.06G075500.3	95.66360053	213	4	368	3616	3258	1	0	4778

TRINITY_DN14654_c0_g2_i1	Glyma.05G208100.2	96.46464646	206	8	279	3524	3243	1	0	4700

TRINITY_DN14654_c0_g2_i1	Glyma.05G208100.3	95.89041096	206	8	279	3524	3243	1	0	4700

TRINITY_DN15073_c0_g1_i2	Glyma.17G201000.1	100	500	16	266	3507	3245	1	0	3070

TRINITY_DN15073_c0_g1_i2	Glyma.17G201000.2	98.18181818	500	16	266	3507	3245	1	0	3070

TRINITY_DN16308_c1_g3_i2	Glyma.20G050700.3	99.07407407	585	37	146	3383	9	3234	0	2462

TRINITY_DN16308_c1_g3_i2	Glyma.20G050700.2	100	585	37	146	3383	9	3234	0	2462

TRINITY_DN15593_c0_g2_i1	Glyma.04G025400.2	97.33333333	539	41	165	3371	1	3237	0	2588

TRINITY_DN15593_c0_g2_i1	Glyma.04G025400.1	100	539	41	165	3371	1	3237	0	2588

TRINITY_DN15847_c1_g2_i1	Glyma.08G302000.1	100	309	3	136	3385	38	3293	0	4263

TRINITY_DN14563_c0_g1_i1	Glyma.18G218900.1	96.55172414	228	18	349	3583	3999	762	0	4497

TRINITY_DN14563_c0_g1_i3	Glyma.18G218900.1	98.66666667	228	18	349	3583	3999	762	0	4497

TRINITY_DN11500_c0_g1_i1	Glyma.18G202700.2	98.95833333	244	4	121	3375	1	3240	0	4562

TRINITY_DN11500_c0_g1_i2	Glyma.18G202700.2	96.22641509	244	5	121	3364	1	3240	0	4492

TRINITY_DN3960_c0_g2_i1	Glyma.11G101700.1	100	207	17	613	3847	948	4179	0	4586

TRINITY_DN3960_c0_g2_i1	Glyma.11G101700.5	100	207	17	613	3847	948	4179	0	4586

TRINITY_DN3960_c0_g2_i1	Glyma.11G101700.3	98.97260274	207	17	613	3847	888	4119	0	4586

TRINITY_DN15073_c0_g1_i3	Glyma.14G132500.1	95.83333333	457	25	383	3616	3234	1	0	3219

TRINITY_DN15073_c0_g1_i1	Glyma.14G132500.1	98.26589595	427	25	1	3234	3234	1	0	3386

TRINITY_DN6214_c0_g2_i1	Glyma.19G200400.1	97.22222222	209	10	211	3452	629	3870	0	4708

TRINITY_DN10787_c0_g1_i1	Glyma.01G244900.1	97.21115538	198	25	20	3246	1	3236	0	4632

TRINITY_DN16254_c0_g1_i3	Glyma.07G004300.1	99.05660377	733	82	397	3594	3207	4	0	1286

TRINITY_DN15073_c0_g1_i2	Glyma.04G074300.15	98.18181818	221	6	265	3507	3252	1	0	4723

TRINITY_DN15073_c0_g1_i2	Glyma.04G074300.16	98.18181818	221	6	265	3507	3252	1	0	4723

TRINITY_DN15073_c0_g1_i2	Glyma.04G074300.12	95.38461538	221	6	265	3507	3252	1	0	4723

TRINITY_DN15073_c0_g1_i2	Glyma.04G074300.13	96.42857143	221	6	265	3507	3252	1	0	4723

TRINITY_DN15073_c0_g1_i2	Glyma.04G074300.14	96.42857143	221	6	265	3507	3252	1	0	4723

TRINITY_DN3960_c0_g2_i1	Glyma.11G101700.4	97.74647887	207	16	613	3847	948	4176	0	4601

TRINITY_DN3960_c0_g2_i1	Glyma.11G101700.2	100	207	16	613	3847	948	4176	0	4601

TRINITY_DN16026_c0_g2_i5	Glyma.11G112700.1	96.32352941	205	6	316	3561	1	3228	0	4676

TRINITY_DN16026_c0_g2_i6	Glyma.11G112700.1	100	205	6	316	3561	1	3228	0	4676

TRINITY_DN16026_c0_g2_i4	Glyma.11G112700.1	96.98078683	327	29	316	3548	1	3221	0	3882

TRINITY_DN16026_c0_g2_i4	Glyma.04G009100.1	99.17695473	296	13	316	3552	1	3213	0	4028

TRINITY_DN16026_c0_g2_i4	Glyma.06G009000.1	99.30313589	289	13	316	3552	1	3213	0	4067

TRINITY_DN6214_c0_g2_i1	Glyma.03G202700.1	97.22222222	208	9	211	3452	635	3876	0	4735

TRINITY_DN16026_c0_g2_i1	Glyma.04G009100.1	96.55172414	246	5	316	3564	1	3213	0	4394

TRINITY_DN16026_c0_g2_i1	Glyma.06G009000.1	100	238	5	316	3564	1	3213	0	4438

TRINITY_DN15073_c0_g1_i3	Glyma.17G201000.1	100	499	16	383	3616	3237	1	0	3061

TRINITY_DN15073_c0_g1_i3	Glyma.17G201000.2	100	499	16	383	3616	3237	1	0	3061

TRINITY_DN9338_c0_g1_i1	Glyma.15G019200.5	97.28506787	509	21	896	4133	3229	1	0	3001

TRINITY_DN9338_c0_g1_i1	Glyma.15G019200.4	98.9010989	509	21	896	4133	3229	1	0	3001

TRINITY_DN9338_c0_g1_i1	Glyma.15G019200.2	97.97297297	509	21	896	4133	3229	1	0	3001

TRINITY_DN16151_c0_g2_i2	Glyma.08G292900.3	95.38461538	403	46	1133	4337	830	4034	0	3221

TRINITY_DN16151_c0_g2_i6	Glyma.08G292900.3	100	403	46	872	4076	830	4034	0	3221

TRINITY_DN9262_c0_g1_i1	Glyma.05G212000.3	99.29078014	227	21	1	3191	616	3855	0	4331

TRINITY_DN9262_c0_g1_i2	Glyma.05G212000.3	100	227	21	1	3191	616	3855	0	4331

TRINITY_DN15073_c0_g1_i3	Glyma.04G074300.15	97.72727273	221	7	380	3616	3247	1	0	4708

TRINITY_DN15073_c0_g1_i3	Glyma.04G074300.16	97.72727273	221	7	380	3616	3247	1	0	4708

TRINITY_DN15073_c0_g1_i3	Glyma.04G074300.12	99.0990991	221	7	380	3616	3247	1	0	4708

TRINITY_DN15073_c0_g1_i3	Glyma.04G074300.13	99.0990991	221	7	380	3616	3247	1	0	4708

TRINITY_DN15073_c0_g1_i3	Glyma.04G074300.14	96.66666667	221	7	380	3616	3247	1	0	4708

TRINITY_DN15073_c0_g1_i2	Glyma.04G074300.10	95.65217391	221	7	270	3507	3246	1	0	4708

TRINITY_DN15073_c0_g1_i2	Glyma.04G074300.11	95.65217391	221	7	270	3507	3246	1	0	4708

TRINITY_DN15073_c0_g1_i2	Glyma.04G074300.3	95.65217391	221	7	270	3507	3246	1	0	4708

TRINITY_DN15073_c0_g1_i2	Glyma.04G074300.4	95.65217391	221	7	270	3507	3246	1	0	4708

TRINITY_DN14118_c0_g2_i1	Glyma.14G218000.1	100	218	15	485	3691	3228	1	0	4407

TRINITY_DN15073_c0_g1_i2	Glyma.06G075500.2	100	213	5	270	3507	3246	1	0	4752

TRINITY_DN15073_c0_g1_i2	Glyma.06G075500.6	96.70588235	213	5	270	3507	3246	1	0	4752

TRINITY_DN15073_c0_g1_i2	Glyma.06G075500.3	98.18181818	213	5	270	3507	3246	1	0	4752

TRINITY_DN15073_c0_g1_i1	Glyma.17G201000.1	98.26589595	476	11	1	3234	3237	1	0	3221

TRINITY_DN15073_c0_g1_i1	Glyma.17G201000.2	100	476	11	1	3234	3237	1	0	3221

TRINITY_DN10077_c0_g1_i1	Glyma.11G102200.1	100	292	22	249	3470	3297	67	0	4124

TRINITY_DN16247_c0_g1_i2	Glyma.12G237000.1	100	277	4	366	3596	3240	1	0	4346

TRINITY_DN15073_c0_g1_i1	Glyma.06G075500.4	100	270	6	1	3234	3243	1	0	4423

TRINITY_DN15073_c0_g1_i1	Glyma.06G075500.5	100	270	6	1	3234	3243	1	0	4423

TRINITY_DN15073_c0_g1_i1	Glyma.06G075500.2	100	270	6	1	3234	3243	1	0	4423

TRINITY_DN15073_c0_g1_i1	Glyma.06G075500.6	96.48241206	270	6	1	3234	3243	1	0	4423

TRINITY_DN15073_c0_g1_i1	Glyma.06G075500.3	98.55072464	270	6	1	3234	3243	1	0	4423

TRINITY_DN15073_c0_g1_i1	Glyma.06G075500.1	97.5308642	270	6	1	3234	3243	1	0	4423

TRINITY_DN15073_c0_g1_i2	Glyma.04G074300.7	100	221	6	273	3507	3244	1	0	4708

TRINITY_DN15073_c0_g1_i2	Glyma.04G074300.8	95.65217391	221	6	273	3507	3244	1	0	4708

TRINITY_DN15073_c0_g1_i2	Glyma.04G074300.9	95.65217391	221	6	273	3507	3244	1	0	4708

TRINITY_DN15073_c0_g1_i2	Glyma.04G074300.5	95.65217391	221	6	273	3507	3244	1	0	4708

TRINITY_DN15073_c0_g1_i2	Glyma.04G074300.6	95.65217391	221	6	273	3507	3244	1	0	4708

TRINITY_DN15073_c0_g1_i2	Glyma.04G074300.2	97.19626168	221	6	273	3507	3244	1	0	4708

TRINITY_DN15073_c0_g1_i2	Glyma.04G074300.1	97.2972973	221	6	273	3507	3244	1	0	4708

TRINITY_DN15073_c0_g1_i2	Glyma.06G075500.4	100	213	4	273	3507	3244	1	0	4752

TRINITY_DN15073_c0_g1_i2	Glyma.06G075500.5	100	213	4	273	3507	3244	1	0	4752

TRINITY_DN15073_c0_g1_i2	Glyma.06G075500.1	98.18181818	213	4	273	3507	3244	1	0	4752

TRINITY_DN16247_c0_g1_i2	Glyma.13G202500.1	98.44632768	282	1	366	3596	3243	1	0	4349

TRINITY_DN16247_c0_g1_i2	Glyma.13G202500.2	98.44660194	282	1	366	3596	3243	1	0	4349

TRINITY_DN15073_c0_g1_i1	Glyma.04G074300.15	100	273	6	1	3234	3243	1	0	4418

TRINITY_DN15073_c0_g1_i1	Glyma.04G074300.16	98.67549669	273	6	1	3234	3243	1	0	4418

TRINITY_DN15073_c0_g1_i1	Glyma.04G074300.12	97.8021978	273	6	1	3234	3243	1	0	4418

TRINITY_DN15073_c0_g1_i1	Glyma.04G074300.13	95.94882729	273	6	1	3234	3243	1	0	4418

TRINITY_DN15073_c0_g1_i1	Glyma.04G074300.14	98.14814815	273	6	1	3234	3243	1	0	4418

TRINITY_DN15073_c0_g1_i1	Glyma.04G074300.7	98.57142857	273	6	1	3234	3243	1	0	4418

TRINITY_DN15073_c0_g1_i1	Glyma.04G074300.8	95.8041958	273	6	1	3234	3243	1	0	4418

TRINITY_DN15073_c0_g1_i1	Glyma.04G074300.9	97.5638051	273	6	1	3234	3243	1	0	4418

TRINITY_DN15073_c0_g1_i1	Glyma.04G074300.10	97.56468798	273	6	1	3234	3243	1	0	4418

TRINITY_DN15073_c0_g1_i1	Glyma.04G074300.11	98.7012987	273	6	1	3234	3243	1	0	4418

TRINITY_DN15073_c0_g1_i1	Glyma.04G074300.3	95.2238806	273	6	1	3234	3243	1	0	4418

TRINITY_DN15073_c0_g1_i1	Glyma.04G074300.4	95.2238806	273	6	1	3234	3243	1	0	4418

TRINITY_DN15073_c0_g1_i1	Glyma.04G074300.5	96.77419355	273	6	1	3234	3243	1	0	4418

TRINITY_DN15073_c0_g1_i1	Glyma.04G074300.6	96.77419355	273	6	1	3234	3243	1	0	4418

TRINITY_DN15073_c0_g1_i1	Glyma.04G074300.2	100	273	6	1	3234	3243	1	0	4418

TRINITY_DN15073_c0_g1_i1	Glyma.04G074300.1	98.26589595	273	6	1	3234	3243	1	0	4418

TRINITY_DN15073_c0_g1_i3	Glyma.04G074300.7	96.66666667	221	6	383	3616	3243	1	0	4706

TRINITY_DN15073_c0_g1_i3	Glyma.04G074300.8	97.71689498	221	6	383	3616	3243	1	0	4706

TRINITY_DN15073_c0_g1_i3	Glyma.04G074300.9	97.85407725	221	6	383	3616	3243	1	0	4706

TRINITY_DN15073_c0_g1_i3	Glyma.04G074300.5	100	221	6	383	3616	3243	1	0	4706

TRINITY_DN15073_c0_g1_i3	Glyma.04G074300.6	95.52238806	221	6	383	3616	3243	1	0	4706

TRINITY_DN15073_c0_g1_i3	Glyma.04G074300.2	95.52238806	221	6	383	3616	3243	1	0	4706

TRINITY_DN15073_c0_g1_i3	Glyma.04G074300.1	100	221	6	383	3616	3243	1	0	4706

TRINITY_DN15073_c0_g1_i3	Glyma.06G075500.4	95.66395664	213	4	383	3616	3243	1	0	4750

TRINITY_DN15073_c0_g1_i3	Glyma.06G075500.5	97.08737864	213	4	383	3616	3243	1	0	4750

TRINITY_DN15073_c0_g1_i3	Glyma.06G075500.1	97.08737864	213	4	383	3616	3243	1	0	4750

TRINITY_DN10715_c0_g1_i2	Glyma.13G261000.1	95.83333333	190	0	1	3243	2656	5898	0	4937

TRINITY_DN10715_c0_g1_i2	Glyma.15G245800.1	95.83333333	182	0	1	3243	2656	5898	0	4981

TRINITY_DN15657_c0_g1_i3	Glyma.03G009200.3	98.38709677	445	27	1	3232	1729	4930	0	3199

TRINITY_DN15657_c0_g1_i3	Glyma.03G009200.1	98.93617021	445	27	1	3232	1729	4930	0	3199

TRINITY_DN15657_c0_g1_i3	Glyma.03G009200.2	98.68995633	445	27	1	3232	1729	4930	0	3199

TRINITY_DN15657_c0_g1_i3	Glyma.09G259400.1	96.11231102	294	9	1	3232	1780	4996	0	4132

TRINITY_DN15657_c0_g1_i3	Glyma.09G259400.2	96.11650485	294	9	1	3232	1780	4996	0	4132

TRINITY_DN15657_c0_g1_i3	Glyma.09G259400.3	95.55555556	294	9	1	3232	1780	4996	0	4132

TRINITY_DN12453_c0_g1_i1	Glyma.11G053900.1	97.32142857	261	8	306	3542	1	3228	0	4429

TRINITY_DN12453_c0_g1_i1	Glyma.01G188200.3	100	248	5	306	3542	1	3237	0	4560

TRINITY_DN12453_c0_g1_i1	Glyma.01G188200.4	98.4375	248	5	306	3542	1	3237	0	4560

TRINITY_DN12453_c0_g1_i1	Glyma.01G188200.1	97.32142857	248	5	306	3542	1	3237	0	4560

TRINITY_DN12453_c0_g1_i1	Glyma.01G188200.2	97.32142857	248	6	306	3542	1	3231	0	4521

TRINITY_DN15657_c0_g1_i3	Glyma.07G070700.1	95.69160998	445	34	1	3232	1729	4915	0	3123

TRINITY_DN15657_c0_g1_i3	Glyma.07G070700.3	95.68965517	445	34	1	3232	883	4069	0	3123

TRINITY_DN15657_c0_g1_i3	Glyma.07G070700.2	95.68965517	445	34	1	3232	883	4069	0	3123

TRINITY_DN14062_c0_g1_i3	Glyma.09G194200.1	95.04132231	227	13	402	3626	3228	1	0	4551

TRINITY_DN14062_c0_g1_i3	Glyma.09G194200.3	96.72131148	225	17	402	3626	3213	1	0	4466

TRINITY_DN15748_c0_g1_i2	Glyma.08G283700.2	100	178	2	680	3898	4062	828	0	4852

TRINITY_DN15748_c0_g1_i3	Glyma.08G283700.2	98.0952381	178	2	37	3255	4062	828	0	4852

TRINITY_DN14062_c0_g1_i3	Glyma.09G194200.4	99.02439024	227	12	402	3626	3225	1	0	4566

TRINITY_DN14062_c0_g1_i3	Glyma.09G194200.2	96.72131148	225	16	402	3626	3210	1	0	4479

TRINITY_DN326_c0_g1_i1	Glyma.03G253000.5	97.59036145	112	1	777	4009	2	3237	0	5336

TRINITY_DN326_c0_g1_i2	Glyma.03G253000.5	97.79411765	112	1	732	3964	2	3237	0	5336

TRINITY_DN13491_c0_g1_i1	Glyma.15G074000.2	98.95287958	222	10	1	3210	2182	5400	0	4479

TRINITY_DN13491_c0_g1_i1	Glyma.15G074000.1	99.04534606	222	10	1	3210	2182	5400	0	4479

TRINITY_DN13491_c0_g1_i1	Glyma.15G074000.4	100	222	10	1	3210	2182	5400	0	4479

TRINITY_DN13491_c0_g1_i1	Glyma.15G074000.3	100	222	10	1	3210	2182	5400	0	4479

TRINITY_DN13491_c0_g1_i2	Glyma.15G074000.2	99.15966387	222	10	1	3210	2182	5400	0	4479

TRINITY_DN13491_c0_g1_i2	Glyma.15G074000.1	99.15966387	222	10	1	3210	2182	5400	0	4479

TRINITY_DN13491_c0_g1_i2	Glyma.15G074000.4	99.15966387	222	10	1	3210	2182	5400	0	4479

TRINITY_DN13491_c0_g1_i2	Glyma.15G074000.3	98.80952381	222	10	1	3210	2182	5400	0	4479

TRINITY_DN13491_c0_g1_i3	Glyma.15G074000.2	97.72727273	222	10	1	3210	2182	5400	0	4479

TRINITY_DN13491_c0_g1_i3	Glyma.15G074000.1	98.92008639	222	10	1	3210	2182	5400	0	4479

TRINITY_DN13491_c0_g1_i3	Glyma.15G074000.4	98.92086331	222	10	1	3210	2182	5400	0	4479

TRINITY_DN13491_c0_g1_i3	Glyma.15G074000.3	98.60788863	222	10	1	3210	2182	5400	0	4479

TRINITY_DN13491_c0_g1_i4	Glyma.15G074000.2	100	222	10	1	3210	2182	5400	0	4479

TRINITY_DN13491_c0_g1_i4	Glyma.15G074000.1	98.9010989	222	10	1	3210	2182	5400	0	4479

TRINITY_DN13491_c0_g1_i4	Glyma.15G074000.4	98.9010989	222	10	1	3210	2182	5400	0	4479

TRINITY_DN13491_c0_g1_i4	Glyma.15G074000.3	96.96969697	222	10	1	3210	2182	5400	0	4479

TRINITY_DN13491_c0_g1_i5	Glyma.15G074000.2	98.55072464	222	10	1	3210	2182	5400	0	4479

TRINITY_DN13491_c0_g1_i5	Glyma.15G074000.1	100	222	10	1	3210	2182	5400	0	4479

TRINITY_DN13491_c0_g1_i5	Glyma.15G074000.4	100	222	10	1	3210	2182	5400	0	4479

TRINITY_DN13491_c0_g1_i5	Glyma.15G074000.3	100	222	10	1	3210	2182	5400	0	4479

TRINITY_DN13491_c0_g1_i6	Glyma.15G074000.2	95.71150097	222	10	1	3210	2182	5400	0	4479

TRINITY_DN13491_c0_g1_i6	Glyma.15G074000.1	100	222	10	1	3210	2182	5400	0	4479

TRINITY_DN13491_c0_g1_i6	Glyma.15G074000.4	100	222	10	1	3210	2182	5400	0	4479

TRINITY_DN13491_c0_g1_i6	Glyma.15G074000.3	100	222	10	1	3210	2182	5400	0	4479

TRINITY_DN14118_c0_g2_i1	Glyma.17G257100.1	98.46938776	221	23	485	3691	3210	1	0	4407

TRINITY_DN14118_c0_g2_i4	Glyma.17G257100.1	99.3902439	221	24	485	3679	3210	1	0	4329

TRINITY_DN14118_c0_g2_i1	Glyma.17G257100.2	98.47328244	221	24	485	3691	3198	1	0	4329

TRINITY_DN14118_c0_g2_i4	Glyma.14G218000.1	97.71167048	218	14	485	3679	3228	1	0	4462

TRINITY_DN15748_c0_g1_i3	Glyma.08G283700.1	98.82352941	180	1	21	3255	4059	828	0	4957

TRINITY_DN15748_c0_g1_i3	Glyma.18G142500.1	96.80365297	171	1	21	3255	4059	828	0	5007

TRINITY_DN15748_c0_g1_i3	Glyma.18G142500.2	96.80365297	171	1	21	3255	4059	828	0	5007

TRINITY_DN16040_c0_g1_i4	Glyma.14G044500.3	99.28315412	223	2	485	3718	3228	1	0	4698

TRINITY_DN16040_c0_g1_i3	Glyma.14G044500.3	97.43589744	196	2	739	3972	3228	1	0	4848

TRINITY_DN16040_c0_g1_i1	Glyma.14G044500.3	96	173	2	485	3718	3228	1	0	4975

TRINITY_DN16040_c0_g1_i2	Glyma.14G044500.3	96.59090909	146	2	739	3972	3228	1	0	5125

TRINITY_DN15815_c0_g2_i2	Glyma.12G147000.2	96.03729604	284	10	466	3689	3213	1	0	4209

TRINITY_DN16247_c0_g1_i2	Glyma.09G103000.1	97.06314244	208	8	366	3596	3225	4	0	4734

TRINITY_DN16247_c0_g1_i2	Glyma.09G103000.2	97.06342991	208	8	366	3596	3225	4	0	4734

TRINITY_DN9957_c0_g1_i1	Glyma.11G039900.1	99.11504425	182	4	579	3809	12966	9736	0	4937

TRINITY_DN16345_c0_g1_i2	Glyma.07G093700.2	95.34883721	159	2	642	3874	3527	299	0	5064

TRINITY_DN15593_c0_g2_i1	Glyma.14G217900.2	96.42857143	247	16	165	3371	1	3216	0	4335

TRINITY_DN15593_c0_g2_i1	Glyma.14G217900.1	96.15384615	247	16	165	3371	1	3216	0	4335

TRINITY_DN16247_c0_g1_i2	Glyma.15G275000.1	100	202	9	366	3594	3222	6	0	4745

TRINITY_DN6009_c0_g1_i1	Glyma.19G087900.1	97.98657718	276	22	309	3500	3213	1	0	4080

TRINITY_DN15847_c1_g2_i3	Glyma.18G119000.3	97.64705882	350	14	179	3394	3	3215	0	3836

TRINITY_DN15593_c0_g2_i1	Glyma.17G256900.1	96.42857143	240	17	165	3371	1	3213	0	4399

TRINITY_DN13547_c0_g1_i2	Glyma.07G045600.2	98.03921569	239	12	2606	5827	2191	5403	0	4501

TRINITY_DN16373_c3_g5_i6	Glyma.07G027800.1	98.05825243	233	30	714	3916	3189	1	0	4257

TRINITY_DN16373_c3_g5_i8	Glyma.07G027800.1	98.05825243	233	30	714	3916	3189	1	0	4257

TRINITY_DN8943_c0_g1_i1	Glyma.20G089300.3	100	377	11	1268	4474	3230	9	0	3709

TRINITY_DN13689_c0_g1_i2	Glyma.06G139800.6	99.00990099	252	21	258	3454	1	3215	0	4292

TRINITY_DN13689_c0_g1_i2	Glyma.06G139800.8	99.00990099	252	21	258	3454	1	3215	0	4292

TRINITY_DN16245_c0_g5_i1	Glyma.06G208900.2	96.34408602	455	17	381	3595	3230	16	0	3306

TRINITY_DN16245_c0_g5_i1	Glyma.06G208900.3	99.11504425	455	17	381	3595	3230	16	0	3306

TRINITY_DN16233_c0_g2_i4	Glyma.14G196800.1	100	325	3	408	3623	3401	180	0	4078

TRINITY_DN16233_c0_g2_i4	Glyma.14G196800.2	100	325	3	408	3623	3284	63	0	4078

TRINITY_DN16233_c0_g2_i4	Glyma.14G196800.3	100	325	3	408	3623	3284	63	0	4078

TRINITY_DN16233_c0_g2_i1	Glyma.14G196800.1	100	241	3	238	3453	3401	180	0	4543

TRINITY_DN16233_c0_g2_i1	Glyma.14G196800.2	95.12195122	241	3	238	3453	3284	63	0	4543

TRINITY_DN16233_c0_g2_i1	Glyma.14G196800.3	95.12195122	241	3	238	3453	3284	63	0	4543

TRINITY_DN14062_c0_g1_i3	Glyma.01G146600.2	97.69585253	213	0	402	3626	3225	1	0	4776

TRINITY_DN15811_c0_g1_i1	Glyma.15G097000.1	98.92473118	170	4	83	3307	1	3219	0	4975

TRINITY_DN15811_c0_g1_i1	Glyma.13G215900.1	98.92473118	159	8	83	3307	1	3204	0	4939

TRINITY_DN16245_c0_g5_i2	Glyma.06G208900.2	95.37037037	404	14	381	3595	3230	16	0	3611

TRINITY_DN16245_c0_g5_i2	Glyma.06G208900.3	96.55172414	404	14	381	3595	3230	16	0	3611

TRINITY_DN16262_c0_g1_i9	Glyma.05G191200.1	96.64179104	290	6	49	3265	120	3339	0	4287

TRINITY_DN14118_c0_g2_i4	Glyma.17G257100.2	98.23434992	221	23	485	3679	3198	1	0	4385

TRINITY_DN16262_c0_g1_i1	Glyma.04G213400.1	96.13733906	392	29	53	3268	124	3300	0	3448

TRINITY_DN16262_c0_g1_i1	Glyma.04G213400.2	96.79300292	390	31	53	3268	124	3297	0	3441

TRINITY_DN13497_c0_g1_i1	Glyma.15G085900.1	96.2962963	210	5	508	3714	1	3222	0	4689

TRINITY_DN13497_c0_g1_i1	Glyma.13G226500.1	100	201	6	508	3714	1	3210	0	4662

TRINITY_DN252_c0_g1_i1	Glyma.08G092000.1	100	194	8	186	3397	3432	216	0	4778

TRINITY_DN16262_c0_g1_i1	Glyma.06G152800.1	96.79300292	385	31	53	3268	124	3288	0	3421

TRINITY_DN15642_c0_g1_i5	Glyma.12G017600.1	100	379	15	1	3217	228	3426	0	3681

TRINITY_DN15642_c0_g1_i5	Glyma.12G017600.2	100	379	15	1	3217	228	3426	0	3681

TRINITY_DN16262_c0_g1_i9	Glyma.08G156400.1	96.82539683	290	1	49	3265	120	3339	0	4320

TRINITY_DN16262_c0_g1_i1	Glyma.05G191200.1	95.29411765	210	0	49	3268	120	3339	0	4783

TRINITY_DN16262_c0_g1_i1	Glyma.08G156400.1	95.3443259	195	0	49	3268	120	3339	0	4867

TRINITY_DN12099_c0_g1_i1	Glyma.04G071900.1	100	301	37	446	3612	161	3336	0	3663

TRINITY_DN13547_c0_g1_i1	Glyma.16G013500.1	98.05825243	228	11	356	3555	1	3212	0	4514

TRINITY_DN13547_c0_g1_i1	Glyma.16G013500.2	98.05825243	228	11	356	3555	1	3212	0	4514

TRINITY_DN13547_c0_g1_i1	Glyma.16G013500.3	98.05825243	228	11	356	3555	1	3212	0	4514

TRINITY_DN15897_c0_g2_i2	Glyma.14G029100.1	97.47634069	212	18	155	3345	3192	1	0	4414

TRINITY_DN16262_c0_g1_i9	Glyma.06G152800.1	95.60117302	306	30	53	3265	124	3288	0	3873

TRINITY_DN16345_c0_g1_i6	Glyma.09G182600.1	98.4375	260	12	440	3652	3717	511	0	4399

TRINITY_DN5451_c0_g1_i1	Glyma.11G095800.2	100	245	8	1	3206	2009	5208	0	4392

TRINITY_DN13547_c0_g1_i1	Glyma.07G045600.1	98.03921569	229	5	356	3555	1	3215	0	4538

TRINITY_DN15070_c0_g1_i3	Glyma.09G256700.1	99.21875	159	0	64	3281	3218	1	0	5062

TRINITY_DN15070_c0_g1_i3	Glyma.18G235800.1	100	134	0	64	3281	3218	1	0	5201

TRINITY_DN16262_c0_g1_i9	Glyma.04G213400.1	98.94179894	319	24	53	3265	124	3300	0	3890

TRINITY_DN16262_c0_g1_i9	Glyma.04G213400.2	100	317	26	53	3265	124	3297	0	3880

TRINITY_DN16345_c0_g1_i6	Glyma.07G093700.1	98.46153846	262	6	440	3652	3729	517	0	4438

TRINITY_DN15642_c0_g1_i5	Glyma.01G232500.2	97.11538462	261	3	1	3217	222	3432	0	4457

TRINITY_DN15642_c0_g1_i5	Glyma.01G232500.1	97.11538462	261	3	1	3217	222	3432	0	4457

TRINITY_DN15642_c0_g1_i5	Glyma.11G010400.1	99.0228013	254	3	1	3217	222	3432	0	4495

TRINITY_DN15847_c1_g2_i1	Glyma.18G119000.3	96.25	309	5	179	3385	3	3215	0	4150

TRINITY_DN8943_c0_g1_i1	Glyma.03G226200.3	100	174	2	1268	4482	3215	1	0	4963

TRINITY_DN16345_c0_g1_i6	Glyma.08G044700.1	98.4375	312	6	446	3652	3699	496	0	4119

TRINITY_DN16345_c0_g1_i6	Glyma.08G044700.6	98.46153846	312	6	446	3652	3420	217	0	4119

TRINITY_DN5451_c0_g1_i1	Glyma.11G095800.1	97.32540862	245	8	1	3200	2009	5202	0	4381

TRINITY_DN5451_c0_g1_i1	Glyma.11G095800.3	96.94656489	245	8	1	3200	2009	5202	0	4381

TRINITY_DN16233_c0_g2_i4	Glyma.02G229700.1	96.44970414	315	2	407	3611	3279	72	0	4126

TRINITY_DN16233_c0_g2_i4	Glyma.02G229700.2	96.55172414	315	2	407	3611	3279	72	0	4126

TRINITY_DN16345_c0_g1_i6	Glyma.05G237500.1	98.4375	305	3	446	3652	3705	496	0	4207

TRINITY_DN16233_c0_g2_i1	Glyma.02G229700.1	98.26839827	243	2	237	3441	3279	72	0	4525

TRINITY_DN16233_c0_g2_i1	Glyma.02G229700.2	100	243	2	237	3441	3279	72	0	4525

TRINITY_DN16426_c2_g1_i3	Glyma.13G152600.1	96.66666667	271	4	358	3561	1	3210	0	4388

TRINITY_DN16426_c2_g1_i1	Glyma.13G152600.1	100	239	4	278	3481	1	3210	0	4566

TRINITY_DN16254_c0_g1_i3	Glyma.08G222200.1	99.45054945	709	80	397	3550	3213	57	0	1306

TRINITY_DN16254_c0_g1_i2	Glyma.08G222200.1	95.08196721	708	80	397	3550	3213	57	0	1323

TRINITY_DN16426_c2_g1_i1	Glyma.13G152700.1	97.26027397	321	2	278	3481	1	3207	0	4124

TRINITY_DN16426_c2_g1_i1	Glyma.18G044600.1	97.26027397	313	2	278	3481	1	3207	0	4169

TRINITY_DN16426_c2_g1_i3	Glyma.13G152700.1	98.57142857	274	2	358	3561	1	3207	0	4385

TRINITY_DN16426_c2_g1_i3	Glyma.18G044600.1	100	270	2	358	3561	1	3207	0	4407

TRINITY_DN6178_c0_g1_i1	Glyma.08G086100.5	100	226	8	1	3205	3308	6495	0	4534

TRINITY_DN6178_c0_g1_i1	Glyma.08G086100.4	96.1038961	226	8	1	3205	3308	6495	0	4534

TRINITY_DN6178_c0_g1_i1	Glyma.08G086100.3	100	226	8	1	3205	3308	6495	0	4534

TRINITY_DN6178_c0_g1_i1	Glyma.08G086100.1	96.875	226	8	1	3205	3308	6495	0	4534

TRINITY_DN6178_c0_g1_i1	Glyma.08G086100.2	100	226	8	1	3205	3191	6378	0	4534

TRINITY_DN14752_c0_g1_i1	Glyma.16G038900.2	96	206	13	530	3706	51	3236	0	4473

TRINITY_DN14752_c0_g1_i1	Glyma.16G038900.1	96.03174603	206	13	530	3706	51	3236	0	4473

TRINITY_DN15070_c0_g1_i3	Glyma.09G256700.2	98.1981982	159	1	64	3268	3200	1	0	5005

TRINITY_DN15070_c0_g1_i3	Glyma.18G235800.2	99.21875	134	1	64	3268	3200	1	0	5144

TRINITY_DN3595_c0_g1_i1	Glyma.19G159900.1	95.83333333	255	7	66	3261	10	3206	0	4407

TRINITY_DN16426_c2_g1_i3	Glyma.18G044500.1	100	232	6	358	3561	1	3195	0	4573

TRINITY_DN16426_c2_g1_i3	Glyma.18G044500.2	96.25	232	6	358	3561	1	3195	0	4573

TRINITY_DN16426_c2_g1_i1	Glyma.18G044500.1	96.34703196	209	3	278	3481	1	3195	0	4700

TRINITY_DN16426_c2_g1_i1	Glyma.18G044500.2	95.96774194	209	3	278	3481	1	3195	0	4700

TRINITY_DN15815_c0_g2_i6	Glyma.12G219400.1	95.84086799	433	19	472	3649	3585	393	0	3301

TRINITY_DN605_c0_g1_i1	Glyma.20G182800.1	100	165	8	422	3598	3198	1	0	4811

TRINITY_DN605_c0_g1_i1	Glyma.10G207900.1	95.77464789	159	9	422	3598	3201	1	0	4865

TRINITY_DN15264_c0_g1_i9	Glyma.02G008000.1	98	300	7	154	3348	1	3198	0	4191

TRINITY_DN12721_c0_g1_i1	Glyma.09G041400.1	98.38709677	174	0	230	3430	3381	181	0	4948

TRINITY_DN12721_c0_g1_i1	Glyma.15G152100.1	100	174	0	230	3430	3372	172	0	4948

TRINITY_DN12721_c0_g1_i2	Glyma.09G041400.1	96.49484536	173	1	230	3411	3381	181	0	4830

TRINITY_DN12721_c0_g1_i2	Glyma.15G152100.1	97.11538462	172	1	230	3411	3372	172	0	4835

TRINITY_DN13164_c0_g1_i2	Glyma.19G113200.1	97.51243781	610	11	110	3301	1	3192	0	2427

TRINITY_DN15264_c0_g1_i9	Glyma.10G137600.3	100	300	7	154	3348	1	3198	0	4202

TRINITY_DN15264_c0_g1_i9	Glyma.10G137600.2	95.96774194	300	7	154	3348	1	3198	0	4202

TRINITY_DN15264_c0_g1_i9	Glyma.10G137600.1	99.57805907	300	7	154	3348	1	3198	0	4202

TRINITY_DN13689_c0_g1_i2	Glyma.06G139800.2	96.15384615	253	9	258	3454	1	3188	0	4410

TRINITY_DN13689_c0_g1_i2	Glyma.06G139800.3	96.66666667	253	9	258	3454	1	3188	0	4410

TRINITY_DN15815_c0_g2_i6	Glyma.12G219400.4	98.39228296	433	18	472	3649	3582	393	0	3315

TRINITY_DN15897_c0_g2_i2	Glyma.18G108100.1	99.46524064	433	35	164	3345	3152	1	0	3107

TRINITY_DN14062_c0_g1_i3	Glyma.01G146600.1	95.97315436	213	0	402	3599	3291	94	0	4726

TRINITY_DN14752_c0_g1_i1	Glyma.19G113700.4	96.03174603	197	9	530	3706	51	3227	0	4543

TRINITY_DN14752_c0_g1_i1	Glyma.19G113700.1	96.03960396	197	9	530	3706	51	3227	0	4543

TRINITY_DN16286_c0_g1_i2	Glyma.09G137200.1	96.96969697	180	2	1046	4243	3230	36	0	4889

TRINITY_DN16286_c0_g1_i3	Glyma.09G137200.1	100	180	2	1079	4276	3230	36	0	4889

TRINITY_DN15815_c0_g2_i6	Glyma.13G281900.1	96.72131148	426	16	472	3649	3582	393	0	3376

TRINITY_DN15264_c0_g1_i2	Glyma.03G224100.1	99.79757085	254	6	201	3383	7	3201	0	4394

TRINITY_DN16094_c0_g1_i7	Glyma.11G004100.1	97.5	203	8	3	3188	3177	1	0	4601

TRINITY_DN13347_c0_g1_i3	Glyma.09G206300.1	95.72192513	180	5	371	3555	1	3194	0	4843

TRINITY_DN13347_c0_g1_i3	Glyma.01G016500.2	97.70114943	171	5	371	3555	1	3194	0	4892

TRINITY_DN13347_c0_g1_i3	Glyma.01G016500.1	96.36363636	171	5	371	3555	1	3194	0	4892

TRINITY_DN16143_c0_g1_i1	Glyma.07G061500.1	99.05992949	429	40	206	3331	1	3147	0	2789

TRINITY_DN14929_c0_g1_i2	Glyma.18G267000.1	99.43502825	570	48	250	3394	3411	246	0	2265

TRINITY_DN8514_c0_g1_i1	Glyma.07G053100.1	100	199	1	327	3518	3222	32	0	4785

TRINITY_DN5981_c0_g1_i1	Glyma.04G065000.3	95.81395349	336	19	687	3844	1	3174	0	3709

TRINITY_DN5981_c0_g1_i1	Glyma.04G065000.1	95.81589958	336	19	687	3844	1	3174	0	3709

TRINITY_DN5981_c0_g1_i1	Glyma.04G065000.6	95.81005587	336	19	687	3844	1	3174	0	3709

TRINITY_DN13941_c0_g1_i1	Glyma.09G228100.1	99.05437352	256	53	1	3094	465	3618	0	3609

TRINITY_DN13941_c0_g1_i1	Glyma.09G228100.2	99.05660377	256	53	1	3094	465	3618	0	3609

TRINITY_DN15162_c0_g1_i1	Glyma.10G201900.1	97.36842105	230	8	85	3271	1	3178	0	4508

TRINITY_DN15162_c0_g1_i1	Glyma.20G188500.1	100	219	7	85	3271	1	3178	0	4569

TRINITY_DN11596_c0_g2_i1	Glyma.01G004100.1	98.93617021	313	18	322	3476	3195	32	0	3762

TRINITY_DN16233_c0_g2_i3	Glyma.18G058900.1	95.3271028	294	5	237	3422	3339	153	0	4217

TRINITY_DN16233_c0_g2_i2	Glyma.18G058900.1	99.82269504	225	5	407	3592	3339	153	0	4599

TRINITY_DN14929_c0_g1_i2	Glyma.08G246100.1	99.42857143	587	47	250	3394	3411	246	0	2204

TRINITY_DN15811_c0_g1_i2	Glyma.17G023600.1	98.92473118	284	8	48	3230	1	3180	0	4218

TRINITY_DN11286_c0_g1_i1	Glyma.09G024900.1	99.44134078	283	0	589	3777	3189	1	0	4322

TRINITY_DN11286_c0_g1_i1	Glyma.09G024900.2	98.85057471	283	0	589	3777	3189	1	0	4322

TRINITY_DN11286_c0_g1_i1	Glyma.09G024900.3	95.23809524	283	0	589	3777	3189	1	0	4322

TRINITY_DN11286_c0_g1_i2	Glyma.09G024900.1	100	283	0	589	3777	3189	1	0	4322

TRINITY_DN11286_c0_g1_i2	Glyma.09G024900.2	97.84172662	283	0	589	3777	3189	1	0	4322

TRINITY_DN11286_c0_g1_i2	Glyma.09G024900.3	97.84172662	283	0	589	3777	3189	1	0	4322

TRINITY_DN16345_c0_g1_i2	Glyma.08G044700.5	95.3271028	415	14	446	3625	3657	484	0	3446

TRINITY_DN16345_c0_g1_i5	Glyma.08G044700.5	98.4375	415	14	446	3625	3657	484	0	3446

TRINITY_DN11286_c0_g1_i1	Glyma.15G130900.1	99.44134078	274	0	590	3777	3188	1	0	4370

TRINITY_DN11286_c0_g1_i2	Glyma.15G130900.1	98.46153846	274	0	590	3777	3188	1	0	4370

TRINITY_DN14332_c0_g1_i1	Glyma.U025000.2	99.68051118	507	37	218	3384	1	3152	0	2721

TRINITY_DN14332_c0_g1_i1	Glyma.U025000.1	96.92307692	507	37	218	3384	1	3152	0	2721

TRINITY_DN16425_c0_g1_i2	Glyma.15G035900.2	100	415	21	494	3652	3165	1	0	3264

TRINITY_DN16425_c0_g1_i5	Glyma.15G035900.2	100	415	21	494	3652	3165	1	0	3264

TRINITY_DN8774_c0_g1_i1	Glyma.10G165000.1	100	193	8	1622	4793	206	3390	0	4706

TRINITY_DN8774_c0_g1_i2	Glyma.10G165000.1	97.40932642	193	8	1622	4793	206	3390	0	4706

TRINITY_DN8774_c0_g1_i3	Glyma.10G165000.1	96.24413146	193	8	1622	4793	206	3390	0	4706

TRINITY_DN15811_c0_g1_i2	Glyma.07G250700.1	98.92473118	273	8	48	3230	1	3177	0	4294

TRINITY_DN15584_c0_g1_i1	Glyma.19G175900.1	95.71428571	233	12	477	3647	1	3183	0	4477

TRINITY_DN13716_c0_g2_i1	Glyma.01G091000.1	99.76635514	425	46	550	3673	3169	1	0	3029

TRINITY_DN16245_c0_g3_i4	Glyma.16G099700.2	100	448	25	672	3841	3200	31	0	3217

TRINITY_DN13164_c0_g1_i4	Glyma.08G114900.1	96.5625	209	2	171	3353	1	3183	0	4710

TRINITY_DN16487_c0_g1_i1	Glyma.08G208600.1	99.28571429	271	10	433	3591	1	3168	0	4124

TRINITY_DN15584_c0_g1_i1	Glyma.03G175200.1	98.37662338	260	6	477	3647	1	3183	0	4361

TRINITY_DN15531_c0_g1_i2	Glyma.06G017700.2	100	184	6	754	3933	1	3180	0	4820

TRINITY_DN16345_c0_g1_i6	Glyma.08G044700.5	98.4375	310	6	446	3622	3657	484	0	4074

TRINITY_DN16422_c0_g2_i4	Glyma.11G005500.1	98.66666667	288	22	1190	4351	780	3933	0	3971

TRINITY_DN16422_c0_g2_i5	Glyma.11G005500.1	97.6744186	288	22	1189	4350	780	3933	0	3971

TRINITY_DN16245_c0_g5_i2	Glyma.01G092900.2	97.8978979	734	74	422	3559	3138	1	0	1253

TRINITY_DN16245_c0_g5_i2	Glyma.01G092900.3	100	734	74	422	3559	3138	1	0	1253

TRINITY_DN13716_c0_g2_i1	Glyma.02G130700.3	99.08256881	442	47	550	3673	3163	1	0	2940

TRINITY_DN14576_c0_g1_i1	Glyma.09G229900.2	96.25	307	13	80	3250	1	3174	0	4063

TRINITY_DN14576_c0_g1_i1	Glyma.09G229900.1	98.11676083	307	13	80	3250	1	3174	0	4063

TRINITY_DN16245_c0_g5_i1	Glyma.01G092900.2	95.80291971	736	73	422	3559	3138	1	0	1253

TRINITY_DN16245_c0_g5_i1	Glyma.01G092900.3	95.8041958	736	73	422	3559	3138	1	0	1253

TRINITY_DN16422_c0_g2_i6	Glyma.11G005500.1	95.29411765	298	29	1189	4351	780	3933	0	3943

TRINITY_DN16422_c0_g2_i7	Glyma.11G005500.1	95.85253456	298	29	1190	4352	780	3933	0	3943

TRINITY_DN14332_c0_g1_i1	Glyma.13G275100.1	98.30508475	296	10	218	3380	1	3175	0	4091

TRINITY_DN15457_c0_g1_i1	Glyma.13G161600.1	99.33774834	224	2	126	3302	1	3180	0	4612

TRINITY_DN15457_c0_g1_i1	Glyma.17G109700.1	100	210	2	126	3302	1	3180	0	4689

TRINITY_DN16422_c0_g2_i1	Glyma.11G005500.1	100	297	28	1190	4351	780	3933	0	3954

TRINITY_DN16422_c0_g2_i2	Glyma.11G005500.1	98.2278481	297	28	1189	4350	780	3933	0	3954

TRINITY_DN15270_c0_g1_i2	Glyma.14G033600.3	97.56097561	187	1	29	3207	688	3863	0	4815

TRINITY_DN15270_c0_g1_i2	Glyma.14G033600.1	98.27586207	187	1	29	3207	688	3863	0	4815

TRINITY_DN15270_c0_g1_i2	Glyma.14G033600.2	98.52941176	187	1	29	3207	688	3863	0	4815

TRINITY_DN15270_c0_g1_i2	Glyma.02G281000.2	100	180	1	29	3207	694	3869	0	4854

TRINITY_DN15270_c0_g1_i2	Glyma.02G281000.1	99.37888199	180	1	29	3207	694	3869	0	4854

TRINITY_DN14332_c0_g1_i1	Glyma.06G238700.1	96.92307692	526	24	218	3384	1	3149	0	2697

TRINITY_DN14332_c0_g1_i1	Glyma.12G225800.1	96.77419355	293	11	218	3380	1	3175	0	4130

TRINITY_DN12563_c0_g1_i2	Glyma.01G002100.2	96.39830508	291	18	268	3412	4	3179	0	4030

TRINITY_DN12563_c0_g1_i3	Glyma.01G002100.2	97	261	11	268	3424	4	3179	0	4274

TRINITY_DN11006_c0_g1_i1	Glyma.03G259000.1	97.43589744	249	8	213	3388	3170	1	0	4425

TRINITY_DN16349_c0_g1_i2	Glyma.02G155100.1	97.4025974	358	43	94	3213	1	3150	0	3341

TRINITY_DN16422_c0_g2_i3	Glyma.01G238200.1	100	289	26	343	3513	1	3120	0	3858

TRINITY_DN16487_c0_g1_i1	Glyma.07G033400.1	98.66666667	256	16	433	3591	1	3162	0	4235

TRINITY_DN318_c0_g1_i1	Glyma.20G229100.4	98.0952381	189	9	1	3172	1770	4911	0	4573

TRINITY_DN318_c0_g1_i1	Glyma.20G229100.5	97.36842105	189	9	1	3172	1770	4911	0	4573

TRINITY_DN318_c0_g1_i1	Glyma.20G229100.2	95.10309278	189	9	1	3172	1767	4908	0	4573

TRINITY_DN318_c0_g1_i1	Glyma.20G229100.3	100	189	9	1	3172	1767	4908	0	4573

TRINITY_DN318_c0_g1_i1	Glyma.20G229100.9	95.10204082	189	9	1	3172	1380	4521	0	4573

TRINITY_DN318_c0_g1_i1	Glyma.20G229100.10	95.10309278	189	9	1	3172	1380	4521	0	4573

TRINITY_DN318_c0_g1_i1	Glyma.20G229100.11	100	189	9	1	3172	765	3906	0	4573

TRINITY_DN318_c0_g1_i1	Glyma.20G229100.12	100	189	9	1	3172	765	3906	0	4573

TRINITY_DN318_c0_g1_i1	Glyma.10G159500.1	100	187	11	1	3172	1770	4923	0	4662

TRINITY_DN318_c0_g1_i1	Glyma.10G159500.2	95.4954955	187	11	1	3172	1767	4920	0	4662

TRINITY_DN16254_c0_g1_i2	Glyma.15G001300.1	99.31034483	767	62	397	3535	3231	96	0	1140

TRINITY_DN14844_c0_g1_i2	Glyma.18G146600.2	100	225	6	484	3639	3174	1	0	4499

TRINITY_DN4758_c0_g1_i1	Glyma.11G085900.1	100	479	21	358	3518	3152	1	0	2992

TRINITY_DN5981_c0_g1_i1	Glyma.04G065000.8	96.55172414	336	18	687	3844	1	3156	0	3792

TRINITY_DN5981_c0_g1_i1	Glyma.04G065000.4	95.83333333	336	18	687	3844	1	3156	0	3792

TRINITY_DN5981_c0_g1_i1	Glyma.04G065000.5	95.83333333	336	18	687	3844	1	3156	0	3792

TRINITY_DN5981_c0_g1_i1	Glyma.04G065000.7	96.55172414	336	18	687	3844	1	3156	0	3792

TRINITY_DN5981_c0_g1_i1	Glyma.04G065000.2	95.83333333	336	18	687	3844	1	3156	0	3792

TRINITY_DN15867_c0_g3_i3	Glyma.17G052600.1	98.41269841	286	8	189	3345	1	3169	0	4146

TRINITY_DN8514_c0_g1_i1	Glyma.16G022200.2	95.65217391	202	0	327	3499	3174	2	0	4741

TRINITY_DN8514_c0_g1_i1	Glyma.16G022200.1	100	202	0	327	3499	3174	2	0	4741

TRINITY_DN8514_c0_g1_i1	Glyma.07G053100.2	95.65217391	196	0	327	3499	3174	2	0	4774

TRINITY_DN5981_c0_g1_i1	Glyma.06G066200.4	95.8041958	362	13	687	3844	1	3160	0	3685

TRINITY_DN5981_c0_g1_i1	Glyma.06G066200.1	95.83333333	362	13	687	3844	1	3160	0	3685

TRINITY_DN5981_c0_g1_i1	Glyma.06G066200.3	95.82909461	362	13	687	3844	1	3160	0	3685

TRINITY_DN5981_c0_g1_i1	Glyma.06G066200.2	95.83333333	362	13	687	3844	1	3160	0	3685

TRINITY_DN15867_c0_g3_i3	Glyma.13G106700.16	96.39175258	291	6	189	3345	1	3169	0	4130

TRINITY_DN15867_c0_g3_i3	Glyma.13G106700.11	98.40546697	291	6	189	3345	1	3169	0	4130

TRINITY_DN15867_c0_g3_i3	Glyma.13G106700.12	95.40229885	291	6	189	3345	1	3169	0	4130

TRINITY_DN15867_c0_g3_i3	Glyma.13G106700.13	96.92307692	291	6	189	3345	1	3169	0	4130

TRINITY_DN15867_c0_g3_i3	Glyma.13G106700.14	97.14285714	291	6	189	3345	1	3169	0	4130

TRINITY_DN15867_c0_g3_i3	Glyma.13G106700.7	98.66666667	291	6	189	3345	1	3169	0	4130

TRINITY_DN15867_c0_g3_i3	Glyma.13G106700.1	96.19410086	291	6	189	3345	1	3169	0	4130

TRINITY_DN15867_c0_g3_i3	Glyma.13G106700.2	96.19450317	291	6	189	3345	1	3169	0	4130

TRINITY_DN15867_c0_g3_i3	Glyma.13G106700.3	95.18072289	291	6	189	3345	1	3169	0	4130

TRINITY_DN15867_c0_g3_i3	Glyma.13G106700.4	100	291	6	189	3345	1	3169	0	4130

TRINITY_DN15867_c0_g3_i3	Glyma.13G106700.17	100	291	6	189	3345	1	3169	0	4130

TRINITY_DN13164_c0_g1_i2	Glyma.16G039900.1	97.5	590	19	140	3301	22	3180	0	2451

TRINITY_DN13164_c0_g1_i2	Glyma.16G039900.2	97.5	590	19	140	3301	22	3180	0	2451

TRINITY_DN10930_c0_g1_i2	Glyma.10G291300.1	99.18032787	208	0	753	3923	3171	1	0	4704

TRINITY_DN10930_c0_g1_i2	Glyma.20G240500.1	98.52941176	199	0	753	3923	3171	1	0	4754

TRINITY_DN16141_c0_g1_i2	Glyma.06G003000.8	100	137	3	691	3843	3171	1	0	4981

TRINITY_DN16141_c0_g1_i2	Glyma.06G003000.1	98.5915493	137	3	691	3843	3171	1	0	4981

TRINITY_DN16141_c0_g1_i5	Glyma.06G003000.8	97.2972973	137	4	691	3841	3171	1	0	4968

TRINITY_DN16141_c0_g1_i5	Glyma.06G003000.1	100	137	4	691	3841	3171	1	0	4968

TRINITY_DN4758_c0_g1_i1	Glyma.11G085900.2	95.77464789	479	19	358	3518	3149	1	0	3007

TRINITY_DN12896_c0_g2_i1	Glyma.18G072400.2	95.84158416	247	5	417	3581	1	3168	0	4440

TRINITY_DN318_c0_g1_i1	Glyma.20G229100.6	99.27007299	188	10	1	3165	1767	4902	0	4560

TRINITY_DN318_c0_g1_i1	Glyma.20G229100.1	99.27007299	188	10	1	3165	1767	4902	0	4560

TRINITY_DN318_c0_g1_i1	Glyma.20G229100.7	97.36842105	188	10	1	3165	1467	4602	0	4560

TRINITY_DN318_c0_g1_i1	Glyma.20G229100.8	99.09090909	188	10	1	3165	1467	4602	0	4560

TRINITY_DN11006_c0_g1_i1	Glyma.19G257700.1	95.8974359	236	13	213	3380	3149	3	0	4396

TRINITY_DN16135_c0_g1_i1	Glyma.01G051400.1	98.96907216	248	30	1468	4603	3115	1	0	3927

TRINITY_DN16141_c0_g1_i2	Glyma.06G003000.7	97.61904762	137	2	691	3843	3168	1	0	4994

TRINITY_DN16141_c0_g1_i2	Glyma.06G003000.4	97.70992366	137	2	691	3843	3168	1	0	4994

TRINITY_DN16141_c0_g1_i5	Glyma.06G003000.7	97.42647059	137	3	691	3841	3168	1	0	4981

TRINITY_DN16141_c0_g1_i5	Glyma.06G003000.4	97.42647059	137	3	691	3841	3168	1	0	4981

TRINITY_DN16254_c0_g1_i3	Glyma.15G001300.1	96.07843137	777	52	397	3535	3231	96	0	1162

TRINITY_DN16425_c0_g1_i2	Glyma.U042000.1	95.6284153	250	15	494	3652	3150	1	0	4301

TRINITY_DN16425_c0_g1_i5	Glyma.U042000.1	100	250	15	494	3652	3150	1	0	4301

TRINITY_DN16425_c0_g1_i2	Glyma.11G136700.1	100	252	18	494	3652	3144	1	0	4263

TRINITY_DN16425_c0_g1_i5	Glyma.11G136700.1	95.92760181	252	18	494	3652	3144	1	0	4263

TRINITY_DN16425_c0_g1_i2	Glyma.12G060600.1	100	251	7	494	3652	3162	1	0	4385

TRINITY_DN16425_c0_g1_i2	Glyma.12G060600.2	100	251	7	494	3652	3162	1	0	4385

TRINITY_DN16425_c0_g1_i2	Glyma.12G060600.3	100	251	7	494	3652	3162	1	0	4385

TRINITY_DN16425_c0_g1_i5	Glyma.12G060600.1	95.08928571	251	7	494	3652	3162	1	0	4385

TRINITY_DN16425_c0_g1_i5	Glyma.12G060600.2	95.08928571	251	7	494	3652	3162	1	0	4385

TRINITY_DN16425_c0_g1_i5	Glyma.12G060600.3	95.08928571	251	7	494	3652	3162	1	0	4385

TRINITY_DN9513_c0_g2_i1	Glyma.06G127600.1	98.60627178	245	13	1	3139	336	3489	0	4237

TRINITY_DN16425_c0_g1_i2	Glyma.11G137100.3	100	244	19	494	3652	3144	1	0	4307

TRINITY_DN16425_c0_g1_i2	Glyma.11G137100.1	100	244	19	494	3652	3144	1	0	4307

TRINITY_DN16425_c0_g1_i2	Glyma.11G137100.2	99.00990099	244	19	494	3652	3144	1	0	4307

TRINITY_DN16425_c0_g1_i5	Glyma.11G137100.3	100	244	19	494	3652	3144	1	0	4307

TRINITY_DN16425_c0_g1_i5	Glyma.11G137100.1	95.87628866	244	19	494	3652	3144	1	0	4307

TRINITY_DN16425_c0_g1_i5	Glyma.11G137100.2	98.4375	244	19	494	3652	3144	1	0	4307

TRINITY_DN12679_c0_g1_i1	Glyma.14G217400.1	95.29411765	225	10	578	3737	451	3610	0	4523

TRINITY_DN12679_c0_g1_i1	Glyma.14G217400.2	95.28301887	225	10	578	3737	451	3610	0	4523

TRINITY_DN12679_c0_g1_i1	Glyma.14G217400.3	95.28301887	225	10	578	3737	451	3610	0	4523

TRINITY_DN12679_c0_g1_i2	Glyma.14G217400.1	95.30201342	225	10	578	3737	451	3610	0	4523

TRINITY_DN12679_c0_g1_i2	Glyma.14G217400.2	95.3125	225	10	578	3737	451	3610	0	4523

TRINITY_DN12679_c0_g1_i2	Glyma.14G217400.3	95.29652352	225	10	578	3737	451	3610	0	4523

TRINITY_DN16153_c0_g1_i1	Glyma.17G217300.1	95.53571429	418	21	1214	4365	3235	90	0	3323

TRINITY_DN16141_c0_g1_i2	Glyma.04G003000.1	95.45454545	133	2	691	3843	3165	1	0	5031

TRINITY_DN16141_c0_g1_i5	Glyma.04G003000.1	95.6043956	133	3	691	3841	3165	1	0	5018

TRINITY_DN16141_c0_g1_i2	Glyma.04G003000.11	100	133	4	691	3843	3156	1	0	4972

TRINITY_DN16141_c0_g1_i5	Glyma.04G003000.11	98.48484848	133	5	691	3841	3156	1	0	4959

TRINITY_DN9338_c0_g1_i1	Glyma.15G019200.6	96.42857143	496	21	896	4050	3154	9	0	2920

TRINITY_DN4758_c0_g1_i1	Glyma.09G198200.1	99.06542056	260	1	355	3518	3161	1	0	4383

TRINITY_DN16245_c0_g5_i2	Glyma.05G015400.2	98.39228296	252	7	347	3508	3159	1	0	4401

TRINITY_DN5846_c0_g1_i1	Glyma.12G024600.2	100	259	7	1	3155	2436	5596	0	4342

TRINITY_DN5846_c0_g1_i1	Glyma.12G024600.4	100	259	7	1	3155	2436	5596	0	4342

TRINITY_DN5846_c0_g1_i1	Glyma.12G024600.3	99.04761905	259	7	1	3155	2436	5596	0	4342

TRINITY_DN16422_c0_g2_i3	Glyma.01G238200.2	100	287	26	343	3498	1	3105	0	3842

TRINITY_DN16245_c0_g5_i1	Glyma.05G015400.2	96.2406015	217	3	347	3508	3159	1	0	4617

TRINITY_DN16141_c0_g1_i2	Glyma.06G003000.6	98.66666667	137	2	691	3843	3162	1	0	5022

TRINITY_DN16141_c0_g1_i2	Glyma.06G003000.3	98.66666667	137	2	691	3843	3162	1	0	5022

TRINITY_DN16141_c0_g1_i5	Glyma.06G003000.6	97.4248927	137	3	691	3841	3162	1	0	5009

TRINITY_DN16141_c0_g1_i5	Glyma.06G003000.3	97.42574257	137	3	691	3841	3162	1	0	5009

TRINITY_DN16141_c0_g1_i2	Glyma.04G003000.12	100	133	1	691	3843	3162	1	0	5044

TRINITY_DN16141_c0_g1_i5	Glyma.04G003000.12	97.26027397	133	2	691	3841	3162	1	0	5031

TRINITY_DN16141_c0_g1_i2	Glyma.04G003000.10	97.4137931	133	3	691	3843	3153	1	0	4987

TRINITY_DN16141_c0_g1_i5	Glyma.04G003000.10	99.52830189	133	4	691	3841	3153	1	0	4974

TRINITY_DN15293_c0_g1_i1	Glyma.12G187700.1	99.57805907	528	61	530	3628	1	3129	0	2305

TRINITY_DN15293_c0_g1_i4	Glyma.12G187700.1	96.96969697	508	61	467	3565	1	3129	0	2416

TRINITY_DN15897_c0_g2_i2	Glyma.08G308600.1	95.18072289	415	38	209	3345	3113	1	0	3073

TRINITY_DN12563_c0_g1_i3	Glyma.07G130400.2	99.47961839	271	6	269	3425	50	3198	0	4233

TRINITY_DN15293_c0_g1_i1	Glyma.13G313500.1	99.57983193	529	58	530	3628	1	3117	0	2233

TRINITY_DN15293_c0_g1_i4	Glyma.13G313500.1	100	506	58	467	3565	1	3117	0	2361

TRINITY_DN16262_c0_g1_i5	Glyma.04G213400.4	99.09090909	410	29	439	3591	1	3114	0	3232

TRINITY_DN16262_c0_g1_i5	Glyma.04G213400.3	98.97435897	408	31	439	3591	1	3111	0	3225

TRINITY_DN16115_c0_g2_i1	Glyma.15G043400.2	99.58333333	337	6	230	3385	3156	1	0	3928

TRINITY_DN16115_c0_g2_i1	Glyma.15G043400.1	99.58333333	337	6	230	3385	3156	1	0	3928

TRINITY_DN16115_c0_g2_i2	Glyma.15G043400.2	98.92473118	300	6	230	3385	3156	1	0	4133

TRINITY_DN16115_c0_g2_i2	Glyma.15G043400.1	97.34513274	300	6	230	3385	3156	1	0	4133

TRINITY_DN16422_c0_g2_i4	Glyma.01G238300.1	95.45454545	289	18	1207	4351	177	3303	0	3936

TRINITY_DN16422_c0_g2_i5	Glyma.01G238300.1	97.6744186	289	18	1206	4350	177	3303	0	3936

TRINITY_DN16398_c1_g1_i1	Glyma.08G256300.2	100	221	6	666	3806	13	3171	0	4494

TRINITY_DN16141_c0_g1_i2	Glyma.06G003000.5	100	137	1	691	3843	3159	1	0	5036

TRINITY_DN16141_c0_g1_i2	Glyma.06G003000.2	97.5	137	1	691	3843	3159	1	0	5036

TRINITY_DN16141_c0_g1_i5	Glyma.06G003000.5	97.26027397	137	2	691	3841	3159	1	0	5024

TRINITY_DN16141_c0_g1_i5	Glyma.06G003000.2	97.26027397	137	2	691	3841	3159	1	0	5024

TRINITY_DN16262_c0_g1_i5	Glyma.06G152800.2	96.04863222	405	31	439	3591	1	3102	0	3193

TRINITY_DN16422_c0_g2_i6	Glyma.01G238300.1	96.98795181	317	26	1206	4351	177	3303	0	3808

TRINITY_DN16422_c0_g2_i7	Glyma.01G238300.1	98.97959184	317	26	1207	4352	177	3303	0	3808

TRINITY_DN15515_c0_g2_i2	Glyma.02G170900.1	98.41269841	295	7	1	3145	252	3405	0	4098

TRINITY_DN15515_c0_g2_i2	Glyma.09G109000.1	96	279	8	1	3145	621	3771	0	4169

TRINITY_DN15515_c0_g2_i2	Glyma.09G109000.2	96.47058824	279	8	1	3145	489	3639	0	4169

TRINITY_DN10448_c0_g1_i1	Glyma.02G232900.2	98	260	6	1	3154	4308	1158	0	4331

TRINITY_DN10448_c0_g1_i1	Glyma.02G232900.1	98	260	6	1	3154	4308	1158	0	4331

TRINITY_DN9513_c0_g2_i1	Glyma.04G236700.1	95.91078067	244	5	1	3139	336	3486	0	4324

TRINITY_DN13689_c0_g1_i1	Glyma.06G139800.6	98.06094183	244	21	258	3384	1	3145	0	4207

TRINITY_DN13689_c0_g1_i1	Glyma.06G139800.8	100	244	21	258	3384	1	3145	0	4207

TRINITY_DN13689_c0_g1_i1	Glyma.06G139800.12	95.52238806	244	21	258	3384	1	3145	0	4207

TRINITY_DN13689_c0_g1_i1	Glyma.06G139800.13	100	244	21	258	3384	1	3145	0	4207

TRINITY_DN9255_c0_g1_i1	Glyma.01G176400.1	100	237	6	315	3468	3154	1	0	4479

TRINITY_DN9255_c0_g1_i1	Glyma.01G176400.2	97.2027972	237	7	315	3468	3151	1	0	4458

TRINITY_DN12679_c0_g1_i1	Glyma.17G256200.2	95.30516432	221	7	581	3737	448	3592	0	4529

TRINITY_DN12679_c0_g1_i1	Glyma.17G256200.1	95.30201342	221	7	581	3737	448	3592	0	4529

TRINITY_DN12679_c0_g1_i1	Glyma.17G256200.4	95.29411765	221	7	581	3737	448	3592	0	4529

TRINITY_DN12679_c0_g1_i1	Glyma.17G256200.3	95.2991453	221	7	581	3737	448	3592	0	4529

TRINITY_DN12679_c0_g1_i2	Glyma.17G256200.2	95.3125	221	7	581	3737	448	3592	0	4529

TRINITY_DN12679_c0_g1_i2	Glyma.17G256200.1	95.29411765	221	7	581	3737	448	3592	0	4529

TRINITY_DN12679_c0_g1_i2	Glyma.17G256200.4	95.3038674	221	7	581	3737	448	3592	0	4529

TRINITY_DN12679_c0_g1_i2	Glyma.17G256200.3	95.30201342	221	7	581	3737	448	3592	0	4529

TRINITY_DN16422_c0_g2_i1	Glyma.01G238300.1	100	317	25	1207	4351	177	3303	0	3814

TRINITY_DN16422_c0_g2_i2	Glyma.01G238300.1	100	317	25	1206	4350	177	3303	0	3814

TRINITY_DN16254_c0_g1_i3	Glyma.15G167500.1	100	280	9	328	3481	3249	105	0	4193

TRINITY_DN16254_c0_g1_i3	Glyma.15G167500.3	100	280	9	328	3481	3249	105	0	4193

TRINITY_DN16254_c0_g1_i3	Glyma.15G167500.2	100	280	9	328	3481	3249	105	0	4193

TRINITY_DN16254_c0_g1_i3	Glyma.15G167500.4	95.72192513	280	9	328	3481	3249	105	0	4193

TRINITY_DN4758_c0_g1_i1	Glyma.16G116200.1	99.34210526	251	4	364	3518	3152	1	0	4405

TRINITY_DN16254_c0_g1_i2	Glyma.15G167500.1	95.51820728	251	7	328	3481	3249	105	0	4353

TRINITY_DN16254_c0_g1_i2	Glyma.15G167500.3	95.52238806	251	7	328	3481	3249	105	0	4353

TRINITY_DN16254_c0_g1_i2	Glyma.15G167500.2	97.82608696	251	7	328	3481	3249	105	0	4353

TRINITY_DN16254_c0_g1_i2	Glyma.15G167500.4	97.75280899	251	7	328	3481	3249	105	0	4353

TRINITY_DN16115_c0_g2_i4	Glyma.15G043400.2	95.21575985	191	0	332	3487	3156	1	0	4771

TRINITY_DN16115_c0_g2_i4	Glyma.15G043400.1	100	191	0	332	3487	3156	1	0	4771

TRINITY_DN16115_c0_g2_i6	Glyma.15G043400.2	100	154	0	332	3487	3156	1	0	4975

TRINITY_DN16115_c0_g2_i6	Glyma.15G043400.1	100	154	0	332	3487	3156	1	0	4975

TRINITY_DN16141_c0_g1_i2	Glyma.04G003000.3	100	133	1	691	3843	3156	1	0	5072

TRINITY_DN16141_c0_g1_i5	Glyma.04G003000.3	97.26027397	133	2	691	3841	3156	1	0	5059

TRINITY_DN16141_c0_g1_i2	Glyma.04G003000.9	95.83333333	133	3	691	3843	3147	1	0	5014

TRINITY_DN16141_c0_g1_i5	Glyma.04G003000.9	97.42574257	133	4	691	3841	3147	1	0	5001

TRINITY_DN16262_c0_g1_i8	Glyma.06G152800.2	100	326	30	439	3588	1	3102	0	3646

TRINITY_DN12563_c0_g1_i2	Glyma.07G130400.2	100	304	8	269	3413	50	3198	0	4039

TRINITY_DN16262_c0_g1_i8	Glyma.04G213400.4	95.83333333	337	24	439	3588	1	3114	0	3674

TRINITY_DN16262_c0_g1_i8	Glyma.04G213400.3	100	335	26	439	3588	1	3111	0	3664

TRINITY_DN13689_c0_g1_i1	Glyma.06G139800.1	98.94736842	244	21	258	3381	1	3142	0	4202

TRINITY_DN16115_c0_g2_i2	Glyma.U033900.1	97.2972973	241	3	234	3385	3146	1	0	4436

TRINITY_DN16115_c0_g2_i1	Glyma.U033900.1	98.76237624	204	3	234	3385	3146	1	0	4641

TRINITY_DN16141_c0_g1_i2	Glyma.04G003000.2	100	133	0	691	3843	3153	1	0	5086

TRINITY_DN16141_c0_g1_i5	Glyma.04G003000.2	100	133	1	691	3841	3153	1	0	5073

TRINITY_DN16141_c0_g1_i2	Glyma.04G003000.8	100	133	2	691	3843	3144	1	0	5027

TRINITY_DN16141_c0_g1_i5	Glyma.04G003000.8	97.4248927	133	3	691	3841	3144	1	0	5014

TRINITY_DN15517_c0_g2_i2	Glyma.02G289700.1	95.92760181	370	13	403	3543	3234	91	0	3648

TRINITY_DN15242_c0_g1_i1	Glyma.20G086700.4	95.67567568	332	23	221	3348	3156	29	0	3683

TRINITY_DN14326_c0_g5_i1	Glyma.06G008300.1	96.21212121	239	12	1223	4345	1291	4431	0	4250

TRINITY_DN16368_c0_g2_i2	Glyma.10G028800.2	100	262	2	1	3149	952	4100	0	4353

TRINITY_DN16368_c0_g2_i2	Glyma.10G028800.1	100	262	2	1	3149	952	4100	0	4353

TRINITY_DN14877_c0_g1_i1	Glyma.13G067800.1	95.16129032	200	2	312	3455	4119	971	0	4663

TRINITY_DN14877_c0_g1_i1	Glyma.13G067800.2	100	200	2	312	3455	4116	968	0	4663

TRINITY_DN16115_c0_g2_i4	Glyma.13G330600.2	95	199	2	339	3487	3149	1	0	4702

TRINITY_DN16115_c0_g2_i4	Glyma.13G330600.1	100	199	2	339	3487	3149	1	0	4702

TRINITY_DN16115_c0_g2_i6	Glyma.13G330600.2	100	160	2	339	3487	3149	1	0	4918

TRINITY_DN16115_c0_g2_i6	Glyma.13G330600.1	99.24242424	160	2	339	3487	3149	1	0	4918

TRINITY_DN16425_c0_g1_i2	Glyma.15G035900.1	98.48484848	413	21	532	3652	3127	1	0	3205

TRINITY_DN16425_c0_g1_i5	Glyma.15G035900.1	100	413	21	532	3652	3127	1	0	3205

TRINITY_DN9255_c0_g1_i1	Glyma.11G068600.1	100	215	9	315	3460	3139	9	0	4488

TRINITY_DN9255_c0_g1_i1	Glyma.11G068600.2	96	215	10	315	3460	3136	9	0	4470

TRINITY_DN16115_c0_g2_i6	Glyma.U033900.1	95.23809524	370	5	342	3487	3140	1	0	3699

TRINITY_DN16115_c0_g2_i4	Glyma.U033900.1	98.30508475	333	5	342	3487	3140	1	0	3904

TRINITY_DN15639_c1_g1_i1	Glyma.12G073200.3	100	231	13	551	3680	3471	330	0	4379

TRINITY_DN15639_c1_g1_i1	Glyma.12G073200.1	95.23809524	231	13	551	3680	3471	330	0	4379

TRINITY_DN15639_c1_g1_i2	Glyma.12G073200.3	97.84946237	231	13	675	3804	3471	330	0	4379

TRINITY_DN15639_c1_g1_i2	Glyma.12G073200.1	100	231	13	675	3804	3471	330	0	4379

TRINITY_DN15639_c1_g1_i3	Glyma.12G073200.3	100	231	13	358	3487	3471	330	0	4379

TRINITY_DN15639_c1_g1_i3	Glyma.12G073200.1	100	231	13	358	3487	3471	330	0	4379

TRINITY_DN14994_c0_g1_i3	Glyma.13G115500.1	97.91666667	197	2	189	3335	1	3147	0	4710

TRINITY_DN11286_c0_g1_i1	Glyma.08G123800.1	99.35064935	529	16	589	3722	3198	62	0	2732

TRINITY_DN11286_c0_g1_i2	Glyma.08G123800.1	97.84172662	529	16	589	3722	3198	62	0	2732

TRINITY_DN11898_c0_g2_i3	Glyma.04G091700.1	98.96907216	337	28	385	3503	3134	1	0	3679

TRINITY_DN14994_c0_g1_i3	Glyma.17G044300.1	97.94520548	201	0	189	3335	1	3147	0	4698

TRINITY_DN16115_c0_g2_i1	Glyma.13G330600.2	100	345	9	246	3385	3140	1	0	3832

TRINITY_DN16115_c0_g2_i1	Glyma.13G330600.1	99.12663755	345	9	246	3385	3140	1	0	3832

TRINITY_DN16115_c0_g2_i2	Glyma.13G330600.2	97.82608696	306	9	246	3385	3140	1	0	4048

TRINITY_DN16115_c0_g2_i2	Glyma.13G330600.1	97.82608696	306	9	246	3385	3140	1	0	4048

TRINITY_DN15384_c0_g1_i1	Glyma.06G122500.1	100	220	10	140	3280	1	3120	0	4401

TRINITY_DN15242_c0_g1_i2	Glyma.20G086700.4	95.39641944	197	11	221	3354	3156	29	0	4536

TRINITY_DN2483_c0_g1_i1	Glyma.17G076400.1	98.7804878	193	2	1	3144	1957	5100	0	4726

TRINITY_DN16254_c0_g1_i2	Glyma.13G372400.1	96.15384615	764	53	410	3523	3233	120	0	1186

TRINITY_DN13900_c0_g1_i1	Glyma.03G262100.1	99.25373134	157	0	1	3144	775	3918	0	4937

TRINITY_DN13900_c0_g1_i1	Glyma.03G262100.5	95.50561798	157	0	1	3144	775	3918	0	4937

TRINITY_DN13900_c0_g1_i1	Glyma.03G262100.6	95.65217391	157	0	1	3144	775	3918	0	4937

TRINITY_DN13900_c0_g1_i1	Glyma.03G262100.4	95.55555556	157	0	1	3144	49	3192	0	4937

TRINITY_DN13900_c0_g1_i1	Glyma.19G261200.1	100	137	0	1	3144	769	3912	0	5048

TRINITY_DN13900_c0_g1_i1	Glyma.19G261200.4	100	137	0	1	3144	769	3912	0	5048

TRINITY_DN13900_c0_g1_i1	Glyma.19G261200.2	96.77419355	137	0	1	3144	769	3912	0	5048

TRINITY_DN13900_c0_g1_i1	Glyma.19G261200.3	96.21451104	137	0	1	3144	769	3912	0	5048

TRINITY_DN13164_c0_g1_i2	Glyma.08G114900.1	97.51552795	286	35	185	3304	70	3183	0	3895

TRINITY_DN15293_c0_g1_i2	Glyma.12G187700.1	100	276	12	530	3646	1	3135	0	4067

TRINITY_DN15293_c0_g1_i3	Glyma.12G187700.1	98.1595092	256	12	467	3583	1	3135	0	4178

TRINITY_DN16262_c0_g1_i2	Glyma.06G152800.2	95.72192513	228	19	537	3674	1	3102	0	4255

TRINITY_DN16349_c0_g1_i2	Glyma.10G019400.1	97.38903394	297	35	94	3213	1	3099	0	3747

TRINITY_DN15293_c0_g1_i2	Glyma.13G313500.1	95.87155963	287	9	530	3646	1	3123	0	3962

TRINITY_DN15293_c0_g1_i3	Glyma.13G313500.1	97.16312057	264	9	467	3583	1	3123	0	4089

TRINITY_DN16374_c1_g1_i5	Glyma.07G069400.1	100	263	22	1	3122	3114	6220	0	4023

TRINITY_DN16262_c0_g1_i2	Glyma.04G213400.4	98.33333333	238	14	537	3674	1	3114	0	4311

TRINITY_DN16262_c0_g1_i2	Glyma.04G213400.3	95.72107765	236	16	537	3674	1	3111	0	4301

TRINITY_DN16254_c0_g1_i3	Glyma.13G372400.1	100	780	44	410	3523	3233	120	0	1164

TRINITY_DN14585_c0_g1_i1	Glyma.07G058200.1	96.11650485	292	20	71	3193	1	3108	0	3886

TRINITY_DN14585_c0_g1_i1	Glyma.07G058200.2	95.40229885	292	20	71	3193	1	3108	0	3886

TRINITY_DN14585_c0_g1_i1	Glyma.07G058200.3	100	292	20	71	3193	1	3108	0	3886

TRINITY_DN16135_c0_g1_i1	Glyma.02G109900.2	95.55555556	263	17	1477	4603	3103	1	0	4041

TRINITY_DN16135_c0_g1_i1	Glyma.02G109900.1	100	263	17	1477	4603	3103	1	0	4041

TRINITY_DN16368_c0_g2_i2	Glyma.02G145200.1	100	226	3	1	3135	952	4086	0	4516

TRINITY_DN16151_c0_g2_i5	Glyma.01G038300.1	100	357	31	253	3341	1	3122	0	3419

TRINITY_DN16420_c0_g1_i4	Glyma.06G201700.1	97.08737864	241	4	92	3220	1	3132	0	4385

TRINITY_DN14953_c0_g3_i2	Glyma.04G123900.1	98.07692308	233	11	549	3677	3162	49	0	4313

TRINITY_DN16420_c0_g1_i7	Glyma.06G201700.1	95	229	4	92	3220	1	3132	0	4451

TRINITY_DN14953_c0_g3_i2	Glyma.04G123900.2	98.07692308	234	11	549	3677	3159	49	0	4300

TRINITY_DN13941_c0_g1_i1	Glyma.12G008500.1	99.05362776	254	32	2	3094	460	3552	0	3862

TRINITY_DN16420_c0_g1_i6	Glyma.06G201700.1	99.05660377	230	10	92	3199	1	3132	0	4355

TRINITY_DN16420_c0_g1_i8	Glyma.06G201700.1	99.12280702	230	10	92	3199	1	3132	0	4355

TRINITY_DN6279_c0_g1_i1	Glyma.12G008200.1	100	225	9	174	3299	1	3114	0	4394

TRINITY_DN13689_c0_g1_i1	Glyma.06G139800.2	96.69811321	245	9	258	3384	1	3118	0	4325

TRINITY_DN13689_c0_g1_i1	Glyma.06G139800.3	96.70103093	245	9	258	3384	1	3118	0	4325

TRINITY_DN13689_c0_g1_i1	Glyma.06G139800.5	96.7032967	245	9	258	3384	1	3118	0	4325

TRINITY_DN13689_c0_g1_i1	Glyma.06G139800.7	96.49681529	245	9	258	3384	1	3118	0	4325

TRINITY_DN13689_c0_g1_i1	Glyma.06G139800.9	96.49805447	245	9	258	3384	1	3118	0	4325

TRINITY_DN13689_c0_g1_i1	Glyma.06G139800.4	98.85057471	243	10	258	3384	1	3109	0	4279

TRINITY_DN6279_c0_g1_i1	Glyma.09G228300.1	100	227	10	174	3299	1	3111	0	4385

TRINITY_DN11569_c0_g1_i1	Glyma.13G059200.1	95.23809524	236	15	148	3249	3117	1	0	4229

TRINITY_DN14517_c0_g1_i3	Glyma.12G027500.1	98.53658537	204	1	198	3325	1	3120	0	4595

TRINITY_DN14517_c0_g1_i3	Glyma.11G101600.1	95.27559055	203	3	198	3325	1	3117	0	4580

TRINITY_DN13716_c0_g2_i1	Glyma.10G205500.1	97.36842105	168	13	548	3673	3099	1	0	4645

TRINITY_DN16151_c0_g2_i7	Glyma.01G038300.1	95.94594595	167	4	318	3436	1	3122	0	4754

TRINITY_DN13689_c0_g1_i1	Glyma.06G139800.11	99.02439024	245	9	258	3381	1	3115	0	4320

TRINITY_DN13689_c0_g1_i1	Glyma.06G139800.10	96.59863946	243	10	258	3381	1	3106	0	4274

TRINITY_DN3417_c0_g1_i1	Glyma.08G174300.1	100	201	16	1	3112	19	3139	0	4525

TRINITY_DN9524_c0_g1_i1	Glyma.05G221500.1	97.90026247	237	7	192	3303	132	3249	0	4318

TRINITY_DN9524_c0_g1_i1	Glyma.08G028000.1	98.26086957	229	12	192	3303	132	3246	0	4342

TRINITY_DN13689_c0_g1_i1	Glyma.04G225000.1	98.98989899	258	2	258	3382	1	3119	0	4303

TRINITY_DN13968_c0_g3_i2	Glyma.13G183900.2	100	361	14	1204	4314	829	3936	0	3581

TRINITY_DN13968_c0_g3_i2	Glyma.13G183900.3	100	361	14	1204	4314	781	3888	0	3581

TRINITY_DN15533_c0_g1_i5	Glyma.13G092700.1	96.07843137	318	21	160	3231	1	3111	0	3587

TRINITY_DN14460_c0_g2_i1	Glyma.17G072200.1	100	306	19	53	3170	1	3103	0	3899

TRINITY_DN334_c0_g1_i1	Glyma.13G158400.2	100	265	8	1	3112	219	3342	0	4224

TRINITY_DN13968_c0_g3_i3	Glyma.13G183900.2	99.20634921	434	22	1204	4308	829	3936	0	3149

TRINITY_DN13968_c0_g3_i3	Glyma.13G183900.3	99.25373134	434	22	1204	4308	781	3888	0	3149

TRINITY_DN14460_c0_g2_i1	Glyma.17G072200.2	100	304	15	53	3170	1	3100	0	3903

TRINITY_DN15405_c0_g1_i6	Glyma.06G323700.1	100	214	11	663	3767	3114	1	0	4407

TRINITY_DN13514_c0_g1_i1	Glyma.17G120500.1	100	163	0	1	3123	5898	2776	0	4865

TRINITY_DN15770_c0_g2_i1	Glyma.19G136400.3	98.61111111	481	31	211	3309	6	3108	0	2841

TRINITY_DN15770_c0_g2_i1	Glyma.19G136400.4	100	481	31	211	3309	6	3108	0	2841

TRINITY_DN15770_c0_g2_i1	Glyma.19G136400.2	97.4025974	481	31	211	3309	6	3108	0	2841

TRINITY_DN15770_c0_g2_i1	Glyma.19G136400.1	100	481	31	211	3309	6	3108	0	2841

TRINITY_DN14460_c0_g2_i1	Glyma.02G205800.1	100	302	15	53	3168	1	3098	0	3910

TRINITY_DN13968_c0_g3_i4	Glyma.13G183900.2	95.09803922	276	10	1204	4317	829	3936	0	4106

TRINITY_DN13968_c0_g3_i4	Glyma.13G183900.3	96.55172414	276	10	1204	4317	781	3888	0	4106

TRINITY_DN13968_c0_g3_i1	Glyma.13G183900.2	98.10126582	349	15	1204	4311	829	3936	0	3674

TRINITY_DN13968_c0_g3_i1	Glyma.13G183900.3	98.36065574	349	15	1204	4311	781	3888	0	3674

TRINITY_DN3417_c0_g1_i1	Glyma.15G252800.1	100	217	10	1	3114	19	3132	0	4482

TRINITY_DN13514_c0_g1_i1	Glyma.05G012400.1	100	167	0	4	3123	5892	2773	0	4837

TRINITY_DN14460_c0_g2_i1	Glyma.04G153700.1	100	143	0	53	3172	1	3120	0	4970

TRINITY_DN14460_c0_g2_i1	Glyma.06G225500.1	100	128	0	53	3172	1	3120	0	5053

TRINITY_DN14460_c0_g2_i1	Glyma.06G225500.2	100	128	0	53	3172	1	3120	0	5053

TRINITY_DN15639_c1_g1_i1	Glyma.15G044500.32	97.12230216	516	45	551	3616	3141	46	0	2410

TRINITY_DN15639_c1_g1_i1	Glyma.15G044500.24	97.12230216	516	45	551	3616	3141	46	0	2410

TRINITY_DN15639_c1_g1_i1	Glyma.15G044500.30	97.12230216	516	45	551	3616	3141	46	0	2410

TRINITY_DN15639_c1_g1_i1	Glyma.15G044500.28	97.70114943	516	45	551	3616	3141	46	0	2410

TRINITY_DN15639_c1_g1_i1	Glyma.15G044500.21	99.38650307	516	45	551	3616	3141	46	0	2410

TRINITY_DN15639_c1_g1_i1	Glyma.15G044500.18	100	516	45	551	3616	3141	46	0	2410

TRINITY_DN15639_c1_g1_i1	Glyma.15G044500.1	95.3271028	516	45	551	3616	3141	46	0	2410

TRINITY_DN15639_c1_g1_i1	Glyma.15G044500.22	97.45454545	516	45	551	3616	3141	46	0	2410

TRINITY_DN15639_c1_g1_i1	Glyma.15G044500.7	97.45762712	516	45	551	3616	3141	46	0	2410

TRINITY_DN15639_c1_g1_i1	Glyma.15G044500.5	98.51190476	516	45	551	3616	3141	46	0	2410

TRINITY_DN15639_c1_g1_i1	Glyma.15G044500.14	99.03846154	516	45	551	3616	3141	46	0	2410

TRINITY_DN15639_c1_g1_i2	Glyma.15G044500.32	96.20535714	516	45	675	3740	3141	46	0	2410

TRINITY_DN15639_c1_g1_i2	Glyma.15G044500.24	95.30364372	516	45	675	3740	3141	46	0	2410

TRINITY_DN15639_c1_g1_i2	Glyma.15G044500.30	95.20295203	516	45	675	3740	3141	46	0	2410

TRINITY_DN15639_c1_g1_i2	Glyma.15G044500.28	96.33507853	516	45	675	3740	3141	46	0	2410

TRINITY_DN15639_c1_g1_i2	Glyma.15G044500.21	100	516	45	675	3740	3141	46	0	2410

TRINITY_DN15639_c1_g1_i2	Glyma.15G044500.18	97.8021978	516	45	675	3740	3141	46	0	2410

TRINITY_DN15639_c1_g1_i2	Glyma.15G044500.1	100	516	45	675	3740	3141	46	0	2410

TRINITY_DN15639_c1_g1_i2	Glyma.15G044500.22	100	516	45	675	3740	3141	46	0	2410

TRINITY_DN15639_c1_g1_i2	Glyma.15G044500.7	97.52066116	516	45	675	3740	3141	46	0	2410

TRINITY_DN15639_c1_g1_i2	Glyma.15G044500.5	97.84946237	516	45	675	3740	3141	46	0	2410

TRINITY_DN15639_c1_g1_i2	Glyma.15G044500.14	96.22641509	516	45	675	3740	3141	46	0	2410

TRINITY_DN15639_c1_g1_i3	Glyma.15G044500.32	99.29577465	516	45	358	3423	3141	46	0	2410

TRINITY_DN15639_c1_g1_i3	Glyma.15G044500.24	99.61977186	516	45	358	3423	3141	46	0	2410

TRINITY_DN15639_c1_g1_i3	Glyma.15G044500.30	99.62121212	516	45	358	3423	3141	46	0	2410

TRINITY_DN15639_c1_g1_i3	Glyma.15G044500.28	96.15384615	516	45	358	3423	3141	46	0	2410

TRINITY_DN15639_c1_g1_i3	Glyma.15G044500.21	98.41269841	516	45	358	3423	3141	46	0	2410

TRINITY_DN15639_c1_g1_i3	Glyma.15G044500.18	98.41427156	516	45	358	3423	3141	46	0	2410

TRINITY_DN15639_c1_g1_i3	Glyma.15G044500.1	95.76719577	516	45	358	3423	3141	46	0	2410

TRINITY_DN15639_c1_g1_i3	Glyma.15G044500.22	99.11504425	516	45	358	3423	3141	46	0	2410

TRINITY_DN15639_c1_g1_i3	Glyma.15G044500.7	97.27272727	516	45	358	3423	3141	46	0	2410

TRINITY_DN15639_c1_g1_i3	Glyma.15G044500.5	100	516	45	358	3423	3141	46	0	2410

TRINITY_DN15639_c1_g1_i3	Glyma.15G044500.14	100	516	45	358	3423	3141	46	0	2410

TRINITY_DN15886_c3_g1_i1	Glyma.15G190200.1	96.55172414	290	12	384	3497	1	3099	0	3991

TRINITY_DN15886_c3_g1_i1	Glyma.15G190200.2	97.77777778	290	12	384	3497	1	3099	0	3991

TRINITY_DN15886_c3_g1_i3	Glyma.15G190200.1	100	290	12	593	3706	1	3099	0	3991

TRINITY_DN15886_c3_g1_i3	Glyma.15G190200.2	98.4375	290	12	593	3706	1	3099	0	3991

TRINITY_DN11567_c0_g1_i1	Glyma.09G052200.1	96.55172414	301	5	704	3809	3684	582	0	3916

TRINITY_DN11567_c0_g1_i2	Glyma.09G052200.1	95.89041096	301	5	352	3457	3684	582	0	3916

TRINITY_DN11567_c0_g1_i3	Glyma.09G052200.1	95.74468085	301	5	440	3545	3684	582	0	3916

TRINITY_DN11567_c0_g1_i4	Glyma.09G052200.1	96.875	301	5	369	3474	3684	582	0	3916

TRINITY_DN11569_c0_g1_i1	Glyma.19G027200.1	100	248	12	148	3249	3111	1	0	4235

TRINITY_DN15994_c0_g1_i2	Glyma.13G367300.4	100	192	11	745	3862	3087	1	0	4494

TRINITY_DN8232_c0_g1_i1	Glyma.08G196100.2	100	318	13	338	3433	1	3111	0	3819

TRINITY_DN15639_c1_g1_i1	Glyma.15G044500.31	99.18699187	516	44	551	3616	3138	46	0	2423

TRINITY_DN15639_c1_g1_i1	Glyma.15G044500.23	99.18699187	516	44	551	3616	3138	46	0	2423

TRINITY_DN15639_c1_g1_i1	Glyma.15G044500.29	97.02970297	516	44	551	3616	3138	46	0	2423

TRINITY_DN15639_c1_g1_i1	Glyma.15G044500.27	98.60788863	516	44	551	3616	3138	46	0	2423

TRINITY_DN15639_c1_g1_i1	Glyma.15G044500.13	95.87231701	516	44	551	3616	3138	46	0	2423

TRINITY_DN15639_c1_g1_i1	Glyma.15G044500.12	95.75671853	516	44	551	3616	3138	46	0	2423

TRINITY_DN15639_c1_g1_i1	Glyma.15G044500.19	99.48979592	516	44	551	3616	3138	46	0	2423

TRINITY_DN15639_c1_g1_i1	Glyma.15G044500.17	97.12121212	516	44	551	3616	3138	46	0	2423

TRINITY_DN15639_c1_g1_i1	Glyma.15G044500.20	97.12230216	516	44	551	3616	3138	46	0	2423

TRINITY_DN15639_c1_g1_i1	Glyma.15G044500.6	97.12230216	516	44	551	3616	3138	46	0	2423

TRINITY_DN15639_c1_g1_i1	Glyma.15G044500.4	97.12230216	516	44	551	3616	3138	46	0	2423

TRINITY_DN15639_c1_g1_i2	Glyma.15G044500.31	97.14285714	516	44	675	3740	3138	46	0	2423

TRINITY_DN15639_c1_g1_i2	Glyma.15G044500.23	97.14285714	516	44	675	3740	3138	46	0	2423

TRINITY_DN15639_c1_g1_i2	Glyma.15G044500.29	97.14285714	516	44	675	3740	3138	46	0	2423

TRINITY_DN15639_c1_g1_i2	Glyma.15G044500.27	100	516	44	675	3740	3138	46	0	2423

TRINITY_DN15639_c1_g1_i2	Glyma.15G044500.13	95.56074766	516	44	675	3740	3138	46	0	2423

TRINITY_DN15639_c1_g1_i2	Glyma.15G044500.12	97.71573604	516	44	675	3740	3138	46	0	2423

TRINITY_DN15639_c1_g1_i2	Glyma.15G044500.19	97.71573604	516	44	675	3740	3138	46	0	2423

TRINITY_DN15639_c1_g1_i2	Glyma.15G044500.17	97.26027397	516	44	675	3740	3138	46	0	2423

TRINITY_DN15639_c1_g1_i2	Glyma.15G044500.20	98.37398374	516	44	675	3740	3138	46	0	2423

TRINITY_DN15639_c1_g1_i2	Glyma.15G044500.6	97.64705882	516	44	675	3740	3138	46	0	2423

TRINITY_DN15639_c1_g1_i2	Glyma.15G044500.4	95.01779359	516	44	675	3740	3138	46	0	2423

TRINITY_DN15639_c1_g1_i3	Glyma.15G044500.31	95.95375723	516	44	358	3423	3138	46	0	2423

TRINITY_DN15639_c1_g1_i3	Glyma.15G044500.23	96.88311688	516	44	358	3423	3138	46	0	2423

TRINITY_DN15639_c1_g1_i3	Glyma.15G044500.29	98.98989899	516	44	358	3423	3138	46	0	2423

TRINITY_DN15639_c1_g1_i3	Glyma.15G044500.27	98.98989899	516	44	358	3423	3138	46	0	2423

TRINITY_DN15639_c1_g1_i3	Glyma.15G044500.13	95.3271028	516	44	358	3423	3138	46	0	2423

TRINITY_DN15639_c1_g1_i3	Glyma.15G044500.12	100	516	44	358	3423	3138	46	0	2423

TRINITY_DN15639_c1_g1_i3	Glyma.15G044500.19	100	516	44	358	3423	3138	46	0	2423

TRINITY_DN15639_c1_g1_i3	Glyma.15G044500.17	98.11320755	516	44	358	3423	3138	46	0	2423

TRINITY_DN15639_c1_g1_i3	Glyma.15G044500.20	99.29577465	516	44	358	3423	3138	46	0	2423

TRINITY_DN15639_c1_g1_i3	Glyma.15G044500.6	100	516	44	358	3423	3138	46	0	2423

TRINITY_DN15639_c1_g1_i3	Glyma.15G044500.4	100	516	44	358	3423	3138	46	0	2423

TRINITY_DN15533_c0_g1_i5	Glyma.17G067700.1	96.07843137	345	19	160	3231	1	3105	0	3487

TRINITY_DN15994_c0_g1_i3	Glyma.13G367300.4	98.55072464	285	13	745	3850	3087	1	0	3923

TRINITY_DN15886_c3_g1_i1	Glyma.09G082000.1	98.4375	277	7	384	3497	1	3099	0	4108

TRINITY_DN15886_c3_g1_i1	Glyma.09G082000.2	95.38461538	277	7	384	3497	1	3099	0	4108

TRINITY_DN15886_c3_g1_i3	Glyma.09G082000.1	100	277	7	593	3706	1	3099	0	4108

TRINITY_DN15886_c3_g1_i3	Glyma.09G082000.2	100	277	7	593	3706	1	3099	0	4108

TRINITY_DN8232_c0_g1_i1	Glyma.07G012400.5	100	327	13	338	3433	1	3108	0	3784

TRINITY_DN8232_c0_g1_i1	Glyma.07G012400.4	100	327	13	338	3433	1	3108	0	3784

TRINITY_DN8232_c0_g1_i1	Glyma.07G012400.3	97.42765273	327	13	338	3433	1	3108	0	3784

TRINITY_DN15639_c1_g1_i1	Glyma.15G044500.26	95.65217391	516	45	551	3610	3090	1	0	2399

TRINITY_DN15639_c1_g1_i1	Glyma.15G044500.16	95.65217391	516	45	551	3610	3090	1	0	2399

TRINITY_DN15639_c1_g1_i1	Glyma.15G044500.10	95.65217391	516	45	551	3610	3090	1	0	2399

TRINITY_DN15639_c1_g1_i1	Glyma.15G044500.11	95.65217391	516	45	551	3610	3090	1	0	2399

TRINITY_DN15639_c1_g1_i1	Glyma.15G044500.3	95.65217391	516	45	551	3610	3090	1	0	2399

TRINITY_DN15639_c1_g1_i2	Glyma.15G044500.26	96.88311688	516	45	675	3734	3090	1	0	2399

TRINITY_DN15639_c1_g1_i2	Glyma.15G044500.16	100	516	45	675	3734	3090	1	0	2399

TRINITY_DN15639_c1_g1_i2	Glyma.15G044500.10	99.33333333	516	45	675	3734	3090	1	0	2399

TRINITY_DN15639_c1_g1_i2	Glyma.15G044500.11	95.20958084	516	45	675	3734	3090	1	0	2399

TRINITY_DN15639_c1_g1_i2	Glyma.15G044500.3	99.13793103	516	45	675	3734	3090	1	0	2399

TRINITY_DN15639_c1_g1_i3	Glyma.15G044500.26	100	516	45	358	3417	3090	1	0	2399

TRINITY_DN15639_c1_g1_i3	Glyma.15G044500.16	95.04716981	516	45	358	3417	3090	1	0	2399

TRINITY_DN15639_c1_g1_i3	Glyma.15G044500.10	97.70723104	516	45	358	3417	3090	1	0	2399

TRINITY_DN15639_c1_g1_i3	Glyma.15G044500.11	97.70833333	516	45	358	3417	3090	1	0	2399

TRINITY_DN15639_c1_g1_i3	Glyma.15G044500.3	99.04534606	516	45	358	3417	3090	1	0	2399

TRINITY_DN15264_c0_g1_i2	Glyma.02G008000.1	98.92473118	435	11	285	3383	91	3198	0	3216

TRINITY_DN16420_c0_g1_i7	Glyma.01G040900.1	99.71098266	388	24	102	3192	11	3092	0	3256

TRINITY_DN16420_c0_g1_i7	Glyma.01G040900.2	100	388	24	102	3192	11	3092	0	3256

TRINITY_DN11567_c0_g1_i1	Glyma.15G158500.1	98.7654321	305	9	704	3809	3681	579	0	3949

TRINITY_DN11567_c0_g1_i1	Glyma.15G158500.2	98.7654321	305	9	704	3809	3681	579	0	3949

TRINITY_DN11567_c0_g1_i2	Glyma.15G158500.1	96.55172414	305	9	352	3457	3681	579	0	3949

TRINITY_DN11567_c0_g1_i2	Glyma.15G158500.2	95.89041096	305	9	352	3457	3681	579	0	3949

TRINITY_DN11567_c0_g1_i3	Glyma.15G158500.1	95.89041096	305	9	440	3545	3681	579	0	3949

TRINITY_DN11567_c0_g1_i3	Glyma.15G158500.2	98.64864865	305	9	440	3545	3681	579	0	3949

TRINITY_DN11567_c0_g1_i4	Glyma.15G158500.1	97.24770642	305	9	369	3474	3681	579	0	3949

TRINITY_DN11567_c0_g1_i4	Glyma.15G158500.2	97.14285714	305	9	369	3474	3681	579	0	3949

TRINITY_DN14991_c0_g2_i1	Glyma.13G082100.5	100	253	12	692	3781	1	3102	0	4128

TRINITY_DN15264_c0_g1_i9	Glyma.03G224100.1	95.55555556	412	12	244	3348	103	3201	0	3336

TRINITY_DN16420_c0_g1_i4	Glyma.01G040900.1	97.08737864	394	22	102	3192	11	3092	0	3234

TRINITY_DN16420_c0_g1_i4	Glyma.01G040900.2	98.61111111	394	22	102	3192	11	3092	0	3234

TRINITY_DN11786_c1_g1_i2	Glyma.18G011200.4	96.96969697	304	6	1	3107	1183	4292	0	4017

TRINITY_DN11786_c1_g1_i2	Glyma.18G011200.3	97.82608696	304	6	1	3107	1183	4292	0	4017

TRINITY_DN11786_c1_g1_i2	Glyma.18G011200.2	96.2962963	304	6	1	3107	1183	4292	0	4017

TRINITY_DN11786_c1_g1_i2	Glyma.18G011200.1	97.5	304	6	1	3107	1183	4292	0	4017

TRINITY_DN11786_c1_g1_i1	Glyma.18G011200.4	98.97260274	303	6	1	3107	1183	4292	0	4023

TRINITY_DN11786_c1_g1_i1	Glyma.18G011200.3	95.97855228	303	6	1	3107	1183	4292	0	4023

TRINITY_DN11786_c1_g1_i1	Glyma.18G011200.2	98.51851852	303	6	1	3107	1183	4292	0	4023

TRINITY_DN11786_c1_g1_i1	Glyma.18G011200.1	96.75324675	303	6	1	3107	1183	4292	0	4023

TRINITY_DN13964_c0_g1_i1	Glyma.13G279600.1	100	291	14	1041	4132	3101	1	0	3934

TRINITY_DN16254_c0_g1_i3	Glyma.09G061200.4	95.43973941	266	10	375	3481	3220	114	0	4209

TRINITY_DN16254_c0_g1_i3	Glyma.09G061200.3	95.65217391	266	10	375	3481	3220	114	0	4209

TRINITY_DN16254_c0_g1_i3	Glyma.09G061200.1	95.40636042	266	10	375	3481	3220	114	0	4209

TRINITY_DN16254_c0_g1_i3	Glyma.09G061200.2	97.12389381	266	10	375	3481	3220	114	0	4209

TRINITY_DN16254_c0_g1_i3	Glyma.09G061200.5	97.12389381	266	10	375	3481	3220	114	0	4209

TRINITY_DN5514_c0_g1_i1	Glyma.19G208700.1	100	248	20	323	3421	3075	1	0	4050

TRINITY_DN16254_c0_g1_i2	Glyma.09G061200.4	97.17514124	237	6	375	3481	3220	114	0	4370

TRINITY_DN16254_c0_g1_i2	Glyma.09G061200.3	97.17514124	237	6	375	3481	3220	114	0	4370

TRINITY_DN16254_c0_g1_i2	Glyma.09G061200.1	97.17514124	237	6	375	3481	3220	114	0	4370

TRINITY_DN16254_c0_g1_i2	Glyma.09G061200.2	97.17647059	237	6	375	3481	3220	114	0	4370

TRINITY_DN16254_c0_g1_i2	Glyma.09G061200.5	97.17741935	237	6	375	3481	3220	114	0	4370

TRINITY_DN15264_c0_g1_i2	Glyma.10G137600.3	99.80314961	438	8	285	3383	91	3198	0	3221

TRINITY_DN15264_c0_g1_i2	Glyma.10G137600.2	100	438	8	285	3383	91	3198	0	3221

TRINITY_DN15264_c0_g1_i2	Glyma.10G137600.1	98.16513761	438	8	285	3383	91	3198	0	3221

TRINITY_DN15847_c1_g2_i2	Glyma.02G280400.1	96.36363636	306	20	1	3085	194	3296	0	3831

TRINITY_DN5514_c0_g1_i1	Glyma.03G211500.1	97.7827051	253	18	323	3421	3075	1	0	4034

TRINITY_DN15639_c1_g1_i1	Glyma.15G044500.25	97.12230216	516	44	551	3610	3087	1	0	2412

TRINITY_DN15639_c1_g1_i1	Glyma.15G044500.15	98.87640449	516	44	551	3610	3087	1	0	2412

TRINITY_DN15639_c1_g1_i1	Glyma.15G044500.8	98.87640449	516	44	551	3610	3087	1	0	2412

TRINITY_DN15639_c1_g1_i1	Glyma.15G044500.9	98.86363636	516	44	551	3610	3087	1	0	2412

TRINITY_DN15639_c1_g1_i1	Glyma.15G044500.2	100	516	44	551	3610	3087	1	0	2412

TRINITY_DN15639_c1_g1_i2	Glyma.15G044500.25	95.94594595	516	44	675	3734	3087	1	0	2412

TRINITY_DN15639_c1_g1_i2	Glyma.15G044500.15	95.23809524	516	44	675	3734	3087	1	0	2412

TRINITY_DN15639_c1_g1_i2	Glyma.15G044500.8	96.1038961	516	44	675	3734	3087	1	0	2412

TRINITY_DN15639_c1_g1_i2	Glyma.15G044500.9	95.40636042	516	44	675	3734	3087	1	0	2412

TRINITY_DN15639_c1_g1_i2	Glyma.15G044500.2	96.16564417	516	44	675	3734	3087	1	0	2412

TRINITY_DN15639_c1_g1_i3	Glyma.15G044500.25	96	516	44	358	3417	3087	1	0	2412

TRINITY_DN15639_c1_g1_i3	Glyma.15G044500.15	99.26470588	516	44	358	3417	3087	1	0	2412

TRINITY_DN15639_c1_g1_i3	Glyma.15G044500.8	97.5	516	44	358	3417	3087	1	0	2412

TRINITY_DN15639_c1_g1_i3	Glyma.15G044500.9	98.79518072	516	44	358	3417	3087	1	0	2412

TRINITY_DN15639_c1_g1_i3	Glyma.15G044500.2	95.23809524	516	44	358	3417	3087	1	0	2412

TRINITY_DN11610_c0_g2_i1	Glyma.16G162200.1	98.59813084	386	27	1	3082	204	3291	0	3293

TRINITY_DN15847_c1_g2_i4	Glyma.02G280400.1	97.2826087	255	12	1	3094	194	3296	0	4193

TRINITY_DN14991_c0_g2_i1	Glyma.13G082100.2	100	253	12	692	3777	1	3098	0	4120

TRINITY_DN9262_c0_g1_i1	Glyma.05G212000.4	100	220	16	1	3068	616	3716	0	4217

TRINITY_DN9262_c0_g1_i2	Glyma.05G212000.4	96.3190184	220	16	1	3068	616	3716	0	4217

TRINITY_DN12627_c0_g1_i2	Glyma.13G317500.1	96.30484988	174	12	313	3411	1	3087	0	4582

TRINITY_DN12627_c0_g1_i2	Glyma.13G317500.2	95.01246883	174	12	313	3411	1	3087	0	4582

TRINITY_DN16031_c1_g2_i2	Glyma.17G037600.1	99.54751131	393	20	297	3392	3081	1	0	3321

TRINITY_DN3701_c0_g1_i1	Glyma.12G191000.1	95.95959596	256	9	1	3091	981	4080	0	4170

TRINITY_DN12627_c0_g1_i2	Glyma.12G183500.1	96.33027523	170	10	313	3411	1	3096	0	4673

TRINITY_DN12627_c0_g1_i2	Glyma.12G183500.2	96.34146341	168	13	313	3411	1	3084	0	4606

TRINITY_DN16031_c1_g2_i2	Glyma.07G235800.1	99.54751131	385	20	297	3392	3081	1	0	3376

TRINITY_DN15847_c1_g2_i2	Glyma.14G034200.1	96.36363636	313	16	1	3085	197	3293	0	3808

TRINITY_DN15847_c1_g2_i2	Glyma.14G034200.2	96.36363636	313	16	1	3085	194	3290	0	3808

TRINITY_DN3701_c0_g1_i1	Glyma.13G310700.1	97.08029197	285	9	1	3091	981	4080	0	4021

TRINITY_DN15384_c0_g1_i1	Glyma.04G240900.1	100	222	14	179	3280	73	3153	0	4318

TRINITY_DN16215_c0_g1_i3	Glyma.19G234300.1	97.19703215	353	17	313	3356	3228	128	0	3358

TRINITY_DN16215_c0_g1_i1	Glyma.19G234300.1	100	346	18	537	3580	3228	128	0	3397

TRINITY_DN5433_c0_g1_i2	Glyma.07G233500.1	99.49494949	263	12	301	3401	3303	235	0	4019

TRINITY_DN2586_c0_g1_i1	Glyma.20G226900.1	97.72370487	234	5	130	3225	3099	1	0	4340

TRINITY_DN14481_c0_g3_i1	Glyma.13G234600.1	100	450	19	1	3089	86	3180	0	3085

TRINITY_DN15914_c0_g2_i2	Glyma.02G271900.1	100	186	0	145	3248	3104	1	0	4702

TRINITY_DN15914_c0_g2_i2	Glyma.14G044900.1	100	181	0	145	3248	3104	1	0	4730

TRINITY_DN15914_c0_g2_i2	Glyma.14G044900.2	100	181	0	145	3248	3104	1	0	4730

TRINITY_DN15847_c1_g2_i4	Glyma.14G034200.1	98.56630824	269	7	1	3094	197	3293	0	4143

TRINITY_DN15847_c1_g2_i4	Glyma.14G034200.2	100	269	7	1	3094	194	3290	0	4143

TRINITY_DN2586_c0_g1_i1	Glyma.10G164700.1	95.55214724	218	2	132	3225	3103	1	0	4464

TRINITY_DN15988_c0_g1_i3	Glyma.08G258700.1	98.97959184	142	3	764	3865	3099	1	0	4911

TRINITY_DN16031_c1_g2_i1	Glyma.15G137000.1	100	379	9	297	3377	3096	1	0	3454

TRINITY_DN16031_c1_g2_i1	Glyma.15G137000.2	100	377	15	297	3377	3087	1	0	3408

TRINITY_DN5433_c0_g1_i2	Glyma.17G038500.1	96.54576857	258	7	301	3401	3333	259	0	4119

TRINITY_DN13926_c0_g1_i2	Glyma.01G234600.1	100	251	29	239	3273	3081	1	0	3770

TRINITY_DN15988_c0_g1_i3	Glyma.18G282800.1	100	147	1	764	3865	3099	1	0	4894

TRINITY_DN15847_c1_g2_i4	Glyma.18G119000.1	95.46165884	245	14	1	3088	200	3284	0	4181

TRINITY_DN15847_c1_g2_i4	Glyma.18G119000.3	96.36913767	245	14	1	3088	131	3215	0	4181

TRINITY_DN14659_c2_g1_i3	Glyma.04G255100.6	100	450	22	493	3582	3666	592	0	3005

TRINITY_DN14659_c2_g1_i3	Glyma.04G255100.4	100	450	22	493	3582	3666	592	0	3005

TRINITY_DN14659_c2_g1_i3	Glyma.04G255100.1	100	450	22	493	3582	3666	592	0	3005

TRINITY_DN14659_c2_g1_i3	Glyma.04G255100.5	95.12195122	450	22	493	3582	3621	547	0	3005

TRINITY_DN14659_c2_g1_i3	Glyma.04G255100.3	98.46153846	450	22	493	3582	3621	547	0	3005

TRINITY_DN14659_c2_g1_i3	Glyma.04G255100.2	97.95918367	450	22	493	3582	3621	547	0	3005

TRINITY_DN16212_c0_g1_i3	Glyma.13G142100.2	100	404	0	986	4083	3309	212	0	3483

TRINITY_DN16212_c0_g1_i2	Glyma.13G142100.2	97.91231733	355	0	986	4083	3309	212	0	3755

TRINITY_DN15847_c1_g2_i4	Glyma.08G302000.1	99.13793103	248	11	1	3088	209	3293	0	4198

TRINITY_DN16245_c0_g2_i1	Glyma.19G007400.3	96.72131148	221	14	318	3410	3207	130	0	4335

TRINITY_DN16332_c0_g3_i1	Glyma.17G239500.5	97.54098361	345	12	582	3670	3093	5	0	3705

TRINITY_DN16332_c0_g3_i1	Glyma.17G239500.1	97.52747253	345	12	582	3670	3093	5	0	3705

TRINITY_DN16252_c0_g1_i1	Glyma.08G293600.11	96.53679654	305	21	592	3675	1	3069	0	3764

TRINITY_DN16252_c0_g1_i3	Glyma.08G293600.11	96.42857143	305	21	592	3675	1	3069	0	3764

TRINITY_DN14659_c2_g1_i2	Glyma.04G255100.6	97.04797048	466	19	332	3421	3666	592	0	2961

TRINITY_DN14659_c2_g1_i2	Glyma.04G255100.4	97.04918033	466	19	332	3421	3666	592	0	2961

TRINITY_DN14659_c2_g1_i2	Glyma.04G255100.1	100	466	19	332	3421	3666	592	0	2961

TRINITY_DN14659_c2_g1_i2	Glyma.04G255100.5	100	466	19	332	3421	3621	547	0	2961

TRINITY_DN14659_c2_g1_i2	Glyma.04G255100.3	95.45454545	466	19	332	3421	3621	547	0	2961

TRINITY_DN14659_c2_g1_i2	Glyma.04G255100.2	97.04797048	466	19	332	3421	3621	547	0	2961

TRINITY_DN14246_c0_g1_i1	Glyma.08G105000.6	100	188	3	789	3878	1	3093	0	4617

TRINITY_DN14246_c0_g1_i1	Glyma.08G105000.7	100	188	3	789	3878	1	3093	0	4617

TRINITY_DN14246_c0_g1_i1	Glyma.08G105000.8	100	188	3	789	3878	1	3093	0	4617

TRINITY_DN14246_c0_g1_i1	Glyma.08G105000.2	100	188	3	789	3878	1	3093	0	4617

TRINITY_DN14246_c0_g1_i1	Glyma.08G105000.3	100	188	3	789	3878	1	3093	0	4617

TRINITY_DN14246_c0_g1_i1	Glyma.08G105000.4	100	188	3	789	3878	1	3093	0	4617

TRINITY_DN14246_c0_g1_i1	Glyma.08G105000.5	100	188	3	789	3878	1	3093	0	4617

TRINITY_DN14246_c0_g1_i1	Glyma.08G105000.1	100	188	3	789	3878	1	3093	0	4617

TRINITY_DN14246_c0_g1_i2	Glyma.08G105000.6	100	188	3	789	3878	1	3093	0	4617

TRINITY_DN14246_c0_g1_i2	Glyma.08G105000.7	100	188	3	789	3878	1	3093	0	4617

TRINITY_DN14246_c0_g1_i2	Glyma.08G105000.8	100	188	3	789	3878	1	3093	0	4617

TRINITY_DN14246_c0_g1_i2	Glyma.08G105000.2	100	188	3	789	3878	1	3093	0	4617

TRINITY_DN14246_c0_g1_i2	Glyma.08G105000.3	100	188	3	789	3878	1	3093	0	4617

TRINITY_DN14246_c0_g1_i2	Glyma.08G105000.4	100	188	3	789	3878	1	3093	0	4617

TRINITY_DN14246_c0_g1_i2	Glyma.08G105000.5	100	188	3	789	3878	1	3093	0	4617

TRINITY_DN14246_c0_g1_i2	Glyma.08G105000.1	100	188	3	789	3878	1	3093	0	4617

TRINITY_DN15225_c0_g1_i1	Glyma.02G094300.1	100	187	8	509	3598	3090	1	0	4604

TRINITY_DN16031_c1_g2_i2	Glyma.15G137000.1	99.54545455	183	0	297	3392	3096	1	0	4704

TRINITY_DN14246_c0_g1_i1	Glyma.05G148100.1	100	181	3	789	3878	1	3093	0	4656

TRINITY_DN14246_c0_g1_i2	Glyma.05G148100.1	100	181	3	789	3878	1	3093	0	4656

TRINITY_DN16031_c1_g2_i2	Glyma.15G137000.2	99.54545455	178	3	297	3392	3087	1	0	4673

TRINITY_DN4996_c0_g1_i1	Glyma.14G032500.1	98.98989899	356	37	286	3340	4289	1244	0	3168

TRINITY_DN16332_c0_g3_i1	Glyma.14G085200.1	97.5308642	350	11	582	3670	3090	5	0	3679

TRINITY_DN8367_c0_g1_i4	Glyma.14G193900.1	98.86363636	334	26	138	3218	3283	224	0	3548

TRINITY_DN11650_c0_g1_i1	Glyma.08G143000.1	99.17355372	248	21	74	3153	1	3076	0	4122

TRINITY_DN11650_c0_g1_i1	Glyma.08G143000.2	97.22222222	248	21	74	3153	1	3076	0	4122

TRINITY_DN15886_c3_g1_i1	Glyma.07G234200.1	100	519	57	452	3497	81	3120	0	2180

TRINITY_DN15886_c3_g1_i1	Glyma.07G234200.3	96.69421488	519	57	452	3497	81	3120	0	2180

TRINITY_DN15886_c3_g1_i3	Glyma.07G234200.1	95.37815126	519	57	661	3706	81	3120	0	2180

TRINITY_DN15886_c3_g1_i3	Glyma.07G234200.3	95.37815126	519	57	661	3706	81	3120	0	2180

TRINITY_DN16420_c0_g1_i6	Glyma.01G040900.1	100	401	17	102	3171	11	3092	0	3260

TRINITY_DN16420_c0_g1_i6	Glyma.01G040900.2	97.91666667	401	17	102	3171	11	3092	0	3260

TRINITY_DN16420_c0_g1_i8	Glyma.01G040900.1	95.94594595	401	17	102	3171	11	3092	0	3260

TRINITY_DN16420_c0_g1_i8	Glyma.01G040900.2	100	401	17	102	3171	11	3092	0	3260

TRINITY_DN16332_c0_g3_i1	Glyma.17G239500.3	97.54098361	346	10	582	3670	3090	5	0	3712

TRINITY_DN16332_c0_g3_i1	Glyma.17G239500.4	97.54601227	346	10	582	3670	3090	5	0	3712

TRINITY_DN16115_c0_g2_i4	Glyma.12G074300.1	100	333	12	332	3410	3129	42	0	3733

TRINITY_DN15576_c0_g2_i1	Glyma.16G030100.1	95.65217391	246	8	292	3375	3093	1	0	4279

TRINITY_DN11610_c0_g2_i1	Glyma.02G078900.1	98.61111111	371	29	11	3080	214	3271	0	3282

TRINITY_DN4996_c0_g1_i1	Glyma.02G281900.1	98.66666667	365	32	290	3340	4305	1244	0	3217

TRINITY_DN16245_c0_g1_i5	Glyma.15G025800.2	97.43589744	382	29	980	4059	3056	1	0	3284

TRINITY_DN15517_c0_g2_i2	Glyma.14G025300.1	97.45762712	354	10	403	3486	3225	139	0	3664

TRINITY_DN15732_c0_g1_i1	Glyma.09G261200.3	97.08295351	632	70	321	3347	3051	1	0	1535

TRINITY_DN15732_c0_g1_i1	Glyma.09G261200.5	97.08404803	632	70	321	3347	3051	1	0	1535

TRINITY_DN15732_c0_g1_i1	Glyma.09G261200.1	100	632	70	321	3347	3051	1	0	1535

TRINITY_DN15732_c0_g1_i1	Glyma.09G261200.2	100	632	70	321	3347	3051	1	0	1535

TRINITY_DN15732_c0_g1_i1	Glyma.09G261200.4	100	632	70	321	3347	3051	1	0	1535

TRINITY_DN15732_c0_g1_i2	Glyma.09G261200.3	98.87640449	632	70	321	3347	3051	1	0	1535

TRINITY_DN15732_c0_g1_i2	Glyma.09G261200.5	99.12280702	632	70	321	3347	3051	1	0	1535

TRINITY_DN15732_c0_g1_i2	Glyma.09G261200.1	96.49415693	632	70	321	3347	3051	1	0	1535

TRINITY_DN15732_c0_g1_i2	Glyma.09G261200.2	96.49446494	632	70	321	3347	3051	1	0	1535

TRINITY_DN15732_c0_g1_i2	Glyma.09G261200.4	96.49484536	632	70	321	3347	3051	1	0	1535

TRINITY_DN15732_c0_g1_i1	Glyma.18G230800.1	95.67039106	638	65	321	3347	3054	1	0	1531

TRINITY_DN15732_c0_g1_i2	Glyma.18G230800.1	98.16849817	638	65	321	3347	3054	1	0	1531

TRINITY_DN3079_c0_g2_i1	Glyma.03G151100.1	100	600	81	899	3915	565	3607	0	1607

TRINITY_DN14481_c0_g3_i1	Glyma.07G202100.1	100	231	8	1	3089	110	3183	0	4316

TRINITY_DN16332_c0_g3_i1	Glyma.06G042400.1	97.54098361	183	3	582	3671	3072	1	0	4577

TRINITY_DN16332_c0_g3_i1	Glyma.04G041300.1	97.54601227	174	2	582	3671	3084	1	0	4704

TRINITY_DN15225_c0_g1_i1	Glyma.18G287400.1	100	172	7	509	3598	3078	1	0	4676

TRINITY_DN15225_c0_g1_i1	Glyma.18G287400.2	100	172	7	509	3598	3078	1	0	4676

TRINITY_DN3079_c0_g1_i1	Glyma.03G151100.1	100	600	80	899	3914	565	3607	0	1611

TRINITY_DN15225_c0_g1_i2	Glyma.02G094300.1	95.32258065	269	8	238	3320	3083	1	0	4137

TRINITY_DN15988_c0_g1_i1	Glyma.08G258700.1	98.30508475	221	5	613	3699	3084	1	0	4434

TRINITY_DN16115_c0_g2_i1	Glyma.12G074300.1	100	204	4	230	3308	3129	42	0	4503

TRINITY_DN16115_c0_g2_i6	Glyma.12G074300.1	100	365	12	332	3404	3129	48	0	3544

TRINITY_DN5433_c0_g1_i1	Glyma.07G233500.1	96.54576857	263	11	301	3384	3303	235	0	4096

TRINITY_DN11650_c0_g1_i1	Glyma.05G185000.4	100	252	11	74	3149	1	3076	0	4152

TRINITY_DN11650_c0_g1_i1	Glyma.05G185000.1	95.45454545	252	11	74	3149	1	3076	0	4152

TRINITY_DN14481_c0_g3_i1	Glyma.13G174200.1	100	229	9	3	3089	112	3183	0	4324

TRINITY_DN15988_c0_g1_i1	Glyma.18G282800.1	96.88581315	227	3	613	3699	3084	1	0	4412

TRINITY_DN15847_c1_g2_i2	Glyma.18G119000.1	97.01492537	204	5	1	3079	200	3284	0	4495

TRINITY_DN15847_c1_g2_i2	Glyma.18G119000.3	98.2300885	204	5	1	3079	131	3215	0	4495

TRINITY_DN12050_c0_g2_i3	Glyma.16G093800.22	100	526	54	351	3384	1	3019	0	2004

TRINITY_DN12050_c0_g2_i3	Glyma.16G093800.20	100	526	54	351	3384	1	3019	0	2004

TRINITY_DN12050_c0_g2_i2	Glyma.16G093800.22	98.47826087	507	54	336	3369	1	3019	0	2109

TRINITY_DN12050_c0_g2_i2	Glyma.16G093800.20	98.46625767	507	54	336	3369	1	3019	0	2109

TRINITY_DN14659_c2_g1_i4	Glyma.04G255100.6	96.91119691	454	14	493	3570	3666	592	0	3049

TRINITY_DN14659_c2_g1_i4	Glyma.04G255100.4	96.91119691	454	14	493	3570	3666	592	0	3049

TRINITY_DN14659_c2_g1_i4	Glyma.04G255100.1	95.85492228	454	14	493	3570	3666	592	0	3049

TRINITY_DN14659_c2_g1_i4	Glyma.04G255100.5	99.21259843	454	14	493	3570	3621	547	0	3049

TRINITY_DN14659_c2_g1_i4	Glyma.04G255100.3	96.91011236	454	14	493	3570	3621	547	0	3049

TRINITY_DN14659_c2_g1_i4	Glyma.04G255100.2	96.91011236	454	14	493	3570	3621	547	0	3049

TRINITY_DN13939_c0_g1_i1	Glyma.03G229000.1	96.76258993	241	16	357	3398	1	3087	0	4074

TRINITY_DN15770_c0_g2_i1	Glyma.10G107700.1	96.50924025	241	0	224	3309	23	3108	0	4364

TRINITY_DN15770_c0_g2_i1	Glyma.02G186100.2	98.14814815	236	0	224	3309	23	3108	0	4392

TRINITY_DN15770_c0_g2_i1	Glyma.02G186100.1	98.14814815	236	0	224	3309	23	3108	0	4392

TRINITY_DN5433_c0_g1_i1	Glyma.17G038500.1	100	258	6	301	3384	3333	259	0	4196

TRINITY_DN13926_c0_g1_i1	Glyma.01G234600.1	98.91304348	252	24	239	3256	3081	1	0	3842

TRINITY_DN15847_c1_g2_i2	Glyma.08G302000.1	98.2300885	206	2	1	3079	209	3293	0	4518

TRINITY_DN15815_c0_g2_i4	Glyma.12G147000.1	95.34883721	271	18	1489	4545	3081	1	0	4000

TRINITY_DN15815_c0_g2_i4	Glyma.12G147000.3	96.44128114	269	21	1489	4545	3078	1	0	3991

TRINITY_DN16094_c0_g1_i3	Glyma.11G004100.1	97.82608696	193	5	3	3086	3177	113	0	4503

TRINITY_DN14659_c2_g1_i1	Glyma.04G255100.6	96.13733906	470	11	332	3409	3666	592	0	3005

TRINITY_DN14659_c2_g1_i1	Glyma.04G255100.4	96.13402062	470	11	332	3409	3666	592	0	3005

TRINITY_DN14659_c2_g1_i1	Glyma.04G255100.1	96.1352657	470	11	332	3409	3666	592	0	3005

TRINITY_DN14659_c2_g1_i1	Glyma.04G255100.5	96.81372549	470	11	332	3409	3621	547	0	3005

TRINITY_DN14659_c2_g1_i1	Glyma.04G255100.3	96.81372549	470	11	332	3409	3621	547	0	3005

TRINITY_DN14659_c2_g1_i1	Glyma.04G255100.2	96.1352657	470	11	332	3409	3621	547	0	3005

TRINITY_DN15225_c0_g1_i2	Glyma.18G287400.1	97.24137931	249	7	238	3320	3071	1	0	4237

TRINITY_DN15225_c0_g1_i2	Glyma.18G287400.2	97.5	249	7	238	3320	3071	1	0	4237

TRINITY_DN15576_c0_g2_i1	Glyma.07G062100.1	100	248	5	302	3375	3083	1	0	4261

TRINITY_DN16115_c0_g2_i2	Glyma.12G074300.1	97.2972973	236	4	230	3302	3129	48	0	4314

TRINITY_DN13939_c0_g1_i1	Glyma.19G226200.2	95.72192513	223	22	357	3398	1	3081	0	4180

TRINITY_DN13939_c0_g1_i1	Glyma.19G226200.1	95.72192513	223	22	357	3398	1	3081	0	4180

TRINITY_DN16031_c1_g2_i1	Glyma.17G037600.1	99.28571429	249	2	297	3377	3081	1	0	4300

TRINITY_DN13411_c0_g1_i1	Glyma.06G098300.5	100	225	5	2626	5701	5961	2881	0	4399

TRINITY_DN16314_c1_g2_i3	Glyma.10G223500.2	98.79518072	295	12	660	3730	3074	1	0	3945

TRINITY_DN16031_c1_g2_i1	Glyma.07G235800.1	99.28571429	237	0	297	3377	3081	1	0	4377

TRINITY_DN16314_c1_g2_i2	Glyma.10G223500.2	100	353	15	660	3712	3074	1	0	3518

TRINITY_DN16314_c1_g2_i1	Glyma.10G223500.2	100	286	5	660	3736	3074	1	0	4056

TRINITY_DN11658_c0_g1_i1	Glyma.10G288200.2	99.12917271	255	8	407	3475	3075	1	0	4183

TRINITY_DN11658_c0_g1_i1	Glyma.10G288200.1	100	255	8	407	3475	3075	1	0	4183

TRINITY_DN15913_c1_g2_i2	Glyma.19G103200.1	100	201	2	340	3415	3075	3	0	4514

TRINITY_DN14362_c0_g1_i1	Glyma.09G218500.1	100	374	37	60	3108	43	3087	0	3212

TRINITY_DN16314_c1_g2_i4	Glyma.10G223500.2	98.7804878	344	8	660	3718	3074	1	0	3629

TRINITY_DN15639_c1_g1_i1	Glyma.U022000.1	100	236	12	551	3610	3075	1	0	4241

TRINITY_DN15639_c1_g1_i2	Glyma.U022000.1	98.7654321	236	12	675	3734	3075	1	0	4241

TRINITY_DN15639_c1_g1_i3	Glyma.U022000.1	98.46153846	236	12	358	3417	3075	1	0	4241

TRINITY_DN15593_c0_g1_i1	Glyma.06G025400.2	98.7012987	265	7	35	3087	173	3243	0	4021

TRINITY_DN15593_c0_g1_i1	Glyma.06G025400.3	100	265	7	35	3087	173	3243	0	4021

TRINITY_DN15593_c0_g1_i1	Glyma.06G025400.1	100	265	7	35	3087	173	3243	0	4021

TRINITY_DN15593_c0_g1_i1	Glyma.06G025400.4	98.52941176	265	7	35	3087	173	3243	0	4021

TRINITY_DN15593_c0_g1_i1	Glyma.04G025400.2	97.84172662	238	7	35	3087	173	3237	0	4132

TRINITY_DN15593_c0_g1_i1	Glyma.04G025400.1	99.29577465	238	7	35	3087	173	3237	0	4132

TRINITY_DN15639_c1_g1_i1	Glyma.12G073200.4	100	229	10	551	3610	3069	1	0	4274

TRINITY_DN15639_c1_g1_i2	Glyma.12G073200.4	100	229	10	675	3734	3069	1	0	4274

TRINITY_DN15639_c1_g1_i3	Glyma.12G073200.4	98.46153846	229	10	358	3417	3069	1	0	4274

TRINITY_DN16254_c0_g1_i2	Glyma.07G004300.2	98.125	690	83	397	3420	3105	88	0	1181

TRINITY_DN16245_c0_g1_i2	Glyma.15G025800.2	98.93992933	380	24	980	4038	3056	1	0	3369

TRINITY_DN12915_c0_g1_i2	Glyma.10G136800.1	97.27272727	324	18	501	3563	3054	1	0	3692

TRINITY_DN12050_c0_g2_i3	Glyma.16G093800.21	96.56992084	526	53	351	3384	1	3004	0	2074

TRINITY_DN12050_c0_g2_i3	Glyma.16G093800.19	100	526	53	351	3384	1	3004	0	2074

TRINITY_DN12050_c0_g2_i2	Glyma.16G093800.21	98.48484848	507	53	336	3369	1	3004	0	2180

TRINITY_DN12050_c0_g2_i2	Glyma.16G093800.19	98.46547315	507	53	336	3369	1	3004	0	2180

TRINITY_DN15458_c0_g1_i2	Glyma.07G269000.1	95.49071618	453	38	403	3429	1	3054	0	2767

TRINITY_DN12915_c0_g1_i2	Glyma.20G086400.1	97.27272727	302	19	501	3563	3054	1	0	3836

TRINITY_DN16245_c0_g1_i4	Glyma.15G025800.2	98.05352798	327	21	898	3953	3056	1	0	3677

TRINITY_DN11658_c0_g1_i1	Glyma.20G100800.1	99.63898917	251	8	407	3466	3084	19	0	4189

TRINITY_DN11658_c0_g1_i1	Glyma.20G100800.2	97.82608696	251	8	407	3466	3084	19	0	4189

TRINITY_DN15183_c0_g1_i3	Glyma.13G320200.1	99.2	160	4	455	3517	3063	1	0	4704

TRINITY_DN15183_c0_g1_i3	Glyma.12G180600.1	98.47328244	143	5	455	3517	3063	1	0	4798

TRINITY_DN6023_c0_g1_i1	Glyma.10G255700.3	98.07692308	203	9	162	3212	1	3054	0	4362

TRINITY_DN16254_c0_g1_i3	Glyma.07G004300.2	95.29411765	709	74	397	3420	3105	88	0	1164

TRINITY_DN16151_c0_g2_i1	Glyma.01G038300.2	96.26865672	355	30	253	3272	1	3050	0	3317

TRINITY_DN12634_c0_g2_i1	Glyma.04G220400.1	98.21428571	287	7	204	3261	3120	60	0	4010

TRINITY_DN15815_c0_g2_i4	Glyma.06G254500.1	100	283	5	1489	4545	3063	1	0	4039

TRINITY_DN16026_c0_g2_i2	Glyma.11G112700.2	95.0310559	243	12	316	3369	1	3048	0	4167

TRINITY_DN6023_c0_g1_i1	Glyma.10G255700.1	98.08306709	203	9	162	3208	1	3050	0	4355

TRINITY_DN11843_c0_g1_i1	Glyma.03G220700.2	100	181	1	135	3194	1	3057	0	4628

TRINITY_DN13807_c0_g2_i1	Glyma.04G045400.1	100	172	4	166	3213	1	3060	0	4621

TRINITY_DN10035_c0_g2_i1	Glyma.10G013500.5	100	219	12	289	3330	21	3077	0	4313

TRINITY_DN16233_c0_g2_i2	Glyma.14G196800.1	96	372	4	408	3463	3401	349	0	3541

TRINITY_DN16233_c0_g2_i2	Glyma.14G196800.2	98.95833333	372	4	408	3463	3284	232	0	3541

TRINITY_DN16233_c0_g2_i2	Glyma.14G196800.3	98.94736842	372	4	408	3463	3284	232	0	3541

TRINITY_DN16233_c0_g2_i1	Glyma.11G166100.1	96.94656489	338	2	237	3293	3285	229	0	3762

TRINITY_DN16233_c0_g2_i1	Glyma.18G058900.1	100	309	2	237	3293	3339	283	0	3923

TRINITY_DN16233_c0_g2_i3	Glyma.14G196800.1	99.07407407	288	4	238	3293	3401	349	0	4006

TRINITY_DN16233_c0_g2_i3	Glyma.14G196800.2	100	288	4	238	3293	3284	232	0	4006

TRINITY_DN16233_c0_g2_i3	Glyma.14G196800.3	95.65217391	288	4	238	3293	3284	232	0	4006

TRINITY_DN16233_c0_g2_i4	Glyma.11G166100.1	96.44970414	267	2	407	3463	3285	229	0	4156

TRINITY_DN16233_c0_g2_i4	Glyma.18G058900.1	96.90346084	240	2	407	3463	3339	283	0	4305

TRINITY_DN13807_c0_g2_i1	Glyma.06G046000.1	97.36070381	175	4	166	3211	1	3058	0	4601

TRINITY_DN16254_c0_g1_i2	Glyma.17G057800.1	95.12195122	447	23	406	3438	3186	147	0	2904

TRINITY_DN16233_c0_g2_i2	Glyma.02G229700.1	95.91836735	365	1	407	3463	3279	226	0	3603

TRINITY_DN16233_c0_g2_i2	Glyma.02G229700.2	95.78947368	365	1	407	3463	3279	226	0	3603

TRINITY_DN14150_c0_g1_i1	Glyma.12G006300.1	96.62921348	352	14	206	3239	3052	1	0	3515

TRINITY_DN16233_c0_g2_i3	Glyma.02G229700.1	95.3125	293	1	237	3293	3279	226	0	4002

TRINITY_DN16233_c0_g2_i3	Glyma.02G229700.2	95.3125	293	1	237	3293	3279	226	0	4002

TRINITY_DN15025_c0_g1_i1	Glyma.15G253000.1	100	212	6	367	3408	1	3048	0	4316

TRINITY_DN15458_c0_g1_i2	Glyma.15G121500.1	95.77464789	209	0	403	3459	1	3057	0	4488

TRINITY_DN15458_c0_g1_i2	Glyma.09G015700.1	98.96907216	208	0	403	3458	1	3056	0	4492

TRINITY_DN16151_c0_g2_i4	Glyma.01G038300.2	100	165	3	318	3367	1	3050	0	4652

TRINITY_DN16151_c0_g2_i3	Glyma.01G038300.1	100	350	30	70	3080	82	3122	0	3328

TRINITY_DN15515_c0_g2_i1	Glyma.02G170900.1	100	291	7	501	3542	355	3405	0	3930

TRINITY_DN15515_c0_g2_i1	Glyma.09G109000.1	100	274	8	501	3542	724	3771	0	4006

TRINITY_DN15515_c0_g2_i1	Glyma.09G109000.2	100	274	8	501	3542	592	3639	0	4006

TRINITY_DN14517_c0_g1_i1	Glyma.12G027500.2	100	199	0	198	3251	1	3054	0	4538

TRINITY_DN14517_c0_g1_i1	Glyma.11G101600.2	99.82300885	197	2	198	3251	1	3051	0	4529

TRINITY_DN16254_c0_g1_i3	Glyma.17G057800.1	99.45054945	419	14	406	3438	3186	147	0	3103

TRINITY_DN15163_c0_g1_i1	Glyma.20G006400.1	97.77777778	265	44	68	3070	1	3030	0	3698

TRINITY_DN13347_c0_g1_i5	Glyma.09G206300.1	100	166	2	114	3163	689	3738	0	4680

TRINITY_DN13347_c0_g1_i5	Glyma.01G016500.2	98.96907216	157	1	114	3163	689	3741	0	4748

TRINITY_DN13347_c0_g1_i5	Glyma.01G016500.1	98.96907216	157	1	114	3163	689	3741	0	4748

TRINITY_DN10640_c0_g1_i1	Glyma.02G008600.1	95.1048951	192	6	259	3306	3033	1	0	4423

TRINITY_DN10640_c0_g1_i3	Glyma.02G008600.1	96.05633803	192	6	259	3306	3033	1	0	4423

TRINITY_DN16131_c0_g2_i2	Glyma.10G041700.4	95.83333333	340	12	1423	4461	31	3078	0	3653

TRINITY_DN14908_c0_g1_i2	Glyma.11G183800.5	95.90909091	224	9	1405	4439	3037	1	0	4200

TRINITY_DN14908_c0_g1_i2	Glyma.11G183800.6	95.91836735	224	9	1405	4439	3037	1	0	4200

TRINITY_DN15981_c0_g1_i19	Glyma.11G048300.1	100	192	3	427	3471	3051	1	0	4532

TRINITY_DN15981_c0_g1_i19	Glyma.17G161300.1	100	489	12	427	3468	3045	1	0	2839

TRINITY_DN15981_c0_g1_i19	Glyma.05G105200.1	100	475	11	427	3468	3045	1	0	2916

TRINITY_DN12634_c0_g2_i1	Glyma.06G145500.1	98.24561404	303	5	204	3248	3120	73	0	3908

TRINITY_DN15025_c0_g1_i1	Glyma.08G174200.1	100	228	9	367	3408	1	3033	0	4229

TRINITY_DN16026_c0_g2_i7	Glyma.11G112700.2	97.91666667	195	2	316	3357	1	3048	0	4510

TRINITY_DN16026_c0_g2_i8	Glyma.11G112700.2	100	195	2	316	3357	1	3048	0	4510

TRINITY_DN12591_c0_g1_i1	Glyma.16G084200.1	97.31707317	189	11	343	3375	3042	1	0	4446

TRINITY_DN12591_c0_g1_i2	Glyma.16G084200.1	95.3125	189	11	315	3347	3042	1	0	4446

TRINITY_DN16254_c0_g1_i2	Glyma.17G057800.4	98.66666667	447	23	406	3438	3174	147	0	2948

TRINITY_DN16254_c0_g1_i2	Glyma.17G057800.3	96.10894942	447	23	406	3438	3174	147	0	2948

TRINITY_DN16254_c0_g1_i2	Glyma.17G057800.2	95.12195122	447	23	406	3438	3174	147	0	2948

TRINITY_DN16254_c0_g1_i2	Glyma.13G101700.1	97.14285714	445	19	405	3438	3175	147	0	2972

TRINITY_DN10648_c0_g1_i2	Glyma.20G090000.2	100	283	6	468	3510	3037	1	0	3980

TRINITY_DN12591_c0_g1_i1	Glyma.03G089000.1	97.45762712	204	7	343	3375	3042	1	0	4385

TRINITY_DN12591_c0_g1_i2	Glyma.03G089000.1	97.4789916	204	7	315	3347	3042	1	0	4385

TRINITY_DN16254_c0_g1_i6	Glyma.15G167500.1	99.74587039	291	5	328	3372	3249	214	0	3952

TRINITY_DN16254_c0_g1_i6	Glyma.15G167500.3	99.74619289	291	5	328	3372	3249	214	0	3952

TRINITY_DN16254_c0_g1_i6	Glyma.15G167500.2	99.16666667	291	5	328	3372	3249	214	0	3952

TRINITY_DN16254_c0_g1_i6	Glyma.15G167500.4	99.16666667	291	5	328	3372	3249	214	0	3952

TRINITY_DN15981_c0_g1_i19	Glyma.01G193600.1	95.34883721	196	1	427	3468	3045	1	0	4518

TRINITY_DN16039_c0_g1_i5	Glyma.09G066000.2	95.91836735	196	0	124	3167	1	3044	0	4536

TRINITY_DN16254_c0_g1_i3	Glyma.13G101700.1	95.72301426	416	14	405	3438	3175	147	0	3177

TRINITY_DN13366_c0_g1_i1	Glyma.06G023600.2	98.7804878	293	16	490	3526	3009	1	0	3749

TRINITY_DN13366_c0_g1_i1	Glyma.06G023600.1	100	293	16	490	3526	3009	1	0	3749

TRINITY_DN5601_c0_g1_i1	Glyma.18G282300.4	96	292	13	8	3022	766	3783	0	3670

TRINITY_DN5601_c0_g1_i1	Glyma.18G282300.1	96.86028257	292	13	8	3022	766	3783	0	3670

TRINITY_DN14460_c0_g2_i1	Glyma.04G153700.2	100	136	0	53	3094	1	3042	0	4865

TRINITY_DN16254_c0_g1_i3	Glyma.17G057800.4	98.56850716	420	13	406	3438	3174	147	0	3153

TRINITY_DN16254_c0_g1_i3	Glyma.17G057800.3	96.22641509	420	13	406	3438	3174	147	0	3153

TRINITY_DN16254_c0_g1_i3	Glyma.17G057800.2	99.45054945	420	13	406	3438	3174	147	0	3153

TRINITY_DN12325_c0_g1_i2	Glyma.16G210500.1	97.22222222	313	22	117	3144	1	3018	0	3661

TRINITY_DN15994_c0_g1_i1	Glyma.13G367300.5	95.29411765	285	7	888	3916	3032	1	0	3923

TRINITY_DN12914_c0_g1_i1	Glyma.06G164500.1	98.52941176	255	5	208	3240	3039	1	0	4161

TRINITY_DN12914_c0_g1_i1	Glyma.04G200900.1	95.65217391	236	5	208	3240	3036	1	0	4246

TRINITY_DN16274_c0_g1_i1	Glyma.12G095300.1	95.80838323	176	24	503	3514	1	3018	0	4327

TRINITY_DN10705_c0_g1_i1	Glyma.17G244600.4	100	152	0	204	3242	1	3039	0	4771

TRINITY_DN10705_c0_g1_i1	Glyma.17G244600.1	100	152	0	204	3242	1	3039	0	4771

TRINITY_DN10705_c0_g1_i1	Glyma.17G244600.5	100	152	0	204	3242	1	3039	0	4771

TRINITY_DN10705_c0_g1_i1	Glyma.14G080800.5	100	145	0	204	3242	1	3039	0	4809

TRINITY_DN10705_c0_g1_i1	Glyma.14G080800.1	100	145	0	204	3242	1	3039	0	4809

TRINITY_DN10705_c0_g1_i1	Glyma.14G080800.4	100	145	0	204	3242	1	3039	0	4809

TRINITY_DN10705_c0_g1_i1	Glyma.14G080800.3	100	145	0	204	3242	1	3039	0	4809

TRINITY_DN10705_c0_g1_i1	Glyma.14G080800.2	100	145	0	204	3242	1	3039	0	4809

TRINITY_DN15148_c0_g1_i1	Glyma.16G012100.1	98.19277108	252	24	1212	4220	3027	7	0	3917

TRINITY_DN15148_c0_g1_i2	Glyma.16G012100.1	98.42519685	252	24	1336	4344	3027	7	0	3917

TRINITY_DN10648_c0_g1_i1	Glyma.20G090000.2	96.8503937	214	2	468	3504	3037	1	0	4412

TRINITY_DN15994_c0_g1_i1	Glyma.15G006100.1	100	308	20	893	3916	3021	1	0	3714

TRINITY_DN15994_c0_g1_i1	Glyma.15G006100.2	99.63898917	308	20	893	3916	3021	1	0	3714

TRINITY_DN16274_c0_g1_i1	Glyma.12G189500.1	95.63758389	287	15	503	3514	1	3024	0	3773

TRINITY_DN16389_c1_g1_i1	Glyma.20G144400.1	95.18716578	287	39	1806	4764	4380	1383	0	3262

TRINITY_DN15148_c0_g1_i1	Glyma.07G043700.1	100	241	27	1216	4212	3029	15	0	3873

TRINITY_DN15148_c0_g1_i2	Glyma.07G043700.1	96.46017699	241	27	1340	4336	3029	15	0	3873

TRINITY_DN16262_c0_g1_i1	Glyma.04G213400.4	97.19626168	384	29	53	3082	124	3114	0	3149

TRINITY_DN16262_c0_g1_i1	Glyma.04G213400.3	100	382	31	53	3082	124	3111	0	3142

TRINITY_DN12325_c0_g1_i2	Glyma.09G161100.1	97.22222222	302	19	117	3144	1	3015	0	3746

TRINITY_DN16168_c0_g1_i6	Glyma.08G057200.2	95.23809524	230	10	524	3558	3282	275	0	4145

TRINITY_DN16168_c0_g1_i6	Glyma.08G057200.3	100	230	10	524	3558	3282	275	0	4145

TRINITY_DN16168_c0_g1_i2	Glyma.08G057200.2	96.85534591	211	10	620	3654	3282	275	0	4250

TRINITY_DN16168_c0_g1_i2	Glyma.08G057200.3	96.66666667	211	10	620	3654	3282	275	0	4250

TRINITY_DN15994_c0_g1_i2	Glyma.13G367300.5	97	192	5	827	3862	3027	1	0	4484

TRINITY_DN13959_c0_g1_i4	Glyma.10G292300.1	99.23664122	184	29	207	3188	3029	1	0	4193

TRINITY_DN16262_c0_g1_i1	Glyma.06G152800.2	96.875	380	31	53	3082	124	3102	0	3105

TRINITY_DN16031_c1_g2_i3	Glyma.17G037600.1	98.58490566	385	20	89	3111	3008	1	0	3230

TRINITY_DN15994_c0_g1_i1	Glyma.13G367300.4	98.49056604	285	7	893	3916	3027	1	0	3914

TRINITY_DN15994_c0_g1_i1	Glyma.13G367300.1	96.73913043	285	7	893	3916	3027	1	0	3914

TRINITY_DN15994_c0_g1_i3	Glyma.13G367300.5	99.55555556	285	7	827	3850	3027	1	0	3914

TRINITY_DN16031_c1_g2_i3	Glyma.07G235800.1	98.95833333	376	20	89	3111	3008	1	0	3291

TRINITY_DN5601_c0_g1_i1	Glyma.18G282300.3	95.67567568	292	12	8	3022	766	3774	0	3712

TRINITY_DN5601_c0_g1_i1	Glyma.18G282300.2	95.67567568	292	12	8	3022	766	3774	0	3712

TRINITY_DN13366_c0_g1_i1	Glyma.04G023600.1	96.66666667	291	12	497	3526	2997	1	0	3736

TRINITY_DN3166_c0_g1_i1	Glyma.02G007700.1	99.42363112	185	6	209	3237	1	3023	0	4486

TRINITY_DN15994_c0_g1_i1	Glyma.08G214600.5	98.55072464	143	1	893	3916	3033	1	0	4750

TRINITY_DN15994_c0_g1_i1	Glyma.08G214600.7	99.0990991	143	1	893	3916	3033	1	0	4750

TRINITY_DN15994_c0_g1_i1	Glyma.08G214600.6	97.13774597	143	1	893	3916	3033	1	0	4750

TRINITY_DN15994_c0_g1_i1	Glyma.08G214600.4	98.5915493	143	1	893	3916	3033	1	0	4750

TRINITY_DN15994_c0_g1_i1	Glyma.08G214600.2	98.75	143	1	893	3916	3033	1	0	4750

TRINITY_DN15994_c0_g1_i1	Glyma.08G214600.1	100	143	1	893	3916	3033	1	0	4750

TRINITY_DN15994_c0_g1_i1	Glyma.08G214600.3	98.64253394	143	1	893	3916	3033	1	0	4750

TRINITY_DN15994_c0_g1_i1	Glyma.07G028000.1	100	142	8	893	3916	3021	1	0	4678

TRINITY_DN16262_c0_g1_i9	Glyma.06G152800.2	96.92307692	301	30	53	3079	124	3102	0	3557

TRINITY_DN15732_c0_g1_i1	Glyma.03G012600.1	100	270	9	321	3347	3012	1	0	3941

TRINITY_DN15732_c0_g1_i2	Glyma.03G012600.1	100	270	9	321	3347	3012	1	0	3941

TRINITY_DN15295_c0_g1_i1	Glyma.10G218400.3	100	237	14	2034	5044	3020	1	0	4072

TRINITY_DN15295_c0_g1_i1	Glyma.10G218400.1	95.55555556	237	14	2034	5044	3020	1	0	4072

TRINITY_DN15295_c0_g1_i1	Glyma.10G218400.4	100	237	14	2034	5044	3020	1	0	4072

TRINITY_DN15295_c0_g1_i1	Glyma.10G218400.2	100	237	14	2034	5044	3020	1	0	4072

TRINITY_DN15295_c0_g1_i2	Glyma.10G218400.3	98.80952381	237	14	2034	5044	3020	1	0	4072

TRINITY_DN15295_c0_g1_i2	Glyma.10G218400.1	96.06060606	237	14	2034	5044	3020	1	0	4072

TRINITY_DN15295_c0_g1_i2	Glyma.10G218400.4	98.66666667	237	14	2034	5044	3020	1	0	4072

TRINITY_DN15295_c0_g1_i2	Glyma.10G218400.2	99.13043478	237	14	2034	5044	3020	1	0	4072

TRINITY_DN10917_c1_g1_i1	Glyma.04G192200.3	98.33333333	213	5	213	3237	14	3041	0	4348

TRINITY_DN10917_c1_g1_i1	Glyma.04G192200.1	98.30508475	213	5	213	3237	14	3041	0	4348

TRINITY_DN10917_c1_g1_i1	Glyma.04G192200.4	99.74683544	213	5	213	3237	14	3041	0	4348

TRINITY_DN10917_c1_g1_i1	Glyma.13G192400.1	98.33333333	196	5	213	3237	14	3041	0	4442

TRINITY_DN16262_c0_g1_i9	Glyma.04G213400.4	98.18181818	311	24	53	3079	124	3114	0	3591

TRINITY_DN16262_c0_g1_i9	Glyma.04G213400.3	96.92307692	309	26	53	3079	124	3111	0	3581

TRINITY_DN12282_c0_g1_i1	Glyma.18G284100.1	96.42857143	215	3	147	3176	3024	1	0	4355

TRINITY_DN3166_c0_g1_i1	Glyma.10G137800.1	95.23809524	182	9	209	3228	1	3020	0	4447

TRINITY_DN12282_c0_g1_i1	Glyma.02G097400.2	96.14243323	219	1	147	3176	3024	1	0	4344

TRINITY_DN12282_c0_g1_i1	Glyma.02G097400.1	96.45390071	219	1	147	3176	3024	1	0	4344

TRINITY_DN12724_c0_g1_i2	Glyma.05G241500.1	97.97297297	173	3	176	3205	1	3021	0	4578

TRINITY_DN12724_c0_g1_i2	Glyma.08G048800.1	96.62921348	165	3	176	3205	1	3021	0	4623

TRINITY_DN16245_c0_g5_i1	Glyma.16G099700.1	96.57534247	694	72	594	3565	3350	352	0	1186

TRINITY_DN13931_c0_g1_i1	Glyma.03G188900.1	98.85057471	184	2	912	3939	3525	498	0	4562

TRINITY_DN16031_c1_g2_i3	Glyma.15G137000.1	100	178	0	83	3111	3029	1	0	4608

TRINITY_DN16031_c1_g2_i3	Glyma.15G137000.2	95.04132231	173	3	83	3111	3020	1	0	4577

TRINITY_DN16345_c0_g1_i2	Glyma.08G044700.3	95.34450652	398	14	446	3465	3504	491	0	3245

TRINITY_DN16345_c0_g1_i5	Glyma.08G044700.3	98.4496124	398	14	446	3465	3504	491	0	3245

TRINITY_DN16345_c0_g1_i2	Glyma.08G044700.7	95.32467532	398	14	446	3465	3225	212	0	3245

TRINITY_DN16345_c0_g1_i5	Glyma.08G044700.7	98.4496124	398	14	446	3465	3225	212	0	3245

TRINITY_DN15732_c0_g1_i1	Glyma.07G072500.1	97.00598802	263	3	321	3347	3009	1	0	4017

TRINITY_DN15732_c0_g1_i2	Glyma.07G072500.1	97.04797048	263	3	321	3347	3009	1	0	4017

TRINITY_DN16274_c0_g1_i1	Glyma.12G095300.4	100	177	21	503	3514	1	3006	0	4377

TRINITY_DN16245_c0_g5_i2	Glyma.16G099700.1	95.13888889	689	69	594	3565	3350	352	0	1247

TRINITY_DN85_c0_g1_i1	Glyma.17G014900.2	99.35483871	261	15	1671	4669	3020	1	0	3928

TRINITY_DN85_c0_g1_i1	Glyma.17G014900.3	95.56786704	261	15	1671	4669	3020	1	0	3928

TRINITY_DN85_c0_g1_i1	Glyma.17G014900.4	97.87234043	261	15	1671	4669	3020	1	0	3928

TRINITY_DN85_c0_g1_i1	Glyma.17G014900.1	97.36070381	261	15	1671	4669	3020	1	0	3928

TRINITY_DN85_c0_g1_i2	Glyma.17G014900.2	100	261	15	1592	4590	3020	1	0	3928

TRINITY_DN85_c0_g1_i2	Glyma.17G014900.3	96.55172414	261	15	1592	4590	3020	1	0	3928

TRINITY_DN85_c0_g1_i2	Glyma.17G014900.4	99.05660377	261	15	1592	4590	3020	1	0	3928

TRINITY_DN85_c0_g1_i2	Glyma.17G014900.1	96.61016949	261	15	1592	4590	3020	1	0	3928

TRINITY_DN16274_c0_g1_i1	Glyma.12G189500.2	98.73417722	288	11	503	3514	1	3012	0	3823

TRINITY_DN13959_c0_g1_i4	Glyma.10G292300.2	99.23664122	185	21	207	3188	3017	1	0	4242

TRINITY_DN11030_c0_g2_i1	Glyma.12G220900.2	97.24770642	155	8	206	3229	1	2994	0	4532

TRINITY_DN11523_c0_g1_i1	Glyma.07G042700.3	98.24561404	263	36	1078	4029	3009	1	0	3576

TRINITY_DN11523_c0_g1_i2	Glyma.07G042700.3	100	263	36	1432	4383	3009	1	0	3576

TRINITY_DN9378_c0_g1_i1	Glyma.07G085500.1	97.62470309	206	16	327	3332	3084	89	0	4157

TRINITY_DN16345_c0_g1_i6	Glyma.08G044700.3	98.4375	293	6	446	3462	3504	491	0	3873

TRINITY_DN16345_c0_g1_i6	Glyma.08G044700.7	98.4496124	293	6	446	3462	3225	212	0	3873

TRINITY_DN15521_c0_g1_i1	Glyma.06G226700.1	100	281	9	134	3135	19	3038	0	3882

TRINITY_DN15521_c0_g1_i4	Glyma.06G226700.1	95.94095941	245	9	134	3135	19	3038	0	4082

TRINITY_DN14994_c0_g1_i2	Glyma.13G115500.1	97.94238683	189	2	189	3209	1	3021	0	4521

TRINITY_DN13882_c0_g2_i1	Glyma.08G310800.1	100	381	68	26	2930	778	3754	0	2435

TRINITY_DN14994_c0_g1_i2	Glyma.17G044300.1	97.91666667	191	0	189	3209	1	3021	0	4521

TRINITY_DN15521_c0_g1_i1	Glyma.12G155900.1	100	277	7	132	3135	17	3035	0	3943

TRINITY_DN15521_c0_g1_i4	Glyma.12G155900.1	100	235	7	132	3135	17	3035	0	4176

TRINITY_DN10760_c0_g1_i1	Glyma.01G053100.1	100	201	5	226	3238	3090	78	0	4385

TRINITY_DN16274_c0_g1_i1	Glyma.12G095300.3	95.80838323	176	24	503	3494	1	2998	0	4290

TRINITY_DN12050_c0_g2_i1	Glyma.03G081300.5	100	499	74	172	3147	3016	5928	0	1858

TRINITY_DN12050_c0_g2_i1	Glyma.03G081300.6	100	499	74	172	3147	3016	5928	0	1858

TRINITY_DN12050_c0_g2_i1	Glyma.03G081300.4	100	499	74	172	3147	3016	5928	0	1858

TRINITY_DN12050_c0_g2_i1	Glyma.03G081300.3	100	499	74	172	3147	3016	5928	0	1858

TRINITY_DN12050_c0_g2_i1	Glyma.03G081300.1	100	499	74	172	3147	3016	5928	0	1858

TRINITY_DN4758_c0_g1_i1	Glyma.11G085900.3	99.47916667	454	15	509	3518	2998	1	0	2889

TRINITY_DN15235_c0_g1_i4	Glyma.16G172500.4	100	514	17	1024	4032	1	3006	0	2612

TRINITY_DN15235_c0_g1_i4	Glyma.16G172500.3	96.15384615	514	17	1024	4032	1	3006	0	2612

TRINITY_DN15235_c0_g1_i4	Glyma.16G172500.2	97.5	514	17	1024	4032	1	3006	0	2612

TRINITY_DN16022_c0_g2_i2	Glyma.01G118900.1	100	340	23	27	3026	1	2976	0	3325

TRINITY_DN16274_c0_g1_i1	Glyma.13G312300.1	95.31981279	280	24	503	3514	1	2967	0	3675

TRINITY_DN85_c0_g1_i1	Glyma.07G259400.8	100	272	17	1671	4647	3026	17	0	3771

TRINITY_DN85_c0_g1_i1	Glyma.07G259400.1	95.71428571	272	17	1671	4647	3026	17	0	3771

TRINITY_DN85_c0_g1_i2	Glyma.07G259400.8	99.46236559	272	17	1592	4568	3026	17	0	3771

TRINITY_DN85_c0_g1_i2	Glyma.07G259400.1	97.48549323	272	17	1592	4568	3026	17	0	3771

TRINITY_DN15235_c0_g1_i2	Glyma.16G172500.4	98.92473118	515	17	1024	4031	1	3005	0	2604

TRINITY_DN15235_c0_g1_i2	Glyma.16G172500.3	95.19230769	515	17	1024	4031	1	3005	0	2604

TRINITY_DN15235_c0_g1_i2	Glyma.16G172500.2	98.91304348	515	17	1024	4031	1	3005	0	2604

TRINITY_DN14092_c0_g1_i1	Glyma.20G151300.1	100	279	12	122	3125	1	3002	0	3868

TRINITY_DN16345_c0_g1_i6	Glyma.07G093700.2	98.4375	245	6	642	3652	3527	517	0	4159

TRINITY_DN14877_c0_g1_i1	Glyma.13G067800.3	95.16129032	190	2	447	3455	3984	971	0	4470

TRINITY_DN10917_c1_g1_i1	Glyma.15G230300.1	98.33333333	190	4	227	3237	1	3014	0	4482

TRINITY_DN15914_c0_g2_i2	Glyma.14G044900.3	100	178	0	145	3159	3074	60	0	4582

TRINITY_DN12050_c0_g2_i1	Glyma.16G093800.38	100	487	75	172	3147	3043	5958	0	1989

TRINITY_DN12050_c0_g2_i1	Glyma.16G093800.1	100	487	75	172	3147	3043	5958	0	1989

TRINITY_DN12050_c0_g2_i1	Glyma.16G093800.33	100	487	75	172	3147	3028	5943	0	1989

TRINITY_DN12050_c0_g2_i1	Glyma.16G093800.34	100	487	75	172	3147	3028	5943	0	1989

TRINITY_DN12050_c0_g2_i1	Glyma.16G093800.7	100	487	75	172	3147	3028	5943	0	1989

TRINITY_DN12050_c0_g2_i1	Glyma.16G093800.31	100	487	75	172	3147	3025	5940	0	1989

TRINITY_DN12050_c0_g2_i1	Glyma.16G093800.5	100	487	75	172	3147	3025	5940	0	1989

TRINITY_DN12050_c0_g2_i1	Glyma.16G093800.36	100	482	80	172	3147	3043	5949	0	1958

TRINITY_DN12050_c0_g2_i1	Glyma.16G093800.15	100	482	80	172	3147	3043	5949	0	1958

TRINITY_DN12050_c0_g2_i1	Glyma.16G093800.28	100	482	80	172	3147	3028	5934	0	1958

TRINITY_DN12050_c0_g2_i1	Glyma.16G093800.29	100	482	80	172	3147	3028	5934	0	1958

TRINITY_DN12050_c0_g2_i1	Glyma.16G093800.13	100	482	80	172	3147	3028	5934	0	1958

TRINITY_DN12050_c0_g2_i1	Glyma.16G093800.25	100	482	80	172	3147	3025	5931	0	1958

TRINITY_DN12050_c0_g2_i1	Glyma.16G093800.18	100	482	80	172	3147	3025	5931	0	1958

TRINITY_DN7713_c0_g1_i1	Glyma.13G370300.2	96.09195402	178	2	199	3209	956	3969	0	4560

TRINITY_DN15815_c0_g2_i8	Glyma.12G219400.1	99.32885906	334	13	472	3462	3585	577	0	3546

TRINITY_DN12050_c0_g2_i1	Glyma.08G352600.1	100	286	23	148	3147	3049	6024	0	3657

TRINITY_DN12050_c0_g2_i1	Glyma.08G352600.4	100	286	23	148	3147	3043	6018	0	3657

TRINITY_DN12050_c0_g2_i1	Glyma.08G352600.2	100	286	23	148	3147	3043	6018	0	3657

TRINITY_DN12050_c0_g2_i1	Glyma.08G352600.3	100	286	23	148	3147	2992	5967	0	3657

TRINITY_DN7713_c0_g1_i1	Glyma.13G370300.3	96.09375	178	2	199	3208	956	3968	0	4558

TRINITY_DN7713_c0_g1_i1	Glyma.13G370300.6	96.09375	178	2	199	3208	956	3968	0	4558

TRINITY_DN7713_c0_g1_i1	Glyma.13G370300.5	99.02912621	178	2	199	3208	956	3968	0	4558

TRINITY_DN7713_c0_g1_i1	Glyma.13G370300.4	97.01492537	178	2	199	3208	956	3968	0	4558

TRINITY_DN15410_c0_g2_i2	Glyma.10G146900.1	100	200	10	416	3419	3000	3	0	4313

TRINITY_DN10760_c0_g1_i1	Glyma.02G111600.1	100	198	2	226	3234	3096	88	0	4427

TRINITY_DN12050_c0_g2_i1	Glyma.16G093800.37	100	488	73	172	3147	3043	5955	0	1997

TRINITY_DN12050_c0_g2_i1	Glyma.16G093800.16	100	488	73	172	3147	3043	5955	0	1997

TRINITY_DN12050_c0_g2_i1	Glyma.16G093800.32	99.2481203	488	73	172	3147	3028	5940	0	1997

TRINITY_DN12050_c0_g2_i1	Glyma.16G093800.41	100	488	73	172	3147	3028	5940	0	1997

TRINITY_DN12050_c0_g2_i1	Glyma.16G093800.6	100	488	73	172	3147	3028	5940	0	1997

TRINITY_DN12050_c0_g2_i1	Glyma.16G093800.39	100	488	73	172	3147	3028	5940	0	1997

TRINITY_DN12050_c0_g2_i1	Glyma.16G093800.2	100	488	73	172	3147	3028	5940	0	1997

TRINITY_DN12050_c0_g2_i1	Glyma.16G093800.30	100	488	73	172	3147	3025	5937	0	1997

TRINITY_DN12050_c0_g2_i1	Glyma.16G093800.4	99.2481203	488	73	172	3147	3025	5937	0	1997

TRINITY_DN12050_c0_g2_i1	Glyma.16G093800.35	99.27007299	483	78	172	3147	3043	5946	0	1967

TRINITY_DN12050_c0_g2_i1	Glyma.16G093800.14	100	483	78	172	3147	3043	5946	0	1967

TRINITY_DN12050_c0_g2_i1	Glyma.16G093800.26	100	483	78	172	3147	3028	5931	0	1967

TRINITY_DN12050_c0_g2_i1	Glyma.16G093800.27	100	483	78	172	3147	3028	5931	0	1967

TRINITY_DN12050_c0_g2_i1	Glyma.16G093800.12	100	483	78	172	3147	3028	5931	0	1967

TRINITY_DN12050_c0_g2_i1	Glyma.16G093800.24	100	483	78	172	3147	3025	5928	0	1967

TRINITY_DN12050_c0_g2_i1	Glyma.16G093800.17	100	483	78	172	3147	3025	5928	0	1967

TRINITY_DN9378_c0_g1_i1	Glyma.09G191400.1	100	200	17	327	3314	3096	99	0	4220

TRINITY_DN15815_c0_g2_i8	Glyma.12G219400.4	98.25327511	334	12	472	3462	3582	577	0	3559

TRINITY_DN8232_c0_g1_i2	Glyma.08G196100.1	95.55950266	311	13	599	3587	681	3684	0	3661

TRINITY_DN8232_c0_g1_i2	Glyma.08G196100.2	97.42813918	311	13	599	3587	108	3111	0	3661

TRINITY_DN15811_c0_g1_i2	Glyma.15G097000.1	98.92473118	219	8	225	3230	214	3219	0	4294

TRINITY_DN15815_c0_g2_i8	Glyma.13G281900.1	99.52606635	332	12	472	3462	3582	577	0	3581

TRINITY_DN15410_c0_g2_i2	Glyma.10G146900.2	99.39759036	200	9	416	3419	2997	3	0	4325

TRINITY_DN15410_c0_g2_i2	Glyma.10G146900.3	96.84210526	200	10	416	3415	2994	1	0	4305

TRINITY_DN16016_c0_g1_i1	Glyma.16G163200.1	97.91666667	400	19	708	3709	3231	245	0	3175

TRINITY_DN16016_c0_g1_i3	Glyma.16G163200.1	98.98167006	400	19	708	3709	3231	245	0	3175

TRINITY_DN8232_c0_g1_i2	Glyma.07G012400.1	95.18072289	319	13	599	3587	687	3687	0	3631

TRINITY_DN8232_c0_g1_i2	Glyma.07G012400.5	98.61111111	319	13	599	3587	108	3108	0	3631

TRINITY_DN8232_c0_g1_i2	Glyma.07G012400.4	97.36842105	319	13	599	3587	108	3108	0	3631

TRINITY_DN8232_c0_g1_i2	Glyma.07G012400.3	97.10144928	319	13	599	3587	108	3108	0	3631

TRINITY_DN16274_c0_g1_i1	Glyma.12G095300.2	100	177	21	503	3494	1	2986	0	4340

TRINITY_DN12822_c0_g1_i4	Glyma.08G102200.1	100	555	59	902	3876	2945	1	0	1882

TRINITY_DN12822_c0_g1_i4	Glyma.08G102200.2	100	555	59	902	3876	2945	1	0	1882

TRINITY_DN12822_c0_g1_i4	Glyma.08G102200.3	100	555	59	902	3876	2945	1	0	1882

TRINITY_DN12822_c0_g1_i4	Glyma.08G102200.4	100	555	59	902	3876	2945	1	0	1882

TRINITY_DN15980_c0_g2_i2	Glyma.20G207700.1	97.94871795	259	9	1022	4021	865	3849	0	3941

TRINITY_DN15068_c0_g1_i6	Glyma.04G080600.1	96.79300292	280	25	182	3155	2997	15	0	3655

TRINITY_DN15410_c0_g2_i2	Glyma.10G146900.4	97.43589744	200	9	416	3415	2991	1	0	4318

TRINITY_DN12822_c0_g1_i1	Glyma.08G102200.1	100	519	54	902	3879	2945	1	0	2122

TRINITY_DN12822_c0_g1_i1	Glyma.08G102200.2	100	519	54	902	3879	2945	1	0	2122

TRINITY_DN12822_c0_g1_i1	Glyma.08G102200.3	100	519	54	902	3879	2945	1	0	2122

TRINITY_DN12822_c0_g1_i1	Glyma.08G102200.4	100	519	54	902	3879	2945	1	0	2122

TRINITY_DN12658_c0_g1_i3	Glyma.05G206100.1	95.91836735	394	33	167	3152	7	2977	0	3035

TRINITY_DN12658_c0_g1_i4	Glyma.05G206100.1	100	394	33	167	3152	7	2977	0	3035

TRINITY_DN14150_c0_g1_i1	Glyma.09G230300.1	100	352	12	255	3239	3003	1	0	3469

TRINITY_DN38330_c0_g1_i1	Glyma.10G118100.2	98.31932773	248	9	321	3316	881	3876	0	4071

TRINITY_DN38330_c0_g1_i1	Glyma.10G118100.4	98.31932773	248	9	321	3316	209	3204	0	4071

TRINITY_DN3417_c0_g1_i1	Glyma.08G174300.2	100	193	16	124	3112	1	2998	0	4342

TRINITY_DN4758_c0_g1_i1	Glyma.16G116200.3	95.8974359	237	2	517	3518	3002	1	0	4220

TRINITY_DN4758_c0_g1_i1	Glyma.16G116200.2	96.44268775	237	2	517	3518	3002	1	0	4220

TRINITY_DN390_c0_g1_i1	Glyma.11G102100.1	100	230	7	7	2997	1	3000	0	4185

TRINITY_DN15068_c0_g1_i6	Glyma.06G082300.1	98.97959184	288	22	182	3155	3000	15	0	3675

TRINITY_DN15484_c0_g1_i3	Glyma.17G105100.1	99.50738916	279	36	303	3242	4167	1210	0	3325

TRINITY_DN16254_c0_g1_i6	Glyma.09G061200.4	95.90909091	273	6	375	3372	3220	223	0	3991

TRINITY_DN16254_c0_g1_i6	Glyma.09G061200.3	98.92473118	273	6	375	3372	3220	223	0	3991

TRINITY_DN16254_c0_g1_i6	Glyma.09G061200.1	98.92473118	273	6	375	3372	3220	223	0	3991

TRINITY_DN16254_c0_g1_i6	Glyma.09G061200.2	100	273	6	375	3372	3220	223	0	3991

TRINITY_DN16254_c0_g1_i6	Glyma.09G061200.5	95.19230769	273	6	375	3372	3220	223	0	3991

TRINITY_DN16428_c1_g1_i1	Glyma.18G210300.6	100	149	2	937	3937	3671	686	0	4619

TRINITY_DN16428_c1_g1_i1	Glyma.18G210300.5	100	149	2	937	3937	3350	365	0	4619

TRINITY_DN16428_c1_g1_i1	Glyma.18G210300.4	100	149	2	937	3937	3329	344	0	4619

TRINITY_DN11523_c0_g1_i1	Glyma.07G042700.4	97.81931464	263	36	1101	4029	2986	1	0	3533

TRINITY_DN11523_c0_g1_i1	Glyma.07G042700.5	97.17514124	263	36	1101	4029	2986	1	0	3533

TRINITY_DN11523_c0_g1_i2	Glyma.07G042700.4	98.83040936	263	36	1455	4383	2986	1	0	3533

TRINITY_DN11523_c0_g1_i2	Glyma.07G042700.5	98.83040936	263	36	1455	4383	2986	1	0	3533

TRINITY_DN12658_c0_g1_i3	Glyma.08G013000.1	100	387	30	167	3152	7	2974	0	3110

TRINITY_DN12658_c0_g1_i4	Glyma.08G013000.1	95.94594595	387	30	167	3152	7	2974	0	3110

TRINITY_DN15016_c0_g1_i2	Glyma.10G234100.1	98.8372093	201	20	295	3271	1	2974	0	4120

TRINITY_DN11500_c0_g1_i1	Glyma.18G202700.3	99.02912621	237	2	121	3118	1	2995	0	4204

TRINITY_DN11500_c0_g1_i2	Glyma.18G202700.3	96.22641509	237	3	121	3107	1	2995	0	4133

TRINITY_DN3417_c0_g1_i1	Glyma.15G252800.3	100	211	10	124	3114	1	2991	0	4289

TRINITY_DN16245_c0_g3_i4	Glyma.06G196400.1	98.71794872	170	5	378	3370	2993	1	0	4542

TRINITY_DN12658_c0_g1_i3	Glyma.01G218100.1	95.94594595	383	18	152	3131	1	2977	0	3184

TRINITY_DN12658_c0_g1_i4	Glyma.01G218100.1	95.94272076	383	18	152	3131	1	2977	0	3184

TRINITY_DN12658_c0_g1_i3	Glyma.11G025100.1	95.92391304	380	28	152	3131	1	2962	0	3105

TRINITY_DN12658_c0_g1_i3	Glyma.11G025100.2	95.93495935	380	28	152	3131	1	2962	0	3105

TRINITY_DN12658_c0_g1_i4	Glyma.11G025100.1	95.94594595	380	28	152	3131	1	2962	0	3105

TRINITY_DN12658_c0_g1_i4	Glyma.11G025100.2	95.91836735	380	28	152	3131	1	2962	0	3105

TRINITY_DN17615_c0_g1_i1	Glyma.08G123300.1	96.61016949	343	14	161	3154	1	2976	0	3491

TRINITY_DN17615_c0_g1_i1	Glyma.08G123300.2	96.61016949	343	14	161	3154	1	2976	0	3491

TRINITY_DN17615_c0_g1_i1	Glyma.08G123300.5	96.61354582	343	14	161	3154	1	2976	0	3491

TRINITY_DN17615_c0_g1_i1	Glyma.08G123300.4	100	343	14	161	3154	1	2976	0	3491

TRINITY_DN11650_c0_g1_i2	Glyma.08G143000.1	100	239	17	74	3053	1	2983	0	4032

TRINITY_DN11650_c0_g1_i2	Glyma.08G143000.2	98.07228916	239	17	74	3053	1	2983	0	4032

TRINITY_DN15016_c0_g1_i2	Glyma.20G160200.1	98.8372093	211	16	295	3271	1	2974	0	4109

TRINITY_DN15295_c0_g1_i1	Glyma.20G173200.1	97.5708502	214	16	2074	5044	2974	1	0	4065

TRINITY_DN15295_c0_g1_i1	Glyma.20G173200.2	99.45945946	214	16	2074	5044	2974	1	0	4065

TRINITY_DN15295_c0_g1_i1	Glyma.20G173200.3	99.46091644	214	16	2074	5044	2974	1	0	4065

TRINITY_DN15295_c0_g1_i2	Glyma.20G173200.1	97.58454106	214	16	2074	5044	2974	1	0	4065

TRINITY_DN15295_c0_g1_i2	Glyma.20G173200.2	99.29577465	214	16	2074	5044	2974	1	0	4065

TRINITY_DN15295_c0_g1_i2	Glyma.20G173200.3	99.54954955	214	16	2074	5044	2974	1	0	4065

TRINITY_DN13959_c0_g1_i4	Glyma.20G242100.1	99.23664122	196	11	235	3188	2990	1	0	4159

TRINITY_DN16245_c0_g3_i4	Glyma.06G196400.3	98.57142857	171	3	378	3370	2990	1	0	4549

TRINITY_DN15634_c2_g1_i3	Glyma.20G187400.2	98.14814815	284	4	1272	4262	2991	1	0	3928

TRINITY_DN14767_c0_g2_i1	Glyma.11G126400.1	100	281	8	352	3327	2991	1	0	3847

TRINITY_DN14767_c0_g2_i2	Glyma.11G126400.1	97.95918367	281	8	223	3198	2991	1	0	3847

TRINITY_DN14767_c0_g2_i3	Glyma.11G126400.1	100	281	8	366	3341	2991	1	0	3847

TRINITY_DN9796_c0_g1_i2	Glyma.11G035800.1	99.2248062	277	13	296	3268	3093	109	0	3812

TRINITY_DN12563_c0_g1_i1	Glyma.01G002100.2	100	255	11	268	3239	4	2994	0	3965

TRINITY_DN12563_c0_g1_i1	Glyma.01G002100.1	96.66666667	255	11	268	3239	4	2994	0	3965

TRINITY_DN11297_c0_g2_i1	Glyma.05G139600.1	100	219	26	100	3057	1	2973	0	3958

TRINITY_DN11422_c0_g1_i1	Glyma.04G187600.2	97.61904762	246	8	340	3321	55	3033	0	4013

TRINITY_DN11650_c0_g1_i2	Glyma.05G185000.4	95.83333333	241	8	74	3053	1	2986	0	4074

TRINITY_DN11650_c0_g1_i2	Glyma.05G185000.1	98.14814815	241	8	74	3053	1	2986	0	4074

TRINITY_DN15811_c0_g1_i2	Glyma.13G215900.1	98.92473118	214	8	243	3230	217	3204	0	4289

TRINITY_DN13959_c0_g1_i4	Glyma.20G242100.2	99.23664122	196	10	235	3188	2987	1	0	4172

TRINITY_DN3274_c0_g1_i1	Glyma.20G218600.1	97.91666667	177	6	419	3400	3430	440	0	4484

TRINITY_DN12325_c0_g1_i1	Glyma.16G078800.1	97.49430524	648	38	71	3028	71	3035	0	1563

TRINITY_DN12325_c0_g1_i2	Glyma.16G078800.1	95.34883721	644	38	208	3165	71	3035	0	1585

TRINITY_DN13640_c1_g2_i2	Glyma.09G255400.1	98.64864865	235	18	441	3374	2982	1	0	3807

TRINITY_DN13640_c1_g2_i3	Glyma.09G255400.1	97.98365123	235	18	441	3374	2982	1	0	3807

TRINITY_DN13640_c1_g2_i4	Glyma.09G255400.1	95.23809524	235	18	441	3374	2982	1	0	3807

TRINITY_DN13640_c1_g2_i5	Glyma.09G255400.1	97.6744186	235	18	441	3374	2982	1	0	3807

TRINITY_DN13640_c1_g2_i6	Glyma.09G255400.1	95.45454545	235	18	441	3374	2982	1	0	3807

TRINITY_DN16406_c0_g1_i2	Glyma.01G006600.1	98.50746269	206	0	89	3078	683	3672	0	4381

TRINITY_DN16406_c0_g1_i2	Glyma.01G006600.2	96.95121951	206	0	89	3078	56	3045	0	4381

TRINITY_DN16406_c0_g1_i2	Glyma.07G125200.2	100	199	0	89	3078	683	3672	0	4420

TRINITY_DN16406_c0_g1_i2	Glyma.07G125200.1	100	199	0	89	3078	683	3672	0	4420

TRINITY_DN10938_c0_g1_i1	Glyma.09G085300.1	100	209	7	158	3136	2985	1	0	4272

TRINITY_DN15674_c0_g1_i3	Glyma.02G258200.1	95.59193955	534	20	462	3430	5111	2131	0	2392

TRINITY_DN11297_c0_g2_i1	Glyma.08G094900.1	100	240	20	100	3057	1	2982	0	3956

TRINITY_DN13640_c1_g2_i2	Glyma.18G237600.1	99.44751381	229	24	441	3374	2976	1	0	3823

TRINITY_DN13640_c1_g2_i3	Glyma.18G237600.1	96.09756098	229	24	441	3374	2976	1	0	3823

TRINITY_DN13640_c1_g2_i4	Glyma.18G237600.1	98.38187702	229	24	441	3374	2976	1	0	3823

TRINITY_DN13640_c1_g2_i5	Glyma.18G237600.1	98.33333333	229	24	441	3374	2976	1	0	3823

TRINITY_DN13640_c1_g2_i6	Glyma.18G237600.1	100	229	24	441	3374	2976	1	0	3823

TRINITY_DN13640_c1_g2_i2	Glyma.09G255400.4	99.44751381	235	17	441	3374	2979	1	0	3819

TRINITY_DN13640_c1_g2_i3	Glyma.09G255400.4	98.66666667	235	17	441	3374	2979	1	0	3819

TRINITY_DN13640_c1_g2_i4	Glyma.09G255400.4	97.22222222	235	17	441	3374	2979	1	0	3819

TRINITY_DN13640_c1_g2_i5	Glyma.09G255400.4	98.96907216	235	17	441	3374	2979	1	0	3819

TRINITY_DN13640_c1_g2_i6	Glyma.09G255400.4	99.10714286	235	17	441	3374	2979	1	0	3819

TRINITY_DN13640_c1_g2_i2	Glyma.09G255400.3	98.64864865	233	18	441	3374	2976	1	0	3812

TRINITY_DN13640_c1_g2_i3	Glyma.09G255400.3	98.66666667	233	18	441	3374	2976	1	0	3812

TRINITY_DN13640_c1_g2_i4	Glyma.09G255400.3	98.71794872	233	18	441	3374	2976	1	0	3812

TRINITY_DN13640_c1_g2_i5	Glyma.09G255400.3	99.24242424	233	18	441	3374	2976	1	0	3812

TRINITY_DN13640_c1_g2_i6	Glyma.09G255400.3	99.10714286	233	18	441	3374	2976	1	0	3812

TRINITY_DN13101_c0_g1_i1	Glyma.14G112200.1	99.25373134	179	3	857	3840	617	3603	0	4505

TRINITY_DN13604_c0_g1_i4	Glyma.11G218000.2	97.46192893	168	2	139	3124	357	3342	0	4573

TRINITY_DN13101_c0_g1_i1	Glyma.17G215700.1	99.27536232	163	6	857	3840	617	3597	0	4554

TRINITY_DN13433_c0_g1_i1	Glyma.10G024500.9	100	315	9	2	2978	2990	21	0	3607

TRINITY_DN13433_c0_g1_i1	Glyma.10G024500.6	97.46136865	315	9	2	2978	2990	21	0	3607

TRINITY_DN13433_c0_g1_i1	Glyma.10G024500.4	100	315	9	2	2978	2990	21	0	3607

TRINITY_DN10938_c0_g1_i1	Glyma.15G193400.1	99.5215311	185	10	158	3136	2979	1	0	4399

TRINITY_DN13604_c0_g1_i4	Glyma.18G039000.1	99.08256881	169	0	139	3124	357	3342	0	4578

TRINITY_DN15521_c0_g1_i4	Glyma.13G266300.1	97.22530522	477	57	182	3119	82	3037	0	2379

TRINITY_DN16151_c0_g2_i7	Glyma.08G292900.1	96.2962963	446	38	1638	4592	1234	4179	0	2595

TRINITY_DN16151_c0_g2_i7	Glyma.08G292900.5	96.96969697	446	38	1638	4592	1234	4179	0	2595

TRINITY_DN16151_c0_g2_i7	Glyma.08G292900.6	95.72086899	446	38	1638	4592	1231	4176	0	2595

TRINITY_DN16151_c0_g2_i7	Glyma.08G292900.4	95.72086899	446	38	1638	4592	1231	4176	0	2595

TRINITY_DN16151_c0_g2_i7	Glyma.08G292900.2	98.31223629	446	38	1638	4592	1231	4176	0	2595

TRINITY_DN12658_c0_g1_i5	Glyma.01G218100.1	95.93345656	381	18	152	3120	1	2966	0	3175

TRINITY_DN12658_c0_g1_i5	Glyma.11G025100.1	95.93495935	377	28	152	3120	1	2951	0	3101

TRINITY_DN12658_c0_g1_i5	Glyma.11G025100.2	95.93495935	377	28	152	3120	1	2951	0	3101

TRINITY_DN15674_c0_g1_i3	Glyma.02G228400.1	95.72953737	282	7	452	3430	5109	2128	0	3892

TRINITY_DN13640_c1_g2_i2	Glyma.18G237600.2	99.44444444	229	23	441	3374	2973	1	0	3836

TRINITY_DN13640_c1_g2_i3	Glyma.18G237600.2	98.38187702	229	23	441	3374	2973	1	0	3836

TRINITY_DN13640_c1_g2_i4	Glyma.18G237600.2	95.52238806	229	23	441	3374	2973	1	0	3836

TRINITY_DN13640_c1_g2_i5	Glyma.18G237600.2	95.70200573	229	23	441	3374	2973	1	0	3836

TRINITY_DN13640_c1_g2_i6	Glyma.18G237600.2	100	229	23	441	3374	2973	1	0	3836

TRINITY_DN14953_c0_g3_i2	Glyma.04G123900.3	98.0861244	214	6	549	3530	2967	1	0	4191

TRINITY_DN3274_c0_g1_i1	Glyma.10G171700.1	97.93814433	173	5	419	3400	3301	320	0	4516

TRINITY_DN3274_c0_g1_i1	Glyma.10G171700.2	97.94238683	173	5	419	3400	3301	320	0	4516

TRINITY_DN15674_c0_g1_i3	Glyma.01G179300.1	95.72649573	138	2	446	3430	5109	2128	0	4728

TRINITY_DN15674_c0_g1_i3	Glyma.11G062900.1	97.25373134	137	2	446	3430	5109	2128	0	4734

TRINITY_DN16094_c0_g1_i1	Glyma.10G250500.1	98.30508475	291	5	282	3263	3219	244	0	3834

TRINITY_DN16094_c0_g1_i6	Glyma.10G250500.1	97.42990654	291	5	282	3263	3219	244	0	3834

TRINITY_DN13197_c0_g1_i1	Glyma.16G024900.1	98.19494585	255	24	473	3430	1	2964	0	3801

TRINITY_DN13197_c0_g1_i1	Glyma.16G024900.2	98.1981982	255	24	473	3430	1	2964	0	3801

TRINITY_DN15811_c0_g1_i1	Glyma.17G023600.1	98.92473118	307	12	331	3307	207	3180	0	3711

TRINITY_DN15811_c0_g1_i1	Glyma.07G250700.1	98.92473118	305	12	331	3307	204	3177	0	3722

TRINITY_DN16175_c0_g1_i1	Glyma.05G110400.1	96.42857143	385	8	283	3254	56	3027	0	3245

TRINITY_DN16175_c0_g1_i4	Glyma.05G110400.1	98.85714286	385	8	267	3238	56	3027	0	3245

TRINITY_DN16175_c0_g1_i5	Glyma.05G110400.1	98.68421053	385	8	283	3254	56	3027	0	3245

TRINITY_DN16175_c0_g1_i6	Glyma.05G110400.1	100	385	8	267	3238	56	3027	0	3245

TRINITY_DN16422_c0_g2_i1	Glyma.11G005300.1	98.24561404	289	11	1240	4210	831	3789	0	3687

TRINITY_DN16422_c0_g2_i2	Glyma.11G005300.1	98.7804878	289	11	1239	4209	831	3789	0	3687

TRINITY_DN12658_c0_g1_i1	Glyma.05G206100.1	100	250	9	291	3258	1	2977	0	3999

TRINITY_DN12658_c0_g1_i6	Glyma.05G206100.1	97.92899408	250	9	291	3258	1	2977	0	3999

TRINITY_DN5517_c0_g1_i1	Glyma.13G259700.4	96.34703196	207	12	153	3123	1	2973	0	4231

TRINITY_DN5517_c0_g1_i1	Glyma.13G259700.1	96.34703196	207	12	153	3123	1	2973	0	4231

TRINITY_DN5517_c0_g1_i4	Glyma.13G259700.4	95.67567568	207	12	230	3200	1	2973	0	4231

TRINITY_DN5517_c0_g1_i4	Glyma.13G259700.1	100	207	12	230	3200	1	2973	0	4231

TRINITY_DN5517_c0_g1_i1	Glyma.13G259700.3	97.93510324	207	13	153	3123	1	2970	0	4211

TRINITY_DN5517_c0_g1_i4	Glyma.13G259700.3	98.8372093	207	13	230	3200	1	2970	0	4211

TRINITY_DN14654_c0_g1_i2	Glyma.11G089400.2	97.41863075	206	5	1	2980	2968	1	0	4263

TRINITY_DN14654_c0_g1_i2	Glyma.11G089400.1	97.41935484	206	5	1	2980	2968	1	0	4263

TRINITY_DN14654_c0_g1_i2	Glyma.11G089400.3	97.41935484	206	5	1	2980	2968	1	0	4263

TRINITY_DN15521_c0_g1_i1	Glyma.13G266300.1	100	471	50	182	3119	82	3037	0	2457

TRINITY_DN16422_c0_g2_i4	Glyma.11G005300.1	98.33333333	269	11	1240	4210	831	3789	0	3808

TRINITY_DN16422_c0_g2_i5	Glyma.11G005300.1	100	269	11	1239	4209	831	3789	0	3808

TRINITY_DN12658_c0_g1_i1	Glyma.08G013000.1	100	236	9	291	3258	1	2974	0	4069

TRINITY_DN12658_c0_g1_i6	Glyma.08G013000.1	97.93459552	236	9	291	3258	1	2974	0	4069

TRINITY_DN9844_c0_g1_i1	Glyma.01G176000.1	99.43661972	203	8	189	3161	3048	73	0	4296

TRINITY_DN9844_c0_g1_i2	Glyma.01G176000.1	100	203	8	178	3150	3048	73	0	4296

TRINITY_DN9844_c0_g1_i4	Glyma.01G176000.1	97.05882353	203	8	181	3153	3048	73	0	4296

TRINITY_DN9844_c0_g1_i5	Glyma.01G176000.1	100	203	8	179	3151	3048	73	0	4296

TRINITY_DN9844_c0_g1_i6	Glyma.01G176000.1	96.70542636	203	8	177	3149	3048	73	0	4296

TRINITY_DN13807_c0_g2_i1	Glyma.01G193600.1	96.46017699	651	47	166	3111	1	2952	0	1496

TRINITY_DN16041_c0_g1_i2	Glyma.11G128000.1	95.34883721	474	32	380	3331	2961	1	0	2573

TRINITY_DN16041_c0_g1_i2	Glyma.11G128000.2	100	474	32	380	3331	2961	1	0	2573

TRINITY_DN16041_c0_g1_i4	Glyma.11G128000.1	95.18072289	474	32	153	3104	2961	1	0	2573

TRINITY_DN16041_c0_g1_i4	Glyma.11G128000.2	95.18072289	474	32	153	3104	2961	1	0	2573

TRINITY_DN16123_c0_g2_i2	Glyma.08G006400.1	96.11650485	421	12	746	3705	2978	1	0	3029

TRINITY_DN14654_c0_g1_i2	Glyma.01G155300.1	97.41602067	197	1	1	2980	2968	1	0	4335

TRINITY_DN15674_c0_g1_i3	Glyma.14G195400.1	95.72649573	280	6	452	3430	5100	2128	0	3912

TRINITY_DN13943_c0_g1_i1	Glyma.14G138300.1	96.76806084	235	6	43	3015	1	2976	0	4141

TRINITY_DN13943_c0_g1_i1	Glyma.14G138300.2	100	235	6	43	3015	1	2976	0	4141

TRINITY_DN13943_c0_g1_i1	Glyma.17G194400.1	98.91304348	230	2	43	3020	1	2978	0	4215

TRINITY_DN16245_c0_g3_i5	Glyma.04G166100.1	96.15384615	178	1	213	3191	2976	1	0	4495

TRINITY_DN12658_c0_g1_i1	Glyma.11G025100.1	100	511	50	297	3237	16	2962	0	2230

TRINITY_DN12658_c0_g1_i1	Glyma.11G025100.2	100	511	50	297	3237	16	2962	0	2230

TRINITY_DN12658_c0_g1_i6	Glyma.11G025100.1	97.9253112	511	50	297	3237	16	2962	0	2230

TRINITY_DN12658_c0_g1_i6	Glyma.11G025100.2	97.91666667	511	50	297	3237	16	2962	0	2230

TRINITY_DN16175_c0_g1_i1	Glyma.17G156300.1	100	398	1	283	3254	56	3033	0	3256

TRINITY_DN16175_c0_g1_i4	Glyma.17G156300.1	95.21276596	398	1	267	3238	56	3033	0	3256

TRINITY_DN16175_c0_g1_i5	Glyma.17G156300.1	98.48484848	398	1	283	3254	56	3033	0	3256

TRINITY_DN16175_c0_g1_i6	Glyma.17G156300.1	97.72727273	398	1	267	3238	56	3033	0	3256

TRINITY_DN16151_c0_g2_i7	Glyma.18G130400.3	95.07186858	432	28	1638	4592	1201	4137	0	2704

TRINITY_DN16151_c0_g2_i7	Glyma.18G130400.1	98.57142857	432	28	1638	4592	1201	4137	0	2704

TRINITY_DN16123_c0_g2_i1	Glyma.08G006400.1	96.2962963	287	8	195	3155	2976	1	0	3797

TRINITY_DN16123_c0_g2_i2	Glyma.04G203000.1	98.79518072	228	7	736	3705	2973	1	0	4163

TRINITY_DN16123_c0_g2_i2	Glyma.06G162500.2	97.4522293	208	5	736	3705	2973	1	0	4274

TRINITY_DN16123_c0_g2_i2	Glyma.06G162500.1	96.05263158	208	5	736	3705	2973	1	0	4274

TRINITY_DN16123_c0_g2_i2	Glyma.06G162500.3	96.11940299	208	5	736	3705	2973	1	0	4274

TRINITY_DN5517_c0_g1_i1	Glyma.15G248200.1	98.13084112	199	7	153	3123	1	2970	0	4311

TRINITY_DN5517_c0_g1_i1	Glyma.15G248200.8	98.13084112	199	7	153	3123	1	2970	0	4311

TRINITY_DN5517_c0_g1_i1	Glyma.15G248200.7	97.14285714	199	7	153	3123	1	2970	0	4311

TRINITY_DN5517_c0_g1_i1	Glyma.15G248200.6	98.91304348	199	7	153	3123	1	2970	0	4311

TRINITY_DN5517_c0_g1_i1	Glyma.15G248200.5	98.36956522	199	7	153	3123	1	2970	0	4311

TRINITY_DN5517_c0_g1_i1	Glyma.15G248200.3	97.5	199	7	153	3123	1	2970	0	4311

TRINITY_DN5517_c0_g1_i1	Glyma.15G248200.2	96.89922481	199	7	153	3123	1	2970	0	4311

TRINITY_DN5517_c0_g1_i1	Glyma.15G248200.9	98.6681465	199	7	153	3123	1	2970	0	4311

TRINITY_DN5517_c0_g1_i4	Glyma.15G248200.1	97.93814433	199	7	230	3200	1	2970	0	4311

TRINITY_DN5517_c0_g1_i4	Glyma.15G248200.8	97.93814433	199	7	230	3200	1	2970	0	4311

TRINITY_DN5517_c0_g1_i4	Glyma.15G248200.7	98.26086957	199	7	230	3200	1	2970	0	4311

TRINITY_DN5517_c0_g1_i4	Glyma.15G248200.6	96.52173913	199	7	230	3200	1	2970	0	4311

TRINITY_DN5517_c0_g1_i4	Glyma.15G248200.5	99.28057554	199	7	230	3200	1	2970	0	4311

TRINITY_DN5517_c0_g1_i4	Glyma.15G248200.3	95.63492063	199	7	230	3200	1	2970	0	4311

TRINITY_DN5517_c0_g1_i4	Glyma.15G248200.2	99.52830189	199	7	230	3200	1	2970	0	4311

TRINITY_DN5517_c0_g1_i4	Glyma.15G248200.9	98.37209302	199	7	230	3200	1	2970	0	4311

TRINITY_DN5517_c0_g1_i1	Glyma.15G248200.4	100	199	9	153	3123	1	2967	0	4292

TRINITY_DN5517_c0_g1_i4	Glyma.15G248200.4	98.23529412	199	9	230	3200	1	2967	0	4292

TRINITY_DN15674_c0_g1_i3	Glyma.14G058300.1	100	540	20	462	3430	5090	2131	0	2355

TRINITY_DN15674_c0_g1_i3	Glyma.11G231100.1	96.62921348	529	18	462	3430	5093	2131	0	2436

TRINITY_DN16041_c0_g1_i2	Glyma.12G052700.4	95.0617284	469	26	380	3331	2961	1	0	2645

TRINITY_DN16041_c0_g1_i2	Glyma.12G052700.1	100	469	26	380	3331	2961	1	0	2645

TRINITY_DN16041_c0_g1_i2	Glyma.12G052700.2	99.55654102	469	26	380	3331	2961	1	0	2645

TRINITY_DN16041_c0_g1_i2	Glyma.12G052700.3	96.47058824	469	26	380	3331	2961	1	0	2645

TRINITY_DN16041_c0_g1_i4	Glyma.12G052700.4	96.96969697	469	26	153	3104	2961	1	0	2645

TRINITY_DN16041_c0_g1_i4	Glyma.12G052700.1	95.29411765	469	26	153	3104	2961	1	0	2645

TRINITY_DN16041_c0_g1_i4	Glyma.12G052700.2	95.70957096	469	26	153	3104	2961	1	0	2645

TRINITY_DN16041_c0_g1_i4	Glyma.12G052700.3	95.71045576	469	26	153	3104	2961	1	0	2645

TRINITY_DN16151_c0_g2_i1	Glyma.02G026700.2	98.64864865	345	32	253	3175	1	2956	0	3112

TRINITY_DN16151_c0_g2_i1	Glyma.02G026700.1	100	345	32	253	3175	1	2956	0	3112

TRINITY_DN16151_c0_g2_i5	Glyma.02G026700.3	99.33774834	345	32	253	3175	1	2956	0	3112

TRINITY_DN16151_c0_g2_i2	Glyma.02G026700.2	100	215	10	1534	4491	1327	4293	0	4130

TRINITY_DN16151_c0_g2_i2	Glyma.02G026700.1	98.80952381	215	10	1534	4491	1327	4293	0	4130

TRINITY_DN16151_c0_g2_i6	Glyma.02G026700.2	100	215	10	1273	4230	1327	4293	0	4130

TRINITY_DN16151_c0_g2_i6	Glyma.02G026700.1	95.41984733	215	10	1273	4230	1327	4293	0	4130

TRINITY_DN16373_c3_g5_i6	Glyma.13G367700.1	98.05996473	446	45	20	2950	3969	1048	0	2396

TRINITY_DN16373_c3_g5_i8	Glyma.13G367700.1	98.03921569	446	45	20	2950	3969	1048	0	2396

TRINITY_DN16254_c0_g1_i6	Glyma.13G101700.1	99.16666667	369	13	405	3372	3175	214	0	3321

TRINITY_DN12563_c0_g1_i1	Glyma.07G130400.2	99.10714286	263	6	269	3239	50	3012	0	3934

TRINITY_DN12563_c0_g1_i1	Glyma.07G130400.1	100	263	6	269	3239	50	3012	0	3934

TRINITY_DN12563_c0_g1_i1	Glyma.07G130400.3	100	263	6	269	3239	41	3003	0	3934

TRINITY_DN15863_c0_g1_i2	Glyma.15G016700.2	98.80952381	225	7	274	3240	1	2967	0	4145

TRINITY_DN15863_c0_g1_i2	Glyma.15G016700.1	98.80952381	225	7	274	3240	1	2967	0	4145

TRINITY_DN16271_c0_g1_i1	Glyma.15G268800.5	100	638	91	2935	5852	2955	37	0	1240

TRINITY_DN16271_c0_g1_i1	Glyma.15G268800.4	100	638	91	2935	5852	2955	37	0	1240

TRINITY_DN16271_c0_g1_i1	Glyma.15G268800.3	100	638	91	2935	5852	2955	37	0	1240

TRINITY_DN16271_c0_g1_i1	Glyma.15G268800.1	96.52777778	638	91	2935	5852	2955	37	0	1240

TRINITY_DN16271_c0_g1_i1	Glyma.15G268800.2	99.6969697	638	91	2935	5852	2955	37	0	1240

TRINITY_DN16271_c0_g1_i1	Glyma.15G268800.6	100	638	91	2935	5852	2955	37	0	1240

TRINITY_DN9093_c0_g1_i1	Glyma.14G005800.1	96.47058824	210	4	200	3169	97	3069	0	4296

TRINITY_DN9093_c0_g1_i1	Glyma.14G005800.2	96.49122807	210	4	200	3169	97	3069	0	4296

TRINITY_DN9093_c0_g1_i1	Glyma.14G005800.3	96.47058824	210	4	200	3169	97	3069	0	4296

TRINITY_DN9093_c0_g1_i1	Glyma.14G005800.4	96.47058824	210	4	200	3169	97	3069	0	4296

TRINITY_DN9093_c0_g1_i1	Glyma.14G005800.5	96.47058824	210	4	200	3169	97	3069	0	4296

TRINITY_DN9093_c0_g1_i1	Glyma.14G005800.6	96.48241206	210	4	200	3169	97	3069	0	4296

TRINITY_DN9844_c0_g1_i1	Glyma.11G068300.1	96.1038961	201	4	189	3155	2970	1	0	4307

TRINITY_DN9844_c0_g1_i2	Glyma.11G068300.1	98.0952381	201	4	178	3144	2970	1	0	4307

TRINITY_DN9844_c0_g1_i4	Glyma.11G068300.1	97.05882353	201	4	181	3147	2970	1	0	4307

TRINITY_DN9844_c0_g1_i5	Glyma.11G068300.1	97.6635514	201	4	179	3145	2970	1	0	4307

TRINITY_DN9844_c0_g1_i6	Glyma.11G068300.1	98.30508475	201	4	177	3143	2970	1	0	4307

TRINITY_DN265_c0_g1_i1	Glyma.04G084300.1	97.46835443	182	10	42	2993	1	2961	0	4257

TRINITY_DN12196_c0_g1_i2	Glyma.12G205800.1	98.26589595	140	0	32	3005	351	3324	0	4717

TRINITY_DN12196_c0_g1_i2	Glyma.12G205800.2	98.26589595	140	0	32	3005	351	3324	0	4717

TRINITY_DN12658_c0_g1_i1	Glyma.01G218100.1	100	533	33	297	3237	16	2977	0	2259

TRINITY_DN12658_c0_g1_i6	Glyma.01G218100.1	97.91666667	533	33	297	3237	16	2977	0	2259

TRINITY_DN15515_c0_g2_i1	Glyma.02G170900.2	99.10714286	286	7	582	3542	1	2970	0	3808

TRINITY_DN15515_c0_g2_i2	Glyma.02G170900.2	98.63013699	286	7	185	3145	1	2970	0	3808

TRINITY_DN15515_c0_g2_i1	Glyma.09G109000.3	99.10714286	269	8	582	3542	1	2967	0	3884

TRINITY_DN15515_c0_g2_i2	Glyma.09G109000.3	96.96969697	269	8	185	3145	1	2967	0	3884

TRINITY_DN15863_c0_g1_i2	Glyma.13G356900.1	98.80952381	223	3	274	3240	1	2970	0	4196

TRINITY_DN15863_c0_g1_i2	Glyma.13G356900.2	98.80952381	223	3	274	3240	1	2970	0	4196

TRINITY_DN12721_c0_g1_i1	Glyma.15G152100.2	97.63779528	161	0	230	3202	2973	1	0	4599

TRINITY_DN12721_c0_g1_i1	Glyma.09G041400.2	96.71814672	160	0	230	3202	2973	1	0	4604

TRINITY_DN12721_c0_g1_i1	Glyma.09G041400.3	97.63779528	160	0	230	3202	2973	1	0	4604

TRINITY_DN12721_c0_g1_i2	Glyma.15G152100.2	96.22166247	159	1	230	3183	2973	1	0	4486

TRINITY_DN12721_c0_g1_i2	Glyma.09G041400.2	95.0617284	159	1	230	3183	2973	1	0	4486

TRINITY_DN12721_c0_g1_i2	Glyma.09G041400.3	95.06292352	159	1	230	3183	2973	1	0	4486

TRINITY_DN12658_c0_g1_i5	Glyma.05G206100.1	97.93814433	391	33	167	3120	7	2945	0	2992

TRINITY_DN16071_c0_g2_i4	Glyma.09G052000.1	98.61111111	224	11	89	3052	1	2959	0	4111

TRINITY_DN15847_c1_g2_i3	Glyma.18G119000.2	100	317	14	437	3394	249	3203	0	3542

TRINITY_DN16422_c0_g2_i6	Glyma.11G005300.1	98.63013699	283	11	1239	4198	831	3778	0	3699

TRINITY_DN16422_c0_g2_i7	Glyma.11G005300.1	100	283	11	1240	4199	831	3778	0	3699

TRINITY_DN15847_c1_g2_i4	Glyma.18G119000.2	96.46393211	239	14	131	3088	249	3203	0	3975

TRINITY_DN16385_c0_g1_i3	Glyma.16G056400.1	100	315	22	589	3522	3453	496	0	3430

TRINITY_DN15748_c0_g1_i2	Glyma.08G283700.3	97.32937685	165	1	929	3898	3794	828	0	4551

TRINITY_DN15748_c0_g1_i3	Glyma.08G283700.3	100	165	1	286	3255	3794	828	0	4551

TRINITY_DN16123_c0_g2_i2	Glyma.05G199100.1	98.79518072	406	9	746	3705	2966	1	0	3157

TRINITY_DN16041_c0_g1_i5	Glyma.12G052700.4	95.34883721	245	16	153	3104	2961	1	0	3964

TRINITY_DN16041_c0_g1_i5	Glyma.12G052700.1	95.34883721	245	16	153	3104	2961	1	0	3964

TRINITY_DN16041_c0_g1_i5	Glyma.12G052700.2	95.34883721	245	16	153	3104	2961	1	0	3964

TRINITY_DN16041_c0_g1_i5	Glyma.12G052700.3	97.43589744	245	16	153	3104	2961	1	0	3964

TRINITY_DN16041_c0_g1_i8	Glyma.12G052700.4	98.9010989	245	16	380	3331	2961	1	0	3964

TRINITY_DN16041_c0_g1_i8	Glyma.12G052700.1	96.68049793	245	16	380	3331	2961	1	0	3964

TRINITY_DN16041_c0_g1_i8	Glyma.12G052700.2	96.68049793	245	16	380	3331	2961	1	0	3964

TRINITY_DN16041_c0_g1_i8	Glyma.12G052700.3	100	245	16	380	3331	2961	1	0	3964

TRINITY_DN16041_c0_g1_i5	Glyma.11G128000.1	100	259	15	153	3104	2961	1	0	3897

TRINITY_DN16041_c0_g1_i5	Glyma.11G128000.2	99.08256881	259	15	153	3104	2961	1	0	3897

TRINITY_DN16041_c0_g1_i8	Glyma.11G128000.1	100	259	15	380	3331	2961	1	0	3897

TRINITY_DN16041_c0_g1_i8	Glyma.11G128000.2	96.56565657	259	15	380	3331	2961	1	0	3897

TRINITY_DN16071_c0_g2_i3	Glyma.09G052000.1	95.55555556	174	4	89	3043	1	2959	0	4375

TRINITY_DN16303_c0_g1_i3	Glyma.13G301100.4	98.79182156	165	4	152	3113	1	2965	0	4508

TRINITY_DN16303_c0_g1_i3	Glyma.13G301100.2	98.79518072	165	4	152	3113	1	2965	0	4508

TRINITY_DN16303_c0_g1_i5	Glyma.13G301100.4	100	165	4	152	3113	1	2965	0	4508

TRINITY_DN16303_c0_g1_i5	Glyma.13G301100.2	100	165	4	152	3113	1	2965	0	4508

TRINITY_DN12658_c0_g1_i2	Glyma.11G025100.1	95.92529711	508	50	297	3226	16	2951	0	2226

TRINITY_DN12658_c0_g1_i2	Glyma.11G025100.2	95.91836735	508	50	297	3226	16	2951	0	2226

TRINITY_DN15521_c0_g1_i4	Glyma.12G231800.1	95.54730983	496	45	182	3111	79	3023	0	2350

TRINITY_DN12658_c0_g1_i5	Glyma.08G013000.1	95.94594595	382	30	167	3120	7	2942	0	3079

TRINITY_DN16151_c0_g2_i1	Glyma.01G038300.1	96.26168224	348	30	253	3175	1	2953	0	3177

TRINITY_DN16151_c0_g2_i5	Glyma.01G038300.2	97.77070064	348	30	253	3175	1	2953	0	3177

TRINITY_DN16374_c1_g1_i5	Glyma.07G069400.2	100	242	22	1	2950	3114	6048	0	3821

TRINITY_DN11513_c0_g1_i1	Glyma.17G105700.1	95.83333333	200	3	601	3561	2967	1	0	4333

TRINITY_DN265_c0_g1_i1	Glyma.06G086000.1	100	186	8	40	2993	68	3021	0	4281

TRINITY_DN16054_c1_g1_i1	Glyma.10G168700.1	95.53264605	154	0	375	3341	2967	1	0	4626

TRINITY_DN16054_c1_g1_i1	Glyma.20G221000.1	96.2962963	146	0	375	3341	2967	1	0	4671

TRINITY_DN16123_c0_g2_i1	Glyma.05G199100.1	100	279	5	195	3155	2964	1	0	3886

TRINITY_DN12325_c0_g1_i1	Glyma.16G078900.1	96.72131148	601	45	64	2996	88	3022	0	1746

TRINITY_DN12325_c0_g1_i1	Glyma.01G007500.1	95.09202454	599	38	59	2996	77	3013	0	1801

TRINITY_DN12526_c0_g1_i1	Glyma.15G075400.1	95.65217391	263	14	1	2955	129	3071	0	3812

TRINITY_DN12526_c0_g1_i1	Glyma.15G075400.2	95.88607595	263	14	1	2955	63	3005	0	3812

TRINITY_DN12526_c0_g1_i1	Glyma.15G075400.3	95.65217391	263	14	1	2955	63	3005	0	3812

TRINITY_DN13497_c0_g1_i1	Glyma.15G085900.2	98.67172676	188	0	508	3472	1	2965	0	4434

TRINITY_DN16303_c0_g1_i3	Glyma.12G201100.1	98.78787879	184	6	152	3113	1	2938	0	4263

TRINITY_DN16303_c0_g1_i3	Glyma.12G201100.2	100	184	6	152	3113	1	2938	0	4263

TRINITY_DN16303_c0_g1_i5	Glyma.12G201100.1	100	184	6	152	3113	1	2938	0	4263

TRINITY_DN16303_c0_g1_i5	Glyma.12G201100.2	100	184	6	152	3113	1	2938	0	4263

TRINITY_DN16151_c0_g2_i4	Glyma.02G026700.2	98.27586207	154	4	318	3270	1	2956	0	4486

TRINITY_DN16151_c0_g2_i4	Glyma.02G026700.1	96.25	154	4	318	3270	1	2956	0	4486

TRINITY_DN16151_c0_g2_i7	Glyma.02G026700.3	99.46091644	154	4	318	3270	1	2956	0	4486

TRINITY_DN15340_c0_g1_i1	Glyma.17G112500.2	98.42519685	140	2	970	3928	2946	1	0	4538

TRINITY_DN11513_c0_g1_i1	Glyma.13G165400.1	95.57739558	202	4	601	3561	2961	1	0	4316

TRINITY_DN6941_c0_g1_i1	Glyma.18G067400.1	97.04142012	167	3	117	3076	4029	1067	0	4516

TRINITY_DN15521_c0_g1_i1	Glyma.12G231800.1	99.25925926	488	39	182	3111	79	3023	0	2438

TRINITY_DN15818_c1_g2_i2	Glyma.03G136500.1	100	430	20	474	3398	2955	1	0	2793

TRINITY_DN15818_c1_g2_i2	Glyma.19G138700.1	97.87234043	425	20	474	3398	2955	1	0	2820

TRINITY_DN15818_c1_g2_i2	Glyma.19G138700.2	100	425	20	474	3398	2955	1	0	2820

TRINITY_DN16094_c0_g1_i1	Glyma.20G143200.1	98.30508475	277	6	282	3242	3222	271	0	3853

TRINITY_DN16094_c0_g1_i6	Glyma.20G143200.1	98.7987988	277	6	282	3242	3222	271	0	3853

TRINITY_DN12658_c0_g1_i2	Glyma.01G218100.1	95.93495935	531	33	297	3226	16	2966	0	2250

TRINITY_DN10628_c0_g1_i2	Glyma.08G031500.1	100	152	1	1	2962	1344	4302	0	4608

TRINITY_DN10628_c0_g1_i2	Glyma.05G224700.1	100	151	1	1	2962	1344	4302	0	4614

TRINITY_DN10628_c0_g1_i2	Glyma.05G224700.2	100	151	1	1	2962	1344	4302	0	4614

TRINITY_DN18060_c0_g1_i1	Glyma.02G312200.1	96.75324675	142	4	1	2955	479	3439	0	4632

TRINITY_DN18060_c0_g1_i1	Glyma.14G000300.1	100	142	6	1	2955	458	3418	0	4632

TRINITY_DN15521_c0_g1_i4	Glyma.12G233000.1	95.54794521	507	39	182	3111	79	3020	0	2337

TRINITY_DN38460_c0_g1_i1	Glyma.18G096800.1	100	300	16	145	3099	3301	6239	0	3626

TRINITY_DN10789_c0_g2_i1	Glyma.03G169700.2	100	222	20	81	3020	1	2940	0	3967

TRINITY_DN10789_c0_g2_i1	Glyma.03G169700.1	100	222	20	81	3020	1	2940	0	3967

TRINITY_DN2211_c0_g1_i1	Glyma.03G264400.1	98.29545455	149	1	79	3036	1	2961	0	4623

TRINITY_DN2211_c0_g1_i1	Glyma.03G264400.3	96.03399433	149	1	79	3036	1	2961	0	4623

TRINITY_DN2211_c0_g1_i1	Glyma.03G264400.6	100	149	1	79	3036	1	2961	0	4623

TRINITY_DN16123_c0_g2_i1	Glyma.04G203000.1	97.4522293	365	6	200	3155	2953	1	0	3373

TRINITY_DN12325_c0_g1_i1	Glyma.16G210500.1	96.11650485	301	19	59	3007	80	3018	0	3592

TRINITY_DN12526_c0_g1_i1	Glyma.13G238000.2	98.71794872	265	11	1	2955	63	3008	0	3875

TRINITY_DN12526_c0_g1_i1	Glyma.13G238000.1	98.71794872	265	11	1	2955	63	3008	0	3875

TRINITY_DN12526_c0_g1_i1	Glyma.13G238000.5	97.01492537	265	11	1	2955	63	3008	0	3875

TRINITY_DN15417_c0_g1_i1	Glyma.05G194500.1	100	240	29	467	3399	2952	16	0	3814

TRINITY_DN15417_c0_g1_i2	Glyma.05G194500.1	97.58241758	240	29	733	3665	2952	16	0	3814

TRINITY_DN10961_c0_g1_i1	Glyma.11G256600.1	97.11538462	283	13	218	3167	1	2938	0	3703

TRINITY_DN10961_c0_g1_i1	Glyma.11G256600.2	97.11538462	283	13	218	3167	1	2938	0	3703

TRINITY_DN16151_c0_g2_i4	Glyma.01G038300.1	98.27586207	158	3	318	3270	1	2953	0	4512

TRINITY_DN16151_c0_g2_i7	Glyma.01G038300.2	96.69421488	158	3	318	3270	1	2953	0	4512

TRINITY_DN15521_c0_g1_i1	Glyma.12G233000.1	96.55172414	499	35	182	3111	79	3020	0	2414

TRINITY_DN16094_c0_g1_i3	Glyma.10G250500.1	97.82608696	335	24	21	2954	3186	244	0	3354

TRINITY_DN16094_c0_g1_i7	Glyma.10G250500.1	100	335	24	21	2954	3186	244	0	3354

TRINITY_DN15847_c1_g2_i1	Glyma.18G119000.2	96.42857143	276	5	437	3385	249	3203	0	3856

TRINITY_DN15639_c1_g1_i1	Glyma.U022000.3	100	233	12	551	3490	2955	1	0	4036

TRINITY_DN15639_c1_g1_i2	Glyma.U022000.3	100	233	12	675	3614	2955	1	0	4036

TRINITY_DN15639_c1_g1_i3	Glyma.U022000.3	97.95918367	233	12	358	3297	2955	1	0	4036

TRINITY_DN15847_c1_g2_i2	Glyma.18G119000.2	96.34703196	198	5	131	3079	249	3203	0	4289

TRINITY_DN2211_c0_g1_i1	Glyma.19G263400.1	100	153	1	79	3036	1	2952	0	4577

TRINITY_DN12325_c0_g1_i2	Glyma.16G078900.1	97.22222222	597	43	208	3133	95	3022	0	1766

TRINITY_DN16245_c0_g3_i5	Glyma.16G099700.2	98.87640449	406	25	213	3155	2973	31	0	3031

TRINITY_DN16245_c0_g3_i5	Glyma.16G099700.1	98.37662338	406	25	213	3155	2973	31	0	3031

TRINITY_DN15334_c0_g1_i2	Glyma.04G204800.1	100	151	9	384	3339	3543	615	0	4436

TRINITY_DN16374_c1_g1_i2	Glyma.07G069400.2	97.36842105	148	18	1	2941	3114	6048	0	4396

TRINITY_DN16123_c0_g2_i1	Glyma.06G162500.2	96.2962963	351	3	201	3155	2952	1	0	3482

TRINITY_DN16123_c0_g2_i1	Glyma.06G162500.1	98.05194805	351	3	201	3155	2952	1	0	3482

TRINITY_DN16123_c0_g2_i1	Glyma.06G162500.3	98.14814815	351	3	201	3155	2952	1	0	3482

TRINITY_DN16298_c0_g1_i1	Glyma.13G308700.1	97.10144928	318	3	297	3248	2955	1	0	3664

TRINITY_DN12325_c0_g1_i1	Glyma.09G161100.1	96.11650485	288	16	59	3007	80	3015	0	3688

TRINITY_DN15815_c0_g2_i7	Glyma.12G147000.1	97.91666667	282	10	466	3407	3543	590	0	3794

TRINITY_DN15815_c0_g2_i7	Glyma.12G147000.3	97.91666667	282	10	466	3407	3540	587	0	3794

TRINITY_DN15815_c0_g2_i7	Glyma.12G147000.2	96.2962963	282	10	466	3407	3213	260	0	3794

TRINITY_DN15645_c0_g1_i4	Glyma.05G209200.1	97.24137931	182	7	37	2979	1	2955	0	4361

TRINITY_DN15334_c0_g1_i2	Glyma.06G160800.1	95.45454545	151	2	384	3339	3567	615	0	4602

TRINITY_DN12325_c0_g1_i1	Glyma.01G007400.1	97.22222222	669	58	77	2993	104	3017	0	1240

TRINITY_DN12325_c0_g1_i2	Glyma.01G007400.1	95.34883721	668	58	214	3130	104	3017	0	1245

TRINITY_DN1785_c0_g1_i1	Glyma.06G086800.1	100	309	20	145	3070	2938	1	0	3448

TRINITY_DN11596_c0_g2_i1	Glyma.01G004100.2	98.93617021	300	18	322	3241	2946	18	0	3400

TRINITY_DN10961_c0_g1_i1	Glyma.18G000500.1	98.97959184	272	9	218	3167	1	2938	0	3808

TRINITY_DN11875_c0_g2_i1	Glyma.05G148300.1	95.3125	245	9	240	3176	1	2952	0	3964

TRINITY_DN11875_c0_g2_i1	Glyma.05G148300.3	95.3125	244	16	240	3176	1	2937	0	3873

TRINITY_DN11875_c0_g2_i1	Glyma.05G148300.2	95.29652352	243	15	240	3176	1	2934	0	3858

TRINITY_DN13629_c0_g2_i1	Glyma.05G143100.1	95.78947368	195	6	333	3287	2942	1	0	4292

TRINITY_DN14862_c0_g1_i3	Glyma.20G181600.1	99.18032787	181	2	412	3363	2952	1	0	4416

TRINITY_DN14862_c0_g1_i3	Glyma.20G181600.4	96.34146341	181	2	412	3363	2952	1	0	4416

TRINITY_DN16298_c0_g1_i2	Glyma.13G308700.1	97.61904762	175	1	345	3296	2955	1	0	4468

TRINITY_DN16132_c0_g1_i4	Glyma.17G226100.1	97.48743719	238	10	641	3592	1	2943	0	4052

TRINITY_DN9513_c0_g2_i1	Glyma.04G236700.4	96.57657658	226	4	198	3139	47	2994	0	4087

TRINITY_DN11989_c0_g1_i1	Glyma.06G083700.1	98.20359281	174	4	108	3056	1	2952	0	4446

TRINITY_DN11989_c0_g1_i1	Glyma.04G082000.1	97.70114943	172	4	108	3056	1	2952	0	4457

TRINITY_DN14862_c0_g1_i3	Glyma.10G209000.1	99.05660377	194	2	412	3363	2952	1	0	4366

TRINITY_DN14862_c0_g1_i3	Glyma.10G209000.2	99.05660377	194	2	412	3363	2952	1	0	4366

TRINITY_DN12325_c0_g1_i2	Glyma.01G007500.1	97.22222222	597	36	208	3133	89	3013	0	1801

TRINITY_DN16385_c0_g1_i3	Glyma.19G091300.1	100	296	16	589	3522	3423	499	0	3520

TRINITY_DN16132_c0_g1_i4	Glyma.14G098600.1	100	253	6	641	3592	1	2943	0	3991

TRINITY_DN10789_c0_g2_i1	Glyma.19G170900.1	100	235	8	81	3020	1	2949	0	4052

TRINITY_DN10789_c0_g2_i1	Glyma.19G170900.2	100	235	8	81	3020	1	2949	0	4052

TRINITY_DN14862_c0_g1_i3	Glyma.20G181600.2	99.18032787	181	1	412	3363	2949	1	0	4429

TRINITY_DN14862_c0_g1_i3	Glyma.20G181600.3	99.18032787	181	1	412	3363	2949	1	0	4429

TRINITY_DN14654_c0_g1_i2	Glyma.09G194900.1	100	398	21	1	2936	2977	44	0	3038

TRINITY_DN15815_c0_g2_i7	Glyma.06G254500.1	96.57534247	282	9	466	3407	3525	578	0	3810

TRINITY_DN16425_c0_g1_i2	Glyma.12G060600.4	96.30390144	240	5	706	3652	2947	1	0	4069

TRINITY_DN16425_c0_g1_i5	Glyma.12G060600.4	100	240	5	706	3652	2947	1	0	4069

TRINITY_DN10917_c1_g1_i1	Glyma.04G192200.5	99.5951417	205	3	288	3237	14	2960	0	4281

TRINITY_DN6780_c0_g1_i2	Glyma.15G166700.2	95.07575758	205	8	300	3233	3447	499	0	4191

TRINITY_DN6780_c0_g1_i2	Glyma.15G166700.3	98.35526316	205	8	300	3233	2967	19	0	4191

TRINITY_DN14862_c0_g1_i1	Glyma.20G181600.1	96.90444145	181	2	412	3359	2952	5	0	4409

TRINITY_DN14862_c0_g1_i1	Glyma.20G181600.4	96.9047619	181	2	412	3359	2952	5	0	4409

TRINITY_DN16373_c3_g5_i6	Glyma.15G005500.2	98.06763285	459	23	20	2950	3936	1015	0	2601

TRINITY_DN16373_c3_g5_i8	Glyma.15G005500.2	98.03921569	459	23	20	2950	3936	1015	0	2601

TRINITY_DN16373_c3_g5_i6	Glyma.15G005500.3	98.06763285	459	23	20	2950	3825	904	0	2601

TRINITY_DN16373_c3_g5_i8	Glyma.15G005500.3	98.05825243	459	23	20	2950	3825	904	0	2601

TRINITY_DN12658_c0_g1_i2	Glyma.05G206100.1	95.91836735	247	9	291	3226	1	2945	0	3956

TRINITY_DN12658_c0_g1_i2	Glyma.08G013000.1	95.93301435	231	9	291	3226	1	2942	0	4037

TRINITY_DN11630_c1_g1_i1	Glyma.08G109600.2	97.29344729	224	17	284	3202	1	2928	0	3875

TRINITY_DN11630_c1_g1_i1	Glyma.08G109600.1	97.5308642	224	17	284	3202	1	2928	0	3875

TRINITY_DN14862_c0_g1_i1	Glyma.10G209000.1	98	194	2	412	3359	2952	5	0	4359

TRINITY_DN14862_c0_g1_i1	Glyma.10G209000.2	96	194	2	412	3359	2952	5	0	4359

TRINITY_DN1785_c0_g1_i1	Glyma.04G085200.1	100	324	14	145	3070	2932	1	0	3404

TRINITY_DN15225_c0_g1_i2	Glyma.20G099200.1	100	293	9	231	3174	3066	126	0	3749

TRINITY_DN11569_c0_g1_i1	Glyma.13G059200.2	99.54545455	222	13	325	3249	2943	1	0	4034

TRINITY_DN14862_c0_g1_i1	Glyma.20G181600.2	96.90402477	181	1	412	3359	2949	5	0	4421

TRINITY_DN14862_c0_g1_i1	Glyma.20G181600.3	96.90402477	181	1	412	3359	2949	5	0	4421

TRINITY_DN14654_c0_g1_i2	Glyma.16G111200.3	99.51219512	407	29	11	2936	2970	44	0	2922

TRINITY_DN14654_c0_g1_i2	Glyma.16G111200.2	96.06299213	407	29	11	2936	2970	44	0	2922

TRINITY_DN14654_c0_g1_i2	Glyma.16G111200.1	100	407	29	11	2936	2970	44	0	2922

TRINITY_DN15225_c0_g1_i1	Glyma.20G099200.1	100	386	7	509	3452	3066	126	0	3245

TRINITY_DN1785_c0_g1_i1	Glyma.06G086800.2	100	310	20	145	3070	2929	1	0	3472

TRINITY_DN16441_c4_g2_i5	Glyma.13G235300.1	99.33110368	254	21	601	3519	5268	2329	0	3808

TRINITY_DN16441_c4_g2_i5	Glyma.13G235300.3	95.04950495	254	21	601	3519	4488	1549	0	3808

TRINITY_DN16441_c4_g2_i5	Glyma.13G235300.2	100	254	21	601	3519	4488	1549	0	3808

TRINITY_DN14837_c0_g3_i2	Glyma.09G284900.1	95.71428571	216	0	1	2947	1089	4035	0	4246

TRINITY_DN15040_c0_g1_i1	Glyma.15G163900.1	99.18032787	221	7	605	3548	2937	3	0	4132

TRINITY_DN15040_c0_g1_i1	Glyma.15G163900.3	100	221	7	605	3548	2937	3	0	4132

TRINITY_DN14432_c0_g1_i5	Glyma.17G233000.1	97.48953975	173	14	210	3125	88	3027	0	4250

TRINITY_DN15040_c0_g1_i1	Glyma.09G057800.1	96.45390071	241	2	605	3548	2946	3	0	4091

TRINITY_DN5178_c0_g1_i1	Glyma.03G148700.1	100	233	11	390	3317	2940	1	0	4017

TRINITY_DN16094_c0_g1_i1	Glyma.11G004100.1	95.67901235	194	7	567	3497	2943	1	0	4272

TRINITY_DN16373_c3_g5_i6	Glyma.08G214900.2	98.05825243	204	6	11	2950	3915	985	0	4207

TRINITY_DN16373_c3_g5_i6	Glyma.08G214900.1	98.04964539	204	6	11	2950	3915	985	0	4207

TRINITY_DN16373_c3_g5_i8	Glyma.08G214900.2	98.05194805	204	6	11	2950	3915	985	0	4207

TRINITY_DN16373_c3_g5_i8	Glyma.08G214900.1	98.05825243	204	6	11	2950	3915	985	0	4207

TRINITY_DN16298_c0_g1_i1	Glyma.12G193800.1	98.14814815	314	1	297	3233	2955	16	0	3670

TRINITY_DN35910_c0_g1_i1	Glyma.13G214400.1	98.50746269	185	9	69	3004	1	2930	0	4314

TRINITY_DN16298_c0_g1_i2	Glyma.12G193800.1	96.26865672	169	1	345	3281	2955	16	0	4473

TRINITY_DN9021_c0_g1_i1	Glyma.04G062200.1	95.47511312	164	5	59	2998	1	2925	0	4423

TRINITY_DN12563_c0_g1_i1	Glyma.08G027800.2	96.92307692	507	37	269	3192	60	2950	0	2211

TRINITY_DN12563_c0_g1_i1	Glyma.08G027800.1	100	507	37	269	3192	60	2950	0	2211

TRINITY_DN35910_c0_g1_i1	Glyma.15G098800.2	98.50746269	196	5	69	3004	1	2933	0	4283

TRINITY_DN35910_c0_g1_i1	Glyma.15G098800.1	98.50746269	196	5	69	3004	1	2933	0	4283

TRINITY_DN10987_c0_g1_i1	Glyma.19G028400.1	100	167	12	138	3037	19	2942	0	4154

TRINITY_DN16286_c0_g1_i2	Glyma.10G234500.2	100	417	18	301	3222	2988	58	0	2966

TRINITY_DN16115_c0_g2_i6	Glyma.U033900.3	100	343	5	552	3487	2930	1	0	3461

TRINITY_DN16094_c0_g1_i3	Glyma.20G143200.1	97.82608696	315	28	21	2933	3189	271	0	3397

TRINITY_DN16094_c0_g1_i7	Glyma.20G143200.1	100	315	28	21	2933	3189	271	0	3397

TRINITY_DN17615_c0_g1_i1	Glyma.05G165600.1	100	313	15	161	3093	1	2924	0	3568

TRINITY_DN13828_c0_g2_i1	Glyma.13G214300.1	95.68965517	313	20	246	3173	1	2907	0	3426

TRINITY_DN13828_c0_g2_i1	Glyma.13G214300.4	95.68965517	313	20	246	3173	1	2907	0	3426

TRINITY_DN13828_c0_g2_i1	Glyma.13G214300.2	95.68965517	313	20	246	3173	1	2907	0	3426

TRINITY_DN16115_c0_g2_i4	Glyma.U033900.3	100	306	5	552	3487	2930	1	0	3666

TRINITY_DN14939_c1_g1_i2	Glyma.06G142500.1	95.46351085	252	8	216	3137	1	2937	0	3919

TRINITY_DN14939_c1_g1_i2	Glyma.04G222800.1	95.47038328	234	8	216	3137	1	2937	0	4019

TRINITY_DN16115_c0_g2_i2	Glyma.U033900.3	97.95918367	231	3	449	3385	2931	1	0	4095

TRINITY_DN16115_c0_g2_i1	Glyma.U033900.3	100	194	3	449	3385	2931	1	0	4300

TRINITY_DN10987_c0_g1_i1	Glyma.13G057800.1	96.2962963	168	24	138	3038	16	2934	0	4133

TRINITY_DN16373_c3_g1_i1	Glyma.08G214900.2	100	453	44	349	3201	3908	982	0	2307

TRINITY_DN16373_c3_g1_i1	Glyma.08G214900.1	100	453	44	349	3201	3908	982	0	2307

TRINITY_DN13828_c0_g2_i1	Glyma.15G098900.1	95.68965517	331	19	246	3173	1	2907	0	3338

TRINITY_DN13436_c0_g1_i1	Glyma.05G237400.1	99.25373134	262	26	254	3158	2934	9	0	3705

TRINITY_DN15040_c0_g1_i1	Glyma.15G163900.6	100	221	7	616	3548	2926	3	0	4111

TRINITY_DN15040_c0_g1_i1	Glyma.15G163900.4	98.83040936	221	7	616	3548	2926	3	0	4111

TRINITY_DN15040_c0_g1_i1	Glyma.15G163900.5	96.09375	221	7	616	3548	2926	3	0	4111

TRINITY_DN10625_c0_g1_i1	Glyma.20G097700.1	97.6744186	166	6	333	3264	2952	33	0	4385

TRINITY_DN12050_c0_g2_i2	Glyma.16G093800.20	98.48484848	493	76	3467	6365	3018	5850	0	1808

TRINITY_DN12050_c0_g2_i3	Glyma.16G093800.20	100	493	76	3482	6380	3018	5850	0	1808

TRINITY_DN12050_c0_g2_i2	Glyma.16G093800.19	99.2481203	493	76	3467	6365	3003	5835	0	1808

TRINITY_DN12050_c0_g2_i3	Glyma.16G093800.19	100	493	76	3482	6380	3003	5835	0	1808

TRINITY_DN15458_c0_g1_i2	Glyma.17G004700.1	95.4887218	438	33	403	3295	1	2920	0	2636

TRINITY_DN12658_c0_g1_i3	Glyma.05G206100.2	95.91836735	382	31	236	3152	7	2908	0	2985

TRINITY_DN12658_c0_g1_i4	Glyma.05G206100.2	95.93495935	382	31	236	3152	7	2908	0	2985

TRINITY_DN12612_c0_g1_i1	Glyma.08G361900.1	97.05882353	210	8	60	2978	13	2946	0	4157

TRINITY_DN11667_c0_g1_i1	Glyma.02G101100.1	100	192	9	305	3223	1	2934	0	4257

TRINITY_DN15919_c0_g1_i3	Glyma.04G201200.1	96.1038961	319	16	521	3433	2916	1	0	3410

TRINITY_DN16071_c0_g2_i4	Glyma.15G158300.1	97.56097561	218	9	127	3052	60	2989	0	4145

TRINITY_DN16016_c0_g1_i4	Glyma.10G221500.1	98.9821883	170	7	1337	4263	2923	6	0	4338

TRINITY_DN16016_c0_g1_i4	Glyma.10G221500.3	96.2962963	170	7	1337	4263	2923	6	0	4338

TRINITY_DN16016_c0_g1_i4	Glyma.10G221500.2	100	170	7	1337	4263	2923	6	0	4338

TRINITY_DN16016_c0_g1_i5	Glyma.10G221500.1	100	170	7	1337	4263	2923	6	0	4338

TRINITY_DN16016_c0_g1_i5	Glyma.10G221500.3	100	170	7	1337	4263	2923	6	0	4338

TRINITY_DN16016_c0_g1_i5	Glyma.10G221500.2	98.96907216	170	7	1337	4263	2923	6	0	4338

TRINITY_DN12196_c0_g1_i2	Glyma.13G295100.1	100	132	0	32	2963	351	3282	0	4684

TRINITY_DN12563_c0_g1_i2	Glyma.08G027800.2	100	498	37	269	3184	60	2942	0	2246

TRINITY_DN12563_c0_g1_i2	Glyma.08G027800.1	96.95290859	498	37	269	3184	60	2942	0	2246

TRINITY_DN16151_c0_g2_i3	Glyma.02G026700.3	100	350	31	34	2914	46	2956	0	3022

TRINITY_DN11630_c1_g1_i1	Glyma.05G152900.1	98.98373984	242	10	284	3202	1	2910	0	3858

TRINITY_DN11630_c1_g1_i1	Glyma.05G152900.2	96.46990741	242	10	284	3202	1	2910	0	3858

TRINITY_DN14432_c0_g1_i5	Glyma.U029000.4	97.48283753	187	9	210	3125	88	3009	0	4222

TRINITY_DN14432_c0_g1_i5	Glyma.U029000.3	97.47474747	187	9	210	3125	88	3009	0	4222

TRINITY_DN14432_c0_g1_i5	Glyma.U029000.1	97.48427673	187	9	210	3125	88	3009	0	4222

TRINITY_DN14432_c0_g1_i5	Glyma.U029000.2	97.4789916	187	9	210	3125	88	3009	0	4222

TRINITY_DN16016_c0_g1_i4	Glyma.20G170000.1	99.01960784	170	6	1337	4258	2926	11	0	4316

TRINITY_DN16016_c0_g1_i5	Glyma.20G170000.1	97.22222222	170	6	1337	4258	2926	11	0	4316

TRINITY_DN16190_c0_g3_i3	Glyma.06G277500.1	95.1048951	582	32	271	3186	1	2901	0	1908

TRINITY_DN12563_c0_g1_i1	Glyma.05G221300.3	100	519	40	269	3186	60	2935	0	2109

TRINITY_DN12563_c0_g1_i1	Glyma.05G221300.1	96.23430962	519	40	269	3186	60	2935	0	2109

TRINITY_DN12563_c0_g1_i3	Glyma.05G221300.3	98.80749574	519	40	269	3186	60	2935	0	2109

TRINITY_DN16168_c0_g1_i8	Glyma.05G248500.1	96.42857143	382	25	524	3420	3300	389	0	2964

TRINITY_DN15919_c0_g1_i3	Glyma.04G201200.2	96.1038961	320	14	521	3433	2913	1	0	3419

TRINITY_DN16071_c0_g2_i3	Glyma.15G158300.1	95.55555556	157	4	127	3043	60	2989	0	4457

TRINITY_DN11667_c0_g1_i1	Glyma.20G185300.1	100	417	49	305	3183	1	2894	0	2549

TRINITY_DN16168_c0_g1_i5	Glyma.05G248500.1	98.31775701	362	26	620	3516	3300	389	0	3086

TRINITY_DN12050_c0_g2_i1	Glyma.16G093800.20	100	478	73	255	3147	3021	5850	0	1899

TRINITY_DN12050_c0_g2_i1	Glyma.16G093800.19	100	478	73	255	3147	3006	5835	0	1899

TRINITY_DN15808_c0_g1_i1	Glyma.04G163600.2	99.14893617	306	13	129	3043	1	2912	0	3524

TRINITY_DN15808_c0_g1_i1	Glyma.04G163600.1	98.13664596	306	13	129	3043	1	2912	0	3524

TRINITY_DN15808_c0_g1_i1	Glyma.04G163600.3	100	306	13	129	3043	1	2912	0	3524

TRINITY_DN16441_c1_g1_i1	Glyma.14G016800.2	95	251	8	837	3762	696	3609	0	3914

TRINITY_DN16190_c0_g3_i7	Glyma.12G210600.1	96.19047619	483	27	339	3257	1	2901	0	2510

TRINITY_DN13629_c0_g2_i2	Glyma.05G143100.1	96.51162791	190	6	283	3208	2913	1	0	4266

TRINITY_DN11667_c0_g1_i1	Glyma.01G089300.1	98.11715481	187	7	305	3218	1	2926	0	4290

TRINITY_DN16168_c0_g1_i8	Glyma.08G057200.2	96.42857143	381	25	524	3420	3282	380	0	2968

TRINITY_DN16168_c0_g1_i8	Glyma.08G057200.3	96.42857143	381	25	524	3420	3282	380	0	2968

TRINITY_DN16168_c0_g1_i5	Glyma.08G057200.2	98.31775701	362	25	620	3516	3282	380	0	3073

TRINITY_DN16168_c0_g1_i5	Glyma.08G057200.3	98.31932773	362	25	620	3516	3282	380	0	3073

TRINITY_DN13622_c0_g1_i1	Glyma.16G182200.1	97.61904762	257	16	401	3300	2928	8	0	3790

TRINITY_DN15818_c1_g2_i2	Glyma.10G099200.2	97.87234043	241	3	475	3398	2921	1	0	4034

TRINITY_DN15818_c1_g2_i2	Glyma.10G099200.1	97.87234043	241	3	475	3398	2921	1	0	4034

TRINITY_DN15818_c1_g2_i2	Glyma.10G099200.3	97.87234043	238	7	475	3398	2909	1	0	3973

TRINITY_DN15818_c1_g2_i2	Glyma.02G184600.1	97.87234043	229	1	474	3398	2922	1	0	4113

TRINITY_DN16033_c0_g1_i3	Glyma.03G238100.2	100	332	12	142	3059	2917	18	0	3367

TRINITY_DN13959_c0_g1_i4	Glyma.10G292300.3	100	167	28	319	3188	2918	1	0	4087

TRINITY_DN15479_c0_g2_i1	Glyma.04G088800.1	98.64864865	382	21	323	3224	2911	1	0	3068

TRINITY_DN14362_c0_g1_i1	Glyma.01G153200.1	98.18181818	342	24	208	3108	1	2901	0	3219

TRINITY_DN16033_c0_g1_i3	Glyma.19G235500.1	100	320	14	142	3059	2914	18	0	3424

TRINITY_DN16033_c0_g1_i4	Glyma.03G238100.2	99.61832061	268	10	142	3059	2917	18	0	3733

TRINITY_DN15919_c0_g1_i2	Glyma.04G201200.1	97.79816514	249	10	521	3427	2916	1	0	3869

TRINITY_DN16033_c0_g1_i2	Glyma.10G154400.1	96.61016949	261	4	378	3294	2923	4	0	3904

TRINITY_DN16033_c0_g1_i2	Glyma.20G233900.1	100	257	4	378	3294	2920	1	0	3927

TRINITY_DN16033_c0_g1_i4	Glyma.19G235500.1	100	254	12	142	3059	2914	18	0	3801

TRINITY_DN11667_c0_g1_i1	Glyma.01G089300.3	100	186	6	305	3218	1	2920	0	4301

TRINITY_DN16033_c0_g1_i1	Glyma.20G233900.1	96.03960396	286	3	378	3294	2920	1	0	3777

TRINITY_DN16033_c0_g1_i1	Glyma.10G154400.1	96.03960396	284	3	378	3294	2923	4	0	3788

TRINITY_DN6279_c0_g1_i1	Glyma.12G008200.2	100	209	9	384	3299	208	3111	0	4095

TRINITY_DN15919_c0_g1_i2	Glyma.04G201200.2	95	250	8	521	3427	2913	1	0	3879

TRINITY_DN11945_c0_g1_i1	Glyma.01G197600.1	96.11111111	329	19	433	3333	28	2937	0	3393

TRINITY_DN15674_c0_g1_i5	Glyma.02G228400.1	100	228	1	1	2919	5045	2128	0	4120

TRINITY_DN673_c0_g2_i1	Glyma.07G224000.1	100	190	2	1	2916	475	3393	0	4318

TRINITY_DN673_c0_g2_i1	Glyma.07G224000.2	100	190	2	1	2916	475	3393	0	4318

TRINITY_DN16190_c0_g3_i9	Glyma.06G277500.1	96.0502693	577	26	271	3174	1	2901	0	1991

TRINITY_DN15479_c0_g2_i1	Glyma.06G090800.1	97.86585366	394	14	326	3224	2908	1	0	3018

TRINITY_DN15618_c0_g1_i2	Glyma.04G051200.1	98.7804878	362	17	415	3323	3095	187	0	3267

TRINITY_DN15618_c0_g1_i2	Glyma.04G051200.1	100	362	17	3314	6222	187	3095	0	3267

TRINITY_DN9107_c0_g1_i1	Glyma.11G057500.3	99.06542056	245	20	1086	3967	2	2907	0	3722

TRINITY_DN16811_c0_g1_i1	Glyma.20G229500.1	96.36363636	178	22	642	3520	2909	1	0	4093

TRINITY_DN15328_c0_g4_i1	Glyma.07G056700.4	98.24561404	480	55	1	2877	529	3389	0	2108

TRINITY_DN16190_c0_g3_i3	Glyma.13G270400.4	100	325	6	271	3186	1	2904	0	3496

TRINITY_DN16190_c0_g3_i3	Glyma.13G270400.2	97.4025974	325	6	271	3186	1	2904	0	3496

TRINITY_DN16190_c0_g3_i3	Glyma.13G270400.3	97.4025974	325	6	271	3186	1	2904	0	3496

TRINITY_DN16190_c0_g3_i3	Glyma.13G270400.1	97.72727273	325	6	271	3186	1	2904	0	3496

TRINITY_DN16190_c0_g3_i3	Glyma.13G270400.5	97.72727273	325	6	271	3186	1	2904	0	3496

TRINITY_DN16190_c0_g3_i3	Glyma.06G229900.1	95.93495935	208	6	271	3186	1	2904	0	4145

TRINITY_DN16190_c0_g3_i3	Glyma.12G161300.1	96.11650485	205	4	271	3186	1	2904	0	4161

TRINITY_DN15618_c0_g1_i2	Glyma.17G228800.1	100	185	0	414	3330	3171	255	0	4362

TRINITY_DN15618_c0_g1_i2	Glyma.17G228800.1	95.23809524	185	0	3307	6223	255	3171	0	4362

TRINITY_DN11667_c0_g1_i1	Glyma.01G089300.2	100	178	12	305	3218	1	2902	0	4285

TRINITY_DN16190_c0_g3_i8	Glyma.12G210600.1	100	483	22	339	3245	1	2901	0	2555

TRINITY_DN16190_c0_g3_i7	Glyma.06G229900.1	95.83333333	334	17	348	3257	10	2904	0	3360

TRINITY_DN16190_c0_g3_i3	Glyma.12G229400.3	95.93495935	315	4	271	3186	1	2904	0	3563

TRINITY_DN16190_c0_g3_i3	Glyma.12G229400.1	96.55172414	315	4	271	3186	1	2904	0	3563

TRINITY_DN16190_c0_g3_i3	Glyma.12G229400.2	96.55172414	315	4	271	3186	1	2904	0	3563

TRINITY_DN9476_c0_g1_i1	Glyma.07G263100.1	99.48717949	197	3	334	3243	2919	24	0	4126

TRINITY_DN15618_c0_g1_i2	Glyma.14G094700.1	98.26086957	161	5	418	3330	3176	264	0	4455

TRINITY_DN15618_c0_g1_i2	Glyma.14G094700.1	98.26086957	161	5	3307	6219	264	3176	0	4455

TRINITY_DN16175_c0_g1_i1	Glyma.11G043800.1	100	515	20	348	3252	136	3037	0	2381

TRINITY_DN16175_c0_g1_i4	Glyma.11G043800.1	98.48484848	515	20	332	3236	136	3037	0	2381

TRINITY_DN16175_c0_g1_i5	Glyma.11G043800.1	100	515	20	348	3252	136	3037	0	2381

TRINITY_DN16175_c0_g1_i6	Glyma.11G043800.1	99.31506849	515	20	332	3236	136	3037	0	2381

TRINITY_DN16190_c0_g3_i2	Glyma.13G270400.4	100	513	24	271	3168	1	2904	0	2361

TRINITY_DN16190_c0_g3_i2	Glyma.13G270400.2	100	513	24	271	3168	1	2904	0	2361

TRINITY_DN16190_c0_g3_i2	Glyma.13G270400.3	100	513	24	271	3168	1	2904	0	2361

TRINITY_DN16190_c0_g3_i2	Glyma.13G270400.1	100	513	24	271	3168	1	2904	0	2361

TRINITY_DN16190_c0_g3_i2	Glyma.13G270400.5	96.2962963	513	24	271	3168	1	2904	0	2361

TRINITY_DN16190_c0_g3_i2	Glyma.12G229400.3	98.45201238	505	24	271	3168	1	2904	0	2405

TRINITY_DN16190_c0_g3_i2	Glyma.12G229400.1	98.45201238	505	24	271	3168	1	2904	0	2405

TRINITY_DN16190_c0_g3_i2	Glyma.12G229400.2	98.45360825	505	24	271	3168	1	2904	0	2405

TRINITY_DN16368_c0_g2_i2	Glyma.19G158900.5	100	499	15	1	2907	964	3870	0	2516

TRINITY_DN16368_c0_g2_i2	Glyma.19G158900.1	100	499	15	1	2907	964	3870	0	2516

TRINITY_DN16368_c0_g2_i2	Glyma.19G158900.2	100	499	15	1	2907	964	3870	0	2516

TRINITY_DN16368_c0_g2_i2	Glyma.19G158900.3	100	499	15	1	2907	964	3870	0	2516

TRINITY_DN16368_c0_g2_i2	Glyma.19G158900.4	100	499	15	1	2907	964	3870	0	2516

TRINITY_DN16190_c0_g3_i7	Glyma.06G277500.1	100	473	26	352	3257	13	2901	0	2536

TRINITY_DN16132_c0_g1_i4	Glyma.04G056200.1	100	354	11	681	3586	41	2952	0	3345

TRINITY_DN15062_c0_g1_i1	Glyma.01G168200.1	95.81151832	459	50	231	3111	2875	1	0	2374

TRINITY_DN15062_c0_g1_i1	Glyma.01G168200.3	97.45762712	459	50	231	3111	2875	1	0	2374

TRINITY_DN15062_c0_g1_i1	Glyma.01G168200.2	95.23809524	458	52	231	3111	2869	1	0	2340

TRINITY_DN16190_c0_g3_i7	Glyma.12G161300.1	96.17486339	336	11	348	3257	10	2904	0	3371

TRINITY_DN15090_c0_g1_i3	Glyma.03G032900.1	95	180	0	309	3222	4644	1731	0	4385

TRINITY_DN12658_c0_g1_i1	Glyma.05G206100.2	100	247	9	360	3258	1	2908	0	3888

TRINITY_DN12658_c0_g1_i6	Glyma.05G206100.2	97.91666667	247	9	360	3258	1	2908	0	3888

TRINITY_DN14341_c0_g1_i1	Glyma.09G274600.1	95.75757576	241	10	148	3057	1	2904	0	3967

TRINITY_DN13512_c0_g1_i1	Glyma.06G101600.3	95.86466165	192	0	221	3133	2913	1	0	4316

TRINITY_DN13512_c0_g1_i1	Glyma.06G101600.4	95.85798817	192	0	221	3133	2913	1	0	4316

TRINITY_DN13512_c0_g1_i1	Glyma.06G101600.2	95.89041096	192	0	221	3133	2913	1	0	4316

TRINITY_DN13512_c0_g1_i1	Glyma.06G101600.1	95.8677686	192	0	221	3133	2913	1	0	4316

TRINITY_DN13512_c0_g1_i3	Glyma.06G101600.3	98.62068966	189	1	221	3113	2913	1	0	4204

TRINITY_DN13512_c0_g1_i3	Glyma.06G101600.4	98.63013699	189	1	221	3113	2913	1	0	4204

TRINITY_DN13512_c0_g1_i3	Glyma.06G101600.2	98.63013699	189	1	221	3113	2913	1	0	4204

TRINITY_DN13512_c0_g1_i3	Glyma.06G101600.1	96.17486339	189	1	221	3113	2913	1	0	4204

TRINITY_DN16168_c0_g1_i6	Glyma.05G248500.1	98.50746269	226	3	524	3432	3300	389	0	4106

TRINITY_DN16168_c0_g1_i2	Glyma.05G248500.1	98.98477157	204	3	620	3528	3300	389	0	4228

TRINITY_DN3084_c0_g1_i1	Glyma.08G266100.1	100	194	4	106	3006	1	2910	0	4229

TRINITY_DN3084_c0_g2_i1	Glyma.08G266100.1	100	194	4	106	3006	1	2910	0	4229

TRINITY_DN16190_c0_g3_i2	Glyma.06G229900.1	99.07407407	495	13	271	3168	1	2904	0	2516

TRINITY_DN16332_c0_g3_i1	Glyma.14G085200.2	97.5308642	325	11	582	3485	2901	1	0	3476

TRINITY_DN13622_c0_g1_i1	Glyma.16G182200.5	96.24413146	260	10	401	3300	2913	8	0	3843

TRINITY_DN15674_c0_g1_i5	Glyma.14G195400.1	96.25	212	1	10	2919	5031	2128	0	4161

TRINITY_DN10625_c0_g1_i1	Glyma.10G147200.1	100	151	6	351	3260	2886	1	0	4383

TRINITY_DN16190_c0_g3_i2	Glyma.12G161300.1	99.23664122	502	11	271	3168	1	2904	0	2488

TRINITY_DN16190_c0_g3_i4	Glyma.06G229900.1	100	586	26	348	3239	10	2904	0	1936

TRINITY_DN15618_c0_g1_i2	Glyma.06G052100.1	99.00744417	380	13	415	3315	3071	170	0	3181

TRINITY_DN15618_c0_g1_i2	Glyma.06G052100.1	99.31034483	380	13	3322	6222	170	3071	0	3181

TRINITY_DN9524_c0_g1_i1	Glyma.05G221500.2	97.90026247	222	7	192	3085	132	3031	0	3999

TRINITY_DN16420_c0_g1_i1	Glyma.06G201700.1	100	215	4	368	3268	229	3132	0	4108

TRINITY_DN16420_c0_g1_i5	Glyma.06G201700.1	97.14285714	215	4	368	3268	229	3132	0	4108

TRINITY_DN15062_c0_g1_i2	Glyma.01G168200.1	100	212	21	375	3233	2901	1	0	3834

TRINITY_DN15062_c0_g1_i2	Glyma.01G168200.3	96.51162791	212	21	375	3233	2901	1	0	3834

TRINITY_DN15062_c0_g1_i2	Glyma.01G168200.2	96.1038961	211	22	375	3233	2895	1	0	3801

TRINITY_DN8232_c0_g1_i2	Glyma.07G012400.2	97.05882353	307	11	599	3488	687	3588	0	3526

TRINITY_DN16266_c0_g2_i1	Glyma.20G239800.1	98.57142857	264	3	1	2903	781	3686	0	3873

TRINITY_DN15305_c1_g1_i2	Glyma.13G330400.1	96.17834395	286	29	258	3112	2903	1	0	3434

TRINITY_DN16266_c0_g2_i1	Glyma.10G290800.1	96.42857143	268	1	1	2903	781	3686	0	3862

TRINITY_DN16266_c0_g2_i1	Glyma.10G290800.2	96.29360465	268	1	1	2903	268	3173	0	3862

TRINITY_DN3084_c0_g1_i1	Glyma.16G101800.1	97.00598802	184	5	106	3006	1	2904	0	4301

TRINITY_DN3084_c0_g1_i1	Glyma.16G101800.2	100	184	5	106	3006	1	2904	0	4301

TRINITY_DN3084_c0_g2_i1	Glyma.16G101800.1	95.23809524	184	5	106	3006	1	2904	0	4301

TRINITY_DN3084_c0_g2_i1	Glyma.16G101800.2	100	184	5	106	3006	1	2904	0	4301

TRINITY_DN14721_c0_g1_i2	Glyma.18G058700.1	97.61904762	178	11	80	2970	5	2874	0	4050

TRINITY_DN14721_c0_g1_i2	Glyma.18G058700.4	98.79518072	178	12	80	2970	5	2865	0	3993

TRINITY_DN16190_c0_g3_i4	Glyma.12G161300.1	99.55555556	595	20	348	3239	10	2904	0	1919

TRINITY_DN13968_c0_g3_i2	Glyma.15G070100.1	100	401	38	1437	4314	1104	3981	0	2795

TRINITY_DN13968_c0_g3_i2	Glyma.15G070100.4	100	401	38	1437	4314	795	3672	0	2795

TRINITY_DN16190_c0_g3_i9	Glyma.13G270400.4	95.05494505	360	2	271	3174	1	2904	0	3358

TRINITY_DN16190_c0_g3_i9	Glyma.13G270400.2	98.27586207	360	2	271	3174	1	2904	0	3358

TRINITY_DN16190_c0_g3_i9	Glyma.13G270400.3	98.27586207	360	2	271	3174	1	2904	0	3358

TRINITY_DN16190_c0_g3_i9	Glyma.13G270400.1	100	360	2	271	3174	1	2904	0	3358

TRINITY_DN16190_c0_g3_i9	Glyma.13G270400.5	96.04938272	360	2	271	3174	1	2904	0	3358

TRINITY_DN16190_c0_g3_i9	Glyma.12G229400.3	95.05300353	351	2	271	3174	1	2904	0	3408

TRINITY_DN16190_c0_g3_i9	Glyma.12G229400.1	95.05494505	351	2	271	3174	1	2904	0	3408

TRINITY_DN16190_c0_g3_i9	Glyma.12G229400.2	95.05494505	351	2	271	3174	1	2904	0	3408

TRINITY_DN16499_c0_g1_i1	Glyma.08G265300.4	98.7654321	169	7	245	3145	1	2892	0	4318

TRINITY_DN16499_c0_g1_i1	Glyma.08G265300.1	98.7654321	169	7	245	3145	1	2892	0	4318

TRINITY_DN16499_c0_g1_i1	Glyma.08G265300.2	97.053407	169	7	245	3145	1	2892	0	4318

TRINITY_DN16499_c0_g1_i1	Glyma.08G265300.3	97.6284585	169	7	245	3145	1	2892	0	4318

TRINITY_DN16245_c0_g3_i5	Glyma.08G268900.1	100	400	21	213	3106	2976	86	0	2998

TRINITY_DN9796_c0_g1_i2	Glyma.01G207400.1	99.21259843	273	7	296	3187	2886	1	0	3657

TRINITY_DN16190_c0_g3_i2	Glyma.06G277500.1	97.72727273	240	7	271	3168	1	2901	0	3975

TRINITY_DN16266_c0_g2_i1	Glyma.18G286600.1	99.0990991	194	2	1	2903	793	3695	0	4276

TRINITY_DN13772_c0_g1_i1	Glyma.03G217500.1	98.30508475	188	0	654	3557	553	3456	0	4322

TRINITY_DN13772_c0_g1_i2	Glyma.03G217500.1	99.15611814	188	0	812	3715	553	3456	0	4322

TRINITY_DN16266_c0_g2_i1	Glyma.02G094800.1	97.45098039	186	2	1	2903	793	3695	0	4320

TRINITY_DN16190_c0_g3_i9	Glyma.06G229900.1	96.91358025	178	0	271	3174	1	2904	0	4377

TRINITY_DN16190_c0_g3_i9	Glyma.12G161300.1	97.6744186	172	0	271	3174	1	2904	0	4410

TRINITY_DN16332_c0_g3_i1	Glyma.06G042400.2	97.5308642	168	3	582	3485	2886	1	0	4316

TRINITY_DN16332_c0_g3_i1	Glyma.04G041300.2	97.5308642	161	2	582	3485	2898	1	0	4433

TRINITY_DN16175_c0_g1_i1	Glyma.01G197800.1	100	519	8	354	3252	142	3037	0	2414

TRINITY_DN16175_c0_g1_i4	Glyma.01G197800.1	98.85714286	519	8	338	3236	142	3037	0	2414

TRINITY_DN16175_c0_g1_i5	Glyma.01G197800.1	100	519	8	354	3252	142	3037	0	2414

TRINITY_DN16175_c0_g1_i6	Glyma.01G197800.1	97.24137931	519	8	338	3236	142	3037	0	2414

TRINITY_DN16190_c0_g3_i8	Glyma.06G277500.1	100	468	20	352	3245	13	2901	0	2619

TRINITY_DN16190_c0_g3_i8	Glyma.06G229900.1	96.19047619	304	11	348	3245	10	2904	0	3592

TRINITY_DN14721_c0_g1_i2	Glyma.18G058700.2	97.61904762	178	10	80	2970	5	2871	0	4065

TRINITY_DN14721_c0_g1_i2	Glyma.18G058700.3	98.79518072	178	11	80	2970	5	2862	0	4006

TRINITY_DN12563_c0_g1_i1	Glyma.05G221300.2	98.68421053	512	42	297	3186	2	2845	0	2071

TRINITY_DN13968_c0_g3_i4	Glyma.15G070100.1	95.66210046	496	35	1437	4317	1104	3981	0	2320

TRINITY_DN13968_c0_g3_i4	Glyma.15G070100.4	95.66160521	496	35	1437	4317	795	3672	0	2320

TRINITY_DN12658_c0_g1_i5	Glyma.05G206100.2	97.92387543	379	31	236	3120	7	2876	0	2942

TRINITY_DN16190_c0_g3_i4	Glyma.12G210600.1	97.5	350	2	339	3239	1	2901	0	3408

TRINITY_DN12547_c0_g2_i1	Glyma.14G154600.1	97.4025974	305	8	189	3082	357	3250	0	3567

TRINITY_DN12050_c0_g2_i1	Glyma.18G169600.1	100	289	19	148	3037	3040	5908	0	3467

TRINITY_DN12050_c0_g2_i1	Glyma.18G169600.2	100	289	19	148	3037	3040	5908	0	3467

TRINITY_DN12050_c0_g2_i1	Glyma.18G169600.3	99.24242424	289	19	148	3037	3040	5908	0	3467

TRINITY_DN12050_c0_g2_i1	Glyma.18G169600.4	100	289	19	148	3037	3040	5908	0	3467

TRINITY_DN12050_c0_g2_i1	Glyma.18G169600.5	100	289	19	148	3037	3040	5908	0	3467

TRINITY_DN14563_c0_g1_i5	Glyma.18G218900.2	100	245	36	1467	4338	2881	19	0	3555

TRINITY_DN12547_c0_g2_i2	Glyma.14G154600.1	100	237	8	189	3082	357	3250	0	3943

TRINITY_DN16285_c1_g1_i2	Glyma.05G178900.5	96.875	227	9	633	3527	1	2898	0	4030

TRINITY_DN16190_c0_g3_i8	Glyma.12G161300.1	96.19047619	303	7	348	3245	10	2904	0	3620

TRINITY_DN11875_c0_g1_i1	Glyma.18G005100.2	100	276	27	186	3041	1	2889	0	3459

TRINITY_DN11875_c0_g1_i1	Glyma.18G005100.1	100	276	27	186	3041	1	2889	0	3459

TRINITY_DN673_c0_g2_i1	Glyma.07G224000.3	97.99691834	190	2	19	2916	478	3378	0	4285

TRINITY_DN6023_c0_g1_i1	Glyma.20G135800.3	98.07692308	188	9	162	3049	1	2900	0	4226

TRINITY_DN6023_c0_g1_i1	Glyma.20G135800.2	98.07692308	188	9	162	3049	1	2900	0	4226

TRINITY_DN6023_c0_g1_i1	Glyma.20G135800.1	98.07692308	188	9	162	3049	1	2900	0	4226

TRINITY_DN6023_c0_g1_i1	Glyma.20G135800.5	98.07692308	188	10	162	3049	1	2897	0	4205

TRINITY_DN14091_c0_g1_i1	Glyma.07G119500.1	100	145	9	35	2917	1	2901	0	4438

TRINITY_DN15602_c0_g1_i1	Glyma.19G223400.2	96	132	5	900	3800	98	2991	0	4580

TRINITY_DN12547_c0_g2_i1	Glyma.13G032400.1	97.4025974	284	4	189	3084	162	3054	0	3711

TRINITY_DN12547_c0_g2_i2	Glyma.13G032400.1	100	225	4	189	3084	162	3054	0	4037

TRINITY_DN16285_c1_g1_i1	Glyma.05G178900.5	100	158	5	633	3527	1	2898	0	4434

TRINITY_DN14362_c0_g1_i1	Glyma.16G076500.1	100	339	15	220	3107	58	2945	0	3334

TRINITY_DN16033_c0_g1_i3	Glyma.10G154400.1	96.72131148	304	10	173	3064	2895	4	0	3579

TRINITY_DN16033_c0_g1_i3	Glyma.20G233900.1	100	301	10	173	3064	2892	1	0	3596

TRINITY_DN15531_c0_g1_i3	Glyma.06G017700.2	99.07120743	168	4	754	3650	1	2897	0	4397

TRINITY_DN15674_c0_g1_i5	Glyma.11G231100.1	100	515	14	29	2919	5021	2131	0	2409

TRINITY_DN16033_c0_g1_i4	Glyma.10G154400.1	100	341	9	173	3064	2895	4	0	3386

TRINITY_DN16033_c0_g1_i4	Glyma.20G233900.1	100	340	9	173	3064	2892	1	0	3391

TRINITY_DN8328_c0_g1_i2	Glyma.11G160300.1	97.61904762	199	8	388	3276	3351	463	0	4133

TRINITY_DN14091_c0_g1_i1	Glyma.03G104400.1	95.52238806	149	5	35	2917	1	2898	0	4429

TRINITY_DN16033_c0_g1_i2	Glyma.03G238100.2	98.7012987	348	18	400	3289	2892	18	0	3234

TRINITY_DN14362_c0_g1_i1	Glyma.16G109200.1	96.6442953	330	11	220	3107	58	2945	0	3406

TRINITY_DN14675_c0_g1_i1	Glyma.16G217300.1	100	292	5	457	3350	3162	278	0	3635

TRINITY_DN16033_c0_g1_i2	Glyma.19G235500.1	98.7012987	340	20	400	3289	2889	18	0	3271

TRINITY_DN16033_c0_g1_i1	Glyma.03G238100.2	96.03960396	307	16	400	3289	2892	18	0	3472

TRINITY_DN13573_c0_g1_i1	Glyma.01G123900.1	97.47292419	267	23	634	3513	2856	1	0	3507

TRINITY_DN9476_c0_g1_i3	Glyma.07G263100.1	100	197	2	334	3223	2919	24	0	4218

TRINITY_DN14146_c1_g1_i3	Glyma.19G242400.1	98.88888889	192	16	504	3383	2874	1	0	4039

TRINITY_DN16033_c0_g1_i1	Glyma.19G235500.1	96.03960396	297	18	400	3289	2889	18	0	3520

TRINITY_DN14245_c0_g2_i1	Glyma.13G181700.1	95.65217391	192	11	438	3308	1	2883	0	4050

TRINITY_DN12365_c0_g1_i1	Glyma.19G139800.1	96.84813754	189	0	191	3085	1	2895	0	4300

TRINITY_DN8328_c0_g1_i2	Glyma.18G053700.1	95.08196721	188	5	388	3276	3333	442	0	4246

TRINITY_DN12365_c0_g1_i1	Glyma.03G137100.1	95.45454545	183	0	191	3085	1	2895	0	4333

TRINITY_DN12794_c0_g1_i1	Glyma.15G176400.2	97.33009709	181	1	424	3315	2895	1	0	4324

TRINITY_DN14670_c0_g1_i3	Glyma.19G019100.1	100	291	18	530	3416	243	3096	0	3428

TRINITY_DN13573_c0_g1_i1	Glyma.03G051300.1	97.48953975	280	20	634	3513	2874	1	0	3574

TRINITY_DN4758_c0_g1_i1	Glyma.11G085900.4	100	438	15	632	3518	2876	1	0	2767

TRINITY_DN10067_c0_g4_i1	Glyma.17G061200.6	96.42857143	406	11	413	3301	1	2883	0	3003

TRINITY_DN10067_c0_g4_i1	Glyma.17G061200.3	99.3006993	406	11	413	3301	1	2883	0	3003

TRINITY_DN10067_c0_g4_i1	Glyma.17G061200.4	100	406	11	413	3301	1	2883	0	3003

TRINITY_DN10067_c0_g4_i1	Glyma.17G061200.1	100	406	11	413	3301	1	2883	0	3003

TRINITY_DN10067_c0_g4_i1	Glyma.17G061200.2	100	406	11	413	3301	1	2883	0	3003

TRINITY_DN10067_c0_g4_i1	Glyma.17G061200.5	98.71794872	406	11	413	3301	1	2883	0	3003

TRINITY_DN10067_c0_g4_i2	Glyma.17G061200.6	99.02912621	406	11	413	3301	1	2883	0	3003

TRINITY_DN10067_c0_g4_i2	Glyma.17G061200.3	100	406	11	413	3301	1	2883	0	3003

TRINITY_DN10067_c0_g4_i2	Glyma.17G061200.4	95.57522124	406	11	413	3301	1	2883	0	3003

TRINITY_DN10067_c0_g4_i2	Glyma.17G061200.1	100	406	11	413	3301	1	2883	0	3003

TRINITY_DN10067_c0_g4_i2	Glyma.17G061200.2	100	406	11	413	3301	1	2883	0	3003

TRINITY_DN10067_c0_g4_i2	Glyma.17G061200.5	96.01677149	406	11	413	3301	1	2883	0	3003

TRINITY_DN10067_c0_g4_i1	Glyma.13G098600.1	100	398	10	413	3301	1	2883	0	3048

TRINITY_DN10067_c0_g4_i2	Glyma.13G098600.1	98.8372093	398	10	413	3301	1	2883	0	3048

TRINITY_DN16268_c1_g9_i1	Glyma.19G027900.2	99.12280702	325	8	314	3201	2888	1	0	3478

TRINITY_DN16268_c1_g9_i1	Glyma.19G027900.1	95.51282051	325	8	314	3201	2888	1	0	3478

TRINITY_DN16268_c1_g9_i1	Glyma.13G058600.1	99.1202346	314	6	314	3201	2888	1	0	3539

TRINITY_DN5792_c0_g1_i1	Glyma.12G087000.1	95.94594595	302	4	270	3160	6621	3734	0	3624

TRINITY_DN15062_c0_g1_i2	Glyma.11G075100.4	96.1038961	217	35	375	3233	2862	1	0	3720

TRINITY_DN15062_c0_g1_i2	Glyma.11G075100.3	96.1038961	217	35	375	3233	2862	1	0	3720

TRINITY_DN15062_c0_g1_i2	Glyma.11G075100.2	96.1038961	217	35	375	3233	2862	1	0	3720

TRINITY_DN15062_c0_g1_i2	Glyma.11G075100.1	96.1038961	217	35	375	3233	2862	1	0	3720

TRINITY_DN14721_c0_g1_i1	Glyma.18G058700.1	97.6	179	7	80	2957	5	2874	0	4106

TRINITY_DN14721_c0_g1_i1	Glyma.18G058700.4	95.92875318	179	8	80	2957	5	2865	0	4047

TRINITY_DN14670_c0_g1_i3	Glyma.09G241100.1	95.58823529	136	2	530	3416	288	3180	0	4551

TRINITY_DN14670_c0_g1_i3	Glyma.09G241100.4	100	136	2	530	3416	288	3180	0	4551

TRINITY_DN14670_c0_g1_i3	Glyma.09G241100.2	97.78597786	136	2	530	3416	270	3162	0	4551

TRINITY_DN14670_c0_g1_i3	Glyma.09G241100.3	97.89473684	136	2	530	3416	270	3162	0	4551

TRINITY_DN15674_c0_g1_i5	Glyma.14G058300.1	99.58158996	530	14	35	2919	5015	2131	0	2314

TRINITY_DN15674_c0_g1_i5	Glyma.02G258200.1	100	518	14	35	2919	5015	2131	0	2381

TRINITY_DN10067_c0_g4_i1	Glyma.09G057400.2	98.52216749	138	1	413	3301	1	2892	0	4556

TRINITY_DN10067_c0_g4_i1	Glyma.09G057400.1	98.7654321	138	1	413	3301	1	2892	0	4556

TRINITY_DN10067_c0_g4_i2	Glyma.09G057400.2	95.21640091	138	1	413	3301	1	2892	0	4556

TRINITY_DN10067_c0_g4_i2	Glyma.09G057400.1	95.2173913	138	1	413	3301	1	2892	0	4556

TRINITY_DN14670_c0_g1_i3	Glyma.18G255000.1	96.96969697	136	3	531	3416	271	3162	0	4549

TRINITY_DN10067_c0_g4_i1	Glyma.15G163700.2	99.31740614	134	1	413	3301	1	2892	0	4578

TRINITY_DN10067_c0_g4_i1	Glyma.15G163700.1	95.34883721	134	1	413	3301	1	2892	0	4578

TRINITY_DN10067_c0_g4_i2	Glyma.15G163700.2	98.71794872	134	1	413	3301	1	2892	0	4578

TRINITY_DN10067_c0_g4_i2	Glyma.15G163700.1	98.50746269	134	1	413	3301	1	2892	0	4578

TRINITY_DN16420_c0_g1_i1	Glyma.01G040900.1	100	348	21	368	3240	232	3092	0	3101

TRINITY_DN16420_c0_g1_i1	Glyma.01G040900.2	97.39130435	348	21	368	3240	232	3092	0	3101

TRINITY_DN16420_c0_g1_i5	Glyma.01G040900.1	95.74468085	348	21	368	3240	232	3092	0	3101

TRINITY_DN16420_c0_g1_i5	Glyma.01G040900.2	97.5	348	21	368	3240	232	3092	0	3101

TRINITY_DN13968_c0_g3_i3	Glyma.15G070100.1	97.69820972	305	20	1437	4308	1104	3981	0	3443

TRINITY_DN13968_c0_g3_i3	Glyma.15G070100.4	97.03703704	305	20	1437	4308	795	3672	0	3443

TRINITY_DN8780_c0_g1_i1	Glyma.15G203600.1	95.65217391	244	17	135	3000	1	2881	0	3760

TRINITY_DN16268_c1_g14_i1	Glyma.19G027900.2	100	218	4	314	3201	2888	1	0	4093

TRINITY_DN16268_c1_g14_i1	Glyma.19G027900.1	99.58847737	218	4	314	3201	2888	1	0	4093

TRINITY_DN16268_c1_g14_i1	Glyma.13G058600.1	96.19205298	210	3	314	3201	2888	1	0	4137

TRINITY_DN14146_c1_g1_i3	Glyma.03G245000.1	98.88888889	186	14	509	3383	2878	1	0	4120

TRINITY_DN13718_c0_g1_i1	Glyma.03G121100.1	96.65653495	182	5	266	3151	2889	1	0	4285

TRINITY_DN15674_c0_g1_i5	Glyma.01G179300.1	98.50746269	169	0	29	2919	5018	2128	0	4403

TRINITY_DN15674_c0_g1_i5	Glyma.11G062900.1	100	162	0	29	2919	5018	2128	0	4442

TRINITY_DN16190_c0_g3_i2	Glyma.12G210600.1	98.7654321	399	3	283	3168	13	2901	0	3094

TRINITY_DN9717_c0_g1_i1	Glyma.08G244900.2	100	190	3	163	3051	2874	1	0	4174

TRINITY_DN9717_c0_g1_i1	Glyma.08G244900.3	100	190	3	163	3051	2874	1	0	4174

TRINITY_DN9717_c0_g1_i1	Glyma.08G244900.1	100	190	3	163	3051	2874	1	0	4174

TRINITY_DN14721_c0_g1_i1	Glyma.18G058700.2	99.43181818	179	6	80	2957	5	2871	0	4119

TRINITY_DN14721_c0_g1_i1	Glyma.18G058700.3	95.92760181	179	7	80	2957	5	2862	0	4061

TRINITY_DN13718_c0_g1_i1	Glyma.19G125800.1	97.8021978	167	3	266	3151	2889	1	0	4379

TRINITY_DN16022_c0_g2_i2	Glyma.13G266100.1	97.85932722	600	72	87	2936	67	2895	0	1371

TRINITY_DN16329_c1_g1_i1	Glyma.12G170100.1	98.32635983	215	0	1419	4307	2889	1	0	4145

TRINITY_DN16329_c1_g1_i1	Glyma.08G188000.1	98.34254144	196	0	1419	4307	2889	1	0	4250

TRINITY_DN16190_c0_g3_i4	Glyma.06G277500.1	97.5	144	1	352	3239	13	2901	0	4530

TRINITY_DN14245_c0_g2_i1	Glyma.15G072000.1	95.65217391	436	27	451	3308	14	2877	0	2569

TRINITY_DN14245_c0_g2_i1	Glyma.13G241400.1	95.65217391	433	44	451	3295	14	2870	0	2457

TRINITY_DN14245_c0_g2_i1	Glyma.13G241400.9	95.65217391	433	44	451	3295	14	2870	0	2457

TRINITY_DN13968_c0_g3_i1	Glyma.15G070100.1	99.34640523	400	17	1437	4311	1104	3981	0	2968

TRINITY_DN13968_c0_g3_i1	Glyma.15G070100.4	100	400	17	1437	4311	795	3672	0	2968

TRINITY_DN14675_c0_g1_i1	Glyma.09G167100.1	95.83333333	299	8	457	3332	3177	293	0	3579

TRINITY_DN9767_c0_g1_i1	Glyma.03G172200.1	98.66666667	227	11	288	3164	2877	1	0	3934

TRINITY_DN9767_c0_g1_i1	Glyma.03G172200.2	98.66666667	227	11	288	3164	2877	1	0	3934

TRINITY_DN9767_c0_g1_i1	Glyma.03G172200.3	97.26027397	227	11	288	3164	2877	1	0	3934

TRINITY_DN16314_c1_g2_i4	Glyma.16G165900.1	98.7804878	189	1	446	3330	3237	350	0	4266

TRINITY_DN16314_c1_g2_i2	Glyma.16G165900.1	100	165	4	446	3324	3237	350	0	4361

TRINITY_DN12000_c0_g1_i1	Glyma.08G366600.1	100	277	43	167	3019	2844	1	0	3299

TRINITY_DN12000_c0_g1_i1	Glyma.08G366600.2	100	275	47	167	3019	2832	1	0	3232

TRINITY_DN5141_c0_g1_i1	Glyma.08G258500.2	98.96907216	181	1	484	3367	381	3267	0	4309

TRINITY_DN5141_c0_g1_i1	Glyma.08G258500.1	99.45652174	181	1	484	3367	381	3267	0	4309

TRINITY_DN5141_c0_g1_i1	Glyma.18G282700.4	100	174	4	484	3367	384	3267	0	4329

TRINITY_DN5141_c0_g1_i1	Glyma.18G282700.1	95.89041096	174	4	484	3367	384	3267	0	4329

TRINITY_DN5141_c0_g1_i1	Glyma.18G282700.2	99.06976744	174	4	484	3367	384	3267	0	4329

TRINITY_DN5141_c0_g1_i1	Glyma.18G282700.3	97.4137931	174	4	484	3367	384	3267	0	4329

TRINITY_DN16094_c0_g1_i3	Glyma.11G004100.2	97.82608696	162	4	3	2889	2871	3	0	4318

TRINITY_DN16094_c0_g1_i7	Glyma.11G004100.2	96.53979239	162	4	3	2889	2871	3	0	4318

TRINITY_DN16151_c0_g2_i3	Glyma.01G038300.2	95.45454545	341	29	70	2914	82	2953	0	3085

TRINITY_DN5981_c0_g1_i1	Glyma.06G066200.5	95.83333333	323	12	687	3562	1	2865	0	3339

TRINITY_DN8780_c0_g1_i1	Glyma.09G097500.1	98.71794872	275	22	135	2995	1	2864	0	3502

TRINITY_DN9206_c0_g1_i1	Glyma.12G033700.1	96.1038961	249	4	358	3240	508	3384	0	3873

TRINITY_DN10980_c0_g1_i2	Glyma.12G002500.1	96.70468948	197	0	631	3516	1	2886	0	4239

TRINITY_DN10980_c0_g1_i2	Glyma.12G002500.2	100	197	0	631	3516	1	2886	0	4239

TRINITY_DN9767_c0_g1_i1	Glyma.19G173100.1	98.66666667	236	9	288	3164	2874	1	0	3908

TRINITY_DN15981_c0_g1_i15	Glyma.11G048300.2	100	192	6	724	3597	2884	1	0	4198

TRINITY_DN16022_c0_g2_i2	Glyma.12G233300.1	100	543	62	96	2955	73	2899	0	1805

TRINITY_DN16314_c1_g2_i1	Glyma.02G080900.1	100	243	13	446	3321	3237	371	0	3829

TRINITY_DN14326_c0_g4_i3	Glyma.11G074600.1	97.22222222	237	21	453	3321	131	2981	0	3714

TRINITY_DN16132_c0_g1_i4	Glyma.17G226100.2	100	231	10	712	3592	21	2892	0	3960

TRINITY_DN16314_c1_g2_i3	Glyma.02G080900.1	98.7854251	222	16	446	3315	3237	371	0	3906

TRINITY_DN14516_c0_g1_i1	Glyma.05G031900.1	95.82366589	133	8	174	3035	1	2880	0	4433

TRINITY_DN14516_c0_g1_i1	Glyma.17G094800.1	99.44444444	131	4	174	3035	1	2883	0	4462

TRINITY_DN14245_c0_g2_i3	Glyma.15G072000.1	95.65217391	425	22	393	3253	14	2877	0	2715

TRINITY_DN14245_c0_g2_i3	Glyma.13G241400.1	95.65217391	419	37	393	3240	14	2870	0	2619

TRINITY_DN14245_c0_g2_i3	Glyma.13G241400.9	95.65217391	419	37	393	3240	14	2870	0	2619

TRINITY_DN12658_c0_g1_i2	Glyma.05G206100.2	95.93495935	244	9	360	3226	1	2876	0	3845

TRINITY_DN15891_c1_g1_i1	Glyma.02G046800.1	100	238	8	1	2869	160	3037	0	3904

TRINITY_DN14245_c0_g2_i1	Glyma.07G194800.1	95.65217391	182	8	434	3308	3	2871	0	4196

TRINITY_DN1011_c0_g1_i1	Glyma.10G014100.1	97.5	293	45	1	2814	3416	579	0	2998

TRINITY_DN1011_c0_g1_i1	Glyma.10G014100.2	97.93103448	293	45	1	2814	3413	576	0	2998

TRINITY_DN16314_c1_g2_i1	Glyma.16G165900.1	100	236	7	446	3321	3237	371	0	3901

TRINITY_DN16314_c1_g2_i3	Glyma.16G165900.1	98.79518072	212	10	446	3315	3237	371	0	3995

TRINITY_DN16262_c0_g1_i3	Glyma.08G156400.1	100	338	23	61	2912	473	3339	0	3192

TRINITY_DN1011_c0_g1_i1	Glyma.02G013500.1	100	302	44	1	2814	3428	576	0	3055

TRINITY_DN14326_c0_g4_i3	Glyma.01G168900.1	98.92473118	252	17	453	3319	107	2955	0	3650

TRINITY_DN15891_c1_g1_i1	Glyma.16G126400.1	100	247	4	1	2869	160	3037	0	3877

TRINITY_DN326_c0_g1_i1	Glyma.03G253000.2	97.16312057	93	1	733	3608	555	3433	0	4782

TRINITY_DN16040_c0_g1_i4	Glyma.20G071200.1	97.22814499	581	45	397	3250	2851	1	0	1766

TRINITY_DN12973_c0_g1_i2	Glyma.03G057600.1	95.04761905	184	9	3	2876	1210	4074	0	4185

TRINITY_DN15645_c0_g1_i3	Glyma.05G209200.1	95.26627219	181	7	118	2982	79	2955	0	4222

TRINITY_DN16262_c0_g1_i3	Glyma.05G191200.1	97.48427673	342	25	61	2912	473	3339	0	3192

TRINITY_DN5408_c0_g1_i1	Glyma.12G083300.1	95.33333333	167	7	160	3026	2874	1	0	4303

TRINITY_DN15994_c0_g1_i1	Glyma.13G158800.1	97.09302326	677	52	1047	3881	2885	37	0	1122

TRINITY_DN16262_c0_g1_i7	Glyma.08G156400.1	99.62264151	249	17	61	2915	473	3339	0	3738

TRINITY_DN14245_c0_g2_i3	Glyma.07G194800.1	95.66473988	201	16	393	3253	20	2871	0	3945

TRINITY_DN15994_c0_g1_i1	Glyma.13G158800.2	96.0950764	677	51	1047	3881	2882	37	0	1123

TRINITY_DN16094_c0_g1_i1	Glyma.10G250500.3	98.27586207	267	5	282	3153	2868	3	0	3764

TRINITY_DN16094_c0_g1_i1	Glyma.10G250500.2	95.41463415	267	5	282	3153	2868	3	0	3764

TRINITY_DN16094_c0_g1_i6	Glyma.10G250500.3	97.4325214	267	5	282	3153	2868	3	0	3764

TRINITY_DN16094_c0_g1_i6	Glyma.10G250500.2	96.18320611	267	5	282	3153	2868	3	0	3764

TRINITY_DN16262_c0_g1_i7	Glyma.05G191200.1	100	262	17	61	2915	473	3339	0	3688

TRINITY_DN2306_c0_g1_i1	Glyma.01G063600.1	96.81528662	257	10	3	2853	2082	4950	0	3703

TRINITY_DN2306_c0_g1_i1	Glyma.01G063600.2	97.84366577	257	10	3	2853	2079	4947	0	3703

TRINITY_DN2306_c0_g1_i1	Glyma.01G063600.3	99.01960784	257	10	3	2853	1704	4572	0	3703

TRINITY_DN14062_c0_g1_i1	Glyma.09G194200.1	97.6744186	193	11	402	3263	3228	367	0	4084

TRINITY_DN14062_c0_g1_i1	Glyma.09G194200.3	100	191	15	402	3263	3213	367	0	3997

TRINITY_DN16158_c0_g1_i6	Glyma.20G102900.1	96.15384615	179	10	300	3172	3513	663	0	4161

TRINITY_DN16158_c0_g1_i6	Glyma.20G102900.2	96.15384615	179	10	300	3172	3513	663	0	4161

TRINITY_DN5408_c0_g1_i1	Glyma.11G191000.2	95.3307393	165	1	156	3026	2874	1	0	4373

TRINITY_DN5408_c0_g1_i1	Glyma.11G191000.1	95.33169533	165	1	156	3026	2874	1	0	4373

TRINITY_DN16158_c0_g1_i3	Glyma.09G284800.1	97.12389381	162	6	1133	3991	2853	1	0	4178

TRINITY_DN16158_c0_g1_i3	Glyma.20G001300.1	100	158	5	1133	3991	2871	1	0	4316

TRINITY_DN16158_c0_g1_i3	Glyma.20G001300.2	97.39130435	158	5	1133	3991	2871	1	0	4316

TRINITY_DN13778_c1_g3_i3	Glyma.19G081200.1	98.24561404	423	31	196	3031	2857	1	0	2619

TRINITY_DN12000_c0_g1_i1	Glyma.18G295800.4	100	286	26	167	3019	2847	1	0	3424

TRINITY_DN12000_c0_g1_i1	Glyma.18G295800.2	100	286	26	167	3019	2847	1	0	3424

TRINITY_DN12000_c0_g1_i1	Glyma.18G295800.1	100	286	26	167	3019	2847	1	0	3424

TRINITY_DN12000_c0_g1_i1	Glyma.18G295800.3	100	286	26	167	3019	2847	1	0	3424

TRINITY_DN16158_c0_g1_i6	Glyma.20G001300.1	96.15384615	275	6	300	3171	3531	685	0	3607

TRINITY_DN16158_c0_g1_i6	Glyma.20G001300.2	96.15384615	275	6	300	3171	3531	685	0	3607

TRINITY_DN16341_c0_g1_i2	Glyma.10G165900.1	100	247	20	172	3018	2871	1	0	3757

TRINITY_DN13629_c0_g2_i1	Glyma.08G099500.1	96.42857143	184	5	420	3292	3012	155	0	4189

TRINITY_DN13629_c0_g2_i2	Glyma.08G099500.1	96.51162791	184	5	341	3213	3012	155	0	4189

TRINITY_DN13629_c0_g2_i1	Glyma.08G099500.3	96.42857143	184	6	420	3292	3009	155	0	4170

TRINITY_DN13629_c0_g2_i1	Glyma.08G099500.2	96.42857143	184	6	420	3292	3009	155	0	4170

TRINITY_DN13629_c0_g2_i2	Glyma.08G099500.3	98.5915493	184	6	341	3213	3009	155	0	4170

TRINITY_DN13629_c0_g2_i2	Glyma.08G099500.2	98.21428571	184	6	341	3213	3009	155	0	4170

TRINITY_DN16158_c0_g1_i6	Glyma.10G286300.1	96.15384615	179	2	300	3172	3531	681	0	4172

TRINITY_DN15891_c1_g1_i1	Glyma.01G165000.1	100	470	21	1	2864	157	3008	0	2519

TRINITY_DN16158_c0_g1_i6	Glyma.09G284800.1	96.15384615	261	4	300	3171	3513	685	0	3579

TRINITY_DN15964_c0_g1_i6	Glyma.06G189900.1	98.5915493	250	2	226	3096	1	2871	0	3906

TRINITY_DN2306_c0_g1_i1	Glyma.02G121200.1	96.2962963	249	10	1	2853	1942	4800	0	3718

TRINITY_DN2306_c0_g1_i1	Glyma.02G121200.3	96.2962963	249	10	1	2853	1942	4800	0	3718

TRINITY_DN2306_c0_g1_i1	Glyma.02G121200.2	98.71794872	249	10	1	2853	1942	4800	0	3718

TRINITY_DN2306_c0_g1_i1	Glyma.02G121200.5	99.27007299	249	10	1	2853	1942	4800	0	3718

TRINITY_DN2306_c0_g1_i1	Glyma.02G121200.4	96.2962963	249	10	1	2853	1939	4797	0	3718

TRINITY_DN15964_c0_g1_i6	Glyma.04G174800.1	96.11650485	243	2	226	3096	1	2871	0	3945

TRINITY_DN15941_c1_g1_i1	Glyma.10G005700.1	96.41025641	215	22	37	2868	1	2865	0	3808

TRINITY_DN12254_c0_g1_i2	Glyma.19G082100.2	100	173	3	968	3835	1	2871	0	4313

TRINITY_DN15748_c0_g1_i2	Glyma.08G283700.4	96.0419092	158	1	1027	3898	3696	828	0	4409

TRINITY_DN15748_c0_g1_i3	Glyma.08G283700.4	100	158	1	384	3255	3696	828	0	4409

TRINITY_DN15964_c0_g1_i5	Glyma.06G189900.1	98.11320755	209	0	226	3096	1	2871	0	4145

TRINITY_DN15964_c0_g1_i5	Glyma.04G174800.1	97.05882353	201	0	226	3096	1	2871	0	4189

TRINITY_DN14062_c0_g1_i1	Glyma.09G194200.4	97.6744186	193	10	402	3263	3225	367	0	4096

TRINITY_DN14062_c0_g1_i1	Glyma.09G194200.2	96.75324675	191	14	402	3263	3210	367	0	4012

TRINITY_DN16004_c1_g1_i4	Glyma.09G056300.1	98.30508475	619	67	243	3062	208	3047	0	1354

TRINITY_DN16004_c1_g1_i4	Glyma.09G056300.2	98.30508475	619	67	243	3062	22	2861	0	1354

TRINITY_DN16040_c0_g1_i3	Glyma.20G071200.1	97.04142012	573	47	660	3504	2842	1	0	1783

TRINITY_DN15980_c0_g2_i1	Glyma.20G207700.1	99.45355191	492	65	3	2804	1030	3849	0	1812

TRINITY_DN15964_c0_g1_i5	Glyma.17G096100.1	95.57195572	222	3	226	3094	1	2866	0	4037

TRINITY_DN15964_c0_g1_i6	Glyma.17G096100.1	96.33027523	179	3	226	3094	1	2866	0	4276

TRINITY_DN265_c0_g1_i1	Glyma.06G086000.3	95.18072289	178	4	128	2993	27	2883	0	4204

TRINITY_DN265_c0_g1_i1	Glyma.06G086000.2	96.33911368	178	4	128	2993	27	2883	0	4204

TRINITY_DN16071_c0_g2_i3	Glyma.08G087300.1	99	423	28	200	3046	127	2985	0	2748

TRINITY_DN15964_c0_g1_i7	Glyma.04G174800.1	99.01639344	380	9	226	3089	1	2864	0	3129

TRINITY_DN15980_c0_g2_i2	Glyma.20G207700.2	99.07407407	248	9	1022	3884	865	3712	0	3749

TRINITY_DN15980_c0_g2_i2	Glyma.20G207700.4	99.07407407	248	9	1022	3884	865	3712	0	3749

TRINITY_DN16413_c0_g1_i2	Glyma.17G188300.2	98.82352941	241	6	255	3116	2865	1	0	3892

TRINITY_DN16413_c0_g1_i2	Glyma.17G188300.1	95.18072289	241	6	255	3116	2865	1	0	3892

TRINITY_DN15964_c0_g1_i8	Glyma.04G174800.1	97.6744186	338	7	226	3089	1	2864	0	3373

TRINITY_DN15964_c0_g1_i8	Glyma.17G096100.1	97.6744186	330	7	226	3089	1	2861	0	3397

TRINITY_DN15964_c0_g1_i8	Glyma.05G030700.2	97.24770642	326	7	226	3089	1	2861	0	3419

TRINITY_DN15964_c0_g1_i8	Glyma.05G030700.1	97.91666667	326	7	226	3089	1	2861	0	3419

TRINITY_DN15964_c0_g1_i7	Glyma.17G096100.1	100	287	7	226	3089	1	2861	0	3635

TRINITY_DN15964_c0_g1_i7	Glyma.05G030700.2	100	285	7	226	3089	1	2861	0	3646

TRINITY_DN15964_c0_g1_i7	Glyma.05G030700.1	98.75	285	7	226	3089	1	2861	0	3646

TRINITY_DN15980_c0_g2_i1	Glyma.10G182300.1	100	486	57	3	2804	1063	3882	0	1879

TRINITY_DN16132_c0_g1_i4	Glyma.06G056400.1	100	361	2	721	3586	84	2949	0	3282

TRINITY_DN16132_c0_g1_i4	Glyma.06G056400.4	99.19354839	361	2	721	3586	84	2949	0	3282

TRINITY_DN16132_c0_g1_i4	Glyma.06G056400.3	98.31932773	361	3	721	3586	84	2943	0	3243

TRINITY_DN14670_c0_g1_i3	Glyma.13G065500.6	99.2	284	18	530	3390	243	3067	0	3411

TRINITY_DN14670_c0_g1_i3	Glyma.13G065500.2	96.86706181	284	18	530	3390	177	3001	0	3411

TRINITY_DN14670_c0_g1_i3	Glyma.13G065500.4	97.18309859	284	19	530	3390	243	3058	0	3352

TRINITY_DN14670_c0_g1_i3	Glyma.13G065500.5	100	282	20	530	3390	243	3064	0	3402

TRINITY_DN14670_c0_g1_i3	Glyma.13G065500.3	96.88715953	282	21	530	3390	243	3055	0	3345

TRINITY_DN16071_c0_g2_i3	Glyma.05G132900.1	98.27586207	426	26	203	3046	130	2985	0	2726

TRINITY_DN15964_c0_g1_i7	Glyma.06G189900.1	100	398	4	226	3089	1	2864	0	3062

TRINITY_DN16071_c0_g2_i4	Glyma.08G087300.1	96.3963964	356	17	200	3055	127	2985	0	3210

TRINITY_DN39130_c0_g1_i1	Glyma.13G253300.1	96.52777778	243	8	146	3005	33	2895	0	3888

TRINITY_DN16413_c0_g1_i2	Glyma.14G139800.1	98.82352941	227	5	255	3116	2862	1	0	3984

TRINITY_DN15964_c0_g1_i8	Glyma.06G189900.1	99.0228013	357	2	226	3089	1	2864	0	3301

TRINITY_DN16071_c0_g2_i4	Glyma.05G132900.1	96.3963964	352	18	203	3055	130	2985	0	3216

TRINITY_DN14670_c0_g1_i2	Glyma.19G019100.1	97.61904762	272	11	530	3386	243	3096	0	3672

TRINITY_DN38330_c0_g1_i1	Glyma.10G118100.5	98.31932773	232	9	461	3316	88	2943	0	3901

TRINITY_DN15964_c0_g1_i5	Glyma.05G030700.2	98.11320755	221	1	226	3089	1	2861	0	4045

TRINITY_DN15964_c0_g1_i5	Glyma.05G030700.1	96.82539683	221	1	226	3089	1	2861	0	4045

TRINITY_DN15964_c0_g1_i6	Glyma.05G030700.2	96.11650485	180	1	226	3089	1	2861	0	4272

TRINITY_DN15964_c0_g1_i6	Glyma.05G030700.1	96.11650485	180	1	226	3089	1	2861	0	4272

TRINITY_DN14437_c0_g2_i1	Glyma.03G009000.1	95.52238806	551	67	1	2812	7	2815	0	1555

TRINITY_DN16086_c0_g2_i2	Glyma.15G162600.1	98.98477157	527	26	279	3126	21	2868	0	2185

TRINITY_DN15242_c0_g1_i1	Glyma.10G008700.4	100	312	22	221	3058	2898	55	0	3286

TRINITY_DN16262_c0_g1_i7	Glyma.04G213400.1	99.84126984	290	19	57	2915	469	3300	0	3465

TRINITY_DN16262_c0_g1_i7	Glyma.04G213400.2	100	288	21	57	2915	469	3297	0	3458

TRINITY_DN14062_c0_g1_i1	Glyma.01G146600.1	96.59763314	179	0	402	3263	3291	430	0	4294

TRINITY_DN14062_c0_g1_i1	Glyma.01G146600.2	96.20253165	179	0	402	3263	3225	364	0	4294

TRINITY_DN12519_c0_g1_i2	Glyma.20G017000.1	100	138	3	161	3016	1	2862	0	4482

TRINITY_DN12519_c0_g1_i1	Glyma.20G017000.1	97.60479042	137	4	161	3000	1	2862	0	4385

TRINITY_DN16086_c0_g2_i2	Glyma.09G056300.1	98.28178694	536	24	279	3126	207	3054	0	2146

TRINITY_DN16086_c0_g2_i2	Glyma.09G056300.2	98.98477157	536	24	279	3126	21	2868	0	2146

TRINITY_DN16086_c0_g2_i5	Glyma.15G162600.1	95	530	26	109	2955	22	2868	0	2167

TRINITY_DN11287_c0_g1_i1	Glyma.17G020600.1	99.03846154	179	16	242	3085	2853	1	0	4130

TRINITY_DN16086_c0_g2_i2	Glyma.13G369300.1	100	641	35	283	3124	7	2848	0	1498

TRINITY_DN13619_c0_g1_i3	Glyma.20G099500.1	100	426	32	1	2817	772	3621	0	2579

TRINITY_DN15639_c1_g1_i1	Glyma.U022000.2	95.05208333	227	12	551	3392	2859	3	0	3888

TRINITY_DN15639_c1_g1_i2	Glyma.U022000.2	97.14285714	227	12	675	3516	2859	3	0	3888

TRINITY_DN15639_c1_g1_i3	Glyma.U022000.2	98.0952381	227	12	358	3199	2859	3	0	3888

TRINITY_DN12591_c0_g1_i1	Glyma.16G084200.6	97.4595843	176	11	531	3375	2854	1	0	4170

TRINITY_DN12591_c0_g1_i2	Glyma.16G084200.6	98.03921569	176	11	503	3347	2854	1	0	4170

TRINITY_DN6075_c0_g1_i1	Glyma.16G101500.1	98.07692308	156	15	249	3086	2856	1	0	4250

TRINITY_DN14122_c0_g1_i1	Glyma.16G156200.1	97.68339768	357	19	224	3061	1	2853	0	3127

TRINITY_DN16086_c0_g2_i5	Glyma.14G134100.1	95	316	6	100	2955	1	2856	0	3491

TRINITY_DN16086_c0_g2_i5	Glyma.17G199200.1	100	312	6	100	2955	1	2856	0	3513

TRINITY_DN10917_c1_g1_i1	Glyma.04G192200.6	100	200	3	380	3237	1	2855	0	4139

TRINITY_DN10917_c1_g1_i1	Glyma.04G192200.2	100	200	3	380	3237	1	2855	0	4139

TRINITY_DN10917_c1_g1_i1	Glyma.15G230300.2	99.74747475	178	2	380	3237	1	2858	0	4281

TRINITY_DN7742_c0_g1_i1	Glyma.17G026600.2	100	154	0	124	2982	1	2859	0	4427

TRINITY_DN12519_c0_g1_i2	Glyma.07G218600.1	99.09090909	127	1	161	3016	1	2859	0	4556

TRINITY_DN12519_c0_g1_i1	Glyma.07G218600.1	100	127	2	161	3000	1	2859	0	4453

TRINITY_DN16086_c0_g2_i5	Glyma.09G056300.1	100	541	20	109	2955	208	3054	0	2139

TRINITY_DN16086_c0_g2_i5	Glyma.09G056300.2	98.10606061	541	20	109	2955	22	2868	0	2139

TRINITY_DN15980_c0_g2_i3	Glyma.20G207700.1	100	381	42	3	2822	1030	3849	0	2676

TRINITY_DN15980_c0_g2_i3	Glyma.10G182300.1	98.61111111	362	44	3	2822	1063	3882	0	2782

TRINITY_DN16086_c0_g2_i2	Glyma.14G134100.1	98.98373984	280	4	271	3126	1	2856	0	3701

TRINITY_DN16086_c0_g2_i2	Glyma.17G199200.1	99.57627119	275	4	271	3126	1	2856	0	3729

TRINITY_DN15941_c1_g1_i1	Glyma.02G003100.1	96.41025641	227	24	37	2868	1	2844	0	3762

TRINITY_DN12537_c0_g1_i1	Glyma.19G015900.1	95.27896996	204	1	3	2860	608	3459	0	4109

TRINITY_DN12537_c0_g1_i1	Glyma.13G068100.1	99.74683544	191	0	3	2860	602	3459	0	4220

TRINITY_DN12537_c0_g1_i1	Glyma.13G068100.2	98.0952381	191	0	3	2860	26	2883	0	4220

TRINITY_DN16086_c0_g2_i2	Glyma.06G076100.1	99.57446809	190	4	271	3126	1	2856	0	4200

TRINITY_DN15140_c0_g1_i1	Glyma.20G166200.1	100	179	17	1	2839	1242	4068	0	3964

TRINITY_DN15140_c0_g1_i2	Glyma.20G166200.1	100	179	17	1	2839	1242	4068	0	3964

TRINITY_DN16054_c1_g1_i5	Glyma.10G168700.1	97.87234043	142	0	557	3414	2858	1	0	4492

TRINITY_DN16054_c1_g1_i5	Glyma.20G221000.1	95.62682216	136	0	557	3414	2858	1	0	4525

TRINITY_DN5601_c0_g1_i1	Glyma.08G257900.1	98.02631579	274	24	172	3022	969	3786	0	3467

TRINITY_DN16262_c0_g1_i3	Glyma.04G213400.1	98.13829787	217	14	57	2912	469	3300	0	3906

TRINITY_DN16262_c0_g1_i3	Glyma.04G213400.2	95.49071618	215	16	57	2912	469	3297	0	3897

TRINITY_DN16086_c0_g2_i2	Glyma.04G075100.1	99.57446809	176	2	271	3126	1	2856	0	4289

TRINITY_DN16086_c0_g2_i5	Glyma.06G076100.1	100	153	2	100	2955	1	2856	0	4416

TRINITY_DN16262_c0_g1_i7	Glyma.06G152800.1	95.23809524	281	20	64	2915	476	3288	0	3413

TRINITY_DN5643_c0_g1_i2	Glyma.03G122400.1	100	156	7	150	2980	1	2832	0	4093

TRINITY_DN16086_c0_g2_i5	Glyma.04G075100.1	99.01380671	144	0	100	2955	1	2856	0	4477

TRINITY_DN16254_c0_g1_i6	Glyma.07G004300.1	99.16666667	671	64	397	3216	3207	394	0	1064

TRINITY_DN16254_c0_g1_i6	Glyma.07G004300.2	99.16666667	671	64	397	3216	3105	292	0	1064

TRINITY_DN14844_c0_g1_i2	Glyma.02G041400.5	99.03846154	375	19	1339	4170	2988	139	0	3025

TRINITY_DN14844_c0_g1_i2	Glyma.02G041400.4	99.18699187	373	20	1339	4170	2985	139	0	3016

TRINITY_DN10912_c0_g1_i1	Glyma.06G061600.1	98.41269841	279	27	1121	3936	2846	1	0	3428

TRINITY_DN15140_c0_g1_i1	Glyma.10G225800.1	100	171	13	1	2839	1218	4035	0	3993

TRINITY_DN15140_c0_g1_i2	Glyma.10G225800.1	97.41602067	171	13	1	2839	1218	4035	0	3993

TRINITY_DN15770_c0_g2_i1	Glyma.03G134200.1	100	413	26	479	3309	275	3114	0	2754

TRINITY_DN15770_c0_g2_i1	Glyma.03G134200.2	98.55072464	413	26	479	3309	275	3114	0	2754

TRINITY_DN10912_c0_g1_i1	Glyma.04G061000.1	96.07843137	282	22	1121	3936	2846	1	0	3422

TRINITY_DN14437_c0_g1_i2	Glyma.07G070500.1	98.63013699	281	13	1010	3838	1	2826	0	3382

TRINITY_DN390_c0_g1_i1	Glyma.12G027800.1	100	210	3	145	2997	1	2829	0	3951

TRINITY_DN16262_c0_g1_i3	Glyma.06G152800.1	95.09981851	202	19	64	2912	476	3288	0	3866

TRINITY_DN15242_c0_g1_i2	Glyma.10G008700.4	100	199	6	221	3064	2898	55	0	4050

TRINITY_DN16158_c0_g1_i2	Glyma.20G102900.1	98	181	2	300	3150	3513	663	0	4252

TRINITY_DN16158_c0_g1_i2	Glyma.20G102900.2	100	181	2	300	3150	3513	663	0	4252

TRINITY_DN16158_c0_g1_i2	Glyma.20G001300.1	100	275	3	300	3149	3531	685	0	3709

TRINITY_DN16158_c0_g1_i2	Glyma.20G001300.2	100	275	3	300	3149	3531	685	0	3709

TRINITY_DN7201_c0_g1_i1	Glyma.08G222400.1	100	201	1	741	3588	2851	1	0	4132

TRINITY_DN7201_c0_g1_i1	Glyma.07G004100.1	98.33333333	194	1	741	3588	2851	1	0	4170

TRINITY_DN16158_c0_g1_i2	Glyma.10G286300.1	100	180	0	300	3150	3531	681	0	4268

TRINITY_DN16086_c0_g2_i5	Glyma.13G369300.1	95.81151832	644	39	124	2953	19	2848	0	1437

TRINITY_DN16158_c0_g1_i2	Glyma.09G284800.1	98.27586207	261	1	300	3149	3513	685	0	3681

TRINITY_DN13071_c1_g2_i1	Glyma.08G081600.1	100	250	7	142	2988	1	2838	0	3781

TRINITY_DN13071_c1_g2_i1	Glyma.08G081600.2	98.75	250	7	142	2988	1	2838	0	3781

TRINITY_DN12399_c0_g1_i2	Glyma.02G040900.1	96.11650485	214	29	190	3018	1	2817	0	3729

TRINITY_DN16190_c0_g3_i7	Glyma.13G270400.4	100	400	14	413	3257	75	2904	0	2896

TRINITY_DN16190_c0_g3_i7	Glyma.13G270400.2	97.98994975	400	14	413	3257	75	2904	0	2896

TRINITY_DN16190_c0_g3_i7	Glyma.13G270400.3	96.15384615	400	14	413	3257	75	2904	0	2896

TRINITY_DN16190_c0_g3_i7	Glyma.13G270400.1	96.15931721	400	14	413	3257	75	2904	0	2896

TRINITY_DN16190_c0_g3_i7	Glyma.13G270400.5	97.38562092	400	14	413	3257	75	2904	0	2896

TRINITY_DN16168_c0_g1_i3	Glyma.08G057200.5	98.27586207	212	10	240	3087	3095	275	0	3899

TRINITY_DN16190_c0_g3_i7	Glyma.12G229400.3	97.36070381	388	12	413	3257	75	2904	0	2974

TRINITY_DN16190_c0_g3_i7	Glyma.12G229400.1	97.36070381	388	12	413	3257	75	2904	0	2974

TRINITY_DN16190_c0_g3_i7	Glyma.12G229400.2	99.50980392	388	12	413	3257	75	2904	0	2974

TRINITY_DN16094_c0_g1_i3	Glyma.10G250500.3	97.82608696	311	24	21	2844	2835	3	0	3284

TRINITY_DN16094_c0_g1_i3	Glyma.10G250500.2	97.82608696	311	24	21	2844	2835	3	0	3284

TRINITY_DN16094_c0_g1_i7	Glyma.10G250500.3	95.45454545	311	24	21	2844	2835	3	0	3284

TRINITY_DN16094_c0_g1_i7	Glyma.10G250500.2	95.45454545	311	24	21	2844	2835	3	0	3284

TRINITY_DN13071_c1_g2_i1	Glyma.05G126600.4	96.2962963	226	8	142	2988	1	2838	0	3936

TRINITY_DN13071_c1_g2_i1	Glyma.05G126600.2	100	226	8	142	2988	1	2838	0	3936

TRINITY_DN13071_c1_g2_i1	Glyma.05G126600.3	95.77464789	226	9	142	2988	1	2835	0	3917

TRINITY_DN4076_c0_g1_i1	Glyma.10G237900.1	98.73417722	223	0	1	2848	963	3810	0	4024

TRINITY_DN4076_c0_g1_i1	Glyma.20G156700.1	99.04761905	217	0	1	2848	960	3807	0	4058

TRINITY_DN16168_c0_g1_i3	Glyma.08G057200.2	97.61904762	212	10	241	3087	3094	275	0	3897

TRINITY_DN16168_c0_g1_i3	Glyma.08G057200.3	97.46136865	212	10	241	3087	3094	275	0	3897

TRINITY_DN16168_c0_g1_i3	Glyma.08G057200.1	98.55072464	212	10	241	3087	3094	275	0	3897

TRINITY_DN16168_c0_g1_i6	Glyma.08G057200.5	96.80511182	212	10	712	3558	3094	275	0	3897

TRINITY_DN16168_c0_g1_i6	Glyma.08G057200.1	96.80511182	212	10	712	3558	3094	275	0	3897

TRINITY_DN16168_c0_g1_i2	Glyma.08G057200.5	96.23655914	203	10	808	3654	3094	275	0	3947

TRINITY_DN16168_c0_g1_i2	Glyma.08G057200.1	96.82539683	203	10	808	3654	3094	275	0	3947

TRINITY_DN12674_c0_g2_i1	Glyma.08G360000.3	100	162	0	215	3062	2871	24	0	4362

TRINITY_DN12674_c0_g2_i1	Glyma.08G360000.1	99.12280702	162	0	215	3062	2871	24	0	4362

TRINITY_DN12674_c0_g2_i1	Glyma.08G360000.4	99.13793103	162	0	215	3062	2871	24	0	4362

TRINITY_DN12674_c0_g2_i1	Glyma.08G360000.2	99.13043478	162	0	215	3062	2871	24	0	4362

TRINITY_DN12674_c0_g2_i1	Glyma.08G360000.5	98.21428571	162	0	215	3062	2871	24	0	4362

TRINITY_DN9350_c0_g1_i1	Glyma.10G019200.1	100	272	9	154	2997	1	2835	0	3653

TRINITY_DN15562_c1_g1_i1	Glyma.08G148300.2	100	133	1	587	3432	1433	4279	0	4514

TRINITY_DN15886_c3_g1_i1	Glyma.17G039600.1	96.69421488	477	59	452	3249	81	2875	0	1973

TRINITY_DN15886_c3_g1_i3	Glyma.17G039600.1	95.38002981	477	59	661	3458	81	2875	0	1973

TRINITY_DN13619_c0_g1_i3	Glyma.10G289700.2	100	423	29	2	2817	788	3621	0	2641

TRINITY_DN13619_c0_g1_i3	Glyma.10G289700.1	100	423	29	2	2817	788	3621	0	2641

TRINITY_DN16004_c1_g1_i4	Glyma.13G369300.1	98.30508475	593	43	254	3062	17	2843	0	1620

TRINITY_DN15964_c0_g1_i7	Glyma.19G019200.1	100	383	17	255	3090	30	2865	0	3016

TRINITY_DN9350_c0_g1_i1	Glyma.02G155200.1	100	263	3	154	2997	1	2841	0	3764

TRINITY_DN14596_c0_g1_i1	Glyma.10G217200.2	100	203	8	455	3294	39	2875	0	4045

TRINITY_DN14596_c0_g1_i2	Glyma.10G217200.2	98.95833333	203	8	438	3277	39	2875	0	4045

TRINITY_DN16475_c0_g1_i1	Glyma.19G005800.1	97.98657718	203	15	27	2868	84	2880	0	3799

TRINITY_DN14437_c0_g1_i2	Glyma.18G233100.5	98.61386139	576	63	1010	3804	1	2802	0	1472

TRINITY_DN14437_c0_g1_i2	Glyma.18G233100.1	96.17486339	576	63	1010	3804	1	2802	0	1472

TRINITY_DN16086_c0_g2_i5	Glyma.13G097900.1	96.26168224	550	30	109	2942	22	2837	0	1960

TRINITY_DN16086_c0_g2_i2	Glyma.13G097900.1	95.6043956	547	30	280	3113	22	2837	0	1977

TRINITY_DN16004_c1_g1_i4	Glyma.17G103700.1	100	230	2	229	3069	1	2844	0	3958

TRINITY_DN15836_c0_g2_i2	Glyma.14G197100.1	98.8372093	219	25	80	2873	32	2867	0	3664

TRINITY_DN14596_c0_g1_i1	Glyma.20G174900.3	100	212	6	455	3294	39	2875	0	4006

TRINITY_DN14596_c0_g1_i1	Glyma.20G174900.2	100	212	6	455	3294	39	2875	0	4006

TRINITY_DN14596_c0_g1_i2	Glyma.20G174900.3	98.95833333	212	6	438	3277	39	2875	0	4006

TRINITY_DN14596_c0_g1_i2	Glyma.20G174900.2	98.95833333	212	6	438	3277	39	2875	0	4006

TRINITY_DN16004_c1_g1_i4	Glyma.13G156000.1	100	210	2	229	3069	1	2844	0	4069

TRINITY_DN12687_c0_g2_i1	Glyma.19G004000.8	97.2972973	207	24	150	2948	1	2838	0	3775

TRINITY_DN12687_c0_g2_i1	Glyma.19G004000.5	97.2972973	207	24	150	2948	1	2838	0	3775

TRINITY_DN12687_c0_g2_i1	Glyma.19G004000.7	97.2826087	207	24	150	2948	1	2838	0	3775

TRINITY_DN12687_c0_g2_i1	Glyma.19G004000.6	97.2972973	207	24	150	2948	1	2838	0	3775

TRINITY_DN14051_c0_g1_i2	Glyma.09G279500.1	96.88581315	193	19	185	3011	2850	24	0	3964

TRINITY_DN16086_c0_g2_i2	Glyma.17G061800.1	98.21428571	547	22	280	3113	22	2849	0	2065

TRINITY_DN16086_c0_g2_i5	Glyma.17G061800.1	96.33027523	546	22	109	2942	22	2849	0	2071

TRINITY_DN16151_c0_g2_i7	Glyma.08G292900.3	95.53956835	412	38	1638	4450	1231	4034	0	2521

TRINITY_DN15934_c0_g1_i1	Glyma.10G212600.3	96.05263158	250	10	954	3784	9	2849	0	3775

TRINITY_DN15645_c0_g1_i3	Glyma.05G209200.2	98.24561404	176	7	153	2982	81	2922	0	4185

TRINITY_DN15645_c0_g1_i4	Glyma.05G209200.2	95.50561798	176	7	150	2979	81	2922	0	4185

TRINITY_DN15964_c0_g1_i6	Glyma.19G019200.1	100	535	13	255	3089	30	2864	0	2194

TRINITY_DN15964_c0_g1_i8	Glyma.19G019200.1	99.0228013	392	11	255	3090	30	2865	0	3000

TRINITY_DN16298_c0_g1_i2	Glyma.12G098900.1	98.95833333	333	5	345	3183	2955	117	0	3365

TRINITY_DN1011_c0_g1_i1	Glyma.02G013500.3	99.01960784	301	44	38	2814	3391	576	0	2992

TRINITY_DN5747_c0_g1_i1	Glyma.13G235600.1	96.875	166	14	242	3055	169	3009	0	4141

TRINITY_DN15964_c0_g1_i5	Glyma.19G019200.1	97.56097561	540	11	255	3089	30	2864	0	2178

TRINITY_DN14042_c0_g1_i1	Glyma.13G332300.10	98.7804878	201	9	446	3268	1	2838	0	3997

TRINITY_DN14042_c0_g1_i1	Glyma.13G332300.8	98.7804878	201	9	446	3268	1	2838	0	3997

TRINITY_DN14042_c0_g1_i1	Glyma.13G332300.1	98.7804878	201	9	446	3268	1	2838	0	3997

TRINITY_DN14042_c0_g1_i3	Glyma.13G332300.10	100	201	9	663	3485	1	2838	0	3997

TRINITY_DN14042_c0_g1_i3	Glyma.13G332300.8	95.83333333	201	9	663	3485	1	2838	0	3997

TRINITY_DN14042_c0_g1_i3	Glyma.13G332300.1	98.05194805	201	9	663	3485	1	2838	0	3997

TRINITY_DN16298_c0_g1_i1	Glyma.12G098900.1	97.2972973	197	3	297	3135	2955	117	0	4130

TRINITY_DN12892_c0_g4_i5	Glyma.11G103100.2	100	197	4	230	3067	34	2862	0	4058

TRINITY_DN12892_c0_g4_i5	Glyma.11G103100.3	100	197	4	230	3067	34	2862	0	4058

TRINITY_DN12892_c0_g4_i5	Glyma.11G102600.1	95.83333333	195	4	230	3067	34	2862	0	4069

TRINITY_DN12892_c0_g4_i5	Glyma.11G102600.2	95.83333333	195	4	230	3067	34	2862	0	4069

TRINITY_DN5894_c0_g1_i1	Glyma.03G237200.1	96.2962963	169	1	144	2982	5979	3139	0	4298

TRINITY_DN5894_c0_g1_i1	Glyma.03G237200.2	100	169	1	144	2982	5931	3091	0	4298

TRINITY_DN5643_c0_g1_i2	Glyma.03G122400.2	100	156	6	150	2980	1	2817	0	4163

TRINITY_DN5643_c0_g1_i2	Glyma.19G126500.1	98.25581395	151	7	150	2980	1	2817	0	4191

TRINITY_DN16190_c0_g3_i8	Glyma.13G270400.4	96	431	13	413	3245	75	2904	0	2758

TRINITY_DN16190_c0_g3_i8	Glyma.13G270400.2	100	431	13	413	3245	75	2904	0	2758

TRINITY_DN16190_c0_g3_i8	Glyma.13G270400.3	100	431	13	413	3245	75	2904	0	2758

TRINITY_DN16190_c0_g3_i8	Glyma.13G270400.1	100	431	13	413	3245	75	2904	0	2758

TRINITY_DN16190_c0_g3_i8	Glyma.13G270400.5	100	431	13	413	3245	75	2904	0	2758

TRINITY_DN16190_c0_g3_i8	Glyma.12G229400.3	100	420	14	413	3245	75	2904	0	2819

TRINITY_DN16190_c0_g3_i8	Glyma.12G229400.1	100	420	14	413	3245	75	2904	0	2819

TRINITY_DN16190_c0_g3_i8	Glyma.12G229400.2	97.5	420	14	413	3245	75	2904	0	2819

TRINITY_DN14437_c0_g1_i2	Glyma.03G009000.1	98.63013699	279	14	1010	3826	1	2817	0	3413

TRINITY_DN5901_c0_g1_i1	Glyma.05G241600.1	97.70114943	219	6	189	3025	1	2834	0	3984

TRINITY_DN5901_c0_g1_i1	Glyma.05G241600.3	100	219	6	189	3025	1	2834	0	3984

TRINITY_DN5901_c0_g1_i1	Glyma.05G241600.2	100	219	6	189	3025	1	2834	0	3984

TRINITY_DN5901_c0_g2_i1	Glyma.05G241600.1	100	206	6	189	3025	1	2834	0	4056

TRINITY_DN5901_c0_g2_i1	Glyma.05G241600.3	100	206	6	189	3025	1	2834	0	4056

TRINITY_DN5901_c0_g2_i1	Glyma.05G241600.2	100	206	6	189	3025	1	2834	0	4056

TRINITY_DN11126_c0_g1_i1	Glyma.17G183400.1	96.875	152	0	441	3279	3509	671	0	4401

TRINITY_DN16043_c0_g2_i3	Glyma.18G129200.1	97.56097561	144	3	871	3708	1	2832	0	4394

TRINITY_DN11126_c0_g1_i1	Glyma.13G192600.1	96.875	143	0	441	3279	3509	671	0	4451

TRINITY_DN14437_c0_g1_i2	Glyma.09G259200.3	98.63013699	569	58	1010	3804	1	2799	0	1559

TRINITY_DN14437_c0_g1_i2	Glyma.09G259200.2	98.63013699	569	58	1010	3804	1	2799	0	1559

TRINITY_DN14437_c0_g1_i2	Glyma.09G259200.1	98.63013699	569	58	1010	3804	1	2799	0	1559

TRINITY_DN16158_c0_g1_i3	Glyma.20G102900.1	100	358	7	1157	3991	2829	1	0	3192

TRINITY_DN16158_c0_g1_i3	Glyma.20G102900.2	97.32620321	358	7	1157	3991	2829	1	0	3192

TRINITY_DN14670_c0_g1_i2	Glyma.13G065500.6	97.61904762	262	10	530	3360	243	3067	0	3672

TRINITY_DN14670_c0_g1_i2	Glyma.13G065500.2	97.61904762	262	10	530	3360	177	3001	0	3672

TRINITY_DN14670_c0_g1_i2	Glyma.13G065500.4	95.95375723	262	11	530	3360	243	3058	0	3613

TRINITY_DN14670_c0_g1_i2	Glyma.13G065500.5	95.95375723	260	12	530	3360	243	3064	0	3663

TRINITY_DN14670_c0_g1_i2	Glyma.13G065500.3	95.83333333	260	13	530	3360	243	3055	0	3605

TRINITY_DN16274_c0_g1_i1	Glyma.U010500.1	95.8073149	142	5	696	3514	209	3045	0	4337

TRINITY_DN16332_c0_g3_i1	Glyma.17G239500.2	97.52650177	313	10	840	3670	2832	5	0	3419

TRINITY_DN12043_c0_g2_i1	Glyma.10G005000.1	98.33679834	273	24	320	3137	158	2958	0	3382

TRINITY_DN14062_c0_g1_i2	Glyma.09G194200.1	99.33665008	204	13	1104	3924	2824	1	0	3932

TRINITY_DN14062_c0_g1_i2	Glyma.09G194200.3	99.38650307	202	17	1104	3924	2809	1	0	3847

TRINITY_DN14042_c0_g1_i1	Glyma.15G042000.8	95.51282051	183	9	446	3268	1	2826	0	4074

TRINITY_DN14042_c0_g1_i1	Glyma.15G042000.1	95.38461538	183	9	446	3268	1	2826	0	4074

TRINITY_DN14042_c0_g1_i3	Glyma.15G042000.8	95.88068182	183	9	663	3485	1	2826	0	4074

TRINITY_DN14042_c0_g1_i3	Glyma.15G042000.1	100	183	9	663	3485	1	2826	0	4074

TRINITY_DN5643_c0_g1_i1	Glyma.03G122400.1	100	156	6	150	2960	1	2832	0	4185

TRINITY_DN13807_c0_g2_i1	Glyma.01G193600.2	98.57142857	619	47	166	2966	1	2807	0	1406

TRINITY_DN6117_c0_g2_i1	Glyma.09G084300.1	95.58359621	183	2	2	2833	1069	3900	0	4183

TRINITY_DN6117_c0_g2_i1	Glyma.09G084300.3	99.5215311	183	2	2	2833	769	3600	0	4183

TRINITY_DN15709_c0_g1_i1	Glyma.10G090000.1	95.50561798	205	2	181	3013	73	2905	0	4085

TRINITY_DN14064_c0_g1_i5	Glyma.13G209800.1	96.90721649	152	1	1676	4506	2852	19	0	4372

TRINITY_DN14064_c0_g1_i5	Glyma.15G102800.1	96.90721649	151	1	1676	4506	2834	1	0	4377

TRINITY_DN14062_c0_g1_i2	Glyma.09G194200.4	98.7804878	204	12	1104	3924	2821	1	0	3947

TRINITY_DN14062_c0_g1_i2	Glyma.09G194200.2	97.82608696	202	16	1104	3924	2806	1	0	3860

TRINITY_DN16094_c0_g1_i6	Glyma.11G004100.1	100	184	4	567	3395	2943	113	0	4174

TRINITY_DN6117_c0_g2_i1	Glyma.15G192700.1	97.69230769	166	2	2	2833	595	3426	0	4300

TRINITY_DN11794_c0_g1_i1	Glyma.10G153000.1	97.84366577	198	23	256	3051	2826	1	0	3862

TRINITY_DN11794_c0_g1_i1	Glyma.10G153000.2	98.61111111	197	26	256	3051	2814	1	0	3790

TRINITY_DN15457_c0_g1_i1	Glyma.13G161600.2	98.33333333	195	2	474	3302	1	2832	0	4130

TRINITY_DN6117_c0_g2_i1	Glyma.09G084300.4	98.41269841	183	1	2	2833	1069	3897	0	4196

TRINITY_DN6117_c0_g2_i1	Glyma.09G084300.2	98.58156028	183	1	2	2833	769	3597	0	4196

TRINITY_DN14437_c0_g1_i2	Glyma.18G233100.3	98.64253394	582	52	1010	3804	1	2790	0	1506

TRINITY_DN14437_c0_g1_i2	Glyma.18G233100.4	98.64253394	582	52	1010	3804	1	2790	0	1506

TRINITY_DN15808_c0_g1_i1	Glyma.05G022300.2	98.41269841	221	21	2636	5441	2522	5322	0	3648

TRINITY_DN15808_c0_g1_i2	Glyma.05G022300.2	100	221	21	2758	5563	2522	5322	0	3648

TRINITY_DN16440_c0_g1_i2	Glyma.01G112300.1	98.21428571	488	33	560	3353	3773	953	0	2231

TRINITY_DN16222_c0_g1_i3	Glyma.03G206400.1	95.20295203	212	23	168	2986	1	2780	0	3657

TRINITY_DN16222_c0_g1_i2	Glyma.03G206400.1	99.5049505	211	23	155	2973	1	2780	0	3663

TRINITY_DN16222_c0_g1_i6	Glyma.03G206400.1	95.54317549	211	23	168	2986	1	2780	0	3663

TRINITY_DN16222_c0_g1_i1	Glyma.03G206400.1	98.93992933	210	23	155	2973	1	2780	0	3668

TRINITY_DN12973_c0_g1_i2	Glyma.01G121400.1	99.15878023	192	8	66	2876	1258	4083	0	4013

TRINITY_DN16314_c1_g1_i3	Glyma.02G080900.1	100	567	42	707	3514	3233	435	0	1753

TRINITY_DN16314_c1_g2_i2	Glyma.08G145600.1	100	549	45	449	3239	3285	477	0	1808

TRINITY_DN16314_c1_g2_i3	Glyma.05G187300.1	98.78787879	546	38	449	3249	3285	479	0	1877

TRINITY_DN8943_c0_g1_i1	Glyma.20G089300.5	100	330	9	741	3552	2827	1	0	3273

TRINITY_DN16254_c0_g1_i6	Glyma.17G057800.1	99.16666667	330	10	406	3215	3186	362	0	3247

TRINITY_DN15345_c0_g1_i1	Glyma.12G194200.2	100	244	4	388	3210	17	2844	0	3827

TRINITY_DN15345_c0_g1_i1	Glyma.12G194200.1	100	244	4	388	3210	17	2844	0	3827

TRINITY_DN15345_c0_g1_i2	Glyma.12G194200.2	99.13793103	244	4	194	3016	17	2844	0	3827

TRINITY_DN15345_c0_g1_i2	Glyma.12G194200.1	100	244	4	194	3016	17	2844	0	3827

TRINITY_DN15345_c0_g1_i1	Glyma.13G308400.5	96.19651347	230	4	388	3210	17	2844	0	3904

TRINITY_DN15345_c0_g1_i1	Glyma.13G308400.2	97.14285714	230	4	388	3210	17	2844	0	3904

TRINITY_DN15345_c0_g1_i1	Glyma.13G308400.1	97.24770642	230	4	388	3210	17	2844	0	3904

TRINITY_DN15345_c0_g1_i2	Glyma.13G308400.5	98.11320755	230	4	194	3016	17	2844	0	3904

TRINITY_DN15345_c0_g1_i2	Glyma.13G308400.2	98.68852459	230	4	194	3016	17	2844	0	3904

TRINITY_DN15345_c0_g1_i2	Glyma.13G308400.1	99.13793103	230	4	194	3016	17	2844	0	3904

TRINITY_DN11794_c0_g1_i1	Glyma.20G235300.1	97.84366577	195	15	256	3051	2823	1	0	3892

TRINITY_DN5816_c0_g1_i1	Glyma.17G121400.1	96.9924812	147	2	90	2906	1	2829	0	4333

TRINITY_DN16314_c1_g2_i1	Glyma.05G187300.1	100	554	32	449	3255	3285	479	0	1882

TRINITY_DN16314_c1_g1_i3	Glyma.16G165900.1	100	551	40	707	3514	3233	435	0	1853

TRINITY_DN16314_c1_g2_i2	Glyma.05G187300.1	98.01324503	541	42	449	3239	3285	477	0	1864

TRINITY_DN15808_c0_g1_i1	Glyma.17G077400.1	97.9020979	222	21	2636	5441	2531	5328	0	3657

TRINITY_DN15808_c0_g1_i2	Glyma.17G077400.1	98.16513761	222	21	2758	5563	2531	5328	0	3657

TRINITY_DN16158_c0_g1_i5	Glyma.20G001300.3	100	157	6	1075	3887	2803	1	0	4095

TRINITY_DN16314_c1_g2_i4	Glyma.08G145600.1	98.7804878	560	37	449	3245	3285	477	0	1808

TRINITY_DN16314_c1_g2_i4	Glyma.05G187300.1	98.78787879	549	36	449	3245	3285	477	0	1869

TRINITY_DN16169_c0_g1_i4	Glyma.20G242400.1	96.44839068	328	15	1954	4772	2789	1	0	3107

TRINITY_DN16169_c0_g1_i4	Glyma.20G242400.2	100	328	15	1954	4772	2789	1	0	3107

TRINITY_DN16314_c1_g2_i3	Glyma.08G145600.1	98.79518072	562	34	449	3249	3285	479	0	1821

TRINITY_DN14341_c0_g1_i1	Glyma.03G034200.1	98	457	19	225	3039	87	2903	0	2558

TRINITY_DN14654_c0_g1_i5	Glyma.09G194900.1	96.96969697	319	15	1	2816	2977	165	0	3302

TRINITY_DN15674_c0_g1_i3	Glyma.02G228400.2	95.27272727	263	0	605	3430	4953	2128	0	3762

TRINITY_DN15674_c0_g1_i5	Glyma.02G228400.2	100	223	0	94	2919	4953	2128	0	3984

TRINITY_DN15981_c0_g1_i15	Glyma.01G193600.2	100	187	1	772	3594	2826	1	0	4163

TRINITY_DN10733_c0_g1_i1	Glyma.05G212500.1	97.22222222	180	1	220	3042	1	2826	0	4202

TRINITY_DN10733_c0_g1_i1	Glyma.08G018900.1	97.22222222	176	1	220	3042	1	2826	0	4224

TRINITY_DN16245_c0_g2_i1	Glyma.05G006800.2	95.65217391	163	0	318	3143	2865	40	0	4316

TRINITY_DN16245_c0_g2_i1	Glyma.19G007400.2	96.58536585	159	4	318	3143	2880	70	0	4241

TRINITY_DN16169_c0_g1_i4	Glyma.10G292400.3	98.55072464	322	14	1954	4772	2792	1	0	3181

TRINITY_DN16169_c0_g1_i4	Glyma.10G292400.1	99.47460595	322	14	1954	4772	2792	1	0	3181

TRINITY_DN15242_c0_g1_i1	Glyma.10G008700.2	97.63033175	308	24	221	3021	3375	571	0	3227

TRINITY_DN16274_c0_g1_i1	Glyma.U010500.2	98.21428571	144	2	696	3514	209	3033	0	4381

TRINITY_DN16314_c1_g2_i1	Glyma.08G145600.1	100	573	26	449	3255	3285	479	0	1821

TRINITY_DN16325_c0_g1_i2	Glyma.19G016600.1	100	337	12	381	3199	3760	948	0	3245

TRINITY_DN16325_c0_g1_i3	Glyma.19G016600.1	100	334	12	198	3016	3760	948	0	3262

TRINITY_DN15163_c0_g1_i1	Glyma.07G159300.1	95.08196721	233	25	275	3070	259	3060	0	3602

TRINITY_DN16297_c0_g2_i1	Glyma.01G232800.1	100	236	11	434	3246	1	2798	0	3679

TRINITY_DN14598_c0_g2_i1	Glyma.14G223100.10	95.23809524	222	28	480	3247	2795	1	0	3446

TRINITY_DN14598_c0_g2_i1	Glyma.14G223100.1	100	222	28	480	3247	2795	1	0	3446

TRINITY_DN14598_c0_g2_i1	Glyma.14G223100.8	98.17351598	220	29	480	3247	2783	1	0	3380

TRINITY_DN14598_c0_g2_i1	Glyma.14G223100.9	98.77300613	220	29	480	3247	2783	1	0	3380

TRINITY_DN15618_c0_g1_i2	Glyma.17G228800.2	96.875	178	0	508	3330	3077	255	0	4228

TRINITY_DN15618_c0_g1_i2	Glyma.17G228800.2	96.55172414	178	0	3307	6129	255	3077	0	4228

TRINITY_DN14287_c0_g1_i1	Glyma.07G260500.1	100	421	18	385	3190	3249	435	0	2730

TRINITY_DN6168_c0_g1_i1	Glyma.20G227000.3	96	216	12	109	2917	22	2821	0	3788

TRINITY_DN16440_c0_g1_i2	Glyma.03G077400.1	97.14285714	456	31	560	3353	3380	575	0	2412

TRINITY_DN14341_c0_g1_i1	Glyma.01G133300.1	99.6031746	439	13	225	3039	87	2900	0	2693

TRINITY_DN14341_c0_g1_i1	Glyma.01G133300.2	98.87640449	439	13	225	3039	87	2900	0	2693

TRINITY_DN14654_c0_g1_i5	Glyma.16G111200.3	98.7804878	327	21	11	2816	2970	165	0	3205

TRINITY_DN14654_c0_g1_i5	Glyma.16G111200.2	98.7804878	327	21	11	2816	2970	165	0	3205

TRINITY_DN14654_c0_g1_i5	Glyma.16G111200.1	98.7804878	327	21	11	2816	2970	165	0	3205

TRINITY_DN12265_c0_g2_i1	Glyma.16G073200.1	95.71428571	247	5	164	2983	1	2814	0	3790

TRINITY_DN15549_c0_g1_i6	Glyma.05G247000.3	100	201	14	837	3626	2814	1	0	3851

TRINITY_DN15016_c0_g1_i1	Glyma.10G234100.1	97.54098361	194	20	295	3093	1	2796	0	3831

TRINITY_DN14062_c0_g1_i2	Glyma.01G146600.2	98.52941176	189	0	1104	3924	2821	1	0	4163

TRINITY_DN15981_c0_g1_i5	Glyma.11G048300.1	98.7854251	171	3	427	3241	3051	231	0	4224

TRINITY_DN5643_c0_g1_i1	Glyma.03G122400.2	100	156	5	150	2960	1	2817	0	4255

TRINITY_DN5643_c0_g1_i1	Glyma.19G126500.1	100	151	6	150	2960	1	2817	0	4283

TRINITY_DN15981_c0_g1_i5	Glyma.17G161300.1	99.23664122	457	10	427	3241	3045	231	0	2612

TRINITY_DN15981_c0_g1_i5	Glyma.05G105200.1	100	441	9	427	3241	3045	231	0	2700

TRINITY_DN13002_c0_g1_i1	Glyma.03G202000.1	99.56521739	328	6	153	2965	23	2838	0	3319

TRINITY_DN13063_c0_g2_i3	Glyma.17G035000.1	99.01960784	234	8	61	2874	1	2808	0	3795

TRINITY_DN13063_c0_g2_i3	Glyma.17G035000.2	99.01960784	234	9	61	2874	1	2802	0	3757

TRINITY_DN6075_c0_g1_i1	Glyma.08G266300.2	96.59863946	153	11	249	3044	2874	58	0	4185

TRINITY_DN6075_c0_g1_i1	Glyma.08G266300.1	98.07692308	153	11	249	3044	2874	58	0	4185

TRINITY_DN16247_c0_g1_i3	Glyma.12G237000.1	95.89041096	142	2	1	2808	2817	1	0	4324

TRINITY_DN16247_c0_g1_i3	Glyma.13G202500.1	95	140	1	1	2808	2820	1	0	4355

TRINITY_DN16247_c0_g1_i3	Glyma.13G202500.2	95	140	1	1	2808	2820	1	0	4355

TRINITY_DN12290_c0_g1_i2	Glyma.19G140000.1	100	533	23	464	3263	2953	145	0	2065

TRINITY_DN14670_c0_g1_i3	Glyma.19G019100.2	97.18309859	281	18	605	3416	3	2781	0	3345

TRINITY_DN13629_c0_g2_i1	Glyma.08G099500.4	96.42857143	181	5	474	3292	2958	155	0	4106

TRINITY_DN13629_c0_g2_i1	Glyma.08G099500.5	96.42857143	181	5	474	3292	2958	155	0	4106

TRINITY_DN13629_c0_g2_i2	Glyma.08G099500.4	97.98994975	181	5	395	3213	2958	155	0	4106

TRINITY_DN13629_c0_g2_i2	Glyma.08G099500.5	95.65217391	181	5	395	3213	2958	155	0	4106

TRINITY_DN8943_c0_g1_i1	Glyma.10G140500.2	100	341	11	741	3540	3709	895	0	3179

TRINITY_DN12687_c0_g2_i1	Glyma.19G004000.1	97.2972973	206	23	150	2922	1	2814	0	3746

TRINITY_DN12687_c0_g2_i1	Glyma.19G004000.2	97.2972973	206	23	150	2922	1	2814	0	3746

TRINITY_DN12687_c0_g2_i1	Glyma.19G004000.4	97.28183119	206	23	150	2922	1	2814	0	3746

TRINITY_DN12687_c0_g2_i1	Glyma.19G004000.3	97.2972973	206	23	150	2922	1	2814	0	3746

TRINITY_DN16274_c0_g1_i2	Glyma.12G095300.3	97.91666667	168	22	503	3296	1	2797	0	3982

TRINITY_DN16274_c0_g1_i2	Glyma.12G095300.1	100	168	22	503	3296	1	2797	0	3982

TRINITY_DN16254_c0_g1_i6	Glyma.08G222200.1	99.16666667	639	67	397	3170	3213	443	0	1086

TRINITY_DN16254_c0_g1_i6	Glyma.17G057800.4	99.16666667	331	9	406	3215	3174	362	0	3297

TRINITY_DN16254_c0_g1_i6	Glyma.17G057800.3	99.16666667	331	9	406	3215	3174	362	0	3297

TRINITY_DN16254_c0_g1_i6	Glyma.17G057800.2	99.16666667	331	9	406	3215	3174	362	0	3297

TRINITY_DN8943_c0_g1_i1	Glyma.20G089300.2	100	327	9	741	3540	3709	895	0	3267

TRINITY_DN15836_c0_g2_i2	Glyma.02G229400.1	100	205	23	121	2873	1	2813	0	3627

TRINITY_DN15016_c0_g1_i1	Glyma.20G160200.1	97.90575916	202	16	295	3093	1	2796	0	3831

TRINITY_DN14882_c0_g1_i1	Glyma.03G219500.1	95.12195122	191	6	52	2853	2865	49	0	4047

TRINITY_DN14882_c0_g1_i2	Glyma.03G219500.1	98.40848806	191	6	729	3530	2865	49	0	4047

TRINITY_DN13619_c0_g1_i3	Glyma.18G288500.2	95.52238806	162	2	1	2817	799	3600	0	4207

TRINITY_DN13619_c0_g1_i3	Glyma.18G288500.1	100	162	2	1	2817	799	3600	0	4207

TRINITY_DN13619_c0_g1_i3	Glyma.18G288500.5	100	162	2	1	2817	799	3600	0	4207

TRINITY_DN13619_c0_g1_i3	Glyma.02G093600.4	100	157	2	1	2817	814	3624	0	4294

TRINITY_DN13619_c0_g1_i3	Glyma.02G093600.1	96.50059312	157	2	1	2817	814	3624	0	4294

TRINITY_DN16254_c0_g1_i2	Glyma.07G004300.3	95.65217391	632	71	397	3170	2790	23	0	1118

TRINITY_DN16274_c0_g1_i2	Glyma.12G189500.1	97.98657718	265	14	503	3296	1	2803	0	3506

TRINITY_DN15242_c0_g1_i2	Glyma.10G008700.2	100	197	8	221	3027	3375	571	0	3980

TRINITY_DN5901_c0_g2_i1	Glyma.07G173700.3	97.72727273	370	25	189	2996	1	2778	0	2865

TRINITY_DN5901_c0_g2_i1	Glyma.07G173700.1	97.72727273	370	25	189	2996	1	2778	0	2865

TRINITY_DN5901_c0_g2_i1	Glyma.07G173700.2	99.42857143	370	25	189	2996	1	2778	0	2865

TRINITY_DN5901_c0_g1_i1	Glyma.07G173700.3	100	338	25	189	2996	1	2778	0	3042

TRINITY_DN5901_c0_g1_i1	Glyma.07G173700.1	100	338	25	189	2996	1	2778	0	3042

TRINITY_DN5901_c0_g1_i1	Glyma.07G173700.2	100	338	25	189	2996	1	2778	0	3042

TRINITY_DN14654_c0_g1_i5	Glyma.01G155300.1	95.58823529	251	3	1	2814	2968	167	0	3718

TRINITY_DN5901_c0_g1_i1	Glyma.08G049000.1	100	220	4	189	3001	1	2810	0	3934

TRINITY_DN5901_c0_g1_i1	Glyma.08G049000.3	100	220	4	189	3001	1	2810	0	3934

TRINITY_DN5901_c0_g1_i1	Glyma.08G049000.4	100	220	4	189	3001	1	2810	0	3934

TRINITY_DN5901_c0_g1_i1	Glyma.08G049000.2	100	220	4	189	3001	1	2810	0	3934

TRINITY_DN5901_c0_g2_i1	Glyma.08G049000.1	100	206	4	189	3001	1	2810	0	4012

TRINITY_DN5901_c0_g2_i1	Glyma.08G049000.3	100	206	4	189	3001	1	2810	0	4012

TRINITY_DN5901_c0_g2_i1	Glyma.08G049000.4	99.43181818	206	4	189	3001	1	2810	0	4012

TRINITY_DN5901_c0_g2_i1	Glyma.08G049000.2	99.42857143	206	4	189	3001	1	2810	0	4012

TRINITY_DN13959_c0_g1_i4	Glyma.20G242100.3	100	183	9	387	3188	2811	1	0	4074

TRINITY_DN14654_c0_g1_i5	Glyma.11G089400.2	97.14285714	255	5	1	2812	2968	169	0	3681

TRINITY_DN14654_c0_g1_i5	Glyma.11G089400.1	97.14285714	255	5	1	2812	2968	169	0	3681

TRINITY_DN14654_c0_g1_i5	Glyma.11G089400.3	97.14285714	255	5	1	2812	2968	169	0	3681

TRINITY_DN9107_c0_g1_i1	Glyma.11G057500.2	99.06542056	242	20	1190	3967	955	3756	0	3546

TRINITY_DN14245_c0_g2_i3	Glyma.13G181700.1	95.65217391	207	14	446	3253	94	2883	0	3856

TRINITY_DN15981_c0_g1_i19	Glyma.11G048300.2	96	181	3	664	3471	2814	1	0	4156

TRINITY_DN15981_c0_g1_i5	Glyma.01G193600.1	95.4954955	171	1	427	3237	3045	232	0	4229

TRINITY_DN5901_c0_g2_i1	Glyma.02G087000.3	99.42196532	379	23	189	2996	1	2778	0	2837

TRINITY_DN5901_c0_g2_i1	Glyma.02G087000.2	99.41860465	379	23	189	2996	1	2778	0	2837

TRINITY_DN5901_c0_g1_i1	Glyma.02G087000.3	100	352	23	189	2996	1	2778	0	2987

TRINITY_DN5901_c0_g1_i1	Glyma.02G087000.2	100	352	23	189	2996	1	2778	0	2987

TRINITY_DN14882_c0_g1_i1	Glyma.19G216400.2	97.91666667	213	7	52	2849	2847	35	0	3917

TRINITY_DN14882_c0_g1_i2	Glyma.19G216400.2	100	213	7	729	3526	2847	35	0	3917

TRINITY_DN15981_c0_g1_i15	Glyma.11G048300.1	98.16513761	181	3	791	3597	2813	1	0	4154

TRINITY_DN15981_c0_g1_i15	Glyma.17G161300.1	100	436	12	791	3594	2807	1	0	2693

TRINITY_DN14882_c0_g1_i1	Glyma.19G216400.1	98.38709677	213	7	53	2849	2846	35	0	3916

TRINITY_DN14882_c0_g1_i2	Glyma.19G216400.1	97.20670391	213	7	730	3526	2846	35	0	3916

TRINITY_DN12399_c0_g1_i2	Glyma.01G023900.1	100	213	23	190	2993	1	2777	0	3735

TRINITY_DN13900_c0_g1_i1	Glyma.03G262100.2	96.84210526	129	0	334	3144	1	2811	0	4477

TRINITY_DN13900_c0_g1_i1	Glyma.03G262100.3	97.72727273	129	0	334	3144	1	2811	0	4477

TRINITY_DN16247_c0_g1_i3	Glyma.09G103000.1	95.0617284	270	8	1	2808	2802	4	0	3609

TRINITY_DN16247_c0_g1_i3	Glyma.09G103000.2	95.86466165	270	8	1	2808	2802	4	0	3609

TRINITY_DN15808_c0_g1_i1	Glyma.05G022300.1	98.80952381	221	21	2636	5420	2522	5301	0	3609

TRINITY_DN15808_c0_g1_i2	Glyma.05G022300.1	100	221	21	2758	5542	2522	5301	0	3609

TRINITY_DN16254_c0_g1_i6	Glyma.07G004300.3	99.16666667	656	64	397	3170	2790	23	0	1062

TRINITY_DN15981_c0_g1_i15	Glyma.05G105200.1	96.25	428	11	794	3594	2804	1	0	2732

TRINITY_DN16129_c0_g2_i1	Glyma.16G144500.1	100	193	6	356	3154	1	2805	0	4028

TRINITY_DN16129_c0_g2_i2	Glyma.16G144500.1	98.68995633	193	6	328	3126	1	2805	0	4028

TRINITY_DN16254_c0_g1_i3	Glyma.07G004300.3	100	651	62	397	3170	2790	23	0	1101

TRINITY_DN16247_c0_g1_i3	Glyma.15G275000.1	95.0617284	261	9	1	2806	2799	6	0	3637

TRINITY_DN15981_c0_g1_i19	Glyma.01G193600.2	100	184	1	664	3468	2808	1	0	4146

TRINITY_DN5134_c0_g1_i1	Glyma.18G203900.1	95.23809524	149	0	1	2808	223	3030	0	4361

TRINITY_DN5134_c0_g1_i1	Glyma.18G203900.3	98.14814815	148	2	1	2808	223	3024	0	4327

TRINITY_DN9858_c0_g1_i2	Glyma.04G221900.1	97.91666667	130	1	309	3113	1	2808	0	4446

TRINITY_DN16298_c0_g1_i2	Glyma.06G305700.1	96.42857143	324	0	345	3151	2940	134	0	3389

TRINITY_DN16298_c0_g1_i1	Glyma.06G305700.1	100	193	0	297	3103	2940	134	0	4115

TRINITY_DN15981_c0_g1_i15	Glyma.01G193600.1	100	184	1	791	3594	2807	1	0	4145

TRINITY_DN14437_c0_g2_i1	Glyma.18G233100.5	95.52238806	252	5	1	2792	7	2802	0	3631

TRINITY_DN14437_c0_g2_i1	Glyma.18G233100.1	96.16182573	252	5	1	2792	7	2802	0	3631

TRINITY_DN16274_c0_g1_i2	Glyma.12G095300.2	97.14285714	169	20	503	3296	1	2785	0	4032

TRINITY_DN16274_c0_g1_i2	Glyma.12G095300.4	95.41284404	169	20	503	3296	1	2785	0	4032

TRINITY_DN16158_c0_g1_i3	Glyma.20G001300.3	97.12313003	157	5	1201	3991	2803	1	0	4196

TRINITY_DN16158_c0_g1_i4	Glyma.20G001300.3	100	157	5	1075	3865	2803	1	0	4196

TRINITY_DN16158_c0_g1_i7	Glyma.20G001300.3	99.5215311	157	5	800	3590	2803	1	0	4196

TRINITY_DN16349_c0_g1_i2	Glyma.10G019300.1	97.39073712	294	18	420	3213	384	3162	0	3312

TRINITY_DN9858_c0_g1_i2	Glyma.06G143500.2	98.68421053	132	0	309	3113	1	2805	0	4449

TRINITY_DN9858_c0_g1_i2	Glyma.06G143500.1	98.68421053	132	0	309	3113	1	2805	0	4449

TRINITY_DN9858_c0_g1_i2	Glyma.06G143500.3	100	132	0	309	3113	1	2805	0	4449

TRINITY_DN9858_c0_g1_i2	Glyma.06G143500.4	97.91666667	132	0	309	3113	1	2805	0	4449

TRINITY_DN16274_c0_g1_i2	Glyma.12G189500.2	99.47368421	266	10	503	3296	1	2791	0	3555

TRINITY_DN14231_c0_g1_i1	Glyma.08G174100.1	98.02631579	194	23	27	2796	45	2820	0	3714

TRINITY_DN15768_c0_g1_i1	Glyma.03G177600.1	98.92473118	199	5	56	2848	1	2799	0	3995

TRINITY_DN5134_c0_g1_i1	Glyma.07G152800.1	95.20958084	160	0	1	2801	223	3023	0	4287

TRINITY_DN5134_c0_g1_i1	Glyma.07G152800.2	99.41176471	160	1	1	2801	223	3020	0	4266

TRINITY_DN14341_c0_g1_i1	Glyma.18G213600.1	99.60159363	230	9	262	3057	1	2793	0	3825

TRINITY_DN13783_c0_g1_i2	Glyma.09G002400.1	100	205	6	386	3166	2793	1	0	3868

TRINITY_DN13783_c0_g1_i2	Glyma.09G002400.2	96.84210526	205	6	386	3166	2793	1	0	3868

TRINITY_DN14231_c0_g1_i1	Glyma.15G253100.1	98.02371542	197	20	27	2796	45	2829	0	3788

TRINITY_DN16129_c0_g2_i1	Glyma.16G144800.1	98.50746269	182	7	356	3142	1	2796	0	4052

TRINITY_DN16129_c0_g2_i2	Glyma.16G144800.1	98.68995633	182	7	328	3114	1	2796	0	4052

TRINITY_DN16268_c1_g14_i1	Glyma.18G264400.1	100	306	7	265	3060	2925	133	0	3415

TRINITY_DN15768_c0_g1_i4	Glyma.03G177600.1	99.47460595	291	17	56	2806	1	2793	0	3208

TRINITY_DN16129_c0_g2_i1	Glyma.02G062500.1	100	214	3	356	3148	1	2796	0	3925

TRINITY_DN16129_c0_g2_i2	Glyma.02G062500.1	98.68995633	214	3	328	3120	1	2796	0	3925

TRINITY_DN16268_c1_g9_i1	Glyma.18G264400.1	95.09536785	178	7	265	3060	2925	133	0	4124

TRINITY_DN8872_c0_g1_i1	Glyma.15G140000.3	98.7012987	366	10	68	2854	150	2939	0	3016

TRINITY_DN8872_c0_g1_i1	Glyma.15G140000.2	98.7012987	366	10	68	2854	150	2939	0	3016

TRINITY_DN8872_c0_g1_i2	Glyma.15G140000.3	98.34254144	366	10	68	2854	150	2939	0	3016

TRINITY_DN8872_c0_g1_i2	Glyma.15G140000.2	96.49122807	366	10	68	2854	150	2939	0	3016

TRINITY_DN9748_c0_g1_i1	Glyma.18G269500.1	97.97297297	277	19	79	2871	1	2760	0	3354

TRINITY_DN16129_c0_g2_i1	Glyma.02G062200.1	95.14824798	220	4	356	3147	1	2792	0	3871

TRINITY_DN16129_c0_g2_i2	Glyma.02G062200.1	98.68995633	220	4	328	3119	1	2792	0	3871

TRINITY_DN1246_c0_g1_i1	Glyma.12G192700.1	97.82608696	287	13	198	2984	2784	1	0	3437

TRINITY_DN8961_c0_g1_i1	Glyma.02G275300.1	97.5	242	16	538	3299	1	2780	0	3500

TRINITY_DN15818_c1_g2_i2	Glyma.02G184600.2	97.87234043	220	1	474	3269	2793	1	0	3925

TRINITY_DN15988_c0_g1_i3	Glyma.10G297700.1	95.83333333	347	10	764	3547	3081	289	0	3155

TRINITY_DN15988_c0_g1_i1	Glyma.10G297700.1	97.79735683	283	10	598	3381	3081	289	0	3509

TRINITY_DN16274_c0_g1_i2	Glyma.13G312300.1	96.71361502	259	23	503	3296	1	2746	0	3404

TRINITY_DN16130_c2_g6_i8	Glyma.10G075300.2	98.82352941	221	7	278	3067	1	2790	0	3873

TRINITY_DN16130_c2_g6_i17	Glyma.10G075300.2	96.36363636	163	7	278	3067	1	2790	0	4194

TRINITY_DN16325_c0_g1_i2	Glyma.19G016700.1	100	358	9	411	3199	3715	930	0	3092

TRINITY_DN16325_c0_g1_i2	Glyma.19G016700.2	100	358	9	411	3199	3379	594	0	3092

TRINITY_DN16325_c0_g1_i3	Glyma.19G016700.1	100	355	9	228	3016	3715	930	0	3109

TRINITY_DN16325_c0_g1_i3	Glyma.19G016700.2	100	355	9	228	3016	3379	594	0	3109

TRINITY_DN16268_c1_g14_i1	Glyma.08G242100.1	100	307	6	265	3056	2925	138	0	3408

TRINITY_DN1246_c0_g1_i1	Glyma.13G309700.1	97.82608696	282	17	198	2984	2775	1	0	3430

TRINITY_DN14437_c0_g2_i1	Glyma.09G259200.3	95.4954955	261	3	1	2792	7	2799	0	3694

TRINITY_DN14437_c0_g2_i1	Glyma.09G259200.2	95.52845528	261	3	1	2792	7	2799	0	3694

TRINITY_DN14437_c0_g2_i1	Glyma.09G259200.1	95.52238806	261	3	1	2792	7	2799	0	3694

TRINITY_DN14437_c0_g2_i1	Glyma.18G233100.3	95.4954955	252	4	1	2792	7	2790	0	3687

TRINITY_DN14437_c0_g2_i1	Glyma.18G233100.4	95.52238806	252	4	1	2792	7	2790	0	3687

TRINITY_DN12872_c0_g2_i1	Glyma.14G214000.1	95.0617284	228	10	38	2818	1	2781	0	3729

TRINITY_DN14062_c0_g1_i2	Glyma.01G146600.1	100	189	0	1104	3897	2887	94	0	4113

TRINITY_DN16268_c1_g9_i1	Glyma.08G242100.1	97.09677419	183	6	265	3056	2925	138	0	4095

TRINITY_DN15506_c0_g2_i11	Glyma.07G196500.1	98.4063745	463	20	954	3734	1	2784	0	2457

TRINITY_DN15506_c0_g2_i12	Glyma.07G196500.1	100	463	20	1268	4048	1	2784	0	2457

TRINITY_DN15506_c0_g2_i13	Glyma.07G196500.1	100	463	20	1036	3816	1	2784	0	2457

TRINITY_DN15506_c0_g2_i3	Glyma.07G196500.1	96.35416667	463	20	871	3651	1	2784	0	2457

TRINITY_DN15506_c0_g2_i5	Glyma.07G196500.1	97.95918367	463	20	1185	3965	1	2784	0	2457

TRINITY_DN15506_c0_g2_i6	Glyma.07G196500.1	99.03846154	463	20	953	3733	1	2784	0	2457

TRINITY_DN1246_c0_g1_i1	Glyma.12G192700.2	97.82608696	288	11	198	2984	2781	1	0	3446

TRINITY_DN15506_c0_g2_i11	Glyma.15G074200.1	95.65217391	241	16	954	3734	1	2775	0	3629

TRINITY_DN15506_c0_g2_i12	Glyma.15G074200.1	100	241	16	1268	4048	1	2775	0	3629

TRINITY_DN15506_c0_g2_i13	Glyma.15G074200.1	100	241	16	1036	3816	1	2775	0	3629

TRINITY_DN15506_c0_g2_i3	Glyma.15G074200.1	98.47560976	241	16	871	3651	1	2775	0	3629

TRINITY_DN15506_c0_g2_i5	Glyma.15G074200.1	95.94594595	241	16	1185	3965	1	2775	0	3629

TRINITY_DN15506_c0_g2_i6	Glyma.15G074200.1	100	241	16	953	3733	1	2775	0	3629

TRINITY_DN16130_c2_g6_i8	Glyma.02G201300.2	100	206	5	278	3067	1	2787	0	3958

TRINITY_DN16130_c2_g6_i8	Glyma.02G201300.4	98.91304348	206	5	278	3067	1	2787	0	3958

TRINITY_DN16130_c2_g6_i8	Glyma.02G201300.1	98.82352941	206	5	278	3067	1	2787	0	3958

TRINITY_DN13783_c0_g1_i2	Glyma.12G241900.1	100	187	6	392	3166	2784	1	0	3947

TRINITY_DN16130_c2_g6_i17	Glyma.02G201300.2	95.62289562	148	5	278	3067	1	2787	0	4279

TRINITY_DN16130_c2_g6_i17	Glyma.02G201300.4	95.625	148	5	278	3067	1	2787	0	4279

TRINITY_DN16130_c2_g6_i17	Glyma.02G201300.1	95.625	148	5	278	3067	1	2787	0	4279

TRINITY_DN12732_c0_g1_i1	Glyma.17G157900.1	96.09756098	222	6	184	2974	22	2796	0	3810

TRINITY_DN12732_c0_g1_i2	Glyma.17G157900.1	98.46153846	222	6	184	2974	22	2796	0	3810

TRINITY_DN16440_c0_g1_i2	Glyma.03G075300.1	100	400	31	592	3353	3246	473	0	2641

TRINITY_DN5346_c0_g1_i1	Glyma.01G092400.1	98.47715736	262	7	1639	4425	1171	3942	0	3554

TRINITY_DN5346_c0_g1_i1	Glyma.01G092400.2	100	262	7	1639	4425	1171	3942	0	3554

TRINITY_DN15549_c0_g1_i2	Glyma.05G247000.2	99	202	14	903	3662	2784	1	0	3790

TRINITY_DN9348_c0_g1_i1	Glyma.10G082300.1	95.96774194	160	4	1	2788	306	3090	0	4209

TRINITY_DN14283_c0_g1_i3	Glyma.18G143900.1	99.2970123	233	20	391	3174	2952	211	0	3513

TRINITY_DN9206_c0_g1_i1	Glyma.09G218800.1	97.08737864	221	2	454	3240	667	3453	0	3890

TRINITY_DN14670_c0_g1_i2	Glyma.19G019100.2	95.83333333	262	11	605	3386	3	2781	0	3589

TRINITY_DN14575_c0_g1_i1	Glyma.14G135900.1	97.43954481	203	17	575	3361	8298	5551	0	3747

TRINITY_DN14575_c0_g1_i1	Glyma.14G135900.2	98.55072464	203	17	575	3361	8274	5527	0	3747

TRINITY_DN13063_c0_g2_i3	Glyma.07G238400.1	98.82352941	221	6	91	2874	10	2790	0	3853

TRINITY_DN14575_c0_g1_i1	Glyma.17G197200.1	100	220	4	575	3361	8406	5626	0	3879

TRINITY_DN14575_c0_g1_i1	Glyma.17G197200.3	97.43589744	220	4	575	3361	8382	5602	0	3879

TRINITY_DN14575_c0_g1_i1	Glyma.17G197200.4	97.14285714	217	10	575	3361	8394	5626	0	3818

TRINITY_DN14575_c0_g1_i1	Glyma.17G197200.2	95.59193955	217	10	575	3361	8370	5602	0	3818

TRINITY_DN14598_c0_g2_i1	Glyma.14G223100.4	100	216	28	515	3247	2760	1	0	3415

TRINITY_DN14598_c0_g2_i1	Glyma.14G223100.5	99.72299169	216	28	515	3247	2760	1	0	3415

TRINITY_DN14598_c0_g2_i1	Glyma.14G223100.2	96.87778769	214	29	515	3247	2748	1	0	3349

TRINITY_DN14598_c0_g2_i1	Glyma.14G223100.3	99.33333333	214	29	515	3247	2748	1	0	3349

TRINITY_DN12732_c0_g1_i1	Glyma.05G109300.1	95.53571429	207	7	184	2969	22	2792	0	3879

TRINITY_DN12732_c0_g1_i2	Glyma.05G109300.1	98.74213836	207	7	184	2969	22	2792	0	3879

TRINITY_DN14283_c0_g1_i3	Glyma.08G282400.5	99.28057554	227	18	391	3168	2964	202	0	3675

TRINITY_DN14283_c0_g1_i1	Glyma.08G282400.5	95.45454545	223	25	391	3144	2964	202	0	3542

TRINITY_DN14283_c0_g1_i3	Glyma.08G282400.3	99.28571429	223	25	391	3168	2940	202	0	3542

TRINITY_DN12780_c0_g2_i1	Glyma.07G039900.1	100	201	10	100	2868	1	2784	0	3897

TRINITY_DN11037_c0_g1_i3	Glyma.20G217800.1	95.54317549	185	2	217	3000	1	2787	0	4102

TRINITY_DN11037_c0_g1_i3	Glyma.10G172400.1	100	181	4	217	3000	1	2781	0	4085

TRINITY_DN15506_c0_g2_i11	Glyma.13G239100.1	95.65217391	230	18	954	3734	1	2760	0	3670

TRINITY_DN15506_c0_g2_i12	Glyma.13G239100.1	100	230	18	1268	4048	1	2760	0	3670

TRINITY_DN15506_c0_g2_i13	Glyma.13G239100.1	100	230	18	1036	3816	1	2760	0	3670

TRINITY_DN15506_c0_g2_i3	Glyma.13G239100.1	98.47328244	230	18	871	3651	1	2760	0	3670

TRINITY_DN15506_c0_g2_i5	Glyma.13G239100.1	95.94594595	230	18	1185	3965	1	2760	0	3670

TRINITY_DN15506_c0_g2_i6	Glyma.13G239100.1	100	230	18	953	3733	1	2760	0	3670

TRINITY_DN5725_c0_g2_i1	Glyma.06G074700.1	97.95918367	140	3	1	2786	4650	7426	0	4311

TRINITY_DN5725_c0_g2_i1	Glyma.06G074700.4	97.95918367	140	3	1	2786	4650	7426	0	4311

TRINITY_DN5725_c0_g2_i1	Glyma.06G074700.3	97.95918367	140	3	1	2786	4623	7399	0	4311

TRINITY_DN5725_c0_g2_i1	Glyma.06G074700.2	97.95918367	140	3	1	2786	3942	6718	0	4311

TRINITY_DN16064_c0_g2_i1	Glyma.13G361900.4	98.85714286	450	30	89	2860	100	2853	0	2366

TRINITY_DN16064_c0_g2_i3	Glyma.13G361900.4	96.55172414	450	30	89	2860	100	2853	0	2366

TRINITY_DN10413_c0_g1_i1	Glyma.04G038200.1	100	306	52	443	3181	2712	1	0	2678

TRINITY_DN10413_c0_g1_i3	Glyma.04G038200.1	98.88268156	306	52	236	2974	2712	1	0	2678

TRINITY_DN15768_c0_g1_i1	Glyma.10G048800.1	100	379	25	72	2848	23	2772	0	2776

TRINITY_DN49_c0_g2_i1	Glyma.20G217700.1	100	302	27	721	3465	3696	931	0	3099

TRINITY_DN14283_c0_g1_i3	Glyma.08G282400.4	99.27536232	227	17	391	3168	2961	202	0	3690

TRINITY_DN14283_c0_g1_i1	Glyma.08G282400.4	95.45454545	223	24	391	3144	2961	202	0	3557

TRINITY_DN14283_c0_g1_i3	Glyma.08G282400.2	99.27797834	223	24	391	3168	2937	202	0	3557

TRINITY_DN15768_c0_g1_i1	Glyma.13G136700.1	98.8372093	369	19	72	2848	101	2853	0	2863

TRINITY_DN15768_c0_g1_i1	Glyma.13G136700.2	97.22222222	369	19	72	2848	26	2778	0	2863

TRINITY_DN15768_c0_g1_i1	Glyma.13G136700.3	98.8372093	369	19	72	2848	26	2778	0	2863

TRINITY_DN16115_c0_g2_i2	Glyma.U033900.2	97.82608696	224	3	604	3385	2776	1	0	3847

TRINITY_DN16115_c0_g2_i1	Glyma.U033900.2	96.20253165	187	3	604	3385	2776	1	0	4052

TRINITY_DN13347_c0_g1_i1	Glyma.09G206300.1	97.95918367	160	5	371	3144	1	2783	0	4194

TRINITY_DN13347_c0_g1_i1	Glyma.01G016500.2	100	152	5	371	3144	1	2783	0	4239

TRINITY_DN13347_c0_g1_i1	Glyma.01G016500.1	99.13419913	152	5	371	3144	1	2783	0	4239

TRINITY_DN16115_c0_g2_i6	Glyma.U033900.2	100	330	5	708	3487	2774	1	0	3245

TRINITY_DN16115_c0_g2_i4	Glyma.U033900.2	100	293	5	708	3487	2774	1	0	3450

TRINITY_DN11160_c0_g1_i1	Glyma.11G060300.1	100	208	18	418	3186	2757	1	0	3740

TRINITY_DN16041_c0_g1_i1	Glyma.11G128000.1	96.42857143	441	35	380	3120	2961	199	0	2318

TRINITY_DN16041_c0_g1_i1	Glyma.11G128000.2	96.42857143	441	35	380	3120	2961	199	0	2318

TRINITY_DN16041_c0_g1_i6	Glyma.11G128000.1	98.51239669	441	35	153	2893	2961	199	0	2318

TRINITY_DN16041_c0_g1_i6	Glyma.11G128000.2	97.61904762	441	35	153	2893	2961	199	0	2318

TRINITY_DN16115_c0_g2_i1	Glyma.15G043400.3	100	293	2	606	3385	2780	1	0	3500

TRINITY_DN16115_c0_g2_i2	Glyma.15G043400.3	97.14285714	256	2	606	3385	2780	1	0	3705

TRINITY_DN15417_c0_g1_i1	Glyma.08G002300.2	100	208	21	467	3223	2805	55	0	3635

TRINITY_DN15417_c0_g1_i1	Glyma.08G002300.1	95.28795812	208	21	467	3223	2805	55	0	3635

TRINITY_DN15417_c0_g1_i2	Glyma.08G002300.2	99.36708861	208	21	733	3489	2805	55	0	3635

TRINITY_DN15417_c0_g1_i2	Glyma.08G002300.1	98.21428571	208	21	733	3489	2805	55	0	3635

TRINITY_DN16115_c0_g2_i4	Glyma.15G043400.3	100	165	0	707	3487	2781	1	0	4222

TRINITY_DN16115_c0_g2_i6	Glyma.15G043400.3	99.24528302	128	0	707	3487	2781	1	0	4427

TRINITY_DN1011_c0_g1_i1	Glyma.02G013500.2	100	295	44	100	2814	3329	576	0	2911

TRINITY_DN16425_c0_g1_i2	Glyma.15G035900.3	96.82539683	375	21	902	3652	2757	1	0	2732

TRINITY_DN16425_c0_g1_i5	Glyma.15G035900.3	97.36842105	375	21	902	3652	2757	1	0	2732

TRINITY_DN11160_c0_g1_i1	Glyma.11G060300.2	95.95959596	208	17	418	3186	2754	1	0	3753

TRINITY_DN15784_c0_g1_i6	Glyma.02G214300.1	98.79518072	193	17	1	2751	34	2805	0	3836

TRINITY_DN9844_c0_g1_i3	Glyma.01G176000.1	95.55555556	192	8	275	3045	2846	73	0	3984

TRINITY_DN16130_c2_g6_i4	Glyma.10G075300.2	97.85714286	241	12	278	3045	1	2771	0	3685

TRINITY_DN12780_c0_g2_i1	Glyma.16G008700.1	100	222	6	100	2868	1	2769	0	3784

TRINITY_DN15784_c0_g1_i6	Glyma.14G181800.3	98.37837838	192	15	1	2751	34	2805	0	3853

TRINITY_DN15784_c0_g1_i6	Glyma.14G181800.2	98.79518072	192	15	1	2751	34	2805	0	3853

TRINITY_DN15784_c0_g1_i6	Glyma.14G181800.1	98.79518072	192	15	1	2751	34	2805	0	3853

TRINITY_DN16130_c2_g6_i14	Glyma.10G075300.2	96.10866373	183	12	278	3045	1	2771	0	4006

TRINITY_DN10067_c0_g4_i1	Glyma.15G163700.3	95.95959596	132	1	413	3187	1	2778	0	4379

TRINITY_DN10067_c0_g4_i2	Glyma.15G163700.3	100	132	1	413	3187	1	2778	0	4379

TRINITY_DN16041_c0_g1_i1	Glyma.12G052700.4	99.31506849	438	29	380	3120	2961	199	0	2379

TRINITY_DN16041_c0_g1_i1	Glyma.12G052700.1	96.42857143	438	29	380	3120	2961	199	0	2379

TRINITY_DN16041_c0_g1_i1	Glyma.12G052700.2	96.42857143	438	29	380	3120	2961	199	0	2379

TRINITY_DN16041_c0_g1_i1	Glyma.12G052700.3	96.42857143	438	29	380	3120	2961	199	0	2379

TRINITY_DN16041_c0_g1_i6	Glyma.12G052700.4	96.80851064	438	29	153	2893	2961	199	0	2379

TRINITY_DN16041_c0_g1_i6	Glyma.12G052700.1	97.63779528	438	29	153	2893	2961	199	0	2379

TRINITY_DN16041_c0_g1_i6	Glyma.12G052700.2	95.34883721	438	29	153	2893	2961	199	0	2379

TRINITY_DN16041_c0_g1_i6	Glyma.12G052700.3	95.68345324	438	29	153	2893	2961	199	0	2379

TRINITY_DN16130_c2_g6_i4	Glyma.02G201300.2	97.64705882	227	9	278	3045	1	2768	0	3766

TRINITY_DN16130_c2_g6_i4	Glyma.02G201300.4	97.36842105	227	9	278	3045	1	2768	0	3766

TRINITY_DN16130_c2_g6_i4	Glyma.02G201300.1	97.85714286	227	9	278	3045	1	2768	0	3766

TRINITY_DN16130_c2_g6_i14	Glyma.02G201300.2	97.87234043	169	9	278	3045	1	2768	0	4087

TRINITY_DN16130_c2_g6_i14	Glyma.02G201300.4	100	169	9	278	3045	1	2768	0	4087

TRINITY_DN16130_c2_g6_i14	Glyma.02G201300.1	100	169	9	278	3045	1	2768	0	4087

TRINITY_DN14585_c0_g1_i1	Glyma.16G027200.4	99.3902439	263	7	419	3193	310	3072	0	3591

TRINITY_DN14585_c0_g1_i1	Glyma.16G027200.3	99.39209726	263	7	419	3193	310	3072	0	3591

TRINITY_DN14585_c0_g1_i1	Glyma.16G027200.2	96.15384615	263	7	419	3193	310	3072	0	3591

TRINITY_DN14585_c0_g1_i1	Glyma.16G027200.1	96.15384615	263	7	419	3193	310	3072	0	3591

TRINITY_DN8961_c0_g1_i1	Glyma.14G040900.1	97.5	252	18	538	3302	1	2753	0	3522

TRINITY_DN16128_c0_g1_i2	Glyma.13G330500.1	97.82608696	233	13	631	3391	4023	1252	0	3723

TRINITY_DN16128_c0_g1_i3	Glyma.13G330500.1	97.82608696	233	13	959	3719	4023	1252	0	3723

TRINITY_DN6220_c0_g1_i1	Glyma.14G001100.1	95.08196721	231	16	205	2968	2744	5	0	3548

TRINITY_DN13056_c0_g1_i1	Glyma.19G141900.5	96.23824451	221	8	1	2756	534	3299	0	3720

TRINITY_DN13056_c0_g1_i1	Glyma.19G141900.4	96.35036496	221	8	1	2756	534	3299	0	3720

TRINITY_DN13056_c0_g1_i1	Glyma.19G141900.1	95	221	8	1	2756	534	3299	0	3720

TRINITY_DN13056_c0_g1_i1	Glyma.19G141900.6	96.25	220	10	1	2756	534	3293	0	3687

TRINITY_DN13056_c0_g1_i1	Glyma.19G141900.3	96.25	220	10	1	2756	534	3293	0	3687

TRINITY_DN13056_c0_g1_i1	Glyma.19G141900.2	96.25	220	10	1	2756	534	3293	0	3687

TRINITY_DN11160_c0_g1_i1	Glyma.01G182000.1	95.95959596	199	13	418	3186	2748	1	0	3808

TRINITY_DN11656_c0_g1_i1	Glyma.09G002100.1	100	166	3	216	2987	1	2769	0	4146

TRINITY_DN12591_c0_g1_i1	Glyma.16G084200.4	97.46031746	159	10	343	3108	2769	1	0	4146

TRINITY_DN12591_c0_g1_i2	Glyma.16G084200.4	100	159	10	315	3080	2769	1	0	4146

TRINITY_DN15549_c0_g1_i6	Glyma.05G247000.2	100	200	14	885	3626	2766	1	0	3768

TRINITY_DN10765_c0_g1_i1	Glyma.11G038500.1	98	191	4	150	2910	2964	200	0	3934

TRINITY_DN15345_c0_g3_i1	Glyma.12G099500.1	100	237	16	381	3134	1	2766	0	3651

TRINITY_DN15345_c0_g3_i1	Glyma.12G099500.4	100	237	16	381	3134	1	2766	0	3651

TRINITY_DN15549_c0_g1_i2	Glyma.05G247000.3	100	200	14	922	3662	2765	1	0	3766

TRINITY_DN9844_c0_g1_i3	Glyma.11G068300.1	100	193	4	275	3039	2768	1	0	3978

TRINITY_DN11656_c0_g1_i1	Glyma.12G242300.1	100	168	1	216	2987	1	2769	0	4169

TRINITY_DN11656_c0_g1_i1	Glyma.12G242300.2	96.72801636	168	1	216	2987	1	2769	0	4169

TRINITY_DN15815_c0_g2_i4	Glyma.12G147000.2	98.56115108	232	10	1489	4250	2751	1	0	3644

TRINITY_DN12540_c0_g1_i2	Glyma.01G009400.1	99.56521739	211	9	420	3163	2877	116	0	3716

TRINITY_DN15768_c0_g1_i4	Glyma.13G136700.1	100	297	24	72	2812	101	2853	0	3173

TRINITY_DN15768_c0_g1_i4	Glyma.13G136700.2	98.30508475	297	24	72	2812	26	2778	0	3173

TRINITY_DN15768_c0_g1_i4	Glyma.13G136700.3	98.61111111	297	24	72	2812	26	2778	0	3173

TRINITY_DN16376_c3_g12_i5	Glyma.05G151100.1	96	224	17	124	2887	5	2750	0	3681

TRINITY_DN10765_c0_g1_i1	Glyma.11G038500.2	98	191	3	150	2910	2961	200	0	3947

TRINITY_DN15768_c0_g1_i4	Glyma.10G048800.1	100	309	23	72	2812	23	2772	0	3097

TRINITY_DN14442_c0_g1_i1	Glyma.08G102900.1	95.47169811	226	30	14	2738	3	2766	0	3544

TRINITY_DN12687_c0_g2_i1	Glyma.05G004100.1	97.27722772	194	14	150	2885	1	2769	0	3825

TRINITY_DN16245_c0_g3_i4	Glyma.16G099700.3	98.41269841	386	29	1090	3841	2778	31	0	2741

TRINITY_DN16245_c0_g3_i5	Glyma.16G099700.3	96.96969697	386	29	404	3155	2778	31	0	2741

TRINITY_DN5901_c0_g2_i1	Glyma.02G087000.4	99.41860465	371	23	189	2951	1	2733	0	2798

TRINITY_DN5901_c0_g2_i1	Glyma.02G087000.1	99.42028986	371	23	189	2951	1	2733	0	2798

TRINITY_DN5901_c0_g1_i1	Glyma.02G087000.4	100	344	23	189	2951	1	2733	0	2948

TRINITY_DN5901_c0_g1_i1	Glyma.02G087000.1	100	344	23	189	2951	1	2733	0	2948

TRINITY_DN8328_c0_g1_i1	Glyma.11G160300.1	95.48022599	179	1	388	3155	3351	587	0	4100

TRINITY_DN8328_c0_g1_i1	Glyma.18G053700.1	95.48022599	169	1	388	3155	3333	569	0	4156

TRINITY_DN16376_c3_g12_i3	Glyma.08G107800.1	96.23059867	147	11	124	2885	5	2751	0	4122

TRINITY_DN16376_c3_g12_i3	Glyma.05G151100.1	96.22641509	147	14	124	2885	5	2751	0	4122

TRINITY_DN16376_c3_g12_i5	Glyma.08G107800.1	95.97315436	224	14	124	2884	5	2747	0	3675

TRINITY_DN15040_c0_g1_i1	Glyma.15G163900.2	100	215	7	784	3548	2758	3	0	3834

TRINITY_DN16397_c0_g1_i2	Glyma.19G065600.2	100	306	6	3933	6695	2830	68	0	3374

TRINITY_DN14283_c0_g1_i1	Glyma.18G143900.1	99	233	15	391	3150	2952	211	0	3624

TRINITY_DN12892_c0_g4_i5	Glyma.11G103100.1	100	189	2	305	3067	139	2898	0	4002

TRINITY_DN12892_c0_g4_i5	Glyma.11G103100.4	100	189	2	305	3067	139	2898	0	4002

TRINITY_DN13071_c1_g2_i1	Glyma.05G126600.1	95.94594595	222	8	225	2988	114	2868	0	3805

TRINITY_DN13071_c1_g2_i1	Glyma.05G126600.5	98.79725086	222	9	225	2988	114	2865	0	3786

TRINITY_DN16080_c0_g1_i1	Glyma.20G052500.1	98.54771784	219	6	105	2866	92	2844	0	3807

TRINITY_DN14516_c0_g1_i2	Glyma.05G031900.1	99.52380952	127	8	209	2951	120	2880	0	4246

TRINITY_DN14516_c0_g1_i2	Glyma.17G094800.1	96.70542636	126	4	209	2951	120	2883	0	4270

TRINITY_DN16331_c0_g2_i2	Glyma.13G360100.1	95.90163934	107	6	97	2850	1	2763	0	4440

TRINITY_DN16331_c0_g2_i2	Glyma.15G013800.3	95.90909091	107	6	97	2850	1	2763	0	4440

TRINITY_DN16331_c0_g2_i2	Glyma.15G013800.1	95.89041096	107	6	97	2850	1	2763	0	4440

TRINITY_DN16331_c0_g2_i5	Glyma.13G360100.1	100	107	6	97	2850	1	2763	0	4440

TRINITY_DN16331_c0_g2_i5	Glyma.15G013800.3	100	107	6	97	2850	1	2763	0	4440

TRINITY_DN16331_c0_g2_i5	Glyma.15G013800.1	100	107	6	97	2850	1	2763	0	4440

TRINITY_DN14283_c0_g1_i1	Glyma.08G282400.3	98.98989899	223	18	391	3144	2940	202	0	3653

TRINITY_DN16349_c0_g1_i2	Glyma.02G155000.1	97.4025974	214	18	465	3213	5	2739	0	3646

TRINITY_DN15040_c0_g1_i3	Glyma.13G099300.2	96.96969697	197	6	406	3144	2763	1	0	3856

TRINITY_DN15709_c0_g1_i2	Glyma.15G276000.1	100	240	25	1922	4661	1722	4455	0	3448

TRINITY_DN15132_c0_g1_i1	Glyma.16G023700.2	100	228	2	457	3217	2889	129	0	3825

TRINITY_DN15132_c0_g1_i1	Glyma.16G023700.1	98.5	228	2	457	3217	2889	129	0	3825

TRINITY_DN15132_c0_g1_i2	Glyma.16G023700.2	99.7716895	228	2	512	3272	2889	129	0	3825

TRINITY_DN15132_c0_g1_i2	Glyma.16G023700.1	95.05703422	228	2	512	3272	2889	129	0	3825

TRINITY_DN15132_c0_g1_i3	Glyma.16G023700.2	97.88359788	228	2	177	2937	2889	129	0	3825

TRINITY_DN15132_c0_g1_i3	Glyma.16G023700.1	97.99270073	228	2	177	2937	2889	129	0	3825

TRINITY_DN13016_c0_g1_i1	Glyma.12G010300.1	100	142	8	571	3320	3294	545	0	4159

TRINITY_DN13016_c0_g1_i1	Glyma.12G010300.2	100	142	8	571	3320	3294	545	0	4159

TRINITY_DN16130_c2_g6_i5	Glyma.19G196000.1	98.91304348	269	7	284	3038	162	2916	0	3531

TRINITY_DN15132_c0_g1_i1	Glyma.07G054900.1	98.80478088	227	0	457	3217	2889	129	0	3842

TRINITY_DN15132_c0_g1_i2	Glyma.07G054900.1	99.7706422	227	0	512	3272	2889	129	0	3842

TRINITY_DN15132_c0_g1_i3	Glyma.07G054900.1	97.02970297	227	0	177	2937	2889	129	0	3842

TRINITY_DN16130_c2_g6_i15	Glyma.19G196000.1	99.5951417	216	7	284	3038	162	2916	0	3825

TRINITY_DN14042_c0_g1_i2	Glyma.15G042000.6	99.52267303	179	20	663	3389	1	2757	0	3862

TRINITY_DN14042_c0_g1_i2	Glyma.15G042000.7	98.26086957	179	20	663	3389	1	2757	0	3862

TRINITY_DN14042_c0_g1_i4	Glyma.15G042000.6	98.46153846	179	20	446	3172	1	2757	0	3862

TRINITY_DN14042_c0_g1_i4	Glyma.15G042000.7	95.38461538	179	20	446	3172	1	2757	0	3862

TRINITY_DN16086_c0_g2_i2	Glyma.04G075100.2	99.57627119	174	2	271	3030	1	2760	0	4122

TRINITY_DN15016_c0_g1_i2	Glyma.09G137000.1	98.8372093	446	40	566	3290	245	2973	0	2200

TRINITY_DN16130_c2_g6_i5	Glyma.19G196000.3	98.91304348	269	7	285	3038	1	2754	0	3530

TRINITY_DN16130_c2_g6_i5	Glyma.19G196000.4	100	269	7	285	3038	1	2754	0	3530

TRINITY_DN2620_c0_g1_i1	Glyma.12G027700.1	100	233	7	87	2834	2757	1	0	3709

TRINITY_DN2620_c0_g1_i1	Glyma.12G027700.3	100	233	7	87	2834	2757	1	0	3709

TRINITY_DN2620_c0_g1_i1	Glyma.12G027700.4	100	233	7	87	2834	2757	1	0	3709

TRINITY_DN2620_c0_g1_i1	Glyma.12G027700.2	100	232	8	87	2834	2751	1	0	3675

TRINITY_DN14283_c0_g1_i1	Glyma.08G282400.2	99	223	17	391	3144	2937	202	0	3668

TRINITY_DN16130_c2_g6_i15	Glyma.19G196000.3	97.5	216	7	285	3038	1	2754	0	3823

TRINITY_DN16130_c2_g6_i15	Glyma.19G196000.4	97.91666667	216	7	285	3038	1	2754	0	3823

TRINITY_DN14042_c0_g1_i2	Glyma.13G332300.12	98.26086957	195	6	663	3389	1	2757	0	3784

TRINITY_DN14042_c0_g1_i2	Glyma.13G332300.5	95.97315436	195	6	663	3389	1	2757	0	3784

TRINITY_DN14042_c0_g1_i2	Glyma.13G332300.6	99.27007299	195	6	663	3389	1	2757	0	3784

TRINITY_DN14042_c0_g1_i4	Glyma.13G332300.12	95.38461538	195	6	446	3172	1	2757	0	3784

TRINITY_DN14042_c0_g1_i4	Glyma.13G332300.5	98	195	6	446	3172	1	2757	0	3784

TRINITY_DN14042_c0_g1_i4	Glyma.13G332300.6	98.68421053	195	6	446	3172	1	2757	0	3784

TRINITY_DN16086_c0_g2_i5	Glyma.04G075100.2	100	142	0	100	2859	1	2760	0	4311

TRINITY_DN15964_c0_g1_i7	Glyma.15G004300.1	99.10714286	640	58	368	3096	131	2850	0	1103

TRINITY_DN15506_c0_g2_i11	Glyma.13G179600.2	97.27891156	447	16	971	3721	18	2768	0	2516

TRINITY_DN15506_c0_g2_i11	Glyma.13G179600.1	98.4063745	447	16	971	3721	18	2768	0	2516

TRINITY_DN15506_c0_g2_i12	Glyma.13G179600.2	96	447	16	1285	4035	18	2768	0	2516

TRINITY_DN15506_c0_g2_i12	Glyma.13G179600.1	98.50187266	447	16	1285	4035	18	2768	0	2516

TRINITY_DN15506_c0_g2_i13	Glyma.13G179600.2	99.1416309	447	16	1053	3803	18	2768	0	2516

TRINITY_DN15506_c0_g2_i13	Glyma.13G179600.1	97.07317073	447	16	1053	3803	18	2768	0	2516

TRINITY_DN15506_c0_g2_i3	Glyma.13G179600.2	99.07407407	447	16	888	3638	18	2768	0	2516

TRINITY_DN15506_c0_g2_i3	Glyma.13G179600.1	99.07407407	447	16	888	3638	18	2768	0	2516

TRINITY_DN15506_c0_g2_i5	Glyma.13G179600.2	100	447	16	1202	3952	18	2768	0	2516

TRINITY_DN15506_c0_g2_i5	Glyma.13G179600.1	97.56097561	447	16	1202	3952	18	2768	0	2516

TRINITY_DN15506_c0_g2_i6	Glyma.13G179600.2	97.08994709	447	16	970	3720	18	2768	0	2516

TRINITY_DN15506_c0_g2_i6	Glyma.13G179600.1	95.3125	447	16	970	3720	18	2768	0	2516

TRINITY_DN15699_c0_g1_i10	Glyma.19G056700.1	95	359	16	173	2917	1	2751	0	2964

TRINITY_DN15699_c0_g1_i1	Glyma.19G056700.1	100	344	16	173	2917	1	2751	0	3048

TRINITY_DN16080_c0_g1_i1	Glyma.20G052500.3	95.1048951	219	6	110	2866	1	2748	0	3797

TRINITY_DN16080_c0_g1_i1	Glyma.20G052500.2	95.1048951	219	6	110	2866	1	2748	0	3797

TRINITY_DN14610_c0_g1_i1	Glyma.16G217200.1	95.85987261	200	3	181	2930	2759	1	0	3928

TRINITY_DN15016_c0_g1_i2	Glyma.16G182400.1	98.28178694	448	42	566	3290	242	2964	0	2172

TRINITY_DN16331_c0_g2_i1	Glyma.13G360100.1	95.89041096	272	4	141	2894	1	2756	0	3548

TRINITY_DN16331_c0_g2_i1	Glyma.15G013800.3	95.89041096	268	4	141	2894	1	2756	0	3570

TRINITY_DN16331_c0_g2_i1	Glyma.15G013800.1	95.91836735	268	4	141	2894	1	2756	0	3570

TRINITY_DN13926_c0_g1_i2	Glyma.01G234600.4	100	232	29	239	2929	2919	183	0	3240

TRINITY_DN13926_c0_g1_i2	Glyma.01G234600.5	98.33333333	232	29	239	2929	2919	183	0	3240

TRINITY_DN5434_c0_g1_i1	Glyma.14G127800.2	100	171	3	82	2836	1	2755	0	4108

TRINITY_DN5434_c0_g1_i1	Glyma.14G127800.1	100	171	3	82	2836	1	2755	0	4108

TRINITY_DN15964_c0_g1_i8	Glyma.15G004300.1	99.5412844	659	55	368	3096	131	2850	0	1020

TRINITY_DN19847_c0_g1_i1	Glyma.09G279900.1	95.83333333	344	18	1	2736	1302	4049	0	2992

TRINITY_DN19847_c0_g1_i1	Glyma.09G279900.2	96.47058824	344	18	1	2736	912	3659	0	2992

TRINITY_DN4758_c0_g1_i1	Glyma.09G198200.2	100	219	1	355	3111	2756	3	0	3858

TRINITY_DN4509_c0_g1_i1	Glyma.15G068100.2	99.1416309	214	3	136	2889	1	2757	0	3886

TRINITY_DN4509_c0_g1_i1	Glyma.15G068100.1	99.14367269	214	3	136	2889	1	2757	0	3886

TRINITY_DN15863_c0_g1_i1	Glyma.15G016700.2	98.81422925	214	8	274	3020	1	2748	0	3794

TRINITY_DN15863_c0_g1_i1	Glyma.15G016700.1	98.80952381	214	8	274	3020	1	2748	0	3794

TRINITY_DN8191_c0_g2_i2	Glyma.03G086000.1	98.0952381	194	1	1	2753	103	2858	0	3995

TRINITY_DN7273_c0_g1_i1	Glyma.20G179400.1	97.72727273	193	29	358	3077	2732	13	0	3555

TRINITY_DN7273_c0_g1_i2	Glyma.20G179400.1	95.72368421	190	34	358	3066	2732	13	0	3500

TRINITY_DN8205_c0_g1_i1	Glyma.20G190500.1	98.38709677	176	7	452	3207	3138	404	0	3978

TRINITY_DN8205_c0_g1_i1	Glyma.10G200000.1	96.77419355	171	7	452	3207	3135	395	0	4045

TRINITY_DN8205_c0_g1_i1	Glyma.10G200000.2	96.86098655	171	8	452	3207	3132	395	0	4026

TRINITY_DN16425_c0_g1_i2	Glyma.12G060600.5	100	221	5	902	3652	2751	1	0	3812

TRINITY_DN16425_c0_g1_i5	Glyma.12G060600.5	99.27007299	221	5	902	3652	2751	1	0	3812

TRINITY_DN4509_c0_g1_i1	Glyma.13G245700.2	95	203	5	142	2889	7	2760	0	3912

TRINITY_DN4509_c0_g1_i1	Glyma.13G245700.1	98.85057471	203	5	142	2889	7	2760	0	3912

TRINITY_DN15789_c0_g1_i1	Glyma.04G032700.1	96.22266402	540	72	1920	4646	2689	5361	0	1397

TRINITY_DN15789_c0_g1_i1	Glyma.04G032700.4	95.56962025	540	72	1920	4646	2413	5085	0	1397

TRINITY_DN15789_c0_g1_i1	Glyma.04G032700.3	95.28508772	540	72	1920	4646	2413	5085	0	1397

TRINITY_DN15789_c0_g1_i1	Glyma.04G032700.2	99.30795848	540	72	1920	4646	2413	5085	0	1397

TRINITY_DN5871_c0_g1_i1	Glyma.09G164300.1	98.64864865	270	19	296	3015	3558	809	0	3345

TRINITY_DN5871_c0_g1_i1	Glyma.09G164300.6	98.87640449	270	19	296	3015	3546	797	0	3345

TRINITY_DN5871_c0_g1_i1	Glyma.09G164300.5	98.87640449	270	19	296	3015	3480	731	0	3345

TRINITY_DN5871_c0_g1_i1	Glyma.09G164300.4	100	270	19	296	3015	3285	536	0	3345

TRINITY_DN5871_c0_g1_i1	Glyma.09G164300.2	96.50924025	270	19	296	3015	3285	536	0	3345

TRINITY_DN5871_c0_g1_i1	Glyma.09G164300.3	98.87640449	270	19	296	3015	3285	536	0	3345

TRINITY_DN15863_c0_g1_i1	Glyma.13G356900.1	100	209	4	274	3020	1	2751	0	3864

TRINITY_DN15863_c0_g1_i1	Glyma.13G356900.2	99.41176471	209	4	274	3020	1	2751	0	3864

TRINITY_DN7739_c0_g2_i1	Glyma.01G081300.1	98.36065574	197	3	317	3067	2785	32	0	3975

TRINITY_DN7739_c0_g2_i1	Glyma.08G262800.1	100	194	6	317	3067	2779	32	0	3952

TRINITY_DN12070_c0_g1_i1	Glyma.18G253400.2	99.02912621	163	2	402	3154	1	2753	0	4170

TRINITY_DN12070_c0_g1_i1	Glyma.18G253400.1	99.02912621	163	2	402	3154	1	2753	0	4170

TRINITY_DN12070_c0_g1_i2	Glyma.18G253400.2	99.02912621	163	2	594	3346	1	2753	0	4170

TRINITY_DN12070_c0_g1_i2	Glyma.18G253400.1	99.01960784	163	2	594	3346	1	2753	0	4170

TRINITY_DN10715_c0_g1_i2	Glyma.15G245800.2	100	155	0	490	3243	3064	5817	0	4228

TRINITY_DN2525_c0_g2_i1	Glyma.12G135700.1	96.66666667	474	20	5	2743	910	3648	0	2278

TRINITY_DN2525_c0_g2_i1	Glyma.12G135700.4	98.828125	474	20	5	2743	778	3516	0	2278

TRINITY_DN2525_c0_g2_i1	Glyma.12G135700.5	98.79032258	474	21	5	2743	778	3507	0	2218

TRINITY_DN10789_c0_g1_i1	Glyma.13G129700.1	100	235	41	277	2945	1	2698	0	2883

TRINITY_DN10789_c0_g1_i1	Glyma.13G129700.2	100	235	41	277	2945	1	2698	0	2883

TRINITY_DN38330_c0_g1_i1	Glyma.20G069700.1	99.21875	228	5	570	3316	1190	3936	0	3744

TRINITY_DN3595_c0_g1_i1	Glyma.03G157700.1	98.64864865	226	4	470	3217	12	2763	0	3794

TRINITY_DN12070_c0_g1_i1	Glyma.09G242200.2	99.02439024	167	0	402	3154	1	2753	0	4159

TRINITY_DN12070_c0_g1_i1	Glyma.09G242200.1	99.01960784	167	0	402	3154	1	2753	0	4159

TRINITY_DN12070_c0_g1_i2	Glyma.09G242200.2	99.02912621	167	0	594	3346	1	2753	0	4159

TRINITY_DN12070_c0_g1_i2	Glyma.09G242200.1	99.0228013	167	0	594	3346	1	2753	0	4159

TRINITY_DN37092_c0_g1_i1	Glyma.11G197900.1	95	231	12	201	2919	2734	1	0	3472

TRINITY_DN37092_c0_g1_i1	Glyma.11G197900.2	100	231	12	201	2919	2734	1	0	3472

TRINITY_DN13926_c0_g1_i2	Glyma.01G234600.2	100	231	29	239	2923	2916	186	0	3234

TRINITY_DN13926_c0_g1_i2	Glyma.01G234600.3	98.01587302	231	29	239	2923	2916	186	0	3234

TRINITY_DN2320_c0_g4_i1	Glyma.10G280700.1	99.35897436	144	4	262	3004	3258	507	0	4226

TRINITY_DN15699_c0_g1_i10	Glyma.19G003200.1	98.34254144	138	2	167	2917	1	2751	0	4305

TRINITY_DN16331_c0_g2_i4	Glyma.13G360100.3	100	108	6	97	2838	1	2751	0	4412

TRINITY_DN16331_c0_g2_i4	Glyma.15G013800.2	100	108	6	97	2838	1	2751	0	4412

TRINITY_DN16130_c2_g6_i16	Glyma.19G196000.1	100	295	8	284	3028	162	2903	0	3349

TRINITY_DN16130_c2_g6_i9	Glyma.19G196000.1	97.22222222	242	8	284	3028	162	2903	0	3642

TRINITY_DN15699_c0_g1_i1	Glyma.19G003200.1	97.84946237	150	0	167	2917	1	2751	0	4250

TRINITY_DN15964_c0_g1_i7	Glyma.13G369300.1	95.79646018	652	54	368	3089	131	2843	0	1046

TRINITY_DN15278_c1_g1_i1	Glyma.08G100100.1	100	318	24	985	3705	2718	1	0	2922

TRINITY_DN16130_c2_g6_i16	Glyma.19G196000.3	97.83549784	295	8	285	3028	1	2741	0	3347

TRINITY_DN16130_c2_g6_i16	Glyma.19G196000.4	97.65886288	295	8	285	3028	1	2741	0	3347

TRINITY_DN16130_c2_g6_i9	Glyma.19G196000.3	97.22222222	242	8	285	3028	1	2741	0	3640

TRINITY_DN16130_c2_g6_i9	Glyma.19G196000.4	100	242	8	285	3028	1	2741	0	3640

TRINITY_DN12540_c0_g1_i2	Glyma.16G077200.1	97.77777778	213	7	420	3163	2856	116	0	3801

TRINITY_DN15964_c0_g1_i8	Glyma.13G369300.1	99.543379	668	52	368	3089	131	2843	0	968

TRINITY_DN14042_c0_g1_i2	Glyma.15G042000.4	100	176	8	663	3389	1	2745	0	3934

TRINITY_DN14042_c0_g1_i2	Glyma.15G042000.5	100	176	8	663	3389	1	2745	0	3934

TRINITY_DN14042_c0_g1_i4	Glyma.15G042000.4	98.92473118	176	8	446	3172	1	2745	0	3934

TRINITY_DN14042_c0_g1_i4	Glyma.15G042000.5	98.46153846	176	8	446	3172	1	2745	0	3934

TRINITY_DN2320_c0_g4_i1	Glyma.20G109000.1	97.08431836	159	2	262	3004	3264	516	0	4157

TRINITY_DN16245_c0_g1_i5	Glyma.13G348200.3	100	345	33	1325	4059	2711	1	0	2841

TRINITY_DN15972_c0_g1_i1	Glyma.04G209000.1	99.1902834	287	10	336	3074	2739	1	0	3369

TRINITY_DN15345_c0_g3_i1	Glyma.06G305000.1	100	261	12	381	3108	1	2746	0	3487

TRINITY_DN15345_c0_g3_i1	Glyma.06G305000.3	96.65738162	261	12	381	3108	1	2746	0	3487

TRINITY_DN2525_c0_g2_i1	Glyma.12G214500.1	100	180	2	6	2741	911	3658	0	4000

TRINITY_DN15278_c1_g1_i1	Glyma.08G100100.2	100	318	23	985	3705	2715	1	0	2937

TRINITY_DN13882_c0_g1_i1	Glyma.14G026900.1	98.94736842	292	24	512	3236	1450	4159	0	3073

TRINITY_DN15867_c0_g3_i3	Glyma.17G052600.2	100	226	8	615	3345	1	2743	0	3692

TRINITY_DN2905_c0_g1_i1	Glyma.09G234500.1	100	210	6	204	2945	1	2745	0	3864

TRINITY_DN12540_c0_g1_i2	Glyma.01G009400.2	100	209	6	420	3163	2853	116	0	3838

TRINITY_DN15964_c0_g1_i5	Glyma.17G199200.1	100	613	58	370	3084	139	2843	0	1210

TRINITY_DN14051_c0_g1_i2	Glyma.18G209600.1	98.30508475	175	0	185	2930	2868	123	0	4102

TRINITY_DN12701_c0_g1_i1	Glyma.08G026700.2	96.07843137	125	6	648	3390	72	2805	0	4281

TRINITY_DN15964_c0_g1_i6	Glyma.17G199200.1	96.01386482	607	56	370	3084	139	2843	0	1254

TRINITY_DN14245_c0_g2_i1	Glyma.13G241400.10	95.65217391	406	38	451	3153	14	2734	0	2394

TRINITY_DN14245_c0_g2_i1	Glyma.13G241400.2	95.65217391	406	38	451	3153	14	2734	0	2394

TRINITY_DN13778_c1_g3_i3	Glyma.16G064100.1	95.53072626	400	25	196	2909	2839	105	0	2599

TRINITY_DN15040_c0_g1_i2	Glyma.13G099300.2	97.75280899	301	9	615	3337	2738	1	0	3225

TRINITY_DN16764_c0_g1_i1	Glyma.11G072100.3	97.26027397	251	16	507	3213	2737	1	0	3393

TRINITY_DN16764_c0_g1_i1	Glyma.11G072100.1	100	251	16	507	3213	2737	1	0	3393

TRINITY_DN16764_c0_g1_i1	Glyma.11G072100.2	100	251	16	507	3213	2737	1	0	3393

TRINITY_DN37092_c0_g1_i1	Glyma.18G052100.4	97.60479042	227	18	201	2919	2725	1	0	3526

TRINITY_DN37092_c0_g1_i1	Glyma.18G052100.2	100	227	18	201	2919	2725	1	0	3526

TRINITY_DN37092_c0_g1_i1	Glyma.18G052100.3	95.61403509	227	18	201	2919	2725	1	0	3526

TRINITY_DN15964_c0_g1_i8	Glyma.17G199200.1	99.18032787	612	52	370	3084	139	2843	0	1249

TRINITY_DN16427_c0_g3_i3	Glyma.13G195200.1	99.23076923	350	8	1	2740	4240	1507	0	3049

TRINITY_DN15458_c0_g1_i4	Glyma.15G121500.1	100	322	17	1	2737	342	3057	0	3068

TRINITY_DN15568_c1_g1_i1	Glyma.12G214600.1	100	273	6	551	3274	1	2742	0	3424

TRINITY_DN16130_c2_g6_i5	Glyma.03G198100.1	100	265	6	300	3036	19	2758	0	3539

TRINITY_DN16130_c2_g6_i5	Glyma.03G198100.2	98.91304348	265	6	300	3036	19	2758	0	3539

TRINITY_DN16764_c0_g1_i1	Glyma.01G171000.2	100	234	20	507	3213	2731	1	0	3459

TRINITY_DN16764_c0_g1_i1	Glyma.01G171000.1	97.26027397	234	20	507	3213	2731	1	0	3459

TRINITY_DN16764_c0_g1_i1	Glyma.01G171000.3	97.26027397	234	20	507	3213	2731	1	0	3459

TRINITY_DN16764_c0_g1_i1	Glyma.01G171000.4	97.26027397	234	20	507	3213	2731	1	0	3459

TRINITY_DN16130_c2_g6_i15	Glyma.03G198100.1	99.76359338	211	6	300	3036	19	2758	0	3838

TRINITY_DN16130_c2_g6_i15	Glyma.03G198100.2	97.72727273	211	6	300	3036	19	2758	0	3838

TRINITY_DN8412_c0_g1_i1	Glyma.08G296800.1	97.59036145	172	1	275	3014	27	2769	0	4093

TRINITY_DN15384_c0_g1_i1	Glyma.06G122500.2	99.06542056	169	6	539	3280	1	2730	0	4039

TRINITY_DN15964_c0_g1_i7	Glyma.17G199200.1	95.23809524	606	50	370	3084	139	2843	0	1293

TRINITY_DN16168_c0_g1_i1	Glyma.05G248500.1	100	360	25	241	2949	3112	389	0	2739

TRINITY_DN9899_c0_g1_i2	Glyma.07G057700.1	98.52941176	360	43	66	2771	1	2685	0	2468

TRINITY_DN9899_c0_g1_i2	Glyma.16G026700.1	96.66666667	351	55	66	2771	1	2679	0	2479

TRINITY_DN14364_c0_g2_i4	Glyma.09G038300.2	97.11538462	278	13	287	3018	2736	5	0	3395

TRINITY_DN14364_c0_g2_i4	Glyma.09G038300.1	100	278	13	287	3018	2736	5	0	3395

TRINITY_DN15458_c0_g1_i1	Glyma.15G121500.1	95.51282051	260	15	1	2737	342	3057	0	3422

TRINITY_DN14283_c0_g1_i3	Glyma.08G282400.1	99.29577465	225	18	436	3168	2919	202	0	3603

TRINITY_DN14283_c0_g1_i1	Glyma.08G282400.1	99.28571429	221	19	436	3144	2919	202	0	3470

TRINITY_DN14283_c0_g1_i3	Glyma.08G282400.11	99.28741093	221	19	436	3168	2895	202	0	3470

TRINITY_DN2525_c0_g2_i1	Glyma.13G287100.1	98.92086331	194	2	2	2737	904	3645	0	3951

TRINITY_DN10789_c0_g2_i1	Glyma.13G129700.1	100	475	90	358	3018	5	2686	0	1531

TRINITY_DN10789_c0_g2_i1	Glyma.13G129700.2	100	475	90	358	3018	5	2686	0	1531

TRINITY_DN16245_c0_g5_i1	Glyma.04G144900.2	100	391	10	1163	3898	2748	13	0	2832

TRINITY_DN16245_c0_g5_i1	Glyma.04G144900.3	100	391	10	1163	3898	2748	13	0	2832

TRINITY_DN16245_c0_g5_i2	Glyma.04G144900.2	97.89473684	342	10	1163	3898	2748	13	0	3103

TRINITY_DN16245_c0_g5_i2	Glyma.04G144900.3	97.89473684	342	10	1163	3898	2748	13	0	3103

TRINITY_DN15458_c0_g1_i4	Glyma.09G015700.1	99.51923077	311	17	1	2736	342	3056	0	3138

TRINITY_DN15458_c0_g1_i1	Glyma.09G015700.1	95.51282051	250	17	1	2736	342	3056	0	3476

TRINITY_DN13926_c0_g1_i1	Glyma.01G234600.4	96.1038961	233	24	239	2912	2919	183	0	3312

TRINITY_DN13926_c0_g1_i1	Glyma.01G234600.5	98.71794872	233	24	239	2912	2919	183	0	3312

TRINITY_DN16427_c0_g3_i3	Glyma.11G157400.1	99.23076923	172	2	1	2740	4888	2149	0	4096

TRINITY_DN16004_c1_g1_i4	Glyma.15G004300.1	98.30508475	588	35	355	3062	118	2843	0	1506

TRINITY_DN16298_c0_g1_i2	Glyma.06G305700.2	97.10144928	317	0	412	3151	2873	134	0	3304

TRINITY_DN14364_c0_g2_i4	Glyma.15G143400.1	100	290	7	287	3018	2736	2	0	3369

TRINITY_DN16298_c0_g1_i1	Glyma.06G305700.2	95.94095941	189	0	364	3103	2873	134	0	4013

TRINITY_DN14245_c0_g2_i3	Glyma.13G241400.10	95.65217391	392	31	393	3098	14	2734	0	2556

TRINITY_DN14245_c0_g2_i3	Glyma.13G241400.2	95.65217391	392	31	393	3098	14	2734	0	2556

TRINITY_DN15568_c1_g1_i1	Glyma.13G287000.3	95.50561798	258	16	551	3274	1	2727	0	3454

TRINITY_DN15568_c1_g1_i1	Glyma.13G287000.1	96.15384615	258	16	551	3274	1	2727	0	3454

TRINITY_DN4283_c0_g1_i1	Glyma.10G210900.2	96.70542636	257	4	151	2883	1	2739	0	3596

TRINITY_DN4283_c0_g1_i1	Glyma.10G210900.1	95.92274678	257	4	151	2883	1	2739	0	3596

TRINITY_DN14767_c0_g2_i1	Glyma.12G051000.1	100	256	18	605	3327	2693	1	0	3240

TRINITY_DN14767_c0_g2_i2	Glyma.12G051000.1	97.99196787	256	18	476	3198	2693	1	0	3240

TRINITY_DN14767_c0_g2_i3	Glyma.12G051000.1	100	256	18	619	3341	2693	1	0	3240

TRINITY_DN14283_c0_g1_i3	Glyma.08G282400.12	99.29577465	225	17	436	3168	2916	202	0	3618

TRINITY_DN14283_c0_g1_i1	Glyma.08G282400.12	99.29078014	221	18	436	3144	2916	202	0	3485

TRINITY_DN14283_c0_g1_i3	Glyma.08G282400.10	99.29577465	221	18	436	3168	2892	202	0	3485

TRINITY_DN2525_c0_g2_i1	Glyma.12G214500.2	100	181	1	6	2741	911	3649	0	4036

TRINITY_DN13707_c0_g1_i1	Glyma.10G157700.1	100	172	10	264	2978	1	2733	0	3912

TRINITY_DN16331_c0_g2_i1	Glyma.07G034700.1	95.89041096	128	1	141	2870	1	2739	0	4290

TRINITY_DN16168_c0_g1_i1	Glyma.08G057200.5	100	363	25	240	2949	3095	380	0	2723

TRINITY_DN14364_c0_g2_i1	Glyma.15G143400.1	95.08196721	241	4	287	3018	2736	2	0	3663

TRINITY_DN11678_c0_g1_i1	Glyma.12G194100.1	100	230	4	132	2867	2730	1	0	3718

TRINITY_DN14364_c0_g2_i1	Glyma.09G038300.2	95.08196721	230	6	287	3018	2736	5	0	3705

TRINITY_DN14364_c0_g2_i1	Glyma.09G038300.1	95.08196721	230	6	287	3018	2736	5	0	3705

TRINITY_DN16168_c0_g1_i1	Glyma.08G057200.2	96.875	363	25	241	2949	3094	380	0	2721

TRINITY_DN16168_c0_g1_i1	Glyma.08G057200.3	96.85314685	363	25	241	2949	3094	380	0	2721

TRINITY_DN16168_c0_g1_i1	Glyma.08G057200.1	96.8537415	363	25	241	2949	3094	380	0	2721

TRINITY_DN16168_c0_g1_i8	Glyma.08G057200.5	96.42857143	363	25	712	3420	3094	380	0	2721

TRINITY_DN16168_c0_g1_i8	Glyma.08G057200.1	98.54368932	363	25	712	3420	3094	380	0	2721

TRINITY_DN16168_c0_g1_i5	Glyma.08G057200.5	100	354	25	808	3516	3094	380	0	2771

TRINITY_DN16168_c0_g1_i5	Glyma.08G057200.1	100	354	25	808	3516	3094	380	0	2771

TRINITY_DN14576_c0_g1_i8	Glyma.09G229900.2	100	273	14	1487	4201	445	3174	0	3354

TRINITY_DN14576_c0_g1_i8	Glyma.09G229900.1	97.36842105	273	14	1487	4201	445	3174	0	3354

TRINITY_DN16331_c0_g2_i2	Glyma.07G034700.1	95.89442815	260	9	97	2815	1	2737	0	3498

TRINITY_DN16331_c0_g2_i4	Glyma.07G034700.1	100	260	9	97	2815	1	2737	0	3498

TRINITY_DN16331_c0_g2_i5	Glyma.07G034700.1	100	260	9	97	2815	1	2737	0	3498

TRINITY_DN16245_c0_g1_i5	Glyma.07G007700.9	97.02380952	183	7	1325	4059	2714	1	0	3879

TRINITY_DN16245_c0_g1_i5	Glyma.07G007700.10	95.88477366	183	7	1325	4059	2714	1	0	3879

TRINITY_DN15836_c0_g2_i1	Glyma.14G197100.1	97.4025974	286	25	80	2765	32	2759	0	3094

TRINITY_DN14518_c0_g1_i1	Glyma.05G022200.1	97.6744186	273	4	178	2904	2736	1	0	3482

TRINITY_DN11678_c0_g1_i1	Glyma.13G308500.1	100	239	1	132	2867	2730	1	0	3690

TRINITY_DN5451_c0_g1_i1	Glyma.12G021900.1	100	197	11	534	3257	2443	5157	0	3747

TRINITY_DN15278_c1_g1_i2	Glyma.08G100100.1	100	193	6	985	3705	2718	1	0	3770

TRINITY_DN15278_c1_g1_i2	Glyma.08G100100.2	99.23954373	193	8	985	3705	2715	1	0	3751

TRINITY_DN14610_c0_g1_i1	Glyma.09G167000.1	96.61399549	191	5	181	2904	2762	27	0	3917

TRINITY_DN16245_c0_g1_i5	Glyma.08G190400.3	99.68454259	180	4	1325	4059	2714	1	0	3906

TRINITY_DN13952_c0_g1_i1	Glyma.08G040500.1	98.4375	151	3	1330	4059	2736	1	0	4178

TRINITY_DN13952_c0_g1_i4	Glyma.08G040500.1	100	151	3	1343	4072	2736	1	0	4178

TRINITY_DN10628_c0_g1_i2	Glyma.05G224700.3	100	138	1	1	2735	1344	4079	0	4281

TRINITY_DN16440_c0_g1_i2	Glyma.03G052800.3	97.5	402	13	633	3352	3836	1107	0	2695

TRINITY_DN16080_c0_g3_i1	Glyma.14G041100.1	95.86563307	337	9	190	2919	1	2721	0	3061

TRINITY_DN16130_c2_g6_i16	Glyma.03G198100.1	98.92473118	290	7	300	3028	19	2747	0	3367

TRINITY_DN16130_c2_g6_i16	Glyma.03G198100.2	99.46808511	290	7	300	3028	19	2747	0	3367

TRINITY_DN13474_c0_g2_i1	Glyma.16G010400.2	98.71794872	247	25	97	2792	97	2801	0	3236

TRINITY_DN16130_c2_g6_i9	Glyma.03G198100.1	97.22222222	236	7	300	3028	19	2747	0	3666

TRINITY_DN16130_c2_g6_i9	Glyma.03G198100.2	97.22222222	236	7	300	3028	19	2747	0	3666

TRINITY_DN13926_c0_g1_i1	Glyma.01G234600.2	95.06398537	232	24	239	2906	2916	186	0	3306

TRINITY_DN13926_c0_g1_i1	Glyma.01G234600.3	96.12903226	232	24	239	2906	2916	186	0	3306

TRINITY_DN14576_c0_g1_i2	Glyma.09G229900.2	97.87685775	273	14	1173	3884	448	3174	0	3349

TRINITY_DN14576_c0_g1_i2	Glyma.09G229900.1	99.50248756	273	14	1173	3884	448	3174	0	3349

TRINITY_DN16252_c0_g1_i3	Glyma.08G293600.5	96.37681159	264	21	1665	4375	21	2731	0	3290

TRINITY_DN14298_c0_g2_i1	Glyma.08G060200.2	96.19047619	261	18	1	2711	428	3144	0	3345

TRINITY_DN14298_c0_g2_i1	Glyma.08G060200.1	96.20253165	261	18	1	2711	428	3144	0	3345

TRINITY_DN15980_c0_g2_i1	Glyma.20G207700.2	99.45355191	481	65	3	2667	1030	3712	0	1620

TRINITY_DN15980_c0_g2_i1	Glyma.20G207700.4	99.45355191	481	65	3	2667	1030	3712	0	1620

TRINITY_DN2731_c0_g2_i1	Glyma.11G130300.1	100	413	27	35	2749	1	2706	0	2468

TRINITY_DN15795_c0_g1_i5	Glyma.16G042600.3	97.13467049	362	54	203	2884	2652	1	0	2189

TRINITY_DN15795_c0_g1_i5	Glyma.16G042600.1	97.13574097	362	54	203	2884	2652	1	0	2189

TRINITY_DN15815_c0_g2_i5	Glyma.12G219400.1	98.4496124	337	14	1489	4210	3117	393	0	3057

TRINITY_DN15278_c1_g1_i2	Glyma.15G091000.4	98.79518072	319	32	987	3705	2692	1	0	2924

TRINITY_DN16331_c0_g2_i1	Glyma.13G360100.3	95.89041096	270	3	141	2872	1	2731	0	3531

TRINITY_DN16331_c0_g2_i1	Glyma.15G013800.2	95.90909091	266	3	141	2872	1	2731	0	3554

TRINITY_DN13474_c0_g2_i4	Glyma.16G010400.2	96.93251534	257	19	97	2810	97	2801	0	3319

TRINITY_DN15278_c1_g1_i2	Glyma.15G091000.1	97.59036145	321	30	987	3705	2695	1	0	2944

TRINITY_DN15795_c0_g1_i1	Glyma.16G042600.3	96.40718563	296	43	151	2844	2652	1	0	2643

TRINITY_DN15795_c0_g1_i1	Glyma.16G042600.1	97.03703704	296	43	151	2844	2652	1	0	2643

TRINITY_DN15795_c0_g1_i3	Glyma.16G042600.3	100	296	43	325	3018	2652	1	0	2643

TRINITY_DN15795_c0_g1_i3	Glyma.16G042600.1	100	296	43	325	3018	2652	1	0	2643

TRINITY_DN16245_c0_g3_i6	Glyma.08G268900.1	100	236	2	301	3032	3569	843	0	3705

TRINITY_DN15768_c0_g1_i1	Glyma.19G178400.2	98.92473118	195	1	117	2848	68	2796	0	3945

TRINITY_DN15768_c0_g1_i1	Glyma.19G178400.1	100	195	1	117	2848	68	2796	0	3945

TRINITY_DN14231_c0_g1_i2	Glyma.08G174100.1	98.03921569	192	23	27	2725	45	2749	0	3594

TRINITY_DN16331_c0_g2_i2	Glyma.13G360100.3	95.89041096	107	6	97	2818	1	2731	0	4381

TRINITY_DN16331_c0_g2_i2	Glyma.15G013800.2	95.89041096	107	6	97	2818	1	2731	0	4381

TRINITY_DN16331_c0_g2_i4	Glyma.13G360100.1	100	107	6	97	2818	1	2731	0	4381

TRINITY_DN16331_c0_g2_i4	Glyma.15G013800.3	100	107	6	97	2818	1	2731	0	4381

TRINITY_DN16331_c0_g2_i4	Glyma.15G013800.1	100	107	6	97	2818	1	2731	0	4381

TRINITY_DN16331_c0_g2_i5	Glyma.13G360100.3	100	107	6	97	2818	1	2731	0	4381

TRINITY_DN16331_c0_g2_i5	Glyma.15G013800.2	100	107	6	97	2818	1	2731	0	4381

TRINITY_DN16080_c0_g3_i1	Glyma.02G274900.1	95.86614173	349	6	190	2919	1	2721	0	3038

TRINITY_DN16252_c0_g1_i3	Glyma.08G293600.3	96.37462236	264	19	1665	4375	21	2728	0	3302

TRINITY_DN14231_c0_g1_i1	Glyma.08G174100.3	98.03921569	192	23	27	2724	45	2748	0	3592

TRINITY_DN14231_c0_g1_i1	Glyma.08G174100.2	98.03921569	192	23	27	2724	45	2748	0	3592

TRINITY_DN14231_c0_g1_i2	Glyma.08G174100.3	98.02259887	192	23	27	2724	45	2748	0	3592

TRINITY_DN14231_c0_g1_i2	Glyma.08G174100.2	98.01324503	192	23	27	2724	45	2748	0	3592

TRINITY_DN15815_c0_g2_i5	Glyma.12G219400.4	100	337	13	1489	4210	3114	393	0	3072

TRINITY_DN15278_c1_g1_i2	Glyma.13G221400.2	100	333	30	987	3705	2686	1	0	2841

TRINITY_DN7971_c0_g1_i2	Glyma.01G133000.1	98.11320755	185	6	489	3209	2727	1	0	3940

TRINITY_DN13619_c0_g1_i3	Glyma.18G288500.6	97.02970297	159	2	1	2730	799	3513	0	4063

TRINITY_DN16331_c0_g2_i1	Glyma.08G207300.1	95.89371981	135	4	141	2870	1	2718	0	4217

TRINITY_DN10834_c0_g1_i1	Glyma.06G315100.1	100	426	37	1	2710	993	3684	0	2318

TRINITY_DN15278_c1_g1_i2	Glyma.13G221400.1	97.61904762	335	28	987	3705	2689	1	0	2861

TRINITY_DN15458_c0_g1_i1	Glyma.07G269000.1	96.79487179	328	25	3	2707	341	3054	0	2970

TRINITY_DN15768_c0_g1_i4	Glyma.19G178400.2	95.42253521	276	11	117	2806	68	2790	0	3219

TRINITY_DN15768_c0_g1_i4	Glyma.19G178400.1	95.21126761	276	11	117	2806	68	2790	0	3219

TRINITY_DN14298_c0_g2_i1	Glyma.07G189100.1	96.20938628	254	20	1	2711	392	3093	0	3341

TRINITY_DN3903_c0_g1_i1	Glyma.15G030000.1	96.25	253	27	567	3266	1	2706	0	3302

TRINITY_DN14231_c0_g1_i2	Glyma.15G253100.1	98.02631579	195	20	27	2725	45	2758	0	3668

TRINITY_DN16440_c0_g1_i2	Glyma.03G052800.1	98.58490566	399	13	633	3345	3944	1222	0	2699

TRINITY_DN16440_c0_g1_i2	Glyma.03G052800.5	100	399	13	633	3345	3863	1141	0	2699

TRINITY_DN16440_c0_g1_i2	Glyma.03G052800.4	100	399	13	633	3345	3839	1117	0	2699

TRINITY_DN16440_c0_g1_i2	Glyma.03G052800.2	98.55072464	399	13	633	3345	3758	1036	0	2699

TRINITY_DN15815_c0_g2_i5	Glyma.13G281900.1	99.71014493	335	10	1489	4210	3114	393	0	3105

TRINITY_DN7273_c0_g1_i1	Glyma.20G179400.2	97.05882353	192	28	358	3049	2702	5	0	3548

TRINITY_DN7273_c0_g1_i1	Glyma.20G179400.3	99.18032787	192	28	358	3049	2702	5	0	3548

TRINITY_DN7273_c0_g1_i2	Glyma.20G179400.2	97.46835443	189	33	358	3038	2702	5	0	3493

TRINITY_DN7273_c0_g1_i2	Glyma.20G179400.3	99.6031746	189	33	358	3038	2702	5	0	3493

TRINITY_DN14042_c0_g1_i2	Glyma.15G042000.2	98.79518072	177	3	663	3389	1	2724	0	4024

TRINITY_DN14042_c0_g1_i2	Glyma.15G042000.3	98.79518072	177	3	663	3389	1	2724	0	4024

TRINITY_DN14042_c0_g1_i4	Glyma.15G042000.2	98.75	177	3	446	3172	1	2724	0	4024

TRINITY_DN14042_c0_g1_i4	Glyma.15G042000.3	98.75	177	3	446	3172	1	2724	0	4024

TRINITY_DN16245_c0_g1_i2	Glyma.13G348200.3	97.24310777	348	24	1325	4038	2711	1	0	2920

TRINITY_DN15538_c0_g1_i5	Glyma.08G333900.3	97.16981132	202	0	443	3169	1	2727	0	3917

TRINITY_DN15538_c0_g1_i5	Glyma.08G333900.1	100	202	0	443	3169	1	2727	0	3917

TRINITY_DN15538_c0_g1_i5	Glyma.08G333900.2	100	202	0	443	3169	1	2727	0	3917

TRINITY_DN15538_c0_g1_i5	Glyma.18G067000.3	98.67549669	197	0	443	3169	1	2727	0	3945

TRINITY_DN15538_c0_g1_i5	Glyma.18G067000.2	98.67549669	197	0	443	3169	1	2727	0	3945

TRINITY_DN15538_c0_g1_i5	Glyma.18G067000.1	97.16981132	197	0	443	3169	1	2727	0	3945

TRINITY_DN14042_c0_g1_i2	Glyma.13G332300.11	95.85798817	195	1	663	3389	1	2724	0	3936

TRINITY_DN14042_c0_g1_i2	Glyma.13G332300.2	95.85798817	195	1	663	3389	1	2724	0	3936

TRINITY_DN14042_c0_g1_i4	Glyma.13G332300.11	100	195	1	446	3172	1	2724	0	3936

TRINITY_DN14042_c0_g1_i4	Glyma.13G332300.2	100	195	1	446	3172	1	2724	0	3936

TRINITY_DN15808_c0_g1_i1	Glyma.06G202000.2	99.11504425	260	9	129	2841	1	2716	0	3445

TRINITY_DN15808_c0_g1_i1	Glyma.06G202000.3	99.11504425	260	9	129	2841	1	2716	0	3445

TRINITY_DN15808_c0_g1_i1	Glyma.06G202000.1	99.11504425	260	9	129	2841	1	2716	0	3445

TRINITY_DN15808_c0_g1_i1	Glyma.06G202000.5	97.14285714	260	9	129	2841	1	2716	0	3445

TRINITY_DN15808_c0_g1_i1	Glyma.06G202000.4	97.39130435	260	9	129	2841	1	2716	0	3445

TRINITY_DN15278_c1_g1_i1	Glyma.13G221400.1	95.75471698	189	14	987	3705	2689	1	0	3714

TRINITY_DN15795_c0_g1_i5	Glyma.19G109500.3	95.8677686	383	35	203	2884	2673	1	0	2307

TRINITY_DN15795_c0_g1_i5	Glyma.19G109500.1	96.58703072	383	35	203	2884	2673	1	0	2307

TRINITY_DN16245_c0_g1_i4	Glyma.13G348200.3	100	318	22	1243	3953	2711	1	0	3101

TRINITY_DN15458_c0_g1_i4	Glyma.07G269000.1	100	292	18	3	2707	341	3054	0	3225

TRINITY_DN14596_c0_g1_i1	Glyma.20G174900.6	99.66555184	212	31	34	2725	1	2695	0	3461

TRINITY_DN16168_c0_g1_i3	Glyma.05G248500.1	96.01769912	204	3	241	2961	3112	389	0	3880

TRINITY_DN9899_c0_g1_i1	Glyma.07G057700.1	95.58823529	320	23	66	2771	1	2685	0	2900

TRINITY_DN15795_c0_g1_i1	Glyma.19G109500.3	96.40718563	308	32	151	2844	2673	1	0	2811

TRINITY_DN15795_c0_g1_i1	Glyma.19G109500.1	96.40718563	308	32	151	2844	2673	1	0	2811

TRINITY_DN15795_c0_g1_i3	Glyma.19G109500.3	96.89440994	308	32	325	3018	2673	1	0	2811

TRINITY_DN15795_c0_g1_i3	Glyma.19G109500.1	96.89440994	308	32	325	3018	2673	1	0	2811

TRINITY_DN15808_c0_g1_i4	Glyma.06G202000.2	95.38461538	256	7	129	2838	1	2716	0	3482

TRINITY_DN15808_c0_g1_i4	Glyma.06G202000.3	97.16312057	256	7	129	2838	1	2716	0	3482

TRINITY_DN15808_c0_g1_i4	Glyma.06G202000.1	97.96511628	256	7	129	2838	1	2716	0	3482

TRINITY_DN15808_c0_g1_i4	Glyma.06G202000.5	98.97435897	256	7	129	2838	1	2716	0	3482

TRINITY_DN15808_c0_g1_i4	Glyma.06G202000.4	98.7012987	256	7	129	2838	1	2716	0	3482

TRINITY_DN15808_c0_g1_i6	Glyma.06G202000.2	95.0617284	256	7	129	2838	1	2716	0	3482

TRINITY_DN15808_c0_g1_i6	Glyma.06G202000.3	100	256	7	129	2838	1	2716	0	3482

TRINITY_DN15808_c0_g1_i6	Glyma.06G202000.1	96.05781866	256	7	129	2838	1	2716	0	3482

TRINITY_DN15808_c0_g1_i6	Glyma.06G202000.5	95.06726457	256	7	129	2838	1	2716	0	3482

TRINITY_DN15808_c0_g1_i6	Glyma.06G202000.4	98.61111111	256	7	129	2838	1	2716	0	3482

TRINITY_DN15867_c0_g3_i2	Glyma.17G052600.3	98.30508475	195	8	1355	4058	987	3699	0	3760

TRINITY_DN9899_c0_g1_i1	Glyma.16G026700.1	99.01960784	316	25	66	2771	1	2679	0	2894

TRINITY_DN13433_c0_g2_i1	Glyma.03G151400.1	97.4137931	315	29	3	2695	3371	694	0	2809

TRINITY_DN13433_c0_g2_i1	Glyma.03G151400.2	97.4137931	315	29	3	2695	3371	694	0	2809

TRINITY_DN11578_c0_g1_i1	Glyma.19G216000.1	98.80952381	206	8	50	2760	568	3279	0	3749

TRINITY_DN15867_c0_g3_i2	Glyma.13G106700.15	100	189	5	1355	4058	987	3705	0	3843

TRINITY_DN15867_c0_g3_i2	Glyma.13G106700.8	97.01492537	189	5	1355	4058	987	3705	0	3843

TRINITY_DN15867_c0_g3_i2	Glyma.13G106700.9	97.43589744	189	5	1355	4058	987	3705	0	3843

TRINITY_DN15867_c0_g3_i2	Glyma.13G106700.10	96.96202532	189	5	1355	4058	987	3705	0	3843

TRINITY_DN15867_c0_g3_i2	Glyma.13G106700.5	98.96907216	189	5	1355	4058	987	3705	0	3843

TRINITY_DN15867_c0_g3_i2	Glyma.13G106700.6	100	189	5	1355	4058	987	3705	0	3843

TRINITY_DN15278_c1_g1_i1	Glyma.13G221400.2	96.69421488	189	13	987	3705	2686	1	0	3727

TRINITY_DN15278_c1_g1_i1	Glyma.15G091000.1	96.25	178	14	987	3705	2695	1	0	3847

TRINITY_DN15980_c0_g2_i3	Glyma.20G207700.2	100	370	42	3	2685	1030	3712	0	2484

TRINITY_DN15980_c0_g2_i3	Glyma.20G207700.4	99.13793103	370	42	3	2685	1030	3712	0	2484

TRINITY_DN14364_c0_g2_i5	Glyma.09G038300.2	96.92307692	306	15	287	2996	2736	27	0	3188

TRINITY_DN14364_c0_g2_i5	Glyma.09G038300.1	98.19277108	306	15	287	2996	2736	27	0	3188

TRINITY_DN16331_c0_g2_i2	Glyma.08G207300.1	95.91527988	270	5	97	2815	1	2716	0	3483

TRINITY_DN16331_c0_g2_i4	Glyma.08G207300.1	100	270	5	97	2815	1	2716	0	3483

TRINITY_DN16331_c0_g2_i5	Glyma.08G207300.1	100	270	5	97	2815	1	2716	0	3483

TRINITY_DN11893_c0_g1_i1	Glyma.10G075900.1	98	174	3	104	2818	1	2721	0	4023

TRINITY_DN11893_c0_g1_i1	Glyma.02G200700.1	98	170	4	104	2818	1	2721	0	4045

TRINITY_DN2211_c0_g1_i1	Glyma.19G263400.2	100	139	1	79	2799	1	2715	0	4217

TRINITY_DN3903_c0_g1_i1	Glyma.13G344400.1	99.68652038	246	20	567	3266	1	2703	0	3421

TRINITY_DN7971_c0_g1_i2	Glyma.03G035200.1	98.11320755	155	7	500	3209	2716	1	0	4074

TRINITY_DN14364_c0_g2_i5	Glyma.15G143400.1	98	317	9	287	2996	2736	24	0	3168

TRINITY_DN13433_c0_g2_i1	Glyma.19G153900.1	97.4137931	297	22	3	2695	3371	679	0	3040

TRINITY_DN13433_c0_g2_i1	Glyma.19G153900.4	97.4137931	297	22	3	2695	2996	304	0	3040

TRINITY_DN15278_c1_g1_i1	Glyma.15G091000.4	98.61111111	178	13	987	3705	2692	1	0	3860

TRINITY_DN15795_c0_g1_i5	Glyma.16G042600.4	100	362	48	203	2884	2637	1	0	2257

TRINITY_DN15795_c0_g1_i5	Glyma.16G042600.2	97.13467049	362	48	203	2884	2637	1	0	2257

TRINITY_DN9812_c0_g2_i1	Glyma.15G021300.1	96.07843137	237	0	246	2963	2718	1	0	3707

TRINITY_DN14283_c0_g1_i1	Glyma.08G282400.11	99	221	18	436	3144	2895	202	0	3581

TRINITY_DN15405_c0_g1_i2	Glyma.06G323700.1	97.22222222	190	11	213	2912	2709	1	0	3792

TRINITY_DN9431_c0_g1_i1	Glyma.05G223700.1	97.04968944	146	3	1	2718	340	3050	0	4165

TRINITY_DN15242_c0_g1_i2	Glyma.10G188600.2	95.01557632	429	36	221	2906	3267	576	0	2279

TRINITY_DN15757_c0_g1_i2	Glyma.18G003000.1	98.7654321	329	9	128	2836	76	2787	0	3110

TRINITY_DN15757_c0_g1_i3	Glyma.18G003000.1	99.07407407	303	8	128	2836	76	2787	0	3254

TRINITY_DN15795_c0_g1_i1	Glyma.16G042600.4	96.40718563	296	34	151	2844	2637	1	0	2712

TRINITY_DN15795_c0_g1_i1	Glyma.16G042600.2	96.40718563	296	34	151	2844	2637	1	0	2712

TRINITY_DN15795_c0_g1_i3	Glyma.16G042600.4	100	296	34	325	3018	2637	1	0	2712

TRINITY_DN15795_c0_g1_i3	Glyma.16G042600.2	100	296	34	325	3018	2637	1	0	2712

TRINITY_DN15757_c0_g1_i5	Glyma.18G003000.1	100	290	9	128	2836	76	2787	0	3326

TRINITY_DN14364_c0_g2_i6	Glyma.15G143400.1	100	268	6	287	2996	2736	24	0	3461

TRINITY_DN14364_c0_g2_i6	Glyma.09G038300.2	100	258	8	287	2996	2736	27	0	3498

TRINITY_DN14364_c0_g2_i6	Glyma.09G038300.1	100	258	8	287	2996	2736	27	0	3498

TRINITY_DN12730_c0_g2_i1	Glyma.17G229100.1	95.91836735	221	20	487	3165	2784	76	0	3496

TRINITY_DN16245_c0_g1_i4	Glyma.07G007700.9	95.23809524	222	5	1243	3953	2714	1	0	3740

TRINITY_DN16245_c0_g1_i4	Glyma.07G007700.10	95.23809524	222	5	1243	3953	2714	1	0	3740

TRINITY_DN16245_c0_g1_i2	Glyma.07G007700.9	96.19047619	184	4	1325	4038	2714	1	0	3971

TRINITY_DN16245_c0_g1_i2	Glyma.07G007700.10	100	184	4	1325	4038	2714	1	0	3971

TRINITY_DN14283_c0_g1_i1	Glyma.08G282400.10	99	221	17	436	3144	2892	202	0	3596

TRINITY_DN16245_c0_g1_i4	Glyma.08G190400.3	100	216	4	1243	3953	2714	1	0	3784

TRINITY_DN16245_c0_g1_i2	Glyma.08G190400.3	97.1291866	180	2	1325	4038	2714	1	0	4004

TRINITY_DN15757_c0_g1_i2	Glyma.11G254000.1	98.7654321	329	4	127	2836	75	2784	0	3138

TRINITY_DN15757_c0_g1_i3	Glyma.11G254000.1	100	304	5	127	2836	75	2784	0	3277

TRINITY_DN15757_c0_g1_i5	Glyma.11G254000.1	99.26470588	291	4	127	2836	75	2784	0	3349

TRINITY_DN13882_c0_g1_i1	Glyma.02G288000.5	100	275	19	548	3236	1432	4129	0	3223

TRINITY_DN13882_c0_g1_i1	Glyma.02G288000.1	98.6013986	275	19	548	3236	1432	4129	0	3223

TRINITY_DN13882_c0_g1_i1	Glyma.02G288000.3	98.75	275	19	548	3236	1384	4081	0	3223

TRINITY_DN13882_c0_g1_i1	Glyma.02G288000.4	100	275	19	548	3236	1384	4081	0	3223

TRINITY_DN15466_c0_g1_i2	Glyma.06G162200.1	97.93814433	203	10	324	3025	149	2853	0	3751

TRINITY_DN3903_c0_g1_i1	Glyma.12G052300.1	96.25	427	33	596	3266	153	2841	0	2230

TRINITY_DN15242_c0_g1_i1	Glyma.10G188600.2	100	320	31	221	2900	3267	576	0	2900

TRINITY_DN14275_c0_g1_i1	Glyma.12G136800.1	100	262	41	282	2930	2922	226	0	3053

TRINITY_DN14275_c0_g1_i1	Glyma.12G136800.2	100	262	41	282	2930	2922	226	0	3053

TRINITY_DN326_c0_g1_i1	Glyma.03G253000.6	100	100	1	1301	4009	1	2712	0	4434

TRINITY_DN326_c0_g1_i2	Glyma.03G253000.6	96.93654267	100	1	1256	3964	1	2712	0	4434

TRINITY_DN15757_c0_g1_i4	Glyma.18G003000.1	97.87234043	331	6	152	2855	81	2787	0	3101

TRINITY_DN15757_c0_g1_i6	Glyma.18G003000.1	100	318	7	152	2855	81	2787	0	3173

TRINITY_DN15757_c0_g1_i7	Glyma.18G003000.1	100	292	6	152	2855	81	2787	0	3317

TRINITY_DN15757_c0_g1_i1	Glyma.18G003000.1	98.98534386	279	7	152	2855	81	2787	0	3389

TRINITY_DN16045_c0_g2_i1	Glyma.15G013000.1	100	330	8	752	3454	2706	1	0	3105

TRINITY_DN14216_c0_g1_i1	Glyma.19G159400.4	100	231	1	104	2807	78	2787	0	3687

TRINITY_DN10834_c0_g1_i1	Glyma.15G215400.3	98.31460674	209	1	1	2710	843	3546	0	3808

TRINITY_DN10834_c0_g1_i1	Glyma.15G215400.1	100	209	1	1	2710	843	3546	0	3808

TRINITY_DN10834_c0_g1_i1	Glyma.15G215400.2	100	208	3	1	2710	843	3543	0	3794

TRINITY_DN10212_c0_g2_i1	Glyma.15G192300.1	98.54368932	184	4	519	3226	3090	383	0	3960

TRINITY_DN15795_c0_g1_i6	Glyma.16G042600.3	98.33333333	363	53	203	2860	2652	1	0	2294

TRINITY_DN15795_c0_g1_i6	Glyma.16G042600.1	98.46153846	363	53	203	2860	2652	1	0	2294

TRINITY_DN15764_c0_g1_i1	Glyma.16G181000.1	98.96907216	339	16	478	3165	1	2697	0	2911

TRINITY_DN15757_c0_g1_i4	Glyma.11G254000.1	100	325	7	152	2855	81	2784	0	3138

TRINITY_DN15757_c0_g1_i6	Glyma.11G254000.1	96	312	6	152	2855	81	2784	0	3210

TRINITY_DN15757_c0_g1_i7	Glyma.11G254000.1	100	287	7	152	2855	81	2784	0	3349

TRINITY_DN15757_c0_g1_i1	Glyma.11G254000.1	98.98534386	274	6	152	2855	81	2784	0	3421

TRINITY_DN15836_c0_g2_i1	Glyma.02G229400.1	96.45776567	263	23	121	2765	1	2705	0	3107

TRINITY_DN10834_c0_g1_i1	Glyma.08G228800.1	100	222	1	2	2710	844	3546	0	3735

TRINITY_DN10834_c0_g1_i1	Glyma.08G228800.3	97.89103691	222	1	2	2710	103	2805	0	3735

TRINITY_DN16045_c0_g2_i2	Glyma.07G034300.1	96.92307692	221	5	752	3457	2706	1	0	3740

TRINITY_DN15433_c0_g2_i3	Glyma.08G214000.1	95.91836735	221	11	766	3442	5	2711	0	3559

TRINITY_DN15433_c0_g2_i4	Glyma.08G214000.1	100	221	11	941	3617	5	2711	0	3559

TRINITY_DN16314_c1_g1_i2	Glyma.05G187300.1	97.99107143	170	1	1833	4541	2706	1	0	4041

TRINITY_DN16314_c1_g1_i2	Glyma.08G145600.1	100	160	1	1833	4541	2706	1	0	4096

TRINITY_DN5798_c0_g1_i1	Glyma.09G079100.3	100	154	5	248	2949	2709	5	0	4078

TRINITY_DN5798_c0_g1_i1	Glyma.09G079100.2	95.75892857	154	5	248	2949	2709	5	0	4078

TRINITY_DN5798_c0_g1_i1	Glyma.09G079100.1	95.75971731	154	5	248	2949	2709	5	0	4078

TRINITY_DN16141_c0_g1_i1	Glyma.06G003000.8	100	106	3	691	3381	3171	463	0	4300

TRINITY_DN16141_c0_g1_i1	Glyma.06G003000.1	100	106	3	691	3381	3171	463	0	4300

TRINITY_DN16141_c0_g1_i3	Glyma.06G003000.8	100	106	3	691	3381	3171	463	0	4300

TRINITY_DN16141_c0_g1_i3	Glyma.06G003000.1	99.12280702	106	3	691	3381	3171	463	0	4300

TRINITY_DN16045_c0_g2_i1	Glyma.13G360900.1	100	342	7	752	3454	2703	1	0	3042

TRINITY_DN15795_c0_g1_i2	Glyma.16G042600.3	96.07843137	297	42	151	2820	2652	1	0	2748

TRINITY_DN15795_c0_g1_i2	Glyma.16G042600.1	96.07843137	297	42	151	2820	2652	1	0	2748

TRINITY_DN15795_c0_g1_i4	Glyma.16G042600.3	98.7394958	297	42	325	2994	2652	1	0	2748

TRINITY_DN15795_c0_g1_i4	Glyma.16G042600.1	98.25581395	297	42	325	2994	2652	1	0	2748

TRINITY_DN16045_c0_g2_i2	Glyma.13G360900.1	96.22641509	273	6	752	3457	2703	1	0	3443

TRINITY_DN16331_c0_g2_i1	Glyma.13G360100.2	95.91194969	266	2	141	2847	1	2707	0	3515

TRINITY_DN16045_c0_g2_i2	Glyma.15G013000.1	96.92307692	262	4	752	3457	2706	1	0	3524

TRINITY_DN14245_c0_g2_i1	Glyma.13G181700.3	95.65217391	174	7	438	3121	1	2702	0	3843

TRINITY_DN16331_c0_g2_i2	Glyma.13G360100.2	95.90909091	107	6	97	2794	1	2707	0	4337

TRINITY_DN16331_c0_g2_i4	Glyma.13G360100.2	100	107	6	97	2794	1	2707	0	4337

TRINITY_DN16331_c0_g2_i5	Glyma.13G360100.2	100	107	6	97	2794	1	2707	0	4337

TRINITY_DN16141_c0_g1_i1	Glyma.04G003000.1	100	104	2	691	3386	3165	458	0	4348

TRINITY_DN16141_c0_g1_i3	Glyma.04G003000.1	100	104	2	691	3386	3165	458	0	4348

TRINITY_DN16141_c0_g1_i1	Glyma.04G003000.11	95.96412556	104	4	691	3386	3156	458	0	4289

TRINITY_DN16141_c0_g1_i3	Glyma.04G003000.11	100	104	4	691	3386	3156	458	0	4289

TRINITY_DN16314_c1_g1_i3	Glyma.16G165900.2	100	514	40	707	3393	2690	13	0	1834

TRINITY_DN14596_c0_g1_i2	Glyma.20G174900.6	98.95833333	214	24	34	2708	1	2695	0	3530

TRINITY_DN9761_c0_g1_i4	Glyma.10G044200.2	96.55797101	175	13	476	3160	1	2676	0	3687

TRINITY_DN9761_c0_g1_i4	Glyma.10G044200.1	96.55172414	175	13	476	3160	1	2676	0	3687

TRINITY_DN16045_c0_g2_i1	Glyma.07G034300.1	100	142	3	752	3454	2706	1	0	4180

TRINITY_DN17837_c0_g1_i1	Glyma.06G116900.1	95.78947368	120	0	1	2707	505	3211	0	4335

TRINITY_DN13778_c1_g3_i3	Glyma.16G064200.1	95.53072626	398	21	218	2909	2787	105	0	2553

TRINITY_DN16314_c1_g2_i1	Glyma.16G165900.2	100	205	7	446	3147	2694	2	0	3751

TRINITY_DN15984_c0_g1_i2	Glyma.11G015700.1	97.26027397	193	11	378	3074	2697	1	0	3812

TRINITY_DN16314_c1_g2_i3	Glyma.16G165900.2	98.79518072	181	10	446	3141	2694	2	0	3845

TRINITY_DN9507_c0_g1_i1	Glyma.11G080600.1	99.22779923	148	0	113	2818	1	2706	0	4178

TRINITY_DN9507_c0_g2_i1	Glyma.11G080600.1	98.7012987	148	0	113	2818	1	2706	0	4178

TRINITY_DN9507_c0_g1_i1	Glyma.01G162800.1	95.8677686	134	0	113	2818	1	2706	0	4255

TRINITY_DN9507_c0_g2_i1	Glyma.01G162800.1	100	134	0	113	2818	1	2706	0	4255

TRINITY_DN16141_c0_g1_i1	Glyma.06G003000.7	97.27272727	106	2	691	3381	3168	463	0	4313

TRINITY_DN16141_c0_g1_i1	Glyma.06G003000.4	96.96969697	106	2	691	3381	3168	463	0	4313

TRINITY_DN16141_c0_g1_i3	Glyma.06G003000.7	100	106	2	691	3381	3168	463	0	4313

TRINITY_DN16141_c0_g1_i3	Glyma.06G003000.4	97.05882353	106	2	691	3381	3168	463	0	4313

TRINITY_DN15634_c2_g1_i3	Glyma.10G203000.1	100	258	4	1272	3974	2703	1	0	3541

TRINITY_DN11755_c0_g1_i1	Glyma.09G273300.1	100	218	7	561	3263	1	2691	0	3685

TRINITY_DN12724_c0_g1_i2	Glyma.05G241500.2	97.97297297	147	3	176	2880	1	2696	0	4122

TRINITY_DN16141_c0_g1_i1	Glyma.04G003000.12	100	104	1	691	3386	3162	458	0	4361

TRINITY_DN16141_c0_g1_i3	Glyma.04G003000.12	100	104	1	691	3386	3162	458	0	4361

TRINITY_DN16141_c0_g1_i1	Glyma.04G003000.10	100	104	3	691	3386	3153	458	0	4303

TRINITY_DN16141_c0_g1_i3	Glyma.04G003000.10	98.48484848	104	3	691	3386	3153	458	0	4303

TRINITY_DN16045_c0_g2_i2	Glyma.08G207800.1	100	217	0	754	3457	2704	1	0	3792

TRINITY_DN14051_c0_g1_i1	Glyma.09G279500.1	100	186	19	627	3313	2710	24	0	3744

TRINITY_DN16045_c0_g2_i1	Glyma.08G207800.1	99.78795589	135	2	754	3454	2704	1	0	4226

TRINITY_DN15795_c0_g1_i1	Glyma.16G152500.1	96.40635798	258	15	151	2844	2661	1	0	3232

TRINITY_DN15795_c0_g1_i3	Glyma.16G152500.1	95.66210046	258	15	325	3018	2661	1	0	3232

TRINITY_DN15634_c2_g1_i3	Glyma.10G203000.2	97.22222222	255	8	1272	3974	2682	1	0	3443

TRINITY_DN2620_c0_g1_i1	Glyma.11G102000.1	100	224	7	87	2771	2745	43	0	3635

TRINITY_DN15054_c0_g1_i1	Glyma.13G298300.1	98.5915493	189	6	1	2703	424	3105	0	3808

TRINITY_DN15054_c0_g1_i1	Glyma.12G203300.1	98.58906526	175	0	1	2703	424	3126	0	4023

TRINITY_DN15054_c0_g1_i1	Glyma.12G203300.2	98.58906526	175	0	1	2703	424	3126	0	4023

TRINITY_DN5798_c0_g1_i1	Glyma.15G187500.8	98.93491124	163	3	248	2949	2703	5	0	4056

TRINITY_DN5798_c0_g1_i1	Glyma.15G187500.7	100	163	3	248	2949	2703	5	0	4056

TRINITY_DN5798_c0_g1_i1	Glyma.15G187500.5	100	163	3	248	2949	2703	5	0	4056

TRINITY_DN5798_c0_g1_i1	Glyma.15G187500.6	97.43589744	163	3	248	2949	2703	5	0	4056

TRINITY_DN5798_c0_g1_i1	Glyma.15G187500.4	95.11494253	163	3	248	2949	2703	5	0	4056

TRINITY_DN5798_c0_g1_i1	Glyma.15G187500.1	95.11568123	163	3	248	2949	2703	5	0	4056

TRINITY_DN5798_c0_g1_i1	Glyma.15G187500.2	98.4375	163	3	248	2949	2703	5	0	4056

TRINITY_DN5798_c0_g1_i1	Glyma.15G187500.3	96.80851064	163	3	248	2949	2703	5	0	4056

TRINITY_DN15764_c0_g1_i5	Glyma.09G135100.1	96.875	169	9	1	2689	3	2694	0	3904

TRINITY_DN16441_c4_g2_i5	Glyma.13G175200.1	95.56451613	438	37	847	3519	5022	2353	0	2180

TRINITY_DN14216_c0_g1_i2	Glyma.19G159400.4	100	380	4	268	2967	91	2787	0	2850

TRINITY_DN10834_c0_g1_i1	Glyma.04G129200.1	97.95918367	421	22	1	2686	927	3606	0	2435

TRINITY_DN10834_c0_g1_i1	Glyma.04G129200.2	100	421	22	1	2686	648	3327	0	2435

TRINITY_DN15795_c0_g1_i6	Glyma.19G109500.3	95.39748954	384	34	203	2860	2673	1	0	2412

TRINITY_DN15795_c0_g1_i6	Glyma.19G109500.1	96.36871508	384	34	203	2860	2673	1	0	2412

TRINITY_DN19847_c0_g1_i1	Glyma.18G209100.1	96.47058824	315	12	1	2675	1299	3987	0	3009

TRINITY_DN11755_c0_g1_i1	Glyma.18G215800.1	96.93877551	228	9	566	3263	3	2685	0	3600

TRINITY_DN15984_c0_g1_i1	Glyma.11G015700.1	100	135	1	378	3077	2697	1	0	4218

TRINITY_DN15984_c0_g1_i1	Glyma.01G227000.1	95.96774194	132	3	378	3077	2676	1	0	4100

TRINITY_DN16141_c0_g1_i1	Glyma.06G003000.6	99.29078014	106	2	691	3381	3162	463	0	4340

TRINITY_DN16141_c0_g1_i1	Glyma.06G003000.3	97.27272727	106	2	691	3381	3162	463	0	4340

TRINITY_DN16141_c0_g1_i3	Glyma.06G003000.6	97.5	106	2	691	3381	3162	463	0	4340

TRINITY_DN16141_c0_g1_i3	Glyma.06G003000.3	95.55555556	106	2	691	3381	3162	463	0	4340

TRINITY_DN15795_c0_g1_i2	Glyma.19G109500.3	96.07843137	309	31	151	2820	2673	1	0	2916

TRINITY_DN15795_c0_g1_i2	Glyma.19G109500.1	96.07843137	309	31	151	2820	2673	1	0	2916

TRINITY_DN15795_c0_g1_i4	Glyma.19G109500.3	100	309	31	325	2994	2673	1	0	2916

TRINITY_DN15795_c0_g1_i4	Glyma.19G109500.1	100	309	31	325	2994	2673	1	0	2916

TRINITY_DN16141_c0_g1_i1	Glyma.04G003000.3	95	104	1	691	3386	3156	458	0	4388

TRINITY_DN16141_c0_g1_i3	Glyma.04G003000.3	100	104	1	691	3386	3156	458	0	4388

TRINITY_DN16141_c0_g1_i1	Glyma.04G003000.9	97.27272727	104	3	691	3386	3147	458	0	4331

TRINITY_DN16141_c0_g1_i3	Glyma.04G003000.9	95	104	3	691	3386	3147	458	0	4331

TRINITY_DN17316_c0_g1_i1	Glyma.13G220400.3	97.52475248	306	32	152	2818	2946	295	0	2789

TRINITY_DN15531_c0_g1_i2	Glyma.02G310900.1	98.7012987	628	44	1224	3886	1122	3796	0	1140

TRINITY_DN15531_c0_g1_i2	Glyma.02G310900.2	98.7012987	628	44	1224	3886	180	2854	0	1140

TRINITY_DN17316_c0_g1_i1	Glyma.15G092000.1	100	315	20	152	2818	4287	1618	0	2867

TRINITY_DN15984_c0_g1_i2	Glyma.01G227000.1	97.08737864	196	10	378	3074	2676	1	0	3759

TRINITY_DN9761_c0_g1_i4	Glyma.13G131800.1	96.55172414	178	10	476	3160	1	2670	0	3742

TRINITY_DN15795_c0_g1_i5	Glyma.16G152500.1	98.36065574	176	13	203	2884	2661	1	0	3675

TRINITY_DN10760_c0_g1_i1	Glyma.02G111600.6	95.86028461	162	1	226	2922	2730	37	0	4063

TRINITY_DN10760_c0_g1_i1	Glyma.02G111600.2	95.86206897	162	1	226	2922	2730	37	0	4063

TRINITY_DN14779_c0_g1_i2	Glyma.17G246600.1	95.39473684	151	8	101	2784	2690	1	0	4015

TRINITY_DN16141_c0_g1_i1	Glyma.06G003000.5	100	106	1	691	3381	3159	463	0	4355

TRINITY_DN16141_c0_g1_i1	Glyma.06G003000.2	98.02631579	106	1	691	3381	3159	463	0	4355

TRINITY_DN16141_c0_g1_i3	Glyma.06G003000.5	100	106	1	691	3381	3159	463	0	4355

TRINITY_DN16141_c0_g1_i3	Glyma.06G003000.2	100	106	1	691	3381	3159	463	0	4355

TRINITY_DN7739_c0_g2_i1	Glyma.10G274700.1	96.96969697	386	24	312	2990	2778	100	0	2621

TRINITY_DN12547_c0_g2_i1	Glyma.13G032400.2	97.84172662	273	4	189	2880	162	2850	0	3395

TRINITY_DN15984_c0_g1_i1	Glyma.12G021600.1	98.61111111	260	5	386	3077	2689	1	0	3467

TRINITY_DN12547_c0_g2_i2	Glyma.13G032400.2	95.72649573	214	4	189	2880	162	2850	0	3722

TRINITY_DN15795_c0_g1_i5	Glyma.02G071300.1	98.26589595	164	13	204	2884	2660	1	0	3740

TRINITY_DN14779_c0_g1_i2	Glyma.09G114500.2	95.45454545	149	5	101	2784	2690	1	0	4037

TRINITY_DN14779_c0_g1_i2	Glyma.09G114500.1	95.39473684	149	5	101	2784	2690	1	0	4037

TRINITY_DN16141_c0_g1_i1	Glyma.04G003000.2	100	104	0	691	3386	3153	458	0	4403

TRINITY_DN16141_c0_g1_i3	Glyma.04G003000.2	99.47643979	104	0	691	3386	3153	458	0	4403

TRINITY_DN16141_c0_g1_i1	Glyma.04G003000.8	98.21428571	104	2	691	3386	3144	458	0	4344

TRINITY_DN16141_c0_g1_i3	Glyma.04G003000.8	96.2962963	104	2	691	3386	3144	458	0	4344

TRINITY_DN16425_c0_g1_i3	Glyma.15G035900.2	97.26027397	364	17	427	3096	3165	484	0	2715

TRINITY_DN16425_c0_g1_i1	Glyma.15G035900.2	96.50582363	358	17	494	3163	3165	484	0	2748

TRINITY_DN9977_c0_g1_i2	Glyma.U028300.4	100	293	16	1	2679	2661	1	0	3031

TRINITY_DN9977_c0_g1_i2	Glyma.U028300.3	100	293	16	1	2679	2661	1	0	3031

TRINITY_DN9977_c0_g1_i2	Glyma.U028300.1	100	293	16	1	2679	2661	1	0	3031

TRINITY_DN9977_c0_g1_i2	Glyma.U028300.2	100	293	16	1	2679	2661	1	0	3031

TRINITY_DN14287_c0_g1_i1	Glyma.15G108000.2	100	161	2	385	3078	2697	4	0	4072

TRINITY_DN14779_c0_g1_i3	Glyma.17G246600.1	95.43147208	112	7	204	2885	2691	1	0	4246

TRINITY_DN16016_c0_g1_i4	Glyma.16G163200.1	97.18309859	363	15	1337	4027	2920	245	0	2828

TRINITY_DN16016_c0_g1_i5	Glyma.16G163200.1	100	363	15	1337	4027	2920	245	0	2828

TRINITY_DN15795_c0_g1_i6	Glyma.16G042600.4	96.36913767	363	47	203	2860	2637	1	0	2362

TRINITY_DN15795_c0_g1_i6	Glyma.16G042600.2	96.36963696	363	47	203	2860	2637	1	0	2362

TRINITY_DN11681_c0_g1_i1	Glyma.06G055800.1	100	311	15	298	2975	1	2678	0	3046

TRINITY_DN16033_c0_g1_i2	Glyma.10G154400.2	96.61016949	240	4	606	3294	2695	4	0	3600

TRINITY_DN16314_c1_g2_i4	Glyma.16G165900.2	98.78787879	176	1	446	3136	2694	1	0	3980

TRINITY_DN16314_c1_g2_i2	Glyma.16G165900.2	100	152	4	446	3130	2694	1	0	4074

TRINITY_DN14779_c0_g1_i3	Glyma.09G114500.2	95.38461538	109	4	204	2885	2691	1	0	4274

TRINITY_DN14779_c0_g1_i3	Glyma.09G114500.1	95.39748954	109	4	204	2885	2691	1	0	4274

TRINITY_DN15795_c0_g1_i2	Glyma.16G042600.4	96.07843137	297	33	151	2820	2637	1	0	2817

TRINITY_DN15795_c0_g1_i2	Glyma.16G042600.2	96.07843137	297	33	151	2820	2637	1	0	2817

TRINITY_DN15795_c0_g1_i4	Glyma.16G042600.4	100	297	33	325	2994	2637	1	0	2817

TRINITY_DN15795_c0_g1_i4	Glyma.16G042600.2	100	297	33	325	2994	2637	1	0	2817

TRINITY_DN16033_c0_g1_i1	Glyma.10G154400.2	96.03960396	263	3	606	3294	2695	4	0	3483

TRINITY_DN16318_c0_g1_i6	Glyma.09G028000.1	95.74468085	259	6	521	3198	2694	26	0	3286

TRINITY_DN11268_c2_g1_i1	Glyma.15G060100.1	100	250	13	54	2724	1	2683	0	3382

TRINITY_DN12617_c0_g1_i2	Glyma.15G170000.11	100	233	10	1817	4505	1230	3900	0	3515

TRINITY_DN12617_c0_g1_i2	Glyma.15G170000.7	98.14814815	233	10	1817	4505	1230	3900	0	3515

TRINITY_DN12617_c0_g1_i2	Glyma.15G170000.5	98.14814815	233	10	1817	4505	1230	3900	0	3515

TRINITY_DN12617_c0_g1_i2	Glyma.15G170000.1	98.14814815	233	10	1817	4505	1230	3900	0	3515

TRINITY_DN12617_c0_g1_i2	Glyma.15G170000.9	100	233	10	1817	4505	1230	3900	0	3515

TRINITY_DN12617_c0_g1_i2	Glyma.15G170000.8	100	233	10	1817	4505	1224	3894	0	3515

TRINITY_DN12617_c0_g1_i2	Glyma.15G170000.6	100	233	10	1817	4505	1224	3894	0	3515

TRINITY_DN12617_c0_g1_i2	Glyma.15G170000.10	98.50746269	233	10	1817	4505	1206	3876	0	3515

TRINITY_DN12617_c0_g1_i2	Glyma.15G170000.4	100	233	10	1817	4505	1206	3876	0	3515

TRINITY_DN12617_c0_g1_i2	Glyma.15G170000.15	98.50746269	233	10	1817	4505	1182	3852	0	3515

TRINITY_DN12617_c0_g1_i2	Glyma.15G170000.3	98.49246231	233	10	1817	4505	1182	3852	0	3515

TRINITY_DN12617_c0_g1_i2	Glyma.15G170000.14	98.5	233	10	1817	4505	1158	3828	0	3515

TRINITY_DN12617_c0_g1_i2	Glyma.15G170000.2	98.50746269	233	10	1817	4505	1158	3828	0	3515

TRINITY_DN12617_c0_g1_i2	Glyma.15G170000.12	98.50746269	233	10	1817	4505	1137	3807	0	3515

TRINITY_DN12617_c0_g1_i2	Glyma.15G170000.13	98.4962406	233	10	1817	4505	1137	3807	0	3515

TRINITY_DN16332_c0_g3_i3	Glyma.17G239500.5	100	143	2	1	2690	2693	1	0	4161

TRINITY_DN16332_c0_g3_i3	Glyma.17G239500.1	100	143	2	1	2690	2693	1	0	4161

TRINITY_DN8943_c0_g1_i2	Glyma.10G140500.3	100	318	10	1859	4533	2695	9	0	3068

TRINITY_DN8943_c0_g1_i2	Glyma.10G140500.4	100	318	10	1859	4533	2695	9	0	3068

TRINITY_DN8943_c0_g1_i2	Glyma.10G140500.1	100	318	10	1859	4533	2695	9	0	3068

TRINITY_DN8943_c0_g1_i2	Glyma.20G089300.3	100	310	10	1859	4533	2695	9	0	3112

TRINITY_DN8943_c0_g1_i2	Glyma.20G089300.4	100	310	10	1859	4533	2695	9	0	3112

TRINITY_DN8943_c0_g1_i2	Glyma.20G089300.1	100	310	10	1859	4533	2695	9	0	3112

TRINITY_DN17316_c0_g1_i1	Glyma.13G220400.2	97.52475248	306	29	152	2818	2940	295	0	2817

TRINITY_DN17316_c0_g1_i1	Glyma.15G092000.2	100	315	19	152	2818	4281	1618	0	2894

TRINITY_DN14450_c1_g1_i1	Glyma.11G110800.5	95.53314121	167	17	330	2992	2678	1	0	3779

TRINITY_DN14450_c1_g1_i1	Glyma.11G110800.7	95.53314121	167	17	330	2992	2678	1	0	3779

TRINITY_DN14450_c1_g1_i1	Glyma.11G110800.3	95.53450609	167	17	330	2992	2678	1	0	3779

TRINITY_DN15235_c0_g1_i3	Glyma.16G172600.1	98.24561404	397	21	34	2710	448	3127	0	2619

TRINITY_DN9977_c0_g1_i2	Glyma.01G107000.1	100	291	32	1	2679	2631	1	0	2904

TRINITY_DN15795_c0_g1_i1	Glyma.02G071300.1	96.40591966	243	15	164	2844	2648	1	0	3291

TRINITY_DN15795_c0_g1_i3	Glyma.02G071300.1	95.66160521	243	15	338	3018	2648	1	0	3291

TRINITY_DN15984_c0_g1_i2	Glyma.12G021600.1	95.04950495	222	2	386	3074	2689	1	0	3725

TRINITY_DN9255_c0_g1_i1	Glyma.11G068600.3	97.2027972	193	2	772	3460	2697	9	0	3886

TRINITY_DN10085_c0_g1_i1	Glyma.05G013400.2	95.23809524	166	1	150	2836	1	2690	0	4028

TRINITY_DN13959_c0_g1_i3	Glyma.10G292300.1	100	166	29	207	2842	3029	347	0	3653

TRINITY_DN16332_c0_g3_i3	Glyma.14G085200.1	100	150	0	1	2690	2690	1	0	4137

TRINITY_DN16332_c0_g3_i3	Glyma.17G239500.2	100	143	0	1	2690	2690	1	0	4176

TRINITY_DN16332_c0_g3_i3	Glyma.17G239500.3	100	143	0	1	2690	2690	1	0	4176

TRINITY_DN16332_c0_g3_i3	Glyma.17G239500.4	100	143	0	1	2690	2690	1	0	4176

TRINITY_DN12523_c0_g3_i1	Glyma.20G106300.1	98.8372093	140	3	183	2868	3	2691	0	4159

TRINITY_DN16441_c4_g2_i5	Glyma.07G201200.1	100	459	21	847	3519	5016	2353	0	2158

TRINITY_DN16441_c4_g2_i5	Glyma.07G201200.2	95.78651685	459	21	847	3519	5013	2350	0	2158

TRINITY_DN8764_c0_g1_i1	Glyma.13G054400.1	98.8372093	389	34	305	2958	20	2691	0	2475

TRINITY_DN15399_c0_g1_i1	Glyma.01G144500.1	98.33333333	345	12	1	2674	5	2690	0	2939

TRINITY_DN15399_c0_g1_i4	Glyma.01G144500.1	99.53917051	295	12	1	2674	5	2690	0	3216

TRINITY_DN11268_c2_g1_i1	Glyma.13G254600.1	95.96774194	250	24	54	2724	1	2653	0	3232

TRINITY_DN15036_c0_g1_i2	Glyma.08G111000.2	95.40229885	175	4	280	2964	2730	43	0	3964

TRINITY_DN15036_c0_g1_i2	Glyma.08G111000.1	95.40229885	175	4	280	2964	2730	43	0	3964

TRINITY_DN3084_c0_g1_i1	Glyma.08G266100.2	98.02955665	174	4	328	3006	1	2688	0	3930

TRINITY_DN3084_c0_g2_i1	Glyma.08G266100.2	96	174	4	328	3006	1	2688	0	3930

TRINITY_DN16341_c0_g1_i1	Glyma.20G223300.1	96.78362573	194	4	665	3345	2682	1	0	3805

TRINITY_DN15305_c1_g1_i2	Glyma.12G074100.6	100	393	35	218	2885	2934	282	0	2442

TRINITY_DN15305_c1_g1_i2	Glyma.12G074100.5	96.7032967	393	35	218	2885	2934	282	0	2442

TRINITY_DN15305_c1_g1_i2	Glyma.12G074100.3	95.83333333	393	35	218	2885	2934	282	0	2442

TRINITY_DN15305_c1_g1_i2	Glyma.12G074100.1	97.43589744	393	35	218	2885	2934	282	0	2442

TRINITY_DN15305_c1_g1_i2	Glyma.12G074100.2	98.73417722	393	35	218	2885	2934	282	0	2442

TRINITY_DN15305_c1_g1_i2	Glyma.12G074100.4	98.73417722	393	35	218	2885	2934	282	0	2442

TRINITY_DN15399_c0_g1_i2	Glyma.01G144500.1	98.31932773	294	8	1	2677	5	2690	0	3262

TRINITY_DN12000_c0_g1_i1	Glyma.18G295800.5	99.53271028	277	24	167	2836	2661	1	0	3149

TRINITY_DN15399_c0_g1_i3	Glyma.01G144500.1	98.33333333	244	8	1	2677	5	2690	0	3539

TRINITY_DN9742_c0_g1_i1	Glyma.03G161300.1	100	171	15	288	2967	2994	336	0	3788

TRINITY_DN9742_c0_g1_i1	Glyma.03G161300.2	100	171	15	288	2967	2994	336	0	3788

TRINITY_DN9742_c0_g1_i1	Glyma.03G161300.3	100	171	15	288	2967	2844	186	0	3788

TRINITY_DN9742_c0_g1_i1	Glyma.03G161300.4	100	171	15	288	2967	2844	186	0	3788

TRINITY_DN16332_c0_g3_i3	Glyma.06G042400.1	100	292	1	1	2686	2684	2	0	3323

TRINITY_DN16332_c0_g3_i3	Glyma.04G041300.1	100	283	1	1	2686	2684	2	0	3373

TRINITY_DN14575_c0_g1_i1	Glyma.17G197200.5	100	213	10	677	3361	8292	5626	0	3651

TRINITY_DN16222_c0_g1_i2	Glyma.08G052900.3	97.94871795	229	2	155	2839	1	2676	0	3631

TRINITY_DN16222_c0_g1_i2	Glyma.08G052900.2	100	229	2	155	2839	1	2676	0	3631

TRINITY_DN16222_c0_g1_i2	Glyma.08G052900.1	96.75456389	229	2	155	2839	1	2676	0	3631

TRINITY_DN16222_c0_g1_i3	Glyma.08G052900.3	95.09954058	228	2	168	2852	1	2676	0	3637

TRINITY_DN16222_c0_g1_i3	Glyma.08G052900.2	98.1878089	228	2	168	2852	1	2676	0	3637

TRINITY_DN16222_c0_g1_i3	Glyma.08G052900.1	100	228	2	168	2852	1	2676	0	3637

TRINITY_DN16222_c0_g1_i1	Glyma.08G052900.3	100	215	3	155	2839	1	2676	0	3709

TRINITY_DN16222_c0_g1_i1	Glyma.08G052900.2	96.90721649	215	3	155	2839	1	2676	0	3709

TRINITY_DN16222_c0_g1_i1	Glyma.08G052900.1	98.93617021	215	3	155	2839	1	2676	0	3709

TRINITY_DN16222_c0_g1_i6	Glyma.08G052900.3	97.43589744	214	3	168	2852	1	2676	0	3714

TRINITY_DN16222_c0_g1_i6	Glyma.08G052900.2	96.5648855	214	3	168	2852	1	2676	0	3714

TRINITY_DN16222_c0_g1_i6	Glyma.08G052900.1	100	214	3	168	2852	1	2676	0	3714

TRINITY_DN16341_c0_g1_i2	Glyma.20G223300.1	100	214	7	344	3018	2682	1	0	3688

TRINITY_DN9872_c0_g1_i1	Glyma.04G253800.1	100	193	16	1766	4449	1709	4353	0	3624

TRINITY_DN13648_c0_g1_i1	Glyma.15G046000.2	99.51690821	141	2	1	2684	462	3145	0	4165

TRINITY_DN6075_c0_g1_i1	Glyma.08G266300.3	98.09160305	139	4	249	2924	2697	13	0	4130

TRINITY_DN15399_c0_g1_i1	Glyma.01G144500.4	98.31460674	341	18	1	2653	5	2685	0	2848

TRINITY_DN15399_c0_g1_i4	Glyma.01G144500.4	99.49494949	291	18	1	2653	5	2685	0	3125

TRINITY_DN15629_c1_g2_i1	Glyma.02G281100.1	96.61016949	220	1	47	2724	44	2727	0	3699

TRINITY_DN9872_c0_g1_i1	Glyma.06G108500.1	100	185	11	1766	4449	1709	4353	0	3679

TRINITY_DN9872_c0_g1_i1	Glyma.06G108500.3	96.62921348	185	11	1766	4449	1568	4212	0	3679

TRINITY_DN9742_c0_g1_i1	Glyma.19G162800.1	100	166	15	288	2967	3510	855	0	3831

TRINITY_DN9742_c0_g1_i1	Glyma.19G162800.2	100	166	15	288	2967	2991	336	0	3831

TRINITY_DN14450_c1_g1_i1	Glyma.12G037100.1	95.50561798	161	12	330	2992	2669	1	0	3832

TRINITY_DN14450_c1_g1_i1	Glyma.12G037100.5	95.53805774	161	12	330	2992	2669	1	0	3832

TRINITY_DN13648_c0_g1_i1	Glyma.08G187000.1	98.91304348	151	0	1	2684	462	3145	0	4120

TRINITY_DN8943_c0_g1_i2	Glyma.03G226200.3	100	149	2	1859	4541	2683	1	0	4119

TRINITY_DN8943_c0_g1_i2	Glyma.03G226200.2	100	149	2	1859	4541	2683	1	0	4119

TRINITY_DN8943_c0_g1_i2	Glyma.03G226200.1	100	149	2	1859	4541	2683	1	0	4119

TRINITY_DN8943_c0_g1_i2	Glyma.19G223200.1	100	143	2	1859	4541	2683	1	0	4152

TRINITY_DN16353_c0_g1_i1	Glyma.10G284500.1	95.83333333	317	22	471	3134	1006	3669	0	2953

TRINITY_DN16353_c0_g1_i8	Glyma.10G284500.1	99.36708861	317	22	389	3052	1006	3669	0	2953

TRINITY_DN16268_c1_g14_i1	Glyma.08G242100.3	100	283	0	265	2947	2916	234	0	3387

TRINITY_DN5451_c0_g1_i1	Glyma.12G021900.2	96.18320611	194	9	534	3206	2443	5109	0	3712

TRINITY_DN16268_c1_g9_i1	Glyma.08G242100.3	99.1202346	159	0	265	2947	2916	234	0	4074

TRINITY_DN15629_c1_g2_i1	Glyma.18G116800.1	96.38554217	433	18	54	2724	29	2700	0	2418

TRINITY_DN16031_c1_g2_i4	Glyma.15G137000.1	100	372	9	1	2661	2676	1	0	2717

TRINITY_DN16031_c1_g2_i4	Glyma.15G137000.2	95.33333333	370	15	1	2661	2667	1	0	2671

TRINITY_DN15399_c0_g1_i2	Glyma.01G144500.4	98.31932773	290	14	1	2656	5	2685	0	3171

TRINITY_DN16033_c0_g1_i3	Glyma.10G154400.2	96.63865546	270	8	390	3064	2681	4	0	3386

TRINITY_DN8285_c0_g1_i1	Glyma.02G107200.1	97.2972973	248	35	587	3180	32	2697	0	2907

TRINITY_DN15399_c0_g1_i3	Glyma.01G144500.4	98.33333333	240	14	1	2656	5	2685	0	3448

TRINITY_DN14044_c0_g1_i1	Glyma.07G220800.7	95.08716323	189	15	209	2884	257	2912	0	3699

TRINITY_DN14044_c0_g1_i1	Glyma.07G220800.1	97.14285714	189	15	209	2884	257	2912	0	3699

TRINITY_DN14044_c0_g1_i2	Glyma.07G220800.7	96.26998224	189	15	209	2884	257	2912	0	3699

TRINITY_DN14044_c0_g1_i2	Glyma.07G220800.1	99.47368421	189	15	209	2884	257	2912	0	3699

TRINITY_DN14044_c0_g1_i1	Glyma.07G220800.6	99.39393939	189	15	209	2884	221	2876	0	3699

TRINITY_DN14044_c0_g1_i2	Glyma.07G220800.6	98.57142857	189	15	209	2884	221	2876	0	3699

TRINITY_DN14044_c0_g1_i1	Glyma.07G220800.4	96.73913043	189	15	209	2884	71	2726	0	3699

TRINITY_DN14044_c0_g1_i1	Glyma.07G220800.5	98.57142857	189	15	209	2884	71	2726	0	3699

TRINITY_DN14044_c0_g1_i2	Glyma.07G220800.4	100	189	15	209	2884	71	2726	0	3699

TRINITY_DN14044_c0_g1_i2	Glyma.07G220800.5	100	189	15	209	2884	71	2726	0	3699

TRINITY_DN16431_c0_g1_i4	Glyma.03G034400.2	96.7032967	444	36	2681	5308	2667	1	0	2058

TRINITY_DN16431_c0_g1_i4	Glyma.03G034400.1	99.05660377	444	36	2681	5308	2667	1	0	2058

TRINITY_DN16431_c0_g1_i5	Glyma.03G034400.2	95.04950495	444	36	2661	5288	2667	1	0	2058

TRINITY_DN16431_c0_g1_i5	Glyma.03G034400.1	97.31343284	444	36	2661	5288	2667	1	0	2058

TRINITY_DN16431_c0_g1_i6	Glyma.03G034400.2	96.49122807	444	36	2324	4951	2667	1	0	2058

TRINITY_DN16431_c0_g1_i6	Glyma.03G034400.1	95.66563467	444	36	2324	4951	2667	1	0	2058

TRINITY_DN16431_c0_g1_i7	Glyma.03G034400.2	100	444	36	2304	4931	2667	1	0	2058

TRINITY_DN16431_c0_g1_i7	Glyma.03G034400.1	97.35849057	444	36	2304	4931	2667	1	0	2058

TRINITY_DN16033_c0_g1_i4	Glyma.10G154400.2	100	307	7	390	3064	2681	4	0	3192

TRINITY_DN11554_c0_g3_i1	Glyma.13G003700.1	100	254	8	149	2821	1	2676	0	3470

TRINITY_DN11554_c0_g3_i1	Glyma.20G065900.1	98.27586207	238	9	153	2821	8	2682	0	3528

TRINITY_DN15629_c1_g2_i1	Glyma.14G033500.1	98.34254144	208	3	48	2724	12	2688	0	3759

TRINITY_DN16071_c0_g2_i7	Glyma.08G087300.1	100	186	7	20	2691	308	2985	0	3855

TRINITY_DN12523_c0_g3_i1	Glyma.10G283400.1	98.8372093	157	3	193	2868	4	2682	0	4047

TRINITY_DN15235_c0_g1_i3	Glyma.10G227400.6	98.8372093	319	14	4	2673	412	3084	0	3083

TRINITY_DN15235_c0_g1_i3	Glyma.10G227400.4	100	319	14	4	2673	412	3084	0	3083

TRINITY_DN15235_c0_g1_i3	Glyma.10G227400.3	100	319	14	4	2673	412	3084	0	3083

TRINITY_DN15235_c0_g1_i3	Glyma.10G227400.1	100	319	14	4	2673	412	3084	0	3083

TRINITY_DN15235_c0_g1_i3	Glyma.10G227400.2	98.55072464	319	14	4	2673	412	3084	0	3083

TRINITY_DN15235_c0_g1_i3	Glyma.10G227400.5	97.2826087	319	14	4	2673	412	3084	0	3083

TRINITY_DN17316_c0_g1_i1	Glyma.13G220400.1	97.52475248	319	28	152	2818	4296	1669	0	2772

TRINITY_DN15795_c0_g1_i2	Glyma.16G152500.1	96.07843137	259	14	151	2820	2661	1	0	3338

TRINITY_DN15795_c0_g1_i4	Glyma.16G152500.1	97.95081967	259	14	325	2994	2661	1	0	3338

TRINITY_DN13324_c0_g2_i1	Glyma.12G236100.1	100	222	26	572	3221	2650	1	0	3343

TRINITY_DN13262_c0_g1_i1	Glyma.08G061700.2	98.79275654	221	19	1	2661	26	2680	0	3452

TRINITY_DN13262_c0_g1_i1	Glyma.08G061700.1	100	221	19	1	2661	26	2680	0	3452

TRINITY_DN13324_c0_g2_i1	Glyma.12G236100.3	100	221	27	572	3221	2644	1	0	3310

TRINITY_DN13324_c0_g2_i1	Glyma.12G236100.2	96.51162791	221	27	572	3221	2644	1	0	3310

TRINITY_DN13262_c0_g1_i1	Glyma.07G187500.1	95.52238806	219	6	1	2661	26	2704	0	3618

TRINITY_DN16425_c0_g1_i1	Glyma.U042000.1	98.03921569	202	12	494	3163	3150	484	0	3692

TRINITY_DN16341_c0_g1_i1	Glyma.10G165900.1	96.77419355	198	2	673	3345	2676	1	0	3792

TRINITY_DN16026_c0_g2_i3	Glyma.11G112700.4	98.26589595	188	5	895	3573	4	2658	0	3751

TRINITY_DN16026_c0_g2_i9	Glyma.11G112700.4	98.7654321	188	5	895	3573	4	2658	0	3751

TRINITY_DN16071_c0_g2_i7	Glyma.05G132900.1	100	182	7	20	2691	308	2985	0	3888

TRINITY_DN14450_c1_g1_i1	Glyma.11G110800.6	95.51282051	167	10	330	2992	2663	1	0	3816

TRINITY_DN14450_c1_g1_i1	Glyma.11G110800.4	95.51282051	167	10	330	2992	2663	1	0	3816

TRINITY_DN14450_c1_g1_i1	Glyma.11G110800.2	95.51282051	167	10	330	2992	2663	1	0	3816

TRINITY_DN16026_c0_g2_i5	Glyma.11G112700.4	100	158	5	883	3561	4	2658	0	3917

TRINITY_DN16026_c0_g2_i6	Glyma.11G112700.4	100	158	5	883	3561	4	2658	0	3917

TRINITY_DN3163_c0_g1_i2	Glyma.11G028700.1	97.36842105	131	6	353	3007	2667	1	0	3989

TRINITY_DN16431_c0_g1_i4	Glyma.03G043600.1	96.70658683	482	54	2681	5308	2643	1	0	1725

TRINITY_DN16431_c0_g1_i5	Glyma.03G043600.1	99.11504425	482	54	2661	5288	2643	1	0	1725

TRINITY_DN16431_c0_g1_i6	Glyma.03G043600.1	100	482	54	2324	4951	2643	1	0	1725

TRINITY_DN16431_c0_g1_i7	Glyma.03G043600.1	100	482	54	2304	4931	2643	1	0	1725

TRINITY_DN15235_c0_g1_i3	Glyma.16G172400.1	98.07321773	451	24	34	2697	517	3180	0	2266

TRINITY_DN15235_c0_g1_i3	Glyma.16G172400.4	96.95121951	451	24	34	2697	475	3138	0	2266

TRINITY_DN15235_c0_g1_i3	Glyma.16G172400.3	99.01639344	451	24	34	2697	448	3111	0	2266

TRINITY_DN16026_c0_g2_i1	Glyma.11G112700.4	97.10144928	310	27	895	3560	4	2651	0	2957

TRINITY_DN16026_c0_g2_i4	Glyma.11G112700.4	97.43589744	280	27	883	3548	4	2651	0	3123

TRINITY_DN16425_c0_g1_i3	Glyma.U042000.1	95.65217391	205	10	427	3096	3150	484	0	3687

TRINITY_DN13959_c0_g1_i3	Glyma.10G292300.2	98.20261438	167	21	207	2842	3017	347	0	3703

TRINITY_DN15345_c0_g3_i1	Glyma.12G194200.2	97.95918367	462	48	374	3020	27	2670	0	1977

TRINITY_DN15345_c0_g3_i1	Glyma.12G194200.1	97.18309859	462	48	374	3020	27	2670	0	1977

TRINITY_DN16158_c0_g1_i3	Glyma.10G286300.1	98.92473118	308	2	1157	3832	2847	172	0	3225

TRINITY_DN16425_c0_g1_i3	Glyma.12G060600.1	96.92307692	204	5	427	3096	3162	487	0	3762

TRINITY_DN16425_c0_g1_i3	Glyma.12G060600.2	97.79411765	204	5	427	3096	3162	487	0	3762

TRINITY_DN16425_c0_g1_i3	Glyma.12G060600.3	98.38709677	204	5	427	3096	3162	487	0	3762

TRINITY_DN16425_c0_g1_i1	Glyma.12G060600.1	98.68421053	200	5	494	3163	3162	487	0	3784

TRINITY_DN16425_c0_g1_i1	Glyma.12G060600.2	96.875	200	5	494	3163	3162	487	0	3784

TRINITY_DN16425_c0_g1_i1	Glyma.12G060600.3	96.87778769	200	5	494	3163	3162	487	0	3784

TRINITY_DN15531_c0_g1_i2	Glyma.14G001500.1	95.79831933	629	36	1240	3886	1138	3796	0	1160

TRINITY_DN15531_c0_g1_i2	Glyma.14G001500.2	96	629	36	1240	3886	1129	3787	0	1160

TRINITY_DN16262_c0_g1_i7	Glyma.04G213400.4	99.08256881	282	18	57	2729	469	3114	0	3166

TRINITY_DN16262_c0_g1_i7	Glyma.04G213400.3	99.08256881	280	20	57	2729	469	3111	0	3158

TRINITY_DN12265_c0_g2_i1	Glyma.19G065400.5	100	247	4	308	2983	118	2784	0	3515

TRINITY_DN12265_c0_g2_i1	Glyma.19G065400.2	100	247	4	308	2983	118	2784	0	3515

TRINITY_DN12265_c0_g2_i1	Glyma.19G065400.1	100	247	4	308	2983	118	2784	0	3515

TRINITY_DN12265_c0_g2_i1	Glyma.19G065400.3	100	247	4	308	2983	118	2784	0	3515

TRINITY_DN12265_c0_g2_i1	Glyma.19G065400.4	100	247	4	308	2983	118	2784	0	3515

TRINITY_DN16425_c0_g1_i1	Glyma.11G136700.1	97.14285714	217	3	494	3163	3144	469	0	3701

TRINITY_DN16425_c0_g1_i3	Glyma.11G136700.1	100	217	3	427	3096	3144	469	0	3701

TRINITY_DN16425_c0_g1_i1	Glyma.11G137100.3	95.81151832	208	10	494	3163	3144	484	0	3653

TRINITY_DN16425_c0_g1_i1	Glyma.11G137100.1	95.94095941	208	10	494	3163	3144	484	0	3653

TRINITY_DN16425_c0_g1_i1	Glyma.11G137100.2	95.94272076	208	10	494	3163	3144	484	0	3653

TRINITY_DN16425_c0_g1_i3	Glyma.11G137100.3	97.11934156	208	10	427	3096	3144	484	0	3653

TRINITY_DN16425_c0_g1_i3	Glyma.11G137100.1	98.55072464	208	10	427	3096	3144	484	0	3653

TRINITY_DN16425_c0_g1_i3	Glyma.11G137100.2	97.10144928	208	10	427	3096	3144	484	0	3653

TRINITY_DN8945_c0_g1_i1	Glyma.11G086000.1	97.01492537	200	14	440	3103	1	2655	0	3620

TRINITY_DN8945_c0_g1_i1	Glyma.11G086000.2	97.02970297	200	14	440	3103	1	2655	0	3620

TRINITY_DN8945_c0_g1_i1	Glyma.01G158600.1	100	193	5	440	3103	1	2667	0	3736

TRINITY_DN8945_c0_g1_i1	Glyma.01G158600.2	97.03703704	193	5	440	3103	1	2667	0	3736

TRINITY_DN16039_c0_g1_i5	Glyma.09G066000.3	100	157	3	1033	3708	1	2670	0	4034

TRINITY_DN14718_c0_g2_i2	Glyma.06G223500.2	96.12403101	150	0	2949	5624	2704	29	0	4111

TRINITY_DN14718_c0_g2_i3	Glyma.06G223500.2	96.09375	150	0	751	3426	2704	29	0	4111

TRINITY_DN8285_c0_g1_i1	Glyma.01G048000.1	98.9010989	262	51	587	3180	383	3024	0	2752

TRINITY_DN8285_c0_g1_i1	Glyma.01G048000.2	96.82539683	262	51	587	3180	32	2673	0	2752

TRINITY_DN11696_c0_g2_i1	Glyma.17G187400.1	97.77777778	256	15	115	2769	1	2670	0	3360

TRINITY_DN13573_c0_g1_i2	Glyma.01G123900.1	97.48953975	252	23	1013	3671	2635	1	0	3182

TRINITY_DN13152_c0_g1_i1	Glyma.02G222900.1	95.82366589	218	15	628	3291	2655	1	0	3531

TRINITY_DN16430_c0_g2_i4	Glyma.11G033000.2	100	301	20	1	2660	25	2675	0	3042

TRINITY_DN16430_c0_g2_i4	Glyma.11G033000.1	100	301	20	1	2660	25	2675	0	3042

TRINITY_DN1088_c0_g2_i1	Glyma.19G211500.1	95	273	15	365	3011	2676	6	0	3243

TRINITY_DN12617_c0_g1_i1	Glyma.09G063200.1	96.52605459	255	21	1872	4538	1210	3846	0	3253

TRINITY_DN12617_c0_g1_i1	Glyma.09G063200.3	96.52928416	255	21	1872	4538	811	3447	0	3253

TRINITY_DN12617_c0_g1_i1	Glyma.09G063200.6	96.52777778	251	27	1872	4538	1210	3822	0	3120

TRINITY_DN15795_c0_g1_i6	Glyma.16G152500.1	97.45762712	177	12	203	2860	2661	1	0	3781

TRINITY_DN14216_c0_g1_i2	Glyma.03G157200.1	100	382	5	304	2967	1	2667	0	2734

TRINITY_DN14216_c0_g1_i2	Glyma.03G157200.2	100	382	5	304	2967	1	2667	0	2734

TRINITY_DN11681_c0_g1_i1	Glyma.04G055700.2	97.14285714	310	12	316	2968	10	2677	0	3068

TRINITY_DN16245_c0_g3_i6	Glyma.06G196400.1	96	304	22	378	3032	2993	333	0	3070

TRINITY_DN16245_c0_g3_i6	Glyma.04G166100.1	99.57983193	301	20	375	3032	3500	840	0	3105

TRINITY_DN13573_c0_g1_i2	Glyma.03G051300.1	97.48283753	264	18	1013	3671	2653	1	0	3265

TRINITY_DN13377_c0_g1_i1	Glyma.13G212200.1	100	252	33	342	2947	5	2652	0	2957

TRINITY_DN13377_c0_g1_i2	Glyma.13G212200.1	100	252	33	381	2986	5	2652	0	2957

TRINITY_DN13377_c0_g1_i3	Glyma.13G212200.1	99.43820225	252	33	176	2781	5	2652	0	2957

TRINITY_DN9844_c0_g1_i1	Glyma.11G068300.2	99.43820225	189	4	491	3155	2668	1	0	3816

TRINITY_DN9844_c0_g1_i1	Glyma.11G068300.4	96.80365297	189	4	491	3155	2668	1	0	3816

TRINITY_DN9844_c0_g1_i2	Glyma.11G068300.2	100	189	4	480	3144	2668	1	0	3816

TRINITY_DN9844_c0_g1_i2	Glyma.11G068300.4	100	189	4	480	3144	2668	1	0	3816

TRINITY_DN9844_c0_g1_i3	Glyma.11G068300.2	97.77777778	189	4	375	3039	2668	1	0	3816

TRINITY_DN9844_c0_g1_i3	Glyma.11G068300.4	100	189	4	375	3039	2668	1	0	3816

TRINITY_DN9844_c0_g1_i4	Glyma.11G068300.2	95.23809524	189	4	483	3147	2668	1	0	3816

TRINITY_DN9844_c0_g1_i4	Glyma.11G068300.4	95.23809524	189	4	483	3147	2668	1	0	3816

TRINITY_DN9844_c0_g1_i5	Glyma.11G068300.2	100	189	4	481	3145	2668	1	0	3816

TRINITY_DN9844_c0_g1_i5	Glyma.11G068300.4	97.43589744	189	4	481	3145	2668	1	0	3816

TRINITY_DN9844_c0_g1_i6	Glyma.11G068300.2	99.52380952	189	4	479	3143	2668	1	0	3816

TRINITY_DN9844_c0_g1_i6	Glyma.11G068300.4	97.16981132	189	4	479	3143	2668	1	0	3816

TRINITY_DN15764_c0_g1_i5	Glyma.16G181000.1	98.47328244	174	8	34	2689	30	2697	0	3842

TRINITY_DN15795_c0_g1_i6	Glyma.02G071300.1	96.34146341	165	12	204	2860	2660	1	0	3845

TRINITY_DN14450_c1_g1_i1	Glyma.12G037100.2	95.52845528	162	8	330	2992	2654	1	0	3862

TRINITY_DN14450_c1_g1_i1	Glyma.12G037100.4	95.51282051	162	8	330	2992	2654	1	0	3862

TRINITY_DN14450_c1_g1_i1	Glyma.12G037100.3	95.50561798	162	9	330	2992	2651	1	0	3843

TRINITY_DN16245_c0_g5_i1	Glyma.16G099700.2	95.65217391	591	65	948	3565	2996	352	0	1147

TRINITY_DN16440_c0_g1_i2	Glyma.01G112200.2	98.26086957	377	28	694	3353	3576	938	0	2566

TRINITY_DN16440_c0_g1_i2	Glyma.01G112200.1	98.56630824	375	28	694	3353	3681	1043	0	2577

TRINITY_DN15964_c0_g1_i7	Glyma.13G065400.1	98.78787879	356	8	430	3096	1	2667	0	2909

TRINITY_DN13650_c0_g1_i1	Glyma.09G253100.3	96.46017699	293	14	214	2874	2730	112	0	2909

TRINITY_DN13650_c0_g1_i1	Glyma.09G253100.2	97.79411765	293	14	214	2874	2730	112	0	2909

TRINITY_DN13650_c0_g1_i1	Glyma.09G253100.1	97.75280899	293	14	214	2874	2730	112	0	2909

TRINITY_DN13650_c0_g1_i2	Glyma.09G253100.3	97.8021978	293	14	198	2858	2730	112	0	2909

TRINITY_DN13650_c0_g1_i2	Glyma.09G253100.2	97.20670391	293	14	198	2858	2730	112	0	2909

TRINITY_DN13650_c0_g1_i2	Glyma.09G253100.1	97.77777778	293	14	198	2858	2730	112	0	2909

TRINITY_DN15421_c1_g3_i1	Glyma.13G264600.1	97.70114943	250	19	584	3237	2793	134	0	3367

TRINITY_DN15421_c1_g3_i1	Glyma.13G264600.6	100	250	19	584	3237	2793	134	0	3367

TRINITY_DN15421_c1_g3_i1	Glyma.13G264600.8	95.71428571	250	19	584	3237	2793	134	0	3367

TRINITY_DN15421_c1_g3_i1	Glyma.13G264600.4	95.71428571	250	19	584	3237	2793	134	0	3367

TRINITY_DN16222_c0_g1_i2	Glyma.05G245100.1	95.95959596	213	5	155	2825	1	2659	0	3675

TRINITY_DN16222_c0_g1_i3	Glyma.05G245100.1	99.29078014	212	5	168	2838	1	2659	0	3681

TRINITY_DN16262_c0_g1_i3	Glyma.04G213400.4	95.10050251	209	14	57	2726	469	3114	0	3607

TRINITY_DN16262_c0_g1_i3	Glyma.04G213400.3	98.12792512	207	16	57	2726	469	3111	0	3598

TRINITY_DN16222_c0_g1_i1	Glyma.05G245100.1	95.61403509	196	6	155	2825	1	2659	0	3770

TRINITY_DN16222_c0_g1_i6	Glyma.05G245100.1	98.7654321	195	4	168	2838	1	2659	0	3775

TRINITY_DN11351_c0_g1_i1	Glyma.05G030900.1	100	424	37	58	2709	1	2631	0	2213

TRINITY_DN14843_c0_g1_i1	Glyma.13G084000.1	100	389	19	68	2722	1	2661	0	2621

TRINITY_DN12547_c0_g2_i1	Glyma.14G154600.2	98.4375	288	9	189	2840	357	3018	0	3168

TRINITY_DN16262_c0_g1_i7	Glyma.06G152800.2	98.94736842	276	20	64	2729	476	3102	0	3097

TRINITY_DN1088_c0_g2_i1	Glyma.19G211500.2	95	273	14	365	3011	2673	6	0	3256

TRINITY_DN13209_c1_g1_i2	Glyma.08G171800.2	98.26086957	261	15	695	3353	2659	1	0	3343

TRINITY_DN14283_c0_g1_i2	Glyma.18G143900.2	99.28825623	227	20	2031	4694	2832	211	0	3325

TRINITY_DN14283_c0_g1_i4	Glyma.18G143900.2	100	227	20	1880	4543	2832	211	0	3325

TRINITY_DN12547_c0_g2_i2	Glyma.14G154600.2	96.63608563	220	9	189	2840	357	3018	0	3544

TRINITY_DN15594_c0_g1_i2	Glyma.01G203100.1	96.77419355	150	2	558	3221	2670	1	0	4061

TRINITY_DN15594_c0_g1_i2	Glyma.01G203100.2	96.77419355	150	2	558	3221	2670	1	0	4061

TRINITY_DN15594_c0_g1_i5	Glyma.01G203100.1	95	143	10	558	3208	2670	1	0	4015

TRINITY_DN15594_c0_g1_i5	Glyma.01G203100.2	98.7654321	143	10	558	3208	2670	1	0	4015

TRINITY_DN3163_c0_g1_i2	Glyma.11G028700.3	100	131	5	353	3007	2658	1	0	4030

TRINITY_DN13377_c0_g1_i1	Glyma.07G253800.13	96.93877551	427	66	371	2942	1	2635	0	1716

TRINITY_DN13377_c0_g1_i1	Glyma.07G253800.2	98.55072464	427	66	371	2942	1	2635	0	1716

TRINITY_DN13377_c0_g1_i2	Glyma.07G253800.13	97.36842105	427	66	410	2981	1	2635	0	1716

TRINITY_DN13377_c0_g1_i2	Glyma.07G253800.2	97.36842105	427	66	410	2981	1	2635	0	1716

TRINITY_DN13377_c0_g1_i3	Glyma.07G253800.13	100	427	66	205	2776	1	2635	0	1716

TRINITY_DN13377_c0_g1_i3	Glyma.07G253800.2	100	427	66	205	2776	1	2635	0	1716

TRINITY_DN15964_c0_g1_i8	Glyma.13G065400.1	95.12195122	360	4	430	3096	1	2667	0	2909

TRINITY_DN15764_c0_g1_i1	Glyma.09G135100.1	97.62711864	337	10	513	3165	30	2694	0	2933

TRINITY_DN16245_c0_g3_i6	Glyma.06G196400.3	98.69791667	304	20	378	3032	2990	333	0	3083

TRINITY_DN15314_c0_g2_i1	Glyma.12G094500.1	100	254	22	118	2742	2874	226	0	3109

TRINITY_DN13377_c0_g1_i1	Glyma.15G100500.1	97.91144528	254	37	342	2947	5	2658	0	3018

TRINITY_DN13377_c0_g1_i1	Glyma.15G100500.2	97.91183295	254	37	342	2947	5	2658	0	3018

TRINITY_DN13377_c0_g1_i2	Glyma.15G100500.1	98.80952381	254	37	381	2986	5	2658	0	3018

TRINITY_DN13377_c0_g1_i2	Glyma.15G100500.2	98.80952381	254	37	381	2986	5	2658	0	3018

TRINITY_DN13377_c0_g1_i3	Glyma.15G100500.1	99.34210526	254	37	176	2781	5	2658	0	3018

TRINITY_DN13377_c0_g1_i3	Glyma.15G100500.2	97.95918367	254	37	176	2781	5	2658	0	3018

TRINITY_DN14283_c0_g1_i2	Glyma.18G143900.1	99.28057554	227	20	2032	4694	2831	211	0	3323

TRINITY_DN14283_c0_g1_i3	Glyma.18G143900.2	99.28315412	227	20	512	3174	2831	211	0	3323

TRINITY_DN14283_c0_g1_i4	Glyma.18G143900.1	100	227	20	1881	4543	2831	211	0	3323

TRINITY_DN15314_c0_g2_i2	Glyma.12G094500.1	95.07389163	223	21	697	3314	2874	226	0	3234

TRINITY_DN15142_c0_g1_i1	Glyma.13G355400.1	99.45054945	215	10	209	2864	2662	1	0	3609

TRINITY_DN16245_c0_g5_i2	Glyma.16G099700.2	95.13991163	586	62	948	3565	2996	352	0	1208

TRINITY_DN15964_c0_g1_i5	Glyma.13G065400.1	96.16580311	496	6	430	3094	1	2665	0	2141

TRINITY_DN15964_c0_g1_i6	Glyma.13G065400.1	100	496	6	430	3094	1	2665	0	2141

TRINITY_DN13209_c1_g1_i2	Glyma.15G255200.1	99.01960784	255	15	695	3353	2659	1	0	3398

TRINITY_DN15421_c1_g3_i1	Glyma.13G264600.2	96.91119691	250	18	584	3237	2790	134	0	3380

TRINITY_DN15421_c1_g3_i1	Glyma.13G264600.3	95.95375723	250	18	584	3237	2790	134	0	3380

TRINITY_DN15421_c1_g3_i1	Glyma.13G264600.5	100	250	18	584	3237	2790	134	0	3380

TRINITY_DN15421_c1_g3_i1	Glyma.13G264600.7	95.83333333	250	18	584	3237	2790	134	0	3380

TRINITY_DN14283_c0_g1_i2	Glyma.08G282400.8	99.29078014	223	18	2030	4688	2845	202	0	3478

TRINITY_DN14283_c0_g1_i4	Glyma.08G282400.8	100	223	18	1879	4537	2845	202	0	3478

TRINITY_DN14283_c0_g1_i5	Glyma.08G282400.8	100	219	19	1879	4513	2845	202	0	3345

TRINITY_DN14283_c0_g1_i6	Glyma.08G282400.8	98.4375	219	19	2030	4664	2845	202	0	3345

TRINITY_DN14283_c0_g1_i2	Glyma.08G282400.7	99.29078014	219	19	2030	4688	2821	202	0	3345

TRINITY_DN14283_c0_g1_i4	Glyma.08G282400.7	100	219	19	1879	4537	2821	202	0	3345

TRINITY_DN9123_c0_g1_i1	Glyma.03G140700.1	95.3125	204	10	477	3131	2667	1	0	3707

TRINITY_DN16802_c0_g1_i1	Glyma.10G168600.1	98.91304348	194	3	361	3027	2664	1	0	3819

TRINITY_DN16802_c0_g1_i1	Glyma.20G221100.1	98.5472155	185	3	361	3027	2664	1	0	3869

TRINITY_DN16802_c0_g1_i1	Glyma.20G221100.2	96.84210526	185	3	361	3027	2664	1	0	3869

TRINITY_DN8764_c0_g1_i1	Glyma.13G053600.2	98.8372093	389	32	323	2958	35	2685	0	2466

TRINITY_DN8764_c0_g1_i1	Glyma.13G053600.1	98.8372093	389	32	323	2958	35	2685	0	2466

TRINITY_DN14216_c0_g1_i1	Glyma.10G029500.1	100	340	10	153	2807	1	2664	0	2944

TRINITY_DN14216_c0_g1_i1	Glyma.02G144600.1	100	338	12	153	2807	1	2661	0	2937

TRINITY_DN14216_c0_g1_i1	Glyma.02G144600.2	100	338	12	153	2807	1	2661	0	2937

TRINITY_DN1088_c0_g2_i1	Glyma.03G214900.1	100	275	14	365	3010	2667	7	0	3227

TRINITY_DN16019_c0_g1_i9	Glyma.06G013100.1	95.77464789	268	12	331	2994	2649	1	0	3304

TRINITY_DN16019_c0_g1_i6	Glyma.06G013100.1	100	261	12	331	2994	2649	1	0	3343

TRINITY_DN14216_c0_g1_i1	Glyma.03G157200.1	100	218	3	153	2807	1	2667	0	3640

TRINITY_DN14216_c0_g1_i1	Glyma.03G157200.2	100	218	3	153	2807	1	2667	0	3640

TRINITY_DN15162_c0_g1_i1	Glyma.10G201900.2	96.91780822	204	8	85	2747	1	2654	0	3685

TRINITY_DN16262_c0_g1_i3	Glyma.06G152800.2	100	197	19	64	2726	476	3102	0	3550

TRINITY_DN16274_c0_g1_i1	Glyma.12G095300.5	95.63636364	163	20	503	3157	1	2646	0	3808

TRINITY_DN16274_c0_g1_i2	Glyma.12G095300.5	96.26998224	163	20	503	3157	1	2646	0	3808

TRINITY_DN16303_c0_g1_i3	Glyma.12G201100.3	100	156	5	152	2818	1	2640	0	3886

TRINITY_DN16303_c0_g1_i5	Glyma.12G201100.3	100	156	5	152	2818	1	2640	0	3886

TRINITY_DN3163_c0_g1_i2	Glyma.01G213200.1	100	141	6	353	3007	2655	1	0	3989

TRINITY_DN16431_c0_g1_i4	Glyma.03G037000.1	100	435	38	2687	5308	2646	1	0	2100

TRINITY_DN16431_c0_g1_i5	Glyma.03G037000.1	95.50561798	435	38	2667	5288	2646	1	0	2100

TRINITY_DN16431_c0_g1_i6	Glyma.03G037000.1	95.78488372	435	38	2330	4951	2646	1	0	2100

TRINITY_DN16431_c0_g1_i7	Glyma.03G037000.1	96.05263158	435	38	2310	4931	2646	1	0	2100

TRINITY_DN15795_c0_g1_i2	Glyma.02G071300.1	96.07843137	244	14	164	2820	2648	1	0	3397

TRINITY_DN15795_c0_g1_i4	Glyma.02G071300.1	97.95081967	244	14	338	2994	2648	1	0	3397

TRINITY_DN15328_c0_g1_i2	Glyma.16G025800.1	98.80952381	225	9	3	2655	1359	4008	0	3489

TRINITY_DN15328_c0_g1_i2	Glyma.16G025800.2	98.79518072	225	10	3	2655	1359	4005	0	3470

TRINITY_DN14283_c0_g1_i2	Glyma.08G282400.5	99.28057554	223	18	2032	4688	2843	202	0	3474

TRINITY_DN14283_c0_g1_i2	Glyma.08G282400.1	99.29577465	223	18	2032	4688	2843	202	0	3474

TRINITY_DN14283_c0_g1_i3	Glyma.08G282400.8	99.29078014	223	18	512	3168	2843	202	0	3474

TRINITY_DN14283_c0_g1_i4	Glyma.08G282400.5	100	223	18	1881	4537	2843	202	0	3474

TRINITY_DN14283_c0_g1_i4	Glyma.08G282400.1	100	223	18	1881	4537	2843	202	0	3474

TRINITY_DN14283_c0_g1_i1	Glyma.08G282400.8	99.27536232	219	19	512	3144	2843	202	0	3341

TRINITY_DN14283_c0_g1_i5	Glyma.08G282400.5	100	219	19	1881	4513	2843	202	0	3341

TRINITY_DN14283_c0_g1_i5	Glyma.08G282400.1	100	219	19	1881	4513	2843	202	0	3341

TRINITY_DN14283_c0_g1_i6	Glyma.08G282400.5	98.4375	219	19	2032	4664	2843	202	0	3341

TRINITY_DN14283_c0_g1_i6	Glyma.08G282400.1	96.23059867	219	19	2032	4664	2843	202	0	3341

TRINITY_DN14283_c0_g1_i2	Glyma.08G282400.3	99.28825623	219	19	2032	4688	2819	202	0	3341

TRINITY_DN14283_c0_g1_i2	Glyma.08G282400.11	99.28571429	219	19	2032	4688	2819	202	0	3341

TRINITY_DN14283_c0_g1_i3	Glyma.08G282400.7	99.29078014	219	19	512	3168	2819	202	0	3341

TRINITY_DN14283_c0_g1_i4	Glyma.08G282400.3	100	219	19	1881	4537	2819	202	0	3341

TRINITY_DN14283_c0_g1_i4	Glyma.08G282400.11	100	219	19	1881	4537	2819	202	0	3341

TRINITY_DN16431_c0_g1_i4	Glyma.03G038800.1	98.57651246	450	39	2693	5308	2652	1	0	2028

TRINITY_DN16431_c0_g1_i5	Glyma.03G038800.1	95	450	39	2673	5288	2652	1	0	2028

TRINITY_DN16431_c0_g1_i6	Glyma.03G038800.1	95.37572254	450	39	2336	4951	2652	1	0	2028

TRINITY_DN16431_c0_g1_i7	Glyma.03G038800.1	97.95918367	450	39	2316	4931	2652	1	0	2028

TRINITY_DN14216_c0_g1_i2	Glyma.19G159400.10	100	374	4	304	2967	1	2661	0	2817

TRINITY_DN14216_c0_g1_i2	Glyma.19G159400.5	100	374	4	304	2967	1	2661	0	2817

TRINITY_DN14216_c0_g1_i2	Glyma.19G159400.6	100	374	4	304	2967	1	2661	0	2817

TRINITY_DN14216_c0_g1_i2	Glyma.19G159400.2	100	374	4	304	2967	1	2661	0	2817

TRINITY_DN15594_c0_g1_i2	Glyma.17G151100.1	98.64253394	302	7	562	3221	2657	1	0	3164

TRINITY_DN16430_c0_g2_i4	Glyma.11G032900.3	98.97959184	294	14	1	2660	25	2669	0	3131

TRINITY_DN16430_c0_g2_i4	Glyma.11G032900.1	97.6119403	294	14	1	2660	25	2669	0	3131

TRINITY_DN16430_c0_g2_i4	Glyma.11G032900.2	95.74468085	294	14	1	2660	25	2669	0	3131

TRINITY_DN16019_c0_g1_i9	Glyma.04G013200.1	95.77464789	275	3	331	2994	2661	1	0	3365

TRINITY_DN16019_c0_g1_i6	Glyma.04G013200.1	96.61691542	268	3	331	2994	2661	1	0	3404

TRINITY_DN14283_c0_g1_i2	Glyma.08G282400.9	99.28057554	223	17	2030	4688	2842	202	0	3493

TRINITY_DN14283_c0_g1_i4	Glyma.08G282400.9	99.28057554	223	17	1879	4537	2842	202	0	3493

TRINITY_DN13152_c0_g1_i1	Glyma.14G189900.1	95.83333333	221	13	628	3291	2637	1	0	3509

TRINITY_DN14283_c0_g1_i5	Glyma.08G282400.9	100	219	18	1879	4513	2842	202	0	3360

TRINITY_DN14283_c0_g1_i6	Glyma.08G282400.9	96.22641509	219	18	2030	4664	2842	202	0	3360

TRINITY_DN14283_c0_g1_i2	Glyma.08G282400.6	99.29577465	219	18	2030	4688	2818	202	0	3360

TRINITY_DN14283_c0_g1_i4	Glyma.08G282400.6	100	219	18	1879	4537	2818	202	0	3360

TRINITY_DN16213_c0_g2_i1	Glyma.12G212500.1	95.38461538	426	24	204	2845	120	2773	0	2353

TRINITY_DN16430_c0_g2_i4	Glyma.01G209200.1	100	316	7	1	2660	25	2678	0	3079

TRINITY_DN15594_c0_g1_i2	Glyma.05G068600.1	98.64253394	301	6	562	3221	2654	1	0	3162

TRINITY_DN1088_c0_g2_i1	Glyma.03G214900.2	99.31506849	275	13	365	3010	2664	7	0	3241

TRINITY_DN11898_c0_g2_i3	Glyma.06G093500.1	98.96907216	259	13	859	3503	2651	1	0	3278

TRINITY_DN11898_c0_g2_i1	Glyma.06G093500.1	98.74213836	228	11	889	3536	2651	1	0	3469

TRINITY_DN14216_c0_g1_i2	Glyma.10G029500.1	100	190	0	304	2967	1	2664	0	3868

TRINITY_DN15458_c0_g1_i2	Glyma.15G121500.3	95.4887218	189	0	796	3459	1	2664	0	3873

TRINITY_DN14216_c0_g1_i2	Glyma.02G144600.1	100	183	1	304	2967	1	2661	0	3886

TRINITY_DN14216_c0_g1_i2	Glyma.02G144600.2	100	183	1	304	2967	1	2661	0	3886

TRINITY_DN145_c0_g1_i1	Glyma.03G225800.1	100	178	2	538	3183	1	2664	0	3818

TRINITY_DN145_c0_g1_i1	Glyma.19G222700.1	100	163	2	538	3183	1	2664	0	3901

TRINITY_DN16019_c0_g1_i6	Glyma.11G118600.1	98.56115108	158	0	331	2994	2664	1	0	4045

TRINITY_DN16019_c0_g1_i9	Glyma.11G118600.1	100	155	0	331	2994	2664	1	0	4061

TRINITY_DN16019_c0_g1_i6	Glyma.12G044000.1	99.36708861	146	0	331	2994	2664	1	0	4111

TRINITY_DN16019_c0_g1_i9	Glyma.12G044000.1	95.0617284	143	0	331	2994	2664	1	0	4128

TRINITY_DN16430_c0_g2_i4	Glyma.01G209300.1	100	301	12	1	2660	25	2654	0	3018

TRINITY_DN11696_c0_g2_i1	Glyma.05G089300.1	98.48484848	264	15	115	2769	1	2634	0	3216

TRINITY_DN14283_c0_g1_i2	Glyma.08G282400.4	99.29078014	223	17	2032	4688	2840	202	0	3489

TRINITY_DN14283_c0_g1_i2	Glyma.08G282400.12	99.29522318	223	17	2032	4688	2840	202	0	3489

TRINITY_DN14283_c0_g1_i3	Glyma.08G282400.9	99.27536232	223	17	512	3168	2840	202	0	3489

TRINITY_DN14283_c0_g1_i4	Glyma.08G282400.4	98.4375	223	17	1881	4537	2840	202	0	3489

TRINITY_DN14283_c0_g1_i4	Glyma.08G282400.12	100	223	17	1881	4537	2840	202	0	3489

TRINITY_DN14283_c0_g1_i1	Glyma.08G282400.9	99.28571429	219	18	512	3144	2840	202	0	3356

TRINITY_DN14283_c0_g1_i5	Glyma.08G282400.4	100	219	18	1881	4513	2840	202	0	3356

TRINITY_DN14283_c0_g1_i5	Glyma.08G282400.12	100	219	18	1881	4513	2840	202	0	3356

TRINITY_DN14283_c0_g1_i6	Glyma.08G282400.4	96.21621622	219	18	2032	4664	2840	202	0	3356

TRINITY_DN14283_c0_g1_i6	Glyma.08G282400.12	96.22641509	219	18	2032	4664	2840	202	0	3356

TRINITY_DN14283_c0_g1_i2	Glyma.08G282400.2	99.28571429	219	18	2032	4688	2816	202	0	3356

TRINITY_DN14283_c0_g1_i2	Glyma.08G282400.10	99.27536232	219	18	2032	4688	2816	202	0	3356

TRINITY_DN14283_c0_g1_i3	Glyma.08G282400.6	99.28571429	219	18	512	3168	2816	202	0	3356

TRINITY_DN14283_c0_g1_i4	Glyma.08G282400.2	100	219	18	1881	4537	2816	202	0	3356

TRINITY_DN14283_c0_g1_i4	Glyma.08G282400.10	100	219	18	1881	4537	2816	202	0	3356

TRINITY_DN14044_c0_g1_i1	Glyma.07G220800.8	95.41284404	186	13	228	2884	1	2639	0	3694

TRINITY_DN14044_c0_g1_i1	Glyma.07G220800.2	95.41284404	186	13	228	2884	1	2639	0	3694

TRINITY_DN14044_c0_g1_i1	Glyma.07G220800.3	97.26027397	186	13	228	2884	1	2639	0	3694

TRINITY_DN14044_c0_g1_i2	Glyma.07G220800.8	97.98657718	186	13	228	2884	1	2639	0	3694

TRINITY_DN14044_c0_g1_i2	Glyma.07G220800.2	97.98657718	186	13	228	2884	1	2639	0	3694

TRINITY_DN14044_c0_g1_i2	Glyma.07G220800.3	96.71052632	186	13	228	2884	1	2639	0	3694

TRINITY_DN14044_c0_g1_i1	Glyma.08G229600.1	97.73462783	181	8	228	2884	1	2645	0	3760

TRINITY_DN14044_c0_g1_i2	Glyma.08G229600.1	97.91666667	181	8	228	2884	1	2645	0	3760

TRINITY_DN13377_c0_g1_i1	Glyma.07G253800.1	98.9010989	425	66	371	2935	1	2628	0	1714

TRINITY_DN13377_c0_g1_i2	Glyma.07G253800.1	97.36842105	425	66	410	2974	1	2628	0	1714

TRINITY_DN13377_c0_g1_i3	Glyma.07G253800.1	100	425	66	205	2769	1	2628	0	1714

TRINITY_DN15016_c0_g1_i2	Glyma.16G182400.2	98.41628959	422	40	661	3290	22	2649	0	2152

TRINITY_DN16397_c0_g1_i2	Glyma.19G065600.3	100	282	4	3933	6592	2686	27	0	3328

TRINITY_DN15122_c0_g2_i2	Glyma.11G221800.2	96.72131148	212	0	1198	3859	3787	1126	0	3742

TRINITY_DN15122_c0_g2_i2	Glyma.11G221800.1	99.71098266	212	0	1198	3859	3787	1126	0	3742

TRINITY_DN6058_c0_g1_i1	Glyma.10G022700.1	97.32142857	155	5	387	3047	2646	1	0	3947

TRINITY_DN15639_c1_g1_i1	Glyma.13G329600.1	97.84946237	480	43	996	3616	2738	103	0	1834

TRINITY_DN15639_c1_g1_i2	Glyma.13G329600.1	97.41935484	480	43	1120	3740	2738	103	0	1834

TRINITY_DN15639_c1_g1_i3	Glyma.13G329600.1	100	480	43	803	3423	2738	103	0	1834

TRINITY_DN14779_c0_g1_i1	Glyma.04G034700.1	95.38188277	355	19	205	2855	2642	1	0	2760

TRINITY_DN14779_c0_g1_i1	Glyma.04G034700.2	95.43568465	355	19	205	2855	2642	1	0	2760

TRINITY_DN14216_c0_g1_i1	Glyma.19G159400.10	100	229	1	153	2807	1	2661	0	3607

TRINITY_DN14216_c0_g1_i1	Glyma.19G159400.5	100	229	1	153	2807	1	2661	0	3607

TRINITY_DN14216_c0_g1_i1	Glyma.19G159400.6	100	229	1	153	2807	1	2661	0	3607

TRINITY_DN14216_c0_g1_i1	Glyma.19G159400.2	100	229	1	153	2807	1	2661	0	3607

TRINITY_DN16031_c1_g2_i4	Glyma.17G037600.1	100	194	0	1	2661	2661	1	0	3840

TRINITY_DN16031_c1_g2_i4	Glyma.07G235800.1	97.5	183	0	1	2661	2661	1	0	3901

TRINITY_DN13152_c0_g1_i4	Glyma.02G222900.1	95.80838323	178	5	628	3279	2655	1	0	3831

TRINITY_DN9657_c0_g1_i1	Glyma.08G051000.1	96.72131148	178	58	206	2754	1	2636	0	3042

TRINITY_DN6058_c0_g1_i1	Glyma.02G151400.1	97.33333333	157	2	387	3047	2646	1	0	3947

TRINITY_DN14779_c0_g1_i4	Glyma.04G034700.1	99.04761905	330	14	100	2754	2643	1	0	2942

TRINITY_DN14779_c0_g1_i4	Glyma.04G034700.2	98.27586207	330	14	100	2754	2643	1	0	2942

TRINITY_DN9123_c0_g1_i1	Glyma.19G143300.1	96.15384615	203	6	477	3131	2658	1	0	3742

TRINITY_DN9030_c0_g1_i2	Glyma.11G028300.1	97.30848861	183	6	198	2848	1	2654	0	3801

TRINITY_DN6074_c0_g1_i1	Glyma.19G140100.1	95.66473988	179	0	465	3124	2660	1	0	3921

TRINITY_DN6074_c0_g1_i1	Glyma.19G140100.2	95.66197183	179	0	465	3124	2660	1	0	3921

TRINITY_DN6074_c0_g1_i2	Glyma.19G140100.1	95.65217391	179	0	465	3124	2660	1	0	3921

TRINITY_DN6074_c0_g1_i2	Glyma.19G140100.2	95.65217391	179	0	465	3124	2660	1	0	3921

TRINITY_DN16431_c0_g1_i4	Glyma.03G047000.1	100	506	47	2681	5293	2643	13	0	1629

TRINITY_DN16431_c0_g1_i5	Glyma.03G047000.1	95.78947368	506	47	2661	5273	2643	13	0	1629

TRINITY_DN16431_c0_g1_i6	Glyma.03G047000.1	96.52509653	506	47	2324	4936	2643	13	0	1629

TRINITY_DN16431_c0_g1_i7	Glyma.03G047000.1	97.72727273	506	47	2304	4916	2643	13	0	1629

TRINITY_DN15757_c0_g1_i2	Glyma.18G003000.2	98.14126394	325	9	128	2778	76	2729	0	3025

TRINITY_DN15594_c0_g1_i5	Glyma.17G151100.1	97.79411765	300	9	562	3208	2657	1	0	3158

TRINITY_DN15757_c0_g1_i3	Glyma.18G003000.2	100	299	8	128	2778	76	2729	0	3169

TRINITY_DN15757_c0_g1_i5	Glyma.18G003000.2	100	286	9	128	2778	76	2729	0	3241

TRINITY_DN14670_c0_g1_i3	Glyma.19G019100.3	99.53488372	266	18	765	3416	1	2619	0	3133

TRINITY_DN12872_c0_g2_i1	Glyma.14G214000.2	95.0617284	217	7	173	2818	91	2748	0	3618

TRINITY_DN11351_c0_g2_i1	Glyma.04G175400.2	98.52941176	393	19	67	2709	1	2649	0	2577

TRINITY_DN11351_c0_g2_i1	Glyma.06G189500.1	100	385	19	67	2709	1	2649	0	2621

TRINITY_DN15594_c0_g1_i5	Glyma.05G068600.1	97.97979798	299	8	562	3208	2654	1	0	3155

TRINITY_DN6074_c0_g1_i1	Glyma.03G137300.1	95.66473988	185	3	471	3124	2657	1	0	3851

TRINITY_DN6074_c0_g1_i2	Glyma.03G137300.1	95.65217391	185	3	471	3124	2657	1	0	3851

TRINITY_DN16425_c0_g1_i3	Glyma.15G035900.1	97.26027397	362	17	465	3096	3127	484	0	2656

TRINITY_DN16425_c0_g1_i1	Glyma.15G035900.1	96.99398798	356	17	532	3163	3127	484	0	2689

TRINITY_DN15242_c0_g1_i1	Glyma.20G086700.5	99.00990099	287	27	221	2851	2631	1	0	2981

TRINITY_DN16130_c2_g6_i9	Glyma.10G075300.2	98.41269841	240	3	393	3047	136	2790	0	3552

TRINITY_DN15328_c0_g1_i4	Glyma.16G025800.1	100	224	9	3	2646	1359	3999	0	3478

TRINITY_DN15328_c0_g1_i4	Glyma.16G025800.2	100	224	10	3	2646	1359	3996	0	3459

TRINITY_DN16384_c0_g1_i1	Glyma.18G212400.3	100	213	7	185	2839	1	2640	0	3603

TRINITY_DN16384_c0_g1_i1	Glyma.18G212400.1	100	213	7	185	2839	1	2640	0	3603

TRINITY_DN16384_c0_g1_i3	Glyma.18G212400.3	100	211	6	183	2837	1	2640	0	3615

TRINITY_DN16384_c0_g1_i3	Glyma.18G212400.1	100	211	6	183	2837	1	2640	0	3615

TRINITY_DN9030_c0_g1_i2	Glyma.01G213600.1	97.31051345	185	6	198	2845	1	2651	0	3784

TRINITY_DN9030_c0_g1_i2	Glyma.01G213600.2	97.30250482	185	6	198	2845	1	2651	0	3784

TRINITY_DN16130_c2_g6_i16	Glyma.10G075300.2	96.54218534	182	3	393	3047	136	2790	0	3873

TRINITY_DN9874_c0_g1_i1	Glyma.19G212900.1	96.46464646	143	13	435	3068	2649	1	0	3914

TRINITY_DN15353_c0_g1_i2	Glyma.18G233500.1	98.76923077	250	4	292	2946	2652	1	0	3487

TRINITY_DN15353_c0_g1_i2	Glyma.09G259100.1	98.37962963	244	4	292	2946	2652	1	0	3520

TRINITY_DN16431_c0_g1_i4	Glyma.03G045700.1	96.03174603	435	23	2684	5308	2644	1	0	2228

TRINITY_DN16431_c0_g1_i5	Glyma.03G045700.1	95.64220183	435	23	2664	5288	2644	1	0	2228

TRINITY_DN16431_c0_g1_i6	Glyma.03G045700.1	99.2	435	23	2327	4951	2644	1	0	2228

TRINITY_DN16431_c0_g1_i7	Glyma.03G045700.1	97.68786127	435	23	2307	4931	2644	1	0	2228

TRINITY_DN15789_c0_g1_i1	Glyma.14G075800.1	97.5	309	12	1996	4646	3011	5649	0	3062

TRINITY_DN11351_c0_g1_i1	Glyma.06G189500.1	95.34883721	241	5	58	2709	1	2649	0	3509

TRINITY_DN16130_c2_g6_i9	Glyma.02G201300.2	99.12280702	228	0	393	3047	133	2787	0	3640

TRINITY_DN16130_c2_g6_i9	Glyma.02G201300.4	99.12280702	228	0	393	3047	133	2787	0	3640

TRINITY_DN16130_c2_g6_i9	Glyma.02G201300.1	99.12280702	228	0	393	3047	133	2787	0	3640

TRINITY_DN11351_c0_g1_i1	Glyma.04G175400.2	100	223	5	58	2709	1	2649	0	3609

TRINITY_DN16384_c0_g1_i4	Glyma.18G212400.3	97.72727273	184	6	185	2833	1	2640	0	3747

TRINITY_DN16384_c0_g1_i4	Glyma.18G212400.1	97.72727273	184	6	185	2833	1	2640	0	3747

TRINITY_DN16384_c0_g1_i2	Glyma.18G212400.3	100	182	6	183	2831	1	2640	0	3759

TRINITY_DN16384_c0_g1_i2	Glyma.18G212400.1	100	182	6	183	2831	1	2640	0	3759

TRINITY_DN16130_c2_g6_i16	Glyma.02G201300.2	99.00990099	170	0	393	3047	133	2787	0	3962

TRINITY_DN16130_c2_g6_i16	Glyma.02G201300.4	100	170	0	393	3047	133	2787	0	3962

TRINITY_DN16130_c2_g6_i16	Glyma.02G201300.1	99.05437352	170	0	393	3047	133	2787	0	3962

TRINITY_DN12988_c0_g2_i1	Glyma.19G168600.1	98.5915493	160	4	1	2645	399	3052	0	3945

TRINITY_DN2580_c0_g1_i1	Glyma.19G022800.2	99.59349593	158	2	200	2852	3600	946	0	4015

TRINITY_DN9874_c0_g1_i1	Glyma.03G216400.1	96.46464646	148	9	435	3068	2649	1	0	3908

TRINITY_DN16431_c0_g1_i4	Glyma.03G034900.1	100	438	37	2706	5308	2639	1	0	2049

TRINITY_DN16431_c0_g1_i5	Glyma.03G034900.1	100	438	37	2686	5288	2639	1	0	2049

TRINITY_DN16431_c0_g1_i6	Glyma.03G034900.1	100	438	37	2349	4951	2639	1	0	2049

TRINITY_DN16431_c0_g1_i7	Glyma.03G034900.1	97.95918367	438	37	2329	4931	2639	1	0	2049

TRINITY_DN11351_c0_g2_i1	Glyma.05G030900.1	99.23664122	225	16	67	2709	1	2631	0	3435

TRINITY_DN15981_c0_g1_i6	Glyma.11G048300.2	100	171	6	724	3367	2884	231	0	3890

TRINITY_DN16431_c0_g1_i4	Glyma.03G075200.1	95	471	53	2688	5293	2633	19	0	1740

TRINITY_DN16431_c0_g1_i5	Glyma.03G075200.1	100	471	53	2668	5273	2633	19	0	1740

TRINITY_DN16431_c0_g1_i6	Glyma.03G075200.1	98.66666667	471	53	2331	4936	2633	19	0	1740

TRINITY_DN16431_c0_g1_i7	Glyma.03G075200.1	100	471	53	2311	4916	2633	19	0	1740

TRINITY_DN13377_c0_g1_i1	Glyma.17G020500.1	100	430	68	371	2935	1	2622	0	1748

TRINITY_DN13377_c0_g1_i2	Glyma.17G020500.1	97.36842105	430	68	410	2974	1	2622	0	1748

TRINITY_DN13377_c0_g1_i3	Glyma.17G020500.1	100	430	68	205	2769	1	2622	0	1748

TRINITY_DN15757_c0_g1_i4	Glyma.18G003000.2	100	327	6	152	2797	81	2729	0	3016

TRINITY_DN15757_c0_g1_i6	Glyma.18G003000.2	100	314	7	152	2797	81	2729	0	3088

TRINITY_DN15757_c0_g1_i7	Glyma.18G003000.2	100	288	6	152	2797	81	2729	0	3232

TRINITY_DN15757_c0_g1_i1	Glyma.18G003000.2	98.988622	275	7	152	2797	81	2729	0	3304

TRINITY_DN4116_c0_g1_i1	Glyma.05G010600.1	97.43589744	261	23	79	2704	2641	1	0	3201

TRINITY_DN12988_c0_g2_i1	Glyma.03G167500.1	98.5915493	166	5	1	2643	408	3059	0	3908

TRINITY_DN22188_c0_g1_i1	Glyma.08G143200.3	95.4887218	151	4	3	2655	5155	7801	0	4024

TRINITY_DN22188_c0_g1_i1	Glyma.08G143200.2	95.4887218	151	4	3	2655	5155	7801	0	4024

TRINITY_DN22188_c0_g1_i1	Glyma.08G143200.1	95.4887218	151	4	3	2655	5155	7801	0	4024

TRINITY_DN22188_c0_g1_i1	Glyma.08G143200.4	95.5399061	151	4	3	2655	5155	7801	0	4024

TRINITY_DN16005_c1_g2_i2	Glyma.08G289100.1	96.05263158	301	17	217	2838	1	2646	0	2998

TRINITY_DN10314_c0_g1_i2	Glyma.06G056300.1	100	184	9	285	2925	2650	1	0	3794

TRINITY_DN13152_c0_g1_i4	Glyma.14G189900.1	95.80838323	173	5	628	3279	2637	1	0	3842

TRINITY_DN22188_c0_g1_i1	Glyma.05G185300.1	95.5399061	159	5	3	2654	4099	6743	0	3971

TRINITY_DN13423_c0_g2_i1	Glyma.07G241500.1	100	151	6	838	3483	2643	1	0	3964

TRINITY_DN14839_c0_g1_i1	Glyma.12G235900.1	96.96969697	289	18	426	3047	2637	1	0	3016

TRINITY_DN12822_c0_g1_i2	Glyma.05G145400.1	100	283	17	357	2996	2613	1	0	3011

TRINITY_DN12822_c0_g1_i3	Glyma.05G145400.1	100	283	17	357	2996	2613	1	0	3011

TRINITY_DN16298_c0_g1_i1	Glyma.13G308700.2	100	280	4	603	3248	2650	1	0	3306

TRINITY_DN16318_c0_g1_i6	Glyma.15G133700.1	95.74468085	251	2	521	3155	2814	165	0	3406

TRINITY_DN15328_c0_g1_i2	Glyma.07G056700.3	98.79518072	228	9	19	2655	1357	3990	0	3443

TRINITY_DN15328_c0_g1_i2	Glyma.07G056700.2	100	228	10	19	2655	1357	3987	0	3424

TRINITY_DN16130_c2_g6_i14	Glyma.19G196000.1	97.42120344	285	1	410	3058	271	2916	0	3293

TRINITY_DN16130_c2_g6_i14	Glyma.19G196000.3	97.7443609	285	1	410	3058	109	2754	0	3293

TRINITY_DN16130_c2_g6_i14	Glyma.19G196000.4	98.06094183	285	1	410	3058	109	2754	0	3293

TRINITY_DN16005_c1_g2_i3	Glyma.08G289100.1	100	267	14	217	2835	1	2646	0	3199

TRINITY_DN13736_c0_g1_i1	Glyma.12G236300.1	95.77464789	262	5	1	2643	234	2876	0	3363

TRINITY_DN13736_c0_g1_i1	Glyma.13G201700.1	95.77464789	233	8	1	2643	234	2870	0	3485

TRINITY_DN16130_c2_g6_i4	Glyma.19G196000.1	95.07772021	232	1	410	3058	271	2916	0	3587

TRINITY_DN16130_c2_g6_i4	Glyma.19G196000.3	99.2481203	232	1	410	3058	109	2754	0	3587

TRINITY_DN16130_c2_g6_i4	Glyma.19G196000.4	100	232	1	410	3058	109	2754	0	3587

TRINITY_DN8806_c0_g1_i1	Glyma.08G254000.1	96.33333333	224	17	183	2798	1	2637	0	3360

TRINITY_DN11351_c0_g1_i1	Glyma.04G175400.1	95.34883721	223	5	64	2709	55	2697	0	3598

TRINITY_DN11650_c0_g1_i1	Glyma.05G185000.5	99.43019943	209	8	74	2710	1	2643	0	3618

TRINITY_DN11650_c0_g1_i1	Glyma.05G185000.6	97.87234043	209	8	74	2710	1	2643	0	3618

TRINITY_DN11650_c0_g1_i2	Glyma.05G185000.5	99.08256881	209	8	74	2710	1	2643	0	3618

TRINITY_DN11650_c0_g1_i2	Glyma.05G185000.6	100	209	8	74	2710	1	2643	0	3618

TRINITY_DN15237_c0_g1_i1	Glyma.20G053500.1	95.74468085	184	2	341	2989	1	2643	0	3834

TRINITY_DN15237_c0_g1_i3	Glyma.20G053500.1	96.17834395	184	2	313	2961	1	2643	0	3834

TRINITY_DN13423_c0_g2_i1	Glyma.17G032400.1	100	161	3	838	3483	2646	1	0	3962

TRINITY_DN12627_c0_g1_i2	Glyma.13G317500.5	99.43502825	154	12	772	3411	1	2628	0	3845

TRINITY_DN16431_c0_g1_i4	Glyma.03G034500.1	100	455	43	2706	5308	2633	1	0	1982

TRINITY_DN16431_c0_g1_i5	Glyma.03G034500.1	100	455	43	2686	5288	2633	1	0	1982

TRINITY_DN16431_c0_g1_i6	Glyma.03G034500.1	98.4375	455	43	2349	4951	2633	1	0	1982

TRINITY_DN16431_c0_g1_i7	Glyma.03G034500.1	95.96774194	455	43	2329	4931	2633	1	0	1982

TRINITY_DN14622_c0_g1_i4	Glyma.10G179700.3	100	202	24	67	2703	3	2598	0	3363

TRINITY_DN3710_c0_g1_i1	Glyma.14G137100.1	99.20634921	201	4	255	2896	580	3224	0	3718

TRINITY_DN15242_c0_g1_i2	Glyma.20G086700.5	96.82539683	186	11	221	2857	2631	1	0	3679

TRINITY_DN13959_c0_g1_i3	Glyma.20G242100.1	95.91194969	175	11	235	2842	2990	347	0	3637

TRINITY_DN15235_c0_g1_i3	Glyma.10G227300.5	98.24561404	415	8	34	2673	454	3096	0	2518

TRINITY_DN15235_c0_g1_i3	Glyma.10G227300.4	98.24561404	415	8	34	2673	454	3096	0	2518

TRINITY_DN15235_c0_g1_i3	Glyma.10G227300.3	98.4375	415	8	34	2673	454	3096	0	2518

TRINITY_DN15235_c0_g1_i3	Glyma.10G227300.1	96.96969697	415	8	34	2673	454	3096	0	2518

TRINITY_DN15235_c0_g1_i3	Glyma.10G227300.2	99.01477833	415	8	34	2673	454	3096	0	2518

TRINITY_DN16130_c2_g6_i14	Glyma.03G198100.1	96.10894942	280	0	410	3056	112	2758	0	3338

TRINITY_DN16130_c2_g6_i14	Glyma.03G198100.2	97.14285714	280	0	410	3056	112	2758	0	3338

TRINITY_DN13425_c0_g1_i1	Glyma.11G130200.1	100	251	21	119	2743	88	2721	0	3271

TRINITY_DN13425_c0_g1_i3	Glyma.11G130200.1	97.5	248	29	119	2731	88	2721	0	3210

TRINITY_DN16130_c2_g6_i4	Glyma.03G198100.1	95.53264605	226	0	410	3056	112	2758	0	3637

TRINITY_DN16130_c2_g6_i4	Glyma.03G198100.2	97.01492537	226	0	410	3056	112	2758	0	3637

TRINITY_DN14463_c0_g1_i1	Glyma.06G134300.1	100	178	25	281	2914	1	2604	0	3541

TRINITY_DN12915_c0_g1_i2	Glyma.10G189200.1	97.83653846	520	53	505	3103	3053	431	0	1554

TRINITY_DN16431_c0_g1_i4	Glyma.03G037300.2	99.6763754	419	42	2706	5308	2627	1	0	2165

TRINITY_DN16431_c0_g1_i4	Glyma.03G037300.4	96.8503937	419	42	2706	5308	2627	1	0	2165

TRINITY_DN16431_c0_g1_i4	Glyma.03G037300.1	96.8503937	419	42	2706	5308	2627	1	0	2165

TRINITY_DN16431_c0_g1_i4	Glyma.03G037300.5	100	419	42	2706	5308	2627	1	0	2165

TRINITY_DN16431_c0_g1_i4	Glyma.03G037300.3	98.25581395	419	42	2706	5308	2627	1	0	2165

TRINITY_DN16431_c0_g1_i5	Glyma.03G037300.2	97.51552795	419	42	2686	5288	2627	1	0	2165

TRINITY_DN16431_c0_g1_i5	Glyma.03G037300.4	100	419	42	2686	5288	2627	1	0	2165

TRINITY_DN16431_c0_g1_i5	Glyma.03G037300.1	96	419	42	2686	5288	2627	1	0	2165

TRINITY_DN16431_c0_g1_i5	Glyma.03G037300.5	96.53465347	419	42	2686	5288	2627	1	0	2165

TRINITY_DN16431_c0_g1_i5	Glyma.03G037300.3	97.44318182	419	42	2686	5288	2627	1	0	2165

TRINITY_DN16431_c0_g1_i6	Glyma.03G037300.2	99.2	419	42	2349	4951	2627	1	0	2165

TRINITY_DN16431_c0_g1_i6	Glyma.03G037300.4	95.54455446	419	42	2349	4951	2627	1	0	2165

TRINITY_DN16431_c0_g1_i6	Glyma.03G037300.1	97.20930233	419	42	2349	4951	2627	1	0	2165

TRINITY_DN16431_c0_g1_i6	Glyma.03G037300.5	100	419	42	2349	4951	2627	1	0	2165

TRINITY_DN16431_c0_g1_i6	Glyma.03G037300.3	98.7804878	419	42	2349	4951	2627	1	0	2165

TRINITY_DN16431_c0_g1_i7	Glyma.03G037300.2	96.76806084	419	42	2329	4931	2627	1	0	2165

TRINITY_DN16431_c0_g1_i7	Glyma.03G037300.4	95.36423841	419	42	2329	4931	2627	1	0	2165

TRINITY_DN16431_c0_g1_i7	Glyma.03G037300.1	100	419	42	2329	4931	2627	1	0	2165

TRINITY_DN16431_c0_g1_i7	Glyma.03G037300.5	100	419	42	2329	4931	2627	1	0	2165

TRINITY_DN16431_c0_g1_i7	Glyma.03G037300.3	96.70103093	419	42	2329	4931	2627	1	0	2165

TRINITY_DN11351_c0_g1_i1	Glyma.06G189500.2	95.34883721	241	5	67	2709	1	2640	0	3493

TRINITY_DN14283_c0_g1_i5	Glyma.18G143900.2	100	227	15	1880	4519	2832	211	0	3435

TRINITY_DN14283_c0_g1_i6	Glyma.18G143900.2	100	227	15	2031	4670	2832	211	0	3435

TRINITY_DN11049_c0_g3_i1	Glyma.10G045500.1	97.4025974	223	8	257	2893	3130	491	0	3554

TRINITY_DN11049_c0_g3_i1	Glyma.10G045500.2	100	223	8	257	2893	2686	47	0	3554

TRINITY_DN15549_c0_g1_i2	Glyma.08G055400.2	95.07389163	195	9	1048	3662	2642	1	0	3579

TRINITY_DN15549_c0_g1_i6	Glyma.08G055400.2	100	195	9	1012	3626	2642	1	0	3579

TRINITY_DN14021_c0_g2_i1	Glyma.11G018700.1	100	171	7	905	3540	3372	740	0	3790

TRINITY_DN14283_c0_g1_i1	Glyma.18G143900.2	99.28057554	227	15	512	3150	2831	211	0	3434

TRINITY_DN14283_c0_g1_i5	Glyma.18G143900.1	100	227	15	1881	4519	2831	211	0	3434

TRINITY_DN14283_c0_g1_i6	Glyma.18G143900.1	100	227	15	2032	4670	2831	211	0	3434

TRINITY_DN13844_c0_g1_i1	Glyma.01G074000.1	97.51243781	194	3	335	2977	1	2643	0	3784

TRINITY_DN13844_c0_g1_i1	Glyma.01G074000.3	96.35535308	194	3	335	2977	1	2643	0	3784

TRINITY_DN13844_c0_g1_i1	Glyma.01G074000.2	96.34369287	191	9	335	2977	1	2622	0	3664

TRINITY_DN14021_c0_g2_i1	Glyma.01G224200.3	96.98630137	186	7	905	3540	3390	752	0	3757

TRINITY_DN14021_c0_g2_i1	Glyma.01G224200.1	100	186	7	905	3540	3390	752	0	3757

TRINITY_DN14021_c0_g2_i1	Glyma.01G224200.2	96.82337992	186	7	905	3540	3390	752	0	3757

TRINITY_DN13959_c0_g1_i3	Glyma.20G242100.2	98.94736842	175	10	235	2842	2987	347	0	3650

TRINITY_DN16431_c0_g1_i4	Glyma.03G043500.1	100	479	41	2706	5308	2615	1	0	1777

TRINITY_DN16431_c0_g1_i5	Glyma.03G043500.1	100	479	41	2686	5288	2615	1	0	1777

TRINITY_DN16431_c0_g1_i6	Glyma.03G043500.1	95	479	41	2349	4951	2615	1	0	1777

TRINITY_DN16431_c0_g1_i7	Glyma.03G043500.1	95.40942928	479	41	2329	4931	2615	1	0	1777

TRINITY_DN16140_c0_g2_i2	Glyma.12G013600.1	98.67109635	273	17	106	2723	1	2621	0	3053

TRINITY_DN16140_c0_g2_i2	Glyma.12G013600.2	99.05913978	273	17	106	2723	1	2621	0	3053

TRINITY_DN16140_c0_g2_i5	Glyma.12G013600.1	98.61111111	273	17	169	2786	1	2621	0	3053

TRINITY_DN16140_c0_g2_i5	Glyma.12G013600.2	97.99691834	273	17	169	2786	1	2621	0	3053

TRINITY_DN14576_c0_g1_i5	Glyma.09G229900.2	96	267	13	1336	3969	538	3174	0	3293

TRINITY_DN14576_c0_g1_i5	Glyma.09G229900.1	96.93251534	267	13	1336	3969	538	3174	0	3293

TRINITY_DN14576_c0_g1_i7	Glyma.09G229900.2	100	267	13	1653	4286	538	3174	0	3293

TRINITY_DN14576_c0_g1_i7	Glyma.09G229900.1	96.82804674	267	13	1653	4286	538	3174	0	3293

TRINITY_DN14283_c0_g1_i5	Glyma.08G282400.7	100	219	18	1879	4513	2821	202	0	3456

TRINITY_DN14283_c0_g1_i6	Glyma.08G282400.7	100	219	18	2030	4664	2821	202	0	3456

TRINITY_DN16406_c0_g1_i1	Glyma.01G006600.1	97.25685786	194	0	89	2732	683	3326	0	3808

TRINITY_DN16406_c0_g1_i1	Glyma.01G006600.2	96.34146341	194	0	89	2732	56	2699	0	3808

TRINITY_DN16406_c0_g1_i1	Glyma.07G125200.2	95.15669516	190	0	89	2732	683	3326	0	3831

TRINITY_DN16406_c0_g1_i1	Glyma.07G125200.1	95.82172702	190	0	89	2732	683	3326	0	3831

TRINITY_DN13110_c0_g2_i2	Glyma.15G067900.1	96.80851064	164	6	481	3120	2637	1	0	3903

TRINITY_DN16298_c0_g1_i2	Glyma.13G308700.2	99.65277778	156	1	656	3296	2644	1	0	3999

TRINITY_DN8963_c0_g1_i1	Glyma.18G055700.1	98.125	417	6	1	2640	332	2971	0	2532

TRINITY_DN13844_c0_g1_i1	Glyma.11G175700.1	95.65217391	201	1	335	2977	1	2640	0	3747

TRINITY_DN13844_c0_g1_i1	Glyma.11G175700.3	96.34146341	201	1	335	2977	1	2640	0	3747

TRINITY_DN11049_c0_g3_i1	Glyma.13G133200.1	95.3271028	201	13	257	2893	3112	491	0	3592

TRINITY_DN15088_c0_g1_i1	Glyma.05G166300.1	100	194	8	617	3256	1095	3719	0	3670

TRINITY_DN13110_c0_g2_i2	Glyma.13G245900.1	96.79487179	165	3	481	3120	2640	1	0	3928

TRINITY_DN11610_c0_g2_i1	Glyma.10G189200.1	97.82608696	574	76	493	3080	450	3055	0	1118

TRINITY_DN11351_c0_g2_i1	Glyma.04G175400.1	99.28057554	389	19	83	2709	65	2697	0	2569

TRINITY_DN16140_c0_g2_i2	Glyma.09G223300.3	95.86466165	260	19	106	2723	1	2615	0	3109

TRINITY_DN16140_c0_g2_i2	Glyma.09G223300.5	95.86466165	260	19	106	2723	1	2615	0	3109

TRINITY_DN16140_c0_g2_i2	Glyma.09G223300.6	96.42857143	260	19	106	2723	1	2615	0	3109

TRINITY_DN16140_c0_g2_i2	Glyma.09G223300.4	95.91836735	260	19	106	2723	1	2615	0	3109

TRINITY_DN16140_c0_g2_i2	Glyma.09G223300.1	96.2962963	260	19	106	2723	1	2615	0	3109

TRINITY_DN16140_c0_g2_i2	Glyma.09G223300.2	97.5	260	19	106	2723	1	2615	0	3109

TRINITY_DN16140_c0_g2_i5	Glyma.09G223300.3	96.41255605	260	19	169	2786	1	2615	0	3109

TRINITY_DN16140_c0_g2_i5	Glyma.09G223300.5	96.41350211	260	19	169	2786	1	2615	0	3109

TRINITY_DN16140_c0_g2_i5	Glyma.09G223300.6	96.11111111	260	19	169	2786	1	2615	0	3109

TRINITY_DN16140_c0_g2_i5	Glyma.09G223300.4	99.24242424	260	19	169	2786	1	2615	0	3109

TRINITY_DN16140_c0_g2_i5	Glyma.09G223300.1	99.24242424	260	19	169	2786	1	2615	0	3109

TRINITY_DN16140_c0_g2_i5	Glyma.09G223300.2	98.61111111	260	19	169	2786	1	2615	0	3109

TRINITY_DN15328_c0_g1_i4	Glyma.07G056700.1	100	227	9	19	2647	1357	3982	0	3434

TRINITY_DN14283_c0_g1_i1	Glyma.08G282400.7	99.29577465	219	18	512	3144	2819	202	0	3452

TRINITY_DN14283_c0_g1_i5	Glyma.08G282400.3	98.4375	219	18	1881	4513	2819	202	0	3452

TRINITY_DN14283_c0_g1_i5	Glyma.08G282400.11	100	219	18	1881	4513	2819	202	0	3452

TRINITY_DN14283_c0_g1_i6	Glyma.08G282400.3	100	219	18	2032	4664	2819	202	0	3452

TRINITY_DN14283_c0_g1_i6	Glyma.08G282400.11	100	219	18	2032	4664	2819	202	0	3452

TRINITY_DN10096_c0_g1_i1	Glyma.01G187400.1	98.02955665	192	10	13	2638	2010	4635	0	3609

TRINITY_DN15237_c0_g1_i1	Glyma.14G043900.1	99.07407407	453	24	365	2987	7	2632	0	2154

TRINITY_DN15237_c0_g1_i3	Glyma.14G043900.1	97.4537037	453	24	337	2959	7	2632	0	2154

TRINITY_DN15629_c1_g2_i1	Glyma.08G302900.1	100	425	26	105	2724	81	2712	0	2329

TRINITY_DN15122_c0_g2_i1	Glyma.11G221800.2	97.85714286	377	6	1227	3865	3758	1126	0	2724

TRINITY_DN15122_c0_g2_i1	Glyma.11G221800.1	100	377	6	1227	3865	3758	1126	0	2724

TRINITY_DN15328_c0_g1_i2	Glyma.07G056700.1	98.40954274	227	9	19	2646	1357	3981	0	3432

TRINITY_DN15328_c0_g1_i4	Glyma.07G056700.3	100	227	9	19	2646	1357	3981	0	3432

TRINITY_DN15328_c0_g1_i4	Glyma.07G056700.2	100	227	10	19	2646	1357	3978	0	3413

TRINITY_DN14283_c0_g1_i5	Glyma.08G282400.6	100	219	17	1879	4513	2818	202	0	3470

TRINITY_DN14283_c0_g1_i6	Glyma.08G282400.6	100	219	17	2030	4664	2818	202	0	3470

TRINITY_DN15311_c0_g1_i1	Glyma.08G363300.1	98.73417722	189	2	804	3443	670	3309	0	3818

TRINITY_DN8963_c0_g1_i1	Glyma.02G233700.1	98.11827957	177	2	3	2642	349	2988	0	3884

TRINITY_DN11037_c0_g1_i1	Glyma.10G172400.1	96.89440994	171	5	217	2853	1	2635	0	3866

TRINITY_DN14245_c0_g2_i1	Glyma.13G181700.4	95.66473988	171	7	438	3054	1	2635	0	3736

TRINITY_DN14431_c0_g1_i1	Glyma.17G089100.1	96.36363636	167	16	224	2844	2639	1	0	3810

TRINITY_DN12915_c0_g1_i2	Glyma.20G201500.1	97.27272727	512	54	508	3102	3032	414	0	1613

TRINITY_DN16431_c0_g1_i4	Glyma.03G034800.1	100	448	26	2706	5308	2627	1	0	2071

TRINITY_DN16431_c0_g1_i5	Glyma.03G034800.1	97.45762712	448	26	2686	5288	2627	1	0	2071

TRINITY_DN16431_c0_g1_i6	Glyma.03G034800.1	96.66666667	448	26	2349	4951	2627	1	0	2071

TRINITY_DN16431_c0_g1_i7	Glyma.03G034800.1	100	448	26	2329	4931	2627	1	0	2071

TRINITY_DN15918_c0_g1_i4	Glyma.18G268900.1	98.90625	314	20	593	3182	1	2629	0	2741

TRINITY_DN15918_c0_g1_i4	Glyma.18G268900.3	98.90710383	314	20	593	3182	1	2629	0	2741

TRINITY_DN15918_c0_g1_i5	Glyma.18G268900.1	95.30916844	314	20	312	2901	1	2629	0	2741

TRINITY_DN15918_c0_g1_i5	Glyma.18G268900.3	98.1981982	314	20	312	2901	1	2629	0	2741

TRINITY_DN15918_c0_g1_i4	Glyma.08G247500.2	100	309	22	593	3182	1	2626	0	2750

TRINITY_DN15918_c0_g1_i4	Glyma.08G247500.1	100	309	22	593	3182	1	2626	0	2750

TRINITY_DN15918_c0_g1_i5	Glyma.08G247500.2	99.41348974	309	22	312	2901	1	2626	0	2750

TRINITY_DN15918_c0_g1_i5	Glyma.08G247500.1	99.41860465	309	22	312	2901	1	2626	0	2750

TRINITY_DN16130_c2_g6_i15	Glyma.10G075300.2	99.57983193	260	8	393	3025	136	2771	0	3363

TRINITY_DN12617_c0_g1_i2	Glyma.09G063200.1	98.50746269	220	5	1872	4505	1210	3846	0	3598

TRINITY_DN12617_c0_g1_i2	Glyma.09G063200.3	98.50746269	220	5	1872	4505	811	3447	0	3598

TRINITY_DN12617_c0_g1_i2	Glyma.09G063200.6	100	216	11	1872	4505	1210	3822	0	3465

TRINITY_DN16130_c2_g6_i5	Glyma.10G075300.2	100	202	8	393	3025	136	2771	0	3685

TRINITY_DN8963_c0_g1_i1	Glyma.14G201500.1	98.125	179	0	3	2642	352	2991	0	3884

TRINITY_DN16130_c2_g6_i17	Glyma.19G196000.1	95.12195122	311	2	410	3048	271	2903	0	3112

TRINITY_DN16130_c2_g6_i17	Glyma.19G196000.3	98.52941176	311	2	410	3048	109	2741	0	3112

TRINITY_DN16130_c2_g6_i17	Glyma.19G196000.4	95.12195122	311	2	410	3048	109	2741	0	3112

TRINITY_DN16130_c2_g6_i17	Glyma.03G198100.1	97.11538462	305	1	410	3048	112	2747	0	3164

TRINITY_DN16130_c2_g6_i17	Glyma.03G198100.2	97.11538462	305	1	410	3048	112	2747	0	3164

TRINITY_DN16130_c2_g6_i8	Glyma.19G196000.1	95.09981851	258	2	410	3048	271	2903	0	3406

TRINITY_DN16130_c2_g6_i8	Glyma.19G196000.3	99.58158996	258	2	410	3048	109	2741	0	3406

TRINITY_DN16130_c2_g6_i8	Glyma.19G196000.4	97.36842105	258	2	410	3048	109	2741	0	3406

TRINITY_DN16130_c2_g6_i8	Glyma.03G198100.1	98.82352941	251	1	410	3048	112	2747	0	3463

TRINITY_DN16130_c2_g6_i8	Glyma.03G198100.2	98.82352941	251	1	410	3048	112	2747	0	3463

TRINITY_DN8806_c0_g1_i1	Glyma.18G276500.1	95.23809524	242	19	183	2792	1	2622	0	3236

TRINITY_DN14283_c0_g1_i1	Glyma.08G282400.6	99.28909953	219	17	512	3144	2816	202	0	3467

TRINITY_DN14283_c0_g1_i5	Glyma.08G282400.2	100	219	17	1881	4513	2816	202	0	3467

TRINITY_DN14283_c0_g1_i5	Glyma.08G282400.10	100	219	17	1881	4513	2816	202	0	3467

TRINITY_DN14283_c0_g1_i6	Glyma.08G282400.2	100	219	17	2032	4664	2816	202	0	3467

TRINITY_DN14283_c0_g1_i6	Glyma.08G282400.10	100	219	17	2032	4664	2816	202	0	3467

TRINITY_DN8781_c0_g1_i1	Glyma.15G190800.1	99.49238579	175	4	511	3139	2666	34	0	3801

TRINITY_DN8781_c0_g1_i1	Glyma.15G190800.2	97.6	175	5	511	3139	2657	34	0	3742

TRINITY_DN11037_c0_g1_i1	Glyma.20G217800.1	96.875	170	3	217	2851	1	2639	0	3906

TRINITY_DN15847_c1_g2_i1	Glyma.14G034200.3	96.36363636	125	0	127	2765	14	2652	0	4181

TRINITY_DN15847_c1_g2_i3	Glyma.14G034200.3	95.12711864	117	0	127	2765	14	2652	0	4226

TRINITY_DN16130_c2_g6_i15	Glyma.02G201300.2	98.38709677	249	4	393	3025	133	2768	0	3446

TRINITY_DN16130_c2_g6_i15	Glyma.02G201300.4	100	249	4	393	3025	133	2768	0	3446

TRINITY_DN16130_c2_g6_i15	Glyma.02G201300.1	100	249	4	393	3025	133	2768	0	3446

TRINITY_DN15891_c1_g1_i1	Glyma.02G046800.2	100	222	8	244	2869	1	2635	0	3544

TRINITY_DN16130_c2_g6_i5	Glyma.02G201300.2	100	191	4	393	3025	133	2768	0	3768

TRINITY_DN16130_c2_g6_i5	Glyma.02G201300.4	98.78787879	191	4	393	3025	133	2768	0	3768

TRINITY_DN16130_c2_g6_i5	Glyma.02G201300.1	100	191	4	393	3025	133	2768	0	3768

TRINITY_DN15459_c0_g1_i1	Glyma.19G113200.1	100	182	0	525	3162	3357	720	0	3864

TRINITY_DN14463_c0_g1_i1	Glyma.06G134300.4	97.82608696	179	20	281	2914	1	2595	0	3576

TRINITY_DN14463_c0_g1_i1	Glyma.06G134300.2	100	178	25	281	2914	1	2589	0	3542

TRINITY_DN11676_c0_g2_i1	Glyma.11G209000.1	100	399	38	503	3113	1	2599	0	2246

TRINITY_DN15736_c0_g1_i2	Glyma.06G162300.1	96.47058824	237	21	296	2897	203	2825	0	3240

TRINITY_DN15459_c0_g1_i1	Glyma.16G039900.1	96.20535714	193	0	525	3161	3345	709	0	3801

TRINITY_DN15459_c0_g1_i1	Glyma.16G039900.2	97.02970297	193	0	525	3161	3345	709	0	3801

TRINITY_DN14463_c0_g1_i1	Glyma.06G134300.3	100	179	20	281	2913	1	2594	0	3574

TRINITY_DN14288_c0_g1_i1	Glyma.09G087900.1	98.96907216	148	1	742	3378	3567	934	0	4030

TRINITY_DN14288_c0_g1_i1	Glyma.15G194000.1	100	141	2	742	3378	3564	934	0	4050

TRINITY_DN9977_c0_g1_i1	Glyma.U028300.4	100	288	16	56	2675	2602	1	0	2950

TRINITY_DN9977_c0_g1_i1	Glyma.U028300.3	100	288	16	56	2675	2602	1	0	2950

TRINITY_DN9977_c0_g1_i1	Glyma.U028300.1	100	288	16	56	2675	2602	1	0	2950

TRINITY_DN9977_c0_g1_i1	Glyma.U028300.2	100	288	16	56	2675	2602	1	0	2950

TRINITY_DN14783_c0_g1_i1	Glyma.01G111300.1	96.96969697	176	3	354	2988	2664	33	0	3860

TRINITY_DN14783_c0_g1_i2	Glyma.01G111300.1	96.96969697	176	3	354	2988	2664	33	0	3860

TRINITY_DN16094_c0_g1_i1	Glyma.11G004100.2	100	153	3	567	3198	2637	3	0	3988

TRINITY_DN16094_c0_g1_i6	Glyma.11G004100.2	97.5483871	153	3	567	3198	2637	3	0	3988

TRINITY_DN14670_c0_g1_i3	Glyma.13G065500.1	96.8879668	248	17	530	3158	243	2838	0	3201

TRINITY_DN14670_c0_g1_i3	Glyma.13G065500.7	99.53379953	248	18	530	3158	243	2829	0	3144

TRINITY_DN14622_c0_g1_i4	Glyma.10G179700.1	95.83333333	200	23	78	2703	97	2679	0	3363

TRINITY_DN14622_c0_g1_i4	Glyma.10G179700.2	100	200	23	78	2703	88	2670	0	3363

TRINITY_DN14783_c0_g1_i1	Glyma.03G076900.1	96.96969697	186	2	354	2988	2661	30	0	3816

TRINITY_DN14783_c0_g1_i2	Glyma.03G076900.1	96.96261682	186	2	354	2988	2661	30	0	3816

TRINITY_DN11610_c0_g2_i1	Glyma.20G201500.1	99.36305732	572	56	493	3080	432	3037	0	1218

TRINITY_DN15568_c1_g1_i1	Glyma.06G267000.1	99.07834101	452	54	720	3299	110	2718	0	1849

TRINITY_DN14839_c0_g1_i1	Glyma.13G201400.1	100	324	23	426	3047	2610	1	0	2837

TRINITY_DN36201_c0_g1_i1	Glyma.16G099100.2	99.39393939	222	10	211	2829	1	2628	0	3498

TRINITY_DN36201_c0_g1_i1	Glyma.16G099100.1	99.39393939	222	10	211	2829	1	2628	0	3498

TRINITY_DN16245_c0_g3_i6	Glyma.16G099700.1	99.24242424	220	0	399	3032	3473	840	0	3646

TRINITY_DN16080_c0_g1_i1	Glyma.02G274900.1	98.57142857	480	19	242	2866	103	2721	0	2061

TRINITY_DN15568_c1_g1_i1	Glyma.12G136000.1	99.08256881	467	46	710	3299	112	2703	0	1733

TRINITY_DN15458_c0_g1_i2	Glyma.17G004700.2	95.4887218	390	32	403	2994	1	2622	0	2366

TRINITY_DN9507_c0_g1_i1	Glyma.11G080600.2	100	141	0	113	2745	1	2633	0	4082

TRINITY_DN9507_c0_g2_i1	Glyma.11G080600.2	96.12403101	141	0	113	2745	1	2633	0	4082

TRINITY_DN16080_c0_g1_i1	Glyma.14G041100.1	95.10638298	476	15	242	2866	103	2721	0	2095

TRINITY_DN14670_c0_g1_i3	Glyma.13G065500.10	97.20670391	258	18	765	3390	1	2590	0	3121

TRINITY_DN14670_c0_g1_i3	Glyma.13G065500.8	96.07142857	258	18	765	3390	1	2590	0	3121

TRINITY_DN14670_c0_g1_i3	Glyma.13G065500.13	96.07250755	256	20	765	3390	1	2587	0	3112

TRINITY_DN13640_c1_g2_i1	Glyma.09G255400.1	96.07843137	212	18	441	3016	2982	359	0	3273

TRINITY_DN16043_c0_g1_i1	Glyma.18G129200.1	100	302	7	107	2732	205	2832	0	3134

TRINITY_DN9977_c0_g1_i1	Glyma.01G107000.1	95.70552147	289	32	56	2675	2572	1	0	2806

TRINITY_DN2601_c0_g1_i1	Glyma.04G089400.1	99	195	7	459	3086	4032	1414	0	3681

TRINITY_DN2601_c0_g1_i1	Glyma.04G089400.3	98.98989899	195	7	459	3086	4023	1405	0	3681

TRINITY_DN2601_c0_g1_i1	Glyma.06G091300.1	99	189	6	459	3086	4035	1417	0	3714

TRINITY_DN2601_c0_g1_i1	Glyma.06G091300.3	98.98989899	189	6	459	3086	3621	1003	0	3714

TRINITY_DN16043_c0_g1_i1	Glyma.08G293900.1	95.45454545	333	5	107	2732	1057	3684	0	2974

TRINITY_DN4116_c0_g1_i1	Glyma.17G118300.1	99.3993994	271	20	96	2692	2618	1	0	3064

TRINITY_DN13941_c0_g1_i2	Glyma.09G228100.1	99.05660377	214	53	1	2533	465	3057	0	2806

TRINITY_DN13941_c0_g1_i2	Glyma.09G228100.2	99.05660377	214	53	1	2533	465	3057	0	2806

TRINITY_DN14622_c0_g1_i6	Glyma.10G179700.3	96.5973535	203	21	67	2685	3	2598	0	3441

TRINITY_DN15237_c0_g1_i1	Glyma.02G273500.1	96.2962963	445	14	365	2978	7	2629	0	2254

TRINITY_DN15237_c0_g1_i1	Glyma.02G273500.2	100	445	14	365	2978	7	2629	0	2254

TRINITY_DN15237_c0_g1_i3	Glyma.02G273500.1	96.1038961	445	14	337	2950	7	2629	0	2254

TRINITY_DN15237_c0_g1_i3	Glyma.02G273500.2	98.0952381	445	14	337	2950	7	2629	0	2254

TRINITY_DN10413_c0_g1_i1	Glyma.04G038200.2	100	294	52	599	3181	2556	1	0	2457

TRINITY_DN10413_c0_g1_i3	Glyma.04G038200.2	98.88888889	294	52	392	2974	2556	1	0	2457

TRINITY_DN14670_c0_g1_i2	Glyma.19G019100.3	96.8	247	11	765	3386	1	2619	0	3376

TRINITY_DN15142_c0_g1_i1	Glyma.15G018800.1	97.6	224	14	209	2835	2650	42	0	3472

TRINITY_DN13640_c1_g2_i1	Glyma.09G255400.4	96.07843137	212	17	441	3016	2979	359	0	3286

TRINITY_DN13640_c1_g2_i1	Glyma.09G255400.3	96.07843137	210	18	441	3016	2976	359	0	3278

TRINITY_DN13640_c1_g2_i1	Glyma.18G237600.1	100	207	19	441	3016	2976	359	0	3295

TRINITY_DN15736_c0_g1_i2	Glyma.04G203200.1	96.47058824	236	20	293	2897	173	2786	0	3306

TRINITY_DN16005_c1_g2_i3	Glyma.02G033300.1	100	191	7	217	2835	1	2622	0	3698

TRINITY_DN16005_c1_g2_i2	Glyma.02G033300.1	96.05263158	132	6	217	2838	1	2622	0	4045

TRINITY_DN14437_c0_g1_i2	Glyma.07G070500.2	98.63013699	264	13	1236	3838	17	2616	0	3059

TRINITY_DN14245_c0_g2_i3	Glyma.13G181700.3	95.65217391	189	10	446	3066	94	2702	0	3650

TRINITY_DN16311_c0_g1_i7	Glyma.17G089200.1	96.63608563	177	9	1	2606	77	2702	0	3729

TRINITY_DN14064_c0_g1_i4	Glyma.13G209800.1	97.8021978	142	1	1893	4516	2645	19	0	4045

TRINITY_DN14064_c0_g1_i4	Glyma.15G102800.1	96.59367397	142	1	1893	4516	2627	1	0	4045

TRINITY_DN16175_c0_g1_i3	Glyma.05G110400.1	95.33333333	426	15	3	2612	415	3027	0	2302

TRINITY_DN16175_c0_g1_i7	Glyma.05G110400.1	100	426	15	3	2612	415	3027	0	2302

TRINITY_DN16175_c0_g1_i2	Glyma.17G156300.1	100	411	10	3	2621	415	3033	0	2483

TRINITY_DN16175_c0_g1_i2	Glyma.05G110400.1	100	400	15	3	2621	415	3027	0	2505

TRINITY_DN16447_c1_g1_i6	Glyma.08G162300.4	96.45833333	225	7	934	3553	1995	4614	0	3526

TRINITY_DN16447_c1_g1_i6	Glyma.08G162300.3	100	224	8	934	3553	1995	4611	0	3511

TRINITY_DN16447_c1_g1_i7	Glyma.08G162300.4	95.10869565	224	8	1168	3784	1995	4614	0	3511

TRINITY_DN16447_c1_g1_i7	Glyma.08G162300.3	95.10961214	223	9	1168	3784	1995	4611	0	3498

TRINITY_DN13640_c1_g2_i1	Glyma.18G237600.2	96.22641509	207	18	441	3016	2973	359	0	3308

TRINITY_DN16311_c0_g1_i5	Glyma.17G089200.1	96.55172414	146	7	1	2606	77	2702	0	3912

TRINITY_DN11351_c0_g2_i1	Glyma.06G189500.2	100	378	17	97	2709	25	2640	0	2617

TRINITY_DN16005_c1_g2_i3	Glyma.02G033300.2	100	192	6	217	2835	1	2619	0	3707

TRINITY_DN16005_c1_g2_i2	Glyma.02G033300.2	96.05055292	132	5	217	2838	1	2619	0	4058

TRINITY_DN15235_c0_g1_i7	Glyma.16G172400.1	98.63760218	442	31	1	2606	575	3180	0	2165

TRINITY_DN15235_c0_g1_i7	Glyma.16G172400.4	95.52238806	442	31	1	2606	533	3138	0	2165

TRINITY_DN15235_c0_g1_i7	Glyma.16G172400.3	100	442	31	1	2606	506	3111	0	2165

TRINITY_DN15458_c0_g1_i4	Glyma.15G121500.3	100	300	10	119	2737	55	2664	0	3061

TRINITY_DN16311_c0_g1_i7	Glyma.17G089200.2	96.63865546	179	7	1	2606	77	2699	0	3731

TRINITY_DN16175_c0_g1_i3	Glyma.17G156300.1	100	436	10	3	2612	415	3033	0	2318

TRINITY_DN16175_c0_g1_i7	Glyma.17G156300.1	96.2962963	436	10	3	2612	415	3033	0	2318

TRINITY_DN15458_c0_g1_i1	Glyma.15G121500.3	96.79420889	238	8	119	2737	55	2664	0	3415

TRINITY_DN16311_c0_g1_i5	Glyma.17G089200.2	96.55172414	148	5	1	2606	77	2699	0	3914

TRINITY_DN10934_c0_g2_i2	Glyma.03G247800.1	100	292	33	388	2975	8	2610	0	2881

TRINITY_DN16450_c0_g1_i1	Glyma.10G079000.1	100	223	25	172	2724	2610	1	0	3083

TRINITY_DN16005_c1_g2_i3	Glyma.01G032100.1	100	187	4	217	2835	1	2616	0	3747

TRINITY_DN13963_c0_g1_i1	Glyma.06G053800.1	96.90721649	181	0	127	2748	1	2622	0	3840

TRINITY_DN16005_c1_g2_i2	Glyma.01G032100.1	96.05055292	130	3	217	2838	1	2616	0	4084

TRINITY_DN2236_c0_g1_i1	Glyma.14G039600.1	97.09677419	382	32	394	2999	2597	1	0	2471

TRINITY_DN16175_c0_g1_i2	Glyma.01G197800.1	100	265	7	1	2619	428	3037	0	3288

TRINITY_DN5178_c0_g1_i1	Glyma.19G152000.1	99.0990991	228	6	703	3317	2617	1	0	3513

TRINITY_DN16071_c0_g2_i7	Glyma.09G052000.1	99.21875	369	16	80	2688	347	2959	0	2678

TRINITY_DN16071_c0_g2_i7	Glyma.15G158300.1	100	363	17	80	2688	377	2989	0	2712

TRINITY_DN16175_c0_g1_i2	Glyma.11G043800.1	100	278	5	1	2619	428	3037	0	3227

TRINITY_DN12309_c0_g1_i3	Glyma.10G012600.1	100	179	5	507	3122	2613	1	0	3775

TRINITY_DN12309_c0_g1_i2	Glyma.10G012600.1	99	178	6	507	3110	2613	1	0	3703

TRINITY_DN16499_c0_g1_i1	Glyma.08G265300.5	96.16519174	154	7	530	3145	1	2607	0	3875

TRINITY_DN15295_c0_g1_i1	Glyma.20G173200.6	100	189	13	2074	4669	2623	13	0	3589

TRINITY_DN15295_c0_g1_i1	Glyma.20G173200.4	96.36363636	189	13	2074	4669	2623	13	0	3589

TRINITY_DN15295_c0_g1_i1	Glyma.20G173200.5	96.36363636	189	13	2074	4669	2623	13	0	3589

TRINITY_DN15295_c0_g1_i2	Glyma.20G173200.6	98.63013699	189	13	2074	4669	2623	13	0	3589

TRINITY_DN15295_c0_g1_i2	Glyma.20G173200.4	100	189	13	2074	4669	2623	13	0	3589

TRINITY_DN15295_c0_g1_i2	Glyma.20G173200.5	100	189	13	2074	4669	2623	13	0	3589

TRINITY_DN12878_c0_g1_i1	Glyma.11G231700.1	100	221	28	33	2627	1	2592	0	3293

TRINITY_DN11676_c0_g2_i1	Glyma.19G193100.3	97.25274725	214	10	503	3113	1	2605	0	3520

TRINITY_DN11676_c0_g2_i1	Glyma.19G193100.6	100	214	10	503	3113	1	2605	0	3520

TRINITY_DN11676_c0_g2_i1	Glyma.19G193100.4	100	214	10	503	3113	1	2605	0	3520

TRINITY_DN11676_c0_g2_i1	Glyma.19G193100.12	95.63492063	214	10	503	3113	1	2605	0	3520

TRINITY_DN11676_c0_g2_i1	Glyma.19G193100.7	100	214	10	503	3113	1	2605	0	3520

TRINITY_DN11676_c0_g2_i1	Glyma.19G193100.10	98.80952381	214	10	503	3113	1	2605	0	3520

TRINITY_DN11676_c0_g2_i1	Glyma.19G193100.8	99.11504425	214	10	503	3113	1	2605	0	3520

TRINITY_DN11676_c0_g2_i1	Glyma.19G193100.5	96.48760331	214	10	503	3113	1	2605	0	3520

TRINITY_DN11676_c0_g2_i1	Glyma.19G193100.9	100	214	10	503	3113	1	2605	0	3520

TRINITY_DN11676_c0_g2_i1	Glyma.19G193100.14	100	214	10	503	3113	1	2605	0	3520

TRINITY_DN11676_c0_g2_i1	Glyma.19G193100.11	100	214	10	503	3113	1	2605	0	3520

TRINITY_DN11676_c0_g2_i1	Glyma.19G193100.1	99.37304075	214	10	503	3113	1	2605	0	3520

TRINITY_DN11676_c0_g2_i1	Glyma.19G193100.2	95.77464789	214	10	503	3113	1	2605	0	3520

TRINITY_DN11676_c0_g2_i1	Glyma.19G193100.13	96.49122807	214	10	503	3113	1	2605	0	3520

TRINITY_DN12309_c0_g1_i3	Glyma.02G012100.1	96.72131148	198	6	507	3122	2607	1	0	3653

TRINITY_DN12309_c0_g1_i2	Glyma.02G012100.1	96.42857143	195	7	507	3110	2607	1	0	3592

TRINITY_DN16078_c0_g1_i1	Glyma.06G289400.1	100	191	0	236	2853	41	2658	0	3777

TRINITY_DN16078_c0_g1_i1	Glyma.12G117600.1	100	187	0	236	2853	41	2658	0	3799

TRINITY_DN16245_c0_g1_i5	Glyma.07G007700.11	100	180	7	1444	4059	2595	1	0	3675

TRINITY_DN16245_c0_g1_i5	Glyma.07G007700.12	100	180	7	1444	4059	2595	1	0	3675

TRINITY_DN15867_c0_g3_i2	Glyma.15G172400.7	97.01492537	347	17	189	2783	1	2601	0	2665

TRINITY_DN15867_c0_g3_i2	Glyma.15G172400.2	98.5915493	347	17	189	2783	1	2601	0	2665

TRINITY_DN15867_c0_g3_i2	Glyma.15G172400.3	95.45454545	347	17	189	2783	1	2601	0	2665

TRINITY_DN15867_c0_g3_i2	Glyma.15G172400.6	100	347	17	189	2783	1	2601	0	2665

TRINITY_DN15867_c0_g3_i2	Glyma.15G172400.1	95.09803922	347	17	189	2783	1	2601	0	2665

TRINITY_DN15867_c0_g3_i3	Glyma.15G172400.7	100	347	17	189	2783	1	2601	0	2665

TRINITY_DN15867_c0_g3_i3	Glyma.15G172400.2	99.33333333	347	17	189	2783	1	2601	0	2665

TRINITY_DN15867_c0_g3_i3	Glyma.15G172400.3	96.11650485	347	17	189	2783	1	2601	0	2665

TRINITY_DN15867_c0_g3_i3	Glyma.15G172400.6	97.56097561	347	17	189	2783	1	2601	0	2665

TRINITY_DN15867_c0_g3_i3	Glyma.15G172400.1	95.3038674	347	17	189	2783	1	2601	0	2665

TRINITY_DN16311_c0_g1_i5	Glyma.13G146900.2	96.55172414	293	16	12	2613	138	2733	0	2977

TRINITY_DN16311_c0_g1_i7	Glyma.13G146900.2	96.62447257	264	9	12	2613	138	2733	0	3138

TRINITY_DN14622_c0_g1_i3	Glyma.10G179700.3	96.06481481	204	17	67	2672	3	2598	0	3496

TRINITY_DN14622_c0_g1_i6	Glyma.10G179700.1	98.97959184	201	20	78	2685	97	2679	0	3441

TRINITY_DN14622_c0_g1_i6	Glyma.10G179700.2	96.59763314	201	20	78	2685	88	2670	0	3441

TRINITY_DN12309_c0_g1_i3	Glyma.10G012600.2	100	180	3	507	3122	2610	1	0	3784

TRINITY_DN12309_c0_g1_i2	Glyma.10G012600.2	96.60493827	179	4	507	3110	2610	1	0	3712

TRINITY_DN9717_c0_g1_i1	Glyma.08G244900.4	100	167	3	436	3051	2601	1	0	3797

TRINITY_DN14949_c0_g1_i2	Glyma.03G224400.2	96.2962963	143	3	424	3036	1	2616	0	4008

TRINITY_DN14949_c0_g1_i2	Glyma.03G224400.1	96.2962963	143	3	424	3036	1	2616	0	4008

TRINITY_DN14949_c0_g1_i2	Glyma.19G221300.2	96.2962963	130	4	424	3036	1	2616	0	4080

TRINITY_DN14949_c0_g1_i2	Glyma.19G221300.1	96.2962963	130	4	424	3036	1	2616	0	4080

TRINITY_DN13046_c0_g3_i1	Glyma.15G165500.1	98	239	12	914	3508	2613	1	0	3352

TRINITY_DN16450_c0_g1_i1	Glyma.02G198000.1	100	223	28	172	2724	2604	1	0	3110

TRINITY_DN11676_c0_g2_i1	Glyma.03G193000.1	100	218	3	503	3114	1	2615	0	3591

TRINITY_DN11676_c0_g2_i1	Glyma.03G193000.2	97.95918367	218	3	503	3114	1	2615	0	3591

TRINITY_DN11676_c0_g2_i1	Glyma.03G193000.3	95.62043796	218	3	503	3114	1	2615	0	3591

TRINITY_DN2792_c0_g1_i1	Glyma.06G131100.1	100	153	0	136	2751	1	2616	0	3984

TRINITY_DN16274_c0_g1_i2	Glyma.U010500.1	97.26027397	133	5	696	3296	209	2824	0	3997

TRINITY_DN15314_c0_g2_i2	Glyma.12G188600.1	97.09677419	459	67	697	3235	2859	293	0	1485

TRINITY_DN13751_c0_g1_i2	Glyma.13G122200.1	96	293	20	543	3132	1	2605	0	2979

TRINITY_DN13751_c0_g1_i2	Glyma.13G122200.2	97.23502304	293	20	543	3132	1	2605	0	2979

TRINITY_DN11254_c0_g1_i1	Glyma.08G189500.1	98.85057471	393	36	142	2729	2603	7	0	2372

TRINITY_DN15867_c0_g3_i2	Glyma.09G066400.1	100	350	24	189	2783	1	2580	0	2545

TRINITY_DN15867_c0_g3_i3	Glyma.09G066400.1	99.10313901	350	24	189	2783	1	2580	0	2545

TRINITY_DN11681_c0_g1_i1	Glyma.04G055700.1	97.18309859	300	12	316	2910	10	2619	0	3016

TRINITY_DN16311_c0_g1_i2	Glyma.06G182100.3	95	200	4	93	2702	127	2739	0	3687

TRINITY_DN16311_c0_g1_i2	Glyma.06G182100.2	95	200	4	93	2702	127	2739	0	3687

TRINITY_DN16311_c0_g1_i2	Glyma.06G182100.1	95	200	4	93	2702	127	2739	0	3687

TRINITY_DN10369_c0_g1_i1	Glyma.17G026900.1	98.61111111	187	4	85	2683	4	2614	0	3675

TRINITY_DN14492_c1_g1_i4	Glyma.19G192800.1	96.55172414	183	6	243	2848	1	2613	0	3755

TRINITY_DN2236_c0_g1_i1	Glyma.02G276200.1	97.09821429	372	37	410	2984	2614	13	0	2447

TRINITY_DN14836_c0_g1_i1	Glyma.13G034600.3	98.98989899	317	28	6	2565	1456	4033	0	2501

TRINITY_DN14836_c0_g1_i1	Glyma.13G034600.2	100	317	28	6	2565	1456	4033	0	2501

TRINITY_DN14836_c0_g1_i1	Glyma.13G034600.1	96	317	28	6	2565	1456	4033	0	2501

TRINITY_DN14836_c0_g1_i2	Glyma.13G034600.3	100	317	28	6	2565	1456	4033	0	2501

TRINITY_DN14836_c0_g1_i2	Glyma.13G034600.2	95.04950495	317	28	6	2565	1456	4033	0	2501

TRINITY_DN14836_c0_g1_i2	Glyma.13G034600.1	100	317	28	6	2565	1456	4033	0	2501

TRINITY_DN14836_c0_g1_i4	Glyma.13G034600.3	97.68637532	317	28	6	2565	1456	4033	0	2501

TRINITY_DN14836_c0_g1_i4	Glyma.13G034600.2	96.05055292	317	28	6	2565	1456	4033	0	2501

TRINITY_DN14836_c0_g1_i4	Glyma.13G034600.1	100	317	28	6	2565	1456	4033	0	2501

TRINITY_DN14836_c0_g1_i7	Glyma.13G034600.3	100	317	28	6	2565	1456	4033	0	2501

TRINITY_DN14836_c0_g1_i7	Glyma.13G034600.2	100	317	28	6	2565	1456	4033	0	2501

TRINITY_DN14836_c0_g1_i7	Glyma.13G034600.1	100	317	28	6	2565	1456	4033	0	2501

TRINITY_DN14836_c0_g1_i9	Glyma.13G034600.1	100	317	28	6	2565	1456	4033	0	2501

TRINITY_DN14836_c0_g1_i9	Glyma.13G034600.3	99.02912621	317	28	6	2565	1456	4033	0	2501

TRINITY_DN14836_c0_g1_i9	Glyma.13G034600.2	97.61904762	317	28	6	2565	1456	4033	0	2501

TRINITY_DN16311_c0_g1_i2	Glyma.13G146900.2	95	195	10	93	2702	139	2733	0	3609

TRINITY_DN14492_c1_g1_i2	Glyma.03G192300.1	97.1875	189	1	243	2852	1	2613	0	3759

TRINITY_DN16038_c0_g1_i1	Glyma.10G266600.1	96.55172414	188	6	231	2827	8	2619	0	3674

TRINITY_DN12653_c0_g1_i1	Glyma.08G025600.1	97.05882353	183	0	817	3429	2613	1	0	3812

TRINITY_DN14415_c0_g1_i2	Glyma.16G154400.4	96.3963964	183	13	240	2846	1	2589	0	3618

TRINITY_DN14415_c0_g1_i2	Glyma.16G154400.3	97.40871614	183	13	240	2846	1	2589	0	3618

TRINITY_DN14415_c0_g1_i2	Glyma.16G154400.1	100	183	13	240	2846	1	2589	0	3618

TRINITY_DN14415_c0_g1_i2	Glyma.16G154400.2	97.18309859	183	13	240	2846	1	2589	0	3618

TRINITY_DN14415_c0_g1_i2	Glyma.16G154400.5	97.18309859	183	13	240	2846	1	2589	0	3618

TRINITY_DN14415_c0_g1_i3	Glyma.16G154400.4	99.19224556	183	13	240	2846	1	2589	0	3618

TRINITY_DN14415_c0_g1_i3	Glyma.16G154400.3	95.89041096	183	13	240	2846	1	2589	0	3618

TRINITY_DN14415_c0_g1_i3	Glyma.16G154400.1	98.61111111	183	13	240	2846	1	2589	0	3618

TRINITY_DN14415_c0_g1_i3	Glyma.16G154400.2	97.5308642	183	13	240	2846	1	2589	0	3618

TRINITY_DN14415_c0_g1_i3	Glyma.16G154400.5	98.54545455	183	13	240	2846	1	2589	0	3618

TRINITY_DN13629_c0_g2_i1	Glyma.05G143100.2	96.42857143	182	5	333	2945	2603	1	0	3753

TRINITY_DN13963_c0_g1_i1	Glyma.04G053300.3	100	177	0	127	2739	1	2613	0	3845

TRINITY_DN13963_c0_g1_i1	Glyma.04G053300.2	96.90721649	177	0	127	2739	1	2613	0	3845

TRINITY_DN12653_c0_g1_i1	Glyma.05G219300.1	97.04797048	176	0	817	3429	2613	1	0	3851

TRINITY_DN16311_c0_g1_i1	Glyma.06G182100.3	95	169	2	93	2702	127	2739	0	3869

TRINITY_DN16311_c0_g1_i1	Glyma.06G182100.2	95	169	2	93	2702	127	2739	0	3869

TRINITY_DN16311_c0_g1_i1	Glyma.06G182100.1	95	169	2	93	2702	127	2739	0	3869

TRINITY_DN10035_c0_g1_i1	Glyma.15G142600.1	100	168	9	1	2592	805	3414	0	3740

TRINITY_DN10035_c0_g1_i1	Glyma.15G142600.3	100	168	9	1	2592	760	3369	0	3740

TRINITY_DN10035_c0_g1_i1	Glyma.15G142600.2	100	168	9	1	2592	754	3363	0	3740

TRINITY_DN16311_c0_g1_i1	Glyma.13G146900.2	95	166	3	93	2702	139	2733	0	3770

TRINITY_DN14778_c0_g1_i3	Glyma.14G035400.1	96.875	153	2	514	3123	1	2604	0	3901

TRINITY_DN14778_c0_g1_i3	Glyma.02G279300.1	96.875	145	1	514	3123	1	2613	0	4002

TRINITY_DN15919_c0_g1_i2	Glyma.08G007700.1	98.23529412	401	14	548	3135	2850	248	0	2388

TRINITY_DN15919_c0_g1_i2	Glyma.08G007700.2	96.1038961	398	19	548	3135	2838	248	0	2327

TRINITY_DN15919_c0_g1_i3	Glyma.08G007700.1	95.15151515	335	11	548	3141	2850	248	0	2793

TRINITY_DN15919_c0_g1_i3	Glyma.08G007700.2	100	332	16	548	3141	2838	248	0	2732

TRINITY_DN16175_c0_g1_i3	Glyma.01G197800.1	98.68421053	252	4	1	2610	428	3037	0	3402

TRINITY_DN16175_c0_g1_i7	Glyma.01G197800.1	97.84946237	252	4	1	2610	428	3037	0	3402

TRINITY_DN9858_c0_g1_i1	Glyma.04G221900.1	97.63779528	122	1	309	2917	1	2612	0	4128

TRINITY_DN15223_c0_g1_i1	Glyma.05G234900.1	97.84946237	267	21	42	2637	1	2593	0	3129

TRINITY_DN16175_c0_g1_i3	Glyma.11G043800.1	98.36956522	264	2	1	2610	428	3037	0	3347

TRINITY_DN16175_c0_g1_i7	Glyma.11G043800.1	97.84946237	264	2	1	2610	428	3037	0	3347

TRINITY_DN36201_c0_g1_i1	Glyma.07G150100.1	97.95918367	220	8	228	2829	24	2628	0	3506

TRINITY_DN36201_c0_g1_i1	Glyma.07G150100.3	100	220	8	228	2829	24	2628	0	3506

TRINITY_DN36201_c0_g1_i1	Glyma.07G150100.2	99.15966387	220	8	228	2829	24	2628	0	3506

TRINITY_DN14622_c0_g1_i4	Glyma.10G179700.5	95.83333333	195	23	102	2703	1	2559	0	3347

TRINITY_DN14622_c0_g1_i4	Glyma.10G179700.4	99.41176471	195	23	102	2703	1	2559	0	3347

TRINITY_DN10369_c0_g1_i1	Glyma.07G247100.1	98.5915493	166	4	85	2683	4	2611	0	3805

TRINITY_DN10369_c0_g1_i1	Glyma.07G247100.3	98.61111111	163	8	85	2683	4	2590	0	3687

TRINITY_DN14622_c0_g1_i4	Glyma.20G210700.1	100	173	22	102	2701	1	2557	0	3454

TRINITY_DN14622_c0_g1_i4	Glyma.20G210700.2	100	173	22	102	2701	1	2557	0	3454

TRINITY_DN14778_c0_g1_i3	Glyma.14G035400.2	96.86098655	153	1	514	3123	1	2601	0	3914

TRINITY_DN14778_c0_g1_i3	Glyma.02G279300.2	95.23809524	145	0	514	3123	1	2610	0	4017

TRINITY_DN16499_c0_g1_i1	Glyma.16G095500.1	97.62931034	145	1	530	3139	1	2607	0	3997

TRINITY_DN12627_c0_g1_i2	Glyma.12G183500.4	95.01246883	135	4	313	2919	1	2601	0	3995

TRINITY_DN12627_c0_g1_i2	Glyma.12G183500.3	100	133	7	313	2919	1	2589	0	3928

TRINITY_DN13016_c0_g1_i1	Glyma.12G010300.3	98.14814815	132	8	571	3168	2598	1	0	3934

TRINITY_DN10934_c0_g2_i2	Glyma.19G245800.1	96.2406015	286	27	387	2973	7	2599	0	2988

TRINITY_DN9858_c0_g1_i1	Glyma.06G143500.2	95.22292994	122	0	309	2917	1	2609	0	4143

TRINITY_DN9858_c0_g1_i1	Glyma.06G143500.1	95.22357724	122	0	309	2917	1	2609	0	4143

TRINITY_DN9858_c0_g1_i1	Glyma.06G143500.3	95.2238806	122	0	309	2917	1	2609	0	4143

TRINITY_DN9858_c0_g1_i1	Glyma.06G143500.4	100	122	0	309	2917	1	2609	0	4143

TRINITY_DN13751_c0_g1_i2	Glyma.10G035800.1	99.45945946	287	7	543	3132	1	2590	0	3016

TRINITY_DN14051_c0_g1_i1	Glyma.18G209600.1	100	169	0	627	3232	2728	123	0	3877

TRINITY_DN9549_c0_g1_i1	Glyma.11G081500.1	96.15384615	236	13	113	2695	1	2601	0	3336

TRINITY_DN14670_c0_g1_i2	Glyma.13G065500.1	95.28301887	226	9	530	3128	243	2838	0	3461

TRINITY_DN14670_c0_g1_i2	Glyma.13G065500.7	98.21428571	226	10	530	3128	243	2829	0	3404

TRINITY_DN13411_c0_g1_i1	Glyma.06G098300.7	100	189	5	3103	5701	5502	2899	0	3718

TRINITY_DN13411_c0_g1_i1	Glyma.06G098300.6	96.60056657	189	5	3103	5701	5484	2881	0	3718

TRINITY_DN8701_c0_g1_i1	Glyma.17G015200.3	98.1981982	177	3	325	2926	3879	1275	0	3810

TRINITY_DN13512_c0_g1_i1	Glyma.06G101600.5	96.18644068	172	0	529	3133	2605	1	0	3858

TRINITY_DN13512_c0_g1_i3	Glyma.06G101600.5	98.64864865	169	1	529	3113	2605	1	0	3746

TRINITY_DN11254_c0_g1_i1	Glyma.07G034800.1	98.85245902	495	41	142	2720	2597	22	0	1724

TRINITY_DN5189_c0_g1_i1	Glyma.20G022900.1	95.91836735	373	26	386	2959	310	2901	0	2471

TRINITY_DN13968_c0_g3_i2	Glyma.15G070100.3	96.40591966	352	40	1437	4001	1104	3680	0	2433

TRINITY_DN14622_c0_g1_i3	Glyma.10G179700.1	96.06557377	202	16	78	2672	97	2679	0	3496

TRINITY_DN14622_c0_g1_i3	Glyma.10G179700.2	96.06557377	202	16	78	2672	88	2670	0	3496

TRINITY_DN16274_c0_g1_i2	Glyma.U010500.2	99.39393939	135	1	696	3296	209	2812	0	4041

TRINITY_DN15305_c1_g2_i1	Glyma.12G074100.6	98.73417722	184	12	218	2802	2934	336	0	3646

TRINITY_DN15305_c1_g2_i1	Glyma.12G074100.5	98.73417722	184	12	218	2802	2934	336	0	3646

TRINITY_DN15305_c1_g2_i1	Glyma.12G074100.3	98.73873874	184	12	218	2802	2934	336	0	3646

TRINITY_DN15305_c1_g2_i1	Glyma.12G074100.1	98.7394958	184	12	218	2802	2934	336	0	3646

TRINITY_DN15305_c1_g2_i1	Glyma.12G074100.2	96.79245283	184	12	218	2802	2934	336	0	3646

TRINITY_DN15305_c1_g2_i1	Glyma.12G074100.4	100	184	12	218	2802	2934	336	0	3646

TRINITY_DN15888_c0_g3_i2	Glyma.02G269200.1	98.75	364	19	76	2668	537	3117	0	2595

TRINITY_DN14670_c0_g1_i2	Glyma.13G065500.10	95.70917759	236	10	765	3360	1	2590	0	3382

TRINITY_DN14670_c0_g1_i2	Glyma.13G065500.8	98.02955665	236	10	765	3360	1	2590	0	3382

TRINITY_DN14670_c0_g1_i2	Glyma.13G065500.13	98.24561404	234	12	765	3360	1	2587	0	3373

TRINITY_DN2658_c0_g1_i1	Glyma.07G232200.7	96.61538462	191	28	243	2802	2580	1	0	3334

TRINITY_DN2658_c0_g1_i1	Glyma.07G232200.1	96.62650602	191	28	243	2802	2580	1	0	3334

TRINITY_DN2658_c0_g1_i1	Glyma.07G232200.3	96.62921348	191	28	243	2802	2580	1	0	3334

TRINITY_DN2658_c0_g1_i1	Glyma.07G232200.4	96.62162162	191	28	243	2802	2580	1	0	3334

TRINITY_DN7273_c0_g1_i1	Glyma.10G211600.1	97	178	6	318	2910	2784	189	0	3722

TRINITY_DN7273_c0_g1_i1	Glyma.10G211600.2	95.4787234	178	6	318	2910	2784	189	0	3722

TRINITY_DN7273_c0_g1_i2	Glyma.10G211600.1	100	175	12	318	2899	2784	189	0	3668

TRINITY_DN7273_c0_g1_i2	Glyma.10G211600.2	100	175	12	318	2899	2784	189	0	3668

TRINITY_DN318_c0_g1_i1	Glyma.10G159500.3	98.58156028	155	9	1	2599	1770	4350	0	3792

TRINITY_DN13968_c0_g3_i4	Glyma.15G070100.3	100	447	37	1437	4004	1104	3680	0	1958

TRINITY_DN12697_c0_g1_i1	Glyma.11G009200.1	97.34219269	247	10	446	3034	2586	1	0	3260

TRINITY_DN12697_c0_g1_i2	Glyma.11G009200.1	97.33333333	239	10	325	2913	2586	1	0	3304

TRINITY_DN15314_c0_g2_i1	Glyma.13G313000.1	98.71794872	437	71	118	2663	2829	293	0	1624

TRINITY_DN13209_c1_g1_i2	Glyma.15G214500.1	99.00744417	426	35	695	3254	2644	73	0	2002

TRINITY_DN6780_c0_g1_i2	Glyma.15G166700.4	96.90721649	194	8	300	2882	2598	1	0	3603

TRINITY_DN8850_c0_g1_i1	Glyma.10G000200.1	99.36708861	174	10	279	2865	255	2850	0	3727

TRINITY_DN15888_c0_g3_i1	Glyma.14G047900.1	100	340	36	499	3081	525	3087	0	2580

TRINITY_DN12617_c0_g1_i1	Glyma.09G063200.5	96.52173913	247	21	1946	4538	1203	3765	0	3160

TRINITY_DN12617_c0_g1_i1	Glyma.09G063200.2	96.53401797	247	21	1946	4538	804	3366	0	3160

TRINITY_DN5257_c0_g2_i1	Glyma.14G074700.1	96.96969697	243	9	601	3189	2586	1	0	3304

TRINITY_DN12617_c0_g1_i1	Glyma.09G063200.4	98.4962406	243	27	1946	4538	1203	3741	0	3027

TRINITY_DN15466_c0_g1_i2	Glyma.04G203300.1	99.54954955	194	4	434	3025	244	2841	0	3674

TRINITY_DN16131_c0_g2_i1	Glyma.19G170700.1	100	354	5	1	2596	1955	4548	0	2798

TRINITY_DN15428_c0_g1_i2	Glyma.13G264100.1	95.92391304	205	10	892	3471	523	3117	0	3526

TRINITY_DN16431_c0_g1_i4	Glyma.03G034900.2	98.57819905	430	37	2706	5251	2600	19	0	1988

TRINITY_DN16431_c0_g1_i5	Glyma.03G034900.2	95	430	37	2686	5231	2600	19	0	1988

TRINITY_DN16431_c0_g1_i6	Glyma.03G034900.2	95.74468085	430	37	2349	4894	2600	19	0	1988

TRINITY_DN16431_c0_g1_i7	Glyma.03G034900.2	98.85057471	430	37	2329	4874	2600	19	0	1988

TRINITY_DN14639_c0_g2_i1	Glyma.13G359500.1	98.4496124	416	27	143	2692	1	2592	0	2156

TRINITY_DN5950_c0_g1_i1	Glyma.13G271100.1	100	221	9	528	3097	3945	1352	0	3378

TRINITY_DN5950_c0_g1_i1	Glyma.13G271100.6	100	221	9	528	3097	3945	1352	0	3378

TRINITY_DN16245_c0_g1_i4	Glyma.07G007700.11	100	212	5	1362	3953	2595	1	0	3576

TRINITY_DN16245_c0_g1_i4	Glyma.07G007700.12	100	212	5	1362	3953	2595	1	0	3576

TRINITY_DN5950_c0_g1_i1	Glyma.12G228700.7	100	203	9	528	3101	3936	1348	0	3470

TRINITY_DN5950_c0_g1_i1	Glyma.12G228700.8	100	203	9	528	3101	3936	1348	0	3470

TRINITY_DN5950_c0_g1_i1	Glyma.12G228700.1	100	203	9	528	3101	3936	1348	0	3470

TRINITY_DN5950_c0_g1_i1	Glyma.12G228700.5	100	203	9	528	3101	3936	1348	0	3470

TRINITY_DN5950_c0_g1_i1	Glyma.12G228700.2	100	203	9	528	3101	3936	1348	0	3470

TRINITY_DN5950_c0_g1_i1	Glyma.12G228700.6	100	203	9	528	3101	3936	1348	0	3470

TRINITY_DN5950_c0_g1_i1	Glyma.12G228700.9	100	203	9	528	3101	3582	994	0	3470

TRINITY_DN5950_c0_g1_i1	Glyma.12G228700.3	100	203	9	528	3101	3582	994	0	3470

TRINITY_DN5950_c0_g1_i1	Glyma.12G228700.4	100	203	9	528	3101	3582	994	0	3470

TRINITY_DN16245_c0_g1_i2	Glyma.07G007700.11	95.87628866	181	4	1444	4038	2595	1	0	3768

TRINITY_DN16245_c0_g1_i2	Glyma.07G007700.12	98.937583	181	4	1444	4038	2595	1	0	3768

TRINITY_DN8850_c0_g1_i1	Glyma.02G001000.1	99.29078014	179	8	279	2865	207	2787	0	3637

TRINITY_DN15754_c0_g1_i3	Glyma.08G178900.2	97.76119403	342	24	304	2893	3114	573	0	2512

TRINITY_DN12878_c0_g1_i1	Glyma.18G025500.1	100	220	14	33	2627	1	2574	0	3426

TRINITY_DN9336_c0_g1_i1	Glyma.05G217800.1	97.95918367	209	15	1	2572	1338	3924	0	3422

TRINITY_DN9336_c0_g1_i1	Glyma.05G217800.3	100	209	15	1	2572	1338	3924	0	3422

TRINITY_DN9336_c0_g1_i1	Glyma.05G217800.4	100	209	15	1	2572	1338	3924	0	3422

TRINITY_DN9336_c0_g1_i1	Glyma.05G217800.6	95.43147208	209	15	1	2572	1338	3924	0	3422

TRINITY_DN9336_c0_g1_i1	Glyma.05G217800.2	100	209	15	1	2572	1326	3912	0	3422

TRINITY_DN9336_c0_g1_i1	Glyma.05G217800.5	100	209	15	1	2572	1131	3717	0	3422

TRINITY_DN15499_c0_g1_i1	Glyma.13G235000.1	96.61016949	452	25	5	2578	420	3002	0	2074

TRINITY_DN15499_c0_g1_i4	Glyma.13G235000.1	98.24561404	428	25	5	2578	420	3002	0	2207

TRINITY_DN15399_c0_g1_i3	Glyma.18G221600.1	98.33333333	277	16	92	2679	77	2659	0	3134

TRINITY_DN12697_c0_g1_i1	Glyma.01G233600.2	97.32824427	239	10	446	3034	2580	1	0	3332

TRINITY_DN12697_c0_g1_i1	Glyma.01G233600.1	97.34513274	239	10	446	3034	2580	1	0	3332

TRINITY_DN12697_c0_g1_i2	Glyma.01G233600.2	97.33333333	231	10	325	2913	2580	1	0	3376

TRINITY_DN12697_c0_g1_i2	Glyma.01G233600.1	97.34042553	231	10	325	2913	2580	1	0	3376

TRINITY_DN15399_c0_g1_i2	Glyma.18G221600.1	98.31932773	218	16	92	2679	77	2659	0	3461

TRINITY_DN15074_c0_g1_i1	Glyma.20G081500.1	96.51741294	172	10	459	3011	2595	1	0	3568

TRINITY_DN15981_c0_g1_i6	Glyma.01G193600.2	96.18320611	162	1	772	3363	2826	232	0	3875

TRINITY_DN7878_c0_g1_i1	Glyma.09G209900.1	99.03381643	149	6	380	2971	2586	1	0	3890

TRINITY_DN7878_c0_g3_i1	Glyma.09G209900.1	99.02912621	149	6	380	2971	2586	1	0	3890

TRINITY_DN15836_c0_g2_i2	Glyma.11G167200.1	95.703125	405	16	271	2854	205	2788	0	2418

TRINITY_DN11254_c0_g1_i1	Glyma.07G007000.1	98.85057471	299	9	142	2729	2594	7	0	3057

TRINITY_DN15399_c0_g1_i3	Glyma.09G268300.1	98.33333333	297	4	94	2684	304	2895	0	3112

TRINITY_DN15399_c0_g1_i2	Glyma.09G268300.1	98.31932773	232	4	94	2684	304	2895	0	3472

TRINITY_DN5950_c0_g1_i1	Glyma.13G271100.4	100	222	7	528	3097	3942	1352	0	3386

TRINITY_DN5950_c0_g1_i1	Glyma.13G271100.5	100	222	7	528	3097	3942	1352	0	3386

TRINITY_DN15399_c0_g1_i4	Glyma.09G268300.1	98.34254144	215	6	94	2681	304	2895	0	3548

TRINITY_DN15399_c0_g1_i1	Glyma.09G268300.1	98.37662338	150	5	94	2681	304	2895	0	3908

TRINITY_DN16080_c0_g3_i1	Glyma.20G052500.1	95.09202454	460	23	340	2919	265	2844	0	2073

TRINITY_DN16080_c0_g3_i1	Glyma.20G052500.3	95.8677686	460	23	340	2919	169	2748	0	2073

TRINITY_DN16080_c0_g3_i1	Glyma.20G052500.2	96.82539683	460	23	340	2919	169	2748	0	2073

TRINITY_DN5257_c0_g2_i1	Glyma.17G250800.1	98.63013699	240	15	601	3189	2565	1	0	3253

TRINITY_DN5257_c0_g2_i1	Glyma.17G250800.2	100	240	15	601	3189	2565	1	0	3253

TRINITY_DN14639_c0_g2_i2	Glyma.15G014400.1	100	200	10	253	2829	1	2592	0	3570

TRINITY_DN14622_c0_g1_i6	Glyma.10G179700.5	98.97959184	196	20	102	2685	1	2559	0	3424

TRINITY_DN14622_c0_g1_i6	Glyma.10G179700.4	97.24576271	196	20	102	2685	1	2559	0	3424

TRINITY_DN15074_c0_g1_i1	Glyma.10G130800.1	96.16161616	177	16	459	3011	2592	1	0	3542

TRINITY_DN15458_c0_g1_i2	Glyma.15G121500.2	95.49019608	172	0	403	2995	1	2593	0	3836

TRINITY_DN666_c0_g1_i1	Glyma.15G136900.1	100	165	3	1	2569	843	3432	0	3699

TRINITY_DN666_c0_g1_i1	Glyma.15G136900.2	100	165	3	1	2569	426	3015	0	3699

TRINITY_DN7878_c0_g1_i1	Glyma.01G011200.3	99.04761905	144	8	380	2971	2583	1	0	3919

TRINITY_DN7878_c0_g1_i1	Glyma.01G011200.1	99.04534606	144	8	380	2971	2583	1	0	3919

TRINITY_DN7878_c0_g1_i1	Glyma.01G011200.2	99.02912621	144	8	380	2971	2583	1	0	3919

TRINITY_DN7878_c0_g3_i1	Glyma.01G011200.3	99.0430622	144	8	380	2971	2583	1	0	3919

TRINITY_DN7878_c0_g3_i1	Glyma.01G011200.1	99.04761905	144	8	380	2971	2583	1	0	3919

TRINITY_DN7878_c0_g3_i1	Glyma.01G011200.2	99.03846154	144	8	380	2971	2583	1	0	3919

TRINITY_DN15836_c0_g2_i2	Glyma.18G059700.1	95.27896996	410	14	269	2854	200	2779	0	2399

TRINITY_DN13968_c0_g3_i2	Glyma.15G070100.2	96.40591966	353	38	1437	4001	1104	3668	0	2483

TRINITY_DN16131_c0_g2_i1	Glyma.03G169500.2	98.7804878	330	9	1	2588	1955	4539	0	2887

TRINITY_DN16131_c0_g2_i1	Glyma.03G169500.1	100	330	9	1	2588	1955	4539	0	2887

TRINITY_DN15458_c0_g1_i1	Glyma.17G004700.1	98.19277108	312	22	3	2573	341	2920	0	2844

TRINITY_DN16311_c0_g1_i1	Glyma.17G089200.1	97.36842105	303	10	116	2695	117	2702	0	2992

TRINITY_DN16311_c0_g1_i5	Glyma.06G182100.3	96.55172414	291	8	37	2613	151	2739	0	3059

TRINITY_DN16311_c0_g1_i5	Glyma.06G182100.2	96.55172414	291	8	37	2613	151	2739	0	3059

TRINITY_DN16311_c0_g1_i5	Glyma.06G182100.1	96.55172414	291	8	37	2613	151	2739	0	3059

TRINITY_DN13968_c0_g3_i3	Glyma.15G070100.3	96.07843137	287	22	1437	3997	1104	3682	0	2913

TRINITY_DN5950_c0_g1_i1	Glyma.13G271100.2	100	225	10	528	3097	3933	1352	0	3334

TRINITY_DN5950_c0_g1_i1	Glyma.13G271100.3	100	225	10	528	3097	3933	1352	0	3334

TRINITY_DN15399_c0_g1_i4	Glyma.18G221600.1	100	208	10	92	2676	77	2659	0	3531

TRINITY_DN14639_c0_g2_i2	Glyma.13G359500.1	100	192	8	253	2829	1	2592	0	3626

TRINITY_DN14639_c0_g2_i2	Glyma.13G359500.2	100	191	14	253	2829	1	2577	0	3535

TRINITY_DN9112_c0_g1_i1	Glyma.19G101600.1	95.04643963	176	2	284	2872	2592	1	0	3792

TRINITY_DN14622_c0_g1_i6	Glyma.20G210700.1	98.73417722	174	19	102	2683	1	2557	0	3531

TRINITY_DN14622_c0_g1_i6	Glyma.20G210700.2	96.06060606	174	19	102	2683	1	2557	0	3531

TRINITY_DN666_c0_g1_i1	Glyma.09G031900.1	96.57320872	163	4	1	2568	843	3431	0	3709

TRINITY_DN666_c0_g1_i1	Glyma.09G031900.6	97.18309859	163	4	1	2568	843	3431	0	3709

TRINITY_DN666_c0_g1_i1	Glyma.09G031900.7	97.09543568	163	4	1	2568	843	3431	0	3709

TRINITY_DN666_c0_g1_i1	Glyma.09G031900.8	95.01039501	163	4	1	2568	843	3431	0	3709

TRINITY_DN666_c0_g1_i1	Glyma.09G031900.2	96.55172414	163	4	1	2568	426	3014	0	3709

TRINITY_DN666_c0_g1_i1	Glyma.09G031900.3	100	163	4	1	2568	426	3014	0	3709

TRINITY_DN666_c0_g1_i1	Glyma.09G031900.4	96.20535714	163	4	1	2568	426	3014	0	3709

TRINITY_DN666_c0_g1_i1	Glyma.09G031900.5	97.18309859	163	4	1	2568	426	3014	0	3709

TRINITY_DN9112_c0_g1_i1	Glyma.16G049600.1	100	162	2	284	2872	2592	1	0	3869

TRINITY_DN15399_c0_g1_i1	Glyma.18G221600.1	98.7654321	149	10	92	2676	77	2659	0	3858

TRINITY_DN16131_c0_g2_i1	Glyma.13G128700.1	100	141	0	3	2594	1960	4551	0	4006

TRINITY_DN16131_c0_g2_i1	Glyma.13G128700.2	100	141	0	3	2594	1762	4353	0	4006

TRINITY_DN13209_c1_g1_i2	Glyma.15G214500.6	100	426	35	695	3254	2635	73	0	2043

TRINITY_DN15235_c0_g1_i7	Glyma.10G227400.6	95	419	14	1	2582	500	3084	0	2366

TRINITY_DN15235_c0_g1_i7	Glyma.10G227400.4	99.40828402	419	14	1	2582	500	3084	0	2366

TRINITY_DN15235_c0_g1_i7	Glyma.10G227400.3	95	419	14	1	2582	500	3084	0	2366

TRINITY_DN15235_c0_g1_i7	Glyma.10G227400.1	97.5308642	419	14	1	2582	500	3084	0	2366

TRINITY_DN15235_c0_g1_i7	Glyma.10G227400.2	98.33333333	419	14	1	2582	500	3084	0	2366

TRINITY_DN15235_c0_g1_i7	Glyma.10G227400.5	98.30508475	419	14	1	2582	500	3084	0	2366

TRINITY_DN14639_c0_g2_i1	Glyma.13G359500.2	100	415	29	143	2692	1	2577	0	2130

TRINITY_DN12988_c0_g2_i1	Glyma.10G040500.1	100	355	11	1	2586	387	2969	0	2734

TRINITY_DN16311_c0_g1_i2	Glyma.17G089200.1	95	272	8	116	2695	117	2702	0	3175

TRINITY_DN16311_c0_g1_i7	Glyma.06G182100.3	96.61654135	260	6	37	2613	151	2739	0	3241

TRINITY_DN16311_c0_g1_i7	Glyma.06G182100.2	96.61835749	260	6	37	2613	151	2739	0	3241

TRINITY_DN16311_c0_g1_i7	Glyma.06G182100.1	96.62447257	260	6	37	2613	151	2739	0	3241

TRINITY_DN37092_c0_g1_i1	Glyma.18G052100.1	96.53916211	215	18	201	2766	2806	235	0	3310

TRINITY_DN37092_c0_g1_i1	Glyma.18G052100.5	100	215	18	201	2766	2806	235	0	3310

TRINITY_DN9073_c0_g2_i1	Glyma.01G014700.1	97.10144928	183	17	413	2945	78	2655	0	3312

TRINITY_DN11681_c0_g1_i2	Glyma.06G055800.1	98.48484848	235	15	298	2871	1	2574	0	3275

TRINITY_DN7977_c0_g1_i1	Glyma.15G177600.1	100	197	8	177	2753	2625	37	0	3602

TRINITY_DN9073_c0_g2_i1	Glyma.01G014700.2	97.11538462	183	17	414	2945	1	2577	0	3310

TRINITY_DN13968_c0_g3_i4	Glyma.15G070100.2	100	448	35	1437	4004	1104	3668	0	2008

TRINITY_DN13968_c0_g3_i1	Glyma.15G070100.3	100	382	19	1437	4000	1104	3682	0	2438

TRINITY_DN14981_c0_g2_i2	Glyma.02G033500.2	98.01980198	326	6	399	2984	2583	1	0	2916

TRINITY_DN14981_c0_g2_i2	Glyma.02G033500.1	98.02513465	326	6	399	2984	2583	1	0	2916

TRINITY_DN14981_c0_g2_i4	Glyma.02G033500.2	98.01980198	326	6	399	2984	2583	1	0	2916

TRINITY_DN14981_c0_g2_i4	Glyma.02G033500.1	98.03921569	326	6	399	2984	2583	1	0	2916

TRINITY_DN14981_c0_g2_i1	Glyma.08G288600.1	95.4787234	322	6	366	2951	2586	1	0	2959

TRINITY_DN14981_c0_g2_i1	Glyma.08G288600.2	95.45454545	322	6	366	2951	2586	1	0	2959

TRINITY_DN14981_c0_g2_i3	Glyma.08G288600.1	98.02955665	322	6	366	2951	2586	1	0	2959

TRINITY_DN14981_c0_g2_i3	Glyma.08G288600.2	98.03921569	322	6	366	2951	2586	1	0	2959

TRINITY_DN16311_c0_g1_i1	Glyma.17G089200.2	95	305	8	116	2695	117	2699	0	2994

TRINITY_DN15235_c0_g1_i7	Glyma.10G227300.5	96.3963964	283	8	1	2582	512	3096	0	3142

TRINITY_DN15235_c0_g1_i7	Glyma.10G227300.4	98.5915493	283	8	1	2582	512	3096	0	3142

TRINITY_DN15235_c0_g1_i7	Glyma.10G227300.3	98.5915493	283	8	1	2582	512	3096	0	3142

TRINITY_DN15235_c0_g1_i7	Glyma.10G227300.1	98.85057471	283	8	1	2582	512	3096	0	3142

TRINITY_DN15235_c0_g1_i7	Glyma.10G227300.2	97.91666667	283	8	1	2582	512	3096	0	3142

TRINITY_DN15458_c0_g1_i4	Glyma.17G004700.1	98.33333333	270	17	3	2573	341	2920	0	3110

TRINITY_DN16254_c0_g1_i5	Glyma.07G004300.1	96.0950764	229	11	1	2576	2588	4	0	3402

TRINITY_DN10035_c0_g1_i1	Glyma.09G037300.1	98.21428571	160	12	1	2567	805	3386	0	3707

TRINITY_DN10035_c0_g1_i1	Glyma.09G037300.2	95.74468085	160	12	1	2567	805	3386	0	3707

TRINITY_DN12591_c0_g1_i1	Glyma.16G084200.2	95.32258065	150	11	343	2922	2709	128	0	3847

TRINITY_DN12591_c0_g1_i1	Glyma.16G084200.3	98.61111111	150	11	343	2922	2709	128	0	3847

TRINITY_DN12591_c0_g1_i2	Glyma.16G084200.2	100	150	11	315	2894	2709	128	0	3847

TRINITY_DN12591_c0_g1_i2	Glyma.16G084200.3	100	150	11	315	2894	2709	128	0	3847

TRINITY_DN16054_c1_g1_i2	Glyma.10G168700.2	96.62162162	122	0	890	3478	2589	1	0	4106

TRINITY_DN16054_c1_g1_i2	Glyma.20G221000.2	98.18181818	119	0	890	3478	2589	1	0	4122

TRINITY_DN14981_c0_g2_i2	Glyma.01G031900.1	98.01980198	338	5	399	2984	2583	1	0	2861

TRINITY_DN14981_c0_g2_i4	Glyma.01G031900.1	98.01980198	338	5	399	2984	2583	1	0	2861

TRINITY_DN14981_c0_g2_i1	Glyma.18G136200.1	95.45454545	317	4	366	2951	2586	1	0	2998

TRINITY_DN14981_c0_g2_i1	Glyma.18G136200.2	95.47511312	317	4	366	2951	2586	1	0	2998

TRINITY_DN14981_c0_g2_i3	Glyma.18G136200.1	98.02631579	317	4	366	2951	2586	1	0	2998

TRINITY_DN14981_c0_g2_i3	Glyma.18G136200.2	98.02955665	317	4	366	2951	2586	1	0	2998

TRINITY_DN16311_c0_g1_i2	Glyma.17G089200.2	95	274	6	116	2695	117	2699	0	3177

TRINITY_DN11786_c1_g1_i1	Glyma.11G246000.1	100	249	2	1	2587	1174	3760	0	3387

TRINITY_DN11786_c1_g1_i2	Glyma.11G246000.1	97.06601467	249	2	1	2587	1174	3760	0	3387

TRINITY_DN9073_c0_g2_i1	Glyma.09G207400.1	97.10144928	187	18	414	2945	1	2577	0	3310

TRINITY_DN9073_c0_g2_i1	Glyma.01G014700.3	97.10467706	183	17	416	2945	9	2583	0	3306

TRINITY_DN2876_c0_g1_i1	Glyma.09G267100.1	100	148	6	1	2586	1890	4466	0	3875

TRINITY_DN14639_c0_g2_i2	Glyma.07G006300.1	96.55172414	404	16	253	2829	1	2574	0	2390

TRINITY_DN14981_c0_g2_i1	Glyma.01G031900.1	95.45454545	179	3	366	2951	2583	1	0	3753

TRINITY_DN14981_c0_g2_i3	Glyma.01G031900.1	98.02631579	179	3	366	2951	2583	1	0	3753

TRINITY_DN9046_c0_g1_i1	Glyma.13G160300.1	96.66666667	170	6	524	3103	2580	1	0	3746

TRINITY_DN9046_c0_g2_i1	Glyma.13G160300.1	96.66666667	170	6	429	3008	2580	1	0	3746

TRINITY_DN7482_c0_g1_i1	Glyma.13G027300.4	97.95221843	170	24	572	3151	2553	1	0	3570

TRINITY_DN7482_c0_g1_i1	Glyma.13G027300.1	95.45454545	170	24	572	3151	2553	1	0	3570

TRINITY_DN7482_c0_g1_i1	Glyma.13G027300.2	95.45454545	170	24	572	3151	2553	1	0	3570

TRINITY_DN14981_c0_g2_i1	Glyma.02G033500.2	95.46539379	167	3	366	2951	2583	1	0	3819

TRINITY_DN14981_c0_g2_i1	Glyma.02G033500.1	95.45454545	167	3	366	2951	2583	1	0	3819

TRINITY_DN14981_c0_g2_i3	Glyma.02G033500.2	98.03921569	167	3	366	2951	2583	1	0	3819

TRINITY_DN14981_c0_g2_i3	Glyma.02G033500.1	98.0349345	167	3	366	2951	2583	1	0	3819

TRINITY_DN14245_c0_g2_i2	Glyma.15G072000.1	95.65217391	361	21	1	2568	310	2877	0	2543

TRINITY_DN14245_c0_g2_i2	Glyma.13G241400.1	95.65217391	348	36	1	2555	310	2870	0	2486

TRINITY_DN14245_c0_g2_i2	Glyma.13G241400.9	95.65217391	348	36	1	2555	310	2870	0	2486

TRINITY_DN16254_c0_g1_i10	Glyma.07G004300.1	98.73417722	170	8	1	2573	2588	4	0	3744

TRINITY_DN14981_c0_g2_i2	Glyma.08G288600.1	95.47038328	135	0	399	2984	2586	1	0	4028

TRINITY_DN14981_c0_g2_i2	Glyma.08G288600.2	95.47169811	135	0	399	2984	2586	1	0	4028

TRINITY_DN14981_c0_g2_i4	Glyma.08G288600.1	98.01980198	135	0	399	2984	2586	1	0	4028

TRINITY_DN14981_c0_g2_i4	Glyma.08G288600.2	98.03921569	135	0	399	2984	2586	1	0	4028

TRINITY_DN16131_c0_g2_i1	Glyma.10G041700.1	96.52173913	134	0	3	2588	1960	4545	0	4034

TRINITY_DN16131_c0_g2_i1	Glyma.10G041700.3	96.51972158	134	0	3	2588	1762	4347	0	4034

TRINITY_DN16131_c0_g2_i1	Glyma.10G041700.2	96.51972158	134	0	3	2588	1762	4347	0	4034

TRINITY_DN16131_c0_g2_i1	Glyma.10G041700.4	99.18367347	134	0	3	2588	493	3078	0	4034

TRINITY_DN14981_c0_g2_i2	Glyma.18G136200.1	95.45454545	128	0	399	2984	2586	1	0	4067

TRINITY_DN14981_c0_g2_i2	Glyma.18G136200.2	95.45454545	128	0	399	2984	2586	1	0	4067

TRINITY_DN14981_c0_g2_i4	Glyma.18G136200.1	98.03921569	128	0	399	2984	2586	1	0	4067

TRINITY_DN14981_c0_g2_i4	Glyma.18G136200.2	98.02631579	128	0	399	2984	2586	1	0	4067

TRINITY_DN16054_c1_g1_i1	Glyma.10G168700.2	95.53314121	122	0	756	3341	2586	1	0	4100

TRINITY_DN16054_c1_g1_i2	Glyma.10G168700.1	100	122	0	893	3478	2586	1	0	4100

TRINITY_DN16054_c1_g1_i5	Glyma.10G168700.2	97.05882353	122	0	829	3414	2586	1	0	4100

TRINITY_DN16054_c1_g1_i1	Glyma.20G221000.2	95.53264605	119	0	756	3341	2586	1	0	4117

TRINITY_DN16054_c1_g1_i2	Glyma.20G221000.1	98.14814815	119	0	893	3478	2586	1	0	4117

TRINITY_DN16054_c1_g1_i5	Glyma.20G221000.2	97.87234043	119	0	829	3414	2586	1	0	4117

TRINITY_DN9073_c0_g2_i1	Glyma.09G207400.2	97.10144928	187	17	414	2945	1	2574	0	3325

TRINITY_DN15479_c0_g2_i2	Glyma.04G088800.1	100	359	21	758	3320	2572	1	0	2569

TRINITY_DN15973_c0_g1_i1	Glyma.05G172000.1	96.02649007	258	4	360	2939	2637	55	0	3310

TRINITY_DN15973_c0_g1_i2	Glyma.05G172000.1	96.13095238	258	4	535	3114	2637	55	0	3310

TRINITY_DN15973_c0_g1_i1	Glyma.05G171900.1	100	257	4	360	2939	2637	55	0	3315

TRINITY_DN15973_c0_g1_i2	Glyma.05G171900.1	98.85057471	257	4	535	3114	2637	55	0	3315

TRINITY_DN11409_c0_g1_i1	Glyma.10G120200.1	95.23809524	240	50	1030	3534	1375	3903	0	2577

TRINITY_DN11409_c0_g1_i2	Glyma.10G120200.1	95.45454545	240	50	1477	3981	1375	3903	0	2577

TRINITY_DN11409_c0_g1_i1	Glyma.10G120200.2	97.64011799	240	50	1030	3534	1366	3894	0	2577

TRINITY_DN11409_c0_g1_i2	Glyma.10G120200.2	98.77300613	240	50	1477	3981	1366	3894	0	2577

TRINITY_DN11606_c0_g1_i1	Glyma.17G123700.1	95.93345656	200	10	997	3571	2578	1	0	3567

TRINITY_DN13629_c0_g2_i2	Glyma.05G143100.2	95.65217391	177	5	283	2866	2574	1	0	3727

TRINITY_DN9046_c0_g1_i1	Glyma.17G111200.1	96.66666667	168	5	524	3103	2577	1	0	3770

TRINITY_DN9046_c0_g1_i1	Glyma.17G111200.5	96.66666667	168	5	524	3103	2577	1	0	3770

TRINITY_DN9046_c0_g2_i1	Glyma.17G111200.1	96.66666667	168	5	429	3008	2577	1	0	3770

TRINITY_DN9046_c0_g2_i1	Glyma.17G111200.5	96.66666667	168	5	429	3008	2577	1	0	3770

TRINITY_DN15981_c0_g1_i5	Glyma.11G048300.2	98.78787879	160	3	664	3241	2814	231	0	3847

TRINITY_DN11254_c0_g1_i1	Glyma.07G006900.1	98.85057471	289	16	142	2717	2579	25	0	2942

TRINITY_DN15981_c0_g1_i6	Glyma.11G048300.1	95.12195122	160	3	791	3367	2813	231	0	3845

TRINITY_DN16181_c0_g1_i4	Glyma.04G034200.1	99.55156951	140	1	312	2894	2580	1	0	3975

TRINITY_DN16181_c0_g1_i4	Glyma.06G034400.2	96.96969697	132	1	312	2894	2580	1	0	4019

TRINITY_DN15981_c0_g1_i6	Glyma.17G161300.1	95.12195122	404	10	791	3367	2807	231	0	2466

TRINITY_DN15479_c0_g2_i2	Glyma.06G090800.1	98.39572193	363	14	758	3320	2572	1	0	2569

TRINITY_DN9549_c0_g1_i1	Glyma.01G162100.1	96.15384615	220	13	127	2695	18	2595	0	3439

TRINITY_DN10262_c0_g2_i3	Glyma.08G295700.1	100	206	17	419	2972	2601	36	0	3343

TRINITY_DN9657_c0_g1_i1	Glyma.05G243200.1	96.72131148	191	17	206	2754	1	2567	0	3400

TRINITY_DN5134_c0_g1_i1	Glyma.16G097100.1	99.41176471	166	14	165	2738	531	3093	0	3674

TRINITY_DN16181_c0_g1_i4	Glyma.17G248400.3	95	303	6	319	2894	2579	1	0	3042

TRINITY_DN16181_c0_g1_i4	Glyma.17G248400.1	97.08029197	303	6	319	2894	2579	1	0	3042

TRINITY_DN16181_c0_g1_i4	Glyma.17G248400.4	97.08029197	303	9	319	2894	2567	1	0	2964

TRINITY_DN15934_c0_g1_i2	Glyma.10G212600.1	99.18367347	240	11	418	2984	1	2580	0	3339

TRINITY_DN15934_c0_g1_i2	Glyma.10G212600.2	97.77777778	240	12	418	2984	1	2577	0	3321

TRINITY_DN16345_c0_g1_i7	Glyma.08G044700.1	98.31223629	234	6	1145	3720	3068	496	0	3386

TRINITY_DN16345_c0_g1_i7	Glyma.08G044700.6	98.31932773	234	6	1145	3720	2789	217	0	3386

TRINITY_DN15934_c0_g1_i2	Glyma.20G178500.1	96.05568445	232	7	418	2984	1	2580	0	3384

TRINITY_DN15934_c0_g1_i2	Glyma.20G178500.2	100	232	8	418	2984	1	2577	0	3365

TRINITY_DN16345_c0_g1_i7	Glyma.09G182600.1	98.31932773	226	12	1145	3720	3080	511	0	3411

TRINITY_DN12309_c0_g1_i3	Glyma.10G012600.3	96.72131148	179	3	507	3086	2595	22	0	3723

TRINITY_DN12309_c0_g1_i2	Glyma.10G012600.3	99	178	4	507	3074	2595	22	0	3651

TRINITY_DN9046_c0_g1_i1	Glyma.17G111200.2	96.66666667	168	4	524	3103	2574	1	0	3784

TRINITY_DN9046_c0_g1_i1	Glyma.17G111200.3	96.66666667	168	4	524	3103	2574	1	0	3784

TRINITY_DN9046_c0_g1_i1	Glyma.17G111200.4	96.66666667	168	4	524	3103	2574	1	0	3784

TRINITY_DN9046_c0_g2_i1	Glyma.17G111200.2	96.66666667	168	4	429	3008	2574	1	0	3784

TRINITY_DN9046_c0_g2_i1	Glyma.17G111200.3	96.67519182	168	4	429	3008	2574	1	0	3784

TRINITY_DN9046_c0_g2_i1	Glyma.17G111200.4	96.66666667	168	4	429	3008	2574	1	0	3784

TRINITY_DN13055_c0_g1_i1	Glyma.13G212900.9	95.74468085	450	18	71	2629	53	2623	0	2078

TRINITY_DN13055_c0_g1_i1	Glyma.13G212900.1	95.74468085	450	18	71	2629	53	2623	0	2078

TRINITY_DN13055_c0_g1_i1	Glyma.13G212900.7	95.74468085	450	18	71	2629	53	2623	0	2078

TRINITY_DN16168_c0_g1_i8	Glyma.08G057200.4	96.42857143	320	16	524	3093	2571	11	0	2804

TRINITY_DN16181_c0_g1_i4	Glyma.14G077200.1	99.25373134	301	6	319	2894	2576	1	0	3046

TRINITY_DN16168_c0_g1_i5	Glyma.08G057200.4	98.65771812	301	16	620	3189	2571	11	0	2909

TRINITY_DN11681_c0_g1_i1	Glyma.04G055700.3	97.18309859	292	9	404	2968	26	2602	0	3031

TRINITY_DN9560_c0_g1_i2	Glyma.08G301200.1	100	291	5	482	3049	3695	1119	0	3055

TRINITY_DN13968_c0_g3_i3	Glyma.15G070100.2	96.07843137	288	20	1437	3997	1104	3670	0	2963

TRINITY_DN15568_c1_g1_i2	Glyma.12G214600.1	95.59748428	261	4	551	3112	1	2580	0	3203

TRINITY_DN16345_c0_g1_i7	Glyma.05G237500.1	98.30508475	231	3	1145	3720	3074	496	0	3452

TRINITY_DN16345_c0_g1_i7	Glyma.07G093700.2	98.30508475	231	5	1145	3720	3092	517	0	3434

TRINITY_DN16345_c0_g1_i7	Glyma.07G093700.1	98.30508475	231	5	1145	3720	3092	517	0	3434

TRINITY_DN6677_c0_g2_i1	Glyma.01G235200.1	95.18072289	205	4	232	2810	2573	1	0	3578

TRINITY_DN6677_c0_g2_i1	Glyma.01G235200.2	98.30985915	205	4	232	2810	2573	1	0	3578

TRINITY_DN6677_c0_g2_i1	Glyma.01G235200.3	97.84313725	205	4	232	2810	2573	1	0	3578

TRINITY_DN6677_c0_g2_i1	Glyma.01G235200.4	98.8372093	205	4	232	2810	2573	1	0	3578

TRINITY_DN6677_c0_g2_i2	Glyma.01G235200.1	98.8372093	205	4	232	2810	2573	1	0	3578

TRINITY_DN6677_c0_g2_i2	Glyma.01G235200.2	100	205	4	232	2810	2573	1	0	3578

TRINITY_DN6677_c0_g2_i2	Glyma.01G235200.3	100	205	4	232	2810	2573	1	0	3578

TRINITY_DN6677_c0_g2_i2	Glyma.01G235200.4	99.12280702	205	4	232	2810	2573	1	0	3578

TRINITY_DN12896_c0_g2_i1	Glyma.18G072400.3	95.83333333	202	4	244	2819	12	2589	0	3607

TRINITY_DN15639_c1_g1_i1	Glyma.12G073200.2	100	198	13	1119	3680	2903	330	0	3513

TRINITY_DN15639_c1_g1_i2	Glyma.12G073200.2	97.14285714	198	13	1243	3804	2903	330	0	3513

TRINITY_DN15639_c1_g1_i3	Glyma.12G073200.2	100	198	13	926	3487	2903	330	0	3513

TRINITY_DN14622_c0_g1_i3	Glyma.10G179700.5	96.06741573	197	16	102	2672	1	2559	0	3480

TRINITY_DN14622_c0_g1_i3	Glyma.10G179700.4	96.06741573	197	16	102	2672	1	2559	0	3480

TRINITY_DN14783_c0_g1_i1	Glyma.01G111300.2	96.96969697	172	3	354	2932	2577	1	0	3786

TRINITY_DN14783_c0_g1_i2	Glyma.01G111300.2	96.96969697	172	3	354	2932	2577	1	0	3786

TRINITY_DN3903_c0_g1_i1	Glyma.11G127700.1	96.25	419	41	729	3266	157	2709	0	2008

TRINITY_DN15981_c0_g1_i6	Glyma.05G105200.1	95.12195122	394	9	794	3367	2804	231	0	2516

TRINITY_DN15499_c0_g1_i4	Glyma.13G174900.1	99.53271028	319	5	6	2579	451	3027	0	2950

TRINITY_DN15568_c1_g1_i2	Glyma.13G287000.3	99.08256881	245	14	551	3115	1	2568	0	3243

TRINITY_DN15568_c1_g1_i2	Glyma.13G287000.1	99.08256881	245	14	551	3115	1	2568	0	3243

TRINITY_DN11681_c0_g1_i2	Glyma.04G055700.2	98.82352941	237	14	316	2874	10	2583	0	3288

TRINITY_DN11681_c0_g1_i2	Glyma.04G055700.1	99.76635514	237	14	316	2874	10	2583	0	3288

TRINITY_DN14912_c0_g1_i2	Glyma.08G296300.1	96.53465347	207	25	64	2611	1	2556	0	3267

TRINITY_DN14622_c0_g1_i3	Glyma.20G210700.1	97.84946237	174	18	102	2670	1	2557	0	3592

TRINITY_DN14622_c0_g1_i3	Glyma.20G210700.2	99.4047619	174	18	102	2670	1	2557	0	3592

TRINITY_DN15789_c0_g1_i1	Glyma.06G032600.1	95.85798817	549	50	1899	4455	2686	5218	0	1293

TRINITY_DN15789_c0_g1_i1	Glyma.06G032600.4	99.55357143	549	50	1899	4455	2626	5158	0	1293

TRINITY_DN15789_c0_g1_i1	Glyma.06G032600.2	100	549	50	1899	4455	2626	5158	0	1293

TRINITY_DN15789_c0_g1_i1	Glyma.06G032600.3	100	549	50	1899	4455	2410	4942	0	1293

TRINITY_DN14639_c0_g2_i2	Glyma.08G206900.1	100	403	13	253	2829	1	2559	0	2399

TRINITY_DN9560_c0_g1_i3	Glyma.08G301200.1	100	291	5	241	2806	3693	1119	0	3051

TRINITY_DN15499_c0_g1_i1	Glyma.13G174900.1	95.06398537	290	3	6	2579	451	3027	0	3121

TRINITY_DN16436_c0_g2_i11	Glyma.11G131600.1	98.41269841	251	10	181	2742	1	2571	0	3221

TRINITY_DN13959_c0_g1_i3	Glyma.10G292300.3	100	149	28	319	2842	2918	347	0	3548

TRINITY_DN13968_c0_g3_i1	Glyma.15G070100.2	100	383	17	1437	4000	1104	3670	0	2488

TRINITY_DN4785_c0_g1_i1	Glyma.07G196800.1	100	287	21	371	2921	2729	173	0	2872

TRINITY_DN15072_c0_g1_i4	Glyma.18G106900.1	96.66666667	237	8	685	3249	2574	1	0	3349

TRINITY_DN16168_c0_g1_i6	Glyma.08G057200.4	96.2962963	182	1	524	3100	2571	1	0	3712

TRINITY_DN16168_c0_g1_i2	Glyma.08G057200.4	96.82539683	163	1	620	3196	2571	1	0	3818

TRINITY_DN15981_c0_g1_i5	Glyma.01G193600.2	98.7854251	159	1	664	3237	2808	232	0	3858

TRINITY_DN497_c0_g1_i1	Glyma.05G029600.1	97.59450172	280	17	234	2770	3	2575	0	2935

TRINITY_DN3649_c0_g1_i1	Glyma.08G055900.1	97.07602339	235	11	195	2756	3132	562	0	3332

TRINITY_DN3649_c0_g2_i1	Glyma.08G055900.1	100	235	12	195	2753	3132	562	0	3314

TRINITY_DN12309_c0_g1_i3	Glyma.02G012100.2	95.215311	194	6	507	3080	2589	25	0	3598

TRINITY_DN12309_c0_g1_i2	Glyma.02G012100.2	98.58657244	191	7	507	3068	2589	25	0	3537

TRINITY_DN15981_c0_g1_i6	Glyma.01G193600.1	99.08256881	159	1	791	3363	2807	232	0	3856

TRINITY_DN12617_c0_g1_i1	Glyma.13G104300.1	98.4962406	362	15	1982	4538	1242	3813	0	2614

TRINITY_DN12617_c0_g1_i1	Glyma.13G104300.18	98.50746269	362	15	1982	4538	1236	3807	0	2614

TRINITY_DN12617_c0_g1_i1	Glyma.13G104300.17	98.4962406	362	15	1982	4538	1218	3789	0	2614

TRINITY_DN12617_c0_g1_i1	Glyma.13G104300.15	98.50746269	362	15	1982	4538	1116	3687	0	2614

TRINITY_DN15964_c0_g1_i7	Glyma.19G019200.2	100	362	17	255	2820	30	2595	0	2634

TRINITY_DN15040_c0_g1_i2	Glyma.13G099300.1	99.38271605	284	9	784	3337	2569	1	0	3007

TRINITY_DN15040_c0_g1_i2	Glyma.13G099300.3	99.00744417	284	9	785	3337	2568	1	0	3005

TRINITY_DN11141_c0_g1_i2	Glyma.06G221400.1	95.12195122	279	12	2	2566	7	2571	0	3092

TRINITY_DN15072_c0_g1_i4	Glyma.08G309400.1	96.66666667	259	7	685	3249	2571	1	0	3241

TRINITY_DN16436_c0_g2_i3	Glyma.11G131600.1	96.48241206	257	7	534	3092	1	2571	0	3214

TRINITY_DN14639_c0_g2_i1	Glyma.07G006300.1	98.86363636	224	11	143	2692	1	2574	0	3358

TRINITY_DN11141_c0_g1_i1	Glyma.06G221400.1	100	207	3	76	2643	1	2571	0	3548

TRINITY_DN15072_c0_g1_i1	Glyma.18G106900.1	95.95959596	168	2	685	3252	2574	1	0	3784

TRINITY_DN15314_c0_g2_i1	Glyma.12G188600.1	97.54060325	424	61	154	2663	2814	293	0	1666

TRINITY_DN12617_c0_g1_i1	Glyma.13G104300.6	98.5	363	17	1982	4538	1242	3804	0	2573

TRINITY_DN12617_c0_g1_i1	Glyma.13G104300.16	98.50746269	363	17	1982	4538	1218	3780	0	2573

TRINITY_DN12617_c0_g1_i1	Glyma.13G104300.14	98.4962406	363	17	1982	4538	1116	3678	0	2573

TRINITY_DN11141_c0_g1_i1	Glyma.02G203600.1	98.29545455	359	18	76	2638	1	2560	0	2614

TRINITY_DN15499_c0_g1_i4	Glyma.07G201600.1	98.01980198	314	7	13	2579	455	3024	0	2953

TRINITY_DN14312_c0_g1_i4	Glyma.11G012000.1	98.80952381	169	6	563	3135	2556	2	0	3699

TRINITY_DN14312_c0_g1_i4	Glyma.01G229200.1	98.80952381	161	6	563	3135	2559	2	0	3762

TRINITY_DN2876_c0_g1_i1	Glyma.18G223300.4	100	152	0	14	2586	1903	4475	0	3910

TRINITY_DN2876_c0_g1_i1	Glyma.18G223300.3	100	152	0	14	2586	1903	4475	0	3910

TRINITY_DN2876_c0_g1_i1	Glyma.18G223300.2	100	152	0	14	2586	1903	4475	0	3910

TRINITY_DN2876_c0_g1_i1	Glyma.18G223300.1	100	152	0	14	2586	1903	4475	0	3910

TRINITY_DN15964_c0_g1_i6	Glyma.19G019200.2	96.01328904	475	11	255	2820	30	2595	0	2041

TRINITY_DN14481_c0_g3_i1	Glyma.13G234600.2	96	387	19	533	3089	246	2808	0	2451

TRINITY_DN15964_c0_g1_i8	Glyma.19G019200.2	98.8372093	371	11	255	2820	30	2595	0	2617

TRINITY_DN15499_c0_g1_i1	Glyma.07G201600.1	100	288	5	13	2579	455	3024	0	3109

TRINITY_DN13941_c0_g1_i2	Glyma.12G008500.1	99.05660377	215	32	2	2533	460	2991	0	3042

TRINITY_DN13622_c0_g1_i1	Glyma.16G182200.2	96.94656489	212	2	401	2971	2571	1	0	3563

TRINITY_DN11843_c0_g1_i1	Glyma.19G217500.2	100	153	1	1149	3720	1	2569	0	3882

TRINITY_DN15964_c0_g1_i5	Glyma.19G019200.2	100	480	9	255	2820	30	2595	0	2025

TRINITY_DN15627_c0_g1_i2	Glyma.01G184500.1	96.15384615	326	24	132	2698	2339	4869	0	2658

TRINITY_DN15627_c0_g1_i2	Glyma.01G184500.2	97.52066116	326	24	132	2698	1043	3573	0	2658

TRINITY_DN14362_c0_g1_i2	Glyma.09G218500.1	100	226	22	60	2586	43	2601	0	3134

TRINITY_DN15252_c0_g1_i1	Glyma.17G143300.1	100	202	22	239	2758	2559	1	0	3221

TRINITY_DN15072_c0_g1_i1	Glyma.08G309400.1	96.92307692	183	1	685	3252	2571	1	0	3714

TRINITY_DN7705_c0_g1_i1	Glyma.11G054100.1	95.38461538	155	7	256	2824	25	2586	0	3818

TRINITY_DN9348_c0_g1_i1	Glyma.02G195500.1	95.96774194	152	4	237	2803	512	3078	0	3855

TRINITY_DN13959_c0_g1_i4	Glyma.10G292300.4	95.65217391	146	17	646	3188	2565	1	0	3720

TRINITY_DN16430_c0_g2_i3	Glyma.11G033000.2	97.64705882	291	16	46	2603	118	2675	0	2979

TRINITY_DN16430_c0_g2_i3	Glyma.11G033000.1	100	291	16	46	2603	118	2675	0	2979

TRINITY_DN16436_c0_g2_i3	Glyma.11G131700.1	95.94594595	239	0	523	3092	44	2613	0	3422

TRINITY_DN16436_c0_g2_i3	Glyma.11G131700.2	98.68421053	239	0	523	3092	23	2592	0	3422

TRINITY_DN16033_c0_g1_i2	Glyma.20G233900.2	98.7012987	219	2	378	2943	2569	1	0	3500

TRINITY_DN14245_c0_g2_i2	Glyma.13G181700.1	95.65217391	198	9	2	2568	326	2883	0	3552

TRINITY_DN14245_c0_g2_i2	Glyma.07G194800.1	95.65217391	193	7	2	2568	314	2871	0	3579

TRINITY_DN16155_c1_g1_i2	Glyma.11G145600.1	95.87628866	250	2	223	2782	2569	1	0	3301

TRINITY_DN16033_c0_g1_i1	Glyma.20G233900.2	98.37398374	248	1	378	2943	2569	1	0	3350

TRINITY_DN15040_c0_g1_i3	Glyma.13G099300.1	100	185	5	597	3144	2569	1	0	3583

TRINITY_DN10314_c0_g1_i1	Glyma.06G056300.1	100	180	9	212	2769	2567	1	0	3663

TRINITY_DN16155_c1_g1_i4	Glyma.11G145600.1	97.265625	170	2	223	2782	2569	1	0	3744

TRINITY_DN7705_c0_g1_i1	Glyma.01G188000.1	95.4954955	157	3	256	2824	25	2586	0	3829

TRINITY_DN15642_c0_g1_i1	Glyma.12G017600.1	99.6031746	315	7	1355	3921	869	3426	0	2926

TRINITY_DN15642_c0_g1_i1	Glyma.12G017600.2	97.61904762	315	7	1355	3921	869	3426	0	2926

TRINITY_DN15642_c0_g1_i2	Glyma.12G017600.1	97.59036145	315	7	1281	3847	869	3426	0	2926

TRINITY_DN15642_c0_g1_i2	Glyma.12G017600.2	100	315	7	1281	3847	869	3426	0	2926

TRINITY_DN15040_c0_g1_i3	Glyma.13G099300.3	99.4011976	185	5	598	3144	2568	1	0	3581

TRINITY_DN12892_c0_g4_i1	Glyma.11G103100.2	100	172	4	230	2794	34	2589	0	3692

TRINITY_DN12892_c0_g4_i1	Glyma.11G103100.3	100	172	4	230	2794	34	2589	0	3692

TRINITY_DN12892_c0_g4_i1	Glyma.11G102600.1	100	172	4	230	2794	34	2589	0	3692

TRINITY_DN12892_c0_g4_i1	Glyma.11G102600.2	100	172	4	230	2794	34	2589	0	3692

TRINITY_DN16155_c1_g1_i4	Glyma.12G174000.1	100	345	8	223	2782	2560	1	0	2739

TRINITY_DN15302_c0_g1_i3	Glyma.17G067800.1	96.03174603	295	16	271	2820	1	2565	0	2983

TRINITY_DN15302_c0_g1_i4	Glyma.17G067800.1	95.77464789	295	16	377	2926	1	2565	0	2983

TRINITY_DN16155_c1_g1_i2	Glyma.12G174000.1	95.87628866	271	8	223	2782	2560	1	0	3149

TRINITY_DN8598_c0_g1_i1	Glyma.13G032300.1	98.30508475	224	29	87	2600	1	2556	0	3086

TRINITY_DN12719_c0_g2_i1	Glyma.13G321300.52	96.96969697	172	29	1	2532	2517	1	0	3238

TRINITY_DN12719_c0_g2_i1	Glyma.13G321300.1	97.61904762	172	29	1	2532	2517	1	0	3238

TRINITY_DN12719_c0_g2_i1	Glyma.13G321300.20	99.10714286	172	29	1	2532	2517	1	0	3238

TRINITY_DN12719_c0_g2_i1	Glyma.13G321300.21	99.10714286	172	29	1	2532	2517	1	0	3238

TRINITY_DN12719_c0_g2_i3	Glyma.13G321300.52	95.93495935	172	29	1	2532	2517	1	0	3238

TRINITY_DN12719_c0_g2_i3	Glyma.13G321300.1	100	172	29	1	2532	2517	1	0	3238

TRINITY_DN12719_c0_g2_i3	Glyma.13G321300.20	98.61111111	172	29	1	2532	2517	1	0	3238

TRINITY_DN12719_c0_g2_i3	Glyma.13G321300.21	98.14814815	172	29	1	2532	2517	1	0	3238

TRINITY_DN15679_c0_g1_i3	Glyma.15G196800.1	97.86096257	169	4	59	2623	1	2562	0	3759

TRINITY_DN15679_c0_g1_i6	Glyma.15G196800.1	100	169	4	1348	3912	1	2562	0	3759

TRINITY_DN15642_c0_g1_i1	Glyma.01G232500.2	97.98657718	167	0	1355	3921	866	3432	0	3816

TRINITY_DN15642_c0_g1_i1	Glyma.01G232500.1	96.17486339	167	0	1355	3921	866	3432	0	3816

TRINITY_DN15642_c0_g1_i2	Glyma.01G232500.2	100	167	0	1281	3847	866	3432	0	3816

TRINITY_DN15642_c0_g1_i2	Glyma.01G232500.1	100	167	0	1281	3847	866	3432	0	3816

TRINITY_DN15642_c0_g1_i1	Glyma.11G010400.1	97.59036145	161	0	1355	3921	866	3432	0	3849

TRINITY_DN15642_c0_g1_i2	Glyma.11G010400.1	100	161	0	1281	3847	866	3432	0	3849

TRINITY_DN15627_c0_g1_i2	Glyma.17G176300.1	97.09193246	160	0	132	2698	2303	4869	0	3855

TRINITY_DN15627_c0_g1_i2	Glyma.05G090500.1	99.1416309	159	0	132	2698	2303	4869	0	3860

TRINITY_DN16297_c0_g1_i1	Glyma.01G232800.1	99.04761905	473	33	1019	3567	271	2798	0	1762

TRINITY_DN16297_c0_g1_i2	Glyma.01G232800.1	98.64253394	473	33	1019	3567	271	2798	0	1762

TRINITY_DN16155_c1_g1_i4	Glyma.13G325400.1	95.62043796	339	12	223	2782	2554	1	0	2745

TRINITY_DN16155_c1_g1_i4	Glyma.13G325400.2	100	338	13	223	2782	2551	1	0	2730

TRINITY_DN16155_c1_g1_i2	Glyma.13G325400.1	95.87628866	266	10	223	2782	2554	1	0	3149

TRINITY_DN16155_c1_g1_i2	Glyma.13G325400.2	96.63120567	265	11	223	2782	2551	1	0	3134

TRINITY_DN11409_c0_g1_i1	Glyma.20G070600.1	95.96541787	263	33	1030	3534	1360	3891	0	2667

TRINITY_DN11409_c0_g1_i2	Glyma.20G070600.1	95.85635359	263	33	1477	3981	1360	3891	0	2667

TRINITY_DN9396_c0_g1_i1	Glyma.15G034300.1	100	244	19	179	2713	2565	4	0	3160

TRINITY_DN16436_c0_g2_i11	Glyma.12G055900.1	96.80851064	221	3	178	2742	49	2610	0	3482

TRINITY_DN12617_c0_g1_i2	Glyma.09G063200.5	100	212	5	1946	4505	1203	3765	0	3506

TRINITY_DN12617_c0_g1_i2	Glyma.09G063200.2	100	212	5	1946	4505	804	3366	0	3506

TRINITY_DN12617_c0_g1_i2	Glyma.09G063200.4	98.18181818	208	11	1946	4505	1203	3741	0	3373

TRINITY_DN15570_c0_g1_i2	Glyma.10G105200.1	95	188	20	597	3116	1	2565	0	3393

TRINITY_DN9971_c0_g1_i1	Glyma.09G120200.1	100	167	2	62	2618	246	2808	0	3736

TRINITY_DN13055_c0_g1_i1	Glyma.13G213000.1	97.2166998	453	25	86	2629	11	2566	0	2034

TRINITY_DN16436_c0_g2_i11	Glyma.11G131700.1	95.62289562	242	1	178	2742	52	2613	0	3376

TRINITY_DN16436_c0_g2_i11	Glyma.11G131700.2	100	242	1	178	2742	31	2592	0	3376

TRINITY_DN16436_c0_g2_i11	Glyma.11G131700.3	96.80851064	242	1	178	2742	7	2568	0	3376

TRINITY_DN14365_c0_g1_i1	Glyma.02G311300.1	99.20634921	213	20	778	3322	36	2577	0	3278

TRINITY_DN2227_c0_g1_i1	Glyma.12G129300.1	100	177	7	240	2804	1	2547	0	3640

TRINITY_DN16811_c0_g1_i1	Glyma.20G229500.2	96.36363636	159	22	995	3520	2556	1	0	3546

TRINITY_DN14734_c0_g1_i1	Glyma.13G363900.1	100	149	0	228	2792	2586	22	0	3912

TRINITY_DN14734_c0_g1_i1	Glyma.15G010000.1	100	144	0	228	2792	2586	22	0	3940

TRINITY_DN12519_c0_g1_i2	Glyma.20G017000.2	96.49122807	118	3	161	2719	1	2565	0	4045

TRINITY_DN12519_c0_g1_i1	Glyma.20G017000.2	97.60479042	117	4	161	2703	1	2565	0	3947

TRINITY_DN7739_c0_g2_i1	Glyma.10G274700.2	98.05825243	367	22	312	2859	2550	3	0	2495

TRINITY_DN16436_c0_g2_i1	Glyma.11G131600.1	100	252	8	181	2729	1	2558	0	3203

TRINITY_DN16436_c0_g2_i10	Glyma.11G131600.1	95.83333333	245	8	181	2729	1	2558	0	3241

TRINITY_DN9396_c0_g1_i1	Glyma.13G340100.1	100	243	22	179	2713	2556	4	0	3131

TRINITY_DN6296_c0_g1_i1	Glyma.05G146500.1	100	228	6	121	2682	1	2559	0	3426

TRINITY_DN16436_c0_g2_i3	Glyma.12G055900.1	95.55555556	220	2	530	3092	48	2610	0	3504

TRINITY_DN15679_c0_g1_i3	Glyma.09G088700.1	97.86324786	163	6	59	2617	1	2556	0	3747

TRINITY_DN15679_c0_g1_i6	Glyma.09G088700.1	97.5	163	6	1348	3906	1	2556	0	3747

TRINITY_DN15016_c0_g1_i1	Glyma.09G137000.1	96.2962963	424	38	566	3093	245	2778	0	1971

TRINITY_DN8611_c0_g1_i1	Glyma.05G100000.1	99.13793103	377	50	146	2673	2517	17	0	2017

TRINITY_DN16286_c0_g1_i3	Glyma.10G234500.2	97.56097561	355	12	700	3255	2613	58	0	2676

TRINITY_DN11141_c0_g1_i2	Glyma.02G203600.1	95.12195122	306	7	1	2561	6	2560	0	2974

TRINITY_DN15652_c1_g3_i2	Glyma.20G144600.1	96	242	8	187	2745	22	2577	0	3321

TRINITY_DN15652_c1_g3_i2	Glyma.20G144600.3	100	242	8	187	2745	22	2577	0	3321

TRINITY_DN15652_c1_g3_i3	Glyma.20G144600.1	97.09677419	242	8	154	2712	22	2577	0	3321

TRINITY_DN15652_c1_g3_i3	Glyma.20G144600.3	96.50485437	242	8	154	2712	22	2577	0	3321

TRINITY_DN16436_c0_g2_i3	Glyma.11G131700.3	100	237	0	530	3092	6	2568	0	3421

TRINITY_DN11262_c0_g1_i1	Glyma.04G056400.1	100	229	7	443	2998	4575	2014	0	3413

TRINITY_DN11262_c0_g1_i1	Glyma.04G056400.4	98.65771812	229	7	443	2998	3675	1114	0	3413

TRINITY_DN16155_c1_g1_i2	Glyma.12G076100.1	97.22222222	226	14	223	2782	2545	1	0	3345

TRINITY_DN2227_c0_g1_i1	Glyma.06G276100.1	100	179	7	240	2802	1	2548	0	3644

TRINITY_DN16155_c1_g1_i4	Glyma.12G076100.1	100	152	14	223	2782	2545	1	0	3755

TRINITY_DN9348_c0_g1_i1	Glyma.02G195500.2	98.72340426	151	4	245	2803	1	2559	0	3845

TRINITY_DN16430_c0_g2_i3	Glyma.11G032900.3	97.82608696	282	9	46	2603	118	2669	0	3079

TRINITY_DN16430_c0_g2_i3	Glyma.11G032900.1	98.73417722	282	9	46	2603	118	2669	0	3079

TRINITY_DN16430_c0_g2_i3	Glyma.11G032900.2	98.73417722	282	9	46	2603	118	2669	0	3079

TRINITY_DN15252_c0_g1_i1	Glyma.17G143300.2	100	202	21	239	2758	2550	1	0	3264

TRINITY_DN9085_c0_g1_i2	Glyma.01G125800.1	95.91836735	201	10	187	2737	27	2580	0	3494

TRINITY_DN7201_c0_g1_i1	Glyma.08G222400.2	100	193	1	1030	3588	2562	1	0	3642

TRINITY_DN11262_c0_g1_i3	Glyma.04G056400.1	95.4954955	186	5	443	2995	4575	2014	0	3642

TRINITY_DN11262_c0_g1_i3	Glyma.04G056400.4	97.6744186	186	5	443	2995	3675	1114	0	3642

TRINITY_DN15016_c0_g1_i1	Glyma.16G182400.1	96.57142857	425	40	566	3093	242	2769	0	1949

TRINITY_DN16430_c0_g2_i3	Glyma.01G209200.1	98.73417722	306	2	46	2603	118	2678	0	3014

TRINITY_DN16026_c0_g2_i4	Glyma.04G009100.2	100	200	4	993	3552	631	3159	0	3408

TRINITY_DN16026_c0_g2_i1	Glyma.04G009100.2	96.55172414	172	4	1005	3564	631	3159	0	3563

TRINITY_DN10252_c0_g1_i1	Glyma.01G229400.2	98.30508475	131	9	169	2724	323	2875	0	3919

TRINITY_DN10252_c0_g1_i1	Glyma.01G229400.3	98.31460674	131	9	169	2724	323	2875	0	3919

TRINITY_DN10252_c0_g1_i1	Glyma.01G229400.1	98.31932773	131	9	169	2724	323	2875	0	3919

TRINITY_DN10252_c0_g1_i2	Glyma.01G229400.2	98.30508475	131	9	169	2724	323	2875	0	3919

TRINITY_DN10252_c0_g1_i2	Glyma.01G229400.3	98.30508475	131	9	169	2724	323	2875	0	3919

TRINITY_DN10252_c0_g1_i2	Glyma.01G229400.1	98.30508475	131	9	169	2724	323	2875	0	3919

TRINITY_DN10252_c0_g2_i1	Glyma.01G229400.2	97.83549784	131	9	169	2724	323	2875	0	3919

TRINITY_DN10252_c0_g2_i1	Glyma.01G229400.3	97.82608696	131	9	169	2724	323	2875	0	3919

TRINITY_DN10252_c0_g2_i1	Glyma.01G229400.1	97.84313725	131	9	169	2724	323	2875	0	3919

TRINITY_DN10252_c0_g2_i2	Glyma.01G229400.2	97.82608696	131	9	169	2724	323	2875	0	3919

TRINITY_DN10252_c0_g2_i2	Glyma.01G229400.3	97.83393502	131	9	169	2724	323	2875	0	3919

TRINITY_DN10252_c0_g2_i2	Glyma.01G229400.1	97.84656797	131	9	169	2724	323	2875	0	3919

TRINITY_DN10252_c0_g2_i3	Glyma.01G229400.2	97.84946237	131	9	169	2724	323	2875	0	3919

TRINITY_DN10252_c0_g2_i3	Glyma.01G229400.3	97.84172662	131	9	169	2724	323	2875	0	3919

TRINITY_DN10252_c0_g2_i3	Glyma.01G229400.1	97.84172662	131	9	169	2724	323	2875	0	3919

TRINITY_DN10252_c0_g2_i4	Glyma.01G229400.2	97.84482759	131	9	169	2724	323	2875	0	3919

TRINITY_DN10252_c0_g2_i4	Glyma.01G229400.3	97.83989835	131	9	169	2724	323	2875	0	3919

TRINITY_DN10252_c0_g2_i4	Glyma.01G229400.1	99.10714286	131	9	169	2724	323	2875	0	3919

TRINITY_DN11262_c0_g1_i3	Glyma.14G098300.1	100	383	11	445	2995	4555	1999	0	2529

TRINITY_DN11262_c0_g1_i3	Glyma.14G098300.2	100	383	11	445	2995	4156	1600	0	2529

TRINITY_DN16436_c0_g2_i7	Glyma.11G131600.1	100	258	5	534	3079	1	2558	0	3195

TRINITY_DN14639_c0_g2_i1	Glyma.08G206900.1	98.86363636	236	4	143	2692	1	2559	0	3349

TRINITY_DN9085_c0_g1_i2	Glyma.03G047100.1	95.93495935	229	11	187	2737	27	2580	0	3362

TRINITY_DN16082_c0_g1_i8	Glyma.09G023600.1	97.95918367	209	3	173	2713	2559	1	0	3441

TRINITY_DN14245_c0_g2_i3	Glyma.13G181700.4	95.65217391	186	10	446	2999	94	2635	0	3542

TRINITY_DN16435_c0_g2_i2	Glyma.05G165300.1	100	183	10	155	2696	1	2538	0	3456

TRINITY_DN16082_c0_g1_i1	Glyma.09G023600.1	97.24137931	175	3	173	2713	2559	1	0	3629

TRINITY_DN14492_c1_g1_i2	Glyma.19G192800.2	96.9924812	171	1	243	2799	1	2560	0	3760

TRINITY_DN16332_c0_g3_i2	Glyma.17G239500.5	100	137	2	1968	4524	2560	1	0	3949

TRINITY_DN16332_c0_g3_i2	Glyma.17G239500.1	95.85492228	137	2	1968	4524	2560	1	0	3949

TRINITY_DN11262_c0_g1_i1	Glyma.14G098300.1	100	348	6	445	2998	4555	1999	0	2752

TRINITY_DN11262_c0_g1_i1	Glyma.14G098300.2	100	348	6	445	2998	4156	1600	0	2752

TRINITY_DN16082_c0_g1_i11	Glyma.09G023600.1	95.23809524	267	1	173	2713	2559	1	0	3131

TRINITY_DN16082_c0_g1_i5	Glyma.09G023600.1	100	233	1	173	2713	2559	1	0	3319

TRINITY_DN3367_c0_g1_i2	Glyma.09G228600.1	98.27586207	205	17	48	2588	1	2553	0	3435

TRINITY_DN16131_c0_g2_i2	Glyma.09G282500.2	100	520	57	102	2624	177	2699	0	1391

TRINITY_DN16131_c0_g2_i2	Glyma.09G282500.1	100	520	57	102	2624	177	2699	0	1391

TRINITY_DN16131_c0_g2_i2	Glyma.09G282500.3	95.92760181	520	57	102	2624	24	2546	0	1391

TRINITY_DN16131_c0_g2_i2	Glyma.09G282500.4	97.58064516	520	57	102	2624	24	2546	0	1391

TRINITY_DN16131_c0_g2_i2	Glyma.09G282500.5	100	520	57	102	2624	24	2546	0	1391

TRINITY_DN14122_c0_g1_i2	Glyma.16G156200.1	98.4962406	334	19	224	2760	1	2552	0	2699

TRINITY_DN15345_c0_g1_i1	Glyma.13G308400.4	99.02439024	198	4	659	3210	1	2550	0	3537

TRINITY_DN15345_c0_g1_i1	Glyma.13G308400.3	99.609375	198	4	659	3210	1	2550	0	3537

TRINITY_DN15345_c0_g1_i2	Glyma.13G308400.4	100	198	4	465	3016	1	2550	0	3537

TRINITY_DN15345_c0_g1_i2	Glyma.13G308400.3	96.30434783	198	4	465	3016	1	2550	0	3537

TRINITY_DN16435_c0_g2_i2	Glyma.08G122700.1	96.85314685	175	23	155	2696	1	2514	0	3367

TRINITY_DN16076_c2_g3_i2	Glyma.05G082200.1	100	404	18	352	2899	2601	69	0	2270

TRINITY_DN16436_c0_g2_i7	Glyma.11G131700.1	100	251	0	523	3079	44	2600	0	3332

TRINITY_DN16436_c0_g2_i7	Glyma.11G131700.2	96.34146341	251	0	523	3079	23	2579	0	3332

TRINITY_DN5366_c0_g1_i1	Glyma.02G159100.2	100	192	9	1295	3844	2598	52	0	3548

TRINITY_DN5366_c0_g2_i1	Glyma.02G159100.2	100	192	9	767	3316	2598	52	0	3548

TRINITY_DN5366_c0_g1_i1	Glyma.02G159100.3	100	192	10	1295	3844	2595	52	0	3530

TRINITY_DN5366_c0_g2_i1	Glyma.02G159100.3	98.14814815	192	10	767	3316	2595	52	0	3530

TRINITY_DN16332_c0_g3_i2	Glyma.14G085200.1	100	142	0	1968	4524	2557	1	0	3936

TRINITY_DN16332_c0_g3_i2	Glyma.17G239500.2	97.5308642	137	0	1968	4524	2557	1	0	3964

TRINITY_DN16332_c0_g3_i2	Glyma.17G239500.3	97.5308642	137	0	1968	4524	2557	1	0	3964

TRINITY_DN16332_c0_g3_i2	Glyma.17G239500.4	100	137	0	1968	4524	2557	1	0	3964

TRINITY_DN3367_c0_g1_i2	Glyma.09G228600.2	96.50145773	205	15	48	2588	1	2550	0	3448

TRINITY_DN10252_c0_g1_i1	Glyma.11G012200.3	98.30508475	150	5	171	2724	335	2884	0	3838

TRINITY_DN10252_c0_g1_i1	Glyma.11G012200.1	98.30508475	150	5	171	2724	335	2884	0	3838

TRINITY_DN10252_c0_g1_i1	Glyma.11G012200.2	98.30508475	150	5	171	2724	335	2884	0	3838

TRINITY_DN10252_c0_g1_i1	Glyma.11G012200.4	98.30508475	150	5	171	2724	335	2884	0	3838

TRINITY_DN10252_c0_g1_i2	Glyma.11G012200.3	98.31932773	150	5	171	2724	335	2884	0	3838

TRINITY_DN10252_c0_g1_i2	Glyma.11G012200.1	98.31932773	150	5	171	2724	335	2884	0	3838

TRINITY_DN10252_c0_g1_i2	Glyma.11G012200.2	97.82608696	150	5	171	2724	335	2884	0	3838

TRINITY_DN10252_c0_g1_i2	Glyma.11G012200.4	97.84102061	150	5	171	2724	335	2884	0	3838

TRINITY_DN10252_c0_g2_i1	Glyma.11G012200.3	97.84946237	150	5	171	2724	335	2884	0	3838

TRINITY_DN10252_c0_g2_i1	Glyma.11G012200.1	97.84946237	150	5	171	2724	335	2884	0	3838

TRINITY_DN10252_c0_g2_i1	Glyma.11G012200.2	97.83549784	150	5	171	2724	335	2884	0	3838

TRINITY_DN10252_c0_g2_i1	Glyma.11G012200.4	97.84172662	150	5	171	2724	335	2884	0	3838

TRINITY_DN10252_c0_g2_i2	Glyma.11G012200.3	97.84560144	150	5	171	2724	335	2884	0	3838

TRINITY_DN10252_c0_g2_i2	Glyma.11G012200.1	97.84836066	150	5	171	2724	335	2884	0	3838

TRINITY_DN10252_c0_g2_i2	Glyma.11G012200.2	97.82945736	150	5	171	2724	335	2884	0	3838

TRINITY_DN10252_c0_g2_i2	Glyma.11G012200.4	97.84482759	150	5	171	2724	335	2884	0	3838

TRINITY_DN10252_c0_g2_i3	Glyma.11G012200.3	97.84946237	150	5	171	2724	335	2884	0	3838

TRINITY_DN10252_c0_g2_i3	Glyma.11G012200.1	97.84946237	150	5	171	2724	335	2884	0	3838

TRINITY_DN10252_c0_g2_i3	Glyma.11G012200.2	97.84946237	150	5	171	2724	335	2884	0	3838

TRINITY_DN10252_c0_g2_i3	Glyma.11G012200.4	97.82608696	150	5	171	2724	335	2884	0	3838

TRINITY_DN10252_c0_g2_i4	Glyma.11G012200.3	99.10714286	150	5	171	2724	335	2884	0	3838

TRINITY_DN10252_c0_g2_i4	Glyma.11G012200.1	99.0990991	150	5	171	2724	335	2884	0	3838

TRINITY_DN10252_c0_g2_i4	Glyma.11G012200.2	99.0990991	150	5	171	2724	335	2884	0	3838

TRINITY_DN10252_c0_g2_i4	Glyma.11G012200.4	99.0990991	150	5	171	2724	335	2884	0	3838

TRINITY_DN15888_c0_g3_i4	Glyma.14G047900.1	100	390	40	499	3038	525	3040	0	2209

TRINITY_DN16435_c0_g2_i2	Glyma.08G122700.2	100	175	22	155	2696	1	2511	0	3380

TRINITY_DN8781_c0_g1_i1	Glyma.09G082300.1	95.40229885	160	7	510	3056	2550	1	0	3747

TRINITY_DN5601_c0_g1_i1	Glyma.18G282300.5	96.33333333	246	12	8	2543	766	3295	0	3083

TRINITY_DN16285_c1_g1_i2	Glyma.05G178900.6	96.53061224	209	9	633	3179	1	2550	0	3487

TRINITY_DN14843_c0_g1_i1	Glyma.14G165000.1	98.52941176	444	19	170	2706	67	2606	0	2067

TRINITY_DN16076_c2_g3_i2	Glyma.05G082200.2	99.66555184	403	18	352	2895	2529	1	0	2268

TRINITY_DN16076_c2_g3_i2	Glyma.05G082200.3	99.66666667	403	18	352	2895	2529	1	0	2268

TRINITY_DN16332_c0_g3_i2	Glyma.06G042400.1	100	276	1	1968	4520	2551	2	0	3166

TRINITY_DN16332_c0_g3_i2	Glyma.04G041300.1	100	270	1	1968	4520	2551	2	0	3199

TRINITY_DN16436_c0_g2_i1	Glyma.12G055900.1	97.92746114	232	3	178	2729	49	2597	0	3397

TRINITY_DN16436_c0_g2_i10	Glyma.12G055900.1	99.50248756	230	3	178	2729	49	2597	0	3408

TRINITY_DN13055_c0_g1_i1	Glyma.17G021400.1	98.98989899	225	1	86	2635	74	2626	0	3448

TRINITY_DN14162_c0_g1_i4	Glyma.10G198600.1	97.85714286	152	2	369	2921	2550	1	0	3853

TRINITY_DN14162_c0_g1_i3	Glyma.10G198600.1	97.64705882	150	5	369	2909	2550	1	0	3786

TRINITY_DN16360_c0_g8_i3	Glyma.16G131800.1	99.35064935	146	9	197	2746	1	2547	0	3847

TRINITY_DN14162_c0_g1_i4	Glyma.20G191400.1	97.36842105	140	4	369	2921	2547	1	0	3901

TRINITY_DN14162_c0_g1_i4	Glyma.20G191400.2	97.85714286	139	8	369	2921	2535	1	0	3829

TRINITY_DN14162_c0_g1_i3	Glyma.20G191400.1	98.37962963	138	8	369	2909	2547	1	0	3834

TRINITY_DN15888_c0_g3_i5	Glyma.02G269200.1	96.26865672	436	17	76	2618	537	3070	0	2122

TRINITY_DN16436_c0_g2_i1	Glyma.11G131700.1	99.609375	254	1	178	2729	52	2600	0	3286

TRINITY_DN16436_c0_g2_i1	Glyma.11G131700.2	98.50746269	254	1	178	2729	31	2579	0	3286

TRINITY_DN16436_c0_g2_i1	Glyma.11G131700.3	97.2972973	254	1	178	2729	7	2555	0	3286

TRINITY_DN12719_c0_g3_i1	Glyma.11G191900.1	96	254	11	1	2532	2550	1	0	3164

TRINITY_DN16436_c0_g2_i10	Glyma.11G131700.1	96.37426901	248	1	178	2729	52	2600	0	3319

TRINITY_DN16436_c0_g2_i10	Glyma.11G131700.2	96.2962963	248	1	178	2729	31	2579	0	3319

TRINITY_DN16436_c0_g2_i10	Glyma.11G131700.3	96.5	248	1	178	2729	7	2555	0	3319

TRINITY_DN4758_c0_g1_i1	Glyma.09G198200.3	96.59863946	202	1	355	2906	2549	1	0	3574

TRINITY_DN15384_c0_g1_i2	Glyma.04G240900.1	98.97959184	158	5	2	2552	541	3082	0	3766

TRINITY_DN15384_c0_g1_i2	Glyma.06G122500.1	96.03174603	151	5	2	2552	508	3049	0	3805

TRINITY_DN15384_c0_g1_i2	Glyma.06G122500.2	96.72131148	151	5	2	2552	118	2659	0	3805

TRINITY_DN16285_c1_g1_i1	Glyma.05G178900.6	97.14285714	148	5	633	3179	1	2550	0	3847

TRINITY_DN12783_c0_g1_i1	Glyma.06G202500.3	97.00598802	107	0	1695	4246	4872	2321	0	4120

TRINITY_DN15655_c2_g1_i3	Glyma.02G239600.1	98.19277108	298	18	130	2658	1	2544	0	2872

TRINITY_DN15484_c0_g1_i1	Glyma.17G105100.1	98.36065574	249	34	4013	6505	3717	1210	0	2678

TRINITY_DN16345_c0_g1_i7	Glyma.08G044700.5	98.31460674	232	6	1145	3690	3026	484	0	3341

TRINITY_DN16436_c0_g2_i7	Glyma.12G055900.1	96.34146341	231	2	530	3079	48	2597	0	3419

TRINITY_DN11262_c0_g1_i1	Glyma.06G056500.1	100	214	4	452	2998	4560	2014	0	3474

TRINITY_DN15421_c1_g3_i2	Glyma.15G210100.8	99.55947137	172	0	557	3107	2748	198	0	3759

TRINITY_DN15421_c1_g3_i2	Glyma.15G210100.7	99.56140351	172	0	557	3107	2748	198	0	3759

TRINITY_DN15421_c1_g3_i2	Glyma.15G210100.6	100	172	0	557	3107	2748	198	0	3759

TRINITY_DN15421_c1_g3_i2	Glyma.15G210100.5	96.17021277	172	0	557	3107	2748	198	0	3759

TRINITY_DN15421_c1_g3_i2	Glyma.15G210100.1	96.17021277	172	0	557	3107	2748	198	0	3759

TRINITY_DN15421_c1_g3_i2	Glyma.15G210100.2	97.4025974	172	0	557	3107	2748	198	0	3759

TRINITY_DN15421_c1_g3_i2	Glyma.15G210100.3	97.4025974	172	0	557	3107	2748	198	0	3759

TRINITY_DN15421_c1_g3_i2	Glyma.15G210100.4	97.4025974	172	0	557	3107	2748	198	0	3759

TRINITY_DN4063_c0_g1_i1	Glyma.12G040000.1	95.23809524	392	10	1	2528	2594	46	0	2388

TRINITY_DN16360_c0_g8_i3	Glyma.01G170200.1	99.35483871	310	14	197	2726	1	2549	0	2856

TRINITY_DN16360_c0_g8_i1	Glyma.01G170200.1	100	287	8	159	2697	1	2549	0	3042

TRINITY_DN12719_c0_g3_i1	Glyma.12G082400.2	96	260	13	1	2532	2532	1	0	3037

TRINITY_DN12719_c0_g3_i1	Glyma.12G082400.1	96	260	13	1	2532	2532	1	0	3037

TRINITY_DN16436_c0_g2_i7	Glyma.11G131700.3	96.66666667	249	0	530	3079	6	2555	0	3330

TRINITY_DN2761_c0_g1_i1	Glyma.04G020400.1	100	231	6	150	2696	1	2544	0	3371

TRINITY_DN16329_c1_g1_i2	Glyma.12G170100.1	98.33333333	194	0	514	3063	2550	1	0	3635

TRINITY_DN11623_c0_g1_i1	Glyma.11G105500.1	100	193	2	1261	3804	1550	4099	0	3602

TRINITY_DN16329_c1_g1_i2	Glyma.08G188000.1	98.33333333	178	0	514	3063	2550	1	0	3723

TRINITY_DN16360_c0_g8_i3	Glyma.02G051700.1	99.36305732	168	2	197	2746	1	2547	0	3759

TRINITY_DN15808_c0_g1_i1	Glyma.17G077400.1	96.75324675	392	48	129	2604	1	2533	0	1960

TRINITY_DN15808_c0_g1_i1	Glyma.17G077400.2	95.23809524	392	48	129	2604	1	2533	0	1960

TRINITY_DN15808_c0_g1_i4	Glyma.17G077400.2	100	392	48	129	2604	1	2533	0	1960

TRINITY_DN15808_c0_g1_i4	Glyma.17G077400.1	95.45454545	392	48	129	2604	1	2533	0	1960

TRINITY_DN15808_c0_g1_i6	Glyma.17G077400.2	100	392	48	129	2604	1	2533	0	1960

TRINITY_DN15808_c0_g1_i6	Glyma.17G077400.1	100	392	48	129	2604	1	2533	0	1960

TRINITY_DN16360_c0_g8_i1	Glyma.11G073100.1	100	280	9	159	2695	1	2546	0	3059

TRINITY_DN16254_c0_g1_i4	Glyma.09G061200.4	97.54098361	207	2	995	3540	2768	223	0	3522

TRINITY_DN16254_c0_g1_i4	Glyma.09G061200.3	96.46924829	207	2	995	3540	2768	223	0	3522

TRINITY_DN16254_c0_g1_i4	Glyma.09G061200.1	100	207	2	995	3540	2768	223	0	3522

TRINITY_DN16254_c0_g1_i4	Glyma.09G061200.2	96.5	207	2	995	3540	2768	223	0	3522

TRINITY_DN16254_c0_g1_i4	Glyma.09G061200.5	95.12195122	207	2	995	3540	2768	223	0	3522

TRINITY_DN3367_c0_g1_i2	Glyma.12G007900.1	100	199	12	48	2588	1	2538	0	3482

TRINITY_DN14487_c0_g1_i1	Glyma.07G172000.1	98.66666667	184	1	533	3078	1	2549	0	3668

TRINITY_DN9717_c0_g1_i1	Glyma.08G244900.5	100	165	3	504	3051	2533	1	0	3683

TRINITY_DN9717_c0_g1_i1	Glyma.08G244900.6	95.83333333	165	3	504	3051	2533	1	0	3683

TRINITY_DN9717_c0_g1_i1	Glyma.08G244900.7	100	165	3	504	3051	2533	1	0	3683

TRINITY_DN9717_c0_g1_i1	Glyma.08G244900.8	100	165	3	504	3051	2533	1	0	3683

TRINITY_DN9717_c0_g1_i1	Glyma.08G244900.9	100	165	3	504	3051	2533	1	0	3683

TRINITY_DN9717_c0_g1_i1	Glyma.08G244900.10	100	165	3	504	3051	2533	1	0	3683

TRINITY_DN9717_c0_g1_i1	Glyma.08G244900.11	100	165	3	504	3051	2533	1	0	3683

TRINITY_DN16426_c2_g1_i2	Glyma.13G152600.1	96	239	2	278	2823	1	2546	0	3356

TRINITY_DN11603_c0_g1_i1	Glyma.19G187900.1	100	203	14	208	2727	1	2535	0	3315

TRINITY_DN16033_c0_g1_i3	Glyma.20G233900.2	99.43181818	267	8	173	2713	2541	1	0	3147

TRINITY_DN2761_c0_g1_i1	Glyma.06G020500.1	100	251	3	150	2695	1	2543	0	3280

TRINITY_DN14675_c0_g1_i2	Glyma.16G217300.1	95.08547009	239	2	896	3439	2821	278	0	3341

TRINITY_DN14216_c0_g1_i1	Glyma.19G159400.1	100	226	2	267	2807	302	2847	0	3406

TRINITY_DN14216_c0_g1_i1	Glyma.19G159400.9	100	226	2	267	2807	176	2721	0	3406

TRINITY_DN5432_c0_g1_i1	Glyma.16G000500.1	100	208	5	3	2546	1	2544	0	3513

TRINITY_DN11262_c0_g1_i3	Glyma.06G056500.1	97.6744186	178	1	452	2995	4560	2014	0	3698

TRINITY_DN14610_c0_g1_i1	Glyma.16G217200.2	96.57142857	164	3	398	2930	2547	1	0	3705

TRINITY_DN16033_c0_g1_i4	Glyma.20G233900.2	98.96907216	302	7	173	2713	2541	1	0	2964

TRINITY_DN11603_c0_g1_i2	Glyma.19G187900.1	100	294	18	208	2724	1	2532	0	2795

TRINITY_DN16426_c2_g1_i2	Glyma.18G044600.1	97.26027397	222	0	278	2823	1	2546	0	3472

TRINITY_DN16426_c2_g1_i2	Glyma.13G152700.1	97.26027397	221	0	278	2823	1	2546	0	3478

TRINITY_DN16426_c2_g1_i2	Glyma.18G044500.1	100	219	3	278	2823	1	2537	0	3430

TRINITY_DN16426_c2_g1_i2	Glyma.18G044500.2	100	219	3	278	2823	1	2537	0	3430

TRINITY_DN16254_c0_g1_i4	Glyma.15G167500.1	98.68421053	214	1	995	3540	2756	214	0	3496

TRINITY_DN16254_c0_g1_i4	Glyma.15G167500.3	98.98989899	214	1	995	3540	2756	214	0	3496

TRINITY_DN16254_c0_g1_i4	Glyma.15G167500.2	96.79487179	214	1	995	3540	2756	214	0	3496

TRINITY_DN16254_c0_g1_i4	Glyma.15G167500.4	98.7654321	214	1	995	3540	2756	214	0	3496

TRINITY_DN9156_c0_g1_i1	Glyma.18G109800.1	99.31972789	205	3	63	2607	1	2532	0	3469

TRINITY_DN11893_c0_g1_i1	Glyma.02G200700.10	98	163	4	104	2643	1	2546	0	3760

TRINITY_DN11893_c0_g1_i1	Glyma.02G200700.2	98.01324503	163	4	104	2643	1	2546	0	3760

TRINITY_DN8611_c0_g1_i1	Glyma.17G166200.1	97.00598802	303	15	146	2669	2532	12	0	2730

TRINITY_DN15252_c0_g1_i1	Glyma.05G061500.1	96.05263158	209	15	239	2758	2538	1	0	3336

TRINITY_DN9156_c0_g1_i1	Glyma.08G307900.1	99.24528302	200	1	63	2607	1	2532	0	3507

TRINITY_DN15815_c0_g2_i3	Glyma.12G219400.1	96.62288931	238	8	1489	4023	3117	577	0	3302

TRINITY_DN16060_c0_g1_i1	Glyma.17G087600.2	98.39572193	192	15	2856	5363	2511	1	0	3188

TRINITY_DN16060_c0_g1_i1	Glyma.17G087600.3	98.68421053	192	15	2856	5363	2511	1	0	3188

TRINITY_DN16060_c0_g1_i1	Glyma.17G087600.1	98	192	15	2856	5363	2511	1	0	3188

TRINITY_DN16060_c0_g1_i2	Glyma.17G087600.2	95.45454545	192	15	2842	5349	2511	1	0	3188

TRINITY_DN16060_c0_g1_i2	Glyma.17G087600.3	96.51741294	192	15	2842	5349	2511	1	0	3188

TRINITY_DN16060_c0_g1_i2	Glyma.17G087600.1	99.3902439	192	15	2842	5349	2511	1	0	3188

TRINITY_DN16060_c0_g1_i3	Glyma.17G087600.2	95.79646018	192	15	2935	5442	2511	1	0	3188

TRINITY_DN16060_c0_g1_i3	Glyma.17G087600.3	96.62921348	192	15	2935	5442	2511	1	0	3188

TRINITY_DN16060_c0_g1_i3	Glyma.17G087600.1	95.88235294	192	15	2935	5442	2511	1	0	3188

TRINITY_DN15062_c0_g1_i2	Glyma.11G075100.5	96.1038961	190	33	375	2885	2514	1	0	3238

TRINITY_DN15655_c2_g1_i2	Glyma.02G239600.1	98.28767123	161	8	130	2655	1	2544	0	3690

TRINITY_DN16086_c0_g2_i5	Glyma.15G207900.1	99.0990991	578	42	116	2635	23	2541	0	1190

TRINITY_DN16222_c0_g1_i8	Glyma.03G206400.1	97.13114754	190	8	168	2706	1	2524	0	3494

TRINITY_DN16222_c0_g1_i5	Glyma.03G206400.1	95.52941176	189	8	155	2693	1	2524	0	3500

TRINITY_DN12719_c0_g2_i1	Glyma.13G321300.51	96.86192469	176	20	1	2532	2493	1	0	3326

TRINITY_DN12719_c0_g2_i1	Glyma.13G321300.48	96.86346863	176	20	1	2532	2493	1	0	3326

TRINITY_DN12719_c0_g2_i1	Glyma.13G321300.18	96.13259669	176	20	1	2532	2493	1	0	3326

TRINITY_DN12719_c0_g2_i1	Glyma.13G321300.19	95	176	20	1	2532	2493	1	0	3326

TRINITY_DN12719_c0_g2_i3	Glyma.13G321300.51	98.14385151	176	20	1	2532	2493	1	0	3326

TRINITY_DN12719_c0_g2_i3	Glyma.13G321300.48	98.1443299	176	20	1	2532	2493	1	0	3326

TRINITY_DN12719_c0_g2_i3	Glyma.13G321300.18	97.41100324	176	20	1	2532	2493	1	0	3326

TRINITY_DN12719_c0_g2_i3	Glyma.13G321300.19	97.41176471	176	20	1	2532	2493	1	0	3326

TRINITY_DN14219_c0_g2_i1	Glyma.10G270800.1	96.19834711	138	2	683	3224	1	2542	0	3919

TRINITY_DN14219_c0_g2_i2	Glyma.10G270800.1	96.19047619	138	2	320	2861	1	2542	0	3919

TRINITY_DN14219_c0_g2_i3	Glyma.10G270800.1	97.59036145	138	2	205	2746	1	2542	0	3919

TRINITY_DN16254_c0_g1_i5	Glyma.08G222200.1	100	216	9	1	2532	2594	57	0	3408

TRINITY_DN16082_c0_g1_i8	Glyma.09G023600.2	100	209	2	173	2713	2541	1	0	3524

TRINITY_DN16237_c0_g1_i4	Glyma.06G164900.1	100	198	24	260	2746	1	2532	0	3177

TRINITY_DN16237_c0_g1_i6	Glyma.06G164900.1	99.55357143	198	24	278	2764	1	2532	0	3177

TRINITY_DN16237_c0_g1_i9	Glyma.06G164900.1	95.9446093	198	24	266	2752	1	2532	0	3177

TRINITY_DN16237_c0_g1_i4	Glyma.04G200600.1	98.06629834	186	22	260	2746	1	2532	0	3243

TRINITY_DN16237_c0_g1_i6	Glyma.04G200600.1	95.08716323	186	22	278	2764	1	2532	0	3243

TRINITY_DN16237_c0_g1_i9	Glyma.04G200600.1	99.31506849	186	22	266	2752	1	2532	0	3243

TRINITY_DN11827_c0_g1_i2	Glyma.07G009800.1	97.22222222	178	2	362	2900	1	2539	0	3670

TRINITY_DN11827_c0_g1_i2	Glyma.07G009800.2	97.22222222	178	2	362	2900	1	2539	0	3670

TRINITY_DN16082_c0_g1_i1	Glyma.09G023600.2	98.52507375	175	2	173	2713	2541	1	0	3712

TRINITY_DN10938_c0_g1_i1	Glyma.15G193400.3	100	137	6	158	2695	2538	1	0	3884

TRINITY_DN15655_c2_g1_i2	Glyma.11G204200.1	100	272	11	130	2655	1	2535	0	3049

TRINITY_DN15655_c2_g1_i2	Glyma.11G204200.2	95.71428571	272	11	130	2655	1	2535	0	3049

TRINITY_DN16082_c0_g1_i11	Glyma.09G023600.2	100	267	0	173	2713	2541	1	0	3214

TRINITY_DN15815_c0_g2_i3	Glyma.12G219400.4	95.62982005	238	7	1489	4023	3114	577	0	3315

TRINITY_DN16082_c0_g1_i5	Glyma.09G023600.2	95.95375723	233	0	173	2713	2541	1	0	3402

TRINITY_DN14940_c1_g2_i1	Glyma.12G148200.1	100	218	2	338	2875	2541	1	0	3465

TRINITY_DN14571_c0_g2_i1	Glyma.09G247600.1	100	212	15	1584	4114	2522	1	0	3330

TRINITY_DN14571_c0_g2_i2	Glyma.09G247600.1	98.18181818	212	15	1605	4135	2522	1	0	3330

TRINITY_DN16082_c0_g1_i8	Glyma.15G129700.1	96.72330097	198	4	175	2713	2539	1	0	3570

TRINITY_DN9476_c0_g1_i1	Glyma.07G263100.3	98.03921569	177	3	709	3243	2544	24	0	3544

TRINITY_DN13972_c0_g1_i2	Glyma.11G212700.1	97.14285714	172	5	187	2721	2538	1	0	3681

TRINITY_DN16082_c0_g1_i1	Glyma.15G129700.1	96.98492462	166	4	175	2713	2539	1	0	3747

TRINITY_DN14162_c0_g1_i3	Glyma.20G191400.2	96.75324675	139	4	369	2909	2535	1	0	3884

TRINITY_DN12780_c0_g2_i1	Glyma.19G263300.1	100	412	23	347	2868	203	2700	0	2021

TRINITY_DN15655_c2_g1_i3	Glyma.14G208500.1	100	297	12	130	2658	1	2532	0	2922

TRINITY_DN16082_c0_g1_i11	Glyma.15G129700.1	95.23809524	261	2	175	2713	2539	1	0	3232

TRINITY_DN15815_c0_g2_i3	Glyma.13G281900.1	100	241	6	1489	4023	3114	577	0	3310

TRINITY_DN15648_c0_g2_i1	Glyma.10G153800.5	95.69892473	237	6	223	2757	3381	844	0	3332

TRINITY_DN15648_c0_g2_i1	Glyma.10G153800.1	95.69892473	237	6	223	2757	3381	844	0	3332

TRINITY_DN15648_c0_g2_i1	Glyma.10G153800.4	95.69892473	237	6	223	2757	3381	844	0	3332

TRINITY_DN15648_c0_g2_i1	Glyma.10G153800.3	100	237	6	223	2757	3381	844	0	3332

TRINITY_DN15648_c0_g2_i1	Glyma.10G153800.2	96.34146341	237	6	223	2757	3381	844	0	3332

TRINITY_DN15648_c0_g2_i1	Glyma.10G153800.11	96.46924829	237	6	223	2757	3381	844	0	3332

TRINITY_DN15648_c0_g2_i1	Glyma.10G153800.17	100	237	6	223	2757	3378	841	0	3332

TRINITY_DN15648_c0_g2_i1	Glyma.10G153800.16	95.69892473	237	6	223	2757	3378	841	0	3332

TRINITY_DN15648_c0_g2_i1	Glyma.10G153800.15	95.69892473	237	6	223	2757	3378	841	0	3332

TRINITY_DN15648_c0_g2_i1	Glyma.10G153800.14	95.69892473	237	6	223	2757	3378	841	0	3332

TRINITY_DN15648_c0_g2_i1	Glyma.10G153800.13	95.69892473	237	6	223	2757	3378	841	0	3332

TRINITY_DN15305_c1_g1_i1	Glyma.13G330400.1	97.61904762	236	23	480	2973	2539	1	0	3079

TRINITY_DN16082_c0_g1_i5	Glyma.15G129700.1	95.95375723	229	2	175	2713	2539	1	0	3410

TRINITY_DN13425_c0_g1_i1	Glyma.12G054700.1	97.77777778	225	8	212	2743	211	2745	0	3360

TRINITY_DN13425_c0_g1_i3	Glyma.12G054700.1	99.25925926	222	12	212	2731	211	2745	0	3299

TRINITY_DN15808_c0_g1_i2	Glyma.17G077400.1	98.16513761	186	6	206	2726	1	2533	0	3493

TRINITY_DN15808_c0_g1_i2	Glyma.17G077400.2	100	186	6	206	2726	1	2533	0	3493

TRINITY_DN15808_c0_g1_i3	Glyma.17G077400.2	98.75	186	6	206	2726	1	2533	0	3493

TRINITY_DN15808_c0_g1_i3	Glyma.17G077400.1	98.98989899	186	6	206	2726	1	2533	0	3493

TRINITY_DN15808_c0_g1_i5	Glyma.17G077400.2	100	186	6	206	2726	1	2533	0	3493

TRINITY_DN15808_c0_g1_i5	Glyma.17G077400.1	95.17241379	186	6	206	2726	1	2533	0	3493

TRINITY_DN15655_c2_g1_i3	Glyma.11G204200.1	98.30508475	170	7	130	2658	1	2535	0	3646

TRINITY_DN15655_c2_g1_i3	Glyma.11G204200.2	98.30508475	170	7	130	2658	1	2535	0	3646

TRINITY_DN12719_c0_g2_i6	Glyma.13G321300.50	96	170	30	1	2505	2475	1	0	3103

TRINITY_DN12719_c0_g2_i6	Glyma.13G321300.47	99.00990099	170	30	1	2505	2475	1	0	3103

TRINITY_DN12719_c0_g2_i6	Glyma.13G321300.16	96.02649007	170	30	1	2505	2475	1	0	3103

TRINITY_DN12719_c0_g2_i6	Glyma.13G321300.17	96.02649007	170	30	1	2505	2475	1	0	3103

TRINITY_DN12719_c0_g2_i8	Glyma.13G321300.50	98.7012987	170	30	1	2505	2475	1	0	3103

TRINITY_DN12719_c0_g2_i8	Glyma.13G321300.47	98.70550162	170	30	1	2505	2475	1	0	3103

TRINITY_DN12719_c0_g2_i8	Glyma.13G321300.16	98.70550162	170	30	1	2505	2475	1	0	3103

TRINITY_DN12719_c0_g2_i8	Glyma.13G321300.17	96.15384615	170	30	1	2505	2475	1	0	3103

TRINITY_DN14219_c0_g2_i1	Glyma.20G120300.1	96.20437956	139	2	683	3221	1	2539	0	3908

TRINITY_DN14219_c0_g2_i1	Glyma.20G120300.2	96.20253165	139	2	683	3221	1	2539	0	3908

TRINITY_DN14219_c0_g2_i2	Glyma.20G120300.1	97.59036145	139	2	320	2858	1	2539	0	3908

TRINITY_DN14219_c0_g2_i2	Glyma.20G120300.2	97.6076555	139	2	320	2858	1	2539	0	3908

TRINITY_DN14219_c0_g2_i3	Glyma.20G120300.1	97.6	139	2	205	2743	1	2539	0	3908

TRINITY_DN14219_c0_g2_i3	Glyma.20G120300.2	97.6	139	2	205	2743	1	2539	0	3908

TRINITY_DN16222_c0_g1_i5	Glyma.08G052900.3	98.30508475	212	1	155	2693	1	2533	0	3476

TRINITY_DN16222_c0_g1_i5	Glyma.08G052900.2	98.07280514	212	1	155	2693	1	2533	0	3476

TRINITY_DN16222_c0_g1_i5	Glyma.08G052900.1	100	212	1	155	2693	1	2533	0	3476

TRINITY_DN16222_c0_g1_i8	Glyma.08G052900.3	96.28140704	211	1	168	2706	1	2533	0	3482

TRINITY_DN16222_c0_g1_i8	Glyma.08G052900.2	97.76674938	211	1	168	2706	1	2533	0	3482

TRINITY_DN16222_c0_g1_i8	Glyma.08G052900.1	96.42857143	211	1	168	2706	1	2533	0	3482

TRINITY_DN16222_c0_g1_i5	Glyma.05G245100.1	95.52845528	201	4	155	2693	1	2530	0	3517

TRINITY_DN16222_c0_g1_i8	Glyma.05G245100.1	98.30508475	200	4	168	2706	1	2530	0	3522

TRINITY_DN16254_c0_g1_i10	Glyma.08G222200.1	97.40871614	151	5	1	2529	2594	57	0	3781

TRINITY_DN14704_c0_g1_i1	Glyma.06G081600.1	95.215311	135	2	192	2727	1	2536	0	3903

TRINITY_DN16360_c0_g4_i1	Glyma.07G010400.1	95	127	3	329	2863	1	2538	0	3952

TRINITY_DN16082_c0_g1_i12	Glyma.08G202000.1	100	504	57	121	2587	2553	42	0	1267

TRINITY_DN16064_c0_g2_i1	Glyma.15G012000.2	95.23809524	381	28	844	3370	3	2511	0	2318

TRINITY_DN16064_c0_g2_i3	Glyma.15G012000.2	99.44444444	381	28	844	3370	3	2511	0	2318

TRINITY_DN16254_c0_g1_i4	Glyma.13G101700.1	96.875	322	18	1014	3540	2734	214	0	2723

TRINITY_DN15648_c0_g2_i1	Glyma.20G234500.6	97.25274725	228	6	223	2757	3420	886	0	3386

TRINITY_DN15648_c0_g2_i1	Glyma.20G234500.4	99.39393939	228	6	223	2757	3378	844	0	3386

TRINITY_DN16303_c0_g1_i2	Glyma.12G201100.4	98.79032258	138	6	152	2687	1	2511	0	3735

TRINITY_DN14704_c0_g1_i1	Glyma.04G080000.1	95.12195122	137	0	192	2729	1	2538	0	3928

TRINITY_DN16082_c0_g1_i7	Glyma.04G085000.1	100	505	42	188	2691	2535	20	0	1539

TRINITY_DN15345_c0_g1_i1	Glyma.06G305000.1	97.17514124	431	35	507	3020	120	2636	0	2019

TRINITY_DN15345_c0_g1_i1	Glyma.06G305000.3	97.17514124	431	35	507	3020	120	2636	0	2019

TRINITY_DN15345_c0_g1_i2	Glyma.06G305000.1	100	431	35	313	2826	120	2636	0	2019

TRINITY_DN15345_c0_g1_i2	Glyma.06G305000.3	100	431	35	313	2826	120	2636	0	2019

TRINITY_DN12697_c0_g1_i1	Glyma.11G108500.1	97.34513274	389	39	446	2946	2556	38	0	2182

TRINITY_DN12697_c0_g1_i2	Glyma.11G108500.1	97.33333333	385	39	325	2825	2556	38	0	2204

TRINITY_DN15655_c2_g1_i2	Glyma.18G046800.1	95.71428571	273	15	130	2655	1	2526	0	3031

TRINITY_DN14216_c0_g1_i2	Glyma.19G159400.1	100	354	4	433	2967	316	2847	0	2689

TRINITY_DN14216_c0_g1_i2	Glyma.19G159400.9	100	354	4	433	2967	190	2721	0	2689

TRINITY_DN9350_c0_g1_i1	Glyma.10G019200.2	96.1038961	258	9	154	2686	1	2524	0	3157

TRINITY_DN14913_c0_g1_i1	Glyma.08G179100.1	95.93495935	228	16	149	2674	1	2529	0	3310

TRINITY_DN16360_c0_g4_i1	Glyma.08G193500.1	95	143	4	329	2863	1	2532	0	3858

TRINITY_DN16082_c0_g1_i9	Glyma.04G085000.1	96.18138425	512	38	142	2645	2535	20	0	1522

TRINITY_DN16022_c0_g2_i2	Glyma.12G233300.2	100	471	53	443	2955	9	2497	0	1633

TRINITY_DN15570_c0_g1_i2	Glyma.03G130900.1	100	357	18	596	3109	3	2528	0	2510

TRINITY_DN16252_c0_g1_i1	Glyma.08G293600.5	100	245	9	1665	4180	21	2554	0	3195

TRINITY_DN14106_c0_g1_i3	Glyma.12G180700.1	98	213	24	1	2503	2325	4845	0	3205

TRINITY_DN14731_c0_g1_i1	Glyma.18G055200.1	97.92207792	195	8	438	2960	2526	1	0	3465

TRINITY_DN15655_c2_g1_i3	Glyma.18G046800.1	99.51456311	174	6	130	2658	1	2526	0	3620

TRINITY_DN15655_c2_g1_i2	Glyma.14G208500.1	98.17351598	165	7	130	2655	1	2532	0	3690

TRINITY_DN15981_c0_g1_i19	Glyma.11G048300.3	97.22222222	154	3	427	2955	2535	1	0	3790

TRINITY_DN15981_c0_g1_i5	Glyma.11G048300.3	98.97959184	154	3	427	2955	2535	1	0	3790

TRINITY_DN9837_c0_g1_i1	Glyma.04G124300.1	99.30795848	135	3	348	2876	2532	1	0	3875

TRINITY_DN16082_c0_g1_i7	Glyma.06G086600.1	96.83544304	511	41	192	2691	2534	20	0	1506

TRINITY_DN15919_c0_g1_i2	Glyma.05G200300.1	96.1038961	375	17	573	3083	2813	300	0	2324

TRINITY_DN16082_c0_g1_i9	Glyma.06G086600.1	96.18138425	517	38	146	2645	2534	20	0	1483

TRINITY_DN16420_c0_g1_i2	Glyma.01G040900.1	96.1352657	341	22	102	2613	11	2513	0	2459

TRINITY_DN16420_c0_g1_i2	Glyma.01G040900.2	96.79715302	341	22	102	2613	11	2513	0	2459

TRINITY_DN15919_c0_g1_i3	Glyma.05G200300.1	100	314	13	573	3089	2813	300	0	2712

TRINITY_DN15068_c0_g1_i3	Glyma.04G080600.1	97.16981132	240	23	275	2779	2525	15	0	3025

TRINITY_DN14913_c0_g1_i1	Glyma.15G053400.1	95.93495935	232	13	149	2674	1	2526	0	3302

TRINITY_DN9156_c0_g1_i2	Glyma.18G109800.1	100	205	2	63	2594	1	2532	0	3530

TRINITY_DN16086_c0_g2_i2	Glyma.15G207900.1	100	581	41	296	2806	32	2541	0	1179

TRINITY_DN16004_c1_g1_i4	Glyma.15G207900.1	98.07692308	327	10	229	2753	1	2528	0	2793

TRINITY_DN13890_c0_g1_i2	Glyma.08G170000.1	99.31972789	285	0	294	2825	1	2532	0	3097

TRINITY_DN13890_c0_g1_i2	Glyma.15G257100.1	98.33333333	284	0	294	2825	1	2532	0	3103

TRINITY_DN16252_c0_g1_i1	Glyma.08G293600.3	97.54098361	245	7	1665	4180	21	2551	0	3208

TRINITY_DN14675_c0_g1_i2	Glyma.09G167100.1	99.20634921	244	4	896	3421	2821	293	0	3265

TRINITY_DN15068_c0_g1_i3	Glyma.06G082300.1	97.16981132	238	22	275	2779	2531	15	0	3086

TRINITY_DN14106_c0_g1_i3	Glyma.12G180700.2	95.86466165	213	23	1	2503	2325	4842	0	3217

TRINITY_DN9156_c0_g1_i2	Glyma.08G307900.1	96.24573379	200	0	63	2594	1	2532	0	3568

TRINITY_DN16214_c0_g1_i1	Glyma.14G031600.1	97.36842105	196	11	500	3017	3495	966	0	3487

TRINITY_DN13890_c0_g1_i1	Glyma.08G170000.1	96.86098655	181	0	121	2652	1	2532	0	3674

TRINITY_DN13890_c0_g1_i1	Glyma.15G257100.1	96.86098655	173	0	121	2652	1	2532	0	3718

TRINITY_DN14913_c0_g2_i1	Glyma.08G179100.1	95.93495935	158	8	149	2674	1	2529	0	3742

TRINITY_DN9742_c0_g1_i1	Glyma.03G161300.6	100	158	15	288	2812	2505	2	0	3574

TRINITY_DN9742_c0_g1_i1	Glyma.03G161300.5	100	158	15	288	2812	2505	2	0	3574

TRINITY_DN9837_c0_g1_i1	Glyma.06G314300.1	98.46153846	139	3	348	2876	2529	1	0	3868

TRINITY_DN16043_c0_g2_i2	Glyma.18G129200.1	96.37681159	118	1	871	3402	1	2526	0	3984

TRINITY_DN20555_c0_g1_i1	Glyma.13G273300.1	97.56097561	256	16	1	2507	3077	5601	0	3062

TRINITY_DN15072_c0_g1_i2	Glyma.08G309400.1	100	249	7	685	3205	2571	45	0	3216

TRINITY_DN9205_c0_g1_i4	Glyma.18G199400.1	100	217	16	449	2931	2528	1	0	3153

TRINITY_DN9205_c0_g1_i4	Glyma.18G199400.2	96.69421488	213	26	449	2931	2504	1	0	3020

TRINITY_DN9810_c0_g1_i2	Glyma.07G234900.1	100	203	13	1	2530	24	2526	0	3373

TRINITY_DN16082_c0_g1_i12	Glyma.08G202000.6	100	505	59	121	2587	2544	42	0	1304

TRINITY_DN15710_c0_g2_i1	Glyma.12G074600.6	100	391	47	36	2537	2508	14	0	2109

TRINITY_DN15710_c0_g2_i1	Glyma.12G074600.1	100	391	47	36	2537	2508	14	0	2109

TRINITY_DN15710_c0_g2_i1	Glyma.12G074600.5	100	391	47	36	2537	2508	14	0	2109

TRINITY_DN15710_c0_g2_i1	Glyma.12G074600.10	96.80851064	391	47	36	2537	2508	14	0	2109

TRINITY_DN15710_c0_g2_i1	Glyma.12G074600.7	97.76357827	391	47	36	2537	2508	14	0	2109

TRINITY_DN15710_c0_g2_i1	Glyma.12G074600.3	100	391	47	36	2537	2508	14	0	2109

TRINITY_DN15710_c0_g2_i1	Glyma.12G074600.2	100	391	47	36	2537	2508	14	0	2109

TRINITY_DN15710_c0_g2_i1	Glyma.12G074600.4	96.2962963	391	47	36	2537	2508	14	0	2109

TRINITY_DN15710_c0_g2_i2	Glyma.12G074600.6	98.55072464	391	47	36	2537	2508	14	0	2109

TRINITY_DN15710_c0_g2_i2	Glyma.12G074600.1	97.56097561	391	47	36	2537	2508	14	0	2109

TRINITY_DN15710_c0_g2_i2	Glyma.12G074600.5	96.21848739	391	47	36	2537	2508	14	0	2109

TRINITY_DN15710_c0_g2_i2	Glyma.12G074600.10	97.2972973	391	47	36	2537	2508	14	0	2109

TRINITY_DN15710_c0_g2_i2	Glyma.12G074600.7	99.12280702	391	47	36	2537	2508	14	0	2109

TRINITY_DN15710_c0_g2_i2	Glyma.12G074600.3	98.24120603	391	47	36	2537	2508	14	0	2109

TRINITY_DN15710_c0_g2_i2	Glyma.12G074600.2	100	391	47	36	2537	2508	14	0	2109

TRINITY_DN15710_c0_g2_i2	Glyma.12G074600.4	98.67109635	391	47	36	2537	2508	14	0	2109

TRINITY_DN16420_c0_g1_i2	Glyma.06G201700.1	95.53571429	200	4	92	2613	1	2525	0	3491

TRINITY_DN11600_c0_g1_i2	Glyma.02G119700.1	95	199	33	60	2555	1	2508	0	3219

TRINITY_DN6291_c0_g1_i1	Glyma.18G016000.1	96.40718563	176	0	1	2529	4560	7088	0	3696

TRINITY_DN6291_c0_g2_i1	Glyma.18G016000.1	96.34146341	176	0	1	2529	4560	7088	0	3696

TRINITY_DN7938_c0_g1_i1	Glyma.12G075800.1	100	163	2	490	3015	2526	1	0	3729

TRINITY_DN14913_c0_g2_i1	Glyma.15G053400.1	95.93495935	161	5	149	2674	1	2526	0	3740

TRINITY_DN7938_c0_g1_i1	Glyma.11G145800.1	100	152	1	490	3015	2529	1	0	3808

TRINITY_DN10085_c0_g1_i1	Glyma.17G121600.2	99.10714286	146	1	150	2675	1	2529	0	3842

TRINITY_DN16415_c1_g1_i1	Glyma.13G327000.1	99.23664122	384	7	1153	3675	2523	1	0	2477

TRINITY_DN16415_c1_g1_i10	Glyma.13G327000.1	97.13193117	384	7	746	3268	2523	1	0	2477

TRINITY_DN20555_c0_g1_i1	Glyma.12G227000.1	99	275	13	1	2507	2933	5448	0	2931

TRINITY_DN20555_c0_g1_i1	Glyma.12G227000.3	97.75280899	275	13	1	2507	1427	3942	0	2931

TRINITY_DN16082_c0_g1_i7	Glyma.07G016700.1	98.20143885	240	11	188	2695	2526	1	0	3197

TRINITY_DN13926_c0_g1_i2	Glyma.01G234600.8	100	221	23	463	2929	2706	183	0	3024

TRINITY_DN13926_c0_g1_i2	Glyma.01G234600.9	95.52845528	221	23	463	2929	2706	183	0	3024

TRINITY_DN13926_c0_g1_i1	Glyma.01G234600.8	100	218	29	463	2912	2706	183	0	2931

TRINITY_DN13926_c0_g1_i1	Glyma.01G234600.9	96.76339286	218	29	463	2912	2706	183	0	2931

TRINITY_DN16082_c0_g1_i9	Glyma.07G016700.1	100	212	11	142	2649	2526	1	0	3352

TRINITY_DN3367_c0_g1_i1	Glyma.09G228600.1	96.47058824	205	17	147	2656	32	2553	0	3378

TRINITY_DN16376_c3_g12_i5	Glyma.05G151100.2	96	197	17	124	2645	5	2508	0	3384

TRINITY_DN15808_c0_g1_i2	Glyma.05G022300.2	99.09090909	172	4	206	2726	1	2524	0	3644

TRINITY_DN15808_c0_g1_i2	Glyma.05G022300.1	99.09090909	172	4	206	2726	1	2524	0	3644

TRINITY_DN15808_c0_g1_i3	Glyma.05G022300.2	95.8677686	172	4	206	2726	1	2524	0	3644

TRINITY_DN15808_c0_g1_i3	Glyma.05G022300.1	100	172	4	206	2726	1	2524	0	3644

TRINITY_DN15808_c0_g1_i5	Glyma.05G022300.2	100	172	4	206	2726	1	2524	0	3644

TRINITY_DN15808_c0_g1_i5	Glyma.05G022300.1	99.03846154	172	4	206	2726	1	2524	0	3644

TRINITY_DN14106_c0_g1_i3	Glyma.13G320100.1	98	225	20	1	2503	2313	4827	0	3188

TRINITY_DN2601_c0_g1_i1	Glyma.06G091300.2	98.99497487	183	8	563	3086	3927	1417	0	3530

TRINITY_DN16499_c0_g1_i1	Glyma.08G265300.6	100	150	7	623	3145	1	2514	0	3725

TRINITY_DN14731_c0_g1_i1	Glyma.11G161500.1	97.92207792	193	9	438	2951	2517	1	0	3459

TRINITY_DN3367_c0_g1_i1	Glyma.09G228600.2	100	205	15	147	2656	32	2550	0	3391

TRINITY_DN16420_c0_g1_i3	Glyma.06G201700.1	100	193	10	92	2592	1	2525	0	3439

TRINITY_DN5366_c0_g1_i1	Glyma.02G159100.1	100	188	9	1327	3844	2566	52	0	3511

TRINITY_DN5366_c0_g2_i1	Glyma.02G159100.1	98.14814815	188	9	799	3316	2566	52	0	3511

TRINITY_DN12719_c0_g2_i5	Glyma.13G321300.50	98.26086957	170	29	1	2490	2475	1	0	3171

TRINITY_DN12719_c0_g2_i5	Glyma.13G321300.47	97.40802676	170	29	1	2490	2475	1	0	3171

TRINITY_DN12719_c0_g2_i5	Glyma.13G321300.16	95	170	29	1	2490	2475	1	0	3171

TRINITY_DN12719_c0_g2_i5	Glyma.13G321300.17	98.77300613	170	29	1	2490	2475	1	0	3171

TRINITY_DN12719_c0_g2_i7	Glyma.13G321300.50	97.91666667	170	29	1	2490	2475	1	0	3171

TRINITY_DN12719_c0_g2_i7	Glyma.13G321300.47	97.91666667	170	29	1	2490	2475	1	0	3171

TRINITY_DN12719_c0_g2_i7	Glyma.13G321300.16	96.96969697	170	29	1	2490	2475	1	0	3171

TRINITY_DN12719_c0_g2_i7	Glyma.13G321300.17	97.2972973	170	29	1	2490	2475	1	0	3171

TRINITY_DN13423_c0_g2_i1	Glyma.17G032400.2	100	154	3	838	3359	2565	44	0	3771

TRINITY_DN16275_c0_g1_i2	Glyma.13G030300.1	95.95588235	455	19	239	2751	245	2757	0	1999

TRINITY_DN16076_c1_g1_i1	Glyma.01G183400.1	96.77419355	292	24	75	2576	1	2508	0	2798

TRINITY_DN16249_c1_g3_i9	Glyma.05G003500.1	97.77777778	188	8	290	2798	5865	3363	0	3387

TRINITY_DN13158_c0_g1_i1	Glyma.04G208500.1	100	145	0	253	2776	2532	9	0	3858

TRINITY_DN16376_c3_g12_i3	Glyma.05G151100.2	96.22641509	139	14	124	2641	5	2507	0	3716

TRINITY_DN4063_c0_g1_i1	Glyma.11G114100.1	97.46835443	411	7	1	2517	2576	57	0	2324

TRINITY_DN16076_c1_g1_i1	Glyma.11G058900.1	100	306	22	75	2576	1	2508	0	2732

TRINITY_DN16076_c1_g1_i1	Glyma.11G058900.2	100	306	22	75	2576	1	2508	0	2732

TRINITY_DN16022_c0_g2_i2	Glyma.01G118900.3	100	278	23	27	2533	1	2483	0	2758

TRINITY_DN16254_c0_g1_i5	Glyma.15G001300.1	97.46835443	230	9	1	2517	2609	96	0	3288

TRINITY_DN16132_c0_g1_i1	Glyma.17G226100.1	98.19277108	207	10	1174	3694	432	2943	0	3428

TRINITY_DN16132_c0_g1_i1	Glyma.17G226100.2	95.52238806	207	10	1174	3694	381	2892	0	3428

TRINITY_DN16082_c0_g1_i9	Glyma.07G016500.1	100	203	11	142	2649	2517	1	0	3398

TRINITY_DN13398_c0_g1_i1	Glyma.16G096600.1	95.18987342	181	9	252	2762	2973	457	0	3541

TRINITY_DN13398_c0_g1_i1	Glyma.08G264900.5	97.46835443	174	8	252	2762	2991	472	0	3598

TRINITY_DN13398_c0_g1_i1	Glyma.08G264900.3	97.46835443	174	8	252	2762	2991	472	0	3598

TRINITY_DN13398_c0_g1_i1	Glyma.08G264900.2	98.01980198	174	8	252	2762	2991	472	0	3598

TRINITY_DN13398_c0_g1_i1	Glyma.08G264900.1	95.18900344	174	8	252	2762	2991	472	0	3598

TRINITY_DN10934_c0_g2_i1	Glyma.10G163200.1	96.95121951	385	25	518	3017	81	2589	0	2298

TRINITY_DN16082_c0_g1_i7	Glyma.07G016500.1	100	235	9	188	2695	2517	1	0	3232

TRINITY_DN13926_c0_g1_i2	Glyma.01G234600.6	100	220	23	463	2923	2703	186	0	3018

TRINITY_DN13926_c0_g1_i2	Glyma.01G234600.7	96.23188406	220	23	463	2923	2703	186	0	3018

TRINITY_DN13926_c0_g1_i1	Glyma.01G234600.6	95.72649573	217	29	463	2906	2703	186	0	2926

TRINITY_DN13926_c0_g1_i1	Glyma.01G234600.7	95.45454545	217	29	463	2906	2703	186	0	2926

TRINITY_DN11678_c0_g1_i1	Glyma.13G308500.2	99.46236559	211	2	132	2653	2508	1	0	3398

TRINITY_DN16415_c1_g1_i1	Glyma.11G147900.1	98.7012987	196	6	1153	3673	2517	5	0	3507

TRINITY_DN16415_c1_g1_i10	Glyma.11G147900.1	100	196	6	746	3266	2517	5	0	3507

TRINITY_DN15421_c1_g3_i1	Glyma.15G210100.8	95.11278195	307	6	557	3069	2748	230	0	2891

TRINITY_DN15421_c1_g3_i1	Glyma.15G210100.7	99.74811083	307	6	557	3069	2748	230	0	2891

TRINITY_DN15421_c1_g3_i1	Glyma.15G210100.6	98.96907216	307	6	557	3069	2748	230	0	2891

TRINITY_DN15421_c1_g3_i1	Glyma.15G210100.5	98.92473118	307	6	557	3069	2748	230	0	2891

TRINITY_DN15421_c1_g3_i1	Glyma.15G210100.1	99.06542056	307	6	557	3069	2748	230	0	2891

TRINITY_DN15421_c1_g3_i1	Glyma.15G210100.2	99.6978852	307	6	557	3069	2748	230	0	2891

TRINITY_DN15421_c1_g3_i1	Glyma.15G210100.3	98.95833333	307	6	557	3069	2748	230	0	2891

TRINITY_DN15421_c1_g3_i1	Glyma.15G210100.4	95.41284404	307	6	557	3069	2748	230	0	2891

TRINITY_DN16254_c0_g1_i10	Glyma.15G001300.1	98.42519685	294	10	1	2514	2609	96	0	2937

TRINITY_DN10651_c0_g1_i1	Glyma.08G106400.1	99.07407407	279	17	317	2801	3443	927	0	2852

TRINITY_DN16275_c0_g1_i2	Glyma.20G054000.1	96.11650485	278	5	239	2751	194	2712	0	3051

TRINITY_DN15416_c1_g1_i2	Glyma.04G093800.3	100	243	24	425	2940	2465	1	0	2915

TRINITY_DN16132_c0_g1_i1	Glyma.14G098600.1	97.72727273	227	6	1174	3694	432	2943	0	3339

TRINITY_DN9476_c0_g1_i3	Glyma.07G263100.3	98.87640449	177	2	709	3223	2544	24	0	3637

TRINITY_DN16275_c0_g1_i5	Glyma.20G054000.1	98.0952381	317	7	239	2749	194	2710	0	2820

TRINITY_DN16022_c0_g2_i2	Glyma.01G118900.2	99.05660377	278	23	27	2530	1	2480	0	2752

TRINITY_DN16360_c0_g4_i2	Glyma.15G023800.1	95	234	5	229	2744	11	2523	0	3286

TRINITY_DN16082_c0_g1_i9	Glyma.08G201900.1	96.1038961	197	8	142	2649	2517	1	0	3465

TRINITY_DN14622_c0_g1_i4	Glyma.10G179700.6	99.41176471	184	15	193	2703	6	2484	0	3312

TRINITY_DN12674_c0_g2_i1	Glyma.08G360000.7	98.2300885	140	0	215	2734	2520	1	0	3879

TRINITY_DN16275_c0_g1_i5	Glyma.13G030300.1	99.5951417	417	13	239	2749	245	2755	0	2239

TRINITY_DN16076_c2_g3_i1	Glyma.05G082200.1	98.19277108	393	18	352	2861	2601	107	0	2261

TRINITY_DN16076_c2_g3_i1	Glyma.05G082200.2	95.52238806	393	18	352	2861	2529	35	0	2261

TRINITY_DN16076_c2_g3_i1	Glyma.05G082200.3	98.71794872	393	18	352	2861	2529	35	0	2261

TRINITY_DN16132_c0_g1_i1	Glyma.04G056200.1	95.75757576	290	7	1175	3688	436	2952	0	3000

TRINITY_DN16082_c0_g1_i7	Glyma.08G201900.1	95.07389163	232	6	188	2695	2517	1	0	3282

TRINITY_DN15345_c0_g1_i1	Glyma.12G194200.3	96.51162791	207	3	698	3210	44	2562	0	3467

TRINITY_DN15345_c0_g1_i2	Glyma.12G194200.3	98.16849817	207	3	504	3016	44	2562	0	3467

TRINITY_DN7705_c0_g1_i1	Glyma.11G054100.2	97.2972973	149	6	308	2824	13	2523	0	3762

TRINITY_DN3367_c0_g1_i1	Glyma.12G007900.1	98.14814815	199	12	147	2656	32	2538	0	3424

TRINITY_DN14622_c0_g1_i7	Glyma.10G179700.3	96.42058166	197	17	67	2573	3	2499	0	3352

TRINITY_DN11991_c0_g1_i1	Glyma.08G229100.2	100	175	4	102	2610	1	2515	0	3603

TRINITY_DN11991_c0_g1_i1	Glyma.08G229100.1	100	175	4	102	2610	1	2515	0	3603

TRINITY_DN16303_c0_g1_i2	Glyma.13G301100.4	98.7804878	129	1	152	2669	1	2517	0	3928

TRINITY_DN16303_c0_g1_i2	Glyma.13G301100.2	98.7804878	129	1	152	2669	1	2517	0	3928

TRINITY_DN3163_c0_g1_i1	Glyma.11G028700.1	99.62546816	127	6	600	3093	2506	1	0	3714

TRINITY_DN16082_c0_g1_i12	Glyma.08G202000.3	100	507	53	121	2587	2532	42	0	1349

TRINITY_DN16216_c0_g3_i7	Glyma.11G012500.1	100	182	17	42	2519	132	2644	0	3362

TRINITY_DN16216_c0_g3_i7	Glyma.11G012500.2	97.38372093	182	17	42	2519	132	2644	0	3362

TRINITY_DN12719_c0_g2_i1	Glyma.13G321300.45	96.33027523	172	29	1	2482	2472	6	0	3145

TRINITY_DN12719_c0_g2_i1	Glyma.13G321300.33	98.49246231	172	29	1	2482	2472	6	0	3145

TRINITY_DN12719_c0_g2_i1	Glyma.13G321300.13	100	172	29	1	2482	2472	6	0	3145

TRINITY_DN12719_c0_g2_i3	Glyma.13G321300.45	100	172	29	1	2482	2472	6	0	3145

TRINITY_DN12719_c0_g2_i3	Glyma.13G321300.33	100	172	29	1	2482	2472	6	0	3145

TRINITY_DN12719_c0_g2_i3	Glyma.13G321300.13	100	172	29	1	2482	2472	6	0	3145

TRINITY_DN15913_c1_g2_i2	Glyma.19G103200.4	95.45454545	159	2	902	3415	2513	3	0	3709

TRINITY_DN12145_c0_g1_i1	Glyma.16G044400.1	100	155	1	400	2916	3036	523	0	3770

TRINITY_DN12145_c0_g1_i1	Glyma.19G107500.2	98.1595092	147	0	400	2916	3039	523	0	3834

TRINITY_DN12145_c0_g1_i1	Glyma.19G107500.1	99.54751131	147	0	400	2916	3039	523	0	3834

TRINITY_DN12145_c0_g1_i1	Glyma.19G107500.3	100	147	0	400	2916	3039	523	0	3834

TRINITY_DN16303_c0_g1_i2	Glyma.12G201100.3	98	137	6	152	2668	1	2489	0	3709

TRINITY_DN16303_c0_g1_i2	Glyma.12G201100.1	98	137	6	152	2668	1	2489	0	3709

TRINITY_DN16303_c0_g1_i2	Glyma.12G201100.2	98.79032258	137	6	152	2668	1	2489	0	3709

TRINITY_DN16082_c0_g1_i7	Glyma.08G202000.1	97.69736842	243	10	188	2671	2553	40	0	3081

TRINITY_DN16214_c0_g1_i1	Glyma.02G282700.1	100	197	5	515	3017	3474	960	0	3465

TRINITY_DN16214_c0_g1_i1	Glyma.02G282700.2	96.03174603	197	5	515	3017	3399	885	0	3465

TRINITY_DN12719_c0_g2_i6	Glyma.13G321300.49	96.40718563	174	21	1	2505	2451	1	0	3192

TRINITY_DN12719_c0_g2_i6	Glyma.13G321300.46	95.0310559	174	21	1	2505	2451	1	0	3192

TRINITY_DN12719_c0_g2_i6	Glyma.13G321300.15	95.0310559	174	21	1	2505	2451	1	0	3192

TRINITY_DN12719_c0_g2_i6	Glyma.13G321300.14	96.82539683	174	21	1	2505	2451	1	0	3192

TRINITY_DN12719_c0_g2_i8	Glyma.13G321300.49	95.11400651	174	21	1	2505	2451	1	0	3192

TRINITY_DN12719_c0_g2_i8	Glyma.13G321300.46	97.19626168	174	21	1	2505	2451	1	0	3192

TRINITY_DN12719_c0_g2_i8	Glyma.13G321300.15	96.55172414	174	21	1	2505	2451	1	0	3192

TRINITY_DN12719_c0_g2_i8	Glyma.13G321300.14	98.7012987	174	21	1	2505	2451	1	0	3192

TRINITY_DN12674_c0_g2_i1	Glyma.08G360000.6	98.21428571	141	0	547	3062	2539	24	0	3866

TRINITY_DN16082_c0_g1_i12	Glyma.07G016700.1	100	510	54	121	2587	2526	36	0	1354

TRINITY_DN16082_c0_g1_i10	Glyma.04G085000.1	100	421	34	188	2661	2535	41	0	1917

TRINITY_DN12697_c0_g1_i1	Glyma.12G016900.1	97.34513274	388	32	446	2929	2553	55	0	2191

TRINITY_DN12697_c0_g1_i1	Glyma.12G016900.3	97.34513274	388	32	446	2929	2502	4	0	2191

TRINITY_DN12697_c0_g1_i1	Glyma.12G016900.2	97.34513274	388	32	446	2929	2502	4	0	2191

TRINITY_DN12697_c0_g1_i2	Glyma.12G016900.1	97.34513274	384	32	325	2808	2553	55	0	2213

TRINITY_DN12697_c0_g1_i2	Glyma.12G016900.3	97.33840304	384	32	325	2808	2502	4	0	2213

TRINITY_DN12697_c0_g1_i2	Glyma.12G016900.2	100	384	32	325	2808	2502	4	0	2213

TRINITY_DN15040_c0_g1_i3	Glyma.15G163900.1	98.71794872	379	47	406	2858	2949	455	0	2015

TRINITY_DN15040_c0_g1_i3	Glyma.15G163900.3	98.71794872	379	47	406	2858	2949	455	0	2015

TRINITY_DN16420_c0_g1_i3	Glyma.01G040900.1	98.52941176	349	17	102	2592	11	2513	0	2479

TRINITY_DN16420_c0_g1_i3	Glyma.01G040900.2	100	349	17	102	2592	11	2513	0	2479

TRINITY_DN16132_c0_g1_i1	Glyma.06G056400.1	96.39175258	301	2	1175	3688	436	2949	0	2964

TRINITY_DN16132_c0_g1_i1	Glyma.06G056400.4	100	301	2	1175	3688	436	2949	0	2964

TRINITY_DN16132_c0_g1_i1	Glyma.06G056400.3	97.5	301	3	1175	3688	436	2943	0	2926

TRINITY_DN14994_c0_g1_i2	Glyma.13G115500.2	97.91666667	155	0	189	2703	1	2515	0	3786

TRINITY_DN14994_c0_g1_i3	Glyma.13G115500.2	97.93814433	155	0	189	2703	1	2515	0	3786

TRINITY_DN16303_c0_g1_i3	Glyma.12G201100.4	100	137	5	152	2666	1	2488	0	3711

TRINITY_DN16303_c0_g1_i5	Glyma.12G201100.4	100	137	5	152	2666	1	2488	0	3711

TRINITY_DN16082_c0_g1_i10	Glyma.08G202000.1	98.63013699	292	14	188	2657	2553	42	0	2728

TRINITY_DN16082_c0_g1_i9	Glyma.08G202000.1	95.79831933	223	7	142	2625	2553	40	0	3214

TRINITY_DN15790_c3_g4_i3	Glyma.06G066900.1	96.28378378	142	1	1330	3837	2527	5040	0	3818

TRINITY_DN16082_c0_g1_i10	Glyma.06G086600.1	100	430	35	192	2661	2534	41	0	1858

TRINITY_DN16082_c0_g1_i13	Glyma.04G085000.1	95.3125	428	30	142	2615	2535	41	0	1901

TRINITY_DN16076_c2_g3_i1	Glyma.17G179200.1	97.72727273	388	24	361	2861	2523	35	0	2259

TRINITY_DN16076_c2_g3_i2	Glyma.17G179200.1	99.66777409	388	24	361	2861	2523	35	0	2259

TRINITY_DN16082_c0_g1_i1	Glyma.05G166400.1	100	334	12	173	2674	2562	55	0	2687

TRINITY_DN1095_c0_g2_i1	Glyma.07G166300.1	96.62921348	207	10	122	2614	2511	1	0	3352

TRINITY_DN16237_c0_g1_i11	Glyma.05G200800.4	100	206	18	256	2744	1	2498	0	3247

TRINITY_DN16237_c0_g1_i11	Glyma.05G200800.1	100	206	18	256	2744	1	2498	0	3247

TRINITY_DN16237_c0_g1_i5	Glyma.05G200800.4	99.77728285	206	18	256	2744	1	2498	0	3247

TRINITY_DN16237_c0_g1_i5	Glyma.05G200800.1	95.57713053	206	18	256	2744	1	2498	0	3247

TRINITY_DN1095_c0_g1_i1	Glyma.07G166300.1	95.2173913	205	17	122	2598	2511	1	0	3260

TRINITY_DN13832_c0_g2_i2	Glyma.10G066000.1	100	184	11	313	2815	2499	3	0	3454

TRINITY_DN15981_c0_g1_i10	Glyma.11G048300.2	100	159	3	1201	3707	2513	1	0	3722

TRINITY_DN15981_c0_g1_i10	Glyma.11G048300.1	98.57142857	159	3	1201	3707	2513	1	0	3722

TRINITY_DN15981_c0_g1_i14	Glyma.11G048300.2	100	159	3	1075	3581	2513	1	0	3722

TRINITY_DN15981_c0_g1_i14	Glyma.11G048300.1	98.46153846	159	3	1075	3581	2513	1	0	3722

TRINITY_DN10715_c0_g1_i3	Glyma.13G261000.1	100	145	0	872	3384	3386	5898	0	3838

TRINITY_DN10715_c0_g1_i3	Glyma.15G245800.1	100	145	0	872	3384	3386	5898	0	3838

TRINITY_DN10715_c0_g1_i3	Glyma.15G245800.2	100	145	0	872	3384	3305	5817	0	3838

TRINITY_DN16082_c0_g1_i13	Glyma.06G086600.1	99.14529915	436	32	146	2615	2534	41	0	1836

TRINITY_DN15040_c0_g1_i3	Glyma.09G057800.1	98.71794872	386	30	406	2858	2958	455	0	2067

TRINITY_DN15421_c1_g3_i2	Glyma.13G264600.1	97.16049383	339	10	584	3085	2793	292	0	2632

TRINITY_DN15421_c1_g3_i2	Glyma.13G264600.6	95.76271186	339	10	584	3085	2793	292	0	2632

TRINITY_DN15421_c1_g3_i2	Glyma.13G264600.8	97.36842105	339	10	584	3085	2793	292	0	2632

TRINITY_DN15421_c1_g3_i2	Glyma.13G264600.4	100	339	10	584	3085	2793	292	0	2632

TRINITY_DN16050_c0_g1_i1	Glyma.07G080400.1	97.59036145	329	15	281	2780	3144	642	0	2680

TRINITY_DN16082_c0_g1_i13	Glyma.08G202000.1	96.6507177	272	11	142	2611	2553	42	0	2861

TRINITY_DN16360_c0_g4_i2	Glyma.13G350700.1	95	236	4	234	2744	22	2526	0	3280

TRINITY_DN16249_c1_g3_i5	Glyma.05G003500.1	99.02439024	188	7	290	2786	5865	3363	0	3443

TRINITY_DN11991_c0_g1_i1	Glyma.15G215600.1	100	169	3	102	2610	1	2509	0	3664

TRINITY_DN13832_c0_g2_i2	Glyma.13G151100.1	100	168	9	313	2815	2499	3	0	3554

TRINITY_DN16303_c0_g1_i2	Glyma.13G301100.3	98.79518072	129	0	152	2663	1	2512	0	3925

TRINITY_DN16303_c0_g1_i2	Glyma.13G301100.1	98.7804878	129	0	152	2663	1	2512	0	3925

TRINITY_DN16303_c0_g1_i3	Glyma.13G301100.3	100	129	0	152	2663	1	2512	0	3925

TRINITY_DN16303_c0_g1_i3	Glyma.13G301100.1	100	129	0	152	2663	1	2512	0	3925

TRINITY_DN16303_c0_g1_i5	Glyma.13G301100.3	100	129	0	152	2663	1	2512	0	3925

TRINITY_DN16303_c0_g1_i5	Glyma.13G301100.1	100	129	0	152	2663	1	2512	0	3925

TRINITY_DN16082_c0_g1_i8	Glyma.05G166400.1	97.50566893	291	10	173	2674	2562	55	0	2948

TRINITY_DN16082_c0_g1_i5	Glyma.05G166400.1	98.36065574	266	7	173	2674	2562	55	0	3086

TRINITY_DN9844_c0_g1_i1	Glyma.11G068300.3	96.80365297	155	1	189	2699	2508	1	0	3759

TRINITY_DN9844_c0_g1_i2	Glyma.11G068300.3	100	155	1	178	2688	2508	1	0	3759

TRINITY_DN9844_c0_g1_i4	Glyma.11G068300.3	95.23809524	155	1	181	2691	2508	1	0	3759

TRINITY_DN9844_c0_g1_i5	Glyma.11G068300.3	95.53072626	155	1	179	2689	2508	1	0	3759

TRINITY_DN9844_c0_g1_i6	Glyma.11G068300.3	97.16981132	155	1	177	2687	2508	1	0	3759

TRINITY_DN16082_c0_g1_i12	Glyma.07G016500.1	100	498	48	121	2587	2517	36	0	1417

TRINITY_DN15981_c0_g1_i10	Glyma.17G161300.1	98.1233244	393	8	1201	3704	2507	1	0	2399

TRINITY_DN15981_c0_g1_i14	Glyma.17G161300.1	97.91666667	393	8	1075	3578	2507	1	0	2399

TRINITY_DN15981_c0_g1_i10	Glyma.05G105200.1	97.58064516	388	7	1201	3704	2507	1	0	2427

TRINITY_DN15981_c0_g1_i14	Glyma.05G105200.1	98.41269841	388	7	1075	3578	2507	1	0	2427

TRINITY_DN10833_c0_g1_i2	Glyma.09G248000.1	97.61904762	257	9	267	2767	2531	31	0	3096

TRINITY_DN1088_c0_g1_i1	Glyma.19G211500.1	96.89922481	254	14	112	2598	2513	6	0	3066

TRINITY_DN13158_c0_g1_i1	Glyma.06G157200.1	100	154	0	253	2762	2538	29	0	3783

TRINITY_DN2408_c0_g1_i1	Glyma.07G233900.1	97.20496894	141	0	13	2522	3388	879	0	3855

TRINITY_DN2408_c0_g1_i1	Glyma.17G039200.1	99.86091794	136	0	13	2522	3382	873	0	3882

TRINITY_DN2408_c0_g1_i1	Glyma.17G039200.8	98.07692308	136	0	13	2522	3241	732	0	3882

TRINITY_DN2408_c0_g1_i1	Glyma.17G039200.7	95.22900763	136	0	13	2522	3241	732	0	3882

TRINITY_DN2408_c0_g1_i1	Glyma.17G039200.5	96	136	0	13	2522	3241	732	0	3882

TRINITY_DN2408_c0_g1_i1	Glyma.17G039200.6	100	136	0	13	2522	3241	732	0	3882

TRINITY_DN2408_c0_g1_i1	Glyma.17G039200.4	100	136	0	13	2522	3241	732	0	3882

TRINITY_DN2408_c0_g1_i1	Glyma.17G039200.3	95	136	0	13	2522	3241	732	0	3882

TRINITY_DN2408_c0_g1_i1	Glyma.17G039200.2	99.513382	136	0	13	2522	3241	732	0	3882

TRINITY_DN15421_c1_g3_i2	Glyma.13G264600.2	97.59036145	339	9	584	3085	2790	292	0	2645

TRINITY_DN15421_c1_g3_i2	Glyma.13G264600.3	96.41693811	339	9	584	3085	2790	292	0	2645

TRINITY_DN15421_c1_g3_i2	Glyma.13G264600.5	97.56097561	339	9	584	3085	2790	292	0	2645

TRINITY_DN15421_c1_g3_i2	Glyma.13G264600.7	95.77464789	339	9	584	3085	2790	292	0	2645

TRINITY_DN10283_c0_g1_i1	Glyma.15G066300.1	95.65217391	329	10	270	2768	2520	16	0	2721

TRINITY_DN13968_c0_g3_i2	Glyma.13G243600.1	96.40522876	305	31	1827	4314	1476	3960	0	2652

TRINITY_DN13968_c0_g3_i2	Glyma.13G243600.2	100	305	31	1827	4314	1167	3651	0	2652

TRINITY_DN16254_c0_g1_i5	Glyma.13G372400.1	95.74468085	245	5	1	2505	2624	120	0	3225

TRINITY_DN16082_c0_g1_i11	Glyma.05G166400.1	96.68508287	223	6	173	2674	2562	55	0	3347

TRINITY_DN12878_c0_g1_i3	Glyma.11G231700.1	100	217	28	33	2518	1	2483	0	3114

TRINITY_DN3163_c0_g1_i1	Glyma.11G028700.3	100	127	5	600	3093	2497	1	0	3755

TRINITY_DN13968_c0_g3_i4	Glyma.13G243600.1	96.89440994	400	30	1827	4317	1476	3960	0	2158

TRINITY_DN13968_c0_g3_i4	Glyma.13G243600.2	96.89440994	400	30	1827	4317	1167	3651	0	2158

TRINITY_DN16050_c0_g1_i2	Glyma.02G093500.1	100	336	10	281	2780	3321	822	0	2667

TRINITY_DN16082_c0_g1_i1	Glyma.08G124400.1	100	326	10	173	2674	2550	49	0	2748

TRINITY_DN14913_c0_g2_i1	Glyma.08G072500.1	97.93814433	295	22	182	2674	55	2544	0	2784

TRINITY_DN13698_c1_g1_i2	Glyma.15G154900.1	96.38554217	252	25	354	2821	2486	1	0	2835

TRINITY_DN13698_c1_g1_i2	Glyma.15G154900.4	96.55172414	250	29	354	2821	2477	1	0	2787

TRINITY_DN15994_c0_g1_i2	Glyma.13G367300.2	95	196	5	455	2962	3333	835	0	3487

TRINITY_DN15314_c0_g2_i3	Glyma.11G179000.1	99.42196532	193	18	624	3108	2664	193	0	3181

TRINITY_DN14044_c0_g1_i1	Glyma.08G229600.2	97.26027397	175	8	228	2729	1	2490	0	3507

TRINITY_DN14044_c0_g1_i2	Glyma.08G229600.2	96.71361502	175	8	228	2729	1	2490	0	3507

TRINITY_DN16131_c0_g2_i2	Glyma.20G002900.1	100	522	44	102	2580	177	2655	0	1376

TRINITY_DN16050_c0_g1_i2	Glyma.18G288600.2	100	325	9	281	2780	3294	795	0	2739

TRINITY_DN16050_c0_g1_i2	Glyma.18G288600.1	100	325	9	281	2780	3294	795	0	2739

TRINITY_DN16254_c0_g1_i10	Glyma.13G372400.1	98.42519685	304	6	1	2502	2624	120	0	2900

TRINITY_DN1088_c0_g1_i1	Glyma.19G211500.2	100	254	13	112	2598	2510	6	0	3081

TRINITY_DN1088_c0_g1_i1	Glyma.03G214900.1	97.87234043	254	14	112	2597	2507	7	0	3048

TRINITY_DN16082_c0_g1_i7	Glyma.08G202000.6	97.69736842	243	13	188	2671	2544	40	0	3121

TRINITY_DN15648_c0_g2_i3	Glyma.10G153800.12	95.65217391	234	6	223	2724	3348	844	0	3288

TRINITY_DN15648_c0_g2_i3	Glyma.10G153800.10	95.19890261	234	6	223	2724	3348	844	0	3288

TRINITY_DN15648_c0_g2_i3	Glyma.10G153800.9	100	234	6	223	2724	3348	844	0	3288

TRINITY_DN15648_c0_g2_i3	Glyma.10G153800.8	97.05014749	234	6	223	2724	3348	844	0	3288

TRINITY_DN15648_c0_g2_i3	Glyma.10G153800.7	97.0505618	234	6	223	2724	3348	844	0	3288

TRINITY_DN15648_c0_g2_i3	Glyma.10G153800.6	95.40229885	234	6	223	2724	3348	844	0	3288

TRINITY_DN15648_c0_g2_i3	Glyma.10G153800.22	97.56097561	234	6	223	2724	3345	841	0	3288

TRINITY_DN15648_c0_g2_i3	Glyma.10G153800.21	97.75280899	234	6	223	2724	3345	841	0	3288

TRINITY_DN15648_c0_g2_i3	Glyma.10G153800.20	99	234	6	223	2724	3345	841	0	3288

TRINITY_DN15648_c0_g2_i3	Glyma.10G153800.19	95.58652729	234	6	223	2724	3345	841	0	3288

TRINITY_DN15648_c0_g2_i3	Glyma.10G153800.18	95.81589958	234	6	223	2724	3345	841	0	3288

TRINITY_DN15072_c0_g1_i2	Glyma.18G106900.1	99.81981982	219	7	685	3179	2574	70	0	3326

TRINITY_DN16082_c0_g1_i9	Glyma.08G202000.6	97.61904762	219	13	142	2625	2544	40	0	3254

TRINITY_DN14913_c0_g1_i1	Glyma.08G072500.1	95.94594595	216	20	182	2674	55	2544	0	3232

TRINITY_DN16237_c0_g1_i11	Glyma.08G008100.1	97.57914339	206	27	256	2744	1	2477	0	3179

TRINITY_DN16237_c0_g1_i5	Glyma.08G008100.1	96.60056657	206	27	256	2744	1	2477	0	3179

TRINITY_DN5876_c0_g1_i1	Glyma.07G270800.1	95.87155963	182	6	2	2495	29	2528	0	3493

TRINITY_DN15981_c0_g1_i10	Glyma.01G193600.2	100	164	1	1201	3704	2507	1	0	3701

TRINITY_DN15981_c0_g1_i10	Glyma.01G193600.1	100	164	1	1201	3704	2507	1	0	3701

TRINITY_DN15981_c0_g1_i14	Glyma.01G193600.2	95.05494505	164	1	1075	3578	2507	1	0	3701

TRINITY_DN15981_c0_g1_i14	Glyma.01G193600.1	100	164	1	1075	3578	2507	1	0	3701

TRINITY_DN15285_c1_g1_i2	Glyma.06G152500.1	98.16513761	157	3	68	2568	44	2544	0	3683

TRINITY_DN15285_c1_g1_i2	Glyma.06G152500.2	100	157	3	68	2568	44	2544	0	3683

TRINITY_DN14913_c0_g2_i1	Glyma.05G117500.1	95.94594595	290	23	182	2674	55	2541	0	2815

TRINITY_DN16082_c0_g1_i5	Glyma.08G124400.1	96.59763314	267	6	173	2674	2550	49	0	3097

TRINITY_DN16195_c0_g1_i2	Glyma.08G077500.1	95.08196721	197	15	52	2521	9	2508	0	3265

TRINITY_DN16216_c0_g3_i1	Glyma.11G012500.1	100	179	16	392	2858	142	2644	0	3365

TRINITY_DN16216_c0_g3_i1	Glyma.11G012500.2	97.84946237	179	16	392	2858	142	2644	0	3365

TRINITY_DN11259_c0_g1_i2	Glyma.08G187600.1	100	178	13	100	2582	1	2505	0	3487

TRINITY_DN12973_c0_g1_i2	Glyma.01G121400.2	96.8	166	5	66	2561	1258	3759	0	3618

TRINITY_DN16811_c0_g1_i1	Glyma.20G229500.3	96.36363636	158	22	1054	3520	2497	1	0	3443

TRINITY_DN3163_c0_g1_i1	Glyma.01G213200.1	99.62264151	137	6	600	3093	2494	1	0	3714

TRINITY_DN15345_c0_g1_i1	Glyma.12G099500.1	96.98492462	397	31	554	3038	158	2642	0	2169

TRINITY_DN15345_c0_g1_i1	Glyma.12G099500.4	97.74647887	397	31	554	3038	158	2642	0	2169

TRINITY_DN15345_c0_g1_i2	Glyma.12G099500.1	97.27272727	397	31	360	2844	158	2642	0	2169

TRINITY_DN15345_c0_g1_i2	Glyma.12G099500.4	95.67901235	397	31	360	2844	158	2642	0	2169

TRINITY_DN16082_c0_g1_i10	Glyma.08G202000.6	98.13084112	292	17	188	2657	2544	42	0	2769

TRINITY_DN16082_c0_g1_i8	Glyma.08G124400.1	97.50297265	282	6	173	2674	2550	49	0	3025

TRINITY_DN16082_c0_g1_i13	Glyma.08G202000.6	96.64948454	268	17	142	2611	2544	42	0	2902

TRINITY_DN15648_c0_g2_i3	Glyma.20G234500.1	97.25274725	226	6	223	2724	3432	931	0	3336

TRINITY_DN15648_c0_g2_i3	Glyma.20G234500.5	97.25274725	226	6	223	2724	3387	886	0	3336

TRINITY_DN15648_c0_g2_i3	Glyma.20G234500.3	97.36842105	226	6	223	2724	3345	844	0	3336

TRINITY_DN14622_c0_g1_i7	Glyma.10G179700.1	97.2972973	195	16	78	2573	97	2580	0	3352

TRINITY_DN14622_c0_g1_i7	Glyma.10G179700.2	98.91304348	195	16	78	2573	88	2571	0	3352

TRINITY_DN4278_c0_g1_i1	Glyma.09G224000.1	95.39170507	152	0	417	2921	2505	1	0	3784

TRINITY_DN15836_c0_g2_i1	Glyma.18G059700.1	100	311	12	269	2767	200	2692	0	2798

TRINITY_DN1088_c0_g1_i1	Glyma.03G214900.2	100	254	13	112	2597	2504	7	0	3062

TRINITY_DN16082_c0_g1_i11	Glyma.08G124400.1	98.98989899	221	4	173	2674	2550	49	0	3374

TRINITY_DN16415_c1_g1_i1	Glyma.U018900.1	98.7012987	221	5	1153	3656	2511	17	0	3341

TRINITY_DN16415_c1_g1_i10	Glyma.U018900.1	100	221	5	746	3249	2511	17	0	3341

TRINITY_DN9205_c0_g1_i4	Glyma.07G148100.1	98.63013699	221	22	472	2931	2502	1	0	3103

TRINITY_DN9205_c0_g1_i4	Glyma.07G148100.8	97.46192893	219	28	472	2931	2478	1	0	2959

TRINITY_DN14913_c0_g1_i1	Glyma.05G117500.1	95.93495935	212	19	182	2674	55	2541	0	3269

TRINITY_DN16050_c0_g1_i1	Glyma.18G288600.2	97.10467706	180	7	281	2780	3294	795	0	3576

TRINITY_DN16050_c0_g1_i1	Glyma.18G288600.1	97.10743802	180	7	281	2780	3294	795	0	3576

TRINITY_DN37264_c0_g1_i1	Glyma.17G244300.1	98.55072464	144	2	361	2863	3588	1086	0	3814

TRINITY_DN16082_c0_g1_i12	Glyma.08G201900.1	96.8503937	504	40	121	2587	2517	36	0	1461

TRINITY_DN10755_c0_g1_i2	Glyma.17G245500.1	96.32352941	421	42	117	2585	1	2463	0	1812

TRINITY_DN16440_c0_g1_i2	Glyma.03G053100.1	97.14285714	377	9	801	3291	2500	1	0	2436

TRINITY_DN16353_c0_g1_i3	Glyma.10G284500.1	96.35499208	307	24	389	2874	1006	3491	0	2702

TRINITY_DN13698_c1_g1_i2	Glyma.09G047300.3	96.55172414	268	31	354	2821	2489	1	0	2820

TRINITY_DN13698_c1_g1_i2	Glyma.09G047300.2	96.55172414	265	33	354	2821	2483	1	0	2798

TRINITY_DN15994_c0_g1_i3	Glyma.13G367300.2	98.27160494	257	4	455	2947	3333	835	0	3109

TRINITY_DN15068_c0_g1_i5	Glyma.04G080600.1	96.25603865	250	24	182	2653	2997	517	0	2894

TRINITY_DN16050_c0_g1_i2	Glyma.07G080400.1	98.39572193	180	1	281	2780	3144	642	0	3605

TRINITY_DN37264_c0_g1_i1	Glyma.14G081100.1	98.55072464	139	0	361	2863	3612	1110	0	3853

TRINITY_DN37264_c0_g1_i1	Glyma.14G081100.6	100	139	0	361	2863	3609	1107	0	3853

TRINITY_DN37264_c0_g1_i1	Glyma.14G081100.3	100	139	0	361	2863	2838	336	0	3853

TRINITY_DN37264_c0_g1_i1	Glyma.14G081100.4	100	139	0	361	2863	2838	336	0	3853

TRINITY_DN37264_c0_g1_i1	Glyma.14G081100.5	100	139	0	361	2863	2838	336	0	3853

TRINITY_DN16082_c0_g1_i12	Glyma.08G201900.2	100	504	40	121	2586	2481	1	0	1459

TRINITY_DN16076_c2_g3_i1	Glyma.17G179200.2	97.72727273	388	24	361	2850	2478	1	0	2239

TRINITY_DN16076_c2_g3_i1	Glyma.17G179200.3	95.75757576	388	24	361	2850	2478	1	0	2239

TRINITY_DN16076_c2_g3_i2	Glyma.17G179200.2	99.66777409	388	24	361	2850	2478	1	0	2239

TRINITY_DN16076_c2_g3_i2	Glyma.17G179200.3	99.66887417	388	24	361	2850	2478	1	0	2239

TRINITY_DN15836_c0_g2_i1	Glyma.11G167200.1	98.4375	319	13	271	2764	205	2698	0	2750

TRINITY_DN9800_c0_g1_i1	Glyma.13G054500.1	96.1038961	291	22	1	2490	886	3363	0	2776

TRINITY_DN16318_c0_g1_i3	Glyma.09G028000.1	95.73459716	231	9	521	3005	2694	219	0	3062

TRINITY_DN14961_c3_g1_i2	Glyma.06G204400.1	100	216	8	844	3327	1	2490	0	3230

TRINITY_DN14622_c0_g1_i6	Glyma.10G179700.6	96.36363636	185	12	193	2685	6	2484	0	3389

TRINITY_DN14933_c1_g2_i1	Glyma.15G167000.1	98.07692308	185	48	373	2787	715	3180	0	2800

TRINITY_DN4972_c0_g1_i1	Glyma.10G270300.1	100	184	2	85	2586	2520	22	0	3581

TRINITY_DN13959_c0_g1_i1	Glyma.10G292300.1	100	162	28	207	2657	3029	535	0	3349

TRINITY_DN13959_c0_g1_i2	Glyma.10G292300.1	96.55172414	162	28	207	2657	3029	535	0	3349

TRINITY_DN10873_c0_g1_i1	Glyma.04G017700.1	100	155	6	343	2842	2488	1	0	3659

TRINITY_DN15285_c1_g1_i2	Glyma.04G213800.2	96.49923896	144	5	68	2568	44	2532	0	3733

TRINITY_DN15285_c1_g1_i2	Glyma.04G213800.1	98.16513761	144	5	68	2568	44	2532	0	3733

TRINITY_DN16254_c0_g1_i10	Glyma.09G061200.4	99.0990991	555	65	1	2460	2576	114	0	1033

TRINITY_DN16254_c0_g1_i10	Glyma.09G061200.3	99.0990991	555	65	1	2460	2576	114	0	1033

TRINITY_DN16254_c0_g1_i10	Glyma.09G061200.1	96.21212121	555	65	1	2460	2576	114	0	1033

TRINITY_DN16254_c0_g1_i10	Glyma.09G061200.2	97.84946237	555	65	1	2460	2576	114	0	1033

TRINITY_DN16254_c0_g1_i10	Glyma.09G061200.5	97.84946237	555	65	1	2460	2576	114	0	1033

TRINITY_DN16332_c0_g3_i3	Glyma.06G042400.2	100	263	1	1	2501	2498	1	0	3142

TRINITY_DN16332_c0_g3_i3	Glyma.04G041300.2	100	257	1	1	2501	2498	1	0	3175

TRINITY_DN16444_c0_g1_i9	Glyma.16G204200.1	100	242	9	339	2823	651	3150	0	3168

TRINITY_DN16444_c0_g1_i9	Glyma.16G204200.2	97.5	242	9	339	2823	558	3057	0	3168

TRINITY_DN16444_c0_g1_i9	Glyma.16G204200.3	97.49478079	242	9	339	2823	21	2520	0	3168

TRINITY_DN16444_c0_g1_i8	Glyma.16G204200.1	100	240	9	339	2823	651	3150	0	3179

TRINITY_DN16444_c0_g1_i8	Glyma.16G204200.2	96.80170576	240	9	339	2823	558	3057	0	3179

TRINITY_DN16444_c0_g1_i8	Glyma.16G204200.3	100	240	9	339	2823	21	2520	0	3179

TRINITY_DN16444_c0_g1_i4	Glyma.16G204200.1	97.2027972	239	9	339	2823	651	3150	0	3184

TRINITY_DN16444_c0_g1_i4	Glyma.16G204200.2	100	239	9	339	2823	558	3057	0	3184

TRINITY_DN16444_c0_g1_i4	Glyma.16G204200.3	100	239	9	339	2823	21	2520	0	3184

TRINITY_DN16050_c0_g1_i1	Glyma.02G093500.1	100	190	2	281	2780	3321	822	0	3554

TRINITY_DN13197_c0_g1_i1	Glyma.07G056000.1	98.1878089	188	2	931	3430	471	2970	0	3565

TRINITY_DN12719_c0_g2_i5	Glyma.13G321300.49	99.02912621	174	20	1	2490	2451	1	0	3260

TRINITY_DN12719_c0_g2_i5	Glyma.13G321300.46	97.61904762	174	20	1	2490	2451	1	0	3260

TRINITY_DN12719_c0_g2_i5	Glyma.13G321300.15	97.61904762	174	20	1	2490	2451	1	0	3260

TRINITY_DN12719_c0_g2_i5	Glyma.13G321300.14	97.61904762	174	20	1	2490	2451	1	0	3260

TRINITY_DN12719_c0_g2_i7	Glyma.13G321300.49	100	174	20	1	2490	2451	1	0	3260

TRINITY_DN12719_c0_g2_i7	Glyma.13G321300.46	100	174	20	1	2490	2451	1	0	3260

TRINITY_DN12719_c0_g2_i7	Glyma.13G321300.15	100	174	20	1	2490	2451	1	0	3260

TRINITY_DN12719_c0_g2_i7	Glyma.13G321300.14	97.72727273	174	20	1	2490	2451	1	0	3260

TRINITY_DN16332_c0_g3_i3	Glyma.14G085200.2	100	138	0	1	2501	2501	1	0	3855

TRINITY_DN15040_c0_g1_i1	Glyma.13G099300.2	98.51851852	378	33	615	3096	2738	287	0	2097

TRINITY_DN16004_c1_g1_i6	Glyma.17G103700.1	98.95833333	326	11	229	2723	1	2492	0	2726

TRINITY_DN16004_c1_g1_i6	Glyma.13G156000.1	98.95833333	315	11	229	2723	1	2492	0	2787

TRINITY_DN15040_c0_g1_i2	Glyma.09G057800.1	100	282	28	605	3051	2946	455	0	2660

TRINITY_DN14670_c0_g1_i3	Glyma.13G065500.11	98.35466179	246	18	897	3390	1	2458	0	2944

TRINITY_DN14670_c0_g1_i3	Glyma.13G065500.12	96.90721649	244	20	897	3390	1	2455	0	2935

TRINITY_DN16318_c0_g1_i3	Glyma.15G133700.1	95.74468085	243	2	521	3005	2814	315	0	3173

TRINITY_DN15314_c0_g2_i3	Glyma.12G094500.1	97.54098361	215	19	641	3108	2704	226	0	3083

TRINITY_DN9378_c0_g1_i1	Glyma.09G191400.4	97.62470309	145	16	835	3314	2585	99	0	3600

TRINITY_DN15040_c0_g1_i2	Glyma.15G163900.1	100	269	37	605	3051	2937	455	0	2686

TRINITY_DN15040_c0_g1_i2	Glyma.15G163900.3	98.63013699	269	37	605	3051	2937	455	0	2686

TRINITY_DN16004_c1_g1_i6	Glyma.15G207900.1	98.95833333	219	5	229	2723	1	2495	0	3350

TRINITY_DN16444_c0_g1_i4	Glyma.17G111500.1	98.51301115	397	30	364	2832	970	3456	0	2156

TRINITY_DN16444_c0_g1_i9	Glyma.17G111500.1	97.25400458	397	30	364	2832	970	3456	0	2156

TRINITY_DN15068_c0_g1_i5	Glyma.06G082300.1	95.92760181	262	19	182	2653	3000	517	0	2904

TRINITY_DN14961_c1_g1_i1	Glyma.08G266700.2	97.04918033	141	1	179	2676	3174	692	0	3735

TRINITY_DN14961_c1_g1_i1	Glyma.08G266700.1	97.05882353	141	1	179	2676	3174	692	0	3735

TRINITY_DN14961_c1_g1_i1	Glyma.08G266700.3	97.05882353	141	2	179	2676	3171	692	0	3716

TRINITY_DN8227_c0_g1_i1	Glyma.08G116000.1	100	257	15	344	2821	4008	1534	0	2922

TRINITY_DN8227_c0_g1_i1	Glyma.08G116000.2	100	257	15	344	2821	3669	1195	0	2922

TRINITY_DN5432_c0_g1_i1	Glyma.12G164200.1	96.68049793	205	7	52	2546	29	2514	0	3391

TRINITY_DN5432_c0_g1_i1	Glyma.12G164200.3	96.68049793	203	8	52	2546	29	2511	0	3384

TRINITY_DN11922_c0_g1_i1	Glyma.10G060000.1	95.65217391	178	14	167	2657	2473	1	0	3432

TRINITY_DN11922_c0_g1_i1	Glyma.13G146700.1	95.65217391	168	17	167	2657	2467	1	0	3448

TRINITY_DN14961_c1_g1_i1	Glyma.16G102300.1	97.05882353	139	1	179	2675	3201	708	0	3821

TRINITY_DN14961_c1_g1_i1	Glyma.16G102300.2	97.04142012	139	2	179	2675	3198	708	0	3803

TRINITY_DN11922_c0_g2_i2	Glyma.10G060000.1	97.22222222	128	9	167	2660	2473	1	0	3727

TRINITY_DN11922_c0_g2_i2	Glyma.13G146700.1	95.91836735	116	12	167	2660	2467	1	0	3755

TRINITY_DN16254_c0_g1_i10	Glyma.15G167500.1	97.84946237	566	61	1	2460	2564	105	0	1009

TRINITY_DN16254_c0_g1_i10	Glyma.15G167500.3	97.84946237	566	61	1	2460	2564	105	0	1009

TRINITY_DN16254_c0_g1_i10	Glyma.15G167500.2	97.18875502	566	61	1	2460	2564	105	0	1009

TRINITY_DN16254_c0_g1_i10	Glyma.15G167500.4	97.18969555	566	61	1	2460	2564	105	0	1009

TRINITY_DN16444_c0_g1_i8	Glyma.17G111500.1	100	407	28	364	2832	970	3456	0	2122

TRINITY_DN16430_c0_g2_i3	Glyma.01G209300.1	100	290	2	111	2603	159	2654	0	2983

TRINITY_DN16268_c1_g3_i3	Glyma.11G116800.1	100	206	8	331	2818	2490	1	0	3378

TRINITY_DN4972_c0_g1_i1	Glyma.20G121000.1	100	172	2	85	2580	2499	7	0	3637

TRINITY_DN16082_c0_g1_i12	Glyma.06G086600.1	96.8503937	138	6	123	2591	2536	41	0	3670

TRINITY_DN16254_c0_g1_i5	Glyma.09G061200.4	100	571	53	1	2463	2576	114	0	1031

TRINITY_DN16254_c0_g1_i5	Glyma.09G061200.3	100	571	53	1	2463	2576	114	0	1031

TRINITY_DN16254_c0_g1_i5	Glyma.09G061200.1	96.93877551	571	53	1	2463	2576	114	0	1031

TRINITY_DN16254_c0_g1_i5	Glyma.09G061200.2	96.93877551	571	53	1	2463	2576	114	0	1031

TRINITY_DN16254_c0_g1_i5	Glyma.09G061200.5	95.45454545	571	53	1	2463	2576	114	0	1031

TRINITY_DN16082_c0_g1_i7	Glyma.08G202000.3	97.6969697	244	8	188	2671	2532	40	0	3171

TRINITY_DN16082_c0_g1_i9	Glyma.08G202000.3	97.61904762	220	8	142	2625	2532	40	0	3304

TRINITY_DN16447_c1_g1_i1	Glyma.08G162300.4	95.23809524	212	8	934	3421	1995	4483	0	3349

TRINITY_DN16447_c1_g1_i1	Glyma.08G162300.1	97.05882353	212	8	934	3421	1995	4483	0	3349

TRINITY_DN16082_c0_g1_i12	Glyma.04G085000.1	100	138	4	121	2591	2535	41	0	3688

TRINITY_DN16254_c0_g1_i5	Glyma.15G167500.1	99.53488372	576	56	1	2463	2564	105	0	994

TRINITY_DN16254_c0_g1_i5	Glyma.15G167500.3	95.89442815	576	56	1	2463	2564	105	0	994

TRINITY_DN16254_c0_g1_i5	Glyma.15G167500.2	95.89442815	576	56	1	2463	2564	105	0	994

TRINITY_DN16254_c0_g1_i5	Glyma.15G167500.4	98.63945578	576	56	1	2463	2564	105	0	994

TRINITY_DN13209_c1_g1_i2	Glyma.15G214500.4	99.05992949	414	24	695	3163	2479	11	0	1989

TRINITY_DN13209_c1_g1_i2	Glyma.15G214500.5	99.28571429	414	24	695	3163	2479	11	0	1989

TRINITY_DN9800_c0_g1_i1	Glyma.19G032700.1	100	306	24	1	2490	886	3342	0	2645

TRINITY_DN16082_c0_g1_i10	Glyma.07G016700.1	100	290	17	188	2657	2526	36	0	2824

TRINITY_DN10651_c0_g1_i1	Glyma.08G106400.2	97.8021978	281	9	317	2801	3416	927	0	2964

TRINITY_DN8227_c0_g1_i1	Glyma.05G158300.1	100	255	15	344	2821	2973	499	0	2966

TRINITY_DN15815_c0_g2_i1	Glyma.12G147000.1	100	230	10	1489	3968	3081	590	0	3229

TRINITY_DN15815_c0_g2_i1	Glyma.12G147000.3	100	230	10	1489	3968	3078	587	0	3229

TRINITY_DN15815_c0_g2_i1	Glyma.12G147000.2	99.35064935	230	10	1489	3968	2751	260	0	3229

TRINITY_DN16447_c1_g1_i1	Glyma.08G162300.3	97.05882353	212	8	934	3421	1995	4480	0	3341

TRINITY_DN16447_c1_g1_i1	Glyma.08G162300.2	95.56650246	212	8	934	3421	1995	4480	0	3341

TRINITY_DN16004_c1_g1_i6	Glyma.04G075100.2	97.10144928	555	37	248	2720	17	2486	0	1251

TRINITY_DN16004_c1_g1_i6	Glyma.04G075100.1	97.10144928	555	37	248	2720	17	2486	0	1251

TRINITY_DN15314_c0_g2_i2	Glyma.13G313000.1	97.09821429	451	65	793	3235	2726	293	0	1400

TRINITY_DN16444_c0_g1_i8	Glyma.13G159800.1	97.05882353	410	30	359	2826	821	3288	0	2010

TRINITY_DN16082_c0_g1_i10	Glyma.08G202000.3	98.13084112	293	12	188	2657	2532	42	0	2819

TRINITY_DN15068_c0_g2_i1	Glyma.17G190900.1	100	228	27	853	3295	2465	1	0	2837

TRINITY_DN13968_c0_g3_i3	Glyma.13G243600.1	99.25187032	213	10	1827	4308	1476	3960	0	3301

TRINITY_DN13968_c0_g3_i3	Glyma.13G243600.2	97.69874477	213	10	1827	4308	1167	3651	0	3301

TRINITY_DN16447_c1_g1_i1	Glyma.15G265300.1	98.52941176	208	5	934	3421	1992	4480	0	3393

TRINITY_DN16447_c1_g1_i1	Glyma.15G265300.2	96.50485437	208	5	934	3421	1914	4402	0	3393

TRINITY_DN16447_c1_g1_i1	Glyma.15G265300.3	100	208	5	934	3421	1068	3556	0	3393

TRINITY_DN2620_c0_g1_i1	Glyma.12G027700.5	100	205	7	354	2834	2490	1	0	3371

TRINITY_DN12719_c0_g2_i1	Glyma.13G321300.44	100	176	20	1	2482	2448	6	0	3234

TRINITY_DN12719_c0_g2_i1	Glyma.13G321300.32	100	176	20	1	2482	2448	6	0	3234

TRINITY_DN12719_c0_g2_i1	Glyma.13G321300.12	100	176	20	1	2482	2448	6	0	3234

TRINITY_DN12719_c0_g2_i3	Glyma.13G321300.44	95.44072948	176	20	1	2482	2448	6	0	3234

TRINITY_DN12719_c0_g2_i3	Glyma.13G321300.32	100	176	20	1	2482	2448	6	0	3234

TRINITY_DN12719_c0_g2_i3	Glyma.13G321300.12	100	176	20	1	2482	2448	6	0	3234

TRINITY_DN12892_c0_g4_i1	Glyma.11G103100.1	100	164	2	305	2794	139	2625	0	3637

TRINITY_DN12892_c0_g4_i1	Glyma.11G103100.4	100	164	2	305	2794	139	2625	0	3637

TRINITY_DN12257_c0_g1_i2	Glyma.09G021400.1	97.5	153	3	278	2746	1	2490	0	3581

TRINITY_DN12257_c0_g1_i2	Glyma.15G127700.1	97.5	151	4	278	2746	1	2490	0	3592

TRINITY_DN15040_c0_g1_i3	Glyma.15G163900.6	98.71794872	374	47	429	2858	2926	455	0	2001

TRINITY_DN15040_c0_g1_i3	Glyma.15G163900.4	100	374	47	429	2858	2926	455	0	2001

TRINITY_DN15040_c0_g1_i3	Glyma.15G163900.5	100	374	47	429	2858	2926	455	0	2001

TRINITY_DN15282_c0_g1_i3	Glyma.05G150200.1	98.13432836	311	29	1251	3700	2474	1	0	2492

TRINITY_DN15282_c0_g1_i4	Glyma.05G150200.1	97.77777778	311	29	1320	3769	2474	1	0	2492

TRINITY_DN15282_c0_g1_i5	Glyma.05G150200.1	100	311	29	706	3155	2474	1	0	2492

TRINITY_DN15282_c0_g1_i3	Glyma.05G150200.2	100	309	33	1251	3700	2468	1	0	2464

TRINITY_DN15282_c0_g1_i4	Glyma.05G150200.2	100	309	33	1320	3769	2468	1	0	2464

TRINITY_DN15282_c0_g1_i5	Glyma.05G150200.2	99.03846154	309	33	706	3155	2468	1	0	2464

TRINITY_DN13968_c0_g3_i1	Glyma.13G243600.1	100	308	9	1827	4311	1476	3960	0	2806

TRINITY_DN13968_c0_g3_i1	Glyma.13G243600.2	97.36842105	308	9	1827	4311	1167	3651	0	2806

TRINITY_DN16065_c0_g1_i2	Glyma.13G244300.1	98.88059701	291	43	299	2731	1	2475	0	2499

TRINITY_DN16082_c0_g1_i13	Glyma.07G016700.1	100	266	14	142	2611	2526	36	0	2979

TRINITY_DN14843_c0_g1_i1	Glyma.14G164900.1	96.875	434	16	239	2707	161	2646	0	2021

TRINITY_DN2451_c0_g1_i1	Glyma.01G077200.1	99.14529915	277	24	451	2928	5106	2656	0	2723

TRINITY_DN2451_c0_g1_i1	Glyma.01G077200.2	96.90140845	277	24	451	2928	4773	2323	0	2723

TRINITY_DN16082_c0_g1_i13	Glyma.08G202000.3	96.64902998	273	9	142	2611	2532	42	0	2952

TRINITY_DN13734_c0_g1_i2	Glyma.01G191100.2	99.31034483	246	10	1	2472	661	3144	0	3070

TRINITY_DN13734_c0_g1_i2	Glyma.01G191100.1	99.09365559	246	10	1	2472	661	3144	0	3070

TRINITY_DN10786_c0_g1_i1	Glyma.10G037300.1	100	226	6	866	3354	3226	741	0	3302

TRINITY_DN15314_c0_g2_i1	Glyma.11G179000.1	96.875	218	18	276	2742	2648	193	0	3011

TRINITY_DN9205_c0_g1_i4	Glyma.07G148100.9	100	218	22	485	2931	2489	1	0	3096

TRINITY_DN9205_c0_g1_i4	Glyma.18G199400.4	100	214	15	485	2931	2489	1	0	3118

TRINITY_DN9205_c0_g1_i4	Glyma.18G199400.3	97.46192893	210	25	485	2931	2465	1	0	2985

TRINITY_DN16447_c1_g1_i1	Glyma.15G265300.4	98.01980198	208	5	936	3421	14	2500	0	3389

TRINITY_DN15314_c0_g2_i2	Glyma.11G179000.1	98.33333333	193	18	848	3314	2648	193	0	3149

TRINITY_DN14779_c0_g1_i3	Glyma.04G034700.1	98.24561404	323	16	205	2686	2642	171	0	2641

TRINITY_DN14779_c0_g1_i3	Glyma.04G034700.2	98.27586207	323	16	205	2686	2642	171	0	2641

TRINITY_DN16065_c0_g1_i2	Glyma.15G069400.1	95.33333333	293	42	299	2731	1	2478	0	2529

TRINITY_DN10833_c0_g1_i2	Glyma.18G244800.1	97.6744186	230	9	270	2743	2540	61	0	3157

TRINITY_DN14961_c3_g1_i2	Glyma.06G204400.2	100	216	7	844	3327	1	2478	0	3286

TRINITY_DN14171_c0_g1_i2	Glyma.15G150900.1	100	209	1	113	2602	1	2487	0	3421

TRINITY_DN14171_c0_g1_i3	Glyma.15G150900.1	100	208	6	113	2591	1	2487	0	3356

TRINITY_DN12719_c0_g2_i6	Glyma.13G321300.43	98.4375	170	30	1	2455	2430	6	0	3011

TRINITY_DN12719_c0_g2_i6	Glyma.13G321300.31	99.15254237	170	30	1	2455	2430	6	0	3011

TRINITY_DN12719_c0_g2_i6	Glyma.13G321300.11	100	170	30	1	2455	2430	6	0	3011

TRINITY_DN12719_c0_g2_i8	Glyma.13G321300.43	96.07843137	170	30	1	2455	2430	6	0	3011

TRINITY_DN12719_c0_g2_i8	Glyma.13G321300.31	96.07843137	170	30	1	2455	2430	6	0	3011

TRINITY_DN12719_c0_g2_i8	Glyma.13G321300.11	96.07843137	170	30	1	2455	2430	6	0	3011

TRINITY_DN13959_c0_g1_i1	Glyma.10G292300.2	100	163	20	207	2657	3017	535	0	3398

TRINITY_DN13959_c0_g1_i2	Glyma.10G292300.2	100	163	20	207	2657	3017	535	0	3398

TRINITY_DN14779_c0_g1_i2	Glyma.04G034700.1	95.38461538	298	11	100	2585	2643	171	0	2822

TRINITY_DN14779_c0_g1_i2	Glyma.04G034700.2	95.4248366	298	11	100	2585	2643	171	0	2822

TRINITY_DN15278_c1_g1_i2	Glyma.15G091000.2	95.55555556	295	32	987	3461	2449	2	0	2606

TRINITY_DN15815_c0_g2_i1	Glyma.06G254500.1	100	234	9	1489	3968	3063	578	0	3223

TRINITY_DN9361_c0_g1_i1	Glyma.10G245700.1	95.25316456	189	6	216	2697	2485	1	0	3478

TRINITY_DN14622_c0_g1_i3	Glyma.10G179700.6	97.72370487	186	8	193	2672	6	2484	0	3443

TRINITY_DN14779_c0_g1_i4	Glyma.17G246600.1	98.27586207	143	5	101	2579	2690	206	0	3714

TRINITY_DN15278_c1_g1_i2	Glyma.15G091000.3	100	297	30	987	3461	2452	2	0	2627

TRINITY_DN15040_c0_g1_i2	Glyma.15G163900.6	95.23809524	269	37	616	3051	2926	455	0	2665

TRINITY_DN15040_c0_g1_i2	Glyma.15G163900.4	100	269	37	616	3051	2926	455	0	2665

TRINITY_DN15040_c0_g1_i2	Glyma.15G163900.5	96	269	37	616	3051	2926	455	0	2665

TRINITY_DN16082_c0_g1_i13	Glyma.07G016500.1	100	246	11	142	2611	2517	36	0	3077

TRINITY_DN11475_c0_g1_i1	Glyma.11G244500.1	96.52173913	230	20	12	2459	1	2481	0	3016

TRINITY_DN13734_c0_g1_i4	Glyma.01G191100.2	95.89041096	202	6	1	2478	661	3144	0	3386

TRINITY_DN13734_c0_g1_i4	Glyma.01G191100.1	96	202	6	1	2478	661	3144	0	3386

TRINITY_DN4194_c0_g2_i1	Glyma.08G041800.1	100	167	8	171	2648	4	2487	0	3579

TRINITY_DN13734_c0_g1_i1	Glyma.01G191100.2	100	156	6	1	2478	661	3144	0	3640

TRINITY_DN13734_c0_g1_i1	Glyma.01G191100.1	100	156	6	1	2478	661	3144	0	3640

TRINITY_DN14779_c0_g1_i4	Glyma.09G114500.2	99.06542056	142	2	101	2579	2690	206	0	3731

TRINITY_DN14779_c0_g1_i4	Glyma.09G114500.1	98.24561404	142	2	101	2579	2690	206	0	3731

TRINITY_DN16082_c0_g1_i10	Glyma.07G016500.1	100	278	9	188	2657	2517	36	0	2911

TRINITY_DN14750_c0_g1_i1	Glyma.13G256100.1	95.21044993	247	14	490	2961	2481	1	0	3088

TRINITY_DN11681_c0_g1_i2	Glyma.04G055700.3	98.92086331	219	11	404	2874	26	2508	0	3251

TRINITY_DN12878_c0_g1_i3	Glyma.18G025500.1	100	215	14	33	2518	1	2465	0	3253

TRINITY_DN14961_c3_g1_i2	Glyma.04G161000.4	100	206	3	844	3327	1	2481	0	3393

TRINITY_DN14961_c3_g1_i2	Glyma.04G161000.3	100	206	3	844	3327	1	2481	0	3393

TRINITY_DN14961_c3_g1_i2	Glyma.04G161000.1	100	206	3	844	3327	1	2481	0	3393

TRINITY_DN14961_c3_g1_i2	Glyma.04G161000.2	100	206	3	844	3327	1	2481	0	3393

TRINITY_DN12514_c0_g1_i2	Glyma.08G334000.1	95.75163399	140	0	476	2962	55	2541	0	3818

TRINITY_DN12514_c0_g1_i2	Glyma.18G067200.1	100	135	0	476	2962	55	2541	0	3845

TRINITY_DN14779_c0_g1_i1	Glyma.17G246600.1	98.38709677	104	4	204	2680	2691	206	0	3945

TRINITY_DN12634_c0_g2_i2	Glyma.04G220400.1	98.24561404	258	7	183	2662	2542	60	0	3103

TRINITY_DN12256_c0_g1_i1	Glyma.13G181800.1	98.92473118	194	19	410	2873	120	2598	0	3328

TRINITY_DN13648_c0_g1_i1	Glyma.15G046000.1	95.4954955	130	2	1	2485	462	2946	0	3858

TRINITY_DN14779_c0_g1_i1	Glyma.09G114500.2	98.85057471	102	1	204	2680	2691	206	0	3967

TRINITY_DN14779_c0_g1_i1	Glyma.09G114500.1	98.82352941	102	1	204	2680	2691	206	0	3967

TRINITY_DN36917_c0_g1_i1	Glyma.14G206300.1	97.43589744	457	45	9	2457	2456	5	0	1611

TRINITY_DN13209_c1_g1_i2	Glyma.15G214500.2	96.875	414	24	695	3163	2470	11	0	2030

TRINITY_DN13209_c1_g1_i2	Glyma.15G214500.3	98.7804878	414	24	695	3163	2470	11	0	2030

TRINITY_DN14750_c0_g1_i1	Glyma.15G058700.1	95.21126761	248	16	490	2961	2472	1	0	3048

TRINITY_DN16206_c0_g1_i3	Glyma.20G130400.2	99.33554817	229	3	132	2613	30	2508	0	3262

TRINITY_DN16206_c0_g1_i1	Glyma.20G130400.2	96.37681159	224	3	132	2613	30	2508	0	3290

TRINITY_DN15614_c1_g1_i1	Glyma.19G021800.1	95	213	13	1201	3654	2484	1	0	3203

TRINITY_DN15614_c1_g1_i1	Glyma.19G021800.2	100	213	13	1201	3654	2484	1	0	3203

TRINITY_DN15614_c1_g1_i1	Glyma.13G063500.1	96.44351464	201	20	1201	3654	2463	1	0	3133

TRINITY_DN16195_c0_g1_i2	Glyma.08G077500.2	95.08196721	198	9	52	2521	9	2487	0	3356

TRINITY_DN15549_c0_g1_i4	Glyma.08G055400.1	98.43137255	187	17	391	2842	3276	821	0	3153

TRINITY_DN14750_c0_g1_i2	Glyma.13G256100.1	95.18388792	140	7	490	2964	2481	1	0	3723

TRINITY_DN15795_c0_g1_i5	Glyma.16G042600.5	100	335	54	452	2884	2403	1	0	1879

TRINITY_DN16082_c0_g1_i10	Glyma.08G201900.1	97.53521127	283	7	188	2657	2517	36	0	2916

TRINITY_DN12865_c0_g1_i2	Glyma.05G202900.1	100	247	20	220	2671	1	2479	0	2979

TRINITY_DN13197_c0_g1_i2	Glyma.16G024900.1	98.21428571	226	24	473	2930	1	2464	0	3038

TRINITY_DN13197_c0_g1_i2	Glyma.16G024900.2	98.19277108	226	24	473	2930	1	2464	0	3038

TRINITY_DN14621_c1_g1_i4	Glyma.05G072300.1	96.59090909	224	13	122	2599	1	2466	0	3192

TRINITY_DN14621_c1_g1_i2	Glyma.05G072300.1	95	184	13	122	2599	1	2466	0	3413

TRINITY_DN13514_c0_g2_i1	Glyma.17G120500.1	96	125	3	1	2482	5898	3417	0	3869

TRINITY_DN16004_c1_g1_i6	Glyma.13G369300.1	96.51741294	542	28	254	2723	17	2483	0	1395

TRINITY_DN13734_c0_g1_i1	Glyma.05G102400.1	100	311	12	1	2478	691	3153	0	2700

TRINITY_DN15282_c0_g1_i3	Glyma.08G107100.2	100	307	19	1251	3700	2447	1	0	2438

TRINITY_DN15282_c0_g1_i3	Glyma.08G107100.3	98.83495146	307	19	1251	3700	2447	1	0	2438

TRINITY_DN15282_c0_g1_i3	Glyma.08G107100.1	100	307	19	1251	3700	2447	1	0	2438

TRINITY_DN15282_c0_g1_i4	Glyma.08G107100.2	98.55072464	307	19	1320	3769	2447	1	0	2438

TRINITY_DN15282_c0_g1_i4	Glyma.08G107100.3	97.77777778	307	19	1320	3769	2447	1	0	2438

TRINITY_DN15282_c0_g1_i4	Glyma.08G107100.1	97.5308642	307	19	1320	3769	2447	1	0	2438

TRINITY_DN15282_c0_g1_i5	Glyma.08G107100.2	100	307	19	706	3155	2447	1	0	2438

TRINITY_DN15282_c0_g1_i5	Glyma.08G107100.3	100	307	19	706	3155	2447	1	0	2438

TRINITY_DN15282_c0_g1_i5	Glyma.08G107100.1	97.23502304	307	19	706	3155	2447	1	0	2438

TRINITY_DN15795_c0_g1_i1	Glyma.16G042600.5	97.69820972	292	43	400	2844	2403	1	0	2206

TRINITY_DN15795_c0_g1_i3	Glyma.16G042600.5	98.62385321	292	43	574	3018	2403	1	0	2206

TRINITY_DN16082_c0_g1_i10	Glyma.08G201900.2	100	283	7	188	2656	2481	1	0	2915

TRINITY_DN16082_c0_g1_i13	Glyma.08G201900.1	97.6744186	252	6	142	2611	2517	36	0	3099

TRINITY_DN16082_c0_g1_i9	Glyma.08G201900.2	100	196	5	142	2621	2481	2	0	3461

TRINITY_DN4194_c0_g1_i1	Glyma.08G041800.1	98.85714286	166	8	171	2643	4	2482	0	3576

TRINITY_DN9431_c0_g1_i1	Glyma.05G223700.2	100	132	4	237	2718	8	2483	0	3803

TRINITY_DN13734_c0_g1_i1	Glyma.17G164600.1	100	314	11	1	2478	667	3129	0	2695

TRINITY_DN15594_c0_g1_i2	Glyma.05G068600.2	98.64253394	277	6	744	3221	2472	1	0	2959

TRINITY_DN13734_c0_g1_i4	Glyma.05G102400.1	98	265	10	1	2478	691	3153	0	2966

TRINITY_DN16082_c0_g1_i13	Glyma.08G201900.2	97.6744186	252	6	142	2610	2481	1	0	3097

TRINITY_DN16082_c0_g1_i7	Glyma.08G201900.2	100	231	3	188	2667	2481	2	0	3278

TRINITY_DN15629_c1_g1_i1	Glyma.08G302900.1	96.66666667	208	14	103	2563	12	2489	0	3269

TRINITY_DN15549_c0_g1_i4	Glyma.05G247000.4	98.98477157	188	13	391	2842	3270	821	0	3142

TRINITY_DN15549_c0_g1_i4	Glyma.05G247000.1	100	188	13	391	2842	3270	821	0	3142

TRINITY_DN15549_c0_g1_i4	Glyma.08G055400.3	95.76271186	188	15	391	2842	3273	821	0	3160

TRINITY_DN14622_c0_g1_i7	Glyma.20G210700.1	96.15384615	169	18	102	2573	1	2460	0	3441

TRINITY_DN14622_c0_g1_i7	Glyma.20G210700.2	99.29078014	169	18	102	2573	1	2460	0	3441

TRINITY_DN14750_c0_g1_i2	Glyma.15G058700.1	95.18518519	141	8	490	2964	2472	1	0	3692

TRINITY_DN16444_c0_g1_i8	Glyma.13G160000.1	97.91921665	411	24	375	2832	1062	3534	0	2104

TRINITY_DN14952_c0_g1_i1	Glyma.13G112300.1	96.2962963	322	6	36	2509	6	2482	0	2726

TRINITY_DN13734_c0_g1_i4	Glyma.17G164600.1	100	268	9	1	2478	667	3129	0	2961

TRINITY_DN13698_c1_g1_i2	Glyma.15G154900.2	97.13261649	253	24	354	2821	2459	1	0	2953

TRINITY_DN13698_c1_g1_i2	Glyma.15G154900.3	95.32293987	250	30	354	2821	2447	1	0	2892

TRINITY_DN16214_c0_g1_i2	Glyma.14G031600.1	97.46835443	196	11	500	2966	3495	1017	0	3393

TRINITY_DN14622_c0_g1_i7	Glyma.10G179700.5	100	190	16	102	2573	1	2460	0	3336

TRINITY_DN14622_c0_g1_i7	Glyma.10G179700.4	100	190	16	102	2573	1	2460	0	3336

TRINITY_DN8292_c0_g1_i2	Glyma.10G212000.1	98.52941176	178	5	204	2675	1	2478	0	3518

TRINITY_DN14563_c0_g1_i6	Glyma.18G218900.2	97.43589744	175	30	1467	3917	2881	428	0	3243

TRINITY_DN16444_c0_g1_i9	Glyma.13G160000.1	99	408	21	375	2832	1062	3534	0	2132

TRINITY_DN16444_c0_g1_i4	Glyma.13G160000.1	97.95918367	405	21	375	2832	1062	3534	0	2148

TRINITY_DN10755_c0_g1_i2	Glyma.17G245600.1	97.56097561	249	5	117	2585	1	2478	0	3127

TRINITY_DN14645_c0_g1_i2	Glyma.09G059000.1	96.36363636	246	15	264	2720	2478	1	0	3066

TRINITY_DN14645_c0_g1_i2	Glyma.09G059000.2	96.36363636	246	15	264	2720	2478	1	0	3066

TRINITY_DN14952_c0_g1_i2	Glyma.13G112300.1	96.30102041	244	0	36	2514	6	2484	0	3227

TRINITY_DN12865_c0_g1_i2	Glyma.08G010300.1	100	236	24	220	2673	1	2463	0	3005

TRINITY_DN14645_c0_g1_i1	Glyma.09G059000.1	95.48022599	217	15	264	2720	2478	1	0	3227

TRINITY_DN14645_c0_g1_i1	Glyma.09G059000.2	96.66666667	217	15	264	2720	2478	1	0	3227

TRINITY_DN16268_c1_g3_i3	Glyma.12G042600.1	98.14814815	212	13	356	2818	2472	1	0	3254

TRINITY_DN8849_c0_g1_i1	Glyma.09G031100.2	97.87234043	207	29	1	2460	2433	1	0	3011

TRINITY_DN8849_c0_g1_i1	Glyma.09G031100.1	97.36842105	207	29	1	2460	2433	1	0	3011

TRINITY_DN14231_c0_g1_i1	Glyma.15G253100.2	98.01699717	183	20	27	2475	45	2508	0	3273

TRINITY_DN14231_c0_g1_i2	Glyma.15G253100.2	98.03149606	183	20	27	2475	45	2508	0	3273

TRINITY_DN15278_c1_g1_i1	Glyma.15G091000.3	99.15254237	173	14	987	3462	2452	1	0	3426

TRINITY_DN16114_c0_g1_i4	Glyma.11G009500.2	95.40229885	154	24	136	2581	1	2467	0	3434

TRINITY_DN16114_c0_g1_i4	Glyma.11G009500.1	100	154	24	136	2581	1	2467	0	3434

TRINITY_DN13514_c0_g2_i1	Glyma.05G012400.1	96	132	0	4	2482	5892	3414	0	3847

TRINITY_DN15399_c0_g1_i2	Glyma.09G268300.2	98.31932773	221	0	207	2684	1	2478	0	3352

TRINITY_DN13734_c0_g1_i2	Glyma.05G102400.1	95.83333333	210	12	1	2472	691	3153	0	3277

TRINITY_DN16065_c0_g1_i5	Glyma.13G244300.1	97.33333333	163	1	299	2776	1	2475	0	3653

TRINITY_DN8292_c0_g1_i2	Glyma.20G179100.1	95.91836735	162	4	204	2675	1	2472	0	3602

TRINITY_DN16065_c0_g1_i5	Glyma.15G069400.1	97.33333333	151	0	299	2776	1	2478	0	3740

TRINITY_DN15399_c0_g1_i1	Glyma.09G268300.2	98.7654321	139	2	207	2681	1	2478	0	3786

TRINITY_DN16181_c0_g1_i4	Glyma.17G248400.2	98.68421053	290	6	423	2894	2475	1	0	2922

TRINITY_DN10009_c0_g1_i2	Glyma.14G223300.1	95.44658494	267	8	47	2509	1	2472	0	2972

TRINITY_DN10009_c0_g1_i1	Glyma.14G223300.1	98.73417722	262	15	47	2498	1	2472	0	2929

TRINITY_DN10755_c0_g1_i2	Glyma.14G079500.1	97.56097561	245	5	114	2585	46	2520	0	3171

TRINITY_DN13734_c0_g1_i2	Glyma.17G164600.1	95.28301887	211	10	1	2472	667	3129	0	3282

TRINITY_DN15385_c0_g1_i3	Glyma.03G107700.1	96.90140845	203	19	1627	4071	2451	1	0	3075

TRINITY_DN15629_c1_g1_i1	Glyma.18G116800.1	95.74468085	201	2	96	2563	1	2477	0	3402

TRINITY_DN15385_c0_g1_i1	Glyma.03G107700.1	98.11320755	192	16	1571	4024	2451	1	0	3193

TRINITY_DN14042_c0_g1_i1	Glyma.15G042000.6	98.7804878	157	3	446	2921	1	2473	0	3672

TRINITY_DN14042_c0_g1_i1	Glyma.15G042000.7	97.00499168	157	3	446	2921	1	2473	0	3672

TRINITY_DN14042_c0_g1_i3	Glyma.15G042000.6	95.19230769	157	3	663	3138	1	2473	0	3672

TRINITY_DN14042_c0_g1_i3	Glyma.15G042000.7	98.16643159	157	3	663	3138	1	2473	0	3672

TRINITY_DN15399_c0_g1_i3	Glyma.09G268300.2	98.32635983	282	0	209	2684	3	2478	0	3011

TRINITY_DN15594_c0_g1_i5	Glyma.05G068600.2	97.97979798	275	8	744	3208	2472	1	0	2952

TRINITY_DN15353_c0_g1_i2	Glyma.09G259100.2	98.7704918	232	4	472	2946	2472	1	0	3254

TRINITY_DN15521_c0_g1_i5	Glyma.06G226700.1	100	213	9	134	2589	19	2492	0	3251

TRINITY_DN15399_c0_g1_i4	Glyma.09G268300.2	99.54954955	200	2	209	2681	3	2478	0	3445

TRINITY_DN15278_c1_g1_i1	Glyma.15G091000.2	99.12280702	173	13	987	3462	2449	1	0	3439

TRINITY_DN14042_c0_g1_i1	Glyma.13G332300.12	98.86363636	170	1	446	2921	1	2473	0	3611

TRINITY_DN14042_c0_g1_i1	Glyma.13G332300.5	98.91304348	170	1	446	2921	1	2473	0	3611

TRINITY_DN14042_c0_g1_i1	Glyma.13G332300.6	98.88888889	170	1	446	2921	1	2473	0	3611

TRINITY_DN14042_c0_g1_i3	Glyma.13G332300.12	95.52238806	170	1	663	3138	1	2473	0	3611

TRINITY_DN14042_c0_g1_i3	Glyma.13G332300.5	95.52238806	170	1	663	3138	1	2473	0	3611

TRINITY_DN14042_c0_g1_i3	Glyma.13G332300.6	95.52238806	170	1	663	3138	1	2473	0	3611

TRINITY_DN12256_c0_g1_i1	Glyma.07G194500.1	98.93617021	190	12	410	2873	120	2589	0	3415

TRINITY_DN5451_c0_g1_i1	Glyma.12G021900.3	96.77419355	180	9	534	2998	2443	4901	0	3406

TRINITY_DN16026_c0_g2_i2	Glyma.11G112700.3	96.82539683	178	0	895	3369	4	2478	0	3585

TRINITY_DN15841_c0_g1_i1	Glyma.12G074800.1	96.86706181	177	3	144	2618	1	2440	0	3363

TRINITY_DN15841_c0_g1_i2	Glyma.12G074800.1	95.64428312	177	3	144	2618	1	2440	0	3363

TRINITY_DN15841_c0_g1_i1	Glyma.12G074800.2	96.86847599	177	7	144	2618	1	2434	0	3325

TRINITY_DN15841_c0_g1_i2	Glyma.12G074800.2	95.64502876	177	7	144	2618	1	2434	0	3325

TRINITY_DN12719_c0_g2_i5	Glyma.13G321300.43	97.64705882	170	29	1	2440	2430	6	0	3079

TRINITY_DN12719_c0_g2_i5	Glyma.13G321300.31	97.05882353	170	29	1	2440	2430	6	0	3079

TRINITY_DN12719_c0_g2_i5	Glyma.13G321300.11	97.34513274	170	29	1	2440	2430	6	0	3079

TRINITY_DN12719_c0_g2_i7	Glyma.13G321300.43	96.46464646	170	29	1	2440	2430	6	0	3079

TRINITY_DN12719_c0_g2_i7	Glyma.13G321300.31	98.93617021	170	29	1	2440	2430	6	0	3079

TRINITY_DN12719_c0_g2_i7	Glyma.13G321300.11	95.34883721	170	29	1	2440	2430	6	0	3079

TRINITY_DN16026_c0_g2_i7	Glyma.11G112700.3	97.91666667	148	0	883	3357	4	2478	0	3751

TRINITY_DN16026_c0_g2_i8	Glyma.11G112700.3	100	148	0	883	3357	4	2478	0	3751

TRINITY_DN16245_c0_g3_i5	Glyma.06G196400.1	97.89473684	131	3	213	2684	2472	1	0	3807

TRINITY_DN36917_c0_g1_i1	Glyma.14G206200.2	96.44808743	479	36	9	2458	2447	1	0	1585

TRINITY_DN36917_c0_g1_i1	Glyma.14G206200.1	98.07692308	479	36	9	2458	2447	1	0	1585

TRINITY_DN16447_c1_g1_i8	Glyma.08G162300.1	96.34146341	272	29	2936	5346	3703	6164	0	2577

TRINITY_DN16447_c1_g1_i8	Glyma.08G162300.2	100	271	30	2936	5346	3703	6161	0	2564

TRINITY_DN1122_c0_g2_i1	Glyma.01G001200.1	98.66666667	262	32	288	2742	2416	1	0	2630

TRINITY_DN10961_c0_g1_i1	Glyma.11G256600.3	97.01492537	237	12	704	3167	1	2461	0	3118

TRINITY_DN15385_c0_g1_i3	Glyma.07G119000.2	100	211	15	1627	4071	2460	1	0	3133

TRINITY_DN15385_c0_g1_i3	Glyma.07G119000.1	100	211	15	1627	4071	2460	1	0	3133

TRINITY_DN15385_c0_g1_i3	Glyma.07G119000.3	100	211	15	1627	4071	2460	1	0	3133

TRINITY_DN15521_c0_g1_i5	Glyma.12G155900.1	97.5	207	7	132	2589	17	2489	0	3323

TRINITY_DN15385_c0_g1_i1	Glyma.07G119000.2	98.50746269	199	12	1571	4024	2460	1	0	3258

TRINITY_DN15385_c0_g1_i1	Glyma.07G119000.1	98.50746269	199	12	1571	4024	2460	1	0	3258

TRINITY_DN15385_c0_g1_i1	Glyma.07G119000.3	98.50746269	199	12	1571	4024	2460	1	0	3258

TRINITY_DN9073_c0_g2_i1	Glyma.01G014700.4	97.10144928	174	17	531	2945	1	2460	0	3144

TRINITY_DN15674_c0_g1_i5	Glyma.14G058300.2	99.58158996	451	12	454	2919	4596	2131	0	1989

TRINITY_DN15570_c0_g1_i2	Glyma.19G132900.1	96.51162791	348	21	597	3046	1	2462	0	2431

TRINITY_DN12634_c0_g2_i2	Glyma.06G145500.1	98.24561404	269	5	183	2649	2542	73	0	3029

TRINITY_DN16245_c0_g3_i5	Glyma.06G196400.3	98.11320755	132	1	213	2684	2469	1	0	3814

TRINITY_DN9404_c0_g1_i1	Glyma.08G257600.1	98.52941176	114	1	404	2872	2472	1	0	3914

TRINITY_DN9404_c0_g1_i1	Glyma.18G282000.2	100	105	1	404	2872	2472	1	0	3964

TRINITY_DN16436_c0_g2_i4	Glyma.11G131600.1	97.63033175	245	5	534	2990	1	2469	0	3103

TRINITY_DN19024_c0_g1_i1	Glyma.02G257800.1	100	199	8	39	2509	2635	183	0	3345

TRINITY_DN11292_c0_g1_i1	Glyma.07G046100.1	96.96969697	136	5	388	2858	2502	38	0	3771

TRINITY_DN12815_c1_g1_i3	Glyma.18G095400.1	96.99570815	385	55	714	3153	2574	168	0	1827

TRINITY_DN16360_c0_g4_i1	Glyma.15G023800.1	95.0044603	286	10	398	2863	64	2523	0	2887

TRINITY_DN14670_c0_g1_i2	Glyma.13G065500.11	95.5801105	224	10	897	3360	1	2458	0	3205

TRINITY_DN14670_c0_g1_i2	Glyma.13G065500.12	98.38709677	222	12	897	3360	1	2455	0	3195

TRINITY_DN16181_c0_g1_i1	Glyma.04G034200.1	97.4025974	219	3	312	2780	2580	115	0	3315

TRINITY_DN16181_c0_g1_i1	Glyma.06G034400.2	100	212	3	312	2780	2580	115	0	3354

TRINITY_DN10814_c0_g2_i1	Glyma.15G038600.1	100	190	18	178	2632	1	2452	0	3295

TRINITY_DN10814_c0_g2_i1	Glyma.13G335700.1	100	188	10	178	2632	1	2464	0	3384

TRINITY_DN10814_c0_g2_i1	Glyma.13G335700.2	100	188	10	178	2632	1	2464	0	3384

TRINITY_DN9361_c0_g1_i1	Glyma.12G070100.1	95.25222552	185	6	234	2697	2467	1	0	3478

TRINITY_DN9361_c0_g1_i1	Glyma.12G070100.3	95.41666667	181	11	234	2697	2455	1	0	3422

TRINITY_DN14062_c0_g1_i4	Glyma.09G194200.1	95.43197617	170	11	1104	3561	2824	367	0	3465

TRINITY_DN14062_c0_g1_i4	Glyma.09G194200.3	99.82993197	168	15	1104	3561	2809	367	0	3378

TRINITY_DN12815_c1_g1_i4	Glyma.18G095400.1	97	323	53	714	3153	2574	168	0	2182

TRINITY_DN16360_c0_g4_i1	Glyma.13G350700.1	95	289	8	398	2863	67	2526	0	2881

TRINITY_DN14645_c0_g1_i2	Glyma.15G165100.1	96.3622291	243	10	264	2714	2484	16	0	3097

TRINITY_DN15521_c0_g1_i1	Glyma.06G226700.2	100	227	5	672	3135	428	2894	0	3256

TRINITY_DN11569_c0_g1_i1	Glyma.19G027200.2	98.5915493	213	8	794	3249	2468	1	0	3290

TRINITY_DN14645_c0_g1_i1	Glyma.15G165100.1	95.93301435	202	10	264	2714	2484	16	0	3325

TRINITY_DN36917_c0_g1_i1	Glyma.11G200300.2	100	198	9	3	2458	2468	1	0	3373

TRINITY_DN36917_c0_g1_i1	Glyma.11G200300.1	96.90265487	198	9	3	2458	2468	1	0	3373

TRINITY_DN15521_c0_g1_i4	Glyma.06G226700.2	97.05882353	191	5	672	3135	428	2894	0	3456

TRINITY_DN16181_c0_g1_i3	Glyma.04G034200.1	96.61016949	175	1	312	2780	2580	115	0	3570

TRINITY_DN16360_c0_g8_i1	Glyma.02G051700.1	100	171	0	249	2717	79	2547	0	3613

TRINITY_DN16181_c0_g1_i3	Glyma.06G034400.2	95.45454545	166	1	312	2780	2580	115	0	3620

TRINITY_DN13959_c0_g1_i3	Glyma.20G242100.3	99.43661972	162	9	387	2842	2811	347	0	3552

TRINITY_DN14244_c0_g4_i1	Glyma.15G044700.1	99.65397924	117	1	609	3077	2469	4	0	3892

TRINITY_DN15674_c0_g1_i3	Glyma.14G058300.2	99.10714286	452	12	969	3430	4592	2131	0	1977

TRINITY_DN16436_c0_g2_i4	Glyma.11G131700.1	99.53271028	236	0	523	2990	44	2511	0	3251

TRINITY_DN16436_c0_g2_i4	Glyma.11G131700.2	95.07323569	236	0	523	2990	23	2490	0	3251

TRINITY_DN16181_c0_g1_i3	Glyma.17G248400.3	99.07407407	235	4	319	2784	2579	114	0	3230

TRINITY_DN16181_c0_g1_i3	Glyma.17G248400.1	96.34146341	235	4	319	2784	2579	114	0	3230

TRINITY_DN16181_c0_g1_i3	Glyma.17G248400.4	99.13793103	235	5	319	2784	2567	114	0	3153

TRINITY_DN16181_c0_g1_i3	Glyma.14G077200.1	97.08029197	231	5	319	2785	2576	113	0	3245

TRINITY_DN12540_c0_g1_i1	Glyma.01G009400.1	95.49839228	187	5	420	2863	2877	416	0	3328

TRINITY_DN11254_c0_g1_i1	Glyma.07G034900.1	99.24242424	465	48	142	2573	2606	169	0	1567

TRINITY_DN12815_c1_g1_i2	Glyma.18G095400.1	97.01492537	364	56	714	3150	2574	168	0	1958

TRINITY_DN16237_c0_g1_i11	Glyma.06G164900.1	99.07407407	336	29	396	2841	90	2532	0	2403

TRINITY_DN16237_c0_g1_i5	Glyma.06G164900.1	98.27586207	336	29	396	2841	90	2532	0	2403

TRINITY_DN15768_c0_g1_i4	Glyma.03G177600.2	98.61111111	241	12	56	2489	1	2464	0	2988

TRINITY_DN16181_c0_g1_i1	Glyma.17G248400.3	100	201	2	319	2784	2579	114	0	3430

TRINITY_DN16181_c0_g1_i1	Glyma.17G248400.1	100	201	2	319	2784	2579	114	0	3430

TRINITY_DN16181_c0_g1_i1	Glyma.17G248400.4	97.86096257	201	3	319	2784	2567	114	0	3352

TRINITY_DN16181_c0_g1_i1	Glyma.14G077200.1	100	195	3	319	2785	2576	113	0	3456

TRINITY_DN15768_c0_g1_i1	Glyma.03G177600.2	100	176	5	56	2513	1	2464	0	3504

TRINITY_DN14062_c0_g1_i4	Glyma.09G194200.4	97.70114943	170	10	1104	3561	2821	367	0	3478

TRINITY_DN14062_c0_g1_i4	Glyma.09G194200.2	100	168	14	1104	3561	2806	367	0	3393

TRINITY_DN12815_c1_g1_i1	Glyma.18G095400.1	96.9837587	302	54	714	3150	2574	168	0	2313

TRINITY_DN36917_c0_g1_i1	Glyma.18G050700.3	100	184	5	3	2458	2465	1	0	3463

TRINITY_DN36917_c0_g1_i1	Glyma.18G050700.2	100	184	5	3	2458	2465	1	0	3463

TRINITY_DN36917_c0_g1_i1	Glyma.18G050700.1	100	184	5	3	2458	2465	1	0	3463

TRINITY_DN39666_c0_g1_i1	Glyma.20G205000.1	100	177	11	395	2845	2474	21	0	3398

TRINITY_DN12719_c0_g2_i6	Glyma.13G321300.42	96.02649007	174	21	1	2455	2406	6	0	3099

TRINITY_DN12719_c0_g2_i6	Glyma.13G321300.30	100	174	21	1	2455	2406	6	0	3099

TRINITY_DN12719_c0_g2_i6	Glyma.13G321300.10	98.4375	174	21	1	2455	2406	6	0	3099

TRINITY_DN12719_c0_g2_i8	Glyma.13G321300.42	95	174	21	1	2455	2406	6	0	3099

TRINITY_DN12719_c0_g2_i8	Glyma.13G321300.30	100	174	21	1	2455	2406	6	0	3099

TRINITY_DN12719_c0_g2_i8	Glyma.13G321300.10	99.69512195	174	21	1	2455	2406	6	0	3099

TRINITY_DN16114_c0_g1_i4	Glyma.01G233300.1	97.81704782	168	15	136	2581	1	2455	0	3422

TRINITY_DN5837_c0_g2_i1	Glyma.10G213200.1	100	151	19	368	2809	2442	1	0	3408

TRINITY_DN16561_c0_g1_i1	Glyma.10G023700.1	96.42857143	140	0	1	2466	4987	7452	0	3779

TRINITY_DN15016_c0_g1_i1	Glyma.16G182400.2	98.8372093	399	38	661	3093	22	2454	0	1929

TRINITY_DN14244_c0_g4_i1	Glyma.12G072700.1	96.20253165	264	10	609	3071	2457	1	0	3025

TRINITY_DN15847_c1_g2_i2	Glyma.14G034200.3	95.67567568	227	5	1	2459	194	2652	0	3217

TRINITY_DN16094_c0_g1_i1	Glyma.20G143200.2	95.41547278	223	6	282	2744	2454	1	0	3232

TRINITY_DN16094_c0_g1_i6	Glyma.20G143200.2	97.77777778	223	6	282	2744	2454	1	0	3232

TRINITY_DN15847_c1_g2_i4	Glyma.14G034200.3	96.58119658	219	5	1	2459	194	2652	0	3262

TRINITY_DN16214_c0_g1_i2	Glyma.02G282700.1	98.5915493	197	5	515	2966	3474	1011	0	3371

TRINITY_DN16214_c0_g1_i2	Glyma.02G282700.2	95.66395664	197	5	515	2966	3399	936	0	3371

TRINITY_DN16561_c0_g1_i1	Glyma.02G150200.1	96.36363636	132	2	1	2465	4987	7448	0	3801

TRINITY_DN16561_c0_g1_i1	Glyma.02G150200.2	96.42857143	132	2	1	2465	4204	6665	0	3801

TRINITY_DN16561_c0_g1_i1	Glyma.02G150200.3	96.42857143	132	2	1	2465	2956	5417	0	3801

TRINITY_DN16561_c0_g1_i1	Glyma.02G150200.4	96.42857143	132	2	1	2465	2956	5417	0	3801

TRINITY_DN16004_c1_g1_i5	Glyma.15G004300.1	100	535	35	91	2522	394	2843	0	1290

TRINITY_DN16237_c0_g1_i11	Glyma.04G200600.1	97.56097561	338	23	396	2841	90	2532	0	2425

TRINITY_DN16237_c0_g1_i5	Glyma.04G200600.1	96.82539683	338	23	396	2841	90	2532	0	2425

TRINITY_DN16447_c1_g1_i8	Glyma.15G265300.1	99.20948617	273	22	2936	5346	3700	6152	0	2625

TRINITY_DN16447_c1_g1_i8	Glyma.15G265300.2	97.84946237	273	22	2936	5346	3622	6074	0	2625

TRINITY_DN16447_c1_g1_i8	Glyma.15G265300.3	95	273	22	2936	5346	2776	5228	0	2625

TRINITY_DN16447_c1_g1_i8	Glyma.15G265300.4	95.40540541	273	22	2936	5346	1720	4172	0	2625

TRINITY_DN360_c0_g1_i1	Glyma.13G283400.1	97.84946237	265	8	105	2549	2457	1	0	2915

TRINITY_DN15980_c0_g2_i2	Glyma.20G207700.3	95.40229885	210	8	1022	3479	865	3310	0	3232

TRINITY_DN14912_c0_g1_i2	Glyma.18G126000.1	96.53465347	194	17	163	2611	127	2571	0	3256

TRINITY_DN14042_c0_g1_i1	Glyma.15G042000.2	97.59036145	157	3	446	2908	1	2460	0	3648

TRINITY_DN14042_c0_g1_i1	Glyma.15G042000.3	97.2027972	157	3	446	2908	1	2460	0	3648

TRINITY_DN14042_c0_g1_i2	Glyma.15G042000.8	95.66473988	157	3	663	3125	1	2460	0	3648

TRINITY_DN14042_c0_g1_i2	Glyma.15G042000.1	100	157	3	663	3125	1	2460	0	3648

TRINITY_DN14042_c0_g1_i3	Glyma.15G042000.2	98.18181818	157	3	663	3125	1	2460	0	3648

TRINITY_DN14042_c0_g1_i3	Glyma.15G042000.3	100	157	3	663	3125	1	2460	0	3648

TRINITY_DN14042_c0_g1_i4	Glyma.15G042000.8	100	157	3	446	2908	1	2460	0	3648

TRINITY_DN14042_c0_g1_i4	Glyma.15G042000.1	97.22222222	157	3	446	2908	1	2460	0	3648

TRINITY_DN9858_c0_g1_i3	Glyma.04G221900.1	98.33333333	120	1	309	2769	1	2464	0	3866

TRINITY_DN13828_c0_g2_i1	Glyma.07G252300.1	95.68965517	443	77	750	3159	475	2863	0	1273

TRINITY_DN16415_c1_g1_i1	Glyma.12G173700.1	100	378	6	1153	3610	2523	63	0	2409

TRINITY_DN16415_c1_g1_i10	Glyma.12G173700.1	95.06726457	378	6	746	3203	2523	63	0	2409

TRINITY_DN16065_c0_g1_i4	Glyma.13G244300.1	95.32163743	325	43	2	2405	30	2475	0	2257

TRINITY_DN14598_c0_g2_i1	Glyma.14G223100.6	98.26086957	196	23	480	2887	2765	322	0	2985

TRINITY_DN14598_c0_g2_i1	Glyma.14G223100.7	98.13084112	196	23	480	2887	2765	322	0	2985

TRINITY_DN9476_c0_g1_i1	Glyma.07G263100.2	95.23809524	175	3	787	3243	2466	24	0	3411

TRINITY_DN14042_c0_g1_i1	Glyma.13G332300.11	98.91304348	170	1	446	2908	1	2460	0	3587

TRINITY_DN14042_c0_g1_i1	Glyma.13G332300.2	98.91304348	170	1	446	2908	1	2460	0	3587

TRINITY_DN14042_c0_g1_i2	Glyma.13G332300.10	95.68965517	170	1	663	3125	1	2460	0	3587

TRINITY_DN14042_c0_g1_i2	Glyma.13G332300.8	95.68965517	170	1	663	3125	1	2460	0	3587

TRINITY_DN14042_c0_g1_i2	Glyma.13G332300.1	95.68965517	170	1	663	3125	1	2460	0	3587

TRINITY_DN14042_c0_g1_i3	Glyma.13G332300.11	96.80851064	170	1	663	3125	1	2460	0	3587

TRINITY_DN14042_c0_g1_i3	Glyma.13G332300.2	100	170	1	663	3125	1	2460	0	3587

TRINITY_DN14042_c0_g1_i4	Glyma.13G332300.10	97.65100671	170	1	446	2908	1	2460	0	3587

TRINITY_DN14042_c0_g1_i4	Glyma.13G332300.8	99.58158996	170	1	446	2908	1	2460	0	3587

TRINITY_DN14042_c0_g1_i4	Glyma.13G332300.1	100	170	1	446	2908	1	2460	0	3587

TRINITY_DN13959_c0_g1_i1	Glyma.20G242100.1	98.87640449	170	11	235	2657	2990	532	0	3323

TRINITY_DN13959_c0_g1_i2	Glyma.20G242100.1	99.4047619	170	11	235	2657	2990	532	0	3323

TRINITY_DN16360_c0_g8_i1	Glyma.16G131800.1	100	153	0	255	2717	85	2547	0	3701

TRINITY_DN14244_c0_g4_i1	Glyma.13G329100.2	99.61685824	122	0	609	3071	2463	1	0	3873

TRINITY_DN14244_c0_g4_i1	Glyma.13G329100.1	98.98534386	122	0	609	3071	2463	1	0	3873

TRINITY_DN16004_c1_g1_i5	Glyma.13G369300.1	98.66666667	509	39	95	2522	398	2843	0	1404

TRINITY_DN16436_c0_g2_i4	Glyma.12G055900.1	97.5	219	2	530	2990	48	2508	0	3321

TRINITY_DN16425_c0_g1_i3	Glyma.12G060600.4	98.30508475	191	3	639	3096	2947	487	0	3456

TRINITY_DN16425_c0_g1_i1	Glyma.12G060600.4	98.37837838	189	3	706	3163	2947	487	0	3467

TRINITY_DN14042_c0_g1_i1	Glyma.15G042000.4	97.20394737	157	3	446	2906	1	2458	0	3644

TRINITY_DN14042_c0_g1_i1	Glyma.15G042000.5	97.20670391	157	3	446	2906	1	2458	0	3644

TRINITY_DN14042_c0_g1_i3	Glyma.15G042000.4	100	157	3	663	3123	1	2458	0	3644

TRINITY_DN14042_c0_g1_i3	Glyma.15G042000.5	98	157	3	663	3123	1	2458	0	3644

TRINITY_DN12251_c0_g1_i1	Glyma.15G124200.1	100	129	1	170	2628	1	2462	0	3812

TRINITY_DN12251_c0_g1_i1	Glyma.09G018300.1	100	118	2	170	2628	1	2462	0	3873

TRINITY_DN11254_c0_g1_i1	Glyma.15G026300.1	99.26470588	516	49	143	2570	2572	142	0	1279

TRINITY_DN16065_c0_g1_i4	Glyma.15G069400.1	100	332	42	2	2405	30	2478	0	2259

TRINITY_DN15736_c0_g1_i4	Glyma.05G198700.1	95.42253521	312	11	266	2723	250	2695	0	2700

TRINITY_DN16436_c0_g2_i4	Glyma.11G131700.3	96	234	0	530	2990	6	2466	0	3249

TRINITY_DN19024_c0_g1_i1	Glyma.02G257800.2	100	199	8	39	2499	2443	1	0	3326

TRINITY_DN9858_c0_g1_i3	Glyma.06G143500.2	96.8879668	119	0	309	2769	1	2461	0	3886

TRINITY_DN9858_c0_g1_i3	Glyma.06G143500.1	96.89265537	119	0	309	2769	1	2461	0	3886

TRINITY_DN9858_c0_g1_i3	Glyma.06G143500.3	95.45454545	119	0	309	2769	1	2461	0	3886

TRINITY_DN9858_c0_g1_i3	Glyma.06G143500.4	95.45454545	119	0	309	2769	1	2461	0	3886

TRINITY_DN15795_c0_g1_i6	Glyma.16G042600.5	98.46153846	336	53	452	2860	2403	1	0	1984

TRINITY_DN15851_c0_g1_i3	Glyma.10G175900.2	100	329	33	226	2655	2430	1	0	2333

TRINITY_DN14146_c1_g1_i3	Glyma.20G228700.1	98.88888889	294	23	933	3383	2427	1	0	2643

TRINITY_DN14146_c1_g1_i3	Glyma.20G228700.4	100	294	23	933	3383	2427	1	0	2643

TRINITY_DN15345_c0_g3_i1	Glyma.12G099500.5	95.5801105	206	15	690	3134	1	2454	0	3265

TRINITY_DN15345_c0_g3_i1	Glyma.12G099500.2	99.29577465	206	15	690	3134	1	2454	0	3265

TRINITY_DN15345_c0_g3_i1	Glyma.12G099500.3	95.34246575	206	15	690	3134	1	2454	0	3265

TRINITY_DN13959_c0_g1_i1	Glyma.20G242100.2	98.87640449	170	10	235	2657	2987	532	0	3336

TRINITY_DN13959_c0_g1_i2	Glyma.20G242100.2	95.63218391	170	10	235	2657	2987	532	0	3336

TRINITY_DN15851_c0_g1_i3	Glyma.20G214600.5	96.36048527	324	30	226	2655	2427	1	0	2351

TRINITY_DN15851_c0_g1_i3	Glyma.20G214600.7	99.14893617	324	30	226	2655	2427	1	0	2351

TRINITY_DN15851_c0_g1_i3	Glyma.20G214600.3	98.03921569	324	30	226	2655	2427	1	0	2351

TRINITY_DN15851_c0_g1_i3	Glyma.20G214600.2	100	324	30	226	2655	2427	1	0	2351

TRINITY_DN15795_c0_g1_i2	Glyma.16G042600.5	96.07843137	293	38	400	2820	2403	1	0	2311

TRINITY_DN15795_c0_g1_i4	Glyma.16G042600.5	97.46835443	293	38	574	2994	2403	1	0	2311

TRINITY_DN3219_c0_g1_i1	Glyma.15G007800.1	100	221	27	181	2613	2445	1	0	3059

TRINITY_DN15549_c0_g1_i7	Glyma.08G055400.1	97.82608696	187	11	391	2806	3276	821	0	3208

TRINITY_DN14492_c1_g1_i2	Glyma.03G192300.3	96.7032967	168	1	243	2698	1	2459	0	3591

TRINITY_DN15679_c0_g1_i1	Glyma.09G088700.1	100	151	5	59	2512	1	2454	0	3640

TRINITY_DN15679_c0_g1_i8	Glyma.09G088700.1	100	151	5	1348	3801	1	2454	0	3640

TRINITY_DN16245_c0_g5_i1	Glyma.16G099700.3	98.73417722	548	66	1162	3565	2778	352	0	953

TRINITY_DN16373_c3_g5_i6	Glyma.15G005500.1	98.03921569	404	22	512	2950	3447	1015	0	2017

TRINITY_DN16373_c3_g5_i8	Glyma.15G005500.1	98.06629834	404	22	512	2950	3447	1015	0	2017

TRINITY_DN37980_c0_g1_i1	Glyma.08G176200.1	99.12280702	254	24	14	2435	1	2451	0	2854

TRINITY_DN37980_c0_g1_i1	Glyma.15G250200.1	100	234	21	14	2435	1	2448	0	2946

TRINITY_DN3219_c0_g1_i1	Glyma.13G365400.1	100	216	27	181	2613	2442	1	0	3077

TRINITY_DN11176_c0_g1_i1	Glyma.05G138900.1	100	189	15	119	2533	1	2454	0	3188

TRINITY_DN15841_c0_g1_i5	Glyma.12G074800.1	100	177	2	144	2601	1	2440	0	3443

TRINITY_DN15841_c0_g1_i6	Glyma.12G074800.1	97.77777778	177	2	144	2601	1	2440	0	3443

TRINITY_DN15841_c0_g1_i5	Glyma.12G074800.2	97.69585253	177	6	144	2601	1	2434	0	3404

TRINITY_DN15841_c0_g1_i6	Glyma.12G074800.2	96.66666667	177	6	144	2601	1	2434	0	3404

TRINITY_DN14062_c0_g1_i4	Glyma.01G146600.1	97.2972973	155	0	1104	3561	2887	430	0	3681

TRINITY_DN14062_c0_g1_i4	Glyma.01G146600.2	98.66666667	155	0	1104	3561	2821	364	0	3681

TRINITY_DN12048_c1_g1_i1	Glyma.12G039300.1	100	137	5	191	2644	1	2457	0	3747

TRINITY_DN11681_c0_g1_i1	Glyma.17G225800.1	100	334	11	552	2991	501	2949	0	2525

TRINITY_DN11681_c0_g1_i1	Glyma.17G225800.2	95.14563107	334	11	552	2991	60	2508	0	2525

TRINITY_DN14146_c1_g1_i3	Glyma.20G228700.2	98.88888889	295	22	933	3383	2424	1	0	2651

TRINITY_DN14244_c0_g4_i1	Glyma.U021500.1	96.20535714	269	9	609	3063	2457	9	0	2983

TRINITY_DN12048_c1_g1_i1	Glyma.11G113400.1	100	134	3	191	2644	1	2457	0	3775

TRINITY_DN16245_c0_g5_i2	Glyma.16G099700.3	96.51972158	541	64	1162	3565	2778	352	0	1014

TRINITY_DN15423_c0_g1_i2	Glyma.12G083500.1	100	504	37	450	2882	342	2779	0	1478

TRINITY_DN16360_c0_g8_i3	Glyma.11G073100.1	99.34210526	286	8	278	2724	94	2546	0	2874

TRINITY_DN15549_c0_g1_i7	Glyma.08G055400.3	99.07407407	188	9	391	2806	3273	821	0	3216

TRINITY_DN15549_c0_g1_i7	Glyma.05G247000.4	97.95221843	188	13	391	2806	3270	821	0	3197

TRINITY_DN15549_c0_g1_i7	Glyma.05G247000.1	97.95321637	188	13	391	2806	3270	821	0	3197

TRINITY_DN15679_c0_g1_i1	Glyma.15G196800.1	100	154	4	59	2512	1	2451	0	3637

TRINITY_DN15679_c0_g1_i8	Glyma.15G196800.1	100	154	4	1348	3801	1	2451	0	3637

TRINITY_DN36127_c0_g1_i1	Glyma.07G042000.1	99.15966387	137	2	393	2847	3	2457	0	3764

TRINITY_DN7932_c0_g1_i2	Glyma.19G208300.1	96.03960396	188	7	317	2764	2760	322	0	3343

TRINITY_DN5876_c0_g1_i1	Glyma.15G124900.1	100	511	64	89	2495	146	2567	0	1190

TRINITY_DN16206_c0_g1_i3	Glyma.20G130400.1	96.94267516	234	1	160	2613	178	2625	0	3197

TRINITY_DN16206_c0_g1_i3	Glyma.10G260900.1	96.94189602	229	1	160	2613	187	2634	0	3225

TRINITY_DN16206_c0_g1_i1	Glyma.20G130400.1	96.37681159	229	1	160	2613	178	2625	0	3225

TRINITY_DN16206_c0_g1_i1	Glyma.10G260900.1	96.37681159	214	1	160	2613	187	2634	0	3308

TRINITY_DN15088_c0_g1_i1	Glyma.08G124300.1	99.4011976	193	2	803	3256	1104	3554	0	3443

TRINITY_DN13758_c0_g1_i2	Glyma.14G219700.4	100	169	10	64	2493	1	2451	0	3421

TRINITY_DN13758_c0_g1_i2	Glyma.14G219700.3	100	168	11	64	2493	1	2448	0	3408

TRINITY_DN15521_c0_g1_i5	Glyma.13G266300.1	97.86324786	418	53	182	2589	82	2507	0	1749

TRINITY_DN8781_c0_g1_i1	Glyma.08G129000.1	98.65642994	350	17	533	2976	2527	87	0	2455

TRINITY_DN8781_c0_g1_i1	Glyma.08G129000.2	97.77777778	350	17	533	2976	2527	87	0	2455

TRINITY_DN8781_c0_g1_i1	Glyma.08G129000.3	96.92307692	350	17	533	2976	2527	87	0	2455

TRINITY_DN15636_c0_g1_i3	Glyma.12G128700.1	95.01246883	315	13	602	3031	2448	1	0	2604

TRINITY_DN15636_c0_g1_i3	Glyma.12G128700.2	96.15384615	315	13	602	3031	2448	1	0	2604

TRINITY_DN15636_c0_g1_i3	Glyma.12G128700.3	95.23809524	315	13	602	3031	2448	1	0	2604

TRINITY_DN15636_c0_g1_i1	Glyma.12G128700.1	95.11494253	282	13	602	3031	2448	1	0	2787

TRINITY_DN15636_c0_g1_i1	Glyma.12G128700.2	96.14457831	282	13	602	3031	2448	1	0	2787

TRINITY_DN15636_c0_g1_i1	Glyma.12G128700.3	96.14873838	282	13	602	3031	2448	1	0	2787

TRINITY_DN16360_c0_g4_i2	Glyma.08G193500.1	95.02222222	189	4	294	2744	82	2532	0	3458

TRINITY_DN16360_c0_g4_i2	Glyma.07G010400.1	95.00509684	179	4	294	2744	88	2538	0	3513

TRINITY_DN14492_c1_g1_i4	Glyma.03G192300.3	95.88235294	167	1	243	2692	1	2453	0	3585

TRINITY_DN14492_c1_g1_i4	Glyma.03G192300.1	98.63013699	167	1	243	2692	1	2453	0	3585

TRINITY_DN14492_c1_g1_i2	Glyma.19G192800.1	95.81993569	161	1	243	2692	1	2453	0	3618

TRINITY_DN14492_c1_g1_i4	Glyma.19G192800.2	100	161	1	243	2692	1	2453	0	3618

TRINITY_DN15867_c0_g3_i1	Glyma.17G052600.1	95.85253456	161	6	1	2440	450	2898	0	3528

TRINITY_DN15867_c0_g3_i1	Glyma.17G052600.2	100	161	6	1	2440	24	2472	0	3528

TRINITY_DN15314_c0_g2_i3	Glyma.12G188600.1	100	434	61	641	3029	2695	293	0	1400

TRINITY_DN11681_c0_g1_i1	Glyma.14G098900.1	97.18309859	334	7	552	2991	483	2928	0	2562

TRINITY_DN15867_c0_g3_i1	Glyma.13G106700.16	96.21451104	162	4	1	2440	450	2898	0	3533

TRINITY_DN15867_c0_g3_i1	Glyma.13G106700.11	100	162	4	1	2440	450	2898	0	3533

TRINITY_DN15867_c0_g3_i1	Glyma.13G106700.12	95.03968254	162	4	1	2440	450	2898	0	3533

TRINITY_DN15867_c0_g3_i1	Glyma.13G106700.13	98.61111111	162	4	1	2440	450	2898	0	3533

TRINITY_DN15867_c0_g3_i1	Glyma.13G106700.14	95	162	4	1	2440	450	2898	0	3533

TRINITY_DN15867_c0_g3_i1	Glyma.13G106700.7	95	162	4	1	2440	450	2898	0	3533

TRINITY_DN15867_c0_g3_i1	Glyma.13G106700.1	100	162	4	1	2440	450	2898	0	3533

TRINITY_DN15867_c0_g3_i1	Glyma.13G106700.2	100	162	4	1	2440	450	2898	0	3533

TRINITY_DN15867_c0_g3_i1	Glyma.13G106700.3	100	162	4	1	2440	450	2898	0	3533

TRINITY_DN15867_c0_g3_i1	Glyma.13G106700.4	98.55072464	162	4	1	2440	450	2898	0	3533

TRINITY_DN15867_c0_g3_i1	Glyma.13G106700.17	96.66666667	162	4	1	2440	450	2898	0	3533

TRINITY_DN13604_c0_g1_i1	Glyma.11G218000.1	100	146	2	147	2597	1	2451	0	3707

TRINITY_DN2525_c0_g2_i1	Glyma.12G135700.6	96.42857143	409	16	5	2448	778	3215	0	2132

TRINITY_DN4162_c0_g1_i1	Glyma.10G116800.2	99.3006993	233	15	198	2636	631	3060	0	3022

TRINITY_DN4162_c0_g1_i1	Glyma.10G116800.1	97.52066116	233	15	198	2636	631	3060	0	3022

TRINITY_DN5837_c0_g2_i1	Glyma.20G177800.1	100	175	10	368	2809	2436	1	0	3402

TRINITY_DN12719_c0_g2_i5	Glyma.13G321300.42	99.12280702	174	20	1	2440	2406	6	0	3168

TRINITY_DN12719_c0_g2_i5	Glyma.13G321300.30	95.78454333	174	20	1	2440	2406	6	0	3168

TRINITY_DN12719_c0_g2_i5	Glyma.13G321300.10	95.55555556	174	20	1	2440	2406	6	0	3168

TRINITY_DN12719_c0_g2_i7	Glyma.13G321300.42	99.86648865	174	20	1	2440	2406	6	0	3168

TRINITY_DN12719_c0_g2_i7	Glyma.13G321300.30	95.87628866	174	20	1	2440	2406	6	0	3168

TRINITY_DN12719_c0_g2_i7	Glyma.13G321300.10	95.87628866	174	20	1	2440	2406	6	0	3168

TRINITY_DN2525_c0_g2_i1	Glyma.12G214500.4	100	153	1	6	2447	911	3361	0	3620

TRINITY_DN10873_c0_g1_i1	Glyma.04G017700.2	95.8677686	152	6	343	2789	2443	2	0	3600

TRINITY_DN15034_c0_g1_i1	Glyma.06G292500.1	96.93094629	135	2	383	2833	2448	1	0	3759

TRINITY_DN15034_c0_g1_i1	Glyma.06G292500.2	99.66101695	135	2	383	2833	2448	1	0	3759

TRINITY_DN12678_c0_g1_i1	Glyma.12G129800.1	95.30201342	132	3	1017	3463	1	2450	0	3762

TRINITY_DN16275_c0_g1_i2	Glyma.13G075900.1	98.1981982	418	21	310	2751	310	2736	0	2008

TRINITY_DN2525_c0_g2_i1	Glyma.12G135700.2	99.609375	409	16	5	2447	910	3346	0	2130

TRINITY_DN15636_c0_g1_i3	Glyma.06G276900.1	98.55072464	327	17	602	3031	2439	1	0	2512

TRINITY_DN15636_c0_g1_i1	Glyma.06G276900.1	96.63658737	291	17	602	3031	2439	1	0	2712

TRINITY_DN16275_c0_g1_i2	Glyma.20G053700.1	100	417	27	317	2751	155	2580	0	1973

TRINITY_DN14639_c0_g2_i1	Glyma.15G014400.1	98.6013986	395	13	143	2570	1	2446	0	2180

TRINITY_DN10934_c0_g2_i1	Glyma.20G225800.1	95.87628866	349	22	518	2947	84	2519	0	2383

TRINITY_DN15888_c0_g3_i1	Glyma.14G048100.1	100	307	14	641	3085	505	2934	0	2673

TRINITY_DN14645_c0_g1_i1	Glyma.17G059800.1	96.63658737	268	3	273	2720	2445	1	0	3005

TRINITY_DN16065_c0_g1_i3	Glyma.13G244300.1	96.2962963	197	1	2	2450	30	2475	0	3411

TRINITY_DN16065_c0_g1_i3	Glyma.15G069400.1	97.84946237	190	0	2	2450	30	2478	0	3470

TRINITY_DN12678_c0_g1_i1	Glyma.06G275600.1	95.3125	135	4	1017	3460	1	2447	0	3729

TRINITY_DN15636_c0_g1_i5	Glyma.06G276900.1	99.61685824	233	7	602	3040	2439	1	0	3114

TRINITY_DN15636_c0_g1_i5	Glyma.12G128700.1	98.38709677	229	5	602	3040	2448	1	0	3193

TRINITY_DN15636_c0_g1_i5	Glyma.12G128700.2	97.34660033	229	5	602	3040	2448	1	0	3193

TRINITY_DN15636_c0_g1_i5	Glyma.12G128700.3	100	229	5	602	3040	2448	1	0	3193

TRINITY_DN4162_c0_g1_i1	Glyma.02G087500.1	98.7804878	227	20	196	2636	641	3048	0	2959

TRINITY_DN14645_c0_g1_i2	Glyma.17G059800.1	95.03311258	216	1	273	2720	2445	1	0	3304

TRINITY_DN15636_c0_g1_i6	Glyma.06G276900.1	98.6013986	197	7	602	3040	2439	1	0	3314

TRINITY_DN15636_c0_g1_i6	Glyma.12G128700.1	100	196	5	602	3040	2448	1	0	3376

TRINITY_DN15636_c0_g1_i6	Glyma.12G128700.2	100	196	5	602	3040	2448	1	0	3376

TRINITY_DN15636_c0_g1_i6	Glyma.12G128700.3	100	196	5	602	3040	2448	1	0	3376

TRINITY_DN13758_c0_g1_i2	Glyma.17G258700.1	100	167	9	64	2493	1	2445	0	3459

TRINITY_DN13758_c0_g1_i2	Glyma.17G258700.2	100	165	11	64	2493	1	2442	0	3452

TRINITY_DN15790_c3_g4_i1	Glyma.06G066900.1	97.84946237	135	1	1299	3739	2594	5040	0	3733

TRINITY_DN16275_c0_g1_i2	Glyma.20G054100.1	97.11538462	273	10	310	2751	154	2583	0	2876

TRINITY_DN14645_c0_g1_i1	Glyma.13G100100.5	96.70781893	252	3	276	2720	2442	1	0	3088

TRINITY_DN14645_c0_g1_i1	Glyma.13G100100.6	96.25279642	252	3	276	2720	2442	1	0	3088

TRINITY_DN14645_c0_g1_i1	Glyma.13G100100.1	97.03947368	252	3	276	2720	2442	1	0	3088

TRINITY_DN14645_c0_g1_i1	Glyma.13G100100.4	100	252	3	276	2720	2442	1	0	3088

TRINITY_DN14645_c0_g1_i1	Glyma.13G100100.2	99.6742671	252	3	276	2720	2442	1	0	3088

TRINITY_DN14645_c0_g1_i1	Glyma.13G100100.3	96.62921348	252	3	276	2720	2442	1	0	3088

TRINITY_DN14645_c0_g1_i1	Glyma.13G100100.7	96.63865546	252	3	276	2720	2442	1	0	3088

TRINITY_DN12540_c0_g1_i1	Glyma.16G077200.2	95	192	2	420	2865	2454	15	0	3415

TRINITY_DN12892_c0_g4_i1	Glyma.11G103100.5	100	162	4	230	2672	34	2467	0	3522

TRINITY_DN12892_c0_g4_i1	Glyma.11G103100.6	100	162	4	230	2672	34	2467	0	3522

TRINITY_DN12892_c0_g4_i5	Glyma.11G103100.5	100	162	4	230	2672	34	2467	0	3522

TRINITY_DN12892_c0_g4_i5	Glyma.11G103100.6	95.84352078	162	4	230	2672	34	2467	0	3522

TRINITY_DN15521_c0_g1_i5	Glyma.12G231800.2	99.77220957	433	43	182	2590	79	2502	0	1748

TRINITY_DN16069_c0_g1_i2	Glyma.05G191600.1	100	318	11	183	2619	25	2464	0	2669

TRINITY_DN15636_c0_g1_i6	Glyma.12G211000.1	95.04132231	282	10	602	3040	2433	1	0	2837

TRINITY_DN15636_c0_g1_i6	Glyma.12G211000.5	98.27586207	282	10	602	3040	2433	1	0	2837

TRINITY_DN15636_c0_g1_i6	Glyma.13G290400.1	100	279	8	602	3040	2433	1	0	2854

TRINITY_DN15636_c0_g1_i6	Glyma.13G290400.4	100	279	8	602	3040	2433	1	0	2854

TRINITY_DN15636_c0_g1_i6	Glyma.13G290400.5	98.11320755	279	8	602	3040	2433	1	0	2854

TRINITY_DN16377_c0_g1_i3	Glyma.11G137700.1	95.76271186	272	25	59	2473	1	2433	0	2737

TRINITY_DN15636_c0_g1_i5	Glyma.12G211000.1	98.03921569	244	10	602	3040	2433	1	0	3048

TRINITY_DN15636_c0_g1_i5	Glyma.12G211000.5	100	244	10	602	3040	2433	1	0	3048

TRINITY_DN15636_c0_g1_i5	Glyma.13G290400.1	96.42857143	243	8	602	3040	2433	1	0	3053

TRINITY_DN15636_c0_g1_i5	Glyma.13G290400.4	97.8021978	243	8	602	3040	2433	1	0	3053

TRINITY_DN15636_c0_g1_i5	Glyma.13G290400.5	98.10810811	243	8	602	3040	2433	1	0	3053

TRINITY_DN14645_c0_g1_i2	Glyma.13G100100.5	96.17755857	200	1	276	2720	2442	1	0	3387

TRINITY_DN14645_c0_g1_i2	Glyma.13G100100.6	96.1852861	200	1	276	2720	2442	1	0	3387

TRINITY_DN14645_c0_g1_i2	Glyma.13G100100.1	96.1852861	200	1	276	2720	2442	1	0	3387

TRINITY_DN14645_c0_g1_i2	Glyma.13G100100.4	98.13664596	200	1	276	2720	2442	1	0	3387

TRINITY_DN14645_c0_g1_i2	Glyma.13G100100.2	99.77973568	200	1	276	2720	2442	1	0	3387

TRINITY_DN14645_c0_g1_i2	Glyma.13G100100.3	99.78070175	200	1	276	2720	2442	1	0	3387

TRINITY_DN14645_c0_g1_i2	Glyma.13G100100.7	99.57983193	200	1	276	2720	2442	1	0	3387

TRINITY_DN7609_c0_g1_i2	Glyma.20G074200.2	100	161	18	306	2733	2783	344	0	3482

TRINITY_DN7609_c0_g1_i2	Glyma.20G074200.1	100	161	18	306	2733	2783	344	0	3482

TRINITY_DN7609_c0_g1_i2	Glyma.20G074200.3	99.25373134	160	19	306	2733	2780	344	0	3467

TRINITY_DN7609_c0_g1_i3	Glyma.20G074200.2	97.4248927	160	26	306	2713	2783	344	0	3358

TRINITY_DN7609_c0_g1_i3	Glyma.20G074200.1	97.42574257	160	26	306	2713	2783	344	0	3358

TRINITY_DN7609_c0_g1_i3	Glyma.20G074200.3	100	159	27	306	2713	2780	344	0	3343

TRINITY_DN35705_c0_g2_i1	Glyma.04G094500.1	95.34883721	122	2	231	2669	1	2445	0	3801

TRINITY_DN15521_c0_g1_i4	Glyma.12G231800.2	100	433	43	182	2589	79	2501	0	1746

TRINITY_DN15521_c0_g1_i5	Glyma.12G231800.1	97.56097561	433	43	182	2589	79	2501	0	1746

TRINITY_DN16275_c0_g1_i5	Glyma.20G054100.1	100	315	10	310	2749	154	2581	0	2639

TRINITY_DN15516_c0_g1_i1	Glyma.12G104100.1	99.41176471	244	11	205	2633	32	2471	0	3038

TRINITY_DN12540_c0_g1_i1	Glyma.16G077200.1	95	191	2	420	2863	2856	419	0	3417

TRINITY_DN12540_c0_g1_i1	Glyma.01G009400.2	95.14018692	185	2	420	2863	2853	416	0	3450

TRINITY_DN11292_c0_g1_i1	Glyma.16G014200.1	95.06849315	130	11	458	2883	2441	1	0	3657

TRINITY_DN15629_c1_g1_i1	Glyma.14G033500.1	100	418	18	139	2563	32	2465	0	2021

TRINITY_DN14245_c0_g2_i2	Glyma.13G241400.10	95.65217391	321	30	1	2413	310	2734	0	2423

TRINITY_DN14245_c0_g2_i2	Glyma.13G241400.2	95.66326531	321	30	1	2413	310	2734	0	2423

TRINITY_DN15918_c0_g1_i4	Glyma.18G268900.2	98.90710383	296	19	787	3182	6	2437	0	2497

TRINITY_DN15918_c0_g1_i5	Glyma.18G268900.2	98.46743295	296	19	506	2901	6	2437	0	2497

TRINITY_DN16377_c0_g1_i1	Glyma.11G137700.1	95.74468085	272	30	59	2452	1	2433	0	2623

TRINITY_DN12540_c0_g1_i2	Glyma.16G077200.2	98.1981982	191	2	420	2862	2454	18	0	3415

TRINITY_DN15851_c0_g1_i3	Glyma.16G207900.1	97.20101781	191	13	227	2655	2411	1	0	3157

TRINITY_DN15851_c0_g1_i3	Glyma.16G207900.3	97.2972973	191	13	227	2655	2411	1	0	3157

TRINITY_DN15851_c0_g1_i3	Glyma.16G207900.2	97.18309859	191	13	227	2655	2411	1	0	3157

TRINITY_DN15851_c0_g1_i3	Glyma.16G207900.4	96.15384615	191	13	227	2655	2411	1	0	3157

TRINITY_DN9476_c0_g1_i3	Glyma.07G263100.2	97.33606557	175	2	787	3223	2466	24	0	3504

TRINITY_DN8367_c0_g1_i4	Glyma.11G174500.1	98.23529412	482	49	136	2533	2562	153	0	1349

TRINITY_DN11254_c0_g1_i1	Glyma.13G347500.1	98.85386819	468	36	142	2561	2600	187	0	1594

TRINITY_DN15235_c0_g1_i7	Glyma.16G172600.1	99.66101695	328	21	192	2619	697	3127	0	2531

TRINITY_DN49_c0_g2_i1	Glyma.20G217700.3	100	283	21	1062	3465	3355	931	0	2586

TRINITY_DN5648_c0_g1_i1	Glyma.10G279400.1	100	221	25	1	2402	1517	3924	0	2808

TRINITY_DN5648_c0_g1_i1	Glyma.10G279400.3	96.55172414	221	25	1	2402	1517	3924	0	2808

TRINITY_DN5648_c0_g1_i1	Glyma.10G279400.2	98.37398374	221	25	1	2402	965	3372	0	2808

TRINITY_DN5648_c0_g1_i1	Glyma.20G110300.1	97.56097561	217	20	1	2402	1553	3945	0	2732

TRINITY_DN5648_c0_g1_i1	Glyma.20G110300.3	100	217	20	1	2402	1505	3897	0	2732

TRINITY_DN5648_c0_g1_i1	Glyma.20G110300.4	98.2300885	217	20	1	2402	1358	3750	0	2732

TRINITY_DN5648_c0_g1_i1	Glyma.20G110300.2	96.55172414	217	20	1	2402	953	3345	0	2732

TRINITY_DN13964_c0_g1_i1	Glyma.12G221800.1	100	191	13	1709	4132	2432	1	0	3271

TRINITY_DN8503_c0_g1_i1	Glyma.09G164700.1	96.41025641	186	8	318	2753	2439	1	0	3421

TRINITY_DN8503_c0_g1_i3	Glyma.09G164700.1	100	186	8	329	2764	2439	1	0	3421

TRINITY_DN15303_c2_g1_i1	Glyma.09G007700.1	97.2972973	185	18	830	3263	2419	1	0	3284

TRINITY_DN15303_c2_g1_i1	Glyma.09G007700.3	97.77777778	185	18	830	3263	2419	1	0	3284

TRINITY_DN15303_c2_g1_i1	Glyma.09G007700.2	98.51485149	185	18	830	3263	2419	1	0	3284

TRINITY_DN15303_c2_g1_i1	Glyma.09G007700.4	100	185	18	830	3263	2419	1	0	3284

TRINITY_DN15303_c2_g1_i2	Glyma.09G007700.1	99.38650307	185	18	1061	3494	2419	1	0	3284

TRINITY_DN15303_c2_g1_i2	Glyma.09G007700.3	99.37888199	185	18	1061	3494	2419	1	0	3284

TRINITY_DN15303_c2_g1_i2	Glyma.09G007700.2	96.95585997	185	18	1061	3494	2419	1	0	3284

TRINITY_DN15303_c2_g1_i2	Glyma.09G007700.4	100	185	18	1061	3494	2419	1	0	3284

TRINITY_DN15303_c2_g1_i3	Glyma.09G007700.1	100	185	18	1155	3588	2419	1	0	3284

TRINITY_DN15303_c2_g1_i3	Glyma.09G007700.3	98.26254826	185	18	1155	3588	2419	1	0	3284

TRINITY_DN15303_c2_g1_i3	Glyma.09G007700.2	98.26388889	185	18	1155	3588	2419	1	0	3284

TRINITY_DN15303_c2_g1_i3	Glyma.09G007700.4	98.26589595	185	18	1155	3588	2419	1	0	3284

TRINITY_DN2525_c0_g2_i1	Glyma.12G214500.3	100	154	0	6	2447	911	3352	0	3657

TRINITY_DN11254_c0_g1_i1	Glyma.15G026400.1	100	564	65	141	2539	2571	173	0	841

TRINITY_DN15521_c0_g1_i1	Glyma.12G231800.2	97.55244755	427	39	182	2589	79	2501	0	1812

TRINITY_DN14106_c0_g1_i2	Glyma.11G190000.1	95.04761905	276	15	989	3408	1731	4165	0	2804

TRINITY_DN15516_c0_g1_i1	Glyma.06G300600.1	100	251	8	205	2633	32	2465	0	2994

TRINITY_DN11469_c0_g1_i1	Glyma.13G094700.1	96.15384615	203	10	217	2645	2466	30	0	3280

TRINITY_DN11469_c0_g1_i3	Glyma.13G094700.1	100	202	16	217	2622	2466	30	0	3136

TRINITY_DN16094_c0_g1_i3	Glyma.20G143200.2	97.82608696	261	28	21	2435	2421	1	0	2776

TRINITY_DN16094_c0_g1_i7	Glyma.20G143200.2	99.375	261	28	21	2435	2421	1	0	2776

TRINITY_DN37980_c0_g1_i1	Glyma.03G090500.1	99.13793103	251	21	14	2434	1	2418	0	2850

TRINITY_DN37980_c0_g1_i1	Glyma.03G090200.1	100	249	25	14	2434	1	2418	0	2861

TRINITY_DN4336_c0_g1_i1	Glyma.01G152900.1	100	245	8	27	2462	1	2430	0	3059

TRINITY_DN8503_c0_g1_i1	Glyma.16G213100.1	99.10313901	208	4	318	2753	2439	1	0	3321

TRINITY_DN8503_c0_g1_i3	Glyma.16G213100.1	100	208	4	329	2764	2439	1	0	3321

TRINITY_DN15531_c0_g1_i3	Glyma.02G310900.1	99.41176471	569	36	1224	3632	1122	3542	0	1042

TRINITY_DN15531_c0_g1_i3	Glyma.02G310900.2	96.66666667	569	36	1224	3632	180	2600	0	1042

TRINITY_DN10934_c0_g2_i1	Glyma.03G247800.1	96.95121951	464	63	624	3017	211	2610	0	1391

TRINITY_DN14843_c0_g1_i1	Glyma.06G109200.1	98.48484848	454	19	272	2694	214	2645	0	1840

TRINITY_DN15521_c0_g1_i5	Glyma.12G233000.3	95.40229885	445	37	182	2590	79	2499	0	1729

TRINITY_DN15314_c0_g2_i3	Glyma.13G313000.1	98.27586207	444	66	641	3029	2671	293	0	1334

TRINITY_DN13226_c0_g1_i1	Glyma.04G003900.1	100	347	19	197	2626	2529	103	0	2446

TRINITY_DN7609_c0_g1_i2	Glyma.10G122100.1	97.4248927	171	18	318	2733	2741	308	0	3376

TRINITY_DN7609_c0_g1_i3	Glyma.10G122100.1	95.41778976	171	19	318	2713	2741	308	0	3247

TRINITY_DN12949_c0_g1_i1	Glyma.19G212800.1	98.50746269	147	0	344	2782	2439	1	0	3690

TRINITY_DN12949_c0_g1_i1	Glyma.03G216300.1	97.33727811	146	0	344	2782	2439	1	0	3696

TRINITY_DN35705_c0_g2_i1	Glyma.06G096300.2	98.53658537	112	0	231	2669	1	2439	0	3884

TRINITY_DN35705_c0_g2_i1	Glyma.06G096300.1	98.98989899	112	0	231	2669	1	2439	0	3884

TRINITY_DN15521_c0_g1_i4	Glyma.12G233000.3	96.73913043	445	37	182	2589	79	2498	0	1727

TRINITY_DN15521_c0_g1_i5	Glyma.12G233000.1	95.40229885	445	37	182	2589	79	2498	0	1727

TRINITY_DN12172_c0_g1_i1	Glyma.12G136600.1	98.7012987	336	32	199	2625	2400	1	0	2324

TRINITY_DN16004_c1_g1_i5	Glyma.17G103700.1	98.55072464	202	2	95	2529	407	2844	0	3363

TRINITY_DN16004_c1_g1_i5	Glyma.13G156000.1	95.4787234	181	2	95	2529	407	2844	0	3480

TRINITY_DN12719_c0_g2_i1	Glyma.13G321300.40	96.0199005	165	29	1	2403	2388	1	0	3038

TRINITY_DN12719_c0_g2_i1	Glyma.13G321300.41	97.22222222	165	29	1	2403	2388	1	0	3038

TRINITY_DN12719_c0_g2_i1	Glyma.13G321300.28	97.22222222	165	29	1	2403	2388	1	0	3038

TRINITY_DN12719_c0_g2_i1	Glyma.13G321300.29	97.22222222	165	29	1	2403	2388	1	0	3038

TRINITY_DN12719_c0_g2_i1	Glyma.13G321300.9	96.82539683	165	29	1	2403	2388	1	0	3038

TRINITY_DN12719_c0_g2_i1	Glyma.13G321300.8	98.88888889	165	29	1	2403	2388	1	0	3038

TRINITY_DN12719_c0_g2_i3	Glyma.13G321300.40	100	165	29	1	2403	2388	1	0	3038

TRINITY_DN12719_c0_g2_i3	Glyma.13G321300.41	98.98989899	165	29	1	2403	2388	1	0	3038

TRINITY_DN12719_c0_g2_i3	Glyma.13G321300.28	98.98989899	165	29	1	2403	2388	1	0	3038

TRINITY_DN12719_c0_g2_i3	Glyma.13G321300.29	100	165	29	1	2403	2388	1	0	3038

TRINITY_DN12719_c0_g2_i3	Glyma.13G321300.9	99.45355191	165	29	1	2403	2388	1	0	3038

TRINITY_DN12719_c0_g2_i3	Glyma.13G321300.8	96.35036496	165	29	1	2403	2388	1	0	3038

TRINITY_DN14949_c0_g1_i1	Glyma.03G224400.2	96.2962963	130	3	93	2526	180	2616	0	3749

TRINITY_DN14949_c0_g1_i1	Glyma.03G224400.1	96.2962963	130	3	93	2526	180	2616	0	3749

TRINITY_DN14949_c0_g1_i1	Glyma.19G221300.2	96.2962963	117	4	93	2526	180	2616	0	3821

TRINITY_DN14949_c0_g1_i1	Glyma.19G221300.1	96.30314233	117	4	93	2526	180	2616	0	3821

TRINITY_DN16275_c0_g1_i5	Glyma.13G075900.1	99.41860465	419	21	310	2738	310	2723	0	1978

TRINITY_DN15568_c1_g1_i2	Glyma.06G267000.1	96	410	53	720	3104	110	2521	0	1731

TRINITY_DN4283_c0_g1_i1	Glyma.10G210900.3	100	237	4	151	2581	1	2437	0	3149

TRINITY_DN15851_c0_g1_i3	Glyma.09G157300.3	98.5915493	208	8	227	2655	2414	1	0	3147

TRINITY_DN15851_c0_g1_i3	Glyma.09G157300.1	98.5915493	208	8	227	2655	2414	1	0	3147

TRINITY_DN15851_c0_g1_i3	Glyma.09G157300.2	100	208	8	227	2655	2414	1	0	3147

TRINITY_DN6780_c0_g1_i1	Glyma.15G166700.2	97.92899408	190	8	300	2719	3447	1013	0	3325

TRINITY_DN6780_c0_g1_i1	Glyma.15G166700.3	95	190	8	300	2719	2967	533	0	3325

TRINITY_DN6780_c0_g1_i1	Glyma.15G166700.4	98.01980198	190	8	300	2719	2598	164	0	3325

TRINITY_DN15303_c2_g1_i1	Glyma.10G264400.12	99.3006993	170	15	830	3260	2404	1	0	3306

TRINITY_DN15303_c2_g1_i1	Glyma.10G264400.11	96	170	15	830	3260	2404	1	0	3306

TRINITY_DN15303_c2_g1_i1	Glyma.10G264400.10	100	170	15	830	3260	2404	1	0	3306

TRINITY_DN15303_c2_g1_i1	Glyma.10G264400.9	100	170	15	830	3260	2404	1	0	3306

TRINITY_DN15303_c2_g1_i1	Glyma.10G264400.8	100	170	15	830	3260	2404	1	0	3306

TRINITY_DN15303_c2_g1_i1	Glyma.10G264400.7	96.59090909	170	15	830	3260	2404	1	0	3306

TRINITY_DN15303_c2_g1_i1	Glyma.10G264400.6	97.8021978	170	15	830	3260	2404	1	0	3306

TRINITY_DN15303_c2_g1_i1	Glyma.10G264400.5	97.95918367	170	15	830	3260	2404	1	0	3306

TRINITY_DN15303_c2_g1_i1	Glyma.10G264400.4	99.58115183	170	15	830	3260	2404	1	0	3306

TRINITY_DN15303_c2_g1_i1	Glyma.10G264400.1	98.24561404	170	15	830	3260	2404	1	0	3306

TRINITY_DN15303_c2_g1_i1	Glyma.10G264400.3	98.36065574	170	15	830	3260	2404	1	0	3306

TRINITY_DN15303_c2_g1_i1	Glyma.10G264400.2	96.42857143	170	15	830	3260	2404	1	0	3306

TRINITY_DN15303_c2_g1_i2	Glyma.10G264400.12	95.00509684	170	15	1061	3491	2404	1	0	3306

TRINITY_DN15303_c2_g1_i2	Glyma.10G264400.11	95	170	15	1061	3491	2404	1	0	3306

TRINITY_DN15303_c2_g1_i2	Glyma.10G264400.10	96.51162791	170	15	1061	3491	2404	1	0	3306

TRINITY_DN15303_c2_g1_i2	Glyma.10G264400.9	99.72144847	170	15	1061	3491	2404	1	0	3306

TRINITY_DN15303_c2_g1_i2	Glyma.10G264400.8	96.95431472	170	15	1061	3491	2404	1	0	3306

TRINITY_DN15303_c2_g1_i2	Glyma.10G264400.7	97.95081967	170	15	1061	3491	2404	1	0	3306

TRINITY_DN15303_c2_g1_i2	Glyma.10G264400.6	96.63865546	170	15	1061	3491	2404	1	0	3306

TRINITY_DN15303_c2_g1_i2	Glyma.10G264400.5	98.21428571	170	15	1061	3491	2404	1	0	3306

TRINITY_DN15303_c2_g1_i2	Glyma.10G264400.4	100	170	15	1061	3491	2404	1	0	3306

TRINITY_DN15303_c2_g1_i2	Glyma.10G264400.1	100	170	15	1061	3491	2404	1	0	3306

TRINITY_DN15303_c2_g1_i2	Glyma.10G264400.3	99.38650307	170	15	1061	3491	2404	1	0	3306

TRINITY_DN15303_c2_g1_i2	Glyma.10G264400.2	99.38650307	170	15	1061	3491	2404	1	0	3306

TRINITY_DN15303_c2_g1_i3	Glyma.10G264400.12	100	170	15	1155	3585	2404	1	0	3306

TRINITY_DN15303_c2_g1_i3	Glyma.10G264400.11	100	170	15	1155	3585	2404	1	0	3306

TRINITY_DN15303_c2_g1_i3	Glyma.10G264400.10	100	170	15	1155	3585	2404	1	0	3306

TRINITY_DN15303_c2_g1_i3	Glyma.10G264400.9	98.89502762	170	15	1155	3585	2404	1	0	3306

TRINITY_DN15303_c2_g1_i3	Glyma.10G264400.8	99.0990991	170	15	1155	3585	2404	1	0	3306

TRINITY_DN15303_c2_g1_i3	Glyma.10G264400.7	100	170	15	1155	3585	2404	1	0	3306

TRINITY_DN15303_c2_g1_i3	Glyma.10G264400.6	100	170	15	1155	3585	2404	1	0	3306

TRINITY_DN15303_c2_g1_i3	Glyma.10G264400.5	100	170	15	1155	3585	2404	1	0	3306

TRINITY_DN15303_c2_g1_i3	Glyma.10G264400.4	100	170	15	1155	3585	2404	1	0	3306

TRINITY_DN15303_c2_g1_i3	Glyma.10G264400.1	100	170	15	1155	3585	2404	1	0	3306

TRINITY_DN15303_c2_g1_i3	Glyma.10G264400.3	100	170	15	1155	3585	2404	1	0	3306

TRINITY_DN15303_c2_g1_i3	Glyma.10G264400.2	100	170	15	1155	3585	2404	1	0	3306

TRINITY_DN13775_c0_g1_i1	Glyma.19G170300.1	100	162	14	146	2539	1	2427	0	3260

TRINITY_DN13775_c0_g1_i1	Glyma.19G170300.4	96.8503937	162	14	146	2539	1	2427	0	3260

TRINITY_DN13775_c0_g1_i1	Glyma.19G170300.2	98.4375	162	14	146	2539	1	2427	0	3260

TRINITY_DN13775_c0_g1_i2	Glyma.19G170300.1	96.19771863	162	14	146	2539	1	2427	0	3260

TRINITY_DN13775_c0_g1_i2	Glyma.19G170300.4	97.05400982	162	14	146	2539	1	2427	0	3260

TRINITY_DN13775_c0_g1_i2	Glyma.19G170300.2	98.8372093	162	14	146	2539	1	2427	0	3260

TRINITY_DN14622_c0_g1_i4	Glyma.20G210700.3	99.41176471	156	11	274	2701	86	2482	0	3319

TRINITY_DN16379_c0_g2_i5	Glyma.13G143600.1	97.81420765	235	1	313	2745	2436	1	0	3177

TRINITY_DN11523_c0_g1_i1	Glyma.07G042700.6	98.11320755	221	45	1659	4029	2415	1	0	2719

TRINITY_DN11523_c0_g1_i1	Glyma.07G042700.7	98.11320755	221	45	1659	4029	2415	1	0	2719

TRINITY_DN11523_c0_g1_i1	Glyma.07G042700.8	98.11676083	221	45	1659	4029	2415	1	0	2719

TRINITY_DN11523_c0_g1_i2	Glyma.07G042700.6	98.83040936	221	45	2013	4383	2415	1	0	2719

TRINITY_DN11523_c0_g1_i2	Glyma.07G042700.7	100	221	45	2013	4383	2415	1	0	2719

TRINITY_DN11523_c0_g1_i2	Glyma.07G042700.8	96.9924812	221	45	2013	4383	2415	1	0	2719

TRINITY_DN2620_c0_g1_i1	Glyma.11G102000.3	100	196	7	354	2771	2478	43	0	3297

TRINITY_DN16379_c0_g2_i4	Glyma.13G143600.1	97.8021978	194	1	274	2706	2436	1	0	3404

TRINITY_DN8558_c0_g1_i1	Glyma.10G001100.1	100	166	18	198	2597	2484	58	0	3288

TRINITY_DN15521_c0_g1_i1	Glyma.12G233000.3	100	441	33	182	2589	79	2498	0	1783

TRINITY_DN15909_c0_g3_i1	Glyma.07G031100.2	95.38461538	313	11	145	2568	1	2430	0	2660

TRINITY_DN15909_c0_g3_i1	Glyma.07G031100.1	98.27586207	313	11	145	2568	1	2430	0	2660

TRINITY_DN15909_c0_g3_i1	Glyma.07G031100.3	96.45669291	313	11	145	2568	1	2430	0	2660

TRINITY_DN15918_c0_g1_i1	Glyma.18G268900.1	99.16666667	296	19	865	3252	206	2629	0	2483

TRINITY_DN15918_c0_g1_i1	Glyma.18G268900.3	99.16666667	296	19	865	3252	206	2629	0	2483

TRINITY_DN15918_c0_g1_i6	Glyma.18G268900.1	100	296	19	584	2971	206	2629	0	2483

TRINITY_DN15918_c0_g1_i6	Glyma.18G268900.3	98.27586207	296	19	584	2971	206	2629	0	2483

TRINITY_DN15918_c0_g1_i1	Glyma.18G268900.2	99.16666667	296	19	865	3252	14	2437	0	2483

TRINITY_DN15918_c0_g1_i6	Glyma.18G268900.2	95.23809524	296	19	584	2971	14	2437	0	2483

TRINITY_DN15918_c0_g1_i1	Glyma.08G247500.2	99.16666667	291	21	865	3252	206	2626	0	2490

TRINITY_DN15918_c0_g1_i1	Glyma.08G247500.1	99.16666667	291	21	865	3252	206	2626	0	2490

TRINITY_DN15918_c0_g1_i6	Glyma.08G247500.2	99.12280702	291	21	584	2971	206	2626	0	2490

TRINITY_DN15918_c0_g1_i6	Glyma.08G247500.1	100	291	21	584	2971	206	2626	0	2490

TRINITY_DN16298_c0_g1_i2	Glyma.12G098900.2	100	280	5	752	3183	2548	117	0	2907

TRINITY_DN13377_c0_g1_i1	Glyma.15G100500.6	100	231	27	552	2947	155	2574	0	2868

TRINITY_DN13377_c0_g1_i2	Glyma.15G100500.6	100	231	27	591	2986	155	2574	0	2868

TRINITY_DN13377_c0_g1_i3	Glyma.15G100500.6	100	231	27	386	2781	155	2574	0	2868

TRINITY_DN16360_c0_g8_i6	Glyma.01G170200.1	100	302	13	197	2611	1	2433	0	2693

TRINITY_DN9350_c0_g1_i1	Glyma.10G019200.5	98.63013699	254	9	154	2584	1	2422	0	2990

TRINITY_DN11705_c0_g2_i1	Glyma.11G013900.1	98.92473118	231	34	202	2607	2388	1	0	2737

TRINITY_DN16298_c0_g1_i1	Glyma.12G098900.2	100	172	3	704	3135	2548	117	0	3517

TRINITY_DN18597_c0_g1_i1	Glyma.15G266300.1	95.90909091	139	6	1	2425	3680	1256	0	3609

TRINITY_DN18597_c0_g1_i1	Glyma.15G266300.2	95.91836735	139	6	1	2425	3671	1247	0	3609

TRINITY_DN15718_c0_g1_i2	Glyma.05G186900.1	100	301	6	179	2605	2430	1	0	2767

TRINITY_DN16379_c0_g2_i4	Glyma.03G182800.1	97.81746032	290	3	274	2706	2427	1	0	2848

TRINITY_DN16379_c0_g2_i4	Glyma.19G183400.1	97.82608696	277	4	274	2706	2427	1	0	2920

TRINITY_DN14106_c0_g1_i2	Glyma.12G084300.1	100	263	18	996	3408	1762	4183	0	2835

TRINITY_DN16379_c0_g2_i5	Glyma.03G182800.1	97.82608696	245	3	313	2745	2427	1	0	3097

TRINITY_DN16379_c0_g2_i5	Glyma.19G183400.1	97.82608696	240	4	313	2745	2427	1	0	3125

TRINITY_DN16436_c0_g2_i11	Glyma.11G131600.3	96.95723684	226	8	325	2742	1	2427	0	3105

TRINITY_DN15636_c0_g1_i1	Glyma.12G211000.1	98.73417722	176	1	602	3031	2433	1	0	3498

TRINITY_DN15636_c0_g1_i1	Glyma.12G211000.5	97.08737864	176	1	602	3031	2433	1	0	3498

TRINITY_DN15636_c0_g1_i1	Glyma.13G290400.1	97.14285714	168	3	602	3031	2433	1	0	3542

TRINITY_DN15636_c0_g1_i1	Glyma.13G290400.4	97.14285714	168	3	602	3031	2433	1	0	3542

TRINITY_DN15636_c0_g1_i1	Glyma.13G290400.5	97.14285714	168	3	602	3031	2433	1	0	3542

TRINITY_DN8558_c0_g1_i1	Glyma.10G001100.2	98.54227405	168	16	198	2597	2481	58	0	3291

TRINITY_DN15636_c0_g1_i3	Glyma.12G211000.1	100	138	1	602	3031	2433	1	0	3709

TRINITY_DN15636_c0_g1_i3	Glyma.12G211000.5	100	138	1	602	3031	2433	1	0	3709

TRINITY_DN15636_c0_g1_i3	Glyma.13G290400.1	98.80952381	132	3	602	3031	2433	1	0	3742

TRINITY_DN15636_c0_g1_i3	Glyma.13G290400.4	98.66666667	132	3	602	3031	2433	1	0	3742

TRINITY_DN15636_c0_g1_i3	Glyma.13G290400.5	100	132	3	602	3031	2433	1	0	3742

TRINITY_DN8367_c0_g1_i4	Glyma.18G062300.1	98.23529412	452	58	136	2534	3270	896	0	1406

TRINITY_DN9350_c0_g1_i1	Glyma.10G019200.3	95.83333333	254	9	154	2582	1	2420	0	2987

TRINITY_DN11705_c0_g2_i2	Glyma.11G013900.1	97.14285714	227	35	202	2601	2388	1	0	2743

TRINITY_DN9205_c0_g1_i2	Glyma.07G148100.21	95.05154639	215	20	634	3027	2430	1	0	3042

TRINITY_DN9205_c0_g1_i2	Glyma.07G148100.17	95.05208333	215	20	634	3027	2430	1	0	3042

TRINITY_DN9205_c0_g1_i2	Glyma.07G148100.14	95.05300353	215	20	634	3027	2430	1	0	3042

TRINITY_DN9205_c0_g1_i2	Glyma.07G148100.15	95.05376344	215	20	634	3027	2430	1	0	3042

TRINITY_DN9205_c0_g1_i2	Glyma.07G148100.20	100	213	26	634	3027	2406	1	0	2898

TRINITY_DN9205_c0_g1_i2	Glyma.07G148100.16	99.3006993	213	26	634	3027	2406	1	0	2898

TRINITY_DN9205_c0_g1_i2	Glyma.07G148100.12	100	213	26	634	3027	2406	1	0	2898

TRINITY_DN9205_c0_g1_i2	Glyma.07G148100.13	100	213	26	634	3027	2406	1	0	2898

TRINITY_DN9205_c0_g1_i2	Glyma.18G199400.7	95.05494505	211	14	634	3027	2430	1	0	3064

TRINITY_DN9205_c0_g1_i2	Glyma.18G199400.8	95.05494505	211	14	634	3027	2430	1	0	3064

TRINITY_DN9205_c0_g1_i2	Glyma.18G199400.9	95.05562423	211	14	634	3027	2430	1	0	3064

TRINITY_DN9205_c0_g1_i2	Glyma.18G199400.5	98.7012987	207	24	634	3027	2406	1	0	2931

TRINITY_DN9205_c0_g1_i2	Glyma.18G199400.6	96	207	24	634	3027	2406	1	0	2931

TRINITY_DN13747_c0_g1_i1	Glyma.03G197300.1	98.57142857	205	2	1	2431	591	3021	0	3343

TRINITY_DN13747_c0_g1_i2	Glyma.03G197300.1	96.61016949	205	2	1	2431	591	3021	0	3343

TRINITY_DN15207_c0_g1_i1	Glyma.11G256000.1	99.71428571	174	24	241	2661	1	2364	0	3016

TRINITY_DN15207_c0_g1_i1	Glyma.11G256000.2	99.71428571	173	26	241	2661	1	2361	0	3003

TRINITY_DN15207_c0_g1_i1	Glyma.11G256000.3	98.5915493	173	26	241	2661	1	2361	0	3003

TRINITY_DN12946_c0_g1_i1	Glyma.17G104100.1	95.73459716	140	3	285	2712	2758	328	0	3683

TRINITY_DN10934_c0_g2_i1	Glyma.19G245800.1	100	473	65	624	3015	205	2599	0	1384

TRINITY_DN11705_c0_g2_i1	Glyma.11G013900.2	96.20253165	231	33	202	2607	2385	1	0	2750

TRINITY_DN13747_c0_g1_i1	Glyma.19G195300.1	95.40636042	187	0	1	2431	591	3021	0	3454

TRINITY_DN13747_c0_g1_i2	Glyma.19G195300.1	99.3220339	187	0	1	2431	591	3021	0	3454

TRINITY_DN12362_c0_g1_i1	Glyma.02G261400.1	99.11504425	181	9	121	2527	2500	73	0	3312

TRINITY_DN12362_c0_g1_i4	Glyma.02G261400.1	98.16513761	181	9	121	2527	2500	73	0	3312

TRINITY_DN15303_c2_g1_i1	Glyma.20G126000.1	97.8021978	165	15	830	3260	2395	1	0	3343

TRINITY_DN15303_c2_g1_i1	Glyma.20G126000.2	97.8021978	165	15	830	3260	2395	1	0	3343

TRINITY_DN15303_c2_g1_i2	Glyma.20G126000.1	99.45054945	165	15	1061	3491	2395	1	0	3343

TRINITY_DN15303_c2_g1_i2	Glyma.20G126000.2	95.0044603	165	15	1061	3491	2395	1	0	3343

TRINITY_DN15303_c2_g1_i3	Glyma.20G126000.1	100	165	15	1155	3585	2395	1	0	3343

TRINITY_DN15303_c2_g1_i3	Glyma.20G126000.2	96.94656489	165	15	1155	3585	2395	1	0	3343

TRINITY_DN12946_c0_g1_i1	Glyma.13G156500.5	95.73459716	158	1	285	2712	2755	325	0	3594

TRINITY_DN12946_c0_g1_i1	Glyma.13G156500.2	95.73459716	158	1	285	2712	2755	325	0	3594

TRINITY_DN12946_c0_g1_i1	Glyma.13G156500.1	100	158	1	285	2712	2755	325	0	3594

TRINITY_DN15967_c0_g1_i1	Glyma.08G193200.1	98.63013699	145	3	120	2549	1	2427	0	3653

TRINITY_DN16436_c0_g2_i3	Glyma.11G131600.3	96.48241206	232	7	678	3092	1	2427	0	3086

TRINITY_DN13116_c0_g1_i1	Glyma.15G176600.1	97.4025974	186	6	301	2715	2430	1	0	3360

TRINITY_DN15410_c0_g2_i1	Glyma.10G146900.1	98.27586207	153	9	1070	3494	2418	3	0	3517

TRINITY_DN15967_c0_g1_i1	Glyma.07G010200.2	100	145	3	120	2549	1	2427	0	3664

TRINITY_DN15967_c0_g1_i1	Glyma.07G010200.1	95.53956835	145	3	120	2549	1	2427	0	3664

TRINITY_DN15636_c0_g1_i6	Glyma.13G290400.6	97.11538462	278	8	618	3040	2417	1	0	2830

TRINITY_DN15718_c0_g1_i1	Glyma.05G186900.1	99.39393939	255	2	209	2636	2428	1	0	3061

TRINITY_DN15636_c0_g1_i5	Glyma.13G290400.6	100	242	8	618	3040	2417	1	0	3029

TRINITY_DN11705_c0_g2_i2	Glyma.11G013900.2	97.14285714	227	34	202	2601	2385	1	0	2756

TRINITY_DN9404_c0_g1_i1	Glyma.18G282000.1	100	105	1	404	2829	2520	92	0	3884

TRINITY_DN10934_c0_g2_i2	Glyma.10G163200.1	100	497	36	570	2975	184	2589	0	1447

TRINITY_DN15718_c0_g1_i1	Glyma.06G148500.1	100	229	1	209	2636	2425	1	0	3195

TRINITY_DN15718_c0_g1_i1	Glyma.04G217300.3	99.15966387	229	1	209	2636	2425	1	0	3195

TRINITY_DN15718_c0_g1_i1	Glyma.04G217300.1	99.39393939	229	1	209	2636	2425	1	0	3195

TRINITY_DN15718_c0_g1_i1	Glyma.04G217300.2	99.39393939	227	2	209	2636	2416	1	0	3149

TRINITY_DN14752_c0_g1_i1	Glyma.19G113700.2	96.03174603	132	4	530	2936	51	2469	0	3559

TRINITY_DN14752_c0_g1_i1	Glyma.19G113700.3	96	132	4	530	2936	51	2469	0	3559

TRINITY_DN16254_c0_g1_i4	Glyma.07G004300.1	95.3271028	542	63	995	3384	2780	394	0	981

TRINITY_DN16254_c0_g1_i4	Glyma.07G004300.2	96.34146341	542	63	995	3384	2678	292	0	981

TRINITY_DN10495_c0_g1_i1	Glyma.17G062200.1	98.44720497	273	22	3	2387	1	2412	0	2601

TRINITY_DN10651_c0_g1_i1	Glyma.05G149700.1	96.2406015	245	6	379	2801	3338	915	0	3079

TRINITY_DN5710_c0_g1_i1	Glyma.14G018000.1	98.73417722	235	9	597	3015	2493	75	0	3077

TRINITY_DN15345_c0_g3_i1	Glyma.06G305000.2	100	228	6	690	3108	1	2425	0	3155

TRINITY_DN11705_c0_g2_i1	Glyma.01G230700.1	100	227	29	204	2607	2383	1	0	2791

TRINITY_DN5710_c0_g1_i1	Glyma.02G295400.2	100	222	6	597	3015	2493	72	0	3168

TRINITY_DN8175_c0_g1_i1	Glyma.15G255000.3	100	190	8	1	2406	637	3063	0	3293

TRINITY_DN8175_c0_g1_i1	Glyma.15G255000.1	96	190	8	1	2406	637	3063	0	3293

TRINITY_DN8175_c0_g1_i2	Glyma.15G255000.3	100	190	8	1	2406	637	3063	0	3293

TRINITY_DN8175_c0_g1_i2	Glyma.15G255000.1	96.79054054	190	8	1	2406	637	3063	0	3293

TRINITY_DN15718_c0_g1_i2	Glyma.04G217300.3	98.08795411	187	0	179	2605	2427	1	0	3446

TRINITY_DN15718_c0_g1_i2	Glyma.04G217300.1	97.72727273	187	0	179	2605	2427	1	0	3446

TRINITY_DN15718_c0_g1_i2	Glyma.04G217300.2	97.5877193	187	1	179	2605	2418	1	0	3387

TRINITY_DN15718_c0_g1_i2	Glyma.06G148500.1	100	184	0	179	2605	2427	1	0	3463

TRINITY_DN15410_c0_g2_i1	Glyma.10G146900.2	95.97315436	153	8	1070	3494	2415	3	0	3531

TRINITY_DN9657_c0_g1_i1	Glyma.16G206400.1	96.72578445	390	61	395	2756	223	2584	0	1493

TRINITY_DN13116_c0_g1_i1	Glyma.17G050100.1	97.4025974	360	25	301	2715	2397	1	0	2228

TRINITY_DN13116_c0_g1_i1	Glyma.17G050100.4	97.4025974	360	25	301	2715	2397	1	0	2228

TRINITY_DN13116_c0_g1_i1	Glyma.17G050100.5	97.38219895	360	25	301	2715	2397	1	0	2228

TRINITY_DN13116_c0_g1_i1	Glyma.17G050100.2	97.38372093	360	25	301	2715	2397	1	0	2228

TRINITY_DN13116_c0_g1_i1	Glyma.17G050100.3	97.4025974	360	25	301	2715	2397	1	0	2228

TRINITY_DN8623_c0_g1_i1	Glyma.05G146000.1	100	339	10	104	2519	2520	99	0	2512

TRINITY_DN13116_c0_g1_i1	Glyma.09G068800.1	97.39130435	177	3	302	2715	2426	1	0	3422

TRINITY_DN15410_c0_g2_i1	Glyma.10G146900.3	100	153	9	1070	3490	2412	1	0	3509

TRINITY_DN16379_c0_g1_i2	Glyma.11G194900.1	97.8021978	265	5	372	2792	2421	1	0	2959

TRINITY_DN16379_c0_g1_i2	Glyma.11G194900.3	97.82608696	265	5	372	2792	2421	1	0	2959

TRINITY_DN16379_c0_g1_i2	Glyma.11G194900.2	97.80487805	265	5	372	2792	2421	1	0	2959

TRINITY_DN10833_c0_g1_i2	Glyma.09G248000.2	98.38709677	251	7	352	2767	2449	31	0	2990

TRINITY_DN16379_c0_g1_i1	Glyma.11G194900.1	95.62624254	231	5	372	2792	2421	1	0	3147

TRINITY_DN16379_c0_g1_i1	Glyma.11G194900.3	95.65217391	231	5	372	2792	2421	1	0	3147

TRINITY_DN16379_c0_g1_i1	Glyma.11G194900.2	95.63318777	231	5	372	2792	2421	1	0	3147

TRINITY_DN16433_c0_g2_i2	Glyma.18G036300.1	98.57142857	178	2	1951	4374	1753	4176	0	3480

TRINITY_DN4278_c0_g1_i1	Glyma.12G013000.1	95.39170507	139	1	497	2921	2424	1	0	3701

TRINITY_DN14836_c0_g2_i1	Glyma.13G034600.3	95	296	27	6	2376	1456	3847	0	2287

TRINITY_DN14836_c0_g2_i1	Glyma.13G034600.2	99.12280702	296	27	6	2376	1456	3847	0	2287

TRINITY_DN14836_c0_g2_i1	Glyma.13G034600.1	95.55555556	296	27	6	2376	1456	3847	0	2287

TRINITY_DN10495_c0_g1_i1	Glyma.13G097400.1	100	265	21	3	2387	1	2409	0	2660

TRINITY_DN11705_c0_g2_i1	Glyma.01G230700.2	99.25373134	227	28	204	2607	2380	1	0	2806

TRINITY_DN15424_c0_g1_i1	Glyma.06G255000.1	100	207	0	448	2871	2424	1	0	3330

TRINITY_DN15424_c0_g1_i1	Glyma.06G255000.2	99.19354839	205	2	448	2871	2406	1	0	3225

TRINITY_DN15424_c0_g1_i2	Glyma.06G255000.1	95.65217391	204	1	448	2846	2424	1	0	3184

TRINITY_DN15424_c0_g1_i1	Glyma.12G146600.1	99.19354839	204	1	448	2871	2421	1	0	3326

TRINITY_DN15424_c0_g1_i2	Glyma.12G146600.1	98.80952381	202	2	448	2846	2421	1	0	3177

TRINITY_DN15424_c0_g1_i2	Glyma.06G255000.2	98.80952381	202	3	448	2846	2406	1	0	3079

TRINITY_DN16379_c0_g1_i2	Glyma.13G323600.1	97.82608696	129	1	372	2792	2424	1	0	3742

TRINITY_DN16379_c0_g1_i2	Glyma.12G177100.1	97.82608696	120	1	372	2792	2424	1	0	3792

TRINITY_DN13116_c0_g1_i1	Glyma.13G109600.12	97.38219895	359	22	301	2715	2397	1	0	2266

TRINITY_DN13116_c0_g1_i1	Glyma.13G109600.2	97.39776952	359	22	301	2715	2397	1	0	2266

TRINITY_DN13116_c0_g1_i1	Glyma.13G109600.13	97.39478958	359	22	301	2715	2397	1	0	2266

TRINITY_DN13116_c0_g1_i1	Glyma.13G109600.6	97.37991266	359	22	301	2715	2397	1	0	2266

TRINITY_DN13116_c0_g1_i1	Glyma.13G109600.3	97.39130435	359	22	301	2715	2397	1	0	2266

TRINITY_DN13116_c0_g1_i1	Glyma.13G109600.1	97.39130435	359	22	301	2715	2397	1	0	2266

TRINITY_DN13116_c0_g1_i1	Glyma.13G109600.7	97.4025974	359	22	301	2715	2397	1	0	2266

TRINITY_DN13116_c0_g1_i1	Glyma.13G109600.8	97.38562092	359	22	301	2715	2397	1	0	2266

TRINITY_DN11705_c0_g2_i2	Glyma.01G230700.1	97.2972973	226	30	204	2601	2383	1	0	2802

TRINITY_DN16237_c0_g1_i15	Glyma.05G200800.2	95.41284404	201	16	256	2655	1	2409	0	3121

TRINITY_DN16237_c0_g1_i15	Glyma.05G200800.3	95.41284404	201	16	256	2655	1	2409	0	3121

TRINITY_DN12362_c0_g1_i1	Glyma.14G055900.1	99.11504425	185	10	121	2527	2479	70	0	3262

TRINITY_DN12362_c0_g1_i4	Glyma.14G055900.1	98.16513761	185	10	121	2527	2479	70	0	3262

TRINITY_DN15909_c0_g3_i1	Glyma.13G364900.1	97.20930233	177	0	145	2567	1	2423	0	3494

TRINITY_DN15909_c0_g3_i1	Glyma.13G364900.2	98.53658537	177	0	145	2567	1	2423	0	3494

TRINITY_DN15410_c0_g2_i1	Glyma.10G146900.4	96.82539683	153	8	1070	3490	2409	1	0	3524

TRINITY_DN10789_c0_g2_i1	Glyma.10G042300.2	100	437	94	685	3004	296	2687	0	1199

TRINITY_DN10789_c0_g2_i1	Glyma.10G042300.1	100	437	94	685	3004	296	2687	0	1199

TRINITY_DN16415_c1_g1_i7	Glyma.13G327000.1	96.68874172	365	7	1364	3779	2416	1	0	2385

TRINITY_DN15570_c0_g1_i2	Glyma.02G190000.1	100	177	5	597	2999	1	2415	0	3336

TRINITY_DN14979_c0_g1_i1	Glyma.14G223400.1	95.3125	162	3	248	2653	2421	1	0	3476

TRINITY_DN12365_c0_g1_i2	Glyma.19G139800.1	96.20253165	159	0	1075	3495	475	2895	0	3591

TRINITY_DN12365_c0_g1_i2	Glyma.03G137100.1	97.10144928	151	0	1075	3495	475	2895	0	3635

TRINITY_DN11705_c0_g2_i2	Glyma.01G230700.2	97.24137931	226	29	204	2601	2380	1	0	2817

TRINITY_DN16237_c0_g1_i11	Glyma.05G200800.2	96	201	16	256	2652	1	2406	0	3116

TRINITY_DN16237_c0_g1_i11	Glyma.05G200800.3	96	201	16	256	2652	1	2406	0	3116

TRINITY_DN16237_c0_g1_i15	Glyma.05G200800.4	96.61016949	201	16	256	2652	1	2406	0	3116

TRINITY_DN16237_c0_g1_i15	Glyma.05G200800.1	98.97959184	201	16	256	2652	1	2406	0	3116

TRINITY_DN16237_c0_g1_i5	Glyma.05G200800.2	98.47715736	201	16	256	2652	1	2406	0	3116

TRINITY_DN16237_c0_g1_i5	Glyma.05G200800.3	100	201	16	256	2652	1	2406	0	3116

TRINITY_DN16016_c0_g1_i6	Glyma.20G170000.1	95	156	4	1	2411	2421	11	0	3489

TRINITY_DN10705_c0_g1_i1	Glyma.17G244600.7	100	110	0	204	2623	1	2420	0	3860

TRINITY_DN10705_c0_g1_i1	Glyma.17G244600.6	100	110	0	204	2623	1	2420	0	3860

TRINITY_DN10705_c0_g1_i1	Glyma.14G080800.6	100	105	0	204	2623	1	2420	0	3888

TRINITY_DN14332_c0_g1_i2	Glyma.13G275100.1	100	385	46	4	2392	781	3175	0	1986

TRINITY_DN16436_c0_g2_i1	Glyma.11G131600.3	100	227	6	325	2729	1	2414	0	3086

TRINITY_DN16436_c0_g2_i10	Glyma.11G131600.3	100	220	6	325	2729	1	2414	0	3125

TRINITY_DN14622_c0_g1_i6	Glyma.20G210700.3	95	157	7	274	2683	86	2482	0	3397

TRINITY_DN15531_c0_g1_i3	Glyma.14G001500.1	100	571	28	1240	3632	1138	3542	0	1057

TRINITY_DN15531_c0_g1_i3	Glyma.14G001500.2	95.45454545	571	28	1240	3632	1129	3533	0	1057

TRINITY_DN16379_c0_g2_i5	Glyma.10G057100.1	97.8021978	227	2	313	2727	2442	25	0	3188

TRINITY_DN7121_c0_g1_i1	Glyma.13G319300.1	97.31182796	210	26	278	2692	2583	211	0	2992

TRINITY_DN16379_c0_g2_i4	Glyma.10G057100.1	97.8021978	187	1	274	2688	2442	25	0	3410

TRINITY_DN9142_c0_g1_i1	Glyma.12G018400.2	96.8	183	11	108	2522	2403	1	0	3336

TRINITY_DN16231_c1_g1_i1	Glyma.17G045800.2	100	160	0	80	2497	1	2418	0	3579

TRINITY_DN16231_c1_g1_i1	Glyma.17G045800.1	100	160	0	80	2497	1	2418	0	3579

TRINITY_DN8623_c0_g1_i1	Glyma.11G248700.2	100	160	2	104	2519	2514	99	0	3554

TRINITY_DN8623_c0_g1_i1	Glyma.11G248700.1	98.7804878	160	2	104	2519	2514	99	0	3554

TRINITY_DN16231_c1_g1_i1	Glyma.13G114000.1	96.96969697	154	0	80	2497	1	2418	0	3613

TRINITY_DN16231_c1_g1_i1	Glyma.13G114000.2	98.2788296	154	0	80	2497	1	2418	0	3613

TRINITY_DN16231_c1_g1_i1	Glyma.13G114000.3	100	154	0	80	2497	1	2418	0	3613

TRINITY_DN16016_c0_g1_i6	Glyma.10G221500.1	96.35416667	153	4	1	2412	2418	10	0	3520

TRINITY_DN16016_c0_g1_i6	Glyma.10G221500.3	96.35499208	153	4	1	2412	2418	10	0	3520

TRINITY_DN16016_c0_g1_i6	Glyma.10G221500.2	96.35568513	153	4	1	2412	2418	10	0	3520

TRINITY_DN16360_c0_g8_i6	Glyma.16G131800.1	100	135	9	197	2611	1	2412	0	3659

TRINITY_DN15636_c0_g1_i1	Glyma.13G290400.6	97.14285714	167	3	618	3031	2417	1	0	3518

TRINITY_DN5301_c0_g2_i1	Glyma.04G232700.1	96.30390144	166	2	1	2416	6081	3666	0	3531

TRINITY_DN5301_c0_g2_i1	Glyma.04G232700.2	100	166	2	1	2416	4443	2028	0	3531

TRINITY_DN13604_c0_g1_i2	Glyma.11G218000.2	100	145	2	804	3219	927	3342	0	3648

TRINITY_DN13604_c0_g1_i3	Glyma.11G218000.2	98.88888889	145	2	1168	3583	927	3342	0	3648

TRINITY_DN15636_c0_g1_i3	Glyma.13G290400.6	97.4025974	131	3	618	3031	2417	1	0	3718

TRINITY_DN14021_c0_g2_i2	Glyma.11G018700.1	96.53465347	281	9	1	2404	3141	740	0	2737

TRINITY_DN16436_c0_g2_i7	Glyma.11G131600.3	95.74468085	233	5	678	3079	1	2414	0	3068

TRINITY_DN13604_c0_g1_i2	Glyma.18G039000.1	98.91891892	149	0	804	3219	927	3342	0	3637

TRINITY_DN13604_c0_g1_i3	Glyma.18G039000.1	97.05882353	149	0	1168	3583	927	3342	0	3637

TRINITY_DN3649_c0_g1_i1	Glyma.09G173000.2	100	428	34	364	2756	3680	1291	0	1784

TRINITY_DN3649_c0_g1_i1	Glyma.09G173000.1	96	428	34	364	2756	3680	1291	0	1784

TRINITY_DN14750_c0_g1_i1	Glyma.04G131700.1	95.23809524	312	9	524	2934	2444	40	0	2641

TRINITY_DN14021_c0_g2_i2	Glyma.01G224200.3	96.53679654	300	9	1	2404	3159	752	0	2682

TRINITY_DN14021_c0_g2_i2	Glyma.01G224200.1	99.07407407	300	9	1	2404	3159	752	0	2682

TRINITY_DN14021_c0_g2_i2	Glyma.01G224200.2	95.04132231	300	9	1	2404	3159	752	0	2682

TRINITY_DN12238_c0_g1_i2	Glyma.19G014100.1	97.59036145	249	26	565	2948	44	2433	0	2719

TRINITY_DN5378_c0_g1_i1	Glyma.17G038800.1	95.93495935	214	11	1	2399	592	3000	0	3133

TRINITY_DN5378_c0_g2_i1	Glyma.17G038800.1	100	214	11	1	2399	592	3000	0	3133

TRINITY_DN9205_c0_g1_i3	Glyma.07G148100.9	95.3038674	214	20	794	3170	2413	1	0	3016

TRINITY_DN9205_c0_g1_i3	Glyma.07G148100.1	96.66666667	214	20	794	3170	2413	1	0	3016

TRINITY_DN9205_c0_g1_i3	Glyma.07G148100.8	100	212	26	794	3170	2389	1	0	2872

TRINITY_DN9205_c0_g1_i3	Glyma.18G199400.4	98.82352941	210	14	794	3170	2413	1	0	3038

TRINITY_DN9205_c0_g1_i3	Glyma.18G199400.1	98.82352941	210	14	794	3170	2413	1	0	3038

TRINITY_DN9205_c0_g1_i3	Glyma.18G199400.3	95.0617284	206	24	794	3170	2389	1	0	2905

TRINITY_DN9205_c0_g1_i3	Glyma.18G199400.2	100	206	24	794	3170	2389	1	0	2905

TRINITY_DN14362_c0_g1_i2	Glyma.01G153200.1	98.70967742	204	18	208	2586	1	2415	0	3097

TRINITY_DN16415_c1_g1_i7	Glyma.11G147900.1	95.951417	187	6	1364	3777	2410	5	0	3360

TRINITY_DN5301_c0_g2_i1	Glyma.06G132300.1	100	178	0	2	2416	6083	3669	0	3474

TRINITY_DN9142_c0_g1_i1	Glyma.12G018400.3	97.72727273	169	1	108	2522	2412	1	0	3504

TRINITY_DN16360_c0_g8_i6	Glyma.02G051700.1	100	156	2	197	2611	1	2412	0	3576

TRINITY_DN13411_c0_g1_i1	Glyma.02G215800.1	98.95287958	421	28	3312	5701	4423	2020	0	1906

TRINITY_DN4336_c0_g1_i1	Glyma.09G218100.1	95.26315789	237	15	63	2462	22	2424	0	2983

TRINITY_DN14750_c0_g1_i2	Glyma.04G131700.1	95.18599562	219	4	524	2937	2444	40	0	3186

TRINITY_DN9205_c0_g1_i2	Glyma.07G148100.19	95.3125	214	20	652	3027	2412	1	0	3014

TRINITY_DN9205_c0_g1_i2	Glyma.07G148100.9	99.17355372	214	20	652	3027	2412	1	0	3014

TRINITY_DN9205_c0_g1_i2	Glyma.07G148100.1	100	214	20	652	3027	2412	1	0	3014

TRINITY_DN9205_c0_g1_i3	Glyma.07G148100.21	97.8021978	214	20	795	3170	2412	1	0	3014

TRINITY_DN9205_c0_g1_i3	Glyma.07G148100.17	97.8021978	214	20	795	3170	2412	1	0	3014

TRINITY_DN9205_c0_g1_i3	Glyma.07G148100.14	97.8021978	214	20	795	3170	2412	1	0	3014

TRINITY_DN9205_c0_g1_i3	Glyma.07G148100.15	97.8021978	214	20	795	3170	2412	1	0	3014

TRINITY_DN9205_c0_g1_i3	Glyma.07G148100.19	97.8021978	214	20	795	3170	2412	1	0	3014

TRINITY_DN9205_c0_g1_i4	Glyma.07G148100.21	96.42857143	214	20	556	2931	2412	1	0	3014

TRINITY_DN9205_c0_g1_i4	Glyma.07G148100.17	98.65771812	214	20	556	2931	2412	1	0	3014

TRINITY_DN9205_c0_g1_i4	Glyma.07G148100.14	97.2972973	214	20	556	2931	2412	1	0	3014

TRINITY_DN9205_c0_g1_i4	Glyma.07G148100.15	97.2972973	214	20	556	2931	2412	1	0	3014

TRINITY_DN9205_c0_g1_i4	Glyma.07G148100.19	100	214	20	556	2931	2412	1	0	3014

TRINITY_DN9205_c0_g1_i2	Glyma.07G148100.18	100	212	26	652	3027	2388	1	0	2870

TRINITY_DN9205_c0_g1_i2	Glyma.07G148100.8	100	212	26	652	3027	2388	1	0	2870

TRINITY_DN9205_c0_g1_i3	Glyma.07G148100.20	100	212	26	795	3170	2388	1	0	2870

TRINITY_DN9205_c0_g1_i3	Glyma.07G148100.16	100	212	26	795	3170	2388	1	0	2870

TRINITY_DN9205_c0_g1_i3	Glyma.07G148100.12	100	212	26	795	3170	2388	1	0	2870

TRINITY_DN9205_c0_g1_i3	Glyma.07G148100.13	98.87640449	212	26	795	3170	2388	1	0	2870

TRINITY_DN9205_c0_g1_i3	Glyma.07G148100.18	100	212	26	795	3170	2388	1	0	2870

TRINITY_DN9205_c0_g1_i4	Glyma.07G148100.20	96.66666667	212	26	556	2931	2388	1	0	2870

TRINITY_DN9205_c0_g1_i4	Glyma.07G148100.16	96.2962963	212	26	556	2931	2388	1	0	2870

TRINITY_DN9205_c0_g1_i4	Glyma.07G148100.12	100	212	26	556	2931	2388	1	0	2870

TRINITY_DN9205_c0_g1_i4	Glyma.07G148100.13	97.18309859	212	26	556	2931	2388	1	0	2870

TRINITY_DN9205_c0_g1_i4	Glyma.07G148100.18	97.18309859	212	26	556	2931	2388	1	0	2870

TRINITY_DN9205_c0_g1_i2	Glyma.18G199400.11	98.50746269	210	14	652	3027	2412	1	0	3037

TRINITY_DN9205_c0_g1_i2	Glyma.18G199400.4	98.50746269	210	14	652	3027	2412	1	0	3037

TRINITY_DN9205_c0_g1_i2	Glyma.18G199400.1	97.88135593	210	14	652	3027	2412	1	0	3037

TRINITY_DN9205_c0_g1_i3	Glyma.18G199400.7	95.38461538	210	14	795	3170	2412	1	0	3037

TRINITY_DN9205_c0_g1_i3	Glyma.18G199400.8	95.38461538	210	14	795	3170	2412	1	0	3037

TRINITY_DN9205_c0_g1_i3	Glyma.18G199400.9	95.38461538	210	14	795	3170	2412	1	0	3037

TRINITY_DN9205_c0_g1_i3	Glyma.18G199400.11	97.84172662	210	14	795	3170	2412	1	0	3037

TRINITY_DN9205_c0_g1_i4	Glyma.18G199400.7	100	210	14	556	2931	2412	1	0	3037

TRINITY_DN9205_c0_g1_i4	Glyma.18G199400.8	100	210	14	556	2931	2412	1	0	3037

TRINITY_DN9205_c0_g1_i4	Glyma.18G199400.9	100	210	14	556	2931	2412	1	0	3037

TRINITY_DN9205_c0_g1_i4	Glyma.18G199400.11	95.29737206	210	14	556	2931	2412	1	0	3037

TRINITY_DN9205_c0_g1_i2	Glyma.18G199400.10	96	206	24	652	3027	2388	1	0	2904

TRINITY_DN9205_c0_g1_i2	Glyma.18G199400.3	96.58119658	206	24	652	3027	2388	1	0	2904

TRINITY_DN9205_c0_g1_i2	Glyma.18G199400.2	100	206	24	652	3027	2388	1	0	2904

TRINITY_DN9205_c0_g1_i3	Glyma.18G199400.5	100	206	24	795	3170	2388	1	0	2904

TRINITY_DN9205_c0_g1_i3	Glyma.18G199400.6	98.18181818	206	24	795	3170	2388	1	0	2904

TRINITY_DN9205_c0_g1_i3	Glyma.18G199400.10	97.22222222	206	24	795	3170	2388	1	0	2904

TRINITY_DN9205_c0_g1_i4	Glyma.18G199400.5	100	206	24	556	2931	2388	1	0	2904

TRINITY_DN9205_c0_g1_i4	Glyma.18G199400.6	98.82352941	206	24	556	2931	2388	1	0	2904

TRINITY_DN9205_c0_g1_i4	Glyma.18G199400.10	97.03703704	206	24	556	2931	2388	1	0	2904

TRINITY_DN16237_c0_g1_i15	Glyma.08G008100.1	100	201	25	256	2652	1	2385	0	3048

TRINITY_DN12719_c0_g2_i1	Glyma.13G321300.38	100	169	20	1	2403	2364	1	0	3127

TRINITY_DN12719_c0_g2_i1	Glyma.13G321300.39	100	169	20	1	2403	2364	1	0	3127

TRINITY_DN12719_c0_g2_i1	Glyma.13G321300.26	100	169	20	1	2403	2364	1	0	3127

TRINITY_DN12719_c0_g2_i1	Glyma.13G321300.27	98.26086957	169	20	1	2403	2364	1	0	3127

TRINITY_DN12719_c0_g2_i1	Glyma.13G321300.7	100	169	20	1	2403	2364	1	0	3127

TRINITY_DN12719_c0_g2_i1	Glyma.13G321300.6	96.0199005	169	20	1	2403	2364	1	0	3127

TRINITY_DN12719_c0_g2_i3	Glyma.13G321300.38	100	169	20	1	2403	2364	1	0	3127

TRINITY_DN12719_c0_g2_i3	Glyma.13G321300.39	100	169	20	1	2403	2364	1	0	3127

TRINITY_DN12719_c0_g2_i3	Glyma.13G321300.26	100	169	20	1	2403	2364	1	0	3127

TRINITY_DN12719_c0_g2_i3	Glyma.13G321300.27	100	169	20	1	2403	2364	1	0	3127

TRINITY_DN12719_c0_g2_i3	Glyma.13G321300.7	98.16513761	169	20	1	2403	2364	1	0	3127

TRINITY_DN12719_c0_g2_i3	Glyma.13G321300.6	96.08938547	169	20	1	2403	2364	1	0	3127

TRINITY_DN12617_c0_g1_i1	Glyma.15G170000.11	98.50746269	211	12	1817	4225	1230	3617	0	3099

TRINITY_DN12617_c0_g1_i1	Glyma.15G170000.7	98.50746269	211	12	1817	4225	1230	3617	0	3099

TRINITY_DN12617_c0_g1_i1	Glyma.15G170000.5	98.48942598	211	12	1817	4225	1230	3617	0	3099

TRINITY_DN12617_c0_g1_i1	Glyma.15G170000.1	98.4984985	211	12	1817	4225	1230	3617	0	3099

TRINITY_DN12617_c0_g1_i1	Glyma.15G170000.9	98.50746269	211	12	1817	4225	1230	3617	0	3099

TRINITY_DN12617_c0_g1_i1	Glyma.15G170000.8	98.4962406	211	12	1817	4225	1224	3611	0	3099

TRINITY_DN12617_c0_g1_i1	Glyma.15G170000.6	98.50340136	211	12	1817	4225	1224	3611	0	3099

TRINITY_DN12617_c0_g1_i1	Glyma.15G170000.10	98.4962406	211	12	1817	4225	1206	3593	0	3099

TRINITY_DN12617_c0_g1_i1	Glyma.15G170000.4	98.50746269	211	12	1817	4225	1206	3593	0	3099

TRINITY_DN12617_c0_g1_i1	Glyma.15G170000.15	96.52351738	211	12	1817	4225	1182	3569	0	3099

TRINITY_DN12617_c0_g1_i1	Glyma.15G170000.3	96.55172414	211	12	1817	4225	1182	3569	0	3099

TRINITY_DN12617_c0_g1_i1	Glyma.15G170000.14	96.52928416	211	12	1817	4225	1158	3545	0	3099

TRINITY_DN12617_c0_g1_i1	Glyma.15G170000.2	97.00598802	211	12	1817	4225	1158	3545	0	3099

TRINITY_DN12617_c0_g1_i1	Glyma.15G170000.12	96.52509653	211	12	1817	4225	1137	3524	0	3099

TRINITY_DN12617_c0_g1_i1	Glyma.15G170000.13	96.52928416	211	12	1817	4225	1137	3524	0	3099

TRINITY_DN15979_c0_g3_i1	Glyma.04G214200.1	96.875	203	17	409	2797	2511	108	0	3118

TRINITY_DN15979_c0_g3_i1	Glyma.04G214200.7	96.875	203	17	409	2797	2511	108	0	3118

TRINITY_DN15979_c0_g3_i1	Glyma.04G214200.4	96	203	17	409	2797	2511	108	0	3118

TRINITY_DN15979_c0_g3_i1	Glyma.04G214200.3	97.8021978	203	17	409	2797	2511	108	0	3118

TRINITY_DN15979_c0_g3_i2	Glyma.04G214200.1	95.86956522	203	17	409	2797	2511	108	0	3118

TRINITY_DN15979_c0_g3_i2	Glyma.04G214200.7	97.89473684	203	17	409	2797	2511	108	0	3118

TRINITY_DN15979_c0_g3_i2	Glyma.04G214200.4	98.66666667	203	17	409	2797	2511	108	0	3118

TRINITY_DN15979_c0_g3_i2	Glyma.04G214200.3	100	203	17	409	2797	2511	108	0	3118

TRINITY_DN1095_c0_g2_i1	Glyma.09G120400.1	96.04810997	179	7	122	2520	2499	89	0	3362

TRINITY_DN1095_c0_g1_i1	Glyma.09G120400.1	95.2173913	176	10	122	2504	2499	89	0	3275

TRINITY_DN8129_c0_g1_i1	Glyma.07G238900.1	97.08029197	151	2	292	2703	2412	1	0	3607

TRINITY_DN3649_c0_g2_i1	Glyma.09G173000.2	95.47553093	430	33	364	2753	3680	1291	0	1788

TRINITY_DN3649_c0_g2_i1	Glyma.09G173000.1	95.94594595	430	33	364	2753	3680	1291	0	1788

TRINITY_DN15629_c1_g1_i1	Glyma.02G281100.1	95.6043956	419	21	169	2563	104	2504	0	1953

TRINITY_DN16254_c0_g1_i4	Glyma.17G057800.1	97.77777778	283	19	995	3383	2765	362	0	2686

TRINITY_DN8129_c0_g1_i1	Glyma.17G034800.1	97.08029197	159	0	292	2703	2412	1	0	3574

TRINITY_DN15876_c0_g1_i2	Glyma.06G040600.1	100	222	7	107	2509	1	2409	0	3158

TRINITY_DN15876_c0_g1_i2	Glyma.06G040600.2	96.66666667	222	7	107	2509	1	2409	0	3158

TRINITY_DN12719_c0_g2_i6	Glyma.13G321300.53	98.26589595	163	30	1	2376	2346	1	0	2904

TRINITY_DN12719_c0_g2_i6	Glyma.13G321300.36	98.26589595	163	30	1	2376	2346	1	0	2904

TRINITY_DN12719_c0_g2_i6	Glyma.13G321300.37	100	163	30	1	2376	2346	1	0	2904

TRINITY_DN12719_c0_g2_i6	Glyma.13G321300.24	99.0521327	163	30	1	2376	2346	1	0	2904

TRINITY_DN12719_c0_g2_i6	Glyma.13G321300.25	98.85057471	163	30	1	2376	2346	1	0	2904

TRINITY_DN12719_c0_g2_i6	Glyma.13G321300.4	95.41984733	163	30	1	2376	2346	1	0	2904

TRINITY_DN12719_c0_g2_i6	Glyma.13G321300.5	96.17834395	163	30	1	2376	2346	1	0	2904

TRINITY_DN12719_c0_g2_i8	Glyma.13G321300.53	100	163	30	1	2376	2346	1	0	2904

TRINITY_DN12719_c0_g2_i8	Glyma.13G321300.36	100	163	30	1	2376	2346	1	0	2904

TRINITY_DN12719_c0_g2_i8	Glyma.13G321300.37	100	163	30	1	2376	2346	1	0	2904

TRINITY_DN12719_c0_g2_i8	Glyma.13G321300.24	100	163	30	1	2376	2346	1	0	2904

TRINITY_DN12719_c0_g2_i8	Glyma.13G321300.25	95.23809524	163	30	1	2376	2346	1	0	2904

TRINITY_DN12719_c0_g2_i8	Glyma.13G321300.4	98.03921569	163	30	1	2376	2346	1	0	2904

TRINITY_DN12719_c0_g2_i8	Glyma.13G321300.5	97.07112971	163	30	1	2376	2346	1	0	2904

TRINITY_DN14332_c0_g1_i2	Glyma.12G225800.1	96.66666667	390	30	4	2392	781	3175	0	2058

TRINITY_DN10733_c0_g1_i1	Glyma.11G020700.1	97.22222222	322	12	639	3042	384	2784	0	2569

TRINITY_DN4996_c0_g1_i1	Glyma.02G281900.2	98.98989899	297	20	290	2685	4224	1842	0	2540

TRINITY_DN10754_c0_g2_i1	Glyma.08G335200.1	95.30201342	176	5	268	2674	2505	100	0	3430

TRINITY_DN42081_c0_g1_i1	Glyma.19G240700.1	98.52941176	160	1	1	2410	2804	404	0	3506

TRINITY_DN42081_c0_g1_i1	Glyma.03G243400.2	95.23809524	156	1	1	2410	2804	404	0	3528

TRINITY_DN42081_c0_g1_i1	Glyma.03G243400.4	97.4137931	156	1	1	2410	2804	404	0	3528

TRINITY_DN42081_c0_g1_i1	Glyma.03G243400.1	97.4137931	156	1	1	2410	2804	404	0	3528

TRINITY_DN42081_c0_g1_i1	Glyma.03G243400.6	100	156	1	1	2410	2804	404	0	3528

TRINITY_DN42081_c0_g1_i1	Glyma.03G243400.5	98.27586207	156	1	1	2410	2789	389	0	3528

TRINITY_DN42081_c0_g1_i1	Glyma.03G243400.3	100	156	1	1	2410	2789	389	0	3528

TRINITY_DN15560_c0_g1_i1	Glyma.19G165200.2	100	375	25	204	2588	1	2388	0	2080

TRINITY_DN13389_c0_g1_i1	Glyma.13G338700.1	99.47643979	275	22	53	2434	1	2403	0	2712

TRINITY_DN14645_c0_g1_i2	Glyma.15G165100.2	96.36363636	232	6	264	2660	2460	52	0	3086

TRINITY_DN14645_c0_g1_i1	Glyma.15G165100.2	96.51741294	191	6	264	2660	2460	52	0	3314

TRINITY_DN16379_c0_g1_i1	Glyma.13G323600.1	95.65217391	189	1	372	2777	2424	16	0	3382

TRINITY_DN16379_c0_g1_i1	Glyma.12G177100.1	97.36842105	184	1	372	2777	2424	16	0	3410

TRINITY_DN2555_c0_g2_i1	Glyma.06G129700.3	97.70114943	168	8	396	2783	1	2406	0	3363

TRINITY_DN2555_c0_g2_i1	Glyma.06G129700.2	100	168	8	396	2783	1	2406	0	3363

TRINITY_DN2555_c0_g2_i1	Glyma.06G129700.1	100	168	8	396	2783	1	2406	0	3363

TRINITY_DN2555_c0_g2_i1	Glyma.06G129700.4	100	168	8	396	2783	1	2406	0	3363

TRINITY_DN14979_c0_g1_i1	Glyma.14G223400.6	95.3125	162	2	248	2653	2409	1	0	3531

TRINITY_DN14979_c0_g1_i1	Glyma.14G223400.7	95.29411765	162	2	248	2653	2409	1	0	3531

TRINITY_DN14979_c0_g1_i1	Glyma.14G223400.2	95.30864198	162	2	248	2653	2409	1	0	3531

TRINITY_DN10733_c0_g1_i1	Glyma.01G222700.1	97.22222222	321	9	639	3042	384	2784	0	2597

TRINITY_DN4162_c0_g1_i1	Glyma.10G116800.3	98.7804878	228	12	198	2596	631	3017	0	2990

TRINITY_DN10754_c0_g3_i1	Glyma.08G335200.1	95.2991453	175	5	268	2672	2505	102	0	3432

TRINITY_DN15888_c0_g3_i4	Glyma.14G048100.1	97.13114754	344	15	641	3045	505	2891	0	2398

TRINITY_DN10789_c0_g1_i1	Glyma.10G042300.2	100	217	48	621	2921	312	2687	0	2357

TRINITY_DN10789_c0_g1_i1	Glyma.10G042300.1	100	217	48	621	2921	312	2687	0	2357

TRINITY_DN325_c0_g1_i1	Glyma.15G213400.3	96.58119658	203	17	1	2404	3036	687	0	2933

TRINITY_DN325_c0_g1_i1	Glyma.15G213400.2	100	203	17	1	2404	3036	687	0	2933

TRINITY_DN325_c0_g1_i1	Glyma.15G213400.4	100	203	17	1	2404	3036	687	0	2933

TRINITY_DN325_c0_g1_i1	Glyma.15G213400.1	100	203	17	1	2404	3036	687	0	2933

TRINITY_DN15851_c0_g1_i2	Glyma.10G175900.1	100	183	5	226	2624	2472	68	0	3367

TRINITY_DN5366_c0_g1_i1	Glyma.02G159100.4	100	179	9	1445	3844	2448	52	0	3343

TRINITY_DN5366_c0_g2_i1	Glyma.02G159100.4	95.67099567	179	9	917	3316	2448	52	0	3343

TRINITY_DN10754_c0_g1_i1	Glyma.08G335200.1	100	175	5	268	2671	2505	103	0	3430

TRINITY_DN15851_c0_g1_i2	Glyma.20G214600.6	98.63945578	170	7	226	2624	2469	68	0	3419

TRINITY_DN15851_c0_g1_i2	Glyma.20G214600.8	97	170	7	226	2624	2469	68	0	3419

TRINITY_DN15851_c0_g1_i2	Glyma.20G214600.1	100	170	7	226	2624	2469	68	0	3419

TRINITY_DN15851_c0_g1_i2	Glyma.20G214600.4	99.05362776	170	7	226	2624	2469	68	0	3419

TRINITY_DN2555_c0_g2_i1	Glyma.04G234800.7	100	163	12	396	2783	1	2403	0	3393

TRINITY_DN2555_c0_g2_i1	Glyma.04G234800.6	100	163	12	396	2783	1	2403	0	3393

TRINITY_DN2555_c0_g2_i1	Glyma.04G234800.5	100	163	12	396	2783	1	2403	0	3393

TRINITY_DN2555_c0_g2_i1	Glyma.04G234800.4	97.5	163	12	396	2783	1	2403	0	3393

TRINITY_DN2555_c0_g2_i1	Glyma.04G234800.2	99.3993994	163	12	396	2783	1	2403	0	3393

TRINITY_DN2555_c0_g2_i1	Glyma.04G234800.3	98.48484848	163	12	396	2783	1	2403	0	3393

TRINITY_DN2555_c0_g2_i1	Glyma.04G234800.1	95.91836735	163	12	396	2783	1	2403	0	3393

TRINITY_DN14622_c0_g1_i3	Glyma.20G210700.3	95.55214724	157	6	274	2670	86	2482	0	3458

TRINITY_DN10975_c0_g1_i2	Glyma.07G001500.2	100	126	0	363	2768	1	2406	0	3746

TRINITY_DN10975_c0_g1_i2	Glyma.07G001500.1	98.94736842	126	0	363	2768	1	2406	0	3746

TRINITY_DN10975_c0_g1_i2	Glyma.08G225500.2	95.39473684	124	0	363	2768	1	2406	0	3757

TRINITY_DN10975_c0_g1_i2	Glyma.08G225500.1	95.39641944	124	0	363	2768	1	2406	0	3757

TRINITY_DN14887_c0_g1_i1	Glyma.01G239000.1	100	111	3	228	2622	2406	1	0	3757

TRINITY_DN14887_c0_g1_i1	Glyma.11G004600.1	96.11650485	102	3	228	2622	2406	1	0	3807

TRINITY_DN14362_c0_g1_i2	Glyma.16G076500.1	99.59432049	341	27	220	2586	58	2448	0	2217

TRINITY_DN15323_c0_g1_i2	Glyma.10G094500.1	96.23529412	286	15	219	2611	1	2390	0	2682

TRINITY_DN15323_c0_g1_i2	Glyma.10G094500.3	100	286	15	219	2611	1	2390	0	2682

TRINITY_DN15323_c0_g1_i2	Glyma.10G094500.2	98.82352941	286	15	219	2611	1	2390	0	2682

TRINITY_DN15323_c0_g1_i2	Glyma.10G094500.4	95.18518519	286	15	219	2611	1	2390	0	2682

TRINITY_DN14622_c0_g1_i2	Glyma.10G179700.3	95.45454545	193	17	67	2460	3	2386	0	3166

TRINITY_DN131_c0_g1_i1	Glyma.20G238000.2	100	137	2	474	2878	3363	962	0	3663

TRINITY_DN131_c0_g1_i1	Glyma.20G238000.1	100	137	2	474	2878	3363	962	0	3663

TRINITY_DN131_c0_g2_i1	Glyma.20G238000.2	100	137	2	228	2632	3363	962	0	3663

TRINITY_DN131_c0_g2_i1	Glyma.20G238000.1	100	137	2	228	2632	3363	962	0	3663

TRINITY_DN131_c0_g1_i1	Glyma.20G238000.3	100	137	2	474	2878	3228	827	0	3663

TRINITY_DN131_c0_g2_i1	Glyma.20G238000.3	100	137	2	228	2632	3228	827	0	3663

TRINITY_DN131_c0_g1_i1	Glyma.20G238000.4	100	137	2	474	2878	2889	488	0	3663

TRINITY_DN131_c0_g2_i1	Glyma.20G238000.4	100	137	2	228	2632	2889	488	0	3663

TRINITY_DN15560_c0_g1_i1	Glyma.03G163900.2	97.06314244	391	30	204	2588	1	2379	0	1989

TRINITY_DN14670_c0_g1_i3	Glyma.13G065500.9	96.90721649	240	18	993	3390	1	2362	0	2800

TRINITY_DN8961_c0_g1_i1	Glyma.14G040900.2	98.11320755	230	19	538	2931	1	2382	0	2959

TRINITY_DN15979_c0_g3_i1	Glyma.04G214200.2	100	204	13	409	2797	2502	108	0	3155

TRINITY_DN15979_c0_g3_i1	Glyma.04G214200.6	96.36363636	204	13	409	2797	2502	108	0	3155

TRINITY_DN15979_c0_g3_i1	Glyma.04G214200.5	96.875	204	13	409	2797	2502	108	0	3155

TRINITY_DN15979_c0_g3_i2	Glyma.04G214200.2	99.14529915	204	13	409	2797	2502	108	0	3155

TRINITY_DN15979_c0_g3_i2	Glyma.04G214200.6	96.58536585	204	13	409	2797	2502	108	0	3155

TRINITY_DN15979_c0_g3_i2	Glyma.04G214200.5	95.86956522	204	13	409	2797	2502	108	0	3155

TRINITY_DN14432_c0_g1_i1	Glyma.17G233000.1	100	138	14	210	2583	88	2485	0	3443

TRINITY_DN14362_c0_g1_i2	Glyma.16G109200.1	99.59349593	334	22	220	2586	58	2448	0	2278

TRINITY_DN16045_c0_g2_i1	Glyma.15G013000.2	100	282	8	1059	3454	2399	1	0	2804

TRINITY_DN15568_c1_g1_i1	Glyma.12G214600.3	98.74476987	238	4	551	2935	1	2403	0	3003

TRINITY_DN15568_c1_g1_i1	Glyma.12G214600.2	98.75	238	4	551	2935	1	2403	0	3003

TRINITY_DN15568_c1_g1_i2	Glyma.12G214600.3	98.68421053	238	4	551	2935	1	2403	0	3003

TRINITY_DN15568_c1_g1_i2	Glyma.12G214600.2	98.11320755	238	4	551	2935	1	2403	0	3003

TRINITY_DN16233_c0_g2_i1	Glyma.18G058900.2	96.90721649	229	0	237	2639	2403	1	0	3169

TRINITY_DN16233_c0_g2_i3	Glyma.18G058900.2	96.90265487	229	0	237	2639	2403	1	0	3169

TRINITY_DN3038_c0_g1_i1	Glyma.17G043400.1	100	218	3	97	2493	19	2421	0	3192

TRINITY_DN16233_c0_g2_i2	Glyma.18G058900.2	97.0917226	160	0	407	2809	2403	1	0	3552

TRINITY_DN16233_c0_g2_i4	Glyma.18G058900.2	100	160	0	407	2809	2403	1	0	3552

TRINITY_DN11254_c0_g1_i1	Glyma.08G189200.1	98.85057471	448	27	142	2525	2582	196	0	1740

TRINITY_DN16254_c0_g1_i5	Glyma.07G004300.2	98.25581395	205	3	1	2402	2486	88	0	3280

TRINITY_DN11259_c0_g1_i2	Glyma.12G170500.1	100	188	1	176	2577	98	2496	0	3374

TRINITY_DN16379_c0_g2_i5	Glyma.12G177100.1	97.8021978	444	15	313	2706	2424	40	0	1825

TRINITY_DN15235_c0_g1_i4	Glyma.16G172400.5	99.09090909	418	20	1643	4032	68	2457	0	1977

TRINITY_DN15235_c0_g1_i7	Glyma.16G172400.2	96.66666667	391	22	219	2606	1	2388	0	2100

TRINITY_DN15235_c0_g1_i3	Glyma.16G172400.2	96.28482972	387	22	310	2697	1	2388	0	2122

TRINITY_DN16254_c0_g1_i4	Glyma.17G057800.4	95	283	19	995	3383	2753	362	0	2730

TRINITY_DN16254_c0_g1_i4	Glyma.17G057800.3	95	283	19	995	3383	2753	362	0	2730

TRINITY_DN16254_c0_g1_i4	Glyma.17G057800.2	97.58064516	283	19	995	3383	2753	362	0	2730

TRINITY_DN16426_c2_g1_i4	Glyma.13G152600.1	97.5	265	2	358	2756	1	2399	0	2940

TRINITY_DN15876_c0_g1_i2	Glyma.04G039400.1	98.16513761	219	6	107	2500	1	2400	0	3169

TRINITY_DN16045_c0_g2_i2	Glyma.15G013000.2	98.48484848	214	4	1059	3457	2399	1	0	3223

TRINITY_DN17367_c0_g1_i1	Glyma.14G001400.1	100	177	0	69	2469	54	2454	0	3454

TRINITY_DN12591_c0_g1_i1	Glyma.16G084200.5	95.53072626	137	11	531	2922	2521	128	0	3572

TRINITY_DN12591_c0_g1_i2	Glyma.16G084200.5	97.73333333	137	11	503	2894	2521	128	0	3572

TRINITY_DN15235_c0_g1_i2	Glyma.16G172400.5	98.8372093	419	20	1643	4031	68	2456	0	1969

TRINITY_DN13423_c0_g2_i1	Glyma.15G143000.1	98.8372093	362	13	1094	3483	2378	1	0	2220

TRINITY_DN7939_c0_g1_i1	Glyma.16G128300.1	100	181	2	225	2621	1	2397	0	3391

TRINITY_DN7939_c0_g1_i2	Glyma.16G128300.1	100	174	13	225	2594	1	2397	0	3254

TRINITY_DN16360_c0_g4_i1	Glyma.13G104000.1	95.01718213	552	56	449	2820	121	2477	0	926

TRINITY_DN16086_c0_g2_i1	Glyma.15G162600.1	97.6284585	425	24	279	2664	21	2406	0	1908

TRINITY_DN16138_c1_g1_i22	Glyma.15G049300.1	95.03546099	382	18	311	2697	1	2387	0	2159

TRINITY_DN15323_c0_g1_i2	Glyma.02G180100.2	96.36363636	275	16	219	2603	1	2382	0	2706

TRINITY_DN15323_c0_g1_i2	Glyma.02G180100.1	99.61977186	275	16	219	2603	1	2382	0	2706

TRINITY_DN16086_c0_g2_i4	Glyma.14G134100.1	98.8	271	6	100	2495	1	2396	0	2891

TRINITY_DN16086_c0_g2_i4	Glyma.17G199200.1	98.7244898	266	6	100	2495	1	2396	0	2918

TRINITY_DN16106_c0_g3_i5	Glyma.14G128300.1	97.77777778	263	18	299	2690	2682	318	0	2708

TRINITY_DN16106_c0_g3_i5	Glyma.14G128300.3	100	263	18	299	2690	2652	288	0	2708

TRINITY_DN16106_c0_g3_i5	Glyma.14G128300.2	98.52941176	263	18	299	2690	2598	234	0	2708

TRINITY_DN16426_c2_g1_i4	Glyma.18G044500.1	99.4047619	253	6	358	2756	1	2390	0	2970

TRINITY_DN16426_c2_g1_i4	Glyma.18G044500.2	95.65916399	253	6	358	2756	1	2390	0	2970

TRINITY_DN3514_c0_g1_i1	Glyma.02G128900.1	100	182	5	1083	3479	2394	1	0	3376

TRINITY_DN16426_c2_g1_i4	Glyma.13G152700.1	99.4047619	170	0	358	2756	1	2399	0	3489

TRINITY_DN16426_c2_g1_i4	Glyma.18G044600.1	95.65217391	166	0	358	2756	1	2399	0	3511

TRINITY_DN666_c0_g1_i1	Glyma.15G136900.3	95.20153551	156	3	1	2375	843	3238	0	3391

TRINITY_DN16254_c0_g1_i10	Glyma.07G004300.2	96	146	0	1	2399	2486	88	0	3622

TRINITY_DN16086_c0_g2_i1	Glyma.09G056300.1	100	433	22	279	2664	207	2592	0	1875

TRINITY_DN16086_c0_g2_i1	Glyma.09G056300.2	98.98989899	433	22	279	2664	21	2406	0	1875

TRINITY_DN16086_c0_g2_i4	Glyma.15G162600.1	100	428	24	109	2493	22	2406	0	1890

TRINITY_DN13055_c0_g1_i1	Glyma.13G212900.5	97.22222222	414	18	253	2629	1	2389	0	1941

TRINITY_DN13055_c0_g1_i1	Glyma.13G212900.3	97.22222222	414	18	253	2629	1	2389	0	1941

TRINITY_DN13055_c0_g1_i1	Glyma.13G212900.2	98.71794872	414	18	253	2629	1	2389	0	1941

TRINITY_DN16138_c1_g1_i21	Glyma.15G049300.1	98.33887043	378	23	311	2685	1	2387	0	2115

TRINITY_DN16031_c1_g2_i5	Glyma.07G235800.2	100	319	20	129	2516	2373	1	0	2435

TRINITY_DN16086_c0_g2_i1	Glyma.14G134100.1	100	235	4	271	2666	1	2396	0	3101

TRINITY_DN16086_c0_g2_i1	Glyma.17G199200.1	100	229	4	271	2666	1	2396	0	3134

TRINITY_DN3038_c0_g1_i1	Glyma.13G116400.1	100	213	2	97	2491	16	2413	0	3229

TRINITY_DN14332_c0_g1_i2	Glyma.U025000.2	99.57754979	208	5	1	2396	760	3152	0	3230

TRINITY_DN14332_c0_g1_i2	Glyma.U025000.1	99.57754979	208	5	1	2396	760	3152	0	3230

TRINITY_DN14332_c0_g1_i2	Glyma.06G238700.1	99.57805907	205	8	1	2396	760	3149	0	3229

TRINITY_DN17367_c0_g1_i1	Glyma.03G215200.1	97.5	180	2	73	2469	1	2397	0	3419

TRINITY_DN16086_c0_g2_i1	Glyma.06G076100.1	100	167	4	271	2666	1	2396	0	3478

TRINITY_DN16138_c1_g1_i11	Glyma.15G049300.1	96.35258359	346	15	311	2697	1	2387	0	2381

TRINITY_DN12238_c0_g1_i2	Glyma.19G014100.2	97.6	251	20	565	2948	44	2415	0	2791

TRINITY_DN9350_c0_g1_i1	Glyma.10G019200.4	97.14285714	240	9	604	2997	1	2385	0	3000

TRINITY_DN16415_c1_g1_i7	Glyma.U018900.1	95.95375723	214	5	1364	3760	2404	17	0	3182

TRINITY_DN7121_c0_g1_i1	Glyma.12G181600.1	100	191	22	302	2692	2577	217	0	3097

TRINITY_DN13448_c0_g1_i6	Glyma.08G100800.1	99.43820225	185	7	1	2389	27	2415	0	3299

TRINITY_DN5558_c0_g2_i1	Glyma.14G216700.1	97.6	184	21	151	2529	2385	1	0	3214

TRINITY_DN13448_c0_g1_i6	Glyma.08G100800.2	98.4375	182	18	1	2389	27	2394	0	3179

TRINITY_DN10862_c0_g1_i1	Glyma.08G357000.1	98.88888889	178	5	89	2479	1	2391	0	3363

TRINITY_DN10862_c0_g1_i1	Glyma.18G175500.1	100	173	5	89	2479	1	2391	0	3391

TRINITY_DN16086_c0_g2_i1	Glyma.04G075100.2	96.92307692	154	2	271	2666	1	2396	0	3561

TRINITY_DN16086_c0_g2_i1	Glyma.04G075100.1	99.64539007	154	2	271	2666	1	2396	0	3561

TRINITY_DN16086_c0_g2_i4	Glyma.06G076100.1	100	130	2	100	2495	1	2396	0	3694

TRINITY_DN15560_c0_g1_i1	Glyma.19G165200.1	100	370	27	223	2588	71	2445	0	2045

TRINITY_DN15560_c0_g1_i2	Glyma.19G165200.1	96.80589681	370	27	291	2656	71	2445	0	2045

TRINITY_DN13972_c0_g1_i2	Glyma.14G209900.1	98.86363636	344	14	276	2664	2443	55	0	2429

TRINITY_DN12865_c0_g1_i2	Glyma.05G202900.2	100	236	20	220	2583	1	2391	0	2878

TRINITY_DN16352_c0_g2_i1	Glyma.08G194600.1	95.32871972	228	10	2521	4897	2395	1	0	3033

TRINITY_DN15568_c1_g1_i1	Glyma.13G287000.2	100	224	12	551	2935	1	2385	0	3042

TRINITY_DN15568_c1_g1_i2	Glyma.13G287000.2	98.68421053	224	12	551	2935	1	2385	0	3042

TRINITY_DN15517_c0_g2_i3	Glyma.02G289700.1	95.49071618	195	6	29	2419	2481	91	0	3280

TRINITY_DN12719_c0_g2_i5	Glyma.13G321300.53	100	163	29	1	2361	2346	1	0	2972

TRINITY_DN12719_c0_g2_i5	Glyma.13G321300.36	100	163	29	1	2361	2346	1	0	2972

TRINITY_DN12719_c0_g2_i5	Glyma.13G321300.37	100	163	29	1	2361	2346	1	0	2972

TRINITY_DN12719_c0_g2_i5	Glyma.13G321300.24	100	163	29	1	2361	2346	1	0	2972

TRINITY_DN12719_c0_g2_i5	Glyma.13G321300.25	100	163	29	1	2361	2346	1	0	2972

TRINITY_DN12719_c0_g2_i5	Glyma.13G321300.4	100	163	29	1	2361	2346	1	0	2972

TRINITY_DN12719_c0_g2_i5	Glyma.13G321300.5	95.8073149	163	29	1	2361	2346	1	0	2972

TRINITY_DN12719_c0_g2_i7	Glyma.13G321300.53	96.03603604	163	29	1	2361	2346	1	0	2972

TRINITY_DN12719_c0_g2_i7	Glyma.13G321300.36	97.54098361	163	29	1	2361	2346	1	0	2972

TRINITY_DN12719_c0_g2_i7	Glyma.13G321300.37	97.70114943	163	29	1	2361	2346	1	0	2972

TRINITY_DN12719_c0_g2_i7	Glyma.13G321300.24	100	163	29	1	2361	2346	1	0	2972

TRINITY_DN12719_c0_g2_i7	Glyma.13G321300.25	100	163	29	1	2361	2346	1	0	2972

TRINITY_DN12719_c0_g2_i7	Glyma.13G321300.4	98.73417722	163	29	1	2361	2346	1	0	2972

TRINITY_DN12719_c0_g2_i7	Glyma.13G321300.5	98.125	163	29	1	2361	2346	1	0	2972

TRINITY_DN16086_c0_g2_i4	Glyma.04G075100.2	98.75	122	0	100	2495	1	2396	0	3749

TRINITY_DN16086_c0_g2_i4	Glyma.04G075100.1	100	122	0	100	2495	1	2396	0	3749

TRINITY_DN16086_c0_g2_i4	Glyma.09G056300.1	96	438	18	109	2493	208	2592	0	1868

TRINITY_DN16086_c0_g2_i4	Glyma.09G056300.2	96	438	18	109	2493	22	2406	0	1868

TRINITY_DN16138_c1_g1_i22	Glyma.08G183400.7	97.72727273	403	12	311	2697	1	2387	0	2087

TRINITY_DN16138_c1_g1_i22	Glyma.08G183400.8	98.67172676	403	12	311	2697	1	2387	0	2087

TRINITY_DN16138_c1_g1_i22	Glyma.08G183400.9	100	403	12	311	2697	1	2387	0	2087

TRINITY_DN16138_c1_g1_i21	Glyma.08G183400.7	95.91836735	400	16	311	2685	1	2387	0	2026

TRINITY_DN16138_c1_g1_i21	Glyma.08G183400.8	97.84946237	400	16	311	2685	1	2387	0	2026

TRINITY_DN16138_c1_g1_i21	Glyma.08G183400.9	96.62921348	400	16	311	2685	1	2387	0	2026

TRINITY_DN16138_c1_g1_i11	Glyma.08G183400.7	98.07692308	359	12	311	2697	1	2387	0	2331

TRINITY_DN16138_c1_g1_i11	Glyma.08G183400.8	96	359	12	311	2697	1	2387	0	2331

TRINITY_DN16138_c1_g1_i11	Glyma.08G183400.9	96	359	12	311	2697	1	2387	0	2331

TRINITY_DN13423_c0_g2_i1	Glyma.09G037900.1	95.61170213	345	18	1101	3483	2368	1	0	2259

TRINITY_DN16138_c1_g1_i18	Glyma.15G049300.1	98	336	15	300	2678	1	2387	0	2407

TRINITY_DN16138_c1_g1_i5	Glyma.15G049300.1	95.82309582	336	15	566	2944	1	2387	0	2407

TRINITY_DN16031_c1_g2_i5	Glyma.17G037600.1	95.2	327	18	132	2516	2370	1	0	2385

TRINITY_DN16031_c1_g2_i2	Glyma.07G235800.2	96.66666667	319	20	1008	3392	2370	1	0	2429

TRINITY_DN16031_c1_g2_i3	Glyma.07G235800.2	98.58490566	319	20	727	3111	2370	1	0	2429

TRINITY_DN16031_c1_g2_i5	Glyma.07G235800.1	95.2	319	20	132	2516	2370	1	0	2429

TRINITY_DN14680_c0_g2_i4	Glyma.19G175600.1	99.56709957	232	20	1	2374	374	2750	0	2885

TRINITY_DN14680_c0_g2_i4	Glyma.19G175600.2	97.92899408	232	20	1	2374	374	2750	0	2885

TRINITY_DN6110_c0_g1_i1	Glyma.09G207500.1	99.09638554	189	17	681	3018	2395	1	0	3007

TRINITY_DN15539_c0_g1_i4	Glyma.17G259200.1	96	498	44	157	2512	18	2385	0	1249

TRINITY_DN16138_c1_g1_i20	Glyma.15G049300.1	100	332	20	300	2666	1	2387	0	2362

TRINITY_DN16138_c1_g1_i7	Glyma.15G049300.1	97.08029197	332	20	566	2932	1	2387	0	2362

TRINITY_DN16169_c0_g1_i2	Glyma.10G292400.2	95.62624254	209	9	513	2882	4071	1690	0	3031

TRINITY_DN16169_c0_g1_i3	Glyma.10G292400.2	100	209	9	513	2882	4071	1690	0	3031

TRINITY_DN4222_c0_g1_i1	Glyma.17G008400.1	96.96969697	208	5	332	2716	211	2604	0	3210

TRINITY_DN5558_c0_g2_i1	Glyma.17G255300.2	99.53271028	192	16	151	2529	2382	1	0	3182

TRINITY_DN13972_c0_g1_i2	Glyma.02G240400.1	96.03960396	350	6	276	2665	2443	54	0	2442

TRINITY_DN16138_c1_g1_i18	Glyma.08G077100.1	96.90402477	233	6	300	2679	1	2391	0	3031

TRINITY_DN16138_c1_g1_i5	Glyma.08G077100.1	96.8	233	6	566	2945	1	2391	0	3031

TRINITY_DN16138_c1_g1_i20	Glyma.08G077100.1	99.39393939	233	7	300	2667	1	2391	0	2953

TRINITY_DN16138_c1_g1_i7	Glyma.08G077100.1	95.04950495	233	7	566	2933	1	2391	0	2953

TRINITY_DN9810_c0_g1_i2	Glyma.07G234900.4	100	186	13	138	2530	83	2448	0	3214

TRINITY_DN9810_c0_g1_i2	Glyma.07G234900.2	100	186	13	138	2530	5	2370	0	3214

TRINITY_DN15878_c0_g2_i2	Glyma.19G260900.1	95.2173913	348	45	491	2844	2460	106	0	2004

TRINITY_DN12988_c0_g2_i1	Glyma.13G127100.1	100	327	12	60	2443	350	2733	0	2503

TRINITY_DN16168_c0_g1_i1	Glyma.08G057200.4	96.85393258	302	16	241	2622	2383	11	0	2556

TRINITY_DN14621_c1_g1_i1	Glyma.05G072300.1	100	257	14	186	2575	89	2466	0	2891

TRINITY_DN16138_c1_g1_i11	Glyma.08G077100.1	95.19650655	220	3	311	2698	1	2391	0	3166

TRINITY_DN14621_c1_g1_i3	Glyma.05G072300.1	98.18181818	217	14	186	2575	89	2466	0	3112

TRINITY_DN14622_c0_g1_i2	Glyma.10G179700.1	99.18032787	191	16	78	2460	97	2467	0	3166

TRINITY_DN14622_c0_g1_i2	Glyma.10G179700.2	97.14285714	191	16	78	2460	88	2458	0	3166

TRINITY_DN4552_c0_g1_i1	Glyma.08G133700.1	98.63013699	189	2	331	2716	2392	1	0	3332

TRINITY_DN15539_c0_g1_i4	Glyma.14G220300.1	100	502	44	157	2512	18	2382	0	1240

TRINITY_DN16082_c0_g1_i6	Glyma.06G086600.1	97.05882353	476	39	552	2909	2392	20	0	1448

TRINITY_DN16082_c0_g1_i6	Glyma.04G085000.1	95.94017094	469	42	552	2909	2389	20	0	1469

TRINITY_DN16138_c1_g1_i18	Glyma.08G183400.7	96	359	9	300	2678	1	2387	0	2324

TRINITY_DN16138_c1_g1_i18	Glyma.08G183400.8	98.63013699	359	9	300	2678	1	2387	0	2324

TRINITY_DN16138_c1_g1_i18	Glyma.08G183400.9	98.63013699	359	9	300	2678	1	2387	0	2324

TRINITY_DN16138_c1_g1_i5	Glyma.08G183400.7	95.55555556	359	9	566	2944	1	2387	0	2324

TRINITY_DN16138_c1_g1_i5	Glyma.08G183400.8	98.98989899	359	9	566	2944	1	2387	0	2324

TRINITY_DN16138_c1_g1_i5	Glyma.08G183400.9	96.2962963	359	9	566	2944	1	2387	0	2324

TRINITY_DN16138_c1_g1_i20	Glyma.08G183400.7	96.79487179	356	13	300	2666	1	2387	0	2263

TRINITY_DN16138_c1_g1_i20	Glyma.08G183400.8	100	356	13	300	2666	1	2387	0	2263

TRINITY_DN16138_c1_g1_i20	Glyma.08G183400.9	100	356	13	300	2666	1	2387	0	2263

TRINITY_DN16138_c1_g1_i7	Glyma.08G183400.7	100	356	13	566	2932	1	2387	0	2263

TRINITY_DN16138_c1_g1_i7	Glyma.08G183400.8	97.76536313	356	13	566	2932	1	2387	0	2263

TRINITY_DN16138_c1_g1_i7	Glyma.08G183400.9	99.62121212	356	13	566	2932	1	2387	0	2263

TRINITY_DN12362_c0_g1_i2	Glyma.18G023500.1	97.39130435	347	18	78	2442	2556	171	0	2292

TRINITY_DN12362_c0_g1_i3	Glyma.18G023500.1	96.49805447	347	18	78	2442	2556	171	0	2292

TRINITY_DN4222_c0_g1_i1	Glyma.07G265600.1	100	216	5	332	2716	223	2604	0	3121

TRINITY_DN16345_c0_g1_i7	Glyma.08G044700.3	98.30508475	215	6	1145	3530	2873	491	0	3140

TRINITY_DN16345_c0_g1_i7	Glyma.08G044700.7	98.31460674	215	6	1145	3530	2594	212	0	3140

TRINITY_DN14252_c0_g1_i1	Glyma.17G109200.1	100	187	1	682	3069	2391	1	0	3360

TRINITY_DN14252_c0_g1_i1	Glyma.17G109200.3	100	187	1	682	3069	2391	1	0	3360

TRINITY_DN14252_c0_g1_i1	Glyma.13G162200.1	100	178	3	682	3069	2388	1	0	3391

TRINITY_DN16138_c1_g1_i22	Glyma.08G077100.1	97.36842105	175	1	311	2698	1	2391	0	3426

TRINITY_DN16138_c1_g1_i21	Glyma.08G077100.1	95.12195122	175	2	311	2686	1	2391	0	3349

TRINITY_DN16106_c0_g3_i3	Glyma.06G072200.1	100	168	15	299	2644	2679	310	0	3059

TRINITY_DN16106_c0_g3_i5	Glyma.06G072200.1	100	168	16	299	2671	2679	310	0	3232

TRINITY_DN13775_c0_g1_i1	Glyma.03G169000.5	100	166	8	179	2539	49	2430	0	3243

TRINITY_DN13775_c0_g1_i1	Glyma.03G169000.3	96.66666667	166	8	179	2539	49	2430	0	3243

TRINITY_DN13775_c0_g1_i1	Glyma.03G169000.1	98.7804878	166	8	179	2539	49	2430	0	3243

TRINITY_DN13775_c0_g1_i1	Glyma.03G169000.4	97.08029197	166	8	179	2539	49	2430	0	3243

TRINITY_DN13775_c0_g1_i2	Glyma.03G169000.5	98.8372093	166	8	179	2539	49	2430	0	3243

TRINITY_DN13775_c0_g1_i2	Glyma.03G169000.3	100	166	8	179	2539	49	2430	0	3243

TRINITY_DN13775_c0_g1_i2	Glyma.03G169000.1	100	166	8	179	2539	49	2430	0	3243

TRINITY_DN13775_c0_g1_i2	Glyma.03G169000.4	100	166	8	179	2539	49	2430	0	3243

TRINITY_DN10291_c0_g1_i2	Glyma.18G274100.1	100	131	11	148	2529	1	2364	0	3458

TRINITY_DN16082_c0_g1_i2	Glyma.06G086600.1	98.36065574	485	36	581	2938	2392	20	0	1410

TRINITY_DN15560_c0_g1_i1	Glyma.19G165200.3	100	371	31	223	2588	71	2439	0	2067

TRINITY_DN15560_c0_g1_i2	Glyma.19G165200.3	97.27891156	371	31	291	2656	71	2439	0	2067

TRINITY_DN15560_c0_g1_i2	Glyma.19G165200.2	97.27891156	371	31	291	2656	20	2388	0	2067

TRINITY_DN10737_c0_g1_i1	Glyma.19G114400.1	98.57142857	216	15	95	2461	1	2385	0	3037

TRINITY_DN11133_c0_g1_i1	Glyma.09G039000.1	99.72677596	205	17	101	2461	2832	452	0	3033

TRINITY_DN11133_c0_g1_i1	Glyma.09G039000.6	100	205	17	101	2461	2832	452	0	3033

TRINITY_DN11133_c0_g1_i1	Glyma.09G039000.4	95.34883721	205	17	101	2461	2808	428	0	3033

TRINITY_DN11133_c0_g1_i1	Glyma.09G039000.5	96.42857143	205	17	101	2461	2808	428	0	3033

TRINITY_DN11133_c0_g1_i1	Glyma.09G039000.2	96.42857143	205	17	101	2461	2754	374	0	3033

TRINITY_DN11133_c0_g1_i1	Glyma.09G039000.3	100	205	17	101	2461	2754	374	0	3033

TRINITY_DN13448_c0_g1_i6	Glyma.05G144400.3	96.54255319	198	3	1	2389	27	2409	0	3265

TRINITY_DN13448_c0_g1_i6	Glyma.05G144400.1	100	198	3	1	2389	27	2409	0	3265

TRINITY_DN9810_c0_g1_i2	Glyma.07G234900.5	100	186	13	141	2530	38	2400	0	3208

TRINITY_DN9810_c0_g1_i2	Glyma.07G234900.3	100	186	13	141	2530	38	2400	0	3208

TRINITY_DN14622_c0_g1_i7	Glyma.10G179700.6	97.05882353	179	8	193	2573	6	2385	0	3299

TRINITY_DN15891_c0_g1_i1	Glyma.16G048100.1	96.91780822	149	0	863	3252	647	3036	0	3589

TRINITY_DN13959_c0_g1_i1	Glyma.10G292300.3	100	145	27	319	2657	2918	535	0	3241

TRINITY_DN13959_c0_g1_i2	Glyma.10G292300.3	96.56750572	145	27	319	2657	2918	535	0	3241

TRINITY_DN11893_c0_g1_i1	Glyma.02G200700.6	98	138	2	432	2818	2	2391	0	3629

TRINITY_DN16082_c0_g1_i2	Glyma.04G085000.1	95.67198178	479	38	581	2938	2389	20	0	1435

TRINITY_DN14245_c0_g2_i1	Glyma.15G072000.3	95.65217391	356	18	451	2811	14	2389	0	2174

TRINITY_DN16168_c0_g1_i3	Glyma.08G057200.4	96.01873536	164	1	241	2629	2383	1	0	3465

TRINITY_DN14432_c0_g1_i1	Glyma.U029000.4	100	152	8	210	2583	88	2473	0	3454

TRINITY_DN14432_c0_g1_i1	Glyma.U029000.3	100	152	8	210	2583	88	2473	0	3454

TRINITY_DN14432_c0_g1_i1	Glyma.U029000.1	100	152	8	210	2583	88	2473	0	3454

TRINITY_DN14432_c0_g1_i1	Glyma.U029000.2	100	152	8	210	2583	88	2473	0	3454

TRINITY_DN15851_c0_g1_i1	Glyma.16G207900.1	95.10086455	325	44	227	2582	2411	68	0	2119

TRINITY_DN15851_c0_g1_i1	Glyma.16G207900.3	100	325	44	227	2582	2411	68	0	2119

TRINITY_DN15851_c0_g1_i1	Glyma.16G207900.2	99.35483871	325	44	227	2582	2411	68	0	2119

TRINITY_DN15851_c0_g1_i1	Glyma.16G207900.4	97.14285714	325	44	227	2582	2411	68	0	2119

TRINITY_DN9549_c0_g1_i1	Glyma.11G081500.2	98.91304348	212	13	113	2478	1	2384	0	3068

TRINITY_DN14252_c0_g1_i1	Glyma.17G109200.2	100	188	2	682	3069	2385	1	0	3349

TRINITY_DN36295_c0_g1_i1	Glyma.16G208400.6	95.23809524	187	16	66	2436	10	2391	0	3225

TRINITY_DN36295_c0_g1_i1	Glyma.16G208400.7	99.35483871	187	16	66	2436	10	2391	0	3225

TRINITY_DN36295_c0_g1_i1	Glyma.16G208400.5	99.35483871	187	16	66	2436	10	2391	0	3225

TRINITY_DN36295_c0_g1_i1	Glyma.16G208400.12	100	187	16	66	2436	10	2391	0	3225

TRINITY_DN36295_c0_g1_i1	Glyma.16G208400.8	100	187	16	66	2436	10	2391	0	3225

TRINITY_DN36295_c0_g1_i1	Glyma.16G208400.1	100	187	16	66	2436	10	2391	0	3225

TRINITY_DN36295_c0_g1_i1	Glyma.16G208400.9	95.04132231	187	16	66	2436	10	2391	0	3225

TRINITY_DN36295_c0_g1_i1	Glyma.16G208400.2	100	187	16	66	2436	10	2391	0	3225

TRINITY_DN36295_c0_g1_i1	Glyma.16G208400.3	95.21178637	187	16	66	2436	10	2391	0	3225

TRINITY_DN36295_c0_g1_i1	Glyma.16G208400.4	95.21276596	187	16	66	2436	10	2391	0	3225

TRINITY_DN9498_c0_g1_i2	Glyma.14G065300.1	99.24528302	166	8	1	2382	10	2385	0	3374

TRINITY_DN14556_c0_g1_i2	Glyma.09G252000.1	98.7804878	147	1	227	2614	1	2382	0	3557

TRINITY_DN14801_c0_g2_i1	Glyma.06G115600.1	97.90026247	399	42	259	2598	2358	1	0	1707

TRINITY_DN14208_c0_g1_i1	Glyma.13G113900.1	97.2972973	224	13	897	3258	2718	5103	0	3000

TRINITY_DN14208_c0_g1_i1	Glyma.13G113900.2	97.2972973	224	13	897	3258	1761	4146	0	3000

TRINITY_DN14208_c0_g1_i1	Glyma.13G113900.3	97.2972973	224	13	897	3258	1719	4104	0	3000

TRINITY_DN14208_c0_g1_i1	Glyma.13G113900.4	96.5785381	224	13	897	3258	1599	3984	0	3000

TRINITY_DN14208_c0_g1_i1	Glyma.13G113900.5	97.2972973	224	13	897	3258	1596	3981	0	3000

TRINITY_DN12719_c0_g2_i6	Glyma.12G179300.5	100	173	14	1	2376	2358	1	0	3192

TRINITY_DN12719_c0_g2_i6	Glyma.12G179300.4	100	173	14	1	2376	2358	1	0	3192

TRINITY_DN12719_c0_g2_i6	Glyma.12G179300.3	95.23809524	173	14	1	2376	2358	1	0	3192

TRINITY_DN12719_c0_g2_i6	Glyma.12G179300.1	96.40718563	173	14	1	2376	2358	1	0	3192

TRINITY_DN12719_c0_g2_i8	Glyma.12G179300.5	100	173	14	1	2376	2358	1	0	3192

TRINITY_DN12719_c0_g2_i8	Glyma.12G179300.4	100	173	14	1	2376	2358	1	0	3192

TRINITY_DN12719_c0_g2_i8	Glyma.12G179300.3	98.98989899	173	14	1	2376	2358	1	0	3192

TRINITY_DN12719_c0_g2_i8	Glyma.12G179300.1	95.11400651	173	14	1	2376	2358	1	0	3192

TRINITY_DN12719_c0_g2_i6	Glyma.13G321300.34	100	167	21	1	2376	2322	1	0	2992

TRINITY_DN12719_c0_g2_i6	Glyma.13G321300.35	100	167	21	1	2376	2322	1	0	2992

TRINITY_DN12719_c0_g2_i6	Glyma.13G321300.22	96.18320611	167	21	1	2376	2322	1	0	2992

TRINITY_DN12719_c0_g2_i6	Glyma.13G321300.23	99.16839917	167	21	1	2376	2322	1	0	2992

TRINITY_DN12719_c0_g2_i6	Glyma.13G321300.2	100	167	21	1	2376	2322	1	0	2992

TRINITY_DN12719_c0_g2_i6	Glyma.13G321300.3	100	167	21	1	2376	2322	1	0	2992

TRINITY_DN12719_c0_g2_i8	Glyma.13G321300.34	96.07843137	167	21	1	2376	2322	1	0	2992

TRINITY_DN12719_c0_g2_i8	Glyma.13G321300.35	96.07843137	167	21	1	2376	2322	1	0	2992

TRINITY_DN12719_c0_g2_i8	Glyma.13G321300.22	96.07843137	167	21	1	2376	2322	1	0	2992

TRINITY_DN12719_c0_g2_i8	Glyma.13G321300.23	96.07843137	167	21	1	2376	2322	1	0	2992

TRINITY_DN12719_c0_g2_i8	Glyma.13G321300.2	96.27659574	167	21	1	2376	2322	1	0	2992

TRINITY_DN12719_c0_g2_i8	Glyma.13G321300.3	100	167	21	1	2376	2322	1	0	2992

TRINITY_DN15751_c0_g1_i10	Glyma.06G172300.2	95.45454545	302	32	890	3232	1	2367	0	2333

TRINITY_DN15751_c0_g1_i10	Glyma.06G172300.3	95.45454545	302	32	890	3232	1	2367	0	2333

TRINITY_DN15751_c0_g1_i10	Glyma.06G172300.4	95.45454545	302	32	890	3232	1	2367	0	2333

TRINITY_DN15751_c0_g1_i10	Glyma.06G172300.1	95.6043956	302	32	890	3232	1	2367	0	2333

TRINITY_DN9851_c0_g1_i1	Glyma.11G215600.1	95.4954955	213	4	1	2382	718	3102	0	3193

TRINITY_DN9851_c0_g1_i2	Glyma.11G215600.1	100	213	4	1	2382	718	3102	0	3193

TRINITY_DN12362_c0_g1_i2	Glyma.02G261400.1	97.14285714	195	8	101	2465	2455	73	0	3171

TRINITY_DN12362_c0_g1_i3	Glyma.02G261400.1	100	195	8	101	2465	2455	73	0	3171

TRINITY_DN16214_c0_g2_i1	Glyma.02G282700.1	100	413	51	1787	4125	3010	648	0	1677

TRINITY_DN16214_c0_g2_i2	Glyma.02G282700.1	100	413	51	1716	4054	3010	648	0	1677

TRINITY_DN16214_c0_g2_i1	Glyma.02G282700.2	99.2	413	51	1787	4125	2935	573	0	1677

TRINITY_DN16214_c0_g2_i2	Glyma.02G282700.2	100	413	51	1716	4054	2935	573	0	1677

TRINITY_DN15560_c0_g1_i2	Glyma.03G163900.2	96.66666667	387	30	291	2656	20	2379	0	1977

TRINITY_DN16231_c1_g1_i1	Glyma.09G073600.3	100	311	10	114	2493	35	2414	0	2617

TRINITY_DN16231_c1_g1_i1	Glyma.09G073600.5	100	311	10	114	2493	35	2414	0	2617

TRINITY_DN16231_c1_g1_i1	Glyma.09G073600.2	100	311	10	114	2493	35	2414	0	2617

TRINITY_DN14146_c1_g1_i3	Glyma.20G228700.3	100	287	18	1005	3383	2364	1	0	2639

TRINITY_DN10737_c0_g1_i1	Glyma.16G038400.1	97.10743802	220	15	95	2461	1	2382	0	3049

TRINITY_DN9851_c0_g1_i1	Glyma.18G041000.1	96.89922481	217	1	1	2382	907	3291	0	3182

TRINITY_DN9851_c0_g1_i2	Glyma.18G041000.1	100	217	1	1	2382	907	3291	0	3182

TRINITY_DN15180_c1_g1_i4	Glyma.17G072500.1	97.2972973	176	10	194	2572	1	2370	0	3293

TRINITY_DN14423_c0_g1_i1	Glyma.10G272800.1	100	164	0	181	2565	1	2385	0	3496

TRINITY_DN17409_c0_g1_i1	Glyma.04G204500.1	95	160	6	17	2388	346	2729	0	3428

TRINITY_DN17409_c0_g1_i1	Glyma.04G204500.2	97.65625	160	6	17	2388	346	2729	0	3428

TRINITY_DN15440_c0_g1_i1	Glyma.02G124500.1	99.10714286	149	4	560	2932	2385	1	0	3502

TRINITY_DN16031_c1_g2_i5	Glyma.15G137000.1	99.10714286	147	0	132	2516	2385	1	0	3591

TRINITY_DN16031_c1_g2_i5	Glyma.15G137000.2	100	142	3	132	2516	2376	1	0	3559

TRINITY_DN15539_c0_g1_i4	Glyma.01G164100.1	97.33333333	118	0	140	2524	1	2385	0	3751

TRINITY_DN16345_c0_g1_i9	Glyma.08G044700.1	99.21259843	306	10	445	2822	3699	1328	0	2591

TRINITY_DN16345_c0_g1_i9	Glyma.08G044700.5	99.20948617	306	10	445	2822	3657	1286	0	2591

TRINITY_DN16345_c0_g1_i9	Glyma.08G044700.3	99.21259843	306	10	445	2822	3504	1133	0	2591

TRINITY_DN16345_c0_g1_i9	Glyma.08G044700.6	99.20634921	306	10	445	2822	3420	1049	0	2591

TRINITY_DN16345_c0_g1_i9	Glyma.08G044700.7	99.23076923	306	10	445	2822	3225	854	0	2591

TRINITY_DN16231_c1_g1_i1	Glyma.15G182600.1	100	299	8	114	2493	35	2414	0	2695

TRINITY_DN15751_c0_g1_i8	Glyma.06G172300.2	97.10982659	222	27	890	3235	1	2367	0	2817

TRINITY_DN15751_c0_g1_i8	Glyma.06G172300.3	97.11111111	222	27	890	3235	1	2367	0	2817

TRINITY_DN15751_c0_g1_i8	Glyma.06G172300.4	97.11191336	222	27	890	3235	1	2367	0	2817

TRINITY_DN15751_c0_g1_i8	Glyma.06G172300.1	98.03921569	222	27	890	3235	1	2367	0	2817

TRINITY_DN16130_c2_g6_i6	Glyma.10G075300.2	96.42857143	197	0	337	2720	407	2790	0	3312

TRINITY_DN16130_c2_g6_i6	Glyma.02G201300.2	98.85386819	188	0	337	2720	404	2787	0	3362

TRINITY_DN16130_c2_g6_i6	Glyma.02G201300.4	99.2481203	188	0	337	2720	404	2787	0	3362

TRINITY_DN16130_c2_g6_i6	Glyma.02G201300.1	100	188	0	337	2720	404	2787	0	3362

TRINITY_DN9498_c0_g1_i2	Glyma.02G251500.1	99.24242424	161	4	2	2382	11	2388	0	3452

TRINITY_DN16038_c0_g1_i1	Glyma.20G124000.1	100	161	6	457	2827	309	2691	0	3421

TRINITY_DN8905_c0_g1_i3	Glyma.17G259200.1	100	141	2	117	2494	13	2396	0	3583

TRINITY_DN16130_c2_g6_i7	Glyma.10G075300.2	100	139	0	337	2720	407	2790	0	3633

TRINITY_DN16130_c2_g6_i7	Glyma.02G201300.2	100	130	0	337	2720	404	2787	0	3683

TRINITY_DN16130_c2_g6_i7	Glyma.02G201300.4	97.04142012	130	0	337	2720	404	2787	0	3683

TRINITY_DN16130_c2_g6_i7	Glyma.02G201300.1	97.04142012	130	0	337	2720	404	2787	0	3683

TRINITY_DN16345_c0_g1_i9	Glyma.07G093700.1	99.23076923	129	1	439	2822	3729	1349	0	3668

TRINITY_DN16345_c0_g1_i9	Glyma.09G182600.1	99.2248062	124	2	439	2822	3717	1343	0	3657

TRINITY_DN13882_c0_g1_i1	Glyma.08G310800.1	98.50746269	450	43	26	2380	778	3111	0	1410

TRINITY_DN14801_c0_g2_i1	Glyma.04G247200.1	100	390	46	259	2598	2358	1	0	1801

TRINITY_DN14245_c0_g2_i3	Glyma.15G072000.3	95.65217391	345	13	393	2756	14	2389	0	2322

TRINITY_DN14556_c0_g1_i2	Glyma.20G219600.2	99.57446809	305	14	227	2605	1	2370	0	2601

TRINITY_DN14556_c0_g1_i2	Glyma.20G219600.1	99.57627119	305	14	227	2605	1	2370	0	2601

TRINITY_DN14245_c0_g2_i2	Glyma.13G181700.3	95.65217391	180	5	2	2381	326	2702	0	3345

TRINITY_DN3323_c0_g1_i1	Glyma.02G181500.1	99.43661972	165	0	502	2884	1	2383	0	3487

TRINITY_DN15539_c0_g1_i4	Glyma.11G079200.1	97.5	118	0	140	2522	1	2383	0	3747

TRINITY_DN4194_c0_g1_i1	Glyma.07G092400.1	97.25400458	389	29	265	2612	86	2442	0	1862

TRINITY_DN4194_c0_g2_i1	Glyma.07G092400.1	100	389	29	265	2612	86	2442	0	1862

TRINITY_DN15851_c0_g1_i1	Glyma.09G157300.3	97.14285714	347	39	227	2582	2414	68	0	2082

TRINITY_DN15851_c0_g1_i1	Glyma.09G157300.1	98.67549669	347	39	227	2582	2414	68	0	2082

TRINITY_DN15851_c0_g1_i1	Glyma.09G157300.2	98.67549669	347	39	227	2582	2414	68	0	2082

TRINITY_DN16345_c0_g1_i9	Glyma.05G237500.1	99.21259843	294	6	445	2822	3705	1328	0	2719

TRINITY_DN16259_c0_g1_i1	Glyma.07G117800.1	96.44268775	266	30	348	2708	1	2337	0	2499

TRINITY_DN16259_c0_g1_i1	Glyma.03G106500.1	96.2962963	261	38	348	2708	1	2328	0	2468

TRINITY_DN16082_c0_g1_i6	Glyma.07G016700.1	96.05425401	228	11	552	2913	2380	1	0	2994

TRINITY_DN16082_c0_g1_i2	Glyma.07G016700.1	99.03846154	206	11	581	2942	2380	1	0	3116

TRINITY_DN15440_c0_g1_i1	Glyma.01G068000.1	100	146	4	560	2932	2382	1	0	3531

TRINITY_DN9308_c0_g2_i1	Glyma.01G159900.1	96.12676056	144	2	599	2977	2382	1	0	3581

TRINITY_DN11023_c0_g1_i1	Glyma.04G018100.1	99.12280702	141	3	76	2451	1	2379	0	3559

TRINITY_DN16254_c0_g1_i4	Glyma.07G004300.3	99.57627119	527	63	995	3338	2363	23	0	979

TRINITY_DN16214_c0_g2_i3	Glyma.02G282700.1	99.42028986	414	44	1716	4054	3010	648	0	1716

TRINITY_DN16214_c0_g2_i4	Glyma.02G282700.1	97.81287971	414	44	1787	4125	3010	648	0	1716

TRINITY_DN16214_c0_g2_i3	Glyma.02G282700.2	99.27007299	414	44	1716	4054	2935	573	0	1716

TRINITY_DN16214_c0_g2_i4	Glyma.02G282700.2	100	414	44	1787	4125	2935	573	0	1716

TRINITY_DN13491_c0_g1_i1	Glyma.15G074000.5	100	165	8	1	2356	2182	4546	0	3217

TRINITY_DN13491_c0_g1_i1	Glyma.15G074000.6	100	165	8	1	2356	2182	4546	0	3217

TRINITY_DN13491_c0_g1_i2	Glyma.15G074000.5	98.80952381	165	8	1	2356	2182	4546	0	3217

TRINITY_DN13491_c0_g1_i2	Glyma.15G074000.6	98.7804878	165	8	1	2356	2182	4546	0	3217

TRINITY_DN13491_c0_g1_i3	Glyma.15G074000.5	98.60788863	165	8	1	2356	2182	4546	0	3217

TRINITY_DN13491_c0_g1_i3	Glyma.15G074000.6	96.51162791	165	8	1	2356	2182	4546	0	3217

TRINITY_DN13491_c0_g1_i4	Glyma.15G074000.5	100	165	8	1	2356	2182	4546	0	3217

TRINITY_DN13491_c0_g1_i4	Glyma.15G074000.6	100	165	8	1	2356	2182	4546	0	3217

TRINITY_DN13491_c0_g1_i5	Glyma.15G074000.5	95.08196721	165	8	1	2356	2182	4546	0	3217

TRINITY_DN13491_c0_g1_i5	Glyma.15G074000.6	100	165	8	1	2356	2182	4546	0	3217

TRINITY_DN13491_c0_g1_i6	Glyma.15G074000.5	100	165	8	1	2356	2182	4546	0	3217

TRINITY_DN13491_c0_g1_i6	Glyma.15G074000.6	96.15384615	165	8	1	2356	2182	4546	0	3217

TRINITY_DN8905_c0_g1_i3	Glyma.14G220300.1	100	145	1	117	2494	13	2393	0	3574

TRINITY_DN16412_c0_g2_i1	Glyma.07G068500.1	98.52941176	278	8	240	2617	110	2481	0	2791

TRINITY_DN16412_c0_g2_i4	Glyma.07G068500.1	100	278	8	270	2647	110	2481	0	2791

TRINITY_DN16377_c0_g1_i6	Glyma.11G137700.1	100	269	18	59	2423	1	2368	0	2730

TRINITY_DN13524_c0_g2_i2	Glyma.15G159000.3	97.08029197	240	3	255	2633	1	2373	0	3014

TRINITY_DN13524_c0_g2_i2	Glyma.15G159000.2	97.05882353	240	3	255	2633	1	2373	0	3014

TRINITY_DN13524_c0_g2_i2	Glyma.15G159000.1	97.08737864	240	3	255	2633	1	2373	0	3014

TRINITY_DN13524_c0_g2_i2	Glyma.09G052700.2	100	236	3	255	2633	1	2373	0	3037

TRINITY_DN13524_c0_g2_i2	Glyma.09G052700.1	97.09302326	236	3	255	2633	1	2373	0	3037

TRINITY_DN16004_c1_g1_i6	Glyma.15G004300.1	97.10144928	531	22	355	2723	118	2483	0	1303

TRINITY_DN5710_c0_g1_i1	Glyma.02G295400.1	97.14285714	219	6	645	3015	2445	72	0	3096

TRINITY_DN12362_c0_g1_i2	Glyma.14G055900.1	96.7032967	196	11	101	2465	2434	70	0	3127

TRINITY_DN12362_c0_g1_i3	Glyma.14G055900.1	95.23809524	196	11	101	2465	2434	70	0	3127

TRINITY_DN13524_c0_g2_i1	Glyma.15G159000.3	100	190	1	255	2633	1	2373	0	3302

TRINITY_DN13524_c0_g2_i1	Glyma.15G159000.2	100	190	1	255	2633	1	2373	0	3302

TRINITY_DN13524_c0_g2_i1	Glyma.15G159000.1	100	190	1	255	2633	1	2373	0	3302

TRINITY_DN13524_c0_g2_i1	Glyma.09G052700.2	100	189	1	255	2633	1	2373	0	3308

TRINITY_DN13524_c0_g2_i1	Glyma.09G052700.1	100	189	1	255	2633	1	2373	0	3308

TRINITY_DN15180_c1_g1_i4	Glyma.02G205500.1	95	179	10	194	2572	1	2355	0	3247

TRINITY_DN11023_c0_g1_i1	Glyma.06G018400.1	100	146	1	76	2451	1	2379	0	3565

TRINITY_DN18293_c0_g1_i1	Glyma.17G204000.1	100	112	2	342	2714	6489	4111	0	3735

TRINITY_DN18293_c0_g1_i1	Glyma.17G204000.2	100	112	2	342	2714	5808	3430	0	3735

TRINITY_DN18293_c0_g1_i1	Glyma.06G073700.1	100	349	41	344	2706	6451	4122	0	2050

TRINITY_DN16377_c0_g1_i5	Glyma.11G137700.1	100	269	23	59	2402	1	2368	0	2617

TRINITY_DN9308_c0_g2_i1	Glyma.11G084200.1	96.12736661	143	1	600	2977	2375	1	0	3579

TRINITY_DN15736_c0_g1_i1	Glyma.06G162300.1	98.96373057	297	24	404	2745	457	2825	0	2466

TRINITY_DN9365_c0_g1_i1	Glyma.15G100800.1	96.13003096	262	21	1	2348	2312	4671	0	2641

TRINITY_DN15979_c0_g3_i1	Glyma.06G152200.2	97.82608696	227	21	409	2755	2487	123	0	2861

TRINITY_DN15979_c0_g3_i1	Glyma.06G152200.1	100	227	21	409	2755	2487	123	0	2861

TRINITY_DN15979_c0_g3_i2	Glyma.06G152200.2	96.8	227	21	409	2755	2487	123	0	2861

TRINITY_DN15979_c0_g3_i2	Glyma.06G152200.1	97.22772277	227	21	409	2755	2487	123	0	2861

TRINITY_DN13209_c1_g1_i2	Glyma.08G171800.1	95.23809524	222	11	695	3067	2815	446	0	3088

TRINITY_DN11133_c0_g1_i1	Glyma.15G144100.1	98.07692308	204	20	111	2461	2805	452	0	2942

TRINITY_DN11133_c0_g1_i1	Glyma.15G144100.3	100	204	20	111	2461	2781	428	0	2942

TRINITY_DN11133_c0_g1_i1	Glyma.15G144100.2	99.06542056	204	20	111	2461	2643	290	0	2942

TRINITY_DN16082_c0_g1_i2	Glyma.07G016500.1	96.57534247	197	11	581	2942	2371	1	0	3162

TRINITY_DN16449_c0_g1_i1	Glyma.01G091800.1	100	158	8	288	2655	1	2374	0	3437

TRINITY_DN14556_c0_g1_i2	Glyma.18G240600.1	97.18309859	157	1	227	2603	1	2371	0	3482

TRINITY_DN10529_c0_g2_i1	Glyma.15G047100.1	99.71346705	156	1	1	2377	81	2454	0	3506

TRINITY_DN11893_c0_g1_i1	Glyma.10G075900.2	98	155	3	104	2474	1	2377	0	3493

TRINITY_DN15659_c0_g2_i4	Glyma.07G184800.1	100	146	7	115	2482	2	2375	0	3504

TRINITY_DN15659_c0_g2_i4	Glyma.07G184800.3	95.08716323	146	7	115	2482	2	2375	0	3504

TRINITY_DN15659_c0_g2_i7	Glyma.07G184800.1	99.08256881	146	7	192	2559	2	2375	0	3504

TRINITY_DN15659_c0_g2_i7	Glyma.07G184800.3	100	146	7	192	2559	2	2375	0	3504

TRINITY_DN10529_c0_g2_i1	Glyma.08G185700.1	99.72752044	145	0	1	2377	87	2463	0	3587

TRINITY_DN16254_c0_g1_i4	Glyma.08G222200.1	96.95945946	534	53	995	3338	2786	443	0	1016

TRINITY_DN14750_c0_g1_i2	Glyma.08G067100.1	95.23809524	315	13	563	2925	2384	22	0	2475

TRINITY_DN9365_c0_g1_i1	Glyma.13G211900.1	96.60056657	284	21	1	2348	2171	4530	0	2531

TRINITY_DN16259_c0_g1_i1	Glyma.07G117800.2	100	263	35	348	2708	1	2319	0	2466

TRINITY_DN16082_c0_g1_i6	Glyma.07G016500.1	95.4	225	9	552	2913	2371	1	0	3018

TRINITY_DN690_c0_g1_i1	Glyma.12G115800.1	100	160	2	188	2554	2606	231	0	3443

TRINITY_DN13976_c0_g1_i1	Glyma.04G221600.7	95.41984733	135	1	268	2643	2373	1	0	3620

TRINITY_DN13976_c0_g1_i1	Glyma.04G221600.1	95.41984733	135	1	268	2643	2373	1	0	3620

TRINITY_DN13976_c0_g1_i1	Glyma.04G221600.6	95.41984733	135	1	268	2643	2373	1	0	3620

TRINITY_DN13976_c0_g1_i1	Glyma.04G221600.3	98.17073171	135	1	268	2643	2373	1	0	3620

TRINITY_DN13976_c0_g1_i1	Glyma.04G221600.4	98.17073171	135	1	268	2643	2373	1	0	3620

TRINITY_DN13976_c0_g1_i1	Glyma.04G221600.5	98.17073171	135	1	268	2643	2373	1	0	3620

TRINITY_DN13976_c0_g1_i1	Glyma.04G221600.2	98.17232376	135	1	268	2643	2373	1	0	3620

TRINITY_DN13976_c0_g1_i1	Glyma.04G221600.8	95.23809524	135	1	268	2643	2373	1	0	3620

TRINITY_DN13976_c0_g1_i2	Glyma.04G221600.7	100	135	1	268	2643	2373	1	0	3620

TRINITY_DN13976_c0_g1_i2	Glyma.04G221600.1	100	135	1	268	2643	2373	1	0	3620

TRINITY_DN13976_c0_g1_i2	Glyma.04G221600.6	100	135	1	268	2643	2373	1	0	3620

TRINITY_DN13976_c0_g1_i2	Glyma.04G221600.3	97.19626168	135	1	268	2643	2373	1	0	3620

TRINITY_DN13976_c0_g1_i2	Glyma.04G221600.4	99.68944099	135	1	268	2643	2373	1	0	3620

TRINITY_DN13976_c0_g1_i2	Glyma.04G221600.5	99.18032787	135	1	268	2643	2373	1	0	3620

TRINITY_DN13976_c0_g1_i2	Glyma.04G221600.2	99.18032787	135	1	268	2643	2373	1	0	3620

TRINITY_DN13976_c0_g1_i2	Glyma.04G221600.8	99.18032787	135	1	268	2643	2373	1	0	3620

TRINITY_DN13976_c0_g1_i1	Glyma.06G143800.8	100	133	1	268	2643	2373	1	0	3631

TRINITY_DN13976_c0_g1_i1	Glyma.06G143800.2	100	133	1	268	2643	2373	1	0	3631

TRINITY_DN13976_c0_g1_i1	Glyma.06G143800.3	100	133	1	268	2643	2373	1	0	3631

TRINITY_DN13976_c0_g1_i1	Glyma.06G143800.4	96.64902998	133	1	268	2643	2373	1	0	3631

TRINITY_DN13976_c0_g1_i1	Glyma.06G143800.7	98.32402235	133	1	268	2643	2373	1	0	3631

TRINITY_DN13976_c0_g1_i1	Glyma.06G143800.10	98.53658537	133	1	268	2643	2373	1	0	3631

TRINITY_DN13976_c0_g1_i1	Glyma.06G143800.1	95.27559055	133	1	268	2643	2373	1	0	3631

TRINITY_DN13976_c0_g1_i1	Glyma.06G143800.9	95.41984733	133	1	268	2643	2373	1	0	3631

TRINITY_DN13976_c0_g1_i1	Glyma.06G143800.5	95.41984733	133	1	268	2643	2373	1	0	3631

TRINITY_DN13976_c0_g1_i1	Glyma.06G143800.6	95.41984733	133	1	268	2643	2373	1	0	3631

TRINITY_DN13976_c0_g1_i2	Glyma.06G143800.8	95.71865443	133	1	268	2643	2373	1	0	3631

TRINITY_DN13976_c0_g1_i2	Glyma.06G143800.2	100	133	1	268	2643	2373	1	0	3631

TRINITY_DN13976_c0_g1_i2	Glyma.06G143800.3	100	133	1	268	2643	2373	1	0	3631

TRINITY_DN13976_c0_g1_i2	Glyma.06G143800.4	97	133	1	268	2643	2373	1	0	3631

TRINITY_DN13976_c0_g1_i2	Glyma.06G143800.7	98.61111111	133	1	268	2643	2373	1	0	3631

TRINITY_DN13976_c0_g1_i2	Glyma.06G143800.10	98.30866808	133	1	268	2643	2373	1	0	3631

TRINITY_DN13976_c0_g1_i2	Glyma.06G143800.1	98.92473118	133	1	268	2643	2373	1	0	3631

TRINITY_DN13976_c0_g1_i2	Glyma.06G143800.9	95.52238806	133	1	268	2643	2373	1	0	3631

TRINITY_DN13976_c0_g1_i2	Glyma.06G143800.5	95.52238806	133	1	268	2643	2373	1	0	3631

TRINITY_DN13976_c0_g1_i2	Glyma.06G143800.6	100	133	1	268	2643	2373	1	0	3631

TRINITY_DN16106_c0_g3_i3	Glyma.14G128300.1	99.09090909	264	14	299	2663	2682	318	0	2795

TRINITY_DN16106_c0_g3_i3	Glyma.14G128300.3	99.1202346	264	14	299	2663	2652	288	0	2795

TRINITY_DN16106_c0_g3_i3	Glyma.14G128300.2	97.24137931	264	14	299	2663	2598	234	0	2795

TRINITY_DN16377_c0_g1_i3	Glyma.12G061200.1	95.76923077	245	14	59	2423	1	2368	0	2918

TRINITY_DN16377_c0_g1_i6	Glyma.12G061200.1	100	245	14	59	2423	1	2368	0	2918

TRINITY_DN16377_c0_g1_i1	Glyma.12G061200.1	95.77167019	239	20	59	2402	1	2368	0	2817

TRINITY_DN16377_c0_g1_i5	Glyma.12G061200.1	100	239	20	59	2402	1	2368	0	2817

TRINITY_DN16130_c2_g6_i10	Glyma.19G196000.1	98.80952381	218	1	337	2711	545	2916	0	3158

TRINITY_DN16130_c2_g6_i10	Glyma.19G196000.3	100	218	1	337	2711	383	2754	0	3158

TRINITY_DN16130_c2_g6_i10	Glyma.19G196000.4	98.80952381	218	1	337	2711	383	2754	0	3158

TRINITY_DN9537_c0_g1_i1	Glyma.06G173800.1	95.07772021	188	11	57	2427	413	2771	0	3216

TRINITY_DN13428_c0_g2_i1	Glyma.20G051100.1	99.05660377	414	51	321	2657	2337	1	0	1613

TRINITY_DN15751_c0_g1_i14	Glyma.04G193800.1	95.85062241	345	27	890	3238	1	2352	0	2169

TRINITY_DN15751_c0_g1_i4	Glyma.04G193800.1	100	345	27	890	3238	1	2352	0	2169

TRINITY_DN13448_c0_g1_i6	Glyma.18G011000.1	98.11320755	323	2	17	2389	43	2415	0	2582

TRINITY_DN16377_c0_g1_i3	Glyma.12G061400.1	95.76547231	278	19	59	2423	1	2356	0	2669

TRINITY_DN16377_c0_g1_i6	Glyma.12G061400.1	100	278	19	59	2423	1	2356	0	2669

TRINITY_DN16377_c0_g1_i1	Glyma.12G061400.1	95.77464789	273	23	59	2402	1	2356	0	2562

TRINITY_DN16377_c0_g1_i5	Glyma.12G061400.1	100	273	23	59	2402	1	2356	0	2562

TRINITY_DN14670_c0_g1_i2	Glyma.13G065500.9	97.72727273	218	10	993	3360	1	2362	0	3061

TRINITY_DN36949_c0_g1_i1	Glyma.04G121100.4	100	194	4	1	2359	60	2433	0	3212

TRINITY_DN36949_c0_g1_i1	Glyma.04G121100.1	97.61904762	194	4	1	2359	60	2433	0	3212

TRINITY_DN16082_c0_g1_i2	Glyma.08G201900.1	99.13793103	194	8	581	2942	2371	1	0	3212

TRINITY_DN36949_c0_g1_i1	Glyma.06G317700.4	98.14814815	192	19	1	2359	27	2376	0	3068

TRINITY_DN36949_c0_g1_i1	Glyma.06G317700.7	95.12953368	190	6	1	2359	27	2400	0	3234

TRINITY_DN36949_c0_g1_i1	Glyma.06G317700.1	100	190	6	1	2359	27	2400	0	3234

TRINITY_DN36949_c0_g1_i1	Glyma.06G317700.3	96.09375	190	6	1	2359	27	2400	0	3234

TRINITY_DN13428_c0_g2_i1	Glyma.14G041800.1	99.6875	185	14	297	2657	2352	1	0	3133

TRINITY_DN13428_c0_g2_i1	Glyma.14G041800.2	99.05660377	185	14	297	2657	2352	1	0	3133

TRINITY_DN13428_c0_g2_i1	Glyma.02G274300.1	99.07407407	183	11	297	2657	2352	1	0	3144

TRINITY_DN13428_c0_g2_i1	Glyma.02G274300.5	99.07407407	183	11	297	2657	2352	1	0	3144

TRINITY_DN13428_c0_g2_i1	Glyma.02G274300.4	97.84313725	183	11	297	2657	2352	1	0	3144

TRINITY_DN13428_c0_g2_i1	Glyma.02G274300.2	96.4556962	183	11	297	2657	2352	1	0	3144

TRINITY_DN13428_c0_g2_i1	Glyma.02G274300.3	98.20359281	183	11	297	2657	2352	1	0	3144

TRINITY_DN15163_c0_g1_i1	Glyma.07G159300.2	95.45454545	183	23	722	3070	628	2979	0	3066

TRINITY_DN21870_c0_g1_i1	Glyma.11G088700.1	95.74468085	176	3	217	2583	2373	1	0	3358

TRINITY_DN11385_c0_g1_i2	Glyma.20G076300.1	97.14285714	147	8	155	2516	2356	1	0	3376

TRINITY_DN16449_c0_g1_i1	Glyma.02G130200.1	98.64864865	143	4	288	2655	1	2368	0	3515

TRINITY_DN16449_c0_g1_i1	Glyma.02G130200.3	100	143	5	288	2655	1	2359	0	3456

TRINITY_DN11385_c0_g1_i2	Glyma.10G124200.1	96.92307692	132	10	155	2516	2368	1	0	3537

TRINITY_DN13448_c0_g1_i6	Glyma.11G246200.9	97.95918367	333	0	17	2389	43	2415	0	2538

TRINITY_DN13448_c0_g1_i6	Glyma.11G246200.1	100	333	0	17	2389	43	2415	0	2538

TRINITY_DN15655_c2_g1_i3	Glyma.02G239600.5	95.23809524	275	15	130	2486	1	2366	0	2710

TRINITY_DN15751_c0_g1_i3	Glyma.04G193800.1	96.66666667	256	23	890	3241	1	2352	0	2693

TRINITY_DN15751_c0_g1_i7	Glyma.04G193800.1	99.12280702	256	23	890	3241	1	2352	0	2693

TRINITY_DN16082_c0_g1_i6	Glyma.08G201900.1	98.7804878	221	6	552	2913	2371	1	0	3073

TRINITY_DN16130_c2_g6_i10	Glyma.03G198100.1	97.44245524	217	0	337	2709	386	2758	0	3181

TRINITY_DN16130_c2_g6_i10	Glyma.03G198100.2	96.77419355	217	0	337	2709	386	2758	0	3181

TRINITY_DN15736_c0_g1_i1	Glyma.04G203200.1	98.96373057	303	24	403	2745	426	2786	0	2444

TRINITY_DN16352_c0_g2_i1	Glyma.07G011600.1	100	270	6	2521	4874	2386	15	0	2769

TRINITY_DN14750_c0_g1_i1	Glyma.08G067100.1	95.21367521	237	11	563	2922	2384	22	0	2931

TRINITY_DN13436_c0_g1_i1	Glyma.08G044600.1	98.91891892	232	16	816	3167	2372	3	0	2953

TRINITY_DN6110_c0_g1_i1	Glyma.01G014600.5	98.7654321	192	27	681	3018	2362	1	0	3033

TRINITY_DN6110_c0_g1_i1	Glyma.01G014600.4	98.7654321	192	27	681	3018	2362	1	0	3033

TRINITY_DN6110_c0_g1_i1	Glyma.01G014600.3	100	192	27	681	3018	2362	1	0	3033

TRINITY_DN6110_c0_g1_i1	Glyma.01G014600.2	99.09365559	192	27	681	3018	2362	1	0	3033

TRINITY_DN6110_c0_g1_i1	Glyma.01G014600.1	99.09502262	192	27	681	3018	2362	1	0	3033

TRINITY_DN12719_c0_g2_i5	Glyma.12G179300.5	97.54601227	173	13	1	2361	2358	1	0	3260

TRINITY_DN12719_c0_g2_i5	Glyma.12G179300.4	97.54689755	173	13	1	2361	2358	1	0	3260

TRINITY_DN12719_c0_g2_i5	Glyma.12G179300.3	100	173	13	1	2361	2358	1	0	3260

TRINITY_DN12719_c0_g2_i5	Glyma.12G179300.1	99.02912621	173	13	1	2361	2358	1	0	3260

TRINITY_DN12719_c0_g2_i7	Glyma.12G179300.5	99.58847737	173	13	1	2361	2358	1	0	3260

TRINITY_DN12719_c0_g2_i7	Glyma.12G179300.4	100	173	13	1	2361	2358	1	0	3260

TRINITY_DN12719_c0_g2_i7	Glyma.12G179300.3	97.22222222	173	13	1	2361	2358	1	0	3260

TRINITY_DN12719_c0_g2_i7	Glyma.12G179300.1	100	173	13	1	2361	2358	1	0	3260

TRINITY_DN12719_c0_g2_i5	Glyma.13G321300.34	97.14285714	167	20	1	2361	2322	1	0	3061

TRINITY_DN12719_c0_g2_i5	Glyma.13G321300.35	97.14285714	167	20	1	2361	2322	1	0	3061

TRINITY_DN12719_c0_g2_i5	Glyma.13G321300.22	98.7012987	167	20	1	2361	2322	1	0	3061

TRINITY_DN12719_c0_g2_i5	Glyma.13G321300.23	97.18309859	167	20	1	2361	2322	1	0	3061

TRINITY_DN12719_c0_g2_i5	Glyma.13G321300.2	97.18309859	167	20	1	2361	2322	1	0	3061

TRINITY_DN12719_c0_g2_i5	Glyma.13G321300.3	95.27559055	167	20	1	2361	2322	1	0	3061

TRINITY_DN12719_c0_g2_i7	Glyma.13G321300.34	97.82608696	167	20	1	2361	2322	1	0	3061

TRINITY_DN12719_c0_g2_i7	Glyma.13G321300.35	96.38554217	167	20	1	2361	2322	1	0	3061

TRINITY_DN12719_c0_g2_i7	Glyma.13G321300.22	96.2962963	167	20	1	2361	2322	1	0	3061

TRINITY_DN12719_c0_g2_i7	Glyma.13G321300.23	100	167	20	1	2361	2322	1	0	3061

TRINITY_DN12719_c0_g2_i7	Glyma.13G321300.2	98.00995025	167	20	1	2361	2322	1	0	3061

TRINITY_DN12719_c0_g2_i7	Glyma.13G321300.3	98.00995025	167	20	1	2361	2322	1	0	3061

TRINITY_DN16216_c0_g3_i1	Glyma.01G229700.1	100	143	6	499	2858	135	2497	0	3452

TRINITY_DN16216_c0_g3_i7	Glyma.01G229700.1	98.77300613	143	6	160	2519	135	2497	0	3452

TRINITY_DN11262_c0_g1_i1	Glyma.04G056400.2	97.6635514	216	10	651	2998	4383	2014	0	3027

TRINITY_DN15345_c0_g1_i3	Glyma.12G194200.2	100	190	1	388	2758	17	2386	0	3319

TRINITY_DN15345_c0_g1_i3	Glyma.12G194200.1	100	190	1	388	2758	17	2386	0	3319

TRINITY_DN15345_c0_g1_i4	Glyma.12G194200.2	97.05882353	190	1	194	2564	17	2386	0	3319

TRINITY_DN15345_c0_g1_i4	Glyma.12G194200.1	97.05882353	190	1	194	2564	17	2386	0	3319

TRINITY_DN12892_c0_g4_i1	Glyma.11G103100.7	100	154	2	305	2672	139	2503	0	3467

TRINITY_DN12892_c0_g4_i1	Glyma.11G103100.8	100	154	2	305	2672	139	2503	0	3467

TRINITY_DN12892_c0_g4_i5	Glyma.11G103100.7	100	154	2	305	2672	139	2503	0	3467

TRINITY_DN12892_c0_g4_i5	Glyma.11G103100.8	100	154	2	305	2672	139	2503	0	3467

TRINITY_DN14521_c1_g1_i2	Glyma.16G148800.1	98.05825243	404	24	665	3019	2358	1	0	1964

TRINITY_DN16082_c0_g1_i4	Glyma.06G086600.1	96.19565217	395	33	552	2879	2392	41	0	1801

TRINITY_DN15235_c0_g1_i3	Glyma.20G165000.1	98.82352941	281	10	310	2672	1	2366	0	2748

TRINITY_DN15235_c0_g1_i3	Glyma.20G165000.2	98.82352941	281	10	310	2672	1	2366	0	2748

TRINITY_DN3202_c0_g1_i1	Glyma.05G098700.1	97.93814433	234	4	48	2412	2919	552	0	3035

TRINITY_DN16082_c0_g1_i6	Glyma.08G202000.1	95.37572254	232	10	552	2889	2407	40	0	2872

TRINITY_DN12679_c0_g1_i1	Glyma.18G004000.2	95.29411765	215	2	1371	3739	470	2838	0	3173

TRINITY_DN12679_c0_g1_i2	Glyma.18G004000.2	95.30791789	215	2	1371	3739	470	2838	0	3173

TRINITY_DN13425_c0_g1_i2	Glyma.11G130200.1	97.68637532	209	11	246	2606	358	2721	0	3121

TRINITY_DN12967_c0_g3_i1	Glyma.08G056100.1	99.32885906	191	8	230	2578	2370	10	0	3125

TRINITY_DN11262_c0_g1_i3	Glyma.04G056400.2	100	173	8	651	2995	4383	2014	0	3256

TRINITY_DN16031_c1_g2_i1	Glyma.07G235800.2	100	171	0	1008	3377	2370	1	0	3430

TRINITY_DN16031_c1_g2_i4	Glyma.07G235800.2	95.59286464	163	0	292	2661	2370	1	0	3474

TRINITY_DN15944_c2_g2_i2	Glyma.06G109500.1	95.65217391	128	4	275	2629	2370	1	0	3570

TRINITY_DN15944_c2_g2_i2	Glyma.06G109500.2	100	128	5	275	2629	2367	1	0	3552

TRINITY_DN13433_c0_g1_i1	Glyma.03G151400.1	95.07042254	444	53	3	2323	3371	1048	0	1314

TRINITY_DN13433_c0_g1_i1	Glyma.03G151400.2	95.07042254	444	53	3	2323	3371	1048	0	1314

TRINITY_DN16082_c0_g1_i14	Glyma.06G086600.1	95.43197617	404	30	581	2908	2392	41	0	1762

TRINITY_DN16082_c0_g1_i4	Glyma.04G085000.1	96.19565217	385	34	552	2879	2389	41	0	1847

TRINITY_DN15235_c0_g1_i7	Glyma.20G165000.1	100	374	9	219	2581	1	2366	0	2244

TRINITY_DN15235_c0_g1_i7	Glyma.20G165000.2	100	374	9	219	2581	1	2366	0	2244

TRINITY_DN13622_c0_g1_i1	Glyma.16G182200.7	95.45454545	184	2	401	2768	2841	474	0	3343

TRINITY_DN13622_c0_g1_i1	Glyma.16G182200.6	100	184	2	401	2768	2826	459	0	3343

TRINITY_DN15345_c0_g1_i3	Glyma.13G308400.5	96.82539683	180	1	388	2756	17	2384	0	3371

TRINITY_DN15345_c0_g1_i3	Glyma.13G308400.2	100	180	1	388	2756	17	2384	0	3371

TRINITY_DN15345_c0_g1_i3	Glyma.13G308400.1	100	180	1	388	2756	17	2384	0	3371

TRINITY_DN15345_c0_g1_i4	Glyma.13G308400.5	98.36065574	180	1	194	2562	17	2384	0	3371

TRINITY_DN15345_c0_g1_i4	Glyma.13G308400.2	98.95833333	180	1	194	2562	17	2384	0	3371

TRINITY_DN15345_c0_g1_i4	Glyma.13G308400.1	100	180	1	194	2562	17	2384	0	3371

TRINITY_DN14622_c0_g1_i2	Glyma.20G210700.1	98.57142857	163	18	102	2460	1	2347	0	3265

TRINITY_DN14622_c0_g1_i2	Glyma.20G210700.2	99.4011976	163	18	102	2460	1	2347	0	3265

TRINITY_DN9122_c0_g1_i2	Glyma.02G248300.2	97.75280899	157	7	10	2374	4	2360	0	3402

TRINITY_DN9122_c0_g1_i2	Glyma.02G248300.1	100	157	7	10	2374	4	2360	0	3402

TRINITY_DN9122_c0_g1_i2	Glyma.14G068200.1	95.4954955	146	5	10	2374	4	2360	0	3463

TRINITY_DN16141_c0_g1_i1	Glyma.04G003000.7	95.91836735	93	1	691	3056	3069	701	0	3840

TRINITY_DN16141_c0_g1_i2	Glyma.04G003000.7	98.66666667	93	1	691	3056	3069	701	0	3840

TRINITY_DN16141_c0_g1_i3	Glyma.04G003000.7	99.48186528	93	1	691	3056	3069	701	0	3840

TRINITY_DN16141_c0_g1_i5	Glyma.04G003000.7	96.12403101	93	1	691	3056	3069	701	0	3840

TRINITY_DN16141_c0_g1_i1	Glyma.04G003000.5	100	93	1	691	3056	3060	692	0	3840

TRINITY_DN16141_c0_g1_i2	Glyma.04G003000.5	95.34883721	93	1	691	3056	3060	692	0	3840

TRINITY_DN16141_c0_g1_i3	Glyma.04G003000.5	95.86330935	93	1	691	3056	3060	692	0	3840

TRINITY_DN16141_c0_g1_i5	Glyma.04G003000.5	98.68421053	93	1	691	3056	3060	692	0	3840

TRINITY_DN18293_c0_g1_i1	Glyma.04G072400.1	100	365	19	344	2706	6457	4128	0	2073

TRINITY_DN18293_c0_g1_i1	Glyma.04G072400.2	100	365	19	344	2706	6358	4029	0	2073

TRINITY_DN12362_c0_g1_i1	Glyma.18G023500.1	99.11504425	346	18	163	2504	2533	171	0	2255

TRINITY_DN12362_c0_g1_i4	Glyma.18G023500.1	100	346	18	163	2504	2533	171	0	2255

TRINITY_DN15751_c0_g1_i10	Glyma.04G193800.1	98.30508475	320	24	890	3232	1	2352	0	2335

TRINITY_DN16082_c0_g1_i4	Glyma.08G202000.1	95.1048951	281	14	552	2875	2407	42	0	2519

TRINITY_DN16332_c0_g3_i2	Glyma.06G042400.2	100	247	1	1968	4335	2365	1	0	2985

TRINITY_DN16332_c0_g3_i2	Glyma.04G041300.2	100	244	1	1968	4335	2365	1	0	3001

TRINITY_DN16082_c0_g1_i2	Glyma.08G202000.1	100	215	7	581	2918	2407	40	0	2988

TRINITY_DN13425_c0_g1_i2	Glyma.12G054700.1	100	194	6	246	2606	382	2745	0	3227

TRINITY_DN14622_c0_g1_i2	Glyma.10G179700.5	97.29344729	186	16	102	2460	1	2347	0	3149

TRINITY_DN14622_c0_g1_i2	Glyma.10G179700.4	95.72864322	186	16	102	2460	1	2347	0	3149

TRINITY_DN15163_c0_g1_i1	Glyma.20G006400.2	97.6744186	175	21	722	3070	601	2949	0	3158

TRINITY_DN2227_c0_g1_i1	Glyma.06G276100.2	98.7012987	168	7	240	2607	1	2353	0	3345

TRINITY_DN16332_c0_g3_i2	Glyma.14G085200.2	100	130	0	1968	4335	2368	1	0	3653

TRINITY_DN13433_c0_g1_i1	Glyma.19G153900.1	95.12195122	430	48	3	2323	3371	1051	0	1395

TRINITY_DN13433_c0_g1_i1	Glyma.19G153900.4	100	430	48	3	2323	2996	676	0	1395

TRINITY_DN16082_c0_g1_i14	Glyma.04G085000.1	97.60479042	395	30	581	2908	2389	41	0	1814

TRINITY_DN14750_c0_g1_i2	Glyma.07G182900.2	95.20295203	297	11	563	2925	2375	22	0	2615

TRINITY_DN14750_c0_g1_i2	Glyma.07G182900.1	95.27896996	297	11	563	2925	2375	22	0	2615

TRINITY_DN13890_c0_g1_i2	Glyma.08G170000.2	98.91304348	266	0	459	2825	1	2367	0	2898

TRINITY_DN9350_c0_g1_i1	Glyma.10G019200.7	96.92307692	247	9	154	2517	1	2355	0	2905

TRINITY_DN9350_c0_g1_i1	Glyma.10G019200.6	96.92307692	247	9	154	2517	1	2355	0	2905

TRINITY_DN15751_c0_g1_i8	Glyma.04G193800.1	97.10982659	231	20	890	3235	1	2352	0	2859

TRINITY_DN15944_c2_g2_i5	Glyma.13G083300.1	100	196	19	196	2526	2364	1	0	3033

TRINITY_DN36295_c0_g1_i1	Glyma.09G157900.1	97.65100671	194	8	73	2436	19	2370	0	3181

TRINITY_DN36295_c0_g1_i1	Glyma.09G157900.3	100	194	8	73	2436	19	2370	0	3181

TRINITY_DN13890_c0_g1_i1	Glyma.08G170000.2	96.86346863	176	0	286	2652	1	2367	0	3397

TRINITY_DN15659_c0_g2_i4	Glyma.08G064600.1	96.6442953	172	5	125	2484	12	2374	0	3347

TRINITY_DN15659_c0_g2_i7	Glyma.08G064600.1	96.89922481	172	5	202	2561	12	2374	0	3347

TRINITY_DN13775_c0_g1_i1	Glyma.19G170300.3	97.08029197	160	8	206	2539	10	2367	0	3214

TRINITY_DN13775_c0_g1_i2	Glyma.19G170300.3	97.56097561	160	8	206	2539	10	2367	0	3214

TRINITY_DN16130_c2_g6_i10	Glyma.10G075300.2	97.44136461	159	5	337	2698	407	2771	0	3445

TRINITY_DN16130_c2_g6_i10	Glyma.02G201300.2	100	151	4	337	2698	404	2768	0	3489

TRINITY_DN16130_c2_g6_i10	Glyma.02G201300.4	97.91666667	151	4	337	2698	404	2768	0	3489

TRINITY_DN16130_c2_g6_i10	Glyma.02G201300.1	97.91666667	151	4	337	2698	404	2768	0	3489

TRINITY_DN9378_c0_g1_i1	Glyma.09G191400.2	96.62921348	139	3	327	2687	2367	1	0	3563

TRINITY_DN9378_c0_g1_i1	Glyma.07G085500.2	97.62611276	137	2	327	2687	2367	1	0	3574

TRINITY_DN11155_c0_g1_i1	Glyma.15G093600.1	100	94	5	377	2737	2367	1	0	3812

TRINITY_DN13116_c0_g1_i2	Glyma.17G050100.6	97.84946237	354	25	123	2477	2337	1	0	2150

TRINITY_DN12362_c0_g1_i2	Glyma.11G233500.1	97.39130435	352	19	78	2422	2541	179	0	2265

TRINITY_DN12362_c0_g1_i3	Glyma.11G233500.1	96.49923896	352	19	78	2422	2541	179	0	2265

TRINITY_DN13524_c0_g2_i1	Glyma.05G131800.2	100	326	12	272	2633	21	2376	0	2473

TRINITY_DN13524_c0_g2_i1	Glyma.05G131800.1	100	326	12	272	2633	21	2376	0	2473

TRINITY_DN13524_c0_g2_i1	Glyma.05G131800.3	100	326	12	272	2633	21	2376	0	2473

TRINITY_DN13524_c0_g2_i1	Glyma.05G131800.4	100	326	12	272	2633	21	2376	0	2473

TRINITY_DN13524_c0_g2_i1	Glyma.05G131800.5	100	326	12	272	2633	21	2376	0	2473

TRINITY_DN16082_c0_g1_i14	Glyma.08G202000.1	100	264	11	581	2904	2407	42	0	2636

TRINITY_DN15944_c2_g2_i3	Glyma.13G083300.1	96.69811321	236	22	287	2614	2361	1	0	2784

TRINITY_DN3202_c0_g1_i1	Glyma.17G166900.1	97.93814433	224	5	48	2410	2964	608	0	3051

TRINITY_DN16134_c0_g3_i1	Glyma.08G354500.1	95.94594595	172	18	109	2433	2343	1	0	3003

TRINITY_DN16134_c0_g3_i1	Glyma.08G354500.3	98.7654321	172	18	109	2433	2343	1	0	3003

TRINITY_DN15655_c2_g1_i2	Glyma.02G239600.5	98.28767123	138	5	130	2483	1	2366	0	3528

TRINITY_DN16141_c0_g1_i1	Glyma.04G003000.6	99.13294798	93	0	691	3056	3066	701	0	3855

TRINITY_DN16141_c0_g1_i2	Glyma.04G003000.6	99.18699187	93	0	691	3056	3066	701	0	3855

TRINITY_DN16141_c0_g1_i3	Glyma.04G003000.6	99.02439024	93	0	691	3056	3066	701	0	3855

TRINITY_DN16141_c0_g1_i5	Glyma.04G003000.6	99.06542056	93	0	691	3056	3066	701	0	3855

TRINITY_DN16141_c0_g1_i1	Glyma.04G003000.4	100	93	0	691	3056	3057	692	0	3855

TRINITY_DN16141_c0_g1_i2	Glyma.04G003000.4	96.17486339	93	0	691	3056	3057	692	0	3855

TRINITY_DN16141_c0_g1_i3	Glyma.04G003000.4	99.02912621	93	0	691	3056	3057	692	0	3855

TRINITY_DN16141_c0_g1_i5	Glyma.04G003000.4	100	93	0	691	3056	3057	692	0	3855

TRINITY_DN16190_c0_g3_i7	Glyma.13G290700.1	96.19047619	423	23	900	3257	596	2937	0	1831

TRINITY_DN15345_c0_g3_i1	Glyma.12G194200.3	96.66666667	405	35	679	3020	44	2388	0	1845

TRINITY_DN13524_c0_g2_i1	Glyma.08G086300.4	100	335	10	272	2633	21	2376	0	2435

TRINITY_DN13524_c0_g2_i1	Glyma.08G086300.2	100	335	10	272	2633	21	2376	0	2435

TRINITY_DN13524_c0_g2_i1	Glyma.08G086300.3	100	332	13	272	2633	21	2367	0	2392

TRINITY_DN14654_c0_g1_i7	Glyma.11G089400.2	96.76258993	271	14	1786	4147	2344	1	0	2712

TRINITY_DN14654_c0_g1_i7	Glyma.11G089400.1	98.19277108	271	14	1786	4147	2344	1	0	2712

TRINITY_DN14654_c0_g1_i7	Glyma.11G089400.3	96.46302251	271	14	1786	4147	2344	1	0	2712

TRINITY_DN13524_c0_g2_i2	Glyma.05G131800.2	97.07446809	265	10	272	2633	21	2376	0	2822

TRINITY_DN13524_c0_g2_i2	Glyma.05G131800.1	97.07865169	265	10	272	2633	21	2376	0	2822

TRINITY_DN13524_c0_g2_i2	Glyma.05G131800.3	97.06601467	265	10	272	2633	21	2376	0	2822

TRINITY_DN13524_c0_g2_i2	Glyma.05G131800.4	97.05882353	265	10	272	2633	21	2376	0	2822

TRINITY_DN13524_c0_g2_i2	Glyma.05G131800.5	97.05882353	265	10	272	2633	21	2376	0	2822

TRINITY_DN16130_c2_g6_i7	Glyma.19G196000.1	100	244	2	337	2701	545	2903	0	2977

TRINITY_DN16130_c2_g6_i7	Glyma.19G196000.3	98.88888889	244	2	337	2701	383	2741	0	2977

TRINITY_DN16130_c2_g6_i7	Glyma.19G196000.4	98.88888889	244	2	337	2701	383	2741	0	2977

TRINITY_DN16130_c2_g6_i7	Glyma.03G198100.1	98.88888889	242	1	337	2701	386	2747	0	3007

TRINITY_DN16130_c2_g6_i7	Glyma.03G198100.2	98.88888889	242	1	337	2701	386	2747	0	3007

TRINITY_DN14151_c0_g1_i1	Glyma.16G030600.1	100	209	9	199	2545	1	2362	0	3073

TRINITY_DN16130_c2_g6_i6	Glyma.19G196000.1	98.48484848	191	2	337	2701	545	2903	0	3271

TRINITY_DN16130_c2_g6_i6	Glyma.19G196000.3	100	191	2	337	2701	383	2741	0	3271

TRINITY_DN16130_c2_g6_i6	Glyma.19G196000.4	95.24281467	191	2	337	2701	383	2741	0	3271

TRINITY_DN16130_c2_g6_i6	Glyma.03G198100.1	95.215311	188	1	337	2701	386	2747	0	3306

TRINITY_DN16130_c2_g6_i6	Glyma.03G198100.2	99.10714286	188	1	337	2701	386	2747	0	3306

TRINITY_DN13116_c0_g1_i2	Glyma.15G176600.1	97.86324786	184	6	128	2477	2365	1	0	3251

TRINITY_DN15851_c0_g1_i1	Glyma.10G175900.2	95.10050251	183	5	226	2582	2430	68	0	3290

TRINITY_DN15851_c0_g1_i1	Glyma.20G214600.5	96.51162791	170	7	226	2582	2427	68	0	3341

TRINITY_DN15851_c0_g1_i1	Glyma.20G214600.7	96.19047619	170	7	226	2582	2427	68	0	3341

TRINITY_DN15851_c0_g1_i1	Glyma.20G214600.3	97.70114943	170	7	226	2582	2427	68	0	3341

TRINITY_DN15851_c0_g1_i1	Glyma.20G214600.2	95.09981851	170	7	226	2582	2427	68	0	3341

TRINITY_DN10934_c0_g2_i2	Glyma.20G225800.1	100	470	36	564	2905	181	2519	0	1458

TRINITY_DN13524_c0_g2_i2	Glyma.08G086300.4	97.07317073	275	8	272	2633	21	2376	0	2778

TRINITY_DN13524_c0_g2_i2	Glyma.08G086300.2	97.07317073	275	8	272	2633	21	2376	0	2778

TRINITY_DN13524_c0_g2_i2	Glyma.08G086300.3	97.07602339	274	12	272	2633	21	2367	0	2724

TRINITY_DN14372_c0_g2_i1	Glyma.20G237900.1	98	232	12	180	2531	1	2352	0	2926

TRINITY_DN16181_c0_g1_i3	Glyma.17G248400.2	99.13793103	222	4	423	2784	2475	114	0	3110

TRINITY_DN15944_c2_g2_i5	Glyma.13G083300.4	95.45454545	196	18	196	2526	2361	1	0	3048

TRINITY_DN14780_c0_g1_i1	Glyma.20G126100.4	100	156	9	137	2482	2346	1	0	3269

TRINITY_DN14780_c0_g1_i1	Glyma.20G126100.1	100	156	9	137	2482	2346	1	0	3269

TRINITY_DN9351_c0_g1_i1	Glyma.17G137600.1	100	143	11	185	2524	2364	1	0	3419

TRINITY_DN7785_c0_g1_i1	Glyma.02G164700.1	100	132	3	672	3029	2364	1	0	3596

TRINITY_DN15944_c2_g2_i2	Glyma.04G253000.1	97.89473684	123	6	275	2629	2352	1	0	3548

TRINITY_DN16214_c0_g2_i1	Glyma.14G031600.1	100	428	47	1806	4125	2998	654	0	1598

TRINITY_DN16214_c0_g2_i2	Glyma.14G031600.1	100	428	47	1735	4054	2998	654	0	1598

TRINITY_DN16065_c0_g1_i6	Glyma.13G244300.1	100	283	43	299	2602	1	2346	0	2305

TRINITY_DN15944_c2_g2_i3	Glyma.13G083300.4	100	236	21	287	2614	2358	1	0	2798

TRINITY_DN14750_c0_g1_i1	Glyma.07G182900.2	95.21126761	230	7	563	2922	2375	22	0	3013

TRINITY_DN14750_c0_g1_i1	Glyma.07G182900.1	95.21276596	230	7	563	2922	2375	22	0	3013

TRINITY_DN14151_c0_g1_i1	Glyma.07G062500.1	100	206	11	199	2545	1	2359	0	3094

TRINITY_DN16181_c0_g1_i1	Glyma.17G248400.2	97.4025974	188	2	423	2784	2475	114	0	3310

TRINITY_DN16214_c0_g2_i3	Glyma.14G031600.1	100	417	45	1735	4054	2998	654	0	1670

TRINITY_DN16214_c0_g2_i4	Glyma.14G031600.1	100	417	45	1806	4125	2998	654	0	1670

TRINITY_DN14654_c0_g1_i7	Glyma.01G155300.1	98.94736842	261	11	1787	4147	2343	1	0	2787

TRINITY_DN16384_c0_g1_i1	Glyma.18G212400.2	100	188	8	185	2544	1	2344	0	3192

TRINITY_DN16384_c0_g1_i3	Glyma.18G212400.2	100	186	7	183	2542	1	2344	0	3203

TRINITY_DN13116_c0_g1_i2	Glyma.09G068800.1	97.84946237	175	3	128	2477	2362	1	0	3315

TRINITY_DN10814_c0_g2_i1	Glyma.11G139700.1	100	410	31	285	2632	33	2359	0	1784

TRINITY_DN13116_c0_g1_i1	Glyma.17G050100.6	97.87234043	354	25	366	2715	2332	1	0	2141

TRINITY_DN13116_c0_g1_i2	Glyma.17G050100.1	97.87234043	354	25	128	2477	2332	1	0	2141

TRINITY_DN13116_c0_g1_i2	Glyma.17G050100.4	97.87234043	354	25	128	2477	2332	1	0	2141

TRINITY_DN13116_c0_g1_i2	Glyma.17G050100.5	97.85714286	354	25	128	2477	2332	1	0	2141

TRINITY_DN13116_c0_g1_i2	Glyma.17G050100.2	97.85714286	354	25	128	2477	2332	1	0	2141

TRINITY_DN13116_c0_g1_i2	Glyma.17G050100.3	97.85714286	354	25	128	2477	2332	1	0	2141

TRINITY_DN16065_c0_g1_i6	Glyma.15G069400.1	99.21875	284	42	299	2602	1	2349	0	2340

TRINITY_DN15944_c2_g2_i3	Glyma.13G083300.3	95	235	22	292	2614	2356	1	0	2780

TRINITY_DN16082_c0_g1_i6	Glyma.08G202000.6	98.7654321	232	13	552	2889	2398	40	0	2913

TRINITY_DN16082_c0_g1_i2	Glyma.08G202000.6	100	211	13	581	2918	2398	40	0	3029

TRINITY_DN15944_c2_g2_i5	Glyma.13G083300.3	97.01492537	196	19	202	2526	2358	1	0	3022

TRINITY_DN14117_c0_g1_i1	Glyma.02G034300.1	98.11320755	178	12	306	2663	2766	436	0	3160

TRINITY_DN14117_c0_g1_i2	Glyma.02G034300.1	100	178	12	306	2663	2766	436	0	3160

TRINITY_DN14117_c0_g1_i1	Glyma.02G034300.4	95.87628866	178	12	306	2663	2457	127	0	3160

TRINITY_DN14117_c0_g1_i2	Glyma.02G034300.4	100	178	12	306	2663	2457	127	0	3160

TRINITY_DN16245_c0_g3_i6	Glyma.16G099700.2	95.96069869	176	0	672	3032	3200	840	0	3386

TRINITY_DN10163_c0_g1_i1	Glyma.01G019800.1	96.66666667	176	25	149	2492	1	2332	0	3088

TRINITY_DN10163_c0_g1_i1	Glyma.01G019800.2	98.52941176	176	25	149	2492	1	2332	0	3088

TRINITY_DN14521_c1_g1_i2	Glyma.16G046200.1	97.56097561	166	6	665	3019	2358	1	0	3382

TRINITY_DN14117_c0_g1_i1	Glyma.01G031400.1	96.92307692	160	11	306	2663	2784	445	0	3319

TRINITY_DN14117_c0_g1_i2	Glyma.01G031400.1	99.2481203	160	11	306	2663	2784	445	0	3319

TRINITY_DN14521_c1_g1_i2	Glyma.19G105900.1	99.20634921	159	6	665	3019	2358	1	0	3421

TRINITY_DN14780_c0_g1_i1	Glyma.20G126100.2	100	156	8	137	2482	2343	1	0	3282

TRINITY_DN14780_c0_g1_i1	Glyma.20G126100.3	100	156	8	137	2482	2343	1	0	3282

TRINITY_DN16271_c0_g1_i3	Glyma.18G300200.1	97.5708502	103	0	27	2387	5877	3517	0	3790

TRINITY_DN16271_c0_g1_i3	Glyma.18G300200.2	97.18309859	103	0	27	2387	5784	3424	0	3790

TRINITY_DN16271_c0_g1_i3	Glyma.08G361500.1	96.95431472	93	0	27	2387	5877	3517	0	3845

TRINITY_DN11957_c0_g2_i1	Glyma.20G045400.1	98.47715736	438	24	288	2632	2357	13	0	1738

TRINITY_DN14680_c0_g2_i4	Glyma.13G134500.1	100	419	41	43	2374	353	2672	0	1598

TRINITY_DN13433_c0_g2_i1	Glyma.10G024500.1	98.71794872	360	47	2	2323	3449	1135	0	1827

TRINITY_DN13433_c0_g2_i1	Glyma.10G024500.9	97.4137931	360	47	2	2323	2990	676	0	1827

TRINITY_DN13433_c0_g2_i1	Glyma.10G024500.6	97.4137931	360	47	2	2323	2990	676	0	1827

TRINITY_DN13433_c0_g2_i1	Glyma.10G024500.4	96.875	360	47	2	2323	2990	676	0	1827

TRINITY_DN16231_c1_g1_i1	Glyma.09G073600.1	100	308	10	114	2468	35	2389	0	2588

TRINITY_DN15385_c0_g1_i5	Glyma.18G248200.2	98.01980198	225	12	1571	3910	2358	10	0	2911

TRINITY_DN15385_c0_g1_i5	Glyma.18G248200.1	98.07692308	225	12	1571	3910	2358	10	0	2911

TRINITY_DN15944_c2_g2_i6	Glyma.06G109500.1	97.14285714	169	7	199	2541	2358	1	0	3299

TRINITY_DN15944_c2_g2_i6	Glyma.06G109500.2	97.5	169	8	199	2541	2355	1	0	3280

TRINITY_DN16134_c0_g3_i1	Glyma.18G171100.1	100	163	16	109	2433	2325	1	0	3003

TRINITY_DN10291_c0_g1_i2	Glyma.08G251400.1	100	134	11	179	2529	26	2364	0	3422

TRINITY_DN7785_c0_g1_i1	Glyma.10G102400.1	99.49238579	115	1	673	3029	2360	1	0	3701

TRINITY_DN7785_c0_g1_i1	Glyma.10G102400.3	99.49238579	114	2	673	3029	2357	1	0	3688

TRINITY_DN7785_c0_g1_i1	Glyma.10G102400.4	97.91921665	114	4	673	3029	2354	1	0	3668

TRINITY_DN7785_c0_g1_i1	Glyma.10G102400.5	97.12286159	113	5	673	3029	2351	1	0	3655

TRINITY_DN16082_c0_g1_i4	Glyma.08G202000.6	95.1048951	281	17	552	2875	2398	42	0	2560

TRINITY_DN16082_c0_g1_i14	Glyma.08G202000.6	95.75892857	260	17	581	2904	2398	42	0	2676

TRINITY_DN10814_c0_g1_i1	Glyma.11G139700.1	96.55172414	231	0	174	2532	1	2359	0	3077

TRINITY_DN14680_c0_g2_i4	Glyma.10G046400.1	99.09090909	418	38	43	2374	353	2675	0	1644

TRINITY_DN14680_c0_g2_i4	Glyma.10G046400.2	100	418	38	43	2374	353	2675	0	1644

TRINITY_DN13116_c0_g1_i2	Glyma.13G109600.12	99.49622166	354	22	128	2477	2332	1	0	2174

TRINITY_DN13116_c0_g1_i2	Glyma.13G109600.2	97.87234043	354	22	128	2477	2332	1	0	2174

TRINITY_DN13116_c0_g1_i2	Glyma.13G109600.13	97.87234043	354	22	128	2477	2332	1	0	2174

TRINITY_DN13116_c0_g1_i2	Glyma.13G109600.6	97.86516854	354	22	128	2477	2332	1	0	2174

TRINITY_DN13116_c0_g1_i2	Glyma.13G109600.3	97.87234043	354	22	128	2477	2332	1	0	2174

TRINITY_DN13116_c0_g1_i2	Glyma.13G109600.1	97.87234043	354	22	128	2477	2332	1	0	2174

TRINITY_DN13116_c0_g1_i2	Glyma.13G109600.7	97.86585366	354	22	128	2477	2332	1	0	2174

TRINITY_DN13116_c0_g1_i2	Glyma.13G109600.8	97.84946237	354	22	128	2477	2332	1	0	2174

TRINITY_DN15944_c2_g2_i3	Glyma.13G083300.2	96.08355091	235	21	292	2614	2353	1	0	2795

TRINITY_DN15944_c2_g2_i5	Glyma.13G083300.2	100	196	18	202	2526	2355	1	0	3037

TRINITY_DN14780_c0_g1_i1	Glyma.10G264200.1	100	165	7	137	2482	2355	1	0	3343

TRINITY_DN16425_c0_g1_i3	Glyma.12G060400.1	98.18181818	158	5	427	2777	3102	746	0	3428

TRINITY_DN9122_c0_g1_i1	Glyma.02G248300.2	100	157	4	10	2363	4	2360	0	3452

TRINITY_DN9122_c0_g1_i1	Glyma.02G248300.1	98.18181818	157	4	10	2363	4	2360	0	3452

TRINITY_DN16425_c0_g1_i1	Glyma.12G060400.1	99.05660377	153	5	494	2844	3102	746	0	3456

TRINITY_DN9122_c0_g1_i1	Glyma.14G068200.1	95.94272076	146	4	10	2363	4	2360	0	3513

TRINITY_DN16163_c0_g1_i2	Glyma.13G308200.2	95.50561798	128	6	470	2824	1	2346	0	3548

TRINITY_DN16163_c0_g1_i2	Glyma.13G308200.1	96.10705596	128	6	470	2824	1	2346	0	3548

TRINITY_DN16163_c0_g1_i6	Glyma.13G308200.2	96	128	6	448	2802	1	2346	0	3548

TRINITY_DN16163_c0_g1_i6	Glyma.13G308200.1	97.18076285	128	6	448	2802	1	2346	0	3548

TRINITY_DN16163_c0_g1_i2	Glyma.12G194400.1	99.51456311	124	6	470	2824	1	2346	0	3570

TRINITY_DN16163_c0_g1_i2	Glyma.12G194400.9	95.50561798	124	6	470	2824	1	2346	0	3570

TRINITY_DN16163_c0_g1_i6	Glyma.12G194400.1	95.23809524	124	6	448	2802	1	2346	0	3570

TRINITY_DN16163_c0_g1_i6	Glyma.12G194400.9	96	124	6	448	2802	1	2346	0	3570

TRINITY_DN16163_c0_g1_i2	Glyma.12G194400.7	96.10778443	122	7	470	2824	1	2331	0	3485

TRINITY_DN16163_c0_g1_i2	Glyma.12G194400.8	95	122	7	470	2824	1	2331	0	3485

TRINITY_DN16163_c0_g1_i6	Glyma.12G194400.7	95.50748752	122	7	448	2802	1	2331	0	3485

TRINITY_DN16163_c0_g1_i6	Glyma.12G194400.8	95.50898204	122	7	448	2802	1	2331	0	3485

TRINITY_DN11262_c0_g1_i1	Glyma.04G056400.3	100	217	7	649	2998	4369	2014	0	3099

TRINITY_DN35829_c0_g1_i1	Glyma.08G028400.1	97.10144928	164	4	435	2785	2391	44	0	3347

TRINITY_DN16425_c0_g1_i2	Glyma.12G060400.1	100	162	3	494	2844	3102	746	0	3417

TRINITY_DN16425_c0_g1_i5	Glyma.12G060400.1	99.27007299	162	3	494	2844	3102	746	0	3417

TRINITY_DN10137_c0_g1_i2	Glyma.13G174600.1	99.32885906	155	3	468	2824	8157	5815	0	3404

TRINITY_DN16415_c1_g1_i7	Glyma.12G173700.1	95.61904762	361	6	1364	3714	2416	63	0	2305

TRINITY_DN14801_c0_g1_i2	Glyma.06G115600.1	95.95375723	211	10	395	2737	2352	1	0	3072

TRINITY_DN17020_c0_g1_i1	Glyma.11G229000.1	95.71984436	186	5	846	3198	2417	65	0	3282

TRINITY_DN16237_c0_g1_i13	Glyma.06G164900.2	98.9010989	178	24	260	2560	1	2346	0	2944

TRINITY_DN16237_c0_g1_i7	Glyma.06G164900.2	95.40229885	178	24	278	2578	1	2346	0	2944

TRINITY_DN11262_c0_g1_i3	Glyma.04G056400.3	96.89922481	174	5	649	2995	4369	2014	0	3328

TRINITY_DN16237_c0_g1_i13	Glyma.04G200600.2	100	169	22	260	2560	1	2346	0	2994

TRINITY_DN16237_c0_g1_i7	Glyma.04G200600.2	100	169	22	278	2578	1	2346	0	2994

TRINITY_DN15621_c0_g1_i2	Glyma.19G089800.1	97.6744186	122	0	1	2356	18	2373	0	3675

TRINITY_DN15621_c0_g1_i3	Glyma.19G089800.1	100	122	0	1	2356	18	2373	0	3675

TRINITY_DN16436_c0_g2_i11	Glyma.15G033000.1	96.9581749	393	33	188	2510	5	2334	0	1803

TRINITY_DN15699_c0_g1_i5	Glyma.19G056700.1	95.6043956	314	16	105	2445	405	2751	0	2468

TRINITY_DN15699_c0_g1_i3	Glyma.19G056700.1	97.95918367	299	16	105	2445	405	2751	0	2551

TRINITY_DN16415_c1_g1_i1	Glyma.U018900.2	99.23664122	209	5	1153	3507	2346	1	0	3133

TRINITY_DN16415_c1_g1_i10	Glyma.U018900.2	100	209	5	746	3100	2346	1	0	3133

TRINITY_DN16415_c1_g1_i1	Glyma.11G147900.3	99.43820225	181	2	1153	3507	2496	148	0	3308

TRINITY_DN16415_c1_g1_i10	Glyma.11G147900.3	98.1595092	181	2	746	3100	2496	148	0	3308

TRINITY_DN16415_c1_g1_i1	Glyma.11G147900.2	99.82993197	181	2	1153	3507	2349	1	0	3308

TRINITY_DN16415_c1_g1_i10	Glyma.11G147900.2	100	181	2	746	3100	2349	1	0	3308

TRINITY_DN15845_c0_g1_i3	Glyma.11G006900.1	99.12280702	174	31	108	2387	1	2325	0	2706

TRINITY_DN15845_c0_g1_i3	Glyma.01G236300.1	99.12280702	173	26	108	2387	1	2325	0	2712

TRINITY_DN14042_c0_g1_i1	Glyma.13G332300.9	100	166	0	446	2800	1	2355	0	3430

TRINITY_DN14042_c0_g1_i1	Glyma.13G332300.7	100	166	0	446	2800	1	2355	0	3430

TRINITY_DN14042_c0_g1_i1	Glyma.13G332300.3	100	166	0	446	2800	1	2355	0	3430

TRINITY_DN14042_c0_g1_i1	Glyma.13G332300.4	98.79518072	166	0	446	2800	1	2355	0	3430

TRINITY_DN14042_c0_g1_i2	Glyma.13G332300.9	95.11568123	166	0	663	3017	1	2355	0	3430

TRINITY_DN14042_c0_g1_i2	Glyma.13G332300.7	99.0521327	166	0	663	3017	1	2355	0	3430

TRINITY_DN14042_c0_g1_i2	Glyma.13G332300.3	95.3125	166	0	663	3017	1	2355	0	3430

TRINITY_DN14042_c0_g1_i2	Glyma.13G332300.4	96.72330097	166	0	663	3017	1	2355	0	3430

TRINITY_DN14042_c0_g1_i3	Glyma.13G332300.9	95	166	0	663	3017	1	2355	0	3430

TRINITY_DN14042_c0_g1_i3	Glyma.13G332300.7	98.05825243	166	0	663	3017	1	2355	0	3430

TRINITY_DN14042_c0_g1_i3	Glyma.13G332300.3	100	166	0	663	3017	1	2355	0	3430

TRINITY_DN14042_c0_g1_i3	Glyma.13G332300.4	99.02912621	166	0	663	3017	1	2355	0	3430

TRINITY_DN14042_c0_g1_i4	Glyma.13G332300.9	96.94323144	166	0	446	2800	1	2355	0	3430

TRINITY_DN14042_c0_g1_i4	Glyma.13G332300.7	98.33759591	166	0	446	2800	1	2355	0	3430

TRINITY_DN14042_c0_g1_i4	Glyma.13G332300.3	100	166	0	446	2800	1	2355	0	3430

TRINITY_DN14042_c0_g1_i4	Glyma.13G332300.4	98.73417722	166	0	446	2800	1	2355	0	3430

TRINITY_DN9404_c0_g1_i1	Glyma.08G257600.4	97.5	110	2	404	2754	2433	79	0	3714

TRINITY_DN10755_c0_g1_i2	Glyma.14G079600.1	96.3190184	367	33	255	2585	139	2451	0	1901

TRINITY_DN12679_c0_g1_i1	Glyma.18G004000.1	95.29411765	213	2	1371	3723	470	2822	0	3155

TRINITY_DN12679_c0_g1_i2	Glyma.18G004000.1	95.29411765	213	2	1371	3723	470	2822	0	3155

TRINITY_DN429_c0_g1_i1	Glyma.19G228100.1	97.87234043	135	0	1	2354	847	3200	0	3600

TRINITY_DN16190_c0_g3_i8	Glyma.13G290700.1	100	427	16	900	3245	596	2937	0	1864

TRINITY_DN14801_c0_g1_i2	Glyma.04G247200.1	100	192	7	395	2737	2352	1	0	3210

TRINITY_DN15207_c0_g1_i1	Glyma.18G001100.1	96.47058824	188	25	318	2661	51	2349	0	2896

TRINITY_DN15207_c0_g1_i1	Glyma.18G001100.2	98.20627803	188	26	318	2661	51	2346	0	2878

TRINITY_DN5703_c0_g1_i1	Glyma.13G210500.1	100	178	5	240	2583	2502	153	0	3282

TRINITY_DN16237_c0_g1_i13	Glyma.06G164900.1	98.51851852	178	24	260	2557	1	2343	0	2939

TRINITY_DN16237_c0_g1_i4	Glyma.06G164900.2	99.38650307	178	24	260	2557	1	2343	0	2939

TRINITY_DN16237_c0_g1_i6	Glyma.06G164900.2	96.18320611	178	24	278	2575	1	2343	0	2939

TRINITY_DN16237_c0_g1_i7	Glyma.06G164900.1	95.40229885	178	24	278	2575	1	2343	0	2939

TRINITY_DN16237_c0_g1_i9	Glyma.06G164900.2	98.26589595	178	24	266	2563	1	2343	0	2939

TRINITY_DN16237_c0_g1_i13	Glyma.04G200600.1	100	169	22	260	2557	1	2343	0	2988

TRINITY_DN16237_c0_g1_i4	Glyma.04G200600.2	99.38650307	169	22	260	2557	1	2343	0	2988

TRINITY_DN16237_c0_g1_i6	Glyma.04G200600.2	96.66666667	169	22	278	2575	1	2343	0	2988

TRINITY_DN16237_c0_g1_i7	Glyma.04G200600.1	99.40828402	169	22	278	2575	1	2343	0	2988

TRINITY_DN16237_c0_g1_i9	Glyma.04G200600.2	98.26589595	169	22	266	2563	1	2343	0	2988

TRINITY_DN10814_c0_g1_i1	Glyma.15G038600.1	100	402	30	204	2532	121	2452	0	1838

TRINITY_DN37246_c0_g1_i1	Glyma.14G011500.1	98.64864865	390	7	1	2337	934	3282	0	2067

TRINITY_DN4552_c0_g1_i1	Glyma.11G241400.1	98.92473118	385	42	366	2686	2342	28	0	1772

TRINITY_DN16285_c1_g1_i1	Glyma.18G017600.1	100	285	9	633	2980	1	2342	0	2675

TRINITY_DN16163_c0_g1_i1	Glyma.12G194400.1	95.54455446	258	14	448	2782	1	2344	0	2752

TRINITY_DN16163_c0_g1_i1	Glyma.12G194400.9	95.54565702	258	14	448	2782	1	2344	0	2752

TRINITY_DN16163_c0_g1_i3	Glyma.12G194400.1	100	258	14	470	2804	1	2344	0	2752

TRINITY_DN16163_c0_g1_i3	Glyma.12G194400.9	98.41017488	258	14	470	2804	1	2344	0	2752

TRINITY_DN16163_c0_g1_i1	Glyma.12G194400.7	96	255	16	448	2782	1	2329	0	2673

TRINITY_DN16163_c0_g1_i1	Glyma.12G194400.8	96	255	16	448	2782	1	2329	0	2673

TRINITY_DN16163_c0_g1_i3	Glyma.12G194400.7	97.54601227	255	16	470	2804	1	2329	0	2673

TRINITY_DN16163_c0_g1_i3	Glyma.12G194400.8	100	255	16	470	2804	1	2329	0	2673

TRINITY_DN16397_c0_g1_i5	Glyma.19G065600.2	98.16513761	241	4	3933	6282	2830	481	0	2983

TRINITY_DN16397_c0_g1_i5	Glyma.19G065600.3	98.16513761	241	4	3933	6282	2686	337	0	2983

TRINITY_DN15944_c2_g2_i6	Glyma.04G253000.1	98.63013699	173	6	199	2541	2340	1	0	3249

TRINITY_DN15944_c2_g2_i5	Glyma.14G164600.4	97.92387543	167	15	196	2526	2346	1	0	3243

TRINITY_DN5747_c0_g1_i1	Glyma.13G235600.2	98.125	138	14	242	2565	169	2519	0	3391

TRINITY_DN16086_c0_g2_i1	Glyma.13G369300.1	100	522	28	283	2619	7	2343	0	1269

TRINITY_DN16323_c0_g2_i1	Glyma.18G249800.1	95.75289575	349	30	327	2650	2402	67	0	2137

TRINITY_DN16163_c0_g1_i1	Glyma.13G308200.2	100	262	13	448	2782	1	2344	0	2741

TRINITY_DN16163_c0_g1_i1	Glyma.13G308200.1	100	262	13	448	2782	1	2344	0	2741

TRINITY_DN16163_c0_g1_i3	Glyma.13G308200.2	100	262	13	470	2804	1	2344	0	2741

TRINITY_DN16163_c0_g1_i3	Glyma.13G308200.1	98.67256637	262	13	470	2804	1	2344	0	2741

TRINITY_DN10681_c1_g2_i1	Glyma.16G157400.1	95.58823529	260	8	105	2454	174	2508	0	2791

TRINITY_DN13366_c0_g1_i1	Glyma.06G023600.3	99.53917051	238	14	1180	3526	2323	1	0	2817

TRINITY_DN16397_c0_g1_i6	Glyma.19G065600.2	98.14814815	214	2	3933	6282	2830	481	0	3144

TRINITY_DN16397_c0_g1_i6	Glyma.19G065600.3	98.1595092	214	2	3933	6282	2686	337	0	3144

TRINITY_DN15944_c2_g2_i3	Glyma.14G164600.4	98.69565217	208	18	287	2614	2343	1	0	2988

TRINITY_DN15385_c0_g1_i2	Glyma.18G248200.2	96.12403101	205	7	1627	3957	2358	10	0	3064

TRINITY_DN15385_c0_g1_i2	Glyma.18G248200.1	96.82539683	205	7	1627	3957	2358	10	0	3064

TRINITY_DN10946_c0_g2_i1	Glyma.16G059700.1	98.11320755	406	29	484	2815	2374	46	0	1838

TRINITY_DN16436_c0_g2_i1	Glyma.15G033000.1	99.53051643	390	35	188	2505	5	2329	0	1810

TRINITY_DN16436_c0_g2_i10	Glyma.15G033000.1	95.2681388	390	37	188	2505	5	2329	0	1810

TRINITY_DN15345_c0_g1_i1	Glyma.12G099500.5	98.88888889	367	31	709	3038	1	2330	0	2049

TRINITY_DN15345_c0_g1_i1	Glyma.12G099500.2	99.5	367	31	709	3038	1	2330	0	2049

TRINITY_DN15345_c0_g1_i1	Glyma.12G099500.3	98.80952381	367	31	709	3038	1	2330	0	2049

TRINITY_DN15345_c0_g1_i2	Glyma.12G099500.5	100	367	31	515	2844	1	2330	0	2049

TRINITY_DN15345_c0_g1_i2	Glyma.12G099500.2	95.0310559	367	31	515	2844	1	2330	0	2049

TRINITY_DN15345_c0_g1_i2	Glyma.12G099500.3	100	367	31	515	2844	1	2330	0	2049

TRINITY_DN10681_c1_g2_i1	Glyma.02G075600.1	97.5	236	4	106	2454	172	2511	0	2961

TRINITY_DN16285_c1_g1_i2	Glyma.18G017600.1	97.33727811	216	5	633	2980	1	2342	0	3079

TRINITY_DN10163_c0_g1_i1	Glyma.09G202600.3	96.05263158	187	13	149	2493	1	2321	0	3085

TRINITY_DN10163_c0_g1_i1	Glyma.09G202600.1	96.05263158	187	13	149	2493	1	2321	0	3085

TRINITY_DN10163_c0_g1_i1	Glyma.09G202600.2	97.36842105	187	13	149	2493	1	2321	0	3085

TRINITY_DN109_c0_g2_i1	Glyma.03G220600.1	97.58064516	174	19	260	2572	2349	1	0	3131

TRINITY_DN12910_c0_g1_i2	Glyma.06G222100.1	97.42574257	157	0	293	2642	5664	3315	0	3470

TRINITY_DN13340_c0_g1_i1	Glyma.07G045100.1	97.09821429	157	7	272	2617	2340	1	0	3380

TRINITY_DN11254_c0_g1_i1	Glyma.15G026400.2	95.89041096	544	59	141	2449	2397	86	0	826

TRINITY_DN16082_c0_g1_i6	Glyma.08G202000.3	96.05633803	233	8	552	2889	2386	40	0	2963

TRINITY_DN16082_c0_g1_i2	Glyma.08G202000.3	97.91666667	212	8	581	2918	2386	40	0	3079

TRINITY_DN13023_c0_g1_i1	Glyma.10G241600.1	98.38709677	209	3	275	2614	1	2349	0	3121

TRINITY_DN15385_c0_g1_i5	Glyma.09G245000.1	96.15384615	205	13	1571	3910	2337	10	0	3009

TRINITY_DN15385_c0_g1_i5	Glyma.09G245000.2	97.2972973	205	13	1571	3910	2337	10	0	3009

TRINITY_DN13023_c0_g1_i2	Glyma.10G241600.1	100	175	2	275	2617	1	2349	0	3330

TRINITY_DN15944_c2_g2_i5	Glyma.14G164600.3	98.94736842	167	14	196	2526	2343	1	0	3258

TRINITY_DN16376_c3_g12_i1	Glyma.05G151100.2	96.2406015	167	17	124	2466	5	2329	0	3219

TRINITY_DN16376_c3_g12_i1	Glyma.05G151100.1	96.21621622	167	17	124	2466	5	2329	0	3219

TRINITY_DN16376_c3_g12_i1	Glyma.08G107800.1	96.21212121	163	14	124	2466	5	2329	0	3241

TRINITY_DN13340_c0_g1_i1	Glyma.16G013100.1	100	162	1	272	2617	2349	1	0	3421

TRINITY_DN13231_c0_g1_i2	Glyma.19G010900.1	97.52066116	160	4	349	2694	2343	1	0	3393

TRINITY_DN13231_c0_g1_i2	Glyma.13G170700.1	100	157	5	349	2694	2340	1	0	3391

TRINITY_DN16065_c0_g1_i1	Glyma.13G244300.1	100	155	1	299	2647	1	2346	0	3459

TRINITY_DN16065_c0_g1_i1	Glyma.15G069400.1	96.42857143	142	0	299	2647	1	2349	0	3552

TRINITY_DN11713_c0_g1_i1	Glyma.13G114900.1	100	123	0	374	2722	2349	1	0	3657

TRINITY_DN16082_c0_g1_i4	Glyma.07G016700.1	97.14285714	278	17	552	2875	2380	36	0	2621

TRINITY_DN15944_c2_g2_i3	Glyma.14G164600.3	98.67549669	208	17	287	2614	2340	1	0	3003

TRINITY_DN16384_c0_g1_i4	Glyma.18G212400.2	100	163	6	185	2526	1	2333	0	3297

TRINITY_DN16384_c0_g1_i2	Glyma.18G212400.2	100	161	6	183	2524	1	2333	0	3308

TRINITY_DN14721_c0_g1_i2	Glyma.11G165900.1	98.79518072	155	9	636	2970	558	2889	0	3290

TRINITY_DN14877_c0_g1_i3	Glyma.13G067800.3	98.88268156	141	2	1196	3537	3317	971	0	3509

TRINITY_DN14877_c0_g1_i3	Glyma.13G067800.1	100	141	2	1196	3537	3317	971	0	3509

TRINITY_DN14877_c0_g1_i3	Glyma.13G067800.2	99.58847737	141	2	1196	3537	3314	968	0	3509

TRINITY_DN16086_c0_g2_i4	Glyma.13G097900.1	99.20948617	443	24	109	2448	22	2343	0	1674

TRINITY_DN16086_c0_g2_i1	Glyma.13G097900.1	96.80851064	440	24	280	2619	22	2343	0	1690

TRINITY_DN16082_c0_g1_i4	Glyma.08G202000.3	97.02970297	282	12	552	2875	2386	42	0	2610

TRINITY_DN12257_c0_g1_i1	Glyma.09G021400.1	97.5	140	4	118	2440	148	2490	0	3378

TRINITY_DN12257_c0_g1_i1	Glyma.15G127700.1	97.5	139	5	118	2440	148	2490	0	3384

TRINITY_DN16086_c0_g2_i1	Glyma.17G061800.1	95.18072289	434	16	280	2619	22	2355	0	1812

TRINITY_DN16086_c0_g2_i4	Glyma.17G061800.1	97.22222222	433	16	109	2448	22	2355	0	1818

TRINITY_DN10946_c0_g2_i1	Glyma.19G087800.1	96.18320611	419	26	484	2815	2371	46	0	1792

TRINITY_DN16082_c0_g1_i14	Glyma.07G016700.1	96.9924812	260	14	581	2904	2380	36	0	2743

TRINITY_DN15886_c3_g1_i1	Glyma.15G190200.3	98.11320755	233	8	1156	3497	74	2406	0	2931

TRINITY_DN15886_c3_g1_i3	Glyma.15G190200.3	100	233	8	1365	3706	74	2406	0	2931

TRINITY_DN15944_c2_g2_i3	Glyma.14G164600.1	95.85492228	207	18	292	2614	2338	1	0	2985

TRINITY_DN13023_c0_g1_i1	Glyma.20G152600.1	98.38709677	193	2	275	2614	1	2346	0	3225

TRINITY_DN9258_c1_g1_i2	Glyma.19G115800.6	99.78118162	162	0	159	2504	2346	1	0	3435

TRINITY_DN9258_c1_g1_i2	Glyma.19G115800.1	96.33507853	162	0	159	2504	2346	1	0	3435

TRINITY_DN9258_c1_g1_i2	Glyma.19G115800.3	97.05882353	162	0	159	2504	2346	1	0	3435

TRINITY_DN9258_c1_g1_i2	Glyma.19G115800.5	97.95918367	162	0	159	2504	2346	1	0	3435

TRINITY_DN9258_c1_g1_i2	Glyma.19G115800.2	98.64253394	162	0	159	2504	2346	1	0	3435

TRINITY_DN13023_c0_g1_i2	Glyma.20G152600.1	97.65886288	155	1	275	2617	1	2346	0	3454

TRINITY_DN5735_c0_g1_i1	Glyma.07G096500.1	98.34254144	128	3	58	2399	2889	545	0	3591

TRINITY_DN5735_c0_g1_i1	Glyma.07G096500.2	98.34254144	128	3	58	2399	2889	545	0	3591

TRINITY_DN5735_c0_g1_i1	Glyma.09G181200.1	95.53450609	118	3	58	2399	2889	545	0	3646

TRINITY_DN16082_c0_g1_i14	Glyma.08G202000.3	96.48760331	265	9	581	2904	2386	42	0	2726

TRINITY_DN5668_c0_g1_i1	Glyma.13G236800.1	100	253	29	8	2339	483	2766	0	2451

TRINITY_DN5668_c0_g1_i1	Glyma.13G236800.2	100	253	29	8	2339	483	2766	0	2451

TRINITY_DN16309_c0_g1_i2	Glyma.11G033600.5	98.21428571	243	31	77	2388	1	2309	0	2538

TRINITY_DN16309_c0_g1_i2	Glyma.11G033600.7	98.21428571	243	31	77	2388	1	2309	0	2538

TRINITY_DN16309_c0_g1_i2	Glyma.11G033600.6	97.5	243	31	77	2388	1	2309	0	2538

TRINITY_DN16309_c0_g1_i2	Glyma.11G033600.2	97.98994975	243	31	77	2388	1	2309	0	2538

TRINITY_DN16309_c0_g1_i2	Glyma.11G033600.1	97.5	243	31	77	2388	1	2309	0	2538

TRINITY_DN16309_c0_g1_i2	Glyma.11G033600.3	97.98994975	243	31	77	2388	1	2309	0	2538

TRINITY_DN16309_c0_g1_i2	Glyma.11G033600.4	97.5	243	31	77	2388	1	2309	0	2538

TRINITY_DN16309_c0_g1_i4	Glyma.11G033600.2	100	243	31	77	2388	1	2309	0	2538

TRINITY_DN16309_c0_g1_i4	Glyma.11G033600.1	99.3006993	243	31	77	2388	1	2309	0	2538

TRINITY_DN16309_c0_g1_i4	Glyma.11G033600.3	99.31506849	243	31	77	2388	1	2309	0	2538

TRINITY_DN16309_c0_g1_i4	Glyma.11G033600.4	99.30555556	243	31	77	2388	1	2309	0	2538

TRINITY_DN16309_c0_g1_i4	Glyma.11G033600.5	99.31972789	243	31	77	2388	1	2309	0	2538

TRINITY_DN16309_c0_g1_i4	Glyma.11G033600.7	99.31271478	243	31	77	2388	1	2309	0	2538

TRINITY_DN16309_c0_g1_i4	Glyma.11G033600.6	99.30555556	243	31	77	2388	1	2309	0	2538

TRINITY_DN5625_c0_g2_i1	Glyma.19G200000.1	100	160	9	617	2940	2364	20	0	3308

TRINITY_DN15699_c0_g1_i5	Glyma.19G003200.1	95.6043956	124	2	102	2445	408	2751	0	3631

TRINITY_DN15944_c2_g2_i5	Glyma.14G164600.1	97.9253112	167	15	204	2526	2338	1	0	3229

TRINITY_DN15699_c0_g1_i3	Glyma.19G003200.1	100	136	0	102	2445	408	2751	0	3576

TRINITY_DN3649_c0_g1_i1	Glyma.07G104700.1	97.34513274	392	35	450	2756	3465	1138	0	1825

TRINITY_DN11681_c0_g1_i2	Glyma.17G225800.1	99.4011976	371	16	552	2874	501	2832	0	2071

TRINITY_DN11681_c0_g1_i2	Glyma.17G225800.2	100	371	16	552	2874	60	2391	0	2071

TRINITY_DN12362_c0_g1_i1	Glyma.11G233500.1	96.66666667	352	19	163	2484	2518	179	0	2222

TRINITY_DN12362_c0_g1_i4	Glyma.11G233500.1	99.09090909	352	19	163	2484	2518	179	0	2222

TRINITY_DN16360_c0_g8_i6	Glyma.11G073100.1	100	276	8	278	2611	94	2433	0	2721

TRINITY_DN11898_c0_g2_i3	Glyma.06G093500.2	98.74213836	236	8	859	3194	2432	103	0	2891

TRINITY_DN14680_c0_g2_i3	Glyma.19G175600.1	98.92473118	229	20	1	2322	374	2698	0	2806

TRINITY_DN14680_c0_g2_i3	Glyma.19G175600.2	100	229	20	1	2322	374	2698	0	2806

TRINITY_DN16309_c0_g1_i2	Glyma.01G208600.1	97.5	221	38	77	2385	1	2270	0	2410

TRINITY_DN16309_c0_g1_i4	Glyma.01G208600.1	100	221	38	77	2385	1	2270	0	2410

TRINITY_DN15944_c2_g2_i3	Glyma.14G164600.2	98.67549669	207	17	292	2614	2335	1	0	3000

TRINITY_DN11898_c0_g2_i1	Glyma.06G093500.2	98.75	205	6	889	3227	2432	103	0	3081

TRINITY_DN15636_c0_g1_i2	Glyma.12G211000.3	100	175	3	602	2941	2343	1	0	3338

TRINITY_DN16106_c0_g3_i3	Glyma.04G070200.1	95.61403509	173	23	299	2593	2748	409	0	3038

TRINITY_DN16106_c0_g3_i3	Glyma.04G070200.2	99.13793103	173	23	299	2593	2739	400	0	3038

TRINITY_DN16106_c0_g3_i5	Glyma.04G070200.1	95.91836735	167	9	299	2620	2748	409	0	3247

TRINITY_DN16106_c0_g3_i5	Glyma.04G070200.2	95.91836735	167	9	299	2620	2739	400	0	3247

TRINITY_DN15636_c0_g1_i2	Glyma.13G290400.2	95.84352078	166	3	602	2941	2343	1	0	3387

TRINITY_DN15636_c0_g1_i2	Glyma.13G290400.3	95.84415584	166	3	602	2941	2343	1	0	3387

TRINITY_DN10137_c0_g1_i1	Glyma.13G174600.1	96.81908549	154	0	468	2810	8157	5815	0	3474

TRINITY_DN15636_c0_g1_i4	Glyma.12G211000.3	99.17012448	137	3	602	2941	2343	1	0	3548

TRINITY_DN9351_c0_g1_i1	Glyma.05G055500.1	100	133	3	185	2524	2343	1	0	3570

TRINITY_DN15636_c0_g1_i4	Glyma.13G290400.2	98.62385321	130	3	602	2941	2343	1	0	3587

TRINITY_DN15636_c0_g1_i4	Glyma.13G290400.3	98.62385321	130	3	602	2941	2343	1	0	3587

TRINITY_DN3642_c0_g1_i1	Glyma.06G022600.1	95.89041096	389	49	23	2329	1	2313	0	1757

TRINITY_DN16190_c0_g3_i4	Glyma.13G290700.1	96.28865979	289	2	900	3239	596	2937	0	2712

TRINITY_DN16082_c0_g1_i14	Glyma.07G016500.1	97.95918367	240	11	581	2904	2371	36	0	2841

TRINITY_DN5651_c0_g1_i1	Glyma.06G308700.1	100	181	4	1	2336	491	2829	0	3264

TRINITY_DN5651_c0_g1_i1	Glyma.06G308700.2	98.94736842	181	4	1	2336	479	2817	0	3264

TRINITY_DN5651_c0_g1_i1	Glyma.12G096100.1	98.94736842	169	2	1	2336	509	2850	0	3350

TRINITY_DN9258_c1_g1_i2	Glyma.16G037500.2	100	166	3	159	2496	2349	9	0	3373

TRINITY_DN9258_c1_g1_i2	Glyma.16G037500.5	98.02631579	166	3	159	2496	2349	9	0	3373

TRINITY_DN9258_c1_g1_i2	Glyma.16G037500.3	96.18717504	166	3	159	2496	2349	9	0	3373

TRINITY_DN9258_c1_g1_i2	Glyma.16G037500.1	96.92307692	166	3	159	2496	2349	9	0	3373

TRINITY_DN15981_c0_g1_i1	Glyma.11G048300.2	95.8677686	153	3	1370	3705	2342	1	0	3439

TRINITY_DN15981_c0_g1_i1	Glyma.11G048300.1	98.14814815	153	3	1370	3705	2342	1	0	3439

TRINITY_DN15981_c0_g1_i23	Glyma.11G048300.2	96.11650485	153	3	1480	3815	2342	1	0	3439

TRINITY_DN15981_c0_g1_i23	Glyma.11G048300.1	96.55172414	153	3	1480	3815	2342	1	0	3439

TRINITY_DN15981_c0_g1_i4	Glyma.11G048300.2	95.18072289	153	3	1244	3579	2342	1	0	3439

TRINITY_DN15981_c0_g1_i4	Glyma.11G048300.1	100	153	3	1244	3579	2342	1	0	3439

TRINITY_DN15981_c0_g1_i7	Glyma.11G048300.2	97.00598802	153	3	1354	3689	2342	1	0	3439

TRINITY_DN15981_c0_g1_i7	Glyma.11G048300.1	100	153	3	1354	3689	2342	1	0	3439

TRINITY_DN16086_c0_g2_i4	Glyma.13G369300.1	99.20948617	525	32	124	2448	19	2343	0	1208

TRINITY_DN15423_c0_g1_i2	Glyma.11G190900.1	100	483	33	459	2780	347	2667	0	1395

TRINITY_DN15423_c0_g1_i2	Glyma.11G190900.2	97.69230769	483	33	459	2780	347	2667	0	1395

TRINITY_DN16323_c0_g2_i1	Glyma.18G250100.1	95.74468085	322	20	327	2650	2393	67	0	2338

TRINITY_DN16323_c0_g2_i1	Glyma.18G250100.2	95.74468085	322	20	327	2650	2348	22	0	2338

TRINITY_DN16082_c0_g1_i4	Glyma.07G016500.1	97.87234043	268	9	552	2875	2371	36	0	2697

TRINITY_DN15944_c2_g2_i5	Glyma.14G164600.2	97.9253112	167	14	204	2526	2335	1	0	3243

TRINITY_DN3649_c0_g2_i1	Glyma.07G104700.1	96.15384615	394	34	450	2753	3465	1138	0	1829

TRINITY_DN10754_c0_g1_i1	Glyma.18G068800.3	100	174	0	268	2607	2340	1	0	3358

TRINITY_DN10754_c0_g1_i1	Glyma.18G068800.1	100	174	0	268	2607	2340	1	0	3358

TRINITY_DN10754_c0_g2_i1	Glyma.18G068800.3	95.3125	174	0	268	2607	2340	1	0	3358

TRINITY_DN10754_c0_g2_i1	Glyma.18G068800.1	95.30791789	174	0	268	2607	2340	1	0	3358

TRINITY_DN10754_c0_g3_i1	Glyma.18G068800.3	95.28301887	174	0	268	2607	2340	1	0	3358

TRINITY_DN10754_c0_g3_i1	Glyma.18G068800.1	95.28936743	174	0	268	2607	2340	1	0	3358

TRINITY_DN16237_c0_g1_i10	Glyma.06G164900.1	100	172	24	116	2400	203	2532	0	2948

TRINITY_DN10754_c0_g1_i1	Glyma.08G335200.3	100	169	1	268	2607	2337	1	0	3365

TRINITY_DN10754_c0_g1_i1	Glyma.08G335200.4	100	169	1	268	2607	2337	1	0	3365

TRINITY_DN10754_c0_g1_i1	Glyma.08G335200.2	100	169	1	268	2607	2337	1	0	3365

TRINITY_DN10754_c0_g2_i1	Glyma.08G335200.3	95.29411765	169	1	268	2607	2337	1	0	3365

TRINITY_DN10754_c0_g2_i1	Glyma.08G335200.4	95.3125	169	1	268	2607	2337	1	0	3365

TRINITY_DN10754_c0_g2_i1	Glyma.08G335200.2	95.30201342	169	1	268	2607	2337	1	0	3365

TRINITY_DN10754_c0_g3_i1	Glyma.08G335200.3	95.29411765	169	1	268	2607	2337	1	0	3365

TRINITY_DN10754_c0_g3_i1	Glyma.08G335200.4	95.29411765	169	1	268	2607	2337	1	0	3365

TRINITY_DN10754_c0_g3_i1	Glyma.08G335200.2	95.29411765	169	1	268	2607	2337	1	0	3365

TRINITY_DN16106_c0_g3_i5	Glyma.04G070200.3	96.92307692	167	12	299	2620	2712	400	0	3105

TRINITY_DN16237_c0_g1_i10	Glyma.04G200600.1	100	155	22	116	2400	203	2532	0	3042

TRINITY_DN15560_c0_g1_i1	Glyma.03G163900.3	95.96774194	382	28	204	2522	1	2317	0	1932

TRINITY_DN15981_c0_g1_i1	Glyma.17G161300.1	95.38950715	377	8	1370	3702	2336	1	0	2172

TRINITY_DN15981_c0_g1_i23	Glyma.17G161300.1	100	377	8	1480	3812	2336	1	0	2172

TRINITY_DN15981_c0_g1_i4	Glyma.17G161300.1	97.5	377	8	1244	3576	2336	1	0	2172

TRINITY_DN15981_c0_g1_i7	Glyma.17G161300.1	99.29577465	377	8	1354	3686	2336	1	0	2172

TRINITY_DN15981_c0_g1_i1	Glyma.05G105200.1	100	372	7	1370	3702	2336	1	0	2200

TRINITY_DN15981_c0_g1_i23	Glyma.05G105200.1	100	372	7	1480	3812	2336	1	0	2200

TRINITY_DN15981_c0_g1_i4	Glyma.05G105200.1	95.97457627	372	7	1244	3576	2336	1	0	2200

TRINITY_DN15981_c0_g1_i7	Glyma.05G105200.1	98.08917197	372	7	1354	3686	2336	1	0	2200

TRINITY_DN8175_c0_g1_i1	Glyma.15G255000.2	100	186	8	1	2318	637	2975	0	3153

TRINITY_DN8175_c0_g1_i2	Glyma.15G255000.2	100	186	8	1	2318	637	2975	0	3153

TRINITY_DN11712_c0_g3_i1	Glyma.09G058000.1	97.95918367	183	22	103	2438	2297	1	0	3014

TRINITY_DN11712_c0_g3_i1	Glyma.09G058000.3	100	183	22	103	2438	2297	1	0	3014

TRINITY_DN11712_c0_g3_i1	Glyma.09G058000.2	100	183	22	103	2438	2297	1	0	3014

TRINITY_DN11712_c0_g3_i1	Glyma.09G058000.4	100	183	22	103	2438	2297	1	0	3014

TRINITY_DN13976_c0_g1_i5	Glyma.04G221600.7	96.28482972	134	1	268	2606	2373	38	0	3557

TRINITY_DN13976_c0_g1_i5	Glyma.04G221600.1	95.97069597	134	1	268	2606	2373	38	0	3557

TRINITY_DN13976_c0_g1_i5	Glyma.04G221600.6	99.06976744	134	1	268	2606	2373	38	0	3557

TRINITY_DN13976_c0_g1_i5	Glyma.04G221600.3	99.06976744	134	1	268	2606	2373	38	0	3557

TRINITY_DN13976_c0_g1_i5	Glyma.04G221600.4	100	134	1	268	2606	2373	38	0	3557

TRINITY_DN13976_c0_g1_i5	Glyma.04G221600.5	100	134	1	268	2606	2373	38	0	3557

TRINITY_DN13976_c0_g1_i5	Glyma.04G221600.2	100	134	1	268	2606	2373	38	0	3557

TRINITY_DN13976_c0_g1_i5	Glyma.04G221600.8	100	134	1	268	2606	2373	38	0	3557

TRINITY_DN13976_c0_g1_i5	Glyma.06G143800.8	97.5	133	1	268	2606	2373	38	0	3563

TRINITY_DN13976_c0_g1_i5	Glyma.06G143800.2	96.02649007	133	1	268	2606	2373	38	0	3563

TRINITY_DN13976_c0_g1_i5	Glyma.06G143800.3	99.35064935	133	1	268	2606	2373	38	0	3563

TRINITY_DN13976_c0_g1_i5	Glyma.06G143800.4	96.44549763	133	1	268	2606	2373	38	0	3563

TRINITY_DN13976_c0_g1_i5	Glyma.06G143800.7	96.44670051	133	1	268	2606	2373	38	0	3563

TRINITY_DN13976_c0_g1_i5	Glyma.06G143800.10	98.01980198	133	1	268	2606	2373	38	0	3563

TRINITY_DN13976_c0_g1_i5	Glyma.06G143800.1	98.67256637	133	1	268	2606	2373	38	0	3563

TRINITY_DN13976_c0_g1_i5	Glyma.06G143800.9	100	133	1	268	2606	2373	38	0	3563

TRINITY_DN13976_c0_g1_i5	Glyma.06G143800.5	95.6097561	133	1	268	2606	2373	38	0	3563

TRINITY_DN13976_c0_g1_i5	Glyma.06G143800.6	96.28482972	133	1	268	2606	2373	38	0	3563

TRINITY_DN14432_c0_g1_i4	Glyma.17G233000.1	97.46835443	126	9	888	3202	692	3027	0	3446

TRINITY_DN16168_c0_g1_i2	Glyma.09G169500.2	99.49494949	375	30	1857	4182	2279	5	0	1755

TRINITY_DN16168_c0_g1_i3	Glyma.09G169500.2	98.28571429	375	30	1290	3615	2279	5	0	1755

TRINITY_DN16168_c0_g1_i6	Glyma.09G169500.2	100	375	30	1761	4086	2279	5	0	1755

TRINITY_DN16230_c0_g1_i5	Glyma.U030200.2	97.62962963	276	26	1	2277	2345	25	0	2285

TRINITY_DN16082_c0_g1_i4	Glyma.08G201900.1	100	272	7	552	2875	2371	36	0	2708

TRINITY_DN10651_c0_g1_i1	Glyma.08G106400.5	99.27272727	239	16	499	2801	3260	927	0	2741

TRINITY_DN15706_c0_g1_i5	Glyma.08G180900.1	95.62043796	227	8	172	2505	1	2331	0	2988

TRINITY_DN15706_c0_g1_i5	Glyma.15G051500.1	100	216	9	172	2505	1	2331	0	3049

TRINITY_DN15706_c0_g1_i3	Glyma.08G180900.1	99.48717949	196	8	172	2505	1	2331	0	3160

TRINITY_DN15385_c0_g1_i2	Glyma.09G245000.1	95.97457627	184	5	1627	3957	2337	10	0	3188

TRINITY_DN15385_c0_g1_i2	Glyma.09G245000.2	96.12403101	184	5	1627	3957	2337	10	0	3188

TRINITY_DN15706_c0_g1_i3	Glyma.15G051500.1	99.48717949	184	9	172	2505	1	2331	0	3227

TRINITY_DN8358_c0_g2_i1	Glyma.02G212700.2	99	179	7	187	2512	1	2320	0	3133

TRINITY_DN8358_c0_g2_i1	Glyma.02G212700.1	100	179	7	187	2512	1	2320	0	3133

TRINITY_DN8358_c0_g2_i1	Glyma.02G212700.4	99.80430528	179	7	187	2512	1	2320	0	3133

TRINITY_DN8358_c0_g2_i1	Glyma.02G212700.3	100	179	9	187	2512	1	2317	0	3112

TRINITY_DN5703_c0_g1_i1	Glyma.15G102300.1	100	173	4	240	2571	2493	159	0	3301

TRINITY_DN9717_c0_g1_i1	Glyma.08G244900.12	100	158	3	715	3051	2322	1	0	3332

TRINITY_DN9717_c0_g1_i1	Glyma.08G244900.13	100	158	3	715	3051	2322	1	0	3332

TRINITY_DN15967_c0_g1_i1	Glyma.15G024100.1	100	328	8	120	2448	1	2332	0	2414

TRINITY_DN16082_c0_g1_i4	Glyma.08G201900.2	96.2962963	272	7	552	2874	2335	1	0	2706

TRINITY_DN16082_c0_g1_i14	Glyma.08G201900.1	95.6043956	249	6	581	2904	2371	36	0	2846

TRINITY_DN16082_c0_g1_i2	Glyma.08G201900.2	98.65771812	193	5	581	2914	2335	2	0	3208

TRINITY_DN37246_c0_g1_i1	Glyma.08G333200.1	98.92473118	173	0	1	2337	943	3279	0	3358

TRINITY_DN14432_c0_g1_i5	Glyma.U029000.5	97.4789916	140	4	798	3125	1	2331	0	3443

TRINITY_DN11782_c0_g1_i1	Glyma.01G009300.1	100	117	1	255	2582	2337	1	0	3609

TRINITY_DN15560_c0_g1_i1	Glyma.03G163900.1	95.96774194	382	27	204	2520	1	2314	0	1932

TRINITY_DN16230_c0_g1_i4	Glyma.U030200.2	99.34640523	283	25	1	2275	2345	11	0	2346

TRINITY_DN613_c0_g1_i1	Glyma.20G129200.1	100	269	17	188	2479	1	2328	0	2488

TRINITY_DN16082_c0_g1_i14	Glyma.08G201900.2	100	249	6	581	2903	2335	1	0	2844

TRINITY_DN16082_c0_g1_i6	Glyma.08G201900.2	95.03546099	220	3	552	2885	2335	2	0	3070

TRINITY_DN12691_c0_g1_i2	Glyma.12G029500.1	97.33333333	178	5	226	2556	2331	1	0	3264

TRINITY_DN11712_c0_g3_i1	Glyma.15G164100.3	98.95833333	177	5	103	2438	2321	1	0	3236

TRINITY_DN11712_c0_g3_i1	Glyma.15G164100.1	96.77419355	177	5	103	2438	2321	1	0	3236

TRINITY_DN11712_c0_g3_i1	Glyma.15G164100.2	96.77419355	177	5	103	2438	2321	1	0	3236

TRINITY_DN12691_c0_g1_i2	Glyma.11G104500.1	97.33333333	176	5	226	2556	2331	1	0	3275

TRINITY_DN15981_c0_g1_i1	Glyma.01G193600.2	95.74468085	157	1	1370	3702	2336	1	0	3424

TRINITY_DN15981_c0_g1_i1	Glyma.01G193600.1	100	157	1	1370	3702	2336	1	0	3424

TRINITY_DN15981_c0_g1_i23	Glyma.01G193600.2	95.96774194	157	1	1480	3812	2336	1	0	3424

TRINITY_DN15981_c0_g1_i23	Glyma.01G193600.1	97.60191847	157	1	1480	3812	2336	1	0	3424

TRINITY_DN15981_c0_g1_i4	Glyma.01G193600.2	100	157	1	1244	3576	2336	1	0	3424

TRINITY_DN15981_c0_g1_i4	Glyma.01G193600.1	99.15730337	157	1	1244	3576	2336	1	0	3424

TRINITY_DN15981_c0_g1_i7	Glyma.01G193600.2	100	157	1	1354	3686	2336	1	0	3424

TRINITY_DN15981_c0_g1_i7	Glyma.01G193600.1	95.49180328	157	1	1354	3686	2336	1	0	3424

TRINITY_DN13775_c0_g1_i1	Glyma.19G170300.5	95.98393574	157	14	146	2438	1	2326	0	3101

TRINITY_DN13775_c0_g1_i2	Glyma.19G170300.5	96.64179104	157	14	146	2438	1	2326	0	3101

TRINITY_DN10732_c0_g1_i1	Glyma.13G340300.1	100	129	2	226	2560	4077	1743	0	3587

TRINITY_DN14670_c0_g1_i3	Glyma.18G255000.2	100	102	3	531	2860	271	2606	0	3711

TRINITY_DN19771_c0_g1_i1	Glyma.10G034200.1	95.20661157	283	19	202	2512	1	2313	0	2447

TRINITY_DN15353_c0_g1_i2	Glyma.18G233500.2	98.7804878	213	2	292	2625	2334	1	0	3120

TRINITY_DN8358_c0_g2_i1	Glyma.14G180600.2	100	177	7	187	2512	1	2317	0	3157

TRINITY_DN8358_c0_g2_i1	Glyma.14G180600.1	100	177	7	187	2512	1	2317	0	3157

TRINITY_DN14721_c0_g1_i1	Glyma.11G165900.1	95.92920354	156	5	636	2957	558	2889	0	3345

TRINITY_DN11681_c0_g1_i2	Glyma.14G098900.1	100	381	8	552	2874	483	2811	0	2097

TRINITY_DN15517_c0_g2_i3	Glyma.14G025300.1	95.49071618	193	0	29	2362	2472	139	0	3241

TRINITY_DN11600_c0_g1_i2	Glyma.01G061500.1	95	191	27	244	2555	77	2388	0	2968

TRINITY_DN7182_c0_g1_i1	Glyma.20G164400.1	100	185	15	1	2299	3428	1108	0	2976

TRINITY_DN2792_c0_g1_i1	Glyma.04G233700.1	100	150	1	136	2468	1	2334	0	3472

TRINITY_DN2792_c0_g1_i1	Glyma.04G233700.2	100	150	1	136	2468	1	2334	0	3472

TRINITY_DN10010_c0_g1_i1	Glyma.19G000300.1	97.36842105	123	17	263	2563	2325	1	0	3358

TRINITY_DN16444_c0_g1_i9	Glyma.13G159800.1	99.00990099	377	20	514	2826	964	3288	0	2039

TRINITY_DN16444_c0_g1_i4	Glyma.13G159800.1	97.95918367	375	20	514	2826	964	3288	0	2050

TRINITY_DN16190_c0_g3_i2	Glyma.13G290700.1	98.7654321	298	0	836	3168	605	2937	0	2658

TRINITY_DN35988_c0_g1_i1	Glyma.05G097400.1	100	294	39	147	2426	2313	1	0	2207

TRINITY_DN16441_c4_g2_i3	Glyma.13G235300.1	97.27272727	281	25	280	2588	5268	2951	0	2499

TRINITY_DN16441_c4_g2_i3	Glyma.13G235300.3	97.27272727	281	25	280	2588	4488	2171	0	2499

TRINITY_DN16441_c4_g2_i3	Glyma.13G235300.2	97.27272727	281	25	280	2588	4488	2171	0	2499

TRINITY_DN16106_c0_g3_i5	Glyma.17G205700.1	95.87628866	276	12	299	2620	2766	442	0	2656

TRINITY_DN16106_c0_g3_i5	Glyma.17G205700.4	100	276	12	299	2620	2766	442	0	2656

TRINITY_DN11875_c0_g1_i1	Glyma.11G251900.1	100	255	31	737	3041	429	2733	0	2534

TRINITY_DN16012_c0_g1_i3	Glyma.17G010500.1	95.22091975	172	11	381	2702	1	2328	0	3253

TRINITY_DN16012_c0_g1_i3	Glyma.17G010500.2	97.56097561	172	11	381	2702	1	2328	0	3253

TRINITY_DN16012_c0_g1_i5	Glyma.17G010500.1	100	172	11	287	2608	1	2328	0	3253

TRINITY_DN16012_c0_g1_i5	Glyma.17G010500.2	100	172	11	287	2608	1	2328	0	3253

TRINITY_DN5625_c0_g2_i1	Glyma.03G202100.1	100	159	4	617	2940	2352	20	0	3369

TRINITY_DN5747_c0_g2_i1	Glyma.13G235600.1	99.40828402	113	0	283	2615	677	3009	0	3683

TRINITY_DN16379_c0_g1_i2	Glyma.19G183400.1	97.81420765	438	9	451	2777	2342	16	0	1816

TRINITY_DN15345_c0_g1_i1	Glyma.06G305000.2	97.17514124	391	29	709	3020	1	2315	0	1901

TRINITY_DN15345_c0_g1_i2	Glyma.06G305000.2	100	391	29	515	2826	1	2315	0	1901

TRINITY_DN15681_c0_g2_i3	Glyma.17G033700.1	100	224	17	287	2604	1	2315	0	2865

TRINITY_DN15718_c0_g1_i4	Glyma.04G217300.3	100	180	0	355	2686	2332	1	0	3310

TRINITY_DN15718_c0_g1_i4	Glyma.04G217300.1	100	180	0	355	2686	2332	1	0	3310

TRINITY_DN15718_c0_g1_i4	Glyma.04G217300.2	100	180	1	355	2686	2323	1	0	3251

TRINITY_DN37246_c0_g1_i1	Glyma.18G073600.1	97.5308642	166	0	1	2332	937	3268	0	3387

TRINITY_DN14670_c0_g1_i3	Glyma.18G255000.3	100	109	3	1091	3416	3	2334	0	3664

TRINITY_DN16379_c0_g1_i1	Glyma.06G180000.1	98.55072464	498	15	451	2774	2363	43	0	1435

TRINITY_DN15040_c0_g1_i1	Glyma.13G099300.1	95.99271403	361	33	784	3096	2569	287	0	1879

TRINITY_DN15956_c0_g2_i1	Glyma.03G142500.1	100	322	9	451	2775	2328	1	0	2462

TRINITY_DN15956_c0_g2_i3	Glyma.03G142500.1	97.4248927	322	9	451	2775	2328	1	0	2462

TRINITY_DN613_c0_g1_i1	Glyma.10G261600.1	100	263	29	188	2459	1	2320	0	2396

TRINITY_DN6245_c0_g1_i1	Glyma.11G242300.1	98.13084112	246	3	1	2327	2882	5211	0	2909

TRINITY_DN6245_c0_g1_i1	Glyma.11G242300.2	98.13084112	246	3	1	2327	2771	5100	0	2909

TRINITY_DN6245_c0_g1_i1	Glyma.11G242300.3	98.13084112	246	3	1	2327	1442	3771	0	2909

TRINITY_DN7182_c0_g1_i1	Glyma.10G227900.1	96.15931721	194	19	1	2299	3422	1108	0	2920

TRINITY_DN16441_c4_g2_i2	Glyma.13G235300.1	99.375	184	8	601	2925	5268	2944	0	3208

TRINITY_DN16441_c4_g2_i2	Glyma.13G235300.3	98.4962406	184	8	601	2925	4488	2164	0	3208

TRINITY_DN16441_c4_g2_i2	Glyma.13G235300.2	99.375	184	8	601	2925	4488	2164	0	3208

TRINITY_DN12877_c0_g3_i1	Glyma.10G280500.1	98.37837838	182	6	207	2525	2325	1	0	3181

TRINITY_DN16398_c1_g1_i2	Glyma.18G280500.3	100	161	4	255	2579	1	2331	0	3374

TRINITY_DN16398_c1_g1_i2	Glyma.18G280500.1	100	161	4	255	2579	1	2331	0	3374

TRINITY_DN16398_c1_g1_i2	Glyma.18G280500.2	100	161	4	255	2579	1	2331	0	3374

TRINITY_DN13088_c0_g1_i3	Glyma.20G156500.1	100	157	8	534	2849	4	2334	0	3338

TRINITY_DN16398_c1_g1_i2	Glyma.08G256300.1	100	155	2	255	2579	1	2331	0	3408

TRINITY_DN15754_c0_g1_i1	Glyma.08G178900.1	98.71794872	377	29	304	2628	3246	971	0	1832

TRINITY_DN15754_c0_g1_i1	Glyma.08G178900.2	98.71794872	377	29	304	2628	3114	839	0	1832

TRINITY_DN15040_c0_g1_i1	Glyma.13G099300.3	95.72192513	361	33	785	3096	2568	287	0	1877

TRINITY_DN16106_c0_g3_i5	Glyma.17G205700.6	98.61111111	275	16	299	2620	2739	442	0	2521

TRINITY_DN16106_c0_g3_i5	Glyma.17G205700.3	98.11320755	275	16	299	2620	2739	442	0	2521

TRINITY_DN16106_c0_g3_i5	Glyma.17G205700.5	100	271	20	299	2620	2724	442	0	2446

TRINITY_DN16106_c0_g3_i5	Glyma.17G205700.2	97.34513274	271	20	299	2620	2724	442	0	2446

TRINITY_DN16164_c1_g1_i4	Glyma.14G119800.1	100	221	28	1	2287	435	2748	0	2697

TRINITY_DN16164_c1_g1_i4	Glyma.14G119800.2	100	221	28	1	2287	435	2748	0	2697

TRINITY_DN16164_c1_g1_i6	Glyma.14G119800.1	97.40932642	217	28	1	2287	435	2748	0	2719

TRINITY_DN16164_c1_g1_i6	Glyma.14G119800.2	96.92307692	217	28	1	2287	435	2748	0	2719

TRINITY_DN15956_c0_g2_i1	Glyma.19G145400.1	100	321	3	451	2775	2328	1	0	2490

TRINITY_DN15956_c0_g2_i3	Glyma.19G145400.1	95.91836735	321	3	451	2775	2328	1	0	2490

TRINITY_DN15886_c3_g1_i1	Glyma.15G190200.4	100	232	8	1173	3497	1	2316	0	2905

TRINITY_DN15886_c3_g1_i3	Glyma.15G190200.4	95.37815126	232	8	1382	3706	1	2316	0	2905

TRINITY_DN15718_c0_g1_i4	Glyma.06G148500.1	100	177	0	358	2686	2329	1	0	3321

TRINITY_DN16164_c1_g1_i7	Glyma.14G119800.1	95.52238806	174	19	1	2293	435	2748	0	3007

TRINITY_DN16164_c1_g1_i7	Glyma.14G119800.2	96.95121951	174	19	1	2293	435	2748	0	3007

TRINITY_DN16164_c1_g1_i2	Glyma.14G119800.1	100	170	19	1	2293	435	2748	0	3029

TRINITY_DN16164_c1_g1_i2	Glyma.14G119800.2	100	170	19	1	2293	435	2748	0	3029

TRINITY_DN15888_c0_g3_i4	Glyma.02G269200.1	100	403	26	772	3085	813	3117	0	1827

TRINITY_DN10877_c0_g1_i2	Glyma.05G040300.1	100	270	19	11	2308	25	2349	0	2590

TRINITY_DN16012_c0_g1_i4	Glyma.17G010500.1	99.18032787	241	6	381	2699	1	2328	0	2905

TRINITY_DN16012_c0_g1_i4	Glyma.17G010500.2	99.18032787	241	6	381	2699	1	2328	0	2905

TRINITY_DN16012_c0_g1_i9	Glyma.17G010500.1	100	241	6	287	2605	1	2328	0	2905

TRINITY_DN16012_c0_g1_i9	Glyma.17G010500.2	95.5654102	241	6	287	2605	1	2328	0	2905

TRINITY_DN13612_c0_g2_i1	Glyma.08G027500.1	98.30508475	209	3	94	2418	1	2325	0	3103

TRINITY_DN16122_c1_g1_i1	Glyma.03G226600.1	100	206	26	78	2369	1	2316	0	2848

TRINITY_DN14117_c0_g1_i1	Glyma.02G034300.3	100	176	12	306	2630	2298	1	0	3110

TRINITY_DN14117_c0_g1_i1	Glyma.02G034300.2	97.34513274	176	12	306	2630	2298	1	0	3110

TRINITY_DN14117_c0_g1_i2	Glyma.02G034300.3	100	176	12	306	2630	2298	1	0	3110

TRINITY_DN14117_c0_g1_i2	Glyma.02G034300.2	100	176	12	306	2630	2298	1	0	3110

TRINITY_DN13758_c0_g1_i1	Glyma.14G219700.1	95.34883721	161	16	64	2372	1	2310	0	3168

TRINITY_DN14117_c0_g1_i1	Glyma.01G031400.4	98.52941176	158	11	306	2630	2307	1	0	3269

TRINITY_DN14117_c0_g1_i1	Glyma.01G031400.2	100	158	11	306	2630	2307	1	0	3269

TRINITY_DN14117_c0_g1_i1	Glyma.01G031400.3	97.77777778	158	11	306	2630	2307	1	0	3269

TRINITY_DN14117_c0_g1_i2	Glyma.01G031400.4	97.89473684	158	11	306	2630	2307	1	0	3269

TRINITY_DN14117_c0_g1_i2	Glyma.01G031400.2	96.50092081	158	11	306	2630	2307	1	0	3269

TRINITY_DN14117_c0_g1_i2	Glyma.01G031400.3	95.74468085	158	11	306	2630	2307	1	0	3269

TRINITY_DN15711_c0_g1_i4	Glyma.03G055900.1	98.30508475	153	1	1	2325	217	2544	0	3432

TRINITY_DN15711_c0_g1_i5	Glyma.03G055900.1	97.6744186	153	1	1	2325	217	2544	0	3432

TRINITY_DN15711_c0_g1_i4	Glyma.01G119500.3	99.44444444	153	1	1	2325	214	2541	0	3432

TRINITY_DN15711_c0_g1_i4	Glyma.01G119500.1	95.82366589	153	1	1	2325	214	2541	0	3432

TRINITY_DN15711_c0_g1_i5	Glyma.01G119500.3	100	153	1	1	2325	214	2541	0	3432

TRINITY_DN15711_c0_g1_i5	Glyma.01G119500.1	97.6744186	153	1	1	2325	214	2541	0	3432

TRINITY_DN14887_c0_g1_i1	Glyma.01G239000.3	100	106	2	301	2622	2328	1	0	3674

TRINITY_DN16379_c0_g1_i2	Glyma.06G180000.1	100	495	7	451	2774	2363	43	0	1496

TRINITY_DN18012_c0_g1_i1	Glyma.18G288700.1	98.18181818	430	36	109	2399	1	2309	0	1567

TRINITY_DN3642_c0_g1_i1	Glyma.04G022500.1	95.89041096	385	25	23	2329	1	2310	0	1925

TRINITY_DN3642_c0_g1_i1	Glyma.04G022300.1	98.76033058	338	20	23	2329	1	2313	0	2206

TRINITY_DN16436_c0_g2_i4	Glyma.11G131600.3	100	220	5	678	2990	1	2325	0	2976

TRINITY_DN8900_c0_g1_i1	Glyma.11G230700.1	95.93495935	218	13	3	2320	946	3252	0	2902

TRINITY_DN139_c0_g1_i1	Glyma.09G248100.1	96.72801636	204	26	443	2744	2298	1	0	2819

TRINITY_DN139_c0_g2_i1	Glyma.09G248100.1	96.71814672	204	26	444	2745	2298	1	0	2819

TRINITY_DN15808_c0_g1_i1	Glyma.17G077400.2	100	195	14	3136	5441	2933	5232	0	2907

TRINITY_DN15808_c0_g1_i2	Glyma.17G077400.2	97.4137931	195	14	3258	5563	2933	5232	0	2907

TRINITY_DN5607_c0_g1_i1	Glyma.09G043900.1	97.95918367	129	2	1	2326	1463	3788	0	3570

TRINITY_DN5607_c0_g1_i1	Glyma.09G043900.2	97.95918367	129	2	1	2326	1457	3782	0	3570

TRINITY_DN15736_c0_g1_i3	Glyma.05G198700.1	97.9498861	379	17	556	2875	388	2695	0	2041

TRINITY_DN16164_c1_g1_i2	Glyma.04G241900.2	95.61170213	239	33	2	2293	445	2736	0	2532

TRINITY_DN16164_c1_g1_i2	Glyma.04G241900.1	98.8372093	239	33	2	2293	445	2736	0	2532

TRINITY_DN16164_c1_g1_i7	Glyma.04G241900.2	97.26027397	234	33	2	2293	445	2736	0	2560

TRINITY_DN16164_c1_g1_i7	Glyma.04G241900.1	97.61904762	234	33	2	2293	445	2736	0	2560

TRINITY_DN14660_c1_g1_i4	Glyma.20G187500.2	95.08928571	234	43	436	2679	1	2295	0	2268

TRINITY_DN14660_c1_g1_i5	Glyma.20G187500.2	98.04270463	234	43	436	2679	1	2295	0	2268

TRINITY_DN15681_c0_g2_i3	Glyma.07G239600.1	100	226	11	287	2604	1	2312	0	2902

TRINITY_DN15956_c0_g2_i1	Glyma.10G091800.4	96.19205298	190	3	451	2775	2322	1	0	3210

TRINITY_DN15956_c0_g2_i1	Glyma.10G091800.3	100	190	3	451	2775	2322	1	0	3210

TRINITY_DN15956_c0_g2_i1	Glyma.10G091800.2	99.58847737	190	3	451	2775	2322	1	0	3210

TRINITY_DN15956_c0_g2_i1	Glyma.10G091800.1	100	190	3	451	2775	2322	1	0	3210

TRINITY_DN15956_c0_g2_i1	Glyma.10G091800.5	100	190	3	451	2775	2322	1	0	3210

TRINITY_DN15956_c0_g2_i3	Glyma.10G091800.4	95.85365854	190	3	451	2775	2322	1	0	3210

TRINITY_DN15956_c0_g2_i3	Glyma.10G091800.3	98.57142857	190	3	451	2775	2322	1	0	3210

TRINITY_DN15956_c0_g2_i3	Glyma.10G091800.2	95.97855228	190	3	451	2775	2322	1	0	3210

TRINITY_DN15956_c0_g2_i3	Glyma.10G091800.1	97.61904762	190	3	451	2775	2322	1	0	3210

TRINITY_DN15956_c0_g2_i3	Glyma.10G091800.5	97.61904762	190	3	451	2775	2322	1	0	3210

TRINITY_DN38193_c0_g1_i1	Glyma.08G227500.4	100	164	1	1	2323	243	2568	0	3367

TRINITY_DN38193_c0_g1_i1	Glyma.08G227500.5	100	164	1	1	2323	243	2568	0	3367

TRINITY_DN38193_c0_g1_i1	Glyma.08G227500.3	100	164	1	1	2323	243	2568	0	3367

TRINITY_DN38193_c0_g1_i1	Glyma.08G227500.2	100	164	1	1	2323	243	2568	0	3367

TRINITY_DN38193_c0_g1_i1	Glyma.08G227500.1	100	164	1	1	2323	243	2568	0	3367

TRINITY_DN37638_c0_g1_i1	Glyma.08G223000.1	100	129	2	275	2599	2577	255	0	3555

TRINITY_DN15888_c0_g3_i5	Glyma.14G047900.1	96.26865672	347	38	355	2664	801	3087	0	2028

TRINITY_DN15789_c0_g1_i1	Glyma.14G075800.1	97.5	203	9	1	2310	713	3016	0	2937

TRINITY_DN15789_c0_g1_i1	Glyma.14G075800.2	100	203	9	1	2310	713	3016	0	2937

TRINITY_DN10946_c0_g2_i1	Glyma.05G075800.1	98.57142857	189	6	482	2794	2316	1	0	3110

TRINITY_DN5192_c0_g2_i1	Glyma.09G268000.1	97.36842105	165	11	1	2314	309	2625	0	3256

TRINITY_DN5192_c0_g2_i1	Glyma.09G268000.2	96.31578947	165	12	1	2314	309	2622	0	3238

TRINITY_DN7482_c0_g1_i1	Glyma.13G027300.3	96.7816092	143	13	834	3151	2309	1	0	3352

TRINITY_DN14887_c0_g1_i1	Glyma.01G239000.2	95.65217391	106	1	301	2622	2325	1	0	3687

TRINITY_DN14887_c0_g1_i1	Glyma.11G004600.2	97.55301794	98	1	301	2622	2325	1	0	3731

TRINITY_DN15040_c0_g1_i3	Glyma.15G163900.2	100	355	47	597	2858	2758	455	0	1796

TRINITY_DN3642_c0_g1_i1	Glyma.06G022500.1	98.76160991	352	28	23	2325	1	2297	0	2032

TRINITY_DN15510_c0_g1_i2	Glyma.09G168500.1	98.48484848	341	23	1	2310	5	2305	0	2163

TRINITY_DN15510_c0_g1_i2	Glyma.09G168500.3	98.9010989	341	23	1	2310	5	2305	0	2163

TRINITY_DN13432_c0_g1_i1	Glyma.18G251800.2	96.70014347	320	29	252	2536	2306	1	0	2150

TRINITY_DN13432_c0_g1_i1	Glyma.18G251800.1	96.70103093	320	29	252	2536	2306	1	0	2150

TRINITY_DN15510_c0_g1_i1	Glyma.16G218200.1	100	303	35	288	2585	1	2301	0	2296

TRINITY_DN15510_c0_g1_i4	Glyma.16G218200.1	98.84393064	303	35	314	2611	1	2301	0	2296

TRINITY_DN16230_c0_g1_i3	Glyma.U030200.2	99.34102142	278	26	1	2264	2345	25	0	2344

TRINITY_DN16164_c1_g1_i2	Glyma.06G121500.1	100	242	29	1	2293	597	2886	0	2531

TRINITY_DN16164_c1_g1_i2	Glyma.06G121500.4	97.34219269	242	29	1	2293	573	2862	0	2531

TRINITY_DN16164_c1_g1_i2	Glyma.06G121500.2	100	242	29	1	2293	444	2733	0	2531

TRINITY_DN16164_c1_g1_i7	Glyma.06G121500.1	95.12195122	237	29	1	2293	597	2886	0	2558

TRINITY_DN16164_c1_g1_i7	Glyma.06G121500.4	95.88068182	237	29	1	2293	573	2862	0	2558

TRINITY_DN16164_c1_g1_i7	Glyma.06G121500.2	100	237	29	1	2293	444	2733	0	2558

TRINITY_DN14654_c0_g1_i7	Glyma.16G111200.3	100	212	13	1787	4103	2355	44	0	2994

TRINITY_DN14654_c0_g1_i7	Glyma.16G111200.2	97.18309859	212	13	1787	4103	2355	44	0	2994

TRINITY_DN14654_c0_g1_i7	Glyma.16G111200.1	99.57627119	212	13	1787	4103	2355	44	0	2994

TRINITY_DN2383_c0_g1_i1	Glyma.U020600.1	99.10714286	161	0	486	2809	3471	1148	0	3400

TRINITY_DN2383_c0_g1_i1	Glyma.U020600.2	99.10714286	161	0	486	2809	2907	584	0	3400

TRINITY_DN15681_c0_g2_i2	Glyma.17G033700.1	99.05660377	160	10	287	2589	1	2315	0	3212

TRINITY_DN14432_c0_g1_i4	Glyma.U029000.4	97.48953975	137	4	888	3202	692	3009	0	3435

TRINITY_DN14432_c0_g1_i4	Glyma.U029000.3	97.46835443	137	4	888	3202	692	3009	0	3435

TRINITY_DN14432_c0_g1_i4	Glyma.U029000.1	97.48822606	137	4	888	3202	692	3009	0	3435

TRINITY_DN14432_c0_g1_i4	Glyma.U029000.2	97.4789916	137	4	888	3202	692	3009	0	3435

TRINITY_DN14432_c0_g1_i4	Glyma.U029000.5	97.47706422	137	4	888	3202	14	2331	0	3435

TRINITY_DN5607_c0_g1_i1	Glyma.15G149200.1	97.95918367	131	2	1	2323	1583	3905	0	3554

TRINITY_DN5607_c0_g1_i1	Glyma.15G149200.2	97.95918367	131	2	1	2323	1325	3647	0	3554

TRINITY_DN16230_c0_g1_i1	Glyma.U030200.2	95.45454545	279	25	1	2264	2345	25	0	2350

TRINITY_DN16274_c0_g1_i1	Glyma.13G312300.2	99.14529915	222	4	1196	3514	1	2295	0	2854

TRINITY_DN14654_c0_g1_i7	Glyma.09G194900.1	97.95918367	202	11	1787	4103	2352	44	0	3042

TRINITY_DN8850_c0_g1_i1	Glyma.02G001000.3	99.29078014	170	6	279	2592	207	2514	0	3193

TRINITY_DN15510_c0_g1_i2	Glyma.16G218000.3	96.88385269	370	20	1	2310	5	2311	0	2063

TRINITY_DN15510_c0_g1_i2	Glyma.16G218000.1	97.77777778	370	20	1	2310	5	2311	0	2063

TRINITY_DN15510_c0_g1_i1	Glyma.09G168600.3	100	293	29	288	2585	1	2301	0	2374

TRINITY_DN15510_c0_g1_i1	Glyma.09G168600.4	96.95740365	293	29	288	2585	1	2301	0	2374

TRINITY_DN15510_c0_g1_i1	Glyma.09G168600.1	97.36842105	293	29	288	2585	1	2301	0	2374

TRINITY_DN15510_c0_g1_i4	Glyma.09G168600.3	98.48101266	293	29	314	2611	1	2301	0	2374

TRINITY_DN15510_c0_g1_i4	Glyma.09G168600.4	100	293	29	314	2611	1	2301	0	2374

TRINITY_DN15510_c0_g1_i4	Glyma.09G168600.1	95.97585513	293	29	314	2611	1	2301	0	2374

TRINITY_DN15517_c0_g2_i2	Glyma.14G025300.2	97.72296015	258	7	403	2718	3144	826	0	2800

TRINITY_DN13432_c0_g2_i1	Glyma.18G251800.2	96.7032967	242	20	252	2548	2306	1	0	2682

TRINITY_DN13432_c0_g2_i1	Glyma.18G251800.1	96.62447257	242	20	252	2548	2306	1	0	2682

TRINITY_DN15506_c0_g2_i11	Glyma.15G074200.3	100	208	16	1425	3734	325	2628	0	2942

TRINITY_DN15506_c0_g2_i12	Glyma.15G074200.3	97.5	208	16	1739	4048	325	2628	0	2942

TRINITY_DN15506_c0_g2_i13	Glyma.15G074200.3	100	208	16	1507	3816	325	2628	0	2942

TRINITY_DN15506_c0_g2_i3	Glyma.15G074200.3	95.08196721	208	16	1342	3651	325	2628	0	2942

TRINITY_DN15506_c0_g2_i5	Glyma.15G074200.3	100	208	16	1656	3965	325	2628	0	2942

TRINITY_DN15506_c0_g2_i6	Glyma.15G074200.3	100	208	16	1424	3733	325	2628	0	2942

TRINITY_DN13474_c0_g2_i4	Glyma.16G010400.1	96.93251534	207	20	97	2397	97	2388	0	2811

TRINITY_DN13474_c0_g2_i1	Glyma.16G010400.1	98	201	25	97	2379	97	2388	0	2728

TRINITY_DN13612_c0_g2_i1	Glyma.05G221000.1	98.58490566	197	6	94	2411	1	2318	0	3145

TRINITY_DN15421_c1_g3_i1	Glyma.12G234200.1	97.33840304	182	11	927	3237	2447	134	0	3157

TRINITY_DN16422_c0_g2_i3	Glyma.11G005500.1	98.7012987	176	19	1189	3498	780	3087	0	3145

TRINITY_DN14244_c0_g4_i1	Glyma.12G072700.3	96.1852861	247	10	609	2927	2334	22	0	2854

TRINITY_DN4160_c0_g2_i1	Glyma.10G201300.1	96.02446483	176	3	1	2317	234	2553	0	3278

TRINITY_DN14432_c0_g1_i4	Glyma.17G233000.2	97.4789916	136	8	906	3202	587	2907	0	3378

TRINITY_DN14432_c0_g1_i5	Glyma.17G233000.2	97.48427673	136	8	829	3125	587	2907	0	3378

TRINITY_DN15560_c0_g1_i2	Glyma.03G163900.3	100	378	28	291	2590	20	2317	0	1919

TRINITY_DN15718_c0_g1_i4	Glyma.05G186900.1	95.55035129	285	2	368	2686	2319	1	0	2693

TRINITY_DN15040_c0_g1_i2	Glyma.15G163900.2	100	263	37	784	3051	2758	455	0	2388

TRINITY_DN15510_c0_g1_i1	Glyma.16G218000.3	100	217	16	288	2592	1	2311	0	2928

TRINITY_DN15510_c0_g1_i1	Glyma.16G218000.1	100	217	16	288	2592	1	2311	0	2928

TRINITY_DN15510_c0_g1_i4	Glyma.16G218000.3	97.08029197	217	16	314	2618	1	2311	0	2928

TRINITY_DN15510_c0_g1_i4	Glyma.16G218000.1	98	217	16	314	2618	1	2311	0	2928

TRINITY_DN18060_c0_g1_i1	Glyma.14G000300.2	98.37962963	93	0	636	2955	1	2320	0	3770

TRINITY_DN13858_c0_g1_i1	Glyma.14G170900.1	100	336	19	18	2323	23	2331	0	2272

TRINITY_DN13432_c0_g1_i1	Glyma.09G242900.1	96.7032967	330	27	252	2536	2300	1	0	2111

TRINITY_DN6296_c0_g1_i1	Glyma.05G146400.1	100	266	3	365	2682	194	2508	0	2776

TRINITY_DN16122_c1_g1_i1	Glyma.19G223600.1	99.27611169	194	24	82	2364	8	2314	0	2898

TRINITY_DN15421_c1_g3_i1	Glyma.12G234200.7	95.71428571	182	10	927	3237	2444	134	0	3171

TRINITY_DN15421_c1_g3_i1	Glyma.12G234200.5	95.71428571	182	10	927	3237	2444	134	0	3171

TRINITY_DN15421_c1_g3_i1	Glyma.12G234200.6	95.71428571	182	10	927	3237	2444	134	0	3171

TRINITY_DN15421_c1_g3_i1	Glyma.12G234200.4	100	182	10	927	3237	2444	134	0	3171

TRINITY_DN15421_c1_g3_i1	Glyma.12G234200.3	100	182	10	927	3237	2444	134	0	3171

TRINITY_DN15421_c1_g3_i1	Glyma.12G234200.2	100	182	10	927	3237	2444	134	0	3171

TRINITY_DN12860_c0_g1_i1	Glyma.16G006000.1	99.32885906	169	11	495	2807	292	2583	0	3133

TRINITY_DN15754_c0_g1_i1	Glyma.05G117000.1	98.6013986	168	3	304	2622	3267	979	0	3158

TRINITY_DN14492_c1_g1_i2	Glyma.03G192300.2	100	167	1	537	2852	1	2319	0	3338

TRINITY_DN7482_c0_g1_i1	Glyma.13G027300.5	98.51851852	162	24	572	2883	2322	38	0	3120

TRINITY_DN12860_c0_g1_i1	Glyma.07G036600.1	99.32885906	158	5	495	2807	286	2592	0	3291

TRINITY_DN109_c0_g2_i1	Glyma.19G217400.1	97.56468798	156	10	260	2572	2307	1	0	3302

TRINITY_DN2784_c0_g2_i1	Glyma.08G092200.1	96.98795181	114	1	211	2523	3411	1093	0	3613

TRINITY_DN13432_c0_g2_i1	Glyma.09G242900.1	96.49923896	256	25	252	2548	2300	1	0	2610

TRINITY_DN188_c0_g1_i1	Glyma.06G045100.1	97.59036145	229	11	133	2448	8	2308	0	2889

TRINITY_DN188_c0_g1_i1	Glyma.04G044600.1	97.58064516	226	11	133	2448	8	2311	0	2926

TRINITY_DN16254_c0_g1_i5	Glyma.13G372400.2	95.74468085	207	5	1	2314	2315	2	0	3083

TRINITY_DN13448_c0_g1_i1	Glyma.08G100800.1	98.85057471	177	7	1	2310	27	2336	0	3197

TRINITY_DN13448_c0_g1_i1	Glyma.08G100800.2	98.06094183	174	18	1	2310	27	2315	0	3077

TRINITY_DN15681_c0_g2_i2	Glyma.07G239600.1	96.42857143	163	5	287	2589	1	2312	0	3241

TRINITY_DN15560_c0_g1_i2	Glyma.03G163900.1	100	378	27	291	2588	20	2314	0	1919

TRINITY_DN10708_c0_g1_i2	Glyma.17G211900.1	96.77419355	257	21	286	2598	2295	1	0	2687

TRINITY_DN16718_c0_g1_i1	Glyma.16G221700.1	100	215	5	270	2582	2333	21	0	3037

TRINITY_DN11634_c0_g1_i2	Glyma.10G083500.1	100	203	12	105	2405	1	2310	0	3005

TRINITY_DN2680_c0_g1_i1	Glyma.17G038700.1	99	192	5	1	2310	4229	6541	0	3144

TRINITY_DN2680_c0_g1_i1	Glyma.17G038700.2	99	192	5	1	2310	2723	5035	0	3144

TRINITY_DN6551_c0_g1_i1	Glyma.09G104000.1	98.8372093	189	6	1	2313	2341	4638	0	3083

TRINITY_DN6551_c0_g1_i1	Glyma.09G104000.4	97.18309859	187	6	1	2313	2341	4626	0	3016

TRINITY_DN12789_c0_g1_i1	Glyma.19G037200.2	96.15384615	138	1	220	2530	6324	4008	0	3476

TRINITY_DN15510_c0_g1_i1	Glyma.09G168400.1	97.50260146	356	25	270	2552	7	2310	0	2013

TRINITY_DN15510_c0_g1_i4	Glyma.09G168400.1	98.68421053	356	25	296	2578	7	2310	0	2013

TRINITY_DN15980_c0_g2_i3	Glyma.20G207700.3	95	332	41	3	2280	1030	3310	0	1965

TRINITY_DN16254_c0_g1_i10	Glyma.13G372400.2	100	266	6	1	2311	2315	2	0	2758

TRINITY_DN6245_c0_g1_i1	Glyma.18G014800.1	98.13084112	251	3	1	2312	2864	5178	0	2854

TRINITY_DN12686_c0_g1_i1	Glyma.16G205900.1	100	249	19	346	2630	1	2303	0	2614

TRINITY_DN12686_c0_g1_i2	Glyma.16G205900.1	100	249	19	367	2651	1	2303	0	2614

TRINITY_DN16033_c0_g1_i3	Glyma.03G238100.1	95.6043956	236	10	142	2452	2950	658	0	2789

TRINITY_DN14245_c0_g2_i2	Glyma.13G181700.4	95.65217391	177	5	2	2314	326	2635	0	3238

TRINITY_DN16106_c0_g3_i3	Glyma.04G070200.3	99.01234568	167	9	299	2593	2712	400	0	3197

TRINITY_DN14920_c0_g2_i1	Glyma.06G128600.1	100	148	2	195	2510	2313	1	0	3437

TRINITY_DN14920_c0_g2_i1	Glyma.04G236000.3	100	141	2	195	2510	2313	1	0	3476

TRINITY_DN14920_c0_g2_i1	Glyma.04G236000.1	100	141	2	195	2510	2313	1	0	3476

TRINITY_DN14920_c0_g2_i1	Glyma.04G236000.2	100	141	2	195	2510	2313	1	0	3476

TRINITY_DN16033_c0_g1_i4	Glyma.03G238100.1	97.93103448	195	8	142	2452	2950	658	0	3027

TRINITY_DN7273_c0_g1_i1	Glyma.20G179400.6	97.00374532	171	29	799	3077	2291	13	0	2863

TRINITY_DN7273_c0_g1_i2	Glyma.20G179400.6	98.48484848	168	34	799	3066	2291	13	0	2808

TRINITY_DN15874_c0_g1_i1	Glyma.11G253500.1	99.20634921	167	11	740	3052	2304	1	0	3265

TRINITY_DN4336_c0_g1_i1	Glyma.16G108900.1	98.91598916	417	37	172	2454	188	2482	0	1640

TRINITY_DN10946_c0_g2_i1	Glyma.16G059700.2	96.93877551	401	25	484	2783	2302	9	0	1832

TRINITY_DN10946_c0_g2_i1	Glyma.16G059700.3	96.93877551	401	25	484	2783	2302	9	0	1832

TRINITY_DN10946_c0_g2_i1	Glyma.16G059700.4	96.94117647	401	25	484	2783	2302	9	0	1832

TRINITY_DN10946_c0_g2_i1	Glyma.16G059700.5	97.6119403	401	25	484	2783	2302	9	0	1832

TRINITY_DN14999_c0_g1_i2	Glyma.13G155100.1	98.08917197	316	24	46	2334	1	2295	0	2239

TRINITY_DN14999_c0_g1_i1	Glyma.13G155100.1	98.11320755	308	32	46	2321	1	2295	0	2198

TRINITY_DN15836_c0_g2_i1	Glyma.14G197100.2	100	243	25	80	2343	32	2337	0	2553

TRINITY_DN15836_c0_g2_i1	Glyma.14G197100.3	98.48484848	243	25	80	2343	32	2337	0	2553

TRINITY_DN11463_c0_g1_i1	Glyma.13G213400.1	100	226	29	159	2427	29	2331	0	2660

TRINITY_DN4211_c0_g1_i1	Glyma.18G259000.1	100	224	34	328	2592	1	2304	0	2651

TRINITY_DN16718_c0_g1_i1	Glyma.16G221700.2	100	216	3	270	2582	2330	21	0	3044

TRINITY_DN15984_c0_g1_i1	Glyma.12G021600.2	100	216	5	778	3077	2308	1	0	2948

TRINITY_DN15510_c0_g1_i1	Glyma.09G168500.1	98.94736842	214	10	288	2592	1	2305	0	2972

TRINITY_DN15510_c0_g1_i1	Glyma.09G168500.3	98.94736842	214	10	288	2592	1	2305	0	2972

TRINITY_DN15510_c0_g1_i4	Glyma.09G168500.1	95.2293578	214	10	314	2618	1	2305	0	2972

TRINITY_DN15510_c0_g1_i4	Glyma.09G168500.3	98.63945578	214	10	314	2618	1	2305	0	2972

TRINITY_DN16164_c1_g1_i6	Glyma.04G241900.2	96.64179104	201	25	2	2287	445	2736	0	2837

TRINITY_DN16164_c1_g1_i6	Glyma.04G241900.1	100	201	25	2	2287	445	2736	0	2837

TRINITY_DN16164_c1_g1_i4	Glyma.04G241900.2	97.2972973	196	25	2	2287	445	2736	0	2865

TRINITY_DN16164_c1_g1_i4	Glyma.04G241900.1	100	196	25	2	2287	445	2736	0	2865

TRINITY_DN15836_c0_g2_i2	Glyma.14G197100.2	100	192	25	80	2343	32	2337	0	2835

TRINITY_DN15836_c0_g2_i2	Glyma.14G197100.3	98.73417722	192	25	80	2343	32	2337	0	2835

TRINITY_DN15874_c0_g1_i1	Glyma.18G003500.1	98.66666667	167	10	740	3052	2301	1	0	3258

TRINITY_DN2784_c0_g2_i1	Glyma.05G136900.1	99.24623116	112	2	211	2523	3522	1210	0	3640

TRINITY_DN15751_c0_g1_i14	Glyma.06G172300.2	95.85253456	320	30	890	3174	1	2288	0	2156

TRINITY_DN15751_c0_g1_i14	Glyma.06G172300.3	96.96202532	320	30	890	3174	1	2288	0	2156

TRINITY_DN15751_c0_g1_i14	Glyma.06G172300.4	100	320	30	890	3174	1	2288	0	2156

TRINITY_DN15751_c0_g1_i14	Glyma.06G172300.1	100	320	30	890	3174	1	2288	0	2156

TRINITY_DN15751_c0_g1_i4	Glyma.06G172300.2	100	320	30	890	3174	1	2288	0	2156

TRINITY_DN15751_c0_g1_i4	Glyma.06G172300.3	100	320	30	890	3174	1	2288	0	2156

TRINITY_DN15751_c0_g1_i4	Glyma.06G172300.4	100	320	30	890	3174	1	2288	0	2156

TRINITY_DN15751_c0_g1_i4	Glyma.06G172300.1	100	320	30	890	3174	1	2288	0	2156

TRINITY_DN15944_c2_g2_i3	Glyma.06G109500.1	95.65217391	244	17	275	2567	2370	69	0	2719

TRINITY_DN16718_c0_g1_i1	Glyma.03G001200.1	100	208	0	270	2582	2333	21	0	3120

TRINITY_DN11634_c0_g1_i2	Glyma.02G193700.1	97.00598802	201	7	105	2405	1	2310	0	3061

TRINITY_DN6551_c0_g1_i1	Glyma.02G168900.1	98.8372093	185	4	1	2313	2353	4647	0	3131

TRINITY_DN6551_c0_g1_i1	Glyma.02G168900.2	98.8372093	185	4	1	2313	2212	4506	0	3131

TRINITY_DN6551_c0_g1_i1	Glyma.02G168900.3	98.82352941	185	5	1	2313	2353	4635	0	3053

TRINITY_DN6551_c0_g1_i1	Glyma.02G168900.4	98.8179669	184	5	1	2313	2353	4641	0	3097

TRINITY_DN16054_c1_g1_i4	Glyma.10G168700.1	95.45454545	125	0	375	2687	2967	655	0	3579

TRINITY_DN10010_c0_g1_i1	Glyma.05G000200.3	97.84946237	125	8	263	2563	2307	1	0	3463

TRINITY_DN10010_c0_g1_i1	Glyma.05G000200.1	95.45454545	125	8	263	2563	2307	1	0	3463

TRINITY_DN10010_c0_g1_i1	Glyma.05G000200.2	97.36842105	125	9	263	2563	2301	1	0	3424

TRINITY_DN16054_c1_g1_i4	Glyma.20G221000.1	100	113	0	375	2687	2967	655	0	3646

TRINITY_DN15754_c0_g1_i1	Glyma.15G053600.1	98.71794872	357	24	304	2604	3267	994	0	1975

TRINITY_DN16164_c1_g1_i6	Glyma.06G121500.1	98.98989899	203	21	1	2287	597	2886	0	2841

TRINITY_DN16164_c1_g1_i6	Glyma.06G121500.4	100	203	21	1	2287	573	2862	0	2841

TRINITY_DN16164_c1_g1_i6	Glyma.06G121500.2	100	203	21	1	2287	444	2733	0	2841

TRINITY_DN16164_c1_g1_i4	Glyma.06G121500.1	96.19047619	198	21	1	2287	597	2886	0	2868

TRINITY_DN16164_c1_g1_i4	Glyma.06G121500.4	100	198	21	1	2287	573	2862	0	2868

TRINITY_DN16164_c1_g1_i4	Glyma.06G121500.2	98.37398374	198	21	1	2287	444	2733	0	2868

TRINITY_DN13448_c0_g1_i1	Glyma.05G144400.4	98.85057471	188	3	1	2311	27	2331	0	3177

TRINITY_DN15633_c0_g1_i2	Glyma.08G016400.1	98.51301115	138	6	140	2446	1	2307	0	3441

TRINITY_DN14887_c0_g1_i2	Glyma.01G239000.1	96.77419355	104	1	436	2744	2312	1	0	3674

TRINITY_DN14887_c0_g1_i2	Glyma.11G004600.1	96	95	1	436	2744	2312	1	0	3723

TRINITY_DN10681_c1_g2_i1	Glyma.20G173600.1	98.71794872	470	57	4	2265	79	2350	0	1096

TRINITY_DN14999_c0_g1_i2	Glyma.17G075700.1	98.11320755	333	30	46	2331	1	2289	0	2119

TRINITY_DN14999_c0_g1_i1	Glyma.17G075700.1	98.11133201	327	37	46	2318	1	2289	0	2069

TRINITY_DN15655_c2_g1_i3	Glyma.02G239600.4	99.05660377	286	19	374	2658	1	2304	0	2470

TRINITY_DN12686_c0_g1_i1	Glyma.09G155400.1	100	250	18	346	2628	1	2298	0	2617

TRINITY_DN12686_c0_g1_i2	Glyma.09G155400.1	100	250	18	367	2649	1	2298	0	2617

TRINITY_DN10651_c0_g1_i1	Glyma.08G106400.4	97.8021978	241	8	499	2801	3233	927	0	2856

TRINITY_DN15751_c0_g1_i3	Glyma.06G172300.2	95.3125	240	25	890	3177	1	2288	0	2641

TRINITY_DN15751_c0_g1_i3	Glyma.06G172300.3	100	240	25	890	3177	1	2288	0	2641

TRINITY_DN15751_c0_g1_i3	Glyma.06G172300.4	100	240	25	890	3177	1	2288	0	2641

TRINITY_DN15751_c0_g1_i3	Glyma.06G172300.1	98.18181818	240	25	890	3177	1	2288	0	2641

TRINITY_DN15751_c0_g1_i7	Glyma.06G172300.2	96.7032967	240	25	890	3177	1	2288	0	2641

TRINITY_DN15751_c0_g1_i7	Glyma.06G172300.3	96.84210526	240	25	890	3177	1	2288	0	2641

TRINITY_DN15751_c0_g1_i7	Glyma.06G172300.4	96.84385382	240	25	890	3177	1	2288	0	2641

TRINITY_DN15751_c0_g1_i7	Glyma.06G172300.1	98.21428571	240	25	890	3177	1	2288	0	2641

TRINITY_DN13448_c0_g1_i1	Glyma.05G144400.3	96.59863946	188	3	1	2310	27	2330	0	3175

TRINITY_DN13448_c0_g1_i1	Glyma.05G144400.1	99.18032787	188	3	1	2310	27	2330	0	3175

TRINITY_DN13448_c0_g1_i6	Glyma.05G144400.4	99.37106918	188	3	1	2310	27	2330	0	3175

TRINITY_DN16379_c0_g2_i4	Glyma.12G177100.1	97.8021978	413	12	365	2667	2342	40	0	1888

TRINITY_DN13421_c0_g1_i1	Glyma.18G008300.1	100	280	18	448	2745	2307	6	0	2586

TRINITY_DN13421_c0_g1_i1	Glyma.18G008300.2	100	280	18	448	2745	2307	6	0	2586

TRINITY_DN15944_c2_g2_i3	Glyma.06G109500.2	100	244	16	275	2567	2367	69	0	2734

TRINITY_DN16130_c2_g6_i12	Glyma.10G075300.2	96.2962963	192	0	665	2974	481	2790	0	3203

TRINITY_DN16130_c2_g6_i2	Glyma.10G075300.2	98.7012987	192	0	338	2647	481	2790	0	3203

TRINITY_DN16130_c2_g6_i12	Glyma.02G201300.2	95.41547278	182	0	665	2974	478	2787	0	3258

TRINITY_DN16130_c2_g6_i12	Glyma.02G201300.4	95.41666667	182	0	665	2974	478	2787	0	3258

TRINITY_DN16130_c2_g6_i12	Glyma.02G201300.1	97.4025974	182	0	665	2974	478	2787	0	3258

TRINITY_DN16130_c2_g6_i2	Glyma.02G201300.2	100	182	0	338	2647	478	2787	0	3258

TRINITY_DN16130_c2_g6_i2	Glyma.02G201300.4	95.3125	182	0	338	2647	478	2787	0	3258

TRINITY_DN16130_c2_g6_i2	Glyma.02G201300.1	96.51695692	182	0	338	2647	478	2787	0	3258

TRINITY_DN15633_c0_g1_i2	Glyma.05G209700.1	99.49494949	135	3	140	2446	1	2307	0	3480

TRINITY_DN16130_c2_g6_i1	Glyma.10G075300.2	100	134	0	338	2647	481	2790	0	3524

TRINITY_DN16130_c2_g6_i11	Glyma.10G075300.2	97.22222222	134	0	665	2974	481	2790	0	3524

TRINITY_DN2483_c0_g1_i1	Glyma.05G023300.1	98.75	132	2	1	2309	1954	4262	0	3522

TRINITY_DN37638_c0_g1_i1	Glyma.07G003600.1	100	131	0	275	2584	2310	1	0	3541

TRINITY_DN37638_c0_g1_i1	Glyma.08G223000.3	99.07407407	128	1	275	2584	2307	1	0	3537

TRINITY_DN37638_c0_g1_i1	Glyma.08G223000.2	99.07407407	128	1	275	2584	2307	1	0	3537

TRINITY_DN16130_c2_g6_i1	Glyma.02G201300.2	100	124	0	338	2647	478	2787	0	3579

TRINITY_DN16130_c2_g6_i1	Glyma.02G201300.4	100	124	0	338	2647	478	2787	0	3579

TRINITY_DN16130_c2_g6_i1	Glyma.02G201300.1	100	124	0	338	2647	478	2787	0	3579

TRINITY_DN16130_c2_g6_i11	Glyma.02G201300.2	100	124	0	665	2974	478	2787	0	3579

TRINITY_DN16130_c2_g6_i11	Glyma.02G201300.4	97.22222222	124	0	665	2974	478	2787	0	3579

TRINITY_DN16130_c2_g6_i11	Glyma.02G201300.1	97.22222222	124	0	665	2974	478	2787	0	3579

TRINITY_DN16323_c0_g2_i1	Glyma.09G243700.1	95.74468085	405	16	351	2650	2324	28	0	1884

TRINITY_DN12815_c1_g1_i3	Glyma.08G318200.1	97	327	35	714	3009	2574	321	0	2013

TRINITY_DN12815_c1_g1_i3	Glyma.08G318200.4	96.9924812	327	35	714	3009	2574	321	0	2013

TRINITY_DN35988_c0_g1_i1	Glyma.17G167600.1	100	312	30	147	2426	2286	1	0	2200

TRINITY_DN16353_c0_g1_i1	Glyma.19G064000.1	100	263	10	749	3050	330	2631	0	2717

TRINITY_DN16353_c0_g1_i8	Glyma.19G064000.1	99.36708861	263	10	667	2968	330	2631	0	2717

TRINITY_DN10651_c0_g1_i1	Glyma.08G106400.3	95.6043956	241	8	501	2801	3231	927	0	2852

TRINITY_DN14622_c0_g1_i7	Glyma.20G210700.3	100	152	6	274	2573	86	2385	0	3306

TRINITY_DN9844_c0_g1_i3	Glyma.11G068300.3	99.86091794	147	1	275	2583	2306	1	0	3430

TRINITY_DN16379_c0_g1_i1	Glyma.04G186000.1	98.55072464	502	11	451	2753	2363	64	0	1397

TRINITY_DN14680_c0_g2_i3	Glyma.13G134500.1	95.74468085	411	41	43	2322	353	2620	0	1546

TRINITY_DN15560_c0_g1_i1	Glyma.03G163900.4	95.96774194	378	30	300	2588	1	2283	0	1884

TRINITY_DN15560_c0_g1_i2	Glyma.03G163900.4	100	378	30	368	2656	1	2283	0	1884

TRINITY_DN11827_c0_g1_i2	Glyma.15G024300.1	97.22222222	353	44	372	2641	11	2286	0	1855

TRINITY_DN16012_c0_g1_i3	Glyma.13G002200.1	98.5915493	293	16	406	2702	26	2325	0	2516

TRINITY_DN16012_c0_g1_i5	Glyma.13G002200.1	100	293	16	312	2608	26	2325	0	2516

TRINITY_DN16012_c0_g1_i3	Glyma.20G066800.1	95	293	18	406	2702	26	2325	0	2516

TRINITY_DN16012_c0_g1_i5	Glyma.20G066800.1	100	293	18	312	2608	26	2325	0	2516

TRINITY_DN12815_c1_g1_i4	Glyma.08G318200.1	97	261	33	714	3009	2574	321	0	2390

TRINITY_DN12815_c1_g1_i4	Glyma.08G318200.4	97	261	33	714	3009	2574	321	0	2390

TRINITY_DN15099_c0_g1_i2	Glyma.13G261200.1	95.65217391	244	12	518	2797	1	2305	0	2710

TRINITY_DN6417_c0_g1_i1	Glyma.04G045100.1	95.83333333	204	11	215	2519	120	2410	0	3003

TRINITY_DN6417_c0_g1_i1	Glyma.04G045100.3	97.40932642	204	11	215	2519	120	2410	0	3003

TRINITY_DN14718_c0_g2_i2	Glyma.04G156200.1	99.06759907	198	6	561	2862	5568	3267	0	3088

TRINITY_DN15984_c0_g1_i2	Glyma.12G021600.2	95.90163934	178	2	778	3074	2308	1	0	3205

TRINITY_DN14206_c1_g1_i1	Glyma.19G201100.1	100	176	4	193	2491	594	2898	0	3210

TRINITY_DN10477_c0_g2_i2	Glyma.05G153400.1	100	171	19	46	2334	1	2286	0	3049

TRINITY_DN13783_c0_g1_i2	Glyma.09G002400.3	97.6635514	169	8	386	2674	2352	52	0	3158

TRINITY_DN15510_c0_g1_i2	Glyma.16G218200.1	96.3592233	159	8	1	2303	5	2301	0	3278

TRINITY_DN16168_c0_g1_i1	Glyma.09G169500.2	97.26027397	152	8	1285	3592	2284	1	0	3265

TRINITY_DN16168_c0_g1_i5	Glyma.09G169500.2	100	152	8	1852	4159	2284	1	0	3265

TRINITY_DN16168_c0_g1_i8	Glyma.09G169500.2	96.42857143	152	8	1756	4063	2284	1	0	3265

TRINITY_DN18012_c0_g1_i1	Glyma.02G093400.1	98.18181818	394	31	125	2399	26	2318	0	1781

TRINITY_DN10814_c0_g1_i1	Glyma.13G335700.1	99.29328622	390	19	247	2532	164	2464	0	1925

TRINITY_DN10814_c0_g1_i1	Glyma.13G335700.2	99.14529915	390	19	247	2532	164	2464	0	1925

TRINITY_DN37246_c0_g1_i1	Glyma.02G302600.1	96.77419355	367	7	1	2292	934	3237	0	2111

TRINITY_DN15517_c0_g2_i2	Glyma.14G025300.3	96.16580311	256	7	403	2703	2304	1	0	2784

TRINITY_DN18012_c0_g1_i1	Glyma.03G019000.1	95.09803922	196	11	109	2400	1	2301	0	3038

TRINITY_DN18012_c0_g1_i1	Glyma.03G019000.2	95.09803922	196	11	109	2400	1	2301	0	3038

TRINITY_DN14206_c1_g1_i1	Glyma.03G203700.1	100	174	8	195	2491	485	2787	0	3206

TRINITY_DN10477_c0_g2_i2	Glyma.08G111100.1	97.70114943	173	19	46	2334	1	2292	0	3088

TRINITY_DN35705_c0_g1_i1	Glyma.04G094500.1	98.73417722	116	2	231	2531	1	2307	0	3579

TRINITY_DN14887_c0_g1_i2	Glyma.01G239000.2	97	104	1	441	2744	2307	1	0	3664

TRINITY_DN14887_c0_g1_i2	Glyma.01G239000.3	98.42519685	104	1	441	2744	2307	1	0	3664

TRINITY_DN14887_c0_g1_i2	Glyma.11G004600.2	96.77419355	95	1	441	2744	2307	1	0	3714

TRINITY_DN16379_c0_g1_i2	Glyma.04G186000.1	100	495	7	451	2753	2363	64	0	1458

TRINITY_DN14680_c0_g2_i3	Glyma.10G046400.1	98.30508475	411	38	43	2322	353	2623	0	1587

TRINITY_DN14680_c0_g2_i3	Glyma.10G046400.2	96.6226138	411	38	43	2322	353	2623	0	1587

TRINITY_DN12815_c1_g1_i3	Glyma.08G318200.3	96.97368421	327	33	714	3009	2565	321	0	1988

TRINITY_DN12815_c1_g1_i3	Glyma.08G318200.2	96.98492462	327	33	714	3009	2565	321	0	1988

TRINITY_DN16106_c0_g3_i3	Glyma.17G205700.6	97.48603352	276	12	299	2593	2739	442	0	2606

TRINITY_DN16106_c0_g3_i3	Glyma.17G205700.3	97.91666667	276	12	299	2593	2739	442	0	2606

TRINITY_DN16106_c0_g3_i3	Glyma.17G205700.5	95.57195572	272	18	299	2593	2724	442	0	2532

TRINITY_DN16106_c0_g3_i3	Glyma.17G205700.2	97.1042471	272	18	299	2593	2724	442	0	2532

TRINITY_DN14933_c1_g1_i1	Glyma.13G101300.1	100	226	21	418	2688	745	3027	0	2632

TRINITY_DN15385_c0_g1_i4	Glyma.03G107700.1	98.55072464	184	18	213	2489	2280	1	0	2883

TRINITY_DN15845_c0_g1_i3	Glyma.01G236300.3	99.12280702	168	26	157	2387	5	2280	0	2649

TRINITY_DN9398_c0_g3_i1	Glyma.09G023200.1	95.96774194	167	18	259	2530	1	2299	0	3068

TRINITY_DN9398_c0_g3_i1	Glyma.09G023200.2	95.96774194	167	18	259	2530	1	2299	0	3068

TRINITY_DN16012_c0_g1_i3	Glyma.07G263300.1	97.5308642	155	13	381	2675	1	2301	0	3297

TRINITY_DN16012_c0_g1_i5	Glyma.07G263300.1	100	155	13	287	2581	1	2301	0	3297

TRINITY_DN12717_c0_g1_i1	Glyma.03G244500.10	99.70760234	151	0	89	2394	14	2319	0	3422

TRINITY_DN15510_c0_g1_i2	Glyma.09G168600.3	96.20253165	145	5	1	2303	5	2301	0	3378

TRINITY_DN15510_c0_g1_i2	Glyma.09G168600.4	96.8	145	5	1	2303	5	2301	0	3378

TRINITY_DN15510_c0_g1_i2	Glyma.09G168600.1	96.35761589	145	5	1	2303	5	2301	0	3378

TRINITY_DN6023_c0_g1_i1	Glyma.10G255700.2	98.07692308	140	4	906	3208	1	2294	0	3386

TRINITY_DN4336_c0_g1_i1	Glyma.16G076300.1	100	410	31	171	2454	192	2473	0	1701

TRINITY_DN11827_c0_g1_i2	Glyma.15G024300.2	97.22222222	354	41	372	2641	11	2283	0	1862

TRINITY_DN13421_c0_g1_i1	Glyma.11G249000.1	97.61904762	265	16	448	2742	2295	1	0	2660

TRINITY_DN12815_c1_g1_i4	Glyma.08G318200.3	96.97841727	261	31	714	3009	2565	321	0	2364

TRINITY_DN12815_c1_g1_i4	Glyma.08G318200.2	96.98113208	261	31	714	3009	2565	321	0	2364

TRINITY_DN6417_c0_g1_i1	Glyma.04G045100.2	96.82539683	204	11	215	2516	120	2407	0	2998

TRINITY_DN7273_c0_g1_i1	Glyma.10G211600.3	95.80152672	158	1	318	2622	2325	27	0	3343

TRINITY_DN7273_c0_g1_i2	Glyma.10G211600.3	98.48484848	155	7	318	2611	2325	27	0	3288

TRINITY_DN12815_c1_g1_i2	Glyma.08G318200.1	96.98275862	305	32	714	3006	2574	321	0	2170

TRINITY_DN12815_c1_g1_i2	Glyma.08G318200.4	97	305	32	714	3006	2574	321	0	2170

TRINITY_DN14604_c0_g1_i1	Glyma.01G155900.1	100	198	1	215	2518	1	2292	0	3081

TRINITY_DN15655_c2_g1_i2	Glyma.02G239600.4	100	149	9	374	2655	1	2304	0	3288

TRINITY_DN15944_c2_g2_i5	Glyma.06G109500.1	98.1595092	285	20	199	2479	2358	69	0	2447

TRINITY_DN12815_c1_g1_i1	Glyma.08G318200.1	96.9924812	239	30	714	3006	2574	321	0	2547

TRINITY_DN12815_c1_g1_i1	Glyma.08G318200.4	97	239	30	714	3006	2574	321	0	2547

TRINITY_DN16012_c0_g1_i4	Glyma.20G066800.1	99.18032787	236	12	406	2699	26	2325	0	2868

TRINITY_DN16012_c0_g1_i9	Glyma.20G066800.1	100	236	12	312	2605	26	2325	0	2868

TRINITY_DN16012_c0_g1_i4	Glyma.13G002200.1	99.18032787	234	11	406	2699	26	2325	0	2880

TRINITY_DN16012_c0_g1_i9	Glyma.13G002200.1	95.5654102	234	11	312	2605	26	2325	0	2880

TRINITY_DN6117_c0_g2_i1	Glyma.09G084300.8	97.76119403	152	3	2	2301	1069	3368	0	3373

TRINITY_DN6117_c0_g2_i1	Glyma.09G084300.7	100	152	3	2	2301	769	3068	0	3373

TRINITY_DN12717_c0_g1_i1	Glyma.03G244500.1	97.08029197	151	0	92	2394	98	2400	0	3417

TRINITY_DN5192_c0_g2_i1	Glyma.18G221900.1	98.14814815	151	13	1	2295	330	2621	0	3293

TRINITY_DN5192_c0_g2_i1	Glyma.18G221900.2	100	151	14	1	2295	330	2618	0	3275

TRINITY_DN7609_c0_g1_i4	Glyma.20G074200.2	95.91836735	149	13	306	2595	2783	485	0	3317

TRINITY_DN7609_c0_g1_i4	Glyma.20G074200.1	96.47058824	149	13	306	2595	2783	485	0	3317

TRINITY_DN7609_c0_g1_i4	Glyma.20G074200.3	95.9798995	148	14	306	2595	2780	485	0	3304

TRINITY_DN7609_c0_g1_i1	Glyma.20G074200.2	100	148	21	306	2575	2783	485	0	3193

TRINITY_DN7609_c0_g1_i1	Glyma.20G074200.1	99.07407407	148	21	306	2575	2783	485	0	3193

TRINITY_DN7609_c0_g1_i1	Glyma.20G074200.3	96.875	147	22	306	2575	2780	485	0	3181

TRINITY_DN15385_c0_g1_i4	Glyma.07G119000.2	98.48053181	191	14	213	2489	2289	1	0	2948

TRINITY_DN15385_c0_g1_i4	Glyma.07G119000.1	100	191	14	213	2489	2289	1	0	2948

TRINITY_DN15385_c0_g1_i4	Glyma.07G119000.3	98.55072464	191	14	213	2489	2289	1	0	2948

TRINITY_DN8273_c0_g1_i1	Glyma.09G169600.1	95.51724138	166	2	1	2302	1212	3507	0	3293

TRINITY_DN16130_c2_g6_i13	Glyma.19G196000.1	99.25925926	164	1	664	2965	618	2916	0	3323

TRINITY_DN16130_c2_g6_i3	Glyma.19G196000.1	96.55172414	164	1	337	2638	618	2916	0	3323

TRINITY_DN16130_c2_g6_i13	Glyma.19G196000.3	100	164	1	664	2965	456	2754	0	3323

TRINITY_DN16130_c2_g6_i13	Glyma.19G196000.4	98.7012987	164	1	664	2965	456	2754	0	3323

TRINITY_DN16130_c2_g6_i3	Glyma.19G196000.3	98.52941176	164	1	337	2638	456	2754	0	3323

TRINITY_DN16130_c2_g6_i3	Glyma.19G196000.4	100	164	1	337	2638	456	2754	0	3323

TRINITY_DN15912_c1_g1_i2	Glyma.13G357100.1	95.51282051	132	16	390	2669	2289	1	0	3293

TRINITY_DN11782_c0_g1_i1	Glyma.16G077300.1	100	124	0	255	2556	2337	36	0	3565

TRINITY_DN12815_c1_g1_i2	Glyma.08G318200.3	97	305	30	714	3006	2565	321	0	2146

TRINITY_DN12815_c1_g1_i2	Glyma.08G318200.2	97	305	30	714	3006	2565	321	0	2146

TRINITY_DN3624_c0_g1_i1	Glyma.17G224700.1	100	233	19	1	2273	987	3274	0	2693

TRINITY_DN16012_c0_g1_i4	Glyma.07G263300.1	99.18032787	227	5	381	2673	1	2301	0	2940

TRINITY_DN16012_c0_g1_i9	Glyma.07G263300.1	100	227	5	287	2579	1	2301	0	2940

TRINITY_DN15877_c0_g1_i1	Glyma.14G047800.1	95.1951952	198	4	47	2340	2364	65	0	3101

TRINITY_DN13421_c0_g1_i1	Glyma.08G103100.1	100	194	8	448	2742	2301	4	0	3116

TRINITY_DN13421_c0_g1_i1	Glyma.08G103100.2	99.28571429	194	8	448	2742	2301	4	0	3116

TRINITY_DN6837_c0_g1_i1	Glyma.20G133900.1	98.61111111	164	5	3	2297	86	2383	0	3282

TRINITY_DN12627_c0_g1_i2	Glyma.13G317500.4	99.43181818	117	6	313	2609	1	2282	0	3452

TRINITY_DN12627_c0_g1_i2	Glyma.13G317500.3	99.43181818	117	6	313	2609	1	2282	0	3452

TRINITY_DN35705_c0_g1_i1	Glyma.06G096300.2	100	106	0	231	2531	1	2301	0	3663

TRINITY_DN35705_c0_g1_i1	Glyma.06G096300.1	95.18518519	106	0	231	2531	1	2301	0	3663

TRINITY_DN13055_c0_g1_i1	Glyma.13G212900.10	97.22222222	376	16	71	2350	53	2344	0	1984

TRINITY_DN15944_c2_g2_i5	Glyma.06G109500.2	99.31034483	285	19	199	2479	2355	69	0	2462

TRINITY_DN12815_c1_g1_i1	Glyma.08G318200.3	97	239	28	714	3006	2565	321	0	2523

TRINITY_DN12815_c1_g1_i1	Glyma.08G318200.2	96.9924812	239	28	714	3006	2565	321	0	2523

TRINITY_DN14933_c1_g1_i1	Glyma.17G058400.1	100	232	22	421	2688	757	3033	0	2606

TRINITY_DN13432_c0_g1_i1	Glyma.07G114400.3	96.69811321	168	10	251	2536	2292	1	0	3175

TRINITY_DN13432_c0_g1_i1	Glyma.07G114400.1	96.69811321	168	10	251	2536	2292	1	0	3175

TRINITY_DN13432_c0_g1_i1	Glyma.07G114400.2	96.69811321	168	10	251	2536	2292	1	0	3175

TRINITY_DN16130_c2_g6_i13	Glyma.03G198100.1	100	162	0	664	2963	459	2758	0	3350

TRINITY_DN16130_c2_g6_i13	Glyma.03G198100.2	99.47089947	162	0	664	2963	459	2758	0	3350

TRINITY_DN16130_c2_g6_i3	Glyma.03G198100.1	100	162	0	337	2636	459	2758	0	3350

TRINITY_DN16130_c2_g6_i3	Glyma.03G198100.2	96.49122807	162	0	337	2636	459	2758	0	3350

TRINITY_DN6117_c0_g2_i1	Glyma.09G084300.6	100	152	1	2	2301	1069	3365	0	3386

TRINITY_DN6117_c0_g2_i1	Glyma.09G084300.5	100	152	1	2	2301	769	3065	0	3386

TRINITY_DN13991_c0_g1_i1	Glyma.13G111500.1	100	451	26	133	2424	2265	1	0	1482

TRINITY_DN13991_c0_g1_i3	Glyma.13G111500.1	99.4011976	446	33	133	2411	2265	1	0	1426

TRINITY_DN15655_c2_g1_i3	Glyma.02G239600.2	95.23809524	285	18	382	2658	1	2292	0	2479

TRINITY_DN15655_c2_g1_i3	Glyma.02G239600.3	98.16513761	285	18	382	2658	1	2292	0	2479

TRINITY_DN16422_c0_g2_i3	Glyma.01G238300.1	100	188	10	1206	3498	177	2469	0	3127

TRINITY_DN13432_c0_g1_i1	Glyma.03G111200.1	96.93251534	169	8	251	2536	2292	1	0	3181

TRINITY_DN13432_c0_g1_i1	Glyma.03G111200.2	96.93251534	169	8	251	2536	2292	1	0	3181

TRINITY_DN13991_c0_g1_i1	Glyma.17G048100.1	100	454	24	133	2424	2265	1	0	1476

TRINITY_DN13991_c0_g1_i3	Glyma.17G048100.1	96.77419355	450	32	133	2411	2265	1	0	1415

TRINITY_DN15967_c0_g1_i1	Glyma.13G350400.1	97.36842105	307	20	120	2404	7	2294	0	2394

TRINITY_DN15501_c0_g2_i1	Glyma.13G162800.1	100	296	4	306	2601	2296	1	0	2579

TRINITY_DN9796_c0_g1_i2	Glyma.01G207400.3	99.21259843	202	5	899	3187	2294	1	0	3040

TRINITY_DN10946_c0_g2_i1	Glyma.19G074900.2	98.71794872	194	4	482	2773	2289	1	0	3072

TRINITY_DN10946_c0_g2_i1	Glyma.19G074900.1	100	194	4	482	2773	2289	1	0	3072

TRINITY_DN7609_c0_g1_i4	Glyma.10G122100.1	100	160	14	318	2598	2741	449	0	3216

TRINITY_DN7609_c0_g1_i1	Glyma.10G122100.1	97.05882353	160	15	318	2578	2741	449	0	3086

TRINITY_DN14423_c0_g1_i1	Glyma.10G272800.2	98.91304348	154	1	181	2478	1	2297	0	3384

TRINITY_DN13088_c0_g1_i3	Glyma.10G238100.1	100	150	8	564	2849	1	2298	0	3336

TRINITY_DN15891_c0_g1_i1	Glyma.16G048100.2	100	145	0	955	3252	1	2298	0	3441

TRINITY_DN15501_c0_g2_i2	Glyma.13G162800.1	99.35483871	134	0	375	2672	2298	1	0	3502

TRINITY_DN15516_c0_g1_i1	Glyma.12G198800.1	95.70637119	484	41	234	2504	57	2327	0	1225

TRINITY_DN14604_c0_g1_i1	Glyma.09G195100.2	100	388	14	228	2518	14	2289	0	1917

TRINITY_DN12172_c0_g1_i1	Glyma.06G266500.1	98.7012987	322	18	199	2490	2343	76	0	2239

TRINITY_DN10413_c0_g1_i2	Glyma.04G038200.2	100	276	52	1239	3489	2224	1	0	1943

TRINITY_DN10413_c0_g1_i2	Glyma.04G038200.1	96.41025641	276	52	1239	3489	2224	1	0	1943

TRINITY_DN10413_c0_g1_i4	Glyma.04G038200.2	96.49122807	276	52	1032	3282	2224	1	0	1943

TRINITY_DN10413_c0_g1_i4	Glyma.04G038200.1	96.49122807	276	52	1032	3282	2224	1	0	1943

TRINITY_DN3624_c0_g1_i1	Glyma.14G100200.1	95	248	16	5	2273	991	3277	0	2623

TRINITY_DN3624_c0_g1_i1	Glyma.14G100200.2	98.00498753	248	16	5	2273	991	3277	0	2623

TRINITY_DN13991_c0_g1_i1	Glyma.09G070900.1	100	222	6	133	2424	2280	1	0	2870

TRINITY_DN13991_c0_g1_i3	Glyma.09G070900.1	100	219	16	133	2411	2280	1	0	2802

TRINITY_DN13421_c0_g1_i1	Glyma.05G146300.2	97.19298246	187	5	448	2742	2295	4	0	3160

TRINITY_DN13421_c0_g1_i1	Glyma.05G146300.1	95.19230769	187	5	448	2742	2295	4	0	3160

TRINITY_DN3879_c0_g1_i1	Glyma.13G161800.1	100	185	2	68	2363	1	2296	0	3205

TRINITY_DN3879_c0_g1_i1	Glyma.13G161800.2	100	185	2	68	2363	1	2296	0	3205

TRINITY_DN2658_c0_g1_i1	Glyma.07G232200.2	96.61835749	170	28	243	2497	2547	273	0	2887

TRINITY_DN15981_c0_g1_i15	Glyma.11G048300.3	97.49103943	143	3	791	3081	2297	1	0	3411

TRINITY_DN15981_c0_g1_i6	Glyma.11G048300.3	95.95959596	143	3	791	3081	2297	1	0	3411

TRINITY_DN6136_c0_g1_i1	Glyma.19G170500.1	100	143	6	258	2549	2361	67	0	3404

TRINITY_DN6136_c0_g2_i1	Glyma.19G170500.1	100	143	6	257	2548	2361	67	0	3404

TRINITY_DN15954_c0_g1_i4	Glyma.19G114500.2	95.50561798	180	8	287	2578	1	2292	0	3192

TRINITY_DN15954_c0_g1_i4	Glyma.19G114500.1	95.50561798	180	8	287	2578	1	2292	0	3192

TRINITY_DN15954_c0_g1_i8	Glyma.19G114500.2	95.44072948	180	8	147	2438	1	2292	0	3192

TRINITY_DN15954_c0_g1_i8	Glyma.19G114500.1	95.44159544	180	8	147	2438	1	2292	0	3192

TRINITY_DN18012_c0_g1_i1	Glyma.07G080500.1	95.98930481	179	4	109	2400	1	2292	0	3197

TRINITY_DN15167_c0_g2_i1	Glyma.18G225800.2	96.69421488	147	15	348	2619	1	2287	0	3212

TRINITY_DN15167_c0_g2_i1	Glyma.18G225800.1	100	147	15	348	2619	1	2287	0	3212

TRINITY_DN15167_c0_g2_i1	Glyma.18G225800.3	100	147	15	348	2619	1	2287	0	3212

TRINITY_DN15167_c0_g2_i2	Glyma.18G225800.2	96.42857143	147	15	550	2821	1	2287	0	3212

TRINITY_DN15167_c0_g2_i2	Glyma.18G225800.1	96.42857143	147	15	550	2821	1	2287	0	3212

TRINITY_DN15167_c0_g2_i2	Glyma.18G225800.3	100	147	15	550	2821	1	2287	0	3212

TRINITY_DN12145_c0_g1_i1	Glyma.16G044400.2	98.27586207	144	1	400	2695	2952	660	0	3422

TRINITY_DN16352_c0_g2_i3	Glyma.08G194700.1	100	441	50	2340	4600	2585	316	0	1413

TRINITY_DN13448_c0_g1_i1	Glyma.18G011000.1	100	315	2	17	2310	43	2336	0	2481

TRINITY_DN16069_c0_g1_i2	Glyma.08G156800.1	95.36231884	293	15	213	2497	130	2417	0	2505

TRINITY_DN9861_c0_g1_i1	Glyma.11G067500.1	96.55172414	212	12	268	2541	2292	1	0	2909

TRINITY_DN9861_c0_g1_i1	Glyma.01G175300.1	95.38461538	204	16	268	2541	2289	1	0	2933

TRINITY_DN14545_c0_g3_i1	Glyma.06G094900.1	100	160	7	100	2382	1	2289	0	3236

TRINITY_DN14545_c0_g3_i2	Glyma.06G094900.1	95.19230769	160	7	100	2382	1	2289	0	3236

TRINITY_DN12717_c0_g1_i1	Glyma.19G241800.1	97.46192893	155	1	100	2394	1	2292	0	3360

TRINITY_DN14545_c0_g3_i1	Glyma.04G093100.2	100	152	4	100	2382	1	2289	0	3280

TRINITY_DN14545_c0_g3_i1	Glyma.04G093100.3	100	152	4	100	2382	1	2289	0	3280

TRINITY_DN14545_c0_g3_i1	Glyma.04G093100.4	98.48484848	152	4	100	2382	1	2289	0	3280

TRINITY_DN14545_c0_g3_i1	Glyma.04G093100.1	99.53379953	152	4	100	2382	1	2289	0	3280

TRINITY_DN14545_c0_g3_i2	Glyma.04G093100.2	100	152	4	100	2382	1	2289	0	3280

TRINITY_DN14545_c0_g3_i2	Glyma.04G093100.3	100	152	4	100	2382	1	2289	0	3280

TRINITY_DN14545_c0_g3_i2	Glyma.04G093100.4	98.85057471	152	4	100	2382	1	2289	0	3280

TRINITY_DN14545_c0_g3_i2	Glyma.04G093100.1	95.19230769	152	4	100	2382	1	2289	0	3280

TRINITY_DN12717_c0_g1_i1	Glyma.03G244500.12	97.08222812	151	0	100	2394	1	2295	0	3402

TRINITY_DN12717_c0_g1_i1	Glyma.03G244500.11	98.48484848	151	0	100	2394	1	2295	0	3402

TRINITY_DN12717_c0_g1_i1	Glyma.03G244500.13	100	151	0	100	2394	1	2295	0	3402

TRINITY_DN12717_c0_g1_i1	Glyma.03G244500.9	100	151	0	100	2394	1	2295	0	3402

TRINITY_DN12717_c0_g1_i1	Glyma.03G244500.7	100	151	0	100	2394	1	2295	0	3402

TRINITY_DN12717_c0_g1_i1	Glyma.03G244500.8	99.07407407	151	0	100	2394	1	2295	0	3402

TRINITY_DN12717_c0_g1_i1	Glyma.03G244500.3	99.07407407	151	0	100	2394	1	2295	0	3402

TRINITY_DN12717_c0_g1_i1	Glyma.03G244500.4	98.98989899	151	0	100	2394	1	2295	0	3402

TRINITY_DN12717_c0_g1_i1	Glyma.03G244500.5	98.98989899	151	0	100	2394	1	2295	0	3402

TRINITY_DN12717_c0_g1_i1	Glyma.03G244500.6	95.83333333	151	0	100	2394	1	2295	0	3402

TRINITY_DN12717_c0_g1_i1	Glyma.03G244500.2	95.83333333	151	0	100	2394	1	2295	0	3402

TRINITY_DN12717_c0_g1_i1	Glyma.19G241300.1	100	147	0	100	2394	1	2295	0	3424

TRINITY_DN12717_c0_g1_i1	Glyma.19G241300.2	98.15100154	147	1	100	2394	1	2289	0	3386

TRINITY_DN12717_c0_g1_i1	Glyma.03G243800.1	97.72727273	142	0	100	2394	1	2295	0	3452

TRINITY_DN12717_c0_g1_i1	Glyma.03G243800.3	98.67172676	142	0	100	2394	1	2295	0	3452

TRINITY_DN6136_c0_g1_i1	Glyma.03G169200.1	97.01492537	138	2	258	2549	2361	67	0	3454

TRINITY_DN6136_c0_g2_i1	Glyma.03G169200.1	100	138	2	257	2548	2361	67	0	3454

TRINITY_DN15954_c0_g1_i8	Glyma.13G028400.1	98.88888889	431	23	142	2423	110	2391	0	1694

TRINITY_DN13448_c0_g1_i1	Glyma.11G246200.9	100	325	0	17	2310	43	2336	0	2436

TRINITY_DN13448_c0_g1_i1	Glyma.11G246200.1	95.52238806	325	0	17	2310	43	2336	0	2436

TRINITY_DN15954_c0_g1_i4	Glyma.16G038300.4	95.50561798	185	4	287	2578	1	2292	0	3186

TRINITY_DN15954_c0_g1_i4	Glyma.16G038300.1	95.50561798	185	4	287	2578	1	2292	0	3186

TRINITY_DN15954_c0_g1_i4	Glyma.16G038300.3	98.69565217	185	4	287	2578	1	2292	0	3186

TRINITY_DN15954_c0_g1_i8	Glyma.16G038300.4	95.89041096	185	4	147	2438	1	2292	0	3186

TRINITY_DN15954_c0_g1_i8	Glyma.16G038300.1	98.88888889	185	4	147	2438	1	2292	0	3186

TRINITY_DN15954_c0_g1_i8	Glyma.16G038300.3	98.88888889	185	4	147	2438	1	2292	0	3186

TRINITY_DN11578_c0_g1_i1	Glyma.19G216000.2	98.80952381	185	8	50	2332	568	2851	0	3075

TRINITY_DN11578_c0_g1_i2	Glyma.19G216000.2	98.40954274	185	8	50	2332	568	2851	0	3075

TRINITY_DN15549_c0_g1_i2	Glyma.05G247000.5	100	180	13	391	2654	2262	1	0	2839

TRINITY_DN15549_c0_g1_i4	Glyma.05G247000.5	98.24561404	180	13	391	2654	2262	1	0	2839

TRINITY_DN15549_c0_g1_i2	Glyma.08G055400.4	97.05882353	178	15	391	2654	2265	1	0	2868

TRINITY_DN15549_c0_g1_i4	Glyma.08G055400.4	98.24561404	178	15	391	2654	2265	1	0	2868

TRINITY_DN8202_c0_g1_i1	Glyma.17G052800.2	97.91666667	157	7	52	2343	1	2283	0	3282

TRINITY_DN8202_c0_g1_i1	Glyma.17G052800.1	98.7012987	157	7	52	2343	1	2283	0	3282

TRINITY_DN145_c0_g1_i1	Glyma.19G222700.3	100	144	2	538	2812	1	2294	0	3315

TRINITY_DN15954_c0_g1_i5	Glyma.16G038300.4	99.3006993	143	4	147	2438	1	2292	0	3419

TRINITY_DN15954_c0_g1_i5	Glyma.16G038300.1	97.95081967	143	4	147	2438	1	2292	0	3419

TRINITY_DN15954_c0_g1_i5	Glyma.16G038300.3	98.61111111	143	4	147	2438	1	2292	0	3419

TRINITY_DN15954_c0_g1_i9	Glyma.16G038300.4	100	143	4	287	2578	1	2292	0	3419

TRINITY_DN15954_c0_g1_i9	Glyma.16G038300.1	98.81656805	143	4	287	2578	1	2292	0	3419

TRINITY_DN15954_c0_g1_i9	Glyma.16G038300.3	97.5	143	4	287	2578	1	2292	0	3419

TRINITY_DN37264_c0_g1_i1	Glyma.17G244300.3	100	133	2	571	2863	3378	1086	0	3487

TRINITY_DN15602_c0_g1_i1	Glyma.19G223400.3	96.68508287	107	2	1507	3800	6	2295	0	3618

TRINITY_DN15954_c0_g1_i8	Glyma.20G055900.1	100	449	21	142	2423	110	2391	0	1605

TRINITY_DN16130_c2_g6_i13	Glyma.10G075300.2	96.49122807	212	5	665	2952	481	2771	0	3014

TRINITY_DN16130_c2_g6_i3	Glyma.10G075300.2	97.84946237	212	5	338	2625	481	2771	0	3014

TRINITY_DN16130_c2_g6_i13	Glyma.02G201300.2	100	203	4	665	2952	478	2768	0	3064

TRINITY_DN16130_c2_g6_i13	Glyma.02G201300.4	96.42857143	203	4	665	2952	478	2768	0	3064

TRINITY_DN16130_c2_g6_i13	Glyma.02G201300.1	96.42857143	203	4	665	2952	478	2768	0	3064

TRINITY_DN16130_c2_g6_i3	Glyma.02G201300.2	97.19626168	203	4	338	2625	478	2768	0	3064

TRINITY_DN16130_c2_g6_i3	Glyma.02G201300.4	97.84946237	203	4	338	2625	478	2768	0	3064

TRINITY_DN16130_c2_g6_i3	Glyma.02G201300.1	97.84946237	203	4	338	2625	478	2768	0	3064

TRINITY_DN8273_c0_g1_i1	Glyma.16G219200.1	95.83333333	170	0	1	2293	1218	3510	0	3293

TRINITY_DN8273_c0_g1_i1	Glyma.16G219200.3	100	170	0	1	2293	681	2973	0	3293

TRINITY_DN15954_c0_g1_i5	Glyma.19G114500.2	100	141	2	147	2438	1	2292	0	3441

TRINITY_DN15954_c0_g1_i5	Glyma.19G114500.1	100	141	2	147	2438	1	2292	0	3441

TRINITY_DN15954_c0_g1_i9	Glyma.19G114500.2	97.35099338	141	2	287	2578	1	2292	0	3441

TRINITY_DN15954_c0_g1_i9	Glyma.19G114500.1	96.34146341	141	2	287	2578	1	2292	0	3441

TRINITY_DN37264_c0_g1_i1	Glyma.14G081100.2	100	127	0	571	2863	2628	336	0	3531

TRINITY_DN13440_c0_g1_i1	Glyma.09G070000.1	96.20535714	413	22	202	2478	2313	40	0	1731

TRINITY_DN13055_c0_g1_i1	Glyma.13G212900.6	98.71794872	399	18	359	2629	161	2443	0	1829

TRINITY_DN15954_c0_g1_i5	Glyma.05G090100.1	96.15384615	295	3	147	2437	1	2290	0	2579

TRINITY_DN15954_c0_g1_i5	Glyma.05G090100.2	100	295	3	147	2437	1	2290	0	2579

TRINITY_DN15954_c0_g1_i9	Glyma.05G090100.1	97.43589744	295	3	287	2577	1	2290	0	2579

TRINITY_DN15954_c0_g1_i9	Glyma.05G090100.2	97.84946237	295	3	287	2577	1	2290	0	2579

TRINITY_DN6691_c0_g2_i1	Glyma.02G055500.1	98.72881356	246	11	366	2648	1	2283	0	2754

TRINITY_DN16130_c2_g6_i1	Glyma.19G196000.1	96.49122807	243	2	337	2628	618	2903	0	2848

TRINITY_DN16130_c2_g6_i11	Glyma.19G196000.1	98.18181818	243	2	664	2955	618	2903	0	2848

TRINITY_DN16130_c2_g6_i1	Glyma.19G196000.3	99.02912621	243	2	337	2628	456	2741	0	2848

TRINITY_DN16130_c2_g6_i1	Glyma.19G196000.4	96.49122807	243	2	337	2628	456	2741	0	2848

TRINITY_DN16130_c2_g6_i11	Glyma.19G196000.3	96.6395112	243	2	664	2955	456	2741	0	2848

TRINITY_DN16130_c2_g6_i11	Glyma.19G196000.4	95.09803922	243	2	664	2955	456	2741	0	2848

TRINITY_DN16130_c2_g6_i1	Glyma.03G198100.1	100	241	1	337	2628	459	2747	0	2878

TRINITY_DN16130_c2_g6_i1	Glyma.03G198100.2	99.02912621	241	1	337	2628	459	2747	0	2878

TRINITY_DN16130_c2_g6_i11	Glyma.03G198100.1	96.63608563	241	1	664	2955	459	2747	0	2878

TRINITY_DN16130_c2_g6_i11	Glyma.03G198100.2	100	241	1	664	2955	459	2747	0	2878

TRINITY_DN14660_c1_g1_i4	Glyma.20G187500.1	96.06299213	233	40	436	2648	1	2261	0	2231

TRINITY_DN14660_c1_g1_i5	Glyma.20G187500.1	97.6744186	233	40	436	2648	1	2261	0	2231

TRINITY_DN15954_c0_g1_i4	Glyma.05G090100.1	97.14285714	219	3	287	2577	1	2290	0	3000

TRINITY_DN15954_c0_g1_i4	Glyma.05G090100.2	100	219	3	287	2577	1	2290	0	3000

TRINITY_DN15954_c0_g1_i8	Glyma.05G090100.1	98.88888889	219	3	147	2437	1	2290	0	3000

TRINITY_DN15954_c0_g1_i8	Glyma.05G090100.2	98.88888889	219	3	147	2437	1	2290	0	3000

TRINITY_DN2154_c0_g1_i1	Glyma.08G225800.1	100	203	5	198	2486	2289	1	0	3070

TRINITY_DN14604_c0_g1_i5	Glyma.01G155900.1	96.04519774	198	0	215	2506	1	2292	0	3136

TRINITY_DN16130_c2_g6_i12	Glyma.19G196000.1	95.91836735	190	2	664	2955	618	2903	0	3142

TRINITY_DN16130_c2_g6_i2	Glyma.19G196000.1	96.04810997	190	2	337	2628	618	2903	0	3142

TRINITY_DN16130_c2_g6_i12	Glyma.19G196000.3	95.74468085	190	2	664	2955	456	2741	0	3142

TRINITY_DN16130_c2_g6_i12	Glyma.19G196000.4	97.68339768	190	2	664	2955	456	2741	0	3142

TRINITY_DN16130_c2_g6_i2	Glyma.19G196000.3	96.04519774	190	2	337	2628	456	2741	0	3142

TRINITY_DN16130_c2_g6_i2	Glyma.19G196000.4	96.04519774	190	2	337	2628	456	2741	0	3142

TRINITY_DN16130_c2_g6_i12	Glyma.03G198100.1	98.10874704	187	1	664	2955	459	2747	0	3177

TRINITY_DN16130_c2_g6_i12	Glyma.03G198100.2	100	187	1	664	2955	459	2747	0	3177

TRINITY_DN16130_c2_g6_i2	Glyma.03G198100.1	96.35036496	187	1	337	2628	459	2747	0	3177

TRINITY_DN16130_c2_g6_i2	Glyma.03G198100.2	95.09043928	187	1	337	2628	459	2747	0	3177

TRINITY_DN8850_c0_g1_i1	Glyma.02G001000.2	100	169	6	279	2561	207	2483	0	3142

TRINITY_DN15405_c0_g1_i1	Glyma.06G323700.1	98.71794872	152	1	663	2951	3114	823	0	3371

TRINITY_DN12172_c0_g1_i1	Glyma.13G286400.2	100	149	1	199	2490	2289	1	0	3387

TRINITY_DN12172_c0_g1_i1	Glyma.13G286400.1	98.98989899	149	1	199	2490	2289	1	0	3387

TRINITY_DN12172_c0_g1_i1	Glyma.12G215200.1	100	148	1	199	2490	2289	1	0	3393

TRINITY_DN15655_c2_g1_i2	Glyma.02G239600.2	96.91629956	148	8	382	2655	1	2292	0	3297

TRINITY_DN15655_c2_g1_i2	Glyma.02G239600.3	100	148	8	382	2655	1	2292	0	3297

TRINITY_DN6023_c0_g1_i1	Glyma.10G255700.4	98.07692308	139	4	920	3208	30	2309	0	3365

TRINITY_DN10760_c0_g1_i1	Glyma.02G111600.5	100	137	1	226	2517	2289	1	0	3454

TRINITY_DN10760_c0_g1_i1	Glyma.02G111600.3	100	137	1	226	2517	2289	1	0	3454

TRINITY_DN10760_c0_g1_i1	Glyma.02G111600.4	100	137	1	226	2517	2289	1	0	3454

TRINITY_DN15954_c0_g1_i4	Glyma.13G028400.1	100	430	23	285	2563	113	2391	0	1694

TRINITY_DN10739_c0_g2_i1	Glyma.03G248300.2	100	277	24	157	2420	2286	5	0	2464

TRINITY_DN15458_c0_g1_i1	Glyma.17G004700.2	100	264	21	3	2272	341	2622	0	2575

TRINITY_DN14171_c0_g1_i2	Glyma.09G042000.1	95	202	10	113	2399	1	2283	0	3035

TRINITY_DN14171_c0_g1_i3	Glyma.09G042000.1	99.05660377	201	16	113	2388	1	2283	0	2968

TRINITY_DN2154_c0_g1_i1	Glyma.07G001300.1	100	190	4	198	2486	2289	1	0	3153

TRINITY_DN16427_c0_g3_i6	Glyma.04G134700.1	100	136	0	2500	4790	2885	595	0	3478

TRINITY_DN15954_c0_g1_i4	Glyma.20G055900.1	100	448	21	285	2563	113	2391	0	1605

TRINITY_DN14604_c0_g1_i1	Glyma.11G088900.1	100	207	5	229	2517	15	2288	0	2972

TRINITY_DN35897_c0_g1_i1	Glyma.07G067100.1	95.72649573	165	3	250	2535	2289	1	0	3282

TRINITY_DN14344_c0_g1_i2	Glyma.20G202200.3	98.73417722	152	4	272	2551	2289	1	0	3315

TRINITY_DN14344_c0_g1_i2	Glyma.20G202200.4	100	152	4	272	2551	2289	1	0	3315

TRINITY_DN14344_c0_g1_i2	Glyma.20G202200.1	100	152	4	272	2551	2289	1	0	3315

TRINITY_DN14344_c0_g1_i2	Glyma.20G202200.2	95.23809524	152	4	272	2551	2289	1	0	3315

TRINITY_DN14344_c0_g1_i2	Glyma.20G202200.5	95.29411765	152	4	272	2551	2289	1	0	3315

TRINITY_DN14344_c0_g1_i4	Glyma.20G202200.3	95.0136612	152	4	272	2551	2289	1	0	3315

TRINITY_DN14344_c0_g1_i4	Glyma.20G202200.4	96.90721649	152	4	272	2551	2289	1	0	3315

TRINITY_DN14344_c0_g1_i4	Glyma.20G202200.1	100	152	4	272	2551	2289	1	0	3315

TRINITY_DN14344_c0_g1_i4	Glyma.20G202200.2	99.16666667	152	4	272	2551	2289	1	0	3315

TRINITY_DN14344_c0_g1_i4	Glyma.20G202200.5	98.54191981	152	4	272	2551	2289	1	0	3315

TRINITY_DN7366_c0_g1_i1	Glyma.13G211200.1	100	140	6	507	2789	1	2289	0	3402

TRINITY_DN7366_c0_g1_i1	Glyma.15G101500.2	100	139	7	507	2789	1	2289	0	3408

TRINITY_DN8781_c0_g1_i1	Glyma.05G170700.1	95.34883721	324	6	533	2818	2566	281	0	2394

TRINITY_DN16033_c0_g1_i2	Glyma.03G238100.1	98.59649123	275	16	400	2682	2925	658	0	2529

TRINITY_DN11699_c0_g1_i1	Glyma.09G208200.1	100	243	13	1	2279	1088	3354	0	2675

TRINITY_DN11699_c0_g1_i1	Glyma.09G208200.2	100	243	13	1	2279	1088	3354	0	2675

TRINITY_DN14718_c0_g2_i2	Glyma.06G223500.1	96.12068966	200	6	583	2862	5567	3285	0	3022

TRINITY_DN14718_c0_g2_i2	Glyma.06G223500.2	96.07843137	200	6	583	2862	4982	2700	0	3022

TRINITY_DN15954_c0_g1_i5	Glyma.20G055900.1	98.15100154	459	12	142	2423	110	2391	0	1605

TRINITY_DN13116_c0_g1_i1	Glyma.13G109600.18	97.84946237	347	22	436	2715	2262	1	0	2084

TRINITY_DN13116_c0_g1_i1	Glyma.13G109600.16	97.85932722	347	22	436	2715	2262	1	0	2084

TRINITY_DN13116_c0_g1_i1	Glyma.13G109600.10	97.86729858	347	22	436	2715	2262	1	0	2084

TRINITY_DN13116_c0_g1_i1	Glyma.13G109600.11	97.87234043	347	22	436	2715	2262	1	0	2084

TRINITY_DN13116_c0_g1_i2	Glyma.13G109600.18	97.85714286	347	22	198	2477	2262	1	0	2084

TRINITY_DN13116_c0_g1_i2	Glyma.13G109600.16	97.84946237	347	22	198	2477	2262	1	0	2084

TRINITY_DN13116_c0_g1_i2	Glyma.13G109600.10	97.85407725	347	22	198	2477	2262	1	0	2084

TRINITY_DN13116_c0_g1_i2	Glyma.13G109600.11	97.87234043	347	22	198	2477	2262	1	0	2084

TRINITY_DN1122_c0_g1_i1	Glyma.01G001200.1	98.64864865	245	30	283	2553	2232	1	0	2396

TRINITY_DN16033_c0_g1_i1	Glyma.03G238100.1	100	234	14	400	2682	2925	658	0	2767

TRINITY_DN15458_c0_g1_i4	Glyma.17G004700.2	96.96312364	222	16	3	2272	341	2622	0	2841

TRINITY_DN8984_c0_g1_i2	Glyma.02G098300.1	97.93814433	137	5	4	2288	2282	1	0	3408

TRINITY_DN9262_c0_g1_i1	Glyma.08G018400.1	95.45454545	130	3	1461	3747	2050	4335	0	3485

TRINITY_DN15954_c0_g1_i5	Glyma.13G028400.1	98.14814815	448	10	142	2423	110	2391	0	1677

TRINITY_DN3954_c0_g2_i1	Glyma.03G251700.2	96.90346084	372	25	449	2719	16	2283	0	1936

TRINITY_DN3954_c0_g1_i1	Glyma.03G251700.2	100	371	26	449	2718	16	2283	0	1936

TRINITY_DN15878_c0_g2_i2	Glyma.19G260900.3	98.7654321	334	44	491	2743	2250	1	0	1914

TRINITY_DN15878_c0_g2_i2	Glyma.19G260900.2	98.72611465	334	44	491	2743	2250	1	0	1914

TRINITY_DN14344_c0_g1_i3	Glyma.20G202200.3	100	329	23	378	2645	2274	1	0	2194

TRINITY_DN14344_c0_g1_i3	Glyma.20G202200.4	100	329	23	378	2645	2274	1	0	2194

TRINITY_DN14344_c0_g1_i3	Glyma.20G202200.1	98.7654321	329	23	378	2645	2274	1	0	2194

TRINITY_DN14344_c0_g1_i3	Glyma.20G202200.2	100	329	23	378	2645	2274	1	0	2194

TRINITY_DN14344_c0_g1_i3	Glyma.20G202200.5	100	329	23	378	2645	2274	1	0	2194

TRINITY_DN14344_c0_g1_i5	Glyma.20G202200.3	98.50746269	329	23	378	2645	2274	1	0	2194

TRINITY_DN14344_c0_g1_i5	Glyma.20G202200.4	99.72752044	329	23	378	2645	2274	1	0	2194

TRINITY_DN14344_c0_g1_i5	Glyma.20G202200.1	97.24137931	329	23	378	2645	2274	1	0	2194

TRINITY_DN14344_c0_g1_i5	Glyma.20G202200.2	100	329	23	378	2645	2274	1	0	2194

TRINITY_DN14344_c0_g1_i5	Glyma.20G202200.5	98.64864865	329	23	378	2645	2274	1	0	2194

TRINITY_DN16425_c0_g1_i3	Glyma.15G035900.3	95.0877193	322	17	835	3096	2757	484	0	2194

TRINITY_DN16425_c0_g1_i1	Glyma.15G035900.3	99.23076923	318	17	902	3163	2757	484	0	2217

TRINITY_DN10754_c0_g1_i1	Glyma.14G009700.1	100	308	11	275	2557	2300	21	0	2446

TRINITY_DN10754_c0_g2_i1	Glyma.14G009700.1	95.2853598	308	11	275	2557	2300	21	0	2446

TRINITY_DN10754_c0_g3_i1	Glyma.14G009700.1	95.30364372	308	11	275	2557	2300	21	0	2446

TRINITY_DN13717_c0_g1_i1	Glyma.16G051500.1	100	201	13	345	2594	2286	1	0	2865

TRINITY_DN3322_c0_g1_i1	Glyma.06G171700.1	98.68421053	171	5	170	2444	2287	1	0	3199

TRINITY_DN3322_c0_g1_i1	Glyma.06G171700.2	97.01149425	171	5	170	2444	2287	1	0	3199

TRINITY_DN3322_c0_g2_i1	Glyma.06G171700.1	99.13043478	171	5	170	2444	2287	1	0	3199

TRINITY_DN3322_c0_g2_i1	Glyma.06G171700.2	96.57142857	171	5	170	2444	2287	1	0	3199

TRINITY_DN7273_c0_g1_i1	Glyma.20G179400.5	97.00460829	170	28	799	3049	2261	5	0	2856

TRINITY_DN7273_c0_g1_i1	Glyma.20G179400.4	97.77777778	170	28	799	3049	2261	5	0	2856

TRINITY_DN7273_c0_g1_i2	Glyma.20G179400.5	100	167	33	799	3038	2261	5	0	2800

TRINITY_DN7273_c0_g1_i2	Glyma.20G179400.4	100	167	33	799	3038	2261	5	0	2800

TRINITY_DN13665_c0_g1_i1	Glyma.10G013400.1	100	164	1	72	2352	12	2298	0	3277

TRINITY_DN15167_c0_g2_i1	Glyma.09G265200.3	98.83449883	158	13	348	2616	1	2275	0	3155

TRINITY_DN15167_c0_g2_i1	Glyma.09G265200.1	98.83495146	158	13	348	2616	1	2275	0	3155

TRINITY_DN15167_c0_g2_i1	Glyma.09G265200.2	98.8372093	158	13	348	2616	1	2275	0	3155

TRINITY_DN15167_c0_g2_i2	Glyma.09G265200.3	95	158	13	550	2818	1	2275	0	3155

TRINITY_DN15167_c0_g2_i2	Glyma.09G265200.1	96.96048632	158	13	550	2818	1	2275	0	3155

TRINITY_DN15167_c0_g2_i2	Glyma.09G265200.2	100	158	13	550	2818	1	2275	0	3155

TRINITY_DN15956_c0_g2_i1	Glyma.10G092200.1	100	593	59	451	2691	2394	136	2.78E-131	472

TRINITY_DN15956_c0_g2_i3	Glyma.10G092200.1	98.01980198	593	59	451	2691	2394	136	3.09E-131	472

TRINITY_DN15956_c0_g2_i1	Glyma.02G176700.1	100	585	61	451	2691	2391	136	1.65E-138	496

TRINITY_DN15956_c0_g2_i3	Glyma.02G176700.1	97.4248927	585	61	451	2691	2391	136	1.83E-138	496

TRINITY_DN16379_c0_g1_i2	Glyma.13G143600.1	97.82608696	408	4	471	2754	2322	39	0	1936

TRINITY_DN10754_c0_g1_i1	Glyma.02G304100.1	95.3125	312	9	275	2557	2300	21	0	2435

TRINITY_DN10754_c0_g1_i1	Glyma.02G304100.2	95.29411765	312	9	275	2557	2300	21	0	2435

TRINITY_DN10754_c0_g2_i1	Glyma.02G304100.1	95.29085873	312	9	275	2557	2300	21	0	2435

TRINITY_DN10754_c0_g2_i1	Glyma.02G304100.2	95.29411765	312	9	275	2557	2300	21	0	2435

TRINITY_DN10754_c0_g3_i1	Glyma.02G304100.1	95.30864198	312	9	275	2557	2300	21	0	2435

TRINITY_DN10754_c0_g3_i1	Glyma.02G304100.2	95.2991453	312	9	275	2557	2300	21	0	2435

TRINITY_DN6691_c0_g2_i1	Glyma.16G138300.1	95.70093458	241	5	366	2648	1	2277	0	2809

TRINITY_DN6691_c0_g2_i1	Glyma.16G138300.9	100	241	5	366	2648	1	2277	0	2809

TRINITY_DN6691_c0_g2_i1	Glyma.16G138300.3	100	241	5	366	2648	1	2277	0	2809

TRINITY_DN6691_c0_g2_i1	Glyma.16G138300.11	98.72611465	241	5	366	2648	1	2277	0	2809

TRINITY_DN9983_c0_g1_i1	Glyma.06G182800.2	98.33679834	194	22	404	2671	2262	1	0	2876

TRINITY_DN9983_c0_g1_i1	Glyma.06G182800.1	98.33333333	194	22	404	2671	2262	1	0	2876

TRINITY_DN9983_c0_g1_i1	Glyma.06G182800.3	98.33333333	194	22	404	2671	2262	1	0	2876

TRINITY_DN13844_c0_g1_i1	Glyma.11G175700.5	99.01960784	174	1	692	2977	1	2283	0	3238

TRINITY_DN13844_c0_g1_i1	Glyma.11G175700.6	100	174	1	692	2977	1	2283	0	3238

TRINITY_DN11072_c0_g1_i1	Glyma.18G245200.1	98.68421053	165	2	315	2597	2286	1	0	3288

TRINITY_DN35897_c0_g1_i1	Glyma.16G033100.1	98.18181818	152	0	250	2535	2286	1	0	3380

TRINITY_DN7366_c0_g1_i1	Glyma.15G101500.1	100	139	7	507	2785	1	2285	0	3400

TRINITY_DN15954_c0_g1_i9	Glyma.20G055900.1	97.87685775	458	12	285	2563	113	2391	0	1605

TRINITY_DN13088_c0_g1_i3	Glyma.09G143700.1	100	408	44	595	2849	89	2331	0	1495

TRINITY_DN14604_c0_g1_i5	Glyma.09G195100.2	95.01557632	388	13	228	2506	14	2289	0	1973

TRINITY_DN11699_c0_g1_i1	Glyma.01G014000.2	95.55236729	248	5	1	2279	1076	3357	0	2787

TRINITY_DN11699_c0_g1_i1	Glyma.01G014000.3	96.42857143	248	5	1	2279	1076	3357	0	2787

TRINITY_DN11699_c0_g1_i1	Glyma.01G014000.1	100	248	5	1	2279	1076	3357	0	2787

TRINITY_DN11699_c0_g1_i1	Glyma.01G014000.4	99.03846154	248	5	1	2279	1076	3357	0	2787

TRINITY_DN15867_c0_g3_i4	Glyma.15G172400.7	100	215	7	189	2460	1	2278	0	2900

TRINITY_DN15867_c0_g3_i4	Glyma.15G172400.2	97.12230216	215	7	189	2460	1	2278	0	2900

TRINITY_DN15867_c0_g3_i4	Glyma.15G172400.3	100	215	7	189	2460	1	2278	0	2900

TRINITY_DN15867_c0_g3_i4	Glyma.15G172400.6	99.24242424	215	7	189	2460	1	2278	0	2900

TRINITY_DN15867_c0_g3_i4	Glyma.15G172400.1	100	215	7	189	2460	1	2278	0	2900

TRINITY_DN15954_c0_g1_i9	Glyma.13G028400.1	98.63013699	447	10	285	2563	113	2391	0	1677

TRINITY_DN13088_c0_g1_i3	Glyma.16G196700.1	99.6031746	405	42	596	2849	90	2328	0	1489

TRINITY_DN14151_c0_g1_i1	Glyma.18G239200.1	96.41693811	370	50	303	2547	87	2316	0	1567

TRINITY_DN15878_c0_g2_i2	Glyma.03G261800.5	95.6043956	339	42	491	2743	2247	1	0	1901

TRINITY_DN15878_c0_g2_i2	Glyma.03G261800.1	99.15254237	339	42	491	2743	2247	1	0	1901

TRINITY_DN15878_c0_g2_i2	Glyma.03G261800.2	98.50746269	339	42	491	2743	2247	1	0	1901

TRINITY_DN15878_c0_g2_i2	Glyma.03G261800.3	95.3271028	339	42	491	2743	2247	1	0	1901

TRINITY_DN15878_c0_g2_i2	Glyma.03G261800.4	100	339	42	491	2743	2247	1	0	1901

TRINITY_DN14660_c1_g1_i4	Glyma.10G202800.1	97.6744186	239	38	436	2648	1	2258	0	2266

TRINITY_DN14660_c1_g1_i4	Glyma.10G202800.2	98.30508475	239	38	436	2648	1	2258	0	2266

TRINITY_DN14660_c1_g1_i5	Glyma.10G202800.1	99.47368421	239	38	436	2648	1	2258	0	2266

TRINITY_DN14660_c1_g1_i5	Glyma.10G202800.2	100	239	38	436	2648	1	2258	0	2266

TRINITY_DN6529_c0_g1_i1	Glyma.18G074300.1	98.1042654	229	10	249	2520	2794	517	0	2833

TRINITY_DN6529_c0_g1_i1	Glyma.18G074300.2	99.15611814	229	10	249	2520	2509	232	0	2833

TRINITY_DN6529_c0_g1_i1	Glyma.18G074300.3	98.0952381	229	10	249	2520	2509	232	0	2833

TRINITY_DN15867_c0_g3_i4	Glyma.09G066400.1	95.256917	228	7	189	2460	1	2272	0	2800

TRINITY_DN11072_c0_g1_i1	Glyma.09G247500.1	95.50561798	170	11	315	2597	2268	1	0	3166

TRINITY_DN13959_c0_g1_i1	Glyma.20G242100.3	98.86363636	157	9	387	2657	2811	532	0	3238

TRINITY_DN13959_c0_g1_i2	Glyma.20G242100.3	99.00990099	157	9	387	2657	2811	532	0	3238

TRINITY_DN6599_c0_g1_i1	Glyma.02G271300.1	98.82352941	104	2	223	2505	1	2283	0	3629

TRINITY_DN6599_c0_g1_i1	Glyma.02G271300.3	98.828125	104	2	223	2505	1	2283	0	3629

TRINITY_DN11254_c0_g1_i1	Glyma.13G347700.1	100	514	59	142	2387	2561	313	0	909

TRINITY_DN15751_c0_g1_i10	Glyma.05G045700.1	97.59036145	347	48	968	3232	7	2220	0	1731

TRINITY_DN15621_c0_g1_i2	Glyma.05G077100.1	100	310	15	56	2335	70	2325	0	2305

TRINITY_DN15621_c0_g1_i3	Glyma.05G077100.1	100	310	15	56	2335	70	2325	0	2305

TRINITY_DN13818_c0_g1_i1	Glyma.10G097600.1	100	177	6	201	2480	1	2280	0	3197

TRINITY_DN145_c0_g1_i1	Glyma.03G225800.2	100	159	7	538	2802	1	2283	0	3219

TRINITY_DN5374_c0_g1_i2	Glyma.18G101600.1	95.91141397	148	14	330	2579	2280	1	0	3164

TRINITY_DN145_c0_g1_i1	Glyma.19G222700.2	100	142	1	538	2802	1	2283	0	3314

TRINITY_DN15981_c0_g1_i20	Glyma.11G048300.2	95.50561798	138	3	1075	3351	2513	231	0	3413

TRINITY_DN15981_c0_g1_i20	Glyma.11G048300.1	100	138	3	1075	3351	2513	231	0	3413

TRINITY_DN15981_c0_g1_i8	Glyma.11G048300.2	96.55172414	138	3	1201	3477	2513	231	0	3413

TRINITY_DN15981_c0_g1_i8	Glyma.11G048300.1	100	138	3	1201	3477	2513	231	0	3413

TRINITY_DN11114_c0_g1_i1	Glyma.05G000600.1	97.84946237	129	4	509	2782	2280	1	0	3424

TRINITY_DN11114_c0_g1_i1	Glyma.19G000400.2	99.46808511	122	3	509	2782	2280	1	0	3463

TRINITY_DN11114_c0_g1_i1	Glyma.19G000400.1	99.47089947	122	3	509	2782	2280	1	0	3463

TRINITY_DN15888_c0_g3_i1	Glyma.02G269200.1	98.75	467	31	772	3035	813	3070	0	1356

TRINITY_DN14604_c0_g1_i1	Glyma.16G116000.1	100	388	11	242	2517	25	2288	0	1910

TRINITY_DN13778_c1_g3_i3	Glyma.16G064800.1	95.48022599	358	22	200	2464	3360	1093	0	2030

TRINITY_DN15302_c0_g1_i2	Glyma.17G067800.2	98.85057471	265	16	377	2641	1	2280	0	2623

TRINITY_DN3954_c0_g2_i1	Glyma.20G221800.1	98.79518072	175	6	444	2719	23	2301	0	3186

TRINITY_DN3954_c0_g1_i1	Glyma.20G221800.1	95.08196721	175	7	444	2718	23	2301	0	3181

TRINITY_DN13818_c0_g1_i1	Glyma.02G184100.1	100	174	4	201	2480	1	2280	0	3225

TRINITY_DN16628_c0_g1_i1	Glyma.10G143700.2	97.42268041	170	6	569	2846	2278	1	0	3221

TRINITY_DN16628_c0_g1_i1	Glyma.10G143700.1	100	170	6	569	2846	2278	1	0	3221

TRINITY_DN15841_c0_g1_i1	Glyma.12G074800.3	96.86888454	167	4	144	2425	1	2247	0	3062

TRINITY_DN15841_c0_g1_i2	Glyma.12G074800.3	95.64766839	167	4	144	2425	1	2247	0	3062

TRINITY_DN13547_c0_g1_i1	Glyma.07G045600.1	98.04719284	166	12	3623	5898	3212	5478	0	3158

TRINITY_DN13547_c0_g1_i1	Glyma.07G045600.2	98.05825243	166	12	3623	5898	3137	5403	0	3158

TRINITY_DN13547_c0_g1_i1	Glyma.07G045600.3	98.03921569	166	12	3623	5898	1694	3960	0	3158

TRINITY_DN13547_c0_g1_i1	Glyma.07G045600.4	98.05825243	166	12	3623	5898	1007	3273	0	3158

TRINITY_DN12388_c0_g2_i1	Glyma.02G082200.1	95.74468085	151	3	320	2600	2277	1	0	3339

TRINITY_DN14064_c0_g1_i5	Glyma.13G209800.2	98.937583	118	1	1676	3954	2282	1	0	3541

TRINITY_DN15888_c0_g3_i2	Glyma.14G047900.1	98.75	397	40	355	2621	801	3040	0	1657

TRINITY_DN16237_c0_g1_i15	Glyma.06G164900.2	97.2972973	316	29	396	2655	90	2346	0	2170

TRINITY_DN3744_c0_g1_i1	Glyma.17G135600.1	100	271	33	222	2451	2274	3	0	2324

TRINITY_DN15652_c1_g3_i2	Glyma.20G144600.2	98.55072464	207	3	469	2745	4	2283	0	3033

TRINITY_DN15652_c1_g3_i3	Glyma.20G144600.2	96.72131148	207	3	436	2712	4	2283	0	3033

TRINITY_DN12388_c0_g2_i1	Glyma.16G167800.1	96.15384615	145	1	320	2600	2277	1	0	3384

TRINITY_DN6691_c0_g2_i1	Glyma.11G069300.1	95.23809524	500	44	400	2648	32	2286	0	1079

TRINITY_DN15568_c1_g1_i2	Glyma.12G136000.1	100	390	33	710	2948	112	2371	0	1655

TRINITY_DN15305_c1_g1_i1	Glyma.12G074100.6	98.79518072	371	30	480	2746	2527	282	0	1851

TRINITY_DN15305_c1_g1_i1	Glyma.12G074100.5	98.79518072	371	30	480	2746	2527	282	0	1851

TRINITY_DN15305_c1_g1_i1	Glyma.12G074100.3	96.34146341	371	30	480	2746	2527	282	0	1851

TRINITY_DN15305_c1_g1_i1	Glyma.12G074100.1	100	371	30	480	2746	2527	282	0	1851

TRINITY_DN15305_c1_g1_i1	Glyma.12G074100.2	99.67213115	371	30	480	2746	2527	282	0	1851

TRINITY_DN15305_c1_g1_i1	Glyma.12G074100.4	98.9010989	371	30	480	2746	2527	282	0	1851

TRINITY_DN13778_c1_g3_i3	Glyma.16G064900.1	97.34660033	366	18	200	2464	3822	1555	0	2008

TRINITY_DN15981_c0_g1_i20	Glyma.17G161300.1	100	361	6	1075	3351	2507	231	0	2172

TRINITY_DN15981_c0_g1_i8	Glyma.17G161300.1	97.01492537	361	6	1201	3477	2507	231	0	2172

TRINITY_DN13778_c1_g3_i3	Glyma.16G065000.1	95.68627451	357	24	200	2464	3522	1270	0	1962

TRINITY_DN13778_c1_g3_i3	Glyma.16G065400.1	98.71794872	356	18	200	2464	4686	2419	0	2063

TRINITY_DN15981_c0_g1_i20	Glyma.05G105200.1	97.43589744	354	5	1075	3351	2507	231	0	2211

TRINITY_DN15981_c0_g1_i8	Glyma.05G105200.1	100	354	5	1201	3477	2507	231	0	2211

TRINITY_DN5374_c0_g1_i2	Glyma.18G101600.2	97.85714286	149	12	330	2579	2277	1	0	3171

TRINITY_DN5910_c0_g1_i1	Glyma.08G288400.1	99.55357143	145	0	2	2281	247	2526	0	3408

TRINITY_DN15912_c1_g1_i2	Glyma.15G016500.1	95.51820728	141	6	390	2669	2256	1	0	3275

TRINITY_DN16019_c0_g1_i8	Glyma.11G118600.2	100	134	0	895	3174	2280	1	0	3469

TRINITY_DN16019_c0_g1_i5	Glyma.11G118600.2	100	131	0	895	3174	2280	1	0	3485

TRINITY_DN13991_c0_g1_i2	Glyma.09G070900.1	96.25	430	18	298	2547	2274	1	0	1607

TRINITY_DN9899_c0_g1_i3	Glyma.07G057700.1	100	358	62	14	2241	458	2685	0	1567

TRINITY_DN15458_c0_g1_i4	Glyma.15G121500.2	95.83333333	285	17	1	2273	342	2593	0	2416

TRINITY_DN9805_c0_g1_i1	Glyma.14G017100.1	99.56521739	250	12	478	2751	2265	1	0	2700

TRINITY_DN16628_c0_g1_i1	Glyma.20G092500.4	100	161	5	569	2846	2275	1	0	3284

TRINITY_DN16628_c0_g1_i1	Glyma.20G092500.2	97.36842105	161	5	569	2846	2275	1	0	3284

TRINITY_DN16628_c0_g1_i1	Glyma.20G092500.1	97.36842105	161	5	569	2846	2275	1	0	3284

TRINITY_DN6467_c0_g1_i1	Glyma.09G112200.7	95.94594595	129	2	2	2280	2152	4424	0	3456

TRINITY_DN6467_c0_g2_i1	Glyma.09G112200.7	98.80952381	129	2	2	2280	2152	4424	0	3456

TRINITY_DN6467_c0_g1_i1	Glyma.09G112200.9	95.74468085	129	2	2	2280	2140	4412	0	3456

TRINITY_DN6467_c0_g2_i1	Glyma.09G112200.9	100	129	2	2	2280	2140	4412	0	3456

TRINITY_DN6467_c0_g1_i1	Glyma.09G112200.6	100	129	2	2	2280	2131	4403	0	3456

TRINITY_DN6467_c0_g2_i1	Glyma.09G112200.6	97.72727273	129	2	2	2280	2131	4403	0	3456

TRINITY_DN6467_c0_g1_i1	Glyma.09G112200.8	95.30685921	129	2	2	2280	2119	4391	0	3456

TRINITY_DN6467_c0_g2_i1	Glyma.09G112200.8	97.8125	129	2	2	2280	2119	4391	0	3456

TRINITY_DN9899_c0_g1_i3	Glyma.16G026700.1	96	357	64	14	2241	458	2679	0	1544

TRINITY_DN16237_c0_g1_i15	Glyma.04G200600.2	98.05825243	321	23	396	2655	90	2346	0	2176

TRINITY_DN16237_c0_g1_i11	Glyma.06G164900.2	98.4375	316	29	396	2652	90	2343	0	2165

TRINITY_DN16237_c0_g1_i15	Glyma.06G164900.1	95.29085873	316	29	396	2652	90	2343	0	2165

TRINITY_DN16237_c0_g1_i5	Glyma.06G164900.2	95.96069869	316	29	396	2652	90	2343	0	2165

TRINITY_DN13310_c0_g2_i1	Glyma.13G160100.1	96.34146341	299	28	274	2528	1	2249	0	2215

TRINITY_DN13310_c0_g2_i2	Glyma.13G160100.1	100	299	28	258	2512	1	2249	0	2215

TRINITY_DN14592_c0_g1_i1	Glyma.20G242000.1	98.26086957	283	22	93	2357	1	2253	0	2394

TRINITY_DN9805_c0_g1_i1	Glyma.02G296400.1	99.44134078	260	11	478	2751	2262	1	0	2638

TRINITY_DN6529_c0_g1_i1	Glyma.08G332700.1	98.11320755	239	4	249	2520	2791	517	0	2824

TRINITY_DN6529_c0_g1_i1	Glyma.08G332700.2	98.0952381	239	4	249	2520	2506	232	0	2824

TRINITY_DN2154_c0_g1_i1	Glyma.03G121300.2	100	231	2	210	2486	2277	1	0	2915

TRINITY_DN2154_c0_g1_i1	Glyma.03G121300.1	100	231	2	210	2486	2277	1	0	2915

TRINITY_DN2154_c0_g1_i1	Glyma.03G121300.3	100	231	2	210	2486	2277	1	0	2915

TRINITY_DN17615_c0_g1_i1	Glyma.05G165600.2	100	229	11	161	2434	1	2268	0	2848

TRINITY_DN15458_c0_g1_i1	Glyma.15G121500.2	100	223	15	1	2273	342	2593	0	2771

TRINITY_DN14604_c0_g1_i5	Glyma.11G088900.1	96.04519774	207	4	229	2505	15	2288	0	3027

TRINITY_DN6147_c0_g1_i1	Glyma.08G229400.1	96	122	0	1	2278	1209	3486	0	3531

TRINITY_DN6147_c0_g1_i1	Glyma.08G229400.2	98.55072464	122	0	1	2278	897	3174	0	3531

TRINITY_DN11827_c0_g1_i2	Glyma.13G350000.1	97.22222222	358	32	372	2627	11	2257	0	1892

TRINITY_DN14622_c0_g1_i2	Glyma.10G179700.6	99.13043478	175	8	193	2460	6	2272	0	3112

TRINITY_DN5758_c0_g1_i1	Glyma.15G144500.1	99.11504425	161	0	303	2579	1	2277	0	3314

TRINITY_DN5758_c0_g1_i1	Glyma.09G039300.1	99.11504425	161	0	303	2579	1	2277	0	3314

TRINITY_DN9498_c0_g1_i2	Glyma.02G251500.2	95.23809524	149	4	2	2275	11	2281	0	3321

TRINITY_DN5374_c0_g1_i2	Glyma.08G313100.1	100	135	13	330	2579	2274	1	0	3264

TRINITY_DN36917_c0_g1_i1	Glyma.02G237300.1	96.68874172	446	43	9	2250	2255	2	0	1371

TRINITY_DN15122_c0_g2_i1	Glyma.18G035700.2	97.8021978	360	27	598	2858	4267	2010	0	1995

TRINITY_DN14898_c0_g2_i1	Glyma.12G011700.1	95.5801105	356	43	12	2248	523	2765	0	1766

TRINITY_DN13310_c0_g2_i1	Glyma.17G111400.2	100	312	27	274	2529	1	2244	0	2139

TRINITY_DN13310_c0_g2_i1	Glyma.17G111400.1	98.44660194	312	27	274	2529	1	2244	0	2139

TRINITY_DN13310_c0_g2_i2	Glyma.17G111400.2	99.13793103	312	27	258	2513	1	2244	0	2139

TRINITY_DN13310_c0_g2_i2	Glyma.17G111400.1	99.14529915	312	27	258	2513	1	2244	0	2139

TRINITY_DN15751_c0_g1_i14	Glyma.05G045700.1	100	310	45	968	3232	7	2214	0	1975

TRINITY_DN15751_c0_g1_i4	Glyma.05G045700.1	96.875	310	45	968	3232	7	2214	0	1975

TRINITY_DN16082_c0_g1_i1	Glyma.05G166400.2	100	302	12	173	2437	2271	1	0	2427

TRINITY_DN15954_c0_g1_i5	Glyma.17G184900.1	98.14814815	293	4	147	2419	1	2273	0	2542

TRINITY_DN15954_c0_g1_i9	Glyma.17G184900.1	97.85276074	293	4	287	2559	1	2273	0	2542

TRINITY_DN15808_c0_g1_i3	Glyma.04G163600.2	100	266	24	2227	4480	1971	4201	0	2296

TRINITY_DN15808_c0_g1_i3	Glyma.04G163600.1	95.53571429	266	24	2227	4480	1971	4201	0	2296

TRINITY_DN15808_c0_g1_i3	Glyma.04G163600.3	100	266	24	2227	4480	1971	4201	0	2296

TRINITY_DN16082_c0_g1_i7	Glyma.08G202000.5	98.7654321	215	10	188	2431	2274	1	0	2793

TRINITY_DN8602_c0_g1_i1	Glyma.03G161800.1	97.40932642	175	17	185	2451	2271	11	0	3079

TRINITY_DN15981_c0_g1_i20	Glyma.01G193600.2	96.55870445	139	1	1075	3347	2507	232	0	3413

TRINITY_DN15981_c0_g1_i20	Glyma.01G193600.1	97	139	1	1075	3347	2507	232	0	3413

TRINITY_DN15981_c0_g1_i8	Glyma.01G193600.2	99.2481203	139	1	1201	3473	2507	232	0	3413

TRINITY_DN15981_c0_g1_i8	Glyma.01G193600.1	100	139	1	1201	3473	2507	232	0	3413

TRINITY_DN14244_c0_g4_i1	Glyma.13G329100.3	96.20253165	114	0	609	2884	2370	95	0	3572

TRINITY_DN13023_c0_g1_i2	Glyma.16G201700.1	98.7394958	333	11	350	2611	70	2337	0	2228

TRINITY_DN16237_c0_g1_i11	Glyma.04G200600.2	99.36708861	321	23	396	2652	90	2343	0	2170

TRINITY_DN16237_c0_g1_i15	Glyma.04G200600.1	100	321	23	396	2652	90	2343	0	2170

TRINITY_DN16237_c0_g1_i5	Glyma.04G200600.2	100	321	23	396	2652	90	2343	0	2170

TRINITY_DN15867_c0_g3_i4	Glyma.13G106700.16	95.25862069	289	6	189	2448	1	2272	0	2484

TRINITY_DN15867_c0_g3_i4	Glyma.13G106700.11	96.18320611	289	6	189	2448	1	2272	0	2484

TRINITY_DN15867_c0_g3_i4	Glyma.13G106700.12	98.73417722	289	6	189	2448	1	2272	0	2484

TRINITY_DN15867_c0_g3_i4	Glyma.13G106700.13	98.30508475	289	6	189	2448	1	2272	0	2484

TRINITY_DN15867_c0_g3_i4	Glyma.13G106700.14	100	289	6	189	2448	1	2272	0	2484

TRINITY_DN15867_c0_g3_i4	Glyma.13G106700.7	100	289	6	189	2448	1	2272	0	2484

TRINITY_DN15867_c0_g3_i4	Glyma.13G106700.1	97.87234043	289	6	189	2448	1	2272	0	2484

TRINITY_DN15867_c0_g3_i4	Glyma.13G106700.2	100	289	6	189	2448	1	2272	0	2484

TRINITY_DN15867_c0_g3_i4	Glyma.13G106700.3	98.7012987	289	6	189	2448	1	2272	0	2484

TRINITY_DN15867_c0_g3_i4	Glyma.13G106700.4	96.58703072	289	6	189	2448	1	2272	0	2484

TRINITY_DN15867_c0_g3_i4	Glyma.13G106700.17	97.58064516	289	6	189	2448	1	2272	0	2484

TRINITY_DN15867_c0_g3_i4	Glyma.17G052600.1	95.25731584	275	6	189	2448	1	2272	0	2562

TRINITY_DN17615_c0_g1_i1	Glyma.08G123300.6	100	247	7	161	2434	1	2262	0	2743

TRINITY_DN17615_c0_g1_i1	Glyma.08G123300.3	100	247	7	161	2434	1	2262	0	2743

TRINITY_DN13665_c0_g1_i1	Glyma.02G012800.1	100	153	0	78	2352	21	2295	0	3354

TRINITY_DN12659_c1_g1_i1	Glyma.01G140600.1	98.11320755	122	3	235	2505	2274	1	0	3493

TRINITY_DN13088_c0_g1_i3	Glyma.09G143700.3	97.4789916	407	44	606	2849	1	2232	0	1480

TRINITY_DN13088_c0_g1_i3	Glyma.09G143700.4	100	407	44	606	2849	1	2232	0	1480

TRINITY_DN13088_c0_g1_i3	Glyma.09G143700.2	96.66666667	407	44	606	2849	1	2232	0	1480

TRINITY_DN13088_c0_g1_i3	Glyma.16G196700.2	100	404	42	606	2849	1	2229	0	1476

TRINITY_DN13088_c0_g1_i3	Glyma.16G196700.3	100	404	42	606	2849	1	2229	0	1476

TRINITY_DN3954_c0_g2_i1	Glyma.03G251700.1	100	371	25	449	2706	16	2270	0	1917

TRINITY_DN3954_c0_g1_i1	Glyma.03G251700.1	100	370	26	449	2705	16	2270	0	1917

TRINITY_DN13448_c0_g1_i1	Glyma.11G246200.6	95.52238806	322	0	17	2290	43	2316	0	2416

TRINITY_DN13448_c0_g1_i6	Glyma.11G246200.6	99.54954955	322	0	17	2290	43	2316	0	2416

TRINITY_DN16082_c0_g1_i8	Glyma.05G166400.2	97.95918367	259	10	173	2437	2271	1	0	2687

TRINITY_DN10708_c0_g1_i1	Glyma.17G211900.1	95.57522124	249	19	286	2558	2295	44	0	2671

TRINITY_DN10708_c0_g1_i3	Glyma.17G211900.1	96.79487179	249	19	286	2558	2295	44	0	2671

TRINITY_DN16082_c0_g1_i5	Glyma.05G166400.2	99.3006993	234	7	173	2437	2271	1	0	2826

TRINITY_DN15954_c0_g1_i4	Glyma.17G184900.1	97.59036145	225	2	287	2559	1	2273	0	2940

TRINITY_DN15954_c0_g1_i8	Glyma.17G184900.1	98.88888889	225	2	147	2419	1	2273	0	2940

TRINITY_DN16082_c0_g1_i9	Glyma.08G202000.5	97.45762712	195	7	142	2385	2274	1	0	2926

TRINITY_DN11431_c0_g2_i2	Glyma.07G040600.1	100	194	3	93	2354	2271	1	0	3027

TRINITY_DN12299_c0_g1_i1	Glyma.09G179500.1	99.31972789	175	6	267	2530	2273	1	0	3158

TRINITY_DN12299_c0_g1_i2	Glyma.09G179500.1	99.31271478	175	6	267	2530	2273	1	0	3158

TRINITY_DN8602_c0_g1_i1	Glyma.19G163300.1	98.5915493	170	10	185	2451	2271	11	0	3129

TRINITY_DN11599_c0_g1_i3	Glyma.20G215200.1	97.10144928	164	15	456	2719	2240	1	0	3007

TRINITY_DN11599_c0_g1_i5	Glyma.20G215200.1	95.70135747	164	15	456	2719	2240	1	0	3007

TRINITY_DN11599_c0_g1_i6	Glyma.20G215200.1	98.74213836	164	15	456	2719	2240	1	0	3007

TRINITY_DN11599_c0_g1_i3	Glyma.20G215200.3	96.31675875	162	24	456	2719	2222	1	0	2902

TRINITY_DN11599_c0_g1_i5	Glyma.20G215200.3	99.0990991	162	24	456	2719	2222	1	0	2902

TRINITY_DN11599_c0_g1_i6	Glyma.20G215200.3	100	162	24	456	2719	2222	1	0	2902

TRINITY_DN37991_c0_g1_i1	Glyma.08G311100.1	97.27722772	150	16	332	2578	2268	1	0	3155

TRINITY_DN16245_c0_g3_i6	Glyma.04G144900.1	95.83333333	501	40	773	3031	3066	835	0	1066

TRINITY_DN3744_c0_g1_i1	Glyma.05G053200.1	98.63636364	256	27	222	2451	2268	3	0	2457

TRINITY_DN16353_c0_g1_i1	Glyma.19G068800.1	100	247	14	796	3047	695	2961	0	2654

TRINITY_DN16353_c0_g1_i8	Glyma.19G068800.1	95.83333333	247	14	714	2965	695	2961	0	2654

TRINITY_DN14621_c1_g1_i1	Glyma.19G055500.1	95.35864979	225	20	314	2575	3	2262	0	2796

TRINITY_DN14675_c0_g1_i3	Glyma.16G217300.1	99.11894273	218	2	95	2364	2547	278	0	2952

TRINITY_DN14621_c1_g1_i3	Glyma.19G055500.1	96.66666667	188	20	314	2575	3	2262	0	3001

TRINITY_DN15423_c0_g1_i1	Glyma.12G083500.1	98.6013986	468	35	450	2699	342	2596	0	1351

TRINITY_DN15423_c0_g1_i3	Glyma.12G083500.1	98.03921569	468	35	450	2699	342	2596	0	1351

TRINITY_DN15122_c0_g2_i2	Glyma.18G035700.2	98.7654321	412	24	598	2852	4267	2010	0	1712

TRINITY_DN15345_c0_g1_i3	Glyma.06G305000.1	96.875	385	28	507	2758	120	2377	0	1844

TRINITY_DN15345_c0_g1_i3	Glyma.06G305000.3	98.82352941	385	28	507	2758	120	2377	0	1844

TRINITY_DN15345_c0_g1_i4	Glyma.06G305000.1	96.11650485	385	28	313	2564	120	2377	0	1844

TRINITY_DN15345_c0_g1_i4	Glyma.06G305000.3	98.11320755	385	28	313	2564	120	2377	0	1844

TRINITY_DN14621_c1_g1_i4	Glyma.19G055500.1	100	200	14	336	2599	1	2262	0	2972

TRINITY_DN16082_c0_g1_i11	Glyma.05G166400.2	97.36842105	191	6	173	2437	2271	1	0	3086

TRINITY_DN14692_c0_g1_i2	Glyma.12G045800.1	95.45454545	170	3	115	2383	1	2266	0	3195

TRINITY_DN14621_c1_g1_i2	Glyma.19G055500.1	100	163	14	336	2599	1	2262	0	3177

TRINITY_DN7939_c0_g1_i1	Glyma.16G128300.2	100	162	2	353	2621	39	2307	0	3260

TRINITY_DN7939_c0_g1_i2	Glyma.16G128300.2	100	155	13	353	2594	39	2307	0	3123

TRINITY_DN12659_c1_g1_i1	Glyma.01G140600.2	98.11133201	122	2	235	2505	2271	1	0	3507

TRINITY_DN13423_c0_g2_i1	Glyma.09G037900.2	100	327	18	1101	3359	2248	5	0	2130

TRINITY_DN13423_c0_g2_i1	Glyma.09G037900.3	97.34219269	327	18	1101	3359	2248	5	0	2130

TRINITY_DN16069_c0_g1_i1	Glyma.05G191600.1	100	266	9	2	2267	199	2464	0	2656

TRINITY_DN12988_c0_g1_i1	Glyma.03G167500.1	95.33898305	232	8	1	2267	408	2674	0	2857

TRINITY_DN13489_c0_g1_i1	Glyma.13G345500.1	100	223	5	1035	3299	2265	1	0	2881

TRINITY_DN13489_c0_g1_i1	Glyma.13G345500.2	100	223	5	1035	3299	2265	1	0	2881

TRINITY_DN13489_c0_g1_i2	Glyma.13G345500.1	100	222	5	1278	3542	2265	1	0	2887

TRINITY_DN13489_c0_g1_i2	Glyma.13G345500.2	100	222	5	1278	3542	2265	1	0	2887

TRINITY_DN16354_c0_g1_i4	Glyma.20G192700.1	95	204	1	2921	5188	31	2301	0	3044

TRINITY_DN13489_c0_g1_i3	Glyma.11G126000.1	95.84245077	202	21	302	2542	2229	1	0	2610

TRINITY_DN13489_c0_g1_i3	Glyma.11G126000.2	97.13261649	202	21	302	2542	2229	1	0	2610

TRINITY_DN13489_c0_g1_i3	Glyma.11G126000.3	100	202	21	302	2542	2229	1	0	2610

TRINITY_DN13489_c0_g1_i3	Glyma.12G050600.1	95.45454545	189	22	302	2542	2229	1	0	2682

TRINITY_DN13489_c0_g1_i3	Glyma.12G050600.2	95.83333333	188	26	302	2542	2217	1	0	2610

TRINITY_DN11599_c0_g1_i3	Glyma.10G175300.1	96.42857143	166	10	456	2719	2252	1	0	3107

TRINITY_DN11599_c0_g1_i5	Glyma.10G175300.1	100	166	10	456	2719	2252	1	0	3107

TRINITY_DN11599_c0_g1_i6	Glyma.10G175300.1	100	166	10	456	2719	2252	1	0	3107

TRINITY_DN11599_c0_g1_i3	Glyma.10G175300.3	100	165	9	456	2719	2246	1	0	3073

TRINITY_DN11599_c0_g1_i5	Glyma.10G175300.3	98.7012987	165	9	456	2719	2246	1	0	3073

TRINITY_DN11599_c0_g1_i6	Glyma.10G175300.3	98.74213836	165	9	456	2719	2246	1	0	3073

TRINITY_DN12659_c1_g1_i1	Glyma.03G026900.2	98.08917197	126	0	235	2505	2271	1	0	3496

TRINITY_DN12659_c1_g1_i1	Glyma.03G026900.1	98.11320755	126	0	235	2505	2271	1	0	3496

TRINITY_DN16379_c0_g1_i2	Glyma.10G057100.1	97.36842105	409	4	471	2738	2328	61	0	1901

TRINITY_DN14604_c0_g1_i5	Glyma.16G116000.1	95.85365854	388	10	242	2505	25	2288	0	1965

TRINITY_DN49_c0_g1_i1	Glyma.20G217700.6	100	273	21	319	2550	3183	931	0	2324

TRINITY_DN12988_c0_g1_i1	Glyma.19G168600.1	100	237	6	1	2267	399	2665	0	2841

TRINITY_DN9796_c0_g1_i1	Glyma.11G035800.1	98.31932773	209	10	296	2553	3093	830	0	2918

TRINITY_DN11599_c0_g1_i3	Glyma.10G175300.2	95.46827795	164	10	456	2719	2249	1	0	3109

TRINITY_DN11599_c0_g1_i5	Glyma.10G175300.2	96.42401022	164	10	456	2719	2249	1	0	3109

TRINITY_DN11599_c0_g1_i6	Glyma.10G175300.2	100	164	10	456	2719	2249	1	0	3109

TRINITY_DN11599_c0_g1_i3	Glyma.10G175300.4	100	163	9	456	2719	2243	1	0	3075

TRINITY_DN11599_c0_g1_i5	Glyma.10G175300.4	100	163	9	456	2719	2243	1	0	3075

TRINITY_DN11599_c0_g1_i6	Glyma.10G175300.4	98.74213836	163	9	456	2719	2243	1	0	3075

TRINITY_DN13023_c0_g1_i1	Glyma.16G201700.1	97.51552795	301	5	350	2608	70	2337	0	2451

TRINITY_DN13389_c0_g1_i1	Glyma.15G035600.1	96.2962963	235	15	179	2434	129	2385	0	2726

TRINITY_DN12865_c0_g1_i2	Glyma.08G010300.2	100	209	24	220	2463	1	2253	0	2767

TRINITY_DN12299_c0_g1_i1	Glyma.07G099400.3	99.30555556	183	6	272	2530	2268	1	0	3105

TRINITY_DN12299_c0_g1_i1	Glyma.07G099400.1	99.30555556	183	6	272	2530	2268	1	0	3105

TRINITY_DN12299_c0_g1_i2	Glyma.07G099400.3	99.3006993	183	6	272	2530	2268	1	0	3105

TRINITY_DN12299_c0_g1_i2	Glyma.07G099400.1	99.31034483	183	6	272	2530	2268	1	0	3105

TRINITY_DN14365_c0_g1_i1	Glyma.14G001200.1	97.18309859	183	24	224	2481	19	2231	0	2743

TRINITY_DN7958_c0_g2_i1	Glyma.07G157900.1	100	170	13	438	2675	2265	1	0	3022

TRINITY_DN7958_c0_g2_i1	Glyma.20G005700.1	100	162	17	438	2675	2259	1	0	3027

TRINITY_DN14692_c0_g1_i2	Glyma.11G120700.1	96.15384615	159	3	115	2380	1	2263	0	3251

TRINITY_DN12678_c0_g1_i1	Glyma.12G129800.2	95.29411765	116	3	1017	3281	1	2268	0	3515

TRINITY_DN8905_c0_g1_i3	Glyma.11G079200.1	97.07317073	491	42	122	2366	18	2256	0	1133

TRINITY_DN15302_c0_g1_i2	Glyma.17G067800.1	98.85057471	263	16	377	2627	1	2266	0	2608

TRINITY_DN15302_c0_g1_i3	Glyma.17G067800.2	100	263	16	271	2521	1	2266	0	2608

TRINITY_DN15302_c0_g1_i4	Glyma.17G067800.2	99.12280702	263	16	377	2627	1	2266	0	2608

TRINITY_DN13489_c0_g1_i1	Glyma.15G028800.2	98.95833333	232	6	1035	3299	2259	1	0	2826

TRINITY_DN13489_c0_g1_i2	Glyma.15G028800.2	100	231	6	1278	3542	2259	1	0	2832

TRINITY_DN12988_c0_g1_i1	Glyma.10G040500.1	95.0617284	214	2	1	2267	387	2653	0	2990

TRINITY_DN16354_c0_g1_i4	Glyma.20G192700.4	95	203	1	2924	5188	1	2268	0	3044

TRINITY_DN16354_c0_g1_i4	Glyma.20G192700.5	95	203	1	2924	5188	1	2268	0	3044

TRINITY_DN16354_c0_g1_i4	Glyma.20G192700.3	95	203	1	2924	5188	1	2268	0	3044

TRINITY_DN16354_c0_g1_i4	Glyma.20G192700.2	95	203	1	2924	5188	1	2268	0	3044

TRINITY_DN13039_c0_g2_i5	Glyma.02G303300.1	99.2248062	185	1	139	2400	1	2268	0	3125

TRINITY_DN15549_c0_g1_i6	Glyma.05G247000.5	100	180	13	391	2618	2262	1	0	2894

TRINITY_DN15549_c0_g1_i7	Glyma.05G247000.5	97.06790123	180	13	391	2618	2262	1	0	2894

TRINITY_DN15549_c0_g1_i6	Glyma.08G055400.4	100	178	9	391	2618	2265	1	0	2924

TRINITY_DN15549_c0_g1_i7	Glyma.08G055400.4	97.5	178	9	391	2618	2265	1	0	2924

TRINITY_DN11176_c0_g1_i1	Glyma.08G094100.1	99.2248062	176	24	295	2533	142	2385	0	2870

TRINITY_DN15538_c0_g1_i6	Glyma.08G333900.3	98.79518072	169	0	997	3264	460	2727	0	3253

TRINITY_DN15538_c0_g1_i6	Glyma.08G333900.1	97.36842105	169	0	997	3264	460	2727	0	3253

TRINITY_DN15538_c0_g1_i6	Glyma.08G333900.2	100	169	0	997	3264	460	2727	0	3253

TRINITY_DN16435_c0_g2_i1	Glyma.05G165300.1	97.98657718	168	8	155	2407	1	2247	0	3025

TRINITY_DN16435_c0_g2_i4	Glyma.05G165300.1	97.61904762	167	8	155	2407	1	2247	0	3031

TRINITY_DN15878_c0_g2_i2	Glyma.16G017400.5	97.05882353	167	12	491	2743	2253	1	0	3070

TRINITY_DN15878_c0_g2_i2	Glyma.16G017400.1	99.31506849	167	12	491	2743	2253	1	0	3070

TRINITY_DN15878_c0_g2_i2	Glyma.16G017400.4	100	167	12	491	2743	2253	1	0	3070

TRINITY_DN15878_c0_g2_i2	Glyma.16G017400.6	100	167	12	491	2743	2253	1	0	3070

TRINITY_DN15878_c0_g2_i2	Glyma.16G017400.3	95.6043956	167	12	491	2743	2253	1	0	3070

TRINITY_DN15878_c0_g2_i2	Glyma.16G017400.2	99.31192661	167	12	491	2743	2253	1	0	3070

TRINITY_DN15538_c0_g1_i6	Glyma.18G067000.3	98	162	0	997	3264	460	2727	0	3291

TRINITY_DN15538_c0_g1_i6	Glyma.18G067000.2	97.72727273	162	0	997	3264	460	2727	0	3291

TRINITY_DN15538_c0_g1_i6	Glyma.18G067000.1	98.7804878	162	0	997	3264	460	2727	0	3291

TRINITY_DN13039_c0_g2_i5	Glyma.14G010500.1	100	162	2	139	2400	1	2268	0	3253

TRINITY_DN5517_c0_g1_i1	Glyma.13G259700.5	95.41284404	160	6	861	3123	1	2262	0	3230

TRINITY_DN5517_c0_g1_i4	Glyma.13G259700.5	98.14814815	160	6	938	3200	1	2262	0	3230

TRINITY_DN11644_c0_g2_i1	Glyma.13G135900.1	100	144	1	313	2577	2268	1	0	3371

TRINITY_DN11644_c0_g2_i2	Glyma.13G135900.1	97.26027397	144	1	313	2577	2268	1	0	3371

TRINITY_DN37991_c0_g1_i1	Glyma.18G104500.3	97.2972973	140	21	332	2578	2241	1	0	3103

TRINITY_DN37991_c0_g1_i1	Glyma.18G104500.1	97.2972973	140	21	332	2578	2241	1	0	3103

TRINITY_DN11393_c0_g1_i1	Glyma.17G129100.1	100	133	0	344	2611	2268	1	0	3452

TRINITY_DN11393_c0_g1_i1	Glyma.05G047300.1	97.05882353	128	0	344	2611	2268	1	0	3480

TRINITY_DN11644_c0_g2_i1	Glyma.10G048100.1	99.07407407	127	4	313	2577	2262	1	0	3426

TRINITY_DN11644_c0_g2_i2	Glyma.10G048100.1	100	127	4	313	2577	2262	1	0	3426

TRINITY_DN13023_c0_g1_i2	Glyma.09G150300.1	98.37962963	348	12	356	2611	61	2322	0	2156

TRINITY_DN15872_c0_g1_i2	Glyma.11G106800.1	99.28400955	346	17	312	2557	2313	56	0	2076

TRINITY_DN16082_c0_g1_i7	Glyma.08G202000.4	97.69820972	215	13	188	2431	2265	1	0	2833

TRINITY_DN16082_c0_g1_i9	Glyma.08G202000.4	95.18072289	191	13	142	2385	2265	1	0	2966

TRINITY_DN16049_c1_g2_i4	Glyma.08G102600.1	95.83333333	178	16	626	2878	2236	1	0	2909

TRINITY_DN13972_c0_g1_i1	Glyma.11G212700.1	96.42857143	156	0	557	2823	2267	1	0	3323

TRINITY_DN5408_c0_g1_i1	Glyma.11G191000.3	95.33333333	115	1	763	3026	2267	1	0	3530

TRINITY_DN16131_c0_g4_i1	Glyma.08G161500.1	100	537	46	225	2464	8	2244	0	854

TRINITY_DN13991_c0_g1_i2	Glyma.13G111500.1	96.72131148	237	10	294	2547	2263	1	0	2774

TRINITY_DN13991_c0_g1_i2	Glyma.17G048100.1	96.72131148	225	11	294	2547	2263	1	0	2841

TRINITY_DN15919_c0_g1_i2	Glyma.06G164300.9	97.37991266	207	10	1181	3427	2259	1	0	2870

TRINITY_DN15919_c0_g1_i2	Glyma.06G164300.1	95.36423841	207	10	1181	3427	2259	1	0	2870

TRINITY_DN15919_c0_g1_i2	Glyma.06G164300.7	95.36423841	207	10	1181	3427	2259	1	0	2870

TRINITY_DN15919_c0_g1_i2	Glyma.06G164300.8	95.36423841	207	10	1181	3427	2259	1	0	2870

TRINITY_DN15919_c0_g1_i2	Glyma.06G164300.5	97.35449735	207	11	1181	3427	2256	1	0	2850

TRINITY_DN39157_c0_g1_i1	Glyma.19G086700.1	96.875	182	6	167	2425	2265	1	0	3125

TRINITY_DN16425_c0_g1_i3	Glyma.12G060600.5	96.66666667	172	3	835	3096	2751	487	0	3199

TRINITY_DN16425_c0_g1_i1	Glyma.12G060600.5	98.16971714	170	3	902	3163	2751	487	0	3210

TRINITY_DN11810_c0_g1_i2	Glyma.11G125100.1	97.56097561	169	11	459	2721	3651	1404	0	3112

TRINITY_DN15878_c0_g2_i2	Glyma.07G048500.2	97.4025974	166	10	491	2743	2253	1	0	3097

TRINITY_DN15878_c0_g2_i2	Glyma.07G048500.1	97.4025974	166	10	491	2743	2253	1	0	3097

TRINITY_DN15878_c0_g2_i2	Glyma.07G048500.3	98.57142857	166	10	491	2743	2253	1	0	3097

TRINITY_DN15878_c0_g2_i2	Glyma.07G048500.5	98.24561404	166	10	491	2743	2253	1	0	3097

TRINITY_DN15878_c0_g2_i2	Glyma.07G048500.6	97.39130435	166	10	491	2743	2253	1	0	3097

TRINITY_DN15878_c0_g2_i2	Glyma.07G048500.7	97.39130435	166	10	491	2743	2253	1	0	3097

TRINITY_DN15878_c0_g2_i2	Glyma.07G048500.4	97.05882353	166	10	491	2743	2253	1	0	3097

TRINITY_DN16435_c0_g2_i1	Glyma.08G122700.1	96.17486339	156	21	155	2407	1	2223	0	2959

TRINITY_DN16435_c0_g2_i4	Glyma.08G122700.1	96.17834395	155	21	155	2407	1	2223	0	2964

TRINITY_DN13858_c0_g1_i1	Glyma.13G088000.1	100	311	19	61	2307	69	2321	0	2266

TRINITY_DN15919_c0_g1_i3	Glyma.06G164300.9	100	255	14	1189	3433	2248	1	0	2531

TRINITY_DN15919_c0_g1_i3	Glyma.06G164300.1	98.52125693	255	14	1189	3433	2248	1	0	2531

TRINITY_DN15919_c0_g1_i3	Glyma.06G164300.7	97.26027397	255	14	1189	3433	2248	1	0	2531

TRINITY_DN15919_c0_g1_i3	Glyma.06G164300.8	97.26027397	255	14	1189	3433	2248	1	0	2531

TRINITY_DN15919_c0_g1_i3	Glyma.06G164300.5	98.61111111	255	15	1189	3433	2245	1	0	2512

TRINITY_DN11599_c0_g1_i3	Glyma.10G175300.5	100	170	15	456	2719	2234	1	0	3035

TRINITY_DN11599_c0_g1_i5	Glyma.10G175300.5	95.70093458	170	15	456	2719	2234	1	0	3035

TRINITY_DN11599_c0_g1_i6	Glyma.10G175300.5	98.74213836	170	15	456	2719	2234	1	0	3035

TRINITY_DN11599_c0_g1_i3	Glyma.10G175300.7	100	169	16	456	2719	2228	1	0	3001

TRINITY_DN11599_c0_g1_i5	Glyma.10G175300.7	99.0990991	169	16	456	2719	2228	1	0	3001

TRINITY_DN11599_c0_g1_i6	Glyma.10G175300.7	97.32142857	169	16	456	2719	2228	1	0	3001

TRINITY_DN15841_c0_g1_i5	Glyma.12G074800.3	97.69585253	167	3	144	2408	1	2247	0	3142

TRINITY_DN15841_c0_g1_i6	Glyma.12G074800.3	99.01960784	167	3	144	2408	1	2247	0	3142

TRINITY_DN7276_c0_g1_i1	Glyma.01G001300.1	95.4887218	153	2	256	2519	2264	1	0	3323

TRINITY_DN7276_c0_g1_i2	Glyma.01G001300.1	98.30508475	153	2	256	2519	2264	1	0	3323

TRINITY_DN7276_c0_g1_i3	Glyma.01G001300.1	100	153	2	256	2519	2264	1	0	3323

TRINITY_DN9745_c0_g1_i2	Glyma.02G238300.2	100	142	16	458	2704	2247	1	0	3164

TRINITY_DN9745_c0_g1_i2	Glyma.02G238300.1	100	142	16	458	2704	2247	1	0	3164

TRINITY_DN9745_c0_g1_i4	Glyma.02G238300.2	100	142	16	334	2580	2247	1	0	3164

TRINITY_DN9745_c0_g1_i4	Glyma.02G238300.1	100	142	16	334	2580	2247	1	0	3164

TRINITY_DN10739_c0_g2_i1	Glyma.10G163600.3	100	133	6	157	2415	2259	1	0	3369

TRINITY_DN10739_c0_g2_i1	Glyma.10G163600.2	100	133	6	157	2415	2259	1	0	3369

TRINITY_DN10739_c0_g2_i1	Glyma.10G163600.1	100	133	6	157	2415	2259	1	0	3369

TRINITY_DN10739_c0_g2_i1	Glyma.20G226200.1	100	123	7	157	2415	2253	1	0	3386

TRINITY_DN13489_c0_g1_i1	Glyma.15G028800.1	98.95833333	231	6	1039	3299	2255	1	0	2824

TRINITY_DN13489_c0_g1_i2	Glyma.15G028800.1	100	231	6	1282	3542	2255	1	0	2824

TRINITY_DN9796_c0_g1_i1	Glyma.01G207400.1	98.30508475	226	5	296	2553	2886	638	0	2793

TRINITY_DN11810_c0_g1_i2	Glyma.12G049700.1	97.56097561	176	8	459	2721	3636	1392	0	3077

TRINITY_DN11810_c0_g1_i2	Glyma.12G049700.2	97.56097561	175	11	459	2721	3633	1392	0	3062

TRINITY_DN11599_c0_g1_i3	Glyma.10G175300.6	100	168	15	456	2719	2231	1	0	3037

TRINITY_DN11599_c0_g1_i5	Glyma.10G175300.6	99.23664122	168	15	456	2719	2231	1	0	3037

TRINITY_DN11599_c0_g1_i6	Glyma.10G175300.6	98.74213836	168	15	456	2719	2231	1	0	3037

TRINITY_DN11599_c0_g1_i3	Glyma.10G175300.8	97.10144928	167	16	456	2719	2225	1	0	3003

TRINITY_DN11599_c0_g1_i5	Glyma.10G175300.8	96.44670051	167	16	456	2719	2225	1	0	3003

TRINITY_DN11599_c0_g1_i6	Glyma.10G175300.8	98.74213836	167	16	456	2719	2225	1	0	3003

TRINITY_DN12949_c0_g1_i1	Glyma.19G212800.2	100	137	0	519	2782	2264	1	0	3422

TRINITY_DN12949_c0_g1_i1	Glyma.03G216300.2	99.17355372	136	0	519	2782	2264	1	0	3428

TRINITY_DN13023_c0_g1_i1	Glyma.09G150300.1	99.31506849	314	9	356	2608	61	2322	0	2368

TRINITY_DN35889_c0_g1_i1	Glyma.19G254900.2	98.4375	270	26	190	2423	2271	23	0	2405

TRINITY_DN11646_c0_g1_i2	Glyma.17G229000.1	100	217	15	228	2476	2406	155	0	2815

TRINITY_DN11646_c0_g1_i2	Glyma.17G229000.2	100	217	15	228	2476	2343	92	0	2815

TRINITY_DN14933_c1_g2_i1	Glyma.09G060500.1	98.08429119	214	47	64	2244	391	2619	0	2244

TRINITY_DN15919_c0_g1_i2	Glyma.06G164300.3	96.96969697	208	9	1181	3427	2256	1	0	2878

TRINITY_DN15919_c0_g1_i2	Glyma.06G164300.4	100	208	9	1181	3427	2256	1	0	2878

TRINITY_DN15919_c0_g1_i2	Glyma.06G164300.6	98.06451613	208	9	1181	3427	2256	1	0	2878

TRINITY_DN15919_c0_g1_i2	Glyma.06G164300.2	98.7804878	208	10	1181	3427	2253	1	0	2859

TRINITY_DN16049_c1_g2_i4	Glyma.05G145800.1	97.88867562	183	7	626	2878	2239	1	0	2946

TRINITY_DN16049_c1_g2_i4	Glyma.05G145800.2	97.89103691	183	7	626	2878	2239	1	0	2946

TRINITY_DN16435_c0_g2_i1	Glyma.08G122700.2	96.17486339	156	20	155	2407	1	2220	0	2972

TRINITY_DN16435_c0_g2_i4	Glyma.08G122700.2	97.61904762	155	20	155	2407	1	2220	0	2977

TRINITY_DN12617_c0_g1_i2	Glyma.13G104300.1	98.14814815	355	16	1982	4222	1242	3500	0	2056

TRINITY_DN12617_c0_g1_i2	Glyma.13G104300.18	100	355	16	1982	4222	1236	3494	0	2056

TRINITY_DN12617_c0_g1_i2	Glyma.13G104300.17	100	355	16	1982	4222	1218	3476	0	2056

TRINITY_DN12617_c0_g1_i2	Glyma.13G104300.15	100	355	16	1982	4222	1116	3374	0	2056

TRINITY_DN13448_c0_g1_i6	Glyma.11G246200.4	98.27586207	314	0	128	2389	1	2262	0	2438

TRINITY_DN13448_c0_g1_i6	Glyma.11G246200.5	97.68786127	314	0	128	2389	1	2262	0	2438

TRINITY_DN15919_c0_g1_i3	Glyma.06G164300.3	98.24561404	256	13	1189	3433	2245	1	0	2540

TRINITY_DN15919_c0_g1_i3	Glyma.06G164300.4	99.05660377	256	13	1189	3433	2245	1	0	2540

TRINITY_DN15919_c0_g1_i3	Glyma.06G164300.6	100	256	13	1189	3433	2245	1	0	2540

TRINITY_DN15919_c0_g1_i3	Glyma.06G164300.2	98.61111111	256	14	1189	3433	2242	1	0	2519

TRINITY_DN39157_c0_g1_i1	Glyma.16G060400.1	96.875	217	2	167	2425	2262	1	0	2955

TRINITY_DN16422_c0_g2_i3	Glyma.11G005300.1	98.7012987	177	8	1239	3498	831	3084	0	3133

TRINITY_DN2173_c0_g1_i1	Glyma.19G107300.1	97.31707317	134	2	25	2286	1	2259	0	3415

TRINITY_DN2173_c0_g1_i1	Glyma.19G107300.2	97.43589744	134	2	25	2286	1	2259	0	3415

TRINITY_DN2173_c0_g1_i1	Glyma.16G044600.1	97.31707317	128	2	25	2286	1	2259	0	3448

TRINITY_DN16141_c0_g1_i2	Glyma.06G003000.12	96.2962963	101	2	1594	3843	2262	1	0	3541

TRINITY_DN16141_c0_g1_i5	Glyma.06G003000.12	100	101	3	1594	3841	2262	1	0	3528

TRINITY_DN16354_c0_g1_i4	Glyma.08G027000.1	95	362	7	2924	5178	1	2258	0	2111

TRINITY_DN14151_c0_g1_i1	Glyma.09G253700.1	96.77419355	361	45	323	2547	107	2316	0	1613

TRINITY_DN18910_c0_g1_i1	Glyma.16G018900.1	97.19101124	321	23	1	2246	17	2253	0	2145

TRINITY_DN12617_c0_g1_i2	Glyma.13G104300.6	100	356	18	1982	4222	1242	3491	0	2013

TRINITY_DN12617_c0_g1_i2	Glyma.13G104300.16	100	356	18	1982	4222	1218	3467	0	2013

TRINITY_DN12617_c0_g1_i2	Glyma.13G104300.14	100	356	18	1982	4222	1116	3365	0	2013

TRINITY_DN18910_c0_g1_i1	Glyma.07G050200.1	97.19101124	327	22	1	2246	17	2259	0	2161

TRINITY_DN9899_c0_g1_i4	Glyma.07G057700.1	96	318	34	14	2241	458	2685	0	1999

TRINITY_DN9801_c0_g1_i1	Glyma.04G155800.1	95.221843	292	7	606	2853	2565	312	0	2440

TRINITY_DN6417_c0_g1_i1	Glyma.06G045600.1	98.64864865	193	6	265	2523	183	2426	0	2994

TRINITY_DN15423_c0_g1_i1	Glyma.11G190900.1	96.63865546	471	30	459	2699	347	2587	0	1330

TRINITY_DN15423_c0_g1_i1	Glyma.11G190900.2	100	471	30	459	2699	347	2587	0	1330

TRINITY_DN15423_c0_g1_i3	Glyma.11G190900.1	98.21428571	471	30	459	2699	347	2587	0	1330

TRINITY_DN15423_c0_g1_i3	Glyma.11G190900.2	100	471	30	459	2699	347	2587	0	1330

TRINITY_DN15872_c0_g1_i3	Glyma.11G106800.1	98.82352941	295	4	312	2566	2313	56	0	2505

TRINITY_DN16955_c0_g1_i1	Glyma.13G362100.1	95.0617284	192	13	45	2291	1	2247	0	2953

TRINITY_DN36076_c0_g1_i1	Glyma.06G107200.1	96.12403101	189	10	214	2460	2238	1	0	2911

TRINITY_DN2582_c0_g1_i1	Glyma.09G074300.2	96.15384615	161	2	947	3202	1	2259	0	3260

TRINITY_DN13088_c0_g1_i3	Glyma.20G156500.3	100	155	8	606	2849	1	2259	0	3216

TRINITY_DN13088_c0_g1_i3	Glyma.20G156500.4	97.28	155	8	606	2849	1	2259	0	3216

TRINITY_DN13088_c0_g1_i3	Glyma.20G156500.2	98.30508475	155	8	606	2849	1	2259	0	3216

TRINITY_DN9745_c0_g1_i2	Glyma.14G207200.1	100	142	5	458	2704	2253	1	0	3269

TRINITY_DN9745_c0_g1_i4	Glyma.14G207200.1	100	142	5	334	2580	2253	1	0	3269

TRINITY_DN16141_c0_g1_i2	Glyma.06G003000.11	96.32352941	101	1	1594	3843	2259	1	0	3554

TRINITY_DN16141_c0_g1_i5	Glyma.06G003000.11	100	101	2	1594	3841	2259	1	0	3541

TRINITY_DN9899_c0_g1_i4	Glyma.16G026700.1	100	322	34	14	2241	458	2679	0	1960

TRINITY_DN15808_c0_g1_i5	Glyma.04G163600.2	97.36842105	267	21	2227	4462	1971	4201	0	2374

TRINITY_DN15808_c0_g1_i5	Glyma.04G163600.1	97.4025974	267	21	2227	4462	1971	4201	0	2374

TRINITY_DN15808_c0_g1_i5	Glyma.04G163600.3	99.09090909	267	21	2227	4462	1971	4201	0	2374

TRINITY_DN14244_c0_g4_i1	Glyma.12G072700.2	96.19883041	244	10	609	2864	2250	1	0	2754

TRINITY_DN14675_c0_g1_i3	Glyma.09G167100.1	95.20958084	221	4	95	2346	2547	293	0	2887

TRINITY_DN11646_c0_g1_i2	Glyma.14G094500.1	95.55555556	182	13	228	2472	2334	93	0	2974

TRINITY_DN11646_c0_g1_i2	Glyma.14G094500.2	100	182	14	228	2472	2328	93	0	2935

TRINITY_DN14064_c0_g1_i1	Glyma.13G209800.1	96.40718563	110	0	317	2574	3967	1710	0	3561

TRINITY_DN14064_c0_g1_i1	Glyma.13G209800.2	96.96969697	110	0	317	2574	3397	1140	0	3561

TRINITY_DN14064_c0_g1_i1	Glyma.15G102800.1	96.15384615	103	0	317	2574	3949	1692	0	3600

TRINITY_DN16230_c0_g1_i5	Glyma.17G233900.1	100	255	19	80	2278	2263	24	0	2270

TRINITY_DN16148_c0_g1_i1	Glyma.02G290500.1	97.34513274	189	18	134	2367	2241	1	0	2868

TRINITY_DN14893_c0_g1_i1	Glyma.18G098800.1	99.15254237	183	12	265	2515	1	2239	0	3000

TRINITY_DN16148_c0_g1_i2	Glyma.02G290500.1	100	183	17	134	2380	2241	1	0	2987

TRINITY_DN14893_c0_g1_i1	Glyma.08G315300.1	99.15254237	180	10	265	2515	1	2248	0	3073

TRINITY_DN14893_c0_g1_i1	Glyma.08G315300.2	99.15254237	180	10	265	2515	1	2248	0	3073

TRINITY_DN15913_c1_g2_i2	Glyma.19G103200.2	100	150	1	340	2596	3003	753	0	3299

TRINITY_DN10739_c0_g2_i1	Glyma.03G248300.1	100	274	24	192	2420	2251	5	0	2416

TRINITY_DN2582_c0_g1_i1	Glyma.15G188300.2	97.6744186	159	0	947	3202	1	2256	0	3286

TRINITY_DN13088_c0_g1_i3	Glyma.10G238100.2	99.55752212	150	8	606	2849	1	2256	0	3258

TRINITY_DN13088_c0_g1_i3	Glyma.10G238100.3	99.55752212	150	8	606	2849	1	2256	0	3258

TRINITY_DN8558_c0_g1_i1	Glyma.10G001000.1	100	431	30	200	2430	2275	36	0	1524

TRINITY_DN15235_c0_g1_i6	Glyma.16G172400.1	96.3963964	400	11	3	2251	2264	4512	0	1871

TRINITY_DN15235_c0_g1_i6	Glyma.16G172400.4	97.39776952	400	11	3	2251	2222	4470	0	1871

TRINITY_DN15235_c0_g1_i6	Glyma.16G172400.3	97.39776952	400	11	3	2251	2195	4443	0	1871

TRINITY_DN15235_c0_g1_i6	Glyma.16G172400.2	97.99196787	400	11	3	2251	1472	3720	0	1871

TRINITY_DN15235_c0_g1_i6	Glyma.16G172400.5	97.99196787	400	11	3	2251	209	2457	0	1871

TRINITY_DN14151_c0_g1_i1	Glyma.09G253700.4	95.40229885	362	42	323	2547	107	2310	0	1635

TRINITY_DN8852_c0_g1_i2	Glyma.12G214200.1	100	226	10	36	2284	1	2243	0	2796

TRINITY_DN16082_c0_g1_i7	Glyma.08G202000.2	97.69736842	216	8	188	2431	2253	1	0	2883

TRINITY_DN16082_c0_g1_i9	Glyma.08G202000.2	95.18072289	192	8	142	2385	2253	1	0	3016

TRINITY_DN3954_c0_g2_i1	Glyma.10G167300.1	98.79518072	158	1	465	2719	53	2304	0	3269

TRINITY_DN3954_c0_g1_i1	Glyma.10G167300.1	96.64082687	158	2	465	2718	53	2304	0	3264

TRINITY_DN16163_c0_g1_i5	Glyma.13G308200.2	96.57320872	124	6	82	2333	104	2346	0	3380

TRINITY_DN16163_c0_g1_i5	Glyma.13G308200.1	100	124	6	82	2333	104	2346	0	3380

TRINITY_DN15235_c0_g1_i5	Glyma.16G172400.1	100	401	11	3	2250	2264	4511	0	1864

TRINITY_DN15235_c0_g1_i5	Glyma.16G172400.4	100	401	11	3	2250	2222	4469	0	1864

TRINITY_DN15235_c0_g1_i5	Glyma.16G172400.3	100	401	11	3	2250	2195	4442	0	1864

TRINITY_DN15235_c0_g1_i5	Glyma.16G172400.2	100	401	11	3	2250	1472	3719	0	1864

TRINITY_DN15235_c0_g1_i5	Glyma.16G172400.5	100	401	11	3	2250	209	2456	0	1864

TRINITY_DN15417_c0_g1_i1	Glyma.06G156500.1	95.29411765	332	27	468	2678	2768	519	0	2019

TRINITY_DN15417_c0_g1_i2	Glyma.06G156500.1	97.43589744	332	27	734	2944	2768	519	0	2019

TRINITY_DN12815_c1_g1_i3	Glyma.18G095200.1	96.9924812	319	32	714	2930	2604	391	0	1897

TRINITY_DN16230_c0_g1_i5	Glyma.17G233900.2	95.32374101	255	18	80	2278	2260	24	0	2285

TRINITY_DN9336_c0_g1_i1	Glyma.08G023800.3	99.35483871	194	8	1	2234	1335	3583	0	2926

TRINITY_DN9336_c0_g1_i1	Glyma.08G023800.4	95	194	8	1	2234	1335	3583	0	2926

TRINITY_DN9336_c0_g1_i1	Glyma.08G023800.1	100	194	8	1	2234	1335	3583	0	2926

TRINITY_DN9336_c0_g1_i1	Glyma.08G023800.2	100	194	8	1	2234	1323	3571	0	2926

TRINITY_DN9336_c0_g1_i1	Glyma.08G023800.5	97.6744186	194	8	1	2234	1323	3571	0	2926

TRINITY_DN9336_c0_g1_i1	Glyma.08G023800.6	98.38709677	194	8	1	2234	1323	3571	0	2926

TRINITY_DN9336_c0_g1_i1	Glyma.08G023800.8	96.47058824	194	8	1	2234	1128	3376	0	2926

TRINITY_DN9336_c0_g1_i1	Glyma.08G023800.7	97.10144928	194	8	1	2234	1116	3364	0	2926

TRINITY_DN16148_c0_g1_i1	Glyma.14G024300.1	97.34693878	190	21	134	2367	2232	1	0	2839

TRINITY_DN16148_c0_g1_i2	Glyma.14G024300.1	100	185	20	134	2380	2232	1	0	2950

TRINITY_DN5720_c0_g1_i1	Glyma.10G200700.1	100	174	2	350	2600	2307	54	0	3179

TRINITY_DN37703_c0_g1_i1	Glyma.09G202100.1	97.14285714	151	8	164	2405	2362	109	0	3249

TRINITY_DN15235_c0_g1_i6	Glyma.16G172600.1	98.7012987	395	4	1	2251	2196	4446	0	1947

TRINITY_DN15872_c0_g1_i2	Glyma.12G031800.1	98.51851852	320	23	312	2540	2322	82	0	2156

TRINITY_DN12815_c1_g1_i4	Glyma.18G095200.1	97	261	30	714	2930	2604	391	0	2230

TRINITY_DN16056_c0_g2_i1	Glyma.03G139100.1	95.57713053	198	3	511	2762	2337	89	0	3031

TRINITY_DN1847_c0_g1_i1	Glyma.20G100400.1	95.68345324	190	6	7	2244	38	2287	0	2992

TRINITY_DN16231_c1_g1_i2	Glyma.17G045800.3	99.32885906	151	0	80	2332	1	2253	0	3325

TRINITY_DN37991_c0_g1_i1	Glyma.08G311100.4	97.2972973	151	7	332	2578	2247	1	0	3247

TRINITY_DN37991_c0_g1_i1	Glyma.08G311100.3	97.2972973	151	7	332	2578	2247	1	0	3247

TRINITY_DN37991_c0_g1_i1	Glyma.08G311100.2	97.28813559	151	7	332	2578	2247	1	0	3247

TRINITY_DN16231_c1_g1_i2	Glyma.13G114000.4	97.95918367	145	0	80	2332	1	2253	0	3358

TRINITY_DN10529_c0_g2_i1	Glyma.08G185700.7	99.68454259	135	0	125	2377	1	2253	0	3413

TRINITY_DN14526_c0_g1_i1	Glyma.01G226700.1	100	133	9	291	2525	2253	1	0	3308

TRINITY_DN16163_c0_g1_i5	Glyma.12G194400.1	97.4522293	120	6	84	2333	106	2346	0	3398

TRINITY_DN16163_c0_g1_i5	Glyma.12G194400.9	97.36842105	120	6	84	2333	106	2346	0	3398

TRINITY_DN16163_c0_g1_i5	Glyma.12G194400.7	100	118	7	84	2333	106	2331	0	3314

TRINITY_DN16163_c0_g1_i5	Glyma.12G194400.8	100	118	7	84	2333	106	2331	0	3314

TRINITY_DN16141_c0_g1_i2	Glyma.06G003000.10	96.63157895	101	1	1594	3843	2253	1	0	3581

TRINITY_DN16141_c0_g1_i5	Glyma.06G003000.10	100	101	2	1594	3841	2253	1	0	3568

TRINITY_DN11155_c0_g1_i1	Glyma.15G093600.2	99.39759036	91	5	491	2737	2253	1	0	3618

TRINITY_DN15235_c0_g1_i5	Glyma.16G172600.1	100	396	4	1	2250	2196	4445	0	1940

TRINITY_DN15235_c0_g1_i6	Glyma.10G227400.6	100	302	2	1	2251	2190	4440	0	2473

TRINITY_DN15235_c0_g1_i6	Glyma.10G227400.4	100	302	2	1	2251	2190	4440	0	2473

TRINITY_DN15235_c0_g1_i6	Glyma.10G227400.3	100	302	2	1	2251	2190	4440	0	2473

TRINITY_DN15235_c0_g1_i6	Glyma.10G227400.1	97.36842105	302	2	1	2251	2190	4440	0	2473

TRINITY_DN15235_c0_g1_i6	Glyma.10G227400.2	100	302	2	1	2251	2190	4440	0	2473

TRINITY_DN15235_c0_g1_i6	Glyma.10G227400.5	100	302	2	1	2251	2190	4440	0	2473

TRINITY_DN9258_c1_g1_i1	Glyma.19G115800.6	100	256	4	1	2248	2251	1	0	2708

TRINITY_DN9258_c1_g1_i1	Glyma.19G115800.1	100	256	4	1	2248	2251	1	0	2708

TRINITY_DN9258_c1_g1_i1	Glyma.19G115800.3	100	256	4	1	2248	2251	1	0	2708

TRINITY_DN9258_c1_g1_i1	Glyma.19G115800.5	100	256	4	1	2248	2251	1	0	2708

TRINITY_DN9258_c1_g1_i1	Glyma.19G115800.2	95.34883721	256	4	1	2248	2251	1	0	2708

TRINITY_DN16231_c1_g1_i1	Glyma.17G045800.3	100	151	0	80	2331	1	2252	0	3323

TRINITY_DN16231_c1_g1_i2	Glyma.17G045800.2	100	151	0	80	2331	1	2252	0	3323

TRINITY_DN16231_c1_g1_i2	Glyma.17G045800.1	95.70247934	151	0	80	2331	1	2252	0	3323

TRINITY_DN16231_c1_g1_i1	Glyma.13G114000.4	100	145	0	80	2331	1	2252	0	3356

TRINITY_DN16231_c1_g1_i2	Glyma.13G114000.1	100	145	0	80	2331	1	2252	0	3356

TRINITY_DN16231_c1_g1_i2	Glyma.13G114000.2	100	145	0	80	2331	1	2252	0	3356

TRINITY_DN16231_c1_g1_i2	Glyma.13G114000.3	95.20153551	145	0	80	2331	1	2252	0	3356

TRINITY_DN15235_c0_g1_i5	Glyma.10G227400.6	95.91836735	303	2	1	2250	2190	4439	0	2466

TRINITY_DN15235_c0_g1_i5	Glyma.10G227400.4	95.91836735	303	2	1	2250	2190	4439	0	2466

TRINITY_DN15235_c0_g1_i5	Glyma.10G227400.3	99.42363112	303	2	1	2250	2190	4439	0	2466

TRINITY_DN15235_c0_g1_i5	Glyma.10G227400.1	95.23809524	303	2	1	2250	2190	4439	0	2466

TRINITY_DN15235_c0_g1_i5	Glyma.10G227400.2	100	303	2	1	2250	2190	4439	0	2466

TRINITY_DN15235_c0_g1_i5	Glyma.10G227400.5	100	303	2	1	2250	2190	4439	0	2466

TRINITY_DN15235_c0_g1_i6	Glyma.20G165000.1	99.5412844	293	2	2	2251	1474	3723	0	2521

TRINITY_DN15235_c0_g1_i6	Glyma.20G165000.2	100	293	2	2	2251	1474	3723	0	2521

TRINITY_DN16436_c0_g2_i13	Glyma.11G131600.1	97.07602339	224	5	534	2770	1	2249	0	2813

TRINITY_DN16019_c0_g1_i1	Glyma.06G013100.1	100	203	4	1	2250	2235	1	0	2922

TRINITY_DN16019_c0_g1_i11	Glyma.06G013100.1	97.61904762	196	4	1	2250	2235	1	0	2961

TRINITY_DN37703_c0_g1_i1	Glyma.01G020300.1	100	155	9	164	2405	2362	118	0	3201

TRINITY_DN15235_c0_g1_i6	Glyma.10G227300.5	99.62264151	142	3	1	2249	2202	4450	0	3345

TRINITY_DN15235_c0_g1_i6	Glyma.10G227300.4	99.62264151	142	3	1	2249	2202	4450	0	3345

TRINITY_DN15235_c0_g1_i6	Glyma.10G227300.3	99.62546816	142	3	1	2249	2202	4450	0	3345

TRINITY_DN15235_c0_g1_i6	Glyma.10G227300.1	99.62546816	142	3	1	2249	2202	4450	0	3345

TRINITY_DN15235_c0_g1_i6	Glyma.10G227300.2	99.5412844	142	3	1	2249	2202	4450	0	3345

TRINITY_DN15235_c0_g1_i5	Glyma.10G227300.5	96.68874172	141	3	1	2249	2202	4450	0	3350

TRINITY_DN15235_c0_g1_i5	Glyma.10G227300.4	100	141	3	1	2249	2202	4450	0	3350

TRINITY_DN15235_c0_g1_i5	Glyma.10G227300.3	100	141	3	1	2249	2202	4450	0	3350

TRINITY_DN15235_c0_g1_i5	Glyma.10G227300.1	98.61111111	141	3	1	2249	2202	4450	0	3350

TRINITY_DN15235_c0_g1_i5	Glyma.10G227300.2	100	141	3	1	2249	2202	4450	0	3350

TRINITY_DN14670_c0_g1_i2	Glyma.09G241100.1	98.55072464	135	2	530	2777	288	2538	0	3389

TRINITY_DN14670_c0_g1_i2	Glyma.09G241100.4	97.36842105	135	2	530	2777	288	2538	0	3389

TRINITY_DN14670_c0_g1_i2	Glyma.09G241100.2	98.21428571	135	2	530	2777	270	2520	0	3389

TRINITY_DN14670_c0_g1_i2	Glyma.09G241100.3	100	135	2	530	2777	270	2520	0	3389

TRINITY_DN15980_c0_g2_i1	Glyma.20G207700.3	97.13467049	408	44	3	2207	1030	3237	0	1332

TRINITY_DN15235_c0_g1_i5	Glyma.20G165000.1	100	294	2	2	2250	1474	3722	0	2514

TRINITY_DN15235_c0_g1_i5	Glyma.20G165000.2	96.33027523	294	2	2	2250	1474	3722	0	2514

TRINITY_DN13614_c0_g1_i1	Glyma.06G272200.1	98.46547315	222	7	348	2589	2242	1	0	2822

TRINITY_DN16019_c0_g1_i1	Glyma.04G013200.1	100	203	1	1	2250	2247	1	0	3011

TRINITY_DN16019_c0_g1_i11	Glyma.04G013200.1	97.61904762	196	1	1	2250	2247	1	0	3049

TRINITY_DN36489_c0_g1_i1	Glyma.06G206000.6	97.2972973	182	12	38	2270	543	2787	0	3005

TRINITY_DN36489_c0_g1_i1	Glyma.06G206000.5	100	182	12	38	2270	543	2787	0	3005

TRINITY_DN36489_c0_g1_i1	Glyma.06G206000.1	96.38554217	182	12	38	2270	543	2787	0	3005

TRINITY_DN36489_c0_g1_i1	Glyma.06G206000.4	100	182	12	38	2270	543	2787	0	3005

TRINITY_DN5720_c0_g1_i1	Glyma.20G189900.1	100	172	0	350	2599	2286	37	0	3203

TRINITY_DN16019_c0_g1_i11	Glyma.11G118600.1	97.61904762	165	0	1	2250	2250	1	0	3241

TRINITY_DN16019_c0_g1_i1	Glyma.11G118600.1	100	162	0	1	2250	2250	1	0	3258

TRINITY_DN16019_c0_g1_i11	Glyma.12G044000.1	97.61904762	158	0	1	2250	2250	1	0	3280

TRINITY_DN16019_c0_g1_i1	Glyma.12G044000.1	100	155	0	1	2250	2250	1	0	3297

TRINITY_DN11644_c0_g2_i1	Glyma.13G135900.2	100	144	1	313	2559	2250	1	0	3338

TRINITY_DN11644_c0_g2_i2	Glyma.13G135900.2	100	144	1	313	2559	2250	1	0	3338

TRINITY_DN14670_c0_g1_i2	Glyma.18G255000.2	97.05882353	132	2	531	2777	271	2520	0	3404

TRINITY_DN14670_c0_g1_i2	Glyma.18G255000.1	100	132	2	531	2777	271	2520	0	3404

TRINITY_DN16141_c0_g1_i2	Glyma.06G003000.9	97.56097561	101	0	1594	3843	2250	1	0	3596

TRINITY_DN16141_c0_g1_i5	Glyma.06G003000.9	95	101	1	1594	3841	2250	1	0	3583

TRINITY_DN16190_c0_g3_i2	Glyma.06G229900.2	96.2962963	361	9	271	2509	1	2245	0	2063

TRINITY_DN12815_c1_g1_i2	Glyma.18G095200.1	97	301	29	714	2927	2604	391	0	2034

TRINITY_DN15718_c0_g1_i3	Glyma.05G186900.1	100	275	2	582	2829	2248	1	0	2617

TRINITY_DN15718_c0_g1_i5	Glyma.05G186900.1	97.5	275	2	663	2910	2248	1	0	2617

TRINITY_DN49_c0_g1_i1	Glyma.20G217700.3	100	271	21	340	2550	3162	931	0	2296

TRINITY_DN49_c0_g1_i1	Glyma.20G217700.1	95.91836735	271	21	340	2550	3162	931	0	2296

TRINITY_DN49_c0_g2_i1	Glyma.20G217700.6	98.55072464	271	21	1255	3465	3162	931	0	2296

TRINITY_DN9258_c1_g1_i1	Glyma.16G037500.2	95.34883721	257	9	1	2240	2254	9	0	2652

TRINITY_DN9258_c1_g1_i1	Glyma.16G037500.5	95.92641261	257	9	1	2240	2254	9	0	2652

TRINITY_DN9258_c1_g1_i1	Glyma.16G037500.3	99.21875	257	9	1	2240	2254	9	0	2652

TRINITY_DN9258_c1_g1_i1	Glyma.16G037500.1	99.21875	257	9	1	2240	2254	9	0	2652

TRINITY_DN16436_c0_g2_i13	Glyma.11G131700.1	97.52475248	219	0	523	2771	44	2292	0	2940

TRINITY_DN16436_c0_g2_i13	Glyma.11G131700.2	96.65970772	219	0	523	2771	23	2271	0	2940

TRINITY_DN16082_c0_g1_i7	Glyma.07G016700.2	95.56650246	212	11	188	2406	2247	1	0	2772

TRINITY_DN8900_c0_g1_i1	Glyma.18G026600.1	95.94594595	206	7	81	2320	604	2835	0	2844

TRINITY_DN16082_c0_g1_i9	Glyma.07G016700.2	95.18072289	184	11	142	2360	2247	1	0	2928

TRINITY_DN14463_c0_g1_i1	Glyma.04G230800.1	100	161	13	311	2546	22	2260	0	3112

TRINITY_DN14463_c0_g1_i1	Glyma.04G230800.4	98.97959184	161	14	311	2546	22	2254	0	3073

TRINITY_DN16163_c0_g1_i4	Glyma.13G308200.2	97.84172662	258	13	82	2313	104	2344	0	2573

TRINITY_DN16163_c0_g1_i4	Glyma.13G308200.1	100	258	13	82	2313	104	2344	0	2573

TRINITY_DN12815_c1_g1_i1	Glyma.18G095200.1	96.98412698	243	27	714	2927	2604	391	0	2366

TRINITY_DN16040_c0_g1_i2	Glyma.20G054600.1	100	209	7	610	2854	2250	7	0	2948

TRINITY_DN11834_c0_g1_i2	Glyma.17G117500.1	97.56097561	208	27	345	2564	2205	1	0	2540

TRINITY_DN16415_c1_g1_i7	Glyma.U018900.2	95.83333333	202	5	1364	3611	2239	1	0	2974

TRINITY_DN16040_c0_g1_i3	Glyma.20G054600.1	98.0952381	198	7	610	2854	2250	7	0	3009

TRINITY_DN15718_c0_g1_i3	Glyma.04G217300.3	100	174	0	582	2829	2248	1	0	3188

TRINITY_DN15718_c0_g1_i3	Glyma.04G217300.1	98.7804878	174	0	582	2829	2248	1	0	3188

TRINITY_DN15718_c0_g1_i5	Glyma.04G217300.3	100	174	0	663	2910	2248	1	0	3188

TRINITY_DN15718_c0_g1_i5	Glyma.04G217300.1	98.57142857	174	0	663	2910	2248	1	0	3188

TRINITY_DN14378_c0_g2_i1	Glyma.06G039600.6	95.89041096	173	19	532	2749	636	2853	0	2806

TRINITY_DN14378_c0_g2_i1	Glyma.06G039600.3	95.59471366	173	19	532	2749	636	2853	0	2806

TRINITY_DN14378_c0_g2_i1	Glyma.06G039600.1	97.79411765	173	19	532	2749	636	2853	0	2806

TRINITY_DN14378_c0_g2_i1	Glyma.06G039600.7	100	173	19	532	2749	636	2853	0	2806

TRINITY_DN16415_c1_g1_i7	Glyma.11G147900.3	100	172	2	1364	3611	2389	148	0	3160

TRINITY_DN16415_c1_g1_i7	Glyma.11G147900.2	100	172	2	1364	3611	2242	1	0	3160

TRINITY_DN15718_c0_g1_i3	Glyma.06G148500.1	100	171	0	582	2829	2248	1	0	3205

TRINITY_DN15718_c0_g1_i5	Glyma.06G148500.1	95.20547945	171	0	663	2910	2248	1	0	3205

TRINITY_DN16190_c0_g3_i4	Glyma.06G229900.2	100	452	18	348	2580	10	2245	0	1483

TRINITY_DN16314_c1_g1_i2	Glyma.02G080900.1	100	443	35	1833	4062	2655	435	0	1417

TRINITY_DN12617_c0_g1_i1	Glyma.13G104300.5	98.50746269	304	11	2303	4538	1362	3606	0	2381

TRINITY_DN12617_c0_g1_i1	Glyma.13G104300.13	98.50746269	304	11	2303	4538	1356	3600	0	2381

TRINITY_DN12617_c0_g1_i1	Glyma.13G104300.12	98.50187266	304	11	2303	4538	1338	3582	0	2381

TRINITY_DN49_c0_g1_i1	Glyma.20G217700.5	99.09090909	271	21	342	2550	3160	931	0	2292

TRINITY_DN49_c0_g2_i1	Glyma.20G217700.5	98.79518072	271	21	1257	3465	3160	931	0	2292

TRINITY_DN16163_c0_g1_i4	Glyma.12G194400.1	97.84172662	254	14	84	2313	106	2344	0	2580

TRINITY_DN16163_c0_g1_i4	Glyma.12G194400.9	97.84172662	254	14	84	2313	106	2344	0	2580

TRINITY_DN16163_c0_g1_i4	Glyma.12G194400.7	100	251	16	84	2313	106	2329	0	2501

TRINITY_DN16163_c0_g1_i4	Glyma.12G194400.8	100	251	16	84	2313	106	2329	0	2501

TRINITY_DN13614_c0_g1_i1	Glyma.12G132800.1	98.46547315	210	6	348	2586	2248	7	0	2902

TRINITY_DN15718_c0_g1_i3	Glyma.04G217300.2	100	174	0	583	2829	2247	1	0	3186

TRINITY_DN15718_c0_g1_i5	Glyma.04G217300.2	96.57794677	174	0	664	2910	2247	1	0	3186

TRINITY_DN16106_c0_g3_i2	Glyma.06G072200.1	99	156	15	533	2734	2535	310	0	2859

TRINITY_DN628_c0_g1_i1	Glyma.08G075100.1	98.27586207	149	0	282	2528	2256	10	0	3325

TRINITY_DN37991_c0_g1_i1	Glyma.18G104500.2	97.28813559	142	11	332	2578	2220	1	0	3188

TRINITY_DN37991_c0_g1_i1	Glyma.18G104500.4	97.28813559	142	11	332	2578	2220	1	0	3188

TRINITY_DN6691_c0_g1_i1	Glyma.11G069300.1	95.34883721	493	44	685	2899	66	2286	0	1055

TRINITY_DN14332_c0_g1_i3	Glyma.13G275100.1	97.46835443	321	43	4	2221	781	3004	0	2047

TRINITY_DN12988_c0_g2_i1	Glyma.13G127100.2	100	307	11	224	2464	22	2259	0	2362

TRINITY_DN16331_c0_g2_i3	Glyma.13G360100.2	95.89041096	209	0	141	2386	1	2246	0	2990

TRINITY_DN16331_c0_g2_i3	Glyma.13G360100.3	95.89041096	209	0	141	2386	1	2246	0	2990

TRINITY_DN16331_c0_g2_i3	Glyma.13G360100.1	95.91836735	209	0	141	2386	1	2246	0	2990

TRINITY_DN16331_c0_g2_i3	Glyma.15G013800.2	95.90361446	204	0	141	2386	1	2246	0	3018

TRINITY_DN16331_c0_g2_i3	Glyma.15G013800.3	95.89041096	204	0	141	2386	1	2246	0	3018

TRINITY_DN16331_c0_g2_i3	Glyma.15G013800.1	95.90909091	204	0	141	2386	1	2246	0	3018

TRINITY_DN16040_c0_g1_i1	Glyma.20G054600.1	97.85407725	192	3	356	2600	2250	7	0	3064

TRINITY_DN16040_c0_g1_i4	Glyma.20G054600.1	95.87301587	181	3	356	2600	2250	7	0	3125

TRINITY_DN11150_c0_g1_i1	Glyma.19G208100.1	95.97315436	149	2	275	2519	24	2268	0	3310

TRINITY_DN11150_c0_g1_i2	Glyma.19G208100.1	97.05882353	149	2	275	2519	24	2268	0	3310

TRINITY_DN11150_c0_g1_i1	Glyma.03G210900.1	95.97315436	137	2	275	2519	186	2430	0	3376

TRINITY_DN11150_c0_g1_i2	Glyma.03G210900.1	97.05882353	137	2	275	2519	186	2430	0	3376

TRINITY_DN16331_c0_g2_i3	Glyma.08G207300.1	95.91836735	103	0	141	2386	1	2246	0	3578

TRINITY_DN16331_c0_g2_i3	Glyma.07G034700.1	95.90163934	95	0	141	2386	1	2246	0	3622

TRINITY_DN14518_c0_g1_i1	Glyma.06G202200.1	97.6744186	376	12	668	2904	2237	1	0	1960

TRINITY_DN12617_c0_g1_i1	Glyma.13G104300.4	98.50746269	305	13	2303	4538	1362	3597	0	2340

TRINITY_DN12617_c0_g1_i1	Glyma.13G104300.11	98.50746269	305	13	2303	4538	1338	3573	0	2340

TRINITY_DN16230_c0_g1_i4	Glyma.17G233900.1	99.78494624	260	18	80	2266	2263	20	0	2324

TRINITY_DN6296_c0_g1_i1	Glyma.05G146500.2	100	202	6	121	2363	1	2240	0	2981

TRINITY_DN3219_c0_g1_i1	Glyma.15G007800.2	100	190	25	181	2400	2367	136	0	2848

TRINITY_DN16190_c0_g3_i3	Glyma.06G229900.2	95.1048951	128	0	271	2515	1	2245	0	3437

TRINITY_DN16190_c0_g3_i9	Glyma.06G229900.2	98.63481229	128	0	271	2515	1	2245	0	3437

TRINITY_DN206_c0_g1_i1	Glyma.11G182800.1	99.41860465	123	3	262	2499	2244	1	0	3413

TRINITY_DN12050_c0_g2_i1	Glyma.16G093800.11	100	349	37	172	2390	3028	5222	0	1733

TRINITY_DN12050_c0_g2_i1	Glyma.16G093800.10	100	349	37	172	2390	3025	5219	0	1733

TRINITY_DN16257_c0_g1_i4	Glyma.12G096900.1	97.18309859	290	7	95	2332	2241	1	0	2479

TRINITY_DN16190_c0_g3_i7	Glyma.06G229900.2	100	254	11	348	2586	10	2245	0	2652

TRINITY_DN16190_c0_g3_i8	Glyma.06G229900.2	100	254	11	348	2586	10	2245	0	2652

TRINITY_DN14670_c0_g1_i3	Glyma.19G019100.4	95	233	15	1178	3416	9	2214	0	2575

TRINITY_DN13746_c0_g2_i1	Glyma.10G155100.1	95.34883721	209	29	458	2665	2695	488	0	2521

TRINITY_DN11819_c0_g3_i3	Glyma.19G103000.1	96.75925926	204	12	53	2278	1	2244	0	2898

TRINITY_DN10489_c0_g1_i1	Glyma.15G137800.1	95	200	9	264	2492	1	2235	0	2881

TRINITY_DN10489_c0_g2_i1	Glyma.15G137800.1	96.30314233	200	9	295	2523	1	2235	0	2881

TRINITY_DN11834_c0_g1_i2	Glyma.05G009500.1	97.56097561	198	12	345	2564	2244	1	0	2892

TRINITY_DN16436_c0_g2_i13	Glyma.12G055900.1	95.45454545	194	4	530	2771	48	2289	0	3044

TRINITY_DN3219_c0_g1_i1	Glyma.13G365400.2	100	189	25	181	2400	2364	136	0	2844

TRINITY_DN16049_c1_g2_i3	Glyma.08G102600.1	98.2300885	180	12	962	3191	2236	1	0	3005

TRINITY_DN9073_c0_g2_i2	Glyma.01G014700.1	97.11538462	153	8	413	2634	78	2314	0	3109

TRINITY_DN9073_c0_g2_i2	Glyma.09G207400.1	97.11934156	150	8	414	2634	1	2239	0	3133

TRINITY_DN3265_c0_g1_i1	Glyma.11G214800.1	96.5	149	1	136	2376	1	2244	0	3299

TRINITY_DN14312_c0_g1_i4	Glyma.11G012000.3	97.40740741	137	1	563	2806	2241	1	0	3365

TRINITY_DN14337_c0_g2_i3	Glyma.13G156600.3	95.16129032	131	8	248	2470	2244	1	0	3282

TRINITY_DN14337_c0_g2_i3	Glyma.13G156600.1	95.16129032	131	8	248	2470	2244	1	0	3282

TRINITY_DN206_c0_g1_i1	Glyma.12G089700.1	98.07692308	114	1	262	2499	2244	1	0	3474

TRINITY_DN15560_c0_g1_i1	Glyma.03G163900.5	98.48484848	369	28	300	2522	1	2221	0	1827

TRINITY_DN15560_c0_g1_i2	Glyma.03G163900.5	100	369	28	368	2590	1	2221	0	1827

TRINITY_DN16388_c0_g2_i2	Glyma.13G360700.1	100	329	31	128	2336	241	2452	0	1899

TRINITY_DN16257_c1_g1_i1	Glyma.12G192100.1	95.78313253	324	18	184	2408	2231	1	0	2154

TRINITY_DN16257_c1_g1_i8	Glyma.12G192100.1	98.29545455	319	18	184	2408	2231	1	0	2182

TRINITY_DN15818_c1_g2_i2	Glyma.03G136500.2	100	310	1	1156	3398	2242	1	0	2418

TRINITY_DN16230_c0_g1_i3	Glyma.17G233900.1	97.03872437	263	21	80	2265	2263	24	0	2298

TRINITY_DN16230_c0_g1_i2	Glyma.U030200.2	99.23076923	262	27	1	2181	2345	121	0	2174

TRINITY_DN9880_c0_g1_i2	Glyma.07G156700.1	99.72375691	215	1	4	2246	477	2713	0	2913

TRINITY_DN15648_c0_g2_i2	Glyma.10G153800.5	97.02970297	215	6	115	2352	3084	844	0	2905

TRINITY_DN15648_c0_g2_i2	Glyma.10G153800.1	95.14285714	215	6	115	2352	3084	844	0	2905

TRINITY_DN15648_c0_g2_i2	Glyma.10G153800.4	95.14563107	215	6	115	2352	3084	844	0	2905

TRINITY_DN15648_c0_g2_i2	Glyma.10G153800.3	97.73755656	215	6	115	2352	3084	844	0	2905

TRINITY_DN15648_c0_g2_i2	Glyma.10G153800.2	100	215	6	115	2352	3084	844	0	2905

TRINITY_DN15648_c0_g2_i2	Glyma.10G153800.11	100	215	6	115	2352	3084	844	0	2905

TRINITY_DN15648_c0_g2_i2	Glyma.10G153800.17	95.83333333	215	6	115	2352	3081	841	0	2905

TRINITY_DN15648_c0_g2_i2	Glyma.10G153800.16	97.6744186	215	6	115	2352	3081	841	0	2905

TRINITY_DN15648_c0_g2_i2	Glyma.10G153800.15	97.6744186	215	6	115	2352	3081	841	0	2905

TRINITY_DN15648_c0_g2_i2	Glyma.10G153800.14	96.98113208	215	6	115	2352	3081	841	0	2905

TRINITY_DN15648_c0_g2_i2	Glyma.10G153800.13	100	215	6	115	2352	3081	841	0	2905

TRINITY_DN2582_c0_g1_i1	Glyma.09G074300.1	99.21259843	161	2	963	3202	77	2319	0	3230

TRINITY_DN9073_c0_g2_i2	Glyma.01G014700.2	97.10144928	153	8	414	2634	1	2236	0	3107

TRINITY_DN16373_c3_g1_i1	Glyma.07G027800.1	100	357	49	1042	3197	3186	958	0	1522

TRINITY_DN5606_c0_g1_i1	Glyma.02G252200.1	95.8073149	300	27	18	2244	154	2371	0	2226

TRINITY_DN16056_c0_g1_i2	Glyma.02G301800.1	96.22641509	273	2	511	2749	2247	6	0	2608

TRINITY_DN15872_c0_g1_i3	Glyma.12G031800.1	98.82352941	270	4	312	2549	2322	82	0	2612

TRINITY_DN35889_c0_g1_i1	Glyma.19G254900.1	97.34513274	269	26	211	2423	2250	23	0	2372

TRINITY_DN16230_c0_g1_i4	Glyma.17G233900.2	100	260	17	80	2266	2260	20	0	2338

TRINITY_DN16230_c0_g1_i1	Glyma.17G233900.1	95.45454545	259	17	80	2265	2263	24	0	2331

TRINITY_DN15225_c0_g1_i2	Glyma.20G099200.2	100	258	9	937	3174	2360	126	0	2639

TRINITY_DN16436_c0_g2_i13	Glyma.11G131700.3	100	217	0	530	2771	6	2247	0	2939

TRINITY_DN15721_c0_g1_i1	Glyma.19G084600.1	97.26027397	202	12	714	2942	1	2229	0	2854

TRINITY_DN15721_c0_g1_i5	Glyma.19G084600.1	95.45454545	202	12	616	2844	1	2229	0	2854

TRINITY_DN10489_c0_g1_i1	Glyma.09G032800.1	99.36708861	195	11	264	2492	1	2232	0	2911

TRINITY_DN10489_c0_g2_i1	Glyma.09G032800.1	96.3011889	195	11	295	2523	1	2232	0	2911

TRINITY_DN14487_c0_g1_i1	Glyma.02G086200.1	96.04130809	164	3	533	2768	1	2241	0	3186

TRINITY_DN15594_c0_g1_i2	Glyma.01G203100.3	100	133	2	986	3221	2242	1	0	3365

TRINITY_DN15594_c0_g1_i5	Glyma.01G203100.3	100	126	10	986	3208	2242	1	0	3319

TRINITY_DN12050_c0_g2_i1	Glyma.16G093800.23	100	350	35	172	2390	3043	5234	0	1740

TRINITY_DN12050_c0_g2_i1	Glyma.16G093800.42	100	350	35	172	2390	3028	5219	0	1740

TRINITY_DN12050_c0_g2_i1	Glyma.16G093800.9	100	350	35	172	2390	3028	5219	0	1740

TRINITY_DN12050_c0_g2_i1	Glyma.16G093800.3	100	350	35	172	2390	3028	5219	0	1740

TRINITY_DN12050_c0_g2_i1	Glyma.16G093800.40	100	350	35	172	2390	3028	5219	0	1740

TRINITY_DN12050_c0_g2_i1	Glyma.16G093800.8	100	350	35	172	2390	3025	5216	0	1740

TRINITY_DN16257_c1_g1_i1	Glyma.13G310300.1	98.7654321	322	8	183	2408	2235	1	0	2218

TRINITY_DN16257_c1_g1_i8	Glyma.13G310300.1	98.29545455	313	8	183	2408	2235	1	0	2268

TRINITY_DN15225_c0_g1_i1	Glyma.20G099200.2	100	304	7	1215	3452	2360	126	0	2396

TRINITY_DN15648_c0_g2_i2	Glyma.20G234500.6	100	205	6	115	2352	3123	886	0	2964

TRINITY_DN15648_c0_g2_i2	Glyma.20G234500.4	100	205	6	115	2352	3081	844	0	2964

TRINITY_DN16216_c0_g3_i1	Glyma.20G241900.1	100	187	21	632	2860	211	2406	0	2734

TRINITY_DN16216_c0_g3_i7	Glyma.20G241900.1	99.06542056	187	21	293	2521	211	2406	0	2734

TRINITY_DN9073_c0_g2_i2	Glyma.01G014700.3	97.11538462	153	8	416	2634	9	2242	0	3103

TRINITY_DN9073_c0_g2_i2	Glyma.09G207400.2	97.11538462	150	7	414	2634	1	2236	0	3145

TRINITY_DN16016_c0_g1_i7	Glyma.10G221500.1	97.03703704	124	5	408	2642	3534	1303	0	3354

TRINITY_DN16016_c0_g1_i7	Glyma.10G221500.3	100	124	5	408	2642	3534	1303	0	3354

TRINITY_DN16016_c0_g1_i7	Glyma.10G221500.2	100	124	5	408	2642	3534	1303	0	3354

TRINITY_DN16016_c0_g1_i8	Glyma.10G221500.1	97.18309859	124	5	408	2642	3534	1303	0	3354

TRINITY_DN16016_c0_g1_i8	Glyma.10G221500.3	97.18309859	124	5	408	2642	3534	1303	0	3354

TRINITY_DN16016_c0_g1_i8	Glyma.10G221500.2	97.18309859	124	5	408	2642	3534	1303	0	3354

TRINITY_DN9404_c0_g1_i1	Glyma.08G257600.3	100	104	1	404	2641	2241	1	0	3542

TRINITY_DN16431_c0_g1_i4	Glyma.03G039200.2	100	381	34	2681	4880	2223	1	0	1657

TRINITY_DN16431_c0_g1_i5	Glyma.03G039200.2	95.78947368	381	34	2661	4860	2223	1	0	1657

TRINITY_DN16431_c0_g1_i6	Glyma.03G039200.2	100	381	34	2324	4523	2223	1	0	1657

TRINITY_DN16431_c0_g1_i7	Glyma.03G039200.2	97.56097561	381	34	2304	4503	2223	1	0	1657

TRINITY_DN15560_c0_g1_i1	Glyma.03G163900.6	96.64948454	369	27	300	2520	1	2218	0	1827

TRINITY_DN15560_c0_g1_i2	Glyma.03G163900.6	100	369	27	368	2588	1	2218	0	1827

TRINITY_DN15888_c0_g3_i5	Glyma.14G048100.1	96.26769627	324	21	437	2668	718	2934	0	2141

TRINITY_DN16230_c0_g1_i3	Glyma.17G233900.2	100	263	20	80	2265	2260	24	0	2311

TRINITY_DN16199_c0_g2_i1	Glyma.03G014000.2	98.42519685	258	5	1670	3901	1375	3612	0	2643

TRINITY_DN16199_c0_g2_i1	Glyma.03G014000.1	98.42519685	258	5	1670	3901	1375	3612	0	2643

TRINITY_DN14651_c0_g3_i1	Glyma.09G038600.1	96.47058824	243	4	88	2315	86	2316	0	2654

TRINITY_DN16049_c1_g2_i3	Glyma.05G145800.1	97.29064039	184	5	962	3191	2239	1	0	3046

TRINITY_DN16049_c1_g2_i3	Glyma.05G145800.2	100	184	5	962	3191	2239	1	0	3046

TRINITY_DN15403_c0_g2_i3	Glyma.10G178100.1	99.19354839	176	9	770	2998	3	2238	0	3064

TRINITY_DN14463_c0_g1_i1	Glyma.04G230800.3	100	162	8	311	2546	22	2251	0	3149

TRINITY_DN14463_c0_g1_i1	Glyma.04G230800.2	100	162	10	311	2546	22	2245	0	3110

TRINITY_DN15410_c0_g2_i1	Glyma.10G146900.5	97.10144928	131	9	1260	3494	2228	3	0	3288

TRINITY_DN15410_c0_g2_i2	Glyma.10G146900.5	97.22222222	131	9	1185	3419	2228	3	0	3288

TRINITY_DN16230_c0_g1_i1	Glyma.17G233900.2	95.45454545	259	16	80	2265	2260	24	0	2344

TRINITY_DN16056_c0_g1_i2	Glyma.14G012400.1	96.22641509	254	0	511	2749	2244	6	0	2728

TRINITY_DN2582_c0_g1_i1	Glyma.15G188300.1	95.87231701	158	0	964	3202	78	2316	0	3260

TRINITY_DN14337_c0_g2_i1	Glyma.13G156600.3	100	140	8	131	2348	2239	1	0	3223

TRINITY_DN14337_c0_g2_i1	Glyma.13G156600.1	100	140	8	131	2348	2239	1	0	3223

TRINITY_DN3265_c0_g1_i1	Glyma.18G041700.1	96.51162791	137	4	136	2370	1	2238	0	3343

TRINITY_DN16199_c0_g2_i1	Glyma.07G073700.1	96.12403101	267	1	1670	3901	1384	3621	0	2615

TRINITY_DN16199_c0_g2_i1	Glyma.07G073700.3	98.96907216	267	1	1670	3901	1384	3621	0	2615

TRINITY_DN6691_c0_g1_i1	Glyma.02G055500.1	95.74898785	241	11	671	2899	55	2283	0	2682

TRINITY_DN17473_c0_g1_i1	Glyma.01G000600.2	98.24561404	125	5	320	2556	100	2331	0	3395

TRINITY_DN10067_c0_g4_i1	Glyma.15G163700.4	95.95959596	107	0	413	2650	1	2238	0	3541

TRINITY_DN10067_c0_g4_i2	Glyma.15G163700.4	100	107	0	413	2650	1	2238	0	3541

TRINITY_DN16388_c0_g2_i2	Glyma.13G360700.2	100	329	30	128	2336	241	2446	0	1927

TRINITY_DN14332_c0_g1_i3	Glyma.12G225800.1	97.46835443	328	27	4	2221	781	3004	0	2108

TRINITY_DN14651_c0_g3_i1	Glyma.15G143700.1	96.47058824	235	8	93	2315	94	2319	0	2667

TRINITY_DN11482_c0_g1_i1	Glyma.04G048800.1	96.12403101	206	5	415	2646	1	2235	0	2944

TRINITY_DN13844_c0_g1_i1	Glyma.01G074000.4	95.96774194	168	3	743	2977	1	2235	0	3175

TRINITY_DN14378_c0_g2_i1	Glyma.04G038600.2	98.24561404	160	20	532	2740	702	2902	0	2832

TRINITY_DN14378_c0_g2_i1	Glyma.04G038600.1	95.34883721	160	20	532	2740	702	2902	0	2832

TRINITY_DN17473_c0_g1_i1	Glyma.14G000400.1	97.14285714	132	2	320	2556	100	2334	0	3387

TRINITY_DN16071_c0_g2_i8	Glyma.09G052000.1	99.21875	124	3	89	2313	1	2228	0	3308

TRINITY_DN16064_c0_g2_i1	Glyma.19G169400.1	95	533	40	92	2304	124	2336	0	880

TRINITY_DN16064_c0_g2_i3	Glyma.19G169400.1	98.64864865	533	40	92	2304	124	2336	0	880

TRINITY_DN16254_c0_g1_i5	Glyma.17G057800.1	98.57142857	494	64	1	2189	2573	370	0	881

TRINITY_DN11999_c1_g1_i1	Glyma.05G103200.2	98.95833333	330	14	174	2401	44	2268	0	2178

TRINITY_DN11999_c1_g1_i1	Glyma.05G103200.1	98.95833333	330	14	174	2401	44	2268	0	2178

TRINITY_DN11999_c1_g1_i1	Glyma.05G103200.3	95.23809524	330	14	174	2401	44	2268	0	2178

TRINITY_DN11999_c1_g1_i2	Glyma.05G103200.2	100	330	14	321	2548	44	2268	0	2178

TRINITY_DN11999_c1_g1_i2	Glyma.05G103200.1	100	330	14	321	2548	44	2268	0	2178

TRINITY_DN11999_c1_g1_i2	Glyma.05G103200.3	96.1038961	330	14	321	2548	44	2268	0	2178

TRINITY_DN16067_c2_g1_i1	Glyma.07G249100.1	96.58703072	200	2	321	2555	1	2235	0	3009

TRINITY_DN16067_c2_g1_i2	Glyma.07G249100.1	98.17351598	200	2	321	2555	1	2235	0	3009

TRINITY_DN9205_c0_g1_i2	Glyma.07G148100.25	100	191	20	830	3027	2234	1	0	2813

TRINITY_DN9205_c0_g1_i2	Glyma.07G148100.5	100	191	20	830	3027	2234	1	0	2813

TRINITY_DN9205_c0_g1_i3	Glyma.07G148100.25	98.97959184	191	20	973	3170	2234	1	0	2813

TRINITY_DN9205_c0_g1_i3	Glyma.07G148100.5	98.97959184	191	20	973	3170	2234	1	0	2813

TRINITY_DN9205_c0_g1_i4	Glyma.07G148100.25	98.7804878	191	20	734	2931	2234	1	0	2813

TRINITY_DN9205_c0_g1_i4	Glyma.07G148100.5	100	191	20	734	2931	2234	1	0	2813

TRINITY_DN16067_c2_g1_i1	Glyma.17G025200.1	96.17834395	189	2	321	2555	1	2235	0	3070

TRINITY_DN16067_c2_g1_i2	Glyma.17G025200.1	99.00990099	189	2	321	2555	1	2235	0	3070

TRINITY_DN9205_c0_g1_i2	Glyma.07G148100.24	99.07407407	189	26	830	3027	2210	1	0	2669

TRINITY_DN9205_c0_g1_i2	Glyma.07G148100.4	99.07407407	189	26	830	3027	2210	1	0	2669

TRINITY_DN9205_c0_g1_i3	Glyma.07G148100.24	100	189	26	973	3170	2210	1	0	2669

TRINITY_DN9205_c0_g1_i3	Glyma.07G148100.4	97.39583333	189	26	973	3170	2210	1	0	2669

TRINITY_DN9205_c0_g1_i4	Glyma.07G148100.24	100	189	26	734	2931	2210	1	0	2669

TRINITY_DN9205_c0_g1_i4	Glyma.07G148100.4	95.62043796	189	26	734	2931	2210	1	0	2669

TRINITY_DN15396_c0_g1_i2	Glyma.10G086800.1	98.25327511	177	6	385	2619	2217	1	0	3020

TRINITY_DN15396_c0_g1_i2	Glyma.10G086800.3	98.40425532	177	6	385	2619	2217	1	0	3020

TRINITY_DN15396_c0_g1_i2	Glyma.02G188300.1	98.25327511	175	4	385	2619	2232	1	0	3127

TRINITY_DN16019_c0_g1_i5	Glyma.06G013100.1	97.88732394	223	12	943	3174	2217	1	0	2756

TRINITY_DN16019_c0_g1_i8	Glyma.06G013100.1	97.5	216	12	943	3174	2217	1	0	2795

TRINITY_DN16067_c2_g1_i3	Glyma.15G154000.2	100	215	0	321	2555	1	2235	0	2937

TRINITY_DN16067_c2_g1_i3	Glyma.15G154000.1	100	215	0	321	2555	1	2235	0	2937

TRINITY_DN16067_c2_g1_i3	Glyma.09G046500.1	98.7854251	211	0	321	2555	1	2235	0	2959

TRINITY_DN16132_c0_g1_i2	Glyma.17G226100.1	95.41984733	195	10	1445	3677	720	2943	0	2963

TRINITY_DN16132_c0_g1_i2	Glyma.17G226100.2	95.65217391	195	10	1445	3677	669	2892	0	2963

TRINITY_DN16067_c2_g1_i1	Glyma.15G154000.2	100	136	0	321	2555	1	2235	0	3374

TRINITY_DN16067_c2_g1_i1	Glyma.15G154000.1	100	136	0	321	2555	1	2235	0	3374

TRINITY_DN16067_c2_g1_i2	Glyma.15G154000.2	99.02912621	136	0	321	2555	1	2235	0	3374

TRINITY_DN16067_c2_g1_i2	Glyma.15G154000.1	99.00990099	136	0	321	2555	1	2235	0	3374

TRINITY_DN16067_c2_g1_i1	Glyma.09G046500.1	97.60479042	131	0	321	2555	1	2235	0	3402

TRINITY_DN16067_c2_g1_i2	Glyma.09G046500.1	99.46236559	131	0	321	2555	1	2235	0	3402

TRINITY_DN16016_c0_g1_i7	Glyma.20G170000.1	99.18032787	123	3	408	2642	3537	1312	0	3387

TRINITY_DN16016_c0_g1_i8	Glyma.20G170000.1	100	123	3	408	2642	3537	1312	0	3387

TRINITY_DN16054_c1_g1_i1	Glyma.10G168700.3	95.53314121	121	0	375	2609	2235	1	0	3458

TRINITY_DN16054_c1_g1_i4	Glyma.10G168700.3	96.57534247	121	0	375	2609	2235	1	0	3458

TRINITY_DN16067_c2_g1_i3	Glyma.07G249100.1	98.7854251	98	0	321	2555	1	2235	0	3585

TRINITY_DN16067_c2_g1_i3	Glyma.17G025200.1	98.36065574	91	0	321	2555	1	2235	0	3624

TRINITY_DN15323_c0_g1_i2	Glyma.10G094500.6	100	278	15	390	2611	1	2219	0	2410

TRINITY_DN15323_c0_g1_i2	Glyma.10G094500.5	98.5915493	278	15	390	2611	1	2219	0	2410

TRINITY_DN14071_c0_g1_i1	Glyma.03G261100.1	100	143	2	167	2399	3	2235	0	3321

TRINITY_DN13778_c1_g3_i3	Glyma.18G198000.2	100	475	50	197	2398	2645	442	0	1105

TRINITY_DN13778_c1_g3_i3	Glyma.18G198000.1	97.70992366	475	50	197	2398	2645	442	0	1105

TRINITY_DN13226_c0_g1_i1	Glyma.11G107600.1	98.07692308	468	48	390	2594	2336	132	0	1170

TRINITY_DN16231_c1_g1_i1	Glyma.09G073600.4	100	290	9	114	2340	35	2263	0	2453

TRINITY_DN16257_c0_g1_i4	Glyma.06G307900.1	97.87234043	274	7	116	2332	2232	1	0	2495

TRINITY_DN15333_c1_g1_i1	Glyma.13G078500.4	100	267	11	100	2322	3	2231	0	2555

TRINITY_DN15333_c1_g1_i1	Glyma.13G078500.3	100	267	11	100	2322	3	2231	0	2555

TRINITY_DN15333_c1_g1_i1	Glyma.13G078500.2	100	267	11	100	2322	3	2231	0	2555

TRINITY_DN15333_c1_g1_i1	Glyma.13G078500.1	96.77419355	267	11	100	2322	3	2231	0	2555

TRINITY_DN16019_c0_g1_i5	Glyma.04G013200.1	99.11816578	224	3	943	3174	2229	1	0	2850

TRINITY_DN16019_c0_g1_i8	Glyma.04G013200.1	96.03960396	217	3	943	3174	2229	1	0	2889

TRINITY_DN16132_c0_g1_i2	Glyma.14G098600.1	98.21428571	211	6	1445	3677	720	2943	0	2896

TRINITY_DN8358_c0_g2_i1	Glyma.02G212700.5	100	173	7	292	2512	1	2215	0	2972

TRINITY_DN14549_c0_g1_i3	Glyma.17G166800.1	99.68454259	167	4	1	2227	222	2454	0	3160

TRINITY_DN14071_c0_g1_i1	Glyma.19G260100.1	97.14285714	151	0	167	2399	27	2259	0	3288

TRINITY_DN14071_c0_g1_i1	Glyma.19G260100.2	97.14285714	151	0	167	2399	3	2235	0	3288

TRINITY_DN15511_c0_g1_i2	Glyma.19G150400.1	97.27891156	142	4	80	2311	2220	1	0	3247

TRINITY_DN35665_c0_g1_i1	Glyma.19G263500.1	100	92	0	1	2233	309	2541	0	3615

TRINITY_DN16132_c0_g1_i2	Glyma.04G056200.1	100	255	7	1445	3671	723	2952	0	2663

TRINITY_DN6691_c0_g1_i1	Glyma.16G138300.1	98.87640449	239	5	671	2899	55	2277	0	2721

TRINITY_DN6691_c0_g1_i1	Glyma.16G138300.9	96.53179191	239	5	671	2899	55	2277	0	2721

TRINITY_DN6691_c0_g1_i1	Glyma.16G138300.3	100	239	5	671	2899	55	2277	0	2721

TRINITY_DN6691_c0_g1_i1	Glyma.16G138300.11	95.66360053	239	5	671	2899	55	2277	0	2721

TRINITY_DN10751_c0_g2_i1	Glyma.17G123400.1	96.77419355	206	31	130	2313	1	2223	0	2612

TRINITY_DN4211_c0_g1_i1	Glyma.09G238900.1	99.31506849	199	24	409	2592	1	2217	0	2612

TRINITY_DN4211_c0_g1_i1	Glyma.09G238900.3	98	199	24	409	2592	1	2217	0	2612

TRINITY_DN4211_c0_g1_i1	Glyma.09G238900.2	97.87234043	199	25	409	2592	1	2214	0	2593

TRINITY_DN15876_c0_g1_i3	Glyma.06G040600.1	99.20634921	195	7	132	2355	180	2409	0	2977

TRINITY_DN15876_c0_g1_i3	Glyma.06G040600.2	100	195	7	132	2355	180	2409	0	2977

TRINITY_DN15721_c0_g1_i1	Glyma.16G062200.1	97.26027397	173	8	723	2942	13	2235	0	3027

TRINITY_DN15721_c0_g1_i5	Glyma.16G062200.1	96.15384615	173	8	625	2844	13	2235	0	3027

TRINITY_DN15511_c0_g1_i2	Glyma.03G147000.1	100	152	2	80	2311	2220	1	0	3203

TRINITY_DN14337_c0_g2_i3	Glyma.17G104200.1	95.16129032	139	5	248	2470	2232	1	0	3293

TRINITY_DN16019_c0_g1_i8	Glyma.11G118600.1	96.27403846	127	0	943	3174	2232	1	0	3419

TRINITY_DN16019_c0_g1_i5	Glyma.11G118600.1	97.61904762	124	0	943	3174	2232	1	0	3435

TRINITY_DN16019_c0_g1_i8	Glyma.12G044000.1	95.23809524	124	0	943	3174	2232	1	0	3435

TRINITY_DN16019_c0_g1_i5	Glyma.12G044000.1	96.27118644	121	0	943	3174	2232	1	0	3452

TRINITY_DN15293_c0_g1_i3	Glyma.11G179600.3	98.55072464	342	46	467	2654	1	2197	0	1727

TRINITY_DN15293_c0_g1_i3	Glyma.11G179600.1	97.96511628	342	46	467	2654	1	2197	0	1727

TRINITY_DN15293_c0_g1_i2	Glyma.11G179600.3	98.50746269	316	46	530	2717	1	2197	0	1871

TRINITY_DN15293_c0_g1_i2	Glyma.11G179600.1	96.62162162	316	46	530	2717	1	2197	0	1871

TRINITY_DN16231_c1_g1_i2	Glyma.09G073600.4	98.12792512	291	8	114	2340	35	2261	0	2457

TRINITY_DN16106_c0_g3_i2	Glyma.14G128300.1	100	246	14	533	2753	2538	318	0	2628

TRINITY_DN16106_c0_g3_i2	Glyma.14G128300.3	95.215311	246	14	533	2753	2508	288	0	2628

TRINITY_DN16106_c0_g3_i2	Glyma.14G128300.2	95.21410579	246	14	533	2753	2454	234	0	2628

TRINITY_DN16060_c0_g1_i1	Glyma.17G087600.2	98.68421053	217	14	618	2823	4713	2514	0	2556

TRINITY_DN16060_c0_g1_i1	Glyma.17G087600.3	98	217	14	618	2823	4713	2514	0	2556

TRINITY_DN16060_c0_g1_i1	Glyma.17G087600.1	95.65217391	217	14	618	2823	4713	2514	0	2556

TRINITY_DN16060_c0_g1_i2	Glyma.17G087600.2	100	217	14	604	2809	4713	2514	0	2556

TRINITY_DN16060_c0_g1_i2	Glyma.17G087600.3	97.70642202	217	14	604	2809	4713	2514	0	2556

TRINITY_DN16060_c0_g1_i2	Glyma.17G087600.1	99.26315789	217	14	604	2809	4713	2514	0	2556

TRINITY_DN16060_c0_g1_i3	Glyma.17G087600.2	96.68367347	217	14	697	2902	4713	2514	0	2556

TRINITY_DN16060_c0_g1_i3	Glyma.17G087600.3	95.88235294	217	14	697	2902	4713	2514	0	2556

TRINITY_DN16060_c0_g1_i3	Glyma.17G087600.1	100	217	14	697	2902	4713	2514	0	2556

TRINITY_DN3806_c0_g1_i1	Glyma.17G215200.1	96.03960396	174	16	1	2204	3878	6099	0	2924

TRINITY_DN3806_c0_g1_i1	Glyma.17G215200.2	98.36065574	174	16	1	2204	2882	5103	0	2924

TRINITY_DN36234_c0_g1_i1	Glyma.14G078800.2	96.40883978	173	12	99	2318	17	2224	0	2942

TRINITY_DN36234_c0_g1_i1	Glyma.14G078800.1	99.513382	173	12	99	2318	17	2224	0	2942

TRINITY_DN16019_c0_g1_i11	Glyma.11G118600.2	97.61904762	164	0	20	2250	2231	1	0	3212

TRINITY_DN16019_c0_g1_i1	Glyma.11G118600.2	100	161	0	20	2250	2231	1	0	3229

TRINITY_DN16019_c0_g1_i6	Glyma.11G118600.2	96.84210526	127	0	764	2994	2231	1	0	3417

TRINITY_DN16019_c0_g1_i9	Glyma.11G118600.2	95.77464789	124	0	764	2994	2231	1	0	3434

TRINITY_DN16254_c0_g1_i10	Glyma.17G057800.1	98.68421053	504	51	1	2186	2573	370	0	874

TRINITY_DN16231_c1_g1_i2	Glyma.15G182600.2	96.43765903	282	6	114	2340	35	2261	0	2518

TRINITY_DN9165_c0_g1_i1	Glyma.14G064700.1	98.63013699	245	30	50	2274	9	2197	0	2464

TRINITY_DN16257_c1_g1_i1	Glyma.12G192000.1	97.00598802	295	12	192	2408	2220	1	0	2346

TRINITY_DN16257_c1_g1_i8	Glyma.12G192000.1	100	291	12	192	2408	2220	1	0	2368

TRINITY_DN226_c0_g1_i1	Glyma.08G291800.1	96.42857143	199	0	232	2460	1858	4086	0	3014

TRINITY_DN14549_c0_g1_i3	Glyma.05G098800.1	100	153	1	2	2227	271	2499	0	3249

TRINITY_DN14841_c0_g1_i2	Glyma.06G000200.1	99.49494949	137	10	467	2671	1	2217	0	3125

TRINITY_DN14841_c0_g1_i2	Glyma.04G000300.2	99.49494949	133	19	467	2671	1	2205	0	3070

TRINITY_DN14841_c0_g1_i2	Glyma.04G000300.1	99.49494949	133	19	467	2671	1	2205	0	3070

TRINITY_DN12798_c0_g2_i1	Glyma.13G326400.1	97.96610169	121	4	385	2607	2229	1	0	3408

TRINITY_DN12798_c0_g2_i1	Glyma.13G326400.3	97.96672828	121	4	385	2607	2229	1	0	3408

TRINITY_DN12798_c0_g2_i1	Glyma.13G326400.2	95.45454545	121	4	385	2607	2229	1	0	3408

TRINITY_DN13226_c0_g1_i1	Glyma.12G032600.1	98.63013699	480	44	390	2594	2336	132	0	1158

TRINITY_DN6136_c0_g1_i1	Glyma.10G041300.1	96.74796748	345	39	258	2462	2262	70	0	1829

TRINITY_DN6136_c0_g2_i1	Glyma.10G041300.1	97.22222222	345	39	257	2461	2262	70	0	1829

TRINITY_DN15323_c0_g1_i2	Glyma.02G180100.4	96.3963964	271	16	390	2603	1	2211	0	2412

TRINITY_DN15323_c0_g1_i2	Glyma.02G180100.3	100	271	16	390	2603	1	2211	0	2412

TRINITY_DN16132_c0_g1_i2	Glyma.06G056400.1	99.12280702	269	2	1445	3671	723	2949	0	2612

TRINITY_DN16132_c0_g1_i2	Glyma.06G056400.4	98.9010989	269	2	1445	3671	723	2949	0	2612

TRINITY_DN16132_c0_g1_i2	Glyma.06G056400.3	98.7012987	269	2	1445	3671	717	2943	0	2612

TRINITY_DN16237_c0_g1_i11	Glyma.05G200800.5	96	172	14	256	2462	1	2216	0	2948

TRINITY_DN16237_c0_g1_i15	Glyma.05G200800.5	100	172	14	256	2462	1	2216	0	2948

TRINITY_DN16237_c0_g1_i5	Glyma.05G200800.5	99.32432432	172	14	256	2462	1	2216	0	2948

TRINITY_DN7905_c0_g1_i1	Glyma.15G207200.1	95.50561798	136	8	272	2468	11	2235	0	3142

TRINITY_DN7905_c0_g1_i2	Glyma.15G207200.1	95.53072626	136	8	171	2367	11	2235	0	3142

TRINITY_DN15180_c1_g1_i4	Glyma.06G225700.1	95.73333333	324	24	365	2572	136	2328	0	1975

TRINITY_DN16257_c1_g1_i1	Glyma.12G191800.1	96.83544304	308	10	193	2408	2219	1	0	2283

TRINITY_DN16257_c1_g1_i8	Glyma.12G191800.1	100	297	10	193	2408	2219	1	0	2344

TRINITY_DN14332_c0_g1_i3	Glyma.U025000.2	97.36842105	211	8	1	2222	760	2978	0	2859

TRINITY_DN14332_c0_g1_i3	Glyma.U025000.1	96.18441971	211	8	1	2222	760	2978	0	2859

TRINITY_DN13021_c0_g1_i1	Glyma.08G256200.1	100	196	31	692	2896	1	2175	0	2549

TRINITY_DN14337_c0_g2_i1	Glyma.17G104200.1	98.48484848	149	5	131	2348	2227	1	0	3229

TRINITY_DN14332_c0_g1_i3	Glyma.06G238700.1	97.46835443	210	11	1	2222	760	2975	0	2857

TRINITY_DN15509_c0_g1_i1	Glyma.07G237900.1	96.8503937	187	12	221	2419	1	2214	0	2822

TRINITY_DN13325_c0_g2_i2	Glyma.19G115700.1	96.76470588	151	1	95	2317	1	2226	0	3254

TRINITY_DN9762_c0_g1_i1	Glyma.04G116700.1	97.97297297	135	17	430	2622	2217	1	0	3092

TRINITY_DN9762_c0_g1_i1	Glyma.04G116700.2	97.95918367	135	17	430	2622	2217	1	0	3092

TRINITY_DN9762_c0_g1_i1	Glyma.04G116700.3	97.97979798	135	17	430	2622	2217	1	0	3092

TRINITY_DN9762_c0_g1_i1	Glyma.04G116700.4	97.97979798	135	17	430	2622	2217	1	0	3092

TRINITY_DN12798_c0_g2_i1	Glyma.12G173200.2	96.55172414	130	1	385	2607	2226	1	0	3371

TRINITY_DN12798_c0_g2_i1	Glyma.12G173200.1	99.46524064	130	1	385	2607	2226	1	0	3371

TRINITY_DN49_c0_g1_i1	Glyma.20G217700.4	99.09090909	271	21	364	2550	3138	931	0	2252

TRINITY_DN49_c0_g1_i1	Glyma.20G217700.2	100	271	21	364	2550	3138	931	0	2252

TRINITY_DN49_c0_g2_i1	Glyma.20G217700.4	95.38461538	271	21	1279	3465	3138	931	0	2252

TRINITY_DN49_c0_g2_i1	Glyma.20G217700.2	100	271	21	1279	3465	3138	931	0	2252

TRINITY_DN14326_c0_g5_i1	Glyma.04G008500.1	99.5951417	204	14	2136	4345	2018	4224	0	2765

TRINITY_DN11332_c0_g2_i1	Glyma.18G130800.1	100	189	30	1	2186	116	2325	0	2713

TRINITY_DN11043_c0_g1_i1	Glyma.10G189300.1	98.57346648	179	8	83	2302	1	2214	0	3014

TRINITY_DN7905_c0_g1_i1	Glyma.04G144600.1	95.45454545	143	5	272	2468	11	2235	0	3136

TRINITY_DN7905_c0_g1_i2	Glyma.04G144600.1	95.45454545	143	5	171	2367	11	2235	0	3136

TRINITY_DN6058_c0_g1_i1	Glyma.02G151400.2	97.33333333	135	2	823	3047	2210	1	0	3264

TRINITY_DN13959_c0_g1_i3	Glyma.10G292300.4	95.14563107	128	17	646	2842	2565	347	0	3181

TRINITY_DN19780_c0_g1_i1	Glyma.19G116700.1	100	119	4	832	3054	2237	15	0	3424

TRINITY_DN16254_c0_g1_i5	Glyma.17G057800.4	98.63945578	496	60	1	2189	2561	370	0	926

TRINITY_DN16254_c0_g1_i5	Glyma.17G057800.3	100	496	60	1	2189	2561	370	0	926

TRINITY_DN16254_c0_g1_i5	Glyma.17G057800.2	100	496	60	1	2189	2561	370	0	926

TRINITY_DN18910_c0_g1_i1	Glyma.16G019300.1	95.57195572	427	59	52	2226	2	2182	0	1147

TRINITY_DN12526_c0_g1_i1	Glyma.07G198200.2	99.70326409	372	43	1	2182	63	2262	0	1620

TRINITY_DN12526_c0_g1_i1	Glyma.07G198200.3	97.5	372	43	1	2182	63	2262	0	1620

TRINITY_DN12526_c0_g1_i1	Glyma.07G198200.4	99.45054945	372	43	1	2182	63	2262	0	1620

TRINITY_DN12526_c0_g1_i1	Glyma.07G198200.5	99.02912621	372	43	1	2182	63	2262	0	1620

TRINITY_DN12526_c0_g1_i1	Glyma.07G198200.1	98.5915493	372	43	1	2182	63	2262	0	1620

TRINITY_DN8710_c0_g1_i1	Glyma.18G027000.1	95.50561798	200	4	236	2457	2634	413	0	2974

TRINITY_DN8710_c0_g1_i2	Glyma.18G027000.1	100	200	5	236	2441	2634	413	0	2870

TRINITY_DN3806_c0_g1_i1	Glyma.14G112700.6	95.08196721	174	15	1	2200	3815	6023	0	2891

TRINITY_DN3806_c0_g1_i1	Glyma.14G112700.1	97.74011299	174	15	1	2200	3815	6023	0	2891

TRINITY_DN3806_c0_g1_i1	Glyma.14G112700.5	95.08196721	174	15	1	2200	3812	6020	0	2891

TRINITY_DN3806_c0_g1_i1	Glyma.14G112700.4	99.24242424	174	15	1	2200	3809	6017	0	2891

TRINITY_DN3806_c0_g1_i1	Glyma.14G112700.2	96.72131148	174	15	1	2200	3407	5615	0	2891

TRINITY_DN11942_c0_g1_i1	Glyma.19G064500.1	100	151	5	149	2362	1	2220	0	3181

TRINITY_DN15352_c0_g1_i3	Glyma.07G267800.1	100	148	2	85	2308	9	2229	0	3267

TRINITY_DN4278_c0_g1_i1	Glyma.12G013000.3	99.13043478	131	0	698	2921	2224	1	0	3382

TRINITY_DN4278_c0_g1_i1	Glyma.12G013000.2	98.02631579	131	0	698	2921	2224	1	0	3382

TRINITY_DN14778_c0_g1_i3	Glyma.14G035400.3	96.875	127	1	514	2734	1	2224	0	3384

TRINITY_DN16254_c0_g1_i5	Glyma.13G101700.1	95.40636042	499	58	1	2189	2561	370	0	920

TRINITY_DN11254_c0_g1_i1	Glyma.13G347800.1	100	495	65	354	2539	2355	173	0	865

TRINITY_DN15137_c0_g1_i5	Glyma.14G222700.1	100	269	8	556	2770	2289	69	0	2551

TRINITY_DN15137_c0_g1_i10	Glyma.14G222700.1	98.94736842	252	8	528	2742	2289	69	0	2645

TRINITY_DN9350_c0_g1_i1	Glyma.10G019200.8	96.40718563	240	9	154	2373	1	2211	0	2678

TRINITY_DN5710_c0_g1_i1	Glyma.02G295400.3	96.66666667	209	6	801	3015	2289	72	0	2863

TRINITY_DN3358_c0_g1_i1	Glyma.08G137900.1	96.23655914	196	9	62	2266	2226	19	0	2806

TRINITY_DN3358_c0_g2_i1	Glyma.08G137900.1	100	196	9	62	2266	2226	19	0	2806

TRINITY_DN3358_c0_g3_i1	Glyma.08G137900.1	100	196	9	62	2266	2226	19	0	2806

TRINITY_DN3358_c0_g4_i1	Glyma.08G137900.1	97.72370487	196	9	62	2266	2226	19	0	2806

TRINITY_DN11025_c0_g2_i1	Glyma.08G125700.1	96.8503937	195	14	208	2415	1	2217	0	2889

TRINITY_DN11025_c0_g2_i2	Glyma.08G125700.1	100	195	14	73	2280	1	2217	0	2889

TRINITY_DN15509_c0_g1_i1	Glyma.17G035500.1	96.38554217	181	14	221	2419	1	2202	0	2811

TRINITY_DN40017_c0_g1_i1	Glyma.10G183700.1	100	173	4	126	2348	14	2232	0	3121

TRINITY_DN15417_c0_g1_i1	Glyma.05G194500.2	100	149	1	467	2686	2226	4	0	3260

TRINITY_DN15417_c0_g1_i2	Glyma.05G194500.2	97.27272727	149	1	733	2952	2226	4	0	3260

TRINITY_DN9762_c0_g1_i1	Glyma.06G320100.2	97.95918367	131	18	430	2622	2211	1	0	3109

TRINITY_DN9762_c0_g1_i1	Glyma.06G320100.1	97.95918367	131	18	430	2622	2211	1	0	3109

TRINITY_DN12647_c0_g1_i1	Glyma.09G002600.17	95.89041096	101	0	180	2402	1	2223	0	3546

TRINITY_DN12647_c0_g1_i1	Glyma.09G002600.12	95.91836735	101	0	180	2402	1	2223	0	3546

TRINITY_DN12647_c0_g1_i1	Glyma.09G002600.11	95.90163934	101	0	180	2402	1	2223	0	3546

TRINITY_DN12647_c0_g1_i1	Glyma.09G002600.13	95.91002045	101	0	180	2402	1	2223	0	3546

TRINITY_DN12647_c0_g1_i1	Glyma.09G002600.14	95.90163934	101	0	180	2402	1	2223	0	3546

TRINITY_DN12647_c0_g1_i1	Glyma.09G002600.1	96.96969697	101	0	180	2402	1	2223	0	3546

TRINITY_DN12647_c0_g1_i1	Glyma.09G002600.5	96.96969697	101	0	180	2402	1	2223	0	3546

TRINITY_DN12647_c0_g1_i1	Glyma.09G002600.3	95.91836735	101	0	180	2402	1	2223	0	3546

TRINITY_DN12647_c0_g1_i1	Glyma.09G002600.15	95.90163934	101	0	180	2402	1	2223	0	3546

TRINITY_DN12647_c0_g1_i5	Glyma.09G002600.17	96.2962963	101	0	180	2402	1	2223	0	3546

TRINITY_DN12647_c0_g1_i5	Glyma.09G002600.12	96.2962963	101	0	180	2402	1	2223	0	3546

TRINITY_DN12647_c0_g1_i5	Glyma.09G002600.11	96.2962963	101	0	180	2402	1	2223	0	3546

TRINITY_DN12647_c0_g1_i5	Glyma.09G002600.13	96.2962963	101	0	180	2402	1	2223	0	3546

TRINITY_DN12647_c0_g1_i5	Glyma.09G002600.14	96.2962963	101	0	180	2402	1	2223	0	3546

TRINITY_DN12647_c0_g1_i5	Glyma.09G002600.1	96.2962963	101	0	180	2402	1	2223	0	3546

TRINITY_DN12647_c0_g1_i5	Glyma.09G002600.5	96.30102041	101	0	180	2402	1	2223	0	3546

TRINITY_DN12647_c0_g1_i5	Glyma.09G002600.3	96.2962963	101	0	180	2402	1	2223	0	3546

TRINITY_DN12647_c0_g1_i5	Glyma.09G002600.15	96.2962963	101	0	180	2402	1	2223	0	3546

TRINITY_DN12647_c0_g1_i7	Glyma.09G002600.17	96.54576857	101	0	150	2372	1	2223	0	3546

TRINITY_DN12647_c0_g1_i7	Glyma.09G002600.12	96.53465347	101	0	150	2372	1	2223	0	3546

TRINITY_DN12647_c0_g1_i7	Glyma.09G002600.11	96.53465347	101	0	150	2372	1	2223	0	3546

TRINITY_DN12647_c0_g1_i7	Glyma.09G002600.13	96.53465347	101	0	150	2372	1	2223	0	3546

TRINITY_DN12647_c0_g1_i7	Glyma.09G002600.14	96.54576857	101	0	150	2372	1	2223	0	3546

TRINITY_DN12647_c0_g1_i7	Glyma.09G002600.1	96.55172414	101	0	150	2372	1	2223	0	3546

TRINITY_DN12647_c0_g1_i7	Glyma.09G002600.5	96.55172414	101	0	150	2372	1	2223	0	3546

TRINITY_DN12647_c0_g1_i7	Glyma.09G002600.3	96.53179191	101	0	150	2372	1	2223	0	3546

TRINITY_DN12647_c0_g1_i7	Glyma.09G002600.15	96.55172414	101	0	150	2372	1	2223	0	3546

TRINITY_DN12647_c0_g1_i1	Glyma.12G241700.2	95.91836735	85	1	180	2402	1	2220	0	3615

TRINITY_DN12647_c0_g1_i1	Glyma.12G241700.1	95.90163934	85	1	180	2402	1	2220	0	3615

TRINITY_DN12647_c0_g1_i1	Glyma.12G241700.4	95.89322382	85	1	180	2402	1	2220	0	3615

TRINITY_DN12647_c0_g1_i5	Glyma.12G241700.2	96.29909366	85	1	180	2402	1	2220	0	3615

TRINITY_DN12647_c0_g1_i5	Glyma.12G241700.1	96.97841727	85	1	180	2402	1	2220	0	3615

TRINITY_DN12647_c0_g1_i5	Glyma.12G241700.4	96.2962963	85	1	180	2402	1	2220	0	3615

TRINITY_DN12647_c0_g1_i7	Glyma.12G241700.2	96.55172414	85	1	150	2372	1	2220	0	3615

TRINITY_DN12647_c0_g1_i7	Glyma.12G241700.1	96.53679654	85	1	150	2372	1	2220	0	3615

TRINITY_DN12647_c0_g1_i7	Glyma.12G241700.4	96.55172414	85	1	150	2372	1	2220	0	3615

TRINITY_DN12647_c0_g1_i1	Glyma.12G241700.3	95.91836735	84	3	180	2402	1	2211	0	3563

TRINITY_DN12647_c0_g1_i5	Glyma.12G241700.3	96.2962963	84	3	180	2402	1	2211	0	3563

TRINITY_DN12647_c0_g1_i7	Glyma.12G241700.3	96.55172414	84	3	150	2372	1	2211	0	3563

TRINITY_DN11021_c0_g1_i1	Glyma.18G120300.1	100	469	42	261	2460	1	2197	0	1201

TRINITY_DN11021_c0_g2_i1	Glyma.18G120300.1	100	469	42	195	2394	1	2197	0	1201

TRINITY_DN11021_c0_g3_i1	Glyma.18G120300.1	96.24776653	469	42	195	2394	1	2197	0	1201

TRINITY_DN15293_c0_g1_i3	Glyma.12G093800.1	97.91666667	362	40	467	2654	1	2188	0	1659

TRINITY_DN15952_c0_g1_i1	Glyma.17G236800.1	100	342	9	344	2558	2334	117	0	2137

TRINITY_DN15293_c0_g1_i2	Glyma.12G093800.1	100	338	40	530	2717	1	2188	0	1792

TRINITY_DN16231_c1_g1_i2	Glyma.09G073600.3	98.12792512	289	8	114	2331	35	2252	0	2451

TRINITY_DN16231_c1_g1_i2	Glyma.09G073600.5	96.42857143	289	8	114	2331	35	2252	0	2451

TRINITY_DN16231_c1_g1_i2	Glyma.09G073600.2	96.42857143	289	8	114	2331	35	2252	0	2451

TRINITY_DN16231_c1_g1_i2	Glyma.09G073600.1	98.38709677	289	8	114	2331	35	2252	0	2451

TRINITY_DN15530_c0_g1_i1	Glyma.12G204400.1	95.65217391	165	5	300	2516	2220	1	0	3144

TRINITY_DN16134_c0_g3_i1	Glyma.08G354500.4	100	159	15	253	2433	2199	1	0	2809

TRINITY_DN16134_c0_g3_i1	Glyma.08G354500.2	97.07865169	159	15	253	2433	2199	1	0	2809

TRINITY_DN11922_c0_g2_i1	Glyma.10G060000.1	98.61111111	108	9	474	2692	2198	1	0	3330

TRINITY_DN11922_c0_g2_i1	Glyma.13G146700.1	95.65217391	101	12	474	2692	2192	1	0	3330

TRINITY_DN16431_c0_g1_i4	Glyma.03G044000.1	100	401	32	2681	4870	2517	322	0	1519

TRINITY_DN16431_c0_g1_i5	Glyma.03G044000.1	95.45454545	401	32	2661	4850	2517	322	0	1519

TRINITY_DN16431_c0_g1_i6	Glyma.03G044000.1	97.93814433	401	32	2324	4513	2517	322	0	1519

TRINITY_DN16431_c0_g1_i7	Glyma.03G044000.1	98.86363636	401	32	2304	4493	2517	322	0	1519

TRINITY_DN15952_c0_g1_i1	Glyma.14G087500.1	95.53571429	330	6	344	2558	2325	108	0	2215

TRINITY_DN15789_c0_g1_i1	Glyma.14G075800.2	100	283	12	1996	4212	3011	5215	0	2405

TRINITY_DN16231_c1_g1_i1	Glyma.15G182600.2	100	280	6	114	2331	35	2252	0	2512

TRINITY_DN16231_c1_g1_i2	Glyma.15G182600.1	98.125	280	6	114	2331	35	2252	0	2512

TRINITY_DN16043_c0_g1_i1	Glyma.08G293900.2	99.05660377	267	1	107	2327	1057	3276	0	2615

TRINITY_DN15710_c0_g2_i1	Glyma.15G042900.1	98.85057471	236	11	333	2548	2204	1	0	2652

TRINITY_DN15710_c0_g2_i2	Glyma.15G042900.1	95.88235294	236	11	333	2548	2204	1	0	2652

TRINITY_DN10255_c0_g1_i2	Glyma.12G039200.1	98.98989899	197	10	1	2209	303	2520	0	2913

TRINITY_DN15876_c0_g1_i3	Glyma.04G039400.1	97.77777778	190	6	133	2346	181	2400	0	2998

TRINITY_DN10255_c0_g1_i2	Glyma.11G113300.1	98.98989899	187	9	1	2209	291	2508	0	2968

TRINITY_DN13772_c0_g1_i1	Glyma.19G214100.1	100	168	1	693	2913	598	2817	0	3164

TRINITY_DN13772_c0_g1_i2	Glyma.19G214100.1	96.91119691	168	1	851	3071	598	2817	0	3164

TRINITY_DN13294_c0_g1_i1	Glyma.02G053800.4	95.92760181	164	3	471	2690	2255	48	0	3103

TRINITY_DN13294_c0_g1_i1	Glyma.02G053800.1	100	164	3	471	2690	2255	48	0	3103

TRINITY_DN13294_c0_g1_i1	Glyma.02G053800.3	99.27007299	164	3	471	2690	2255	48	0	3103

TRINITY_DN13294_c0_g1_i1	Glyma.02G053800.2	99.27007299	164	3	471	2690	2255	48	0	3103

TRINITY_DN15511_c0_g1_i1	Glyma.19G150400.1	99.54397394	143	2	80	2299	2220	1	0	3297

TRINITY_DN16382_c0_g3_i3	Glyma.11G169700.1	96.6442953	133	7	177	2397	1	2199	0	3223

TRINITY_DN16382_c0_g3_i3	Glyma.18G060600.1	96.6507177	126	7	177	2397	1	2199	0	3262

TRINITY_DN11021_c0_g1_i1	Glyma.08G300100.1	100	467	39	261	2460	1	2197	0	1234

TRINITY_DN11021_c0_g1_i1	Glyma.08G300100.2	100	467	39	261	2460	1	2197	0	1234

TRINITY_DN11021_c0_g2_i1	Glyma.08G300100.1	100	467	39	195	2394	1	2197	0	1234

TRINITY_DN11021_c0_g2_i1	Glyma.08G300100.2	100	467	39	195	2394	1	2197	0	1234

TRINITY_DN11021_c0_g3_i1	Glyma.08G300100.1	97.43589744	467	39	195	2394	1	2197	0	1234

TRINITY_DN11021_c0_g3_i1	Glyma.08G300100.2	100	467	39	195	2394	1	2197	0	1234

TRINITY_DN36917_c0_g1_i1	Glyma.14G206200.4	100	425	35	9	2204	2213	15	0	1454

TRINITY_DN15345_c0_g1_i3	Glyma.12G099500.1	100	349	20	554	2758	158	2365	0	1991

TRINITY_DN15345_c0_g1_i3	Glyma.12G099500.4	97.08029197	349	20	554	2758	158	2365	0	1991

TRINITY_DN15345_c0_g1_i4	Glyma.12G099500.1	98.66666667	349	20	360	2564	158	2365	0	1991

TRINITY_DN15345_c0_g1_i4	Glyma.12G099500.4	98.26086957	349	20	360	2564	158	2365	0	1991

TRINITY_DN10733_c0_g1_i1	Glyma.11G020700.2	97.22222222	301	12	639	2852	384	2594	0	2335

TRINITY_DN13432_c0_g2_i1	Glyma.07G114400.3	96.49681529	250	10	343	2548	2218	1	0	2612

TRINITY_DN13432_c0_g2_i1	Glyma.07G114400.1	96.49805447	250	10	343	2548	2218	1	0	2612

TRINITY_DN13432_c0_g2_i1	Glyma.07G114400.2	96.49805447	250	10	343	2548	2218	1	0	2612

TRINITY_DN10217_c0_g2_i1	Glyma.01G128700.1	97.16312057	204	23	728	2916	5085	2885	0	2647

TRINITY_DN10217_c0_g2_i1	Glyma.01G128700.2	97.14285714	204	23	728	2916	5085	2885	0	2647

TRINITY_DN15849_c0_g1_i2	Glyma.08G297400.1	97.68637532	201	3	660	2873	1	2217	0	2928

TRINITY_DN12860_c0_g1_i2	Glyma.16G006000.2	99.32885906	166	11	495	2708	292	2484	0	2966

TRINITY_DN15511_c0_g1_i1	Glyma.03G147000.1	98.66666667	153	0	80	2299	2220	1	0	3253

TRINITY_DN8660_c0_g1_i2	Glyma.16G182300.1	100	149	1	26	2245	1	2217	0	3254

TRINITY_DN8660_c0_g1_i2	Glyma.09G136900.1	100	136	0	26	2245	1	2220	0	3347

TRINITY_DN14841_c0_g1_i1	Glyma.06G000200.1	97.30033746	130	3	467	2680	1	2217	0	3321

TRINITY_DN14841_c0_g1_i1	Glyma.04G000300.2	97.65395894	122	4	467	2680	1	2205	0	3288

TRINITY_DN14841_c0_g1_i1	Glyma.04G000300.1	95.23809524	122	4	467	2680	1	2205	0	3288

TRINITY_DN16065_c0_g1_i4	Glyma.13G244300.2	95.32163743	284	43	246	2405	1	2202	0	2034

TRINITY_DN16065_c0_g1_i2	Glyma.13G244300.2	95.21276596	270	43	572	2731	1	2202	0	2111

TRINITY_DN13432_c0_g2_i1	Glyma.03G111200.1	99.02439024	255	9	343	2548	2218	1	0	2595

TRINITY_DN13432_c0_g2_i1	Glyma.03G111200.2	98.48484848	255	9	343	2548	2218	1	0	2595

TRINITY_DN11713_c0_g1_i1	Glyma.13G114900.2	100	119	0	504	2722	2219	1	0	3439

TRINITY_DN16254_c0_g1_i10	Glyma.17G057800.4	95.16129032	505	49	1	2186	2561	370	0	924

TRINITY_DN16254_c0_g1_i10	Glyma.17G057800.3	95.16129032	505	49	1	2186	2561	370	0	924

TRINITY_DN16254_c0_g1_i10	Glyma.17G057800.2	95.16129032	505	49	1	2186	2561	370	0	924

TRINITY_DN15530_c0_g1_i1	Glyma.13G296900.1	100	166	2	300	2516	2217	1	0	3164

TRINITY_DN15994_c0_g1_i2	Glyma.13G367300.3	96.2962963	153	11	745	2962	3021	835	0	3048

TRINITY_DN15352_c0_g1_i3	Glyma.17G006100.1	97.87234043	146	3	92	2308	13	2226	0	3254

TRINITY_DN14680_c0_g1_i1	Glyma.19G175600.1	97.36842105	380	29	3	2187	549	2751	0	1692

TRINITY_DN14680_c0_g1_i1	Glyma.19G175600.2	97.36842105	380	29	3	2187	549	2751	0	1692

TRINITY_DN17528_c0_g1_i1	Glyma.05G036100.2	100	380	31	20	2215	2193	1	0	1698

TRINITY_DN17528_c0_g1_i1	Glyma.05G036100.1	100	380	31	20	2215	2193	1	0	1698

TRINITY_DN15601_c0_g2_i1	Glyma.09G091200.4	95.78947368	335	34	652	2832	1	2193	0	1851

TRINITY_DN15601_c0_g2_i1	Glyma.09G091200.3	98.82352941	335	34	652	2832	1	2193	0	1851

TRINITY_DN15601_c0_g2_i1	Glyma.09G091200.2	96.96969697	335	34	652	2832	1	2193	0	1851

TRINITY_DN15601_c0_g2_i1	Glyma.09G091200.1	100	335	34	652	2832	1	2193	0	1851

TRINITY_DN13972_c0_g1_i1	Glyma.14G209900.1	97.14285714	320	14	557	2766	2264	55	0	2231

TRINITY_DN2154_c0_g1_i1	Glyma.03G121300.4	100	223	3	271	2486	2199	1	0	2736

TRINITY_DN14670_c0_g1_i3	Glyma.13G065500.15	99.32432432	223	17	1178	3390	9	2182	0	2555

TRINITY_DN15849_c0_g1_i2	Glyma.18G124500.1	99.48586118	186	4	660	2873	1	2214	0	3025

TRINITY_DN8101_c0_g1_i1	Glyma.04G015000.1	97.84946237	182	2	140	2350	1	2217	0	3048

TRINITY_DN11025_c0_g2_i1	Glyma.05G167300.1	96.8503937	181	9	208	2415	1	2211	0	2994

TRINITY_DN11025_c0_g2_i2	Glyma.05G167300.1	98.73417722	181	9	73	2280	1	2211	0	2994

TRINITY_DN15909_c0_g4_i1	Glyma.07G031100.2	99.5951417	151	0	1	2217	214	2430	0	3258

TRINITY_DN15909_c0_g4_i1	Glyma.07G031100.1	100	151	0	1	2217	214	2430	0	3258

TRINITY_DN15909_c0_g4_i1	Glyma.07G031100.3	98.63013699	151	0	1	2217	214	2430	0	3258

TRINITY_DN14688_c0_g2_i2	Glyma.13G325000.1	95.38461538	127	7	478	2679	2217	1	0	3293

TRINITY_DN7785_c0_g1_i1	Glyma.10G102400.2	100	106	1	816	3029	2217	1	0	3487

TRINITY_DN14756_c0_g1_i3	Glyma.16G209600.2	97.05882353	407	42	429	2616	2448	277	0	1373

TRINITY_DN14756_c0_g1_i3	Glyma.16G209600.1	97.05882353	407	42	429	2616	2448	277	0	1373

TRINITY_DN14604_c0_g1_i1	Glyma.09G195100.1	97.5257732	371	12	309	2518	95	2292	0	1882

TRINITY_DN15706_c0_g1_i3	Glyma.08G074700.1	98.97959184	299	9	295	2505	88	2298	0	2372

TRINITY_DN13116_c0_g1_i1	Glyma.09G068800.2	97.39130435	166	4	512	2715	2212	1	0	3070

TRINITY_DN13116_c0_g1_i2	Glyma.09G068800.2	97.85867238	166	4	274	2477	2212	1	0	3070

TRINITY_DN13778_c1_g3_i3	Glyma.16G065700.1	100	351	20	200	2398	3606	1405	0	1958

TRINITY_DN630_c0_g1_i1	Glyma.14G012900.3	97.43589744	303	28	77	2257	2187	1	0	2012

TRINITY_DN630_c0_g1_i1	Glyma.14G012900.1	97.43589744	303	28	77	2257	2187	1	0	2012

TRINITY_DN14622_c0_g1_i1	Glyma.10G179700.3	99.03846154	178	17	67	2270	3	2196	0	2898

TRINITY_DN17528_c0_g1_i1	Glyma.06G184200.4	100	174	1	1	2215	2212	1	0	3107

TRINITY_DN17528_c0_g1_i1	Glyma.06G184200.3	100	174	1	1	2215	2212	1	0	3107

TRINITY_DN17528_c0_g1_i1	Glyma.06G184200.2	100	174	1	1	2215	2212	1	0	3107

TRINITY_DN17528_c0_g1_i1	Glyma.06G184200.1	100	174	1	1	2215	2212	1	0	3107

TRINITY_DN17528_c0_g1_i1	Glyma.06G184200.5	100	174	1	1	2215	2212	1	0	3107

TRINITY_DN17528_c0_g1_i1	Glyma.04G180900.6	100	172	2	1	2215	2212	1	0	3118

TRINITY_DN17528_c0_g1_i1	Glyma.04G180900.4	100	172	2	1	2215	2212	1	0	3118

TRINITY_DN17528_c0_g1_i1	Glyma.04G180900.1	100	172	2	1	2215	2212	1	0	3118

TRINITY_DN7921_c1_g1_i1	Glyma.07G248800.1	100	151	16	1	2207	584	2775	0	3053

TRINITY_DN7921_c1_g1_i1	Glyma.07G248800.3	100	151	16	1	2207	584	2775	0	3053

TRINITY_DN7921_c1_g1_i1	Glyma.07G248800.4	100	151	16	1	2207	584	2775	0	3053

TRINITY_DN7921_c1_g1_i2	Glyma.07G248800.1	100	151	16	1	2207	584	2775	0	3053

TRINITY_DN7921_c1_g1_i2	Glyma.07G248800.3	100	151	16	1	2207	584	2775	0	3053

TRINITY_DN7921_c1_g1_i2	Glyma.07G248800.4	100	151	16	1	2207	584	2775	0	3053

TRINITY_DN7921_c1_g1_i3	Glyma.07G248800.1	100	151	16	1	2207	584	2775	0	3053

TRINITY_DN7921_c1_g1_i3	Glyma.07G248800.3	100	151	16	1	2207	584	2775	0	3053

TRINITY_DN7921_c1_g1_i3	Glyma.07G248800.4	100	151	16	1	2207	584	2775	0	3053

TRINITY_DN7921_c1_g1_i1	Glyma.07G248800.2	100	151	16	1	2207	86	2277	0	3053

TRINITY_DN7921_c1_g1_i2	Glyma.07G248800.2	100	151	16	1	2207	86	2277	0	3053

TRINITY_DN7921_c1_g1_i3	Glyma.07G248800.2	100	151	16	1	2207	86	2277	0	3053

TRINITY_DN14882_c0_g1_i1	Glyma.03G219500.2	98.40764331	145	6	655	2853	2263	49	0	3184

TRINITY_DN14882_c0_g1_i2	Glyma.03G219500.2	98.24561404	145	6	1332	3530	2263	49	0	3184

TRINITY_DN16382_c0_g3_i4	Glyma.11G169700.1	96.66666667	132	4	177	2391	1	2199	0	3256

TRINITY_DN11893_c0_g1_i1	Glyma.02G200700.8	98.01192843	131	2	432	2643	2	2216	0	3345

TRINITY_DN16382_c0_g3_i4	Glyma.11G169700.2	96.66666667	131	5	177	2391	1	2190	0	3203

TRINITY_DN16382_c0_g3_i4	Glyma.18G060600.1	96.6442953	125	4	177	2391	1	2199	0	3295

TRINITY_DN13972_c0_g1_i1	Glyma.02G240400.1	95	324	6	557	2767	2264	54	0	2255

TRINITY_DN15706_c0_g1_i5	Glyma.08G074700.1	96.15384615	270	6	295	2505	88	2298	0	2555

TRINITY_DN14670_c0_g1_i2	Glyma.19G019100.4	100	214	8	1178	3386	9	2214	0	2819

TRINITY_DN8101_c0_g1_i1	Glyma.06G015000.2	98.34710744	188	2	140	2350	1	2214	0	3027

TRINITY_DN8101_c0_g1_i1	Glyma.06G015000.1	99.68847352	188	2	140	2350	1	2214	0	3027

TRINITY_DN13032_c0_g2_i1	Glyma.08G019600.1	100	165	1	537	2747	2376	163	0	3155

TRINITY_DN15373_c0_g1_i1	Glyma.13G097000.1	95.69892473	154	3	229	2436	2211	1	0	3177

TRINITY_DN15373_c0_g1_i2	Glyma.13G097000.1	95.70093458	154	3	229	2436	2211	1	0	3177

TRINITY_DN15373_c0_g1_i4	Glyma.13G097000.1	95.12195122	154	3	229	2436	2211	1	0	3177

TRINITY_DN15373_c0_g1_i1	Glyma.17G062500.1	95.69892473	149	2	229	2436	2214	1	0	3225

TRINITY_DN15373_c0_g1_i2	Glyma.17G062500.1	95.70063694	149	2	229	2436	2214	1	0	3225

TRINITY_DN15373_c0_g1_i4	Glyma.17G062500.1	95.12195122	149	2	229	2436	2214	1	0	3225

TRINITY_DN15909_c0_g4_i1	Glyma.13G364900.1	98.828125	299	0	4	2216	211	2423	0	2431

TRINITY_DN15909_c0_g4_i1	Glyma.13G364900.2	100	299	0	4	2216	211	2423	0	2431

TRINITY_DN16523_c0_g1_i1	Glyma.19G178700.1	100	246	32	91	2247	1	2190	0	2213

TRINITY_DN16523_c0_g1_i1	Glyma.19G178700.3	100	246	33	91	2247	1	2187	0	2194

TRINITY_DN15994_c0_g1_i3	Glyma.13G367300.3	96.52928416	214	10	745	2947	3021	835	0	2669

TRINITY_DN10751_c0_g2_i1	Glyma.05G014900.1	96.78362573	212	25	130	2313	1	2199	0	2634

TRINITY_DN10217_c0_g2_i1	Glyma.03G041600.1	97.14285714	207	19	728	2916	5067	2873	0	2669

TRINITY_DN10217_c0_g2_i1	Glyma.03G041600.2	97.14828897	207	19	728	2916	4872	2678	0	2669

TRINITY_DN16254_c0_g1_i10	Glyma.13G101700.1	95.16129032	510	42	1	2186	2561	370	0	963

TRINITY_DN12949_c0_g1_i1	Glyma.15G151000.1	95.52238806	498	29	362	2556	2400	206	0	1107

TRINITY_DN12949_c0_g1_i1	Glyma.15G151000.3	96.55172414	498	29	362	2556	2340	146	0	1107

TRINITY_DN13324_c0_g2_i1	Glyma.13G201800.1	98.42519685	218	18	572	2775	2860	675	0	2658

TRINITY_DN13324_c0_g2_i1	Glyma.13G201800.2	98.42519685	218	18	572	2775	2656	471	0	2658

TRINITY_DN12988_c0_g1_i1	Glyma.13G127100.1	99.13793103	201	3	60	2267	350	2560	0	2939

TRINITY_DN13046_c0_g3_i1	Glyma.09G059200.1	98.98989899	186	15	1323	3508	2210	1	0	2874

TRINITY_DN12674_c0_g2_i1	Glyma.01G000900.1	98.2300885	124	0	215	2426	2292	81	0	3398

TRINITY_DN16004_c1_g1_i4	Glyma.03G113400.1	98.30508475	526	48	284	2470	5	2188	0	839

TRINITY_DN15516_c0_g1_i1	Glyma.13G303400.1	100	446	34	318	2504	291	2477	0	1303

TRINITY_DN5606_c0_g1_i1	Glyma.14G064400.1	96.79715302	310	29	51	2248	1	2189	0	2139

TRINITY_DN14377_c0_g1_i2	Glyma.10G090600.1	100	210	14	522	2708	1	2205	0	2726

TRINITY_DN14377_c0_g1_i3	Glyma.10G090600.1	100	210	14	580	2766	1	2205	0	2726

TRINITY_DN13032_c0_g2_i1	Glyma.05G213100.1	98.41269841	171	0	537	2747	2373	163	0	3136

TRINITY_DN3358_c0_g1_i1	Glyma.05G180200.1	97.87234043	170	10	62	2254	2208	1	0	3005

TRINITY_DN3358_c0_g2_i1	Glyma.05G180200.1	100	170	10	62	2254	2208	1	0	3005

TRINITY_DN3358_c0_g3_i1	Glyma.05G180200.1	99.3006993	170	10	62	2254	2208	1	0	3005

TRINITY_DN3358_c0_g4_i1	Glyma.05G180200.1	96.64634146	170	10	62	2254	2208	1	0	3005

TRINITY_DN9393_c0_g1_i2	Glyma.09G172000.1	98.80952381	155	10	255	2463	2691	492	0	3140

TRINITY_DN15293_c0_g1_i4	Glyma.11G179600.3	100	154	15	467	2658	1	2198	0	3024

TRINITY_DN15293_c0_g1_i4	Glyma.11G179600.1	95.45454545	154	15	467	2658	1	2198	0	3024

TRINITY_DN16408_c2_g1_i2	Glyma.03G069800.1	96.3060686	138	7	213	2402	2208	1	0	3164

TRINITY_DN15293_c0_g1_i1	Glyma.11G179600.3	97.91666667	128	15	530	2721	1	2198	0	3168

TRINITY_DN15293_c0_g1_i1	Glyma.11G179600.1	97.91666667	128	15	530	2721	1	2198	0	3168

TRINITY_DN14688_c0_g2_i2	Glyma.12G174400.2	99.06542056	125	3	478	2679	2211	1	0	3332

TRINITY_DN14688_c0_g2_i2	Glyma.12G174400.1	99.06542056	125	3	478	2679	2211	1	0	3332

TRINITY_DN263_c0_g1_i1	Glyma.01G002100.2	98.01980198	404	27	3	2161	2884	681	0	1474

TRINITY_DN263_c0_g1_i1	Glyma.01G002100.1	100	404	27	3	2161	2884	681	0	1474

TRINITY_DN16353_c0_g1_i3	Glyma.19G064000.1	96.34615385	233	4	667	2874	330	2537	0	2765

TRINITY_DN9446_c0_g1_i1	Glyma.19G166500.1	97.61904762	223	2	293	2496	2210	1	0	2808

TRINITY_DN15648_c0_g2_i4	Glyma.10G153800.12	96.12211221	212	6	115	2319	3051	844	0	2861

TRINITY_DN15648_c0_g2_i4	Glyma.10G153800.10	97.46136865	212	6	115	2319	3051	844	0	2861

TRINITY_DN15648_c0_g2_i4	Glyma.10G153800.9	95.86330935	212	6	115	2319	3051	844	0	2861

TRINITY_DN15648_c0_g2_i4	Glyma.10G153800.8	97.43589744	212	6	115	2319	3051	844	0	2861

TRINITY_DN15648_c0_g2_i4	Glyma.10G153800.7	96.34146341	212	6	115	2319	3051	844	0	2861

TRINITY_DN15648_c0_g2_i4	Glyma.10G153800.6	98.99280576	212	6	115	2319	3051	844	0	2861

TRINITY_DN15648_c0_g2_i4	Glyma.10G153800.22	97.95918367	212	6	115	2319	3048	841	0	2861

TRINITY_DN15648_c0_g2_i4	Glyma.10G153800.21	96.12068966	212	6	115	2319	3048	841	0	2861

TRINITY_DN15648_c0_g2_i4	Glyma.10G153800.20	96.12068966	212	6	115	2319	3048	841	0	2861

TRINITY_DN15648_c0_g2_i4	Glyma.10G153800.19	96.12188366	212	6	115	2319	3048	841	0	2861

TRINITY_DN15648_c0_g2_i4	Glyma.10G153800.18	96.12188366	212	6	115	2319	3048	841	0	2861

TRINITY_DN12691_c0_g1_i2	Glyma.12G029500.2	97.33333333	171	5	226	2430	2205	1	0	3070

TRINITY_DN12691_c0_g1_i2	Glyma.11G104500.3	97.35099338	167	5	226	2430	2205	1	0	3092

TRINITY_DN12691_c0_g1_i2	Glyma.11G104500.2	97.33333333	167	5	226	2430	2205	1	0	3092

TRINITY_DN17450_c0_g1_i1	Glyma.11G078300.3	100	319	34	312	2498	3735	1552	0	2008

TRINITY_DN17450_c0_g1_i1	Glyma.11G078300.1	99.53488372	319	34	312	2498	3735	1552	0	2008

TRINITY_DN17450_c0_g1_i1	Glyma.11G078300.2	100	319	34	312	2498	3678	1495	0	2008

TRINITY_DN3533_c0_g1_i1	Glyma.10G112500.2	95.48611111	268	20	783	2972	2193	1	0	2368

TRINITY_DN3533_c0_g1_i1	Glyma.10G112500.1	96.66666667	268	20	783	2972	2193	1	0	2368

TRINITY_DN9393_c0_g1_i2	Glyma.07G106100.1	98.80952381	153	4	255	2463	2625	426	0	3173

TRINITY_DN15329_c0_g2_i5	Glyma.02G085000.3	96.47058824	232	23	377	2566	2182	1	0	2508

TRINITY_DN15329_c0_g2_i5	Glyma.02G085000.1	96.47058824	232	23	377	2566	2182	1	0	2508

TRINITY_DN16130_c2_g6_i16	Glyma.19G196000.2	100	205	7	285	2485	1	2205	0	2878

TRINITY_DN16130_c2_g6_i5	Glyma.19G196000.2	98.67021277	205	7	285	2485	1	2205	0	2878

TRINITY_DN15648_c0_g2_i4	Glyma.20G234500.1	97.59036145	203	6	115	2319	3135	931	0	2915

TRINITY_DN15648_c0_g2_i4	Glyma.20G234500.5	96.73913043	203	6	115	2319	3090	886	0	2915

TRINITY_DN15648_c0_g2_i4	Glyma.20G234500.3	96.55172414	203	6	115	2319	3048	844	0	2915

TRINITY_DN8969_c0_g1_i1	Glyma.19G167900.1	96.96969697	203	18	264	2463	1	2167	0	2636

TRINITY_DN8969_c0_g1_i1	Glyma.19G167900.2	96.94656489	203	18	264	2463	1	2167	0	2636

TRINITY_DN8969_c0_g1_i1	Glyma.19G167900.8	96.96969697	203	18	264	2463	1	2167	0	2636

TRINITY_DN8969_c0_g1_i1	Glyma.19G167900.3	96.9581749	203	18	264	2463	1	2167	0	2636

TRINITY_DN16130_c2_g6_i15	Glyma.19G196000.2	99.57983193	157	7	285	2485	1	2205	0	3144

TRINITY_DN16130_c2_g6_i9	Glyma.19G196000.2	96.42857143	157	7	285	2485	1	2205	0	3144

TRINITY_DN16408_c2_g1_i2	Glyma.03G069800.3	99.77426637	138	6	213	2402	2205	1	0	3177

TRINITY_DN5791_c0_g1_i1	Glyma.08G313200.1	97.46835443	137	5	1	2199	580	2781	0	3221

TRINITY_DN16408_c2_g1_i2	Glyma.03G069800.6	97.11111111	137	8	213	2402	2196	1	0	3125

TRINITY_DN5791_c0_g1_i1	Glyma.18G101400.1	100	133	7	1	2199	574	2772	0	3225

TRINITY_DN12257_c0_g1_i2	Glyma.09G021400.2	97.5	125	1	278	2485	1	2205	0	3365

TRINITY_DN17450_c0_g1_i1	Glyma.01G164800.1	100	325	25	312	2498	3744	1555	0	2036

TRINITY_DN17450_c0_g1_i1	Glyma.01G164800.2	99.25373134	325	25	312	2498	3741	1552	0	2036

TRINITY_DN13600_c1_g1_i2	Glyma.15G205600.1	96.42857143	177	9	1	2196	832	3021	0	2915

TRINITY_DN13600_c1_g1_i3	Glyma.15G205600.1	97.44680851	177	9	1	2196	832	3021	0	2915

TRINITY_DN13826_c0_g3_i1	Glyma.04G058300.1	100	226	26	194	2332	2229	25	0	2383

TRINITY_DN11155_c0_g1_i1	Glyma.08G096100.1	100	209	0	377	2582	2310	105	0	2916

TRINITY_DN15474_c0_g1_i2	Glyma.02G146900.6	100	198	11	1739	3921	1445	3645	0	2796

TRINITY_DN15474_c0_g1_i2	Glyma.02G146900.1	98.2300885	198	11	1739	3921	1445	3645	0	2796

TRINITY_DN15474_c0_g1_i2	Glyma.02G146900.4	98.2300885	198	11	1739	3921	1445	3645	0	2796

TRINITY_DN15474_c0_g1_i4	Glyma.02G146900.6	100	198	11	1706	3888	1445	3645	0	2796

TRINITY_DN15474_c0_g1_i4	Glyma.02G146900.1	95.55555556	198	11	1706	3888	1445	3645	0	2796

TRINITY_DN15474_c0_g1_i4	Glyma.02G146900.4	96.80851064	198	11	1706	3888	1445	3645	0	2796

TRINITY_DN15474_c0_g1_i2	Glyma.02G146900.5	100	198	11	1739	3921	1442	3642	0	2796

TRINITY_DN15474_c0_g1_i2	Glyma.02G146900.3	96.33699634	198	11	1739	3921	1442	3642	0	2796

TRINITY_DN15474_c0_g1_i2	Glyma.02G146900.2	98.6013986	198	11	1739	3921	1442	3642	0	2796

TRINITY_DN15474_c0_g1_i4	Glyma.02G146900.5	100	198	11	1706	3888	1442	3642	0	2796

TRINITY_DN15474_c0_g1_i4	Glyma.02G146900.3	99.02912621	198	11	1706	3888	1442	3642	0	2796

TRINITY_DN15474_c0_g1_i4	Glyma.02G146900.2	100	198	11	1706	3888	1442	3642	0	2796

TRINITY_DN3076_c0_g1_i1	Glyma.02G203200.3	100	190	28	180	2353	1	2181	0	2652

TRINITY_DN3076_c0_g1_i1	Glyma.02G203200.2	100	190	28	180	2353	1	2181	0	2652

TRINITY_DN3076_c0_g1_i1	Glyma.02G203200.1	98.13084112	190	28	180	2353	1	2181	0	2652

TRINITY_DN3076_c0_g1_i1	Glyma.02G203200.4	100	190	28	180	2353	1	2181	0	2652

TRINITY_DN11915_c0_g1_i1	Glyma.11G233800.1	95.04950495	178	26	634	2790	2245	40	0	2771

TRINITY_DN15293_c0_g1_i4	Glyma.12G093800.1	98.7012987	160	7	467	2662	1	2193	0	3038

TRINITY_DN15293_c0_g1_i1	Glyma.12G093800.1	96.55172414	136	7	530	2725	1	2193	0	3171

TRINITY_DN16163_c0_g1_i2	Glyma.12G194400.4	100	120	6	622	2824	102	2295	0	3312

TRINITY_DN16163_c0_g1_i2	Glyma.12G194400.5	96.61016949	120	6	622	2824	102	2295	0	3312

TRINITY_DN16163_c0_g1_i2	Glyma.12G194400.6	97.64309764	120	6	622	2824	102	2295	0	3312

TRINITY_DN16163_c0_g1_i5	Glyma.12G194400.4	100	120	6	131	2333	102	2295	0	3312

TRINITY_DN16163_c0_g1_i5	Glyma.12G194400.5	96.72131148	120	6	131	2333	102	2295	0	3312

TRINITY_DN16163_c0_g1_i5	Glyma.12G194400.6	100	120	6	131	2333	102	2295	0	3312

TRINITY_DN16163_c0_g1_i6	Glyma.12G194400.4	95.51020408	120	6	600	2802	102	2295	0	3312

TRINITY_DN16163_c0_g1_i6	Glyma.12G194400.5	97.70992366	120	6	600	2802	102	2295	0	3312

TRINITY_DN16163_c0_g1_i6	Glyma.12G194400.6	97.71167048	120	6	600	2802	102	2295	0	3312

TRINITY_DN16163_c0_g1_i2	Glyma.12G194400.2	97.78597786	118	7	622	2824	102	2280	0	3227

TRINITY_DN16163_c0_g1_i2	Glyma.12G194400.3	99.14893617	118	7	622	2824	102	2280	0	3227

TRINITY_DN16163_c0_g1_i5	Glyma.12G194400.2	98.70967742	118	7	131	2333	102	2280	0	3227

TRINITY_DN16163_c0_g1_i5	Glyma.12G194400.3	99.51690821	118	7	131	2333	102	2280	0	3227

TRINITY_DN16163_c0_g1_i6	Glyma.12G194400.2	99.3902439	118	7	600	2802	102	2280	0	3227

TRINITY_DN16163_c0_g1_i6	Glyma.12G194400.3	100	118	7	600	2802	102	2280	0	3227

TRINITY_DN14377_c0_g1_i2	Glyma.15G276400.3	97.8021978	193	13	522	2708	1	2199	0	2848

TRINITY_DN14377_c0_g1_i2	Glyma.15G276400.1	96.76549865	193	13	522	2708	1	2199	0	2848

TRINITY_DN14377_c0_g1_i2	Glyma.15G276400.4	96.76549865	193	13	522	2708	1	2199	0	2848

TRINITY_DN14377_c0_g1_i2	Glyma.15G276400.2	96.76616915	193	13	522	2708	1	2199	0	2848

TRINITY_DN14377_c0_g1_i3	Glyma.15G276400.3	100	193	13	580	2766	1	2199	0	2848

TRINITY_DN14377_c0_g1_i3	Glyma.15G276400.1	100	193	13	580	2766	1	2199	0	2848

TRINITY_DN14377_c0_g1_i3	Glyma.15G276400.4	100	193	13	580	2766	1	2199	0	2848

TRINITY_DN14377_c0_g1_i3	Glyma.15G276400.2	100	193	13	580	2766	1	2199	0	2848

TRINITY_DN11155_c0_g1_i1	Glyma.05G140800.1	100	187	0	378	2582	2327	123	0	3037

TRINITY_DN13600_c1_g1_i2	Glyma.09G097600.1	100	161	4	1	2196	871	3063	0	3044

TRINITY_DN13600_c1_g1_i3	Glyma.09G097600.1	100	161	4	1	2196	871	3063	0	3044

TRINITY_DN13600_c1_g1_i2	Glyma.09G097600.2	97.63205829	161	4	1	2196	865	3057	0	3044

TRINITY_DN13600_c1_g1_i3	Glyma.09G097600.2	95.16129032	161	4	1	2196	865	3057	0	3044

TRINITY_DN16065_c0_g1_i3	Glyma.13G244300.2	100	156	1	246	2450	1	2202	0	3188

TRINITY_DN16408_c2_g1_i2	Glyma.01G099600.1	97.69230769	143	7	213	2402	2178	1	0	3009

TRINITY_DN16065_c0_g1_i5	Glyma.13G244300.2	97.33333333	142	1	572	2776	1	2202	0	3265

TRINITY_DN16408_c2_g1_i2	Glyma.03G069800.2	96.83544304	139	4	213	2402	2202	1	0	3186

TRINITY_DN16352_c0_g2_i1	Glyma.08G194700.1	100	426	50	2521	4690	2494	316	0	1328

TRINITY_DN15499_c0_g1_i3	Glyma.13G235000.1	97.09302326	416	25	5	2187	420	2611	0	1552

TRINITY_DN14604_c0_g1_i5	Glyma.09G195100.1	95.65217391	371	11	309	2506	95	2292	0	1938

TRINITY_DN15474_c0_g1_i2	Glyma.10G027000.1	98.93617021	211	5	1739	3921	1412	3612	0	2747

TRINITY_DN15474_c0_g1_i4	Glyma.10G027000.1	98.38709677	211	5	1706	3888	1412	3612	0	2747

TRINITY_DN8780_c0_g1_i1	Glyma.09G097500.2	97.60479042	205	11	804	2995	1	2183	0	2721

TRINITY_DN9192_c0_g1_i3	Glyma.10G248400.1	100	160	6	687	2888	2193	1	0	3099

TRINITY_DN2525_c0_g2_i1	Glyma.12G135700.3	100	367	9	5	2203	910	3105	0	1964

TRINITY_DN3533_c0_g1_i1	Glyma.02G160100.1	100	278	28	785	2972	2179	1	0	2276

TRINITY_DN11893_c0_g1_i1	Glyma.02G200700.5	98	131	2	619	2818	54	2256	0	3323

TRINITY_DN11893_c0_g1_i1	Glyma.02G200700.3	98.00664452	131	2	619	2818	54	2256	0	3323

TRINITY_DN263_c0_g1_i1	Glyma.07G130400.2	98.12286689	404	32	3	2160	2917	727	0	1472

TRINITY_DN263_c0_g1_i1	Glyma.07G130400.1	96.06060606	404	32	3	2160	2917	727	0	1472

TRINITY_DN263_c0_g1_i1	Glyma.07G130400.3	96.0591133	404	32	3	2160	2908	718	0	1472

TRINITY_DN15510_c0_g1_i2	Glyma.09G168400.1	99.31506849	355	19	96	2270	115	2310	0	1886

TRINITY_DN13826_c0_g3_i1	Glyma.06G059000.1	100	230	35	194	2335	2205	19	0	2307

TRINITY_DN16274_c0_g1_i1	Glyma.12G189500.3	100	210	10	503	2694	1	2189	0	2754

TRINITY_DN16274_c0_g1_i2	Glyma.12G189500.3	100	210	10	503	2694	1	2189	0	2754

TRINITY_DN15310_c0_g1_i2	Glyma.09G102900.1	100	201	21	316	2492	1	2186	0	2687

TRINITY_DN6753_c0_g1_i1	Glyma.13G178200.1	100	189	18	1	2182	756	2931	0	2723

TRINITY_DN6753_c0_g1_i1	Glyma.13G178200.9	95.35864979	189	18	1	2182	756	2931	0	2723

TRINITY_DN9693_c0_g1_i1	Glyma.13G156400.1	96.73913043	183	2	42	2243	1	2199	0	3033

TRINITY_DN9693_c0_g1_i1	Glyma.17G104000.1	96.72897196	179	2	42	2243	1	2199	0	3055

TRINITY_DN9693_c0_g1_i1	Glyma.17G104000.2	96.74556213	178	3	42	2243	1	2184	0	2964

TRINITY_DN14622_c0_g1_i1	Glyma.10G179700.1	96.34551495	176	16	78	2270	97	2277	0	2898

TRINITY_DN14622_c0_g1_i1	Glyma.10G179700.2	99.27536232	176	16	78	2270	88	2268	0	2898

TRINITY_DN9192_c0_g1_i3	Glyma.20G145800.1	99.04761905	153	3	687	2888	2193	1	0	3160

TRINITY_DN16408_c2_g1_i2	Glyma.03G069800.8	95.65217391	144	8	213	2402	2199	1	0	3171

TRINITY_DN11021_c0_g1_i1	Glyma.19G021600.1	100	121	0	261	2462	1	2202	0	3397

TRINITY_DN11021_c0_g1_i1	Glyma.19G021600.2	100	121	0	261	2462	1	2202	0	3397

TRINITY_DN11021_c0_g2_i1	Glyma.19G021600.1	100	121	0	195	2396	1	2202	0	3397

TRINITY_DN11021_c0_g2_i1	Glyma.19G021600.2	100	121	0	195	2396	1	2202	0	3397

TRINITY_DN11021_c0_g3_i1	Glyma.19G021600.1	95.6043956	121	0	195	2396	1	2202	0	3397

TRINITY_DN11021_c0_g3_i1	Glyma.19G021600.2	99.56896552	121	0	195	2396	1	2202	0	3397

TRINITY_DN11021_c0_g1_i1	Glyma.13G063600.2	100	116	0	261	2462	1	2202	0	3424

TRINITY_DN11021_c0_g1_i1	Glyma.13G063600.1	100	116	0	261	2462	1	2202	0	3424

TRINITY_DN11021_c0_g2_i1	Glyma.13G063600.2	100	116	0	195	2396	1	2202	0	3424

TRINITY_DN11021_c0_g2_i1	Glyma.13G063600.1	100	116	0	195	2396	1	2202	0	3424

TRINITY_DN11021_c0_g3_i1	Glyma.13G063600.2	100	116	0	195	2396	1	2202	0	3424

TRINITY_DN11021_c0_g3_i1	Glyma.13G063600.1	100	116	0	195	2396	1	2202	0	3424

TRINITY_DN15634_c2_g1_i2	Glyma.02G159900.1	98.9010989	440	16	2195	4387	2443	254	0	1504

TRINITY_DN15634_c2_g1_i3	Glyma.02G159900.1	97.95918367	440	16	2306	4498	2443	254	0	1504

TRINITY_DN13377_c0_g1_i1	Glyma.07G253800.14	98.9010989	351	59	371	2474	1	2181	0	1363

TRINITY_DN13377_c0_g1_i1	Glyma.07G253800.8	96.73590504	351	59	371	2474	1	2181	0	1363

TRINITY_DN13377_c0_g1_i1	Glyma.07G253800.3	95.16129032	351	59	371	2474	1	2181	0	1363

TRINITY_DN13377_c0_g1_i2	Glyma.07G253800.14	96.61654135	351	59	410	2513	1	2181	0	1363

TRINITY_DN13377_c0_g1_i2	Glyma.07G253800.8	95.83333333	351	59	410	2513	1	2181	0	1363

TRINITY_DN13377_c0_g1_i2	Glyma.07G253800.3	100	351	59	410	2513	1	2181	0	1363

TRINITY_DN13377_c0_g1_i3	Glyma.07G253800.14	97.84946237	351	59	205	2308	1	2181	0	1363

TRINITY_DN13377_c0_g1_i3	Glyma.07G253800.8	97.84946237	351	59	205	2308	1	2181	0	1363

TRINITY_DN13377_c0_g1_i3	Glyma.07G253800.3	97.82608696	351	59	205	2308	1	2181	0	1363

TRINITY_DN15385_c0_g1_i1	Glyma.18G248200.2	99.54751131	304	45	1571	3718	2358	184	0	1869

TRINITY_DN15385_c0_g1_i1	Glyma.18G248200.1	99.67532468	304	45	1571	3718	2358	184	0	1869

TRINITY_DN15455_c0_g1_i3	Glyma.12G152300.1	99.07407407	269	5	157	2352	1	2199	0	2529

TRINITY_DN15455_c0_g1_i3	Glyma.12G152300.2	96.66666667	269	5	157	2352	1	2199	0	2529

TRINITY_DN14637_c0_g1_i1	Glyma.03G217000.3	98.02955665	160	14	1	2174	516	2710	0	2964

TRINITY_DN14637_c0_g1_i1	Glyma.03G217000.1	97.70992366	160	14	1	2174	516	2710	0	2964

TRINITY_DN12815_c1_g1_i3	Glyma.08G318100.1	96.98275862	313	49	1009	3158	2309	148	0	1760

TRINITY_DN16163_c0_g1_i1	Glyma.12G194400.4	96	254	15	600	2782	102	2293	0	2494

TRINITY_DN16163_c0_g1_i1	Glyma.12G194400.5	96	254	15	600	2782	102	2293	0	2494

TRINITY_DN16163_c0_g1_i1	Glyma.12G194400.6	96	254	15	600	2782	102	2293	0	2494

TRINITY_DN16163_c0_g1_i3	Glyma.12G194400.4	96.66666667	254	15	622	2804	102	2293	0	2494

TRINITY_DN16163_c0_g1_i3	Glyma.12G194400.5	100	254	15	622	2804	102	2293	0	2494

TRINITY_DN16163_c0_g1_i3	Glyma.12G194400.6	98.46938776	254	15	622	2804	102	2293	0	2494

TRINITY_DN16163_c0_g1_i4	Glyma.12G194400.4	100	254	15	131	2313	102	2293	0	2494

TRINITY_DN16163_c0_g1_i4	Glyma.12G194400.5	100	254	15	131	2313	102	2293	0	2494

TRINITY_DN16163_c0_g1_i4	Glyma.12G194400.6	100	254	15	131	2313	102	2293	0	2494

TRINITY_DN16163_c0_g1_i1	Glyma.12G194400.2	98.71794872	251	17	600	2782	102	2278	0	2414

TRINITY_DN16163_c0_g1_i1	Glyma.12G194400.3	95.48387097	251	17	600	2782	102	2278	0	2414

TRINITY_DN16163_c0_g1_i3	Glyma.12G194400.2	95.92875318	251	17	622	2804	102	2278	0	2414

TRINITY_DN16163_c0_g1_i3	Glyma.12G194400.3	95.65217391	251	17	622	2804	102	2278	0	2414

TRINITY_DN16163_c0_g1_i4	Glyma.12G194400.2	100	251	17	131	2313	102	2278	0	2414

TRINITY_DN16163_c0_g1_i4	Glyma.12G194400.3	100	251	17	131	2313	102	2278	0	2414

TRINITY_DN15206_c0_g1_i5	Glyma.03G081800.1	99.48586118	179	5	207	2402	201	2399	0	3038

TRINITY_DN15206_c0_g1_i5	Glyma.03G081800.2	95.97855228	179	5	207	2402	201	2399	0	3038

TRINITY_DN15691_c0_g1_i2	Glyma.10G028700.1	96.95121951	138	8	166	2346	1	2196	0	3149

TRINITY_DN15691_c0_g1_i2	Glyma.10G028700.3	96.95121951	138	8	166	2346	1	2196	0	3149

TRINITY_DN16132_c0_g1_i4	Glyma.14G098600.2	97.43589744	131	0	641	2840	1	2200	0	3338

TRINITY_DN15699_c0_g1_i8	Glyma.19G003200.1	98.03921569	105	0	167	2366	1	2200	0	3482

TRINITY_DN15699_c0_g1_i9	Glyma.19G003200.1	99.4011976	105	0	167	2366	1	2200	0	3482

TRINITY_DN6753_c0_g1_i1	Glyma.13G178200.4	97.05882353	189	16	1	2182	756	2928	0	2736

TRINITY_DN6753_c0_g1_i1	Glyma.13G178200.5	100	189	16	1	2182	756	2928	0	2736

TRINITY_DN6753_c0_g1_i1	Glyma.13G178200.6	98.41269841	189	16	1	2182	756	2928	0	2736

TRINITY_DN6753_c0_g1_i1	Glyma.13G178200.7	98.41269841	189	16	1	2182	756	2928	0	2736

TRINITY_DN6753_c0_g1_i1	Glyma.13G178200.8	100	189	16	1	2182	756	2928	0	2736

TRINITY_DN4160_c0_g2_i1	Glyma.20G189100.1	95.89041096	173	2	1	2196	237	2432	0	3064

TRINITY_DN4160_c0_g2_i1	Glyma.20G189100.4	95.50561798	173	2	1	2196	165	2360	0	3064

TRINITY_DN15403_c0_g2_i3	Glyma.20G212100.1	99.19354839	172	10	815	2998	1	2184	0	2915

TRINITY_DN16106_c0_g3_i2	Glyma.04G070200.1	95.34883721	162	23	533	2683	2604	409	0	2833

TRINITY_DN16106_c0_g3_i2	Glyma.04G070200.2	100	162	23	533	2683	2595	400	0	2833

TRINITY_DN15937_c0_g1_i1	Glyma.12G224600.3	100	153	5	239	2430	4	2199	0	3149

TRINITY_DN15937_c0_g1_i3	Glyma.12G224600.3	95.41284404	153	5	239	2430	4	2199	0	3149

TRINITY_DN15937_c0_g1_i2	Glyma.12G224600.3	100	152	5	239	2430	4	2199	0	3155

TRINITY_DN15937_c0_g1_i5	Glyma.12G224600.3	100	152	5	239	2430	4	2199	0	3155

TRINITY_DN16408_c2_g1_i2	Glyma.01G099600.3	96.20253165	151	9	213	2402	2172	1	0	2992

TRINITY_DN16408_c2_g1_i2	Glyma.03G069800.7	95.09202454	145	6	213	2402	2196	1	0	3181

TRINITY_DN5145_c0_g3_i1	Glyma.U029300.1	99.55555556	143	10	1	2183	284	2475	0	3120

TRINITY_DN15826_c0_g1_i3	Glyma.06G088500.1	97.04433498	142	7	162	2351	1	2199	0	3216

TRINITY_DN11893_c0_g1_i1	Glyma.10G075900.3	98	135	2	623	2818	82	2280	0	3293

TRINITY_DN16382_c0_g3_i1	Glyma.11G169700.1	100	133	0	177	2375	1	2199	0	3325

TRINITY_DN16382_c0_g3_i1	Glyma.11G169700.2	100	132	1	177	2375	1	2190	0	3271

TRINITY_DN16382_c0_g3_i1	Glyma.18G060600.1	100	126	0	177	2375	1	2199	0	3363

TRINITY_DN15621_c0_g1_i2	Glyma.16G057600.1	97.6744186	123	3	1	2196	18	2214	0	3347

TRINITY_DN15621_c0_g1_i3	Glyma.16G057600.1	100	123	3	1	2196	18	2214	0	3347

TRINITY_DN16071_c0_g2_i8	Glyma.15G158300.1	99.21875	117	3	127	2313	60	2258	0	3336

TRINITY_DN16314_c1_g1_i1	Glyma.10G223500.1	100	422	27	4	2183	3125	943	0	1507

TRINITY_DN16314_c1_g1_i1	Glyma.10G223500.3	100	422	27	4	2183	3119	937	0	1507

TRINITY_DN15888_c0_g3_i2	Glyma.14G048100.1	98.75	361	22	437	2628	718	2891	0	1866

TRINITY_DN15764_c0_g1_i6	Glyma.16G181000.1	98.7654321	298	15	478	2654	1	2189	0	2215

TRINITY_DN15699_c0_g1_i8	Glyma.19G056700.1	100	273	6	173	2366	1	2194	0	2495

TRINITY_DN15699_c0_g1_i9	Glyma.19G056700.1	99.0990991	273	6	173	2366	1	2194	0	2495

TRINITY_DN15736_c0_g1_i1	Glyma.06G162300.2	95.215311	263	14	573	2745	2	2195	0	2414

TRINITY_DN12815_c1_g1_i4	Glyma.08G318100.1	97	249	45	1009	3158	2309	148	0	2137

TRINITY_DN15736_c0_g1_i2	Glyma.06G162300.2	100	181	10	724	2897	1	2195	0	2881

TRINITY_DN14637_c0_g1_i1	Glyma.03G217000.2	99.28315412	160	13	1	2174	516	2707	0	2979

TRINITY_DN14637_c0_g1_i1	Glyma.03G217000.4	100	160	13	1	2174	516	2707	0	2979

TRINITY_DN5145_c0_g3_i1	Glyma.17G233200.1	96.34146341	159	9	1	2183	284	2472	0	3024

TRINITY_DN14654_c0_g1_i4	Glyma.16G111200.3	98.57142857	132	5	1787	3983	2355	165	0	3277

TRINITY_DN14654_c0_g1_i4	Glyma.16G111200.2	100	132	5	1787	3983	2355	165	0	3277

TRINITY_DN14654_c0_g1_i4	Glyma.16G111200.1	97.85714286	132	5	1787	3983	2355	165	0	3277

TRINITY_DN14654_c0_g1_i4	Glyma.09G194900.1	96.38554217	123	5	1787	3983	2352	165	0	3306

TRINITY_DN3215_c0_g1_i1	Glyma.02G017400.1	98.27586207	106	0	580	2777	2198	1	0	3472

TRINITY_DN12526_c0_g1_i1	Glyma.13G178200.1	96.78571429	391	36	1	2159	63	2242	0	1535

TRINITY_DN12526_c0_g1_i1	Glyma.13G178200.9	99.49494949	391	36	1	2159	63	2242	0	1535

TRINITY_DN15062_c0_g1_i1	Glyma.11G075100.4	100	330	20	926	3111	2174	1	0	2010

TRINITY_DN15062_c0_g1_i1	Glyma.11G075100.3	100	330	20	926	3111	2174	1	0	2010

TRINITY_DN15062_c0_g1_i1	Glyma.11G075100.2	97.00996678	330	20	926	3111	2174	1	0	2010

TRINITY_DN15062_c0_g1_i1	Glyma.11G075100.1	97.68786127	330	20	926	3111	2174	1	0	2010

TRINITY_DN14654_c0_g1_i4	Glyma.11G089400.2	95.08196721	320	14	1786	3979	2344	169	0	2130

TRINITY_DN14654_c0_g1_i4	Glyma.11G089400.1	96.93877551	320	14	1786	3979	2344	169	0	2130

TRINITY_DN14654_c0_g1_i4	Glyma.11G089400.3	95.37366548	320	14	1786	3979	2344	169	0	2130

TRINITY_DN14654_c0_g1_i4	Glyma.01G155300.1	95.08196721	315	13	1787	3981	2343	167	0	2170

TRINITY_DN12815_c1_g1_i3	Glyma.08G318100.2	96.98492462	313	47	1009	3158	2300	148	0	1735

TRINITY_DN15565_c0_g1_i1	Glyma.09G044300.1	96.48241206	157	14	449	2612	1	2185	0	2896

TRINITY_DN15187_c0_g1_i2	Glyma.15G271800.1	99.07120743	145	7	1801	3985	2191	1	0	3138

TRINITY_DN15187_c0_g1_i3	Glyma.15G271800.1	98.61111111	145	7	2663	4847	2191	1	0	3138

TRINITY_DN15187_c0_g1_i4	Glyma.15G271800.1	100	145	7	1576	3760	2191	1	0	3138

TRINITY_DN13758_c0_g1_i1	Glyma.14G219700.4	95.34883721	143	6	64	2242	1	2197	0	3149

TRINITY_DN13758_c0_g1_i2	Glyma.14G219700.1	100	143	6	64	2242	1	2197	0	3149

TRINITY_DN13758_c0_g1_i1	Glyma.14G219700.3	95.34883721	142	7	64	2242	1	2194	0	3134

TRINITY_DN13758_c0_g1_i1	Glyma.17G258700.1	100	137	6	64	2242	1	2197	0	3182

TRINITY_DN13758_c0_g1_i1	Glyma.17G258700.2	95.34883721	135	8	64	2242	1	2194	0	3173

TRINITY_DN38275_c0_g1_i1	Glyma.06G088900.1	98.5915493	127	15	1	2167	4196	2000	0	3160

TRINITY_DN38275_c0_g1_i1	Glyma.06G088900.2	98.5915493	127	15	1	2167	4196	2000	0	3160

TRINITY_DN3215_c0_g1_i1	Glyma.10G017800.1	98.30097087	100	1	584	2777	2197	1	0	3483

TRINITY_DN3954_c0_g2_i1	Glyma.19G249100.1	96.46017699	373	17	449	2633	16	2196	0	1821

TRINITY_DN3954_c0_g1_i1	Glyma.19G249100.1	100	372	18	449	2632	16	2196	0	1820

TRINITY_DN15850_c2_g3_i2	Glyma.03G245500.1	99.18032787	289	31	230	2384	2199	26	0	2047

TRINITY_DN15329_c0_g2_i5	Glyma.02G085000.2	95.58823529	236	14	377	2566	2170	1	0	2542

TRINITY_DN15329_c0_g2_i5	Glyma.02G085000.5	100	236	14	377	2566	2170	1	0	2542

TRINITY_DN16436_c0_g2_i11	Glyma.11G131600.2	96.95740365	197	8	562	2742	1	2190	0	2828

TRINITY_DN40017_c0_g1_i1	Glyma.10G183700.3	100	170	3	153	2348	1	2193	0	3094

TRINITY_DN40017_c0_g1_i1	Glyma.10G183700.2	100	170	3	153	2348	1	2193	0	3094

TRINITY_DN14680_c0_g1_i1	Glyma.10G046400.1	99.41860465	166	11	1	2187	484	2676	0	3059

TRINITY_DN14680_c0_g1_i1	Glyma.10G046400.2	98.39572193	166	11	1	2187	484	2676	0	3059

TRINITY_DN12719_c0_g2_i6	Glyma.12G179300.2	100	162	14	192	2376	2167	1	0	2900

TRINITY_DN12719_c0_g2_i8	Glyma.12G179300.2	95.83333333	162	14	192	2376	2167	1	0	2900

TRINITY_DN14622_c0_g1_i2	Glyma.20G210700.3	95.53571429	146	6	274	2460	86	2272	0	3131

TRINITY_DN7839_c0_g1_i1	Glyma.08G025800.1	100	137	0	140	2335	1	2196	0	3297

TRINITY_DN7839_c0_g1_i1	Glyma.05G219500.1	100	132	0	140	2335	1	2196	0	3325

TRINITY_DN15455_c0_g1_i3	Glyma.02G204300.1	97.43589744	110	3	157	2352	1	2187	0	3387

TRINITY_DN15455_c0_g1_i3	Glyma.17G073500.3	97.01492537	103	4	157	2352	1	2187	0	3426

TRINITY_DN15455_c0_g1_i3	Glyma.17G073500.2	99.74358974	103	4	157	2352	1	2187	0	3426

TRINITY_DN15455_c0_g1_i3	Glyma.17G073500.1	97.43589744	103	4	157	2352	1	2187	0	3426

TRINITY_DN12815_c1_g1_i2	Glyma.08G318100.1	96.98189135	295	48	1009	3155	2309	148	0	1895

TRINITY_DN12815_c1_g1_i4	Glyma.08G318100.2	96.97885196	249	43	1009	3158	2300	148	0	2111

TRINITY_DN10549_c0_g1_i2	Glyma.08G363700.1	98.21428571	201	16	421	2598	2178	1	0	2721

TRINITY_DN36949_c0_g1_i1	Glyma.04G121100.3	96.2962963	185	4	180	2359	197	2391	0	2931

TRINITY_DN36949_c0_g1_i1	Glyma.04G121100.2	96.2962963	185	4	180	2359	197	2391	0	2931

TRINITY_DN36949_c0_g1_i1	Glyma.06G317700.5	98.56230032	178	6	180	2359	164	2358	0	2970

TRINITY_DN36949_c0_g1_i1	Glyma.06G317700.6	97.12837838	178	6	180	2359	164	2358	0	2970

TRINITY_DN36949_c0_g1_i1	Glyma.06G317700.2	97.12837838	178	6	180	2359	164	2358	0	2970

TRINITY_DN13547_c0_g1_i1	Glyma.07G045600.2	98.04560261	150	5	356	2532	1	2192	0	3086

TRINITY_DN13547_c0_g1_i2	Glyma.07G045600.2	98.05825243	150	5	356	2532	1	2192	0	3086

TRINITY_DN19016_c0_g1_i1	Glyma.02G309200.1	100	136	0	1	2195	2987	793	0	3301

TRINITY_DN19016_c0_g1_i1	Glyma.02G309200.3	100	136	0	1	2195	2954	760	0	3301

TRINITY_DN19016_c0_g1_i1	Glyma.02G309200.5	100	136	0	1	2195	2711	517	0	3301

TRINITY_DN19016_c0_g1_i1	Glyma.02G309200.8	100	136	1	1	2195	2984	793	0	3280

TRINITY_DN19016_c0_g1_i1	Glyma.02G309200.2	100	136	1	1	2195	2951	760	0	3280

TRINITY_DN19016_c0_g1_i1	Glyma.02G309200.4	100	136	1	1	2195	2708	517	0	3280

TRINITY_DN12526_c0_g1_i1	Glyma.13G178200.4	100	392	34	1	2159	63	2239	0	1544

TRINITY_DN12526_c0_g1_i1	Glyma.13G178200.5	96.47058824	392	34	1	2159	63	2239	0	1544

TRINITY_DN12526_c0_g1_i1	Glyma.13G178200.6	96.47058824	392	34	1	2159	63	2239	0	1544

TRINITY_DN12526_c0_g1_i1	Glyma.13G178200.7	96.78571429	392	34	1	2159	63	2239	0	1544

TRINITY_DN12526_c0_g1_i1	Glyma.13G178200.8	96.78571429	392	34	1	2159	63	2239	0	1544

TRINITY_DN12886_c0_g1_i1	Glyma.04G194900.1	96.1038961	264	11	476	2656	3705	1519	0	2460

TRINITY_DN12886_c0_g1_i1	Glyma.04G194900.3	96.09756098	264	11	476	2656	3606	1420	0	2460

TRINITY_DN12886_c0_g1_i1	Glyma.04G194900.2	96.07843137	260	15	476	2656	3600	1420	0	2444

TRINITY_DN15565_c0_g1_i1	Glyma.15G148800.1	100	156	9	449	2612	1	2179	0	2896

TRINITY_DN14680_c0_g1_i1	Glyma.13G134500.1	97.50260146	154	9	1	2187	484	2673	0	3127

TRINITY_DN11366_c0_g1_i1	Glyma.07G195900.1	96.96969697	135	5	99	2282	49	2241	0	3232

TRINITY_DN15415_c0_g1_i3	Glyma.08G282800.1	99.43820225	125	2	70	2263	111	2301	0	3339

TRINITY_DN15415_c0_g1_i3	Glyma.18G143600.1	99.43820225	123	2	70	2263	105	2295	0	3350

TRINITY_DN8887_c0_g1_i1	Glyma.15G160300.1	96.2406015	358	33	79	2247	1	2166	0	1736

TRINITY_DN8887_c0_g1_i2	Glyma.15G160300.1	100	358	33	253	2421	1	2166	0	1736

TRINITY_DN12815_c1_g1_i1	Glyma.08G318100.1	96.996997	231	44	1009	3155	2309	148	0	2272

TRINITY_DN16436_c0_g2_i3	Glyma.11G131600.2	96.48281375	202	7	915	3092	1	2190	0	2815

TRINITY_DN15516_c0_g1_i1	Glyma.12G198600.1	100	410	28	468	2630	219	2399	0	1519

TRINITY_DN12815_c1_g1_i2	Glyma.08G318100.2	97.00598802	295	46	1009	3155	2300	148	0	1871

TRINITY_DN16345_c0_g1_i8	Glyma.09G182600.1	98.30508475	211	10	1553	3739	2694	511	0	2795

TRINITY_DN13538_c0_g1_i2	Glyma.02G013700.1	97.54601227	207	40	249	2428	1	2115	0	2326

TRINITY_DN16314_c1_g1_i1	Glyma.08G145600.1	100	197	7	1	2183	3122	934	0	2880

TRINITY_DN16314_c1_g1_i1	Glyma.05G187300.1	100	197	7	1	2183	3122	934	0	2880

TRINITY_DN15691_c0_g1_i2	Glyma.10G028700.2	96.95121951	138	8	166	2338	1	2188	0	3134

TRINITY_DN19016_c0_g1_i1	Glyma.14G003600.1	100	137	0	4	2195	3002	811	0	3290

TRINITY_DN19016_c0_g1_i1	Glyma.14G003600.2	100	137	1	4	2195	2999	811	0	3269

TRINITY_DN37264_c0_g1_i1	Glyma.17G244300.2	100	129	2	673	2863	3276	1086	0	3321

TRINITY_DN16056_c0_g2_i1	Glyma.02G301800.1	97.6945245	370	11	511	2691	2247	64	0	1886

TRINITY_DN12886_c0_g1_i1	Glyma.06G171000.1	97	281	10	476	2656	3717	1534	0	2379

TRINITY_DN12886_c0_g1_i1	Glyma.06G171000.2	97	281	10	476	2656	3618	1435	0	2379

TRINITY_DN16345_c0_g1_i8	Glyma.07G093700.2	98.31223629	218	4	1553	3739	2706	517	0	2806

TRINITY_DN16345_c0_g1_i8	Glyma.07G093700.1	97.26027397	218	4	1553	3739	2706	517	0	2806

TRINITY_DN18139_c0_g1_i1	Glyma.16G213300.1	100	132	4	273	2454	2202	12	0	3256

TRINITY_DN37264_c0_g1_i1	Glyma.14G081100.7	95.08196721	123	0	673	2863	3300	1110	0	3365

TRINITY_DN15329_c0_g2_i2	Glyma.02G085000.3	95.01246883	232	20	377	2548	2182	1	0	2591

TRINITY_DN15329_c0_g2_i2	Glyma.02G085000.1	95.49019608	232	20	377	2548	2182	1	0	2591

TRINITY_DN12815_c1_g1_i1	Glyma.08G318100.2	96.9924812	231	42	1009	3155	2300	148	0	2248

TRINITY_DN10900_c0_g1_i1	Glyma.18G286300.1	99.26470588	219	58	891	3005	2160	1	0	2152

TRINITY_DN15084_c0_g3_i1	Glyma.08G113600.1	99.38650307	205	19	245	2416	2187	7	0	2734

TRINITY_DN6753_c0_g1_i1	Glyma.07G198200.2	99.78540773	191	9	1	2178	756	2936	0	2850

TRINITY_DN6753_c0_g1_i1	Glyma.07G198200.3	96.31578947	191	9	1	2178	756	2936	0	2850

TRINITY_DN6753_c0_g1_i1	Glyma.07G198200.4	98.11320755	191	9	1	2178	756	2936	0	2850

TRINITY_DN6753_c0_g1_i1	Glyma.07G198200.5	98.06362379	191	9	1	2178	756	2936	0	2850

TRINITY_DN6753_c0_g1_i1	Glyma.07G198200.1	98.06451613	191	9	1	2178	756	2936	0	2850

TRINITY_DN18139_c0_g1_i1	Glyma.09G165000.1	97.60191847	145	0	273	2462	2190	1	0	3241

TRINITY_DN12789_c0_g1_i1	Glyma.19G037200.2	96.08247423	94	0	2599	4788	4008	1819	0	3524

TRINITY_DN16431_c0_g1_i4	Glyma.03G037400.1	100	377	33	2684	4822	2608	433	0	1546

TRINITY_DN16431_c0_g1_i5	Glyma.03G037400.1	95.45454545	377	33	2664	4802	2608	433	0	1546

TRINITY_DN16431_c0_g1_i6	Glyma.03G037400.1	97.93814433	377	33	2327	4465	2608	433	0	1546

TRINITY_DN16431_c0_g1_i7	Glyma.03G037400.1	97.59036145	377	33	2307	4445	2608	433	0	1546

TRINITY_DN11999_c1_g1_i1	Glyma.17G164100.1	95.21276596	357	8	218	2401	100	2283	0	2001

TRINITY_DN11999_c1_g1_i2	Glyma.17G164100.1	99.543379	357	8	365	2548	100	2283	0	2001

TRINITY_DN5189_c0_g1_i1	Glyma.20G022900.2	100	329	23	386	2547	310	2486	0	1967

TRINITY_DN15385_c0_g1_i3	Glyma.18G248200.2	98.7654321	290	33	1627	3765	2358	184	0	2023

TRINITY_DN15385_c0_g1_i3	Glyma.18G248200.1	98.7654321	290	33	1627	3765	2358	184	0	2023

TRINITY_DN3062_c0_g1_i1	Glyma.04G146900.1	98.70967742	253	28	152	2304	2187	23	0	2254

TRINITY_DN12692_c0_g1_i1	Glyma.08G117500.1	100	234	8	363	2548	2970	788	0	2687

TRINITY_DN12692_c0_g1_i1	Glyma.08G117500.2	100	234	8	363	2548	2967	785	0	2687

TRINITY_DN13828_c0_g2_i1	Glyma.13G214300.3	95.68965517	226	10	246	2426	1	2178	0	2667

TRINITY_DN15310_c0_g1_i2	Glyma.15G274800.1	97.46376812	198	18	316	2492	1	2168	0	2732

TRINITY_DN16345_c0_g1_i8	Glyma.08G044700.1	98.32214765	189	5	1553	3739	2676	496	0	2931

TRINITY_DN16345_c0_g1_i8	Glyma.08G044700.6	98.30508475	189	5	1553	3739	2397	217	0	2931

TRINITY_DN15599_c0_g3_i3	Glyma.08G232100.1	96	172	9	259	2437	2187	1	0	3013

TRINITY_DN13444_c0_g1_i2	Glyma.20G160900.1	100	167	14	427	2581	2217	30	0	2891

TRINITY_DN11366_c0_g1_i1	Glyma.13G180100.1	96.96969697	138	5	99	2277	40	2227	0	3206

TRINITY_DN16056_c0_g1_i1	Glyma.03G139100.1	100	388	16	511	2691	2337	163	0	1762

TRINITY_DN16056_c0_g1_i2	Glyma.03G139100.1	96.22641509	388	16	511	2691	2337	163	0	1762

TRINITY_DN15084_c0_g3_i1	Glyma.05G157600.1	100	286	24	245	2417	2178	6	0	2263

TRINITY_DN9859_c0_g1_i1	Glyma.13G366600.1	98.16849817	235	18	40	2205	7	2184	0	2532

TRINITY_DN15944_c2_g2_i3	Glyma.04G253000.1	100	223	7	275	2456	2352	171	0	2728

TRINITY_DN15991_c0_g1_i5	Glyma.19G234800.1	100	198	23	656	2791	2174	4	0	2497

TRINITY_DN16345_c0_g1_i8	Glyma.05G237500.1	98.31775701	186	2	1553	3739	2682	496	0	2998

TRINITY_DN38723_c0_g1_i1	Glyma.20G243000.5	99.21875	181	4	107	2279	1	2188	0	2940

TRINITY_DN38723_c0_g1_i1	Glyma.20G243000.4	99.21875	181	4	107	2279	1	2188	0	2940

TRINITY_DN38723_c0_g1_i1	Glyma.20G243000.3	97.33009709	181	4	107	2279	1	2188	0	2940

TRINITY_DN38723_c0_g1_i1	Glyma.20G243000.2	97.72727273	181	4	107	2279	1	2188	0	2940

TRINITY_DN38723_c0_g1_i1	Glyma.20G243000.1	99.13793103	181	4	107	2279	1	2188	0	2940

TRINITY_DN38723_c0_g1_i1	Glyma.20G243000.6	99.13793103	181	4	107	2279	1	2188	0	2940

TRINITY_DN15466_c0_g1_i1	Glyma.06G162200.1	96.2962963	170	10	324	2499	149	2327	0	2963

TRINITY_DN20106_c0_g1_i1	Glyma.02G077200.3	98.4962406	167	2	318	2504	1	2187	0	3103

TRINITY_DN20106_c0_g1_i1	Glyma.02G077200.1	98.4962406	167	2	318	2504	1	2187	0	3103

TRINITY_DN20106_c0_g1_i1	Glyma.02G077200.2	98.48484848	167	2	318	2504	1	2187	0	3103

TRINITY_DN20106_c0_g2_i1	Glyma.02G077200.3	98.50746269	167	2	318	2504	1	2187	0	3103

TRINITY_DN20106_c0_g2_i1	Glyma.02G077200.1	98.48484848	167	2	318	2504	1	2187	0	3103

TRINITY_DN20106_c0_g2_i1	Glyma.02G077200.2	98.4962406	167	2	318	2504	1	2187	0	3103

TRINITY_DN20106_c0_g1_i1	Glyma.16G160000.1	98.50746269	163	2	318	2504	1	2187	0	3125

TRINITY_DN20106_c0_g1_i1	Glyma.16G160000.2	98.48484848	163	2	318	2504	1	2187	0	3125

TRINITY_DN20106_c0_g2_i1	Glyma.16G160000.1	98.50746269	163	2	318	2504	1	2187	0	3125

TRINITY_DN20106_c0_g2_i1	Glyma.16G160000.2	98.49246231	163	2	318	2504	1	2187	0	3125

TRINITY_DN10873_c0_g1_i2	Glyma.04G017700.1	96.47058824	122	4	263	2448	2183	1	0	3319

TRINITY_DN11875_c0_g2_i1	Glyma.11G251900.1	95.30201342	408	44	1017	3158	550	2691	0	1197

TRINITY_DN16056_c0_g2_i1	Glyma.14G012400.1	98.57142857	361	6	511	2691	2244	61	0	1980

TRINITY_DN16132_c0_g1_i3	Glyma.17G226100.1	95.38461538	230	4	641	2826	1	2183	0	2732

TRINITY_DN16132_c0_g1_i3	Glyma.14G098600.2	100	226	5	641	2826	1	2183	0	2754

TRINITY_DN16132_c0_g1_i3	Glyma.14G098600.1	96.68615984	226	5	641	2826	1	2183	0	2754

TRINITY_DN5148_c0_g1_i1	Glyma.05G158800.1	96.55172414	218	23	450	2603	1	2163	0	2462

TRINITY_DN5148_c0_g1_i1	Glyma.05G158800.3	100	218	23	450	2603	1	2163	0	2462

TRINITY_DN8360_c0_g2_i1	Glyma.08G324300.1	99.09090909	213	1	248	2434	1	2184	0	2839

TRINITY_DN15991_c0_g1_i5	Glyma.03G237100.1	95.94594595	209	37	656	2791	2159	4	0	2351

TRINITY_DN14670_c0_g1_i2	Glyma.13G065500.15	99.54086318	201	9	1178	3360	9	2182	0	2815

TRINITY_DN12719_c0_g2_i2	Glyma.13G321300.51	100	162	20	458	2633	2137	1	0	2747

TRINITY_DN12719_c0_g2_i2	Glyma.13G321300.48	96.875	162	20	458	2633	2137	1	0	2747

TRINITY_DN12719_c0_g2_i2	Glyma.13G321300.18	98.0806142	162	20	458	2633	2137	1	0	2747

TRINITY_DN12719_c0_g2_i2	Glyma.13G321300.19	99.18032787	162	20	458	2633	2137	1	0	2747

TRINITY_DN12719_c0_g2_i2	Glyma.13G321300.52	99.18032787	162	20	458	2633	2137	1	0	2747

TRINITY_DN12719_c0_g2_i2	Glyma.13G321300.1	95.89041096	162	20	458	2633	2137	1	0	2747

TRINITY_DN12719_c0_g2_i2	Glyma.13G321300.20	97.36842105	162	20	458	2633	2137	1	0	2747

TRINITY_DN12719_c0_g2_i2	Glyma.13G321300.21	95.34883721	162	20	458	2633	2137	1	0	2747

TRINITY_DN12719_c0_g2_i4	Glyma.13G321300.51	100	162	20	458	2633	2137	1	0	2747

TRINITY_DN12719_c0_g2_i4	Glyma.13G321300.48	97.00598802	162	20	458	2633	2137	1	0	2747

TRINITY_DN12719_c0_g2_i4	Glyma.13G321300.18	98.96907216	162	20	458	2633	2137	1	0	2747

TRINITY_DN12719_c0_g2_i4	Glyma.13G321300.19	100	162	20	458	2633	2137	1	0	2747

TRINITY_DN12719_c0_g2_i4	Glyma.13G321300.52	96.26168224	162	20	458	2633	2137	1	0	2747

TRINITY_DN12719_c0_g2_i4	Glyma.13G321300.1	98.61111111	162	20	458	2633	2137	1	0	2747

TRINITY_DN12719_c0_g2_i4	Glyma.13G321300.20	97.82608696	162	20	458	2633	2137	1	0	2747

TRINITY_DN12719_c0_g2_i4	Glyma.13G321300.21	100	162	20	458	2633	2137	1	0	2747

TRINITY_DN10477_c0_g2_i3	Glyma.08G111100.1	99.57081545	161	18	165	2333	118	2292	0	2953

TRINITY_DN8503_c0_g1_i5	Glyma.16G213100.1	96.20253165	159	2	318	2501	2439	253	0	3138

TRINITY_DN8503_c0_g1_i6	Glyma.16G213100.1	99.19093851	159	2	329	2512	2439	253	0	3138

TRINITY_DN668_c0_g1_i1	Glyma.02G257600.1	100	158	9	156	2312	1	2184	0	2950

TRINITY_DN13758_c0_g1_i2	Glyma.14G219700.2	100	152	10	331	2493	1	2184	0	3022

TRINITY_DN5129_c0_g1_i1	Glyma.17G112700.1	95.13888889	148	14	304	2484	2166	1	0	3044

TRINITY_DN8503_c0_g1_i5	Glyma.09G164700.1	100	141	2	318	2501	2439	253	0	3238

TRINITY_DN8503_c0_g1_i6	Glyma.09G164700.1	99.1902834	141	2	329	2512	2439	253	0	3238

TRINITY_DN14432_c0_g1_i4	Glyma.17G233000.3	97.47155499	124	6	1037	3202	1	2187	0	3216

TRINITY_DN14432_c0_g1_i5	Glyma.17G233000.3	97.48427673	124	6	960	3125	1	2187	0	3216

TRINITY_DN14516_c0_g1_i1	Glyma.17G094800.2	98.30508475	92	4	174	2339	1	2187	0	3393

TRINITY_DN14981_c0_g2_i4	Glyma.05G036100.2	98.03921569	519	68	603	2747	2379	241	5.70E-168	593

TRINITY_DN14981_c0_g2_i4	Glyma.05G036100.1	98.03921569	519	68	603	2747	2379	241	5.70E-168	593

TRINITY_DN14981_c0_g2_i2	Glyma.05G036100.2	98.01980198	519	68	603	2747	2379	241	7.18E-168	593

TRINITY_DN14981_c0_g2_i2	Glyma.05G036100.1	98.16513761	519	68	603	2747	2379	241	7.18E-168	593

TRINITY_DN15499_c0_g1_i3	Glyma.13G174900.1	98.80952381	265	3	6	2187	451	2635	0	2536

TRINITY_DN16056_c0_g1_i1	Glyma.02G301800.1	100	258	0	511	2696	2247	62	0	2608

TRINITY_DN4972_c0_g1_i1	Glyma.10G270300.2	100	159	2	85	2270	2184	2	0	3136

TRINITY_DN15036_c0_g1_i4	Glyma.08G111000.2	96.96969697	125	2	319	2501	2228	43	0	3325

TRINITY_DN15036_c0_g1_i4	Glyma.08G111000.1	100	125	2	319	2501	2228	43	0	3325

TRINITY_DN12692_c0_g1_i1	Glyma.04G063800.1	100	122	0	363	2548	2931	746	0	3362

TRINITY_DN12692_c0_g1_i1	Glyma.06G065000.1	100	110	0	363	2548	2928	743	0	3428

TRINITY_DN16314_c1_g1_i1	Glyma.02G080900.1	100	414	18	13	2183	3059	883	0	1600

TRINITY_DN15510_c0_g1_i1	Glyma.09G168600.2	100	259	27	424	2585	8	2172	0	2322

TRINITY_DN15510_c0_g1_i4	Glyma.09G168600.2	97.59036145	259	27	450	2611	8	2172	0	2322

TRINITY_DN9428_c0_g1_i1	Glyma.16G067700.1	95.40636042	202	2	94	2277	1	2184	0	2904

TRINITY_DN12892_c0_g4_i5	Glyma.12G028100.1	100	139	3	889	3067	564	2748	0	3227

TRINITY_DN5641_c0_g2_i1	Glyma.05G193600.1	98.39572193	132	13	133	2305	1	2176	0	3168

TRINITY_DN16056_c0_g1_i1	Glyma.14G012400.1	95.6043956	240	0	511	2694	2244	61	0	2704

TRINITY_DN15876_c0_g1_i1	Glyma.06G040600.1	96.52777778	186	7	398	2573	228	2409	0	2939

TRINITY_DN15876_c0_g1_i1	Glyma.06G040600.2	96.52777778	186	7	398	2573	228	2409	0	2939

TRINITY_DN15601_c0_g2_i1	Glyma.17G107300.1	96.95121951	178	3	652	2832	1	2178	0	2988

TRINITY_DN16130_c2_g6_i12	Glyma.02G201300.3	97.84172662	172	0	791	2974	1	2184	0	3081

TRINITY_DN16130_c2_g6_i2	Glyma.02G201300.3	96.04863222	172	0	464	2647	1	2184	0	3081

TRINITY_DN16130_c2_g6_i6	Glyma.02G201300.3	98.41427156	172	0	537	2720	1	2184	0	3081

TRINITY_DN16130_c2_g6_i9	Glyma.02G201300.3	96.42857143	172	0	864	3047	1	2184	0	3081

TRINITY_DN16130_c2_g6_i8	Glyma.02G201300.3	98.33333333	164	0	884	3067	1	2184	0	3125

TRINITY_DN17450_c0_g1_i1	Glyma.16G125100.1	97.57281553	153	4	316	2499	3662	1494	0	3088

TRINITY_DN17450_c0_g1_i1	Glyma.02G046600.1	99.43820225	151	0	316	2499	3677	1494	0	3197

TRINITY_DN14243_c0_g1_i1	Glyma.19G077600.1	96.20253165	145	3	244	2421	1	2178	0	3153

TRINITY_DN14243_c0_g1_i1	Glyma.05G073400.1	96.19047619	143	5	244	2421	1	2178	0	3164

TRINITY_DN5129_c0_g1_i1	Glyma.13G158600.1	95.13888889	135	12	304	2484	2163	1	0	3131

TRINITY_DN14128_c0_g1_i1	Glyma.05G022500.1	100	134	0	543	2726	1	2184	0	3291

TRINITY_DN14128_c0_g1_i1	Glyma.05G022500.2	99.66555184	134	0	543	2726	1	2184	0	3291

TRINITY_DN14128_c0_g1_i1	Glyma.17G077100.3	99.12280702	133	0	543	2726	1	2184	0	3297

TRINITY_DN14128_c0_g1_i1	Glyma.17G077100.1	98.52459016	133	0	543	2726	1	2184	0	3297

TRINITY_DN14128_c0_g1_i1	Glyma.17G077100.2	98.9010989	133	0	543	2726	1	2184	0	3297

TRINITY_DN16130_c2_g6_i1	Glyma.02G201300.3	100	114	0	464	2647	1	2184	0	3402

TRINITY_DN16130_c2_g6_i11	Glyma.02G201300.3	97.22222222	114	0	791	2974	1	2184	0	3402

TRINITY_DN16130_c2_g6_i16	Glyma.02G201300.3	98.74715262	114	0	864	3047	1	2184	0	3402

TRINITY_DN16130_c2_g6_i7	Glyma.02G201300.3	98.18181818	114	0	537	2720	1	2184	0	3402

TRINITY_DN16130_c2_g6_i17	Glyma.02G201300.3	97.11538462	106	0	884	3067	1	2184	0	3446

TRINITY_DN11001_c0_g1_i2	Glyma.01G147800.1	99.10714286	84	2	388	2565	1	2184	0	3530

TRINITY_DN16314_c1_g1_i1	Glyma.16G165900.1	100	409	15	13	2183	3059	883	0	1650

TRINITY_DN16314_c1_g1_i1	Glyma.16G165900.2	100	409	15	13	2183	2516	340	0	1650

TRINITY_DN13448_c0_g1_i1	Glyma.11G246200.4	100	306	0	128	2310	1	2183	0	2337

TRINITY_DN13448_c0_g1_i1	Glyma.11G246200.5	96.15384615	306	0	128	2310	1	2183	0	2337

TRINITY_DN14670_c0_g1_i3	Glyma.13G065500.16	98.80952381	222	15	1212	3390	1	2143	0	2518

TRINITY_DN13746_c0_g2_i1	Glyma.20G233200.1	100	205	21	465	2619	2664	507	0	2553

TRINITY_DN13378_c0_g2_i1	Glyma.13G241100.1	100	200	3	1	2180	224	2403	0	2885

TRINITY_DN13378_c0_g2_i3	Glyma.13G241100.1	100	165	3	1	2180	224	2403	0	3079

TRINITY_DN15981_c0_g1_i11	Glyma.11G048300.2	100	146	3	1761	3937	2183	1	0	3184

TRINITY_DN15981_c0_g1_i11	Glyma.11G048300.1	97.05882353	146	3	1761	3937	2183	1	0	3184

TRINITY_DN15981_c0_g1_i16	Glyma.11G048300.2	100	146	3	1543	3719	2183	1	0	3184

TRINITY_DN15981_c0_g1_i16	Glyma.11G048300.1	97.07602339	146	3	1543	3719	2183	1	0	3184

TRINITY_DN15981_c0_g1_i17	Glyma.11G048300.2	100	146	3	1527	3703	2183	1	0	3184

TRINITY_DN15981_c0_g1_i17	Glyma.11G048300.1	95.45454545	146	3	1527	3703	2183	1	0	3184

TRINITY_DN15981_c0_g1_i21	Glyma.11G048300.2	96.59090909	146	3	1417	3593	2183	1	0	3184

TRINITY_DN15981_c0_g1_i21	Glyma.11G048300.1	99.47229551	146	3	1417	3593	2183	1	0	3184

TRINITY_DN15981_c0_g1_i22	Glyma.11G048300.2	97.08029197	146	3	1635	3811	2183	1	0	3184

TRINITY_DN15981_c0_g1_i22	Glyma.11G048300.1	100	146	3	1635	3811	2183	1	0	3184

TRINITY_DN15981_c0_g1_i3	Glyma.11G048300.2	97.6	146	3	1525	3701	2183	1	0	3184

TRINITY_DN15981_c0_g1_i3	Glyma.11G048300.1	99.23076923	146	3	1525	3701	2183	1	0	3184

TRINITY_DN12886_c0_g1_i1	Glyma.17G129200.1	96.09375	116	2	476	2658	3675	1496	0	3369

TRINITY_DN12886_c0_g1_i1	Glyma.05G047400.1	96.1038961	112	2	476	2658	3675	1496	0	3391

TRINITY_DN12886_c0_g1_i1	Glyma.05G047400.2	96.1038961	112	2	476	2658	3591	1412	0	3391

TRINITY_DN16431_c0_g1_i4	Glyma.03G039200.1	97.2972973	376	33	2681	4822	2601	433	0	1604

TRINITY_DN16431_c0_g1_i5	Glyma.03G039200.1	98.57142857	376	33	2661	4802	2601	433	0	1604

TRINITY_DN16431_c0_g1_i6	Glyma.03G039200.1	97.93814433	376	33	2324	4465	2601	433	0	1604

TRINITY_DN16431_c0_g1_i7	Glyma.03G039200.1	96.02409639	376	33	2304	4445	2601	433	0	1604

TRINITY_DN11771_c0_g2_i1	Glyma.08G035200.1	98.7654321	224	19	4	2166	1	2172	0	2601

TRINITY_DN16436_c0_g2_i1	Glyma.11G131600.2	99.53051643	198	6	562	2729	1	2177	0	2809

TRINITY_DN16436_c0_g2_i10	Glyma.11G131600.2	100	191	6	562	2729	1	2177	0	2848

TRINITY_DN14486_c0_g1_i1	Glyma.09G230800.1	98.6013986	188	11	2617	4795	2303	155	0	2756

TRINITY_DN7825_c0_g1_i1	Glyma.12G063100.1	95.7278481	188	30	295	2415	2163	1	0	2471

TRINITY_DN4427_c0_g1_i1	Glyma.20G125600.1	97.5	136	11	325	2505	2148	1	0	3049

TRINITY_DN16345_c0_g1_i9	Glyma.07G093700.2	99.21259843	112	1	641	2822	3527	1349	0	3389

TRINITY_DN13619_c0_g1_i1	Glyma.20G099500.1	100	313	18	1	2162	772	2942	0	2106

TRINITY_DN13619_c0_g1_i2	Glyma.20G099500.1	95.1171875	313	18	1	2162	772	2942	0	2106

TRINITY_DN13619_c0_g1_i4	Glyma.20G099500.1	99.69040248	313	18	1	2162	772	2942	0	2106

TRINITY_DN13619_c0_g1_i5	Glyma.20G099500.1	97.6	313	18	1	2162	772	2942	0	2106

TRINITY_DN15385_c0_g1_i1	Glyma.09G245000.1	95.83333333	299	37	1571	3718	2337	184	0	1984

TRINITY_DN15385_c0_g1_i1	Glyma.09G245000.2	100	299	37	1571	3718	2337	184	0	1984

TRINITY_DN15944_c2_g2_i6	Glyma.13G083300.1	99.07407407	274	7	196	2369	2364	188	0	2438

TRINITY_DN15944_c2_g2_i6	Glyma.13G083300.4	100	274	8	196	2369	2361	188	0	2420

TRINITY_DN15815_c0_g2_i6	Glyma.12G219400.3	100	270	11	472	2633	3243	1067	0	2383

TRINITY_DN15815_c0_g2_i8	Glyma.12G219400.3	96.12903226	270	11	472	2633	3243	1067	0	2383

TRINITY_DN16268_c1_g14_i1	Glyma.08G242100.2	100	226	0	265	2445	2181	1	0	2776

TRINITY_DN15601_c0_g2_i1	Glyma.13G163600.1	96.95121951	181	1	652	2832	1	2175	0	2987

TRINITY_DN14118_c0_g2_i1	Glyma.14G218000.2	95.92875318	168	15	485	2625	3063	902	0	2715

TRINITY_DN12719_c0_g2_i5	Glyma.12G179300.2	99.2	162	13	192	2361	2167	1	0	2968

TRINITY_DN12719_c0_g2_i7	Glyma.12G179300.2	98.93048128	162	13	192	2361	2167	1	0	2968

TRINITY_DN16341_c0_g1_i2	Glyma.20G223300.3	100	160	1	839	3018	2181	1	0	3134

TRINITY_DN16268_c1_g9_i1	Glyma.08G242100.2	95.51820728	126	0	265	2445	2181	1	0	3330

TRINITY_DN4427_c0_g1_i1	Glyma.10G264900.1	97.5	125	1	325	2505	2178	1	0	3315

TRINITY_DN16899_c0_g1_i1	Glyma.03G127300.1	96.77419355	113	3	168	2342	1	2181	0	3363

TRINITY_DN15944_c0_g1_i1	Glyma.18G012500.1	99.75	93	8	1	2175	295	2472	0	3454

TRINITY_DN15944_c0_g1_i1	Glyma.11G244600.1	99.74937343	91	7	1	2175	298	2475	0	3465

TRINITY_DN11001_c0_g1_i2	Glyma.01G147800.3	95.23809524	84	1	388	2565	1	2181	0	3542

TRINITY_DN11001_c0_g1_i2	Glyma.01G147800.4	97.70114943	84	1	388	2565	1	2181	0	3542

TRINITY_DN11001_c0_g1_i2	Glyma.09G212100.1	99.10714286	84	1	388	2565	1	2181	0	3542

TRINITY_DN15421_c1_g3_i2	Glyma.08G120800.1	95.45454545	468	31	914	3073	2412	253	0	1175

TRINITY_DN11875_c0_g1_i1	Glyma.05G148300.1	95.3125	392	42	829	2970	751	2905	0	1447

TRINITY_DN15981_c0_g1_i11	Glyma.17G161300.1	100	363	8	1761	3934	2177	1	0	1956

TRINITY_DN15981_c0_g1_i16	Glyma.17G161300.1	97.38372093	363	8	1543	3716	2177	1	0	1956

TRINITY_DN15981_c0_g1_i17	Glyma.17G161300.1	100	363	8	1527	3700	2177	1	0	1956

TRINITY_DN15981_c0_g1_i21	Glyma.17G161300.1	96.66666667	363	8	1417	3590	2177	1	0	1956

TRINITY_DN15981_c0_g1_i22	Glyma.17G161300.1	98.91304348	363	8	1635	3808	2177	1	0	1956

TRINITY_DN15981_c0_g1_i3	Glyma.17G161300.1	98.11133201	363	8	1525	3698	2177	1	0	1956

TRINITY_DN15016_c0_g1_i2	Glyma.09G137000.2	100	359	31	1141	3290	10	2163	0	1676

TRINITY_DN15981_c0_g1_i11	Glyma.05G105200.1	100	358	7	1761	3934	2177	1	0	1984

TRINITY_DN15981_c0_g1_i16	Glyma.05G105200.1	99.10714286	358	7	1543	3716	2177	1	0	1984

TRINITY_DN15981_c0_g1_i17	Glyma.05G105200.1	95.45454545	358	7	1527	3700	2177	1	0	1984

TRINITY_DN15981_c0_g1_i21	Glyma.05G105200.1	98.4375	358	7	1417	3590	2177	1	0	1984

TRINITY_DN15981_c0_g1_i22	Glyma.05G105200.1	95.3125	358	7	1635	3808	2177	1	0	1984

TRINITY_DN15981_c0_g1_i3	Glyma.05G105200.1	97.32142857	358	7	1525	3698	2177	1	0	1984

TRINITY_DN16143_c0_g1_i1	Glyma.16G030000.1	97.81420765	321	38	1331	3445	1081	3213	0	1524

TRINITY_DN15944_c2_g2_i2	Glyma.13G083300.1	96.05263158	314	10	287	2457	2361	188	0	2189

TRINITY_DN15944_c2_g2_i2	Glyma.13G083300.4	98.7804878	314	11	287	2457	2358	188	0	2170

TRINITY_DN15499_c0_g1_i3	Glyma.07G201600.1	99.52606635	260	5	13	2187	455	2632	0	2540

TRINITY_DN16016_c0_g1_i6	Glyma.16G163200.1	95	251	3	1	2180	2421	245	0	2615

TRINITY_DN16353_c0_g1_i3	Glyma.19G068800.1	96.34146341	234	11	714	2874	695	2870	0	2580

TRINITY_DN14437_c0_g2_i1	Glyma.18G233100.2	96.16306954	196	3	1	2179	7	2176	0	2868

TRINITY_DN14216_c0_g1_i1	Glyma.19G159400.3	100	164	1	104	2277	78	2257	0	3079

TRINITY_DN16408_c2_g1_i2	Glyma.01G099600.2	97.14285714	142	7	238	2402	2153	1	0	2968

TRINITY_DN15434_c1_g2_i1	Glyma.07G184200.1	100	342	19	133	2302	2233	70	0	1975

TRINITY_DN16436_c0_g2_i7	Glyma.11G131600.2	95.74468085	203	5	915	3079	1	2177	0	2796

TRINITY_DN14486_c0_g1_i1	Glyma.12G005900.1	98.6013986	163	10	2617	4795	2303	158	0	2907

TRINITY_DN14622_c0_g1_i1	Glyma.20G210700.1	98.75776398	149	18	102	2270	1	2157	0	2992

TRINITY_DN14622_c0_g1_i1	Glyma.20G210700.2	96.15384615	149	18	102	2270	1	2157	0	2992

TRINITY_DN5641_c0_g2_i1	Glyma.08G001700.1	98.39572193	136	17	133	2307	1	2160	0	3121

TRINITY_DN15421_c1_g3_i2	Glyma.05G163600.1	99.43820225	440	36	914	3073	2418	268	0	1293

TRINITY_DN8367_c0_g1_i3	Glyma.11G174500.1	95.47325103	417	47	136	2272	2562	417	0	1240

TRINITY_DN15815_c0_g2_i6	Glyma.12G219400.2	100	270	10	472	2633	3240	1067	0	2398

TRINITY_DN15815_c0_g2_i8	Glyma.12G219400.2	95.625	270	10	472	2633	3240	1067	0	2398

TRINITY_DN15329_c0_g2_i2	Glyma.02G085000.2	98.7012987	236	11	377	2548	2170	1	0	2625

TRINITY_DN15329_c0_g2_i2	Glyma.02G085000.5	98.7012987	236	11	377	2548	2170	1	0	2625

TRINITY_DN15434_c1_g2_i1	Glyma.15G059700.1	99.6031746	207	2	125	2302	2214	43	0	2837

TRINITY_DN14622_c0_g1_i1	Glyma.10G179700.5	95.13184584	171	16	102	2270	1	2157	0	2881

TRINITY_DN14622_c0_g1_i1	Glyma.10G179700.4	97.64705882	171	16	102	2270	1	2157	0	2881

TRINITY_DN16341_c0_g1_i1	Glyma.20G223300.3	96.7806841	143	2	1174	3345	2175	1	0	3171

TRINITY_DN16341_c0_g1_i1	Glyma.20G223300.2	100	143	2	1174	3345	2175	1	0	3171

TRINITY_DN15306_c0_g1_i1	Glyma.20G114300.3	99.47368421	136	13	418	2580	2970	817	0	3016

TRINITY_DN15306_c0_g1_i1	Glyma.20G114300.1	100	136	13	418	2580	2970	817	0	3016

TRINITY_DN15306_c0_g1_i2	Glyma.20G114300.3	99.6124031	136	13	2231	4393	2970	817	0	3016

TRINITY_DN15306_c0_g1_i2	Glyma.20G114300.1	100	136	13	2231	4393	2970	817	0	3016

TRINITY_DN15306_c0_g1_i1	Glyma.20G114300.5	100	136	13	418	2580	2280	127	0	3016

TRINITY_DN15306_c0_g1_i2	Glyma.20G114300.5	95	136	13	2231	4393	2280	127	0	3016

TRINITY_DN15306_c0_g1_i1	Glyma.20G114300.2	100	136	14	418	2580	2967	817	0	2998

TRINITY_DN15306_c0_g1_i2	Glyma.20G114300.2	97.05882353	136	14	2231	4393	2967	817	0	2998

TRINITY_DN15306_c0_g1_i1	Glyma.20G114300.4	100	136	14	418	2580	2277	127	0	2998

TRINITY_DN15306_c0_g1_i2	Glyma.20G114300.4	99.56616052	136	14	2231	4393	2277	127	0	2998

TRINITY_DN12011_c0_g2_i2	Glyma.13G010100.2	100	127	0	179	2356	1	2178	0	3319

TRINITY_DN12011_c0_g2_i2	Glyma.13G010100.1	100	127	0	179	2356	1	2178	0	3319

TRINITY_DN12011_c0_g2_i4	Glyma.13G010100.2	100	127	0	110	2287	1	2178	0	3319

TRINITY_DN12011_c0_g2_i4	Glyma.13G010100.1	100	127	0	110	2287	1	2178	0	3319

TRINITY_DN12011_c0_g2_i2	Glyma.20G060100.3	100	118	0	179	2356	1	2178	0	3369

TRINITY_DN12011_c0_g2_i2	Glyma.20G060100.2	100	118	0	179	2356	1	2178	0	3369

TRINITY_DN12011_c0_g2_i2	Glyma.20G060100.1	100	118	0	179	2356	1	2178	0	3369

TRINITY_DN12011_c0_g2_i4	Glyma.20G060100.3	100	118	0	110	2287	1	2178	0	3369

TRINITY_DN12011_c0_g2_i4	Glyma.20G060100.2	100	118	0	110	2287	1	2178	0	3369

TRINITY_DN12011_c0_g2_i4	Glyma.20G060100.1	100	118	0	110	2287	1	2178	0	3369

TRINITY_DN14526_c0_g1_i1	Glyma.11G016000.1	98.63013699	117	2	291	2462	2253	76	0	3336

TRINITY_DN11001_c0_g1_i2	Glyma.01G147800.2	100	84	0	388	2565	1	2178	0	3557

TRINITY_DN11001_c0_g1_i2	Glyma.09G212100.2	100	84	0	388	2565	1	2178	0	3557

TRINITY_DN14750_c0_g1_i1	Glyma.04G131700.2	95.16616314	284	9	762	2934	2206	40	0	2357

TRINITY_DN15944_c2_g2_i6	Glyma.14G164600.4	98.63013699	262	2	196	2369	2346	173	0	2531

TRINITY_DN15944_c2_g2_i6	Glyma.14G164600.3	97.66081871	262	3	196	2369	2343	173	0	2510

TRINITY_DN13474_c0_g2_i3	Glyma.16G010400.2	100	190	20	97	2252	97	2243	0	2638

TRINITY_DN13474_c0_g2_i3	Glyma.16G010400.1	98.29059829	190	20	97	2252	97	2243	0	2638

TRINITY_DN11672_c0_g2_i1	Glyma.01G180100.1	97.5	160	1	1	2171	2303	4479	0	3096

TRINITY_DN11672_c0_g2_i1	Glyma.01G180100.2	98.55072464	160	1	1	2171	2303	4479	0	3096

TRINITY_DN11672_c0_g2_i1	Glyma.01G180100.3	98.9010989	160	1	1	2171	2303	4479	0	3096

TRINITY_DN11672_c0_g2_i1	Glyma.01G180100.4	95.47038328	160	1	1	2171	1346	3522	0	3096

TRINITY_DN15981_c0_g1_i11	Glyma.01G193600.2	96.42857143	149	1	1761	3934	2177	1	0	3175

TRINITY_DN15981_c0_g1_i11	Glyma.01G193600.1	96.77419355	149	1	1761	3934	2177	1	0	3175

TRINITY_DN15981_c0_g1_i16	Glyma.01G193600.2	95	149	1	1543	3716	2177	1	0	3175

TRINITY_DN15981_c0_g1_i16	Glyma.01G193600.1	96.15384615	149	1	1543	3716	2177	1	0	3175

TRINITY_DN15981_c0_g1_i17	Glyma.01G193600.2	100	149	1	1527	3700	2177	1	0	3175

TRINITY_DN15981_c0_g1_i17	Glyma.01G193600.1	98.92473118	149	1	1527	3700	2177	1	0	3175

TRINITY_DN15981_c0_g1_i21	Glyma.01G193600.2	97.47706422	149	1	1417	3590	2177	1	0	3175

TRINITY_DN15981_c0_g1_i21	Glyma.01G193600.1	100	149	1	1417	3590	2177	1	0	3175

TRINITY_DN15981_c0_g1_i22	Glyma.01G193600.2	98.8372093	149	1	1635	3808	2177	1	0	3175

TRINITY_DN15981_c0_g1_i22	Glyma.01G193600.1	97.56097561	149	1	1635	3808	2177	1	0	3175

TRINITY_DN15981_c0_g1_i3	Glyma.01G193600.2	99.2	149	1	1525	3698	2177	1	0	3175

TRINITY_DN15981_c0_g1_i3	Glyma.01G193600.1	97.87234043	149	1	1525	3698	2177	1	0	3175

TRINITY_DN15466_c0_g1_i2	Glyma.06G162200.3	99.55156951	134	1	849	3025	45	2220	0	3271

TRINITY_DN15619_c0_g2_i3	Glyma.06G181900.1	98.10606061	310	14	912	3069	1	2172	0	2159

TRINITY_DN15007_c0_g1_i4	Glyma.11G242000.1	100	285	23	246	2404	2153	1	0	2182

TRINITY_DN15007_c0_g1_i5	Glyma.11G242000.1	100	285	23	246	2404	2153	1	0	2182

TRINITY_DN15944_c2_g2_i5	Glyma.04G253000.1	96.66666667	273	7	199	2368	2340	171	0	2429

TRINITY_DN16005_c1_g2_i2	Glyma.08G289100.2	100	273	17	693	2838	63	2232	0	2274

TRINITY_DN14750_c0_g1_i2	Glyma.04G131700.2	95.20295203	191	4	762	2937	2206	40	0	2902

TRINITY_DN11672_c0_g2_i1	Glyma.11G062100.3	98.57142857	146	3	3	2171	2164	4338	0	3158

TRINITY_DN11672_c0_g2_i1	Glyma.11G062100.1	100	146	3	3	2171	2164	4338	0	3158

TRINITY_DN11672_c0_g2_i1	Glyma.11G062100.6	100	146	3	3	2171	2062	4236	0	3158

TRINITY_DN11672_c0_g2_i1	Glyma.11G062100.4	98.10606061	146	3	3	2171	2062	4236	0	3158

TRINITY_DN11672_c0_g2_i1	Glyma.11G062100.5	95.45454545	146	3	3	2171	2059	4233	0	3158

TRINITY_DN11672_c0_g2_i1	Glyma.11G062100.2	100	146	3	3	2171	2059	4233	0	3158

TRINITY_DN16069_c0_g1_i2	Glyma.04G213100.1	98.8372093	140	9	435	2587	418	2591	0	3081

TRINITY_DN12145_c0_g1_i1	Glyma.16G044400.3	99.38650307	128	1	741	2916	2695	523	0	3290

TRINITY_DN15964_c0_g1_i8	Glyma.07G113500.1	98.8372093	486	10	250	2420	25	2192	0	1253

TRINITY_DN11672_c0_g2_i1	Glyma.16G143100.2	98.56850716	367	13	2	2171	2328	4491	0	1881

TRINITY_DN11672_c0_g2_i1	Glyma.16G143100.6	95.46165884	367	13	2	2171	2328	4491	0	1881

TRINITY_DN11672_c0_g2_i1	Glyma.16G143100.3	95.85987261	367	13	2	2171	2328	4491	0	1881

TRINITY_DN11672_c0_g2_i1	Glyma.16G143100.7	98.39142091	367	13	2	2171	2328	4491	0	1881

TRINITY_DN11672_c0_g2_i1	Glyma.16G143100.4	96.82539683	367	13	2	2171	2328	4491	0	1881

TRINITY_DN11672_c0_g2_i1	Glyma.16G143100.5	97.88359788	367	13	2	2171	2328	4491	0	1881

TRINITY_DN11672_c0_g2_i1	Glyma.16G143100.1	97.69585253	367	13	2	2171	2328	4491	0	1881

TRINITY_DN15137_c0_g1_i12	Glyma.14G222700.1	100	340	9	556	2721	2289	118	0	2056

TRINITY_DN15137_c0_g1_i2	Glyma.14G222700.1	97.14285714	323	9	528	2693	2289	118	0	2150

TRINITY_DN15944_c2_g2_i2	Glyma.13G083300.3	100	313	10	292	2457	2356	188	0	2185

TRINITY_DN15944_c2_g2_i2	Glyma.13G083300.2	98.7804878	313	11	292	2457	2353	188	0	2167

TRINITY_DN15944_c2_g2_i2	Glyma.14G164600.4	96.05263158	305	4	287	2457	2343	173	0	2276

TRINITY_DN15944_c2_g2_i2	Glyma.14G164600.3	96.05263158	305	5	287	2457	2340	173	0	2255

TRINITY_DN15944_c2_g2_i6	Glyma.13G083300.3	100	274	7	202	2369	2358	188	0	2427

TRINITY_DN15944_c2_g2_i6	Glyma.13G083300.2	100	274	8	202	2369	2355	188	0	2409

TRINITY_DN15853_c0_g1_i3	Glyma.02G010500.1	100	267	4	363	2537	2169	1	0	2499

TRINITY_DN13474_c0_g2_i3	Glyma.07G042100.1	98.7804878	183	15	97	2252	97	2255	0	2776

TRINITY_DN7825_c0_g1_i1	Glyma.11G139500.1	95.74468085	182	36	295	2415	2163	1	0	2582

TRINITY_DN9058_c0_g1_i1	Glyma.09G191700.1	95.57522124	173	0	164	2338	1	2175	0	3059

TRINITY_DN16341_c0_g1_i2	Glyma.20G223300.2	100	160	0	844	3018	2175	1	0	3131

TRINITY_DN13902_c0_g1_i3	Glyma.11G219700.1	96.84813754	160	3	103	2271	1	2175	0	3092

TRINITY_DN9058_c0_g1_i1	Glyma.07G085200.1	95.58652729	153	0	164	2338	1	2175	0	3169

TRINITY_DN3145_c0_g1_i1	Glyma.20G150500.1	99.44903581	144	3	237	2408	1	2172	0	3181

TRINITY_DN3145_c0_g1_i1	Glyma.10G243900.1	98.97959184	138	4	237	2408	1	2169	0	3193

TRINITY_DN15510_c0_g1_i2	Glyma.09G168600.2	96.36048527	132	2	132	2303	1	2172	0	3247

TRINITY_DN15853_c0_g1_i3	Glyma.19G218200.1	97.05882353	125	2	363	2537	2166	1	0	3265

TRINITY_DN15853_c0_g1_i3	Glyma.03G221200.1	99.60159363	119	4	363	2537	2163	1	0	3280

TRINITY_DN10010_c0_g1_i1	Glyma.05G000200.4	96.96969697	118	8	401	2563	2169	1	0	3247

TRINITY_DN16899_c0_g1_i1	Glyma.19G130000.1	96.12903226	116	0	168	2342	1	2175	0	3374

TRINITY_DN13349_c0_g1_i1	Glyma.13G323500.1	97.95918367	103	1	306	2477	1	2175	0	3426

TRINITY_DN13349_c0_g1_i1	Glyma.12G177200.3	96.12403101	97	1	306	2477	1	2175	0	3459

TRINITY_DN13349_c0_g1_i1	Glyma.12G177200.1	96.12403101	97	1	306	2477	1	2175	0	3459

TRINITY_DN13349_c0_g1_i1	Glyma.12G177200.2	95.69892473	97	1	306	2477	1	2175	0	3459

TRINITY_DN15964_c0_g1_i7	Glyma.07G113500.1	95.23809524	493	8	250	2420	25	2192	0	1225

TRINITY_DN8905_c0_g1_i1	Glyma.11G079200.1	100	471	38	122	2274	18	2164	0	1096

TRINITY_DN15416_c1_g1_i2	Glyma.06G095600.1	100	199	10	378	2548	2581	420	0	2815

TRINITY_DN15416_c1_g1_i2	Glyma.06G095600.2	100	197	12	378	2548	2578	420	0	2808

TRINITY_DN16254_c0_g1_i5	Glyma.07G004300.3	100	193	3	1	2174	2171	1	0	2926

TRINITY_DN15619_c0_g2_i3	Glyma.06G181900.2	99.12280702	310	12	912	3069	1	2169	0	2172

TRINITY_DN16005_c1_g2_i3	Glyma.08G289100.2	97.5	239	14	693	2835	63	2232	0	2475

TRINITY_DN15681_c0_g2_i1	Glyma.17G033700.2	96.75516224	210	22	287	2443	1	2148	0	2584

TRINITY_DN15007_c0_g1_i3	Glyma.11G242000.1	97.61904762	199	17	246	2404	2153	1	0	2691

TRINITY_DN15007_c0_g1_i9	Glyma.11G242000.1	97.97979798	199	17	246	2404	2153	1	0	2691

TRINITY_DN15876_c0_g1_i1	Glyma.04G039400.1	96.2962963	182	6	399	2564	229	2400	0	2953

TRINITY_DN12306_c0_g1_i2	Glyma.13G099600.2	96.51162791	181	1	137	2306	26	2198	0	2990

TRINITY_DN12306_c0_g1_i2	Glyma.13G099600.1	97.19626168	181	1	137	2306	26	2198	0	2990

TRINITY_DN5999_c0_g1_i1	Glyma.05G175300.1	100	163	0	402	2574	2898	726	0	3110

TRINITY_DN5999_c0_g1_i1	Glyma.05G175300.2	100	163	0	402	2574	2847	675	0	3110

TRINITY_DN5999_c0_g1_i1	Glyma.05G175300.3	100	163	0	402	2574	2592	420	0	3110

TRINITY_DN5999_c0_g1_i1	Glyma.05G175300.4	100	163	0	402	2574	2592	420	0	3110

TRINITY_DN4427_c0_g2_i1	Glyma.20G125600.1	97.5	135	11	408	2579	2139	1	0	3038

TRINITY_DN15655_c1_g3_i1	Glyma.02G281700.1	98.30508475	107	3	1	2170	273	2442	0	3382

TRINITY_DN15655_c1_g3_i1	Glyma.02G281700.2	95.27559055	107	3	1	2170	273	2442	0	3382

TRINITY_DN15655_c1_g3_i1	Glyma.02G281700.3	99.5412844	107	3	1	2170	21	2190	0	3382

TRINITY_DN13902_c0_g1_i1	Glyma.02G248700.2	100	183	1	103	2274	1	2169	0	2977

TRINITY_DN13902_c0_g1_i1	Glyma.02G248700.1	100	183	1	103	2274	1	2169	0	2977

TRINITY_DN6660_c0_g2_i2	Glyma.20G099900.1	97.24770642	178	14	191	2347	1	2169	0	2909

TRINITY_DN6293_c0_g1_i1	Glyma.05G220000.1	95.20153551	166	3	463	2625	1	2163	0	2976

TRINITY_DN6293_c0_g1_i1	Glyma.08G026300.1	95.2	162	3	463	2625	1	2163	0	2998

TRINITY_DN16106_c0_g3_i2	Glyma.04G070200.3	95.43378995	156	9	533	2683	2568	400	0	2992

TRINITY_DN13902_c0_g1_i3	Glyma.18G037700.1	96.84684685	155	1	103	2271	1	2172	0	3133

TRINITY_DN14695_c0_g1_i1	Glyma.04G065700.1	100	133	0	357	2528	1	2172	0	3275

TRINITY_DN14432_c0_g1_i4	Glyma.U029000.6	97.47416762	130	2	1037	3202	1	2166	0	3214

TRINITY_DN14432_c0_g1_i5	Glyma.U029000.6	97.4789916	130	2	960	3125	1	2166	0	3214

TRINITY_DN11190_c0_g1_i1	Glyma.03G222300.1	98.29059829	124	0	344	2515	2172	1	0	3325

TRINITY_DN4427_c0_g2_i1	Glyma.10G264900.1	97.5	123	1	408	2579	2169	1	0	3310

TRINITY_DN11190_c0_g1_i1	Glyma.19G219500.1	96.55172414	121	0	344	2515	2172	1	0	3341

TRINITY_DN15655_c1_g3_i1	Glyma.14G032700.2	100	99	3	2	2170	274	2442	0	3424

TRINITY_DN15655_c1_g3_i1	Glyma.14G032700.1	100	99	3	2	2170	274	2442	0	3424

TRINITY_DN8367_c0_g1_i3	Glyma.18G062300.1	98.82629108	387	61	136	2272	3270	1158	0	1271

TRINITY_DN14216_c0_g1_i2	Glyma.19G159400.3	100	313	4	268	2437	91	2257	0	2242

TRINITY_DN15529_c1_g1_i2	Glyma.05G232400.2	100	294	10	20	2179	2166	1	0	2278

TRINITY_DN15529_c1_g1_i2	Glyma.05G232400.1	100	294	10	20	2179	2166	1	0	2278

TRINITY_DN15529_c1_g1_i2	Glyma.05G232400.4	98.80952381	294	10	20	2179	2166	1	0	2278

TRINITY_DN15529_c1_g1_i4	Glyma.05G232400.2	98.95287958	294	10	20	2179	2166	1	0	2278

TRINITY_DN15529_c1_g1_i4	Glyma.05G232400.1	98.95287958	294	10	20	2179	2166	1	0	2278

TRINITY_DN15529_c1_g1_i4	Glyma.05G232400.4	99.04534606	294	10	20	2179	2166	1	0	2278

TRINITY_DN3172_c0_g1_i1	Glyma.14G008100.1	99.04153355	277	34	235	2371	948	3090	0	2074

TRINITY_DN10833_c0_g1_i1	Glyma.09G248000.2	96.47058824	235	7	882	3043	2195	31	0	2610

TRINITY_DN10833_c0_g1_i1	Glyma.09G248000.1	100	235	7	882	3043	2195	31	0	2610

TRINITY_DN13378_c0_g2_i3	Glyma.13G181200.1	100	200	0	3	2173	307	2477	0	2902

TRINITY_DN10000_c0_g1_i1	Glyma.09G228800.2	100	188	7	168	2335	3901	1749	0	2832

TRINITY_DN10000_c0_g1_i1	Glyma.09G228800.1	100	188	7	168	2335	3901	1749	0	2832

TRINITY_DN10000_c0_g1_i2	Glyma.09G228800.2	96.06557377	188	7	168	2335	3901	1749	0	2832

TRINITY_DN10000_c0_g1_i2	Glyma.09G228800.1	95.14563107	188	7	168	2335	3901	1749	0	2832

TRINITY_DN10000_c0_g1_i3	Glyma.09G228800.2	100	188	7	438	2605	3901	1749	0	2832

TRINITY_DN10000_c0_g1_i3	Glyma.09G228800.1	100	188	7	438	2605	3901	1749	0	2832

TRINITY_DN10000_c0_g1_i4	Glyma.09G228800.2	99.11504425	188	7	438	2605	3901	1749	0	2832

TRINITY_DN10000_c0_g1_i4	Glyma.09G228800.1	98.66666667	188	7	438	2605	3901	1749	0	2832

TRINITY_DN6753_c0_g1_i1	Glyma.13G178200.3	95	188	18	32	2182	664	2808	0	2671

TRINITY_DN13378_c0_g2_i1	Glyma.13G181200.1	100	164	0	3	2173	307	2477	0	3101

TRINITY_DN15306_c0_g1_i1	Glyma.10G275400.1	100	157	12	425	2580	2966	814	0	2926

TRINITY_DN15306_c0_g1_i2	Glyma.10G275400.1	100	157	12	2238	4393	2966	814	0	2926

TRINITY_DN16254_c0_g1_i10	Glyma.07G004300.3	95.74468085	134	0	1	2171	2171	1	0	3267

TRINITY_DN12815_c1_g1_i3	Glyma.18G095400.3	96.98795181	334	40	1009	3153	2297	168	0	1736

TRINITY_DN12815_c1_g1_i3	Glyma.18G095400.2	97.00499168	334	40	1009	3153	2297	168	0	1736

TRINITY_DN15944_c2_g2_i2	Glyma.14G164600.1	96.05263158	304	4	292	2457	2338	173	0	2272

TRINITY_DN15944_c2_g2_i2	Glyma.14G164600.2	96.05263158	304	5	292	2457	2335	173	0	2252

TRINITY_DN15385_c0_g1_i3	Glyma.09G245000.1	98.80095923	278	29	1627	3765	2337	184	0	2163

TRINITY_DN15385_c0_g1_i3	Glyma.09G245000.2	98.80136986	278	29	1627	3765	2337	184	0	2163

TRINITY_DN15529_c1_g1_i1	Glyma.07G089000.1	95.34883721	236	8	568	2733	2160	1	0	2610

TRINITY_DN15529_c1_g1_i1	Glyma.07G089000.3	100	236	8	568	2733	2160	1	0	2610

TRINITY_DN15529_c1_g1_i1	Glyma.07G089000.4	100	236	8	568	2733	2160	1	0	2610

TRINITY_DN15529_c1_g1_i1	Glyma.07G089000.2	99.20634921	236	8	568	2733	2160	1	0	2610

TRINITY_DN11610_c0_g1_i1	Glyma.10G189200.1	100	222	2	1	2169	887	3055	0	2765

TRINITY_DN11610_c0_g1_i1	Glyma.20G201500.1	100	220	2	1	2169	869	3037	0	2776

TRINITY_DN10000_c0_g1_i1	Glyma.12G007800.3	98.00995025	194	6	168	2334	4042	1888	0	2817

TRINITY_DN10000_c0_g1_i1	Glyma.12G007800.2	97.60479042	194	6	168	2334	4042	1888	0	2817

TRINITY_DN10000_c0_g1_i1	Glyma.12G007800.1	96.06299213	194	6	168	2334	4042	1888	0	2817

TRINITY_DN10000_c0_g1_i1	Glyma.12G007800.6	96.06440072	194	6	168	2334	4042	1888	0	2817

TRINITY_DN10000_c0_g1_i2	Glyma.12G007800.3	98.84393064	194	6	168	2334	4042	1888	0	2817

TRINITY_DN10000_c0_g1_i2	Glyma.12G007800.2	96.66666667	194	6	168	2334	4042	1888	0	2817

TRINITY_DN10000_c0_g1_i2	Glyma.12G007800.1	99.60474308	194	6	168	2334	4042	1888	0	2817

TRINITY_DN10000_c0_g1_i2	Glyma.12G007800.6	100	194	6	168	2334	4042	1888	0	2817

TRINITY_DN10000_c0_g1_i3	Glyma.12G007800.3	95.02222222	194	6	438	2604	4042	1888	0	2817

TRINITY_DN10000_c0_g1_i3	Glyma.12G007800.2	96.86098655	194	6	438	2604	4042	1888	0	2817

TRINITY_DN10000_c0_g1_i3	Glyma.12G007800.1	96.34146341	194	6	438	2604	4042	1888	0	2817

TRINITY_DN10000_c0_g1_i3	Glyma.12G007800.6	100	194	6	438	2604	4042	1888	0	2817

TRINITY_DN10000_c0_g1_i4	Glyma.12G007800.3	100	194	6	438	2604	4042	1888	0	2817

TRINITY_DN10000_c0_g1_i4	Glyma.12G007800.2	98.57142857	194	6	438	2604	4042	1888	0	2817

TRINITY_DN10000_c0_g1_i4	Glyma.12G007800.1	97.39130435	194	6	438	2604	4042	1888	0	2817

TRINITY_DN10000_c0_g1_i4	Glyma.12G007800.6	96.84210526	194	6	438	2604	4042	1888	0	2817

TRINITY_DN10000_c0_g1_i1	Glyma.12G007800.5	95.23809524	194	6	168	2334	3898	1744	0	2817

TRINITY_DN10000_c0_g1_i1	Glyma.12G007800.4	98.68421053	194	6	168	2334	3898	1744	0	2817

TRINITY_DN10000_c0_g1_i2	Glyma.12G007800.5	95.83333333	194	6	168	2334	3898	1744	0	2817

TRINITY_DN10000_c0_g1_i2	Glyma.12G007800.4	97.8021978	194	6	168	2334	3898	1744	0	2817

TRINITY_DN10000_c0_g1_i3	Glyma.12G007800.5	97.6344086	194	6	438	2604	3898	1744	0	2817

TRINITY_DN10000_c0_g1_i3	Glyma.12G007800.4	100	194	6	438	2604	3898	1744	0	2817

TRINITY_DN10000_c0_g1_i4	Glyma.12G007800.5	95.6043956	194	6	438	2604	3898	1744	0	2817

TRINITY_DN10000_c0_g1_i4	Glyma.12G007800.4	98.68421053	194	6	438	2604	3898	1744	0	2817

TRINITY_DN16135_c0_g1_i3	Glyma.01G051400.1	99.16666667	146	14	2323	4471	2149	1	0	2928

TRINITY_DN16041_c0_g1_i1	Glyma.15G030400.1	96.9924812	143	12	380	2533	2904	742	0	3066

TRINITY_DN16041_c0_g1_i2	Glyma.15G030400.1	100	143	12	380	2533	2904	742	0	3066

TRINITY_DN16041_c0_g1_i4	Glyma.15G030400.1	96.55172414	143	12	153	2306	2904	742	0	3066

TRINITY_DN16041_c0_g1_i6	Glyma.15G030400.1	95.81395349	143	12	153	2306	2904	742	0	3066

TRINITY_DN16023_c0_g1_i3	Glyma.14G193300.1	95.41547278	443	25	2250	4402	2410	258	0	1345

TRINITY_DN14756_c0_g1_i3	Glyma.09G160200.1	96.13095238	385	46	473	2625	2391	275	0	1434

TRINITY_DN13902_c0_g1_i3	Glyma.02G248700.2	100	298	0	103	2271	1	2169	0	2355

TRINITY_DN13902_c0_g1_i3	Glyma.02G248700.1	100	298	0	103	2271	1	2169	0	2355

TRINITY_DN12815_c1_g1_i4	Glyma.18G095400.3	98.36065574	272	38	1009	3153	2297	168	0	2091

TRINITY_DN12815_c1_g1_i4	Glyma.18G095400.2	98.38709677	272	38	1009	3153	2297	168	0	2091

TRINITY_DN15944_c2_g2_i6	Glyma.14G164600.1	100	262	2	204	2369	2338	173	0	2516

TRINITY_DN15944_c2_g2_i6	Glyma.14G164600.2	97.66081871	262	3	204	2369	2335	173	0	2495

TRINITY_DN15137_c0_g1_i8	Glyma.14G222700.2	100	247	8	668	2828	2235	69	0	2573

TRINITY_DN15750_c1_g1_i7	Glyma.09G062900.1	100	218	1	547	2715	2166	1	0	2778

TRINITY_DN15750_c1_g1_i7	Glyma.09G062900.2	100	218	1	547	2715	2166	1	0	2778

TRINITY_DN15750_c1_g1_i7	Glyma.15G169500.2	100	212	1	547	2715	2166	1	0	2811

TRINITY_DN15750_c1_g1_i7	Glyma.15G169500.1	100	212	1	547	2715	2166	1	0	2811

TRINITY_DN15750_c1_g1_i6	Glyma.09G062900.1	96.90444145	200	1	547	2715	2166	1	0	2878

TRINITY_DN15750_c1_g1_i6	Glyma.09G062900.2	97.95918367	200	1	547	2715	2166	1	0	2878

TRINITY_DN15750_c1_g1_i6	Glyma.15G169500.2	100	191	1	547	2715	2166	1	0	2928

TRINITY_DN15750_c1_g1_i6	Glyma.15G169500.1	100	191	1	547	2715	2166	1	0	2928

TRINITY_DN15529_c1_g1_i1	Glyma.05G232400.2	100	188	3	568	2733	2166	1	0	2926

TRINITY_DN15529_c1_g1_i1	Glyma.05G232400.1	100	188	3	568	2733	2166	1	0	2926

TRINITY_DN15529_c1_g1_i1	Glyma.05G232400.4	97.68637532	188	3	568	2733	2166	1	0	2926

TRINITY_DN9767_c0_g1_i1	Glyma.03G172200.4	98.66666667	173	0	996	3164	2169	1	0	3048

TRINITY_DN14118_c0_g2_i4	Glyma.14G218000.2	100	168	14	485	2613	3063	902	0	2771

TRINITY_DN9398_c0_g3_i1	Glyma.15G129300.1	95.96774194	154	7	259	2411	1	2165	0	3024

TRINITY_DN14292_c0_g1_i1	Glyma.09G155200.2	95.60853199	154	22	316	2458	2140	1	0	2796

TRINITY_DN14292_c0_g1_i1	Glyma.09G155200.1	96.77419355	154	22	316	2458	2140	1	0	2796

TRINITY_DN15910_c0_g1_i5	Glyma.19G217300.1	96.90265487	130	6	248	2407	1	2166	0	3208

TRINITY_DN6753_c0_g1_i1	Glyma.13G178200.2	100	188	16	32	2182	664	2805	0	2684

TRINITY_DN12739_c0_g1_i2	Glyma.13G220900.1	100	186	11	308	2457	1	2159	0	2798

TRINITY_DN16041_c0_g1_i1	Glyma.13G343900.4	99.58158996	136	13	380	2531	2898	744	0	3062

TRINITY_DN16041_c0_g1_i1	Glyma.13G343900.3	100	136	13	380	2531	2898	744	0	3062

TRINITY_DN16041_c0_g1_i1	Glyma.13G343900.2	97.33333333	136	13	380	2531	2898	744	0	3062

TRINITY_DN16041_c0_g1_i1	Glyma.13G343900.1	99.0990991	136	13	380	2531	2898	744	0	3062

TRINITY_DN16041_c0_g1_i2	Glyma.13G343900.4	96.42857143	136	13	380	2531	2898	744	0	3062

TRINITY_DN16041_c0_g1_i2	Glyma.13G343900.3	99.42528736	136	13	380	2531	2898	744	0	3062

TRINITY_DN16041_c0_g1_i2	Glyma.13G343900.2	95.16129032	136	13	380	2531	2898	744	0	3062

TRINITY_DN16041_c0_g1_i2	Glyma.13G343900.1	95.71984436	136	13	380	2531	2898	744	0	3062

TRINITY_DN16041_c0_g1_i4	Glyma.13G343900.4	100	136	13	153	2304	2898	744	0	3062

TRINITY_DN16041_c0_g1_i4	Glyma.13G343900.3	100	136	13	153	2304	2898	744	0	3062

TRINITY_DN16041_c0_g1_i4	Glyma.13G343900.2	95.71865443	136	13	153	2304	2898	744	0	3062

TRINITY_DN16041_c0_g1_i4	Glyma.13G343900.1	99.18032787	136	13	153	2304	2898	744	0	3062

TRINITY_DN16041_c0_g1_i6	Glyma.13G343900.4	100	136	13	153	2304	2898	744	0	3062

TRINITY_DN16041_c0_g1_i6	Glyma.13G343900.3	100	136	13	153	2304	2898	744	0	3062

TRINITY_DN16041_c0_g1_i6	Glyma.13G343900.2	100	136	13	153	2304	2898	744	0	3062

TRINITY_DN16041_c0_g1_i6	Glyma.13G343900.1	100	136	13	153	2304	2898	744	0	3062

TRINITY_DN16314_c1_g1_i2	Glyma.16G165900.1	100	405	33	1912	4062	2576	435	0	1493

TRINITY_DN14337_c0_g2_i1	Glyma.15G208700.1	100	352	18	126	2274	2199	48	0	1838

TRINITY_DN12815_c1_g1_i2	Glyma.18G095400.3	96.99769053	313	41	1009	3150	2297	168	0	1868

TRINITY_DN12815_c1_g1_i2	Glyma.18G095400.2	96.9924812	313	41	1009	3150	2297	168	0	1868

TRINITY_DN13619_c0_g1_i1	Glyma.10G289700.2	97.4522293	310	15	2	2162	788	2942	0	2169

TRINITY_DN13619_c0_g1_i1	Glyma.10G289700.1	96.57320872	310	15	2	2162	788	2942	0	2169

TRINITY_DN13619_c0_g1_i2	Glyma.10G289700.2	99.3902439	310	15	2	2162	788	2942	0	2169

TRINITY_DN13619_c0_g1_i2	Glyma.10G289700.1	100	310	15	2	2162	788	2942	0	2169

TRINITY_DN13619_c0_g1_i4	Glyma.10G289700.2	100	310	15	2	2162	788	2942	0	2169

TRINITY_DN13619_c0_g1_i4	Glyma.10G289700.1	97.5	310	15	2	2162	788	2942	0	2169

TRINITY_DN13619_c0_g1_i5	Glyma.10G289700.2	98.35164835	310	15	2	2162	788	2942	0	2169

TRINITY_DN13619_c0_g1_i5	Glyma.10G289700.1	100	310	15	2	2162	788	2942	0	2169

TRINITY_DN16130_c2_g6_i13	Glyma.02G201300.3	100	193	4	791	2952	1	2165	0	2887

TRINITY_DN16130_c2_g6_i15	Glyma.02G201300.3	100	193	4	864	3025	1	2165	0	2887

TRINITY_DN16130_c2_g6_i3	Glyma.02G201300.3	99.05660377	193	4	464	2625	1	2165	0	2887

TRINITY_DN16130_c2_g6_i4	Glyma.02G201300.3	100	185	4	884	3045	1	2165	0	2931

TRINITY_DN668_c0_g1_i1	Glyma.14G058900.1	100	160	21	156	2312	1	2133	0	2832

TRINITY_DN10864_c0_g1_i1	Glyma.19G029100.1	96.36363636	145	5	2957	5123	2881	721	0	3160

TRINITY_DN10864_c0_g1_i2	Glyma.19G029100.1	98.55072464	145	5	2970	5136	2881	721	0	3160

TRINITY_DN10864_c0_g1_i3	Glyma.19G029100.1	100	145	5	2880	5046	2881	721	0	3160

TRINITY_DN14162_c0_g1_i2	Glyma.10G198600.1	99.05660377	139	1	706	2872	2164	1	0	3212

TRINITY_DN16130_c2_g6_i10	Glyma.02G201300.3	97.44186047	135	4	537	2698	1	2165	0	3208

TRINITY_DN16130_c2_g6_i5	Glyma.02G201300.3	98.66962306	135	4	864	3025	1	2165	0	3208

TRINITY_DN14162_c0_g1_i2	Glyma.20G191400.2	97.84946237	131	3	706	2872	2161	1	0	3238

TRINITY_DN14162_c0_g1_i2	Glyma.20G191400.1	97.84946237	131	3	706	2872	2161	1	0	3238

TRINITY_DN16130_c2_g6_i14	Glyma.02G201300.3	97.42173112	127	4	884	3045	1	2165	0	3253

TRINITY_DN14695_c0_g1_i1	Glyma.06G067100.1	100	124	3	357	2519	1	2166	0	3282

TRINITY_DN11672_c0_g2_i1	Glyma.02G060500.1	95.34883721	363	10	10	2171	2327	4485	0	1917

TRINITY_DN11672_c0_g2_i1	Glyma.02G060500.8	100	363	10	10	2171	2327	4485	0	1917

TRINITY_DN11672_c0_g2_i1	Glyma.02G060500.7	95.3271028	363	10	10	2171	2327	4485	0	1917

TRINITY_DN11672_c0_g2_i1	Glyma.02G060500.5	99.31506849	363	10	10	2171	2327	4485	0	1917

TRINITY_DN11672_c0_g2_i1	Glyma.02G060500.6	100	363	10	10	2171	2327	4485	0	1917

TRINITY_DN11672_c0_g2_i1	Glyma.02G060500.2	97.08029197	363	10	10	2171	2327	4485	0	1917

TRINITY_DN11672_c0_g2_i1	Glyma.02G060500.3	99.31506849	363	10	10	2171	2327	4485	0	1917

TRINITY_DN11672_c0_g2_i1	Glyma.02G060500.4	100	363	10	10	2171	2327	4485	0	1917

TRINITY_DN12815_c1_g1_i1	Glyma.18G095400.3	96.9924812	251	39	1009	3150	2297	168	0	2222

TRINITY_DN12815_c1_g1_i1	Glyma.18G095400.2	96.99074074	251	39	1009	3150	2297	168	0	2222

TRINITY_DN15353_c0_g1_i1	Glyma.18G233500.1	98.92473118	194	3	292	2455	2652	488	0	2905

TRINITY_DN15353_c0_g1_i1	Glyma.18G233500.2	98.92473118	194	3	292	2455	2334	170	0	2905

TRINITY_DN15353_c0_g1_i1	Glyma.09G259100.1	98.95833333	193	0	292	2457	2652	487	0	2931

TRINITY_DN8957_c0_g1_i5	Glyma.17G102800.2	100	165	17	87	2237	2123	1	0	2712

TRINITY_DN8957_c0_g1_i5	Glyma.17G102800.1	95.68345324	165	17	87	2237	2123	1	0	2712

TRINITY_DN10754_c0_g1_i1	Glyma.18G068800.2	100	157	0	268	2433	2166	1	0	3131

TRINITY_DN10754_c0_g2_i1	Glyma.18G068800.2	95.29411765	157	0	268	2433	2166	1	0	3131

TRINITY_DN10754_c0_g3_i1	Glyma.18G068800.2	95.3125	157	0	268	2433	2166	1	0	3131

TRINITY_DN11433_c0_g1_i2	Glyma.05G163800.4	97.05882353	150	11	230	2376	1	2162	0	3020

TRINITY_DN11433_c0_g1_i2	Glyma.05G163800.5	97.05882353	150	11	230	2376	1	2162	0	3020

TRINITY_DN15385_c0_g1_i2	Glyma.03G107700.1	98.41269841	291	22	1627	3769	2451	312	0	2082

TRINITY_DN15385_c0_g1_i5	Glyma.03G107700.1	100	280	19	1571	3722	2451	312	0	2202

TRINITY_DN263_c0_g1_i1	Glyma.08G027800.2	100	235	3	1	2161	2898	735	0	2663

TRINITY_DN263_c0_g1_i1	Glyma.08G027800.1	98.36065574	235	3	1	2161	2898	735	0	2663

TRINITY_DN13975_c0_g1_i1	Glyma.19G143600.3	100	173	9	1302	3452	1	2160	0	2916

TRINITY_DN13975_c0_g1_i1	Glyma.19G143600.5	95.91836735	173	9	1302	3452	1	2160	0	2916

TRINITY_DN13975_c0_g1_i1	Glyma.19G143600.2	96.77419355	173	9	1302	3452	1	2160	0	2916

TRINITY_DN13975_c0_g1_i1	Glyma.19G143600.4	100	173	9	1302	3452	1	2160	0	2916

TRINITY_DN13975_c0_g1_i1	Glyma.19G143600.1	98.77300613	173	9	1302	3452	1	2160	0	2916

TRINITY_DN13975_c0_g1_i2	Glyma.19G143600.3	98.4375	173	9	261	2411	1	2160	0	2916

TRINITY_DN13975_c0_g1_i2	Glyma.19G143600.5	97.02970297	173	9	261	2411	1	2160	0	2916

TRINITY_DN13975_c0_g1_i2	Glyma.19G143600.2	99.07407407	173	9	261	2411	1	2160	0	2916

TRINITY_DN13975_c0_g1_i2	Glyma.19G143600.4	99.21259843	173	9	261	2411	1	2160	0	2916

TRINITY_DN13975_c0_g1_i2	Glyma.19G143600.1	99.21259843	173	9	261	2411	1	2160	0	2916

TRINITY_DN5514_c0_g2_i1	Glyma.19G208700.1	98.62068966	167	6	213	2369	2161	1	0	2996

TRINITY_DN16279_c0_g3_i2	Glyma.08G315400.1	96.06126915	166	5	36	2194	62	2223	0	3020

TRINITY_DN8957_c0_g1_i1	Glyma.17G102800.2	100	148	12	87	2246	2123	1	0	2874

TRINITY_DN8957_c0_g1_i1	Glyma.17G102800.1	100	148	12	87	2246	2123	1	0	2874

TRINITY_DN8957_c0_g1_i2	Glyma.17G102800.2	98.44617092	148	12	87	2246	2123	1	0	2874

TRINITY_DN8957_c0_g1_i2	Glyma.17G102800.1	98.46153846	148	12	87	2246	2123	1	0	2874

TRINITY_DN15681_c0_g2_i5	Glyma.17G033700.2	95.65217391	146	15	287	2428	1	2148	0	2931

TRINITY_DN12050_c0_g2_i2	Glyma.16G093800.22	98.48484848	355	38	3467	5608	3018	5129	0	1552

TRINITY_DN12050_c0_g2_i3	Glyma.16G093800.22	100	355	38	3482	5623	3018	5129	0	1552

TRINITY_DN12050_c0_g2_i2	Glyma.16G093800.21	98.47328244	355	38	3467	5608	3003	5114	0	1552

TRINITY_DN12050_c0_g2_i3	Glyma.16G093800.21	100	355	38	3482	5623	3003	5114	0	1552

TRINITY_DN15137_c0_g1_i10	Glyma.04G019700.1	98.94736842	328	8	528	2687	2184	25	0	2128

TRINITY_DN12815_c1_g1_i3	Glyma.13G066200.1	97	326	38	1009	3159	2281	162	0	1821

TRINITY_DN15137_c0_g1_i5	Glyma.04G019700.1	95.92391304	300	8	556	2715	2184	25	0	2283

TRINITY_DN15171_c0_g1_i2	Glyma.10G012200.1	100	246	11	201	2359	2172	20	0	2531

TRINITY_DN15710_c0_g2_i1	Glyma.15G042900.2	98.85057471	231	11	390	2548	2147	1	0	2575

TRINITY_DN15710_c0_g2_i2	Glyma.15G042900.2	100	231	11	390	2548	2147	1	0	2575

TRINITY_DN9205_c0_g1_i4	Glyma.07G148100.7	100	197	8	472	2613	2163	1	0	2756

TRINITY_DN9205_c0_g1_i4	Glyma.07G148100.6	100	195	14	472	2613	2139	1	0	2612

TRINITY_DN3218_c0_g1_i1	Glyma.04G107100.2	97.88732394	179	10	1	2136	3805	5961	0	2778

TRINITY_DN3218_c0_g1_i1	Glyma.04G107100.1	97.89473684	179	10	1	2136	3805	5961	0	2778

TRINITY_DN5514_c0_g2_i1	Glyma.03G211500.1	97.14285714	168	5	213	2369	2158	1	0	2981

TRINITY_DN15937_c0_g1_i1	Glyma.13G276700.1	95.89041096	165	8	239	2393	4	2161	0	2985

TRINITY_DN15937_c0_g1_i2	Glyma.13G276700.1	100	165	8	239	2393	4	2161	0	2985

TRINITY_DN15937_c0_g1_i3	Glyma.13G276700.1	100	165	8	239	2393	4	2161	0	2985

TRINITY_DN15937_c0_g1_i5	Glyma.13G276700.1	95.09803922	165	8	239	2393	4	2161	0	2985

TRINITY_DN8905_c0_g1_i1	Glyma.17G259200.1	100	125	2	117	2274	13	2176	0	3265

TRINITY_DN10625_c0_g1_i1	Glyma.10G147200.2	99.44444444	110	3	1097	3260	2149	1	0	3290

TRINITY_DN15421_c1_g3_i2	Glyma.12G234200.1	99.43820225	271	5	927	3085	2447	292	0	2422

TRINITY_DN13902_c0_g1_i1	Glyma.11G219700.1	100	242	8	103	2256	1	2157	0	2556

TRINITY_DN10422_c0_g1_i2	Glyma.10G210400.1	95.94594595	212	7	372	2525	2160	1	0	2743

TRINITY_DN12739_c0_g1_i2	Glyma.15G091600.1	98.38709677	188	1	308	2464	1	2163	0	2915

TRINITY_DN11664_c0_g1_i1	Glyma.12G044200.2	95.40229885	183	10	1	2142	50	2200	0	2767

TRINITY_DN11664_c0_g1_i1	Glyma.12G044200.1	95.40540541	183	10	1	2142	50	2200	0	2767

TRINITY_DN11664_c0_g1_i2	Glyma.12G044200.2	97.56097561	183	10	1	2142	50	2200	0	2767

TRINITY_DN11664_c0_g1_i2	Glyma.12G044200.1	97.5	183	10	1	2142	50	2200	0	2767

TRINITY_DN11664_c0_g1_i3	Glyma.12G044200.2	100	183	10	1	2142	50	2200	0	2767

TRINITY_DN11664_c0_g1_i3	Glyma.12G044200.1	95.55555556	183	10	1	2142	50	2200	0	2767

TRINITY_DN11664_c0_g1_i4	Glyma.12G044200.2	95.04643963	183	10	1	2142	50	2200	0	2767

TRINITY_DN11664_c0_g1_i4	Glyma.12G044200.1	98.67256637	183	10	1	2142	50	2200	0	2767

TRINITY_DN11664_c0_g1_i5	Glyma.12G044200.2	99.06103286	183	10	1	2142	50	2200	0	2767

TRINITY_DN11664_c0_g1_i5	Glyma.12G044200.1	98.65771812	183	10	1	2142	50	2200	0	2767

TRINITY_DN3218_c0_g1_i1	Glyma.06G106900.1	98.2300885	173	8	1	2136	4399	6558	0	2843

TRINITY_DN3218_c0_g1_i1	Glyma.06G106900.2	98.24561404	173	8	1	2136	4399	6558	0	2843

TRINITY_DN13654_c0_g2_i1	Glyma.16G015400.1	96.72131148	169	13	437	2590	2148	1	0	2904

TRINITY_DN7530_c0_g1_i1	Glyma.03G219200.1	98.92473118	161	0	39	2201	1	2163	0	3103

TRINITY_DN7530_c0_g1_i1	Glyma.19G216100.1	98.92473118	152	0	39	2201	1	2163	0	3153

TRINITY_DN11433_c0_g1_i2	Glyma.05G163800.2	96.36363636	151	9	230	2376	1	2159	0	3029

TRINITY_DN11433_c0_g1_i2	Glyma.05G163800.3	96.36363636	151	9	230	2376	1	2159	0	3029

TRINITY_DN9106_c0_g1_i1	Glyma.07G054800.1	99.58333333	137	24	108	2220	1	2148	0	2815

TRINITY_DN9106_c0_g1_i1	Glyma.07G054800.4	100	137	25	108	2220	1	2139	0	2758

TRINITY_DN15910_c0_g1_i5	Glyma.03G220500.3	95.3125	131	3	248	2407	1	2163	0	3249

TRINITY_DN9237_c0_g1_i1	Glyma.11G254100.1	98.79518072	128	5	134	2290	1	2163	0	3247

TRINITY_DN6599_c0_g1_i1	Glyma.13G002200.1	98.82352941	507	50	244	2371	16	2149	0	784

TRINITY_DN13778_c1_g3_i3	Glyma.18G198800.1	97.68683274	424	54	199	2327	3286	1155	0	1236

TRINITY_DN14043_c0_g2_i3	Glyma.15G096200.14	97.48427673	354	27	251	2371	1	2145	0	1657

TRINITY_DN14043_c0_g2_i3	Glyma.15G096200.15	95.38461538	354	27	251	2371	1	2145	0	1657

TRINITY_DN14043_c0_g2_i3	Glyma.15G096200.2	95.38461538	354	27	251	2371	1	2145	0	1657

TRINITY_DN15867_c0_g3_i1	Glyma.15G172400.7	100	349	17	9	2149	458	2601	0	1807

TRINITY_DN15867_c0_g3_i1	Glyma.15G172400.2	100	349	17	9	2149	458	2601	0	1807

TRINITY_DN15867_c0_g3_i1	Glyma.15G172400.3	100	349	17	9	2149	458	2601	0	1807

TRINITY_DN15867_c0_g3_i1	Glyma.15G172400.6	100	349	17	9	2149	458	2601	0	1807

TRINITY_DN15867_c0_g3_i1	Glyma.15G172400.1	100	349	17	9	2149	458	2601	0	1807

TRINITY_DN12815_c1_g1_i4	Glyma.13G066200.1	97	264	34	1009	3159	2281	162	0	2187

TRINITY_DN16106_c0_g3_i2	Glyma.17G205700.6	96.96969697	257	12	533	2683	2595	442	0	2446

TRINITY_DN16106_c0_g3_i2	Glyma.17G205700.3	100	257	12	533	2683	2595	442	0	2446

TRINITY_DN16106_c0_g3_i2	Glyma.17G205700.5	98.63013699	253	16	533	2683	2580	442	0	2372

TRINITY_DN16106_c0_g3_i2	Glyma.17G205700.2	98.63013699	253	16	533	2683	2580	442	0	2372

TRINITY_DN263_c0_g1_i1	Glyma.05G221300.3	97.70114943	240	5	1	2161	2889	735	0	2612

TRINITY_DN263_c0_g1_i1	Glyma.05G221300.1	100	240	5	1	2161	2889	735	0	2612

TRINITY_DN263_c0_g1_i1	Glyma.05G221300.2	100	240	5	1	2161	2799	645	0	2612

TRINITY_DN12719_c0_g2_i6	Glyma.13G321300.51	96.27249357	160	21	356	2505	2112	1	0	2706

TRINITY_DN12719_c0_g2_i6	Glyma.13G321300.48	97	160	21	356	2505	2112	1	0	2706

TRINITY_DN12719_c0_g2_i6	Glyma.13G321300.18	97.41935484	160	21	356	2505	2112	1	0	2706

TRINITY_DN12719_c0_g2_i6	Glyma.13G321300.19	96.42857143	160	21	356	2505	2112	1	0	2706

TRINITY_DN12719_c0_g2_i6	Glyma.13G321300.52	96.34146341	160	21	356	2505	2112	1	0	2706

TRINITY_DN12719_c0_g2_i6	Glyma.13G321300.1	99.17695473	160	21	356	2505	2112	1	0	2706

TRINITY_DN12719_c0_g2_i6	Glyma.13G321300.20	100	160	21	356	2505	2112	1	0	2706

TRINITY_DN12719_c0_g2_i6	Glyma.13G321300.21	97.43589744	160	21	356	2505	2112	1	0	2706

TRINITY_DN12719_c0_g2_i8	Glyma.13G321300.51	100	160	21	356	2505	2112	1	0	2706

TRINITY_DN12719_c0_g2_i8	Glyma.13G321300.48	99.63768116	160	21	356	2505	2112	1	0	2706

TRINITY_DN12719_c0_g2_i8	Glyma.13G321300.18	100	160	21	356	2505	2112	1	0	2706

TRINITY_DN12719_c0_g2_i8	Glyma.13G321300.19	100	160	21	356	2505	2112	1	0	2706

TRINITY_DN12719_c0_g2_i8	Glyma.13G321300.52	100	160	21	356	2505	2112	1	0	2706

TRINITY_DN12719_c0_g2_i8	Glyma.13G321300.1	100	160	21	356	2505	2112	1	0	2706

TRINITY_DN12719_c0_g2_i8	Glyma.13G321300.20	100	160	21	356	2505	2112	1	0	2706

TRINITY_DN12719_c0_g2_i8	Glyma.13G321300.21	100	160	21	356	2505	2112	1	0	2706

TRINITY_DN14292_c0_g1_i1	Glyma.16G205700.1	96.77419355	159	18	316	2458	2134	1	0	2808

TRINITY_DN12319_c0_g1_i1	Glyma.19G224000.3	98.64864865	156	11	65	2206	1	2157	0	2966

TRINITY_DN12319_c0_g1_i1	Glyma.19G224000.1	100	156	11	65	2206	1	2157	0	2966

TRINITY_DN12319_c0_g1_i1	Glyma.19G224000.2	100	156	11	65	2206	1	2157	0	2966

TRINITY_DN12319_c0_g1_i2	Glyma.19G224000.3	98.81422925	156	11	109	2250	1	2157	0	2966

TRINITY_DN12319_c0_g1_i2	Glyma.19G224000.1	98.80952381	156	11	109	2250	1	2157	0	2966

TRINITY_DN12319_c0_g1_i2	Glyma.19G224000.2	98.80952381	156	11	109	2250	1	2157	0	2966

TRINITY_DN9106_c0_g1_i1	Glyma.16G023600.1	99.58333333	135	32	108	2220	1	2148	0	2837

TRINITY_DN9106_c0_g1_i1	Glyma.16G023600.3	99.5049505	134	36	108	2220	1	2139	0	2785

TRINITY_DN6599_c0_g1_i1	Glyma.20G066800.1	98.82352941	508	48	244	2371	16	2149	0	789

TRINITY_DN14043_c0_g2_i3	Glyma.15G096200.1	98.13829787	355	23	251	2371	1	2154	0	1720

TRINITY_DN15510_c0_g1_i2	Glyma.09G168400.2	98	349	19	137	2270	6	2160	0	1844

TRINITY_DN16230_c0_g1_i2	Glyma.17G233900.1	96.7032967	243	23	80	2181	2263	121	0	2146

TRINITY_DN15937_c0_g1_i1	Glyma.13G276700.4	100	165	7	239	2393	4	2158	0	3000

TRINITY_DN15937_c0_g1_i1	Glyma.13G276700.3	100	165	7	239	2393	4	2158	0	3000

TRINITY_DN15937_c0_g1_i1	Glyma.13G276700.2	100	165	7	239	2393	4	2158	0	3000

TRINITY_DN15937_c0_g1_i2	Glyma.13G276700.4	97.5	165	7	239	2393	4	2158	0	3000

TRINITY_DN15937_c0_g1_i2	Glyma.13G276700.3	96.36871508	165	7	239	2393	4	2158	0	3000

TRINITY_DN15937_c0_g1_i2	Glyma.13G276700.2	100	165	7	239	2393	4	2158	0	3000

TRINITY_DN15937_c0_g1_i3	Glyma.13G276700.4	99.27007299	165	7	239	2393	4	2158	0	3000

TRINITY_DN15937_c0_g1_i3	Glyma.13G276700.3	100	165	7	239	2393	4	2158	0	3000

TRINITY_DN15937_c0_g1_i3	Glyma.13G276700.2	99.48717949	165	7	239	2393	4	2158	0	3000

TRINITY_DN15937_c0_g1_i5	Glyma.13G276700.4	98.74476987	165	7	239	2393	4	2158	0	3000

TRINITY_DN15937_c0_g1_i5	Glyma.13G276700.3	100	165	7	239	2393	4	2158	0	3000

TRINITY_DN15937_c0_g1_i5	Glyma.13G276700.2	96.55172414	165	7	239	2393	4	2158	0	3000

TRINITY_DN11664_c0_g1_i1	Glyma.11G118900.1	98.11320755	162	7	1	2140	137	2288	0	2900

TRINITY_DN11664_c0_g1_i1	Glyma.11G118900.3	95	162	7	1	2140	137	2288	0	2900

TRINITY_DN11664_c0_g1_i2	Glyma.11G118900.1	100	162	7	1	2140	137	2288	0	2900

TRINITY_DN11664_c0_g1_i2	Glyma.11G118900.3	99.40652819	162	7	1	2140	137	2288	0	2900

TRINITY_DN11664_c0_g1_i3	Glyma.11G118900.1	97.21577726	162	7	1	2140	137	2288	0	2900

TRINITY_DN11664_c0_g1_i3	Glyma.11G118900.3	100	162	7	1	2140	137	2288	0	2900

TRINITY_DN11664_c0_g1_i4	Glyma.11G118900.1	99.31506849	162	7	1	2140	137	2288	0	2900

TRINITY_DN11664_c0_g1_i4	Glyma.11G118900.3	100	162	7	1	2140	137	2288	0	2900

TRINITY_DN11664_c0_g1_i5	Glyma.11G118900.1	97.57575758	162	7	1	2140	137	2288	0	2900

TRINITY_DN11664_c0_g1_i5	Glyma.11G118900.3	96.77419355	162	7	1	2140	137	2288	0	2900

TRINITY_DN15937_c0_g1_i1	Glyma.12G224600.5	100	152	4	239	2393	4	2161	0	3090

TRINITY_DN15937_c0_g1_i1	Glyma.12G224600.4	97.89473684	152	4	239	2393	4	2161	0	3090

TRINITY_DN15937_c0_g1_i1	Glyma.12G224600.1	95.83333333	152	4	239	2393	4	2161	0	3090

TRINITY_DN15937_c0_g1_i1	Glyma.12G224600.2	99.09365559	152	4	239	2393	4	2161	0	3090

TRINITY_DN15937_c0_g1_i2	Glyma.12G224600.5	97.69736842	152	4	239	2393	4	2161	0	3090

TRINITY_DN15937_c0_g1_i2	Glyma.12G224600.4	100	152	4	239	2393	4	2161	0	3090

TRINITY_DN15937_c0_g1_i2	Glyma.12G224600.1	99.53703704	152	4	239	2393	4	2161	0	3090

TRINITY_DN15937_c0_g1_i2	Glyma.12G224600.2	100	152	4	239	2393	4	2161	0	3090

TRINITY_DN15937_c0_g1_i3	Glyma.12G224600.5	95.41284404	152	4	239	2393	4	2161	0	3090

TRINITY_DN15937_c0_g1_i3	Glyma.12G224600.4	98.86363636	152	4	239	2393	4	2161	0	3090

TRINITY_DN15937_c0_g1_i3	Glyma.12G224600.1	97.5308642	152	4	239	2393	4	2161	0	3090

TRINITY_DN15937_c0_g1_i3	Glyma.12G224600.2	97.05882353	152	4	239	2393	4	2161	0	3090

TRINITY_DN15937_c0_g1_i5	Glyma.12G224600.5	98.48484848	152	4	239	2393	4	2161	0	3090

TRINITY_DN15937_c0_g1_i5	Glyma.12G224600.4	95.23809524	152	4	239	2393	4	2161	0	3090

TRINITY_DN15937_c0_g1_i5	Glyma.12G224600.1	95.11111111	152	4	239	2393	4	2161	0	3090

TRINITY_DN15937_c0_g1_i5	Glyma.12G224600.2	99.35064935	152	4	239	2393	4	2161	0	3090

TRINITY_DN12717_c0_g1_i1	Glyma.19G241800.2	97.26027397	147	1	234	2394	3	2160	0	3157

TRINITY_DN15906_c0_g1_i5	Glyma.19G168100.1	98.67549669	138	5	347	2495	2487	330	0	3129

TRINITY_DN8905_c0_g1_i1	Glyma.14G220300.1	100	131	1	117	2274	13	2173	0	3245

TRINITY_DN13526_c0_g1_i1	Glyma.01G214700.1	97.61904762	129	15	157	2301	49	2181	0	2992

TRINITY_DN16314_c1_g1_i1	Glyma.10G223500.2	100	417	23	40	2183	3083	937	0	1491

TRINITY_DN15867_c0_g3_i1	Glyma.09G066400.1	100	346	27	9	2149	458	2580	0	1711

TRINITY_DN16245_c0_g3_i4	Glyma.02G129500.1	99.60784314	293	6	262	2419	3596	1444	0	2307

TRINITY_DN16245_c0_g3_i4	Glyma.02G129500.2	95.8677686	293	6	262	2419	3221	1069	0	2307

TRINITY_DN16041_c0_g1_i5	Glyma.15G030400.1	100	285	18	153	2292	2904	756	0	2209

TRINITY_DN16041_c0_g1_i8	Glyma.15G030400.1	96.03960396	285	18	380	2519	2904	756	0	2209

TRINITY_DN15421_c1_g3_i2	Glyma.12G234200.7	100	271	4	927	3085	2444	292	0	2436

TRINITY_DN15421_c1_g3_i2	Glyma.12G234200.5	99.05660377	271	4	927	3085	2444	292	0	2436

TRINITY_DN15421_c1_g3_i2	Glyma.12G234200.6	96.62162162	271	4	927	3085	2444	292	0	2436

TRINITY_DN15421_c1_g3_i2	Glyma.12G234200.4	100	271	4	927	3085	2444	292	0	2436

TRINITY_DN15421_c1_g3_i2	Glyma.12G234200.3	100	271	4	927	3085	2444	292	0	2436

TRINITY_DN15421_c1_g3_i2	Glyma.12G234200.2	99.71098266	271	4	927	3085	2444	292	0	2436

TRINITY_DN16041_c0_g1_i5	Glyma.13G343900.4	96.44444444	270	20	153	2292	2898	756	0	2254

TRINITY_DN16041_c0_g1_i5	Glyma.13G343900.3	100	270	20	153	2292	2898	756	0	2254

TRINITY_DN16041_c0_g1_i5	Glyma.13G343900.2	98.27586207	270	20	153	2292	2898	756	0	2254

TRINITY_DN16041_c0_g1_i5	Glyma.13G343900.1	100	270	20	153	2292	2898	756	0	2254

TRINITY_DN16041_c0_g1_i8	Glyma.13G343900.4	96.03960396	270	20	380	2519	2898	756	0	2254

TRINITY_DN16041_c0_g1_i8	Glyma.13G343900.3	96.03960396	270	20	380	2519	2898	756	0	2254

TRINITY_DN16041_c0_g1_i8	Glyma.13G343900.2	96.03960396	270	20	380	2519	2898	756	0	2254

TRINITY_DN16041_c0_g1_i8	Glyma.13G343900.1	96.03960396	270	20	380	2519	2898	756	0	2254

TRINITY_DN15991_c0_g1_i1	Glyma.03G237100.1	96.93094629	207	35	701	2812	2132	4	0	2331

TRINITY_DN15991_c0_g1_i4	Glyma.03G237100.1	96.3800905	207	35	117	2228	2132	4	0	2331

TRINITY_DN15991_c0_g1_i6	Glyma.03G237100.1	100	207	35	701	2812	2132	4	0	2331

TRINITY_DN6479_c0_g1_i1	Glyma.02G045200.1	100	203	25	149	2290	1	2118	0	2477

TRINITY_DN15991_c0_g1_i1	Glyma.19G234800.1	96.92923899	195	21	702	2812	2146	4	0	2483

TRINITY_DN15991_c0_g1_i4	Glyma.19G234800.1	99.09090909	195	21	118	2228	2146	4	0	2483

TRINITY_DN15991_c0_g1_i6	Glyma.19G234800.1	97.02048417	195	21	702	2812	2146	4	0	2483

TRINITY_DN8193_c0_g1_i1	Glyma.03G243100.1	100	181	3	152	2311	12	2165	0	2948

TRINITY_DN15137_c0_g1_i2	Glyma.04G019700.1	98.26086957	173	1	528	2684	2184	25	0	3011

TRINITY_DN10093_c0_g1_i1	Glyma.03G002400.1	97.91666667	171	1	347	2506	2157	1	0	3022

TRINITY_DN16185_c4_g1_i13	Glyma.07G049700.1	95.52238806	165	2	129	2279	5742	3583	0	3016

TRINITY_DN15529_c1_g1_i2	Glyma.07G089000.1	97.05882353	164	0	20	2179	2160	1	0	3081

TRINITY_DN15529_c1_g1_i2	Glyma.07G089000.3	98.63636364	164	0	20	2179	2160	1	0	3081

TRINITY_DN15529_c1_g1_i2	Glyma.07G089000.4	98.63636364	164	0	20	2179	2160	1	0	3081

TRINITY_DN15529_c1_g1_i2	Glyma.07G089000.2	95.34883721	164	0	20	2179	2160	1	0	3081

TRINITY_DN15529_c1_g1_i4	Glyma.07G089000.1	96.12403101	164	0	20	2179	2160	1	0	3081

TRINITY_DN15529_c1_g1_i4	Glyma.07G089000.3	95.69892473	164	0	20	2179	2160	1	0	3081

TRINITY_DN15529_c1_g1_i4	Glyma.07G089000.4	97.95918367	164	0	20	2179	2160	1	0	3081

TRINITY_DN15529_c1_g1_i4	Glyma.07G089000.2	96.32768362	164	0	20	2179	2160	1	0	3081

TRINITY_DN10093_c0_g1_i1	Glyma.07G107700.1	98.24561404	159	1	347	2506	2157	1	0	3088

TRINITY_DN15220_c0_g1_i1	Glyma.16G008400.1	98.820059	155	13	517	2655	1	2148	0	2916

TRINITY_DN12319_c0_g1_i1	Glyma.03G227100.1	96.5	154	15	65	2206	1	2157	0	3000

TRINITY_DN12319_c0_g1_i2	Glyma.03G227100.1	98.80952381	154	15	109	2250	1	2157	0	3000

TRINITY_DN15137_c0_g1_i12	Glyma.04G019700.1	100	145	1	556	2712	2184	25	0	3166

TRINITY_DN11644_c0_g2_i1	Glyma.13G135900.4	99.08256881	140	0	418	2577	2160	1	0	3214

TRINITY_DN11644_c0_g2_i1	Glyma.13G135900.3	100	140	0	418	2577	2160	1	0	3214

TRINITY_DN11644_c0_g2_i2	Glyma.13G135900.4	100	140	0	418	2577	2160	1	0	3214

TRINITY_DN11644_c0_g2_i2	Glyma.13G135900.3	98.61111111	140	0	418	2577	2160	1	0	3214

TRINITY_DN15007_c0_g1_i10	Glyma.11G242000.1	100	129	3	664	2822	2150	1	0	3203

TRINITY_DN15007_c0_g1_i7	Glyma.11G242000.1	98.57142857	129	3	664	2822	2150	1	0	3203

TRINITY_DN14891_c0_g1_i1	Glyma.08G119700.1	95.65217391	405	26	321	2466	1	2137	0	1517

TRINITY_DN14043_c0_g2_i3	Glyma.15G096200.9	98.12792512	354	26	251	2371	1	2142	0	1670

TRINITY_DN14043_c0_g2_i3	Glyma.15G096200.12	100	354	26	251	2371	1	2142	0	1670

TRINITY_DN14043_c0_g2_i3	Glyma.15G096200.13	100	354	26	251	2371	1	2142	0	1670

TRINITY_DN4116_c0_g1_i1	Glyma.17G118300.2	99.3993994	215	20	96	2221	2147	1	0	2505

TRINITY_DN13377_c0_g1_i4	Glyma.15G100500.5	98.34024896	205	31	381	2485	5	2151	0	2425

TRINITY_DN13377_c0_g1_i5	Glyma.15G100500.5	97.2972973	205	31	176	2280	5	2151	0	2425

TRINITY_DN16345_c0_g1_i8	Glyma.08G044700.5	98.30508475	187	5	1553	3709	2634	484	0	2887

TRINITY_DN14492_c1_g1_i4	Glyma.03G192300.2	97.5	145	1	537	2692	1	2159	0	3164

TRINITY_DN15007_c0_g1_i10	Glyma.18G015100.1	100	135	1	664	2822	2150	1	0	3181

TRINITY_DN15007_c0_g1_i7	Glyma.18G015100.1	98.80952381	135	1	664	2822	2150	1	0	3181

TRINITY_DN12050_c0_g2_i1	Glyma.16G093800.22	100	340	35	255	2390	3021	5129	0	1642

TRINITY_DN12050_c0_g2_i1	Glyma.16G093800.21	100	340	35	255	2390	3006	5114	0	1642

TRINITY_DN16230_c0_g1_i2	Glyma.17G233900.2	100	243	22	80	2181	2260	121	0	2161

TRINITY_DN6479_c0_g1_i1	Glyma.16G122800.1	99.42857143	193	22	149	2290	1	2121	0	2573

TRINITY_DN6479_c0_g1_i1	Glyma.16G122800.2	100	193	22	149	2290	1	2121	0	2573

TRINITY_DN6479_c0_g1_i1	Glyma.16G122800.3	95.04132231	193	22	149	2290	1	2121	0	2573

TRINITY_DN6479_c0_g1_i1	Glyma.16G122800.4	97.15189873	193	22	149	2290	1	2121	0	2573

TRINITY_DN15378_c1_g4_i2	Glyma.02G267300.1	98.99497487	182	0	202	2359	2646	489	0	2977

TRINITY_DN3076_c0_g1_i1	Glyma.17G074500.1	100	178	20	186	2312	1	2143	0	2702

TRINITY_DN15378_c1_g4_i2	Glyma.14G050200.1	98.99497487	167	0	202	2359	2646	489	0	3061

TRINITY_DN15378_c1_g4_i2	Glyma.14G050200.2	98.99497487	167	0	202	2359	2640	483	0	3061

TRINITY_DN15378_c1_g4_i2	Glyma.14G050200.3	98.99497487	167	0	202	2359	2553	396	0	3061

TRINITY_DN16182_c0_g1_i2	Glyma.17G027600.1	95.45454545	159	4	110	2266	16	2169	0	3072

TRINITY_DN16182_c0_g1_i3	Glyma.17G027600.1	95.80514208	159	4	110	2266	16	2169	0	3072

TRINITY_DN15220_c0_g1_i1	Glyma.07G039600.1	98.820059	159	12	517	2655	1	2145	0	2898

TRINITY_DN14891_c0_g1_i1	Glyma.05G162100.1	95.65217391	398	25	321	2465	1	2136	0	1565

TRINITY_DN16443_c1_g1_i2	Glyma.16G125800.1	96.73590504	381	34	7	2142	3203	1065	0	1620

TRINITY_DN12815_c1_g1_i2	Glyma.13G066200.1	96.98113208	311	33	1009	3156	2281	162	0	1964

TRINITY_DN15137_c0_g1_i5	Glyma.14G222700.2	100	262	8	622	2770	2223	69	0	2468

TRINITY_DN13902_c0_g1_i1	Glyma.18G037700.1	100	247	2	103	2256	1	2154	0	2577

TRINITY_DN12815_c1_g1_i1	Glyma.13G066200.1	96.98492462	245	31	1009	3156	2281	162	0	2329

TRINITY_DN15434_c0_g1_i2	Glyma.20G067300.1	97.00374532	197	1	180	2336	2250	95	0	2885

TRINITY_DN14043_c0_g2_i3	Glyma.07G250400.2	96.27659574	193	12	251	2377	1	2148	0	2662

TRINITY_DN13654_c0_g2_i1	Glyma.07G046800.1	100	159	6	437	2590	2142	1	0	2987

TRINITY_DN15906_c0_g1_i5	Glyma.03G167000.1	100	125	2	347	2494	2487	331	0	3232

TRINITY_DN11749_c0_g1_i1	Glyma.06G002400.4	96.74796748	169	4	1	2156	299	2431	0	2896

TRINITY_DN11749_c0_g1_i2	Glyma.06G002400.4	96.19686801	169	4	1	2156	299	2431	0	2896

TRINITY_DN11749_c0_g1_i1	Glyma.06G002400.3	100	167	6	1	2156	299	2425	0	2868

TRINITY_DN11749_c0_g1_i2	Glyma.06G002400.3	96.74796748	167	6	1	2156	299	2425	0	2868

TRINITY_DN12612_c0_g1_i2	Glyma.08G361900.1	97.05882353	163	8	60	2200	13	2168	0	2981

TRINITY_DN15691_c0_g1_i2	Glyma.02G145300.1	96.95121951	159	5	196	2338	46	2200	0	3011

TRINITY_DN13619_c0_g1_i1	Glyma.18G288500.6	100	135	10	1	2156	799	2924	0	3040

TRINITY_DN13619_c0_g1_i1	Glyma.18G288500.2	100	135	10	1	2156	799	2924	0	3040

TRINITY_DN13619_c0_g1_i1	Glyma.18G288500.1	98.71794872	135	10	1	2156	799	2924	0	3040

TRINITY_DN13619_c0_g1_i1	Glyma.18G288500.5	100	135	10	1	2156	799	2924	0	3040

TRINITY_DN13619_c0_g1_i2	Glyma.18G288500.6	95.50748752	135	10	1	2156	799	2924	0	3040

TRINITY_DN13619_c0_g1_i2	Glyma.18G288500.2	95.51020408	135	10	1	2156	799	2924	0	3040

TRINITY_DN13619_c0_g1_i2	Glyma.18G288500.1	97.70992366	135	10	1	2156	799	2924	0	3040

TRINITY_DN13619_c0_g1_i2	Glyma.18G288500.5	97.71167048	135	10	1	2156	799	2924	0	3040

TRINITY_DN13619_c0_g1_i4	Glyma.18G288500.6	99.14529915	135	10	1	2156	799	2924	0	3040

TRINITY_DN13619_c0_g1_i4	Glyma.18G288500.2	96.44128114	135	10	1	2156	799	2924	0	3040

TRINITY_DN13619_c0_g1_i4	Glyma.18G288500.1	100	135	10	1	2156	799	2924	0	3040

TRINITY_DN13619_c0_g1_i4	Glyma.18G288500.5	100	135	10	1	2156	799	2924	0	3040

TRINITY_DN13619_c0_g1_i5	Glyma.18G288500.6	98.64864865	135	10	1	2156	799	2924	0	3040

TRINITY_DN13619_c0_g1_i5	Glyma.18G288500.2	97.59615385	135	10	1	2156	799	2924	0	3040

TRINITY_DN13619_c0_g1_i5	Glyma.18G288500.1	99.15254237	135	10	1	2156	799	2924	0	3040

TRINITY_DN13619_c0_g1_i5	Glyma.18G288500.5	99.56331878	135	10	1	2156	799	2924	0	3040

TRINITY_DN13619_c0_g1_i1	Glyma.02G093600.4	100	127	10	1	2156	814	2948	0	3142

TRINITY_DN13619_c0_g1_i1	Glyma.02G093600.1	100	127	10	1	2156	814	2948	0	3142

TRINITY_DN13619_c0_g1_i2	Glyma.02G093600.4	95.23809524	127	10	1	2156	814	2948	0	3142

TRINITY_DN13619_c0_g1_i2	Glyma.02G093600.1	96	127	10	1	2156	814	2948	0	3142

TRINITY_DN13619_c0_g1_i4	Glyma.02G093600.4	100	127	10	1	2156	814	2948	0	3142

TRINITY_DN13619_c0_g1_i4	Glyma.02G093600.1	96.875	127	10	1	2156	814	2948	0	3142

TRINITY_DN13619_c0_g1_i5	Glyma.02G093600.4	98.93617021	127	10	1	2156	814	2948	0	3142

TRINITY_DN13619_c0_g1_i5	Glyma.02G093600.1	96.55172414	127	10	1	2156	814	2948	0	3142

TRINITY_DN16443_c1_g1_i2	Glyma.16G171400.1	100	389	26	7	2142	3047	906	0	1618

TRINITY_DN16443_c1_g1_i2	Glyma.16G126000.1	100	368	32	7	2142	3200	1065	0	1696

TRINITY_DN14337_c0_g2_i3	Glyma.15G208700.1	98.24561404	352	18	260	2396	2187	48	0	1816

TRINITY_DN15764_c0_g1_i6	Glyma.09G135100.1	100	300	8	513	2654	30	2183	0	2228

TRINITY_DN15329_c0_g2_i7	Glyma.02G085000.3	100	197	27	377	2513	2182	68	0	2514

TRINITY_DN15329_c0_g2_i7	Glyma.02G085000.1	97.91666667	197	27	377	2513	2182	68	0	2514

TRINITY_DN14891_c0_g1_i1	Glyma.13G225900.1	98.24561404	183	6	321	2471	1	2151	0	2915

TRINITY_DN4333_c0_g1_i1	Glyma.02G111500.6	95.45454545	154	7	321	2472	2139	1	0	3003

TRINITY_DN4333_c0_g1_i1	Glyma.02G111500.1	98.21428571	154	7	321	2472	2139	1	0	3003

TRINITY_DN3274_c0_g1_i1	Glyma.20G218600.4	97.91666667	132	7	1255	3400	2593	440	0	3184

TRINITY_DN9051_c1_g1_i1	Glyma.18G297900.1	99.00990099	124	2	46	2199	4989	2836	0	3280

TRINITY_DN15137_c0_g1_i10	Glyma.14G222700.2	98.94736842	245	8	597	2742	2220	69	0	2556

TRINITY_DN15137_c0_g1_i8	Glyma.14G222700.1	100	245	8	683	2828	2220	69	0	2556

TRINITY_DN10833_c0_g1_i1	Glyma.18G244800.1	97.10144928	207	9	882	3019	2204	61	0	2663

TRINITY_DN14891_c0_g1_i1	Glyma.15G086200.1	97.88732394	196	4	321	2471	1	2151	0	2854

TRINITY_DN11274_c0_g1_i1	Glyma.09G251800.1	97.08737864	182	0	683	2836	2154	1	0	2970

TRINITY_DN15332_c0_g2_i1	Glyma.19G243900.2	95.6043956	144	2	359	2509	2148	1	0	3121

TRINITY_DN15332_c0_g2_i2	Glyma.19G243900.2	99.69512195	144	2	457	2607	2148	1	0	3121

TRINITY_DN15332_c0_g2_i3	Glyma.19G243900.2	97.77777778	144	2	220	2370	2148	1	0	3121

TRINITY_DN14727_c0_g1_i1	Glyma.06G320900.1	98.33333333	140	9	563	2716	2298	160	0	3105

TRINITY_DN15187_c0_g1_i1	Glyma.15G271800.1	100	136	7	2628	4769	2148	1	0	3109

TRINITY_DN15910_c0_g1_i5	Glyma.03G220500.1	96.90265487	130	3	257	2407	67	2220	0	3238

TRINITY_DN14779_c0_g1_i2	Glyma.17G246600.2	95.45454545	88	7	644	2784	2150	1	0	3380

TRINITY_DN14779_c0_g1_i3	Glyma.17G246600.2	95.43147208	88	7	745	2885	2150	1	0	3380

TRINITY_DN16443_c1_g1_i2	Glyma.16G171200.1	96.20991254	377	26	7	2142	3206	1065	0	1707

TRINITY_DN13377_c0_g1_i1	Glyma.07G253800.11	98.9010989	346	54	344	2413	7	2139	0	1393

TRINITY_DN13377_c0_g1_i1	Glyma.07G253800.12	98.9010989	346	54	344	2413	7	2139	0	1393

TRINITY_DN13377_c0_g1_i1	Glyma.07G253800.7	98.9010989	346	54	344	2413	7	2139	0	1393

TRINITY_DN13377_c0_g1_i1	Glyma.07G253800.6	98.9010989	346	54	344	2413	7	2139	0	1393

TRINITY_DN13377_c0_g1_i2	Glyma.07G253800.11	97.36842105	346	54	383	2452	7	2139	0	1393

TRINITY_DN13377_c0_g1_i2	Glyma.07G253800.12	97.36842105	346	54	383	2452	7	2139	0	1393

TRINITY_DN13377_c0_g1_i2	Glyma.07G253800.7	95.18518519	346	54	383	2452	7	2139	0	1393

TRINITY_DN13377_c0_g1_i2	Glyma.07G253800.6	100	346	54	383	2452	7	2139	0	1393

TRINITY_DN13377_c0_g1_i3	Glyma.07G253800.11	100	346	54	178	2247	7	2139	0	1393

TRINITY_DN13377_c0_g1_i3	Glyma.07G253800.12	100	346	54	178	2247	7	2139	0	1393

TRINITY_DN13377_c0_g1_i3	Glyma.07G253800.7	100	346	54	178	2247	7	2139	0	1393

TRINITY_DN13377_c0_g1_i3	Glyma.07G253800.6	98.72881356	346	54	178	2247	7	2139	0	1393

TRINITY_DN13377_c0_g1_i4	Glyma.07G253800.11	96.55172414	346	54	383	2452	7	2139	0	1393

TRINITY_DN13377_c0_g1_i4	Glyma.07G253800.12	100	346	54	383	2452	7	2139	0	1393

TRINITY_DN13377_c0_g1_i4	Glyma.07G253800.7	98.57142857	346	54	383	2452	7	2139	0	1393

TRINITY_DN13377_c0_g1_i4	Glyma.07G253800.6	100	346	54	383	2452	7	2139	0	1393

TRINITY_DN13377_c0_g1_i5	Glyma.07G253800.11	95.91836735	346	54	178	2247	7	2139	0	1393

TRINITY_DN13377_c0_g1_i5	Glyma.07G253800.12	95.06398537	346	54	178	2247	7	2139	0	1393

TRINITY_DN13377_c0_g1_i5	Glyma.07G253800.7	95.06578947	346	54	178	2247	7	2139	0	1393

TRINITY_DN13377_c0_g1_i5	Glyma.07G253800.6	98.1595092	346	54	178	2247	7	2139	0	1393

TRINITY_DN9794_c0_g2_i1	Glyma.06G046600.1	95.63567362	301	24	256	2382	1	2142	0	2069

TRINITY_DN16069_c0_g1_i1	Glyma.08G156800.1	99.14529915	253	13	2	2145	271	2417	0	2477

TRINITY_DN15501_c0_g2_i1	Glyma.07G028500.1	100	243	6	303	2452	2305	156	0	2591

TRINITY_DN15501_c0_g2_i1	Glyma.08G214300.1	100	240	6	303	2452	2305	156	0	2608

TRINITY_DN653_c0_g1_i1	Glyma.18G235900.1	97.04968944	217	9	139	2261	2153	1	0	2580

TRINITY_DN14670_c0_g1_i2	Glyma.13G065500.16	97.6744186	200	7	1212	3360	1	2143	0	2778

TRINITY_DN15979_c0_g3_i1	Glyma.04G214200.8	98.85057471	179	14	666	2797	2251	108	0	2791

TRINITY_DN15979_c0_g3_i2	Glyma.04G214200.8	97.22891566	179	14	666	2797	2251	108	0	2791

TRINITY_DN13654_c0_g1_i1	Glyma.19G261700.1	98.85057471	165	2	325	2477	2166	20	0	3024

TRINITY_DN13398_c0_g1_i1	Glyma.08G264900.4	95.19094767	145	8	622	2762	2621	472	0	3075

TRINITY_DN9165_c0_g1_i1	Glyma.11G224600.1	98.63013699	410	36	123	2257	94	2204	0	1328

TRINITY_DN15510_c0_g1_i1	Glyma.09G168400.2	96.61016949	321	21	433	2552	17	2160	0	1938

TRINITY_DN15510_c0_g1_i4	Glyma.09G168400.2	98.68421053	321	21	459	2578	17	2160	0	1938

TRINITY_DN16132_c0_g1_i3	Glyma.04G056200.1	98.46547315	226	10	681	2824	41	2187	0	2638

TRINITY_DN421_c0_g1_i1	Glyma.05G113300.1	99.39393939	213	15	6	2144	1	2133	0	2588

TRINITY_DN15893_c0_g4_i11	Glyma.12G010800.1	99.02912621	189	25	342	2473	1	2096	0	2436

TRINITY_DN15893_c0_g4_i3	Glyma.12G010800.1	97.61904762	189	25	342	2473	1	2096	0	2436

TRINITY_DN15893_c0_g4_i4	Glyma.12G010800.1	99.57081545	189	25	777	2908	1	2096	0	2436

TRINITY_DN6786_c0_g1_i1	Glyma.08G201600.1	95	182	22	9	2141	1	2136	0	2739

TRINITY_DN8957_c0_g1_i5	Glyma.05G024500.2	98.46153846	162	11	100	2237	2123	1	0	2798

TRINITY_DN8957_c0_g1_i5	Glyma.05G024500.5	98.4375	162	11	100	2237	2123	1	0	2798

TRINITY_DN8957_c0_g1_i5	Glyma.05G024500.3	95.68345324	162	11	100	2237	2123	1	0	2798

TRINITY_DN8957_c0_g1_i5	Glyma.05G024500.4	95.66666667	162	11	100	2237	2123	1	0	2798

TRINITY_DN8957_c0_g1_i5	Glyma.05G024500.1	95.69892473	162	11	100	2237	2123	1	0	2798

TRINITY_DN9714_c0_g2_i1	Glyma.03G099800.1	100	155	12	337	2467	4702	2557	0	2940

TRINITY_DN9046_c0_g1_i1	Glyma.17G111200.6	96.66666667	133	3	524	2674	2148	1	0	3205

TRINITY_DN9046_c0_g1_i1	Glyma.17G111200.7	96.66666667	133	3	524	2674	2148	1	0	3205

TRINITY_DN9046_c0_g2_i1	Glyma.17G111200.6	95.47169811	133	3	429	2579	2148	1	0	3205

TRINITY_DN9046_c0_g2_i1	Glyma.17G111200.7	95.45454545	133	3	429	2579	2148	1	0	3205

TRINITY_DN15501_c0_g2_i2	Glyma.08G214300.1	100	371	8	377	2523	2302	156	0	1866

TRINITY_DN16163_c0_g1_i2	Glyma.12G100100.1	98.52941176	258	15	679	2819	150	2285	0	2381

TRINITY_DN16163_c0_g1_i5	Glyma.12G100100.1	98.7012987	258	15	188	2328	150	2285	0	2381

TRINITY_DN16163_c0_g1_i6	Glyma.12G100100.1	100	258	15	657	2797	150	2285	0	2381

TRINITY_DN16163_c0_g1_i2	Glyma.12G100100.2	99.00990099	258	15	679	2819	48	2183	0	2381

TRINITY_DN16163_c0_g1_i2	Glyma.12G100100.3	99.00990099	258	15	679	2819	48	2183	0	2381

TRINITY_DN16163_c0_g1_i5	Glyma.12G100100.2	98.7012987	258	15	188	2328	48	2183	0	2381

TRINITY_DN16163_c0_g1_i5	Glyma.12G100100.3	98.7012987	258	15	188	2328	48	2183	0	2381

TRINITY_DN16163_c0_g1_i6	Glyma.12G100100.2	100	258	15	657	2797	48	2183	0	2381

TRINITY_DN16163_c0_g1_i6	Glyma.12G100100.3	100	258	15	657	2797	48	2183	0	2381

TRINITY_DN11257_c0_g1_i1	Glyma.04G053100.1	95.85253456	235	18	144	2273	1	2136	0	2438

TRINITY_DN15950_c1_g1_i1	Glyma.17G231600.1	98.68421053	226	16	227	2366	2131	1	0	2519

TRINITY_DN9363_c0_g1_i1	Glyma.20G084100.1	95	193	5	213	2358	2409	261	0	2857

TRINITY_DN10255_c0_g1_i3	Glyma.12G039200.1	100	192	10	1	2139	303	2450	0	2811

TRINITY_DN10255_c0_g1_i3	Glyma.11G113300.1	100	182	9	1	2139	291	2438	0	2867

TRINITY_DN16185_c4_g1_i13	Glyma.16G018400.1	100	174	2	129	2279	5709	3577	0	2892

TRINITY_DN16185_c4_g1_i13	Glyma.16G018400.8	96.2962963	174	2	129	2279	5694	3562	0	2892

TRINITY_DN16185_c4_g1_i13	Glyma.16G018400.7	100	174	2	129	2279	4374	2242	0	2892

TRINITY_DN16185_c4_g1_i13	Glyma.16G018400.6	100	174	2	129	2279	4323	2191	0	2892

TRINITY_DN16185_c4_g1_i13	Glyma.16G018400.5	97.36842105	174	2	129	2279	4308	2176	0	2892

TRINITY_DN16185_c4_g1_i13	Glyma.16G018400.3	98.9010989	174	2	129	2279	3933	1801	0	2892

TRINITY_DN16185_c4_g1_i13	Glyma.16G018400.4	99.19354839	174	2	129	2279	3933	1801	0	2892

TRINITY_DN16185_c4_g1_i13	Glyma.16G018400.2	97.95918367	174	2	129	2279	3918	1786	0	2892

TRINITY_DN11433_c0_g1_i2	Glyma.08G121100.5	97.05426357	167	6	230	2376	1	2144	0	2976

TRINITY_DN11433_c0_g1_i2	Glyma.08G121100.4	100	167	6	230	2376	1	2144	0	2976

TRINITY_DN12218_c0_g1_i1	Glyma.10G049200.1	100	156	25	52	2148	1	2151	0	2760

TRINITY_DN16237_c0_g1_i10	Glyma.06G164900.2	100	152	24	116	2211	203	2343	0	2710

TRINITY_DN8957_c0_g1_i1	Glyma.05G024500.2	100	141	7	100	2246	2123	1	0	2985

TRINITY_DN8957_c0_g1_i1	Glyma.05G024500.5	100	141	7	100	2246	2123	1	0	2985

TRINITY_DN8957_c0_g1_i1	Glyma.05G024500.3	100	141	7	100	2246	2123	1	0	2985

TRINITY_DN8957_c0_g1_i1	Glyma.05G024500.4	100	141	7	100	2246	2123	1	0	2985

TRINITY_DN8957_c0_g1_i1	Glyma.05G024500.1	100	141	7	100	2246	2123	1	0	2985

TRINITY_DN8957_c0_g1_i2	Glyma.05G024500.2	98.46153846	141	7	100	2246	2123	1	0	2985

TRINITY_DN8957_c0_g1_i2	Glyma.05G024500.5	98.4496124	141	7	100	2246	2123	1	0	2985

TRINITY_DN8957_c0_g1_i2	Glyma.05G024500.3	98.4496124	141	7	100	2246	2123	1	0	2985

TRINITY_DN8957_c0_g1_i2	Glyma.05G024500.4	98.4375	141	7	100	2246	2123	1	0	2985

TRINITY_DN8957_c0_g1_i2	Glyma.05G024500.1	98.46153846	141	7	100	2246	2123	1	0	2985

TRINITY_DN16237_c0_g1_i10	Glyma.04G200600.2	98.50746269	138	22	116	2211	203	2343	0	2787

TRINITY_DN15826_c0_g1_i3	Glyma.04G086800.1	97.44897959	137	6	162	2300	1	2151	0	3136

TRINITY_DN16443_c1_g1_i2	Glyma.16G170800.1	97.05882353	382	16	7	2142	2594	453	0	1712

TRINITY_DN14043_c0_g2_i3	Glyma.15G096200.6	95.49071618	355	23	251	2371	1	2133	0	1707

TRINITY_DN14043_c0_g2_i3	Glyma.15G096200.8	95.09981851	355	23	251	2371	1	2133	0	1707

TRINITY_DN14043_c0_g2_i3	Glyma.15G096200.10	95.10050251	355	23	251	2371	1	2133	0	1707

TRINITY_DN16245_c0_g3_i3	Glyma.06G196400.1	98.63945578	265	20	213	2346	2472	333	0	2335

TRINITY_DN4598_c0_g1_i1	Glyma.18G048000.1	95.20766773	246	25	157	2259	43	2178	0	2213

TRINITY_DN39405_c0_g1_i1	Glyma.20G151800.1	99.12280702	238	4	215	2352	2980	831	0	2575

TRINITY_DN15893_c0_g4_i11	Glyma.09G225600.5	99.42857143	192	18	342	2473	1	2108	0	2519

TRINITY_DN15893_c0_g4_i11	Glyma.09G225600.4	96.51376147	192	18	342	2473	1	2108	0	2519

TRINITY_DN15893_c0_g4_i11	Glyma.09G225600.1	99.68847352	192	18	342	2473	1	2108	0	2519

TRINITY_DN15893_c0_g4_i3	Glyma.09G225600.5	97.26027397	192	18	342	2473	1	2108	0	2519

TRINITY_DN15893_c0_g4_i3	Glyma.09G225600.4	97.61904762	192	18	342	2473	1	2108	0	2519

TRINITY_DN15893_c0_g4_i3	Glyma.09G225600.1	97.61904762	192	18	342	2473	1	2108	0	2519

TRINITY_DN15893_c0_g4_i4	Glyma.09G225600.5	98.52941176	192	18	777	2908	1	2108	0	2519

TRINITY_DN15893_c0_g4_i4	Glyma.09G225600.4	99.56896552	192	18	777	2908	1	2108	0	2519

TRINITY_DN15893_c0_g4_i4	Glyma.09G225600.1	99.57081545	192	18	777	2908	1	2108	0	2519

TRINITY_DN15958_c0_g1_i3	Glyma.13G276800.1	95.23809524	184	24	379	2490	2139	1	0	2634

TRINITY_DN15958_c0_g1_i4	Glyma.13G276800.1	95.73033708	184	24	379	2490	2139	1	0	2634

TRINITY_DN12717_c0_g1_i2	Glyma.19G241300.1	98.64864865	151	8	3	2150	163	2295	0	3011

TRINITY_DN12717_c0_g1_i2	Glyma.19G241300.2	96.60056657	151	9	3	2150	163	2289	0	2972

TRINITY_DN5517_c0_g1_i1	Glyma.13G259700.2	98.7654321	149	3	977	3123	6	2151	0	3099

TRINITY_DN5517_c0_g1_i4	Glyma.13G259700.2	99.00497512	149	3	1054	3200	6	2151	0	3099

TRINITY_DN9119_c0_g1_i1	Glyma.11G248400.1	99.0990991	122	0	233	2382	105	2254	0	3295

TRINITY_DN15964_c0_g1_i5	Glyma.03G113400.1	95.38461538	470	18	280	2420	1	2138	0	1242

TRINITY_DN14043_c0_g2_i3	Glyma.15G096200.11	95.72107765	356	19	251	2371	1	2142	0	1770

TRINITY_DN139_c0_g1_i1	Glyma.18G244700.1	96.72131148	205	9	443	2586	2232	92	0	2748

TRINITY_DN139_c0_g2_i1	Glyma.18G244700.1	96.72897196	205	9	444	2587	2232	92	0	2748

TRINITY_DN12378_c0_g1_i1	Glyma.08G091000.1	98.91304348	191	33	184	2287	8	2129	0	2446

TRINITY_DN9363_c0_g1_i1	Glyma.10G132800.1	95.89041096	179	1	213	2358	2409	261	0	2957

TRINITY_DN15533_c0_g1_i3	Glyma.18G019700.1	99.04534606	169	20	448	2577	1	2127	0	2767

TRINITY_DN14378_c0_g2_i1	Glyma.06G039600.5	99.29577465	163	19	631	2749	669	2787	0	2678

TRINITY_DN14378_c0_g2_i1	Glyma.06G039600.4	95	163	19	631	2749	669	2787	0	2678

TRINITY_DN14378_c0_g2_i1	Glyma.06G039600.2	99.37304075	163	19	631	2749	669	2787	0	2678

TRINITY_DN12717_c0_g1_i2	Glyma.03G244500.1	98.66666667	158	7	3	2150	268	2400	0	2983

TRINITY_DN12717_c0_g1_i2	Glyma.03G244500.10	95.41666667	158	7	3	2150	187	2319	0	2983

TRINITY_DN12717_c0_g1_i2	Glyma.03G244500.12	98.64864865	158	7	3	2150	163	2295	0	2983

TRINITY_DN12717_c0_g1_i2	Glyma.03G244500.11	96.80851064	158	7	3	2150	163	2295	0	2983

TRINITY_DN12717_c0_g1_i2	Glyma.03G244500.13	95.67901235	158	7	3	2150	163	2295	0	2983

TRINITY_DN12717_c0_g1_i2	Glyma.03G244500.9	100	158	7	3	2150	163	2295	0	2983

TRINITY_DN12717_c0_g1_i2	Glyma.03G244500.7	100	158	7	3	2150	163	2295	0	2983

TRINITY_DN12717_c0_g1_i2	Glyma.03G244500.8	98.68995633	158	7	3	2150	163	2295	0	2983

TRINITY_DN12717_c0_g1_i2	Glyma.03G244500.3	98.30508475	158	7	3	2150	163	2295	0	2983

TRINITY_DN12717_c0_g1_i2	Glyma.03G244500.4	98.30508475	158	7	3	2150	163	2295	0	2983

TRINITY_DN12717_c0_g1_i2	Glyma.03G244500.5	98.27586207	158	7	3	2150	163	2295	0	2983

TRINITY_DN12717_c0_g1_i2	Glyma.03G244500.6	95.41463415	158	7	3	2150	163	2295	0	2983

TRINITY_DN12717_c0_g1_i2	Glyma.03G244500.2	95.41547278	158	7	3	2150	163	2295	0	2983

TRINITY_DN12717_c0_g1_i2	Glyma.19G241800.1	96.60056657	156	6	3	2150	163	2292	0	2976

TRINITY_DN12717_c0_g1_i2	Glyma.19G241800.2	99.48186528	156	6	3	2150	31	2160	0	2976

TRINITY_DN16135_c0_g1_i3	Glyma.02G109900.2	98.68421053	149	9	2323	4471	2128	1	0	3007

TRINITY_DN16135_c0_g1_i3	Glyma.02G109900.1	100	149	9	2323	4471	2128	1	0	3007

TRINITY_DN12717_c0_g1_i2	Glyma.03G243800.1	95.59471366	143	6	3	2150	163	2295	0	3066

TRINITY_DN12717_c0_g1_i2	Glyma.03G243800.3	100	143	6	3	2150	163	2295	0	3066

TRINITY_DN15332_c0_g2_i1	Glyma.03G246500.1	95.6043956	142	2	364	2509	2143	1	0	3123

TRINITY_DN15332_c0_g2_i2	Glyma.03G246500.1	98.16849817	142	2	462	2607	2143	1	0	3123

TRINITY_DN15332_c0_g2_i3	Glyma.03G246500.1	100	142	2	225	2370	2143	1	0	3123

TRINITY_DN13225_c0_g1_i1	Glyma.04G237400.3	97.36842105	120	2	163	2310	2148	1	0	3291

TRINITY_DN13225_c0_g1_i1	Glyma.04G237400.2	95.93495935	120	2	163	2310	2148	1	0	3291

TRINITY_DN13225_c0_g1_i1	Glyma.04G237400.1	96.51162791	120	2	163	2310	2148	1	0	3291

TRINITY_DN15617_c0_g2_i2	Glyma.02G225300.1	100	113	10	2	2135	268	2410	0	3206

TRINITY_DN15617_c0_g2_i2	Glyma.02G225300.2	100	113	10	2	2135	268	2410	0	3206

TRINITY_DN15964_c0_g1_i6	Glyma.03G113400.1	98.50746269	477	16	280	2420	1	2138	0	1214

TRINITY_DN14043_c0_g2_i3	Glyma.13G216400.3	95.38461538	367	29	251	2371	1	2130	0	1642

TRINITY_DN14043_c0_g2_i3	Glyma.13G216400.1	100	367	29	251	2371	1	2130	0	1642

TRINITY_DN16245_c0_g3_i1	Glyma.06G196400.1	99.12790698	282	18	1	2134	2472	333	0	2263

TRINITY_DN15958_c0_g1_i2	Glyma.06G242600.3	100	273	15	379	2496	2133	1	0	2163

TRINITY_DN15958_c0_g1_i2	Glyma.06G242600.1	100	273	15	379	2496	2133	1	0	2163

TRINITY_DN15958_c0_g1_i5	Glyma.06G242600.3	95.16129032	273	15	379	2496	2133	1	0	2163

TRINITY_DN15958_c0_g1_i5	Glyma.06G242600.1	96.875	273	15	379	2496	2133	1	0	2163

TRINITY_DN15958_c0_g1_i2	Glyma.06G242600.2	97.6744186	273	18	379	2496	2130	1	0	2145

TRINITY_DN15958_c0_g1_i5	Glyma.06G242600.2	96.875	273	18	379	2496	2130	1	0	2145

TRINITY_DN16245_c0_g3_i3	Glyma.04G166100.1	100	259	20	213	2346	2976	840	0	2370

TRINITY_DN16377_c0_g1_i4	Glyma.11G137700.1	100	240	18	196	2328	1	2136	0	2462

TRINITY_DN4333_c0_g2_i1	Glyma.02G111500.6	100	154	6	321	2465	2139	1	0	3037

TRINITY_DN4333_c0_g2_i1	Glyma.02G111500.1	100	154	6	321	2465	2139	1	0	3037

TRINITY_DN15826_c0_g1_i3	Glyma.06G088500.6	97.44680851	135	7	162	2300	1	2148	0	3160

TRINITY_DN15826_c0_g1_i3	Glyma.06G088500.7	97.44680851	135	7	162	2300	1	2148	0	3160

TRINITY_DN15826_c0_g1_i3	Glyma.06G088500.3	97.44680851	135	7	162	2300	1	2148	0	3160

TRINITY_DN15826_c0_g1_i3	Glyma.06G088500.4	97.44779582	135	7	162	2300	1	2148	0	3160

TRINITY_DN15826_c0_g1_i3	Glyma.06G088500.5	97.44779582	135	7	162	2300	1	2148	0	3160

TRINITY_DN15826_c0_g1_i3	Glyma.06G088500.2	97.44816587	135	7	162	2300	1	2148	0	3160

TRINITY_DN13225_c0_g1_i1	Glyma.06G126600.2	99.59839357	127	0	163	2310	2148	1	0	3264

TRINITY_DN13225_c0_g1_i1	Glyma.06G126600.1	99.2481203	127	0	163	2310	2148	1	0	3264

TRINITY_DN15964_c0_g1_i8	Glyma.03G113400.1	99.18032787	474	14	280	2420	1	2138	0	1242

TRINITY_DN14043_c0_g2_i3	Glyma.15G096200.4	95.72192513	355	22	251	2371	1	2130	0	1720

TRINITY_DN14043_c0_g2_i3	Glyma.15G096200.5	98.7012987	355	22	251	2371	1	2130	0	1720

TRINITY_DN14043_c0_g2_i3	Glyma.15G096200.7	98.00796813	355	22	251	2371	1	2130	0	1720

TRINITY_DN6151_c0_g1_i1	Glyma.06G041000.2	100	355	39	354	2447	1	2124	0	1507

TRINITY_DN6151_c0_g1_i1	Glyma.06G041000.3	100	355	39	354	2447	1	2124	0	1507

TRINITY_DN6151_c0_g1_i1	Glyma.06G041000.1	100	355	39	354	2447	1	2124	0	1507

TRINITY_DN6151_c0_g1_i1	Glyma.06G041000.4	100	355	39	354	2447	1	2124	0	1507

TRINITY_DN6151_c0_g1_i1	Glyma.06G041000.5	98.5042735	355	39	354	2447	1	2124	0	1507

TRINITY_DN16245_c0_g3_i3	Glyma.06G196400.3	100	265	18	213	2346	2469	333	0	2348

TRINITY_DN38405_c0_g1_i1	Glyma.11G138600.1	100	215	15	217	2341	105	2229	0	2490

TRINITY_DN15415_c0_g1_i3	Glyma.02G038500.1	98.95833333	214	2	118	2261	135	2281	0	2760

TRINITY_DN15893_c0_g4_i5	Glyma.12G010800.1	97.6119403	178	23	342	2468	1	2097	0	2527

TRINITY_DN15893_c0_g4_i8	Glyma.12G010800.1	96.35416667	178	23	777	2903	1	2097	0	2527

TRINITY_DN11915_c0_g1_i1	Glyma.18G023200.1	95	157	16	634	2744	2242	98	0	2850

TRINITY_DN7715_c0_g1_i1	Glyma.05G007800.1	95.91836735	149	24	106	2239	1	2107	0	2796

TRINITY_DN7715_c0_g1_i2	Glyma.05G007800.1	100	149	24	103	2236	1	2107	0	2796

TRINITY_DN17087_c0_g1_i1	Glyma.11G253100.1	98.46743295	130	0	1	2147	368	2514	0	3245

TRINITY_DN17087_c0_g1_i1	Glyma.11G253100.2	98.46153846	130	0	1	2147	182	2328	0	3245

TRINITY_DN14961_c1_g1_i2	Glyma.08G266700.6	100	122	1	1123	3269	2823	692	0	3192

TRINITY_DN15958_c0_g1_i2	Glyma.13G276800.1	99.14367269	112	17	379	2496	2139	1	0	3105

TRINITY_DN15958_c0_g1_i5	Glyma.13G276800.1	99.55357143	112	17	379	2496	2139	1	0	3105

TRINITY_DN15964_c0_g1_i7	Glyma.03G113400.1	100	481	12	280	2420	1	2138	0	1214

TRINITY_DN9794_c0_g2_i1	Glyma.04G045800.2	95.62682216	306	20	256	2376	1	2136	0	2041

TRINITY_DN9794_c0_g2_i1	Glyma.04G045800.1	95.625	306	20	256	2376	1	2136	0	2041

TRINITY_DN15385_c0_g1_i2	Glyma.07G119000.2	97.05882353	299	32	1627	3752	2460	350	0	1951

TRINITY_DN15385_c0_g1_i2	Glyma.07G119000.1	99.29577465	299	32	1627	3752	2460	350	0	1951

TRINITY_DN15385_c0_g1_i2	Glyma.07G119000.3	98.36065574	299	32	1627	3752	2460	350	0	1951

TRINITY_DN15619_c0_g2_i3	Glyma.04G184000.1	99.00990099	295	10	912	3043	1	2143	0	2218

TRINITY_DN15619_c0_g2_i3	Glyma.04G184000.3	99.23076923	295	10	912	3043	1	2143	0	2218

TRINITY_DN15385_c0_g1_i5	Glyma.07G119000.2	100	287	29	1571	3705	2460	350	0	2076

TRINITY_DN15385_c0_g1_i5	Glyma.07G119000.1	100	287	29	1571	3705	2460	350	0	2076

TRINITY_DN15385_c0_g1_i5	Glyma.07G119000.3	95.90361446	287	29	1571	3705	2460	350	0	2076

TRINITY_DN16245_c0_g3_i1	Glyma.04G166100.1	100	275	18	1	2134	2976	840	0	2303

TRINITY_DN15736_c0_g1_i3	Glyma.06G162300.1	97.61904762	194	13	296	2428	203	2335	0	2721

TRINITY_DN12378_c0_g1_i1	Glyma.05G135800.1	96.77419355	189	33	195	2289	28	2149	0	2431

TRINITY_DN15533_c0_g1_i3	Glyma.11G237500.3	95.86466165	181	15	448	2577	1	2118	0	2676

TRINITY_DN15533_c0_g1_i3	Glyma.11G237500.1	100	181	15	448	2577	1	2118	0	2676

TRINITY_DN15533_c0_g1_i3	Glyma.11G237500.2	100	180	18	448	2577	1	2115	0	2662

TRINITY_DN6660_c0_g2_i2	Glyma.10G289000.1	100	166	12	191	2324	1	2143	0	2946

TRINITY_DN10664_c0_g1_i2	Glyma.18G212600.1	97.95918367	165	3	170	2311	1	2145	0	3016

TRINITY_DN10664_c0_g1_i2	Glyma.03G131800.1	97.50566893	155	3	170	2311	1	2145	0	3072

TRINITY_DN16069_c0_g1_i1	Glyma.04G213100.1	100	151	9	113	2235	448	2591	0	2964

TRINITY_DN14431_c0_g1_i2	Glyma.17G089100.1	96.05263158	117	6	38	2180	2143	1	0	3277

TRINITY_DN14967_c1_g1_i2	Glyma.11G004300.2	100	106	5	62	2200	1	2126	0	3201

TRINITY_DN14967_c1_g1_i2	Glyma.11G004300.1	100	106	5	62	2200	1	2126	0	3201

TRINITY_DN16245_c0_g3_i3	Glyma.04G144900.1	99.60784314	473	38	213	2345	2940	835	0	1011

TRINITY_DN6151_c0_g1_i1	Glyma.04G039800.2	95.62043796	378	46	354	2447	1	2121	0	1382

TRINITY_DN6151_c0_g1_i1	Glyma.04G039800.3	95.62043796	378	46	354	2447	1	2121	0	1382

TRINITY_DN6151_c0_g1_i1	Glyma.04G039800.4	97.43589744	378	46	354	2447	1	2121	0	1382

TRINITY_DN16245_c0_g3_i1	Glyma.06G196400.3	97.90575916	282	16	1	2134	2469	333	0	2276

TRINITY_DN15893_c0_g4_i5	Glyma.09G225600.5	96.93877551	183	16	342	2468	1	2109	0	2599

TRINITY_DN15893_c0_g4_i5	Glyma.09G225600.4	96.93877551	183	16	342	2468	1	2109	0	2599

TRINITY_DN15893_c0_g4_i5	Glyma.09G225600.1	96.94117647	183	16	342	2468	1	2109	0	2599

TRINITY_DN15893_c0_g4_i8	Glyma.09G225600.5	100	183	16	777	2903	1	2109	0	2599

TRINITY_DN15893_c0_g4_i8	Glyma.09G225600.4	100	183	16	777	2903	1	2109	0	2599

TRINITY_DN15893_c0_g4_i8	Glyma.09G225600.1	100	183	16	777	2903	1	2109	0	2599

TRINITY_DN12719_c0_g2_i1	Glyma.13G321300.49	100	160	20	399	2532	2095	1	0	2680

TRINITY_DN12719_c0_g2_i1	Glyma.13G321300.46	100	160	20	399	2532	2095	1	0	2680

TRINITY_DN12719_c0_g2_i1	Glyma.13G321300.15	95.6185567	160	20	399	2532	2095	1	0	2680

TRINITY_DN12719_c0_g2_i1	Glyma.13G321300.14	96	160	20	399	2532	2095	1	0	2680

TRINITY_DN12719_c0_g2_i1	Glyma.13G321300.50	99.44444444	160	20	399	2532	2095	1	0	2680

TRINITY_DN12719_c0_g2_i1	Glyma.13G321300.47	95.22058824	160	20	399	2532	2095	1	0	2680

TRINITY_DN12719_c0_g2_i1	Glyma.13G321300.16	100	160	20	399	2532	2095	1	0	2680

TRINITY_DN12719_c0_g2_i1	Glyma.13G321300.17	96.875	160	20	399	2532	2095	1	0	2680

TRINITY_DN12719_c0_g2_i2	Glyma.13G321300.49	95.45454545	160	20	500	2633	2095	1	0	2680

TRINITY_DN12719_c0_g2_i2	Glyma.13G321300.46	100	160	20	500	2633	2095	1	0	2680

TRINITY_DN12719_c0_g2_i2	Glyma.13G321300.15	95.14285714	160	20	500	2633	2095	1	0	2680

TRINITY_DN12719_c0_g2_i2	Glyma.13G321300.14	95.14563107	160	20	500	2633	2095	1	0	2680

TRINITY_DN12719_c0_g2_i2	Glyma.13G321300.50	97.26027397	160	20	500	2633	2095	1	0	2680

TRINITY_DN12719_c0_g2_i2	Glyma.13G321300.47	96.89440994	160	20	500	2633	2095	1	0	2680

TRINITY_DN12719_c0_g2_i2	Glyma.13G321300.16	97.26027397	160	20	500	2633	2095	1	0	2680

TRINITY_DN12719_c0_g2_i2	Glyma.13G321300.17	96.55172414	160	20	500	2633	2095	1	0	2680

TRINITY_DN12719_c0_g2_i3	Glyma.13G321300.49	99.3902439	160	20	399	2532	2095	1	0	2680

TRINITY_DN12719_c0_g2_i3	Glyma.13G321300.46	97.75910364	160	20	399	2532	2095	1	0	2680

TRINITY_DN12719_c0_g2_i3	Glyma.13G321300.15	95.63862928	160	20	399	2532	2095	1	0	2680

TRINITY_DN12719_c0_g2_i3	Glyma.13G321300.14	97.24137931	160	20	399	2532	2095	1	0	2680

TRINITY_DN12719_c0_g2_i3	Glyma.13G321300.50	100	160	20	399	2532	2095	1	0	2680

TRINITY_DN12719_c0_g2_i3	Glyma.13G321300.47	99.38650307	160	20	399	2532	2095	1	0	2680

TRINITY_DN12719_c0_g2_i3	Glyma.13G321300.16	97.95918367	160	20	399	2532	2095	1	0	2680

TRINITY_DN12719_c0_g2_i3	Glyma.13G321300.17	98.7012987	160	20	399	2532	2095	1	0	2680

TRINITY_DN12719_c0_g2_i4	Glyma.13G321300.49	98.52941176	160	20	500	2633	2095	1	0	2680

TRINITY_DN12719_c0_g2_i4	Glyma.13G321300.46	99.18699187	160	20	500	2633	2095	1	0	2680

TRINITY_DN12719_c0_g2_i4	Glyma.13G321300.15	99.18699187	160	20	500	2633	2095	1	0	2680

TRINITY_DN12719_c0_g2_i4	Glyma.13G321300.14	99.18699187	160	20	500	2633	2095	1	0	2680

TRINITY_DN12719_c0_g2_i4	Glyma.13G321300.50	99.18699187	160	20	500	2633	2095	1	0	2680

TRINITY_DN12719_c0_g2_i4	Glyma.13G321300.47	99.18699187	160	20	500	2633	2095	1	0	2680

TRINITY_DN12719_c0_g2_i4	Glyma.13G321300.16	98.68421053	160	20	500	2633	2095	1	0	2680

TRINITY_DN12719_c0_g2_i4	Glyma.13G321300.17	97.65258216	160	20	500	2633	2095	1	0	2680

TRINITY_DN12719_c0_g2_i5	Glyma.13G321300.51	95.41666667	160	20	357	2490	2095	1	0	2680

TRINITY_DN12719_c0_g2_i5	Glyma.13G321300.48	95.25547445	160	20	357	2490	2095	1	0	2680

TRINITY_DN12719_c0_g2_i5	Glyma.13G321300.18	95.25547445	160	20	357	2490	2095	1	0	2680

TRINITY_DN12719_c0_g2_i5	Glyma.13G321300.19	100	160	20	357	2490	2095	1	0	2680

TRINITY_DN12719_c0_g2_i5	Glyma.13G321300.52	96.59574468	160	20	357	2490	2095	1	0	2680

TRINITY_DN12719_c0_g2_i5	Glyma.13G321300.1	95.90909091	160	20	357	2490	2095	1	0	2680

TRINITY_DN12719_c0_g2_i5	Glyma.13G321300.20	99.61832061	160	20	357	2490	2095	1	0	2680

TRINITY_DN12719_c0_g2_i5	Glyma.13G321300.21	98.5915493	160	20	357	2490	2095	1	0	2680

TRINITY_DN12719_c0_g2_i7	Glyma.13G321300.51	97.5308642	160	20	357	2490	2095	1	0	2680

TRINITY_DN12719_c0_g2_i7	Glyma.13G321300.48	95.5801105	160	20	357	2490	2095	1	0	2680

TRINITY_DN12719_c0_g2_i7	Glyma.13G321300.18	99.19354839	160	20	357	2490	2095	1	0	2680

TRINITY_DN12719_c0_g2_i7	Glyma.13G321300.19	95.71428571	160	20	357	2490	2095	1	0	2680

TRINITY_DN12719_c0_g2_i7	Glyma.13G321300.52	100	160	20	357	2490	2095	1	0	2680

TRINITY_DN12719_c0_g2_i7	Glyma.13G321300.1	98.03921569	160	20	357	2490	2095	1	0	2680

TRINITY_DN12719_c0_g2_i7	Glyma.13G321300.20	98.35390947	160	20	357	2490	2095	1	0	2680

TRINITY_DN12719_c0_g2_i7	Glyma.13G321300.21	98.7654321	160	20	357	2490	2095	1	0	2680

TRINITY_DN14492_c1_g1_i2	Glyma.03G192300.5	97.2972973	147	1	243	2384	1	2145	0	3127

TRINITY_DN14492_c1_g1_i4	Glyma.03G192300.5	95.65217391	147	1	243	2384	1	2145	0	3127

TRINITY_DN11347_c0_g1_i2	Glyma.10G039200.3	100	138	4	317	2452	2145	1	0	3138

TRINITY_DN11347_c0_g1_i2	Glyma.10G039200.1	96.34703196	138	4	317	2452	2145	1	0	3138

TRINITY_DN11347_c0_g1_i2	Glyma.10G039200.2	96.34920635	138	4	317	2452	2145	1	0	3138

TRINITY_DN11026_c0_g2_i1	Glyma.04G123700.1	100	124	2	270	2411	2145	1	0	3254

TRINITY_DN37638_c0_g1_i1	Glyma.07G003600.2	98.98989899	119	0	440	2584	2145	1	0	3302

TRINITY_DN12659_c1_g1_i1	Glyma.03G026900.3	98.10066477	118	0	235	2379	2187	43	0	3308

TRINITY_DN5134_c0_g1_i1	Glyma.18G203900.2	96.40718563	114	0	1	2145	223	2367	0	3330

TRINITY_DN15964_c0_g1_i5	Glyma.07G113500.1	97.36842105	466	14	250	2387	25	2159	0	1280

TRINITY_DN5668_c0_g1_i1	Glyma.15G076800.1	100	208	14	196	2339	530	2646	0	2632

TRINITY_DN15876_c0_g1_i2	Glyma.06G040600.3	100	199	5	107	2243	1	2143	0	2806

TRINITY_DN15876_c0_g1_i2	Glyma.06G040600.4	98.83449883	199	5	107	2243	1	2143	0	2806

TRINITY_DN15893_c0_g4_i11	Glyma.09G225600.2	95.55555556	192	17	342	2473	1	2102	0	2547

TRINITY_DN15893_c0_g4_i11	Glyma.09G225600.3	98.18181818	192	17	342	2473	1	2102	0	2547

TRINITY_DN15893_c0_g4_i3	Glyma.09G225600.2	98.93617021	192	17	342	2473	1	2102	0	2547

TRINITY_DN15893_c0_g4_i3	Glyma.09G225600.3	98.93617021	192	17	342	2473	1	2102	0	2547

TRINITY_DN15893_c0_g4_i4	Glyma.09G225600.2	96.875	192	17	777	2908	1	2102	0	2547

TRINITY_DN15893_c0_g4_i4	Glyma.09G225600.3	98.52941176	192	17	777	2908	1	2102	0	2547

TRINITY_DN6730_c0_g1_i3	Glyma.01G039000.1	98.80952381	181	5	266	2409	4140	2018	0	2820

TRINITY_DN10168_c0_g1_i2	Glyma.09G202900.1	100	172	5	31	2174	207	2343	0	2961

TRINITY_DN10168_c0_g1_i1	Glyma.09G202900.1	100	170	10	31	2155	207	2343	0	2850

TRINITY_DN9702_c0_g2_i1	Glyma.04G069600.1	95	165	3	191	2313	23	2166	0	2909

TRINITY_DN16182_c0_g1_i2	Glyma.17G027700.1	100	146	3	109	2249	13	2155	0	3125

TRINITY_DN16182_c0_g1_i3	Glyma.17G027700.1	95.80514208	146	3	109	2249	13	2155	0	3125

TRINITY_DN15187_c0_g1_i1	Glyma.08G152400.1	99.53271028	132	5	2638	4769	2144	1	0	3151

TRINITY_DN16382_c0_g3_i3	Glyma.18G060600.3	96.6442953	124	7	177	2320	1	2122	0	3131

TRINITY_DN15964_c0_g1_i6	Glyma.07G113500.1	98.50746269	473	12	250	2387	25	2159	0	1253

TRINITY_DN16377_c0_g1_i4	Glyma.12G061400.1	100	244	19	196	2328	1	2124	0	2418

TRINITY_DN11124_c0_g1_i1	Glyma.10G270200.1	100	229	18	51	2150	1	2124	0	2289

TRINITY_DN16330_c0_g1_i2	Glyma.10G097800.1	95.18716578	223	2	1	2142	2014	4155	0	2710

TRINITY_DN16377_c0_g1_i4	Glyma.12G061200.1	95.77464789	212	14	196	2328	1	2136	0	2673

TRINITY_DN15329_c0_g2_i7	Glyma.02G085000.2	98.5915493	201	18	377	2513	2170	68	0	2547

TRINITY_DN15329_c0_g2_i7	Glyma.02G085000.5	96.34489222	201	18	377	2513	2170	68	0	2547

TRINITY_DN15736_c0_g1_i3	Glyma.04G203200.1	98	189	13	293	2431	173	2302	0	2800

TRINITY_DN16086_c0_g2_i4	Glyma.15G207900.1	99.20948617	476	25	116	2243	23	2150	0	1138

TRINITY_DN15016_c0_g1_i3	Glyma.16G182400.1	97.95918367	354	38	112	2222	856	2964	0	1581

TRINITY_DN15016_c0_g1_i3	Glyma.16G182400.2	98.82352941	354	38	112	2222	541	2649	0	1581

TRINITY_DN13225_c0_g1_i1	Glyma.14G161600.1	98.85057471	289	4	163	2298	2157	16	0	2316

TRINITY_DN13225_c0_g1_i1	Glyma.14G161600.2	100	289	4	163	2298	2157	16	0	2316

TRINITY_DN13975_c0_g1_i1	Glyma.03G140900.3	98.48484848	165	8	1325	3450	9	2149	0	2931

TRINITY_DN13975_c0_g1_i1	Glyma.03G140900.1	98.98989899	165	8	1325	3450	9	2149	0	2931

TRINITY_DN13975_c0_g1_i1	Glyma.03G140900.2	100	165	8	1325	3450	9	2149	0	2931

TRINITY_DN13975_c0_g1_i2	Glyma.03G140900.3	96.34551495	165	8	284	2409	9	2149	0	2931

TRINITY_DN13975_c0_g1_i2	Glyma.03G140900.1	100	165	8	284	2409	9	2149	0	2931

TRINITY_DN13975_c0_g1_i2	Glyma.03G140900.2	100	165	8	284	2409	9	2149	0	2931

TRINITY_DN16330_c0_g1_i2	Glyma.03G137000.1	95.18900344	156	0	1	2142	2014	4155	0	3092

TRINITY_DN8994_c0_g1_i1	Glyma.12G227800.1	100	155	17	48	2171	7	2136	0	2904

TRINITY_DN16330_c0_g1_i2	Glyma.19G139300.1	95.16129032	152	0	1	2142	2011	4152	0	3114

TRINITY_DN13676_c0_g1_i1	Glyma.15G111600.1	96.93251534	135	5	221	2362	1	2133	0	3149

TRINITY_DN13526_c0_g1_i1	Glyma.11G027200.1	97.61904762	130	19	176	2301	65	2172	0	2913

TRINITY_DN15617_c0_g2_i2	Glyma.14G192100.1	100	116	7	2	2137	256	2388	0	3216

TRINITY_DN15529_c1_g1_i2	Glyma.05G232400.10	100	292	10	20	2149	2151	16	0	2233

TRINITY_DN15529_c1_g1_i4	Glyma.05G232400.10	100	292	10	20	2149	2151	16	0	2233

TRINITY_DN39405_c0_g1_i1	Glyma.10G242300.1	100	249	6	215	2352	2968	831	0	2536

TRINITY_DN10900_c0_g1_i1	Glyma.02G095100.1	98.92473118	212	24	899	3005	2122	1	0	2436

TRINITY_DN2614_c0_g1_i1	Glyma.08G012600.1	96.19047619	206	22	164	2272	64	2169	0	2379

TRINITY_DN15329_c0_g2_i4	Glyma.02G085000.3	100	198	23	377	2499	2182	68	0	2573

TRINITY_DN15329_c0_g2_i4	Glyma.02G085000.1	98.86363636	198	23	377	2499	2182	68	0	2573

TRINITY_DN13698_c1_g1_i1	Glyma.15G154900.4	99.25925926	196	12	791	2896	2133	1	0	2590

TRINITY_DN13698_c1_g1_i1	Glyma.15G154900.1	99.2481203	196	12	791	2896	2133	1	0	2590

TRINITY_DN13698_c1_g1_i3	Glyma.15G154900.4	100	196	12	872	2977	2133	1	0	2590

TRINITY_DN13698_c1_g1_i3	Glyma.15G154900.1	100	196	12	872	2977	2133	1	0	2590

TRINITY_DN16158_c0_g1_i6	Glyma.20G001300.3	96.54255319	188	4	1032	3171	2799	685	0	2737

TRINITY_DN19948_c0_g1_i1	Glyma.11G040000.1	96.67590028	114	0	270	2410	4356	2216	0	3323

TRINITY_DN19948_c0_g1_i1	Glyma.11G040000.2	100	114	0	270	2410	4347	2207	0	3323

TRINITY_DN19948_c0_g1_i1	Glyma.11G040000.3	96.66666667	114	0	270	2410	4347	2207	0	3323

TRINITY_DN19948_c0_g1_i1	Glyma.11G040000.8	96.66666667	114	0	270	2410	4347	2207	0	3323

TRINITY_DN19948_c0_g1_i1	Glyma.11G040000.11	100	114	0	270	2410	3603	1463	0	3323

TRINITY_DN19948_c0_g1_i1	Glyma.01G203000.1	100	112	0	270	2410	4359	2219	0	3334

TRINITY_DN19948_c0_g1_i1	Glyma.01G203000.3	100	112	0	270	2410	4359	2219	0	3334

TRINITY_DN19948_c0_g1_i1	Glyma.01G203000.2	100	112	0	270	2410	4359	2219	0	3334

TRINITY_DN10975_c0_g1_i2	Glyma.08G225500.3	98.95013123	110	1	363	2503	1	2133	0	3293

TRINITY_DN13097_c0_g1_i3	Glyma.18G094400.1	98.57142857	355	43	169	2263	2129	22	0	1487

TRINITY_DN16245_c0_g3_i6	Glyma.02G129500.1	98.41269841	303	6	262	2399	3596	1464	0	2215

TRINITY_DN16245_c0_g3_i6	Glyma.02G129500.2	98.95833333	303	6	262	2399	3221	1089	0	2215

TRINITY_DN15958_c0_g1_i3	Glyma.06G242600.3	100	225	9	379	2490	2133	1	0	2479

TRINITY_DN15958_c0_g1_i3	Glyma.06G242600.1	96.55172414	225	9	379	2490	2133	1	0	2479

TRINITY_DN15958_c0_g1_i4	Glyma.06G242600.3	97.6	225	9	379	2490	2133	1	0	2479

TRINITY_DN15958_c0_g1_i4	Glyma.06G242600.1	98.68421053	225	9	379	2490	2133	1	0	2479

TRINITY_DN15958_c0_g1_i3	Glyma.06G242600.2	100	225	11	379	2490	2130	1	0	2460

TRINITY_DN15958_c0_g1_i4	Glyma.06G242600.2	98.68421053	225	11	379	2490	2130	1	0	2460

TRINITY_DN11347_c0_g1_i2	Glyma.13G125600.4	97.75280899	145	3	317	2452	2139	1	0	3116

TRINITY_DN11347_c0_g1_i2	Glyma.13G125600.3	97.38219895	145	3	317	2452	2139	1	0	3116

TRINITY_DN11347_c0_g1_i2	Glyma.13G125600.1	97.38219895	145	3	317	2452	2139	1	0	3116

TRINITY_DN11347_c0_g1_i2	Glyma.13G125600.2	100	145	3	317	2452	2139	1	0	3116

TRINITY_DN9349_c0_g1_i1	Glyma.19G025900.2	100	145	3	232	2367	1	2139	0	3116

TRINITY_DN9349_c0_g1_i1	Glyma.19G025900.1	100	145	3	232	2367	1	2139	0	3116

TRINITY_DN9028_c0_g1_i2	Glyma.07G186700.1	96.71641791	189	16	1	2129	248	2364	0	2697

TRINITY_DN15529_c1_g1_i1	Glyma.05G232400.10	99.25925926	186	3	568	2703	2151	16	0	2881

TRINITY_DN15893_c0_g4_i5	Glyma.09G225600.2	100	183	15	342	2468	1	2103	0	2627

TRINITY_DN15893_c0_g4_i5	Glyma.09G225600.3	98.11320755	183	15	342	2468	1	2103	0	2627

TRINITY_DN15893_c0_g4_i8	Glyma.09G225600.2	98.17629179	183	15	777	2903	1	2103	0	2627

TRINITY_DN15893_c0_g4_i8	Glyma.09G225600.3	98.18181818	183	15	777	2903	1	2103	0	2627

TRINITY_DN12996_c0_g1_i1	Glyma.20G066600.1	96.11650485	172	0	658	2796	2139	1	0	2998

TRINITY_DN11749_c0_g1_i3	Glyma.06G002400.4	100	171	2	1	2139	299	2431	0	2964

TRINITY_DN11749_c0_g1_i4	Glyma.06G002400.4	96.32653061	171	2	1	2139	299	2431	0	2964

TRINITY_DN11749_c0_g1_i3	Glyma.06G002400.3	95.61643836	169	4	1	2139	299	2425	0	2937

TRINITY_DN11749_c0_g1_i4	Glyma.06G002400.3	96.69421488	169	4	1	2139	299	2425	0	2937

TRINITY_DN12996_c0_g1_i1	Glyma.13G002500.1	100	160	0	658	2796	2139	1	0	3064

TRINITY_DN12996_c0_g1_i1	Glyma.13G002500.2	100	160	0	658	2796	2139	1	0	3064

TRINITY_DN15970_c0_g2_i11	Glyma.03G069400.1	95.12195122	160	3	80	2212	1	2139	0	3025

TRINITY_DN17020_c0_g2_i1	Glyma.11G229000.1	95.71045576	159	3	846	2982	2417	281	0	3044

TRINITY_DN2153_c0_g1_i1	Glyma.15G048100.1	100	148	7	1	2139	4354	6480	0	3053

TRINITY_DN2153_c0_g1_i1	Glyma.15G048100.2	100	148	7	1	2139	4351	6477	0	3053

TRINITY_DN16182_c0_g1_i2	Glyma.07G246300.1	99.01960784	138	0	110	2248	16	2154	0	3186

TRINITY_DN16182_c0_g1_i3	Glyma.07G246300.1	95.8041958	138	0	110	2248	16	2154	0	3186

TRINITY_DN14967_c1_g1_i2	Glyma.01G239200.1	100	129	1	62	2200	1	2120	0	3112

TRINITY_DN10575_c0_g1_i1	Glyma.15G236500.1	100	105	7	47	2185	16	2142	0	3291

TRINITY_DN10575_c0_g1_i1	Glyma.15G236500.2	100	105	7	47	2185	13	2139	0	3291

TRINITY_DN15016_c0_g1_i3	Glyma.09G137000.1	97.36842105	352	31	115	2222	862	2973	0	1637

TRINITY_DN15016_c0_g1_i3	Glyma.09G137000.2	98.55072464	352	31	115	2222	52	2163	0	1637

TRINITY_DN15750_c1_g1_i6	Glyma.13G104000.1	99.36708861	309	11	550	2681	2184	53	0	2159

TRINITY_DN16245_c0_g3_i4	Glyma.01G092900.1	100	282	5	262	2397	3596	1466	0	2327

TRINITY_DN16245_c0_g3_i4	Glyma.01G092900.2	98.98477157	282	5	262	2397	3221	1091	0	2327

TRINITY_DN16245_c0_g3_i4	Glyma.01G092900.3	96.2962963	282	5	262	2397	3221	1091	0	2327

TRINITY_DN15750_c1_g1_i7	Glyma.13G104000.1	98.4	258	11	550	2681	2184	53	0	2442

TRINITY_DN39405_c0_g1_i1	Glyma.20G151800.5	95.94356261	239	0	215	2352	2968	831	0	2625

TRINITY_DN39405_c0_g1_i1	Glyma.20G151800.4	95.42253521	239	0	215	2352	2896	759	0	2625

TRINITY_DN15970_c0_g1_i3	Glyma.02G251100.1	95.74468085	190	2	137	2273	6	2142	0	2883

TRINITY_DN15970_c0_g1_i3	Glyma.14G065600.1	98.35294118	188	2	137	2273	6	2142	0	2894

TRINITY_DN9794_c0_g2_i1	Glyma.17G235000.5	95.625	165	4	256	2388	1	2136	0	2988

TRINITY_DN9794_c0_g2_i1	Glyma.17G235000.1	95.65217391	165	4	256	2388	1	2136	0	2988

TRINITY_DN14563_c0_g1_i1	Glyma.18G218900.2	96.55172414	131	21	1467	3583	2881	762	0	2970

TRINITY_DN14563_c0_g1_i3	Glyma.18G218900.2	98.66666667	131	21	1467	3583	2881	762	0	2970

TRINITY_DN15565_c0_g1_i3	Glyma.17G027200.1	98.18181818	127	13	514	2638	1	2128	0	3096

TRINITY_DN15565_c0_g1_i4	Glyma.17G027200.1	97.72727273	127	13	527	2651	1	2128	0	3096

TRINITY_DN15970_c0_g1_i4	Glyma.02G251100.1	98.7012987	123	2	137	2273	6	2142	0	3254

TRINITY_DN16382_c0_g3_i4	Glyma.18G060600.3	96.6442953	123	4	177	2314	1	2122	0	3164

TRINITY_DN15750_c1_g1_i6	Glyma.17G055100.1	100	311	9	550	2681	2184	53	0	2159

TRINITY_DN16245_c0_g3_i6	Glyma.01G092900.1	100	299	7	262	2395	3596	1468	0	2218

TRINITY_DN16245_c0_g3_i6	Glyma.01G092900.2	98.60465116	299	7	262	2395	3221	1093	0	2218

TRINITY_DN16245_c0_g3_i6	Glyma.01G092900.3	99.85465116	299	7	262	2395	3221	1093	0	2218

TRINITY_DN15501_c0_g2_i6	Glyma.13G162800.1	98.50746269	269	2	1	2136	2136	1	0	2444

TRINITY_DN15750_c1_g1_i7	Glyma.17G055100.1	100	265	9	550	2681	2184	53	0	2414

TRINITY_DN16163_c0_g1_i2	Glyma.06G303700.1	100	252	19	699	2819	68	2189	0	2350

TRINITY_DN16163_c0_g1_i5	Glyma.06G303700.1	98.7012987	252	19	208	2328	68	2189	0	2350

TRINITY_DN16163_c0_g1_i6	Glyma.06G303700.1	100	252	19	677	2797	68	2189	0	2350

TRINITY_DN16332_c0_g3_i5	Glyma.17G239500.5	100	233	8	298	2427	3093	961	0	2584

TRINITY_DN16332_c0_g3_i5	Glyma.17G239500.1	100	233	8	298	2427	3093	961	0	2584

TRINITY_DN15815_c0_g2_i5	Glyma.12G147000.1	97.91666667	233	15	2470	4587	2133	1	0	2507

TRINITY_DN15815_c0_g2_i6	Glyma.12G147000.1	98.95833333	233	15	1909	4026	2133	1	0	2507

TRINITY_DN15815_c0_g2_i5	Glyma.12G147000.3	97.91666667	231	18	2470	4587	2130	1	0	2499

TRINITY_DN15815_c0_g2_i6	Glyma.12G147000.3	100	231	18	1909	4026	2130	1	0	2499

TRINITY_DN15329_c0_g2_i3	Glyma.02G085000.3	95	197	24	377	2495	2182	68	0	2597

TRINITY_DN15329_c0_g2_i3	Glyma.02G085000.1	95	197	24	377	2495	2182	68	0	2597

TRINITY_DN8367_c0_g1_i3	Glyma.14G193900.1	98	185	13	138	2272	3283	1164	0	2798

TRINITY_DN15958_c0_g1_i3	Glyma.12G224500.1	96.42857143	179	17	379	2490	2127	1	0	2728

TRINITY_DN15958_c0_g1_i4	Glyma.12G224500.1	100	179	17	379	2490	2127	1	0	2728

TRINITY_DN12719_c0_g2_i2	Glyma.13G321300.44	100	162	20	458	2583	2092	6	0	2654

TRINITY_DN12719_c0_g2_i2	Glyma.13G321300.32	100	162	20	458	2583	2092	6	0	2654

TRINITY_DN12719_c0_g2_i2	Glyma.13G321300.12	96.62921348	162	20	458	2583	2092	6	0	2654

TRINITY_DN12719_c0_g2_i2	Glyma.13G321300.45	98.66666667	162	20	458	2583	2092	6	0	2654

TRINITY_DN12719_c0_g2_i2	Glyma.13G321300.33	100	162	20	458	2583	2092	6	0	2654

TRINITY_DN12719_c0_g2_i2	Glyma.13G321300.13	100	162	20	458	2583	2092	6	0	2654

TRINITY_DN12719_c0_g2_i4	Glyma.13G321300.44	96.81528662	162	20	458	2583	2092	6	0	2654

TRINITY_DN12719_c0_g2_i4	Glyma.13G321300.32	100	162	20	458	2583	2092	6	0	2654

TRINITY_DN12719_c0_g2_i4	Glyma.13G321300.12	100	162	20	458	2583	2092	6	0	2654

TRINITY_DN12719_c0_g2_i4	Glyma.13G321300.45	97.23865878	162	20	458	2583	2092	6	0	2654

TRINITY_DN12719_c0_g2_i4	Glyma.13G321300.33	96.51741294	162	20	458	2583	2092	6	0	2654

TRINITY_DN12719_c0_g2_i4	Glyma.13G321300.13	96.13259669	162	20	458	2583	2092	6	0	2654

TRINITY_DN8994_c0_g1_i1	Glyma.13G272300.1	100	154	14	48	2170	7	2138	0	2970

TRINITY_DN15970_c0_g1_i4	Glyma.14G065600.1	100	120	0	137	2273	6	2142	0	3282

TRINITY_DN10873_c0_g1_i2	Glyma.04G017700.2	99.25373134	119	4	263	2395	2138	2	0	3262

TRINITY_DN16443_c1_g1_i2	Glyma.16G171500.1	100	385	30	7	2122	3134	1013	0	1592

TRINITY_DN16443_c1_g1_i2	Glyma.16G106200.1	96.21212121	366	30	7	2122	3203	1082	0	1698

TRINITY_DN9028_c0_g1_i2	Glyma.13G251100.1	96.68874172	329	22	1	2120	269	2391	0	1934

TRINITY_DN14967_c1_g1_i2	Glyma.10G250100.2	100	242	15	62	2188	1	2109	0	2372

TRINITY_DN14967_c1_g1_i2	Glyma.10G250100.1	100	242	15	62	2188	1	2109	0	2372

TRINITY_DN15310_c0_g1_i1	Glyma.12G237300.2	97.8021978	215	16	356	2476	1	2115	0	2521

TRINITY_DN15310_c0_g1_i1	Glyma.12G237300.1	97.8021978	215	16	356	2476	1	2115	0	2521

TRINITY_DN15310_c0_g1_i3	Glyma.12G237300.2	95.23809524	215	16	227	2347	1	2115	0	2521

TRINITY_DN15310_c0_g1_i3	Glyma.12G237300.1	98.71794872	215	16	227	2347	1	2115	0	2521

TRINITY_DN10755_c0_g1_i2	Glyma.17G245600.2	97.56097561	204	3	459	2585	1	2136	0	2756

TRINITY_DN15893_c0_g4_i11	Glyma.12G010900.1	95.55555556	196	10	342	2473	1	2108	0	2652

TRINITY_DN15893_c0_g4_i3	Glyma.12G010900.1	98.93617021	196	10	342	2473	1	2108	0	2652

TRINITY_DN15893_c0_g4_i4	Glyma.12G010900.1	96.875	196	10	777	2908	1	2108	0	2652

TRINITY_DN9028_c0_g1_i2	Glyma.07G186700.2	96.70658683	189	15	1	2129	248	2361	0	2710

TRINITY_DN9028_c0_g1_i2	Glyma.08G062500.1	96.71361502	189	16	1	2129	260	2373	0	2710

TRINITY_DN12786_c0_g1_i1	Glyma.17G001700.1	100	173	7	24	2159	1	2112	0	2832

TRINITY_DN12786_c0_g1_i1	Glyma.17G001700.2	100	172	8	24	2159	1	2109	0	2817

TRINITY_DN15970_c0_g2_i11	Glyma.01G099800.1	95.12195122	157	2	80	2212	1	2136	0	3055

TRINITY_DN9794_c0_g2_i1	Glyma.U031200.1	95.64502876	149	4	256	2388	1	2133	0	3081

TRINITY_DN9794_c0_g2_i1	Glyma.U031200.2	95.65217391	149	4	256	2388	1	2133	0	3081

TRINITY_DN9794_c0_g2_i1	Glyma.U031200.3	95.62982005	149	4	256	2388	1	2133	0	3081

TRINITY_DN16163_c0_g1_i1	Glyma.12G100100.1	95.0877193	149	6	657	2782	150	2284	0	3048

TRINITY_DN16163_c0_g1_i3	Glyma.12G100100.1	98.9010989	149	6	679	2804	150	2284	0	3048

TRINITY_DN16163_c0_g1_i4	Glyma.12G100100.1	99.47089947	149	6	188	2313	150	2284	0	3048

TRINITY_DN16163_c0_g1_i1	Glyma.12G100100.2	96.92874693	149	6	657	2782	48	2182	0	3048

TRINITY_DN16163_c0_g1_i1	Glyma.12G100100.3	96.92923899	149	6	657	2782	48	2182	0	3048

TRINITY_DN16163_c0_g1_i3	Glyma.12G100100.2	99.50738916	149	6	679	2804	48	2182	0	3048

TRINITY_DN16163_c0_g1_i3	Glyma.12G100100.3	98.11827957	149	6	679	2804	48	2182	0	3048

TRINITY_DN16163_c0_g1_i4	Glyma.12G100100.2	100	149	6	188	2313	48	2182	0	3048

TRINITY_DN16163_c0_g1_i4	Glyma.12G100100.3	95.2991453	149	6	188	2313	48	2182	0	3048

TRINITY_DN14973_c1_g2_i5	Glyma.09G031400.2	100	116	4	512	2647	2127	1	0	3243

TRINITY_DN14973_c1_g2_i5	Glyma.09G031400.3	96.99074074	116	4	512	2647	2127	1	0	3243

TRINITY_DN14973_c1_g2_i5	Glyma.09G031400.1	100	116	4	512	2647	2127	1	0	3243

TRINITY_DN14973_c1_g2_i6	Glyma.09G031400.2	100	116	4	512	2647	2127	1	0	3243

TRINITY_DN14973_c1_g2_i6	Glyma.09G031400.3	100	116	4	512	2647	2127	1	0	3243

TRINITY_DN14973_c1_g2_i6	Glyma.09G031400.1	100	116	4	512	2647	2127	1	0	3243

TRINITY_DN10575_c0_g1_i1	Glyma.13G198200.3	100	115	6	50	2185	19	2139	0	3210

TRINITY_DN16071_c0_g2_i8	Glyma.08G087300.1	95.65217391	341	26	200	2313	127	2252	0	1860

TRINITY_DN15958_c0_g1_i2	Glyma.12G155400.2	99.04761905	286	17	389	2496	2123	1	0	2106

TRINITY_DN15958_c0_g1_i5	Glyma.12G155400.2	95	286	17	389	2496	2123	1	0	2106

TRINITY_DN15774_c0_g1_i2	Glyma.04G086000.1	97.39130435	242	15	1424	3529	2133	1	0	2401

TRINITY_DN15774_c0_g1_i3	Glyma.04G086000.1	96	242	15	2269	4374	2133	1	0	2401

TRINITY_DN14043_c0_g2_i3	Glyma.17G024000.1	98.80952381	179	8	251	2377	1	2127	0	2848

TRINITY_DN36371_c0_g1_i2	Glyma.08G082600.2	99.07407407	154	1	1	2132	839	2973	0	3070

TRINITY_DN36371_c0_g1_i2	Glyma.08G082600.3	99.21259843	154	1	1	2132	839	2973	0	3070

TRINITY_DN36371_c0_g1_i2	Glyma.08G082600.1	99.21259843	154	1	1	2132	839	2973	0	3070

TRINITY_DN13349_c1_g3_i2	Glyma.08G309000.1	99.15878023	258	17	716	2825	2198	83	0	2241

TRINITY_DN16332_c0_g3_i5	Glyma.14G085200.1	100	245	6	298	2427	3090	961	0	2532

TRINITY_DN16332_c0_g3_i5	Glyma.14G085200.2	100	245	6	298	2427	2901	772	0	2532

TRINITY_DN16332_c0_g3_i5	Glyma.17G239500.3	100	234	6	298	2427	3090	961	0	2593

TRINITY_DN16332_c0_g3_i5	Glyma.17G239500.4	100	234	6	298	2427	3090	961	0	2593

TRINITY_DN13152_c0_g1_i2	Glyma.02G222900.1	95.82577132	196	20	976	3098	2102	1	0	2577

TRINITY_DN16245_c0_g3_i1	Glyma.16G099700.2	100	192	0	1	2134	2973	840	0	2878

TRINITY_DN16245_c0_g3_i1	Glyma.16G099700.1	98.18181818	192	0	1	2134	2973	840	0	2878

TRINITY_DN16245_c0_g3_i1	Glyma.08G268900.1	95.52238806	191	0	1	2134	2976	843	0	2883

TRINITY_DN16425_c0_g1_i2	Glyma.11G137000.1	97.75280899	186	19	1524	3652	2111	1	0	2730

TRINITY_DN16425_c0_g1_i5	Glyma.11G137000.1	97.36842105	186	19	1524	3652	2111	1	0	2730

TRINITY_DN16245_c0_g3_i3	Glyma.16G099700.2	95.95959596	134	0	213	2346	2973	840	0	3199

TRINITY_DN16245_c0_g3_i3	Glyma.16G099700.1	97.39970282	134	0	213	2346	2973	840	0	3199

TRINITY_DN16245_c0_g3_i3	Glyma.08G268900.1	98.41269841	132	0	213	2346	2976	843	0	3210

TRINITY_DN9476_c0_g1_i4	Glyma.07G263100.1	96.55172414	122	1	334	2467	2919	806	0	3136

TRINITY_DN15958_c0_g1_i2	Glyma.12G224500.1	99.14367269	110	10	379	2496	2127	1	0	3182

TRINITY_DN15958_c0_g1_i5	Glyma.12G224500.1	96.81528662	110	10	379	2496	2127	1	0	3182

TRINITY_DN15293_c0_g1_i3	Glyma.11G179600.2	98.97435897	329	46	565	2654	42	2140	0	1618

TRINITY_DN16217_c2_g1_i1	Glyma.13G007200.1	95.83333333	313	13	158	2283	2127	5	0	2095

TRINITY_DN16217_c2_g1_i10	Glyma.13G007200.1	98.50746269	313	13	158	2283	2127	5	0	2095

TRINITY_DN15293_c0_g1_i2	Glyma.11G179600.2	95.38461538	312	46	628	2717	42	2140	0	1712

TRINITY_DN15276_c0_g1_i4	Glyma.08G001200.2	100	225	12	688	2809	407	2529	0	2556

TRINITY_DN15276_c0_g1_i4	Glyma.08G001200.1	100	225	12	688	2809	407	2529	0	2556

TRINITY_DN8360_c0_g2_i1	Glyma.18G085500.1	99.09090909	204	1	248	2380	1	2130	0	2789

TRINITY_DN15098_c1_g1_i2	Glyma.06G049400.1	100	201	14	283	2394	1	2121	0	2612

TRINITY_DN13407_c0_g1_i1	Glyma.20G120700.1	99.10714286	163	0	319	2451	2133	1	0	3037

TRINITY_DN15276_c0_g1_i2	Glyma.08G001200.2	100	159	12	688	2809	407	2529	0	2922

TRINITY_DN15276_c0_g1_i2	Glyma.08G001200.1	98.41269841	159	12	688	2809	407	2529	0	2922

TRINITY_DN8503_c0_g1_i1	Glyma.09G164700.2	99.10313901	139	2	318	2447	2133	1	0	3149

TRINITY_DN8503_c0_g1_i3	Glyma.09G164700.2	97.43589744	139	2	329	2458	2133	1	0	3149

TRINITY_DN8503_c0_g1_i5	Glyma.09G164700.2	96.53679654	139	2	318	2447	2133	1	0	3149

TRINITY_DN8503_c0_g1_i6	Glyma.09G164700.2	99.1902834	139	2	329	2458	2133	1	0	3149

TRINITY_DN14961_c1_g1_i1	Glyma.08G266700.6	97.05882353	122	1	544	2676	2809	692	0	3166

TRINITY_DN14961_c1_g1_i2	Glyma.08G266700.2	100	122	1	1137	3269	2809	692	0	3166

TRINITY_DN14961_c1_g1_i2	Glyma.08G266700.1	100	122	1	1137	3269	2809	692	0	3166

TRINITY_DN14961_c1_g1_i2	Glyma.08G266700.3	100	122	2	1137	3269	2806	692	0	3147

TRINITY_DN9028_c0_g1_i2	Glyma.15G063200.1	96.71052632	342	12	1	2120	272	2394	0	1906

TRINITY_DN16071_c0_g2_i8	Glyma.05G132900.1	99.21875	339	24	203	2313	130	2252	0	1866

TRINITY_DN11610_c0_g2_i1	Glyma.16G162200.2	98.57142857	283	16	1	2128	204	2316	0	2220

TRINITY_DN13198_c0_g1_i1	Glyma.20G180800.1	97.71167048	207	5	73	2203	26	2150	0	2739

TRINITY_DN14596_c0_g1_i1	Glyma.20G174900.7	98.68421053	167	27	34	2134	1	2108	0	2656

TRINITY_DN9702_c0_g2_i1	Glyma.06G071300.4	95	162	6	191	2313	23	2151	0	2963

TRINITY_DN9702_c0_g2_i1	Glyma.06G071300.3	95	162	6	191	2313	23	2151	0	2963

TRINITY_DN9702_c0_g2_i1	Glyma.06G071300.1	95	162	6	191	2313	23	2151	0	2963

TRINITY_DN36371_c0_g1_i2	Glyma.05G127800.1	97.02970297	149	0	1	2132	839	2970	0	3112

TRINITY_DN7715_c0_g1_i1	Glyma.05G007800.2	95.62043796	148	24	121	2239	1	2092	0	2774

TRINITY_DN7715_c0_g1_i2	Glyma.05G007800.2	99.16666667	148	24	118	2236	1	2092	0	2774

TRINITY_DN14961_c1_g1_i2	Glyma.16G102300.1	97.05882353	122	1	1137	3268	2836	708	0	3241

TRINITY_DN14961_c1_g1_i2	Glyma.16G102300.2	97.04142012	122	2	1137	3268	2833	708	0	3223

TRINITY_DN16086_c0_g2_i1	Glyma.15G207900.1	98.18181818	479	24	296	2414	32	2150	0	1127

TRINITY_DN14216_c0_g1_i2	Glyma.19G159400.7	100	306	4	304	2433	1	2127	0	2207

TRINITY_DN14216_c0_g1_i2	Glyma.19G159400.8	100	306	4	304	2433	1	2127	0	2207

TRINITY_DN10955_c0_g1_i1	Glyma.09G233500.1	98.53658537	219	12	188	2308	2127	1	0	2632

TRINITY_DN15994_c0_g1_i1	Glyma.13G367300.3	97.82608696	214	4	893	3013	2961	835	0	2660

TRINITY_DN15994_c0_g1_i1	Glyma.13G367300.2	100	214	4	893	3013	2961	835	0	2660

TRINITY_DN15024_c0_g1_i1	Glyma.06G321000.1	97	193	48	295	2355	2097	1	0	2194

TRINITY_DN15024_c0_g1_i2	Glyma.06G321000.1	98.70689655	193	48	463	2523	2097	1	0	2194

TRINITY_DN15893_c0_g4_i5	Glyma.12G010900.1	98.71794872	184	8	342	2468	1	2109	0	2748

TRINITY_DN15893_c0_g4_i8	Glyma.12G010900.1	98.17518248	184	8	777	2903	1	2109	0	2748

TRINITY_DN14641_c2_g2_i2	Glyma.13G112600.1	97.81818182	138	20	95	2192	2107	1	0	2802

TRINITY_DN9119_c0_g1_i1	Glyma.11G248400.8	98.93617021	122	0	252	2382	1	2131	0	3260

TRINITY_DN9119_c0_g1_i1	Glyma.11G248400.6	95.62982005	122	0	252	2382	1	2131	0	3260

TRINITY_DN9119_c0_g1_i1	Glyma.11G248400.7	95.91194969	122	0	252	2382	1	2131	0	3260

TRINITY_DN9119_c0_g1_i1	Glyma.11G248400.5	96.13733906	122	0	252	2382	1	2131	0	3260

TRINITY_DN9119_c0_g1_i1	Glyma.11G248400.3	96.13733906	122	0	252	2382	1	2131	0	3260

TRINITY_DN9119_c0_g1_i1	Glyma.11G248400.4	100	122	0	252	2382	1	2131	0	3260

TRINITY_DN9119_c0_g1_i1	Glyma.11G248400.2	97.87234043	122	0	252	2382	1	2131	0	3260

TRINITY_DN15565_c0_g1_i3	Glyma.07G246800.2	95.5654102	122	4	514	2638	1	2125	0	3182

TRINITY_DN15565_c0_g1_i3	Glyma.07G246800.1	100	122	4	514	2638	1	2125	0	3182

TRINITY_DN15565_c0_g1_i4	Glyma.07G246800.2	100	122	4	527	2651	1	2125	0	3182

TRINITY_DN15565_c0_g1_i4	Glyma.07G246800.1	97.72727273	122	4	527	2651	1	2125	0	3182

TRINITY_DN12047_c0_g1_i1	Glyma.16G041500.1	95.38461538	114	4	589	2715	2130	1	0	3271

TRINITY_DN15007_c0_g1_i4	Glyma.18G015100.1	100	281	14	286	2404	2113	1	0	2196

TRINITY_DN15007_c0_g1_i5	Glyma.18G015100.1	96.875	281	14	286	2404	2113	1	0	2196

TRINITY_DN16155_c1_g1_i3	Glyma.11G145600.1	100	232	11	1	2108	2129	1	0	2499

TRINITY_DN16082_c0_g1_i6	Glyma.08G202000.5	95.93495935	204	10	552	2649	2128	1	0	2584

TRINITY_DN15007_c0_g1_i3	Glyma.18G015100.1	97.84482759	191	14	286	2404	2113	1	0	2695

TRINITY_DN15007_c0_g1_i9	Glyma.18G015100.1	100	191	14	286	2404	2113	1	0	2695

TRINITY_DN13654_c0_g1_i2	Glyma.19G261700.1	98.11320755	176	6	461	2586	2142	20	0	2887

TRINITY_DN13448_c0_g1_i1	Glyma.08G100800.3	96.44351464	167	7	1	2122	27	2148	0	2905

TRINITY_DN13448_c0_g1_i6	Glyma.08G100800.3	98.41269841	167	7	1	2122	27	2148	0	2905

TRINITY_DN11201_c0_g1_i2	Glyma.17G079400.1	96.23655914	133	7	319	2415	1	2127	0	2964

TRINITY_DN11201_c0_g1_i3	Glyma.17G079400.1	95.93023256	133	7	196	2292	1	2127	0	2964

TRINITY_DN16332_c0_g3_i5	Glyma.06G042400.1	100	127	2	298	2427	3072	958	0	3133

TRINITY_DN16332_c0_g3_i5	Glyma.06G042400.2	100	127	2	298	2427	2886	772	0	3133

TRINITY_DN16332_c0_g3_i5	Glyma.04G041300.1	100	121	1	298	2427	3084	958	0	3243

TRINITY_DN16332_c0_g3_i5	Glyma.04G041300.2	100	121	1	298	2427	2898	772	0	3243

TRINITY_DN15396_c0_g1_i2	Glyma.03G204800.1	99.82142857	313	25	399	2509	2209	99	0	1965

TRINITY_DN14967_c1_g1_i2	Glyma.20G143500.1	100	249	18	62	2182	1	2102	0	2326

TRINITY_DN15958_c0_g1_i3	Glyma.12G155400.2	99.2248062	230	15	389	2490	2123	1	0	2444

TRINITY_DN15958_c0_g1_i4	Glyma.12G155400.2	98.68421053	230	15	389	2490	2123	1	0	2444

TRINITY_DN6691_c0_g1_i1	Glyma.16G138300.2	98.13084112	224	2	774	2899	5	2130	0	2652

TRINITY_DN6691_c0_g1_i1	Glyma.16G138300.8	97.61904762	224	2	774	2899	5	2130	0	2652

TRINITY_DN6691_c0_g1_i1	Glyma.16G138300.10	96.83544304	224	2	774	2899	5	2130	0	2652

TRINITY_DN6691_c0_g2_i1	Glyma.16G138300.2	98.72611465	224	2	523	2648	5	2130	0	2652

TRINITY_DN6691_c0_g2_i1	Glyma.16G138300.8	98.72727273	224	2	523	2648	5	2130	0	2652

TRINITY_DN6691_c0_g2_i1	Glyma.16G138300.10	98.72881356	224	2	523	2648	5	2130	0	2652

TRINITY_DN10955_c0_g1_i1	Glyma.12G003300.1	96.21212121	221	8	188	2308	2127	1	0	2643

TRINITY_DN15329_c0_g2_i4	Glyma.02G085000.2	100	202	14	377	2499	2170	68	0	2606

TRINITY_DN15329_c0_g2_i4	Glyma.02G085000.5	98.82352941	202	14	377	2499	2170	68	0	2606

TRINITY_DN13377_c0_g1_i1	Glyma.15G100500.9	98.66666667	202	31	342	2416	5	2121	0	2386

TRINITY_DN13377_c0_g1_i1	Glyma.15G100500.4	98.66666667	202	31	342	2416	5	2121	0	2386

TRINITY_DN13377_c0_g1_i2	Glyma.15G100500.9	95.6284153	202	31	381	2455	5	2121	0	2386

TRINITY_DN13377_c0_g1_i2	Glyma.15G100500.4	95.6284153	202	31	381	2455	5	2121	0	2386

TRINITY_DN13377_c0_g1_i3	Glyma.15G100500.9	100	202	31	176	2250	5	2121	0	2386

TRINITY_DN13377_c0_g1_i3	Glyma.15G100500.4	97.91666667	202	31	176	2250	5	2121	0	2386

TRINITY_DN9702_c0_g2_i1	Glyma.04G069600.2	95	165	2	191	2313	23	2151	0	2979

TRINITY_DN5837_c0_g2_i1	Glyma.20G177800.2	98.38709677	154	10	690	2809	2114	1	0	2924

TRINITY_DN13676_c0_g1_i1	Glyma.09G007200.1	96.93251534	133	3	234	2362	14	2136	0	3157

TRINITY_DN10506_c0_g1_i1	Glyma.06G127800.1	98.87640449	125	32	474	2548	1	2108	0	2754

TRINITY_DN13778_c1_g3_i3	Glyma.15G237200.1	95.32163743	312	21	218	2327	3330	1218	0	1988

TRINITY_DN15310_c0_g1_i1	Glyma.13G202800.1	100	213	17	356	2476	1	2106	0	2562

TRINITY_DN15310_c0_g1_i3	Glyma.13G202800.1	95.46165884	213	17	227	2347	1	2106	0	2562

TRINITY_DN13377_c0_g1_i1	Glyma.15G100500.5	98.26589595	202	31	342	2415	5	2120	0	2385

TRINITY_DN13377_c0_g1_i2	Glyma.15G100500.5	95.6284153	202	31	381	2454	5	2120	0	2385

TRINITY_DN13377_c0_g1_i3	Glyma.15G100500.5	100	202	31	176	2249	5	2120	0	2385

TRINITY_DN13377_c0_g1_i4	Glyma.15G100500.9	100	202	31	381	2454	5	2120	0	2385

TRINITY_DN13377_c0_g1_i4	Glyma.15G100500.4	98.46153846	202	31	381	2454	5	2120	0	2385

TRINITY_DN13377_c0_g1_i5	Glyma.15G100500.9	96.20253165	202	31	176	2249	5	2120	0	2385

TRINITY_DN13377_c0_g1_i5	Glyma.15G100500.4	96.20253165	202	31	176	2249	5	2120	0	2385

TRINITY_DN11155_c0_g1_i1	Glyma.08G096100.2	98.35526316	196	0	377	2504	2169	42	0	2844

TRINITY_DN16082_c0_g1_i2	Glyma.08G202000.5	95.32163743	187	7	581	2678	2128	1	0	2700

TRINITY_DN16217_c2_g1_i1	Glyma.14G004500.1	98.26589595	177	12	158	2275	2091	1	0	2645

TRINITY_DN16217_c2_g1_i10	Glyma.14G004500.1	96.13733906	177	12	158	2275	2091	1	0	2645

TRINITY_DN15501_c0_g2_i3	Glyma.13G162800.2	98.80239521	129	4	518	2641	2127	1	0	3182

TRINITY_DN13377_c0_g1_i4	Glyma.07G253800.14	99.07407407	343	54	410	2453	1	2107	0	1362

TRINITY_DN13377_c0_g1_i4	Glyma.07G253800.8	95.95959596	343	54	410	2453	1	2107	0	1362

TRINITY_DN13377_c0_g1_i4	Glyma.07G253800.3	99.11894273	343	54	410	2453	1	2107	0	1362

TRINITY_DN13377_c0_g1_i4	Glyma.07G253800.13	97.72727273	343	54	410	2453	1	2107	0	1362

TRINITY_DN13377_c0_g1_i4	Glyma.07G253800.2	100	343	54	410	2453	1	2107	0	1362

TRINITY_DN13377_c0_g1_i4	Glyma.07G253800.1	95.98108747	343	54	410	2453	1	2107	0	1362

TRINITY_DN13377_c0_g1_i5	Glyma.07G253800.14	98.1595092	343	54	205	2248	1	2107	0	1362

TRINITY_DN13377_c0_g1_i5	Glyma.07G253800.8	100	343	54	205	2248	1	2107	0	1362

TRINITY_DN13377_c0_g1_i5	Glyma.07G253800.3	95.83333333	343	54	205	2248	1	2107	0	1362

TRINITY_DN13377_c0_g1_i5	Glyma.07G253800.13	97.07602339	343	54	205	2248	1	2107	0	1362

TRINITY_DN13377_c0_g1_i5	Glyma.07G253800.2	97.07602339	343	54	205	2248	1	2107	0	1362

TRINITY_DN13377_c0_g1_i5	Glyma.07G253800.1	98.33333333	343	54	205	2248	1	2107	0	1362

TRINITY_DN13377_c0_g1_i4	Glyma.13G212200.1	97.56097561	207	26	381	2453	5	2113	0	2316

TRINITY_DN13377_c0_g1_i5	Glyma.13G212200.1	97.50692521	207	26	176	2248	5	2113	0	2316

TRINITY_DN13377_c0_g1_i4	Glyma.15G100500.1	98.46153846	202	31	381	2453	5	2119	0	2383

TRINITY_DN13377_c0_g1_i4	Glyma.15G100500.2	98.49246231	202	31	381	2453	5	2119	0	2383

TRINITY_DN13377_c0_g1_i5	Glyma.15G100500.1	100	202	31	176	2248	5	2119	0	2383

TRINITY_DN13377_c0_g1_i5	Glyma.15G100500.2	100	202	31	176	2248	5	2119	0	2383

TRINITY_DN15098_c1_g1_i2	Glyma.04G048100.1	99.31972789	191	8	283	2394	1	2127	0	2772

TRINITY_DN15187_c0_g1_i1	Glyma.08G152400.1	100	182	7	540	2655	4329	2205	0	2835

TRINITY_DN11835_c0_g1_i1	Glyma.03G119900.1	100	181	2	425	2548	2127	1	0	2905

TRINITY_DN11835_c0_g1_i2	Glyma.03G119900.1	100	181	2	391	2514	2127	1	0	2905

TRINITY_DN14345_c0_g2_i2	Glyma.08G124600.1	98.53372434	166	3	672	2795	2124	1	0	2970

TRINITY_DN14216_c0_g1_i1	Glyma.19G159400.7	100	161	1	153	2273	1	2127	0	2998

TRINITY_DN14216_c0_g1_i1	Glyma.19G159400.8	100	161	1	153	2273	1	2127	0	2998

TRINITY_DN16303_c0_g1_i4	Glyma.12G201100.4	100	125	5	152	2278	1	2100	0	3061

TRINITY_DN16303_c0_g1_i4	Glyma.12G201100.3	100	125	5	152	2278	1	2100	0	3061

TRINITY_DN16303_c0_g1_i4	Glyma.12G201100.1	100	125	5	152	2278	1	2100	0	3061

TRINITY_DN16303_c0_g1_i4	Glyma.12G201100.2	100	125	5	152	2278	1	2100	0	3061

TRINITY_DN16303_c0_g1_i4	Glyma.13G301100.3	100	118	0	152	2278	1	2127	0	3275

TRINITY_DN16303_c0_g1_i4	Glyma.13G301100.4	98	118	0	152	2278	1	2127	0	3275

TRINITY_DN16303_c0_g1_i4	Glyma.13G301100.2	100	118	0	152	2278	1	2127	0	3275

TRINITY_DN16303_c0_g1_i4	Glyma.13G301100.1	100	118	0	152	2278	1	2127	0	3275

TRINITY_DN14967_c1_g1_i3	Glyma.11G004300.2	100	106	4	62	2181	1	2126	0	3290

TRINITY_DN14967_c1_g1_i3	Glyma.11G004300.1	100	106	4	62	2181	1	2126	0	3290

TRINITY_DN13377_c0_g1_i1	Glyma.07G253800.9	95.16129032	343	54	371	2413	1	2106	0	1360

TRINITY_DN13377_c0_g1_i1	Glyma.07G253800.15	96.52173913	343	54	371	2413	1	2106	0	1360

TRINITY_DN13377_c0_g1_i1	Glyma.07G253800.10	96.52173913	343	54	371	2413	1	2106	0	1360

TRINITY_DN13377_c0_g1_i1	Glyma.07G253800.16	96.52173913	343	54	371	2413	1	2106	0	1360

TRINITY_DN13377_c0_g1_i1	Glyma.07G253800.4	96.52173913	343	54	371	2413	1	2106	0	1360

TRINITY_DN13377_c0_g1_i1	Glyma.07G253800.5	96.52173913	343	54	371	2413	1	2106	0	1360

TRINITY_DN13377_c0_g1_i2	Glyma.07G253800.9	97.81704782	343	54	410	2452	1	2106	0	1360

TRINITY_DN13377_c0_g1_i2	Glyma.07G253800.15	96.15384615	343	54	410	2452	1	2106	0	1360

TRINITY_DN13377_c0_g1_i2	Glyma.07G253800.10	96.15384615	343	54	410	2452	1	2106	0	1360

TRINITY_DN13377_c0_g1_i2	Glyma.07G253800.16	96.15384615	343	54	410	2452	1	2106	0	1360

TRINITY_DN13377_c0_g1_i2	Glyma.07G253800.4	96.45776567	343	54	410	2452	1	2106	0	1360

TRINITY_DN13377_c0_g1_i2	Glyma.07G253800.5	96.17486339	343	54	410	2452	1	2106	0	1360

TRINITY_DN13377_c0_g1_i3	Glyma.07G253800.9	97.82608696	343	54	205	2247	1	2106	0	1360

TRINITY_DN13377_c0_g1_i3	Glyma.07G253800.15	97.82608696	343	54	205	2247	1	2106	0	1360

TRINITY_DN13377_c0_g1_i3	Glyma.07G253800.10	100	343	54	205	2247	1	2106	0	1360

TRINITY_DN13377_c0_g1_i3	Glyma.07G253800.16	96	343	54	205	2247	1	2106	0	1360

TRINITY_DN13377_c0_g1_i3	Glyma.07G253800.4	99.29577465	343	54	205	2247	1	2106	0	1360

TRINITY_DN13377_c0_g1_i3	Glyma.07G253800.5	96.0199005	343	54	205	2247	1	2106	0	1360

TRINITY_DN13377_c0_g1_i4	Glyma.07G253800.9	97.82608696	343	54	410	2452	1	2106	0	1360

TRINITY_DN13377_c0_g1_i4	Glyma.07G253800.15	98.05447471	343	54	410	2452	1	2106	0	1360

TRINITY_DN13377_c0_g1_i4	Glyma.07G253800.10	98.74213836	343	54	410	2452	1	2106	0	1360

TRINITY_DN13377_c0_g1_i4	Glyma.07G253800.16	96.2962963	343	54	410	2452	1	2106	0	1360

TRINITY_DN13377_c0_g1_i4	Glyma.07G253800.4	96.2962963	343	54	410	2452	1	2106	0	1360

TRINITY_DN13377_c0_g1_i4	Glyma.07G253800.5	100	343	54	410	2452	1	2106	0	1360

TRINITY_DN13377_c0_g1_i5	Glyma.07G253800.9	97.65258216	343	54	205	2247	1	2106	0	1360

TRINITY_DN13377_c0_g1_i5	Glyma.07G253800.15	100	343	54	205	2247	1	2106	0	1360

TRINITY_DN13377_c0_g1_i5	Glyma.07G253800.10	100	343	54	205	2247	1	2106	0	1360

TRINITY_DN13377_c0_g1_i5	Glyma.07G253800.16	100	343	54	205	2247	1	2106	0	1360

TRINITY_DN13377_c0_g1_i5	Glyma.07G253800.4	99.43820225	343	54	205	2247	1	2106	0	1360

TRINITY_DN13377_c0_g1_i5	Glyma.07G253800.5	95.4954955	343	54	205	2247	1	2106	0	1360

TRINITY_DN38924_c0_g1_i1	Glyma.10G084300.1	98.24561404	237	8	234	2351	1	2121	0	2529

TRINITY_DN13377_c0_g1_i1	Glyma.15G100500.8	100	202	31	342	2413	5	2118	0	2381

TRINITY_DN13377_c0_g1_i1	Glyma.15G100500.3	96.55172414	202	31	342	2413	5	2118	0	2381

TRINITY_DN13377_c0_g1_i2	Glyma.15G100500.8	95.6284153	202	31	381	2452	5	2118	0	2381

TRINITY_DN13377_c0_g1_i2	Glyma.15G100500.3	95.6284153	202	31	381	2452	5	2118	0	2381

TRINITY_DN13377_c0_g1_i3	Glyma.15G100500.8	96.13402062	202	31	176	2247	5	2118	0	2381

TRINITY_DN13377_c0_g1_i3	Glyma.15G100500.3	96.13402062	202	31	176	2247	5	2118	0	2381

TRINITY_DN13377_c0_g1_i4	Glyma.15G100500.8	96.2962963	202	31	381	2452	5	2118	0	2381

TRINITY_DN13377_c0_g1_i4	Glyma.15G100500.3	97.56097561	202	31	381	2452	5	2118	0	2381

TRINITY_DN13377_c0_g1_i5	Glyma.15G100500.8	97.87234043	202	31	176	2247	5	2118	0	2381

TRINITY_DN13377_c0_g1_i5	Glyma.15G100500.3	98.0952381	202	31	176	2247	5	2118	0	2381

TRINITY_DN85_c0_g1_i1	Glyma.07G259400.4	100	198	18	2565	4647	2142	17	0	2551

TRINITY_DN85_c0_g1_i1	Glyma.07G259400.6	98.16513761	198	18	2565	4647	2142	17	0	2551

TRINITY_DN85_c0_g1_i2	Glyma.07G259400.4	96.96969697	198	18	2486	4568	2142	17	0	2551

TRINITY_DN85_c0_g1_i2	Glyma.07G259400.6	100	198	18	2486	4568	2142	17	0	2551

TRINITY_DN9073_c0_g2_i2	Glyma.01G014700.4	97.11538462	144	8	531	2634	1	2119	0	2940

TRINITY_DN16054_c1_g1_i5	Glyma.10G168700.3	100	109	0	557	2682	2126	1	0	3323

TRINITY_DN16181_c0_g1_i4	Glyma.04G034200.2	100	103	0	312	2437	2130	5	0	3356

TRINITY_DN16309_c0_g1_i1	Glyma.05G060300.1	98.1981982	343	53	2925	4994	2897	4990	0	1469

TRINITY_DN16309_c0_g1_i1	Glyma.05G060300.2	98.00399202	343	53	2925	4994	2897	4990	0	1469

TRINITY_DN16309_c0_g1_i1	Glyma.05G060300.3	98.20359281	343	53	2925	4994	2897	4990	0	1469

TRINITY_DN16309_c0_g1_i4	Glyma.05G060300.1	99.31506849	343	53	2991	5060	2897	4990	0	1469

TRINITY_DN16309_c0_g1_i4	Glyma.05G060300.2	99.3006993	343	53	2991	5060	2897	4990	0	1469

TRINITY_DN16309_c0_g1_i4	Glyma.05G060300.3	99.29577465	343	53	2991	5060	2897	4990	0	1469

TRINITY_DN3323_c0_g1_i1	Glyma.03G139300.1	98.66666667	295	7	752	2870	215	2333	0	2213

TRINITY_DN15371_c0_g2_i1	Glyma.16G079400.1	100	271	12	1	2117	311	2424	0	2300

TRINITY_DN38924_c0_g1_i1	Glyma.02G192500.1	95.43378995	221	5	234	2351	1	2121	0	2628

TRINITY_DN15329_c0_g2_i3	Glyma.02G085000.2	95	201	15	377	2495	2170	68	0	2630

TRINITY_DN15329_c0_g2_i3	Glyma.02G085000.5	95	201	15	377	2495	2170	68	0	2630

TRINITY_DN15416_c1_g1_i2	Glyma.04G093800.1	97.71863118	186	5	425	2548	2522	414	0	2784

TRINITY_DN17528_c0_g1_i1	Glyma.04G180900.5	100	162	2	1	2125	2122	1	0	3007

TRINITY_DN17528_c0_g1_i1	Glyma.04G180900.2	100	162	2	1	2125	2122	1	0	3007

TRINITY_DN17528_c0_g1_i1	Glyma.04G180900.3	100	162	2	1	2125	2122	1	0	3007

TRINITY_DN13095_c0_g1_i1	Glyma.17G063000.1	100	160	4	381	2495	2124	1	0	2966

TRINITY_DN13095_c0_g1_i1	Glyma.13G096500.1	96.94656489	157	3	381	2495	2124	1	0	2983

TRINITY_DN5646_c0_g1_i1	Glyma.03G133500.2	98.61386139	155	28	963	3050	1776	3867	0	2614

TRINITY_DN5646_c0_g1_i1	Glyma.03G133500.1	99.3006993	155	28	963	3050	1776	3867	0	2614

TRINITY_DN5646_c0_g1_i2	Glyma.03G133500.2	95.45454545	155	28	963	3050	1776	3867	0	2614

TRINITY_DN5646_c0_g1_i2	Glyma.03G133500.1	95.45454545	155	28	963	3050	1776	3867	0	2614

TRINITY_DN16093_c0_g2_i1	Glyma.06G284000.1	100	239	5	1120	3235	836	2958	0	2540

TRINITY_DN16093_c0_g2_i2	Glyma.06G284000.1	95.32258065	239	5	1120	3235	836	2958	0	2540

TRINITY_DN13152_c0_g1_i2	Glyma.14G189900.1	95.82909461	203	18	976	3098	2084	1	0	2532

TRINITY_DN16354_c0_g1_i4	Glyma.20G214100.1	95	164	4	531	2651	2118	1	0	2955

TRINITY_DN16354_c0_g1_i4	Glyma.10G176200.1	95	162	4	531	2651	2118	1	0	2966

TRINITY_DN14724_c1_g1_i1	Glyma.03G004800.1	100	154	12	2340	4447	2111	1	0	2881

TRINITY_DN14830_c0_g1_i1	Glyma.19G004600.1	95.74468085	147	0	139	2262	1	2124	0	3109

TRINITY_DN14641_c2_g2_i2	Glyma.17G047100.1	100	145	13	99	2192	2124	1	0	2926

TRINITY_DN14830_c0_g1_i1	Glyma.05G003600.4	95.45454545	136	0	139	2262	1	2124	0	3169

TRINITY_DN14830_c0_g1_i1	Glyma.05G003600.1	100	136	0	139	2262	1	2124	0	3169

TRINITY_DN14464_c0_g1_i3	Glyma.09G260100.1	95.16129032	114	7	384	2492	2118	1	0	3155

TRINITY_DN14464_c0_g1_i4	Glyma.09G260100.1	100	114	7	384	2492	2118	1	0	3155

TRINITY_DN4545_c0_g1_i1	Glyma.03G252000.1	95	108	0	172	2295	1	2124	0	3325

TRINITY_DN2211_c0_g1_i1	Glyma.03G264400.2	100	105	0	79	2202	1	2124	0	3341

TRINITY_DN13716_c0_g2_i1	Glyma.02G130700.1	95	303	30	550	2643	3166	1061	0	1945

TRINITY_DN16004_c1_g1_i5	Glyma.15G207900.1	100	272	8	95	2213	410	2528	0	2362

TRINITY_DN7321_c0_g1_i1	Glyma.02G034200.1	95.62043796	250	22	120	2210	2142	31	0	2257

TRINITY_DN15815_c0_g2_i5	Glyma.06G254500.1	98.4496124	241	5	2470	4587	2121	1	0	2540

TRINITY_DN15815_c0_g2_i6	Glyma.06G254500.1	98.61111111	241	5	1909	4026	2121	1	0	2540

TRINITY_DN15529_c1_g1_i5	Glyma.05G232400.2	96.51162791	228	10	20	2131	2166	49	0	2555

TRINITY_DN15529_c1_g1_i5	Glyma.05G232400.1	95.23809524	228	10	20	2131	2166	49	0	2555

TRINITY_DN15529_c1_g1_i5	Glyma.05G232400.4	96.25	228	10	20	2131	2166	49	0	2555

TRINITY_DN15529_c1_g1_i5	Glyma.05G232400.10	99.61685824	228	10	20	2131	2151	34	0	2555

TRINITY_DN14801_c0_g2_i1	Glyma.14G153000.1	95.57522124	218	13	259	2373	2331	223	0	2571

TRINITY_DN15329_c0_g2_i6	Glyma.02G085000.3	95.59286464	198	20	377	2481	2182	68	0	2656

TRINITY_DN15329_c0_g2_i6	Glyma.02G085000.1	97.61904762	198	20	377	2481	2182	68	0	2656

TRINITY_DN16181_c0_g1_i1	Glyma.04G034200.2	100	164	2	312	2433	2130	9	0	3000

TRINITY_DN16429_c0_g2_i2	Glyma.17G228100.1	96.62288931	163	17	189	2296	38	2145	0	2824

TRINITY_DN15572_c0_g1_i1	Glyma.02G137100.1	98.13829787	162	37	213	2316	79	2151	0	2577

TRINITY_DN14862_c0_g1_i2	Glyma.20G181600.1	100	145	2	1344	3463	2124	5	0	3079

TRINITY_DN14862_c0_g1_i2	Glyma.20G181600.4	100	145	2	1344	3463	2124	5	0	3079

TRINITY_DN5216_c0_g1_i3	Glyma.16G158700.1	98.28571429	132	11	171	2292	1	2090	0	2970

TRINITY_DN5216_c0_g1_i2	Glyma.16G158700.1	98.28473413	132	12	171	2273	1	2090	0	2846

TRINITY_DN13707_c0_g1_i1	Glyma.20G230900.1	97.34513274	129	5	264	2362	1	2123	0	3051

TRINITY_DN36012_c0_g1_i1	Glyma.15G125500.3	100	126	4	78	2198	3	2121	0	3184

TRINITY_DN36012_c0_g1_i1	Glyma.15G125500.4	96.15384615	126	4	78	2198	3	2121	0	3184

TRINITY_DN36012_c0_g1_i1	Glyma.15G125500.1	95	126	4	78	2198	3	2121	0	3184

TRINITY_DN36012_c0_g1_i1	Glyma.15G125500.2	100	126	4	78	2198	3	2121	0	3184

TRINITY_DN15396_c0_g1_i2	Glyma.19G202200.1	98.27586207	323	18	397	2509	2199	93	0	1975

TRINITY_DN15529_c1_g1_i3	Glyma.07G089000.1	97.61904762	298	8	568	2685	2160	49	0	2178

TRINITY_DN15529_c1_g1_i3	Glyma.07G089000.3	96	298	8	568	2685	2160	49	0	2178

TRINITY_DN15529_c1_g1_i3	Glyma.07G089000.4	96	298	8	568	2685	2160	49	0	2178

TRINITY_DN15529_c1_g1_i3	Glyma.07G089000.2	100	298	8	568	2685	2160	49	0	2178

TRINITY_DN15276_c0_g1_i4	Glyma.05G193200.3	100	221	6	688	2809	386	2496	0	2623

TRINITY_DN15276_c0_g1_i4	Glyma.05G193200.2	96.96969697	221	6	688	2809	386	2496	0	2623

TRINITY_DN15276_c0_g1_i4	Glyma.05G193200.1	100	221	6	688	2809	386	2496	0	2623

TRINITY_DN12659_c0_g1_i1	Glyma.U025600.1	98.50746269	158	6	164	2266	1	2118	0	2894

TRINITY_DN15276_c0_g1_i2	Glyma.05G193200.3	99.15966387	156	6	688	2809	386	2496	0	2983

TRINITY_DN15276_c0_g1_i2	Glyma.05G193200.2	100	156	6	688	2809	386	2496	0	2983

TRINITY_DN15276_c0_g1_i2	Glyma.05G193200.1	98.93617021	156	6	688	2809	386	2496	0	2983

TRINITY_DN14345_c0_g2_i2	Glyma.05G166600.1	100	155	3	678	2795	2121	1	0	3027

TRINITY_DN17930_c0_g1_i1	Glyma.06G059800.1	100	153	1	179	2297	2122	1	0	3051

TRINITY_DN16163_c0_g1_i1	Glyma.06G303700.1	95.03546099	148	6	677	2782	68	2188	0	2988

TRINITY_DN16163_c0_g1_i3	Glyma.06G303700.1	98.52579853	148	6	699	2804	68	2188	0	2988

TRINITY_DN16163_c0_g1_i4	Glyma.06G303700.1	100	148	6	208	2313	68	2188	0	2988

TRINITY_DN16382_c0_g3_i1	Glyma.18G060600.3	100	124	0	177	2298	1	2122	0	3232

TRINITY_DN16181_c0_g1_i3	Glyma.04G034200.2	100	120	0	312	2433	2130	9	0	3254

TRINITY_DN12659_c1_g1_i1	Glyma.01G140600.7	98.0952381	113	3	235	2352	2121	1	0	3260

TRINITY_DN4545_c0_g1_i1	Glyma.19G249400.1	96.2962963	111	0	172	2293	1	2122	0	3304

TRINITY_DN15137_c0_g1_i3	Glyma.14G222700.2	97.5	318	9	668	2779	2235	118	0	2078

TRINITY_DN13407_c0_g1_i1	Glyma.19G063800.2	95.65217391	308	21	321	2427	2131	23	0	2043

TRINITY_DN13407_c0_g1_i1	Glyma.19G063800.1	95.65217391	308	21	321	2427	2131	23	0	2043

TRINITY_DN2205_c0_g1_i1	Glyma.02G163200.2	98.21428571	239	18	4	2108	2481	370	0	2431

TRINITY_DN2205_c0_g1_i1	Glyma.02G163200.1	97.6744186	239	18	4	2108	2481	370	0	2431

TRINITY_DN6726_c0_g1_i1	Glyma.13G277400.1	100	238	12	88	2198	55	2159	0	2431

TRINITY_DN6726_c0_g1_i1	Glyma.13G277400.3	100	238	12	88	2198	1	2105	0	2431

TRINITY_DN6726_c0_g1_i1	Glyma.12G224200.1	100	225	15	88	2196	1	2100	0	2457

TRINITY_DN16082_c0_g1_i6	Glyma.08G202000.4	95.37750385	204	13	552	2649	2119	1	0	2625

TRINITY_DN16082_c0_g1_i2	Glyma.08G202000.4	100	183	13	581	2678	2119	1	0	2741

TRINITY_DN14862_c0_g1_i2	Glyma.10G209000.1	97.08737864	156	2	1344	3463	2124	5	0	3040

TRINITY_DN14862_c0_g1_i2	Glyma.10G209000.2	95.45454545	156	2	1344	3463	2124	5	0	3040

TRINITY_DN9883_c0_g2_i1	Glyma.15G103100.1	98.10126582	153	4	288	2396	2151	31	0	2992

TRINITY_DN17930_c0_g1_i1	Glyma.04G059100.1	100	147	4	179	2297	2119	1	0	3077

TRINITY_DN14979_c0_g1_i1	Glyma.14G223400.3	95.3125	136	2	536	2653	2121	1	0	3144

TRINITY_DN8008_c0_g1_i1	Glyma.01G060700.1	95.78947368	122	2	459	2579	2118	1	0	3221

TRINITY_DN15529_c1_g1_i3	Glyma.05G232400.2	96.41791045	122	3	568	2685	2166	49	0	3203

TRINITY_DN15529_c1_g1_i3	Glyma.05G232400.1	96.41833811	122	3	568	2685	2166	49	0	3203

TRINITY_DN15529_c1_g1_i3	Glyma.05G232400.4	96.55172414	122	3	568	2685	2166	49	0	3203

TRINITY_DN15529_c1_g1_i3	Glyma.05G232400.10	96.41693811	122	3	568	2685	2151	34	0	3203

TRINITY_DN8008_c0_g1_i1	Glyma.02G119000.1	99.6875	120	2	459	2579	2118	1	0	3232

TRINITY_DN14464_c0_g1_i3	Glyma.18G232400.1	98.71794872	100	5	384	2492	2115	1	0	3247

TRINITY_DN14464_c0_g1_i4	Glyma.18G232400.1	97.14285714	100	5	384	2492	2115	1	0	3247

TRINITY_DN13716_c0_g2_i1	Glyma.02G130700.2	100	306	28	550	2643	3163	1061	0	1941

TRINITY_DN16155_c1_g1_i3	Glyma.12G076100.1	97.6109215	220	7	1	2108	2120	1	0	2619

TRINITY_DN13152_c0_g1_i3	Glyma.02G222900.1	95.82417582	156	10	976	3086	2102	1	0	2876

TRINITY_DN14862_c0_g1_i2	Glyma.20G181600.2	98.63013699	145	1	1344	3463	2121	5	0	3092

TRINITY_DN14862_c0_g1_i2	Glyma.20G181600.3	98.125	145	1	1344	3463	2121	5	0	3092

TRINITY_DN13902_c0_g1_i5	Glyma.02G248700.2	98.19277108	132	2	2	2120	53	2169	0	3158

TRINITY_DN13902_c0_g1_i5	Glyma.02G248700.1	100	132	2	2	2120	53	2169	0	3158

TRINITY_DN14967_c1_g1_i3	Glyma.01G239200.1	100	129	0	62	2181	1	2120	0	3201

TRINITY_DN5141_c0_g1_i1	Glyma.18G282700.5	96.55172414	127	4	484	2600	384	2500	0	3173

TRINITY_DN16158_c0_g1_i2	Glyma.20G001300.3	98.27586207	188	3	1032	3149	2799	685	0	2839

TRINITY_DN12659_c0_g1_i1	Glyma.U025600.14	100	158	5	164	2266	1	2115	0	2909

TRINITY_DN14188_c0_g1_i1	Glyma.09G160000.1	96	140	4	576	2682	6	2124	0	3061

TRINITY_DN14188_c0_g1_i1	Glyma.16G209400.1	96	137	6	576	2682	6	2121	0	3057

TRINITY_DN3145_c0_g1_i1	Glyma.10G003000.1	100	342	15	280	2381	20	2130	0	1868

TRINITY_DN11825_c0_g2_i1	Glyma.04G191500.2	98.57819905	251	9	373	2479	2116	1	0	2436

TRINITY_DN11825_c0_g2_i1	Glyma.04G191500.1	98.57142857	251	9	373	2479	2116	1	0	2436

TRINITY_DN11825_c0_g2_i3	Glyma.04G191500.2	98.55769231	251	9	373	2479	2116	1	0	2436

TRINITY_DN11825_c0_g2_i3	Glyma.04G191500.1	98.57142857	251	9	373	2479	2116	1	0	2436

TRINITY_DN15681_c0_g2_i1	Glyma.17G033700.1	95.61752988	208	18	287	2389	1	2101	0	2553

TRINITY_DN15681_c0_g2_i3	Glyma.17G033700.2	100	208	18	287	2390	1	2100	0	2553

TRINITY_DN12675_c0_g1_i1	Glyma.17G186500.1	100	186	3	238	2352	2115	1	0	2843

TRINITY_DN13902_c0_g1_i6	Glyma.11G219700.1	96.34146341	183	3	6	2117	58	2175	0	2859

TRINITY_DN2153_c0_g1_i1	Glyma.08G184200.1	100	159	4	1	2118	4408	6516	0	2972

TRINITY_DN2153_c0_g1_i1	Glyma.08G184200.2	100	159	5	1	2118	4408	6513	0	2953

TRINITY_DN10853_c0_g2_i1	Glyma.17G106000.3	100	151	3	221	2329	31	2148	0	3016

TRINITY_DN10853_c0_g2_i1	Glyma.17G106000.1	99.23664122	151	3	221	2329	31	2148	0	3016

TRINITY_DN15598_c0_g1_i3	Glyma.08G332900.1	100	425	19	43	2148	1	2100	0	1375

TRINITY_DN16082_c0_g1_i12	Glyma.08G202000.5	98.61111111	421	48	121	2187	2274	184	0	1086

TRINITY_DN13377_c0_g1_i4	Glyma.17G020500.1	99.62686567	356	49	410	2453	1	2101	0	1362

TRINITY_DN13377_c0_g1_i5	Glyma.17G020500.1	98.33333333	356	49	205	2248	1	2101	0	1362

TRINITY_DN13654_c0_g1_i2	Glyma.16G015400.1	97.68786127	301	33	437	2525	2148	72	0	1803

TRINITY_DN16440_c0_g1_i2	Glyma.01G112200.3	97.5	290	10	1243	3353	3153	1043	0	2226

TRINITY_DN13902_c0_g1_i6	Glyma.02G248700.2	99.45945946	247	1	2	2117	53	2169	0	2534

TRINITY_DN13902_c0_g1_i6	Glyma.02G248700.1	99.45945946	247	1	2	2117	53	2169	0	2534

TRINITY_DN14967_c1_g1_i3	Glyma.10G250100.2	100	242	14	62	2169	1	2109	0	2459

TRINITY_DN14967_c1_g1_i3	Glyma.10G250100.1	100	242	14	62	2169	1	2109	0	2459

TRINITY_DN20935_c0_g1_i1	Glyma.16G105100.1	96.1038961	210	12	1	2099	5	2100	0	2494

TRINITY_DN15979_c0_g3_i1	Glyma.06G152200.3	99.14529915	201	15	409	2513	2109	5	0	2641

TRINITY_DN15979_c0_g3_i2	Glyma.06G152200.3	97.22792608	201	15	409	2513	2109	5	0	2641

TRINITY_DN14746_c0_g2_i2	Glyma.02G095500.1	99.51923077	156	13	129	2225	1	2109	0	2865

TRINITY_DN13407_c0_g1_i1	Glyma.10G270600.1	99.36708861	146	5	319	2430	2139	25	0	3055

TRINITY_DN14961_c1_g1_i1	Glyma.08G266700.4	97.05882353	109	2	179	2295	2100	2	0	3190

TRINITY_DN14961_c1_g1_i1	Glyma.08G266700.5	97.05159705	109	2	179	2295	2100	2	0	3190

TRINITY_DN15451_c0_g1_i7	Glyma.10G053500.1	100	328	16	395	2499	2103	14	0	1851

TRINITY_DN15451_c0_g1_i8	Glyma.10G053500.1	100	328	16	395	2499	2103	14	0	1851

TRINITY_DN16132_c0_g1_i3	Glyma.17G226100.2	98.46547315	223	4	712	2826	21	2132	0	2639

TRINITY_DN13698_c1_g1_i1	Glyma.09G047300.3	98.31932773	213	10	791	2896	2109	1	0	2617

TRINITY_DN13698_c1_g1_i1	Glyma.09G047300.1	98.4375	213	10	791	2896	2109	1	0	2617

TRINITY_DN13698_c1_g1_i2	Glyma.09G047300.1	96.55172414	213	10	716	2821	2109	1	0	2617

TRINITY_DN13698_c1_g1_i3	Glyma.09G047300.3	97.87234043	213	10	872	2977	2109	1	0	2617

TRINITY_DN13698_c1_g1_i3	Glyma.09G047300.1	97.70114943	213	10	872	2977	2109	1	0	2617

TRINITY_DN13698_c1_g1_i1	Glyma.09G047300.2	98.4375	210	12	791	2896	2103	1	0	2595

TRINITY_DN13698_c1_g1_i1	Glyma.09G047300.4	99.25925926	210	12	791	2896	2103	1	0	2595

TRINITY_DN13698_c1_g1_i2	Glyma.09G047300.4	95	210	12	716	2821	2103	1	0	2595

TRINITY_DN13698_c1_g1_i3	Glyma.09G047300.2	95.86374696	210	12	872	2977	2103	1	0	2595

TRINITY_DN13698_c1_g1_i3	Glyma.09G047300.4	100	210	12	872	2977	2103	1	0	2595

TRINITY_DN7048_c0_g1_i1	Glyma.05G196100.1	95.12534819	175	2	36	2150	1	2115	0	2926

TRINITY_DN12858_c0_g1_i2	Glyma.06G146900.1	100	138	16	315	2429	2076	1	0	2878

TRINITY_DN10369_c0_g1_i1	Glyma.07G247100.2	98.60465116	136	4	580	2683	7	2119	0	3057

TRINITY_DN15660_c0_g1_i3	Glyma.16G178800.1	96.22641509	387	27	256	2355	2100	1	0	1568

TRINITY_DN15660_c0_g1_i3	Glyma.16G178800.2	99.42196532	387	27	256	2355	2100	1	0	1568

TRINITY_DN15660_c0_g1_i2	Glyma.16G178800.1	100	384	27	256	2355	2100	1	0	1585

TRINITY_DN15660_c0_g1_i2	Glyma.16G178800.2	99.30555556	384	27	256	2355	2100	1	0	1585

TRINITY_DN15310_c0_g1_i2	Glyma.09G102900.2	99.38650307	197	19	403	2492	1	2105	0	2588

TRINITY_DN13902_c0_g1_i6	Glyma.18G037700.1	100	179	1	6	2117	58	2172	0	2894

TRINITY_DN14596_c0_g1_i2	Glyma.20G174900.7	98.95833333	169	20	34	2117	1	2108	0	2724

TRINITY_DN12858_c0_g1_i2	Glyma.04G218900.1	100	152	6	315	2429	2091	1	0	2909

TRINITY_DN10853_c0_g2_i1	Glyma.17G106000.2	96.48760331	151	2	221	2329	31	2145	0	3031

TRINITY_DN10853_c0_g2_i1	Glyma.17G106000.4	97.75280899	151	2	221	2329	31	2145	0	3031

TRINITY_DN19429_c0_g1_i1	Glyma.05G012400.1	100	126	1	1	2115	2794	683	0	3188

TRINITY_DN19429_c0_g1_i1	Glyma.17G120500.1	100	124	0	1	2115	2797	683	0	3219

TRINITY_DN15647_c0_g1_i6	Glyma.05G232200.1	100	282	27	515	2597	2092	1	0	1999

TRINITY_DN14563_c0_g1_i4	Glyma.18G218900.1	98.24561404	207	24	2143	4247	2093	19	0	2447

TRINITY_DN14563_c0_g1_i4	Glyma.18G218900.2	100	207	24	2143	4247	2093	19	0	2447

TRINITY_DN13698_c1_g1_i1	Glyma.15G154900.3	95.45454545	197	11	791	2896	2106	1	0	2710

TRINITY_DN13698_c1_g1_i1	Glyma.15G154900.2	100	197	11	791	2896	2106	1	0	2710

TRINITY_DN13698_c1_g1_i3	Glyma.15G154900.3	97.87234043	197	11	872	2977	2106	1	0	2710

TRINITY_DN13698_c1_g1_i3	Glyma.15G154900.2	97.87234043	197	11	872	2977	2106	1	0	2710

TRINITY_DN16429_c0_g2_i2	Glyma.14G096100.1	96.49122807	173	10	189	2296	38	2142	0	2848

TRINITY_DN16429_c0_g2_i2	Glyma.14G096000.1	95	162	10	189	2296	38	2142	0	2909

TRINITY_DN10228_c0_g1_i1	Glyma.07G017700.1	98.31223629	161	5	311	2413	1	2106	0	2889

TRINITY_DN15907_c0_g2_i2	Glyma.01G008900.1	98.55072464	157	9	395	2488	2112	1	0	2892

TRINITY_DN15907_c0_g2_i2	Glyma.01G008900.2	96.77419355	157	9	395	2488	2112	1	0	2892

TRINITY_DN16155_c1_g1_i3	Glyma.12G174000.1	95.83333333	145	3	1	2108	2114	1	0	3062

TRINITY_DN9872_c0_g1_i1	Glyma.06G108500.7	97.18309859	139	11	1766	3879	1709	3783	0	2881

TRINITY_DN9872_c0_g1_i1	Glyma.06G108500.5	97.5	139	11	1766	3879	1568	3642	0	2881

TRINITY_DN10506_c0_g1_i1	Glyma.04G236500.1	98.87640449	135	28	474	2548	1	2087	0	2730

TRINITY_DN9476_c0_g1_i5	Glyma.07G263100.1	97.09543568	122	0	334	2447	2919	806	0	3229

TRINITY_DN15660_c0_g1_i3	Glyma.09G131500.1	98.26086957	397	22	256	2355	2100	1	0	1535

TRINITY_DN15660_c0_g1_i2	Glyma.09G131500.1	95.56313993	395	20	256	2355	2100	1	0	1546

TRINITY_DN15451_c0_g1_i1	Glyma.19G181900.1	99.39393939	253	30	511	2607	2103	40	0	2080

TRINITY_DN15451_c0_g1_i3	Glyma.19G181900.1	96.66666667	253	30	511	2607	2103	40	0	2080

TRINITY_DN16429_c0_g2_i4	Glyma.17G228100.1	96.34703196	174	12	189	2286	38	2144	0	2802

TRINITY_DN10477_c0_g2_i3	Glyma.05G153400.1	97.21115538	153	11	240	2333	175	2286	0	2926

TRINITY_DN15293_c0_g1_i4	Glyma.11G179600.2	99	141	15	565	2658	42	2141	0	2915

TRINITY_DN15293_c0_g1_i1	Glyma.11G179600.2	97.91666667	124	15	628	2721	42	2141	0	3009

TRINITY_DN18060_c0_g1_i1	Glyma.02G312200.2	97.64705882	77	4	853	2955	1	2113	0	3411

TRINITY_DN16138_c1_g1_i19	Glyma.15G049300.1	96.55172414	331	21	311	2402	1	2101	0	1866

TRINITY_DN15647_c0_g1_i6	Glyma.08G039600.1	100	295	29	515	2597	2089	1	0	1930

TRINITY_DN15958_c0_g1_i2	Glyma.12G155400.1	96.62162162	285	17	412	2496	2100	1	0	2069

TRINITY_DN15958_c0_g1_i5	Glyma.12G155400.1	95.49295775	285	17	412	2496	2100	1	0	2069

TRINITY_DN15529_c1_g1_i5	Glyma.07G089000.1	98.92008639	226	0	20	2131	2160	49	0	2649

TRINITY_DN15529_c1_g1_i5	Glyma.07G089000.3	98.92086331	226	0	20	2131	2160	49	0	2649

TRINITY_DN15529_c1_g1_i5	Glyma.07G089000.4	98.60788863	226	0	20	2131	2160	49	0	2649

TRINITY_DN15529_c1_g1_i5	Glyma.07G089000.2	98.60788863	226	0	20	2131	2160	49	0	2649

TRINITY_DN15681_c0_g2_i1	Glyma.07G239600.1	100	212	12	287	2389	1	2098	0	2577

TRINITY_DN11201_c0_g1_i2	Glyma.12G151100.1	96.23655914	162	2	319	2415	1	2112	0	2905

TRINITY_DN11201_c0_g1_i3	Glyma.12G151100.1	100	162	2	196	2292	1	2112	0	2905

TRINITY_DN12719_c0_g2_i6	Glyma.13G321300.44	97.4025974	160	21	356	2455	2067	6	0	2614

TRINITY_DN12719_c0_g2_i6	Glyma.13G321300.32	97.4025974	160	21	356	2455	2067	6	0	2614

TRINITY_DN12719_c0_g2_i6	Glyma.13G321300.12	97.48427673	160	21	356	2455	2067	6	0	2614

TRINITY_DN12719_c0_g2_i6	Glyma.13G321300.45	96.22641509	160	21	356	2455	2067	6	0	2614

TRINITY_DN12719_c0_g2_i6	Glyma.13G321300.33	96.22641509	160	21	356	2455	2067	6	0	2614

TRINITY_DN12719_c0_g2_i6	Glyma.13G321300.13	100	160	21	356	2455	2067	6	0	2614

TRINITY_DN12719_c0_g2_i8	Glyma.13G321300.44	100	160	21	356	2455	2067	6	0	2614

TRINITY_DN12719_c0_g2_i8	Glyma.13G321300.32	100	160	21	356	2455	2067	6	0	2614

TRINITY_DN12719_c0_g2_i8	Glyma.13G321300.12	97.17868339	160	21	356	2455	2067	6	0	2614

TRINITY_DN12719_c0_g2_i8	Glyma.13G321300.45	96.57534247	160	21	356	2455	2067	6	0	2614

TRINITY_DN12719_c0_g2_i8	Glyma.13G321300.33	98.64864865	160	21	356	2455	2067	6	0	2614

TRINITY_DN12719_c0_g2_i8	Glyma.13G321300.13	100	160	21	356	2455	2067	6	0	2614

TRINITY_DN10477_c0_g2_i2	Glyma.05G153400.2	97.56097561	156	19	46	2138	1	2090	0	2771

TRINITY_DN12172_c0_g1_i1	Glyma.13G286400.3	95.11400651	139	1	379	2490	2109	1	0	3110

TRINITY_DN15981_c0_g1_i13	Glyma.11G048300.2	97.25609756	132	3	1354	3459	2342	231	0	3131

TRINITY_DN15981_c0_g1_i13	Glyma.11G048300.1	97.61904762	132	3	1354	3459	2342	231	0	3131

TRINITY_DN15981_c0_g1_i18	Glyma.11G048300.2	100	132	3	1244	3349	2342	231	0	3131

TRINITY_DN15981_c0_g1_i18	Glyma.11G048300.1	100	132	3	1244	3349	2342	231	0	3131

TRINITY_DN15981_c0_g1_i9	Glyma.11G048300.2	98.11320755	132	3	1480	3585	2342	231	0	3131

TRINITY_DN15981_c0_g1_i9	Glyma.11G048300.1	98.38709677	132	3	1480	3585	2342	231	0	3131

TRINITY_DN13164_c0_g1_i1	Glyma.05G156900.2	96.5648855	103	0	1398	3509	1	2112	0	3330

TRINITY_DN13164_c0_g1_i3	Glyma.05G156900.2	97.5	103	0	1349	3460	1	2112	0	3330

TRINITY_DN16352_c0_g2_i1	Glyma.08G190700.2	100	319	16	2521	4613	2353	246	0	1995

TRINITY_DN16352_c0_g2_i1	Glyma.08G190700.1	100	319	16	2521	4613	2353	246	0	1995

TRINITY_DN7394_c0_g1_i1	Glyma.02G298400.1	95.38461538	314	16	1	2099	2261	4362	0	2023

TRINITY_DN7394_c0_g1_i1	Glyma.02G298500.1	98.38709677	304	15	1	2099	1658	3759	0	2078

TRINITY_DN13654_c0_g1_i2	Glyma.07G046800.1	98.27586207	299	22	437	2525	2142	69	0	1860

TRINITY_DN16254_c0_g1_i8	Glyma.17G057800.1	97.0624235	268	11	618	2709	3060	955	0	2259

TRINITY_DN15329_c0_g2_i6	Glyma.02G085000.2	98.91304348	202	11	377	2481	2170	68	0	2689

TRINITY_DN15329_c0_g2_i6	Glyma.02G085000.5	100	202	11	377	2481	2170	68	0	2689

TRINITY_DN10228_c0_g1_i1	Glyma.08G202900.1	98.30508475	175	5	311	2413	1	2103	0	2826

TRINITY_DN14027_c0_g1_i1	Glyma.20G217200.2	95.97855228	170	3	560	2669	2115	7	0	2937

TRINITY_DN15907_c0_g2_i2	Glyma.16G077500.1	98.55072464	155	7	395	2488	2109	1	0	2916

TRINITY_DN13152_c0_g1_i3	Glyma.14G189900.1	95.81749049	155	10	976	3086	2084	1	0	2865

TRINITY_DN16155_c1_g1_i3	Glyma.13G325400.1	95.83333333	143	1	1	2108	2111	1	0	3086

TRINITY_DN16155_c1_g1_i3	Glyma.13G325400.2	95.83333333	143	2	1	2108	2108	1	0	3068

TRINITY_DN14967_c1_g1_i3	Glyma.20G143500.1	100	249	13	62	2163	1	2102	0	2414

TRINITY_DN2205_c0_g1_i1	Glyma.10G209200.3	95.90361446	195	4	4	2108	2490	385	0	2758

TRINITY_DN2205_c0_g1_i1	Glyma.10G209200.1	98.62068966	195	4	4	2108	2490	385	0	2758

TRINITY_DN2205_c0_g1_i1	Glyma.10G209200.2	100	195	4	4	2108	2214	109	0	2758

TRINITY_DN16429_c0_g2_i4	Glyma.14G096100.1	100	179	9	189	2286	38	2141	0	2789

TRINITY_DN16429_c0_g2_i4	Glyma.14G096000.1	96.75324675	169	11	189	2286	38	2141	0	2844

TRINITY_DN12659_c0_g1_i1	Glyma.U025600.13	100	159	5	164	2266	1	2106	0	2944

TRINITY_DN9498_c0_g1_i5	Glyma.14G065300.1	100	145	8	1	2104	10	2107	0	2977

TRINITY_DN15143_c0_g2_i4	Glyma.09G187400.1	98.7012987	144	8	234	2342	1	2094	0	2988

TRINITY_DN15681_c0_g2_i2	Glyma.17G033700.2	98.98989899	144	11	287	2375	1	2100	0	2898

TRINITY_DN15681_c0_g2_i5	Glyma.17G033700.1	100	144	11	287	2374	1	2101	0	2898

TRINITY_DN16138_c1_g1_i19	Glyma.08G183400.7	99.49494949	351	14	311	2402	1	2101	0	1788

TRINITY_DN16138_c1_g1_i19	Glyma.08G183400.8	99.49494949	351	14	311	2402	1	2101	0	1788

TRINITY_DN16138_c1_g1_i19	Glyma.08G183400.9	99.49494949	351	14	311	2402	1	2101	0	1788

TRINITY_DN15310_c0_g1_i2	Glyma.13G202800.1	98.68421053	351	19	407	2493	3	2106	0	1775

TRINITY_DN15981_c0_g1_i13	Glyma.17G161300.1	100	345	6	1354	3459	2336	231	0	1945

TRINITY_DN15981_c0_g1_i18	Glyma.17G161300.1	99.31506849	345	6	1244	3349	2336	231	0	1945

TRINITY_DN15981_c0_g1_i9	Glyma.17G161300.1	97.24409449	345	6	1480	3585	2336	231	0	1945

TRINITY_DN15981_c0_g1_i13	Glyma.05G105200.1	100	338	5	1354	3459	2336	231	0	1984

TRINITY_DN15981_c0_g1_i18	Glyma.05G105200.1	99	338	5	1244	3349	2336	231	0	1984

TRINITY_DN15981_c0_g1_i9	Glyma.05G105200.1	98.50746269	338	5	1480	3585	2336	231	0	1984

TRINITY_DN15137_c0_g1_i12	Glyma.14G222700.2	100	333	9	622	2721	2223	118	0	1973

TRINITY_DN15598_c0_g1_i3	Glyma.09G131500.1	97.89473684	272	7	43	2148	1	2100	0	2311

TRINITY_DN15598_c0_g1_i3	Glyma.16G178800.1	97.89473684	267	9	43	2148	1	2100	0	2338

TRINITY_DN15598_c0_g1_i3	Glyma.16G178800.2	97.89473684	267	9	43	2148	1	2100	0	2338

TRINITY_DN15660_c0_g1_i3	Glyma.18G074100.1	98.82352941	264	4	256	2355	2109	1	0	2374

TRINITY_DN15660_c0_g1_i2	Glyma.18G074100.1	96.812749	250	4	256	2355	2109	1	0	2451

TRINITY_DN16138_c1_g1_i8	Glyma.08G077100.1	98.41986456	213	7	300	2383	1	2107	0	2540

TRINITY_DN16138_c1_g1_i9	Glyma.08G077100.1	95.36082474	213	7	285	2368	1	2107	0	2540

TRINITY_DN16351_c0_g1_i3	Glyma.11G153500.1	96.61971831	205	6	80	2177	2103	1	0	2649

TRINITY_DN16351_c0_g1_i3	Glyma.11G153500.2	96.63865546	205	6	80	2177	2103	1	0	2649

TRINITY_DN16082_c0_g1_i6	Glyma.08G202000.2	95.37572254	205	8	552	2649	2107	1	0	2675

TRINITY_DN16351_c0_g1_i4	Glyma.11G153500.1	96.62921348	199	6	80	2177	2103	1	0	2682

TRINITY_DN16351_c0_g1_i4	Glyma.11G153500.2	96.61538462	199	6	80	2177	2103	1	0	2682

TRINITY_DN16351_c0_g1_i2	Glyma.11G153500.1	96.61654135	197	7	80	2164	2103	1	0	2610

TRINITY_DN16351_c0_g1_i2	Glyma.11G153500.2	96.63366337	197	7	80	2164	2103	1	0	2610

TRINITY_DN16082_c0_g1_i2	Glyma.08G202000.2	96.42857143	184	8	581	2678	2107	1	0	2791

TRINITY_DN7983_c0_g1_i1	Glyma.13G184200.1	95.79831933	184	8	171	2276	1	2100	0	2798

TRINITY_DN2205_c0_g1_i1	Glyma.20G181400.1	100	180	4	5	2108	2486	382	0	2839

TRINITY_DN15683_c0_g2_i1	Glyma.15G015300.1	95.73459716	175	4	166	2265	1	2106	0	2848

TRINITY_DN14027_c0_g1_i1	Glyma.10G173000.3	97.61904762	161	7	560	2666	2100	10	0	2874

TRINITY_DN14027_c0_g1_i1	Glyma.10G173000.2	95.91836735	161	7	560	2666	2100	10	0	2874

TRINITY_DN14027_c0_g1_i1	Glyma.10G173000.1	97.61904762	161	9	560	2666	2103	10	0	2892

TRINITY_DN14027_c0_g1_i1	Glyma.10G173000.4	97.4248927	161	10	560	2666	2097	10	0	2854

TRINITY_DN15143_c0_g2_i4	Glyma.07G089400.1	100	148	3	234	2342	1	2106	0	3055

TRINITY_DN16082_c0_g1_i12	Glyma.08G202000.4	100	422	50	121	2187	2265	184	0	1122

TRINITY_DN7394_c0_g1_i1	Glyma.14G015300.1	95.45454545	323	14	1	2098	2264	4364	0	1993

TRINITY_DN16138_c1_g1_i8	Glyma.15G049300.1	96.9924812	285	19	300	2383	1	2101	0	2113

TRINITY_DN16138_c1_g1_i9	Glyma.15G049300.1	97.93814433	285	19	285	2368	1	2101	0	2113

TRINITY_DN15099_c0_g1_i2	Glyma.13G261200.2	95.65217391	218	12	718	2797	126	2230	0	2484

TRINITY_DN3562_c0_g1_i1	Glyma.14G008900.1	98.5915493	126	4	414	2515	1	2105	0	3136

TRINITY_DN15683_c0_g2_i5	Glyma.15G015300.1	100	110	4	166	2264	1	2105	0	3206

TRINITY_DN13895_c0_g1_i1	Glyma.03G181400.4	100	376	54	556	2604	3858	1783	0	1232

TRINITY_DN13895_c0_g1_i1	Glyma.03G181400.1	98.7804878	376	54	556	2604	3858	1783	0	1232

TRINITY_DN13895_c0_g1_i1	Glyma.03G181400.2	95.94594595	376	54	556	2604	3858	1783	0	1232

TRINITY_DN13895_c0_g1_i1	Glyma.03G181400.3	96.96356275	376	54	556	2604	3858	1783	0	1232

TRINITY_DN5582_c0_g4_i1	Glyma.08G057400.1	97.56097561	351	26	1	2073	3	2099	0	1663

TRINITY_DN2169_c0_g1_i1	Glyma.11G009900.1	97.89473684	242	26	747	2819	2088	1	0	2207

TRINITY_DN16132_c0_g1_i3	Glyma.06G056400.1	97.05882353	226	4	721	2826	84	2186	0	2606

TRINITY_DN16132_c0_g1_i3	Glyma.06G056400.4	97.91666667	226	4	721	2826	84	2186	0	2606

TRINITY_DN16132_c0_g1_i3	Glyma.06G056400.3	95.80838323	226	5	721	2826	84	2180	0	2567

TRINITY_DN16436_c0_g2_i13	Glyma.11G131600.3	100	199	5	678	2770	1	2105	0	2686

TRINITY_DN6786_c0_g1_i1	Glyma.07G016100.1	99.70760234	182	15	55	2139	74	2170	0	2676

TRINITY_DN15683_c0_g2_i1	Glyma.13G358700.1	95.73459716	177	3	166	2263	1	2104	0	2833

TRINITY_DN7983_c0_g1_i1	Glyma.07G192200.1	95.78947368	172	5	171	2276	1	2100	0	2887

TRINITY_DN4747_c0_g1_i1	Glyma.13G360500.1	96.66666667	170	3	99	2201	2106	1	0	2916

TRINITY_DN4747_c0_g1_i1	Glyma.15G013400.2	100	167	3	99	2201	2106	1	0	2933

TRINITY_DN4747_c0_g1_i1	Glyma.15G013400.3	96.875	167	3	99	2201	2106	1	0	2933

TRINITY_DN4747_c0_g1_i1	Glyma.15G013400.1	100	167	3	99	2201	2106	1	0	2933

TRINITY_DN12553_c0_g1_i2	Glyma.08G012400.1	96.15384615	166	14	48	2141	33	2123	0	2784

TRINITY_DN12553_c0_g1_i3	Glyma.08G012400.1	98.58429858	165	18	48	2122	33	2123	0	2667

TRINITY_DN12659_c0_g1_i1	Glyma.06G238000.12	98.50746269	163	3	164	2266	1	2106	0	2955

TRINITY_DN12659_c0_g1_i1	Glyma.06G238000.1	98.50498339	163	3	164	2266	1	2106	0	2955

TRINITY_DN12659_c0_g1_i1	Glyma.06G238000.7	98.4962406	163	3	164	2266	1	2106	0	2955

TRINITY_DN12659_c0_g1_i1	Glyma.06G238000.3	98.50746269	163	3	164	2266	1	2106	0	2955

TRINITY_DN12659_c0_g1_i1	Glyma.U025600.12	100	159	4	164	2266	1	2103	0	2959

TRINITY_DN16138_c1_g1_i19	Glyma.08G077100.1	100	155	2	311	2402	1	2107	0	2935

TRINITY_DN14727_c0_g1_i1	Glyma.04G114800.1	98.33333333	152	3	570	2676	2285	185	0	3011

TRINITY_DN15683_c0_g2_i5	Glyma.13G358700.1	96.47887324	121	3	166	2263	1	2104	0	3144

TRINITY_DN16275_c0_g1_i2	Glyma.13G030300.2	98.11320755	359	14	652	2751	19	2115	0	1803

TRINITY_DN15137_c0_g1_i2	Glyma.14G222700.2	97.14285714	316	9	597	2693	2220	118	0	2061

TRINITY_DN15137_c0_g1_i3	Glyma.14G222700.1	97.82945736	316	9	683	2779	2220	118	0	2061

TRINITY_DN13448_c0_g1_i1	Glyma.11G246200.8	98.94736842	300	0	17	2122	43	2148	0	2228

TRINITY_DN13448_c0_g1_i6	Glyma.11G246200.8	97.82608696	300	0	17	2122	43	2148	0	2228

TRINITY_DN13902_c0_g1_i5	Glyma.11G219700.1	100	265	8	6	2102	58	2157	0	2324

TRINITY_DN15958_c0_g1_i3	Glyma.12G155400.1	96.14457831	229	15	412	2490	2100	1	0	2407

TRINITY_DN15958_c0_g1_i4	Glyma.12G155400.1	98.33887043	229	15	412	2490	2100	1	0	2407

TRINITY_DN15681_c0_g2_i1	Glyma.07G239600.2	98.48484848	212	11	287	2384	1	2092	0	2573

TRINITY_DN15681_c0_g2_i3	Glyma.07G239600.2	100	212	11	287	2384	1	2092	0	2573

TRINITY_DN35853_c0_g1_i1	Glyma.18G175700.1	99.33774834	172	18	30	2111	1	2100	0	2743

TRINITY_DN9498_c0_g1_i5	Glyma.02G251500.2	99.20634921	141	4	2	2104	11	2110	0	3049

TRINITY_DN9498_c0_g1_i5	Glyma.02G251500.1	100	141	4	2	2104	11	2110	0	3049

TRINITY_DN15451_c0_g1_i1	Glyma.10G053500.1	96.83257919	356	36	536	2603	2078	14	0	1417

TRINITY_DN15451_c0_g1_i3	Glyma.10G053500.1	98.7012987	356	36	536	2603	2078	14	0	1417

TRINITY_DN16138_c1_g1_i8	Glyma.08G183400.7	99.59839357	307	11	300	2383	1	2101	0	2025

TRINITY_DN16138_c1_g1_i8	Glyma.08G183400.8	100	307	11	300	2383	1	2101	0	2025

TRINITY_DN16138_c1_g1_i8	Glyma.08G183400.9	100	307	11	300	2383	1	2101	0	2025

TRINITY_DN16138_c1_g1_i9	Glyma.08G183400.7	97.87234043	307	11	285	2368	1	2101	0	2025

TRINITY_DN16138_c1_g1_i9	Glyma.08G183400.8	95.04950495	307	11	285	2368	1	2101	0	2025

TRINITY_DN16138_c1_g1_i9	Glyma.08G183400.9	95.04950495	307	11	285	2368	1	2101	0	2025

TRINITY_DN12149_c0_g1_i1	Glyma.17G251300.1	97.36842105	215	51	221	2272	2082	1	0	2206

TRINITY_DN15521_c0_g1_i2	Glyma.06G226700.1	100	155	4	1129	3231	936	3038	0	3003

TRINITY_DN15521_c0_g1_i2	Glyma.06G226700.2	98.95833333	155	4	1129	3231	792	2894	0	3003

TRINITY_DN15981_c0_g1_i13	Glyma.01G193600.2	99.34210526	132	1	1354	3455	2336	232	0	3136

TRINITY_DN15981_c0_g1_i13	Glyma.01G193600.1	99.0521327	132	1	1354	3455	2336	232	0	3136

TRINITY_DN15981_c0_g1_i18	Glyma.01G193600.2	99.76635514	132	1	1244	3345	2336	232	0	3136

TRINITY_DN15981_c0_g1_i18	Glyma.01G193600.1	95	132	1	1244	3345	2336	232	0	3136

TRINITY_DN15981_c0_g1_i9	Glyma.01G193600.2	95.9697733	132	1	1480	3581	2336	232	0	3136

TRINITY_DN15981_c0_g1_i9	Glyma.01G193600.1	97.77777778	132	1	1480	3581	2336	232	0	3136

TRINITY_DN3562_c0_g1_i1	Glyma.02G305000.2	98.5915493	125	4	414	2515	1	2099	0	3136

TRINITY_DN3562_c0_g1_i1	Glyma.02G305000.1	96.01910828	125	4	414	2515	1	2099	0	3136

TRINITY_DN16071_c0_g2_i1	Glyma.05G132900.1	98.7654321	120	1	180	2283	881	2985	0	3216

TRINITY_DN16071_c0_g2_i1	Glyma.08G087300.1	96	118	1	180	2283	881	2985	0	3227

TRINITY_DN15521_c0_g1_i2	Glyma.13G266300.1	100	312	34	1129	3215	957	3037	0	1899

TRINITY_DN15403_c0_g2_i4	Glyma.10G178100.1	97.6744186	222	22	770	2838	3	2102	0	2403

TRINITY_DN5558_c0_g2_i1	Glyma.17G255300.3	95	176	16	441	2529	2092	1	0	2736

TRINITY_DN5558_c0_g2_i1	Glyma.17G255300.1	98.98989899	176	16	441	2529	2092	1	0	2736

TRINITY_DN12659_c0_g1_i1	Glyma.06G238000.6	98.5042735	163	2	164	2266	1	2103	0	2970

TRINITY_DN12659_c0_g1_i1	Glyma.06G238000.15	98.50187266	163	2	164	2266	1	2103	0	2970

TRINITY_DN12659_c0_g1_i1	Glyma.06G238000.2	98.50746269	163	2	164	2266	1	2103	0	2970

TRINITY_DN12659_c0_g1_i1	Glyma.06G238000.11	98.48484848	163	2	164	2266	1	2103	0	2970

TRINITY_DN2658_c0_g1_i1	Glyma.07G232200.6	99	158	28	243	2304	2148	67	0	2597

TRINITY_DN14972_c0_g1_i2	Glyma.17G038400.2	98.02306425	154	15	340	2412	1	2085	0	2710

TRINITY_DN14972_c0_g1_i2	Glyma.17G038400.1	98.03921569	154	15	340	2412	1	2085	0	2710

TRINITY_DN15681_c0_g2_i5	Glyma.07G239600.1	95.65217391	149	6	287	2374	1	2098	0	2918

TRINITY_DN143_c0_g1_i1	Glyma.08G001900.1	98.89135255	140	3	219	2321	3093	997	0	3059

TRINITY_DN11825_c0_g2_i1	Glyma.05G044200.1	98.57142857	126	1	376	2479	2101	1	0	3168

TRINITY_DN11825_c0_g2_i3	Glyma.05G044200.1	98.57142857	126	1	376	2479	2101	1	0	3168

TRINITY_DN15912_c1_g1_i2	Glyma.08G204700.1	100	310	10	390	2482	2247	150	0	2069

TRINITY_DN15970_c0_g1_i4	Glyma.18G034300.1	96.12403101	284	6	165	2264	40	2139	0	2272

TRINITY_DN15852_c0_g1_i2	Glyma.10G229900.1	96	276	12	430	2517	1	2094	0	2200

TRINITY_DN15852_c0_g1_i3	Glyma.10G229900.1	97.43589744	276	12	337	2424	1	2094	0	2200

TRINITY_DN15852_c0_g1_i6	Glyma.10G229900.1	97.33333333	276	12	414	2501	1	2094	0	2200

TRINITY_DN15918_c0_g1_i2	Glyma.08G247500.2	99.16666667	244	8	312	2395	1	2093	0	2344

TRINITY_DN15918_c0_g1_i2	Glyma.08G247500.1	99.16666667	244	8	312	2395	1	2093	0	2344

TRINITY_DN15918_c0_g1_i3	Glyma.08G247500.2	97.70114943	244	8	593	2676	1	2093	0	2344

TRINITY_DN15918_c0_g1_i3	Glyma.08G247500.1	96.93251534	244	8	593	2676	1	2093	0	2344

TRINITY_DN16082_c0_g1_i6	Glyma.07G016700.2	95.9375	200	11	552	2624	2101	1	0	2569

TRINITY_DN12460_c0_g1_i2	Glyma.13G346200.9	99.5951417	189	1	1213	3312	2309	207	0	2817

TRINITY_DN12460_c0_g1_i2	Glyma.13G346200.10	97.95918367	189	1	1213	3312	2309	207	0	2817

TRINITY_DN12460_c0_g1_i2	Glyma.13G346200.1	95.45454545	189	1	1213	3312	2309	207	0	2817

TRINITY_DN12460_c0_g1_i2	Glyma.13G346200.4	100	189	1	1213	3312	2309	207	0	2817

TRINITY_DN12460_c0_g1_i1	Glyma.13G346200.9	95.6043956	188	2	1213	3300	2309	207	0	2745

TRINITY_DN12460_c0_g1_i1	Glyma.13G346200.10	99.41860465	188	2	1213	3300	2309	207	0	2745

TRINITY_DN12460_c0_g1_i1	Glyma.13G346200.1	98.31460674	188	2	1213	3300	2309	207	0	2745

TRINITY_DN12460_c0_g1_i1	Glyma.13G346200.4	95.45454545	188	2	1213	3300	2309	207	0	2745

TRINITY_DN12460_c0_g1_i2	Glyma.13G346200.7	100	188	2	1213	3312	2297	207	0	2745

TRINITY_DN12460_c0_g1_i2	Glyma.13G346200.8	100	188	2	1213	3312	2297	207	0	2745

TRINITY_DN12460_c0_g1_i2	Glyma.13G346200.2	99.15966387	188	2	1213	3312	2297	207	0	2745

TRINITY_DN12460_c0_g1_i2	Glyma.13G346200.3	95.97585513	188	2	1213	3312	2297	207	0	2745

TRINITY_DN16082_c0_g1_i2	Glyma.07G016700.2	96.91991786	178	11	581	2653	2101	1	0	2691

TRINITY_DN14972_c0_g1_i2	Glyma.07G233400.1	98.03921569	152	10	340	2412	1	2088	0	2750

TRINITY_DN14415_c0_g1_i2	Glyma.02G073100.2	95.65217391	151	13	246	2342	3	2079	0	2841

TRINITY_DN14415_c0_g1_i2	Glyma.02G073100.3	100	151	13	246	2342	3	2079	0	2841

TRINITY_DN14415_c0_g1_i2	Glyma.02G073100.1	100	151	13	246	2342	3	2079	0	2841

TRINITY_DN14415_c0_g1_i3	Glyma.02G073100.2	98.5472155	151	13	246	2342	3	2079	0	2841

TRINITY_DN14415_c0_g1_i3	Glyma.02G073100.3	97.40932642	151	13	246	2342	3	2079	0	2841

TRINITY_DN14415_c0_g1_i3	Glyma.02G073100.1	97	151	13	246	2342	3	2079	0	2841

TRINITY_DN15521_c0_g1_i2	Glyma.12G155900.1	98.95833333	145	0	1129	3231	933	3035	0	3081

TRINITY_DN15415_c0_g1_i3	Glyma.08G282800.2	100	116	0	161	2263	1	2103	0	3241

TRINITY_DN9349_c0_g1_i1	Glyma.18G262600.1	100	293	10	232	2318	1	2099	0	2143

TRINITY_DN9349_c0_g1_i1	Glyma.18G262600.2	97.52066116	293	10	232	2318	1	2099	0	2143

TRINITY_DN15918_c0_g1_i2	Glyma.18G268900.1	99.16666667	250	5	312	2395	1	2096	0	2342

TRINITY_DN15918_c0_g1_i2	Glyma.18G268900.3	99.16666667	250	5	312	2395	1	2096	0	2342

TRINITY_DN15918_c0_g1_i3	Glyma.18G268900.1	96.93251534	250	5	593	2676	1	2096	0	2342

TRINITY_DN15918_c0_g1_i3	Glyma.18G268900.3	98.84057971	250	5	593	2676	1	2096	0	2342

TRINITY_DN15970_c0_g1_i3	Glyma.18G034300.1	96.875	228	3	165	2264	40	2139	0	2593

TRINITY_DN16274_c0_g1_i2	Glyma.13G312300.2	100	201	3	1196	3296	1	2074	0	2580

TRINITY_DN4160_c0_g2_i1	Glyma.20G189100.3	98.30508475	163	2	98	2196	262	2360	0	2940

TRINITY_DN4160_c0_g2_i1	Glyma.20G189100.2	98.92473118	163	2	98	2196	190	2288	0	2940

TRINITY_DN15660_c0_g1_i2	Glyma.02G302500.1	96.41255605	139	5	256	2351	2109	8	0	3073

TRINITY_DN15660_c0_g1_i3	Glyma.02G302500.1	100	123	5	256	2351	2109	8	0	3162

TRINITY_DN14813_c0_g1_i1	Glyma.U016100.1	98.73873874	103	4	584	2681	2093	1	0	3227

TRINITY_DN16275_c0_g1_i5	Glyma.13G030300.2	99.5951417	321	8	652	2749	19	2113	0	2043

TRINITY_DN15660_c0_g1_i3	Glyma.08G332900.1	100	272	2	256	2355	2100	1	0	2361

TRINITY_DN15751_c0_g1_i1	Glyma.06G172300.2	96.875	272	28	890	2962	1	2082	0	2069

TRINITY_DN15751_c0_g1_i1	Glyma.06G172300.3	96.875	272	28	890	2962	1	2082	0	2069

TRINITY_DN15751_c0_g1_i1	Glyma.06G172300.4	96.875	272	28	890	2962	1	2082	0	2069

TRINITY_DN15751_c0_g1_i1	Glyma.06G172300.1	96.875	272	28	890	2962	1	2082	0	2069

TRINITY_DN13490_c0_g3_i2	Glyma.12G223200.1	98.82352941	267	1	241	2341	2098	1	0	2381

TRINITY_DN15660_c0_g1_i2	Glyma.08G332900.1	100	261	2	256	2355	2100	1	0	2422

TRINITY_DN13490_c0_g3_i2	Glyma.13G278300.2	95.44072948	251	1	241	2341	2098	1	0	2470

TRINITY_DN13490_c0_g3_i2	Glyma.13G278300.1	98.9010989	251	1	241	2341	2098	1	0	2470

TRINITY_DN6168_c0_g1_i1	Glyma.20G227000.4	98.7012987	187	12	109	2196	22	2100	0	2617

TRINITY_DN7394_c0_g1_i1	Glyma.08G326600.1	95.43568465	158	14	3	2099	2299	4377	0	2837

TRINITY_DN15016_c0_g1_i3	Glyma.20G160200.1	95.85858586	146	11	115	2203	886	2974	0	2916

TRINITY_DN15931_c0_g2_i2	Glyma.10G065900.1	98.86363636	144	8	3772	5845	3938	6035	0	2889

TRINITY_DN8924_c0_g1_i1	Glyma.01G227500.1	100	295	23	2	2078	706	2797	0	2043

TRINITY_DN8924_c0_g1_i1	Glyma.01G227500.3	99.41860465	295	23	2	2078	703	2794	0	2043

TRINITY_DN15451_c0_g1_i7	Glyma.19G181900.1	98.62068966	279	33	420	2503	2078	40	0	1834

TRINITY_DN15451_c0_g1_i8	Glyma.19G181900.1	100	279	33	420	2503	2078	40	0	1834

TRINITY_DN13902_c0_g1_i5	Glyma.18G037700.1	95.3038674	271	2	6	2102	58	2154	0	2338

TRINITY_DN16360_c0_g8_i5	Glyma.01G170200.1	99.34640523	257	13	197	2277	1	2099	0	2326

TRINITY_DN10463_c0_g1_i1	Glyma.19G235100.1	100	175	5	374	2471	2089	1	0	2824

TRINITY_DN8924_c0_g1_i1	Glyma.11G094500.1	99.44134078	168	4	2	2095	736	2835	0	2909

TRINITY_DN8924_c0_g1_i1	Glyma.11G094500.2	99.43181818	168	4	2	2095	163	2262	0	2909

TRINITY_DN8924_c0_g1_i1	Glyma.11G094500.3	99.42196532	168	4	2	2095	163	2262	0	2909

TRINITY_DN7394_c0_g1_i1	Glyma.08G326500.1	95.38188277	152	2	3	2099	2299	4398	0	3016

TRINITY_DN13490_c0_g3_i2	Glyma.06G246600.1	100	113	0	242	2341	2100	1	0	3253

TRINITY_DN13490_c0_g3_i2	Glyma.12G150800.1	100	111	0	242	2341	2100	1	0	3264

TRINITY_DN10575_c0_g1_i1	Glyma.15G236500.3	100	105	7	47	2146	16	2103	0	3219

TRINITY_DN16082_c0_g1_i1	Glyma.08G124400.2	100	286	8	581	2674	2142	49	0	2228

TRINITY_DN12659_c0_g1_i1	Glyma.13G274500.5	98.48484848	282	13	164	2255	1	2077	0	2126

TRINITY_DN12659_c0_g1_i1	Glyma.13G274500.3	98.0952381	282	13	164	2255	1	2077	0	2126

TRINITY_DN12659_c0_g1_i1	Glyma.13G274500.4	98.11320755	282	13	164	2255	1	2077	0	2126

TRINITY_DN12659_c0_g1_i1	Glyma.13G274500.2	98.0952381	282	13	164	2255	1	2077	0	2126

TRINITY_DN12659_c0_g1_i1	Glyma.13G274500.1	98.11320755	282	13	164	2255	1	2077	0	2126

TRINITY_DN16254_c0_g1_i8	Glyma.17G057800.4	95.86206897	269	10	618	2709	3048	955	0	2309

TRINITY_DN16254_c0_g1_i8	Glyma.17G057800.3	100	269	10	618	2709	3048	955	0	2309

TRINITY_DN16254_c0_g1_i8	Glyma.17G057800.2	100	269	10	618	2709	3048	955	0	2309

TRINITY_DN3099_c0_g1_i1	Glyma.12G001300.1	95.52238806	227	14	366	2449	2104	12	0	2483

TRINITY_DN15751_c0_g1_i6	Glyma.06G172300.2	97.36842105	192	23	890	2965	1	2082	0	2555

TRINITY_DN15751_c0_g1_i6	Glyma.06G172300.3	95.09803922	192	23	890	2965	1	2082	0	2555

TRINITY_DN15751_c0_g1_i6	Glyma.06G172300.4	95.09803922	192	23	890	2965	1	2082	0	2555

TRINITY_DN15751_c0_g1_i6	Glyma.06G172300.1	95.09803922	192	23	890	2965	1	2082	0	2555

TRINITY_DN12460_c0_g1_i2	Glyma.13G346200.5	100	188	1	1213	3308	2099	1	0	2815

TRINITY_DN12460_c0_g1_i2	Glyma.13G346200.6	100	188	1	1213	3308	2099	1	0	2815

TRINITY_DN12460_c0_g1_i1	Glyma.13G346200.5	98.46153846	187	2	1213	3296	2099	1	0	2743

TRINITY_DN12460_c0_g1_i1	Glyma.13G346200.6	97.87234043	187	2	1213	3296	2099	1	0	2743

TRINITY_DN14801_c0_g2_i1	Glyma.13G031300.1	99.05660377	187	22	285	2373	2296	229	0	2575

TRINITY_DN15241_c0_g1_i2	Glyma.08G165800.1	100	184	6	1986	4072	2096	1	0	2760

TRINITY_DN15241_c0_g1_i2	Glyma.08G165800.2	100	184	6	1986	4072	2096	1	0	2760

TRINITY_DN12460_c0_g1_i2	Glyma.15G028000.1	99.5951417	183	2	1213	3308	2096	1	0	2824

TRINITY_DN12460_c0_g1_i1	Glyma.15G028000.1	96.15384615	182	3	1213	3296	2096	1	0	2752

TRINITY_DN13398_c0_g1_i1	Glyma.16G096600.2	100	153	9	676	2762	2549	457	0	2913

TRINITY_DN2636_c0_g2_i1	Glyma.02G032800.1	95.99198397	149	9	256	2348	2127	38	0	2953

TRINITY_DN15016_c0_g1_i3	Glyma.10G234100.1	95.48022599	143	12	115	2203	892	2974	0	2916

TRINITY_DN14312_c0_g1_i4	Glyma.01G229200.2	97.40740741	131	6	1037	3135	2085	2	0	3053

TRINITY_DN15852_c0_g1_i2	Glyma.20G163400.3	99.44134078	277	5	430	2517	1	2094	0	2250

TRINITY_DN15852_c0_g1_i2	Glyma.20G163400.1	99.44134078	277	5	430	2517	1	2094	0	2250

TRINITY_DN15852_c0_g1_i2	Glyma.20G163400.2	96	277	5	430	2517	1	2094	0	2250

TRINITY_DN15852_c0_g1_i3	Glyma.20G163400.3	96.20253165	277	5	337	2424	1	2094	0	2250

TRINITY_DN15852_c0_g1_i3	Glyma.20G163400.1	97.43589744	277	5	337	2424	1	2094	0	2250

TRINITY_DN15852_c0_g1_i3	Glyma.20G163400.2	97.43589744	277	5	337	2424	1	2094	0	2250

TRINITY_DN15852_c0_g1_i6	Glyma.20G163400.3	97.33333333	277	5	414	2501	1	2094	0	2250

TRINITY_DN15852_c0_g1_i6	Glyma.20G163400.1	97.33333333	277	5	414	2501	1	2094	0	2250

TRINITY_DN15852_c0_g1_i6	Glyma.20G163400.2	97.33333333	277	5	414	2501	1	2094	0	2250

TRINITY_DN16082_c0_g1_i5	Glyma.08G124400.2	99.3006993	245	6	581	2674	2142	49	0	2466

TRINITY_DN11332_c0_g1_i1	Glyma.18G130800.1	95.91141397	205	27	1	2059	116	2198	0	2390

TRINITY_DN35682_c0_g1_i1	Glyma.06G175200.1	98.26589595	202	8	229	2323	2118	27	0	2697

TRINITY_DN13746_c0_g2_i1	Glyma.20G233200.2	96	195	21	550	2619	2579	507	0	2451

TRINITY_DN8924_c0_g1_i1	Glyma.12G020900.1	99.41860465	164	3	2	2095	721	2817	0	2933

TRINITY_DN6859_c0_g1_i2	Glyma.18G010200.1	97.64705882	155	2	99	2195	2127	31	0	3003

TRINITY_DN15681_c0_g2_i2	Glyma.07G239600.2	98.98989899	149	5	287	2369	1	2092	0	2913

TRINITY_DN15681_c0_g2_i5	Glyma.07G239600.2	100	149	5	287	2369	1	2092	0	2913

TRINITY_DN15345_c0_g1_i3	Glyma.13G308400.4	97.88135593	148	1	659	2756	1	2090	0	3003

TRINITY_DN15345_c0_g1_i3	Glyma.13G308400.3	95.76271186	148	1	659	2756	1	2090	0	3003

TRINITY_DN15345_c0_g1_i4	Glyma.13G308400.4	95.12195122	148	1	465	2562	1	2090	0	3003

TRINITY_DN15345_c0_g1_i4	Glyma.13G308400.3	100	148	1	465	2562	1	2090	0	3003

TRINITY_DN11385_c0_g1_i2	Glyma.10G124200.2	96.77419355	104	0	155	2252	2098	1	0	3299

TRINITY_DN11176_c0_g1_i1	Glyma.20G012600.1	99.18699187	368	38	464	2533	490	2541	0	1369

TRINITY_DN13654_c0_g1_i1	Glyma.07G046800.1	97.15909091	312	27	343	2416	2123	69	0	1724

TRINITY_DN16130_c2_g6_i14	Glyma.19G196000.2	100	221	1	410	2505	109	2205	0	2641

TRINITY_DN16130_c2_g6_i17	Glyma.19G196000.2	100	221	1	410	2505	109	2205	0	2641

TRINITY_DN16130_c2_g6_i4	Glyma.19G196000.2	98.80952381	173	1	410	2505	109	2205	0	2907

TRINITY_DN16130_c2_g6_i8	Glyma.19G196000.2	98.33333333	173	1	410	2505	109	2205	0	2907

TRINITY_DN2638_c0_g2_i1	Glyma.10G249700.1	96.64179104	163	3	147	2243	1	2091	0	2931

TRINITY_DN2638_c0_g2_i1	Glyma.20G143900.1	96.66666667	156	0	147	2243	1	2097	0	3009

TRINITY_DN11082_c0_g1_i1	Glyma.15G023200.2	98.30508475	134	3	1012	3099	2098	2	0	3072

TRINITY_DN11082_c0_g1_i1	Glyma.15G023200.1	98.30508475	134	3	1012	3099	2098	2	0	3072

TRINITY_DN3145_c0_g1_i1	Glyma.10G243900.2	95.69892473	134	4	237	2330	1	2091	0	3072

TRINITY_DN5798_c0_g1_i1	Glyma.09G079100.4	96.80851064	112	3	860	2949	2100	5	0	3201

TRINITY_DN5798_c0_g1_i1	Glyma.09G079100.5	100	112	3	860	2949	2100	5	0	3201

TRINITY_DN16082_c0_g1_i12	Glyma.08G202000.2	100	424	44	121	2187	2253	184	0	1166

TRINITY_DN11176_c0_g1_i1	Glyma.07G174100.1	100	361	43	464	2533	463	2508	0	1380

TRINITY_DN13654_c0_g1_i1	Glyma.16G015400.1	97.1563981	309	34	347	2416	2123	72	0	1707

TRINITY_DN15501_c0_g2_i6	Glyma.13G162800.3	100	268	2	42	2136	2095	1	0	2374

TRINITY_DN15501_c0_g2_i1	Glyma.13G162800.3	100	265	2	507	2601	2095	1	0	2390

TRINITY_DN16082_c0_g1_i8	Glyma.08G124400.2	98.11320755	242	4	581	2674	2142	49	0	2505

TRINITY_DN16071_c0_g2_i1	Glyma.09G052000.1	98.7654321	237	10	193	2280	868	2959	0	2481

TRINITY_DN13751_c0_g1_i2	Glyma.19G163500.1	98.13664596	218	12	543	2632	1	2084	0	2547

TRINITY_DN16082_c0_g1_i11	Glyma.08G124400.2	97.36842105	199	4	581	2674	2142	49	0	2743

TRINITY_DN16252_c0_g1_i1	Glyma.08G293600.4	100	195	8	1665	3747	21	2115	0	2700

TRINITY_DN16252_c0_g1_i3	Glyma.08G293600.4	97.37991266	195	8	1665	3747	21	2115	0	2700

TRINITY_DN13097_c0_g1_i3	Glyma.02G292300.1	100	195	10	194	2278	2091	1	0	2687

TRINITY_DN15024_c0_g1_i3	Glyma.04G114300.1	100	193	44	521	2545	2064	1	0	2135

TRINITY_DN15660_c0_g1_i2	Glyma.14G011600.1	97.72727273	158	0	256	2351	2103	8	0	2996

TRINITY_DN14801_c0_g3_i3	Glyma.06G195700.1	100	145	7	366	2459	2133	43	0	3022

TRINITY_DN15660_c0_g1_i3	Glyma.14G011600.1	98.82352941	141	0	256	2351	2103	8	0	3090

TRINITY_DN13604_c0_g1_i4	Glyma.11G218000.1	100	113	2	139	2233	357	2451	0	3232

TRINITY_DN14464_c0_g1_i1	Glyma.09G260100.1	99.14529915	113	7	319	2399	2090	1	0	3109

TRINITY_DN14464_c0_g1_i2	Glyma.09G260100.1	96.74796748	113	7	319	2399	2090	1	0	3109

TRINITY_DN9638_c0_g1_i2	Glyma.16G181700.1	100	397	40	338	2383	2156	90	0	1171

TRINITY_DN15506_c0_g2_i1	Glyma.07G196500.1	100	364	20	871	2953	1	2086	0	1716

TRINITY_DN15506_c0_g2_i10	Glyma.07G196500.1	97.52475248	364	20	954	3036	1	2086	0	1716

TRINITY_DN15506_c0_g2_i14	Glyma.07G196500.1	100	364	20	953	3035	1	2086	0	1716

TRINITY_DN15506_c0_g2_i2	Glyma.07G196500.1	95.53571429	364	20	1268	3350	1	2086	0	1716

TRINITY_DN15506_c0_g2_i7	Glyma.07G196500.1	96.55172414	364	20	1036	3118	1	2086	0	1716

TRINITY_DN15506_c0_g2_i9	Glyma.07G196500.1	100	364	20	1185	3267	1	2086	0	1716

TRINITY_DN15137_c0_g1_i8	Glyma.04G019700.1	97.48653501	318	8	683	2773	2115	25	0	2056

TRINITY_DN16308_c1_g3_i2	Glyma.13G072200.1	100	316	20	906	2988	112	2194	0	1964

TRINITY_DN16071_c0_g2_i1	Glyma.15G158300.1	98.7654321	237	9	193	2280	898	2989	0	2492

TRINITY_DN9350_c0_g1_i1	Glyma.10G019200.10	96.40831758	227	7	154	2246	1	2084	0	2527

TRINITY_DN15024_c0_g1_i3	Glyma.06G321000.4	100	193	48	521	2545	2061	1	0	2128

TRINITY_DN15024_c0_g1_i3	Glyma.06G321000.2	98.5915493	193	48	521	2545	2061	1	0	2128

TRINITY_DN15024_c0_g1_i3	Glyma.06G321000.3	96.58385093	193	48	521	2545	2061	1	0	2128

TRINITY_DN9796_c0_g1_i4	Glyma.11G035800.1	99.21259843	192	10	296	2378	3093	1005	0	2689

TRINITY_DN9796_c0_g1_i5	Glyma.11G035800.1	99.21875	192	10	296	2378	3093	1005	0	2689

TRINITY_DN16254_c0_g1_i8	Glyma.09G061200.4	98.1981982	180	7	618	2709	3063	973	0	2826

TRINITY_DN16254_c0_g1_i8	Glyma.09G061200.3	99.12280702	180	7	618	2709	3063	973	0	2826

TRINITY_DN16254_c0_g1_i8	Glyma.09G061200.1	100	180	7	618	2709	3063	973	0	2826

TRINITY_DN16254_c0_g1_i8	Glyma.09G061200.2	95.23809524	180	7	618	2709	3063	973	0	2826

TRINITY_DN16254_c0_g1_i8	Glyma.09G061200.5	98.27586207	180	7	618	2709	3063	973	0	2826

TRINITY_DN15241_c0_g1_i2	Glyma.15G261200.4	97.89473684	179	11	1991	4072	2086	1	0	2736

TRINITY_DN15241_c0_g1_i2	Glyma.15G261200.2	96.15384615	179	11	1991	4072	2086	1	0	2736

TRINITY_DN15241_c0_g1_i2	Glyma.15G261200.1	95.08196721	179	11	1991	4072	2086	1	0	2736

TRINITY_DN15241_c0_g1_i2	Glyma.15G261200.3	95.19650655	179	11	1991	4072	2086	1	0	2736

TRINITY_DN16006_c0_g1_i1	Glyma.09G052600.4	97.02380952	175	13	266	2353	2070	1	0	2693

TRINITY_DN5646_c0_g1_i1	Glyma.19G135300.2	98.61386139	168	13	969	3050	1780	3843	0	2654

TRINITY_DN5646_c0_g1_i2	Glyma.19G135300.2	100	168	13	969	3050	1780	3843	0	2654

TRINITY_DN13639_c0_g1_i2	Glyma.12G181900.1	100	161	12	318	2378	2085	1	0	2693

TRINITY_DN11692_c0_g1_i2	Glyma.13G349600.1	97.43589744	160	8	34	2126	21	2104	0	2898

TRINITY_DN12719_c0_g2_i1	Glyma.13G321300.42	96.875	160	20	399	2482	2050	6	0	2588

TRINITY_DN12719_c0_g2_i1	Glyma.13G321300.30	95.68965517	160	20	399	2482	2050	6	0	2588

TRINITY_DN12719_c0_g2_i1	Glyma.13G321300.10	96	160	20	399	2482	2050	6	0	2588

TRINITY_DN12719_c0_g2_i1	Glyma.13G321300.43	97.84482759	160	20	399	2482	2050	6	0	2588

TRINITY_DN12719_c0_g2_i1	Glyma.13G321300.31	98.73417722	160	20	399	2482	2050	6	0	2588

TRINITY_DN12719_c0_g2_i1	Glyma.13G321300.11	96.82539683	160	20	399	2482	2050	6	0	2588

TRINITY_DN12719_c0_g2_i2	Glyma.13G321300.42	100	160	20	500	2583	2050	6	0	2588

TRINITY_DN12719_c0_g2_i2	Glyma.13G321300.30	97.14285714	160	20	500	2583	2050	6	0	2588

TRINITY_DN12719_c0_g2_i2	Glyma.13G321300.10	98.63013699	160	20	500	2583	2050	6	0	2588

TRINITY_DN12719_c0_g2_i2	Glyma.13G321300.43	98.47715736	160	20	500	2583	2050	6	0	2588

TRINITY_DN12719_c0_g2_i2	Glyma.13G321300.31	97.36842105	160	20	500	2583	2050	6	0	2588

TRINITY_DN12719_c0_g2_i2	Glyma.13G321300.11	97.36842105	160	20	500	2583	2050	6	0	2588

TRINITY_DN12719_c0_g2_i3	Glyma.13G321300.42	98.7012987	160	20	399	2482	2050	6	0	2588

TRINITY_DN12719_c0_g2_i3	Glyma.13G321300.30	100	160	20	399	2482	2050	6	0	2588

TRINITY_DN12719_c0_g2_i3	Glyma.13G321300.10	100	160	20	399	2482	2050	6	0	2588

TRINITY_DN12719_c0_g2_i3	Glyma.13G321300.43	96.35416667	160	20	399	2482	2050	6	0	2588

TRINITY_DN12719_c0_g2_i3	Glyma.13G321300.31	98.68421053	160	20	399	2482	2050	6	0	2588

TRINITY_DN12719_c0_g2_i3	Glyma.13G321300.11	97.03703704	160	20	399	2482	2050	6	0	2588

TRINITY_DN12719_c0_g2_i4	Glyma.13G321300.42	96.13259669	160	20	500	2583	2050	6	0	2588

TRINITY_DN12719_c0_g2_i4	Glyma.13G321300.30	99.49748744	160	20	500	2583	2050	6	0	2588

TRINITY_DN12719_c0_g2_i4	Glyma.13G321300.10	95.45454545	160	20	500	2583	2050	6	0	2588

TRINITY_DN12719_c0_g2_i4	Glyma.13G321300.43	98.82352941	160	20	500	2583	2050	6	0	2588

TRINITY_DN12719_c0_g2_i4	Glyma.13G321300.31	100	160	20	500	2583	2050	6	0	2588

TRINITY_DN12719_c0_g2_i4	Glyma.13G321300.11	95.6043956	160	20	500	2583	2050	6	0	2588

TRINITY_DN12719_c0_g2_i5	Glyma.13G321300.44	98.5915493	160	20	357	2440	2050	6	0	2588

TRINITY_DN12719_c0_g2_i5	Glyma.13G321300.32	95.71428571	160	20	357	2440	2050	6	0	2588

TRINITY_DN12719_c0_g2_i5	Glyma.13G321300.12	97.72727273	160	20	357	2440	2050	6	0	2588

TRINITY_DN12719_c0_g2_i5	Glyma.13G321300.45	100	160	20	357	2440	2050	6	0	2588

TRINITY_DN12719_c0_g2_i5	Glyma.13G321300.33	100	160	20	357	2440	2050	6	0	2588

TRINITY_DN12719_c0_g2_i5	Glyma.13G321300.13	97.98657718	160	20	357	2440	2050	6	0	2588

TRINITY_DN12719_c0_g2_i7	Glyma.13G321300.44	98.32402235	160	20	357	2440	2050	6	0	2588

TRINITY_DN12719_c0_g2_i7	Glyma.13G321300.32	99.53703704	160	20	357	2440	2050	6	0	2588

TRINITY_DN12719_c0_g2_i7	Glyma.13G321300.12	99.53703704	160	20	357	2440	2050	6	0	2588

TRINITY_DN12719_c0_g2_i7	Glyma.13G321300.45	100	160	20	357	2440	2050	6	0	2588

TRINITY_DN12719_c0_g2_i7	Glyma.13G321300.33	100	160	20	357	2440	2050	6	0	2588

TRINITY_DN12719_c0_g2_i7	Glyma.13G321300.13	95.23809524	160	20	357	2440	2050	6	0	2588

TRINITY_DN8743_c0_g1_i1	Glyma.02G296300.2	100	142	5	623	2716	1	2088	0	3031

TRINITY_DN8743_c0_g1_i1	Glyma.02G296300.1	96.30769231	142	5	623	2716	1	2088	0	3031

TRINITY_DN8743_c0_g1_i1	Glyma.14G017400.1	100	139	5	623	2716	1	2088	0	3048

TRINITY_DN8743_c0_g1_i1	Glyma.14G017400.2	95.7957958	139	5	623	2716	1	2088	0	3048

TRINITY_DN13347_c0_g1_i4	Glyma.09G206300.1	100	121	1	114	2205	689	2783	0	3179

TRINITY_DN15501_c0_g2_i2	Glyma.13G162800.3	97.08737864	119	0	578	2672	2095	1	0	3210

TRINITY_DN13347_c0_g1_i4	Glyma.01G016500.2	98.96907216	112	1	114	2205	689	2783	0	3229

TRINITY_DN13347_c0_g1_i4	Glyma.01G016500.1	96.69117647	112	1	114	2205	689	2783	0	3229

TRINITY_DN20489_c0_g1_i1	Glyma.19G121400.1	100	100	6	463	2550	2094	1	0	3264

TRINITY_DN15757_c0_g1_i3	Glyma.05G150800.1	97.99196787	378	41	773	2834	703	2764	0	1360

TRINITY_DN15757_c0_g1_i7	Glyma.05G150800.1	100	378	41	792	2853	703	2764	0	1360

TRINITY_DN15757_c0_g1_i4	Glyma.05G150800.1	100	344	41	792	2853	703	2764	0	1548

TRINITY_DN13097_c0_g1_i3	Glyma.14G022300.1	100	194	10	194	2276	2097	9	0	2689

TRINITY_DN16006_c0_g1_i2	Glyma.08G086600.1	100	141	5	381	2468	2091	1	0	3027

TRINITY_DN15987_c0_g2_i1	Glyma.04G237600.1	97	127	4	358	2451	1	2076	0	3048

TRINITY_DN12436_c0_g1_i1	Glyma.01G170600.1	95.8073149	118	5	116	2204	1	2092	0	3168

TRINITY_DN20489_c0_g1_i1	Glyma.03G004500.1	95.03546099	103	3	463	2550	2094	1	0	3258

TRINITY_DN16082_c0_g1_i12	Glyma.07G016700.2	95.56650246	433	40	121	2187	2247	178	0	1149

TRINITY_DN16254_c0_g1_i8	Glyma.13G101700.1	98.97959184	270	11	618	2703	3048	961	0	2292

TRINITY_DN16082_c0_g1_i10	Glyma.08G202000.5	100	209	5	188	2257	2274	184	0	2545

TRINITY_DN16252_c0_g1_i1	Glyma.08G293600.2	95.89041096	195	6	1665	3747	21	2112	0	2713

TRINITY_DN16252_c0_g1_i3	Glyma.08G293600.2	95.56451613	195	6	1665	3747	21	2112	0	2713

TRINITY_DN13097_c0_g1_i3	Glyma.02G292300.3	100	195	9	194	2278	2088	1	0	2700

TRINITY_DN13097_c0_g1_i3	Glyma.02G292300.2	98.30508475	195	9	194	2278	2088	1	0	2700

TRINITY_DN15506_c0_g2_i1	Glyma.15G074200.1	96.27329193	189	11	871	2953	1	2077	0	2651

TRINITY_DN15506_c0_g2_i10	Glyma.15G074200.1	100	189	11	954	3036	1	2077	0	2651

TRINITY_DN15506_c0_g2_i14	Glyma.15G074200.1	100	189	11	953	3035	1	2077	0	2651

TRINITY_DN15506_c0_g2_i2	Glyma.15G074200.1	100	189	11	1268	3350	1	2077	0	2651

TRINITY_DN15506_c0_g2_i7	Glyma.15G074200.1	99.23076923	189	11	1036	3118	1	2077	0	2651

TRINITY_DN15506_c0_g2_i9	Glyma.15G074200.1	100	189	11	1185	3267	1	2077	0	2651

TRINITY_DN11678_c0_g1_i1	Glyma.13G308500.3	95.83333333	176	0	775	2867	2093	1	0	2891

TRINITY_DN16006_c0_g1_i1	Glyma.09G052600.1	100	176	11	266	2354	2088	21	0	2702

TRINITY_DN16360_c0_g4_i7	Glyma.07G010400.1	100	102	3	329	2417	1	2092	0	3267

TRINITY_DN14464_c0_g1_i1	Glyma.18G232400.1	97.59036145	100	5	319	2399	2087	1	0	3195

TRINITY_DN14464_c0_g1_i2	Glyma.18G232400.1	95.83333333	100	5	319	2399	2087	1	0	3195

TRINITY_DN16004_c1_g1_i4	Glyma.17G096100.1	98.30508475	465	44	352	2420	127	2195	0	990

TRINITY_DN15757_c0_g1_i1	Glyma.05G150800.1	98.6013986	369	44	792	2853	703	2764	0	1432

TRINITY_DN15757_c0_g1_i5	Glyma.05G150800.1	98.8372093	369	44	773	2834	703	2764	0	1432

TRINITY_DN15757_c0_g1_i2	Glyma.05G150800.1	100	335	44	773	2834	703	2764	0	1620

TRINITY_DN15757_c0_g1_i6	Glyma.05G150800.1	99.29577465	335	44	792	2853	703	2764	0	1620

TRINITY_DN13654_c0_g2_i1	Glyma.19G261700.1	96.54255319	295	25	461	2522	2142	78	0	1860

TRINITY_DN15751_c0_g1_i1	Glyma.04G193800.1	96.875	287	22	890	2962	1	2076	0	2047

TRINITY_DN9205_c0_g1_i2	Glyma.07G148100.11	99.07407407	191	6	634	2709	2091	1	0	2695

TRINITY_DN9205_c0_g1_i2	Glyma.07G148100.10	100	189	12	634	2709	2067	1	0	2551

TRINITY_DN16254_c0_g1_i8	Glyma.15G167500.1	100	186	4	618	2709	3051	964	0	2808

TRINITY_DN16254_c0_g1_i8	Glyma.15G167500.3	98.48053181	186	4	618	2709	3051	964	0	2808

TRINITY_DN16254_c0_g1_i8	Glyma.15G167500.2	95.93301435	186	4	618	2709	3051	964	0	2808

TRINITY_DN16254_c0_g1_i8	Glyma.15G167500.4	100	186	4	618	2709	3051	964	0	2808

TRINITY_DN12878_c0_g1_i1	Glyma.18G025500.2	100	183	14	33	2123	1	2070	0	2700

TRINITY_DN12878_c0_g1_i1	Glyma.18G025500.3	100	183	14	33	2123	1	2070	0	2700

TRINITY_DN12878_c0_g1_i3	Glyma.18G025500.2	100	183	14	33	2123	1	2070	0	2700

TRINITY_DN12878_c0_g1_i3	Glyma.18G025500.3	100	183	14	33	2123	1	2070	0	2700

TRINITY_DN16006_c0_g1_i1	Glyma.09G052600.5	99.62121212	176	11	266	2353	2067	1	0	2700

TRINITY_DN16006_c0_g1_i1	Glyma.09G052600.3	100	176	11	266	2353	2067	1	0	2700

TRINITY_DN16006_c0_g1_i1	Glyma.09G052600.2	100	176	11	266	2353	2067	1	0	2700

TRINITY_DN15127_c0_g1_i1	Glyma.16G003700.1	96.96969697	148	4	188	2275	67	2157	0	3011

TRINITY_DN15127_c0_g1_i1	Glyma.12G167800.1	97.10144928	146	7	188	2275	76	2151	0	2926

TRINITY_DN14801_c0_g3_i4	Glyma.06G195700.1	99	141	3	366	2447	2133	43	0	3011

TRINITY_DN9577_c0_g1_i1	Glyma.20G238800.1	100	140	14	1	2068	455	2537	0	2874

TRINITY_DN9577_c0_g1_i1	Glyma.20G238800.4	100	140	14	1	2068	455	2537	0	2874

TRINITY_DN16303_c0_g1_i3	Glyma.13G301100.5	100	118	4	1028	3113	1	2089	0	3151

TRINITY_DN16303_c0_g1_i5	Glyma.13G301100.5	100	118	4	1028	3113	1	2089	0	3151

TRINITY_DN15757_c0_g1_i3	Glyma.08G107700.1	95.82309582	376	44	779	2832	901	2957	0	1319

TRINITY_DN15757_c0_g1_i7	Glyma.08G107700.1	100	376	44	798	2851	901	2957	0	1319

TRINITY_DN15757_c0_g1_i4	Glyma.08G107700.1	100	344	44	798	2851	901	2957	0	1496

TRINITY_DN15912_c1_g1_i2	Glyma.07G019500.1	100	293	6	390	2468	2256	166	0	2161

TRINITY_DN12569_c0_g1_i6	Glyma.15G061700.1	100	229	23	69	2128	1	2084	0	2348

TRINITY_DN12569_c0_g1_i6	Glyma.13G253200.1	96.55172414	225	25	69	2116	1	2084	0	2292

TRINITY_DN15751_c0_g1_i6	Glyma.04G193800.1	100	198	18	890	2965	1	2076	0	2571

TRINITY_DN8969_c0_g1_i1	Glyma.19G167900.4	96.96969697	196	16	264	2349	1	2050	0	2479

TRINITY_DN8969_c0_g1_i1	Glyma.19G167900.5	96.96969697	196	16	264	2349	1	2050	0	2479

TRINITY_DN8969_c0_g1_i1	Glyma.19G167900.6	96.96969697	196	16	264	2349	1	2050	0	2479

TRINITY_DN8969_c0_g1_i1	Glyma.19G167900.7	97.08029197	196	16	264	2349	1	2050	0	2479

TRINITY_DN13097_c0_g1_i3	Glyma.14G022300.4	96.36363636	194	9	194	2276	2094	9	0	2702

TRINITY_DN16082_c0_g1_i13	Glyma.08G202000.5	100	189	2	142	2211	2274	184	0	2678

TRINITY_DN12460_c0_g1_i1	Glyma.13G346200.7	98.64864865	188	1	1213	3300	2297	207	0	2800

TRINITY_DN12460_c0_g1_i1	Glyma.13G346200.8	98.64864865	188	1	1213	3300	2297	207	0	2800

TRINITY_DN12460_c0_g1_i1	Glyma.13G346200.2	98.64864865	188	1	1213	3300	2297	207	0	2800

TRINITY_DN12460_c0_g1_i1	Glyma.13G346200.3	97.26027397	188	1	1213	3300	2297	207	0	2800

TRINITY_DN15893_c0_g4_i9	Glyma.12G010800.1	100	182	25	342	2412	1	2035	0	2362

TRINITY_DN16006_c0_g1_i1	Glyma.15G158900.1	100	181	7	266	2353	2073	1	0	2723

TRINITY_DN16006_c0_g1_i1	Glyma.15G158900.2	100	181	7	266	2353	2073	1	0	2723

TRINITY_DN15137_c0_g1_i3	Glyma.04G019700.1	98.21428571	163	1	683	2770	2115	25	0	2939

TRINITY_DN9063_c0_g1_i1	Glyma.11G102800.1	95.91836735	162	0	230	2320	3336	1246	0	2964

TRINITY_DN9063_c0_g4_i1	Glyma.11G102800.1	95.91836735	162	0	172	2262	3336	1246	0	2964

TRINITY_DN9063_c0_g1_i1	Glyma.12G027900.1	95.92944369	154	0	230	2320	3321	1231	0	3009

TRINITY_DN9063_c0_g4_i1	Glyma.12G027900.1	95.93023256	154	0	172	2262	3321	1231	0	3009

TRINITY_DN2513_c0_g1_i1	Glyma.01G149800.4	97.14285714	148	3	219	2306	1	2085	0	2983

TRINITY_DN2513_c0_g1_i1	Glyma.01G149800.1	96.42058166	148	3	219	2306	1	2085	0	2983

TRINITY_DN2513_c0_g2_i1	Glyma.01G149800.4	100	148	3	219	2306	1	2085	0	2983

TRINITY_DN2513_c0_g2_i1	Glyma.01G149800.1	100	148	3	219	2306	1	2085	0	2983

TRINITY_DN2513_c0_g1_i1	Glyma.09G213900.1	99.29078014	143	3	219	2306	1	2085	0	3011

TRINITY_DN2513_c0_g2_i1	Glyma.09G213900.1	96.51741294	143	3	219	2306	1	2085	0	3011

TRINITY_DN12436_c0_g1_i1	Glyma.11G072700.1	96.79715302	128	1	116	2206	1	2082	0	3094

TRINITY_DN16006_c0_g1_i2	Glyma.05G132000.1	100	124	2	381	2468	2088	1	0	3136

TRINITY_DN15521_c0_g1_i2	Glyma.12G231800.1	100	323	24	1129	3207	951	3023	0	1890

TRINITY_DN16065_c0_g1_i6	Glyma.13G244300.2	98.30508475	262	43	572	2602	1	2073	0	1917

TRINITY_DN16353_c0_g1_i2	Glyma.10G284500.1	100	255	17	389	2463	1006	3077	0	2233

TRINITY_DN16353_c0_g1_i7	Glyma.10G284500.1	99.35897436	255	17	471	2545	1006	3077	0	2233

TRINITY_DN14680_c0_g2_i1	Glyma.19G175600.4	99.36305732	210	22	1	2069	374	2443	0	2431

TRINITY_DN14680_c0_g2_i1	Glyma.19G175600.3	99.36305732	210	22	1	2069	374	2443	0	2431

TRINITY_DN14680_c0_g2_i2	Glyma.19G175600.4	100	210	22	1	2069	374	2443	0	2431

TRINITY_DN14680_c0_g2_i2	Glyma.19G175600.3	98.34710744	210	22	1	2069	374	2443	0	2431

TRINITY_DN14680_c0_g2_i5	Glyma.19G175600.4	96.90721649	210	22	1	2069	374	2443	0	2431

TRINITY_DN14680_c0_g2_i5	Glyma.19G175600.3	96.90721649	210	22	1	2069	374	2443	0	2431

TRINITY_DN14680_c0_g2_i6	Glyma.19G175600.4	100	210	22	1	2069	374	2443	0	2431

TRINITY_DN14680_c0_g2_i6	Glyma.19G175600.3	98.7804878	210	22	1	2069	374	2443	0	2431

TRINITY_DN14972_c0_g1_i1	Glyma.07G233400.1	98.03921569	192	15	231	2284	14	2088	0	2466

TRINITY_DN16436_c0_g2_i4	Glyma.11G131600.2	97.04142012	190	5	915	2990	1	2088	0	2704

TRINITY_DN8815_c0_g1_i1	Glyma.07G182200.1	100	154	16	967	3026	2100	11	0	2813

TRINITY_DN15852_c0_g1_i2	Glyma.16G176100.4	95.55555556	138	3	430	2517	1	2088	0	3070

TRINITY_DN15852_c0_g1_i2	Glyma.16G176100.1	99.44134078	138	3	430	2517	1	2088	0	3070

TRINITY_DN15852_c0_g1_i2	Glyma.16G176100.2	99.44134078	138	3	430	2517	1	2088	0	3070

TRINITY_DN15852_c0_g1_i2	Glyma.16G176100.3	99.44134078	138	3	430	2517	1	2088	0	3070

TRINITY_DN15852_c0_g1_i2	Glyma.16G176100.5	99.44134078	138	3	430	2517	1	2088	0	3070

TRINITY_DN15852_c0_g1_i3	Glyma.16G176100.4	98.64864865	138	3	337	2424	1	2088	0	3070

TRINITY_DN15852_c0_g1_i3	Glyma.16G176100.1	100	138	3	337	2424	1	2088	0	3070

TRINITY_DN15852_c0_g1_i3	Glyma.16G176100.2	96.23188406	138	3	337	2424	1	2088	0	3070

TRINITY_DN15852_c0_g1_i3	Glyma.16G176100.3	97.54601227	138	3	337	2424	1	2088	0	3070

TRINITY_DN15852_c0_g1_i3	Glyma.16G176100.5	96.20253165	138	3	337	2424	1	2088	0	3070

TRINITY_DN15852_c0_g1_i6	Glyma.16G176100.4	99.74683544	138	3	414	2501	1	2088	0	3070

TRINITY_DN15852_c0_g1_i6	Glyma.16G176100.1	96.27959414	138	3	414	2501	1	2088	0	3070

TRINITY_DN15852_c0_g1_i6	Glyma.16G176100.2	97.33333333	138	3	414	2501	1	2088	0	3070

TRINITY_DN15852_c0_g1_i6	Glyma.16G176100.3	97.33333333	138	3	414	2501	1	2088	0	3070

TRINITY_DN15852_c0_g1_i6	Glyma.16G176100.5	97.33333333	138	3	414	2501	1	2088	0	3070

TRINITY_DN15764_c0_g1_i4	Glyma.09G135100.1	98.30508475	130	6	1	2083	3	2085	0	3049

TRINITY_DN15764_c0_g1_i7	Glyma.09G135100.1	95.83333333	130	6	1	2083	3	2085	0	3049

TRINITY_DN15764_c0_g1_i9	Glyma.09G135100.1	96.45714286	130	6	1	2083	3	2085	0	3049

TRINITY_DN9106_c0_g1_i1	Glyma.07G054800.3	97.61904762	129	26	178	2220	1	2061	0	2654

TRINITY_DN9106_c0_g1_i1	Glyma.07G054800.2	98.93617021	129	27	178	2220	1	2052	0	2595

TRINITY_DN16360_c0_g4_i7	Glyma.08G193500.1	100	116	4	329	2417	1	2086	0	3184

TRINITY_DN15757_c0_g1_i1	Glyma.08G107700.1	100	368	46	798	2851	901	2957	0	1386

TRINITY_DN15757_c0_g1_i5	Glyma.08G107700.1	96.90721649	368	46	779	2832	901	2957	0	1386

TRINITY_DN15757_c0_g1_i2	Glyma.08G107700.1	100	336	46	779	2832	901	2957	0	1563

TRINITY_DN15757_c0_g1_i6	Glyma.08G107700.1	99.29577465	336	46	798	2851	901	2957	0	1563

TRINITY_DN11082_c0_g1_i1	Glyma.08G194500.5	95.28301887	268	6	1012	3097	2086	1	0	2335

TRINITY_DN11082_c0_g1_i1	Glyma.08G194500.1	95.28301887	268	6	1012	3097	2086	1	0	2335

TRINITY_DN11082_c0_g1_i1	Glyma.08G194500.2	97.35576923	268	6	1012	3097	2086	1	0	2335

TRINITY_DN11082_c0_g1_i1	Glyma.08G194500.6	98.0952381	268	6	1012	3097	2086	1	0	2335

TRINITY_DN11082_c0_g1_i1	Glyma.08G194500.4	98.5915493	268	6	1012	3097	2086	1	0	2335

TRINITY_DN11082_c0_g1_i1	Glyma.08G194500.3	100	268	6	1012	3097	2086	1	0	2335

TRINITY_DN11082_c0_g1_i1	Glyma.07G011300.1	95.28301887	258	6	1012	3097	2086	1	0	2390

TRINITY_DN9796_c0_g1_i4	Glyma.01G207400.1	99.23076923	213	5	296	2378	2886	813	0	2542

TRINITY_DN9796_c0_g1_i5	Glyma.01G207400.1	99.21875	213	5	296	2378	2886	813	0	2542

TRINITY_DN15893_c0_g4_i9	Glyma.09G225600.5	95.34883721	184	18	342	2412	1	2047	0	2451

TRINITY_DN15893_c0_g4_i9	Glyma.09G225600.4	95.34883721	184	18	342	2412	1	2047	0	2451

TRINITY_DN15893_c0_g4_i9	Glyma.09G225600.1	95.34883721	184	18	342	2412	1	2047	0	2451

TRINITY_DN19780_c0_g1_i1	Glyma.16G036100.1	96.0199005	117	2	1048	3132	2432	344	0	3184

TRINITY_DN16441_c4_g2_i2	Glyma.07G201200.1	98.84868421	399	17	847	2925	5016	2953	0	1432

TRINITY_DN16441_c4_g2_i2	Glyma.07G201200.2	100	399	17	847	2925	5013	2950	0	1432

TRINITY_DN15980_c0_g2_i3	Glyma.10G004100.1	96.91780822	340	34	764	2822	1680	3720	0	1482

TRINITY_DN15980_c0_g2_i3	Glyma.10G004100.2	96.91714836	340	34	764	2822	1260	3300	0	1482

TRINITY_DN15521_c0_g1_i2	Glyma.12G233000.1	100	328	21	1129	3207	948	3020	0	1884

TRINITY_DN10739_c0_g2_i1	Glyma.19G246300.1	100	260	18	342	2420	2076	5	0	2254

TRINITY_DN10739_c0_g2_i1	Glyma.19G246300.2	100	260	18	342	2420	2076	5	0	2254

TRINITY_DN10739_c0_g2_i1	Glyma.19G246300.3	100	260	18	342	2420	2076	5	0	2254

TRINITY_DN10009_c0_g1_i2	Glyma.14G223300.3	95.45454545	238	5	47	2124	1	2084	0	2447

TRINITY_DN10009_c0_g1_i1	Glyma.14G223300.3	98.73417722	233	12	47	2113	1	2084	0	2403

TRINITY_DN14972_c0_g1_i1	Glyma.17G038400.2	98.02371542	195	12	231	2284	14	2085	0	2453

TRINITY_DN14972_c0_g1_i1	Glyma.17G038400.1	98.02631579	195	12	231	2284	14	2085	0	2453

TRINITY_DN12043_c0_g2_i3	Glyma.10G005000.1	98.33333333	171	17	320	2392	158	2228	0	2702

TRINITY_DN38879_c0_g1_i1	Glyma.06G188900.1	98.57142857	156	4	108	2184	1	2085	0	2902

TRINITY_DN15450_c1_g1_i1	Glyma.01G079100.2	96.72131148	149	14	290	2368	2080	5	0	2894

TRINITY_DN15450_c1_g1_i1	Glyma.01G079100.1	99.24242424	149	14	290	2368	2080	5	0	2894

TRINITY_DN15450_c1_g1_i3	Glyma.01G079100.2	98.41269841	149	14	237	2315	2080	5	0	2894

TRINITY_DN15450_c1_g1_i3	Glyma.01G079100.1	99.14529915	149	14	237	2315	2080	5	0	2894

TRINITY_DN13956_c0_g1_i3	Glyma.09G196100.1	99.46524064	147	14	83	2147	1	2086	0	2880

TRINITY_DN15852_c0_g1_i2	Glyma.09G128500.1	99.08256881	137	0	430	2517	1	2088	0	3097

TRINITY_DN15852_c0_g1_i3	Glyma.09G128500.1	100	137	0	337	2424	1	2088	0	3097

TRINITY_DN15852_c0_g1_i6	Glyma.09G128500.1	98.64864865	137	0	414	2501	1	2088	0	3097

TRINITY_DN15852_c0_g1_i2	Glyma.09G128500.2	99.08256881	137	1	430	2517	1	2085	0	3077

TRINITY_DN15852_c0_g1_i3	Glyma.09G128500.2	100	137	1	337	2424	1	2085	0	3077

TRINITY_DN15852_c0_g1_i6	Glyma.09G128500.2	98.30508475	137	1	414	2501	1	2085	0	3077

TRINITY_DN5316_c0_g1_i1	Glyma.05G013900.1	100	137	4	149	2230	2108	24	0	3038

TRINITY_DN15987_c0_g2_i1	Glyma.06G126400.2	95	134	3	358	2445	1	2079	0	3055

TRINITY_DN15987_c0_g2_i1	Glyma.06G126400.1	97	134	3	358	2445	1	2079	0	3055

TRINITY_DN3145_c0_g1_i1	Glyma.15G201600.1	100	339	24	280	2342	20	2097	0	1762

TRINITY_DN5582_c0_g4_i1	Glyma.05G248800.1	95.67901235	334	19	19	2073	9	2084	0	1725

TRINITY_DN16415_c1_g1_i15	Glyma.13G327000.3	100	298	7	1688	3769	2082	1	0	2139

TRINITY_DN16415_c1_g1_i4	Glyma.13G327000.3	98.21428571	298	7	1792	3873	2082	1	0	2139

TRINITY_DN14245_c0_g2_i2	Glyma.15G072000.3	95.65217391	281	12	1	2071	310	2389	0	2148

TRINITY_DN15506_c0_g2_i1	Glyma.13G239100.1	99.15966387	182	16	871	2953	1	2062	0	2658

TRINITY_DN15506_c0_g2_i10	Glyma.13G239100.1	95.52238806	182	16	954	3036	1	2062	0	2658

TRINITY_DN15506_c0_g2_i14	Glyma.13G239100.1	95.19230769	182	16	953	3035	1	2062	0	2658

TRINITY_DN15506_c0_g2_i2	Glyma.13G239100.1	100	182	16	1268	3350	1	2062	0	2658

TRINITY_DN15506_c0_g2_i7	Glyma.13G239100.1	95.71865443	182	16	1036	3118	1	2062	0	2658

TRINITY_DN15506_c0_g2_i9	Glyma.13G239100.1	98.1595092	182	16	1185	3267	1	2062	0	2658

TRINITY_DN15968_c0_g1_i1	Glyma.16G051200.1	100	169	20	94	2154	1	2076	0	2678

TRINITY_DN14622_c0_g1_i1	Glyma.10G179700.6	100	160	8	193	2270	6	2082	0	2844

TRINITY_DN8240_c0_g1_i1	Glyma.01G224300.1	98.98989899	142	4	101	2185	1	2085	0	3042

TRINITY_DN12617_c0_g1_i1	Glyma.13G104300.2	98.50746269	264	7	2462	4538	1347	3432	0	2331

TRINITY_DN12617_c0_g1_i1	Glyma.13G104300.10	98.49056604	264	7	2462	4538	1323	3408	0	2331

TRINITY_DN12617_c0_g1_i1	Glyma.13G104300.8	98.50746269	264	7	2462	4538	1221	3306	0	2331

TRINITY_DN14621_c1_g1_i3	Glyma.16G063900.2	98.18181818	258	4	104	2187	1	2084	0	2398

TRINITY_DN14621_c1_g1_i3	Glyma.16G063900.1	98.18181818	258	4	104	2187	1	2084	0	2398

TRINITY_DN14621_c1_g1_i1	Glyma.16G063900.2	95	238	4	104	2187	1	2084	0	2508

TRINITY_DN14621_c1_g1_i1	Glyma.16G063900.1	97.39130435	238	4	104	2187	1	2084	0	2508

TRINITY_DN13371_c0_g1_i2	Glyma.13G089700.1	100	204	3	535	2616	2085	1	0	2689

TRINITY_DN13371_c0_g1_i2	Glyma.13G089700.2	95	204	3	535	2616	2085	1	0	2689

TRINITY_DN35682_c0_g1_i1	Glyma.04G190300.1	98.26589595	197	9	229	2310	2097	19	0	2689

TRINITY_DN13639_c0_g1_i1	Glyma.12G181900.1	98.95833333	187	12	1	2052	2076	1	0	2532

TRINITY_DN15968_c0_g1_i1	Glyma.19G099400.1	100	180	13	94	2154	1	2079	0	2649

TRINITY_DN15968_c0_g1_i1	Glyma.19G099400.5	100	180	13	94	2154	1	2079	0	2649

TRINITY_DN15910_c0_g1_i5	Glyma.03G220500.2	96.90346084	125	3	325	2407	3	2088	0	3140

TRINITY_DN15329_c0_g2_i5	Glyma.05G238700.2	96.73913043	106	1	481	2566	2068	1	0	3149

TRINITY_DN9549_c0_g1_i1	Glyma.01G162100.2	100	189	13	127	2198	18	2098	0	2693

TRINITY_DN15893_c0_g4_i1	Glyma.12G010800.1	95.95375723	173	23	342	2406	1	2035	0	2440

TRINITY_DN15893_c0_g4_i2	Glyma.12G010800.1	96.58703072	173	23	342	2406	1	2035	0	2440

TRINITY_DN13324_c0_g2_i1	Glyma.12G236100.4	98.42519685	160	9	572	2650	2119	47	0	2848

TRINITY_DN8240_c0_g1_i1	Glyma.11G018600.1	95.45454545	157	0	101	2185	1	2085	0	2981

TRINITY_DN13371_c0_g1_i4	Glyma.13G089700.1	100	152	1	535	2616	2085	1	0	2988

TRINITY_DN13371_c0_g1_i4	Glyma.13G089700.2	100	152	1	535	2616	2085	1	0	2988

TRINITY_DN17075_c0_g1_i1	Glyma.09G215200.1	98.48484848	133	0	156	2240	2085	1	0	3114

TRINITY_DN17075_c0_g1_i1	Glyma.09G215200.2	98.47328244	131	1	156	2240	2070	1	0	3027

TRINITY_DN10929_c0_g1_i1	Glyma.04G206300.2	98.80952381	115	10	35	2116	1	2070	0	3097

TRINITY_DN10929_c0_g1_i1	Glyma.06G159400.1	97.40740741	109	12	35	2116	1	2064	0	3092

TRINITY_DN10929_c0_g1_i1	Glyma.06G159400.2	97.40802676	106	17	35	2116	1	2040	0	2953

TRINITY_DN12617_c0_g1_i1	Glyma.13G104300.3	98.50746269	265	9	2462	4538	1347	3423	0	2290

TRINITY_DN12617_c0_g1_i1	Glyma.13G104300.9	98.50746269	265	9	2462	4538	1323	3399	0	2290

TRINITY_DN12617_c0_g1_i1	Glyma.13G104300.7	98.50746269	265	9	2462	4538	1221	3297	0	2290

TRINITY_DN16082_c0_g1_i10	Glyma.08G202000.4	100	209	8	188	2257	2265	184	0	2588

TRINITY_DN19139_c0_g1_i1	Glyma.17G006500.1	98.61111111	192	23	47	2079	59	2139	0	2436

TRINITY_DN15223_c0_g1_i2	Glyma.05G234900.1	100	191	10	49	2118	512	2593	0	2687

TRINITY_DN5469_c0_g1_i1	Glyma.03G146200.3	95.41284404	188	0	28	2111	452	2535	0	2808

TRINITY_DN5469_c0_g1_i1	Glyma.03G146200.1	97.08520179	188	0	28	2111	452	2535	0	2808

TRINITY_DN5469_c0_g1_i1	Glyma.03G146200.2	97.08737864	188	0	28	2111	452	2535	0	2808

TRINITY_DN5469_c0_g2_i1	Glyma.03G146200.3	98	188	0	28	2111	452	2535	0	2808

TRINITY_DN5469_c0_g2_i1	Glyma.03G146200.1	96.35701275	188	0	28	2111	452	2535	0	2808

TRINITY_DN5469_c0_g2_i1	Glyma.03G146200.2	96.35722679	188	0	28	2111	452	2535	0	2808

TRINITY_DN16082_c0_g1_i13	Glyma.08G202000.4	100	185	8	142	2211	2265	184	0	2721

TRINITY_DN9651_c0_g1_i1	Glyma.04G185300.1	100	148	9	349	2423	2066	1	0	2854

TRINITY_DN12831_c0_g1_i1	Glyma.05G146100.1	100	130	1	67	2147	9	2092	0	3109

TRINITY_DN11508_c1_g2_i1	Glyma.09G183600.1	97.36842105	127	4	147	2228	2082	1	0	3120

TRINITY_DN11508_c1_g2_i1	Glyma.07G092600.1	100	126	4	147	2228	2082	1	0	3125

TRINITY_DN16360_c0_g8_i5	Glyma.16G131800.1	99.35897436	115	9	197	2277	1	2078	0	3153

TRINITY_DN15980_c0_g2_i3	Glyma.02G004200.1	99.28571429	328	43	764	2811	1716	3750	0	1493

TRINITY_DN15980_c0_g2_i3	Glyma.02G004200.2	95.83333333	328	43	764	2811	1698	3732	0	1493

TRINITY_DN15510_c0_g1_i2	Glyma.16G218100.1	100	303	27	234	2303	185	2244	0	1936

TRINITY_DN15510_c0_g1_i2	Glyma.16G218100.3	100	303	27	234	2303	185	2244	0	1936

TRINITY_DN14940_c1_g2_i1	Glyma.12G220400.1	100	298	16	798	2875	2068	1	0	2067

TRINITY_DN13817_c0_g1_i1	Glyma.02G290700.1	98.91304348	246	15	612	2678	2076	1	0	2335

TRINITY_DN16171_c1_g2_i3	Glyma.13G270900.1	96.66666667	205	27	631	2655	2079	1	0	2311

TRINITY_DN16171_c1_g2_i5	Glyma.13G270900.1	96.51022864	203	30	631	2641	2079	1	0	2231

TRINITY_DN16171_c1_g2_i1	Glyma.13G270900.1	96.93877551	200	27	669	2693	2079	1	0	2338

TRINITY_DN16171_c1_g2_i6	Glyma.13G270900.1	97.59036145	198	30	669	2679	2079	1	0	2259

TRINITY_DN13371_c0_g1_i2	Glyma.14G174500.2	98.85245902	196	2	535	2616	2082	1	0	2748

TRINITY_DN13371_c0_g1_i2	Glyma.14G174500.1	99.50980392	196	2	535	2616	2082	1	0	2748

TRINITY_DN15893_c0_g4_i9	Glyma.09G225600.2	100	184	17	342	2412	1	2041	0	2479

TRINITY_DN15893_c0_g4_i9	Glyma.09G225600.3	100	184	17	342	2412	1	2041	0	2479

TRINITY_DN15893_c0_g4_i1	Glyma.09G225600.5	100	177	16	342	2406	1	2047	0	2518

TRINITY_DN15893_c0_g4_i1	Glyma.09G225600.4	100	177	16	342	2406	1	2047	0	2518

TRINITY_DN15893_c0_g4_i1	Glyma.09G225600.1	99.28057554	177	16	342	2406	1	2047	0	2518

TRINITY_DN15893_c0_g4_i2	Glyma.09G225600.5	98.7654321	177	16	342	2406	1	2047	0	2518

TRINITY_DN15893_c0_g4_i2	Glyma.09G225600.4	98.7654321	177	16	342	2406	1	2047	0	2518

TRINITY_DN15893_c0_g4_i2	Glyma.09G225600.1	96.58634538	177	16	342	2406	1	2047	0	2518

TRINITY_DN15206_c0_g1_i5	Glyma.18G151600.1	97.61904762	169	4	343	2419	78	2158	0	2859

TRINITY_DN12916_c0_g1_i1	Glyma.17G158300.1	98.30508475	145	34	312	2306	1	2055	0	2294

TRINITY_DN15078_c0_g1_i1	Glyma.18G223300.3	99.46808511	124	0	28	2110	4482	6564	0	3160

TRINITY_DN15235_c0_g1_i1	Glyma.16G172400.1	100	373	17	2044	4116	2440	4512	0	1663

TRINITY_DN15235_c0_g1_i1	Glyma.16G172400.4	96.91119691	373	17	2044	4116	2398	4470	0	1663

TRINITY_DN15235_c0_g1_i1	Glyma.16G172400.3	99.25373134	373	17	2044	4116	2371	4443	0	1663

TRINITY_DN15235_c0_g1_i1	Glyma.16G172400.2	95	373	17	2044	4116	1648	3720	0	1663

TRINITY_DN15235_c0_g1_i1	Glyma.16G172400.5	96.77419355	373	17	2044	4116	385	2457	0	1663

TRINITY_DN16441_c4_g2_i3	Glyma.13G175200.1	98.62068966	316	20	526	2588	5022	2960	0	1849

TRINITY_DN9907_c0_g1_i1	Glyma.02G000700.1	100	227	7	51	2123	1	2082	0	2529

TRINITY_DN16067_c2_g1_i3	Glyma.15G154000.3	100	192	0	474	2555	1	2082	0	2782

TRINITY_DN13817_c0_g1_i2	Glyma.02G290700.1	99	184	6	552	2627	2076	1	0	2748

TRINITY_DN16138_c1_g1_i18	Glyma.08G077100.2	96.90402477	181	4	608	2679	3	2082	0	2765

TRINITY_DN16138_c1_g1_i5	Glyma.08G077100.2	96.20253165	181	4	874	2945	3	2082	0	2765

TRINITY_DN16138_c1_g1_i20	Glyma.08G077100.2	100	181	5	608	2667	3	2082	0	2687

TRINITY_DN16138_c1_g1_i7	Glyma.08G077100.2	99.66887417	181	5	874	2933	3	2082	0	2687

TRINITY_DN13371_c0_g1_i4	Glyma.14G174500.2	100	140	0	535	2616	2082	1	0	3070

TRINITY_DN13371_c0_g1_i4	Glyma.14G174500.1	100	140	0	535	2616	2082	1	0	3070

TRINITY_DN14695_c0_g1_i1	Glyma.04G065700.3	97.12837838	127	0	447	2528	61	2142	0	3142

TRINITY_DN15670_c0_g1_i1	Glyma.07G245500.1	99.543379	126	0	435	2516	1	2082	0	3147

TRINITY_DN3247_c0_g1_i1	Glyma.06G001100.1	100	119	0	3	2084	862	2943	0	3186

TRINITY_DN3247_c0_g1_i1	Glyma.06G001100.2	100	119	0	3	2084	502	2583	0	3186

TRINITY_DN11541_c0_g1_i1	Glyma.11G090100.1	96.1038961	117	0	39	2120	1	2082	0	3197

TRINITY_DN16067_c2_g1_i1	Glyma.15G154000.3	96.17755857	113	0	474	2555	1	2082	0	3219

TRINITY_DN16067_c2_g1_i2	Glyma.15G154000.3	99.00990099	113	0	474	2555	1	2082	0	3219

TRINITY_DN3247_c0_g1_i1	Glyma.04G001600.4	99.42857143	110	0	3	2084	811	2892	0	3236

TRINITY_DN3247_c0_g1_i1	Glyma.04G001600.1	99.41860465	110	0	3	2084	811	2892	0	3236

TRINITY_DN3247_c0_g1_i1	Glyma.04G001600.3	99.42196532	110	0	3	2084	808	2889	0	3236

TRINITY_DN3247_c0_g1_i1	Glyma.04G001600.2	99.44134078	110	0	3	2084	808	2889	0	3236

TRINITY_DN15235_c0_g1_i1	Glyma.16G172600.1	95.62043796	367	14	2044	4116	2374	4446	0	1707

TRINITY_DN16415_c1_g1_i1	Glyma.13G327000.3	100	297	7	1600	3675	2076	1	0	2134

TRINITY_DN16415_c1_g1_i10	Glyma.13G327000.3	100	297	7	1193	3268	2076	1	0	2134

TRINITY_DN16415_c1_g1_i15	Glyma.13G327000.1	96.38554217	297	7	1694	3769	2076	1	0	2134

TRINITY_DN16415_c1_g1_i4	Glyma.13G327000.1	96.42857143	297	7	1798	3873	2076	1	0	2134

TRINITY_DN16415_c1_g1_i7	Glyma.13G327000.3	95.03546099	297	7	1704	3779	2076	1	0	2134

TRINITY_DN15329_c0_g2_i1	Glyma.02G085000.4	98.6013986	219	6	846	2924	2076	1	0	2584

TRINITY_DN16292_c0_g1_i2	Glyma.01G234300.1	98.66666667	217	9	1	2066	2366	4440	0	2505

TRINITY_DN16292_c0_g1_i2	Glyma.01G234300.3	100	217	9	1	2066	2366	4440	0	2505

TRINITY_DN16292_c0_g1_i2	Glyma.01G234300.2	100	217	9	1	2066	2348	4422	0	2505

TRINITY_DN16292_c0_g1_i2	Glyma.11G008600.1	100	216	9	1	2066	2357	4431	0	2510

TRINITY_DN16138_c1_g1_i11	Glyma.08G077100.2	95.19774011	200	2	619	2698	3	2082	0	2723

TRINITY_DN15754_c0_g1_i1	Glyma.08G072100.1	99.45355191	165	8	304	2384	3240	1190	0	2736

TRINITY_DN13013_c0_g1_i1	Glyma.16G002500.1	97.36842105	161	15	468	2537	2070	1	0	2809

TRINITY_DN15490_c0_g1_i1	Glyma.15G039500.1	96.16161616	148	5	133	2201	13	2093	0	2946

TRINITY_DN16360_c0_g8_i5	Glyma.02G051700.1	99.35897436	136	2	197	2277	1	2078	0	3070

TRINITY_DN12831_c0_g1_i1	Glyma.05G146100.2	100	130	0	67	2147	9	2089	0	3123

TRINITY_DN14641_c2_g2_i3	Glyma.13G112600.1	96.69421488	286	30	95	2135	2107	58	0	1810

TRINITY_DN15339_c0_g1_i1	Glyma.10G058200.1	100	201	2	252	2330	2154	76	0	2715

TRINITY_DN14595_c0_g1_i1	Glyma.12G103900.2	97.14285714	197	8	495	2564	1	2070	0	2621

TRINITY_DN14595_c0_g1_i1	Glyma.12G103900.1	96.03960396	197	8	495	2564	1	2070	0	2621

TRINITY_DN13817_c0_g1_i2	Glyma.14G024000.1	96.47058824	183	7	552	2627	2073	1	0	2756

TRINITY_DN10580_c0_g1_i1	Glyma.09G067200.1	100	177	13	359	2437	3669	1612	0	2712

TRINITY_DN10580_c0_g1_i1	Glyma.09G067200.2	100	177	13	359	2437	3624	1567	0	2712

TRINITY_DN10580_c0_g1_i1	Glyma.09G067200.3	100	177	13	359	2437	3468	1411	0	2712

TRINITY_DN16138_c1_g1_i22	Glyma.08G077100.2	97.36842105	155	0	619	2698	3	2082	0	2983

TRINITY_DN16138_c1_g1_i21	Glyma.08G077100.2	95.12195122	155	1	619	2686	3	2082	0	2905

TRINITY_DN14146_c1_g1_i1	Glyma.03G245000.1	98.91304348	110	14	1397	3460	2067	1	0	3044

TRINITY_DN15235_c0_g1_i1	Glyma.16G172500.4	97.32142857	369	12	2044	4116	937	3006	0	1698

TRINITY_DN15235_c0_g1_i1	Glyma.16G172500.3	96.36363636	369	12	2044	4116	937	3006	0	1698

TRINITY_DN15235_c0_g1_i1	Glyma.16G172500.2	100	369	12	2044	4116	937	3006	0	1698

TRINITY_DN15339_c0_g1_i1	Glyma.03G181700.1	100	320	2	253	2330	2141	64	0	2054

TRINITY_DN13817_c0_g1_i1	Glyma.14G024000.1	98.91304348	251	13	612	2678	2073	1	0	2333

TRINITY_DN15339_c0_g1_i1	Glyma.13G145000.1	96.36363636	192	0	252	2330	2151	73	0	2776

TRINITY_DN15897_c0_g2_i3	Glyma.14G029100.1	97.12230216	171	12	155	2220	3192	1141	0	2634

TRINITY_DN13444_c0_g1_i3	Glyma.10G233500.1	97.36842105	165	10	427	2469	2118	40	0	2693

TRINITY_DN13444_c0_g1_i3	Glyma.10G233500.2	100	163	12	427	2469	2115	40	0	2684

TRINITY_DN13956_c0_g1_i3	Glyma.16G112900.1	99.46236559	147	12	83	2147	1	2077	0	2922

TRINITY_DN1464_c0_g1_i1	Glyma.07G086500.1	98.7804878	142	0	301	2379	1	2079	0	3053

TRINITY_DN14146_c1_g1_i1	Glyma.19G242400.1	100	109	13	1397	3460	2061	1	0	3022

TRINITY_DN16382_c0_g3_i3	Glyma.14G195700.1	96.65427509	297	19	343	2397	152	2226	0	2025

TRINITY_DN14389_c1_g2_i2	Glyma.01G103500.2	95.93023256	268	36	312	2349	24	2079	0	1953

TRINITY_DN14389_c1_g2_i2	Glyma.01G103500.1	99.41348974	268	36	312	2349	24	2079	0	1953

TRINITY_DN9496_c0_g1_i1	Glyma.08G160200.1	96.40718563	182	5	109	2181	1	2076	0	2784

TRINITY_DN13926_c0_g1_i2	Glyma.11G008300.3	95.36679537	167	19	239	2262	3054	994	0	2453

TRINITY_DN13926_c0_g1_i2	Glyma.11G008300.1	95.34700315	167	19	239	2262	3054	994	0	2453

TRINITY_DN13926_c0_g1_i2	Glyma.11G008300.4	96.42857143	167	19	239	2262	2361	301	0	2453

TRINITY_DN13926_c0_g1_i2	Glyma.11G008300.2	98.71794872	167	19	239	2262	2361	301	0	2453

TRINITY_DN10765_c0_g1_i1	Glyma.11G038500.3	98	163	6	845	2910	2269	200	0	2806

TRINITY_DN10575_c0_g1_i1	Glyma.13G198200.1	100	114	6	50	2127	19	2081	0	3109

TRINITY_DN15510_c0_g1_i3	Glyma.09G168400.1	95.24281467	314	25	296	2339	7	2071	0	1805

TRINITY_DN15510_c0_g1_i6	Glyma.09G168400.1	100	314	25	270	2313	7	2071	0	1805

TRINITY_DN13116_c0_g1_i1	Glyma.13G109600.4	97.87234043	304	10	301	2370	2067	1	0	2041

TRINITY_DN13116_c0_g1_i1	Glyma.13G109600.14	97.85714286	304	10	301	2370	2067	1	0	2041

TRINITY_DN13116_c0_g1_i1	Glyma.13G109600.15	97.86096257	304	10	301	2370	2067	1	0	2041

TRINITY_DN13116_c0_g1_i1	Glyma.13G109600.5	97.87234043	304	10	301	2370	2067	1	0	2041

TRINITY_DN14677_c0_g1_i5	Glyma.13G213500.1	100	246	4	985	3059	2166	92	0	2447

TRINITY_DN15024_c0_g1_i1	Glyma.04G114300.1	100	193	44	350	2355	2045	1	0	2100

TRINITY_DN15024_c0_g1_i2	Glyma.04G114300.1	98.70689655	193	44	518	2523	2045	1	0	2100

TRINITY_DN1068_c0_g1_i1	Glyma.06G077300.1	95.21640091	188	7	80	2155	1	2058	0	2665

TRINITY_DN16006_c0_g1_i1	Glyma.08G086600.1	97.51861042	182	9	285	2353	2072	1	0	2743

TRINITY_DN13658_c0_g1_i1	Glyma.02G221900.1	99.09090909	182	15	154	2214	1	2061	0	2621

TRINITY_DN15893_c0_g4_i1	Glyma.09G225600.2	100	177	15	342	2406	1	2041	0	2545

TRINITY_DN15893_c0_g4_i1	Glyma.09G225600.3	97.70992366	177	15	342	2406	1	2041	0	2545

TRINITY_DN15893_c0_g4_i2	Glyma.09G225600.2	95.35232384	177	15	342	2406	1	2041	0	2545

TRINITY_DN15893_c0_g4_i2	Glyma.09G225600.3	99.31192661	177	15	342	2406	1	2041	0	2545

TRINITY_DN15944_c2_g2_i7	Glyma.13G083300.1	100	175	19	269	2309	2074	1	0	2614

TRINITY_DN15944_c2_g2_i7	Glyma.13G083300.3	100	175	19	269	2309	2074	1	0	2614

TRINITY_DN15187_c0_g1_i3	Glyma.15G271800.1	100	173	1	617	2690	4201	2125	0	2857

TRINITY_DN9426_c0_g1_i1	Glyma.06G097500.1	99.03381643	170	2	55	2130	1	2076	0	2881

TRINITY_DN9426_c0_g1_i1	Glyma.04G095800.1	98.14814815	164	2	55	2130	1	2076	0	2915

TRINITY_DN16415_c1_g1_i15	Glyma.11G147900.1	98.41269841	158	4	1692	3767	2078	5	0	2935

TRINITY_DN16415_c1_g1_i4	Glyma.11G147900.1	98.16513761	158	4	1796	3871	2078	5	0	2935

TRINITY_DN5372_c0_g1_i1	Glyma.06G097200.2	99.4140625	136	2	172	2247	1	2076	0	3070

TRINITY_DN5372_c0_g1_i1	Glyma.06G097200.1	99.41520468	136	2	172	2247	1	2076	0	3070

TRINITY_DN13491_c0_g1_i1	Glyma.13G239300.1	99.15966387	125	0	1134	3210	2265	4341	0	3144

TRINITY_DN13491_c0_g1_i2	Glyma.13G239300.1	96.66666667	125	0	1134	3210	2265	4341	0	3144

TRINITY_DN13491_c0_g1_i3	Glyma.13G239300.1	96.25	125	0	1134	3210	2265	4341	0	3144

TRINITY_DN13491_c0_g1_i4	Glyma.13G239300.1	100	125	0	1134	3210	2265	4341	0	3144

TRINITY_DN13491_c0_g1_i5	Glyma.13G239300.1	100	125	0	1134	3210	2265	4341	0	3144

TRINITY_DN13491_c0_g1_i6	Glyma.13G239300.1	97.36842105	125	0	1134	3210	2265	4341	0	3144

TRINITY_DN2525_c0_g2_i1	Glyma.12G214500.5	98.828125	125	0	6	2082	911	2987	0	3144

TRINITY_DN142_c0_g1_i1	Glyma.18G243300.1	96.72131148	119	11	124	2196	1	2055	0	3009

TRINITY_DN16382_c0_g3_i3	Glyma.02G228700.1	96.66136725	291	19	344	2397	153	2223	0	2047

TRINITY_DN15024_c0_g1_i1	Glyma.06G321000.4	99.70760234	192	48	350	2355	2042	1	0	2098

TRINITY_DN15024_c0_g1_i1	Glyma.06G321000.2	100	192	48	350	2355	2042	1	0	2098

TRINITY_DN15024_c0_g1_i1	Glyma.06G321000.3	96.61016949	192	48	350	2355	2042	1	0	2098

TRINITY_DN15024_c0_g1_i2	Glyma.06G321000.4	97.61904762	192	48	518	2523	2042	1	0	2098

TRINITY_DN15024_c0_g1_i2	Glyma.06G321000.2	100	192	48	518	2523	2042	1	0	2098

TRINITY_DN15024_c0_g1_i2	Glyma.06G321000.3	100	192	48	518	2523	2042	1	0	2098

TRINITY_DN15024_c0_g1_i3	Glyma.06G321000.1	95.71428571	192	48	540	2545	2042	1	0	2098

TRINITY_DN17873_c0_g1_i1	Glyma.17G235900.2	97.79411765	171	14	1	2055	1397	3436	0	2518

TRINITY_DN17873_c0_g1_i1	Glyma.17G235900.1	96.62921348	171	14	1	2055	1397	3436	0	2518

TRINITY_DN16065_c0_g1_i1	Glyma.13G244300.2	100	134	1	572	2647	1	2073	0	3072

TRINITY_DN5372_c0_g1_i1	Glyma.04G095500.1	99.41348974	133	0	172	2247	1	2076	0	3097

TRINITY_DN10929_c0_g1_i1	Glyma.04G206300.1	97.40740741	115	10	35	2107	1	2061	0	3081

TRINITY_DN14677_c0_g1_i5	Glyma.17G021800.1	100	108	9	989	3055	2135	64	0	3151

TRINITY_DN14566_c0_g1_i1	Glyma.03G027500.1	97.72727273	285	3	1	2071	113	2186	0	2220

TRINITY_DN14021_c0_g2_i2	Glyma.01G224200.4	95.65217391	263	7	340	2404	2820	752	0	2272

TRINITY_DN14003_c0_g1_i1	Glyma.06G007500.1	97.44245524	244	21	190	2215	2074	1	0	2158

TRINITY_DN14003_c0_g1_i1	Glyma.06G007500.3	96.55172414	244	21	190	2215	2074	1	0	2158

TRINITY_DN14003_c0_g1_i1	Glyma.06G007500.2	96.55172414	244	21	190	2215	2074	1	0	2158

TRINITY_DN16006_c0_g1_i2	Glyma.09G052600.4	97.81420765	226	15	402	2468	2049	1	0	2361

TRINITY_DN9205_c0_g1_i3	Glyma.07G148100.7	100	190	6	794	2852	2074	1	0	2669

TRINITY_DN15893_c0_g4_i9	Glyma.12G010900.1	98.92473118	188	10	342	2412	1	2047	0	2584

TRINITY_DN9205_c0_g1_i3	Glyma.07G148100.6	97.05882353	188	12	794	2852	2050	1	0	2525

TRINITY_DN5823_c0_g1_i1	Glyma.04G088700.1	95.39170507	186	3	1	2071	828	2901	0	2769

TRINITY_DN5823_c0_g1_i1	Glyma.06G090700.1	95.39170507	182	3	1	2071	819	2892	0	2791

TRINITY_DN14566_c0_g1_i2	Glyma.03G027500.1	97.59036145	181	3	1	2071	113	2186	0	2796

TRINITY_DN13021_c0_g1_i1	Glyma.18G280200.1	100	169	19	867	2896	209	2271	0	2527

TRINITY_DN10765_c0_g1_i1	Glyma.11G038500.4	98	163	5	845	2910	2266	200	0	2819

TRINITY_DN11819_c0_g3_i3	Glyma.16G048700.1	96.76806084	162	13	224	2278	49	2121	0	2793

TRINITY_DN14021_c0_g2_i1	Glyma.01G224200.4	100	155	7	1475	3540	2820	752	0	2876

TRINITY_DN7895_c0_g2_i1	Glyma.03G091800.1	100	155	8	1	2059	245	2304	0	2772

TRINITY_DN12976_c0_g1_i3	Glyma.03G245900.1	98.68421053	143	13	55	2111	1	2069	0	2885

TRINITY_DN14677_c0_g1_i5	Glyma.07G252600.1	100	126	3	986	3060	2201	130	0	3114

TRINITY_DN14064_c0_g1_i4	Glyma.13G209800.2	95.61510353	108	1	1893	3964	2075	1	0	3214

TRINITY_DN16441_c4_g2_i3	Glyma.07G201200.1	100	322	16	526	2588	5016	2960	0	1866

TRINITY_DN16441_c4_g2_i3	Glyma.07G201200.2	97.27272727	322	16	526	2588	5013	2957	0	1866

TRINITY_DN9205_c0_g1_i2	Glyma.07G148100.7	99.53703704	190	6	652	2709	2073	1	0	2667

TRINITY_DN9205_c0_g1_i3	Glyma.07G148100.11	98.57142857	190	6	795	2852	2073	1	0	2667

TRINITY_DN9205_c0_g1_i4	Glyma.07G148100.11	95.62043796	190	6	556	2613	2073	1	0	2667

TRINITY_DN9205_c0_g1_i2	Glyma.07G148100.6	97.01492537	188	12	652	2709	2049	1	0	2523

TRINITY_DN9205_c0_g1_i3	Glyma.07G148100.10	96.20991254	188	12	795	2852	2049	1	0	2523

TRINITY_DN9205_c0_g1_i4	Glyma.07G148100.10	100	188	12	556	2613	2049	1	0	2523

TRINITY_DN16360_c0_g4_i3	Glyma.15G023800.1	95	182	3	229	2298	11	2083	0	2789

TRINITY_DN15944_c2_g2_i7	Glyma.13G083300.2	100	175	18	269	2309	2071	1	0	2628

TRINITY_DN15944_c2_g2_i7	Glyma.13G083300.4	100	175	18	269	2309	2071	1	0	2628

TRINITY_DN15235_c0_g1_i1	Glyma.10G227300.5	100	307	4	2044	4114	2380	4450	0	2102

TRINITY_DN15235_c0_g1_i1	Glyma.10G227300.4	96.10778443	307	4	2044	4114	2380	4450	0	2102

TRINITY_DN15235_c0_g1_i1	Glyma.10G227300.3	97.65100671	307	4	2044	4114	2380	4450	0	2102

TRINITY_DN15235_c0_g1_i1	Glyma.10G227300.1	95.12195122	307	4	2044	4114	2380	4450	0	2102

TRINITY_DN15235_c0_g1_i1	Glyma.10G227300.2	96.19047619	307	4	2044	4114	2380	4450	0	2102

TRINITY_DN14641_c2_g2_i3	Glyma.17G047100.1	99.65635739	290	23	99	2135	2124	58	0	1951

TRINITY_DN16006_c0_g1_i2	Glyma.09G052600.1	95.45454545	227	13	402	2469	2067	21	0	2370

TRINITY_DN11257_c0_g1_i1	Glyma.04G053100.2	95.58823529	223	18	223	2273	1	2058	0	2353

TRINITY_DN14958_c0_g1_i2	Glyma.08G053300.1	98.07692308	187	6	192	2249	1	2070	0	2676

TRINITY_DN14958_c0_g1_i2	Glyma.05G244800.1	98.07692308	177	4	192	2249	1	2070	0	2732

TRINITY_DN16006_c0_g1_i1	Glyma.05G132000.1	97.5177305	174	4	285	2353	2069	1	0	2813

TRINITY_DN9496_c0_g1_i1	Glyma.09G237200.1	96.38554217	162	0	109	2181	1	2073	0	2931

TRINITY_DN15235_c0_g1_i1	Glyma.20G165000.1	95.13677812	161	0	2044	4116	1651	3723	0	2937

TRINITY_DN15235_c0_g1_i1	Glyma.20G165000.2	95.78059072	161	0	2044	4116	1651	3723	0	2937

TRINITY_DN15466_c0_g1_i1	Glyma.04G203300.1	95.52238806	160	4	434	2499	244	2315	0	2891

TRINITY_DN15235_c0_g1_i1	Glyma.10G227400.6	98.95833333	156	0	2044	4116	2368	4440	0	2964

TRINITY_DN15235_c0_g1_i1	Glyma.10G227400.4	95.48022599	156	0	2044	4116	2368	4440	0	2964

TRINITY_DN15235_c0_g1_i1	Glyma.10G227400.3	97.81718963	156	0	2044	4116	2368	4440	0	2964

TRINITY_DN15235_c0_g1_i1	Glyma.10G227400.1	100	156	0	2044	4116	2368	4440	0	2964

TRINITY_DN15235_c0_g1_i1	Glyma.10G227400.2	97.46835443	156	0	2044	4116	2368	4440	0	2964

TRINITY_DN15235_c0_g1_i1	Glyma.10G227400.5	95.13381995	156	0	2044	4116	2368	4440	0	2964

TRINITY_DN38193_c0_g1_i1	Glyma.08G227500.6	100	150	1	1	2070	243	2315	0	2977

TRINITY_DN38193_c0_g1_i1	Glyma.08G227500.7	100	150	1	1	2070	243	2315	0	2977

TRINITY_DN38193_c0_g1_i1	Glyma.08G227500.8	100	150	1	1	2070	243	2315	0	2977

TRINITY_DN7713_c0_g1_i2	Glyma.13G370300.3	98.50746269	122	2	199	2268	956	3028	0	3133

TRINITY_DN7713_c0_g1_i2	Glyma.13G370300.6	98.38709677	122	2	199	2268	956	3028	0	3133

TRINITY_DN7713_c0_g1_i2	Glyma.13G370300.5	100	122	2	199	2268	956	3028	0	3133

TRINITY_DN7713_c0_g1_i2	Glyma.13G370300.2	100	122	2	199	2268	956	3028	0	3133

TRINITY_DN7713_c0_g1_i2	Glyma.13G370300.4	100	122	2	199	2268	956	3028	0	3133

TRINITY_DN7713_c0_g1_i2	Glyma.13G370300.1	100	122	2	199	2268	956	3028	0	3133

TRINITY_DN7491_c0_g1_i1	Glyma.07G089600.1	100	103	0	233	2305	2073	1	0	3258

TRINITY_DN12822_c0_g1_i1	Glyma.05G145400.1	100	422	48	902	2949	2030	1	0	1062

TRINITY_DN12822_c0_g1_i4	Glyma.05G145400.1	95.87628866	422	48	902	2949	2030	1	0	1062

TRINITY_DN2573_c0_g1_i1	Glyma.03G089100.1	100	367	17	158	2221	2367	307	0	1670

TRINITY_DN15506_c0_g2_i1	Glyma.13G179600.2	100	364	16	888	2951	18	2081	0	1707

TRINITY_DN15506_c0_g2_i1	Glyma.13G179600.1	99.03846154	364	16	888	2951	18	2081	0	1707

TRINITY_DN15506_c0_g2_i10	Glyma.13G179600.2	99.07407407	364	16	971	3034	18	2081	0	1707

TRINITY_DN15506_c0_g2_i10	Glyma.13G179600.1	95.83333333	364	16	971	3034	18	2081	0	1707

TRINITY_DN15506_c0_g2_i14	Glyma.13G179600.2	100	364	16	970	3033	18	2081	0	1707

TRINITY_DN15506_c0_g2_i14	Glyma.13G179600.1	100	364	16	970	3033	18	2081	0	1707

TRINITY_DN15506_c0_g2_i2	Glyma.13G179600.2	95.53571429	364	16	1285	3348	18	2081	0	1707

TRINITY_DN15506_c0_g2_i2	Glyma.13G179600.1	95.53571429	364	16	1285	3348	18	2081	0	1707

TRINITY_DN15506_c0_g2_i7	Glyma.13G179600.2	98.73417722	364	16	1053	3116	18	2081	0	1707

TRINITY_DN15506_c0_g2_i7	Glyma.13G179600.1	100	364	16	1053	3116	18	2081	0	1707

TRINITY_DN15506_c0_g2_i9	Glyma.13G179600.2	100	364	16	1202	3265	18	2081	0	1707

TRINITY_DN15506_c0_g2_i9	Glyma.13G179600.1	96.59090909	364	16	1202	3265	18	2081	0	1707

TRINITY_DN8174_c0_g2_i1	Glyma.15G091500.1	96	303	22	662	2713	2067	1	0	1986

TRINITY_DN14389_c1_g2_i2	Glyma.03G070500.1	99.41520468	285	30	313	2349	25	2076	0	1892

TRINITY_DN16006_c0_g1_i2	Glyma.09G052600.5	96.55172414	227	13	402	2468	2046	1	0	2368

TRINITY_DN16006_c0_g1_i2	Glyma.09G052600.3	99.46524064	227	13	402	2468	2046	1	0	2368

TRINITY_DN16006_c0_g1_i2	Glyma.09G052600.2	97.0505618	227	13	402	2468	2046	1	0	2368

TRINITY_DN16082_c0_g1_i10	Glyma.07G016700.2	100	213	3	188	2257	2247	178	0	2621

TRINITY_DN16082_c0_g1_i10	Glyma.08G202000.2	100	210	3	188	2257	2253	184	0	2638

TRINITY_DN6354_c0_g2_i2	Glyma.08G239300.1	99.16666667	167	4	38	2106	3063	996	0	2856

TRINITY_DN6354_c0_g2_i1	Glyma.08G239300.1	99.16317992	166	5	258	2325	3063	996	0	2856

TRINITY_DN6354_c0_g2_i2	Glyma.12G001500.1	99.1643454	165	4	38	2106	3033	966	0	2867

TRINITY_DN6354_c0_g2_i1	Glyma.12G001500.1	96.92307692	164	5	258	2325	3033	966	0	2867

TRINITY_DN36295_c0_g1_i1	Glyma.16G208400.14	100	153	6	373	2436	1	2070	0	2915

TRINITY_DN14516_c0_g1_i5	Glyma.17G094800.2	96.89922481	89	4	174	2224	1	2072	0	3197

TRINITY_DN14516_c0_g1_i5	Glyma.17G094800.1	100	89	4	174	2224	1	2072	0	3197

TRINITY_DN14516_c0_g1_i5	Glyma.05G031900.1	100	89	8	174	2224	1	2069	0	3179

TRINITY_DN2573_c0_g1_i1	Glyma.16G084100.1	100	362	15	158	2221	2367	307	0	1709

TRINITY_DN13097_c0_g1_i3	Glyma.18G094400.2	97.10144928	339	40	236	2263	2061	22	0	1469

TRINITY_DN21836_c0_g2_i1	Glyma.01G019300.1	97.70114943	257	36	113	2146	2043	1	0	1980

TRINITY_DN14531_c1_g1_i1	Glyma.14G212100.1	95.37815126	236	10	424	2484	2826	757	0	2446

TRINITY_DN16006_c0_g1_i2	Glyma.15G158900.1	97.96610169	227	9	402	2468	2052	1	0	2418

TRINITY_DN16006_c0_g1_i2	Glyma.15G158900.2	97.96672828	227	9	402	2468	2052	1	0	2418

TRINITY_DN16345_c0_g1_i6	Glyma.08G044700.4	98.4375	193	1	446	2513	3594	1524	0	2736

TRINITY_DN16345_c0_g1_i6	Glyma.08G044700.2	98.4375	193	1	446	2513	3399	1329	0	2736

TRINITY_DN3038_c0_g1_i1	Glyma.13G116400.2	96.07843137	168	2	424	2491	1	2071	0	2874

TRINITY_DN13654_c0_g1_i1	Glyma.19G261700.3	98.85057471	155	1	325	2395	2091	24	0	2946

TRINITY_DN13654_c0_g1_i1	Glyma.19G261700.2	98.85057471	155	1	325	2395	2091	24	0	2946

TRINITY_DN15944_c2_g2_i1	Glyma.06G109500.1	100	133	4	269	2324	2071	1	0	2990

TRINITY_DN15944_c2_g2_i1	Glyma.06G109500.2	100	133	5	269	2324	2068	1	0	2972

TRINITY_DN8174_c0_g2_i1	Glyma.13G221000.1	99.09090909	307	17	662	2713	2067	1	0	1986

TRINITY_DN9907_c0_g1_i1	Glyma.10G000500.1	100	228	9	64	2123	14	2082	0	2488

TRINITY_DN14645_c0_g1_i3	Glyma.09G059000.1	96.88385269	217	15	49	2096	2069	1	0	2471

TRINITY_DN14645_c0_g1_i3	Glyma.09G059000.2	95.04132231	217	15	49	2096	2069	1	0	2471

TRINITY_DN14531_c1_g1_i2	Glyma.14G212100.1	96.4028777	214	8	424	2484	2826	757	0	2579

TRINITY_DN14680_c0_g2_i1	Glyma.19G175600.1	99.36305732	209	20	1	2049	374	2425	0	2412

TRINITY_DN14680_c0_g2_i1	Glyma.19G175600.2	100	209	20	1	2049	374	2425	0	2412

TRINITY_DN14680_c0_g2_i2	Glyma.19G175600.1	99	209	20	1	2049	374	2425	0	2412

TRINITY_DN14680_c0_g2_i2	Glyma.19G175600.2	100	209	20	1	2049	374	2425	0	2412

TRINITY_DN14680_c0_g2_i3	Glyma.19G175600.4	100	209	20	1	2049	374	2425	0	2412

TRINITY_DN14680_c0_g2_i3	Glyma.19G175600.3	100	209	20	1	2049	374	2425	0	2412

TRINITY_DN14680_c0_g2_i4	Glyma.19G175600.4	99.08536585	209	20	1	2049	374	2425	0	2412

TRINITY_DN14680_c0_g2_i4	Glyma.19G175600.3	99.08675799	209	20	1	2049	374	2425	0	2412

TRINITY_DN14680_c0_g2_i5	Glyma.19G175600.1	96.90721649	209	20	1	2049	374	2425	0	2412

TRINITY_DN14680_c0_g2_i5	Glyma.19G175600.2	95.40229885	209	20	1	2049	374	2425	0	2412

TRINITY_DN14680_c0_g2_i6	Glyma.19G175600.1	98.7804878	209	20	1	2049	374	2425	0	2412

TRINITY_DN14680_c0_g2_i6	Glyma.19G175600.2	98.7804878	209	20	1	2049	374	2425	0	2412

TRINITY_DN10838_c0_g1_i1	Glyma.15G083500.2	97.36842105	198	22	640	2694	2034	1	0	2396

TRINITY_DN10838_c0_g1_i1	Glyma.15G083500.1	96.41350211	198	22	640	2694	2034	1	0	2396

TRINITY_DN10838_c0_g1_i2	Glyma.15G083500.2	97.08737864	198	22	1080	3134	2034	1	0	2396

TRINITY_DN10838_c0_g1_i2	Glyma.15G083500.1	98.10126582	198	22	1080	3134	2034	1	0	2396

TRINITY_DN10838_c0_g1_i3	Glyma.15G083500.2	98.13084112	198	22	1080	3134	2034	1	0	2396

TRINITY_DN10838_c0_g1_i3	Glyma.15G083500.1	95.4954955	198	22	1080	3134	2034	1	0	2396

TRINITY_DN10838_c0_g1_i4	Glyma.15G083500.2	98.9821883	198	22	640	2694	2034	1	0	2396

TRINITY_DN10838_c0_g1_i4	Glyma.15G083500.1	95.89041096	198	22	640	2694	2034	1	0	2396

TRINITY_DN10838_c0_g1_i5	Glyma.15G083500.2	98.7012987	198	22	687	2741	2034	1	0	2396

TRINITY_DN10838_c0_g1_i5	Glyma.15G083500.1	96.2962963	198	22	687	2741	2034	1	0	2396

TRINITY_DN5469_c0_g1_i1	Glyma.19G149500.1	100	191	0	42	2111	460	2529	0	2765

TRINITY_DN5469_c0_g2_i1	Glyma.19G149500.1	100	191	0	42	2111	460	2529	0	2765

TRINITY_DN16082_c0_g1_i13	Glyma.08G202000.2	100	190	0	142	2211	2253	184	0	2771

TRINITY_DN9812_c0_g2_i1	Glyma.15G021300.2	96.07843137	190	0	894	2963	2070	1	0	2771

TRINITY_DN16082_c0_g1_i13	Glyma.07G016700.2	100	189	0	142	2211	2247	178	0	2776

TRINITY_DN8710_c0_g1_i1	Glyma.18G027000.4	100	188	4	390	2457	2480	413	0	2756

TRINITY_DN8710_c0_g1_i2	Glyma.18G027000.4	100	188	5	390	2441	2480	413	0	2652

TRINITY_DN21836_c0_g1_i1	Glyma.01G019300.1	99.03846154	257	35	113	2143	2043	1	0	1984

TRINITY_DN15893_c0_g4_i1	Glyma.12G010900.1	97.40932642	180	8	342	2406	1	2047	0	2656

TRINITY_DN15893_c0_g4_i2	Glyma.12G010900.1	97.59036145	180	8	342	2406	1	2047	0	2656

TRINITY_DN9236_c0_g1_i1	Glyma.17G193800.1	100	175	4	254	2318	2097	30	0	2819

TRINITY_DN13658_c0_g1_i1	Glyma.14G189100.1	99.76851852	175	12	154	2214	1	2049	0	2671

TRINITY_DN7895_c0_g2_i1	Glyma.16G082400.1	100	158	7	1	2059	245	2298	0	2784

TRINITY_DN2154_c0_g1_i1	Glyma.15G101800.1	100	354	21	230	2284	2059	2	0	1709

TRINITY_DN16251_c0_g3_i7	Glyma.09G060600.1	96.57947686	261	16	357	2415	2523	472	0	2211

TRINITY_DN16251_c0_g3_i7	Glyma.09G060600.4	100	261	16	357	2415	2508	457	0	2211

TRINITY_DN16251_c0_g3_i7	Glyma.09G060600.3	96.41693811	260	17	357	2415	2502	472	0	2082

TRINITY_DN16251_c0_g3_i7	Glyma.09G060600.2	96.41577061	260	17	357	2415	2487	457	0	2082

TRINITY_DN16251_c0_g3_i7	Glyma.09G060600.5	96.59090909	259	18	357	2415	2520	472	0	2204

TRINITY_DN16151_c0_g2_i1	Glyma.08G292900.1	95	238	36	1133	3175	830	2848	0	2023

TRINITY_DN16151_c0_g2_i1	Glyma.08G292900.5	100	238	36	1133	3175	830	2848	0	2023

TRINITY_DN16151_c0_g2_i1	Glyma.08G292900.3	98.56115108	238	37	1133	3175	830	2845	0	2002

TRINITY_DN16151_c0_g2_i1	Glyma.08G292900.6	100	238	37	1133	3175	830	2845	0	2002

TRINITY_DN16151_c0_g2_i1	Glyma.08G292900.4	95.34883721	238	37	1133	3175	830	2845	0	2002

TRINITY_DN16151_c0_g2_i1	Glyma.08G292900.2	95.96774194	238	37	1133	3175	830	2845	0	2002

TRINITY_DN14146_c1_g1_i1	Glyma.20G228700.1	98.85386819	234	16	1398	3460	2048	1	0	2361

TRINITY_DN14146_c1_g1_i1	Glyma.20G228700.4	100	234	16	1398	3460	2048	1	0	2361

TRINITY_DN8710_c0_g1_i1	Glyma.18G027000.2	100	188	4	392	2457	2478	413	0	2752

TRINITY_DN8710_c0_g1_i1	Glyma.18G027000.3	100	188	4	392	2457	2478	413	0	2752

TRINITY_DN8710_c0_g1_i2	Glyma.18G027000.2	96.84210526	188	5	392	2441	2478	413	0	2649

TRINITY_DN8710_c0_g1_i2	Glyma.18G027000.3	100	188	5	392	2441	2478	413	0	2649

TRINITY_DN12257_c0_g1_i2	Glyma.15G127700.2	97.5	122	2	278	2345	1	2055	0	3059

TRINITY_DN15329_c0_g2_i1	Glyma.05G238700.2	100	106	0	857	2924	2068	1	0	3232

TRINITY_DN15329_c0_g2_i2	Glyma.05G238700.2	95.07874016	106	0	481	2548	2068	1	0	3232

TRINITY_DN14516_c0_g1_i2	Glyma.17G094800.2	97.16981132	87	4	209	2255	120	2187	0	3201

TRINITY_DN15345_c0_g1_i3	Glyma.06G305000.2	100	345	22	709	2758	1	2056	0	1725

TRINITY_DN15345_c0_g1_i4	Glyma.06G305000.2	99	345	22	515	2564	1	2056	0	1725

TRINITY_DN14043_c0_g2_i3	Glyma.07G250400.1	98.33333333	185	11	251	2290	1	2058	0	2560

TRINITY_DN9290_c0_g1_i3	Glyma.13G237200.1	98.55072464	166	2	6	2063	184	2250	0	2839

TRINITY_DN15328_c0_g1_i1	Glyma.16G025800.2	98.79518072	166	7	3	2057	1359	3410	0	2723

TRINITY_DN15328_c0_g1_i3	Glyma.16G025800.2	100	166	7	3	2057	1359	3410	0	2723

TRINITY_DN14473_c0_g2_i1	Glyma.03G164400.2	98.66071429	146	1	79	2145	1	2064	0	2988

TRINITY_DN14473_c0_g2_i1	Glyma.03G164400.3	98.66071429	146	1	79	2145	1	2064	0	2988

TRINITY_DN14473_c0_g2_i1	Glyma.03G164400.1	98.66156788	146	1	79	2145	1	2064	0	2988

TRINITY_DN12036_c0_g1_i2	Glyma.12G010000.1	98.96907216	145	7	141	2207	4	2055	0	2916

TRINITY_DN16376_c3_g12_i1	Glyma.05G151100.3	96.23015873	134	17	124	2184	5	2047	0	2881

TRINITY_DN16376_c3_g12_i5	Glyma.05G151100.3	96	134	17	124	2184	5	2047	0	2881

TRINITY_DN9805_c0_g1_i1	Glyma.08G324500.1	99.44134078	407	37	614	2655	2147	103	0	1269

TRINITY_DN11482_c0_g1_i1	Glyma.14G090700.1	96.73202614	359	7	415	2479	1	2059	0	1775

TRINITY_DN11482_c0_g1_i1	Glyma.U038600.2	96.79849341	358	7	415	2479	1	2059	0	1781

TRINITY_DN11482_c0_g1_i1	Glyma.U038600.1	100	358	7	415	2479	1	2059	0	1781

TRINITY_DN11482_c0_g1_i1	Glyma.U038600.3	100	358	7	415	2479	1	2059	0	1781

TRINITY_DN12848_c0_g1_i1	Glyma.14G062000.1	98	220	13	172	2217	1	2061	0	2435

TRINITY_DN16360_c0_g4_i3	Glyma.13G350700.1	95.0166113	177	2	234	2298	22	2086	0	2822

TRINITY_DN15764_c0_g1_i4	Glyma.16G181000.1	95.98930481	130	6	34	2086	30	2094	0	3005

TRINITY_DN15764_c0_g1_i7	Glyma.16G181000.1	98.85057471	130	6	34	2086	30	2094	0	3005

TRINITY_DN15764_c0_g1_i9	Glyma.16G181000.1	98.9010989	130	6	34	2086	30	2094	0	3005

TRINITY_DN16257_c0_g1_i4	Glyma.12G192100.1	96.8503937	487	59	293	2315	2055	21	0	651

TRINITY_DN15909_c0_g3_i1	Glyma.06G068600.1	100	471	68	286	2309	193	2213	0	654

TRINITY_DN15345_c0_g1_i3	Glyma.12G099500.5	100	319	20	709	2758	1	2053	0	1871

TRINITY_DN15345_c0_g1_i3	Glyma.12G099500.2	100	319	20	709	2758	1	2053	0	1871

TRINITY_DN15345_c0_g1_i3	Glyma.12G099500.3	100	319	20	709	2758	1	2053	0	1871

TRINITY_DN15345_c0_g1_i4	Glyma.12G099500.5	96.11650485	319	20	515	2564	1	2053	0	1871

TRINITY_DN15345_c0_g1_i4	Glyma.12G099500.2	96.11650485	319	20	515	2564	1	2053	0	1871

TRINITY_DN15345_c0_g1_i4	Glyma.12G099500.3	96.11650485	319	20	515	2564	1	2053	0	1871

TRINITY_DN14146_c1_g1_i1	Glyma.20G228700.3	98.67021277	235	15	1398	3460	2045	1	0	2370

TRINITY_DN14146_c1_g1_i1	Glyma.20G228700.2	96.17834395	235	15	1398	3460	2045	1	0	2370

TRINITY_DN12848_c0_g1_i1	Glyma.02G254400.1	98	209	17	172	2217	1	2052	0	2449

TRINITY_DN15750_c1_g1_i3	Glyma.09G062900.1	96.40718563	193	1	736	2800	2062	1	0	2724

TRINITY_DN15750_c1_g1_i3	Glyma.09G062900.2	96.40718563	193	1	736	2800	2062	1	0	2724

TRINITY_DN13751_c0_g1_i2	Glyma.03G162100.4	100	188	16	543	2595	1	2059	0	2656

TRINITY_DN13751_c0_g1_i2	Glyma.03G162100.3	98.24561404	188	16	543	2595	1	2059	0	2656

TRINITY_DN13751_c0_g1_i2	Glyma.03G162100.1	96.79487179	188	16	543	2595	1	2059	0	2656

TRINITY_DN13751_c0_g1_i2	Glyma.03G162100.2	98.48484848	188	16	543	2595	1	2059	0	2656

TRINITY_DN13751_c0_g1_i2	Glyma.03G162100.5	97.91144528	188	16	543	2595	1	2059	0	2656

TRINITY_DN15750_c1_g1_i3	Glyma.15G169500.2	100	183	1	736	2800	2062	1	0	2780

TRINITY_DN15750_c1_g1_i3	Glyma.15G169500.1	96.69421488	183	1	736	2800	2062	1	0	2780

TRINITY_DN2619_c0_g1_i1	Glyma.13G348300.1	99	174	3	235	2298	1	2063	0	2830

TRINITY_DN15328_c0_g1_i1	Glyma.16G025800.1	98.4	166	7	3	2055	1359	3408	0	2719

TRINITY_DN15328_c0_g1_i3	Glyma.16G025800.1	100	166	7	3	2055	1359	3408	0	2719

TRINITY_DN12976_c0_g1_i3	Glyma.19G243400.1	98.68421053	159	8	55	2111	1	2060	0	2848

TRINITY_DN14473_c0_g2_i1	Glyma.19G165800.1	98.98989899	146	0	79	2143	1	2065	0	3005

TRINITY_DN15143_c0_g2_i2	Glyma.07G089400.1	98.20359281	145	1	236	2300	45	2106	0	2990

TRINITY_DN15143_c0_g2_i2	Glyma.09G187400.1	96.68049793	141	6	236	2300	45	2094	0	2935

TRINITY_DN4333_c0_g1_i1	Glyma.02G111500.4	100	141	7	411	2472	2049	1	0	2909

TRINITY_DN4333_c0_g1_i1	Glyma.02G111500.5	98.57142857	141	7	411	2472	2049	1	0	2909

TRINITY_DN15944_c2_g2_i1	Glyma.04G253000.1	97.43589744	133	6	269	2324	2053	1	0	2940

TRINITY_DN15980_c0_g2_i1	Glyma.10G004100.1	98.7804878	239	24	764	2804	1680	3720	0	2191

TRINITY_DN15980_c0_g2_i1	Glyma.10G004100.2	96.15384615	239	24	764	2804	1260	3300	0	2191

TRINITY_DN15897_c0_g2_i3	Glyma.18G108100.1	99.28057554	239	29	164	2220	3152	1132	0	2165

TRINITY_DN16151_c0_g2_i1	Glyma.18G130400.3	97.70491803	233	30	1133	3175	797	2809	0	2056

TRINITY_DN16151_c0_g2_i1	Glyma.18G130400.1	100	233	30	1133	3175	797	2809	0	2056

TRINITY_DN7308_c0_g1_i2	Glyma.08G153100.1	100	199	7	1222	3279	2046	1	0	2555

TRINITY_DN7308_c0_g1_i2	Glyma.08G153100.2	99.02912621	199	7	1222	3279	2046	1	0	2555

TRINITY_DN7308_c0_g1_i1	Glyma.08G153100.1	97.97297297	198	8	1222	3264	2046	1	0	2462

TRINITY_DN7308_c0_g1_i1	Glyma.08G153100.2	97.97297297	198	8	1222	3264	2046	1	0	2462

TRINITY_DN7308_c0_g1_i2	Glyma.08G153100.3	100	198	8	1222	3279	2031	1	0	2462

TRINITY_DN1079_c0_g2_i1	Glyma.05G125700.1	98.07692308	174	3	198	2255	2064	1	0	2809

TRINITY_DN2573_c0_g1_i1	Glyma.10G112400.1	100	170	0	158	2221	2370	307	0	2870

TRINITY_DN2573_c0_g1_i1	Glyma.02G160300.1	100	160	0	158	2221	2370	307	0	2926

TRINITY_DN5903_c0_g1_i1	Glyma.11G093200.1	96.71457906	151	2	460	2514	2064	1	0	2916

TRINITY_DN17473_c0_g1_i1	Glyma.01G000600.1	97.54716981	122	4	320	2382	100	2160	0	3110

TRINITY_DN16376_c3_g12_i3	Glyma.05G151100.3	96.21621622	119	14	124	2181	5	2047	0	2977

TRINITY_DN12519_c0_g1_i2	Glyma.20G017000.3	96.92780338	101	0	953	3016	1	2064	0	3253

TRINITY_DN12519_c0_g1_i1	Glyma.20G017000.3	97.60609358	100	1	953	3000	1	2064	0	3155

TRINITY_DN4045_c0_g1_i1	Glyma.08G098100.2	95	224	17	65	2094	160	2216	0	2316

TRINITY_DN4045_c0_g1_i1	Glyma.08G098100.1	98.92473118	224	17	65	2094	160	2216	0	2316

TRINITY_DN15302_c0_g1_i3	Glyma.13G092500.1	100	223	7	733	2788	1	2059	0	2503

TRINITY_DN15302_c0_g1_i4	Glyma.13G092500.1	95.55555556	223	7	839	2894	1	2059	0	2503

TRINITY_DN14621_c1_g1_i1	Glyma.19G055500.2	100	208	19	314	2365	3	2055	0	2521

TRINITY_DN15970_c0_g1_i5	Glyma.02G251100.1	96.12903226	181	2	137	2198	6	2067	0	2795

TRINITY_DN15970_c0_g1_i5	Glyma.14G065600.1	96.12903226	180	2	137	2198	6	2067	0	2800

TRINITY_DN14621_c1_g1_i3	Glyma.19G055500.2	96.66666667	171	19	314	2365	3	2055	0	2726

TRINITY_DN14643_c0_g1_i1	Glyma.12G168900.1	96.80851064	164	2	107	2168	9	2070	0	2889

TRINITY_DN11579_c0_g1_i2	Glyma.13G340700.1	100	136	7	73	2127	1	2058	0	2972

TRINITY_DN15970_c0_g1_i1	Glyma.02G251100.1	100	114	2	137	2198	6	2067	0	3166

TRINITY_DN15200_c0_g1_i4	Glyma.09G154600.1	100	294	17	252	2294	2052	1	0	1991

TRINITY_DN14750_c0_g1_i1	Glyma.15G058700.2	95.23809524	216	16	913	2961	2049	1	0	2444

TRINITY_DN11814_c0_g2_i1	Glyma.02G185000.1	97.76119403	195	9	508	2559	2058	1	0	2638

TRINITY_DN14621_c1_g1_i4	Glyma.19G055500.2	96.2962963	183	13	336	2389	1	2055	0	2697

TRINITY_DN13448_c0_g1_i2	Glyma.08G100800.3	96.66666667	164	7	1	2054	27	2080	0	2796

TRINITY_DN13448_c0_g1_i2	Glyma.08G100800.2	97	164	7	1	2054	27	2080	0	2796

TRINITY_DN13448_c0_g1_i2	Glyma.08G100800.1	97.5	164	7	1	2054	27	2080	0	2796

TRINITY_DN11811_c0_g2_i1	Glyma.01G068800.1	97.10144928	162	6	159	2205	1	2046	0	2710

TRINITY_DN11811_c0_g2_i2	Glyma.01G068800.1	97.11538462	162	6	159	2205	1	2046	0	2710

TRINITY_DN11811_c0_g2_i1	Glyma.02G125400.11	97.11538462	152	6	159	2205	1	2046	0	2765

TRINITY_DN11811_c0_g2_i1	Glyma.02G125400.5	97.11538462	152	6	159	2205	1	2046	0	2765

TRINITY_DN11811_c0_g2_i2	Glyma.02G125400.11	97.11538462	152	6	159	2205	1	2046	0	2765

TRINITY_DN11811_c0_g2_i2	Glyma.02G125400.5	97.10982659	152	6	159	2205	1	2046	0	2765

TRINITY_DN14621_c1_g1_i2	Glyma.19G055500.2	100	146	13	336	2389	1	2055	0	2902

TRINITY_DN15944_c2_g2_i7	Glyma.14G164600.4	100	146	14	269	2309	2059	1	0	2844

TRINITY_DN15944_c2_g2_i7	Glyma.14G164600.1	100	146	14	269	2309	2059	1	0	2844

TRINITY_DN12916_c0_g1_i1	Glyma.17G158300.3	98.30508475	145	34	312	2306	1	2034	0	2390

TRINITY_DN20293_c0_g1_i1	Glyma.14G001500.1	96.17346939	130	3	64	2125	1	2056	0	3049

TRINITY_DN20293_c0_g1_i1	Glyma.14G001500.2	98.26989619	128	7	64	2125	1	2047	0	3001

TRINITY_DN15970_c0_g1_i1	Glyma.14G065600.1	100	112	0	137	2198	6	2067	0	3188

TRINITY_DN12257_c0_g1_i1	Glyma.09G021400.2	97.5	112	2	118	2179	148	2205	0	3162

TRINITY_DN11456_c0_g1_i1	Glyma.15G183400.1	98.7987988	108	4	285	2342	2058	1	0	3158

TRINITY_DN15200_c0_g1_i3	Glyma.09G154600.1	98.7654321	268	15	252	2294	2052	1	0	2146

TRINITY_DN16149_c1_g1_i1	Glyma.17G191000.1	96.73913043	257	37	407	2415	2018	1	0	1768

TRINITY_DN13198_c0_g1_i1	Glyma.10G209800.1	97.78597786	195	2	144	2203	1	2060	0	2713

TRINITY_DN13926_c0_g1_i1	Glyma.11G008300.3	100	169	15	239	2245	3054	994	0	2521

TRINITY_DN13926_c0_g1_i1	Glyma.11G008300.1	97.05882353	169	15	239	2245	3054	994	0	2521

TRINITY_DN13926_c0_g1_i1	Glyma.11G008300.4	100	169	15	239	2245	2361	301	0	2521

TRINITY_DN13926_c0_g1_i1	Glyma.11G008300.2	98.30508475	169	15	239	2245	2361	301	0	2521

TRINITY_DN7895_c0_g1_i1	Glyma.03G091800.1	100	155	7	1	2045	245	2304	0	2837

TRINITY_DN15345_c0_g1_i3	Glyma.12G194200.3	97.77777778	153	0	698	2758	44	2104	0	2959

TRINITY_DN15345_c0_g1_i4	Glyma.12G194200.3	97.49103943	153	0	504	2564	44	2104	0	2959

TRINITY_DN14881_c0_g2_i1	Glyma.08G019800.1	95.10961214	148	5	430	2478	1	2049	0	2832

TRINITY_DN14881_c0_g2_i1	Glyma.08G019800.2	95.83333333	148	5	430	2478	1	2049	0	2832

TRINITY_DN14881_c0_g2_i1	Glyma.08G019800.3	97	148	5	430	2478	1	2049	0	2832

TRINITY_DN14881_c0_g2_i2	Glyma.08G019800.1	98.5915493	148	5	462	2510	1	2049	0	2832

TRINITY_DN14881_c0_g2_i2	Glyma.08G019800.2	98.5915493	148	5	462	2510	1	2049	0	2832

TRINITY_DN14881_c0_g2_i2	Glyma.08G019800.3	95.18072289	148	5	462	2510	1	2049	0	2832

TRINITY_DN14881_c0_g2_i3	Glyma.08G019800.1	98.4	148	5	1566	3614	1	2049	0	2832

TRINITY_DN14881_c0_g2_i3	Glyma.08G019800.2	97.82608696	148	5	1566	3614	1	2049	0	2832

TRINITY_DN14881_c0_g2_i3	Glyma.08G019800.3	99.25925926	148	5	1566	3614	1	2049	0	2832

TRINITY_DN13775_c0_g1_i1	Glyma.03G169000.2	100	148	8	179	2209	49	2100	0	2734

TRINITY_DN13775_c0_g1_i2	Glyma.03G169000.2	99.03846154	148	8	179	2209	49	2100	0	2734

TRINITY_DN16138_c1_g1_i17	Glyma.15G049300.1	98.14814815	320	21	311	2350	1	2049	0	1831

TRINITY_DN16190_c0_g3_i5	Glyma.12G229400.3	96.49122807	228	7	50	2106	857	2904	0	2444

TRINITY_DN16190_c0_g3_i5	Glyma.12G229400.1	100	228	7	50	2106	857	2904	0	2444

TRINITY_DN16190_c0_g3_i5	Glyma.12G229400.2	100	228	7	50	2106	857	2904	0	2444

TRINITY_DN14645_c0_g1_i3	Glyma.15G165100.1	96.88385269	218	10	49	2090	2075	16	0	2481

TRINITY_DN15403_c0_g2_i4	Glyma.20G212100.1	100	216	25	815	2838	1	2048	0	2298

TRINITY_DN19139_c0_g1_i1	Glyma.07G267500.1	97.01492537	188	18	58	2079	1	2058	0	2505

TRINITY_DN5582_c0_g4_i1	Glyma.09G168000.2	95.86206897	149	10	47	2081	58	2116	0	2811

TRINITY_DN5582_c0_g4_i1	Glyma.09G168000.1	100	149	10	47	2081	58	2116	0	2811

TRINITY_DN20293_c0_g1_i1	Glyma.02G310900.1	98.26839827	136	3	64	2123	1	2054	0	3013

TRINITY_DN8905_c0_g1_i2	Glyma.11G079200.1	100	445	38	122	2159	18	2049	0	1027

TRINITY_DN10172_c0_g3_i1	Glyma.01G091100.1	98.61111111	369	27	148	2193	2037	1	0	1531

TRINITY_DN15200_c0_g1_i4	Glyma.16G205100.1	100	297	17	256	2294	2051	1	0	1977

TRINITY_DN16138_c1_g1_i18	Glyma.08G077100.4	96.90444145	207	6	300	2345	1	2057	0	2558

TRINITY_DN16138_c1_g1_i5	Glyma.08G077100.4	96.20253165	207	6	566	2611	1	2057	0	2558

TRINITY_DN16138_c1_g1_i20	Glyma.08G077100.4	96.96969697	207	7	300	2333	1	2057	0	2481

TRINITY_DN16138_c1_g1_i7	Glyma.08G077100.4	98.20224719	207	7	566	2599	1	2057	0	2481

TRINITY_DN16138_c1_g1_i8	Glyma.08G077100.4	100	207	7	300	2333	1	2057	0	2481

TRINITY_DN16138_c1_g1_i9	Glyma.08G077100.4	100	207	7	285	2318	1	2057	0	2481

TRINITY_DN16415_c1_g1_i15	Glyma.U018900.1	96.55172414	180	5	1692	3750	2066	17	0	2747

TRINITY_DN16415_c1_g1_i4	Glyma.U018900.1	100	180	5	1796	3854	2066	17	0	2747

TRINITY_DN10172_c0_g3_i1	Glyma.10G205600.1	100	172	7	148	2193	2055	1	0	2739

TRINITY_DN14389_c1_g2_i1	Glyma.01G103500.2	100	169	2	312	2367	24	2079	0	2828

TRINITY_DN14389_c1_g2_i1	Glyma.01G103500.1	96	169	2	312	2367	24	2079	0	2828

TRINITY_DN3079_c0_g1_i1	Glyma.19G001500.3	95.74468085	166	13	554	2603	566	2605	0	2702

TRINITY_DN3079_c0_g2_i1	Glyma.19G001500.3	96.34369287	166	13	554	2603	566	2605	0	2702

TRINITY_DN7530_c0_g1_i1	Glyma.03G219200.2	98.92473118	150	0	39	2097	1	2059	0	2972

TRINITY_DN7530_c0_g1_i1	Glyma.03G219200.3	98.92473118	150	0	39	2097	1	2059	0	2972

TRINITY_DN15944_c2_g2_i7	Glyma.14G164600.3	96.52777778	146	13	269	2309	2056	1	0	2857

TRINITY_DN15944_c2_g2_i7	Glyma.14G164600.2	99.6969697	146	13	269	2309	2056	1	0	2857

TRINITY_DN12916_c0_g1_i1	Glyma.17G158300.2	96.43564356	145	33	312	2306	1	2031	0	2405

TRINITY_DN6272_c0_g1_i1	Glyma.14G007700.1	96.46017699	127	17	1	2041	2082	31	0	2937

TRINITY_DN36415_c0_g1_i1	Glyma.20G201400.1	97.1875	115	3	1	2053	528	2586	0	3127

TRINITY_DN14750_c0_g1_i2	Glyma.15G058700.2	95.18716578	109	8	913	2964	2049	1	0	3088

TRINITY_DN7791_c0_g1_i1	Glyma.17G090900.1	95.64393939	321	27	28	2067	1	2031	0	1731

TRINITY_DN14245_c0_g2_i1	Glyma.13G241400.4	95.65217391	314	35	1269	3295	1	2033	0	1700

TRINITY_DN14245_c0_g2_i3	Glyma.13G241400.4	97.32142857	314	35	1214	3240	1	2033	0	1700

TRINITY_DN14245_c0_g2_i2	Glyma.13G241400.4	95.65217391	313	35	529	2555	1	2033	0	1705

TRINITY_DN15200_c0_g1_i3	Glyma.16G205100.1	98.7654321	261	15	256	2294	2051	1	0	2187

TRINITY_DN16138_c1_g1_i10	Glyma.08G077100.4	100	207	6	285	2329	1	2056	0	2556

TRINITY_DN16138_c1_g1_i10	Glyma.08G077100.1	95.15151515	207	6	285	2329	1	2056	0	2556

TRINITY_DN16138_c1_g1_i2	Glyma.08G077100.4	96.34146341	207	7	300	2332	1	2056	0	2479

TRINITY_DN16138_c1_g1_i2	Glyma.08G077100.1	100	207	7	300	2332	1	2056	0	2479

TRINITY_DN16138_c1_g1_i3	Glyma.08G077100.4	100	207	7	566	2598	1	2056	0	2479

TRINITY_DN16138_c1_g1_i3	Glyma.08G077100.1	96.42857143	207	7	566	2598	1	2056	0	2479

TRINITY_DN16138_c1_g1_i11	Glyma.08G077100.4	95.19774011	194	3	311	2364	1	2057	0	2693

TRINITY_DN14389_c1_g2_i1	Glyma.03G070500.1	96.38554217	186	5	313	2367	25	2076	0	2712

TRINITY_DN12719_c0_g2_i2	Glyma.13G321300.38	100	155	20	458	2504	2008	1	0	2547

TRINITY_DN12719_c0_g2_i2	Glyma.13G321300.39	96.30769231	155	20	458	2504	2008	1	0	2547

TRINITY_DN12719_c0_g2_i2	Glyma.13G321300.26	96	155	20	458	2504	2008	1	0	2547

TRINITY_DN12719_c0_g2_i2	Glyma.13G321300.27	97.03703704	155	20	458	2504	2008	1	0	2547

TRINITY_DN12719_c0_g2_i2	Glyma.13G321300.7	95.45454545	155	20	458	2504	2008	1	0	2547

TRINITY_DN12719_c0_g2_i2	Glyma.13G321300.6	98.8372093	155	20	458	2504	2008	1	0	2547

TRINITY_DN12719_c0_g2_i2	Glyma.13G321300.40	95.75757576	155	20	458	2504	2008	1	0	2547

TRINITY_DN12719_c0_g2_i2	Glyma.13G321300.41	100	155	20	458	2504	2008	1	0	2547

TRINITY_DN12719_c0_g2_i2	Glyma.13G321300.28	98.63013699	155	20	458	2504	2008	1	0	2547

TRINITY_DN12719_c0_g2_i2	Glyma.13G321300.29	100	155	20	458	2504	2008	1	0	2547

TRINITY_DN12719_c0_g2_i2	Glyma.13G321300.9	100	155	20	458	2504	2008	1	0	2547

TRINITY_DN12719_c0_g2_i2	Glyma.13G321300.8	100	155	20	458	2504	2008	1	0	2547

TRINITY_DN12719_c0_g2_i4	Glyma.13G321300.38	96.80851064	155	20	458	2504	2008	1	0	2547

TRINITY_DN12719_c0_g2_i4	Glyma.13G321300.39	100	155	20	458	2504	2008	1	0	2547

TRINITY_DN12719_c0_g2_i4	Glyma.13G321300.26	95.3271028	155	20	458	2504	2008	1	0	2547

TRINITY_DN12719_c0_g2_i4	Glyma.13G321300.27	100	155	20	458	2504	2008	1	0	2547

TRINITY_DN12719_c0_g2_i4	Glyma.13G321300.7	95.99109131	155	20	458	2504	2008	1	0	2547

TRINITY_DN12719_c0_g2_i4	Glyma.13G321300.6	98.64864865	155	20	458	2504	2008	1	0	2547

TRINITY_DN12719_c0_g2_i4	Glyma.13G321300.40	98.8372093	155	20	458	2504	2008	1	0	2547

TRINITY_DN12719_c0_g2_i4	Glyma.13G321300.41	98.8372093	155	20	458	2504	2008	1	0	2547

TRINITY_DN12719_c0_g2_i4	Glyma.13G321300.28	100	155	20	458	2504	2008	1	0	2547

TRINITY_DN12719_c0_g2_i4	Glyma.13G321300.29	100	155	20	458	2504	2008	1	0	2547

TRINITY_DN12719_c0_g2_i4	Glyma.13G321300.9	100	155	20	458	2504	2008	1	0	2547

TRINITY_DN12719_c0_g2_i4	Glyma.13G321300.8	96.22641509	155	20	458	2504	2008	1	0	2547

TRINITY_DN4333_c0_g2_i1	Glyma.02G111500.4	100	141	6	411	2465	2049	1	0	2942

TRINITY_DN4333_c0_g2_i1	Glyma.02G111500.5	98.33333333	141	6	411	2465	2049	1	0	2942

TRINITY_DN11456_c0_g1_i1	Glyma.09G075400.1	99.10313901	121	0	285	2342	2058	1	0	3131

TRINITY_DN16190_c0_g3_i5	Glyma.06G277500.1	100	418	25	58	2106	871	2901	0	1264

TRINITY_DN16138_c1_g1_i17	Glyma.08G183400.7	95.72649573	340	14	311	2350	1	2049	0	1753

TRINITY_DN16138_c1_g1_i17	Glyma.08G183400.8	97.4025974	340	14	311	2350	1	2049	0	1753

TRINITY_DN16138_c1_g1_i17	Glyma.08G183400.9	98.88888889	340	14	311	2350	1	2049	0	1753

TRINITY_DN16138_c1_g1_i10	Glyma.15G049300.1	97.09090909	278	14	285	2328	1	2049	0	2122

TRINITY_DN16251_c0_g3_i8	Glyma.09G060600.1	97.38219895	233	12	357	2405	2523	472	0	2423

TRINITY_DN16251_c0_g3_i8	Glyma.09G060600.4	97.38219895	233	12	357	2405	2508	457	0	2423

TRINITY_DN16251_c0_g3_i8	Glyma.09G060600.3	98.97959184	232	13	357	2405	2502	472	0	2294

TRINITY_DN16251_c0_g3_i8	Glyma.09G060600.2	99.6031746	232	13	357	2405	2487	457	0	2294

TRINITY_DN16251_c0_g3_i8	Glyma.09G060600.5	97.38219895	231	14	357	2405	2520	472	0	2416

TRINITY_DN15980_c0_g2_i1	Glyma.02G004200.1	95.34883721	230	22	764	2793	1716	3750	0	2207

TRINITY_DN15980_c0_g2_i1	Glyma.02G004200.2	96.25	230	22	764	2793	1698	3732	0	2207

TRINITY_DN15329_c0_g2_i5	Glyma.02G085000.4	96.36363636	206	9	512	2566	2034	1	0	2495

TRINITY_DN11749_c0_g1_i1	Glyma.06G002400.1	97.65258216	159	3	1	2057	299	2335	0	2789

TRINITY_DN11749_c0_g1_i2	Glyma.06G002400.1	100	159	3	1	2057	299	2335	0	2789

TRINITY_DN16138_c1_g1_i22	Glyma.08G077100.4	97.36842105	149	1	311	2364	1	2057	0	2953

TRINITY_DN16138_c1_g1_i19	Glyma.08G077100.4	100	149	2	311	2352	1	2057	0	2876

TRINITY_DN16138_c1_g1_i21	Glyma.08G077100.4	96.80851064	149	2	311	2352	1	2057	0	2876

TRINITY_DN5316_c0_g1_i1	Glyma.05G013900.2	98.48484848	133	3	149	2199	2057	1	0	3024

TRINITY_DN8905_c0_g1_i2	Glyma.17G259200.1	100	120	4	117	2165	13	2069	0	3083

TRINITY_DN16138_c1_g1_i2	Glyma.15G049300.1	95.65217391	274	19	300	2331	1	2049	0	2078

TRINITY_DN16138_c1_g1_i3	Glyma.15G049300.1	100	274	19	566	2597	1	2049	0	2078

TRINITY_DN16345_c0_g1_i2	Glyma.08G044700.4	95.34050179	258	4	446	2496	3594	1540	0	2329

TRINITY_DN16345_c0_g1_i5	Glyma.08G044700.4	98.46153846	258	4	446	2496	3594	1540	0	2329

TRINITY_DN16345_c0_g1_i9	Glyma.08G044700.4	99.20948617	258	4	445	2495	3594	1540	0	2329

TRINITY_DN16345_c0_g1_i2	Glyma.08G044700.2	95.3271028	258	4	446	2496	3399	1345	0	2329

TRINITY_DN16345_c0_g1_i5	Glyma.08G044700.2	98.4375	258	4	446	2496	3399	1345	0	2329

TRINITY_DN16345_c0_g1_i9	Glyma.08G044700.2	99.23076923	258	4	445	2495	3399	1345	0	2329

TRINITY_DN17873_c0_g1_i1	Glyma.14G088600.2	98.9010989	179	6	1	2047	1397	3431	0	2612

TRINITY_DN17873_c0_g1_i1	Glyma.14G088600.1	98.01324503	179	6	1	2047	1397	3431	0	2612

TRINITY_DN17873_c0_g1_i1	Glyma.14G088600.3	95.29411765	179	6	1	2047	1397	3431	0	2612

TRINITY_DN10172_c0_g3_i1	Glyma.20G184900.1	98.61111111	178	7	148	2193	2049	1	0	2700

TRINITY_DN11696_c0_g2_i1	Glyma.17G187400.2	98.15602837	178	15	115	2153	1	2046	0	2636

TRINITY_DN8174_c0_g2_i1	Glyma.08G100300.1	98.67549669	156	3	662	2713	2055	1	0	2900

TRINITY_DN12659_c0_g1_i1	Glyma.U025600.15	98.4962406	151	8	164	2198	1	2052	0	2798

TRINITY_DN16138_c1_g1_i17	Glyma.08G077100.4	95.23809524	149	2	311	2351	1	2056	0	2874

TRINITY_DN16138_c1_g1_i17	Glyma.08G077100.1	100	149	2	311	2351	1	2056	0	2874

TRINITY_DN12049_c0_g1_i1	Glyma.08G091900.1	100	127	5	122	2173	124	2172	0	3022

TRINITY_DN36415_c0_g1_i1	Glyma.10G189400.1	100	125	1	1	2053	528	2583	0	3085

TRINITY_DN16356_c0_g1_i1	Glyma.08G025900.1	100	119	9	72	2115	78	2127	0	3022

TRINITY_DN16360_c0_g8_i1	Glyma.04G028600.1	100	470	34	240	2278	79	2111	0	946

TRINITY_DN13198_c0_g1_i1	Glyma.10G058200.1	97.78761062	424	28	170	2206	120	2153	0	1194

TRINITY_DN15980_c0_g2_i2	Glyma.02G004200.1	99.19354839	369	51	1993	4010	1761	3750	0	1092

TRINITY_DN15980_c0_g2_i2	Glyma.02G004200.2	95	369	51	1993	4010	1743	3732	0	1092

TRINITY_DN15510_c0_g1_i3	Glyma.16G218200.1	95.23809524	292	35	314	2342	1	2032	0	1860

TRINITY_DN15510_c0_g1_i6	Glyma.16G218200.1	100	292	35	288	2316	1	2032	0	1860

TRINITY_DN15451_c0_g1_i5	Glyma.19G181900.1	96.25	271	33	555	2593	2033	40	0	1796

TRINITY_DN15451_c0_g1_i6	Glyma.19G181900.1	100	271	33	555	2593	2033	40	0	1796

TRINITY_DN15095_c2_g2_i4	Glyma.03G202300.1	99.06542056	269	35	597	2597	1	2040	0	1858

TRINITY_DN15095_c2_g2_i4	Glyma.03G202300.2	95.65217391	269	35	597	2597	1	2040	0	1858

TRINITY_DN13544_c0_g1_i1	Glyma.02G120600.1	98.06451613	264	29	127	2160	2034	1	0	2061

TRINITY_DN15095_c2_g2_i4	Glyma.19G199500.1	100	263	33	597	2597	1	2043	0	1912

TRINITY_DN15264_c0_g1_i7	Glyma.10G137600.3	97.14285714	256	6	64	2107	1797	3849	0	2292

TRINITY_DN15264_c0_g1_i7	Glyma.10G137600.2	97.14285714	256	6	64	2107	1797	3849	0	2292

TRINITY_DN15264_c0_g1_i7	Glyma.10G137600.1	97.14285714	256	6	64	2107	1797	3849	0	2292

TRINITY_DN7308_c0_g1_i1	Glyma.15G272900.1	97.99196787	214	15	1225	3264	2019	1	0	2279

TRINITY_DN13448_c0_g1_i2	Glyma.05G144400.4	96.15384615	170	3	1	2054	27	2074	0	2802

TRINITY_DN13448_c0_g1_i2	Glyma.05G144400.3	96.15384615	170	3	1	2054	27	2074	0	2802

TRINITY_DN13448_c0_g1_i2	Glyma.05G144400.1	96.0443038	170	3	1	2054	27	2074	0	2802

TRINITY_DN15328_c0_g1_i1	Glyma.07G056700.4	98.79518072	161	7	19	2061	1357	3396	0	2728

TRINITY_DN15328_c0_g1_i3	Glyma.07G056700.4	98.4	161	7	19	2061	1357	3396	0	2728

TRINITY_DN7895_c0_g1_i1	Glyma.16G082400.1	100	159	4	1	2045	245	2298	0	2843

TRINITY_DN13709_c0_g1_i1	Glyma.01G019100.1	95.05494505	158	1	200	2248	2055	1	0	2881

TRINITY_DN15572_c0_g1_i1	Glyma.07G208300.1	96.52173913	153	27	277	2316	1	2007	0	2540

TRINITY_DN14746_c0_g2_i2	Glyma.18G286000.1	99.49494949	140	8	187	2225	44	2097	0	2909

TRINITY_DN7491_c0_g1_i1	Glyma.09G187100.1	98.47328244	109	0	233	2287	2079	25	0	3192

TRINITY_DN15329_c0_g2_i5	Glyma.05G238700.1	98.19277108	100	1	512	2566	2037	1	0	3125

TRINITY_DN8764_c0_g1_i1	Glyma.13G053800.1	98.8372093	322	26	310	2339	19	2060	0	1777

TRINITY_DN15264_c0_g1_i7	Glyma.02G008000.1	97.14285714	251	4	64	2107	1797	3849	0	2331

TRINITY_DN15135_c0_g1_i2	Glyma.13G156900.1	96.49122807	190	20	433	2475	2028	1	0	2501

TRINITY_DN16354_c0_g1_i3	Glyma.20G192700.1	95	182	1	823	2873	31	2084	0	2765

TRINITY_DN15200_c0_g1_i1	Glyma.09G154600.1	99.29577465	181	3	301	2352	2052	1	0	2765

TRINITY_DN13639_c0_g1_i2	Glyma.13G318900.1	95.76271186	145	13	318	2349	2085	46	0	2758

TRINITY_DN13639_c0_g1_i2	Glyma.13G318900.2	98.8372093	145	13	318	2349	2085	46	0	2758

TRINITY_DN14881_c0_g2_i1	Glyma.05G213300.1	95.10703364	143	5	438	2478	9	2052	0	2852

TRINITY_DN14881_c0_g2_i1	Glyma.05G213300.2	95.10869565	143	5	438	2478	9	2052	0	2852

TRINITY_DN14881_c0_g2_i1	Glyma.05G213300.3	95.10869565	143	5	438	2478	9	2052	0	2852

TRINITY_DN14881_c0_g2_i2	Glyma.05G213300.1	100	143	5	470	2510	9	2052	0	2852

TRINITY_DN14881_c0_g2_i2	Glyma.05G213300.2	95.95015576	143	5	470	2510	9	2052	0	2852

TRINITY_DN14881_c0_g2_i2	Glyma.05G213300.3	98.5915493	143	5	470	2510	9	2052	0	2852

TRINITY_DN14881_c0_g2_i3	Glyma.05G213300.1	96.9924812	143	5	1574	3614	9	2052	0	2852

TRINITY_DN14881_c0_g2_i3	Glyma.05G213300.2	99.45652174	143	5	1574	3614	9	2052	0	2852

TRINITY_DN14881_c0_g2_i3	Glyma.05G213300.3	98.4	143	5	1574	3614	9	2052	0	2852

TRINITY_DN8905_c0_g1_i2	Glyma.14G220300.1	100	127	3	117	2165	13	2066	0	3057

TRINITY_DN16360_c0_g8_i3	Glyma.17G254100.1	99.36305732	448	34	274	2307	78	2108	0	1044

TRINITY_DN16360_c0_g8_i6	Glyma.17G254100.1	100	448	34	274	2307	78	2108	0	1044

TRINITY_DN16138_c1_g1_i10	Glyma.08G183400.7	99.21259843	299	7	285	2328	1	2049	0	2050

TRINITY_DN16138_c1_g1_i10	Glyma.08G183400.8	99.21414538	299	7	285	2328	1	2049	0	2050

TRINITY_DN16138_c1_g1_i10	Glyma.08G183400.9	99.21875	299	7	285	2328	1	2049	0	2050

TRINITY_DN16138_c1_g1_i2	Glyma.08G183400.7	95.91836735	296	11	300	2331	1	2049	0	1989

TRINITY_DN16138_c1_g1_i2	Glyma.08G183400.8	98.46153846	296	11	300	2331	1	2049	0	1989

TRINITY_DN16138_c1_g1_i2	Glyma.08G183400.9	97.5	296	11	300	2331	1	2049	0	1989

TRINITY_DN16138_c1_g1_i3	Glyma.08G183400.7	96.92307692	296	11	566	2597	1	2049	0	1989

TRINITY_DN16138_c1_g1_i3	Glyma.08G183400.8	97.46835443	296	11	566	2597	1	2049	0	1989

TRINITY_DN16138_c1_g1_i3	Glyma.08G183400.9	100	296	11	566	2597	1	2049	0	1989

TRINITY_DN15239_c0_g1_i3	Glyma.13G089200.1	96.47058824	293	11	245	2290	1	2046	0	2078

TRINITY_DN15239_c0_g1_i1	Glyma.13G089200.1	100	292	11	245	2290	1	2046	0	2084

TRINITY_DN15510_c0_g1_i3	Glyma.09G168600.3	98.02631579	285	29	314	2342	1	2032	0	1921

TRINITY_DN15510_c0_g1_i3	Glyma.09G168600.4	95.83333333	285	29	314	2342	1	2032	0	1921

TRINITY_DN15510_c0_g1_i3	Glyma.09G168600.1	100	285	29	314	2342	1	2032	0	1921

TRINITY_DN15510_c0_g1_i6	Glyma.09G168600.3	98.48484848	285	29	288	2316	1	2032	0	1921

TRINITY_DN15510_c0_g1_i6	Glyma.09G168600.4	98.38709677	285	29	288	2316	1	2032	0	1921

TRINITY_DN15510_c0_g1_i6	Glyma.09G168600.1	97.44186047	285	29	288	2316	1	2032	0	1921

TRINITY_DN15239_c0_g1_i3	Glyma.14G174200.1	100	284	13	245	2290	1	2046	0	2128

TRINITY_DN15239_c0_g1_i1	Glyma.14G174200.1	99.3006993	283	13	245	2290	1	2046	0	2134

TRINITY_DN13971_c0_g1_i1	Glyma.04G088100.1	98.26086957	192	24	1167	3185	2037	1	0	2405

TRINITY_DN13971_c0_g1_i1	Glyma.04G088100.2	98.26086957	192	24	1167	3185	2037	1	0	2405

TRINITY_DN13971_c0_g1_i1	Glyma.04G088100.3	98.26086957	192	24	1167	3185	2037	1	0	2405

TRINITY_DN13971_c0_g1_i1	Glyma.04G088100.4	95.36423841	192	24	1167	3185	2037	1	0	2405

TRINITY_DN13971_c0_g1_i2	Glyma.04G088100.1	97.87234043	192	24	228	2246	2037	1	0	2405

TRINITY_DN13971_c0_g1_i2	Glyma.04G088100.2	97.87234043	192	24	228	2246	2037	1	0	2405

TRINITY_DN13971_c0_g1_i2	Glyma.04G088100.3	97.43589744	192	24	228	2246	2037	1	0	2405

TRINITY_DN13971_c0_g1_i2	Glyma.04G088100.4	96.62162162	192	24	228	2246	2037	1	0	2405

TRINITY_DN13971_c0_g1_i3	Glyma.04G088100.1	100	192	24	1331	3349	2037	1	0	2405

TRINITY_DN13971_c0_g1_i3	Glyma.04G088100.2	100	192	24	1331	3349	2037	1	0	2405

TRINITY_DN13971_c0_g1_i3	Glyma.04G088100.3	98.48484848	192	24	1331	3349	2037	1	0	2405

TRINITY_DN13971_c0_g1_i3	Glyma.04G088100.4	98.50746269	192	24	1331	3349	2037	1	0	2405

TRINITY_DN16363_c0_g3_i1	Glyma.16G222000.1	96.63865546	186	29	95	2139	73	2096	0	2521

TRINITY_DN14643_c0_g1_i1	Glyma.08G188800.1	96.80851064	180	7	114	2164	16	2063	0	2748

TRINITY_DN15264_c0_g1_i1	Glyma.10G137600.3	95.16129032	178	2	64	2107	1797	3849	0	2747

TRINITY_DN15264_c0_g1_i1	Glyma.10G137600.2	95.16129032	178	2	64	2107	1797	3849	0	2747

TRINITY_DN15264_c0_g1_i1	Glyma.10G137600.1	95.16129032	178	2	64	2107	1797	3849	0	2747

TRINITY_DN15264_c0_g1_i1	Glyma.02G008000.1	100	169	2	64	2107	1797	3849	0	2796

TRINITY_DN11875_c0_g2_i1	Glyma.08G105200.1	95.30201342	168	5	1129	3175	854	2897	0	2763

TRINITY_DN36182_c0_g1_i1	Glyma.04G007400.1	96.82539683	164	9	361	2402	2121	71	0	2798

TRINITY_DN14836_c0_g1_i9	Glyma.13G034600.1	97.54689755	162	23	2557	4598	3984	5997	0	2571

TRINITY_DN14836_c0_g1_i9	Glyma.14G155900.1	95	162	23	2557	4598	3897	5910	0	2571

TRINITY_DN11259_c0_g1_i3	Glyma.08G187600.1	96	149	3	101	2148	453	2505	0	2933

TRINITY_DN14979_c0_g1_i5	Glyma.14G223400.1	96.49122807	133	3	679	2716	2053	1	0	2957

TRINITY_DN16360_c0_g8_i3	Glyma.14G070600.1	99.34210526	450	33	274	2307	78	2108	0	1044

TRINITY_DN16360_c0_g8_i6	Glyma.14G070600.1	100	450	33	274	2307	78	2108	0	1044

TRINITY_DN16354_c0_g1_i3	Glyma.08G027000.1	95	328	7	826	2871	1	2049	0	1914

TRINITY_DN39405_c0_g1_i1	Glyma.16G203300.2	95.53956835	316	8	309	2352	2877	828	0	1975

TRINITY_DN13654_c0_g2_i1	Glyma.19G261700.3	100	282	25	461	2482	2067	43	0	1858

TRINITY_DN13654_c0_g2_i1	Glyma.19G261700.2	98.4375	282	25	461	2482	2067	43	0	1858

TRINITY_DN15853_c0_g1_i12	Glyma.02G010500.3	98.7654321	254	4	823	2874	2046	1	0	2344

TRINITY_DN15239_c0_g1_i4	Glyma.13G089200.1	100	231	9	245	2290	1	2046	0	2433

TRINITY_DN15239_c0_g1_i5	Glyma.13G089200.1	100	230	9	245	2290	1	2046	0	2438

TRINITY_DN15239_c0_g1_i4	Glyma.14G174200.1	100	220	11	245	2290	1	2046	0	2494

TRINITY_DN15239_c0_g1_i5	Glyma.14G174200.1	99.21875	219	11	245	2290	1	2046	0	2499

TRINITY_DN16164_c1_g1_i8	Glyma.13G037600.1	99.66329966	216	24	44	2048	744	2787	0	2237

TRINITY_DN16164_c1_g1_i5	Glyma.13G037600.1	100	212	24	44	2048	744	2787	0	2259

TRINITY_DN2189_c0_g2_i1	Glyma.09G028600.1	97.33727811	176	6	131	2177	56	2099	0	2730

TRINITY_DN15337_c0_g2_i2	Glyma.11G219500.1	100	171	10	1309	3348	1	2040	0	2687

TRINITY_DN15647_c0_g1_i4	Glyma.07G088500.2	100	169	4	513	2558	2046	1	0	2776

TRINITY_DN15647_c0_g1_i4	Glyma.07G088500.1	96.92307692	169	4	513	2558	2046	1	0	2776

TRINITY_DN15200_c0_g1_i1	Glyma.16G205100.1	100	165	5	305	2352	2051	1	0	2843

TRINITY_DN15572_c0_g1_i1	Glyma.07G208300.3	98.13907933	153	26	277	2316	1	2004	0	2555

TRINITY_DN15572_c0_g1_i1	Glyma.07G208300.2	96.52351738	153	28	277	2316	1	2001	0	2534

TRINITY_DN8174_c0_g2_i1	Glyma.05G144000.1	100	149	0	662	2713	2052	1	0	2964

TRINITY_DN10039_c0_g1_i1	Glyma.10G103000.1	96.18768328	123	16	80	2107	1	2043	0	2894

TRINITY_DN10529_c0_g2_i1	Glyma.08G185700.2	99.73544974	118	0	326	2377	1	2052	0	3136

TRINITY_DN15778_c0_g1_i1	Glyma.03G076800.2	100	117	0	998	3049	2052	1	0	3142

TRINITY_DN15778_c0_g1_i1	Glyma.03G076800.1	97.12230216	117	0	998	3049	2052	1	0	3142

TRINITY_DN15778_c0_g1_i1	Glyma.03G076800.5	97.12230216	117	0	998	3049	2052	1	0	3142

TRINITY_DN15778_c0_g1_i1	Glyma.01G111200.2	97.84482759	110	0	998	3049	2052	1	0	3181

TRINITY_DN15778_c0_g1_i1	Glyma.01G111200.1	97.84482759	110	0	998	3049	2052	1	0	3181

TRINITY_DN15778_c0_g1_i1	Glyma.01G111200.3	97.73584906	110	0	998	3049	2052	1	0	3181

TRINITY_DN15239_c0_g1_i1	Glyma.13G089200.2	100	292	11	245	2288	1	2044	0	2080

TRINITY_DN15239_c0_g1_i1	Glyma.14G174200.2	100	283	13	245	2288	1	2044	0	2130

TRINITY_DN16190_c0_g3_i1	Glyma.12G229400.3	98.99497487	260	9	50	2094	857	2904	0	2289

TRINITY_DN16190_c0_g3_i1	Glyma.12G229400.1	98.79518072	260	9	50	2094	857	2904	0	2289

TRINITY_DN16190_c0_g3_i1	Glyma.12G229400.2	98.24561404	260	9	50	2094	857	2904	0	2289

TRINITY_DN16076_c2_g3_i1	Glyma.05G082400.1	100	239	3	352	2401	2064	18	0	2431

TRINITY_DN16076_c2_g3_i1	Glyma.05G082400.2	95.25862069	239	3	352	2401	2064	18	0	2431

TRINITY_DN16076_c2_g3_i2	Glyma.05G082400.1	98.52459016	239	3	352	2401	2064	18	0	2431

TRINITY_DN16076_c2_g3_i2	Glyma.05G082400.2	98.9010989	239	3	352	2401	2064	18	0	2431

TRINITY_DN15877_c0_g1_i2	Glyma.14G047800.1	99.09090909	234	18	47	2079	2364	323	0	2316

TRINITY_DN15129_c0_g1_i1	Glyma.15G150700.1	100	195	17	381	2426	2025	1	0	2507

TRINITY_DN16354_c0_g1_i3	Glyma.20G192700.4	95	181	1	826	2873	1	2051	0	2765

TRINITY_DN16354_c0_g1_i3	Glyma.20G192700.5	95	181	1	826	2873	1	2051	0	2765

TRINITY_DN16354_c0_g1_i3	Glyma.20G192700.3	97.72727273	181	1	826	2873	1	2051	0	2765

TRINITY_DN16354_c0_g1_i3	Glyma.20G192700.2	97.72727273	181	1	826	2873	1	2051	0	2765

TRINITY_DN13140_c0_g1_i2	Glyma.07G195700.1	98.38709677	164	8	1	2036	698	2745	0	2763

TRINITY_DN15328_c0_g1_i1	Glyma.07G056700.2	98.38709677	161	7	19	2057	1357	3392	0	2721

TRINITY_DN15328_c0_g1_i3	Glyma.07G056700.2	100	161	7	19	2057	1357	3392	0	2721

TRINITY_DN5582_c0_g4_i1	Glyma.16G217800.2	100	159	9	47	2081	58	2104	0	2778

TRINITY_DN5582_c0_g4_i1	Glyma.16G217800.1	100	159	9	47	2081	58	2104	0	2778

TRINITY_DN15970_c0_g2_i10	Glyma.03G069400.1	99.38271605	155	3	80	2124	1	2051	0	2891

TRINITY_DN15970_c0_g2_i4	Glyma.03G069400.1	97.48283753	155	3	80	2124	1	2051	0	2891

TRINITY_DN12976_c0_g1_i2	Glyma.03G245900.1	99.06542056	142	13	55	2087	1	2045	0	2846

TRINITY_DN2636_c0_g2_i1	Glyma.01G032500.1	95.9798995	136	2	256	2305	2148	99	0	3022

TRINITY_DN16015_c0_g3_i3	Glyma.03G137900.1	100	136	4	254	2300	1	2050	0	3001

TRINITY_DN2367_c0_g1_i1	Glyma.14G005200.1	95	119	6	477	2516	2054	6	0	3044

TRINITY_DN15969_c0_g5_i1	Glyma.07G065500.3	97	364	33	120	2146	63	2062	0	1297

TRINITY_DN15969_c0_g5_i1	Glyma.07G065500.2	95.22357724	364	33	120	2146	63	2062	0	1297

TRINITY_DN15969_c0_g5_i1	Glyma.07G065500.1	100	364	33	120	2146	63	2062	0	1297

TRINITY_DN15048_c0_g2_i2	Glyma.17G030600.1	98.52216749	313	32	584	2611	2025	1	0	1748

TRINITY_DN10834_c0_g1_i1	Glyma.06G315100.2	100	302	37	1	2032	993	3006	0	1764

TRINITY_DN16388_c0_g2_i4	Glyma.13G360700.1	100	299	30	398	2413	428	2452	0	1748

TRINITY_DN15853_c0_g1_i12	Glyma.02G010500.1	98.7654321	254	4	825	2874	2044	1	0	2340

TRINITY_DN15853_c0_g1_i3	Glyma.02G010500.3	95.42253521	254	4	488	2537	2044	1	0	2340

TRINITY_DN16190_c0_g3_i5	Glyma.13G270400.4	96.76470588	235	6	58	2106	868	2904	0	2394

TRINITY_DN16190_c0_g3_i5	Glyma.13G270400.2	95.62043796	235	6	58	2106	868	2904	0	2394

TRINITY_DN16190_c0_g3_i5	Glyma.13G270400.3	100	235	6	58	2106	868	2904	0	2394

TRINITY_DN16190_c0_g3_i5	Glyma.13G270400.1	99.12280702	235	6	58	2106	868	2904	0	2394

TRINITY_DN16190_c0_g3_i5	Glyma.13G270400.5	96.31578947	235	6	58	2106	868	2904	0	2394

TRINITY_DN15239_c0_g1_i5	Glyma.13G089200.2	100	230	9	245	2288	1	2044	0	2435

TRINITY_DN15239_c0_g1_i5	Glyma.14G174200.2	96.875	219	11	245	2288	1	2044	0	2495

TRINITY_DN15129_c0_g1_i1	Glyma.09G042300.1	98.64864865	203	16	381	2426	2022	1	0	2455

TRINITY_DN16436_c0_g2_i5	Glyma.11G131600.1	97.01492537	184	5	3	2038	202	2249	0	2663

TRINITY_DN16436_c0_g2_i5	Glyma.11G131600.3	97.26027397	184	5	3	2038	58	2105	0	2663

TRINITY_DN16190_c0_g3_i5	Glyma.06G229900.1	100	165	6	58	2106	868	2904	0	2782

TRINITY_DN16190_c0_g3_i5	Glyma.12G161300.1	98.59649123	164	4	58	2106	868	2904	0	2787

TRINITY_DN14937_c0_g1_i1	Glyma.18G266600.1	95.58359621	163	4	197	2245	2043	1	0	2832

TRINITY_DN13095_c0_g1_i3	Glyma.17G063000.2	96.15384615	154	4	720	2759	2049	1	0	2861

TRINITY_DN15841_c0_g1_i1	Glyma.U033300.1	96.80851064	144	4	569	2618	273	2293	0	2800

TRINITY_DN15841_c0_g1_i2	Glyma.U033300.1	97.72727273	144	4	569	2618	273	2293	0	2800

TRINITY_DN11366_c0_g1_i1	Glyma.07G195900.2	100	127	5	99	2138	49	2097	0	3011

TRINITY_DN15853_c0_g1_i12	Glyma.19G218200.1	98.64864865	118	2	825	2874	2041	1	0	3073

TRINITY_DN15853_c0_g1_i12	Glyma.03G221200.1	100	112	4	825	2874	2038	1	0	3088

TRINITY_DN12519_c0_g1_i2	Glyma.20G017000.5	96.92832765	102	3	161	2204	1	2050	0	3182

TRINITY_DN12519_c0_g1_i1	Glyma.20G017000.5	97.6076555	101	4	161	2188	1	2050	0	3085

TRINITY_DN15048_c0_g2_i2	Glyma.07G243300.1	100	312	26	584	2611	2031	1	0	1803

TRINITY_DN13116_c0_g1_i1	Glyma.13G109600.17	97.85002622	307	26	677	2715	2021	1	0	1838

TRINITY_DN13116_c0_g1_i1	Glyma.13G109600.19	97.87234043	307	26	677	2715	2021	1	0	1838

TRINITY_DN13116_c0_g1_i2	Glyma.13G109600.17	97.85714286	307	26	439	2477	2021	1	0	1838

TRINITY_DN13116_c0_g1_i2	Glyma.13G109600.19	97.85407725	307	26	439	2477	2021	1	0	1838

TRINITY_DN16076_c1_g1_i1	Glyma.01G183300.1	98.61111111	303	26	555	2576	439	2469	0	1814

TRINITY_DN16396_c2_g1_i3	Glyma.12G062000.1	97.26027397	271	46	246	2252	40	2043	0	1720

TRINITY_DN9151_c0_g1_i1	Glyma.07G013500.2	100	210	10	187	2227	3	2040	0	2497

TRINITY_DN9151_c0_g1_i1	Glyma.07G013500.1	100	210	10	187	2227	3	2040	0	2497

TRINITY_DN9151_c0_g2_i1	Glyma.07G013500.2	97.32142857	210	10	187	2227	3	2040	0	2497

TRINITY_DN9151_c0_g2_i1	Glyma.07G013500.1	98.01980198	210	10	187	2227	3	2040	0	2497

TRINITY_DN16836_c0_g1_i1	Glyma.04G185000.1	95.14563107	204	3	1	2048	1277	3321	0	2621

TRINITY_DN7308_c0_g1_i1	Glyma.08G153100.3	97.97979798	198	7	1222	3264	2031	1	0	2532

TRINITY_DN18475_c0_g1_i1	Glyma.U020000.1	97.05882353	189	6	19	2055	1	2046	0	2639

TRINITY_DN18475_c0_g1_i1	Glyma.U020000.2	95.12195122	189	6	19	2055	1	2046	0	2639

TRINITY_DN15239_c0_g1_i4	Glyma.06G103200.6	96.2962963	183	1	245	2290	1	2049	0	2750

TRINITY_DN15239_c0_g1_i4	Glyma.06G103200.2	96.31147541	183	1	245	2290	1	2049	0	2750

TRINITY_DN15239_c0_g1_i4	Glyma.06G103200.3	97.91666667	183	1	245	2290	1	2049	0	2750

TRINITY_DN15239_c0_g1_i4	Glyma.06G103200.1	98.41269841	183	1	245	2290	1	2049	0	2750

TRINITY_DN15239_c0_g1_i4	Glyma.06G103200.4	98.41269841	183	1	245	2290	1	2049	0	2750

TRINITY_DN15239_c0_g1_i4	Glyma.06G103200.5	95.77464789	183	1	245	2290	1	2049	0	2750

TRINITY_DN15239_c0_g1_i5	Glyma.06G103200.6	100	182	1	245	2290	1	2049	0	2756

TRINITY_DN15239_c0_g1_i5	Glyma.06G103200.2	100	182	1	245	2290	1	2049	0	2756

TRINITY_DN15239_c0_g1_i5	Glyma.06G103200.3	100	182	1	245	2290	1	2049	0	2756

TRINITY_DN15239_c0_g1_i5	Glyma.06G103200.1	100	182	1	245	2290	1	2049	0	2756

TRINITY_DN15239_c0_g1_i5	Glyma.06G103200.4	100	182	1	245	2290	1	2049	0	2756

TRINITY_DN15239_c0_g1_i5	Glyma.06G103200.5	100	182	1	245	2290	1	2049	0	2756

TRINITY_DN2189_c0_g2_i1	Glyma.15G134500.1	97.42647059	175	6	131	2177	53	2093	0	2750

TRINITY_DN16164_c1_g1_i3	Glyma.13G037600.1	99.05660377	172	18	44	2054	744	2787	0	2553

TRINITY_DN15647_c0_g1_i4	Glyma.09G188300.3	95.75471698	170	3	513	2558	2043	1	0	2784

TRINITY_DN15647_c0_g1_i4	Glyma.09G188300.1	96.64634146	170	3	513	2558	2043	1	0	2784

TRINITY_DN15095_c2_g2_i1	Glyma.19G199500.1	97.01492537	169	11	597	2624	1	2043	0	2673

TRINITY_DN16164_c1_g1_i1	Glyma.13G037600.1	100	168	18	44	2054	744	2787	0	2575

TRINITY_DN15095_c2_g2_i1	Glyma.03G202300.1	100	165	12	597	2624	1	2040	0	2676

TRINITY_DN15095_c2_g2_i1	Glyma.03G202300.2	98.8372093	165	12	597	2624	1	2040	0	2676

TRINITY_DN15328_c0_g1_i1	Glyma.07G056700.3	98.80952381	161	7	19	2055	1357	3390	0	2717

TRINITY_DN15328_c0_g1_i1	Glyma.07G056700.1	98.80952381	161	7	19	2055	1357	3390	0	2717

TRINITY_DN15328_c0_g1_i2	Glyma.07G056700.4	98.4	161	7	19	2055	1357	3390	0	2717

TRINITY_DN15328_c0_g1_i3	Glyma.07G056700.3	98.40255591	161	7	19	2055	1357	3390	0	2717

TRINITY_DN15328_c0_g1_i3	Glyma.07G056700.1	100	161	7	19	2055	1357	3390	0	2717

TRINITY_DN15328_c0_g1_i4	Glyma.07G056700.4	100	161	7	19	2055	1357	3390	0	2717

TRINITY_DN13709_c0_g1_i1	Glyma.01G019100.2	98.33333333	158	0	200	2248	2049	1	0	2909

TRINITY_DN15239_c0_g1_i3	Glyma.06G103200.6	95.45454545	150	1	245	2290	1	2049	0	2933

TRINITY_DN15239_c0_g1_i3	Glyma.06G103200.2	95.45454545	150	1	245	2290	1	2049	0	2933

TRINITY_DN15239_c0_g1_i3	Glyma.06G103200.3	96.12068966	150	1	245	2290	1	2049	0	2933

TRINITY_DN15239_c0_g1_i3	Glyma.06G103200.1	96.12068966	150	1	245	2290	1	2049	0	2933

TRINITY_DN15239_c0_g1_i3	Glyma.06G103200.4	100	150	1	245	2290	1	2049	0	2933

TRINITY_DN15239_c0_g1_i3	Glyma.06G103200.5	100	150	1	245	2290	1	2049	0	2933

TRINITY_DN15239_c0_g1_i1	Glyma.06G103200.6	98.7654321	149	1	245	2290	1	2049	0	2939

TRINITY_DN15239_c0_g1_i1	Glyma.06G103200.2	100	149	1	245	2290	1	2049	0	2939

TRINITY_DN15239_c0_g1_i1	Glyma.06G103200.3	98.61386139	149	1	245	2290	1	2049	0	2939

TRINITY_DN15239_c0_g1_i1	Glyma.06G103200.1	98.61386139	149	1	245	2290	1	2049	0	2939

TRINITY_DN15239_c0_g1_i1	Glyma.06G103200.4	98.61386139	149	1	245	2290	1	2049	0	2939

TRINITY_DN15239_c0_g1_i1	Glyma.06G103200.5	98.61386139	149	1	245	2290	1	2049	0	2939

TRINITY_DN14937_c0_g1_i1	Glyma.08G245400.1	95.58823529	146	1	197	2245	2046	1	0	2955

TRINITY_DN14979_c0_g1_i5	Glyma.14G223400.4	96.47798742	134	2	671	2716	2049	1	0	3022

TRINITY_DN14979_c0_g1_i5	Glyma.14G223400.5	96.47058824	134	2	671	2716	2049	1	0	3022

TRINITY_DN15910_c0_g1_i1	Glyma.19G217300.2	100	119	3	248	2293	1	2049	0	3105

TRINITY_DN14337_c0_g2_i1	Glyma.13G156600.2	100	117	8	321	2348	2049	1	0	3000

TRINITY_DN14337_c0_g2_i3	Glyma.13G156600.2	100	117	8	443	2470	2049	1	0	3000

TRINITY_DN19642_c0_g1_i1	Glyma.12G094500.1	97.34513274	381	79	179	2164	1	1977	0	811

TRINITY_DN13471_c0_g2_i1	Glyma.07G053500.1	95.97315436	248	18	211	2237	2122	90	0	2176

TRINITY_DN6249_c0_g1_i2	Glyma.05G052700.1	100	225	13	87	2126	1	2031	0	2374

TRINITY_DN11630_c1_g1_i1	Glyma.05G152900.3	96.42521167	174	4	1155	3202	785	2811	0	2682

TRINITY_DN13654_c0_g1_i2	Glyma.19G261700.3	97.95918367	166	5	461	2504	2067	24	0	2811

TRINITY_DN13654_c0_g1_i2	Glyma.19G261700.2	100	166	5	461	2504	2067	24	0	2811

TRINITY_DN14645_c0_g1_i3	Glyma.17G059800.1	96.96969697	155	0	49	2096	2048	1	0	2924

TRINITY_DN15970_c0_g2_i10	Glyma.01G099800.1	98.61386139	151	2	80	2124	1	2048	0	2926

TRINITY_DN15970_c0_g2_i4	Glyma.01G099800.1	96.875	151	2	80	2124	1	2048	0	2926

TRINITY_DN14596_c0_g1_i1	Glyma.10G217200.3	98.68421053	145	4	1251	3294	142	2185	0	2928

TRINITY_DN14596_c0_g1_i2	Glyma.10G217200.3	98.95833333	145	4	1234	3277	142	2185	0	2928

TRINITY_DN14645_c0_g1_i3	Glyma.13G100100.5	96.07843137	141	0	49	2096	2048	1	0	3001

TRINITY_DN14645_c0_g1_i3	Glyma.13G100100.6	96.07843137	141	0	49	2096	2048	1	0	3001

TRINITY_DN14645_c0_g1_i3	Glyma.13G100100.1	96.47355164	141	0	49	2096	2048	1	0	3001

TRINITY_DN14645_c0_g1_i3	Glyma.13G100100.4	98.8372093	141	0	49	2096	2048	1	0	3001

TRINITY_DN14645_c0_g1_i3	Glyma.13G100100.2	98.8372093	141	0	49	2096	2048	1	0	3001

TRINITY_DN14645_c0_g1_i3	Glyma.13G100100.3	98.8372093	141	0	49	2096	2048	1	0	3001

TRINITY_DN14645_c0_g1_i3	Glyma.13G100100.7	98.8372093	141	0	49	2096	2048	1	0	3001

TRINITY_DN18597_c0_g1_i1	Glyma.08G161500.1	95.91836735	121	1	381	2425	3303	1256	0	3092

TRINITY_DN16360_c0_g8_i3	Glyma.04G028600.1	99.36305732	460	34	277	2307	87	2111	0	987

TRINITY_DN16360_c0_g8_i6	Glyma.04G028600.1	100	460	34	277	2307	87	2111	0	987

TRINITY_DN12997_c0_g1_i2	Glyma.13G032500.1	100	354	45	390	2385	2268	240	0	1373

TRINITY_DN16396_c2_g1_i5	Glyma.12G062000.1	97.23756906	276	39	246	2252	40	2043	0	1714

TRINITY_DN15264_c0_g1_i1	Glyma.03G224100.1	100	270	6	64	2107	1809	3852	0	2246

TRINITY_DN13971_c0_g1_i1	Glyma.06G090100.5	95.36423841	205	17	1167	3185	2031	1	0	2361

TRINITY_DN13971_c0_g1_i1	Glyma.06G090100.4	95.36423841	205	17	1167	3185	2031	1	0	2361

TRINITY_DN13971_c0_g1_i1	Glyma.06G090100.3	95.36423841	205	17	1167	3185	2031	1	0	2361

TRINITY_DN13971_c0_g1_i1	Glyma.06G090100.2	96.91358025	205	17	1167	3185	2031	1	0	2361

TRINITY_DN13971_c0_g1_i1	Glyma.06G090100.1	100	205	17	1167	3185	2031	1	0	2361

TRINITY_DN13971_c0_g1_i2	Glyma.06G090100.5	95.28936743	205	17	228	2246	2031	1	0	2361

TRINITY_DN13971_c0_g1_i2	Glyma.06G090100.4	95.12195122	205	17	228	2246	2031	1	0	2361

TRINITY_DN13971_c0_g1_i2	Glyma.06G090100.3	100	205	17	228	2246	2031	1	0	2361

TRINITY_DN13971_c0_g1_i2	Glyma.06G090100.2	96.82539683	205	17	228	2246	2031	1	0	2361

TRINITY_DN13971_c0_g1_i2	Glyma.06G090100.1	97.07602339	205	17	228	2246	2031	1	0	2361

TRINITY_DN13971_c0_g1_i3	Glyma.06G090100.5	98.57142857	205	17	1331	3349	2031	1	0	2361

TRINITY_DN13971_c0_g1_i3	Glyma.06G090100.4	98.34254144	205	17	1331	3349	2031	1	0	2361

TRINITY_DN13971_c0_g1_i3	Glyma.06G090100.3	96.96969697	205	17	1331	3349	2031	1	0	2361

TRINITY_DN13971_c0_g1_i3	Glyma.06G090100.2	96.6442953	205	17	1331	3349	2031	1	0	2361

TRINITY_DN13971_c0_g1_i3	Glyma.06G090100.1	99.1011236	205	17	1331	3349	2031	1	0	2361

TRINITY_DN16333_c0_g1_i3	Glyma.07G184300.1	97.82608696	119	6	191	2233	2034	1	0	3011

TRINITY_DN16333_c0_g1_i3	Glyma.08G065100.1	97.83693844	118	6	191	2233	2034	1	0	3016

TRINITY_DN11021_c0_g1_i1	Glyma.08G300100.3	100	438	39	435	2460	1	2023	0	1074

TRINITY_DN11021_c0_g2_i1	Glyma.08G300100.3	100	438	39	369	2394	1	2023	0	1074

TRINITY_DN11021_c0_g3_i1	Glyma.08G300100.3	96.875	438	39	369	2394	1	2023	0	1074

TRINITY_DN16190_c0_g3_i6	Glyma.06G277500.1	96.17486339	400	18	271	2306	1	2033	0	1413

TRINITY_DN16314_c1_g1_i2	Glyma.16G165900.2	100	368	33	1912	3941	2033	13	0	1474

TRINITY_DN15451_c0_g1_i5	Glyma.10G053500.1	99.45054945	321	16	555	2589	2033	14	0	1760

TRINITY_DN15451_c0_g1_i6	Glyma.10G053500.1	100	321	16	555	2589	2033	14	0	1760

TRINITY_DN15510_c0_g1_i5	Glyma.09G168500.1	99.06542056	302	19	1	2034	5	2029	0	1892

TRINITY_DN15510_c0_g1_i5	Glyma.09G168500.3	99.21875	302	19	1	2034	5	2029	0	1892

TRINITY_DN16254_c0_g1_i7	Glyma.17G057800.1	98.90710383	279	23	89	2110	2948	912	0	2019

TRINITY_DN15239_c0_g1_i4	Glyma.04G101500.1	100	192	0	245	2290	1	2046	0	2715

TRINITY_DN15239_c0_g1_i5	Glyma.04G101500.1	99.21875	191	0	245	2290	1	2046	0	2721

TRINITY_DN15264_c0_g1_i7	Glyma.03G224100.1	97.14285714	167	3	64	2107	1809	3852	0	2828

TRINITY_DN13654_c0_g2_i1	Glyma.16G015400.3	100	156	11	437	2476	2031	1	0	2778

TRINITY_DN15239_c0_g1_i3	Glyma.04G101500.1	100	154	0	245	2290	1	2046	0	2926

TRINITY_DN15239_c0_g1_i1	Glyma.04G101500.1	100	153	0	245	2290	1	2046	0	2931

TRINITY_DN11821_c0_g1_i2	Glyma.12G178700.1	98.57142857	152	8	2080	4122	2376	347	0	2813

TRINITY_DN14730_c0_g1_i3	Glyma.07G236000.1	100	144	6	292	2328	2040	1	0	2883

TRINITY_DN4234_c0_g1_i1	Glyma.15G017900.1	95.74468085	141	1	185	2230	2841	799	0	2977

TRINITY_DN4234_c0_g2_i1	Glyma.15G017900.1	96	141	1	185	2230	2841	799	0	2977

TRINITY_DN4234_c0_g1_i1	Glyma.13G356200.1	96.6507177	137	1	185	2230	2829	787	0	3000

TRINITY_DN4234_c0_g2_i1	Glyma.13G356200.1	98.85057471	137	1	185	2230	2829	787	0	3000

TRINITY_DN11400_c0_g1_i1	Glyma.09G171000.1	100	135	8	58	2087	2045	1	0	2920

TRINITY_DN11400_c0_g1_i1	Glyma.09G171000.2	96.57794677	135	8	58	2087	2045	1	0	2920

TRINITY_DN6058_c0_g1_i1	Glyma.02G151400.4	97.33333333	100	2	387	2432	2031	1	0	3127

TRINITY_DN16190_c0_g3_i1	Glyma.06G277500.1	95.23809524	413	19	58	2094	871	2901	0	1347

TRINITY_DN13705_c0_g2_i1	Glyma.10G100400.1	99.57805907	293	18	1	2011	89	2129	0	1908

TRINITY_DN12988_c0_g1_i1	Glyma.13G127100.2	98.5915493	179	2	224	2267	22	2065	0	2772

TRINITY_DN13639_c0_g1_i1	Glyma.13G318900.1	100	174	13	1	2023	2076	46	0	2580

TRINITY_DN13639_c0_g1_i1	Glyma.13G318900.2	98.61111111	174	13	1	2023	2076	46	0	2580

TRINITY_DN13095_c0_g1_i1	Glyma.17G063000.2	98.33333333	154	4	461	2495	2044	1	0	2852

TRINITY_DN13095_c0_g1_i3	Glyma.17G063000.1	96.15384615	154	4	725	2759	2044	1	0	2852

TRINITY_DN13095_c0_g1_i3	Glyma.13G096500.1	98.9010989	151	3	725	2759	2044	1	0	2868

TRINITY_DN11400_c0_g1_i1	Glyma.07G106900.1	100	141	4	58	2087	2045	1	0	2898

TRINITY_DN16427_c0_g3_i6	Glyma.04G134800.1	99.06976744	123	2	456	2499	2151	108	0	3083

TRINITY_DN12997_c0_g1_i2	Glyma.13G032500.8	100	354	44	390	2385	2265	240	0	1387

TRINITY_DN15510_c0_g1_i5	Glyma.16G218000.3	98.29268293	332	16	1	2034	5	2035	0	1786

TRINITY_DN15510_c0_g1_i5	Glyma.16G218000.1	95.00509684	332	16	1	2034	5	2035	0	1786

TRINITY_DN16388_c0_g2_i4	Glyma.13G360700.2	100	299	29	398	2413	428	2446	0	1775

TRINITY_DN16237_c0_g1_i3	Glyma.06G164900.1	100	281	29	923	2945	513	2532	0	1927

TRINITY_DN12437_c1_g2_i1	Glyma.04G173000.1	95.97315436	175	5	1	2043	856	2889	0	2734

TRINITY_DN12437_c1_g2_i1	Glyma.04G173000.2	100	175	5	1	2043	856	2889	0	2734

TRINITY_DN12437_c1_g2_i1	Glyma.04G173000.3	99.55555556	174	11	1	2043	856	2877	0	2662

TRINITY_DN12437_c1_g2_i2	Glyma.04G173000.1	98.41269841	173	5	1	2043	856	2889	0	2745

TRINITY_DN12437_c1_g2_i2	Glyma.04G173000.2	99.36305732	173	5	1	2043	856	2889	0	2745

TRINITY_DN12437_c1_g2_i2	Glyma.04G173000.3	97.6744186	172	11	1	2043	856	2877	0	2673

TRINITY_DN11259_c0_g1_i3	Glyma.12G170500.1	96	162	2	101	2143	456	2496	0	2852

TRINITY_DN14730_c0_g1_i3	Glyma.17G037400.1	97.36842105	161	3	292	2328	2043	1	0	2832

TRINITY_DN9158_c0_g1_i1	Glyma.09G164500.1	100	154	2	329	2372	2112	72	0	2902

TRINITY_DN5316_c0_g1_i1	Glyma.17G122200.1	100	127	1	149	2189	2117	74	0	3051

TRINITY_DN5316_c0_g1_i1	Glyma.17G122200.2	100	127	1	149	2189	2066	23	0	3051

TRINITY_DN16039_c0_g1_i3	Glyma.09G066000.1	96.61016949	118	3	895	2938	1542	3579	0	3083

TRINITY_DN16039_c0_g1_i4	Glyma.09G066000.1	97.26027397	118	3	920	2963	1542	3579	0	3083

TRINITY_DN16039_c0_g1_i3	Glyma.09G066000.3	100	118	3	895	2938	633	2670	0	3083

TRINITY_DN16039_c0_g1_i4	Glyma.09G066000.3	98.87640449	118	3	920	2963	633	2670	0	3083

TRINITY_DN16039_c0_g1_i3	Glyma.15G172100.1	96.61016949	110	1	895	2938	1542	3582	0	3145

TRINITY_DN16039_c0_g1_i4	Glyma.15G172100.1	100	110	1	920	2963	1542	3582	0	3145

TRINITY_DN15239_c0_g1_i3	Glyma.13G089200.2	98.50746269	290	11	245	2280	1	2036	0	2076

TRINITY_DN15619_c0_g2_i3	Glyma.04G184000.4	100	283	10	1015	3043	41	2080	0	2095

TRINITY_DN15239_c0_g1_i3	Glyma.14G174200.2	100	281	13	245	2280	1	2036	0	2126

TRINITY_DN15451_c0_g1_i2	Glyma.19G181900.1	97.24409449	239	30	670	2696	2033	40	0	2028

TRINITY_DN15451_c0_g1_i4	Glyma.19G181900.1	96.68367347	239	30	670	2696	2033	40	0	2028

TRINITY_DN15774_c0_g1_i1	Glyma.04G086000.1	97.69230769	220	13	4	2018	2042	1	0	2366

TRINITY_DN15337_c0_g2_i2	Glyma.11G219500.2	100	172	8	1309	3348	1	2031	0	2723

TRINITY_DN15337_c0_g2_i2	Glyma.18G037900.3	95.67723343	164	8	1309	3348	1	2025	0	2728

TRINITY_DN15337_c0_g2_i2	Glyma.18G037900.1	96.42324888	164	8	1309	3348	1	2025	0	2728

TRINITY_DN15337_c0_g2_i2	Glyma.18G037900.6	96.42401022	164	8	1309	3348	1	2025	0	2728

TRINITY_DN12437_c1_g2_i1	Glyma.06G191300.1	96.32352941	156	4	1	2043	868	2904	0	2870

TRINITY_DN12437_c1_g2_i2	Glyma.06G191300.1	98.41269841	154	4	1	2043	868	2904	0	2881

TRINITY_DN9158_c0_g1_i1	Glyma.16G212900.1	100	152	3	329	2371	2109	70	0	2911

TRINITY_DN15048_c0_g1_i2	Glyma.07G243300.1	98.7654321	152	3	889	2931	2040	1	0	2911

TRINITY_DN15048_c0_g1_i2	Glyma.17G030600.1	100	152	4	889	2931	2034	1	0	2872

TRINITY_DN36309_c0_g1_i1	Glyma.11G024300.3	96.72131148	145	0	1	2043	979	3021	0	2970

TRINITY_DN36309_c0_g1_i1	Glyma.11G024300.5	97.9020979	145	0	1	2043	979	3021	0	2970

TRINITY_DN36309_c0_g1_i1	Glyma.11G024300.4	99.13793103	145	0	1	2043	979	3021	0	2970

TRINITY_DN36309_c0_g1_i1	Glyma.11G024300.2	99.02439024	145	0	1	2043	979	3021	0	2970

TRINITY_DN36309_c0_g1_i1	Glyma.11G024300.1	99.02755267	145	0	1	2043	979	3021	0	2970

TRINITY_DN8660_c0_g1_i1	Glyma.16G182300.1	99.30555556	133	1	26	2068	1	2040	0	3016

TRINITY_DN8335_c0_g1_i1	Glyma.05G222700.3	96.55172414	124	4	385	2421	1	2040	0	3027

TRINITY_DN8335_c0_g1_i1	Glyma.05G222700.2	95.40229885	124	4	385	2421	1	2040	0	3027

TRINITY_DN8660_c0_g1_i1	Glyma.09G136900.1	100	123	0	26	2068	1	2043	0	3092

TRINITY_DN15228_c0_g1_i4	Glyma.19G260400.6	97.73462783	389	60	290	2287	64	2058	0	1027

TRINITY_DN15228_c0_g1_i4	Glyma.19G260400.1	99.62546816	389	60	290	2287	64	2058	0	1027

TRINITY_DN16446_c2_g2_i1	Glyma.01G035400.1	100	374	42	294	2301	2255	239	0	1317

TRINITY_DN16446_c2_g2_i1	Glyma.02G026200.1	98.66666667	359	40	300	2301	2300	281	0	1382

TRINITY_DN15306_c0_g1_i1	Glyma.01G082700.1	97.30458221	339	60	873	2863	2577	570	0	1343

TRINITY_DN15306_c0_g1_i2	Glyma.01G082700.1	95.91836735	339	60	2686	4676	2577	570	0	1343

TRINITY_DN15565_c0_g1_i3	Glyma.09G044300.1	97.08737864	238	16	609	2638	51	2071	0	2239

TRINITY_DN15565_c0_g1_i4	Glyma.09G044300.1	99.0990991	238	16	622	2651	51	2071	0	2239

TRINITY_DN15239_c0_g1_i4	Glyma.13G089200.2	100	228	9	245	2280	1	2036	0	2431

TRINITY_DN15239_c0_g1_i4	Glyma.14G174200.2	96.2890625	217	11	245	2280	1	2036	0	2492

TRINITY_DN15934_c0_g1_i2	Glyma.10G212600.3	100	184	8	954	2984	9	2049	0	2675

TRINITY_DN15510_c0_g1_i3	Glyma.16G218000.3	95.39170507	179	12	314	2342	1	2035	0	2651

TRINITY_DN15510_c0_g1_i3	Glyma.16G218000.1	99.13043478	179	12	314	2342	1	2035	0	2651

TRINITY_DN15510_c0_g1_i6	Glyma.16G218000.3	100	179	12	288	2316	1	2035	0	2651

TRINITY_DN15510_c0_g1_i6	Glyma.16G218000.1	99.35064935	179	12	288	2316	1	2035	0	2651

TRINITY_DN15187_c0_g1_i1	Glyma.15G271800.1	100	165	1	617	2655	4201	2160	0	2837

TRINITY_DN9813_c0_g1_i1	Glyma.02G271700.4	100	142	1	440	2478	1	2042	0	2964

TRINITY_DN9813_c0_g1_i1	Glyma.02G271700.2	100	142	1	440	2478	1	2042	0	2964

TRINITY_DN9813_c0_g1_i1	Glyma.02G271700.3	96.69421488	142	1	440	2478	1	2042	0	2964

TRINITY_DN15339_c0_g1_i1	Glyma.03G181600.1	95.69892473	310	2	253	2292	2150	111	0	2039

TRINITY_DN16237_c0_g1_i3	Glyma.04G200600.1	100	283	23	923	2945	513	2532	0	1949

TRINITY_DN14003_c0_g1_i1	Glyma.04G007700.1	97.44186047	229	24	219	2215	2039	1	0	2204

TRINITY_DN5432_c0_g1_i1	Glyma.16G000500.2	96.68246445	171	5	3	2040	1	2038	0	2784

TRINITY_DN12976_c0_g1_i2	Glyma.19G243400.1	99.06542056	157	8	55	2087	1	2036	0	2815

TRINITY_DN16069_c0_g1_i1	Glyma.04G213100.1	100	149	0	2276	4316	2587	4627	0	2944

TRINITY_DN13694_c0_g1_i3	Glyma.07G103000.3	95.75892857	146	1	102	2141	1	2041	0	2953

TRINITY_DN13694_c0_g1_i3	Glyma.07G103000.1	96.32516704	146	1	102	2141	1	2041	0	2953

TRINITY_DN13694_c0_g1_i3	Glyma.07G103000.2	95.68345324	146	1	102	2141	1	2041	0	2953

TRINITY_DN15619_c0_g2_i3	Glyma.05G038300.1	100	142	0	912	2952	1	2041	0	2983

TRINITY_DN9813_c0_g1_i1	Glyma.02G271700.1	96.69421488	142	1	440	2477	1	2041	0	2963

TRINITY_DN14979_c0_g1_i1	Glyma.14G223400.4	95.30201342	133	2	616	2653	2041	1	0	3013

TRINITY_DN14979_c0_g1_i1	Glyma.14G223400.5	95.30685921	133	2	616	2653	2041	1	0	3013

TRINITY_DN14979_c0_g1_i5	Glyma.14G223400.3	96.4673913	133	2	679	2716	2041	1	0	3013

TRINITY_DN14979_c0_g1_i5	Glyma.14G223400.6	96.47058824	133	2	679	2716	2041	1	0	3013

TRINITY_DN14979_c0_g1_i5	Glyma.14G223400.7	96.47887324	133	2	679	2716	2041	1	0	3013

TRINITY_DN14979_c0_g1_i5	Glyma.14G223400.2	96.47058824	133	2	679	2716	2041	1	0	3013

TRINITY_DN16333_c0_g1_i4	Glyma.08G065100.1	97.82608696	125	3	191	2230	2034	7	0	2987

TRINITY_DN16333_c0_g1_i4	Glyma.07G184300.1	97.82945736	125	3	191	2230	2034	7	0	2987

TRINITY_DN11825_c0_g2_i1	Glyma.17G126700.1	98.57142857	122	2	376	2415	2038	1	0	3068

TRINITY_DN11825_c0_g2_i3	Glyma.17G126700.1	98.57142857	122	2	376	2415	2038	1	0	3068

TRINITY_DN15339_c0_g1_i1	Glyma.19G182300.1	96.15384615	319	2	254	2292	2137	99	0	1988

TRINITY_DN16190_c0_g3_i1	Glyma.13G270400.4	98.24561404	266	5	58	2094	868	2904	0	2255

TRINITY_DN16190_c0_g3_i1	Glyma.13G270400.2	98.24561404	266	5	58	2094	868	2904	0	2255

TRINITY_DN16190_c0_g3_i1	Glyma.13G270400.3	98.24561404	266	5	58	2094	868	2904	0	2255

TRINITY_DN16190_c0_g3_i1	Glyma.13G270400.1	100	266	5	58	2094	868	2904	0	2255

TRINITY_DN16190_c0_g3_i1	Glyma.13G270400.5	95.47738693	266	5	58	2094	868	2904	0	2255

TRINITY_DN15565_c0_g1_i3	Glyma.15G148800.1	95.66360053	240	14	609	2637	51	2067	0	2218

TRINITY_DN15565_c0_g1_i4	Glyma.15G148800.1	96.66666667	240	14	622	2650	51	2067	0	2218

TRINITY_DN231_c0_g2_i1	Glyma.02G213400.1	98.16513761	224	36	275	2273	1	1987	0	1919

TRINITY_DN15565_c0_g1_i1	Glyma.07G246800.2	96.77419355	215	17	496	2500	96	2127	0	2298

TRINITY_DN15565_c0_g1_i1	Glyma.07G246800.1	98.26589595	215	17	496	2500	96	2127	0	2298

TRINITY_DN8879_c0_g2_i1	Glyma.18G239500.1	96.46017699	189	5	34	2072	9	2041	0	2669

TRINITY_DN14345_c0_g2_i1	Glyma.08G124600.2	100	165	3	672	2708	2037	1	0	2815

TRINITY_DN11749_c0_g1_i3	Glyma.06G002400.1	95.23809524	161	1	1	2040	299	2335	0	2856

TRINITY_DN11749_c0_g1_i4	Glyma.06G002400.1	95.28301887	161	1	1	2040	299	2335	0	2856

TRINITY_DN9565_c0_g1_i1	Glyma.19G077000.1	100	161	14	434	2430	2035	4	0	2545

TRINITY_DN9813_c0_g1_i1	Glyma.14G044700.1	97.77777778	142	2	440	2478	1	2039	0	2968

TRINITY_DN9813_c0_g1_i1	Glyma.14G044700.3	98.11320755	142	2	440	2478	1	2039	0	2968

TRINITY_DN14268_c0_g1_i1	Glyma.05G006700.1	100	140	7	681	2696	1	2031	0	2778

TRINITY_DN14268_c0_g1_i2	Glyma.05G006700.1	100	140	7	670	2685	1	2031	0	2778

TRINITY_DN6026_c0_g1_i1	Glyma.06G223800.1	97.27272727	134	2	226	2262	103	2142	0	3005

TRINITY_DN12070_c0_g1_i1	Glyma.18G253400.3	99.03225806	116	2	402	2440	1	2039	0	3112

TRINITY_DN12070_c0_g1_i1	Glyma.18G253400.4	99.0228013	116	2	402	2440	1	2039	0	3112

TRINITY_DN12070_c0_g1_i2	Glyma.18G253400.3	99.02439024	116	2	594	2632	1	2039	0	3112

TRINITY_DN12070_c0_g1_i2	Glyma.18G253400.4	99.01960784	116	2	594	2632	1	2039	0	3112

TRINITY_DN10039_c0_g1_i1	Glyma.02G182800.1	98.5915493	116	9	80	2107	1	2031	0	2988

TRINITY_DN5443_c0_g1_i1	Glyma.10G001400.2	95	83	0	149	2188	1	2040	0	3308

TRINITY_DN5443_c0_g1_i1	Glyma.10G001400.1	95.56962025	83	0	149	2188	1	2040	0	3308

TRINITY_DN16446_c2_g2_i1	Glyma.02G030700.1	100	413	25	292	2301	2263	239	0	1199

TRINITY_DN14680_c0_g2_i1	Glyma.13G134500.1	96.59090909	375	42	43	2053	353	2350	0	1242

TRINITY_DN14680_c0_g2_i2	Glyma.13G134500.1	100	375	42	43	2053	353	2350	0	1242

TRINITY_DN14680_c0_g2_i5	Glyma.13G134500.1	98.75776398	375	42	43	2053	353	2350	0	1242

TRINITY_DN14680_c0_g2_i6	Glyma.13G134500.1	97.51243781	375	42	43	2053	353	2350	0	1242

TRINITY_DN13448_c0_g1_i2	Glyma.18G011000.1	98.30508475	277	2	17	2054	43	2080	0	2218

TRINITY_DN16397_c0_g1_i3	Glyma.19G065600.2	100	227	2	2406	4443	2830	793	0	2495

TRINITY_DN16397_c0_g1_i3	Glyma.19G065600.3	100	227	2	2406	4443	2686	649	0	2495

TRINITY_DN16397_c0_g1_i4	Glyma.19G065600.2	98.14814815	217	2	2244	4281	2830	793	0	2551

TRINITY_DN16397_c0_g1_i4	Glyma.19G065600.3	100	217	2	2244	4281	2686	649	0	2551

TRINITY_DN15329_c0_g2_i1	Glyma.02G085000.2	100	206	6	888	2924	2034	1	0	2579

TRINITY_DN15329_c0_g2_i1	Glyma.02G085000.5	100	206	6	888	2924	2034	1	0	2579

TRINITY_DN15329_c0_g2_i1	Glyma.02G085000.3	98.11320755	206	6	888	2924	2034	1	0	2579

TRINITY_DN15329_c0_g2_i1	Glyma.02G085000.1	95.04132231	206	6	888	2924	2034	1	0	2579

TRINITY_DN15329_c0_g2_i2	Glyma.02G085000.4	100	206	6	512	2548	2034	1	0	2579

TRINITY_DN35694_c0_g1_i1	Glyma.20G094700.1	99.35897436	205	5	196	2232	176	2209	0	2584

TRINITY_DN14026_c0_g2_i1	Glyma.13G062700.1	98.95833333	202	14	1	2022	2034	1	0	2505

TRINITY_DN14026_c0_g4_i1	Glyma.13G062700.1	100	202	14	1	2022	2034	1	0	2505

TRINITY_DN15510_c0_g1_i5	Glyma.16G218200.1	100	148	8	1	2034	5	2032	0	2843

TRINITY_DN12997_c0_g1_i2	Glyma.13G032500.3	100	355	41	390	2385	2259	240	0	1410

TRINITY_DN12997_c0_g1_i2	Glyma.13G032500.7	98.64864865	355	41	390	2385	2259	240	0	1410

TRINITY_DN16352_c0_g2_i1	Glyma.08G190700.1	100	292	21	427	2435	4381	2349	0	1927

TRINITY_DN13448_c0_g1_i2	Glyma.11G246200.8	100	287	0	17	2054	43	2080	0	2174

TRINITY_DN13448_c0_g1_i2	Glyma.11G246200.6	100	287	0	17	2054	43	2080	0	2174

TRINITY_DN13448_c0_g1_i2	Glyma.11G246200.9	99.00990099	287	0	17	2054	43	2080	0	2174

TRINITY_DN13448_c0_g1_i2	Glyma.11G246200.1	99.00990099	287	0	17	2054	43	2080	0	2174

TRINITY_DN13654_c0_g1_i2	Glyma.16G015400.3	99.54954955	286	32	437	2446	2031	28	0	1779

TRINITY_DN14897_c0_g1_i1	Glyma.08G263800.2	95.09803922	268	27	166	2184	4	2010	0	1956

TRINITY_DN4222_c0_g1_i1	Glyma.07G265700.1	98.52941176	207	5	332	2363	193	2221	0	2519

TRINITY_DN4222_c0_g1_i1	Glyma.07G265700.2	98.52941176	207	5	332	2363	46	2074	0	2519

TRINITY_DN19139_c0_g2_i1	Glyma.17G006500.1	97.82608696	187	23	47	2033	59	2093	0	2379

TRINITY_DN14027_c0_g1_i1	Glyma.20G217200.1	97.4248927	163	3	633	2669	2042	7	0	2841

TRINITY_DN16436_c0_g2_i5	Glyma.12G055900.1	100	160	2	3	2039	253	2289	0	2865

TRINITY_DN8498_c0_g1_i1	Glyma.08G101800.1	95.34883721	158	22	121	2124	2031	1	0	2623

TRINITY_DN8335_c0_g1_i1	Glyma.08G029500.1	95.40229885	140	10	385	2421	1	2022	0	2878

TRINITY_DN11579_c0_g1_i2	Glyma.15G033700.1	98.88888889	135	5	97	2127	1	2034	0	2944

TRINITY_DN6272_c0_g1_i1	Glyma.02G305600.1	99.0625	122	14	1	2029	2064	45	0	2913

TRINITY_DN14680_c0_g2_i1	Glyma.10G046400.1	100	374	39	43	2053	353	2353	0	1290

TRINITY_DN14680_c0_g2_i1	Glyma.10G046400.2	95.22058824	374	39	43	2053	353	2353	0	1290

TRINITY_DN14680_c0_g2_i2	Glyma.10G046400.1	99.80430528	374	39	43	2053	353	2353	0	1290

TRINITY_DN14680_c0_g2_i2	Glyma.10G046400.2	96.01226994	374	39	43	2053	353	2353	0	1290

TRINITY_DN14680_c0_g2_i5	Glyma.10G046400.1	95.59748428	374	39	43	2053	353	2353	0	1290

TRINITY_DN14680_c0_g2_i5	Glyma.10G046400.2	98.75776398	374	39	43	2053	353	2353	0	1290

TRINITY_DN14680_c0_g2_i6	Glyma.10G046400.1	99.16201117	374	39	43	2053	353	2353	0	1290

TRINITY_DN14680_c0_g2_i6	Glyma.10G046400.2	97.51166407	374	39	43	2053	353	2353	0	1290

TRINITY_DN15970_c0_g1_i1	Glyma.18G034300.1	95.8677686	269	6	165	2198	40	2073	0	2233

TRINITY_DN15565_c0_g1_i1	Glyma.17G027200.1	98.26589595	221	16	496	2498	96	2128	0	2292

TRINITY_DN15812_c0_g1_i2	Glyma.05G011300.1	97.05882353	196	10	495	2526	2727	696	0	2612

TRINITY_DN15812_c0_g1_i2	Glyma.05G011300.2	100	196	10	495	2526	2727	696	0	2612

TRINITY_DN15812_c0_g1_i7	Glyma.05G011300.1	96.92307692	196	10	578	2609	2727	696	0	2612

TRINITY_DN15812_c0_g1_i7	Glyma.05G011300.2	99.08256881	196	10	578	2609	2727	696	0	2612

TRINITY_DN13508_c0_g1_i3	Glyma.18G210100.1	98.7654321	180	40	94	2036	1	2012	0	1995

TRINITY_DN7983_c0_g1_i2	Glyma.13G184200.2	95.78947368	176	8	171	2204	1	2028	0	2710

TRINITY_DN15200_c0_g1_i2	Glyma.10G177300.1	98.94736842	169	1	252	2288	2034	1	0	2806

TRINITY_DN15200_c0_g1_i2	Glyma.20G212800.1	98.94736842	167	1	252	2288	2034	1	0	2817

TRINITY_DN12059_c0_g1_i1	Glyma.11G133600.2	95.37037037	166	3	623	2659	2025	1	0	2765

TRINITY_DN12059_c0_g1_i1	Glyma.11G133600.1	95.37037037	166	3	623	2659	2025	1	0	2765

TRINITY_DN12059_c0_g1_i2	Glyma.11G133600.2	95.52238806	166	3	623	2659	2025	1	0	2765

TRINITY_DN12059_c0_g1_i2	Glyma.11G133600.1	95.52238806	166	3	623	2659	2025	1	0	2765

TRINITY_DN12059_c0_g1_i3	Glyma.11G133600.2	95.52845528	166	3	426	2462	2025	1	0	2765

TRINITY_DN12059_c0_g1_i3	Glyma.11G133600.1	95.52845528	166	3	426	2462	2025	1	0	2765

TRINITY_DN12059_c0_g1_i4	Glyma.11G133600.2	95.48387097	166	3	426	2462	2025	1	0	2765

TRINITY_DN12059_c0_g1_i4	Glyma.11G133600.1	95.48387097	166	3	426	2462	2025	1	0	2765

TRINITY_DN14562_c0_g1_i6	Glyma.04G236600.4	99.08536585	161	5	26	2062	1	2022	0	2772

TRINITY_DN14562_c0_g1_i6	Glyma.04G236600.1	99.08536585	161	5	26	2062	1	2022	0	2772

TRINITY_DN14562_c0_g1_i6	Glyma.04G236600.2	100	161	5	26	2062	1	2022	0	2772

TRINITY_DN14562_c0_g1_i6	Glyma.04G236600.3	100	161	5	26	2062	1	2022	0	2772

TRINITY_DN15812_c0_g1_i3	Glyma.05G011300.1	98.64253394	161	10	578	2609	2727	696	0	2806

TRINITY_DN15812_c0_g1_i3	Glyma.05G011300.2	98.64253394	161	10	578	2609	2727	696	0	2806

TRINITY_DN15812_c0_g1_i8	Glyma.05G011300.1	99.05660377	161	10	495	2526	2727	696	0	2806

TRINITY_DN15812_c0_g1_i8	Glyma.05G011300.2	99.05660377	161	10	495	2526	2727	696	0	2806

TRINITY_DN12059_c0_g1_i1	Glyma.12G057800.1	95.38461538	159	3	623	2659	2025	1	0	2804

TRINITY_DN12059_c0_g1_i1	Glyma.12G057800.2	95.40983607	159	3	623	2659	2025	1	0	2804

TRINITY_DN12059_c0_g1_i1	Glyma.12G057800.3	95.44072948	159	3	623	2659	2025	1	0	2804

TRINITY_DN12059_c0_g1_i2	Glyma.12G057800.1	95.52238806	159	3	623	2659	2025	1	0	2804

TRINITY_DN12059_c0_g1_i2	Glyma.12G057800.2	95.5017301	159	3	623	2659	2025	1	0	2804

TRINITY_DN12059_c0_g1_i2	Glyma.12G057800.3	95.41984733	159	3	623	2659	2025	1	0	2804

TRINITY_DN12059_c0_g1_i3	Glyma.12G057800.1	95.52715655	159	3	426	2462	2025	1	0	2804

TRINITY_DN12059_c0_g1_i3	Glyma.12G057800.2	95.52845528	159	3	426	2462	2025	1	0	2804

TRINITY_DN12059_c0_g1_i3	Glyma.12G057800.3	95.52845528	159	3	426	2462	2025	1	0	2804

TRINITY_DN12059_c0_g1_i4	Glyma.12G057800.1	95.47511312	159	3	426	2462	2025	1	0	2804

TRINITY_DN12059_c0_g1_i4	Glyma.12G057800.2	95.47738693	159	3	426	2462	2025	1	0	2804

TRINITY_DN12059_c0_g1_i4	Glyma.12G057800.3	95.47738693	159	3	426	2462	2025	1	0	2804

TRINITY_DN14562_c0_g1_i6	Glyma.06G127700.4	97.10144928	155	4	26	2062	1	2022	0	2806

TRINITY_DN14562_c0_g1_i6	Glyma.06G127700.3	97.10144928	155	4	26	2062	1	2022	0	2806

TRINITY_DN14562_c0_g1_i6	Glyma.06G127700.2	98.92473118	155	4	26	2062	1	2022	0	2806

TRINITY_DN14562_c0_g1_i6	Glyma.06G127700.1	96.28378378	155	4	26	2062	1	2022	0	2806

TRINITY_DN36309_c0_g1_i1	Glyma.01G219200.1	99.0990991	140	0	1	2037	868	2904	0	2987

TRINITY_DN15510_c0_g1_i5	Glyma.09G168600.3	100	137	5	1	2034	5	2032	0	2926

TRINITY_DN15510_c0_g1_i5	Glyma.09G168600.4	97.82608696	137	5	1	2034	5	2032	0	2926

TRINITY_DN15510_c0_g1_i5	Glyma.09G168600.1	95.28936743	137	5	1	2034	5	2032	0	2926

TRINITY_DN16190_c0_g3_i1	Glyma.06G229900.1	100	135	0	58	2094	868	2904	0	3014

TRINITY_DN16190_c0_g3_i1	Glyma.12G161300.1	96.66666667	131	0	58	2094	868	2904	0	3037

TRINITY_DN16333_c0_g1_i2	Glyma.07G184300.1	97.84313725	124	5	191	2224	2034	1	0	3037

TRINITY_DN13959_c0_g1_i1	Glyma.10G292300.4	98.4496124	124	16	646	2657	2565	535	0	2874

TRINITY_DN13959_c0_g1_i2	Glyma.10G292300.4	95.65217391	124	16	646	2657	2565	535	0	2874

TRINITY_DN16333_c0_g1_i2	Glyma.08G065100.1	97.82608696	120	5	191	2224	2034	1	0	3059

TRINITY_DN15329_c0_g2_i1	Glyma.05G238700.1	100	100	0	888	2924	2037	1	0	3208

TRINITY_DN15329_c0_g2_i2	Glyma.05G238700.1	98.41269841	100	0	512	2548	2037	1	0	3208

TRINITY_DN15451_c0_g1_i2	Glyma.10G053500.1	98.00443459	339	36	694	2692	2009	14	0	1384

TRINITY_DN15451_c0_g1_i4	Glyma.10G053500.1	97.70114943	339	36	694	2692	2009	14	0	1384

TRINITY_DN12290_c0_g1_i2	Glyma.08G013600.1	100	326	2	464	2498	2035	1	0	1941

TRINITY_DN15750_c1_g1_i3	Glyma.13G104000.1	100	294	9	736	2766	2083	53	0	2067

TRINITY_DN16190_c0_g3_i6	Glyma.13G270400.4	95.23809524	216	0	271	2306	1	2036	0	2564

TRINITY_DN16190_c0_g3_i6	Glyma.13G270400.2	100	216	0	271	2306	1	2036	0	2564

TRINITY_DN16190_c0_g3_i6	Glyma.13G270400.3	100	216	0	271	2306	1	2036	0	2564

TRINITY_DN16190_c0_g3_i6	Glyma.13G270400.1	100	216	0	271	2306	1	2036	0	2564

TRINITY_DN16190_c0_g3_i6	Glyma.13G270400.5	98.4375	216	0	271	2306	1	2036	0	2564

TRINITY_DN14026_c0_g2_i1	Glyma.19G022900.1	98.95833333	214	11	1	2022	2028	1	0	2433

TRINITY_DN4045_c0_g1_i1	Glyma.05G142300.1	95.97315436	214	18	68	2083	163	2190	0	2394

TRINITY_DN15970_c0_g1_i5	Glyma.18G034300.1	96.12903226	213	3	165	2198	40	2073	0	2555

TRINITY_DN16190_c0_g3_i6	Glyma.12G229400.3	98.97959184	206	0	271	2306	1	2036	0	2619

TRINITY_DN16190_c0_g3_i6	Glyma.12G229400.1	100	206	0	271	2306	1	2036	0	2619

TRINITY_DN16190_c0_g3_i6	Glyma.12G229400.2	95.23809524	206	0	271	2306	1	2036	0	2619

TRINITY_DN16436_c0_g2_i5	Glyma.11G131700.1	97.72727273	184	0	4	2039	257	2292	0	2741

TRINITY_DN16436_c0_g2_i5	Glyma.11G131700.2	96.68874172	184	0	4	2039	236	2271	0	2741

TRINITY_DN16436_c0_g2_i5	Glyma.11G131700.3	99.13043478	184	0	4	2039	212	2247	0	2741

TRINITY_DN9723_c0_g1_i1	Glyma.10G153900.1	100	181	4	125	2158	2598	565	0	2732

TRINITY_DN15506_c0_g2_i11	Glyma.15G074200.2	100	180	16	1711	3734	458	2475	0	2569

TRINITY_DN15506_c0_g2_i12	Glyma.15G074200.2	97.36842105	180	16	2025	4048	458	2475	0	2569

TRINITY_DN15506_c0_g2_i13	Glyma.15G074200.2	100	180	16	1793	3816	458	2475	0	2569

TRINITY_DN15506_c0_g2_i3	Glyma.15G074200.2	100	180	16	1628	3651	458	2475	0	2569

TRINITY_DN15506_c0_g2_i5	Glyma.15G074200.2	100	180	16	1942	3965	458	2475	0	2569

TRINITY_DN15506_c0_g2_i6	Glyma.15G074200.2	98.73417722	180	16	1710	3733	458	2475	0	2569

TRINITY_DN15510_c0_g1_i3	Glyma.09G168500.1	97.22222222	175	6	314	2342	1	2029	0	2700

TRINITY_DN15510_c0_g1_i3	Glyma.09G168500.3	97.22222222	175	6	314	2342	1	2029	0	2700

TRINITY_DN15510_c0_g1_i6	Glyma.09G168500.1	96.51162791	175	6	288	2316	1	2029	0	2700

TRINITY_DN15510_c0_g1_i6	Glyma.09G168500.3	100	175	6	288	2316	1	2029	0	2700

TRINITY_DN13056_c0_g1_i1	Glyma.19G141900.7	96.25	170	0	721	2756	1087	3122	0	2819

TRINITY_DN7209_c0_g2_i1	Glyma.07G085600.1	100	170	35	74	2062	2016	1	0	2385

TRINITY_DN7209_c0_g2_i2	Glyma.07G085600.1	100	170	35	219	2207	2016	1	0	2385

TRINITY_DN16190_c0_g3_i6	Glyma.06G229900.2	95.20547945	151	0	271	2306	1	2036	0	2924

TRINITY_DN16190_c0_g3_i6	Glyma.06G229900.1	98.07692308	151	0	271	2306	1	2036	0	2924

TRINITY_DN16190_c0_g3_i6	Glyma.12G161300.1	98.1981982	148	0	271	2306	1	2036	0	2940

TRINITY_DN216_c0_g1_i1	Glyma.05G117400.1	100	134	0	1	2036	179	2214	0	3018

TRINITY_DN12997_c0_g1_i2	Glyma.13G032500.2	99.60474308	355	40	390	2385	2256	240	0	1423

TRINITY_DN12997_c0_g1_i2	Glyma.13G032500.6	96.34146341	355	40	390	2385	2256	240	0	1423

TRINITY_DN11160_c0_g1_i1	Glyma.16G144600.1	96.19047619	332	11	418	2440	2688	657	0	1821

TRINITY_DN15750_c1_g1_i3	Glyma.17G055100.1	100	296	7	736	2766	2083	53	0	2067

TRINITY_DN16254_c0_g1_i7	Glyma.13G101700.1	98.90625	285	19	89	2110	2936	912	0	2030

TRINITY_DN16254_c0_g1_i7	Glyma.17G057800.4	100	279	20	89	2110	2936	912	0	2063

TRINITY_DN16254_c0_g1_i7	Glyma.17G057800.3	100	279	20	89	2110	2936	912	0	2063

TRINITY_DN16254_c0_g1_i7	Glyma.17G057800.2	100	279	20	89	2110	2936	912	0	2063

TRINITY_DN15501_c0_g2_i6	Glyma.13G162800.2	100	260	2	103	2136	2034	1	0	2305

TRINITY_DN12290_c0_g1_i2	Glyma.01G219000.1	95.45454545	259	18	1363	3377	2021	1	0	2104

TRINITY_DN15501_c0_g2_i1	Glyma.13G162800.2	96.61016949	257	2	568	2601	2034	1	0	2322

TRINITY_DN13471_c0_g2_i1	Glyma.16G022600.1	100	251	6	205	2236	2020	1	0	2252

TRINITY_DN13471_c0_g2_i1	Glyma.16G022600.5	97.81420765	251	7	205	2236	2017	1	0	2231

TRINITY_DN14026_c0_g4_i1	Glyma.19G022900.1	99.03381643	216	9	1	2022	2028	1	0	2433

TRINITY_DN14345_c0_g2_i1	Glyma.05G166600.2	100	151	3	678	2708	2034	1	0	2889

TRINITY_DN14345_c0_g2_i1	Glyma.05G166600.3	98.7654321	149	9	678	2708	2016	1	0	2784

TRINITY_DN14790_c0_g1_i1	Glyma.08G329100.1	97.6744186	147	37	329	2321	2223	237	0	2362

TRINITY_DN14790_c0_g1_i2	Glyma.08G329100.1	95.91836735	147	37	329	2321	2223	237	0	2362

TRINITY_DN16064_c0_g2_i1	Glyma.03G168200.1	100	469	37	264	2277	263	2276	0	900

TRINITY_DN16064_c0_g2_i3	Glyma.03G168200.1	99.44751381	469	37	264	2277	263	2276	0	900

TRINITY_DN16360_c0_g8_i1	Glyma.17G254100.1	100	452	34	264	2278	97	2108	0	987

TRINITY_DN11254_c0_g1_i1	Glyma.13G347600.2	97.51332149	423	42	551	2559	2106	99	0	1083

TRINITY_DN6249_c0_g1_i2	Glyma.11G030000.1	100	352	25	130	2133	47	2065	0	1515

TRINITY_DN9237_c0_g1_i1	Glyma.05G151200.2	100	263	14	264	2290	176	2202	0	2209

TRINITY_DN9237_c0_g1_i1	Glyma.05G151200.1	100	263	14	264	2290	176	2202	0	2209

TRINITY_DN231_c0_g2_i1	Glyma.14G181100.1	100	210	33	275	2273	1	1990	0	2082

TRINITY_DN15812_c0_g1_i2	Glyma.17G119400.5	96.1038961	199	4	495	2526	2712	681	0	2628

TRINITY_DN15812_c0_g1_i2	Glyma.17G119400.3	96.93877551	199	4	495	2526	2712	681	0	2628

TRINITY_DN15812_c0_g1_i2	Glyma.17G119400.2	96.93877551	199	4	495	2526	2712	681	0	2628

TRINITY_DN15812_c0_g1_i2	Glyma.17G119400.1	100	199	4	495	2526	2712	681	0	2628

TRINITY_DN15812_c0_g1_i2	Glyma.17G119400.4	100	199	4	495	2526	2712	681	0	2628

TRINITY_DN15812_c0_g1_i2	Glyma.17G119400.6	98.93617021	199	4	495	2526	2712	681	0	2628

TRINITY_DN15812_c0_g1_i7	Glyma.17G119400.5	100	199	4	578	2609	2712	681	0	2628

TRINITY_DN15812_c0_g1_i7	Glyma.17G119400.3	96.92307692	199	4	578	2609	2712	681	0	2628

TRINITY_DN15812_c0_g1_i7	Glyma.17G119400.2	96.92307692	199	4	578	2609	2712	681	0	2628

TRINITY_DN15812_c0_g1_i7	Glyma.17G119400.1	96.92307692	199	4	578	2609	2712	681	0	2628

TRINITY_DN15812_c0_g1_i7	Glyma.17G119400.4	96.92307692	199	4	578	2609	2712	681	0	2628

TRINITY_DN15812_c0_g1_i7	Glyma.17G119400.6	96.92307692	199	4	578	2609	2712	681	0	2628

TRINITY_DN15812_c0_g1_i3	Glyma.17G119400.5	98.46153846	162	4	578	2609	2712	681	0	2833

TRINITY_DN15812_c0_g1_i3	Glyma.17G119400.3	98.46153846	162	4	578	2609	2712	681	0	2833

TRINITY_DN15812_c0_g1_i3	Glyma.17G119400.2	98.61111111	162	4	578	2609	2712	681	0	2833

TRINITY_DN15812_c0_g1_i3	Glyma.17G119400.1	98.61111111	162	4	578	2609	2712	681	0	2833

TRINITY_DN15812_c0_g1_i3	Glyma.17G119400.4	100	162	4	578	2609	2712	681	0	2833

TRINITY_DN15812_c0_g1_i3	Glyma.17G119400.6	100	162	4	578	2609	2712	681	0	2833

TRINITY_DN15812_c0_g1_i8	Glyma.17G119400.5	97.6744186	162	4	495	2526	2712	681	0	2833

TRINITY_DN15812_c0_g1_i8	Glyma.17G119400.3	100	162	4	495	2526	2712	681	0	2833

TRINITY_DN15812_c0_g1_i8	Glyma.17G119400.2	95.68345324	162	4	495	2526	2712	681	0	2833

TRINITY_DN15812_c0_g1_i8	Glyma.17G119400.1	95.68345324	162	4	495	2526	2712	681	0	2833

TRINITY_DN15812_c0_g1_i8	Glyma.17G119400.4	95.68345324	162	4	495	2526	2712	681	0	2833

TRINITY_DN15812_c0_g1_i8	Glyma.17G119400.6	95.68345324	162	4	495	2526	2712	681	0	2833

TRINITY_DN15710_c0_g1_i1	Glyma.12G074600.6	95.09803922	161	2	182	2212	2514	484	0	2826

TRINITY_DN15710_c0_g1_i1	Glyma.12G074600.1	99.0990991	161	2	182	2212	2514	484	0	2826

TRINITY_DN15710_c0_g1_i1	Glyma.12G074600.5	95.96412556	161	2	182	2212	2514	484	0	2826

TRINITY_DN15710_c0_g1_i1	Glyma.12G074600.10	95.96541787	161	2	182	2212	2514	484	0	2826

TRINITY_DN15710_c0_g1_i1	Glyma.12G074600.7	99.07407407	161	2	182	2212	2514	484	0	2826

TRINITY_DN15710_c0_g1_i1	Glyma.12G074600.3	97.64011799	161	2	182	2212	2514	484	0	2826

TRINITY_DN15710_c0_g1_i1	Glyma.12G074600.2	97.93814433	161	2	182	2212	2514	484	0	2826

TRINITY_DN15710_c0_g1_i1	Glyma.12G074600.4	95.23809524	161	2	182	2212	2514	484	0	2826

TRINITY_DN15431_c0_g1_i2	Glyma.08G241600.1	100	157	11	401	2420	2157	126	0	2784

TRINITY_DN15431_c0_g1_i7	Glyma.08G241600.1	100	157	11	401	2420	2157	126	0	2784

TRINITY_DN15431_c0_g1_i2	Glyma.08G241600.2	100	157	11	401	2420	2151	120	0	2784

TRINITY_DN15431_c0_g1_i7	Glyma.08G241600.2	96.34551495	157	11	401	2420	2151	120	0	2784

TRINITY_DN15431_c0_g1_i2	Glyma.18G263900.1	97.59036145	154	11	401	2419	2145	115	0	2787

TRINITY_DN15431_c0_g1_i7	Glyma.18G263900.1	100	154	11	401	2419	2145	115	0	2787

TRINITY_DN16382_c0_g1_i7	Glyma.14G195700.1	98.91304348	130	1	3	2033	193	2226	0	3016

TRINITY_DN15501_c0_g2_i2	Glyma.13G162800.2	100	115	0	639	2672	2034	1	0	3120

TRINITY_DN15501_c0_g2_i3	Glyma.13G162800.3	98.79518072	115	0	608	2641	2034	1	0	3120

TRINITY_DN15501_c0_g2_i3	Glyma.13G162800.1	100	115	0	608	2641	2034	1	0	3120

TRINITY_DN16360_c0_g8_i1	Glyma.14G070600.1	100	454	33	264	2278	97	2108	0	987

TRINITY_DN2216_c0_g1_i1	Glyma.20G204800.1	97.05882353	362	34	215	2233	1989	1	0	1375

TRINITY_DN15768_c0_g1_i1	Glyma.10G048800.2	100	286	9	819	2848	2	2016	0	2034

TRINITY_DN15778_c0_g1_i1	Glyma.18G196200.1	99.24242424	267	16	1026	3049	2015	1	0	2100

TRINITY_DN16333_c0_g1_i3	Glyma.15G059900.1	97.83393502	216	3	193	2224	2023	4	0	2468

TRINITY_DN16333_c0_g1_i3	Glyma.13G254900.1	97.82608696	215	3	193	2224	2023	4	0	2473

TRINITY_DN8602_c0_g1_i1	Glyma.03G161800.6	98.67549669	159	17	428	2451	2028	11	0	2719

TRINITY_DN8602_c0_g1_i1	Glyma.03G161800.7	95.83333333	159	17	428	2451	2028	11	0	2719

TRINITY_DN13515_c0_g1_i2	Glyma.07G036400.1	100	153	10	257	2278	1	2031	0	2822

TRINITY_DN12719_c0_g2_i6	Glyma.13G321300.38	100	153	21	356	2376	1983	1	0	2507

TRINITY_DN12719_c0_g2_i6	Glyma.13G321300.39	98.31932773	153	21	356	2376	1983	1	0	2507

TRINITY_DN12719_c0_g2_i6	Glyma.13G321300.26	95.6043956	153	21	356	2376	1983	1	0	2507

TRINITY_DN12719_c0_g2_i6	Glyma.13G321300.27	100	153	21	356	2376	1983	1	0	2507

TRINITY_DN12719_c0_g2_i6	Glyma.13G321300.7	98.27586207	153	21	356	2376	1983	1	0	2507

TRINITY_DN12719_c0_g2_i6	Glyma.13G321300.6	100	153	21	356	2376	1983	1	0	2507

TRINITY_DN12719_c0_g2_i6	Glyma.13G321300.40	95.16129032	153	21	356	2376	1983	1	0	2507

TRINITY_DN12719_c0_g2_i6	Glyma.13G321300.41	95.16129032	153	21	356	2376	1983	1	0	2507

TRINITY_DN12719_c0_g2_i6	Glyma.13G321300.28	100	153	21	356	2376	1983	1	0	2507

TRINITY_DN12719_c0_g2_i6	Glyma.13G321300.29	95.88235294	153	21	356	2376	1983	1	0	2507

TRINITY_DN12719_c0_g2_i6	Glyma.13G321300.9	98.66666667	153	21	356	2376	1983	1	0	2507

TRINITY_DN12719_c0_g2_i6	Glyma.13G321300.8	95.95744681	153	21	356	2376	1983	1	0	2507

TRINITY_DN12719_c0_g2_i8	Glyma.13G321300.38	100	153	21	356	2376	1983	1	0	2507

TRINITY_DN12719_c0_g2_i8	Glyma.13G321300.39	100	153	21	356	2376	1983	1	0	2507

TRINITY_DN12719_c0_g2_i8	Glyma.13G321300.26	100	153	21	356	2376	1983	1	0	2507

TRINITY_DN12719_c0_g2_i8	Glyma.13G321300.27	95.63218391	153	21	356	2376	1983	1	0	2507

TRINITY_DN12719_c0_g2_i8	Glyma.13G321300.7	95.41984733	153	21	356	2376	1983	1	0	2507

TRINITY_DN12719_c0_g2_i8	Glyma.13G321300.6	100	153	21	356	2376	1983	1	0	2507

TRINITY_DN12719_c0_g2_i8	Glyma.13G321300.40	97.85932722	153	21	356	2376	1983	1	0	2507

TRINITY_DN12719_c0_g2_i8	Glyma.13G321300.41	97.32142857	153	21	356	2376	1983	1	0	2507

TRINITY_DN12719_c0_g2_i8	Glyma.13G321300.28	96.03174603	153	21	356	2376	1983	1	0	2507

TRINITY_DN12719_c0_g2_i8	Glyma.13G321300.29	97.54098361	153	21	356	2376	1983	1	0	2507

TRINITY_DN12719_c0_g2_i8	Glyma.13G321300.9	100	153	21	356	2376	1983	1	0	2507

TRINITY_DN12719_c0_g2_i8	Glyma.13G321300.8	97.58713137	153	21	356	2376	1983	1	0	2507

TRINITY_DN15841_c0_g1_i5	Glyma.U033300.1	95.97315436	144	3	569	2601	273	2293	0	2880

TRINITY_DN15841_c0_g1_i6	Glyma.U033300.1	99.01960784	144	3	569	2601	273	2293	0	2880

TRINITY_DN15329_c0_g2_i7	Glyma.05G238700.2	96.86520376	137	8	481	2513	2068	68	0	2789

TRINITY_DN14688_c0_g2_i2	Glyma.13G325000.2	95.38461538	115	2	478	2504	2037	5	0	3079

TRINITY_DN2216_c0_g1_i1	Glyma.10G185600.1	98.41269841	356	33	215	2233	1989	1	0	1419

TRINITY_DN16333_c0_g1_i4	Glyma.15G059900.1	97.84482759	218	3	193	2223	2023	5	0	2455

TRINITY_DN16333_c0_g1_i4	Glyma.13G254900.1	97.82608696	217	3	193	2223	2023	5	0	2460

TRINITY_DN3906_c0_g1_i1	Glyma.14G007100.1	98.38709677	196	2	1	2032	1653	3678	0	2628

TRINITY_DN3906_c0_g1_i1	Glyma.14G007100.2	96.62921348	196	2	1	2032	1653	3678	0	2628

TRINITY_DN16040_c0_g1_i2	Glyma.13G027800.1	95.85858586	196	6	610	2638	2034	6	0	2628

TRINITY_DN16040_c0_g1_i3	Glyma.13G027800.1	99.05660377	189	6	610	2638	2034	6	0	2667

TRINITY_DN3906_c0_g1_i1	Glyma.02G305900.2	97.6744186	184	3	1	2032	1650	3666	0	2636

TRINITY_DN3906_c0_g1_i1	Glyma.02G305900.3	97.37609329	184	3	1	2032	1650	3666	0	2636

TRINITY_DN3906_c0_g1_i1	Glyma.02G305900.1	100	184	3	1	2032	1650	3666	0	2636

TRINITY_DN15812_c0_g1_i3	Glyma.04G150900.1	100	167	0	578	2609	2730	699	0	2828

TRINITY_DN15812_c0_g1_i8	Glyma.04G150900.1	95.3125	167	0	495	2526	2730	699	0	2828

TRINITY_DN15812_c0_g1_i3	Glyma.06G213300.3	99.45054945	156	0	578	2609	2730	699	0	2889

TRINITY_DN15812_c0_g1_i3	Glyma.06G213300.1	97.54601227	156	0	578	2609	2730	699	0	2889

TRINITY_DN15812_c0_g1_i3	Glyma.06G213300.4	95.3271028	156	0	578	2609	2730	699	0	2889

TRINITY_DN15812_c0_g1_i3	Glyma.06G213300.2	100	156	0	578	2609	2730	699	0	2889

TRINITY_DN15812_c0_g1_i8	Glyma.06G213300.3	100	156	0	495	2526	2730	699	0	2889

TRINITY_DN15812_c0_g1_i8	Glyma.06G213300.1	100	156	0	495	2526	2730	699	0	2889

TRINITY_DN15812_c0_g1_i8	Glyma.06G213300.4	100	156	0	495	2526	2730	699	0	2889

TRINITY_DN15812_c0_g1_i8	Glyma.06G213300.2	97.14285714	156	0	495	2526	2730	699	0	2889

TRINITY_DN8498_c0_g1_i1	Glyma.05G145200.1	95.34883721	154	18	121	2124	2025	1	0	2673

TRINITY_DN10917_c1_g1_i1	Glyma.04G192200.7	98.1981982	144	2	380	2410	1	2031	0	2942

TRINITY_DN15812_c0_g1_i2	Glyma.04G150900.1	96.1038961	118	0	495	2526	2730	699	0	3099

TRINITY_DN15812_c0_g1_i7	Glyma.04G150900.1	100	118	0	578	2609	2730	699	0	3099

TRINITY_DN15812_c0_g1_i2	Glyma.06G213300.3	96.10866373	107	0	495	2526	2730	699	0	3160

TRINITY_DN15812_c0_g1_i2	Glyma.06G213300.1	96.10894942	107	0	495	2526	2730	699	0	3160

TRINITY_DN15812_c0_g1_i2	Glyma.06G213300.4	97.93103448	107	0	495	2526	2730	699	0	3160

TRINITY_DN15812_c0_g1_i2	Glyma.06G213300.2	96.1038961	107	0	495	2526	2730	699	0	3160

TRINITY_DN15812_c0_g1_i7	Glyma.06G213300.3	100	107	0	578	2609	2730	699	0	3160

TRINITY_DN15812_c0_g1_i7	Glyma.06G213300.1	100	107	0	578	2609	2730	699	0	3160

TRINITY_DN15812_c0_g1_i7	Glyma.06G213300.4	100	107	0	578	2609	2730	699	0	3160

TRINITY_DN15812_c0_g1_i7	Glyma.06G213300.2	100	107	0	578	2609	2730	699	0	3160

TRINITY_DN6860_c0_g1_i1	Glyma.14G049700.1	96.55172414	103	4	483	2508	2026	1	0	3105

TRINITY_DN17837_c0_g1_i1	Glyma.06G116900.2	95.38461538	87	0	1	2032	505	2536	0	3271

TRINITY_DN13371_c0_g1_i4	Glyma.06G102500.6	100	299	20	540	2558	2068	56	0	1901

TRINITY_DN13371_c0_g1_i4	Glyma.06G102500.1	100	299	20	540	2558	2068	56	0	1901

TRINITY_DN13371_c0_g1_i4	Glyma.06G102500.5	100	299	20	540	2558	2068	56	0	1901

TRINITY_DN13371_c0_g1_i4	Glyma.06G102500.3	100	299	20	540	2558	2068	56	0	1901

TRINITY_DN13371_c0_g1_i4	Glyma.06G102500.4	96.23430962	299	20	540	2558	2068	56	0	1901

TRINITY_DN13371_c0_g1_i4	Glyma.06G102500.2	100	299	20	540	2558	2068	56	0	1901

TRINITY_DN13371_c0_g1_i2	Glyma.06G102500.6	99.36708861	242	20	540	2558	2068	56	0	2217

TRINITY_DN13371_c0_g1_i2	Glyma.06G102500.1	98.32635983	242	20	540	2558	2068	56	0	2217

TRINITY_DN13371_c0_g1_i2	Glyma.06G102500.5	96.15384615	242	20	540	2558	2068	56	0	2217

TRINITY_DN13371_c0_g1_i2	Glyma.06G102500.3	97.62611276	242	20	540	2558	2068	56	0	2217

TRINITY_DN13371_c0_g1_i2	Glyma.06G102500.4	100	242	20	540	2558	2068	56	0	2217

TRINITY_DN13371_c0_g1_i2	Glyma.06G102500.2	100	242	20	540	2558	2068	56	0	2217

TRINITY_DN231_c0_g1_i1	Glyma.02G213400.1	95.12195122	224	34	275	2264	1	1987	0	1960

TRINITY_DN6249_c0_g1_i2	Glyma.17G135000.1	100	217	11	119	2138	27	2046	0	2407

TRINITY_DN15068_c0_g1_i4	Glyma.04G080600.1	97.16981132	210	22	275	2277	2525	517	0	2265

TRINITY_DN14790_c0_g1_i1	Glyma.18G077700.1	97.6744186	167	34	329	2313	2208	209	0	2327

TRINITY_DN14790_c0_g1_i1	Glyma.18G077700.4	95.77464789	167	34	329	2313	2208	209	0	2327

TRINITY_DN14790_c0_g1_i2	Glyma.18G077700.1	95.89322382	167	34	329	2313	2208	209	0	2327

TRINITY_DN14790_c0_g1_i2	Glyma.18G077700.4	95.91836735	167	34	329	2313	2208	209	0	2327

TRINITY_DN10834_c0_g1_i1	Glyma.08G228800.2	100	162	1	2	2032	844	2868	0	2815

TRINITY_DN10990_c0_g1_i1	Glyma.11G222800.1	96.1038961	156	31	130	2128	1	2005	0	2512

TRINITY_DN12892_c0_g4_i1	Glyma.11G103100.14	100	143	4	230	2257	34	2052	0	2861

TRINITY_DN12892_c0_g4_i1	Glyma.11G103100.13	100	143	4	230	2257	34	2052	0	2861

TRINITY_DN12892_c0_g4_i5	Glyma.11G103100.14	100	143	4	230	2257	34	2052	0	2861

TRINITY_DN12892_c0_g4_i5	Glyma.11G103100.13	100	143	4	230	2257	34	2052	0	2861

TRINITY_DN12892_c0_g4_i1	Glyma.11G102600.3	100	142	4	230	2257	34	2052	0	2867

TRINITY_DN12892_c0_g4_i5	Glyma.11G102600.3	100	142	4	230	2257	34	2052	0	2867

TRINITY_DN12152_c0_g1_i2	Glyma.03G120000.4	96.90265487	136	3	232	2256	1	2031	0	2959

TRINITY_DN12152_c0_g1_i2	Glyma.03G120000.3	99.80916031	136	3	232	2256	1	2031	0	2959

TRINITY_DN12152_c0_g1_i2	Glyma.03G120000.2	100	136	3	232	2256	1	2031	0	2959

TRINITY_DN12152_c0_g1_i2	Glyma.03G120000.1	100	136	3	232	2256	1	2031	0	2959

TRINITY_DN13494_c0_g1_i1	Glyma.03G250400.1	100	134	2	468	2495	2031	1	0	2988

TRINITY_DN16333_c0_g1_i1	Glyma.07G184300.1	98.31932773	130	2	191	2221	2034	7	0	3011

TRINITY_DN12152_c0_g1_i2	Glyma.19G124500.1	96.90189329	130	3	232	2256	1	2031	0	2992

TRINITY_DN16333_c0_g1_i1	Glyma.08G065100.1	98.30508475	127	2	191	2221	2034	7	0	3027

TRINITY_DN16382_c0_g1_i7	Glyma.02G228700.1	98.91598916	123	0	3	2033	193	2223	0	3070

TRINITY_DN9956_c0_g1_i1	Glyma.12G020400.2	97.26027397	119	3	173	2197	60	2087	0	3033

TRINITY_DN9956_c0_g1_i1	Glyma.12G020400.1	97.22222222	119	3	173	2197	60	2087	0	3033

TRINITY_DN9956_c0_g1_i1	Glyma.12G020400.3	97.24770642	119	3	173	2197	60	2087	0	3033

TRINITY_DN15750_c1_g1_i7	Glyma.07G010400.1	100	459	37	587	2589	2156	148	0	896

TRINITY_DN5596_c0_g1_i1	Glyma.17G249900.1	97.5	397	47	1	1966	3496	1498	0	935

TRINITY_DN13639_c0_g1_i2	Glyma.12G085600.1	97.46835443	257	20	320	2339	2050	40	0	2137

TRINITY_DN13639_c0_g1_i2	Glyma.12G085600.2	98.30508475	257	20	320	2339	2050	40	0	2137

TRINITY_DN16164_c1_g1_i4	Glyma.13G037600.1	100	196	19	298	2287	768	2787	0	2340

TRINITY_DN16164_c1_g1_i6	Glyma.13G037600.1	96.92307692	192	19	298	2287	768	2787	0	2362

TRINITY_DN16040_c0_g1_i1	Glyma.13G027800.1	97.85407725	178	2	356	2384	2034	6	0	2750

TRINITY_DN16040_c0_g1_i4	Glyma.13G027800.1	95.74468085	171	2	356	2384	2034	6	0	2789

TRINITY_DN15683_c0_g2_i4	Glyma.07G005200.2	96.11650485	159	15	82	2087	1	2024	0	2675

TRINITY_DN15683_c0_g2_i4	Glyma.07G005200.1	96.11829945	159	15	82	2087	1	2024	0	2675

TRINITY_DN15565_c0_g1_i1	Glyma.09G044300.3	98.67549669	150	12	613	2612	9	2026	0	2647

TRINITY_DN14503_c0_g1_i2	Glyma.10G294200.1	100	147	19	376	2388	2019	1	0	2754

TRINITY_DN12892_c0_g4_i1	Glyma.11G103100.9	100	143	4	230	2256	34	2051	0	2859

TRINITY_DN12892_c0_g4_i1	Glyma.11G103100.10	100	143	4	230	2256	34	2051	0	2859

TRINITY_DN12892_c0_g4_i5	Glyma.11G103100.9	100	143	4	230	2256	34	2051	0	2859

TRINITY_DN12892_c0_g4_i5	Glyma.11G103100.10	100	143	4	230	2256	34	2051	0	2859

TRINITY_DN11114_c0_g1_i1	Glyma.19G000400.3	97.84946237	108	3	762	2782	2027	1	0	3073

TRINITY_DN15464_c0_g1_i3	Glyma.17G083300.1	100	101	5	187	2210	1	1986	0	2867

TRINITY_DN15464_c0_g1_i3	Glyma.17G083300.2	97.08029197	101	5	187	2210	1	1986	0	2867

TRINITY_DN15750_c1_g1_i6	Glyma.07G010400.1	96.15384615	474	35	587	2589	2156	148	0	824

TRINITY_DN12997_c0_g1_i2	Glyma.13G032500.10	100	355	41	390	2376	2013	3	0	1393

TRINITY_DN16396_c2_g1_i5	Glyma.12G062100.1	97.24770642	333	36	249	2252	40	2031	0	1502

TRINITY_DN16396_c2_g1_i5	Glyma.12G062100.4	97.24137931	333	36	249	2252	40	2031	0	1502

TRINITY_DN13471_c0_g2_i1	Glyma.07G053500.3	100	250	10	211	2236	2017	1	0	2265

TRINITY_DN13471_c0_g2_i1	Glyma.07G053500.2	100	250	11	211	2236	2014	1	0	2246

TRINITY_DN15242_c0_g1_i1	Glyma.10G008700.3	100	249	11	1612	3634	2020	1	0	2270

TRINITY_DN16069_c0_g1_i2	Glyma.05G191600.1	98.51973684	229	11	2625	4631	2542	4563	0	2290

TRINITY_DN15068_c0_g1_i4	Glyma.06G082300.1	97.16981132	212	21	275	2277	2531	517	0	2314

TRINITY_DN16069_c0_g1_i1	Glyma.05G191600.1	100	200	11	2273	4279	2542	4563	0	2451

TRINITY_DN15683_c0_g2_i4	Glyma.08G205700.1	96.11872146	187	10	82	2087	1	2024	0	2531

TRINITY_DN11875_c0_g2_i2	Glyma.05G148300.1	95.3125	156	9	1091	3101	927	2952	0	2747

TRINITY_DN11875_c0_g2_i2	Glyma.05G148300.3	95.29085873	155	16	1091	3101	927	2937	0	2656

TRINITY_DN11875_c0_g2_i2	Glyma.05G148300.2	95.29411765	154	15	1091	3101	927	2934	0	2641

TRINITY_DN15565_c0_g1_i1	Glyma.09G044300.2	98.57142857	150	12	614	2612	40	2056	0	2645

TRINITY_DN9956_c0_g1_i1	Glyma.11G093900.1	97.25557461	119	6	188	2197	72	2099	0	2959

TRINITY_DN0_c0_g3_i1	Glyma.04G210700.3	96.47058824	111	3	2	2026	2033	6	0	3099

TRINITY_DN0_c0_g3_i1	Glyma.04G210700.2	96.47058824	111	3	2	2026	2033	6	0	3099

TRINITY_DN0_c0_g3_i1	Glyma.04G210700.1	96.47058824	111	3	2	2026	2033	6	0	3099

TRINITY_DN0_c0_g1_i1	Glyma.04G210700.3	98.50746269	108	3	2	2026	2033	6	0	3116

TRINITY_DN0_c0_g1_i1	Glyma.04G210700.2	100	108	3	2	2026	2033	6	0	3116

TRINITY_DN0_c0_g1_i1	Glyma.04G210700.1	97.07317073	108	3	2	2026	2033	6	0	3116

TRINITY_DN5372_c0_g1_i1	Glyma.14G182100.1	100	275	11	226	2245	46	2068	0	2137

TRINITY_DN13371_c0_g1_i2	Glyma.04G100700.1	100	235	16	540	2558	2068	56	0	2289

TRINITY_DN13371_c0_g1_i2	Glyma.04G100700.2	98.57142857	235	16	540	2558	2068	56	0	2289

TRINITY_DN15936_c0_g2_i1	Glyma.06G285400.1	97.265625	175	9	89	2112	8	2028	0	2704

TRINITY_DN13519_c0_g1_i1	Glyma.03G157900.1	96.68508287	169	15	175	2169	1	2007	0	2460

TRINITY_DN14790_c0_g1_i1	Glyma.18G077700.2	97.67054908	167	33	329	2313	2205	209	0	2342

TRINITY_DN14790_c0_g1_i1	Glyma.18G077700.3	97.65886288	167	33	329	2313	2205	209	0	2342

TRINITY_DN14790_c0_g1_i2	Glyma.18G077700.2	95.93495935	167	33	329	2313	2205	209	0	2342

TRINITY_DN14790_c0_g1_i2	Glyma.18G077700.3	95.93495935	167	33	329	2313	2205	209	0	2342

TRINITY_DN12659_c0_g1_i1	Glyma.U025600.10	98.4962406	154	6	258	2266	8	2031	0	2743

TRINITY_DN12659_c0_g1_i1	Glyma.U025600.11	98.50746269	154	6	258	2266	8	2031	0	2743

TRINITY_DN4792_c0_g1_i1	Glyma.08G252900.1	98.01980198	144	4	149	2176	1	2019	0	2889

TRINITY_DN15683_c0_g2_i3	Glyma.08G205700.1	100	138	6	82	2088	1	2025	0	2826

TRINITY_DN11450_c0_g1_i1	Glyma.09G258100.4	95.98930481	131	3	59	2076	1	2028	0	2955

TRINITY_DN11450_c0_g1_i1	Glyma.09G258100.2	98.33333333	131	3	59	2076	1	2028	0	2955

TRINITY_DN11450_c0_g1_i1	Glyma.09G258100.1	95.58823529	131	3	59	2076	1	2028	0	2955

TRINITY_DN11450_c0_g2_i1	Glyma.09G258100.4	98.47328244	131	3	59	2076	1	2028	0	2955

TRINITY_DN11450_c0_g2_i1	Glyma.09G258100.2	100	131	3	59	2076	1	2028	0	2955

TRINITY_DN11450_c0_g2_i1	Glyma.09G258100.1	98.27586207	131	3	59	2076	1	2028	0	2955

TRINITY_DN4792_c0_g1_i1	Glyma.18G275500.1	96.34146341	125	2	149	2176	1	2022	0	3014

TRINITY_DN11893_c0_g1_i1	Glyma.02G200700.9	98.00399202	124	2	619	2643	54	2081	0	3038

TRINITY_DN11893_c0_g1_i1	Glyma.02G200700.7	98	124	2	619	2643	54	2081	0	3038

TRINITY_DN11893_c0_g1_i1	Glyma.02G200700.4	98	124	2	619	2643	54	2081	0	3038

TRINITY_DN15683_c0_g2_i3	Glyma.07G005200.2	97.57412399	117	9	82	2088	1	2025	0	2942

TRINITY_DN15683_c0_g2_i3	Glyma.07G005200.1	95.91836735	117	9	82	2088	1	2025	0	2942

TRINITY_DN16396_c2_g1_i3	Glyma.12G062100.1	97.24770642	336	32	249	2252	40	2031	0	1507

TRINITY_DN16396_c2_g1_i3	Glyma.12G062100.4	97.25	336	32	249	2252	40	2031	0	1507

TRINITY_DN13371_c0_g1_i4	Glyma.04G100700.1	96.92307692	298	14	540	2558	2068	56	0	1951

TRINITY_DN13371_c0_g1_i4	Glyma.04G100700.2	96.92307692	298	14	540	2558	2068	56	0	1951

TRINITY_DN5871_c0_g1_i1	Glyma.16G212800.1	98.73417722	229	8	993	3015	2825	809	0	2385

TRINITY_DN5871_c0_g1_i1	Glyma.16G212800.3	98.73417722	229	8	993	3015	2813	797	0	2385

TRINITY_DN5871_c0_g1_i1	Glyma.16G212800.4	99.13793103	229	8	993	3015	2552	536	0	2385

TRINITY_DN5871_c0_g1_i1	Glyma.16G212800.2	98.73417722	229	8	993	3015	2552	536	0	2385

TRINITY_DN14389_c1_g2_i2	Glyma.07G134800.1	100	223	26	328	2349	25	1995	0	2113

TRINITY_DN7939_c0_g1_i1	Glyma.16G128300.3	100	155	2	598	2621	59	2082	0	2846

TRINITY_DN16164_c1_g1_i7	Glyma.13G037600.1	97.26027397	152	13	298	2293	768	2787	0	2656

TRINITY_DN16164_c1_g1_i2	Glyma.13G037600.1	100	148	13	298	2293	768	2787	0	2678

TRINITY_DN7939_c0_g1_i2	Glyma.16G128300.3	100	148	13	598	2594	59	2082	0	2710

TRINITY_DN14779_c0_g1_i2	Glyma.09G114500.3	95.45454545	115	1	101	2127	2024	1	0	3086

TRINITY_DN14779_c0_g1_i4	Glyma.09G114500.3	98.27586207	115	1	101	2127	2024	1	0	3086

TRINITY_DN15464_c0_g1_i3	Glyma.05G016700.1	99.0430622	104	4	187	2210	1	1983	0	2863

TRINITY_DN15464_c0_g1_i3	Glyma.05G016700.6	99.17808219	104	4	187	2210	1	1983	0	2863

TRINITY_DN12997_c0_g1_i2	Glyma.13G032500.9	98.64864865	355	40	390	2376	2010	3	0	1406

TRINITY_DN16181_c0_g1_i2	Glyma.17G248400.5	96.04810997	239	4	103	2124	2025	1	0	2385

TRINITY_DN14525_c0_g1_i1	Glyma.11G071000.1	98.33333333	233	16	6	2025	378	2376	0	2237

TRINITY_DN14503_c0_g1_i1	Glyma.18G300100.1	100	227	8	376	2397	2019	1	0	2412

TRINITY_DN15415_c0_g1_i3	Glyma.02G038500.2	100	196	2	118	2140	135	2160	0	2636

TRINITY_DN13515_c0_g1_i2	Glyma.16G005800.1	100	140	14	257	2278	1	2007	0	2817

TRINITY_DN8335_c0_g1_i1	Glyma.05G222700.1	95.40229885	122	4	385	2404	1	2023	0	3007

TRINITY_DN0_c0_g3_i1	Glyma.06G155300.2	96.47058824	111	0	1	2026	2031	6	0	3127

TRINITY_DN0_c0_g3_i1	Glyma.06G155300.1	96.47058824	111	0	1	2026	2031	6	0	3127

TRINITY_DN0_c0_g1_i1	Glyma.06G155300.2	95.34883721	108	0	1	2026	2031	6	0	3144

TRINITY_DN0_c0_g1_i1	Glyma.06G155300.1	100	108	0	1	2026	2031	6	0	3144

TRINITY_DN13639_c0_g1_i2	Glyma.11G189000.1	97.6635514	248	33	320	2323	2056	56	0	2074

TRINITY_DN16164_c1_g1_i8	Glyma.14G119800.1	98	214	23	47	2048	738	2748	0	2314

TRINITY_DN16164_c1_g1_i8	Glyma.14G119800.2	98.28571429	214	23	47	2048	738	2748	0	2314

TRINITY_DN16164_c1_g1_i5	Glyma.14G119800.1	100	210	23	47	2048	738	2748	0	2337

TRINITY_DN16164_c1_g1_i5	Glyma.14G119800.2	100	210	23	47	2048	738	2748	0	2337

TRINITY_DN231_c0_g1_i1	Glyma.14G181100.1	95.12195122	210	28	275	2264	1	1990	0	2124

TRINITY_DN37038_c0_g1_i1	Glyma.03G165600.1	98.11320755	207	5	278	2299	2037	16	0	2555

TRINITY_DN14298_c0_g2_i2	Glyma.08G060200.2	96.20901639	195	8	781	2803	1131	3144	0	2575

TRINITY_DN14298_c0_g2_i2	Glyma.08G060200.1	96.20253165	195	8	781	2803	1131	3144	0	2575

TRINITY_DN8852_c0_g1_i1	Glyma.12G214200.1	99.34102142	186	1	215	2236	219	2243	0	2689

TRINITY_DN15936_c0_g2_i1	Glyma.12G120300.1	98.24561404	175	7	89	2112	11	2028	0	2719

TRINITY_DN12659_c0_g1_i1	Glyma.U025600.8	98.50746269	154	5	258	2266	8	2028	0	2758

TRINITY_DN12659_c0_g1_i1	Glyma.U025600.9	98.48484848	154	5	258	2266	8	2028	0	2758

TRINITY_DN16149_c1_g1_i2	Glyma.17G191000.1	95.40229885	134	10	482	2467	2019	1	0	2706

TRINITY_DN14779_c0_g1_i1	Glyma.09G114500.3	98.8372093	75	0	204	2228	2025	1	0	3325

TRINITY_DN14779_c0_g1_i3	Glyma.09G114500.3	95.42253521	75	0	204	2228	2025	1	0	3325

TRINITY_DN16360_c0_g8_i3	Glyma.17G254100.3	99.35064935	441	34	274	2278	78	2079	0	1029

TRINITY_DN16360_c0_g8_i6	Glyma.17G254100.3	100	441	34	274	2278	78	2079	0	1029

TRINITY_DN13197_c0_g1_i1	Glyma.03G215600.1	98.19494585	344	18	1413	3430	947	2952	0	1677

TRINITY_DN14881_c0_g2_i1	Glyma.01G223400.7	96.3622291	320	37	430	2429	1	1973	0	1480

TRINITY_DN14881_c0_g2_i1	Glyma.01G223400.6	96.36363636	320	37	430	2429	1	1973	0	1480

TRINITY_DN14881_c0_g2_i1	Glyma.01G223400.1	96.36363636	320	37	430	2429	1	1973	0	1480

TRINITY_DN14881_c0_g2_i1	Glyma.01G223400.4	98.14814815	320	37	430	2429	1	1973	0	1480

TRINITY_DN14881_c0_g2_i1	Glyma.01G223400.2	99.22779923	320	37	430	2429	1	1973	0	1480

TRINITY_DN14881_c0_g2_i1	Glyma.01G223400.3	100	320	37	430	2429	1	1973	0	1480

TRINITY_DN14881_c0_g2_i1	Glyma.01G223400.5	97.48427673	320	37	430	2429	1	1973	0	1480

TRINITY_DN14881_c0_g2_i2	Glyma.01G223400.7	97.5	320	37	462	2461	1	1973	0	1480

TRINITY_DN14881_c0_g2_i2	Glyma.01G223400.6	96.62650602	320	37	462	2461	1	1973	0	1480

TRINITY_DN14881_c0_g2_i2	Glyma.01G223400.1	98.4496124	320	37	462	2461	1	1973	0	1480

TRINITY_DN14881_c0_g2_i2	Glyma.01G223400.4	99.01960784	320	37	462	2461	1	1973	0	1480

TRINITY_DN14881_c0_g2_i2	Glyma.01G223400.2	98.38709677	320	37	462	2461	1	1973	0	1480

TRINITY_DN14881_c0_g2_i2	Glyma.01G223400.3	96.77419355	320	37	462	2461	1	1973	0	1480

TRINITY_DN14881_c0_g2_i2	Glyma.01G223400.5	100	320	37	462	2461	1	1973	0	1480

TRINITY_DN14881_c0_g2_i3	Glyma.01G223400.7	97.5	320	37	1566	3565	1	1973	0	1480

TRINITY_DN14881_c0_g2_i3	Glyma.01G223400.6	100	320	37	1566	3565	1	1973	0	1480

TRINITY_DN14881_c0_g2_i3	Glyma.01G223400.1	96	320	37	1566	3565	1	1973	0	1480

TRINITY_DN14881_c0_g2_i3	Glyma.01G223400.4	96	320	37	1566	3565	1	1973	0	1480

TRINITY_DN14881_c0_g2_i3	Glyma.01G223400.2	96	320	37	1566	3565	1	1973	0	1480

TRINITY_DN14881_c0_g2_i3	Glyma.01G223400.3	100	320	37	1566	3565	1	1973	0	1480

TRINITY_DN14881_c0_g2_i3	Glyma.01G223400.5	97.56097561	320	37	1566	3565	1	1973	0	1480

TRINITY_DN15778_c0_g1_i1	Glyma.07G144500.1	99.24242424	281	18	1032	3049	2000	1	0	1988

TRINITY_DN15385_c0_g1_i4	Glyma.18G248200.2	100	273	40	213	2183	2190	184	0	1773

TRINITY_DN15385_c0_g1_i4	Glyma.18G248200.1	95.85798817	273	40	213	2183	2190	184	0	1773

TRINITY_DN16360_c0_g4_i7	Glyma.15G023800.1	100	234	8	398	2417	64	2083	0	2390

TRINITY_DN14503_c0_g1_i1	Glyma.08G361700.1	100	227	10	378	2397	2017	1	0	2409

TRINITY_DN16333_c0_g1_i2	Glyma.15G059900.1	97.84313725	223	4	193	2215	2023	4	0	2470

TRINITY_DN16333_c0_g1_i2	Glyma.13G254900.1	97.83653846	220	4	193	2215	2023	4	0	2486

TRINITY_DN37038_c0_g1_i1	Glyma.19G166900.1	95.83333333	198	9	278	2299	2028	16	0	2556

TRINITY_DN16164_c1_g1_i3	Glyma.14G119800.1	99.6875	167	14	47	2054	738	2748	0	2625

TRINITY_DN16164_c1_g1_i3	Glyma.14G119800.2	99.05660377	167	14	47	2054	738	2748	0	2625

TRINITY_DN11473_c0_g1_i1	Glyma.18G288300.1	96.20938628	163	7	157	2177	3018	5033	0	2763

TRINITY_DN16164_c1_g1_i1	Glyma.14G119800.1	98.80952381	163	14	47	2054	738	2748	0	2647

TRINITY_DN16164_c1_g1_i1	Glyma.14G119800.2	100	163	14	47	2054	738	2748	0	2647

TRINITY_DN11393_c0_g1_i1	Glyma.17G129100.3	95.42253521	117	0	588	2611	2024	1	0	3090

TRINITY_DN16245_c0_g5_i1	Glyma.08G268900.1	98.7804878	456	50	477	2468	3470	1476	0	828

TRINITY_DN16360_c0_g8_i5	Glyma.17G254100.3	99.34640523	441	34	274	2277	78	2078	0	1027

TRINITY_DN16360_c0_g8_i5	Glyma.17G254100.1	99.35483871	441	34	274	2277	78	2078	0	1027

TRINITY_DN9723_c0_g1_i1	Glyma.03G237300.1	100	377	4	137	2157	2565	545	0	1622

TRINITY_DN4336_c0_g1_i1	Glyma.16G108900.2	97.86096257	374	35	172	2164	188	2192	0	1354

TRINITY_DN13639_c0_g1_i1	Glyma.12G085600.1	100	237	20	1	2013	2043	40	0	2235

TRINITY_DN13639_c0_g1_i1	Glyma.12G085600.2	96.71532847	237	20	1	2013	2043	40	0	2235

TRINITY_DN16360_c0_g4_i7	Glyma.13G350700.1	100	230	6	398	2417	67	2086	0	2423

TRINITY_DN16333_c0_g1_i1	Glyma.15G059900.1	97.82608696	225	4	193	2214	2023	5	0	2457

TRINITY_DN16333_c0_g1_i1	Glyma.13G254900.1	97.82608696	222	4	193	2214	2023	5	0	2473

TRINITY_DN19817_c0_g1_i1	Glyma.05G189800.1	98.27586207	198	11	283	2285	1	2021	0	2497

TRINITY_DN14298_c0_g2_i2	Glyma.07G189100.1	96.20253165	188	6	781	2803	1083	3093	0	2617

TRINITY_DN12916_c0_g1_i1	Glyma.05G108600.1	98.41269841	151	22	312	2306	1	1998	0	2556

TRINITY_DN14503_c0_g1_i2	Glyma.20G244000.1	100	147	14	376	2388	2010	1	0	2772

TRINITY_DN15807_c0_g2_i1	Glyma.18G299500.1	100	146	9	62	2080	2013	1	0	2837

TRINITY_DN15807_c0_g2_i3	Glyma.18G299500.1	95.23809524	146	9	62	2080	2013	1	0	2837

TRINITY_DN16254_c0_g1_i7	Glyma.09G061200.4	99.16666667	143	2	91	2110	2949	930	0	2905

TRINITY_DN16254_c0_g1_i7	Glyma.09G061200.3	99.16666667	143	2	91	2110	2949	930	0	2905

TRINITY_DN16254_c0_g1_i7	Glyma.09G061200.1	97.69736842	143	2	91	2110	2949	930	0	2905

TRINITY_DN16254_c0_g1_i7	Glyma.09G061200.2	97.70114943	143	2	91	2110	2949	930	0	2905

TRINITY_DN16254_c0_g1_i7	Glyma.09G061200.5	96.93251534	143	2	91	2110	2949	930	0	2905

TRINITY_DN14245_c0_g2_i1	Glyma.13G181700.2	95.66197183	125	4	438	2439	1	2020	0	2889

TRINITY_DN16360_c0_g8_i5	Glyma.14G070600.1	99.35897436	443	33	274	2277	78	2078	0	1027

TRINITY_DN10348_c0_g1_i1	Glyma.02G262300.1	97.82608696	368	25	206	2202	2819	815	0	1424

TRINITY_DN8872_c0_g1_i1	Glyma.15G140000.5	98.11320755	317	11	68	2073	150	2163	0	1823

TRINITY_DN8872_c0_g1_i1	Glyma.15G140000.4	96.15384615	317	11	68	2073	150	2163	0	1823

TRINITY_DN8872_c0_g1_i2	Glyma.15G140000.5	96.49122807	317	11	68	2073	150	2163	0	1823

TRINITY_DN8872_c0_g1_i2	Glyma.15G140000.4	96.49122807	317	11	68	2073	150	2163	0	1823

TRINITY_DN16207_c0_g1_i2	Glyma.20G105400.1	98.86363636	206	5	274	2293	2833	817	0	2547

TRINITY_DN1847_c0_g1_i1	Glyma.20G100400.2	100	176	6	7	2013	38	2056	0	2643

TRINITY_DN15759_c0_g2_i2	Glyma.04G231900.2	96.38554217	168	2	540	2560	2021	1	0	2791

TRINITY_DN15759_c0_g2_i2	Glyma.04G231900.1	100	168	2	540	2560	2021	1	0	2791

TRINITY_DN15759_c0_g2_i5	Glyma.04G231900.2	97	168	2	540	2560	2021	1	0	2791

TRINITY_DN15759_c0_g2_i5	Glyma.04G231900.1	97	168	2	540	2560	2021	1	0	2791

TRINITY_DN7121_c0_g2_i1	Glyma.13G319300.1	95.95375723	161	23	278	2298	2583	605	0	2558

TRINITY_DN15759_c0_g2_i2	Glyma.06G133000.3	97.69230769	158	2	540	2560	2021	1	0	2846

TRINITY_DN15759_c0_g2_i2	Glyma.06G133000.2	95.10086455	158	2	540	2560	2021	1	0	2846

TRINITY_DN15759_c0_g2_i2	Glyma.06G133000.1	95.98765432	158	2	540	2560	2021	1	0	2846

TRINITY_DN15759_c0_g2_i5	Glyma.06G133000.3	97.8021978	158	2	540	2560	2021	1	0	2846

TRINITY_DN15759_c0_g2_i5	Glyma.06G133000.2	97.5	158	2	540	2560	2021	1	0	2846

TRINITY_DN15759_c0_g2_i5	Glyma.06G133000.1	97	158	2	540	2560	2021	1	0	2846

TRINITY_DN1021_c0_g1_i1	Glyma.15G209800.4	100	119	4	462	2483	2016	1	0	3037

TRINITY_DN1021_c0_g1_i1	Glyma.15G209800.2	100	119	4	462	2483	2016	1	0	3037

TRINITY_DN1021_c0_g1_i1	Glyma.15G209800.3	100	119	4	462	2483	2016	1	0	3037

TRINITY_DN1021_c0_g1_i1	Glyma.15G209800.1	97.36842105	119	4	462	2483	2016	1	0	3037

TRINITY_DN1021_c0_g1_i1	Glyma.09G099400.4	98.18181818	116	1	462	2483	2019	1	0	3072

TRINITY_DN1021_c0_g1_i1	Glyma.09G099400.3	98.14814815	116	1	462	2483	2019	1	0	3072

TRINITY_DN1021_c0_g1_i1	Glyma.09G099400.1	95.35315985	116	1	462	2483	2019	1	0	3072

TRINITY_DN1021_c0_g1_i1	Glyma.09G099400.2	96.62162162	116	1	462	2483	2019	1	0	3072

TRINITY_DN14688_c0_g2_i1	Glyma.13G325000.1	100	116	7	241	2247	2022	1	0	2994

TRINITY_DN11782_c0_g1_i2	Glyma.01G009300.1	95.89041096	96	1	78	2090	2022	1	0	3144

TRINITY_DN10348_c0_g1_i1	Glyma.14G056600.1	98.11320755	362	32	206	2203	2039	43	0	1423

TRINITY_DN14043_c0_g2_i3	Glyma.15G096200.19	100	333	26	251	2230	1	2007	0	1531

TRINITY_DN14043_c0_g2_i3	Glyma.15G096200.20	97.05882353	333	26	251	2230	1	2007	0	1531

TRINITY_DN14043_c0_g2_i3	Glyma.15G096200.3	100	333	26	251	2230	1	2007	0	1531

TRINITY_DN16245_c0_g3_i2	Glyma.02G129500.1	100	291	6	262	2280	3596	1583	0	2061

TRINITY_DN16245_c0_g3_i2	Glyma.02G129500.2	100	291	6	262	2280	3221	1208	0	2061

TRINITY_DN16245_c0_g3_i2	Glyma.01G092900.1	95	283	5	262	2280	3596	1583	0	2106

TRINITY_DN16245_c0_g3_i2	Glyma.01G092900.2	100	283	5	262	2280	3221	1208	0	2106

TRINITY_DN16245_c0_g3_i2	Glyma.01G092900.3	100	283	5	262	2280	3221	1208	0	2106

TRINITY_DN14525_c0_g1_i1	Glyma.01G172200.6	98.18181818	254	2	6	2025	399	2418	0	2313

TRINITY_DN14525_c0_g1_i1	Glyma.01G172200.3	100	254	2	6	2025	399	2418	0	2313

TRINITY_DN14525_c0_g1_i1	Glyma.01G172200.1	98.41269841	254	2	6	2025	399	2418	0	2313

TRINITY_DN14525_c0_g1_i1	Glyma.01G172200.4	98.31932773	254	2	6	2025	399	2418	0	2313

TRINITY_DN14525_c0_g1_i1	Glyma.01G172200.2	98.26086957	254	2	6	2025	399	2418	0	2313

TRINITY_DN14525_c0_g1_i1	Glyma.01G172200.5	95.96774194	254	2	6	2025	399	2418	0	2313

TRINITY_DN16207_c0_g1_i2	Glyma.10G284300.1	97.71573604	197	4	274	2293	2782	766	0	2608

TRINITY_DN12858_c0_g1_i1	Glyma.06G146900.1	100	136	16	476	2495	1981	1	0	2713

TRINITY_DN8191_c0_g2_i2	Glyma.03G086000.5	95.52238806	135	1	1	2018	103	2123	0	2964

TRINITY_DN9106_c0_g1_i1	Glyma.16G023600.2	97.61904762	126	32	108	2079	1	2004	0	2608

TRINITY_DN16356_c0_g1_i1	Glyma.05G219600.1	100	112	4	101	2115	87	2106	0	3066

TRINITY_DN15242_c0_g1_i2	Glyma.10G008700.3	95.01525941	108	3	1621	3637	2020	1	0	3101

TRINITY_DN14043_c0_g2_i3	Glyma.15G096200.21	100	334	22	251	2230	1	2016	0	1596

TRINITY_DN15872_c0_g1_i1	Glyma.11G106800.1	100	325	23	312	2306	2313	304	0	1703

TRINITY_DN15872_c0_g1_i1	Glyma.12G031800.1	100	311	21	312	2306	2322	313	0	1781

TRINITY_DN15897_c0_g2_i3	Glyma.08G308600.1	98.82352941	224	28	209	2220	3113	1138	0	2154

TRINITY_DN16232_c0_g1_i2	Glyma.15G000600.1	98.7804878	221	27	69	2061	1995	3	0	2158

TRINITY_DN16396_c1_g1_i1	Glyma.13G337400.1	98.33333333	218	28	239	2242	266	2230	0	2063

TRINITY_DN16396_c1_g1_i2	Glyma.13G337400.1	98.33333333	218	28	239	2242	266	2230	0	2063

TRINITY_DN9565_c0_g1_i1	Glyma.05G073800.1	100	165	16	434	2430	2014	4	0	2610

TRINITY_DN16254_c0_g1_i7	Glyma.15G167500.1	96.93251534	148	1	91	2110	2937	921	0	2891

TRINITY_DN16254_c0_g1_i7	Glyma.15G167500.3	98.84057971	148	1	91	2110	2937	921	0	2891

TRINITY_DN16254_c0_g1_i7	Glyma.15G167500.2	96.70781893	148	1	91	2110	2937	921	0	2891

TRINITY_DN16254_c0_g1_i7	Glyma.15G167500.4	100	148	1	91	2110	2937	921	0	2891

TRINITY_DN12858_c0_g1_i1	Glyma.04G218900.1	100	147	6	476	2495	1996	1	0	2761

TRINITY_DN14378_c0_g1_i1	Glyma.14G082600.1	98.82352941	256	20	968	2955	594	2608	0	2084

TRINITY_DN14378_c0_g1_i1	Glyma.14G082600.2	100	256	20	968	2955	594	2608	0	2084

TRINITY_DN15768_c0_g1_i4	Glyma.10G048800.2	96	213	5	819	2812	2	2016	0	2361

TRINITY_DN16149_c1_g1_i1	Glyma.06G082800.1	98.10606061	208	7	407	2415	2000	1	0	2388

TRINITY_DN16149_c1_g1_i1	Glyma.04G081100.1	95.54794521	196	12	407	2415	2000	1	0	2455

TRINITY_DN16235_c0_g1_i1	Glyma.04G101000.3	100	174	5	795	2807	2013	1	0	2687

TRINITY_DN16235_c0_g1_i1	Glyma.04G101000.2	97.77777778	174	5	795	2807	2013	1	0	2687

TRINITY_DN16235_c0_g1_i3	Glyma.04G101000.3	98.4	174	5	795	2807	2013	1	0	2687

TRINITY_DN16235_c0_g1_i3	Glyma.04G101000.2	100	174	5	795	2807	2013	1	0	2687

TRINITY_DN16235_c0_g1_i4	Glyma.04G101000.3	98.80952381	173	5	794	2806	2013	1	0	2693

TRINITY_DN16235_c0_g1_i4	Glyma.04G101000.2	98.63636364	173	5	794	2806	2013	1	0	2693

TRINITY_DN16235_c0_g1_i1	Glyma.06G102700.1	100	165	3	795	2807	2019	1	0	2776

TRINITY_DN16235_c0_g1_i3	Glyma.06G102700.1	100	165	3	795	2807	2019	1	0	2776

TRINITY_DN16235_c0_g1_i4	Glyma.06G102700.1	100	164	3	794	2806	2019	1	0	2782

TRINITY_DN16983_c0_g1_i1	Glyma.02G102600.1	99.11504425	147	1	242	2257	1	2019	0	2894

TRINITY_DN16427_c0_g3_i3	Glyma.04G134800.1	95.23809524	142	2	1	2018	2125	108	0	2929

TRINITY_DN14268_c0_g1_i1	Glyma.19G007300.1	100	141	2	681	2696	1	2016	0	2909

TRINITY_DN14268_c0_g1_i1	Glyma.19G007300.2	100	141	2	681	2696	1	2016	0	2909

TRINITY_DN14268_c0_g1_i1	Glyma.19G007300.3	100	141	2	681	2696	1	2016	0	2909

TRINITY_DN14268_c0_g1_i2	Glyma.19G007300.1	100	141	2	670	2685	1	2016	0	2909

TRINITY_DN14268_c0_g1_i2	Glyma.19G007300.2	100	141	2	670	2685	1	2016	0	2909

TRINITY_DN14268_c0_g1_i2	Glyma.19G007300.3	100	141	2	670	2685	1	2016	0	2909

TRINITY_DN14268_c0_g1_i1	Glyma.05G006700.2	100	141	5	681	2696	1	2010	0	2870

TRINITY_DN14268_c0_g1_i2	Glyma.05G006700.2	100	141	5	670	2685	1	2010	0	2870

TRINITY_DN15329_c0_g2_i4	Glyma.05G238700.2	97.02380952	138	1	481	2499	2068	68	0	2848

TRINITY_DN14542_c0_g2_i2	Glyma.08G187100.1	96.55172414	135	4	189	2174	1	2019	0	2767

TRINITY_DN8121_c0_g1_i1	Glyma.17G202300.1	96.6442953	129	11	125	2110	1	2016	0	2782

TRINITY_DN12798_c0_g2_i1	Glyma.12G173200.4	97.0505618	115	1	592	2607	2019	1	0	3072

TRINITY_DN12798_c0_g2_i1	Glyma.12G173200.3	97.81420765	115	1	592	2607	2019	1	0	3072

TRINITY_DN10705_c0_g1_i1	Glyma.17G244600.2	100	90	0	204	2222	1	2019	0	3230

TRINITY_DN10705_c0_g1_i1	Glyma.17G244600.3	100	90	0	204	2222	1	2019	0	3230

TRINITY_DN16245_c0_g5_i2	Glyma.08G268900.1	100	452	43	477	2468	3470	1476	0	905

TRINITY_DN9638_c0_g1_i2	Glyma.16G181700.2	100	383	36	338	2306	2060	62	0	1166

TRINITY_DN9638_c0_g1_i2	Glyma.16G181700.3	100	383	36	338	2306	2060	62	0	1166

TRINITY_DN16354_c0_g1_i6	Glyma.08G027000.1	97.53914989	347	11	173	2182	1	2013	0	1720

TRINITY_DN14043_c0_g2_i3	Glyma.15G096200.16	100	333	25	251	2230	1	2004	0	1546

TRINITY_DN14043_c0_g2_i3	Glyma.15G096200.17	100	333	25	251	2230	1	2004	0	1546

TRINITY_DN14043_c0_g2_i3	Glyma.15G096200.18	95.03546099	333	25	251	2230	1	2004	0	1546

TRINITY_DN11347_c0_g1_i2	Glyma.03G165400.1	99.16666667	322	21	317	2310	2199	197	0	1690

TRINITY_DN14389_c1_g2_i2	Glyma.18G184500.1	95.93023256	225	25	337	2349	31	1995	0	2106

TRINITY_DN13639_c0_g1_i1	Glyma.11G189000.1	96.71532847	224	33	1	1997	2049	56	0	2194

TRINITY_DN15048_c0_g2_i1	Glyma.15G145200.1	96.59090909	199	10	584	2596	2010	1	0	2540

TRINITY_DN9761_c0_g1_i3	Glyma.10G044200.2	96.55172414	132	13	476	2471	1	1987	0	2652

TRINITY_DN9761_c0_g1_i3	Glyma.10G044200.1	96.55172414	132	13	476	2471	1	1987	0	2652

TRINITY_DN10747_c0_g1_i1	Glyma.08G027700.1	100	112	0	1	2018	914	2931	0	3107

TRINITY_DN16360_c0_g8_i5	Glyma.04G028600.1	99.35691318	452	34	277	2277	87	2081	0	976

TRINITY_DN13654_c0_g1_i1	Glyma.16G015400.3	97.15909091	294	33	347	2337	2006	28	0	1683

TRINITY_DN8761_c0_g1_i1	Glyma.13G336500.1	95.54140127	276	31	245	2239	2019	19	0	1951

TRINITY_DN8761_c0_g1_i1	Glyma.13G336500.4	99.60159363	276	31	245	2239	2016	16	0	1951

TRINITY_DN8761_c0_g1_i1	Glyma.13G336500.3	98.29351536	276	31	245	2239	2016	16	0	1951

TRINITY_DN8761_c0_g1_i1	Glyma.13G336500.2	100	276	31	245	2239	2016	16	0	1951

TRINITY_DN16207_c0_g1_i3	Glyma.10G284300.1	99.38461538	206	4	230	2245	2778	766	0	2551

TRINITY_DN15048_c0_g2_i1	Glyma.09G040000.1	98.24561404	205	6	584	2596	2013	1	0	2538

TRINITY_DN9956_c0_g1_i1	Glyma.11G093900.2	97.25609756	119	5	188	2197	72	2087	0	3014

TRINITY_DN16354_c0_g1_i2	Glyma.08G027000.1	95	326	7	173	2182	1	2013	0	1858

TRINITY_DN11347_c0_g1_i2	Glyma.19G166600.6	100	325	17	317	2310	2193	191	0	1696

TRINITY_DN11347_c0_g1_i2	Glyma.19G166600.1	100	325	17	317	2310	2193	191	0	1696

TRINITY_DN15521_c0_g1_i2	Glyma.12G233000.2	100	315	19	1200	3207	947	2948	0	1836

TRINITY_DN15521_c0_g1_i4	Glyma.12G233000.2	98.10606061	315	19	1104	3111	947	2948	0	1836

TRINITY_DN16415_c1_g1_i15	Glyma.12G173700.1	97.22222222	294	5	1694	3704	2076	63	0	2049

TRINITY_DN16415_c1_g1_i4	Glyma.12G173700.1	95.74468085	294	5	1798	3808	2076	63	0	2049

TRINITY_DN12925_c0_g1_i1	Glyma.01G212200.3	100	279	15	263	2275	1992	1	0	2002

TRINITY_DN16050_c0_g1_i2	Glyma.18G288600.3	100	259	9	772	2780	2803	795	0	2198

TRINITY_DN12925_c0_g1_i1	Glyma.11G029600.1	100	253	12	263	2275	1992	1	0	2146

TRINITY_DN12925_c0_g1_i1	Glyma.11G029600.2	100	252	13	263	2275	1989	1	0	2134

TRINITY_DN16354_c0_g1_i6	Glyma.20G192700.1	97.5308642	206	1	170	2182	31	2046	0	2562

TRINITY_DN16354_c0_g1_i2	Glyma.20G192700.1	95	182	1	170	2182	31	2046	0	2695

TRINITY_DN5598_c0_g1_i1	Glyma.14G012200.2	97.31182796	160	15	1	2010	301	2298	0	2682

TRINITY_DN5598_c0_g1_i1	Glyma.02G302000.1	97.31543624	159	16	1	2010	301	2295	0	2667

TRINITY_DN5598_c0_g1_i1	Glyma.02G302000.2	97.16312057	156	21	1	2010	301	2283	0	2606

TRINITY_DN12659_c0_g1_i1	Glyma.U025600.5	98.48484848	155	5	258	2266	8	2019	0	2793

TRINITY_DN12659_c0_g1_i1	Glyma.U025600.6	98.50746269	155	5	258	2266	8	2019	0	2793

TRINITY_DN12659_c0_g1_i1	Glyma.U025600.7	98.49246231	155	5	258	2266	8	2019	0	2793

TRINITY_DN12719_c0_g2_i1	Glyma.12G179300.2	95.45454545	155	13	399	2403	2002	1	0	2702

TRINITY_DN12719_c0_g2_i1	Glyma.12G179300.5	99.50738916	155	13	399	2403	2002	1	0	2702

TRINITY_DN12719_c0_g2_i1	Glyma.12G179300.4	95.68106312	155	13	399	2403	2002	1	0	2702

TRINITY_DN12719_c0_g2_i1	Glyma.12G179300.3	95.68181818	155	13	399	2403	2002	1	0	2702

TRINITY_DN12719_c0_g2_i1	Glyma.12G179300.1	95.68345324	155	13	399	2403	2002	1	0	2702

TRINITY_DN12719_c0_g2_i2	Glyma.12G179300.2	99.10714286	155	13	500	2504	2002	1	0	2702

TRINITY_DN12719_c0_g2_i2	Glyma.12G179300.5	100	155	13	500	2504	2002	1	0	2702

TRINITY_DN12719_c0_g2_i2	Glyma.12G179300.4	100	155	13	500	2504	2002	1	0	2702

TRINITY_DN12719_c0_g2_i2	Glyma.12G179300.3	100	155	13	500	2504	2002	1	0	2702

TRINITY_DN12719_c0_g2_i2	Glyma.12G179300.1	97.66081871	155	13	500	2504	2002	1	0	2702

TRINITY_DN12719_c0_g2_i3	Glyma.12G179300.2	96.35036496	155	13	399	2403	2002	1	0	2702

TRINITY_DN12719_c0_g2_i3	Glyma.12G179300.5	99.29078014	155	13	399	2403	2002	1	0	2702

TRINITY_DN12719_c0_g2_i3	Glyma.12G179300.4	99.54751131	155	13	399	2403	2002	1	0	2702

TRINITY_DN12719_c0_g2_i3	Glyma.12G179300.3	100	155	13	399	2403	2002	1	0	2702

TRINITY_DN12719_c0_g2_i3	Glyma.12G179300.1	95.83333333	155	13	399	2403	2002	1	0	2702

TRINITY_DN12719_c0_g2_i4	Glyma.12G179300.2	100	155	13	500	2504	2002	1	0	2702

TRINITY_DN12719_c0_g2_i4	Glyma.12G179300.5	96.39175258	155	13	500	2504	2002	1	0	2702

TRINITY_DN12719_c0_g2_i4	Glyma.12G179300.4	100	155	13	500	2504	2002	1	0	2702

TRINITY_DN12719_c0_g2_i4	Glyma.12G179300.3	100	155	13	500	2504	2002	1	0	2702

TRINITY_DN12719_c0_g2_i4	Glyma.12G179300.1	99	155	13	500	2504	2002	1	0	2702

TRINITY_DN12719_c0_g2_i1	Glyma.13G321300.34	96.82539683	153	20	399	2403	1966	1	0	2481

TRINITY_DN12719_c0_g2_i1	Glyma.13G321300.35	96.82899207	153	20	399	2403	1966	1	0	2481

TRINITY_DN12719_c0_g2_i1	Glyma.13G321300.22	96.83195592	153	20	399	2403	1966	1	0	2481

TRINITY_DN12719_c0_g2_i1	Glyma.13G321300.23	100	153	20	399	2403	1966	1	0	2481

TRINITY_DN12719_c0_g2_i1	Glyma.13G321300.2	96.90721649	153	20	399	2403	1966	1	0	2481

TRINITY_DN12719_c0_g2_i1	Glyma.13G321300.3	96.90721649	153	20	399	2403	1966	1	0	2481

TRINITY_DN12719_c0_g2_i1	Glyma.13G321300.53	100	153	20	399	2403	1966	1	0	2481

TRINITY_DN12719_c0_g2_i1	Glyma.13G321300.36	99.51456311	153	20	399	2403	1966	1	0	2481

TRINITY_DN12719_c0_g2_i1	Glyma.13G321300.37	96.52777778	153	20	399	2403	1966	1	0	2481

TRINITY_DN12719_c0_g2_i1	Glyma.13G321300.24	96.55172414	153	20	399	2403	1966	1	0	2481

TRINITY_DN12719_c0_g2_i1	Glyma.13G321300.25	96.05263158	153	20	399	2403	1966	1	0	2481

TRINITY_DN12719_c0_g2_i1	Glyma.13G321300.4	98.7804878	153	20	399	2403	1966	1	0	2481

TRINITY_DN12719_c0_g2_i1	Glyma.13G321300.5	100	153	20	399	2403	1966	1	0	2481

TRINITY_DN12719_c0_g2_i2	Glyma.13G321300.34	100	153	20	500	2504	1966	1	0	2481

TRINITY_DN12719_c0_g2_i2	Glyma.13G321300.35	100	153	20	500	2504	1966	1	0	2481

TRINITY_DN12719_c0_g2_i2	Glyma.13G321300.22	96.21621622	153	20	500	2504	1966	1	0	2481

TRINITY_DN12719_c0_g2_i2	Glyma.13G321300.23	95.45454545	153	20	500	2504	1966	1	0	2481

TRINITY_DN12719_c0_g2_i2	Glyma.13G321300.2	100	153	20	500	2504	1966	1	0	2481

TRINITY_DN12719_c0_g2_i2	Glyma.13G321300.3	96.98492462	153	20	500	2504	1966	1	0	2481

TRINITY_DN12719_c0_g2_i2	Glyma.13G321300.53	98.27586207	153	20	500	2504	1966	1	0	2481

TRINITY_DN12719_c0_g2_i2	Glyma.13G321300.36	98.4375	153	20	500	2504	1966	1	0	2481

TRINITY_DN12719_c0_g2_i2	Glyma.13G321300.37	96.59442724	153	20	500	2504	1966	1	0	2481

TRINITY_DN12719_c0_g2_i2	Glyma.13G321300.24	96.59574468	153	20	500	2504	1966	1	0	2481

TRINITY_DN12719_c0_g2_i2	Glyma.13G321300.25	98.73417722	153	20	500	2504	1966	1	0	2481

TRINITY_DN12719_c0_g2_i2	Glyma.13G321300.4	97.19626168	153	20	500	2504	1966	1	0	2481

TRINITY_DN12719_c0_g2_i2	Glyma.13G321300.5	98.14241486	153	20	500	2504	1966	1	0	2481

TRINITY_DN12719_c0_g2_i3	Glyma.13G321300.34	98.79518072	153	20	399	2403	1966	1	0	2481

TRINITY_DN12719_c0_g2_i3	Glyma.13G321300.35	95.3125	153	20	399	2403	1966	1	0	2481

TRINITY_DN12719_c0_g2_i3	Glyma.13G321300.22	96.875	153	20	399	2403	1966	1	0	2481

TRINITY_DN12719_c0_g2_i3	Glyma.13G321300.23	100	153	20	399	2403	1966	1	0	2481

TRINITY_DN12719_c0_g2_i3	Glyma.13G321300.2	99.72527473	153	20	399	2403	1966	1	0	2481

TRINITY_DN12719_c0_g2_i3	Glyma.13G321300.3	100	153	20	399	2403	1966	1	0	2481

TRINITY_DN12719_c0_g2_i3	Glyma.13G321300.53	99.20634921	153	20	399	2403	1966	1	0	2481

TRINITY_DN12719_c0_g2_i3	Glyma.13G321300.36	96	153	20	399	2403	1966	1	0	2481

TRINITY_DN12719_c0_g2_i3	Glyma.13G321300.37	100	153	20	399	2403	1966	1	0	2481

TRINITY_DN12719_c0_g2_i3	Glyma.13G321300.24	100	153	20	399	2403	1966	1	0	2481

TRINITY_DN12719_c0_g2_i3	Glyma.13G321300.25	97.68786127	153	20	399	2403	1966	1	0	2481

TRINITY_DN12719_c0_g2_i3	Glyma.13G321300.4	95	153	20	399	2403	1966	1	0	2481

TRINITY_DN12719_c0_g2_i3	Glyma.13G321300.5	95.70063694	153	20	399	2403	1966	1	0	2481

TRINITY_DN12719_c0_g2_i4	Glyma.13G321300.34	96.22641509	153	20	500	2504	1966	1	0	2481

TRINITY_DN12719_c0_g2_i4	Glyma.13G321300.35	95.94594595	153	20	500	2504	1966	1	0	2481

TRINITY_DN12719_c0_g2_i4	Glyma.13G321300.22	98.57142857	153	20	500	2504	1966	1	0	2481

TRINITY_DN12719_c0_g2_i4	Glyma.13G321300.23	100	153	20	500	2504	1966	1	0	2481

TRINITY_DN12719_c0_g2_i4	Glyma.13G321300.2	95.95375723	153	20	500	2504	1966	1	0	2481

TRINITY_DN12719_c0_g2_i4	Glyma.13G321300.3	98.63945578	153	20	500	2504	1966	1	0	2481

TRINITY_DN12719_c0_g2_i4	Glyma.13G321300.53	97.61904762	153	20	500	2504	1966	1	0	2481

TRINITY_DN12719_c0_g2_i4	Glyma.13G321300.36	96.96969697	153	20	500	2504	1966	1	0	2481

TRINITY_DN12719_c0_g2_i4	Glyma.13G321300.37	100	153	20	500	2504	1966	1	0	2481

TRINITY_DN12719_c0_g2_i4	Glyma.13G321300.24	100	153	20	500	2504	1966	1	0	2481

TRINITY_DN12719_c0_g2_i4	Glyma.13G321300.25	97.59036145	153	20	500	2504	1966	1	0	2481

TRINITY_DN12719_c0_g2_i4	Glyma.13G321300.4	100	153	20	500	2504	1966	1	0	2481

TRINITY_DN12719_c0_g2_i4	Glyma.13G321300.5	100	153	20	500	2504	1966	1	0	2481

TRINITY_DN12719_c0_g2_i5	Glyma.13G321300.38	95	153	20	357	2361	1966	1	0	2481

TRINITY_DN12719_c0_g2_i5	Glyma.13G321300.39	100	153	20	357	2361	1966	1	0	2481

TRINITY_DN12719_c0_g2_i5	Glyma.13G321300.26	97.61904762	153	20	357	2361	1966	1	0	2481

TRINITY_DN12719_c0_g2_i5	Glyma.13G321300.27	100	153	20	357	2361	1966	1	0	2481

TRINITY_DN12719_c0_g2_i5	Glyma.13G321300.7	100	153	20	357	2361	1966	1	0	2481

TRINITY_DN12719_c0_g2_i5	Glyma.13G321300.6	100	153	20	357	2361	1966	1	0	2481

TRINITY_DN12719_c0_g2_i5	Glyma.13G321300.40	100	153	20	357	2361	1966	1	0	2481

TRINITY_DN12719_c0_g2_i5	Glyma.13G321300.41	100	153	20	357	2361	1966	1	0	2481

TRINITY_DN12719_c0_g2_i5	Glyma.13G321300.28	100	153	20	357	2361	1966	1	0	2481

TRINITY_DN12719_c0_g2_i5	Glyma.13G321300.29	100	153	20	357	2361	1966	1	0	2481

TRINITY_DN12719_c0_g2_i5	Glyma.13G321300.9	96.55172414	153	20	357	2361	1966	1	0	2481

TRINITY_DN12719_c0_g2_i5	Glyma.13G321300.8	97.10144928	153	20	357	2361	1966	1	0	2481

TRINITY_DN12719_c0_g2_i7	Glyma.13G321300.38	97.27272727	153	20	357	2361	1966	1	0	2481

TRINITY_DN12719_c0_g2_i7	Glyma.13G321300.39	100	153	20	357	2361	1966	1	0	2481

TRINITY_DN12719_c0_g2_i7	Glyma.13G321300.26	95.29411765	153	20	357	2361	1966	1	0	2481

TRINITY_DN12719_c0_g2_i7	Glyma.13G321300.27	98.21428571	153	20	357	2361	1966	1	0	2481

TRINITY_DN12719_c0_g2_i7	Glyma.13G321300.7	95.97585513	153	20	357	2361	1966	1	0	2481

TRINITY_DN12719_c0_g2_i7	Glyma.13G321300.6	98.5	153	20	357	2361	1966	1	0	2481

TRINITY_DN12719_c0_g2_i7	Glyma.13G321300.40	100	153	20	357	2361	1966	1	0	2481

TRINITY_DN12719_c0_g2_i7	Glyma.13G321300.41	98.11320755	153	20	357	2361	1966	1	0	2481

TRINITY_DN12719_c0_g2_i7	Glyma.13G321300.28	100	153	20	357	2361	1966	1	0	2481

TRINITY_DN12719_c0_g2_i7	Glyma.13G321300.29	100	153	20	357	2361	1966	1	0	2481

TRINITY_DN12719_c0_g2_i7	Glyma.13G321300.9	100	153	20	357	2361	1966	1	0	2481

TRINITY_DN12719_c0_g2_i7	Glyma.13G321300.8	100	153	20	357	2361	1966	1	0	2481

TRINITY_DN36817_c0_g1_i1	Glyma.09G019300.1	98.87640449	151	12	1	1996	7	2019	0	2737

TRINITY_DN36817_c0_g1_i1	Glyma.09G019300.2	98.87640449	151	12	1	1996	7	2019	0	2737

TRINITY_DN9578_c0_g1_i1	Glyma.17G259100.1	100	140	1	1	2016	1741	3753	0	2928

TRINITY_DN9578_c0_g1_i1	Glyma.17G259100.2	100	140	1	1	2016	1570	3582	0	2928

TRINITY_DN9578_c0_g1_i1	Glyma.14G220200.1	99.33774834	133	1	1	2016	1741	3753	0	2966

TRINITY_DN6026_c0_g1_i1	Glyma.04G157200.1	97.27272727	122	2	226	2240	103	2117	0	3035

TRINITY_DN14688_c0_g2_i1	Glyma.12G174400.2	100	116	3	241	2247	2016	1	0	3022

TRINITY_DN14688_c0_g2_i1	Glyma.12G174400.1	100	116	3	241	2247	2016	1	0	3022

TRINITY_DN12925_c0_g1_i1	Glyma.17G135500.1	95.46539379	99	10	263	2275	2001	1	0	3059

TRINITY_DN15634_c2_g1_i4	Glyma.10G203000.1	100	196	6	237	2248	3627	1616	0	2597

TRINITY_DN5003_c0_g1_i1	Glyma.18G150600.1	98.97959184	190	12	1	1997	39	2050	0	2532

TRINITY_DN5003_c0_g1_i1	Glyma.18G150600.2	95.91836735	189	13	1	1997	39	2047	0	2519

TRINITY_DN11696_c0_g2_i1	Glyma.05G089300.2	100	182	9	115	2121	1	2007	0	2610

TRINITY_DN14239_c0_g2_i1	Glyma.04G125500.2	98.50746269	176	8	260	2268	2003	1	0	2630

TRINITY_DN14239_c0_g2_i1	Glyma.04G125500.1	98.52125693	176	8	260	2268	2003	1	0	2630

TRINITY_DN12786_c0_g1_i1	Glyma.07G272700.4	100	168	0	145	2159	92	2106	0	2791

TRINITY_DN10990_c0_g1_i1	Glyma.18G034800.1	96.1038961	156	21	130	2134	1	1990	0	2630

TRINITY_DN15841_c0_g1_i1	Glyma.U033300.3	98.4375	138	4	604	2618	2	1987	0	2769

TRINITY_DN15841_c0_g1_i2	Glyma.U033300.3	100	138	4	604	2618	2	1987	0	2769

TRINITY_DN15329_c0_g2_i3	Glyma.05G238700.2	97.64705882	137	7	481	2495	2068	68	0	2872

TRINITY_DN19642_c0_g1_i1	Glyma.11G179000.1	97.34513274	382	73	179	2164	1	1920	0	815

TRINITY_DN6249_c0_g1_i2	Glyma.01G211900.1	100	335	30	134	2115	51	2047	0	1546

TRINITY_DN2513_c0_g1_i1	Glyma.16G105400.2	97.2972973	304	7	291	2299	73	2078	0	1951

TRINITY_DN2513_c0_g1_i1	Glyma.16G105400.1	97.2972973	304	7	291	2299	73	2078	0	1951

TRINITY_DN2513_c0_g2_i1	Glyma.16G105400.2	97.05882353	304	7	291	2299	73	2078	0	1951

TRINITY_DN2513_c0_g2_i1	Glyma.16G105400.1	97.05882353	304	7	291	2299	73	2078	0	1951

TRINITY_DN15944_c2_g2_i7	Glyma.06G109500.1	100	249	17	269	2262	2071	69	0	2139

TRINITY_DN16432_c1_g2_i6	Glyma.20G204500.1	98.82352941	248	6	52	2060	1	2012	0	2300

TRINITY_DN15634_c2_g1_i4	Glyma.20G187400.1	97.87234043	200	4	237	2248	4425	2414	0	2586

TRINITY_DN15634_c2_g1_i4	Glyma.20G187400.2	97.87234043	200	4	237	2248	3915	1904	0	2586

TRINITY_DN19139_c0_g2_i1	Glyma.07G267500.1	98.03921569	183	18	58	2033	1	2012	0	2447

TRINITY_DN11650_c0_g1_i1	Glyma.05G185000.2	96	171	8	74	2075	1	2008	0	2656

TRINITY_DN11650_c0_g1_i1	Glyma.05G185000.3	100	171	8	74	2075	1	2008	0	2656

TRINITY_DN11650_c0_g1_i2	Glyma.05G185000.2	97.65625	171	8	74	2075	1	2008	0	2656

TRINITY_DN11650_c0_g1_i2	Glyma.05G185000.3	99.41520468	171	8	74	2075	1	2008	0	2656

TRINITY_DN11469_c0_g1_i2	Glyma.13G094700.2	96.15384615	162	10	94	2095	2039	30	0	2719

TRINITY_DN8121_c0_g1_i1	Glyma.14G133600.2	100	124	13	125	2110	1	2007	0	2806

TRINITY_DN8121_c0_g1_i1	Glyma.14G133600.1	100	124	13	125	2110	1	2007	0	2806

TRINITY_DN15521_c0_g1_i1	Glyma.12G233000.2	95.9553696	307	15	1104	3111	947	2948	0	1914

TRINITY_DN16432_c1_g2_i11	Glyma.20G204500.1	100	280	4	52	2060	1	2012	0	2134

TRINITY_DN13838_c0_g1_i1	Glyma.12G206800.1	99.46808511	266	14	1	2005	2377	379	0	2102

TRINITY_DN4794_c0_g1_i1	Glyma.09G213500.1	100	241	14	259	2249	2509	513	0	2137

TRINITY_DN15655_c0_g1_i3	Glyma.07G272800.1	99.16666667	213	9	1058	3053	2015	5	0	2414

TRINITY_DN16354_c0_g1_i6	Glyma.20G192700.4	97.54601227	205	1	173	2182	1	2013	0	2562

TRINITY_DN16354_c0_g1_i6	Glyma.20G192700.5	97.52475248	205	1	173	2182	1	2013	0	2562

TRINITY_DN16354_c0_g1_i6	Glyma.20G192700.3	97.54098361	205	1	173	2182	1	2013	0	2562

TRINITY_DN16354_c0_g1_i6	Glyma.20G192700.2	97.54601227	205	1	173	2182	1	2013	0	2562

TRINITY_DN15634_c2_g1_i4	Glyma.10G203000.2	97.87234043	193	11	237	2248	3606	1616	0	2499

TRINITY_DN16354_c0_g1_i2	Glyma.20G192700.4	95	181	1	173	2182	1	2013	0	2695

TRINITY_DN16354_c0_g1_i2	Glyma.20G192700.5	95	181	1	173	2182	1	2013	0	2695

TRINITY_DN16354_c0_g1_i2	Glyma.20G192700.3	95	181	1	173	2182	1	2013	0	2695

TRINITY_DN16354_c0_g1_i2	Glyma.20G192700.2	95	181	1	173	2182	1	2013	0	2695

TRINITY_DN9237_c0_g1_i2	Glyma.11G254100.1	97.91666667	177	5	134	2140	1	2013	0	2699

TRINITY_DN16352_c0_g2_i1	Glyma.07G011600.1	100	173	1	423	2435	4393	2382	0	2752

TRINITY_DN12455_c0_g1_i1	Glyma.17G129700.1	95.13888889	170	14	216	2216	1	2001	0	2621

TRINITY_DN16167_c0_g1_i2	Glyma.04G141400.6	100	164	6	452	2452	1974	1	0	2479

TRINITY_DN16167_c0_g1_i2	Glyma.04G141400.1	100	164	6	452	2452	1974	1	0	2479

TRINITY_DN16167_c0_g1_i2	Glyma.04G141400.7	100	164	6	452	2452	1974	1	0	2479

TRINITY_DN16167_c0_g1_i4	Glyma.04G141400.6	95.95375723	164	6	607	2607	1974	1	0	2479

TRINITY_DN16167_c0_g1_i4	Glyma.04G141400.1	98.93617021	164	6	607	2607	1974	1	0	2479

TRINITY_DN16167_c0_g1_i4	Glyma.04G141400.7	98.63945578	164	6	607	2607	1974	1	0	2479

TRINITY_DN16167_c0_g1_i6	Glyma.04G141400.6	96.05263158	164	6	508	2508	1974	1	0	2479

TRINITY_DN16167_c0_g1_i6	Glyma.04G141400.1	96.05263158	164	6	508	2508	1974	1	0	2479

TRINITY_DN16167_c0_g1_i6	Glyma.04G141400.7	100	164	6	508	2508	1974	1	0	2479

TRINITY_DN16167_c0_g1_i2	Glyma.11G154700.1	97.65258216	164	10	452	2452	1986	1	0	2556

TRINITY_DN16167_c0_g1_i2	Glyma.11G154700.6	97.65258216	164	10	452	2452	1986	1	0	2556

TRINITY_DN16167_c0_g1_i2	Glyma.11G154700.7	96.81397739	164	10	452	2452	1986	1	0	2556

TRINITY_DN16167_c0_g1_i2	Glyma.11G154700.8	96.81528662	164	10	452	2452	1986	1	0	2556

TRINITY_DN16167_c0_g1_i4	Glyma.11G154700.1	95.94594595	164	10	607	2607	1986	1	0	2556

TRINITY_DN16167_c0_g1_i4	Glyma.11G154700.6	95.57713053	164	10	607	2607	1986	1	0	2556

TRINITY_DN16167_c0_g1_i4	Glyma.11G154700.7	98.57142857	164	10	607	2607	1986	1	0	2556

TRINITY_DN16167_c0_g1_i4	Glyma.11G154700.8	97.6945245	164	10	607	2607	1986	1	0	2556

TRINITY_DN16167_c0_g1_i6	Glyma.11G154700.1	97.68637532	164	10	508	2508	1986	1	0	2556

TRINITY_DN16167_c0_g1_i6	Glyma.11G154700.6	97.68637532	164	10	508	2508	1986	1	0	2556

TRINITY_DN16167_c0_g1_i6	Glyma.11G154700.7	97.68637532	164	10	508	2508	1986	1	0	2556

TRINITY_DN16167_c0_g1_i6	Glyma.11G154700.8	96.0502693	164	10	508	2508	1986	1	0	2556

TRINITY_DN11259_c0_g1_i2	Glyma.12G170500.3	100	160	1	565	2577	1	2010	0	2811

TRINITY_DN11259_c0_g1_i3	Glyma.12G170500.3	100	160	1	131	2143	1	2010	0	2811

TRINITY_DN14452_c0_g1_i2	Glyma.08G201000.1	100	160	20	266	2236	1	2001	0	2483

TRINITY_DN5903_c0_g1_i1	Glyma.12G019400.1	96.71361502	156	0	462	2474	2065	53	0	2854

TRINITY_DN12659_c0_g1_i1	Glyma.U025600.2	98.50746269	155	4	258	2266	8	2016	0	2808

TRINITY_DN12659_c0_g1_i1	Glyma.U025600.3	98.48484848	155	4	258	2266	8	2016	0	2808

TRINITY_DN12659_c0_g1_i1	Glyma.U025600.4	98.4962406	155	4	258	2266	8	2016	0	2808

TRINITY_DN9863_c0_g1_i1	Glyma.08G008600.1	98.71794872	150	5	1	2010	748	2754	0	2828

TRINITY_DN9863_c0_g1_i1	Glyma.08G008600.2	98.71794872	150	5	1	2010	742	2748	0	2828

TRINITY_DN9863_c0_g1_i1	Glyma.08G008600.3	98.71794872	150	5	1	2010	742	2748	0	2828

TRINITY_DN10566_c0_g1_i2	Glyma.05G180100.1	100	139	0	50	2062	19	2031	0	2948

TRINITY_DN10566_c0_g1_i3	Glyma.05G180100.1	100	139	0	50	2062	19	2031	0	2948

TRINITY_DN6848_c0_g2_i1	Glyma.14G183000.3	100	139	5	1	2007	4956	2947	0	2889

TRINITY_DN6848_c0_g2_i1	Glyma.14G183000.1	100	139	5	1	2007	4956	2947	0	2889

TRINITY_DN6848_c0_g2_i1	Glyma.14G183000.2	100	139	5	1	2007	4956	2947	0	2889

TRINITY_DN15765_c0_g1_i2	Glyma.17G174500.1	100	136	4	94	2100	1	2010	0	2905

TRINITY_DN6848_c0_g2_i1	Glyma.02G216000.1	100	127	8	1	2007	4947	2944	0	2916

TRINITY_DN6848_c0_g2_i1	Glyma.02G216000.2	100	127	8	1	2007	4947	2944	0	2916

TRINITY_DN11291_c0_g2_i1	Glyma.04G061600.1	100	120	7	536	2533	1	2010	0	2937

TRINITY_DN12925_c0_g1_i1	Glyma.05G053100.1	95.46485261	94	10	263	2275	1998	1	0	3099

TRINITY_DN3274_c0_g1_i1	Glyma.07G273800.1	97.93814433	455	60	611	2576	3202	1216	0	736

TRINITY_DN13116_c0_g1_i2	Glyma.13G109600.4	97.87234043	299	10	128	2132	2002	1	0	1949

TRINITY_DN13116_c0_g1_i2	Glyma.13G109600.14	97.85714286	299	10	128	2132	2002	1	0	1949

TRINITY_DN13116_c0_g1_i2	Glyma.13G109600.15	97.84946237	299	10	128	2132	2002	1	0	1949

TRINITY_DN13116_c0_g1_i2	Glyma.13G109600.5	97.87234043	299	10	128	2132	2002	1	0	1949

TRINITY_DN13423_c0_g2_i1	Glyma.09G037900.5	100	297	6	1475	3483	2006	1	0	2012

TRINITY_DN2617_c0_g1_i1	Glyma.06G158100.2	96.62921348	279	44	231	2226	7	1954	0	1653

TRINITY_DN2617_c0_g1_i1	Glyma.06G158100.3	96.61835749	279	44	231	2226	7	1954	0	1653

TRINITY_DN2617_c0_g1_i1	Glyma.06G158100.1	96.62162162	279	44	231	2226	7	1954	0	1653

TRINITY_DN12011_c0_g2_i2	Glyma.20G060300.1	100	214	2	348	2356	14	2025	0	2510

TRINITY_DN12011_c0_g2_i4	Glyma.20G060300.1	100	214	2	279	2287	14	2025	0	2510

TRINITY_DN11025_c0_g2_i1	Glyma.08G125700.2	95.04132231	179	10	208	2207	1	2006	0	2608

TRINITY_DN11025_c0_g2_i2	Glyma.08G125700.2	95	179	10	73	2072	1	2006	0	2608

TRINITY_DN16352_c0_g2_i1	Glyma.08G194600.1	100	172	5	427	2435	4396	2391	0	2704

TRINITY_DN16352_c0_g2_i1	Glyma.08G194600.2	100	172	5	427	2435	3643	1638	0	2704

TRINITY_DN14641_c1_g1_i1	Glyma.14G217700.1	99.43661972	166	24	403	2392	2793	813	0	2453

TRINITY_DN11469_c0_g1_i1	Glyma.13G094700.2	96.15384615	162	10	646	2645	2037	30	0	2715

TRINITY_DN11469_c0_g1_i2	Glyma.13G094700.1	96.22641509	162	10	96	2095	2037	30	0	2715

TRINITY_DN11469_c0_g1_i3	Glyma.13G094700.2	100	161	16	646	2622	2037	30	0	2571

TRINITY_DN16050_c0_g1_i1	Glyma.18G288600.3	95.76923077	132	5	772	2780	2803	795	0	2946

TRINITY_DN15464_c0_g1_i5	Glyma.17G083300.1	100	102	3	187	2192	1	1986	0	2944

TRINITY_DN15464_c0_g1_i5	Glyma.17G083300.2	97.50673854	102	3	187	2192	1	1986	0	2944

TRINITY_DN7243_c0_g1_i2	Glyma.10G253200.1	100	305	32	305	2281	2091	106	0	1642

TRINITY_DN14897_c0_g1_i1	Glyma.01G082400.1	95.65217391	277	12	192	2184	21	2025	0	2025

TRINITY_DN13838_c0_g1_i1	Glyma.13G294100.1	98.71794872	256	14	1	2005	2368	370	0	2180

TRINITY_DN16432_c1_g2_i6	Glyma.20G204500.2	97.26027397	249	4	52	2060	1	2009	0	2309

TRINITY_DN15944_c2_g2_i7	Glyma.06G109500.2	100	249	16	269	2262	2068	69	0	2154

TRINITY_DN11695_c0_g1_i1	Glyma.09G204500.1	100	246	25	547	2521	2194	201	0	2008

TRINITY_DN16194_c1_g1_i7	Glyma.06G109800.1	100	210	26	1	1942	270	2268	0	2026

TRINITY_DN16194_c1_g1_i7	Glyma.06G109800.3	100	210	26	1	1942	270	2268	0	2026

TRINITY_DN16194_c1_g1_i7	Glyma.06G109800.2	100	210	26	1	1942	180	2178	0	2026

TRINITY_DN7209_c0_g2_i1	Glyma.09G191300.1	100	191	21	74	2062	1995	1	0	2410

TRINITY_DN7209_c0_g2_i2	Glyma.09G191300.1	100	191	21	219	2207	1995	1	0	2410

TRINITY_DN14926_c0_g1_i1	Glyma.08G130900.4	100	187	13	527	2533	1986	1	0	2490

TRINITY_DN14926_c0_g1_i1	Glyma.08G130900.1	100	187	13	527	2533	1986	1	0	2490

TRINITY_DN14926_c0_g1_i1	Glyma.08G130900.2	100	187	13	527	2533	1986	1	0	2490

TRINITY_DN14926_c0_g1_i1	Glyma.08G130900.3	100	187	13	527	2533	1986	1	0	2490

TRINITY_DN14926_c0_g1_i1	Glyma.05G173600.1	100	178	10	527	2533	1986	1	0	2540

TRINITY_DN15085_c0_g1_i1	Glyma.18G041900.1	99.4047619	139	10	131	2140	1	1992	0	2815

TRINITY_DN8849_c0_g1_i1	Glyma.15G136300.1	95.8	135	11	1	2011	2529	546	0	2791

TRINITY_DN15281_c0_g1_i1	Glyma.07G269900.1	97.60191847	294	26	462	2452	1	1994	0	1857

TRINITY_DN15281_c0_g1_i6	Glyma.07G269900.1	100	294	26	676	2666	1	1994	0	1857

TRINITY_DN16432_c1_g2_i11	Glyma.20G204500.2	97.14285714	281	2	52	2060	1	2009	0	2143

TRINITY_DN16251_c0_g3_i10	Glyma.09G060600.1	100	232	12	357	2358	2523	519	0	2342

TRINITY_DN16251_c0_g3_i10	Glyma.09G060600.4	95	232	12	357	2358	2508	504	0	2342

TRINITY_DN16251_c0_g3_i10	Glyma.09G060600.3	96.28378378	231	13	357	2358	2502	519	0	2213

TRINITY_DN16251_c0_g3_i10	Glyma.09G060600.2	100	231	13	357	2358	2487	504	0	2213

TRINITY_DN16251_c0_g3_i10	Glyma.09G060600.5	99.86091794	230	14	357	2358	2520	519	0	2335

TRINITY_DN12306_c0_g1_i2	Glyma.17G060400.1	100	197	1	300	2306	6	2015	0	2601

TRINITY_DN36194_c0_g1_i1	Glyma.07G076000.2	100	175	0	217	2226	1	2010	0	2743

TRINITY_DN36194_c0_g1_i1	Glyma.07G076000.1	100	175	0	217	2226	1	2010	0	2743

TRINITY_DN16167_c0_g1_i2	Glyma.04G141400.4	100	164	5	452	2452	1971	1	0	2494

TRINITY_DN16167_c0_g1_i2	Glyma.04G141400.5	100	164	5	452	2452	1971	1	0	2494

TRINITY_DN16167_c0_g1_i4	Glyma.04G141400.4	100	164	5	607	2607	1971	1	0	2494

TRINITY_DN16167_c0_g1_i4	Glyma.04G141400.5	98.21428571	164	5	607	2607	1971	1	0	2494

TRINITY_DN16167_c0_g1_i6	Glyma.04G141400.4	96.05055292	164	5	508	2508	1971	1	0	2494

TRINITY_DN16167_c0_g1_i6	Glyma.04G141400.5	96.05055292	164	5	508	2508	1971	1	0	2494

TRINITY_DN16167_c0_g1_i2	Glyma.11G154700.2	99.18699187	164	9	452	2452	1983	1	0	2571

TRINITY_DN16167_c0_g1_i2	Glyma.11G154700.4	99.18699187	164	9	452	2452	1983	1	0	2571

TRINITY_DN16167_c0_g1_i2	Glyma.11G154700.3	99.18699187	164	9	452	2452	1983	1	0	2571

TRINITY_DN16167_c0_g1_i2	Glyma.11G154700.5	98.68421053	164	9	452	2452	1983	1	0	2571

TRINITY_DN16167_c0_g1_i4	Glyma.11G154700.2	96.22641509	164	9	607	2607	1983	1	0	2571

TRINITY_DN16167_c0_g1_i4	Glyma.11G154700.4	96.22641509	164	9	607	2607	1983	1	0	2571

TRINITY_DN16167_c0_g1_i4	Glyma.11G154700.3	96.22641509	164	9	607	2607	1983	1	0	2571

TRINITY_DN16167_c0_g1_i4	Glyma.11G154700.5	96.22641509	164	9	607	2607	1983	1	0	2571

TRINITY_DN16167_c0_g1_i6	Glyma.11G154700.2	98.46153846	164	9	508	2508	1983	1	0	2571

TRINITY_DN16167_c0_g1_i6	Glyma.11G154700.4	95.50561798	164	9	508	2508	1983	1	0	2571

TRINITY_DN16167_c0_g1_i6	Glyma.11G154700.3	95.50561798	164	9	508	2508	1983	1	0	2571

TRINITY_DN16167_c0_g1_i6	Glyma.11G154700.5	97.68421053	164	9	508	2508	1983	1	0	2571

TRINITY_DN11875_c0_g2_i2	Glyma.08G105200.1	95.29411765	161	5	1097	3100	897	2897	0	2723

TRINITY_DN418_c0_g1_i1	Glyma.18G222100.1	96.15384615	144	13	9	1994	70	2070	0	2700

TRINITY_DN15085_c0_g1_i1	Glyma.11G214700.1	99.4047619	140	0	131	2140	1	2010	0	2937

TRINITY_DN15200_c0_g1_i1	Glyma.20G212800.1	96.19047619	337	9	301	2305	2034	42	0	1714

TRINITY_DN15281_c0_g1_i4	Glyma.07G269900.1	99.12280702	243	24	462	2452	1	1994	0	2150

TRINITY_DN15281_c0_g1_i5	Glyma.07G269900.1	100	243	24	676	2666	1	1994	0	2150

TRINITY_DN16181_c0_g1_i2	Glyma.14G077200.1	99.15966387	242	6	120	2124	2005	1	0	2318

TRINITY_DN16181_c0_g1_i2	Glyma.17G248400.2	96.04863222	235	4	120	2124	2008	1	0	2375

TRINITY_DN16181_c0_g1_i2	Glyma.17G248400.3	98.88888889	235	4	120	2124	2008	1	0	2375

TRINITY_DN16181_c0_g1_i2	Glyma.17G248400.1	97.5	235	4	120	2124	2008	1	0	2375

TRINITY_DN16181_c0_g1_i4	Glyma.17G248400.5	97.2972973	235	4	890	2894	2008	1	0	2375

TRINITY_DN16181_c0_g1_i2	Glyma.17G248400.4	100	235	7	120	2124	1996	1	0	2298

TRINITY_DN16360_c0_g8_i5	Glyma.11G073100.1	99.34640523	235	8	278	2277	94	2099	0	2331

TRINITY_DN13117_c0_g1_i1	Glyma.17G207700.1	100	226	18	232	2217	1992	3	0	2187

TRINITY_DN13117_c0_g1_i2	Glyma.17G207700.1	99.51690821	226	18	668	2653	1992	3	0	2187

TRINITY_DN16207_c0_g1_i3	Glyma.20G105400.1	97.4025974	215	5	239	2245	2820	817	0	2473

TRINITY_DN15207_c0_g1_i2	Glyma.11G256000.1	98.63013699	149	13	100	2100	397	2364	0	2567

TRINITY_DN8335_c0_g2_i1	Glyma.05G222700.3	98.13664596	120	4	385	2387	1	2006	0	2987

TRINITY_DN8335_c0_g2_i1	Glyma.05G222700.2	96.15384615	120	4	385	2387	1	2006	0	2987

TRINITY_DN8335_c0_g2_i1	Glyma.05G222700.1	98.48484848	120	4	385	2387	1	2006	0	2987

TRINITY_DN15464_c0_g1_i5	Glyma.05G016700.1	97.50692521	105	2	187	2192	1	1983	0	2940

TRINITY_DN15464_c0_g1_i5	Glyma.05G016700.6	97.50692521	105	2	187	2192	1	1983	0	2940

TRINITY_DN15415_c0_g1_i3	Glyma.17G085500.1	98.80952381	386	18	173	2176	148	2133	0	1402

TRINITY_DN15710_c0_g2_i1	Glyma.U033500.1	95.54140127	296	41	36	2016	2430	458	0	1668

TRINITY_DN15710_c0_g2_i2	Glyma.U033500.1	97.09821429	296	41	36	2016	2430	458	0	1668

TRINITY_DN14621_c1_g1_i2	Glyma.16G063900.2	97.69357496	286	6	207	2211	80	2084	0	2085

TRINITY_DN14621_c1_g1_i2	Glyma.16G063900.1	97.6945245	286	6	207	2211	80	2084	0	2085

TRINITY_DN14621_c1_g1_i4	Glyma.16G063900.2	100	266	6	207	2211	80	2084	0	2196

TRINITY_DN14621_c1_g1_i4	Glyma.16G063900.1	100	266	6	207	2211	80	2084	0	2196

TRINITY_DN11346_c0_g1_i1	Glyma.20G197800.1	95.54140127	186	10	4	2007	1513	3499	0	2516

TRINITY_DN8762_c0_g1_i1	Glyma.20G202800.1	98.95833333	185	10	313	2303	2006	1	0	2560

TRINITY_DN14641_c1_g1_i1	Glyma.17G256500.1	95.91194969	167	14	403	2392	2802	822	0	2492

TRINITY_DN9761_c0_g1_i3	Glyma.13G131800.1	96.55172414	128	10	476	2471	1	1981	0	2747

TRINITY_DN13694_c0_g1_i3	Glyma.07G103000.3	97.18309859	115	0	2137	4144	2124	4131	0	3072

TRINITY_DN13694_c0_g1_i3	Glyma.07G103000.1	95.75971731	115	0	2137	4144	2124	4131	0	3072

TRINITY_DN13694_c0_g1_i3	Glyma.07G103000.2	100	115	0	2137	4144	2124	4131	0	3072

TRINITY_DN14043_c0_g2_i3	Glyma.13G216400.2	100	344	27	251	2230	1	1992	0	1530

TRINITY_DN15200_c0_g1_i1	Glyma.10G177300.1	95.36679537	329	13	301	2301	2034	46	0	1729

TRINITY_DN15751_c0_g1_i1	Glyma.05G045700.1	96.07843137	287	42	968	2962	7	1944	0	1592

TRINITY_DN14938_c0_g1_i2	Glyma.10G026000.1	100	243	27	318	2299	1982	1	0	2037

TRINITY_DN16309_c0_g1_i2	Glyma.11G033600.2	97.5	223	31	3063	5031	2835	4823	0	2109

TRINITY_DN16309_c0_g1_i2	Glyma.11G033600.1	97.5	223	31	3063	5031	2835	4823	0	2109

TRINITY_DN16309_c0_g1_i2	Glyma.11G033600.3	97.5	223	31	3063	5031	2835	4823	0	2109

TRINITY_DN16309_c0_g1_i2	Glyma.11G033600.4	97.5	223	31	3063	5031	2835	4823	0	2109

TRINITY_DN16309_c0_g1_i3	Glyma.11G033600.2	100	223	31	2997	4965	2835	4823	0	2109

TRINITY_DN16309_c0_g1_i3	Glyma.11G033600.1	100	223	31	2997	4965	2835	4823	0	2109

TRINITY_DN16309_c0_g1_i3	Glyma.11G033600.3	100	223	31	2997	4965	2835	4823	0	2109

TRINITY_DN16309_c0_g1_i3	Glyma.11G033600.4	100	223	31	2997	4965	2835	4823	0	2109

TRINITY_DN15207_c0_g1_i2	Glyma.18G001100.1	97.93577982	164	16	100	2100	382	2349	0	2507

TRINITY_DN16360_c0_g4_i3	Glyma.08G193500.1	95.0310559	162	4	294	2298	82	2086	0	2784

TRINITY_DN13816_c0_g1_i1	Glyma.20G113300.1	97.01492537	162	5	278	2275	1	2004	0	2732

TRINITY_DN13816_c0_g1_i2	Glyma.20G113300.1	95.76868829	162	5	278	2275	1	2004	0	2732

TRINITY_DN13816_c0_g1_i1	Glyma.20G113300.2	100	162	6	278	2275	1	2001	0	2712

TRINITY_DN13816_c0_g1_i2	Glyma.20G113300.2	95.76868829	162	6	278	2275	1	2001	0	2712

TRINITY_DN16360_c0_g4_i3	Glyma.07G010400.1	95.0310559	154	4	294	2298	88	2092	0	2828

TRINITY_DN14820_c0_g1_i2	Glyma.01G207500.1	98.7012987	142	2	54	2059	185	2190	0	2907

TRINITY_DN16356_c0_g1_i1	Glyma.05G219400.1	100	142	3	118	2115	1	2007	0	2861

TRINITY_DN16356_c0_g1_i2	Glyma.05G219400.1	100	138	1	168	2171	1	2007	0	2922

TRINITY_DN16356_c0_g1_i2	Glyma.08G025700.1	100	135	3	168	2171	1	2004	0	2920

TRINITY_DN16356_c0_g1_i5	Glyma.05G219400.1	99.51690821	132	3	168	2165	1	2007	0	2916

TRINITY_DN11291_c0_g2_i1	Glyma.06G062300.1	100	124	13	536	2533	1	1992	0	2865

TRINITY_DN16217_c2_g1_i7	Glyma.06G145700.1	100	122	12	523	2521	2259	265	0	2902

TRINITY_DN16217_c2_g1_i7	Glyma.06G145700.2	100	122	12	523	2521	2259	265	0	2902

TRINITY_DN14244_c0_g4_i2	Glyma.15G044700.1	96.19565217	105	0	186	2192	2010	4	0	3125

TRINITY_DN16396_c2_g1_i3	Glyma.11G138500.1	97.22222222	297	48	267	2252	58	2010	0	1587

TRINITY_DN16259_c0_g2_i2	Glyma.03G106500.1	97.64705882	296	36	3021	5008	2146	4106	0	1657

TRINITY_DN16259_c0_g2_i3	Glyma.03G106500.1	97.84102061	296	36	3027	5014	2146	4106	0	1657

TRINITY_DN8761_c0_g1_i1	Glyma.15G037800.1	100	276	24	245	2239	1992	16	0	1917

TRINITY_DN9382_c0_g2_i1	Glyma.20G026800.1	95.75471698	257	25	33	2012	1	1989	0	2002

TRINITY_DN11554_c0_g1_i1	Glyma.13G003700.1	99.36708861	195	5	149	2147	1	2002	0	2553

TRINITY_DN9359_c0_g1_i2	Glyma.10G251100.1	96.2962963	181	19	309	2311	2769	812	0	2372

TRINITY_DN9359_c0_g1_i2	Glyma.10G251100.3	100	181	19	309	2311	2472	515	0	2372

TRINITY_DN12455_c0_g1_i1	Glyma.05G047800.1	100	178	12	220	2216	8	2004	0	2603

TRINITY_DN5413_c0_g1_i2	Glyma.10G215800.1	95.89041096	154	13	563	2553	2136	155	0	2599

TRINITY_DN5413_c0_g1_i2	Glyma.20G176000.1	100	149	12	563	2553	2100	119	0	2627

TRINITY_DN15207_c0_g1_i2	Glyma.11G256000.2	96.03174603	149	13	103	2100	397	2361	0	2562

TRINITY_DN15207_c0_g1_i2	Glyma.11G256000.3	96.03174603	149	13	103	2100	397	2361	0	2562

TRINITY_DN9872_c0_g1_i1	Glyma.06G108500.6	100	137	11	1766	3771	1709	3675	0	2693

TRINITY_DN9872_c0_g1_i1	Glyma.06G108500.4	98.41269841	137	11	1766	3771	1568	3534	0	2693

TRINITY_DN9872_c0_g1_i1	Glyma.06G108500.2	96.35258359	137	11	1766	3771	1568	3534	0	2693

TRINITY_DN16102_c0_g2_i3	Glyma.12G234300.2	100	133	0	149	2154	1	2006	0	2968

TRINITY_DN16102_c0_g2_i3	Glyma.12G234300.1	100	133	0	149	2154	1	2006	0	2968

TRINITY_DN16102_c0_g2_i3	Glyma.13G264500.1	100	132	0	149	2154	1	2006	0	2974

TRINITY_DN14622_c0_g1_i1	Glyma.20G210700.3	96.58634538	132	6	274	2270	86	2082	0	2857

TRINITY_DN16360_c0_g8_i1	Glyma.17G254100.3	100	445	34	264	2249	97	2079	0	972

TRINITY_DN11529_c1_g2_i3	Glyma.11G024700.1	95.07186858	306	12	278	2272	1992	1	0	1858

TRINITY_DN16396_c2_g1_i5	Glyma.11G138500.1	97.23577236	302	46	267	2252	58	2010	0	1570

TRINITY_DN15970_c0_g2_i11	Glyma.18G188000.1	96.72131148	266	12	230	2212	31	2031	0	2061

TRINITY_DN11346_c0_g1_i1	Glyma.10G192700.1	95.54140127	183	13	6	2007	1555	3526	0	2457

TRINITY_DN3187_c0_g1_i1	Glyma.08G294300.1	100	170	23	170	2126	1	1996	0	2392

TRINITY_DN36194_c0_g1_i1	Glyma.03G015700.1	100	167	5	217	2216	1	1995	0	2680

TRINITY_DN16194_c1_g1_i1	Glyma.06G109800.1	95.16129032	165	18	1	1942	270	2268	0	2342

TRINITY_DN16194_c1_g1_i1	Glyma.06G109800.3	95.16129032	165	18	1	1942	270	2268	0	2342

TRINITY_DN16194_c1_g1_i1	Glyma.06G109800.2	95.16129032	165	18	1	1942	180	2178	0	2342

TRINITY_DN14672_c0_g3_i3	Glyma.01G169100.1	99.12280702	149	9	241	2230	6	2004	0	2741

TRINITY_DN16356_c0_g1_i2	Glyma.05G219600.1	100	123	2	167	2171	105	2106	0	3001

TRINITY_DN16181_c0_g1_i2	Glyma.04G034200.1	98.24561404	103	1	120	2124	2002	1	0	3112

TRINITY_DN16181_c0_g1_i2	Glyma.06G034400.2	98.82352941	100	1	120	2124	2002	1	0	3129

TRINITY_DN16379_c0_g2_i6	Glyma.12G177100.1	97.36842105	371	13	313	2310	2424	436	0	1509

TRINITY_DN14801_c0_g1_i2	Glyma.14G153000.1	95.95375723	368	24	395	2381	2325	339	0	1443

TRINITY_DN16354_c0_g1_i6	Glyma.08G027000.2	97.52066116	346	11	173	2168	1	1999	0	1700

TRINITY_DN15200_c0_g1_i2	Glyma.09G154600.1	98.33333333	302	16	252	2237	2052	58	0	1853

TRINITY_DN15007_c0_g1_i4	Glyma.11G242000.2	100	276	21	246	2233	1982	1	0	1927

TRINITY_DN15007_c0_g1_i5	Glyma.11G242000.2	100	276	21	246	2233	1982	1	0	1927

TRINITY_DN15529_c1_g1_i5	Glyma.05G232400.9	100	222	10	20	2012	2001	3	0	2368

TRINITY_DN15529_c1_g1_i5	Glyma.05G232400.8	100	222	10	20	2012	2001	3	0	2368

TRINITY_DN15529_c1_g1_i5	Glyma.05G232400.7	100	222	10	20	2012	2001	3	0	2368

TRINITY_DN15529_c1_g1_i5	Glyma.05G232400.6	98.55072464	222	10	20	2012	2001	3	0	2368

TRINITY_DN15529_c1_g1_i5	Glyma.05G232400.5	98.55072464	222	10	20	2012	2001	3	0	2368

TRINITY_DN15529_c1_g1_i5	Glyma.05G232400.3	100	222	10	20	2012	2001	3	0	2368

TRINITY_DN16309_c0_g1_i2	Glyma.01G208600.1	97.5	221	28	3065	5031	2842	4829	0	2134

TRINITY_DN16309_c0_g1_i3	Glyma.01G208600.1	100	221	28	2999	4965	2842	4829	0	2134

TRINITY_DN15501_c0_g2_i1	Glyma.08G214300.2	100	219	4	303	2304	2002	1	0	2462

TRINITY_DN5003_c0_g1_i1	Glyma.08G275500.2	95.23809524	207	12	10	1998	54	2054	0	2438

TRINITY_DN5003_c0_g1_i1	Glyma.08G275500.1	97.5	207	12	10	1998	54	2054	0	2438

TRINITY_DN35853_c0_g1_i1	Glyma.08G357200.1	96.77419355	175	19	30	2000	1	1998	0	2479

TRINITY_DN15329_c0_g2_i7	Glyma.02G085000.4	95.35714286	171	13	512	2513	2034	68	0	2501

TRINITY_DN15560_c0_g1_i1	Glyma.10G037500.1	98.93617021	165	6	204	2205	1	1992	0	2697

TRINITY_DN20853_c0_g1_i1	Glyma.05G164500.2	95.14563107	165	12	36	2030	1	1983	0	2593

TRINITY_DN15207_c0_g1_i2	Glyma.18G001100.2	97.12643678	164	16	103	2100	382	2346	0	2501

TRINITY_DN15594_c0_g1_i4	Glyma.01G203100.3	97.22222222	151	3	1	2000	2003	1	0	2832

TRINITY_DN15594_c0_g1_i4	Glyma.01G203100.1	97.22222222	151	3	1	2000	2003	1	0	2832

TRINITY_DN15594_c0_g1_i4	Glyma.01G203100.2	97.22222222	151	3	1	2000	2003	1	0	2832

TRINITY_DN9863_c0_g1_i1	Glyma.05G201200.1	99.31972789	151	3	1	2004	757	2754	0	2826

TRINITY_DN9863_c0_g1_i1	Glyma.05G201200.2	97.77777778	151	3	1	2004	751	2748	0	2826

TRINITY_DN9863_c0_g1_i1	Glyma.05G201200.3	98.71794872	151	3	1	2004	406	2403	0	2826

TRINITY_DN15867_c0_g3_i3	Glyma.17G052600.3	98.7654321	148	6	1355	3345	987	2986	0	2771

TRINITY_DN13844_c0_g1_i1	Glyma.11G175700.2	95.45454545	143	1	335	2338	1	2001	0	2889

TRINITY_DN8335_c0_g2_i1	Glyma.08G029500.1	98.61751152	137	10	385	2387	1	1988	0	2832

TRINITY_DN16356_c0_g1_i1	Glyma.08G025700.1	100	134	3	118	2115	1	2004	0	2920

TRINITY_DN16356_c0_g1_i5	Glyma.08G025700.1	100	125	3	168	2165	1	2004	0	2970

TRINITY_DN15464_c0_g1_i4	Glyma.17G083300.1	95.99271403	101	4	187	2184	1	1986	0	2987

TRINITY_DN15464_c0_g1_i4	Glyma.17G083300.2	95.72192513	101	4	187	2184	1	1986	0	2987

TRINITY_DN15994_c0_g1_i1	Glyma.17G112500.2	97.09480122	454	43	1911	3881	2012	37	0	802

TRINITY_DN15994_c0_g1_i1	Glyma.17G112500.1	95.16129032	454	43	1911	3881	2012	37	0	802

TRINITY_DN15994_c0_g1_i3	Glyma.17G112500.2	100	454	43	1845	3815	2012	37	0	802

TRINITY_DN15994_c0_g1_i3	Glyma.17G112500.1	95.4674221	454	43	1845	3815	2012	37	0	802

TRINITY_DN3145_c0_g1_i1	Glyma.10G003000.6	95.58823529	321	13	280	2267	20	2016	0	1784

TRINITY_DN11529_c1_g2_i3	Glyma.01G218800.1	95.07186858	309	10	278	2272	1992	1	0	1864

TRINITY_DN9172_c0_g1_i1	Glyma.10G185700.1	99.18032787	302	29	219	2191	2001	23	0	1677

TRINITY_DN15529_c1_g1_i2	Glyma.05G232400.9	97.01492537	276	10	20	2011	2001	4	0	2067

TRINITY_DN15529_c1_g1_i2	Glyma.05G232400.8	97.0845481	276	10	20	2011	2001	4	0	2067

TRINITY_DN15529_c1_g1_i2	Glyma.05G232400.7	97.0845481	276	10	20	2011	2001	4	0	2067

TRINITY_DN15529_c1_g1_i2	Glyma.05G232400.6	97.08520179	276	10	20	2011	2001	4	0	2067

TRINITY_DN15529_c1_g1_i2	Glyma.05G232400.5	97.08737864	276	10	20	2011	2001	4	0	2067

TRINITY_DN15529_c1_g1_i2	Glyma.05G232400.3	100	276	10	20	2011	2001	4	0	2067

TRINITY_DN15529_c1_g1_i4	Glyma.05G232400.9	100	276	10	20	2011	2001	4	0	2067

TRINITY_DN15529_c1_g1_i4	Glyma.05G232400.8	100	276	10	20	2011	2001	4	0	2067

TRINITY_DN15529_c1_g1_i4	Glyma.05G232400.7	100	276	10	20	2011	2001	4	0	2067

TRINITY_DN15529_c1_g1_i4	Glyma.05G232400.6	100	276	10	20	2011	2001	4	0	2067

TRINITY_DN15529_c1_g1_i4	Glyma.05G232400.5	99.15878023	276	10	20	2011	2001	4	0	2067

TRINITY_DN15529_c1_g1_i4	Glyma.05G232400.3	99.15966387	276	10	20	2011	2001	4	0	2067

TRINITY_DN16259_c0_g2_i1	Glyma.03G106500.1	99.13793103	250	34	3027	5011	2146	4106	0	1927

TRINITY_DN16102_c0_g2_i3	Glyma.08G148100.1	96.55172414	225	4	149	2149	1	2001	0	2427

TRINITY_DN14820_c0_g1_i4	Glyma.11G035200.1	99.3006993	221	5	75	2072	1	2001	0	2429

TRINITY_DN14244_c0_g4_i2	Glyma.12G072700.1	95.57522124	214	7	186	2186	1998	1	0	2468

TRINITY_DN16102_c0_g2_i3	Glyma.15G210300.1	100	212	4	149	2149	1	2001	0	2499

TRINITY_DN16102_c0_g2_i3	Glyma.15G210300.3	100	212	4	149	2149	1	2001	0	2499

TRINITY_DN16102_c0_g2_i3	Glyma.15G210300.2	99.25925926	212	4	149	2149	1	2001	0	2499

TRINITY_DN11554_c0_g1_i1	Glyma.20G065900.1	99.36708861	186	7	153	2147	8	2005	0	2584

TRINITY_DN14820_c0_g1_i2	Glyma.11G035200.1	100	147	5	62	2059	1	2001	0	2839

TRINITY_DN15867_c0_g3_i3	Glyma.13G106700.15	95.45454545	142	4	1355	3345	987	2986	0	2815

TRINITY_DN15867_c0_g3_i3	Glyma.13G106700.8	95.04950495	142	4	1355	3345	987	2986	0	2815

TRINITY_DN15867_c0_g3_i3	Glyma.13G106700.9	95.45454545	142	4	1355	3345	987	2986	0	2815

TRINITY_DN15867_c0_g3_i3	Glyma.13G106700.10	98.92473118	142	4	1355	3345	987	2986	0	2815

TRINITY_DN15867_c0_g3_i3	Glyma.13G106700.5	95.6284153	142	4	1355	3345	987	2986	0	2815

TRINITY_DN15867_c0_g3_i3	Glyma.13G106700.6	100	142	4	1355	3345	987	2986	0	2815

TRINITY_DN7121_c0_g2_i1	Glyma.12G181600.1	97.31182796	141	22	302	2298	2577	611	0	2647

TRINITY_DN16217_c2_g1_i3	Glyma.06G145700.1	100	83	5	523	2521	2259	265	0	3162

TRINITY_DN16217_c2_g1_i3	Glyma.06G145700.2	96.1038961	83	5	523	2521	2259	265	0	3162

TRINITY_DN6691_c0_g2_i1	Glyma.01G173800.1	96.60194175	413	44	400	2375	32	2004	0	1053

TRINITY_DN15501_c0_g2_i2	Glyma.08G214300.2	100	350	6	377	2375	1999	1	0	1720

TRINITY_DN16354_c0_g1_i2	Glyma.08G027000.2	95	325	7	173	2168	1	1999	0	1838

TRINITY_DN16354_c0_g1_i3	Glyma.08G027000.2	95	325	7	826	2821	1	1999	0	1838

TRINITY_DN16354_c0_g1_i4	Glyma.08G027000.2	95	325	7	2924	4919	1	1999	0	1838

TRINITY_DN15281_c0_g1_i1	Glyma.17G003900.2	95.32467532	298	21	462	2453	1	1986	0	1879

TRINITY_DN15281_c0_g1_i1	Glyma.17G003900.1	97.10144928	298	21	462	2453	1	1986	0	1879

TRINITY_DN15281_c0_g1_i6	Glyma.17G003900.2	98.08917197	298	21	676	2667	1	1986	0	1879

TRINITY_DN15281_c0_g1_i6	Glyma.17G003900.1	100	298	21	676	2667	1	1986	0	1879

TRINITY_DN15385_c0_g1_i4	Glyma.09G245000.1	97.86516854	269	29	213	2183	2169	184	0	1903

TRINITY_DN15385_c0_g1_i4	Glyma.09G245000.2	98.36065574	269	29	213	2183	2169	184	0	1903

TRINITY_DN14820_c0_g1_i1	Glyma.11G035200.1	99.38650307	248	5	75	2071	1	2000	0	2278

TRINITY_DN15944_c1_g1_i2	Glyma.01G210600.1	96.51162791	247	4	621	2618	2306	309	0	2278

TRINITY_DN5259_c0_g3_i1	Glyma.08G344100.1	96.22641509	236	5	98	2098	1	1998	0	2357

TRINITY_DN13713_c0_g1_i1	Glyma.15G042700.1	100	222	10	296	2281	1	1992	0	2300

TRINITY_DN15594_c0_g1_i4	Glyma.17G151100.1	97.22222222	172	6	1	2000	1994	1	0	2680

TRINITY_DN6702_c0_g1_i1	Glyma.11G019900.4	100	169	1	281	2282	2359	361	0	2741

TRINITY_DN6702_c0_g1_i1	Glyma.11G019900.3	100	169	1	281	2282	2359	361	0	2741

TRINITY_DN6702_c0_g1_i1	Glyma.11G019900.2	100	169	1	281	2282	2359	361	0	2741

TRINITY_DN6702_c0_g1_i1	Glyma.11G019900.1	100	169	1	281	2282	2359	361	0	2741

TRINITY_DN6702_c0_g1_i1	Glyma.01G223600.5	96.875	151	0	281	2282	2362	361	0	2861

TRINITY_DN6702_c0_g1_i1	Glyma.01G223600.2	96.875	151	0	281	2282	2362	361	0	2861

TRINITY_DN6702_c0_g1_i1	Glyma.01G223600.1	98.98989899	151	0	281	2282	2362	361	0	2861

TRINITY_DN6702_c0_g1_i1	Glyma.01G223600.4	98.98989899	151	0	281	2282	2362	361	0	2861

TRINITY_DN6702_c0_g1_i1	Glyma.01G223600.3	98.98989899	151	0	281	2282	2362	361	0	2861

TRINITY_DN16078_c0_g1_i2	Glyma.06G289400.1	100	141	5	55	2056	676	2658	0	2793

TRINITY_DN16078_c0_g1_i2	Glyma.12G117600.1	100	139	5	55	2056	676	2658	0	2804

TRINITY_DN36817_c0_g1_i1	Glyma.15G125400.1	97.12230216	135	4	1	1996	7	2007	0	2904

TRINITY_DN15329_c0_g2_i7	Glyma.05G238700.1	98.5915493	131	8	512	2513	2037	68	0	2765

TRINITY_DN16356_c0_g1_i5	Glyma.05G219600.1	100	123	1	167	2165	105	2106	0	2996

TRINITY_DN5259_c0_g3_i1	Glyma.16G083100.1	100	118	2	98	2098	1	2001	0	3031

TRINITY_DN5259_c0_g3_i1	Glyma.16G083100.2	95.83333333	118	2	98	2098	1	2001	0	3031

TRINITY_DN15529_c1_g1_i3	Glyma.05G232400.9	99.36305732	116	3	568	2566	2001	3	0	3016

TRINITY_DN15529_c1_g1_i3	Glyma.05G232400.8	100	116	3	568	2566	2001	3	0	3016

TRINITY_DN15529_c1_g1_i3	Glyma.05G232400.7	96.19205298	116	3	568	2566	2001	3	0	3016

TRINITY_DN15529_c1_g1_i3	Glyma.05G232400.6	96.19377163	116	3	568	2566	2001	3	0	3016

TRINITY_DN15529_c1_g1_i3	Glyma.05G232400.5	96.19377163	116	3	568	2566	2001	3	0	3016

TRINITY_DN15529_c1_g1_i3	Glyma.05G232400.3	100	116	3	568	2566	2001	3	0	3016

TRINITY_DN15782_c0_g1_i3	Glyma.04G087300.1	97.65258216	109	2	27	2027	2344	4344	0	3081

TRINITY_DN15782_c0_g1_i3	Glyma.06G089000.2	97.64705882	100	3	27	2027	2347	4344	0	3110

TRINITY_DN15782_c0_g1_i3	Glyma.06G089000.1	100	100	3	27	2027	2347	4344	0	3110

TRINITY_DN16354_c0_g1_i7	Glyma.08G027000.2	97.5308642	324	7	826	2820	1	1998	0	1842

TRINITY_DN15281_c0_g1_i4	Glyma.17G003900.2	99.12280702	244	19	462	2453	1	1986	0	2189

TRINITY_DN15281_c0_g1_i4	Glyma.17G003900.1	99.12280702	244	19	462	2453	1	1986	0	2189

TRINITY_DN15281_c0_g1_i5	Glyma.17G003900.2	98.7654321	244	19	676	2667	1	1986	0	2189

TRINITY_DN15281_c0_g1_i5	Glyma.17G003900.1	98.73417722	244	19	676	2667	1	1986	0	2189

TRINITY_DN14820_c0_g1_i4	Glyma.01G207500.1	95.22900763	210	6	75	2072	193	2190	0	2494

TRINITY_DN16379_c0_g2_i6	Glyma.10G057100.1	97.36842105	202	2	313	2310	2442	442	0	2556

TRINITY_DN16379_c0_g2_i6	Glyma.13G143600.1	97.8021978	200	1	313	2310	2436	436	0	2567

TRINITY_DN15007_c0_g1_i3	Glyma.11G242000.2	97.11111111	190	15	246	2233	1982	1	0	2436

TRINITY_DN15007_c0_g1_i9	Glyma.11G242000.2	96.77419355	190	15	246	2233	1982	1	0	2436

TRINITY_DN38405_c0_g1_i1	Glyma.12G062200.1	100	186	16	371	2341	1	1983	0	2355

TRINITY_DN16354_c0_g1_i7	Glyma.20G192700.1	97.54601227	181	1	823	2820	31	2031	0	2673

TRINITY_DN14820_c0_g1_i3	Glyma.11G035200.1	97.03703704	176	3	62	2058	1	2000	0	2687

TRINITY_DN15594_c0_g1_i4	Glyma.05G068600.2	97.22222222	172	5	1	2000	1991	1	0	2671

TRINITY_DN15594_c0_g1_i4	Glyma.05G068600.1	97.22222222	172	5	1	2000	1991	1	0	2671

TRINITY_DN11431_c0_g2_i2	Glyma.16G009200.1	96.15384615	170	1	93	2093	1998	1	0	2734

TRINITY_DN15529_c1_g1_i1	Glyma.05G232400.9	97.5	170	3	568	2565	2001	4	0	2715

TRINITY_DN15529_c1_g1_i1	Glyma.05G232400.8	95.23809524	170	3	568	2565	2001	4	0	2715

TRINITY_DN15529_c1_g1_i1	Glyma.05G232400.7	99.2481203	170	3	568	2565	2001	4	0	2715

TRINITY_DN15529_c1_g1_i1	Glyma.05G232400.6	99.2481203	170	3	568	2565	2001	4	0	2715

TRINITY_DN15529_c1_g1_i1	Glyma.05G232400.5	100	170	3	568	2565	2001	4	0	2715

TRINITY_DN15529_c1_g1_i1	Glyma.05G232400.3	96.20535714	170	3	568	2565	2001	4	0	2715

TRINITY_DN16379_c0_g2_i1	Glyma.10G057100.1	98.55072464	162	1	274	2271	2442	442	0	2778

TRINITY_DN16379_c0_g2_i1	Glyma.13G143600.1	98.56115108	159	1	274	2271	2436	436	0	2795

TRINITY_DN16983_c0_g1_i1	Glyma.07G213900.1	99.10802775	143	1	260	2257	19	2019	0	2883

TRINITY_DN15329_c0_g2_i6	Glyma.05G238700.2	97.22222222	138	0	481	2481	2068	68	0	2931

TRINITY_DN16217_c2_g1_i7	Glyma.04G220200.1	100	129	8	525	2521	2242	253	0	2883

TRINITY_DN5259_c0_g3_i1	Glyma.03G090000.1	100	112	0	98	2098	1	2001	0	3075

TRINITY_DN5259_c0_g3_i1	Glyma.03G090000.2	97.43589744	112	0	98	2098	1	2001	0	3075

TRINITY_DN14244_c0_g4_i2	Glyma.13G329100.2	96.19410086	106	0	186	2186	2001	1	0	3109

TRINITY_DN14244_c0_g4_i2	Glyma.13G329100.1	96.20253165	106	0	186	2186	2001	1	0	3109

TRINITY_DN15464_c0_g1_i4	Glyma.05G016700.1	95.8974359	104	3	187	2184	1	1983	0	2983

TRINITY_DN15464_c0_g1_i4	Glyma.05G016700.6	95.89905363	104	3	187	2184	1	1983	0	2983

TRINITY_DN16217_c2_g1_i3	Glyma.04G220200.1	100	84	8	525	2521	2242	253	0	3133

TRINITY_DN15634_c2_g1_i2	Glyma.19G192900.2	97.4025974	514	33	2284	4264	2339	359	5.22E-170	601

TRINITY_DN15634_c2_g1_i2	Glyma.19G192900.1	95.45454545	514	33	2284	4264	2339	359	5.22E-170	601

TRINITY_DN15634_c2_g1_i3	Glyma.19G192900.2	96.2962963	514	33	2395	4375	2339	359	5.35E-170	601

TRINITY_DN15634_c2_g1_i3	Glyma.19G192900.1	100	514	33	2395	4375	2339	359	5.35E-170	601

TRINITY_DN14561_c1_g1_i1	Glyma.05G197200.2	97.91666667	322	39	410	2375	2139	177	0	1450

TRINITY_DN14561_c1_g1_i1	Glyma.05G197200.1	97.91666667	322	39	410	2375	2139	177	0	1450

TRINITY_DN15200_c0_g1_i2	Glyma.16G205100.1	100	299	16	256	2237	2051	61	0	1862

TRINITY_DN16259_c0_g2_i2	Glyma.07G117800.1	97.69585253	296	36	3029	5008	2163	4124	0	1679

TRINITY_DN16259_c0_g2_i3	Glyma.07G117800.1	95.65217391	296	36	3035	5014	2163	4124	0	1679

TRINITY_DN16259_c0_g2_i2	Glyma.07G117800.2	99.42857143	296	36	3029	5008	2145	4106	0	1679

TRINITY_DN16259_c0_g2_i3	Glyma.07G117800.2	100	296	36	3035	5014	2145	4106	0	1679

TRINITY_DN14820_c0_g1_i3	Glyma.05G062200.1	98.79518072	244	4	62	2059	1	1998	0	2316

TRINITY_DN16102_c0_g2_i1	Glyma.12G234300.2	98.13953488	243	8	141	2136	1	1996	0	2296

TRINITY_DN16102_c0_g2_i1	Glyma.12G234300.1	98.13953488	243	8	141	2136	1	1996	0	2296

TRINITY_DN16102_c0_g2_i1	Glyma.13G264500.1	100	240	7	141	2136	1	1996	0	2313

TRINITY_DN36798_c0_g1_i1	Glyma.02G003800.1	99.06542056	240	36	294	2245	2193	209	0	1956

TRINITY_DN14820_c0_g1_i4	Glyma.11G035200.2	100	221	4	75	2072	1	1998	0	2444

TRINITY_DN9075_c0_g2_i1	Glyma.04G018900.1	100	212	39	71	1988	1989	1	0	1917

TRINITY_DN14645_c0_g1_i3	Glyma.15G165100.2	96.62921348	207	6	49	2036	2051	52	0	2470

TRINITY_DN14377_c0_g1_i2	Glyma.10G090600.2	100	190	14	522	2497	1	1994	0	2447

TRINITY_DN14377_c0_g1_i3	Glyma.10G090600.2	100	190	14	580	2555	1	1994	0	2447

TRINITY_DN14218_c0_g3_i1	Glyma.12G020300.1	100	162	7	211	2201	2067	74	0	2699

TRINITY_DN14218_c0_g3_i1	Glyma.11G093800.1	100	161	5	211	2201	2079	86	0	2704

TRINITY_DN12691_c0_g1_i2	Glyma.11G104500.4	97.34513274	155	5	226	2220	1995	1	0	2771

TRINITY_DN15907_c0_g2_i5	Glyma.01G008900.1	100	151	9	112	2091	1998	1	0	2715

TRINITY_DN15907_c0_g2_i5	Glyma.01G008900.2	96.20253165	151	9	112	2091	1998	1	0	2715

TRINITY_DN15549_c0_g1_i7	Glyma.05G247000.3	96.62921348	150	12	837	2806	2814	821	0	2630

TRINITY_DN14820_c0_g1_i2	Glyma.11G035200.2	98.7012987	147	4	62	2059	1	1998	0	2854

TRINITY_DN15944_c1_g1_i4	Glyma.01G210600.1	96.14873838	134	2	521	2517	2305	309	0	2913

TRINITY_DN6136_c0_g1_i1	Glyma.10G041300.2	95.76719577	305	34	258	2237	2163	196	0	1679

TRINITY_DN6136_c0_g2_i1	Glyma.10G041300.2	100	305	34	257	2236	2163	196	0	1679

TRINITY_DN14820_c0_g1_i1	Glyma.11G035200.2	99.38650307	248	4	75	2071	1	1997	0	2292

TRINITY_DN15944_c1_g1_i2	Glyma.01G210600.2	96.51972158	247	3	621	2618	2303	309	0	2290

TRINITY_DN13713_c0_g1_i1	Glyma.15G042700.8	97.56097561	222	9	296	2281	1	1989	0	2313

TRINITY_DN13097_c0_g1_i2	Glyma.02G292300.1	98.9010989	189	9	194	2184	2091	98	0	2560

TRINITY_DN13097_c0_g1_i2	Glyma.14G022300.1	100	187	9	194	2184	2097	104	0	2571

TRINITY_DN7043_c0_g1_i1	Glyma.01G204900.1	100	187	14	890	2871	1259	3246	0	2475

TRINITY_DN16345_c0_g1_i8	Glyma.08G044700.3	98.30508475	170	5	1553	3549	2481	491	0	2686

TRINITY_DN16345_c0_g1_i8	Glyma.08G044700.7	98.30508475	170	5	1553	3549	2202	212	0	2686

TRINITY_DN418_c0_g1_i1	Glyma.09G267800.1	97.87234043	151	8	23	1994	9	2001	0	2641

TRINITY_DN13702_c0_g1_i1	Glyma.05G236700.1	100	117	6	323	2314	1	1998	0	2992

TRINITY_DN6026_c0_g1_i1	Glyma.06G077600.1	97.28813559	439	57	289	2244	187	2160	0	832

TRINITY_DN10651_c0_g1_i1	Glyma.18G003800.1	97.4137931	273	39	213	2171	3738	1758	0	1816

TRINITY_DN14314_c0_g1_i1	Glyma.08G245700.1	100	253	23	199	2181	1	1974	0	2036

TRINITY_DN14510_c0_g1_i1	Glyma.16G041600.1	96.57534247	217	8	177	2156	1992	1	0	2333

TRINITY_DN21554_c0_g1_i1	Glyma.17G140800.1	100	208	8	201	2187	2283	288	0	2453

TRINITY_DN11529_c1_g2_i2	Glyma.11G024700.1	95.8677686	199	6	278	2266	1992	1	0	2490

TRINITY_DN13097_c0_g1_i2	Glyma.02G292300.3	96.8	189	9	194	2183	2088	96	0	2558

TRINITY_DN13097_c0_g1_i2	Glyma.02G292300.2	99.41176471	189	9	194	2183	2088	96	0	2558

TRINITY_DN13097_c0_g1_i2	Glyma.14G022300.4	98.9010989	187	9	194	2183	2094	102	0	2569

TRINITY_DN16379_c0_g2_i1	Glyma.03G182800.1	98.56850716	186	3	274	2271	2427	436	0	2621

TRINITY_DN12607_c0_g1_i1	Glyma.17G065700.1	97.05882353	186	10	1546	3527	1301	3291	0	2510

TRINITY_DN14820_c0_g1_i1	Glyma.05G062200.1	100	181	0	75	2072	1	1998	0	2687

TRINITY_DN16354_c0_g1_i7	Glyma.20G192700.4	97.54098361	180	1	826	2820	1	1998	0	2673

TRINITY_DN16354_c0_g1_i7	Glyma.20G192700.5	97.5308642	180	1	826	2820	1	1998	0	2673

TRINITY_DN16354_c0_g1_i7	Glyma.20G192700.3	97.7443609	180	1	826	2820	1	1998	0	2673

TRINITY_DN16354_c0_g1_i7	Glyma.20G192700.2	97.7443609	180	1	826	2820	1	1998	0	2673

TRINITY_DN14820_c0_g1_i3	Glyma.11G035200.2	98.7804878	176	2	62	2058	1	1997	0	2702

TRINITY_DN16379_c0_g2_i1	Glyma.19G183400.1	98.55072464	176	4	274	2271	2427	436	0	2676

TRINITY_DN16379_c0_g2_i6	Glyma.03G182800.1	97.8021978	141	3	313	2310	2427	436	0	2870

TRINITY_DN16379_c0_g2_i6	Glyma.19G183400.1	97.82608696	139	4	313	2310	2427	436	0	2881

TRINITY_DN15841_c0_g1_i5	Glyma.U033300.3	99.47089947	138	3	604	2601	2	1987	0	2848

TRINITY_DN15841_c0_g1_i6	Glyma.U033300.3	100	138	3	604	2601	2	1987	0	2848

TRINITY_DN15281_c0_g1_i1	Glyma.15G122600.1	96.84813754	138	11	462	2453	1	1974	0	2732

TRINITY_DN15281_c0_g1_i6	Glyma.15G122600.1	96.39065817	138	11	676	2667	1	1974	0	2732

TRINITY_DN40621_c0_g1_i1	Glyma.03G090100.1	97.46136865	135	1	184	2181	1995	1	0	2922

TRINITY_DN40621_c0_g1_i1	Glyma.16G082900.1	100	132	1	184	2181	1995	1	0	2939

TRINITY_DN40621_c0_g1_i1	Glyma.16G082900.2	100	132	1	184	2181	1995	1	0	2939

TRINITY_DN40621_c0_g1_i1	Glyma.16G082900.4	100	132	1	184	2181	1995	1	0	2939

TRINITY_DN40621_c0_g1_i1	Glyma.16G082900.3	97.46031746	132	1	184	2181	1995	1	0	2939

TRINITY_DN15048_c0_g2_i1	Glyma.17G030600.1	98.8372093	333	32	584	2558	2025	54	0	1539

TRINITY_DN16354_c0_g1_i7	Glyma.08G027000.1	97.5308642	324	7	826	2816	1	1994	0	1834

TRINITY_DN16432_c1_g2_i1	Glyma.20G204500.1	97.19626168	292	9	244	2233	20	2012	0	1999

TRINITY_DN10651_c0_g1_i1	Glyma.11G253200.1	96.32352941	281	27	213	2171	3741	1758	0	1801

TRINITY_DN15878_c0_g2_i1	Glyma.19G260900.1	100	281	40	60	2027	2065	106	0	1705

TRINITY_DN15970_c0_g2_i11	Glyma.07G137300.1	100	266	8	230	2206	166	2160	0	2073

TRINITY_DN16279_c0_g3_i2	Glyma.18G098400.4	96.06299213	255	11	200	2191	31	2019	0	2191

TRINITY_DN16279_c0_g3_i2	Glyma.18G098400.1	96.06299213	255	11	200	2191	31	2019	0	2191

TRINITY_DN16279_c0_g3_i2	Glyma.18G098400.2	96.06299213	254	12	200	2191	31	2013	0	2158

TRINITY_DN16279_c0_g3_i2	Glyma.18G098400.3	96.06299213	254	12	200	2191	31	2013	0	2158

TRINITY_DN16259_c0_g2_i1	Glyma.07G117800.1	100	247	34	3035	5011	2163	4124	0	1964

TRINITY_DN16259_c0_g2_i1	Glyma.07G117800.2	97.71863118	247	34	3035	5011	2145	4106	0	1964

TRINITY_DN15200_c0_g1_i3	Glyma.20G212800.1	98.7654321	222	3	252	2247	2034	42	0	2425

TRINITY_DN21719_c0_g1_i1	Glyma.04G255100.5	95.12195122	212	7	1	1970	1997	1	0	2338

TRINITY_DN21719_c0_g1_i1	Glyma.04G255100.3	95.12195122	212	7	1	1970	1997	1	0	2338

TRINITY_DN21719_c0_g1_i1	Glyma.04G255100.2	95.12195122	212	7	1	1970	1997	1	0	2338

TRINITY_DN15281_c0_g1_i4	Glyma.15G122600.1	99.12280702	182	9	462	2453	1	1974	0	2499

TRINITY_DN15281_c0_g1_i5	Glyma.15G122600.1	99.12280702	182	9	676	2667	1	1974	0	2499

TRINITY_DN14820_c0_g1_i3	Glyma.01G207500.1	98.68421053	168	2	54	2049	185	2180	0	2745

TRINITY_DN11819_c0_g3_i3	Glyma.16G048700.2	96.75090253	155	13	302	2278	1	1995	0	2687

TRINITY_DN15944_c1_g1_i4	Glyma.01G210600.2	100	134	1	521	2517	2302	309	0	2926

TRINITY_DN15981_c0_g1_i10	Glyma.11G048300.3	98.88888889	121	3	1201	3191	1997	1	0	2979

TRINITY_DN15981_c0_g1_i14	Glyma.11G048300.3	96.41025641	121	3	1075	3065	1997	1	0	2979

TRINITY_DN15981_c0_g1_i20	Glyma.11G048300.3	96.72897196	121	3	1075	3065	1997	1	0	2979

TRINITY_DN15981_c0_g1_i8	Glyma.11G048300.3	96.96969697	121	3	1201	3191	1997	1	0	2979

TRINITY_DN15048_c0_g2_i1	Glyma.07G243300.1	98.75	332	26	584	2558	2031	54	0	1594

TRINITY_DN14314_c0_g1_i1	Glyma.08G246300.1	97.59036145	289	29	199	2181	1	1962	0	1781

TRINITY_DN14820_c0_g1_i2	Glyma.05G062200.1	98.41269841	270	4	62	2055	1	1994	0	2165

TRINITY_DN15617_c0_g2_i2	Glyma.18G065600.1	100	256	18	109	2081	330	2323	0	2106

TRINITY_DN15759_c0_g2_i1	Glyma.04G231900.2	99.17355372	252	16	15	2005	1979	1	0	2148

TRINITY_DN15759_c0_g2_i1	Glyma.04G231900.1	97.6744186	252	16	15	2005	1979	1	0	2148

TRINITY_DN15759_c0_g2_i4	Glyma.04G231900.2	100	252	16	15	2005	1979	1	0	2148

TRINITY_DN15759_c0_g2_i4	Glyma.04G231900.1	100	252	16	15	2005	1979	1	0	2148

TRINITY_DN16432_c1_g2_i7	Glyma.20G204500.1	95.47738693	226	8	244	2233	20	2012	0	2375

TRINITY_DN11529_c1_g2_i2	Glyma.01G218800.1	99.15254237	214	4	278	2266	1992	1	0	2429

TRINITY_DN9850_c0_g1_i1	Glyma.18G207500.1	100	204	1	110	2091	47	2042	0	2466

TRINITY_DN15281_c0_g1_i4	Glyma.09G017000.1	99.12280702	190	8	462	2453	1	1974	0	2466

TRINITY_DN15281_c0_g1_i5	Glyma.09G017000.1	100	190	8	676	2667	1	1974	0	2466

TRINITY_DN15200_c0_g1_i4	Glyma.20G212800.1	97.18309859	189	1	252	2247	2034	42	0	2619

TRINITY_DN15228_c0_g2_i1	Glyma.03G261300.3	100	187	7	111	2076	30	2022	0	2436

TRINITY_DN15228_c0_g2_i1	Glyma.03G261300.2	100	187	7	111	2076	30	2022	0	2436

TRINITY_DN13519_c0_g1_i1	Glyma.19G160200.3	96.66666667	181	14	175	2160	1	1971	0	2457

TRINITY_DN13519_c0_g1_i1	Glyma.19G160200.1	96.68246445	181	14	175	2160	1	1971	0	2457

TRINITY_DN14790_c0_g1_i1	Glyma.08G329200.6	95.77464789	175	44	657	2623	1925	1	0	2071

TRINITY_DN14790_c0_g1_i1	Glyma.08G329200.5	95.83333333	175	44	657	2623	1925	1	0	2071

TRINITY_DN14790_c0_g1_i1	Glyma.08G329200.3	95.77167019	175	44	657	2623	1925	1	0	2071

TRINITY_DN14790_c0_g1_i1	Glyma.08G329200.2	95.78947368	175	44	657	2623	1925	1	0	2071

TRINITY_DN14790_c0_g1_i1	Glyma.08G329200.1	95.78947368	175	44	657	2623	1925	1	0	2071

TRINITY_DN14790_c0_g1_i1	Glyma.08G329200.4	95.78947368	175	44	657	2623	1925	1	0	2071

TRINITY_DN14790_c0_g1_i2	Glyma.08G329200.6	95.89041096	175	44	657	2623	1925	1	0	2071

TRINITY_DN14790_c0_g1_i2	Glyma.08G329200.5	95.89632829	175	44	657	2623	1925	1	0	2071

TRINITY_DN14790_c0_g1_i2	Glyma.08G329200.3	95.91836735	175	44	657	2623	1925	1	0	2071

TRINITY_DN14790_c0_g1_i2	Glyma.08G329200.2	95.88477366	175	44	657	2623	1925	1	0	2071

TRINITY_DN14790_c0_g1_i2	Glyma.08G329200.1	95.8974359	175	44	657	2623	1925	1	0	2071

TRINITY_DN14790_c0_g1_i2	Glyma.08G329200.4	95.90163934	175	44	657	2623	1925	1	0	2071

TRINITY_DN3187_c0_g1_i1	Glyma.18G128600.1	100	167	17	170	2126	1	1987	0	2451

TRINITY_DN15560_c0_g1_i2	Glyma.10G037500.1	98.31775701	164	6	280	2273	9	1992	0	2687

TRINITY_DN16164_c1_g1_i1	Glyma.04G241900.2	100	162	11	65	2054	759	2736	0	2634

TRINITY_DN16164_c1_g1_i1	Glyma.04G241900.1	97.39130435	162	11	65	2054	759	2736	0	2634

TRINITY_DN16164_c1_g1_i3	Glyma.04G241900.2	99.07407407	157	11	65	2054	759	2736	0	2662

TRINITY_DN16164_c1_g1_i3	Glyma.04G241900.1	99.07407407	157	11	65	2054	759	2736	0	2662

TRINITY_DN16102_c0_g2_i1	Glyma.08G148100.1	98.33333333	150	0	141	2136	1	1996	0	2856

TRINITY_DN15907_c0_g2_i5	Glyma.16G077500.1	99.30555556	150	7	113	2091	1994	1	0	2732

TRINITY_DN16102_c0_g2_i1	Glyma.15G210300.1	96.46017699	139	0	141	2136	1	1996	0	2916

TRINITY_DN16102_c0_g2_i1	Glyma.15G210300.3	100	139	0	141	2136	1	1996	0	2916

TRINITY_DN16102_c0_g2_i1	Glyma.15G210300.2	98.33333333	139	0	141	2136	1	1996	0	2916

TRINITY_DN2707_c0_g1_i1	Glyma.12G167300.1	97.76119403	87	0	1	1996	600	2595	0	3205

TRINITY_DN15759_c0_g2_i1	Glyma.06G133000.3	97.6744186	267	14	15	2005	1979	1	0	2076

TRINITY_DN15759_c0_g2_i1	Glyma.06G133000.2	100	267	14	15	2005	1979	1	0	2076

TRINITY_DN15759_c0_g2_i1	Glyma.06G133000.1	100	267	14	15	2005	1979	1	0	2076

TRINITY_DN15759_c0_g2_i4	Glyma.06G133000.3	99.4011976	267	14	15	2005	1979	1	0	2076

TRINITY_DN15759_c0_g2_i4	Glyma.06G133000.2	100	267	14	15	2005	1979	1	0	2076

TRINITY_DN15759_c0_g2_i4	Glyma.06G133000.1	100	267	14	15	2005	1979	1	0	2076

TRINITY_DN16432_c1_g2_i5	Glyma.20G204500.1	98.49246231	260	6	244	2233	20	2012	0	2198

TRINITY_DN12659_c0_g1_i1	Glyma.12G226300.1	98.11320755	250	18	269	2255	58	2029	0	2098

TRINITY_DN15302_c0_g1_i1	Glyma.17G067800.2	100	247	16	377	2354	1	1993	0	2193

TRINITY_DN15302_c0_g1_i1	Glyma.17G067800.1	97.5	247	16	377	2354	1	1993	0	2193

TRINITY_DN15944_c1_g1_i2	Glyma.11G031400.3	97.86700125	246	5	617	2609	2301	318	0	2237

TRINITY_DN15944_c1_g1_i2	Glyma.11G031400.1	95.6284153	246	5	617	2609	2301	318	0	2237

TRINITY_DN21554_c0_g1_i1	Glyma.05G058400.1	100	224	8	201	2186	2280	289	0	2366

TRINITY_DN15200_c0_g1_i3	Glyma.10G177300.1	98.33333333	216	7	252	2243	2034	46	0	2429

TRINITY_DN14244_c0_g4_i2	Glyma.U021500.1	95.57713053	215	7	186	2178	1998	9	0	2447

TRINITY_DN14510_c0_g1_i1	Glyma.19G110800.1	96.57534247	182	15	177	2156	1980	1	0	2483

TRINITY_DN11529_c1_g2_i3	Glyma.08G013200.1	95.07042254	153	2	278	2272	1989	1	0	2798

TRINITY_DN11529_c1_g2_i3	Glyma.08G013200.2	95.07042254	153	2	278	2272	1989	1	0	2798

TRINITY_DN15281_c0_g1_i1	Glyma.09G017000.1	96.84813754	153	6	462	2453	1	1974	0	2682

TRINITY_DN15281_c0_g1_i6	Glyma.09G017000.1	98.2300885	153	6	676	2667	1	1974	0	2682

TRINITY_DN11529_c1_g2_i3	Glyma.05G206300.1	95.07119387	138	7	278	2272	1971	1	0	2765

TRINITY_DN14156_c0_g1_i3	Glyma.02G298200.1	100	126	0	361	2355	2	1996	0	2987

TRINITY_DN9320_c0_g2_i1	Glyma.11G090200.1	97.24770642	123	2	84	2075	1	1983	0	2905

TRINITY_DN14156_c0_g1_i3	Glyma.14G015600.2	96.49122807	112	0	361	2355	2	1996	0	3064

TRINITY_DN13197_c0_g1_i1	Glyma.19G212100.2	98.20359281	328	22	1445	3430	988	2958	0	1664

TRINITY_DN39405_c0_g1_i1	Glyma.16G203300.3	98.81305638	311	8	309	2294	1992	1	0	1895

TRINITY_DN2577_c0_g1_i1	Glyma.10G000400.1	99.21259843	304	44	268	2242	1953	12	0	1539

TRINITY_DN16432_c1_g2_i1	Glyma.20G204500.2	95.34883721	293	7	244	2233	20	2009	0	2008

TRINITY_DN14314_c0_g1_i1	Glyma.18G266900.1	98.96907216	246	26	199	2181	1	1962	0	2041

TRINITY_DN11169_c0_g1_i1	Glyma.10G253000.1	100	219	27	266	2238	13	1968	0	2087

TRINITY_DN12713_c0_g1_i1	Glyma.11G187500.1	99.48051948	212	5	8	1988	2116	124	0	2418

TRINITY_DN14820_c0_g1_i4	Glyma.05G062200.1	96.875	207	0	75	2068	1	1994	0	2536

TRINITY_DN2707_c0_g1_i1	Glyma.16G003300.1	95.14285714	110	4	3	1996	590	2559	0	2918

TRINITY_DN13197_c0_g1_i1	Glyma.19G212100.1	98.20359281	332	19	1445	3430	988	2961	0	1674

TRINITY_DN2577_c0_g1_i1	Glyma.10G000400.2	99.21259843	302	44	268	2242	1950	12	0	1543

TRINITY_DN11529_c1_g2_i2	Glyma.08G013200.1	100	246	5	278	2266	1989	1	0	2266

TRINITY_DN11529_c1_g2_i2	Glyma.08G013200.2	100	246	5	278	2266	1989	1	0	2266

TRINITY_DN15736_c0_g1_i1	Glyma.05G198700.1	95.215311	228	3	266	2256	250	2240	0	2392

TRINITY_DN16432_c1_g2_i7	Glyma.20G204500.2	100	227	6	244	2233	20	2009	0	2385

TRINITY_DN13713_c0_g1_i6	Glyma.15G042700.1	99.23664122	227	12	296	2264	1	1992	0	2261

TRINITY_DN16194_c1_g1_i6	Glyma.06G109800.1	98.71794872	206	27	440	2362	288	2268	0	2010

TRINITY_DN16194_c1_g1_i6	Glyma.06G109800.3	98.5	206	27	440	2362	288	2268	0	2010

TRINITY_DN16194_c1_g1_i6	Glyma.06G109800.2	95.97585513	206	27	440	2362	198	2178	0	2010

TRINITY_DN15200_c0_g1_i4	Glyma.10G177300.1	95.04132231	185	3	252	2243	2034	46	0	2623

TRINITY_DN16164_c1_g1_i1	Glyma.06G121500.1	100	164	10	68	2054	912	2886	0	2617

TRINITY_DN16164_c1_g1_i1	Glyma.06G121500.4	97.5	164	10	68	2054	888	2862	0	2617

TRINITY_DN16164_c1_g1_i1	Glyma.06G121500.2	97.39130435	164	10	68	2054	759	2733	0	2617

TRINITY_DN16164_c1_g1_i3	Glyma.06G121500.1	98.20359281	159	10	68	2054	912	2886	0	2645

TRINITY_DN16164_c1_g1_i3	Glyma.06G121500.4	96.4556962	159	10	68	2054	888	2862	0	2645

TRINITY_DN16164_c1_g1_i3	Glyma.06G121500.2	97.84313725	159	10	68	2054	759	2733	0	2645

TRINITY_DN15944_c1_g1_i4	Glyma.11G031400.3	100	137	1	516	2508	2301	318	0	2863

TRINITY_DN15944_c1_g1_i4	Glyma.11G031400.1	100	137	1	516	2508	2301	318	0	2863

TRINITY_DN259_c0_g1_i1	Glyma.18G105600.1	99.60474308	125	2	162	2153	1	1992	0	2976

TRINITY_DN14314_c0_g1_i2	Glyma.13G048800.1	100	454	55	215	2166	191	2154	0	725

TRINITY_DN15048_c0_g1_i2	Glyma.09G040000.1	95.62937063	343	25	889	2865	2022	55	0	1526

TRINITY_DN14314_c0_g1_i1	Glyma.18G267100.1	98.30508475	279	25	203	2181	11	1968	0	1829

TRINITY_DN16432_c1_g2_i5	Glyma.20G204500.2	100	261	4	244	2233	20	2009	0	2207

TRINITY_DN9058_c0_g1_i1	Glyma.09G191700.2	95.59322034	162	0	164	2155	1	1992	0	2782

TRINITY_DN14452_c0_g1_i2	Glyma.07G015500.3	100	159	12	266	2236	1	1980	0	2584

TRINITY_DN14452_c0_g1_i2	Glyma.07G015500.1	96.01769912	159	12	266	2236	1	1980	0	2584

TRINITY_DN5598_c0_g1_i1	Glyma.14G012200.1	97.31543624	159	15	25	2010	349	2322	0	2643

TRINITY_DN16414_c0_g1_i3	Glyma.11G017900.1	96.23762376	157	2	142	2130	1992	1	0	2789

TRINITY_DN16414_c0_g1_i4	Glyma.11G017900.1	96.25	155	2	142	2130	1992	1	0	2800

TRINITY_DN16414_c0_g1_i11	Glyma.11G017900.1	98.77300613	154	2	334	2322	1992	1	0	2806

TRINITY_DN16414_c0_g1_i10	Glyma.11G017900.1	96.42857143	151	2	334	2322	1992	1	0	2822

TRINITY_DN11604_c0_g1_i1	Glyma.04G229200.1	100	141	2	514	2499	1992	1	0	2859

TRINITY_DN14409_c0_g1_i2	Glyma.12G063700.1	96.27192982	141	3	108	2093	1	1989	0	2839

TRINITY_DN7387_c0_g1_i3	Glyma.10G218000.1	100	131	10	209	2182	1980	1	0	2760

TRINITY_DN11604_c0_g1_i1	Glyma.06G135600.1	97.97752809	127	3	514	2499	1992	1	0	2937

TRINITY_DN9320_c0_g2_i1	Glyma.01G154400.1	96.23430962	117	1	84	2075	1	1980	0	2953

TRINITY_DN259_c0_g1_i1	Glyma.08G310300.2	96.05263158	116	0	162	2153	1	1992	0	3037

TRINITY_DN259_c0_g1_i1	Glyma.08G310300.1	96.05263158	116	0	162	2153	1	1992	0	3037

TRINITY_DN6136_c0_g1_i1	Glyma.13G128200.1	95.76868829	327	22	258	2237	2127	160	0	1646

TRINITY_DN6136_c0_g2_i1	Glyma.13G128200.1	98.55072464	327	22	257	2236	2127	160	0	1646

TRINITY_DN6136_c0_g1_i1	Glyma.13G128200.3	95.76868829	327	22	258	2237	2091	124	0	1646

TRINITY_DN6136_c0_g2_i1	Glyma.13G128200.3	100	327	22	257	2236	2091	124	0	1646

TRINITY_DN2903_c0_g1_i1	Glyma.10G169000.8	96.65809769	267	32	7	1953	88	2064	0	1823

TRINITY_DN2903_c0_g1_i1	Glyma.10G169000.2	95.65217391	267	32	7	1953	88	2064	0	1823

TRINITY_DN2903_c0_g1_i1	Glyma.10G169000.3	95.65217391	267	32	7	1953	88	2064	0	1823

TRINITY_DN2903_c0_g1_i1	Glyma.10G169000.4	97.04968944	267	32	7	1953	88	2064	0	1823

TRINITY_DN2903_c0_g1_i1	Glyma.10G169000.5	97.27891156	267	32	7	1953	88	2064	0	1823

TRINITY_DN2903_c0_g1_i1	Glyma.10G169000.6	97.72727273	267	32	7	1953	88	2064	0	1823

TRINITY_DN2903_c0_g1_i1	Glyma.10G169000.1	95.40229885	267	32	7	1953	88	2064	0	1823

TRINITY_DN2903_c0_g1_i1	Glyma.10G169000.7	99.05660377	267	32	7	1953	88	2064	0	1823

TRINITY_DN2903_c0_g2_i1	Glyma.10G169000.8	95.45454545	267	32	7	1953	88	2064	0	1823

TRINITY_DN2903_c0_g2_i1	Glyma.10G169000.2	95.45454545	267	32	7	1953	88	2064	0	1823

TRINITY_DN2903_c0_g2_i1	Glyma.10G169000.3	95.45454545	267	32	7	1953	88	2064	0	1823

TRINITY_DN2903_c0_g2_i1	Glyma.10G169000.4	97.08737864	267	32	7	1953	88	2064	0	1823

TRINITY_DN2903_c0_g2_i1	Glyma.10G169000.5	97.08737864	267	32	7	1953	88	2064	0	1823

TRINITY_DN2903_c0_g2_i1	Glyma.10G169000.6	100	267	32	7	1953	88	2064	0	1823

TRINITY_DN2903_c0_g2_i1	Glyma.10G169000.1	98.85057471	267	32	7	1953	88	2064	0	1823

TRINITY_DN2903_c0_g2_i1	Glyma.10G169000.7	96.68874172	267	32	7	1953	88	2064	0	1823

TRINITY_DN14820_c0_g1_i1	Glyma.01G207500.1	100	236	6	75	2062	193	2180	0	2331

TRINITY_DN16217_c2_g1_i3	Glyma.05G183100.1	100	227	13	523	2499	2271	286	0	2296

TRINITY_DN16151_c0_g2_i2	Glyma.08G293000.1	98.50746269	217	30	253	2225	1	1943	0	2049

TRINITY_DN12713_c0_g1_i1	Glyma.11G187500.2	95.45454545	215	4	8	1988	2113	124	0	2414

TRINITY_DN16151_c0_g2_i2	Glyma.08G293000.2	95.38461538	215	32	253	2225	1	1925	0	1943

TRINITY_DN14561_c1_g1_i1	Glyma.04G206100.1	95.07042254	199	20	410	2390	2100	129	0	2386

TRINITY_DN15159_c0_g1_i1	Glyma.11G199700.2	98.63013699	194	7	280	2260	2013	27	0	2512

TRINITY_DN15159_c0_g1_i1	Glyma.11G199700.3	97.7443609	194	7	280	2260	2013	27	0	2512

TRINITY_DN3370_c0_g1_i1	Glyma.08G187400.1	100	174	32	733	2676	1986	1	0	2377

TRINITY_DN8924_c0_g1_i1	Glyma.12G020900.2	99.42857143	160	3	2	1988	721	2710	0	2758

TRINITY_DN143_c0_g1_i1	Glyma.05G193900.1	98.87640449	146	5	333	2321	2354	367	0	2835

TRINITY_DN15659_c0_g2_i1	Glyma.08G064600.3	99.18032787	138	6	202	2185	12	1993	0	2809

TRINITY_DN15659_c0_g2_i1	Glyma.08G064600.2	100	138	6	202	2185	12	1993	0	2809

TRINITY_DN15659_c0_g2_i3	Glyma.08G064600.3	98.67256637	138	6	125	2108	12	1993	0	2809

TRINITY_DN15659_c0_g2_i3	Glyma.08G064600.2	100	138	6	125	2108	12	1993	0	2809

TRINITY_DN3419_c0_g1_i1	Glyma.18G092600.1	100	135	0	5	1995	710	2700	0	2929

TRINITY_DN10874_c0_g1_i1	Glyma.14G000500.1	100	117	0	289	2279	236	2226	0	3029

TRINITY_DN10874_c0_g1_i4	Glyma.14G000500.1	97.07317073	117	0	283	2273	236	2226	0	3029

TRINITY_DN15415_c0_g1_i3	Glyma.18G143600.2	100	102	2	70	2060	105	2092	0	3092

TRINITY_DN5133_c0_g1_i1	Glyma.03G237600.1	100	102	8	101	2070	1	1988	0	2957

TRINITY_DN9565_c0_g1_i1	Glyma.19G083600.1	99.01960784	301	58	475	2425	1931	9	0	1323

TRINITY_DN15619_c0_g2_i1	Glyma.06G181900.1	97.82608696	289	14	1193	3164	187	2172	0	1932

TRINITY_DN14566_c0_g1_i2	Glyma.20G243500.1	99.46524064	275	2	93	2081	235	2223	0	2139

TRINITY_DN14566_c0_g1_i2	Glyma.10G293500.1	99.31972789	273	2	93	2081	241	2229	0	2150

TRINITY_DN14314_c0_g1_i2	Glyma.08G245700.1	98.5915493	264	20	199	2169	1	1974	0	1986

TRINITY_DN16061_c0_g1_i2	Glyma.09G266500.1	95.3271028	256	17	548	2527	1993	20	0	2089

TRINITY_DN13713_c0_g1_i6	Glyma.15G042700.8	98.50746269	227	11	296	2264	1	1989	0	2276

TRINITY_DN15228_c0_g2_i1	Glyma.19G260400.4	100	192	15	109	2076	19	2001	0	2423

TRINITY_DN13072_c0_g1_i1	Glyma.17G249600.1	95.12195122	192	19	134	2089	1	1980	0	2327

TRINITY_DN15501_c0_g2_i6	Glyma.07G028500.1	98.9010989	175	8	2	1987	2141	156	0	2654

TRINITY_DN15501_c0_g2_i6	Glyma.08G214300.1	98.55072464	173	7	1	1987	2141	156	0	2671

TRINITY_DN15329_c0_g2_i4	Glyma.02G085000.4	98.8005997	172	9	512	2499	2034	68	0	2560

TRINITY_DN16374_c1_g1_i2	Glyma.16G035100.1	100	156	17	1616	3580	4657	6612	0	2429

TRINITY_DN14566_c0_g1_i1	Glyma.20G243500.1	97.72727273	144	2	93	2081	235	2223	0	2865

TRINITY_DN14409_c0_g1_i2	Glyma.11G140100.1	98.61111111	139	4	108	2093	1	1986	0	2854

TRINITY_DN9431_c0_g1_i1	Glyma.08G030800.1	100	110	1	1	1990	340	2325	0	3040

TRINITY_DN6136_c0_g1_i1	Glyma.13G128200.2	99.28057554	327	22	258	2235	2013	48	0	1642

TRINITY_DN6136_c0_g2_i1	Glyma.13G128200.2	98.80952381	327	22	257	2234	2013	48	0	1642

TRINITY_DN14314_c0_g1_i1	Glyma.08G245600.1	98.48484848	267	29	206	2181	5	1959	0	1890

TRINITY_DN16257_c0_g1_i1	Glyma.12G096900.1	95.74036511	257	7	95	2077	2241	256	0	2191

TRINITY_DN11529_c1_g2_i2	Glyma.05G206300.1	97.63779528	239	11	278	2266	1971	1	0	2233

TRINITY_DN7043_c0_g1_i1	Glyma.11G038000.1	97.51243781	205	10	890	2871	1343	3321	0	2427

TRINITY_DN10651_c0_g2_i1	Glyma.11G253200.1	100	172	4	213	2185	3741	1757	0	2591

TRINITY_DN13197_c0_g1_i2	Glyma.07G056000.1	100	159	0	931	2919	471	2459	0	2793

TRINITY_DN14566_c0_g1_i1	Glyma.10G293500.1	97.72727273	158	0	93	2081	241	2229	0	2798

TRINITY_DN16061_c0_g1_i2	Glyma.07G081600.1	100	155	2	546	2531	1998	13	0	2776

TRINITY_DN16061_c0_g1_i2	Glyma.07G081600.2	95.3271028	155	2	546	2531	1998	13	0	2776

TRINITY_DN9001_c0_g1_i1	Glyma.05G144700.1	98.07692308	150	0	72	2060	1	1989	0	2843

TRINITY_DN9001_c0_g1_i1	Glyma.08G101100.1	98.07692308	144	0	72	2060	1	1989	0	2876

TRINITY_DN2365_c0_g1_i1	Glyma.09G023400.2	95.8	133	1	372	2357	1	1989	0	2916

TRINITY_DN2365_c0_g1_i1	Glyma.09G023400.1	95.6937799	133	1	372	2357	1	1989	0	2916

TRINITY_DN2365_c0_g2_i1	Glyma.09G023400.2	95.83333333	133	1	180	2165	1	1989	0	2916

TRINITY_DN2365_c0_g2_i1	Glyma.09G023400.1	98.22485207	133	1	180	2165	1	1989	0	2916

TRINITY_DN15633_c0_g1_i5	Glyma.05G209700.1	99.50738916	108	3	140	2125	1	1986	0	3037

TRINITY_DN15633_c0_g1_i5	Glyma.08G016400.1	95.65217391	106	3	140	2125	1	1986	0	3048

TRINITY_DN15048_c0_g1_i2	Glyma.15G145200.1	99.06103286	323	29	889	2859	2019	61	0	1585

TRINITY_DN9565_c0_g1_i1	Glyma.16G062700.2	99.01960784	304	62	475	2426	1925	8	0	1303

TRINITY_DN9565_c0_g1_i1	Glyma.16G062700.1	98.7804878	304	62	475	2426	1925	8	0	1303

TRINITY_DN13903_c1_g1_i2	Glyma.10G184300.1	96.88195991	296	44	70	2024	1264	3209	0	1546

TRINITY_DN16385_c0_g2_i1	Glyma.08G186800.1	98.27586207	225	24	332	2293	406	2376	0	2146

TRINITY_DN10090_c1_g1_i1	Glyma.18G185500.1	98.79518072	198	2	367	2354	2025	41	0	2555

TRINITY_DN15135_c0_g1_i2	Glyma.17G104500.1	96.12903226	194	18	433	2403	2043	70	0	2396

TRINITY_DN16217_c2_g1_i7	Glyma.05G183100.1	100	185	9	523	2499	2271	286	0	2562

TRINITY_DN16374_c1_g1_i4	Glyma.16G035100.1	100	173	17	4589	6551	4659	6612	0	2331

TRINITY_DN16374_c1_g1_i6	Glyma.16G035100.1	100	173	17	4383	6345	4659	6612	0	2331

TRINITY_DN10651_c0_g2_i1	Glyma.18G003800.1	96.72131148	169	12	213	2185	3738	1757	0	2599

TRINITY_DN11155_c0_g1_i1	Glyma.05G140800.2	98.35916135	162	0	378	2365	1988	1	0	2774

TRINITY_DN13640_c1_g2_i7	Glyma.09G255400.1	95.13888889	149	13	441	2379	2982	1002	0	2484

TRINITY_DN15329_c0_g2_i4	Glyma.05G238700.1	98.79518072	132	1	512	2499	2037	68	0	2824

TRINITY_DN15007_c0_g1_i10	Glyma.11G242000.2	95	120	1	664	2651	1979	1	0	2948

TRINITY_DN15007_c0_g1_i7	Glyma.11G242000.2	98.80952381	120	1	664	2651	1979	1	0	2948

TRINITY_DN13097_c0_g1_i2	Glyma.18G094400.1	95.0166113	323	34	169	2116	2129	169	0	1459

TRINITY_DN15619_c0_g2_i1	Glyma.06G181900.2	96.58536585	289	12	1193	3164	187	2169	0	1945

TRINITY_DN15970_c0_g2_i11	Glyma.18G188000.2	100	267	8	230	2212	31	2013	0	2139

TRINITY_DN3291_c0_g1_i1	Glyma.14G112700.3	100	234	22	12	1975	1868	3831	0	2076

TRINITY_DN3291_c0_g1_i1	Glyma.14G112700.6	97.28813559	234	22	12	1975	1868	3831	0	2076

TRINITY_DN3291_c0_g1_i1	Glyma.14G112700.1	97.28813559	234	22	12	1975	1868	3831	0	2076

TRINITY_DN3291_c0_g1_i1	Glyma.14G112700.5	97.2972973	234	22	12	1975	1865	3828	0	2076

TRINITY_DN3291_c0_g1_i1	Glyma.14G112700.4	97.2972973	234	22	12	1975	1862	3825	0	2076

TRINITY_DN3291_c0_g1_i1	Glyma.14G112700.2	100	234	22	12	1975	1460	3423	0	2076

TRINITY_DN1088_c0_g1_i1	Glyma.03G214900.3	99.07407407	220	13	629	2597	1987	7	0	2296

TRINITY_DN1088_c0_g2_i1	Glyma.03G214900.3	100	220	13	1042	3010	1987	7	0	2296

TRINITY_DN15820_c0_g2_i2	Glyma.14G003500.1	100	193	2	436	2421	1986	1	0	2588

TRINITY_DN15159_c0_g1_i1	Glyma.11G199700.1	97.36842105	193	7	280	2256	2016	34	0	2510

TRINITY_DN15159_c0_g1_i1	Glyma.11G199700.4	97.36842105	193	7	280	2256	2016	34	0	2510

TRINITY_DN16413_c0_g1_i5	Glyma.17G188300.2	96	187	6	277	2256	2822	840	0	2562

TRINITY_DN16413_c0_g1_i5	Glyma.17G188300.1	97.95918367	187	6	277	2256	2822	840	0	2562

TRINITY_DN15710_c0_g1_i1	Glyma.U033500.1	95.85492228	168	8	182	2163	2436	458	0	2654

TRINITY_DN16061_c0_g1_i2	Glyma.03G019700.1	95.3271028	161	6	546	2527	2001	20	0	2713

TRINITY_DN16194_c1_g1_i9	Glyma.06G109800.1	100	161	19	440	2362	288	2268	0	2326

TRINITY_DN16194_c1_g1_i9	Glyma.06G109800.3	98.0952381	161	19	440	2362	288	2268	0	2326

TRINITY_DN16194_c1_g1_i9	Glyma.06G109800.2	95.83333333	161	19	440	2362	198	2178	0	2326

TRINITY_DN12776_c0_g2_i2	Glyma.07G037100.1	100	148	23	289	2259	1	1947	0	2488

TRINITY_DN8977_c0_g1_i1	Glyma.06G094000.5	98.37398374	147	25	1	1953	489	2441	0	2416

TRINITY_DN15074_c0_g1_i1	Glyma.10G130800.3	100	132	11	459	2420	1986	1	0	2771

TRINITY_DN11782_c0_g1_i2	Glyma.16G077300.1	95.89041096	101	0	78	2064	2022	36	0	3110

TRINITY_DN14314_c0_g1_i2	Glyma.13G048600.1	97.91666667	432	37	206	2161	218	2185	0	965

TRINITY_DN39405_c0_g1_i1	Glyma.09G152400.1	96.19047619	306	3	371	2352	2815	831	0	1940

TRINITY_DN39405_c0_g1_i1	Glyma.09G152400.2	97.46835443	306	3	371	2352	2680	696	0	1940

TRINITY_DN15848_c1_g4_i1	Glyma.20G007900.1	100	222	8	247	2227	1	1981	0	2374

TRINITY_DN16061_c0_g1_i1	Glyma.09G266500.1	96.80851064	195	14	332	2307	1995	20	0	2459

TRINITY_DN15820_c0_g2_i2	Glyma.02G309400.1	99.12280702	187	0	436	2421	1986	1	0	2632

TRINITY_DN15353_c0_g1_i1	Glyma.09G259100.2	98.92473118	181	0	472	2457	2472	487	0	2665

TRINITY_DN8173_c0_g1_i3	Glyma.08G018700.1	95	176	5	306	2288	1983	1	0	2654

TRINITY_DN15329_c0_g2_i3	Glyma.02G085000.4	96.66666667	171	10	512	2495	2034	68	0	2584

TRINITY_DN16222_c0_g1_i4	Glyma.04G066400.2	100	169	21	179	2155	1	1959	0	2499

TRINITY_DN16222_c0_g1_i4	Glyma.04G066400.1	99.13793103	169	21	179	2155	1	1959	0	2499

TRINITY_DN16222_c0_g1_i7	Glyma.04G066400.2	100	169	21	245	2221	1	1959	0	2499

TRINITY_DN16222_c0_g1_i7	Glyma.04G066400.1	95.1048951	169	21	245	2221	1	1959	0	2499

TRINITY_DN16222_c0_g1_i9	Glyma.04G066400.2	99.02912621	169	21	333	2309	1	1959	0	2499

TRINITY_DN16222_c0_g1_i9	Glyma.04G066400.1	97.14285714	169	21	333	2309	1	1959	0	2499

TRINITY_DN4072_c0_g1_i1	Glyma.17G038300.1	98.95833333	165	2	423	2399	205	2190	0	2695

TRINITY_DN12776_c0_g2_i2	Glyma.16G006500.1	96.49122807	157	17	289	2259	1	1953	0	2488

TRINITY_DN12776_c0_g2_i2	Glyma.16G006500.2	96.49122807	157	17	289	2259	1	1953	0	2488

TRINITY_DN12776_c0_g2_i2	Glyma.16G006500.6	96.49122807	157	17	289	2259	1	1953	0	2488

TRINITY_DN12776_c0_g2_i2	Glyma.16G006500.9	99.56331878	156	20	289	2259	1	1941	0	2416

TRINITY_DN12776_c0_g2_i2	Glyma.16G006500.7	95.61403509	156	20	289	2259	1	1941	0	2416

TRINITY_DN12776_c0_g2_i2	Glyma.16G006500.3	96.2962963	156	20	289	2259	1	1941	0	2416

TRINITY_DN12776_c0_g2_i1	Glyma.16G006500.1	100	156	21	289	2247	1	1953	0	2416

TRINITY_DN12776_c0_g2_i1	Glyma.16G006500.2	95.45454545	156	21	289	2247	1	1953	0	2416

TRINITY_DN12776_c0_g2_i1	Glyma.16G006500.6	95.45454545	156	21	289	2247	1	1953	0	2416

TRINITY_DN12776_c0_g2_i2	Glyma.16G006500.4	99.56331878	155	21	289	2259	1	1944	0	2440

TRINITY_DN12776_c0_g2_i2	Glyma.16G006500.10	96.2962963	154	24	289	2259	1	1932	0	2368

TRINITY_DN12776_c0_g2_i2	Glyma.16G006500.8	100	154	24	289	2259	1	1932	0	2368

TRINITY_DN12776_c0_g2_i2	Glyma.16G006500.5	99.65870307	154	24	289	2259	1	1932	0	2368

TRINITY_DN12776_c0_g2_i1	Glyma.16G006500.4	95.45454545	154	25	289	2247	1	1944	0	2368

TRINITY_DN10185_c0_g1_i1	Glyma.17G117600.1	96.78362573	152	3	431	2413	1983	1	0	2787

TRINITY_DN10185_c0_g1_i1	Glyma.17G117600.5	98.95287958	152	3	431	2413	1983	1	0	2787

TRINITY_DN10185_c0_g1_i1	Glyma.17G117600.2	96.15384615	152	3	431	2413	1983	1	0	2787

TRINITY_DN10185_c0_g1_i1	Glyma.17G117600.3	97.68683274	152	3	431	2413	1983	1	0	2787

TRINITY_DN10185_c0_g1_i1	Glyma.17G117600.4	98.01587302	152	3	431	2413	1983	1	0	2787

TRINITY_DN11749_c0_g1_i1	Glyma.06G002400.5	100	150	3	1	1986	299	2264	0	2708

TRINITY_DN11749_c0_g1_i1	Glyma.06G002400.2	96.19565217	150	3	1	1986	299	2264	0	2708

TRINITY_DN11749_c0_g1_i2	Glyma.06G002400.5	100	150	3	1	1986	299	2264	0	2708

TRINITY_DN11749_c0_g1_i2	Glyma.06G002400.2	98.25581395	150	3	1	1986	299	2264	0	2708

TRINITY_DN216_c0_g1_i1	Glyma.08G072400.1	100	135	0	1	1986	794	2779	0	2920

TRINITY_DN216_c0_g1_i1	Glyma.08G072400.3	100	135	0	1	1986	251	2236	0	2920

TRINITY_DN216_c0_g1_i1	Glyma.08G072400.2	100	135	0	1	1986	251	2236	0	2920

TRINITY_DN15074_c0_g1_i1	Glyma.20G081500.2	98.86363636	129	5	459	2420	1986	1	0	2798

TRINITY_DN14759_c0_g1_i7	Glyma.09G203500.1	100	124	3	224	2200	1986	1	0	2922

TRINITY_DN19_c0_g1_i1	Glyma.06G173200.1	100	115	2	17	1999	1	1983	0	2992

TRINITY_DN15464_c0_g1_i2	Glyma.17G083300.1	100	102	2	187	2166	1	1986	0	3064

TRINITY_DN15464_c0_g1_i2	Glyma.17G083300.2	100	102	2	187	2166	1	1986	0	3064

TRINITY_DN13713_c0_g1_i1	Glyma.U033400.8	97.52066116	78	2	296	2281	1	1983	0	3216

TRINITY_DN13713_c0_g1_i1	Glyma.U033400.1	97.63033175	78	2	296	2281	1	1983	0	3216

TRINITY_DN13713_c0_g1_i6	Glyma.U033400.8	100	76	4	296	2264	1	1983	0	3118

TRINITY_DN13713_c0_g1_i6	Glyma.U033400.1	96.81528662	76	4	296	2264	1	1983	0	3118

TRINITY_DN13713_c0_g1_i1	Glyma.12G074700.8	99.0990991	68	3	296	2281	1	1977	0	3232

TRINITY_DN13713_c0_g1_i1	Glyma.12G074700.1	97.5	68	3	296	2281	1	1977	0	3232

TRINITY_DN13713_c0_g1_i6	Glyma.12G074700.8	96.55172414	66	5	296	2264	1	1977	0	3134

TRINITY_DN13713_c0_g1_i6	Glyma.12G074700.1	100	66	5	296	2264	1	1977	0	3134

TRINITY_DN3649_c0_g1_i1	Glyma.07G104700.2	96	316	31	450	2400	3384	1413	0	1611

TRINITY_DN15868_c0_g2_i4	Glyma.09G257700.6	98.63013699	247	24	100	2073	2	1956	0	2032

TRINITY_DN15868_c0_g2_i4	Glyma.09G257700.5	98.63013699	247	24	100	2073	2	1956	0	2032

TRINITY_DN15868_c0_g2_i4	Glyma.09G257700.1	98.63387978	247	24	100	2073	2	1956	0	2032

TRINITY_DN15868_c0_g2_i4	Glyma.09G257700.3	100	247	24	100	2073	2	1956	0	2032

TRINITY_DN15868_c0_g2_i4	Glyma.09G257700.4	95.4954955	247	24	100	2073	2	1956	0	2032

TRINITY_DN15868_c0_g2_i3	Glyma.09G257700.6	100	245	27	100	2067	2	1956	0	2004

TRINITY_DN15868_c0_g2_i3	Glyma.09G257700.5	100	245	27	100	2067	2	1956	0	2004

TRINITY_DN15868_c0_g2_i3	Glyma.09G257700.1	100	245	27	100	2067	2	1956	0	2004

TRINITY_DN15868_c0_g2_i3	Glyma.09G257700.3	100	245	27	100	2067	2	1956	0	2004

TRINITY_DN15868_c0_g2_i3	Glyma.09G257700.4	100	245	27	100	2067	2	1956	0	2004

TRINITY_DN16171_c1_g2_i3	Glyma.12G228800.1	96.60056657	179	26	631	2562	2097	118	0	2300

TRINITY_DN16171_c1_g2_i5	Glyma.12G228800.1	97.36842105	178	29	631	2548	2097	118	0	2215

TRINITY_DN16171_c1_g2_i1	Glyma.12G228800.1	95.04950495	171	26	669	2600	2097	118	0	2344

TRINITY_DN16171_c1_g2_i6	Glyma.12G228800.1	97.59036145	170	29	669	2586	2097	118	0	2259

TRINITY_DN13640_c1_g2_i7	Glyma.09G255400.4	96.93877551	149	12	441	2379	2979	1002	0	2497

TRINITY_DN15450_c1_g1_i1	Glyma.01G079100.4	95.08196721	147	14	393	2368	1977	5	0	2715

TRINITY_DN15450_c1_g1_i3	Glyma.01G079100.4	96.875	147	14	340	2315	1977	5	0	2715

TRINITY_DN9376_c0_g1_i1	Glyma.10G010800.4	97.05882353	138	5	531	2512	1983	1	0	2868

TRINITY_DN9376_c0_g1_i1	Glyma.10G010800.2	98.27586207	138	5	531	2512	1983	1	0	2868

TRINITY_DN9376_c0_g1_i1	Glyma.10G010800.1	96.63299663	138	5	531	2512	1983	1	0	2868

TRINITY_DN9376_c0_g1_i1	Glyma.10G010800.3	95.08196721	138	5	531	2512	1983	1	0	2868

TRINITY_DN9376_c0_g1_i2	Glyma.10G010800.4	97.07112971	138	5	531	2512	1983	1	0	2868

TRINITY_DN9376_c0_g1_i2	Glyma.10G010800.2	100	138	5	531	2512	1983	1	0	2868

TRINITY_DN9376_c0_g1_i2	Glyma.10G010800.1	97.87234043	138	5	531	2512	1983	1	0	2868

TRINITY_DN9376_c0_g1_i2	Glyma.10G010800.3	99.32432432	138	5	531	2512	1983	1	0	2868

TRINITY_DN15808_c0_g1_i1	Glyma.05G022300.2	95.87628866	318	20	129	2081	1	1971	0	1618

TRINITY_DN15808_c0_g1_i1	Glyma.05G022300.1	98.70550162	318	20	129	2081	1	1971	0	1618

TRINITY_DN15808_c0_g1_i4	Glyma.05G022300.2	99	318	20	129	2081	1	1971	0	1618

TRINITY_DN15808_c0_g1_i4	Glyma.05G022300.1	100	318	20	129	2081	1	1971	0	1618

TRINITY_DN15808_c0_g1_i6	Glyma.05G022300.2	96.15384615	318	20	129	2081	1	1971	0	1618

TRINITY_DN15808_c0_g1_i6	Glyma.05G022300.1	100	318	20	129	2081	1	1971	0	1618

TRINITY_DN9130_c0_g2_i2	Glyma.03G171500.1	99.18032787	301	45	213	2162	2520	595	0	1402

TRINITY_DN15681_c0_g2_i3	Glyma.09G035400.1	100	273	12	627	2601	206	2175	0	2002

TRINITY_DN14031_c0_g1_i1	Glyma.07G168900.1	95.44072948	243	12	62	2026	128	2107	0	2169

TRINITY_DN14938_c0_g1_i2	Glyma.02G147800.1	100	241	23	318	2299	1949	1	0	2089

TRINITY_DN13713_c0_g1_i3	Glyma.15G042700.1	95.8677686	220	10	31	1998	19	1992	0	2278

TRINITY_DN8173_c0_g1_i3	Glyma.05G212200.1	98.4496124	184	3	306	2288	1980	1	0	2612

TRINITY_DN16413_c0_g1_i5	Glyma.14G139800.1	96	174	5	277	2256	2819	840	0	2649

TRINITY_DN14481_c0_g3_i1	Glyma.07G202100.2	100	157	2	1	1983	110	2092	0	2782

TRINITY_DN9376_c0_g1_i1	Glyma.02G010300.5	97.70114943	153	3	531	2512	1983	1	0	2796

TRINITY_DN9376_c0_g1_i1	Glyma.02G010300.1	97.4325214	153	3	531	2512	1983	1	0	2796

TRINITY_DN9376_c0_g1_i1	Glyma.02G010300.4	97.43589744	153	3	531	2512	1983	1	0	2796

TRINITY_DN9376_c0_g1_i1	Glyma.02G010300.2	98.55072464	153	3	531	2512	1983	1	0	2796

TRINITY_DN9376_c0_g1_i1	Glyma.02G010300.3	99.14529915	153	3	531	2512	1983	1	0	2796

TRINITY_DN9376_c0_g1_i2	Glyma.02G010300.5	96.61016949	153	3	531	2512	1983	1	0	2796

TRINITY_DN9376_c0_g1_i2	Glyma.02G010300.1	100	153	3	531	2512	1983	1	0	2796

TRINITY_DN9376_c0_g1_i2	Glyma.02G010300.4	100	153	3	531	2512	1983	1	0	2796

TRINITY_DN9376_c0_g1_i2	Glyma.02G010300.2	100	153	3	531	2512	1983	1	0	2796

TRINITY_DN9376_c0_g1_i2	Glyma.02G010300.3	100	153	3	531	2512	1983	1	0	2796

TRINITY_DN13640_c1_g2_i7	Glyma.18G237600.1	97.13114754	150	12	441	2379	2976	1002	0	2484

TRINITY_DN15848_c1_g4_i8	Glyma.20G007900.1	98.68421053	135	1	247	2230	1	1981	0	2896

TRINITY_DN15329_c0_g2_i3	Glyma.05G238700.1	97.64705882	131	7	512	2495	2037	68	0	2848

TRINITY_DN16164_c1_g1_i5	Glyma.04G241900.2	98.42271293	124	3	65	2048	759	2736	0	2939

TRINITY_DN16164_c1_g1_i5	Glyma.04G241900.1	100	124	3	65	2048	759	2736	0	2939

TRINITY_DN19_c0_g1_i1	Glyma.04G192600.1	96.12903226	119	2	17	1999	1	1983	0	2992

TRINITY_DN16164_c1_g1_i8	Glyma.04G241900.2	100	119	3	65	2048	759	2736	0	2966

TRINITY_DN16164_c1_g1_i8	Glyma.04G241900.1	100	119	3	65	2048	759	2736	0	2966

TRINITY_DN10825_c0_g3_i1	Glyma.16G077100.1	98.71794872	296	36	463	2390	2190	218	0	1602

TRINITY_DN10825_c0_g3_i1	Glyma.16G077100.2	96.90721649	296	36	463	2390	2190	218	0	1602

TRINITY_DN10825_c0_g3_i2	Glyma.16G077100.1	100	296	36	291	2218	2190	218	0	1602

TRINITY_DN10825_c0_g3_i2	Glyma.16G077100.2	99.29577465	296	36	291	2218	2190	218	0	1602

TRINITY_DN15848_c1_g4_i1	Glyma.07G161900.1	100	230	8	247	2224	1	1978	0	2324

TRINITY_DN15848_c1_g4_i1	Glyma.07G161900.2	100	230	8	247	2224	1	1978	0	2324

TRINITY_DN9075_c0_g2_i1	Glyma.06G019200.1	100	208	50	109	1988	1970	1	0	1760

TRINITY_DN14439_c1_g2_i1	Glyma.09G096700.1	95.92944369	187	0	262	2244	1983	1	0	2627

TRINITY_DN14439_c1_g2_i1	Glyma.15G203000.1	97.5	177	0	262	2244	1983	1	0	2682

TRINITY_DN8710_c0_g1_i1	Glyma.11G230200.1	95.50561798	154	2	236	2217	2559	578	0	2796

TRINITY_DN8710_c0_g1_i2	Glyma.11G230200.1	100	154	3	236	2201	2559	578	0	2693

TRINITY_DN3358_c0_g1_i1	Glyma.05G180200.2	96.25	153	10	62	2026	2139	160	0	2678

TRINITY_DN3358_c0_g2_i1	Glyma.05G180200.2	97.03703704	153	10	62	2026	2139	160	0	2678

TRINITY_DN3358_c0_g3_i1	Glyma.05G180200.2	100	153	10	62	2026	2139	160	0	2678

TRINITY_DN3358_c0_g4_i1	Glyma.05G180200.2	97.05882353	153	10	62	2026	2139	160	0	2678

TRINITY_DN10185_c0_g1_i1	Glyma.05G009700.1	100	144	1	431	2413	1980	1	0	2844

TRINITY_DN15970_c0_g2_i11	Glyma.01G099800.2	95.12195122	131	0	230	2212	31	2013	0	2937

TRINITY_DN14759_c0_g1_i7	Glyma.01G019200.1	100	122	2	224	2200	1983	1	0	2948

TRINITY_DN19948_c0_g1_i1	Glyma.11G040000.10	96.68246445	105	0	428	2410	4198	2216	0	3081

TRINITY_DN19948_c0_g1_i1	Glyma.11G040000.6	96.66666667	105	0	428	2410	4198	2216	0	3081

TRINITY_DN15464_c0_g1_i2	Glyma.05G016700.1	95.51282051	105	1	187	2166	1	1983	0	3061

TRINITY_DN15464_c0_g1_i2	Glyma.05G016700.6	100	105	1	187	2166	1	1983	0	3061

TRINITY_DN3649_c0_g2_i1	Glyma.07G104700.2	98.66666667	318	30	450	2397	3384	1413	0	1615

TRINITY_DN14314_c0_g1_i2	Glyma.08G245600.1	95.45454545	282	24	206	2169	5	1959	0	1807

TRINITY_DN36798_c0_g1_i1	Glyma.10G004700.1	100	230	41	294	2233	2178	227	0	1921

TRINITY_DN12713_c0_g1_i1	Glyma.12G087100.1	100	203	3	19	1988	2132	151	0	2459

TRINITY_DN1607_c0_g2_i1	Glyma.10G202600.1	99.56331878	176	12	138	2111	1	1962	0	2505

TRINITY_DN15970_c0_g2_i1	Glyma.03G069400.1	99.55555556	153	3	80	2055	1	1982	0	2774

TRINITY_DN15970_c0_g2_i9	Glyma.03G069400.1	100	153	3	80	2055	1	1982	0	2774

TRINITY_DN13640_c1_g2_i7	Glyma.09G255400.3	98.64864865	148	12	441	2376	2976	1002	0	2497

TRINITY_DN35829_c0_g1_i1	Glyma.08G028400.3	96.55172414	141	4	810	2785	2016	44	0	2782

TRINITY_DN7387_c0_g1_i3	Glyma.20G174000.1	100	122	8	209	2182	1965	1	0	2822

TRINITY_DN6151_c0_g1_i1	Glyma.04G039700.1	97.43589744	358	34	507	2447	1	1962	0	1293

TRINITY_DN12718_c0_g2_i1	Glyma.18G216800.2	97.88235294	310	43	3	1955	916	2862	0	1541

TRINITY_DN12718_c0_g2_i1	Glyma.18G216800.1	97.88235294	310	43	3	1955	916	2862	0	1541

TRINITY_DN9376_c0_g1_i1	Glyma.19G218300.1	96.76470588	302	1	524	2503	1989	9	0	1978

TRINITY_DN9376_c0_g1_i1	Glyma.19G218300.2	98	302	1	524	2503	1989	9	0	1978

TRINITY_DN9376_c0_g1_i2	Glyma.19G218300.1	100	302	1	524	2503	1989	9	0	1978

TRINITY_DN9376_c0_g1_i2	Glyma.19G218300.2	100	302	1	524	2503	1989	9	0	1978

TRINITY_DN15853_c0_g1_i12	Glyma.02G010500.2	98.85057471	246	4	894	2874	1975	1	0	2257

TRINITY_DN15853_c0_g1_i3	Glyma.02G010500.2	95.21126761	246	4	557	2537	1975	1	0	2257

TRINITY_DN15868_c0_g2_i1	Glyma.09G257700.6	95.74468085	245	23	405	2370	4	1956	0	2023

TRINITY_DN15868_c0_g2_i1	Glyma.09G257700.5	96.2962963	245	23	405	2370	4	1956	0	2023

TRINITY_DN15868_c0_g2_i1	Glyma.09G257700.1	100	245	23	405	2370	4	1956	0	2023

TRINITY_DN15868_c0_g2_i1	Glyma.09G257700.3	99.07407407	245	23	405	2370	4	1956	0	2023

TRINITY_DN15868_c0_g2_i1	Glyma.09G257700.4	99.13043478	245	23	405	2370	4	1956	0	2023

TRINITY_DN15868_c0_g2_i5	Glyma.09G257700.6	97.2972973	245	23	405	2370	4	1956	0	2023

TRINITY_DN15868_c0_g2_i5	Glyma.09G257700.5	96.77419355	245	23	405	2370	4	1956	0	2023

TRINITY_DN15868_c0_g2_i5	Glyma.09G257700.1	100	245	23	405	2370	4	1956	0	2023

TRINITY_DN15868_c0_g2_i5	Glyma.09G257700.3	100	245	23	405	2370	4	1956	0	2023

TRINITY_DN15868_c0_g2_i5	Glyma.09G257700.4	98.97785349	245	23	405	2370	4	1956	0	2023

TRINITY_DN13713_c0_g1_i3	Glyma.15G042700.8	95.8677686	220	9	31	1998	19	1989	0	2290

TRINITY_DN11596_c0_g2_i1	Glyma.07G127800.1	98.93617021	199	10	1505	3476	2003	32	0	2440

TRINITY_DN11596_c0_g2_i1	Glyma.07G127800.3	98.76923077	199	10	1505	3476	2003	32	0	2440

TRINITY_DN11596_c0_g2_i1	Glyma.07G127800.2	98.7704918	199	10	1505	3476	2003	32	0	2440

TRINITY_DN12874_c0_g1_i1	Glyma.17G121100.3	98.37398374	195	3	553	2532	1	1977	0	2545

TRINITY_DN12874_c0_g1_i1	Glyma.17G121100.2	98.36065574	195	3	553	2532	1	1977	0	2545

TRINITY_DN12874_c0_g1_i1	Glyma.17G121100.5	98.38709677	195	3	553	2532	1	1977	0	2545

TRINITY_DN12874_c0_g1_i1	Glyma.17G121100.1	98.3805668	195	3	553	2532	1	1977	0	2545

TRINITY_DN12874_c0_g1_i4	Glyma.17G121100.3	95.83333333	195	3	553	2532	1	1977	0	2545

TRINITY_DN12874_c0_g1_i4	Glyma.17G121100.2	95.83333333	195	3	553	2532	1	1977	0	2545

TRINITY_DN12874_c0_g1_i4	Glyma.17G121100.5	95.83333333	195	3	553	2532	1	1977	0	2545

TRINITY_DN12874_c0_g1_i4	Glyma.17G121100.1	95.84199584	195	3	553	2532	1	1977	0	2545

TRINITY_DN12874_c0_g1_i1	Glyma.17G121100.4	98.38709677	194	5	553	2532	1	1971	0	2512

TRINITY_DN12874_c0_g1_i4	Glyma.17G121100.4	96.99570815	194	5	553	2532	1	1971	0	2512

TRINITY_DN14975_c0_g2_i1	Glyma.08G003500.1	100	163	32	283	2230	214	2165	0	2355

TRINITY_DN10803_c0_g1_i2	Glyma.02G162300.1	99.24242424	150	3	164	2140	1980	1	0	2795

TRINITY_DN13640_c1_g2_i7	Glyma.18G237600.2	98.64498645	150	11	441	2379	2973	1002	0	2497

TRINITY_DN15848_c1_g4_i8	Glyma.07G161900.1	98.68421053	144	1	247	2227	1	1978	0	2841

TRINITY_DN15848_c1_g4_i8	Glyma.07G161900.2	98.18181818	144	1	247	2227	1	1978	0	2841

TRINITY_DN12226_c0_g2_i3	Glyma.19G033700.1	98.93617021	141	7	624	2597	1977	1	0	2806

TRINITY_DN16164_c1_g1_i5	Glyma.06G121500.1	96.2962963	125	3	68	2048	912	2886	0	2928

TRINITY_DN16164_c1_g1_i5	Glyma.06G121500.4	98.1865285	125	3	68	2048	888	2862	0	2928

TRINITY_DN16164_c1_g1_i5	Glyma.06G121500.2	96	125	3	68	2048	759	2733	0	2928

TRINITY_DN16164_c1_g1_i8	Glyma.06G121500.1	96.53179191	120	3	68	2048	912	2886	0	2955

TRINITY_DN16164_c1_g1_i8	Glyma.06G121500.4	97.09543568	120	3	68	2048	888	2862	0	2955

TRINITY_DN16164_c1_g1_i8	Glyma.06G121500.2	97.51381215	120	3	68	2048	759	2733	0	2955

TRINITY_DN15980_c0_g2_i2	Glyma.10G004100.1	97.46192893	361	44	2077	4021	1794	3720	0	1088

TRINITY_DN15980_c0_g2_i2	Glyma.10G004100.2	97.46192893	361	44	2077	4021	1374	3300	0	1088

TRINITY_DN14314_c0_g1_i2	Glyma.18G266900.1	98.37925446	271	20	199	2169	1	1962	0	1980

TRINITY_DN15868_c0_g2_i4	Glyma.18G234900.1	95.4954955	264	15	100	2073	2	1956	0	1993

TRINITY_DN15868_c0_g2_i4	Glyma.18G234900.3	95.4954955	264	15	100	2073	2	1956	0	1993

TRINITY_DN15868_c0_g2_i4	Glyma.18G234900.2	95.4954955	264	15	100	2073	2	1956	0	1993

TRINITY_DN15868_c0_g2_i4	Glyma.18G234900.4	95.4954955	262	18	100	2073	2	1950	0	1965

TRINITY_DN15868_c0_g2_i3	Glyma.18G234900.1	100	259	16	100	2067	2	1956	0	1982

TRINITY_DN15868_c0_g2_i3	Glyma.18G234900.3	100	259	16	100	2067	2	1956	0	1982

TRINITY_DN15868_c0_g2_i3	Glyma.18G234900.2	100	259	16	100	2067	2	1956	0	1982

TRINITY_DN15868_c0_g2_i3	Glyma.18G234900.4	100	257	19	100	2067	2	1950	0	1954

TRINITY_DN16086_c0_g2_i3	Glyma.14G134100.1	95.51724138	210	6	100	2076	1	1977	0	2455

TRINITY_DN16086_c0_g2_i3	Glyma.17G199200.1	97.33333333	208	6	100	2076	1	1977	0	2466

TRINITY_DN18011_c0_g1_i1	Glyma.04G250200.1	98.41427156	188	26	1	1953	1681	3627	0	2228

TRINITY_DN14790_c0_g1_i1	Glyma.08G329100.2	97.65625	139	33	329	2269	2151	217	0	2344

TRINITY_DN14790_c0_g1_i2	Glyma.08G329100.2	95.93495935	139	33	329	2269	2151	217	0	2344

TRINITY_DN15655_c0_g1_i3	Glyma.16G000300.1	97.05882353	125	3	1050	3023	1996	17	0	2926

TRINITY_DN15655_c0_g1_i3	Glyma.12G164100.1	97.05882353	115	3	1050	3023	1996	17	0	2981

TRINITY_DN5133_c0_g1_i1	Glyma.19G235200.1	100	115	5	101	2071	1	1977	0	2942

TRINITY_DN15848_c1_g4_i8	Glyma.11G122500.1	100	279	12	247	2218	1	1966	0	1980

TRINITY_DN15970_c0_g2_i11	Glyma.07G137300.2	100	267	4	230	2206	166	2142	0	2150

TRINITY_DN11099_c0_g2_i1	Glyma.20G068300.1	96.90265487	254	13	86	2048	36	2010	0	2119

TRINITY_DN16245_c0_g3_i2	Glyma.08G268900.1	97.14285714	237	4	301	2278	3569	1597	0	2296

TRINITY_DN21719_c0_g1_i1	Glyma.04G255100.6	95.12195122	208	7	1	1952	2042	64	0	2327

TRINITY_DN21719_c0_g1_i1	Glyma.04G255100.4	97.03264095	208	7	1	1952	2042	64	0	2327

TRINITY_DN21719_c0_g1_i1	Glyma.04G255100.1	95.37366548	208	7	1	1952	2042	64	0	2327

TRINITY_DN12713_c0_g1_i1	Glyma.12G087100.2	96.11650485	204	2	19	1988	2129	151	0	2466

TRINITY_DN16061_c0_g1_i1	Glyma.07G081600.1	95.45454545	189	7	342	2311	1988	13	0	2531

TRINITY_DN16061_c0_g1_i1	Glyma.07G081600.2	95.6043956	189	7	342	2311	1988	13	0	2531

TRINITY_DN14975_c0_g2_i1	Glyma.05G195900.1	100	165	31	284	2231	228	2178	0	2359

TRINITY_DN15970_c0_g2_i1	Glyma.01G099800.1	96.12403101	149	2	80	2055	1	1979	0	2809

TRINITY_DN15970_c0_g2_i9	Glyma.01G099800.1	98.27586207	149	2	80	2055	1	1979	0	2809

TRINITY_DN9072_c0_g2_i2	Glyma.06G232100.1	95.92760181	140	10	161	2136	1	1949	0	2665

TRINITY_DN9376_c0_g1_i1	Glyma.03G221400.1	100	294	9	524	2495	1989	18	0	1947

TRINITY_DN9376_c0_g1_i1	Glyma.03G221400.2	100	294	9	524	2495	1989	18	0	1947

TRINITY_DN9376_c0_g1_i2	Glyma.03G221400.1	100	294	9	524	2495	1989	18	0	1947

TRINITY_DN9376_c0_g1_i2	Glyma.03G221400.2	98.08917197	294	9	524	2495	1989	18	0	1947

TRINITY_DN15868_c0_g2_i1	Glyma.18G234900.1	99.20318725	254	16	405	2370	4	1956	0	2006

TRINITY_DN15868_c0_g2_i1	Glyma.18G234900.3	96.24573379	254	16	405	2370	4	1956	0	2006

TRINITY_DN15868_c0_g2_i1	Glyma.18G234900.2	96.17755857	254	16	405	2370	4	1956	0	2006

TRINITY_DN15868_c0_g2_i5	Glyma.18G234900.1	97.13574097	254	16	405	2370	4	1956	0	2006

TRINITY_DN15868_c0_g2_i5	Glyma.18G234900.3	96.72364672	254	16	405	2370	4	1956	0	2006

TRINITY_DN15868_c0_g2_i5	Glyma.18G234900.2	97.70114943	254	16	405	2370	4	1956	0	2006

TRINITY_DN15868_c0_g2_i1	Glyma.18G234900.4	96.17834395	252	19	405	2370	4	1950	0	1978

TRINITY_DN15868_c0_g2_i5	Glyma.18G234900.4	95.41984733	252	19	405	2370	4	1950	0	1978

TRINITY_DN16061_c0_g1_i1	Glyma.03G019700.1	96.80851064	190	13	342	2307	1991	20	0	2484

TRINITY_DN10803_c0_g1_i2	Glyma.02G162300.9	95.45454545	150	2	164	2140	1977	1	0	2809

TRINITY_DN10803_c0_g1_i2	Glyma.02G162300.8	96.15384615	148	3	164	2140	1971	1	0	2782

TRINITY_DN16086_c0_g2_i3	Glyma.15G162600.1	98.31932773	341	17	109	2076	22	1989	0	1646

TRINITY_DN15848_c1_g4_i8	Glyma.12G047400.1	100	281	10	247	2218	1	1966	0	1991

TRINITY_DN2703_c0_g1_i1	Glyma.12G080900.1	100	233	10	446	2418	2328	359	0	2289

TRINITY_DN5257_c0_g3_i1	Glyma.14G074700.1	100	201	9	261	2227	1973	1	0	2447

TRINITY_DN16086_c0_g2_i3	Glyma.06G076100.1	97.33333333	112	0	100	2076	1	1977	0	3031

TRINITY_DN16086_c0_g2_i3	Glyma.09G056300.1	95.51569507	342	14	109	2076	208	2175	0	1652

TRINITY_DN16086_c0_g2_i3	Glyma.09G056300.2	95.51495017	342	14	109	2076	22	1989	0	1652

TRINITY_DN3291_c0_g1_i1	Glyma.17G215200.1	98.30508475	219	19	12	1971	1952	3890	0	2093

TRINITY_DN3291_c0_g1_i1	Glyma.17G215200.2	98.28571429	219	19	12	1971	956	2894	0	2093

TRINITY_DN7424_c0_g1_i1	Glyma.08G054900.1	98.75	195	6	34	2004	295	2268	0	2523

TRINITY_DN7424_c0_g1_i1	Glyma.08G054900.2	98.75	191	8	34	2004	295	2262	0	2507

TRINITY_DN16086_c0_g2_i3	Glyma.04G075100.2	98.41269841	99	0	100	2075	1	1976	0	3101

TRINITY_DN16086_c0_g2_i3	Glyma.04G075100.1	95.11111111	99	0	100	2075	1	1976	0	3101

TRINITY_DN16086_c0_g2_i3	Glyma.15G207900.1	96	436	25	116	2076	23	1983	0	1051

TRINITY_DN15681_c0_g2_i2	Glyma.09G035400.1	98.98989899	307	10	627	2586	206	2175	0	1818

TRINITY_DN16396_c2_g1_i5	Glyma.11G138400.3	97.23756906	273	37	319	2252	80	2022	0	1663

TRINITY_DN16396_c2_g1_i5	Glyma.11G138400.1	97.24770642	273	37	319	2252	80	2022	0	1663

TRINITY_DN16396_c2_g1_i3	Glyma.11G138400.3	97.25609756	267	36	319	2252	80	2022	0	1696

TRINITY_DN16396_c2_g1_i3	Glyma.11G138400.1	97.25557461	267	36	319	2252	80	2022	0	1696

TRINITY_DN16257_c0_g1_i1	Glyma.06G307900.1	95.73863636	243	6	116	2077	2232	259	0	2211

TRINITY_DN14644_c0_g2_i1	Glyma.10G271900.1	100	235	10	310	2269	2006	41	0	2191

TRINITY_DN14314_c0_g1_i2	Glyma.08G246300.1	98.7012987	235	11	199	2169	1	1962	0	2235

TRINITY_DN18489_c0_g1_i1	Glyma.01G215700.3	97.74647887	192	1	450	2421	4370	2396	0	2564

TRINITY_DN18489_c0_g1_i1	Glyma.01G215700.1	98.88888889	192	1	450	2421	4370	2396	0	2564

TRINITY_DN18489_c0_g1_i1	Glyma.01G215700.2	99.5	190	5	450	2421	4361	2396	0	2518

TRINITY_DN38438_c0_g1_i1	Glyma.08G332200.1	100	189	14	143	2098	1	1968	0	2433

TRINITY_DN18489_c0_g1_i1	Glyma.11G026700.2	96.51162791	179	5	450	2421	4361	2396	0	2579

TRINITY_DN18489_c0_g1_i1	Glyma.11G026700.1	96.98492462	179	5	450	2421	4361	2396	0	2579

TRINITY_DN18489_c0_g1_i1	Glyma.11G026700.3	99.609375	179	5	450	2421	4130	2165	0	2579

TRINITY_DN8503_c0_g1_i1	Glyma.16G213100.2	100	172	3	781	2753	1974	1	0	2675

TRINITY_DN8503_c0_g1_i3	Glyma.16G213100.2	95.11494253	172	3	792	2764	1974	1	0	2675

TRINITY_DN12776_c0_g2_i1	Glyma.07G037100.1	95.07575758	149	20	289	2247	1	1947	0	2538

TRINITY_DN10640_c0_g1_i1	Glyma.10G009200.1	95.1048951	130	5	1339	3306	1971	1	0	2856

TRINITY_DN10640_c0_g1_i3	Glyma.10G009200.1	96.05781866	130	5	1339	3306	1971	1	0	2856

TRINITY_DN2628_c0_g1_i1	Glyma.04G098900.1	100	128	5	179	2149	1	1968	0	2867

TRINITY_DN13831_c0_g1_i1	Glyma.07G013000.1	95.45454545	126	0	447	2421	1	1975	0	2950

TRINITY_DN10975_c0_g1_i2	Glyma.08G225500.4	98.95013123	101	0	363	2337	1	1975	0	3088

TRINITY_DN16245_c0_g2_i1	Glyma.16G099700.3	100	511	59	1617	3557	2289	352	5.01E-100	368

TRINITY_DN16245_c0_g2_i1	Glyma.16G099700.2	100	511	59	1617	3557	2289	352	5.01E-100	368

TRINITY_DN16245_c0_g2_i1	Glyma.16G099700.1	98.63013699	511	59	1617	3557	2289	352	5.01E-100	368

TRINITY_DN16086_c0_g2_i3	Glyma.13G097900.1	97.95918367	360	18	109	2076	22	1977	0	1496

TRINITY_DN14912_c0_g1_i2	Glyma.02G021900.1	96.54576857	356	40	657	2610	504	2441	0	1312

TRINITY_DN14912_c0_g1_i2	Glyma.02G021900.2	96.53465347	356	40	657	2610	327	2264	0	1312

TRINITY_DN16057_c0_g1_i3	Glyma.07G078100.1	98.66666667	292	50	3056	4998	1306	3230	0	1511

TRINITY_DN11099_c0_g2_i1	Glyma.13G001500.4	96.26168224	281	8	86	2048	36	2007	0	2004

TRINITY_DN11099_c0_g2_i1	Glyma.13G001500.1	98	281	8	86	2048	36	2007	0	2004

TRINITY_DN11099_c0_g2_i1	Glyma.13G001500.2	95.7537155	280	10	86	2048	36	2004	0	1991

TRINITY_DN11099_c0_g2_i1	Glyma.13G001500.3	100	280	10	86	2048	36	2004	0	1991

TRINITY_DN15848_c1_g4_i1	Glyma.11G122500.1	97.07112971	191	8	247	2215	1	1966	0	2503

TRINITY_DN15848_c1_g4_i1	Glyma.12G047400.1	97.07112971	185	9	247	2215	1	1966	0	2536

TRINITY_DN13572_c0_g2_i1	Glyma.05G002800.1	97.48283753	172	18	916	2868	1962	1	0	2479

TRINITY_DN13572_c0_g2_i3	Glyma.05G002800.1	97.48427673	172	18	895	2847	1962	1	0	2479

TRINITY_DN12776_c0_g2_i1	Glyma.16G006500.9	99.08256881	157	17	289	2247	1	1941	0	2466

TRINITY_DN12776_c0_g2_i1	Glyma.16G006500.7	96.875	157	17	289	2247	1	1941	0	2466

TRINITY_DN12776_c0_g2_i1	Glyma.16G006500.3	96.875	157	17	289	2247	1	1941	0	2466

TRINITY_DN12776_c0_g2_i1	Glyma.16G006500.10	100	155	21	289	2247	1	1932	0	2418

TRINITY_DN12776_c0_g2_i1	Glyma.16G006500.8	100	155	21	289	2247	1	1932	0	2418

TRINITY_DN12776_c0_g2_i1	Glyma.16G006500.5	95.8677686	155	21	289	2247	1	1932	0	2418

TRINITY_DN15820_c0_g2_i1	Glyma.13G026100.1	99.11504425	141	0	436	2409	1974	1	0	2865

TRINITY_DN15820_c0_g2_i4	Glyma.13G026100.1	99.00990099	141	0	436	2409	1974	1	0	2865

TRINITY_DN15820_c0_g2_i1	Glyma.20G059000.1	95.66210046	131	0	436	2409	1974	1	0	2920

TRINITY_DN15820_c0_g2_i1	Glyma.20G059000.2	95.34883721	131	0	436	2409	1974	1	0	2920

TRINITY_DN15820_c0_g2_i4	Glyma.20G059000.1	100	131	0	436	2409	1974	1	0	2920

TRINITY_DN15820_c0_g2_i4	Glyma.20G059000.2	96.6442953	131	0	436	2409	1974	1	0	2920

TRINITY_DN13831_c0_g1_i1	Glyma.08G196700.1	99.05660377	122	2	447	2419	1	1973	0	2957

TRINITY_DN16059_c0_g3_i1	Glyma.13G182500.1	100	105	2	204	2177	1	1971	0	3044

TRINITY_DN16059_c0_g3_i2	Glyma.13G182500.1	95.38461538	105	2	483	2456	1	1971	0	3044

TRINITY_DN16059_c0_g3_i1	Glyma.07G194200.1	96.72131148	103	2	204	2177	1	1971	0	3055

TRINITY_DN16059_c0_g3_i2	Glyma.07G194200.1	95.38461538	103	2	483	2456	1	1971	0	3055

TRINITY_DN16086_c0_g2_i3	Glyma.17G061800.1	100	354	10	109	2076	22	1989	0	1618

TRINITY_DN13903_c1_g1_i1	Glyma.10G184300.1	97.44897959	299	42	70	2009	1264	3194	0	1502

TRINITY_DN12718_c0_g1_i1	Glyma.18G216800.2	100	272	36	3	1946	916	2862	0	1781

TRINITY_DN12718_c0_g1_i1	Glyma.18G216800.1	95.56451613	272	36	3	1946	916	2862	0	1781

TRINITY_DN11312_c0_g1_i2	Glyma.20G235500.1	100	241	14	206	2166	1	1953	0	2102

TRINITY_DN18011_c0_g1_i1	Glyma.06G112600.1	98.9010989	217	21	1	1953	1669	3618	0	2163

TRINITY_DN19642_c0_g1_i1	Glyma.12G188600.1	97.34513274	204	8	179	2148	1	1955	0	2377

TRINITY_DN13572_c0_g2_i1	Glyma.19G003000.1	97.47474747	179	16	916	2868	1962	1	0	2451

TRINITY_DN13572_c0_g2_i3	Glyma.19G003000.1	97.4789916	179	16	895	2847	1962	1	0	2451

TRINITY_DN16414_c0_g1_i11	Glyma.05G216600.1	98.22616408	287	11	357	2322	1966	1	0	1975

TRINITY_DN10090_c1_g1_i1	Glyma.07G135600.1	98.79518072	207	2	383	2354	2009	41	0	2475

TRINITY_DN15721_c0_g1_i4	Glyma.19G084600.1	97.7443609	181	12	616	2574	1	1959	0	2471

TRINITY_DN15721_c0_g1_i6	Glyma.19G084600.1	100	181	12	714	2672	1	1959	0	2471

TRINITY_DN11346_c0_g1_i1	Glyma.10G192700.2	99.78540773	178	13	6	1974	1555	3493	0	2423

TRINITY_DN15329_c0_g2_i6	Glyma.02G085000.4	97.62470309	172	6	512	2481	2034	68	0	2643

TRINITY_DN9702_c0_g2_i1	Glyma.04G069600.3	95	159	2	191	2156	23	1994	0	2723

TRINITY_DN2307_c0_g1_i1	Glyma.11G001900.1	95.45454545	139	4	82	2050	75	2037	0	2795

TRINITY_DN15808_c0_g1_i2	Glyma.04G163600.2	100	312	25	206	2161	1	1935	0	1581

TRINITY_DN15808_c0_g1_i2	Glyma.04G163600.1	100	312	25	206	2161	1	1935	0	1581

TRINITY_DN15808_c0_g1_i2	Glyma.04G163600.3	100	312	25	206	2161	1	1935	0	1581

TRINITY_DN15808_c0_g1_i3	Glyma.04G163600.2	98.7012987	312	25	206	2161	1	1935	0	1581

TRINITY_DN15808_c0_g1_i3	Glyma.04G163600.1	99.5	312	25	206	2161	1	1935	0	1581

TRINITY_DN15808_c0_g1_i3	Glyma.04G163600.3	98.57819905	312	25	206	2161	1	1935	0	1581

TRINITY_DN15808_c0_g1_i5	Glyma.04G163600.2	97.65013055	312	25	206	2161	1	1935	0	1581

TRINITY_DN15808_c0_g1_i5	Glyma.04G163600.1	97.4025974	312	25	206	2161	1	1935	0	1581

TRINITY_DN15808_c0_g1_i5	Glyma.04G163600.3	99.09090909	312	25	206	2161	1	1935	0	1581

TRINITY_DN10825_c0_g3_i1	Glyma.16G110400.1	95.72649573	305	31	463	2390	2154	191	0	1628

TRINITY_DN10825_c0_g3_i2	Glyma.16G110400.1	99.36305732	305	31	291	2218	2154	191	0	1628

TRINITY_DN5257_c0_g3_i1	Glyma.17G250800.1	98.52941176	191	12	261	2227	1952	1	0	2433

TRINITY_DN5257_c0_g3_i1	Glyma.17G250800.2	98.52941176	191	12	261	2227	1952	1	0	2433

TRINITY_DN16063_c0_g1_i1	Glyma.04G139400.1	96.42857143	176	32	681	2618	1902	1	0	2006

TRINITY_DN16063_c0_g1_i2	Glyma.04G139400.1	95.65217391	176	32	925	2862	1902	1	0	2006

TRINITY_DN14127_c0_g1_i1	Glyma.20G025400.1	100	169	17	8	1960	1	1959	0	2510

TRINITY_DN15159_c0_g1_i1	Glyma.14G206000.2	95.09043928	322	38	281	2222	1967	19	0	1537

TRINITY_DN15159_c0_g1_i1	Glyma.14G206000.1	95.09202454	322	38	281	2222	1967	19	0	1537

TRINITY_DN15778_c0_g1_i4	Glyma.18G196200.3	99.15966387	261	16	1026	2986	1952	1	0	2017

TRINITY_DN14314_c0_g1_i2	Glyma.18G267100.1	100	250	6	203	2169	11	1968	0	2156

TRINITY_DN6018_c0_g2_i2	Glyma.20G171200.1	100	183	7	207	2171	1	1959	0	2521

TRINITY_DN15549_c0_g1_i4	Glyma.05G247000.2	95.83333333	151	12	903	2842	2784	821	0	2569

TRINITY_DN15329_c0_g2_i6	Glyma.05G238700.1	98.91304348	132	0	512	2481	2037	68	0	2907

TRINITY_DN16384_c0_g1_i1	Glyma.09G277700.1	100	308	17	883	2839	1	1959	0	1788

TRINITY_DN16384_c0_g1_i3	Glyma.09G277700.1	100	307	17	881	2837	1	1959	0	1794

TRINITY_DN12607_c0_g1_i1	Glyma.17G065700.2	97.05882353	183	10	1546	3498	1301	3262	0	2473

TRINITY_DN20930_c0_g1_i1	Glyma.07G261900.1	100	175	11	66	2018	1	1968	0	2556

TRINITY_DN16235_c0_g1_i5	Glyma.04G101000.1	100	170	5	795	2757	2259	297	0	2617

TRINITY_DN15519_c1_g1_i2	Glyma.07G218900.1	96.54255319	155	2	341	2306	1980	12	0	2758

TRINITY_DN11749_c0_g1_i3	Glyma.06G002400.5	95.23809524	152	1	1	1969	299	2264	0	2774

TRINITY_DN11749_c0_g1_i3	Glyma.06G002400.2	95.23809524	152	1	1	1969	299	2264	0	2774

TRINITY_DN11749_c0_g1_i4	Glyma.06G002400.5	95.28301887	152	1	1	1969	299	2264	0	2774

TRINITY_DN11749_c0_g1_i4	Glyma.06G002400.2	100	152	1	1	1969	299	2264	0	2774

TRINITY_DN14764_c2_g1_i2	Glyma.17G240400.1	97.36842105	145	8	345	2303	1965	1	0	2743

TRINITY_DN11312_c0_g1_i2	Glyma.03G236500.1	95.19230769	140	9	200	2166	1	1950	0	2724

TRINITY_DN11312_c0_g1_i2	Glyma.03G236500.4	100	139	10	200	2166	1	1947	0	2712

TRINITY_DN17367_c0_g2_i1	Glyma.03G215200.1	97.5	138	6	1	1966	432	2397	0	2833

TRINITY_DN15624_c0_g1_i3	Glyma.14G169300.1	98.73015873	129	4	181	2148	1	1965	0	2889

TRINITY_DN15624_c0_g1_i5	Glyma.14G169300.1	95.96412556	129	4	181	2148	1	1965	0	2889

TRINITY_DN15624_c0_g1_i2	Glyma.14G169300.1	99.38461538	127	8	181	2135	1	1965	0	2817

TRINITY_DN12320_c0_g1_i3	Glyma.19G012700.1	100	112	1	273	2238	13	1981	0	2996

TRINITY_DN12320_c0_g1_i3	Glyma.13G070300.1	95.94594595	110	1	273	2238	13	1981	0	3007

TRINITY_DN6691_c0_g1_i1	Glyma.01G173800.1	95.34883721	406	44	685	2626	66	2004	0	1029

TRINITY_DN11214_c0_g1_i1	Glyma.13G048600.1	98.9010989	373	18	3	1954	2237	280	0	1400

TRINITY_DN13622_c0_g1_i1	Glyma.10G234000.1	100	324	23	421	2377	2818	877	0	1600

TRINITY_DN13622_c0_g1_i1	Glyma.10G234000.2	100	324	23	421	2377	2719	778	0	1600

TRINITY_DN13622_c0_g1_i1	Glyma.10G234000.3	100	324	23	421	2377	2494	553	0	1600

TRINITY_DN16414_c0_g1_i4	Glyma.05G216600.1	96.25	288	9	168	2130	1963	1	0	1975

TRINITY_DN16414_c0_g1_i10	Glyma.05G216600.1	96.2962963	277	12	357	2316	1966	5	0	2010

TRINITY_DN42531_c0_g1_i1	Glyma.15G019900.1	97.22222222	180	12	259	2222	1	1946	0	2470

TRINITY_DN14127_c0_g1_i1	Glyma.20G025400.2	98.10606061	169	16	8	1960	1	1956	0	2523

TRINITY_DN9519_c0_g1_i1	Glyma.08G330800.2	97.16981132	141	7	312	2267	1962	1	0	2737

TRINITY_DN9519_c0_g1_i1	Glyma.08G330800.1	96.57142857	141	7	312	2267	1962	1	0	2737

TRINITY_DN9519_c0_g1_i1	Glyma.18G075600.1	97.16981132	137	4	312	2267	1962	1	0	2760

TRINITY_DN14682_c0_g1_i3	Glyma.12G026600.1	100	136	0	1	1968	3491	1524	0	2881

TRINITY_DN17367_c0_g2_i1	Glyma.14G001400.1	97.5	134	4	1	1966	489	2454	0	2867

TRINITY_DN15624_c0_g1_i3	Glyma.13G087100.1	98.63013699	129	2	181	2148	1	1965	0	2900

TRINITY_DN15624_c0_g1_i5	Glyma.13G087100.1	98.5915493	129	2	181	2148	1	1965	0	2900

TRINITY_DN15624_c0_g1_i2	Glyma.13G087100.1	98.98989899	128	5	181	2135	1	1965	0	2822

TRINITY_DN2367_c0_g1_i1	Glyma.02G307900.2	99.29078014	121	3	505	2463	1968	1	0	2905

TRINITY_DN2367_c0_g1_i1	Glyma.02G307900.1	98.1595092	121	3	505	2463	1968	1	0	2905

TRINITY_DN13713_c0_g1_i3	Glyma.U033400.8	98.21428571	78	2	31	1998	19	1983	0	3182

TRINITY_DN13713_c0_g1_i3	Glyma.U033400.1	98.21428571	78	2	31	1998	19	1983	0	3182

TRINITY_DN13713_c0_g1_i3	Glyma.12G074700.8	99.83870968	68	3	31	1998	19	1977	0	3199

TRINITY_DN13713_c0_g1_i3	Glyma.12G074700.1	95.215311	68	3	31	1998	19	1977	0	3199

TRINITY_DN16254_c0_g1_i8	Glyma.07G004300.1	98.18181818	469	38	697	2643	2994	1054	0	737

TRINITY_DN16254_c0_g1_i8	Glyma.07G004300.2	95.74468085	469	38	697	2643	2892	952	0	737

TRINITY_DN16254_c0_g1_i8	Glyma.07G004300.3	97.82608696	469	38	697	2643	2577	637	0	737

TRINITY_DN16086_c0_g2_i3	Glyma.13G369300.1	95.6185567	427	28	124	2076	19	1971	0	1086

TRINITY_DN12758_c0_g1_i1	Glyma.08G209200.1	96.35036496	310	23	388	2327	1958	1	0	1683

TRINITY_DN16384_c0_g1_i4	Glyma.09G277700.1	100	281	16	883	2833	1	1959	0	1921

TRINITY_DN16384_c0_g1_i2	Glyma.09G277700.1	100	280	16	881	2831	1	1959	0	1927

TRINITY_DN16807_c0_g1_i1	Glyma.08G235800.1	96.07843137	196	2	232	2198	3106	1149	0	2488

TRINITY_DN16235_c0_g1_i1	Glyma.04G101000.1	99.28057554	170	5	795	2755	2259	299	0	2614

TRINITY_DN16235_c0_g1_i3	Glyma.04G101000.1	100	170	5	795	2755	2259	299	0	2614

TRINITY_DN16235_c0_g1_i5	Glyma.04G101000.3	98.72340426	170	5	795	2755	2013	53	0	2614

TRINITY_DN16235_c0_g1_i5	Glyma.04G101000.2	96.46017699	170	5	795	2755	2013	53	0	2614

TRINITY_DN16235_c0_g1_i4	Glyma.04G101000.1	97.05882353	169	5	794	2754	2259	299	0	2619

TRINITY_DN16235_c0_g1_i5	Glyma.06G102700.1	96.3622291	160	3	795	2755	2019	53	0	2708

TRINITY_DN11433_c0_g1_i2	Glyma.08G121100.2	100	145	1	410	2376	1	1964	0	2809

TRINITY_DN5950_c0_g1_i2	Glyma.12G228700.7	97.5	142	0	1205	3171	3314	1348	0	2846

TRINITY_DN5950_c0_g1_i2	Glyma.12G228700.8	97.52475248	142	0	1205	3171	3314	1348	0	2846

TRINITY_DN5950_c0_g1_i2	Glyma.12G228700.1	97.51552795	142	0	1205	3171	3314	1348	0	2846

TRINITY_DN5950_c0_g1_i2	Glyma.12G228700.5	96.55172414	142	0	1205	3171	3314	1348	0	2846

TRINITY_DN5950_c0_g1_i2	Glyma.12G228700.2	97.50297265	142	0	1205	3171	3314	1348	0	2846

TRINITY_DN5950_c0_g1_i2	Glyma.12G228700.6	96.56357388	142	0	1205	3171	3314	1348	0	2846

TRINITY_DN5950_c0_g1_i2	Glyma.12G228700.9	100	142	0	1205	3171	2960	994	0	2846

TRINITY_DN5950_c0_g1_i2	Glyma.12G228700.3	97.52747253	142	0	1205	3171	2960	994	0	2846

TRINITY_DN5950_c0_g1_i2	Glyma.12G228700.4	97.5	142	0	1205	3171	2960	994	0	2846

TRINITY_DN15970_c0_g2_i11	Glyma.01G099800.3	100	128	0	246	2212	23	1989	0	2924

TRINITY_DN15529_c1_g1_i2	Glyma.08G039800.11	100	275	11	20	1969	1959	1	0	1958

TRINITY_DN15529_c1_g1_i2	Glyma.08G039800.5	96.35258359	275	11	20	1969	1959	1	0	1958

TRINITY_DN15529_c1_g1_i2	Glyma.08G039800.6	100	275	11	20	1969	1959	1	0	1958

TRINITY_DN15529_c1_g1_i2	Glyma.08G039800.7	95.74468085	275	11	20	1969	1959	1	0	1958

TRINITY_DN15529_c1_g1_i2	Glyma.08G039800.8	99.12280702	275	11	20	1969	1959	1	0	1958

TRINITY_DN15529_c1_g1_i2	Glyma.08G039800.9	96.7032967	275	11	20	1969	1959	1	0	1958

TRINITY_DN15529_c1_g1_i2	Glyma.08G039800.3	97.75280899	275	11	20	1969	1959	1	0	1958

TRINITY_DN15529_c1_g1_i2	Glyma.08G039800.4	98.96907216	275	11	20	1969	1959	1	0	1958

TRINITY_DN15529_c1_g1_i2	Glyma.08G039800.1	98.96907216	275	11	20	1969	1959	1	0	1958

TRINITY_DN15529_c1_g1_i2	Glyma.08G039800.2	95.4248366	275	11	20	1969	1959	1	0	1958

TRINITY_DN15529_c1_g1_i2	Glyma.08G039800.10	95.4248366	275	11	20	1969	1959	1	0	1958

TRINITY_DN15529_c1_g1_i4	Glyma.08G039800.11	99.15966387	275	11	20	1969	1959	1	0	1958

TRINITY_DN15529_c1_g1_i4	Glyma.08G039800.5	99.15966387	275	11	20	1969	1959	1	0	1958

TRINITY_DN15529_c1_g1_i4	Glyma.08G039800.6	99.15966387	275	11	20	1969	1959	1	0	1958

TRINITY_DN15529_c1_g1_i4	Glyma.08G039800.7	99.15966387	275	11	20	1969	1959	1	0	1958

TRINITY_DN15529_c1_g1_i4	Glyma.08G039800.8	98.80952381	275	11	20	1969	1959	1	0	1958

TRINITY_DN15529_c1_g1_i4	Glyma.08G039800.9	98.80952381	275	11	20	1969	1959	1	0	1958

TRINITY_DN15529_c1_g1_i4	Glyma.08G039800.3	98.80952381	275	11	20	1969	1959	1	0	1958

TRINITY_DN15529_c1_g1_i4	Glyma.08G039800.4	98.7804878	275	11	20	1969	1959	1	0	1958

TRINITY_DN15529_c1_g1_i4	Glyma.08G039800.1	100	275	11	20	1969	1959	1	0	1958

TRINITY_DN15529_c1_g1_i4	Glyma.08G039800.2	96.66666667	275	11	20	1969	1959	1	0	1958

TRINITY_DN15529_c1_g1_i4	Glyma.08G039800.10	99.53917051	275	11	20	1969	1959	1	0	1958

TRINITY_DN12718_c0_g1_i1	Glyma.09G272300.5	97.36842105	246	17	3	1946	919	2871	0	2041

TRINITY_DN12718_c0_g1_i1	Glyma.09G272300.8	97.36842105	246	17	3	1946	919	2871	0	2041

TRINITY_DN12718_c0_g1_i1	Glyma.09G272300.1	98.42519685	246	17	3	1946	919	2871	0	2041

TRINITY_DN12718_c0_g1_i1	Glyma.09G272300.6	95.23809524	246	17	3	1946	919	2871	0	2041

TRINITY_DN7216_c0_g1_i1	Glyma.20G080700.1	100	232	9	144	2101	2991	1031	0	2261

TRINITY_DN15519_c1_g1_i2	Glyma.20G017100.1	96.54255319	177	0	341	2306	1977	12	0	2651

TRINITY_DN8503_c0_g1_i1	Glyma.16G213100.3	100	171	2	789	2753	1965	1	0	2671

TRINITY_DN8503_c0_g1_i3	Glyma.16G213100.3	95.11568123	171	2	800	2764	1965	1	0	2671

TRINITY_DN14549_c0_g1_i5	Glyma.17G166800.1	100	143	4	1	1960	222	2187	0	2800

TRINITY_DN14682_c0_g1_i3	Glyma.11G100400.3	97.61904762	142	0	1	1966	3488	1523	0	2844

TRINITY_DN14682_c0_g1_i3	Glyma.11G100400.1	97.66536965	142	0	1	1966	3488	1523	0	2844

TRINITY_DN14682_c0_g1_i3	Glyma.11G100400.2	100	142	0	1	1966	3488	1523	0	2844

TRINITY_DN37437_c0_g1_i1	Glyma.08G000800.1	96.1038961	109	14	176	2108	3	1965	0	2795

TRINITY_DN12758_c0_g1_i1	Glyma.07G033000.1	96.35036496	314	19	388	2327	1958	1	0	1683

TRINITY_DN15808_c0_g1_i2	Glyma.06G202000.2	98.8372093	304	25	206	2154	1	1934	0	1640

TRINITY_DN15808_c0_g1_i2	Glyma.06G202000.3	100	304	25	206	2154	1	1934	0	1640

TRINITY_DN15808_c0_g1_i2	Glyma.06G202000.1	96.84210526	304	25	206	2154	1	1934	0	1640

TRINITY_DN15808_c0_g1_i2	Glyma.06G202000.5	96.34551495	304	25	206	2154	1	1934	0	1640

TRINITY_DN15808_c0_g1_i2	Glyma.06G202000.4	98.91891892	304	25	206	2154	1	1934	0	1640

TRINITY_DN15808_c0_g1_i3	Glyma.06G202000.2	100	304	25	206	2154	1	1934	0	1640

TRINITY_DN15808_c0_g1_i3	Glyma.06G202000.3	96.25	304	25	206	2154	1	1934	0	1640

TRINITY_DN15808_c0_g1_i3	Glyma.06G202000.1	96.55172414	304	25	206	2154	1	1934	0	1640

TRINITY_DN15808_c0_g1_i3	Glyma.06G202000.5	97.91666667	304	25	206	2154	1	1934	0	1640

TRINITY_DN15808_c0_g1_i3	Glyma.06G202000.4	97.91666667	304	25	206	2154	1	1934	0	1640

TRINITY_DN15808_c0_g1_i5	Glyma.06G202000.2	98.5915493	304	25	206	2154	1	1934	0	1640

TRINITY_DN15808_c0_g1_i5	Glyma.06G202000.3	99.543379	304	25	206	2154	1	1934	0	1640

TRINITY_DN15808_c0_g1_i5	Glyma.06G202000.1	97.5	304	25	206	2154	1	1934	0	1640

TRINITY_DN15808_c0_g1_i5	Glyma.06G202000.5	100	304	25	206	2154	1	1934	0	1640

TRINITY_DN15808_c0_g1_i5	Glyma.06G202000.4	95.65217391	304	25	206	2154	1	1934	0	1640

TRINITY_DN15529_c1_g1_i5	Glyma.08G039800.11	100	224	13	20	1969	1959	1	0	2252

TRINITY_DN15529_c1_g1_i5	Glyma.08G039800.5	100	224	13	20	1969	1959	1	0	2252

TRINITY_DN15529_c1_g1_i5	Glyma.08G039800.6	95.08196721	224	13	20	1969	1959	1	0	2252

TRINITY_DN15529_c1_g1_i5	Glyma.08G039800.7	95.08196721	224	13	20	1969	1959	1	0	2252

TRINITY_DN15529_c1_g1_i5	Glyma.08G039800.8	100	224	13	20	1969	1959	1	0	2252

TRINITY_DN15529_c1_g1_i5	Glyma.08G039800.9	100	224	13	20	1969	1959	1	0	2252

TRINITY_DN15529_c1_g1_i5	Glyma.08G039800.3	100	224	13	20	1969	1959	1	0	2252

TRINITY_DN15529_c1_g1_i5	Glyma.08G039800.4	95.71045576	224	13	20	1969	1959	1	0	2252

TRINITY_DN15529_c1_g1_i5	Glyma.08G039800.1	95.71150097	224	13	20	1969	1959	1	0	2252

TRINITY_DN15529_c1_g1_i5	Glyma.08G039800.2	100	224	13	20	1969	1959	1	0	2252

TRINITY_DN15529_c1_g1_i5	Glyma.08G039800.10	100	224	13	20	1969	1959	1	0	2252

TRINITY_DN15369_c0_g6_i2	Glyma.19G172200.1	95.94594595	203	9	515	2464	1965	1	0	2407

TRINITY_DN16807_c0_g1_i1	Glyma.08G235900.1	98.27586207	181	2	234	2198	3101	1146	0	2567

TRINITY_DN16807_c0_g1_i1	Glyma.08G235900.2	96.07843137	181	2	234	2198	3101	1146	0	2567

TRINITY_DN12718_c0_g2_i1	Glyma.09G272300.5	97.8858351	174	13	3	1955	919	2871	0	2510

TRINITY_DN12718_c0_g2_i1	Glyma.09G272300.8	97.88732394	174	13	3	1955	919	2871	0	2510

TRINITY_DN12718_c0_g2_i1	Glyma.09G272300.1	97.88732394	174	13	3	1955	919	2871	0	2510

TRINITY_DN12718_c0_g2_i1	Glyma.09G272300.6	97.88732394	174	13	3	1955	919	2871	0	2510

TRINITY_DN15876_c0_g1_i3	Glyma.06G040600.3	95.65217391	172	5	132	2089	180	2143	0	2625

TRINITY_DN15876_c0_g1_i3	Glyma.06G040600.4	98.55072464	172	5	132	2089	180	2143	0	2625

TRINITY_DN5950_c0_g1_i2	Glyma.13G271100.1	97.51243781	161	2	1206	3167	3316	1352	0	2717

TRINITY_DN5950_c0_g1_i2	Glyma.13G271100.6	96.5625	161	2	1206	3167	3316	1352	0	2717

TRINITY_DN15369_c0_g6_i1	Glyma.19G172200.1	100	157	9	515	2464	1965	1	0	2662

TRINITY_DN5209_c0_g1_i1	Glyma.02G165800.2	95.40229885	155	10	144	2099	28	1983	0	2654

TRINITY_DN5209_c0_g1_i1	Glyma.02G165800.1	95.40229885	155	10	144	2099	28	1983	0	2654

TRINITY_DN14764_c2_g1_i2	Glyma.14G084500.1	100	151	8	345	2303	1959	1	0	2715

TRINITY_DN16414_c0_g1_i3	Glyma.05G216600.1	96.35036496	280	10	168	2124	1963	5	0	1999

TRINITY_DN14257_c0_g2_i1	Glyma.02G037100.5	97.16088328	273	16	134	2087	1	1957	0	2004

TRINITY_DN14257_c0_g2_i1	Glyma.02G037100.6	97.14285714	273	16	134	2087	1	1957	0	2004

TRINITY_DN14257_c0_g2_i1	Glyma.02G037100.1	97.14285714	273	16	134	2087	1	1957	0	2004

TRINITY_DN14257_c0_g2_i1	Glyma.02G037100.4	96.56750572	273	16	134	2087	1	1957	0	2004

TRINITY_DN14257_c0_g2_i1	Glyma.02G037100.2	97.14285714	273	16	134	2087	1	1957	0	2004

TRINITY_DN14257_c0_g2_i1	Glyma.02G037100.3	97.14285714	273	16	134	2087	1	1957	0	2004

TRINITY_DN14257_c0_g2_i1	Glyma.02G037100.8	97.14285714	273	16	134	2087	1	1957	0	2004

TRINITY_DN16256_c1_g6_i3	Glyma.U032300.1	95.28985507	243	18	545	2492	1952	1	0	2100

TRINITY_DN16255_c1_g1_i3	Glyma.15G014000.1	98.31223629	186	6	360	2321	1959	1	0	2551

TRINITY_DN13914_c0_g1_i2	Glyma.20G013200.1	97.43589744	147	21	388	2336	1956	20	0	2542

TRINITY_DN15410_c0_g2_i1	Glyma.20G097000.1	95.5	125	9	1528	3490	1948	1	0	2824

TRINITY_DN15410_c0_g2_i2	Glyma.20G097000.1	95	125	9	1453	3415	1948	1	0	2824

TRINITY_DN11865_c0_g1_i1	Glyma.09G048300.1	100	349	27	325	2277	3771	1834	0	1465

TRINITY_DN11865_c0_g1_i2	Glyma.09G048300.1	100	349	27	312	2264	3771	1834	0	1465

TRINITY_DN15510_c0_g1_i5	Glyma.09G168400.1	99.08256881	313	19	96	2031	115	2071	0	1677

TRINITY_DN15944_c1_g1_i4	Glyma.17G139200.1	99.44751381	302	11	521	2476	2302	350	0	1842

TRINITY_DN14257_c0_g2_i1	Glyma.01G028100.4	97.16262976	268	11	134	2087	1	1957	0	2043

TRINITY_DN14257_c0_g2_i1	Glyma.01G028100.2	97.15302491	268	11	134	2087	1	1957	0	2043

TRINITY_DN14257_c0_g2_i1	Glyma.01G028100.3	97.14285714	268	11	134	2087	1	1957	0	2043

TRINITY_DN14257_c0_g2_i1	Glyma.01G028100.1	97.14285714	268	11	134	2087	1	1957	0	2043

TRINITY_DN12718_c0_g1_i1	Glyma.18G217000.4	98.61111111	252	11	3	1946	919	2874	0	2061

TRINITY_DN12718_c0_g1_i1	Glyma.18G217000.3	96.62921348	252	11	3	1946	919	2874	0	2061

TRINITY_DN12718_c0_g1_i1	Glyma.18G217000.1	100	252	11	3	1946	919	2874	0	2061

TRINITY_DN12718_c0_g1_i1	Glyma.18G217000.2	96.15384615	252	11	3	1946	919	2874	0	2061

TRINITY_DN12718_c0_g1_i1	Glyma.09G272300.7	98.68421053	246	15	3	1946	919	2868	0	2056

TRINITY_DN12718_c0_g1_i1	Glyma.09G272300.3	98.68421053	246	15	3	1946	919	2868	0	2056

TRINITY_DN12718_c0_g1_i1	Glyma.09G272300.4	97.36842105	246	15	3	1946	919	2868	0	2056

TRINITY_DN12718_c0_g1_i1	Glyma.09G272300.2	97.36842105	246	15	3	1946	919	2868	0	2056

TRINITY_DN15495_c0_g1_i1	Glyma.02G120000.2	95.65217391	239	25	129	2060	9	1949	0	1958

TRINITY_DN15495_c0_g1_i1	Glyma.02G120000.1	95.65217391	239	25	129	2060	9	1949	0	1958

TRINITY_DN15495_c0_g1_i2	Glyma.02G120000.2	95.5654102	239	25	129	2060	9	1949	0	1958

TRINITY_DN15495_c0_g1_i2	Glyma.02G120000.1	97.08737864	239	25	129	2060	9	1949	0	1958

TRINITY_DN15495_c0_g1_i3	Glyma.02G120000.1	98.13664596	239	25	129	2060	9	1949	0	1958

TRINITY_DN15495_c0_g1_i3	Glyma.02G120000.2	99.0990991	239	25	129	2060	9	1949	0	1958

TRINITY_DN15048_c0_g2_i2	Glyma.09G040000.1	100	223	4	584	2545	2013	55	0	2357

TRINITY_DN13713_c0_g1_i4	Glyma.15G042700.7	100	219	10	296	2242	1	1953	0	2244

TRINITY_DN15369_c0_g6_i2	Glyma.03G171100.1	97.9757085	208	6	515	2464	1962	1	0	2383

TRINITY_DN13072_c0_g1_i1	Glyma.14G076200.1	96.66666667	194	6	134	2089	1	1953	0	2440

TRINITY_DN16255_c1_g1_i3	Glyma.13G360000.1	98.31223629	190	10	360	2321	1950	1	0	2483

TRINITY_DN5950_c0_g1_i2	Glyma.13G271100.2	97.5	162	0	1205	3167	3314	1352	0	2728

TRINITY_DN5950_c0_g1_i2	Glyma.13G271100.3	97.5	162	0	1205	3167	3314	1352	0	2728

TRINITY_DN15235_c0_g1_i1	Glyma.10G227400.6	97.64705882	158	5	4	1964	412	2371	0	2717

TRINITY_DN15235_c0_g1_i1	Glyma.10G227400.4	97.25274725	158	5	4	1964	412	2371	0	2717

TRINITY_DN15235_c0_g1_i1	Glyma.10G227400.3	98.20051414	158	5	4	1964	412	2371	0	2717

TRINITY_DN15235_c0_g1_i1	Glyma.10G227400.1	96.86346863	158	5	4	1964	412	2371	0	2717

TRINITY_DN15235_c0_g1_i1	Glyma.10G227400.2	95.13274336	158	5	4	1964	412	2371	0	2717

TRINITY_DN15235_c0_g1_i1	Glyma.10G227400.5	95.13513514	158	5	4	1964	412	2371	0	2717

TRINITY_DN1479_c0_g1_i2	Glyma.04G016900.1	100	156	19	142	2079	1950	1	0	2516

TRINITY_DN15369_c0_g6_i1	Glyma.03G171100.1	99.23664122	150	6	515	2464	1962	1	0	2704

TRINITY_DN11025_c0_g1_i1	Glyma.08G125700.1	98.77675841	145	9	1	1954	261	2217	0	2724

TRINITY_DN10502_c0_g2_i1	Glyma.17G038100.3	98.86363636	137	10	288	2241	5	1958	0	2750

TRINITY_DN15970_c0_g2_i11	Glyma.01G099800.4	100	127	0	250	2212	3	1965	0	2922

TRINITY_DN12523_c0_g1_i1	Glyma.20G106300.1	95.28301887	100	3	183	2141	3	1964	0	3038

TRINITY_DN14314_c0_g1_i2	Glyma.19G043200.1	100	462	33	232	2167	211	2155	0	785

TRINITY_DN14314_c0_g1_i2	Glyma.13G048700.1	100	441	31	232	2167	214	2158	0	902

TRINITY_DN6249_c0_g1_i2	Glyma.01G211900.2	100	324	30	186	2115	1	1945	0	1511

TRINITY_DN15361_c1_g1_i1	Glyma.04G147000.1	95.73170732	302	30	189	2117	1	1953	0	1679

TRINITY_DN12718_c0_g2_i1	Glyma.18G217000.4	97.88732394	192	10	3	1955	919	2874	0	2462

TRINITY_DN12718_c0_g2_i1	Glyma.18G217000.3	97.88732394	192	10	3	1955	919	2874	0	2462

TRINITY_DN12718_c0_g2_i1	Glyma.18G217000.1	97.88732394	192	10	3	1955	919	2874	0	2462

TRINITY_DN12718_c0_g2_i1	Glyma.18G217000.2	97.88235294	192	10	3	1955	919	2874	0	2462

TRINITY_DN18011_c0_g1_i1	Glyma.04G250200.2	97.95918367	187	26	19	1953	1693	3621	0	2200

TRINITY_DN20930_c0_g1_i1	Glyma.17G012200.1	100	182	5	66	2018	1	1950	0	2479

TRINITY_DN9359_c0_g1_i1	Glyma.10G251100.1	100	179	19	555	2513	2725	812	0	2302

TRINITY_DN9359_c0_g1_i1	Glyma.10G251100.3	100	179	19	555	2513	2428	515	0	2302

TRINITY_DN12718_c0_g2_i1	Glyma.09G272300.7	97.88135593	174	12	3	1955	919	2868	0	2523

TRINITY_DN12718_c0_g2_i1	Glyma.09G272300.3	97.88359788	174	12	3	1955	919	2868	0	2523

TRINITY_DN12718_c0_g2_i1	Glyma.09G272300.4	97.88359788	174	12	3	1955	919	2868	0	2523

TRINITY_DN12718_c0_g2_i1	Glyma.09G272300.2	97.88359788	174	12	3	1955	919	2868	0	2523

TRINITY_DN15529_c1_g1_i1	Glyma.08G039800.11	97.38372093	173	3	568	2523	1959	1	0	2606

TRINITY_DN15529_c1_g1_i1	Glyma.08G039800.5	100	173	3	568	2523	1959	1	0	2606

TRINITY_DN15529_c1_g1_i1	Glyma.08G039800.6	98.37837838	173	3	568	2523	1959	1	0	2606

TRINITY_DN15529_c1_g1_i1	Glyma.08G039800.7	98.09160305	173	3	568	2523	1959	1	0	2606

TRINITY_DN15529_c1_g1_i1	Glyma.08G039800.8	100	173	3	568	2523	1959	1	0	2606

TRINITY_DN15529_c1_g1_i1	Glyma.08G039800.9	100	173	3	568	2523	1959	1	0	2606

TRINITY_DN15529_c1_g1_i1	Glyma.08G039800.3	96.46017699	173	3	568	2523	1959	1	0	2606

TRINITY_DN15529_c1_g1_i1	Glyma.08G039800.4	96.03174603	173	3	568	2523	1959	1	0	2606

TRINITY_DN15529_c1_g1_i1	Glyma.08G039800.1	96.03174603	173	3	568	2523	1959	1	0	2606

TRINITY_DN15529_c1_g1_i1	Glyma.08G039800.2	96.03174603	173	3	568	2523	1959	1	0	2606

TRINITY_DN15529_c1_g1_i1	Glyma.08G039800.10	96.03174603	173	3	568	2523	1959	1	0	2606

TRINITY_DN15624_c0_g1_i1	Glyma.06G106200.1	100	165	0	189	2150	1	1962	0	2710

TRINITY_DN5950_c0_g1_i2	Glyma.13G271100.4	97.5	162	0	1206	3167	3313	1352	0	2726

TRINITY_DN5950_c0_g1_i2	Glyma.13G271100.5	97.5	162	0	1206	3167	3313	1352	0	2726

TRINITY_DN3370_c0_g1_i1	Glyma.15G045600.1	95.66666667	159	11	733	2678	1950	5	0	2536

TRINITY_DN15721_c0_g1_i4	Glyma.16G062200.1	97.7443609	156	8	625	2574	13	1965	0	2623

TRINITY_DN15721_c0_g1_i6	Glyma.16G062200.1	98.98989899	156	8	723	2672	13	1965	0	2623

TRINITY_DN15913_c1_g2_i2	Glyma.19G103200.3	100	139	1	340	2301	1956	1	0	2815

TRINITY_DN14549_c0_g1_i5	Glyma.05G098800.1	97.5	129	1	2	1960	271	2232	0	2889

TRINITY_DN15529_c1_g1_i3	Glyma.08G039800.11	100	120	3	568	2523	1959	1	0	2900

TRINITY_DN15529_c1_g1_i3	Glyma.08G039800.5	98.6013986	120	3	568	2523	1959	1	0	2900

TRINITY_DN15529_c1_g1_i3	Glyma.08G039800.6	99.25187032	120	3	568	2523	1959	1	0	2900

TRINITY_DN15529_c1_g1_i3	Glyma.08G039800.7	96.05263158	120	3	568	2523	1959	1	0	2900

TRINITY_DN15529_c1_g1_i3	Glyma.08G039800.8	96.05263158	120	3	568	2523	1959	1	0	2900

TRINITY_DN15529_c1_g1_i3	Glyma.08G039800.9	96.05263158	120	3	568	2523	1959	1	0	2900

TRINITY_DN15529_c1_g1_i3	Glyma.08G039800.3	96.05263158	120	3	568	2523	1959	1	0	2900

TRINITY_DN15529_c1_g1_i3	Glyma.08G039800.4	96.58536585	120	3	568	2523	1959	1	0	2900

TRINITY_DN15529_c1_g1_i3	Glyma.08G039800.1	98.58490566	120	3	568	2523	1959	1	0	2900

TRINITY_DN15529_c1_g1_i3	Glyma.08G039800.2	98.58490566	120	3	568	2523	1959	1	0	2900

TRINITY_DN15529_c1_g1_i3	Glyma.08G039800.10	97.61904762	120	3	568	2523	1959	1	0	2900

TRINITY_DN9745_c0_g1_i1	Glyma.02G238300.2	100	111	6	334	2286	2247	292	0	2911

TRINITY_DN9745_c0_g1_i1	Glyma.02G238300.1	100	111	6	334	2286	2247	292	0	2911

TRINITY_DN9745_c0_g1_i3	Glyma.02G238300.2	100	111	6	458	2410	2247	292	0	2911

TRINITY_DN9745_c0_g1_i3	Glyma.02G238300.1	100	111	6	458	2410	2247	292	0	2911

TRINITY_DN15944_c1_g1_i4	Glyma.05G056800.1	98.85057471	315	7	521	2478	2308	354	0	1818

TRINITY_DN15369_c0_g6_i1	Glyma.18G289100.1	95.34883721	315	16	515	2464	1950	1	0	1735

TRINITY_DN15369_c0_g6_i2	Glyma.18G289100.1	100	314	17	515	2464	1950	1	0	1740

TRINITY_DN15778_c0_g1_i4	Glyma.07G144500.2	98.03921569	275	18	1032	2986	1937	1	0	1905

TRINITY_DN9527_c0_g1_i1	Glyma.08G199600.1	97.61904762	228	28	264	2186	1923	1	0	1868

TRINITY_DN9527_c0_g1_i1	Glyma.08G199600.2	100	228	28	264	2186	1923	1	0	1868

TRINITY_DN9850_c0_g1_i1	Glyma.18G207500.4	100	203	0	110	2070	56	2016	0	2497

TRINITY_DN9850_c0_g1_i1	Glyma.18G207500.3	95	203	0	110	2070	47	2007	0	2497

TRINITY_DN9850_c0_g1_i1	Glyma.18G207500.6	97.01492537	203	1	110	2070	56	2007	0	2438

TRINITY_DN9850_c0_g1_i1	Glyma.18G207500.2	100	203	1	110	2070	47	1998	0	2438

TRINITY_DN13018_c0_g1_i1	Glyma.16G093400.1	100	203	29	391	2274	1959	1	0	1986

TRINITY_DN7424_c0_g1_i1	Glyma.05G246700.2	98.75	200	2	47	2006	71	2030	0	2501

TRINITY_DN15944_c1_g1_i2	Glyma.17G139200.1	95.52238806	180	8	621	2577	2303	350	0	2553

TRINITY_DN2601_c0_g1_i1	Glyma.06G091300.4	99.00497512	160	6	1129	3086	2951	1003	0	2638

TRINITY_DN13058_c0_g1_i1	Glyma.09G193300.1	100	157	4	575	2528	3408	1452	0	2680

TRINITY_DN9072_c0_g2_i1	Glyma.06G232100.1	95.92760181	141	7	161	2118	1	1949	0	2743

TRINITY_DN11865_c0_g1_i1	Glyma.15G155600.1	100	349	22	325	2277	3771	1837	0	1480

TRINITY_DN11865_c0_g1_i2	Glyma.15G155600.1	100	349	22	312	2264	3771	1837	0	1480

TRINITY_DN11708_c0_g1_i1	Glyma.02G219800.1	100	338	36	265	2181	1947	1	0	1386

TRINITY_DN11708_c0_g1_i2	Glyma.02G219800.1	95.75289575	338	36	265	2181	1947	1	0	1386

TRINITY_DN11708_c0_g1_i3	Glyma.02G219800.1	95.38461538	338	36	265	2181	1947	1	0	1386

TRINITY_DN15529_c1_g1_i3	Glyma.07G089000.6	99.15966387	282	8	568	2523	1950	1	0	1967

TRINITY_DN15529_c1_g1_i3	Glyma.07G089000.5	96.12403101	282	8	568	2523	1950	1	0	1967

TRINITY_DN15619_c0_g2_i1	Glyma.04G184000.1	97.79507134	275	10	1193	3138	187	2143	0	1986

TRINITY_DN15619_c0_g2_i1	Glyma.04G184000.3	97.70114943	275	10	1193	3138	187	2143	0	1986

TRINITY_DN15619_c0_g2_i1	Glyma.04G184000.4	98.42519685	275	10	1193	3138	124	2080	0	1986

TRINITY_DN15529_c1_g1_i1	Glyma.07G089000.6	96.8	226	8	568	2523	1950	1	0	2278

TRINITY_DN15529_c1_g1_i1	Glyma.07G089000.5	96.8	226	8	568	2523	1950	1	0	2278

TRINITY_DN13713_c0_g1_i4	Glyma.15G042700.6	100	219	9	296	2242	1	1950	0	2257

TRINITY_DN15944_c1_g1_i2	Glyma.05G056800.1	95.52238806	183	3	621	2579	2309	354	0	2573

TRINITY_DN14372_c0_g2_i1	Glyma.10G150500.1	95.55555556	173	14	585	2531	1	1947	0	2494

TRINITY_DN11865_c0_g1_i1	Glyma.05G134800.1	100	144	2	319	2277	3816	1858	0	2809

TRINITY_DN11865_c0_g1_i2	Glyma.05G134800.1	100	144	2	306	2264	3816	1858	0	2809

TRINITY_DN11025_c0_g1_i1	Glyma.05G167300.1	98.7755102	130	4	1	1954	255	2211	0	2841

TRINITY_DN14695_c0_g1_i1	Glyma.06G067100.2	97.1291866	114	3	564	2519	4	1962	0	2955

TRINITY_DN14578_c0_g1_i2	Glyma.12G001200.1	99.19354839	107	4	154	2108	2754	797	0	2981

TRINITY_DN15369_c0_g1_i1	Glyma.12G064000.1	98.27586207	342	16	313	2262	1950	1	0	1607

TRINITY_DN15369_c0_g1_i1	Glyma.11G140500.1	95.65217391	330	17	313	2262	1950	1	0	1674

TRINITY_DN15992_c0_g1_i1	Glyma.05G035100.1	95.65217391	298	15	2858	4809	1941	2	0	1799

TRINITY_DN15992_c0_g1_i2	Glyma.05G035100.1	95.54030875	298	15	2726	4677	1941	2	0	1799

TRINITY_DN15992_c0_g1_i3	Glyma.05G035100.1	100	298	15	2659	4610	1941	2	0	1799

TRINITY_DN15510_c0_g1_i1	Glyma.16G218100.1	98.79518072	279	27	653	2585	300	2244	0	1814

TRINITY_DN15510_c0_g1_i1	Glyma.16G218100.3	99.5951417	279	27	653	2585	300	2244	0	1814

TRINITY_DN15510_c0_g1_i4	Glyma.16G218100.1	98.68421053	279	27	679	2611	300	2244	0	1814

TRINITY_DN15510_c0_g1_i4	Glyma.16G218100.3	98.8700565	279	27	679	2611	300	2244	0	1814

TRINITY_DN395_c0_g1_i1	Glyma.17G085000.1	96.36363636	238	7	355	2304	1996	44	0	2202

TRINITY_DN36824_c0_g1_i1	Glyma.06G027100.1	97.8021978	205	19	612	2545	609	2526	0	2056

TRINITY_DN36824_c0_g1_i1	Glyma.06G027100.2	100	204	20	612	2545	609	2514	0	1984

TRINITY_DN36824_c0_g1_i1	Glyma.04G027100.1	96.33507853	199	31	612	2545	609	2526	0	2089

TRINITY_DN37038_c0_g1_i1	Glyma.19G166900.3	98.36956522	194	9	343	2299	1963	16	0	2459

TRINITY_DN12874_c0_g1_i2	Glyma.04G152200.1	95.83333333	164	4	519	2450	1	1959	0	2534

TRINITY_DN12874_c0_g1_i2	Glyma.04G152200.6	95.83333333	164	4	519	2450	1	1959	0	2534

TRINITY_DN12874_c0_g1_i3	Glyma.04G152200.1	95.83333333	164	4	519	2450	1	1959	0	2534

TRINITY_DN12874_c0_g1_i3	Glyma.04G152200.6	95.83333333	164	4	519	2450	1	1959	0	2534

TRINITY_DN12874_c0_g1_i2	Glyma.04G152200.4	95.83333333	164	5	519	2450	1	1953	0	2495

TRINITY_DN12874_c0_g1_i2	Glyma.04G152200.5	95.83333333	164	5	519	2450	1	1953	0	2495

TRINITY_DN12874_c0_g1_i3	Glyma.04G152200.4	95.83333333	164	5	519	2450	1	1953	0	2495

TRINITY_DN12874_c0_g1_i3	Glyma.04G152200.5	95.83333333	164	5	519	2450	1	1953	0	2495

TRINITY_DN11865_c0_g1_i1	Glyma.08G090000.1	100	161	0	319	2277	3828	1870	0	2726

TRINITY_DN11865_c0_g1_i2	Glyma.08G090000.1	100	161	0	306	2264	3828	1870	0	2726

TRINITY_DN13842_c0_g1_i1	Glyma.08G127700.4	100	120	0	1	1959	373	2331	0	2953

TRINITY_DN13842_c0_g1_i1	Glyma.08G127700.3	99.01960784	120	0	1	1959	373	2331	0	2953

TRINITY_DN13842_c0_g1_i1	Glyma.08G127700.1	100	120	0	1	1959	373	2331	0	2953

TRINITY_DN13842_c0_g1_i1	Glyma.08G127700.5	96.25	120	0	1	1959	367	2325	0	2953

TRINITY_DN13842_c0_g1_i1	Glyma.08G127700.2	99.05660377	120	0	1	1959	367	2325	0	2953

TRINITY_DN13842_c0_g1_i1	Glyma.05G169400.3	100	119	1	1	1959	367	2322	0	2939

TRINITY_DN13842_c0_g1_i1	Glyma.05G169400.1	100	119	1	1	1959	367	2322	0	2939

TRINITY_DN13842_c0_g1_i1	Glyma.05G169400.2	100	119	1	1	1959	367	2322	0	2939

TRINITY_DN9745_c0_g1_i1	Glyma.14G207200.1	100	113	4	334	2286	2253	301	0	2915

TRINITY_DN9745_c0_g1_i3	Glyma.14G207200.1	100	113	4	458	2410	2253	301	0	2915

TRINITY_DN14578_c0_g1_i2	Glyma.08G238800.1	97.89473684	110	3	154	2108	2754	797	0	2976

TRINITY_DN15048_c0_g2_i2	Glyma.15G145200.1	100	214	8	584	2539	2010	61	0	2366

TRINITY_DN11318_c0_g1_i1	Glyma.11G020900.1	97.82608696	207	18	1283	3192	1958	7	0	2121

TRINITY_DN8957_c0_g1_i4	Glyma.17G102800.2	98.4375	198	20	87	2027	2123	211	0	2119

TRINITY_DN8957_c0_g1_i4	Glyma.17G102800.1	98.46153846	198	20	87	2027	2123	211	0	2119

TRINITY_DN15540_c0_g1_i6	Glyma.01G109800.1	100	198	35	629	2560	1920	1	0	2106

TRINITY_DN13914_c0_g1_i2	Glyma.07G216200.1	97.50692521	157	10	388	2336	1965	20	0	2610

TRINITY_DN8298_c0_g1_i1	Glyma.11G181000.1	99.356085	156	21	318	2231	1989	38	0	2429

TRINITY_DN1744_c0_g1_i1	Glyma.15G032800.1	98.11676083	147	2	1	1957	273	2229	0	2789

TRINITY_DN15619_c0_g2_i3	Glyma.17G088800.1	97.26027397	141	3	912	2867	1	1957	0	2815

TRINITY_DN10502_c0_g2_i1	Glyma.17G038100.5	98.86363636	136	10	293	2241	25	1973	0	2747

TRINITY_DN10502_c0_g2_i1	Glyma.17G038100.1	98.86792453	136	10	293	2241	25	1973	0	2747

TRINITY_DN10502_c0_g2_i1	Glyma.17G038100.2	98.8700565	136	10	293	2241	25	1973	0	2747

TRINITY_DN1744_c0_g1_i1	Glyma.13G341600.1	98.13084112	131	2	1	1957	273	2229	0	2878

TRINITY_DN16356_c0_g1_i2	Glyma.08G025900.1	100	116	0	214	2171	170	2127	0	2974

TRINITY_DN16356_c0_g1_i5	Glyma.08G025900.1	100	113	2	214	2165	170	2127	0	2952

TRINITY_DN16807_c0_g1_i1	Glyma.09G099900.1	98.8372093	312	9	246	2198	3065	1119	0	1796

TRINITY_DN16807_c0_g1_i1	Glyma.09G099900.2	98.8372093	312	9	246	2198	2993	1047	0	1796

TRINITY_DN16807_c0_g1_i1	Glyma.09G099900.3	96.47355164	312	9	246	2198	2645	699	0	1796

TRINITY_DN13842_c0_g1_i1	Glyma.15G046400.5	96.5625	301	20	1	1941	367	2304	0	1720

TRINITY_DN13842_c0_g1_i1	Glyma.15G046400.6	98.41269841	301	20	1	1941	367	2304	0	1720

TRINITY_DN13842_c0_g1_i1	Glyma.15G046400.3	98.41269841	301	20	1	1941	367	2304	0	1720

TRINITY_DN13842_c0_g1_i1	Glyma.15G046400.4	99.01477833	301	20	1	1941	367	2304	0	1720

TRINITY_DN13842_c0_g1_i1	Glyma.15G046400.1	98.71794872	301	20	1	1941	367	2304	0	1720

TRINITY_DN16256_c1_g6_i2	Glyma.U032300.1	100	211	15	545	2480	1952	1	0	2278

TRINITY_DN8957_c0_g1_i3	Glyma.17G102800.2	98.46153846	181	15	87	2036	2123	211	0	2281

TRINITY_DN8957_c0_g1_i3	Glyma.17G102800.1	98.46153846	181	15	87	2036	2123	211	0	2281

TRINITY_DN15028_c0_g1_i3	Glyma.04G203600.1	97.12230216	175	7	1304	3257	2017	68	0	2580

TRINITY_DN16255_c1_g1_i2	Glyma.15G014000.1	99.51456311	165	4	213	2168	1953	1	0	2667

TRINITY_DN38682_c0_g1_i1	Glyma.01G077400.3	96.72131148	113	0	1	1957	978	2934	0	2988

TRINITY_DN38682_c0_g1_i1	Glyma.01G077400.2	96.72131148	113	0	1	1957	978	2934	0	2988

TRINITY_DN38682_c0_g1_i1	Glyma.01G077400.1	96.72578445	113	0	1	1957	978	2934	0	2988

TRINITY_DN9051_c1_g1_i1	Glyma.18G297900.3	97.10144928	110	2	244	2199	4791	2836	0	2992

TRINITY_DN9051_c1_g1_i1	Glyma.18G297900.2	97.11191336	110	2	244	2199	4791	2836	0	2992

TRINITY_DN9051_c1_g2_i1	Glyma.18G297900.3	97.11538462	110	2	1626	3581	4791	2836	0	2992

TRINITY_DN9051_c1_g2_i1	Glyma.18G297900.2	97.11538462	110	2	1626	3581	4791	2836	0	2992

TRINITY_DN9519_c0_g1_i1	Glyma.02G301400.2	96.21993127	366	15	337	2267	1949	1	0	1378

TRINITY_DN9519_c0_g1_i1	Glyma.02G301400.1	96.21993127	366	15	337	2267	1949	1	0	1378

TRINITY_DN12951_c0_g1_i1	Glyma.06G068400.1	96.41693811	364	42	894	2825	1917	1	0	1188

TRINITY_DN15369_c0_g1_i1	Glyma.18G289600.1	100	323	14	313	2262	1944	1	0	1707

TRINITY_DN15369_c0_g6_i1	Glyma.18G289600.1	100	316	8	515	2464	1944	1	0	1746

TRINITY_DN15495_c0_g1_i1	Glyma.01G061700.1	95.65217391	247	37	129	2060	240	2147	0	1779

TRINITY_DN15495_c0_g1_i2	Glyma.01G061700.1	97.08737864	247	37	129	2060	240	2147	0	1779

TRINITY_DN15495_c0_g1_i3	Glyma.01G061700.1	97.72727273	247	37	129	2060	240	2147	0	1779

TRINITY_DN13842_c0_g1_i2	Glyma.15G046400.5	95	219	15	1	1932	367	2316	0	2206

TRINITY_DN13842_c0_g1_i2	Glyma.15G046400.6	95	219	15	1	1932	367	2316	0	2206

TRINITY_DN13842_c0_g1_i2	Glyma.15G046400.3	98.13084112	219	15	1	1932	367	2316	0	2206

TRINITY_DN13842_c0_g1_i2	Glyma.15G046400.4	100	219	15	1	1932	367	2316	0	2206

TRINITY_DN13842_c0_g1_i2	Glyma.15G046400.1	95.67901235	219	15	1	1932	367	2316	0	2206

TRINITY_DN9125_c0_g1_i2	Glyma.15G001500.1	98.75311721	209	8	247	2189	3261	1310	0	2344

TRINITY_DN15529_c1_g1_i5	Glyma.09G188000.3	97.61336516	204	5	20	1969	1956	1	0	2444

TRINITY_DN15529_c1_g1_i5	Glyma.09G188000.4	100	204	5	20	1969	1956	1	0	2444

TRINITY_DN15529_c1_g1_i5	Glyma.09G188000.1	98.9010989	204	5	20	1969	1956	1	0	2444

TRINITY_DN15529_c1_g1_i5	Glyma.09G188000.2	98.9010989	204	5	20	1969	1956	1	0	2444

TRINITY_DN15529_c1_g1_i5	Glyma.09G188000.5	96.96969697	204	5	20	1969	1956	1	0	2444

TRINITY_DN2777_c0_g1_i1	Glyma.02G037200.1	95.86206897	176	8	148	2100	1947	1	0	2560

TRINITY_DN2777_c0_g2_i1	Glyma.02G037200.1	96.21621622	174	8	148	2100	1947	1	0	2571

TRINITY_DN16255_c1_g1_i2	Glyma.13G360000.1	99.51456311	167	8	213	2168	1944	1	0	2610

TRINITY_DN12675_c0_g1_i1	Glyma.17G186500.2	100	164	3	400	2352	1953	1	0	2665

TRINITY_DN16256_c1_g6_i2	Glyma.20G194100.1	97.72727273	156	14	543	2480	1941	1	0	2534

TRINITY_DN4009_c0_g1_i1	Glyma.19G004200.1	99.00990099	155	14	70	2022	1	1923	0	2521

TRINITY_DN15529_c1_g1_i2	Glyma.09G188000.3	95.34883721	147	5	20	1969	1956	1	0	2760

TRINITY_DN15529_c1_g1_i2	Glyma.09G188000.4	99.6	147	5	20	1969	1956	1	0	2760

TRINITY_DN15529_c1_g1_i2	Glyma.09G188000.1	99.74811083	147	5	20	1969	1956	1	0	2760

TRINITY_DN15529_c1_g1_i2	Glyma.09G188000.2	97.5	147	5	20	1969	1956	1	0	2760

TRINITY_DN15529_c1_g1_i2	Glyma.09G188000.5	96.04938272	147	5	20	1969	1956	1	0	2760

TRINITY_DN15529_c1_g1_i4	Glyma.09G188000.3	96.66666667	147	5	20	1969	1956	1	0	2760

TRINITY_DN15529_c1_g1_i4	Glyma.09G188000.4	97.2972973	147	5	20	1969	1956	1	0	2760

TRINITY_DN15529_c1_g1_i4	Glyma.09G188000.1	100	147	5	20	1969	1956	1	0	2760

TRINITY_DN15529_c1_g1_i4	Glyma.09G188000.2	96.60056657	147	5	20	1969	1956	1	0	2760

TRINITY_DN15529_c1_g1_i4	Glyma.09G188000.5	100	147	5	20	1969	1956	1	0	2760

TRINITY_DN12892_c0_g4_i1	Glyma.11G103100.15	100	135	2	305	2257	139	2088	0	2806

TRINITY_DN12892_c0_g4_i1	Glyma.11G103100.16	100	135	2	305	2257	139	2088	0	2806

TRINITY_DN12892_c0_g4_i5	Glyma.11G103100.15	100	135	2	305	2257	139	2088	0	2806

TRINITY_DN12892_c0_g4_i5	Glyma.11G103100.16	100	135	2	305	2257	139	2088	0	2806

TRINITY_DN13164_c0_g1_i2	Glyma.05G156900.2	97.5	92	0	1349	3304	1	1956	0	3103

TRINITY_DN13164_c0_g1_i4	Glyma.05G156900.2	97.5	92	0	1398	3353	1	1956	0	3103

TRINITY_DN8879_c0_g2_i1	Glyma.10G169800.1	98.95833333	363	29	114	2045	89	2026	0	1341

TRINITY_DN13842_c0_g1_i1	Glyma.08G186400.3	100	300	14	1	1946	370	2304	0	1760

TRINITY_DN13842_c0_g1_i1	Glyma.08G186400.2	100	300	14	1	1946	370	2304	0	1760

TRINITY_DN13842_c0_g1_i1	Glyma.08G186400.1	97.6744186	300	14	1	1946	370	2304	0	1760

TRINITY_DN15048_c0_g2_i1	Glyma.15G145200.4	98.24561404	193	10	647	2596	1947	1	0	2457

TRINITY_DN15048_c0_g2_i1	Glyma.15G145200.3	98.42519685	193	10	647	2596	1947	1	0	2457

TRINITY_DN15048_c0_g2_i1	Glyma.15G145200.2	98.42519685	193	10	647	2596	1947	1	0	2457

TRINITY_DN14499_c1_g1_i2	Glyma.02G108100.1	98.27586207	142	6	448	2397	2	1952	0	2765

TRINITY_DN12892_c0_g4_i1	Glyma.11G103100.11	100	135	2	305	2256	139	2087	0	2804

TRINITY_DN12892_c0_g4_i1	Glyma.11G103100.12	100	135	2	305	2256	139	2087	0	2804

TRINITY_DN12892_c0_g4_i5	Glyma.11G103100.11	100	135	2	305	2256	139	2087	0	2804

TRINITY_DN12892_c0_g4_i5	Glyma.11G103100.12	100	135	2	305	2256	139	2087	0	2804

TRINITY_DN12945_c0_g1_i3	Glyma.17G112200.1	100	128	4	280	2234	1	1949	0	2863

TRINITY_DN11214_c0_g1_i1	Glyma.13G048700.1	98.9010989	124	0	1	1955	2203	249	0	2924

TRINITY_DN12945_c0_g1_i3	Glyma.13G159100.1	100	121	3	280	2234	1	1952	0	2920

TRINITY_DN16345_c0_g1_i4	Glyma.09G182600.1	98.44632768	98	2	440	2394	3717	1772	0	3009

TRINITY_DN15276_c0_g1_i2	Glyma.04G211300.1	99.03846154	288	8	860	2809	485	2433	0	1954

TRINITY_DN15276_c0_g1_i4	Glyma.04G211300.1	100	247	8	860	2809	485	2433	0	2182

TRINITY_DN868_c0_g1_i1	Glyma.02G301400.2	100	214	11	167	2109	1	1949	0	2320

TRINITY_DN868_c0_g1_i1	Glyma.02G301400.1	100	214	11	167	2109	1	1949	0	2320

TRINITY_DN18789_c0_g1_i1	Glyma.07G040300.1	100	196	4	322	2271	1953	1	0	2490

TRINITY_DN4433_c0_g1_i1	Glyma.08G139400.1	99.3006993	165	7	217	2169	1	1941	0	2604

TRINITY_DN4433_c0_g1_i1	Glyma.08G139400.4	99.2970123	165	8	217	2169	1	1938	0	2584

TRINITY_DN13058_c0_g1_i1	Glyma.01G145400.1	100	163	2	575	2528	3402	1455	0	2667

TRINITY_DN10472_c0_g1_i1	Glyma.11G051400.1	96.2962963	163	11	127	2063	13	1947	0	2473

TRINITY_DN12564_c0_g2_i1	Glyma.03G257800.1	98.40989399	151	2	580	2527	1954	1	0	2734

TRINITY_DN4433_c0_g1_i1	Glyma.05G181700.1	99.3006993	149	6	217	2169	1	1926	0	2595

TRINITY_DN4433_c0_g1_i1	Glyma.05G181700.2	99.3006993	149	7	217	2169	1	1923	0	2577

TRINITY_DN12564_c0_g2_i1	Glyma.19G256300.1	100	148	2	580	2527	1954	1	0	2750

TRINITY_DN10989_c0_g1_i1	Glyma.02G272700.1	100	138	0	357	2310	1954	1	0	2844

TRINITY_DN14726_c0_g2_i1	Glyma.14G070600.1	97.10144928	133	4	524	2472	2199	247	0	2833

TRINITY_DN16256_c1_g6_i3	Glyma.20G194100.1	98.63013699	112	5	543	2492	1941	1	0	2878

TRINITY_DN16345_c0_g1_i4	Glyma.07G093700.1	98.44357977	97	1	440	2393	3729	1779	0	3051

TRINITY_DN15820_c0_g2_i1	Glyma.14G003500.1	99.12280702	273	6	436	2381	1986	35	0	2043

TRINITY_DN15820_c0_g2_i4	Glyma.14G003500.1	100	273	6	436	2381	1986	35	0	2043

TRINITY_DN16345_c0_g1_i4	Glyma.08G044700.1	98.45360825	237	3	446	2394	3699	1748	0	2261

TRINITY_DN16345_c0_g1_i4	Glyma.08G044700.5	98.4496124	237	3	446	2394	3657	1706	0	2261

TRINITY_DN16345_c0_g1_i4	Glyma.08G044700.4	98.44559585	237	3	446	2394	3594	1643	0	2261

TRINITY_DN16345_c0_g1_i4	Glyma.08G044700.3	98.4375	237	3	446	2394	3504	1553	0	2261

TRINITY_DN16345_c0_g1_i4	Glyma.08G044700.6	98.4375	237	3	446	2394	3420	1469	0	2261

TRINITY_DN16345_c0_g1_i4	Glyma.08G044700.2	98.4496124	237	3	446	2394	3399	1448	0	2261

TRINITY_DN16345_c0_g1_i4	Glyma.08G044700.7	98.4375	237	3	446	2394	3225	1274	0	2261

TRINITY_DN16345_c0_g1_i4	Glyma.05G237500.1	98.4375	230	3	446	2394	3705	1754	0	2300

TRINITY_DN16385_c0_g1_i7	Glyma.16G056400.1	98.27586207	227	18	589	2506	3453	1506	0	2091

TRINITY_DN16309_c0_g1_i1	Glyma.01G208600.1	98.94736842	187	26	77	1999	1	1920	0	2163

TRINITY_DN16309_c0_g1_i3	Glyma.01G208600.1	100	187	26	77	1999	1	1920	0	2163

TRINITY_DN15159_c0_g1_i1	Glyma.18G051100.1	98.4375	186	9	292	2234	2004	56	0	2486

TRINITY_DN15159_c0_g1_i1	Glyma.18G051100.2	97.34513274	186	9	292	2234	2001	53	0	2486

TRINITY_DN15159_c0_g1_i1	Glyma.18G051100.6	100	186	9	292	2234	1989	41	0	2486

TRINITY_DN15159_c0_g1_i1	Glyma.18G051100.5	97.36842105	186	9	292	2234	1962	14	0	2486

TRINITY_DN15159_c0_g1_i1	Glyma.18G051100.3	100	186	9	292	2234	1962	14	0	2486

TRINITY_DN3700_c0_g1_i1	Glyma.06G118300.4	97.07602339	172	5	449	2397	1941	2	0	2543

TRINITY_DN3700_c0_g1_i1	Glyma.06G118300.3	99.26470588	172	5	449	2397	1941	2	0	2543

TRINITY_DN3700_c0_g1_i1	Glyma.06G118300.5	95.43147208	172	5	449	2397	1941	2	0	2543

TRINITY_DN3700_c0_g1_i1	Glyma.06G118300.1	95.43147208	172	5	449	2397	1941	2	0	2543

TRINITY_DN3700_c0_g1_i1	Glyma.06G118300.2	98.36065574	172	5	449	2397	1941	2	0	2543

TRINITY_DN11214_c0_g1_i1	Glyma.19G043200.1	98.9010989	144	0	1	1953	2200	248	0	2809

TRINITY_DN14726_c0_g2_i1	Glyma.17G254100.1	95.47038328	144	2	524	2472	2199	247	0	2784

TRINITY_DN15981_c0_g1_i12	Glyma.11G048300.2	99.6	125	3	1635	3581	2183	231	0	2876

TRINITY_DN15981_c0_g1_i12	Glyma.11G048300.1	99.5049505	125	3	1635	3581	2183	231	0	2876

TRINITY_DN15981_c0_g1_i2	Glyma.11G048300.2	98.11320755	125	3	1417	3363	2183	231	0	2876

TRINITY_DN15981_c0_g1_i2	Glyma.11G048300.1	95.71428571	125	3	1417	3363	2183	231	0	2876

TRINITY_DN15826_c0_g1_i10	Glyma.06G088500.8	97.2972973	125	4	162	2108	1	1953	0	2876

TRINITY_DN15826_c0_g1_i10	Glyma.06G088500.9	100	125	4	162	2108	1	1953	0	2876

TRINITY_DN15826_c0_g1_i2	Glyma.06G088500.8	99.10714286	125	4	162	2108	1	1953	0	2876

TRINITY_DN15826_c0_g1_i2	Glyma.06G088500.9	96.31578947	125	4	162	2108	1	1953	0	2876

TRINITY_DN15826_c0_g1_i6	Glyma.06G088500.8	98.64864865	125	4	162	2108	1	1953	0	2876

TRINITY_DN15826_c0_g1_i6	Glyma.06G088500.9	98.73417722	125	4	162	2108	1	1953	0	2876

TRINITY_DN15826_c0_g1_i9	Glyma.06G088500.8	97.22222222	125	4	162	2108	1	1953	0	2876

TRINITY_DN15826_c0_g1_i9	Glyma.06G088500.9	97.22222222	125	4	162	2108	1	1953	0	2876

TRINITY_DN12523_c0_g1_i1	Glyma.10G283400.1	99.01380671	110	3	193	2141	4	1955	0	2964

TRINITY_DN16432_c1_g2_i7	Glyma.20G204400.1	97.09193246	317	18	284	2216	54	1998	0	1681

TRINITY_DN13544_c0_g1_i1	Glyma.01G062400.1	98.06451613	287	23	127	2068	2019	84	0	1847

TRINITY_DN14314_c0_g1_i1	Glyma.08G246000.1	97.01492537	282	33	241	2181	1	1896	0	1609

TRINITY_DN15820_c0_g2_i1	Glyma.02G309400.1	98.80239521	268	4	436	2381	1986	35	0	2082

TRINITY_DN15820_c0_g2_i4	Glyma.02G309400.1	100	268	4	436	2381	1986	35	0	2082

TRINITY_DN16309_c0_g1_i1	Glyma.11G033600.2	98.95833333	206	21	77	1999	1	1938	0	2185

TRINITY_DN16309_c0_g1_i1	Glyma.11G033600.1	98.95013123	206	21	77	1999	1	1938	0	2185

TRINITY_DN16309_c0_g1_i1	Glyma.11G033600.3	98.94736842	206	21	77	1999	1	1938	0	2185

TRINITY_DN16309_c0_g1_i1	Glyma.11G033600.4	98.95833333	206	21	77	1999	1	1938	0	2185

TRINITY_DN16309_c0_g1_i1	Glyma.11G033600.5	98.20359281	206	21	77	1999	1	1938	0	2185

TRINITY_DN16309_c0_g1_i1	Glyma.11G033600.7	98.2078853	206	21	77	1999	1	1938	0	2185

TRINITY_DN16309_c0_g1_i1	Glyma.11G033600.6	98.1981982	206	21	77	1999	1	1938	0	2185

TRINITY_DN16309_c0_g1_i3	Glyma.11G033600.5	97.5	206	21	77	1999	1	1938	0	2185

TRINITY_DN16309_c0_g1_i3	Glyma.11G033600.7	97.5	206	21	77	1999	1	1938	0	2185

TRINITY_DN16309_c0_g1_i3	Glyma.11G033600.6	100	206	21	77	1999	1	1938	0	2185

TRINITY_DN16309_c0_g1_i3	Glyma.11G033600.2	100	206	21	77	1999	1	1938	0	2185

TRINITY_DN16309_c0_g1_i3	Glyma.11G033600.1	100	206	21	77	1999	1	1938	0	2185

TRINITY_DN16309_c0_g1_i3	Glyma.11G033600.3	100	206	21	77	1999	1	1938	0	2185

TRINITY_DN16309_c0_g1_i3	Glyma.11G033600.4	100	206	21	77	1999	1	1938	0	2185

TRINITY_DN35741_c0_g1_i1	Glyma.08G014700.1	100	174	7	281	2224	1	1949	0	2569

TRINITY_DN35741_c0_g1_i1	Glyma.08G014700.3	100	174	7	281	2224	1	1949	0	2569

TRINITY_DN35741_c0_g1_i1	Glyma.08G014700.4	100	174	7	281	2224	1	1949	0	2569

TRINITY_DN15549_c0_g1_i7	Glyma.05G247000.2	96.86520376	149	12	885	2806	2766	821	0	2547

TRINITY_DN16245_c0_g3_i4	Glyma.04G166100.2	100	122	0	1926	3877	1952	1	0	2929

TRINITY_DN16245_c0_g3_i5	Glyma.04G166100.2	97.87234043	122	0	1240	3191	1952	1	0	2929

TRINITY_DN16245_c0_g3_i3	Glyma.04G144900.2	95.43325527	428	34	405	2345	2748	835	0	928

TRINITY_DN16245_c0_g3_i3	Glyma.04G144900.3	100	428	34	405	2345	2748	835	0	928

TRINITY_DN16245_c0_g3_i6	Glyma.04G144900.2	99.23076923	428	34	1091	3031	2748	835	0	928

TRINITY_DN16245_c0_g3_i6	Glyma.04G144900.3	100	428	34	1091	3031	2748	835	0	928

TRINITY_DN16432_c1_g2_i6	Glyma.20G204400.1	96	323	16	111	2043	54	1998	0	1659

TRINITY_DN18910_c0_g1_i1	Glyma.16G019000.1	95.55555556	262	15	312	2246	1	1944	0	2002

TRINITY_DN15706_c0_g1_i3	Glyma.08G074700.2	97.63313609	246	7	559	2505	1	1947	0	2189

TRINITY_DN15549_c0_g1_i4	Glyma.05G247000.3	100	149	12	922	2842	2765	821	0	2545

TRINITY_DN15841_c0_g1_i1	Glyma.U033300.2	95.64393939	138	4	569	2519	273	2194	0	2651

TRINITY_DN15841_c0_g1_i2	Glyma.U033300.2	98	138	4	569	2519	273	2194	0	2651

TRINITY_DN37437_c0_g1_i1	Glyma.05G192700.1	96.10182975	109	6	176	2108	3	1950	0	2863

TRINITY_DN15981_c0_g1_i12	Glyma.17G161300.1	96.6442953	331	6	1635	3581	2177	231	0	1729

TRINITY_DN15981_c0_g1_i2	Glyma.17G161300.1	98.6013986	331	6	1417	3363	2177	231	0	1729

TRINITY_DN15981_c0_g1_i12	Glyma.05G105200.1	95.74468085	324	5	1635	3581	2177	231	0	1768

TRINITY_DN15981_c0_g1_i2	Glyma.05G105200.1	96	324	5	1417	3363	2177	231	0	1768

TRINITY_DN14726_c0_g3_i1	Glyma.14G070600.1	95.08196721	245	11	368	2312	2188	247	0	2159

TRINITY_DN15718_c0_g1_i7	Glyma.05G186900.1	97.64705882	237	2	100	2048	1949	1	0	2276

TRINITY_DN16151_c0_g2_i6	Glyma.08G293000.1	100	218	32	34	1964	43	1943	0	1954

TRINITY_DN16151_c0_g2_i6	Glyma.08G293000.2	98.61111111	216	34	34	1964	43	1925	0	1849

TRINITY_DN15529_c1_g1_i5	Glyma.07G089000.6	100	210	0	20	1969	1950	1	0	2438

TRINITY_DN15529_c1_g1_i5	Glyma.07G089000.5	100	210	0	20	1969	1950	1	0	2438

TRINITY_DN4217_c0_g1_i1	Glyma.07G135400.1	99.5951417	194	2	1	1947	58	2004	0	2488

TRINITY_DN4217_c0_g1_i1	Glyma.18G185400.1	98.79518072	188	2	1	1947	58	2004	0	2521

TRINITY_DN11956_c0_g1_i1	Glyma.17G004800.1	97.70114943	175	4	364	2306	2121	173	0	2580

TRINITY_DN15369_c0_g1_i1	Glyma.18G289100.1	100	167	0	313	2262	1950	1	0	2676

TRINITY_DN15529_c1_g1_i2	Glyma.07G089000.6	99.65156794	154	0	20	1969	1950	1	0	2748

TRINITY_DN15529_c1_g1_i2	Glyma.07G089000.5	99.65156794	154	0	20	1969	1950	1	0	2748

TRINITY_DN15529_c1_g1_i4	Glyma.07G089000.6	98.95287958	154	0	20	1969	1950	1	0	2748

TRINITY_DN15529_c1_g1_i4	Glyma.07G089000.5	98.95287958	154	0	20	1969	1950	1	0	2748

TRINITY_DN3322_c0_g1_i1	Glyma.06G171700.3	100	146	6	507	2444	1950	1	0	2715

TRINITY_DN3322_c0_g2_i1	Glyma.06G171700.3	96.66666667	146	6	507	2444	1950	1	0	2715

TRINITY_DN16298_c0_g1_i1	Glyma.12G098900.3	100	143	3	1188	3135	2064	117	0	2784

TRINITY_DN12702_c0_g1_i1	Glyma.04G033200.1	99.5	108	0	252	2201	1950	1	0	3003

TRINITY_DN12702_c0_g1_i1	Glyma.06G033200.1	95.95959596	104	0	252	2201	1950	1	0	3025

TRINITY_DN4747_c0_g1_i1	Glyma.07G034500.2	97.10144928	312	39	99	2027	2082	175	0	1476

TRINITY_DN4747_c0_g1_i1	Glyma.07G034500.1	97.22222222	312	39	99	2027	2082	175	0	1476

TRINITY_DN8924_c0_g1_i1	Glyma.01G227500.2	100	275	23	2	1927	703	2643	0	1875

TRINITY_DN15706_c0_g1_i5	Glyma.08G074700.2	100	217	4	559	2505	1	1947	0	2372

TRINITY_DN13050_c0_g1_i2	Glyma.08G178400.2	95.45454545	185	22	285	2206	2445	512	0	2303

TRINITY_DN13050_c0_g1_i2	Glyma.08G178400.1	97.42268041	185	22	285	2206	2445	512	0	2303

TRINITY_DN35741_c0_g1_i1	Glyma.05G208000.2	100	174	11	281	2224	1	1928	0	2468

TRINITY_DN35741_c0_g1_i1	Glyma.05G208000.4	100	174	11	281	2224	1	1928	0	2468

TRINITY_DN35741_c0_g1_i1	Glyma.05G208000.1	98.66666667	174	11	281	2224	1	1928	0	2468

TRINITY_DN35741_c0_g1_i1	Glyma.05G208000.3	96.55172414	174	11	281	2224	1	1928	0	2468

TRINITY_DN15718_c0_g1_i7	Glyma.04G217300.2	98.828125	152	0	100	2048	1949	1	0	2758

TRINITY_DN15718_c0_g1_i7	Glyma.04G217300.3	100	152	0	100	2048	1949	1	0	2758

TRINITY_DN15718_c0_g1_i7	Glyma.04G217300.1	99.71264368	152	0	100	2048	1949	1	0	2758

TRINITY_DN15718_c0_g1_i7	Glyma.06G148500.1	98.82629108	150	0	100	2048	1949	1	0	2769

TRINITY_DN8977_c0_g1_i1	Glyma.06G094000.4	98.36065574	147	25	39	1953	476	2390	0	2346

TRINITY_DN12226_c0_g2_i1	Glyma.19G033700.1	98.93617021	136	8	513	2454	1944	1	0	2769

TRINITY_DN16432_c1_g2_i6	Glyma.10G185800.1	100	333	19	111	2043	54	1992	0	1598

TRINITY_DN16230_c0_g1_i2	Glyma.17G233900.3	95.9752322	218	22	80	1968	1930	1	0	1892

TRINITY_DN40856_c0_g1_i1	Glyma.13G114200.2	100	157	4	34	1977	2853	907	0	2695

TRINITY_DN40856_c0_g1_i1	Glyma.13G114200.1	99.46236559	157	4	34	1977	2853	907	0	2695

TRINITY_DN40856_c0_g1_i1	Glyma.13G114200.3	100	157	4	34	1977	2472	526	0	2695

TRINITY_DN9085_c0_g1_i2	Glyma.01G125800.2	95.91836735	155	8	187	2124	27	1967	0	2628

TRINITY_DN1445_c0_g1_i1	Glyma.10G124300.2	100	149	0	539	2486	1948	1	0	2772

TRINITY_DN1445_c0_g1_i1	Glyma.10G124300.1	99.34640523	149	0	539	2486	1948	1	0	2772

TRINITY_DN12161_c0_g1_i1	Glyma.05G007600.2	98.4	143	5	276	2219	1944	1	0	2754

TRINITY_DN12161_c0_g1_i1	Glyma.05G007600.1	98.4	143	5	276	2219	1944	1	0	2754

TRINITY_DN1445_c0_g1_i1	Glyma.20G076500.2	99.49494949	133	0	539	2486	1948	1	0	2861

TRINITY_DN1445_c0_g1_i1	Glyma.20G076500.1	99.68701095	133	0	539	2486	1948	1	0	2861

TRINITY_DN10212_c0_g2_i1	Glyma.09G083800.1	98.53372434	128	2	519	2465	1947	1	0	2876

TRINITY_DN12319_c0_g1_i3	Glyma.19G224000.3	98.80952381	127	11	155	2082	215	2157	0	2732

TRINITY_DN12319_c0_g1_i3	Glyma.19G224000.1	98.80952381	127	11	155	2082	215	2157	0	2732

TRINITY_DN12319_c0_g1_i3	Glyma.19G224000.2	98.81305638	127	11	155	2082	215	2157	0	2732

TRINITY_DN13088_c0_g1_i2	Glyma.20G156500.1	96.77419355	119	3	534	2475	4	1951	0	2900

TRINITY_DN11827_c0_g1_i3	Glyma.07G009800.1	99.34210526	114	1	151	2098	595	2539	0	2946

TRINITY_DN11827_c0_g1_i3	Glyma.07G009800.2	99.31972789	114	1	151	2098	595	2539	0	2946

TRINITY_DN6860_c0_g1_i1	Glyma.02G267800.1	99.25373134	110	0	483	2430	2008	61	0	2988

TRINITY_DN16432_c1_g2_i7	Glyma.10G185800.1	97.52066116	332	17	284	2216	54	1992	0	1615

TRINITY_DN16432_c1_g2_i5	Glyma.20G204400.1	95.1536643	272	9	284	2216	54	1998	0	1986

TRINITY_DN16230_c0_g1_i5	Glyma.17G233900.3	99.21875	217	19	80	1968	1930	1	0	1908

TRINITY_DN16082_c0_g1_i4	Glyma.08G202000.5	100	198	5	552	2475	2128	184	0	2337

TRINITY_DN14726_c0_g3_i1	Glyma.04G028600.1	100	171	3	368	2314	2191	251	0	2610

TRINITY_DN11692_c0_g1_i2	Glyma.13G349600.2	96.3190184	147	8	34	1978	21	1956	0	2697

TRINITY_DN12724_c0_g1_i1	Glyma.05G241500.2	99.47916667	114	2	176	2122	1	1941	0	2926

TRINITY_DN12724_c0_g1_i1	Glyma.05G241500.1	100	114	2	176	2122	1	1941	0	2926

TRINITY_DN9431_c0_g1_i1	Glyma.05G223700.3	95.53571429	108	2	1	1947	340	2282	0	2972

TRINITY_DN12724_c0_g1_i1	Glyma.08G048800.1	97.05882353	108	2	176	2122	1	1941	0	2959

TRINITY_DN13713_c0_g1_i4	Glyma.U033400.3	95.38950715	78	2	296	2242	1	1944	0	3144

TRINITY_DN13713_c0_g1_i4	Glyma.U033400.7	97.91666667	78	2	296	2242	1	1944	0	3144

TRINITY_DN16432_c1_g2_i7	Glyma.20G204400.2	99.1416309	319	15	284	2216	54	1992	0	1698

TRINITY_DN16432_c1_g2_i11	Glyma.10G185800.1	95.2020202	279	15	111	2043	54	1992	0	1919

TRINITY_DN16432_c1_g2_i11	Glyma.20G204400.1	95.91141397	277	7	111	2043	54	1998	0	1969

TRINITY_DN16432_c1_g2_i5	Glyma.10G185800.1	95.15418502	276	13	284	2216	54	1992	0	1936

TRINITY_DN16298_c0_g1_i2	Glyma.12G098900.3	100	222	3	1240	3183	2060	117	0	2338

TRINITY_DN2519_c0_g1_i1	Glyma.16G066300.1	98.63013699	192	2	200	2145	485	2427	0	2510

TRINITY_DN13842_c0_g1_i2	Glyma.08G127700.4	100	181	11	1	1923	373	2316	0	2429

TRINITY_DN13842_c0_g1_i2	Glyma.08G127700.3	99.15966387	181	11	1	1923	373	2316	0	2429

TRINITY_DN13842_c0_g1_i2	Glyma.08G127700.1	98.7854251	181	11	1	1923	373	2316	0	2429

TRINITY_DN13842_c0_g1_i2	Glyma.08G127700.5	95.62043796	181	11	1	1923	367	2310	0	2429

TRINITY_DN13842_c0_g1_i2	Glyma.08G127700.2	98.41269841	181	11	1	1923	367	2310	0	2429

TRINITY_DN9085_c0_g1_i2	Glyma.03G047100.2	95.92391304	178	9	187	2124	27	1967	0	2523

TRINITY_DN9601_c0_g2_i1	Glyma.03G009500.1	100	175	15	325	2257	1944	18	0	2418

TRINITY_DN9601_c0_g2_i1	Glyma.03G009500.2	100	173	16	325	2257	1929	18	0	2331

TRINITY_DN6551_c0_g1_i1	Glyma.09G104000.3	98.82352941	153	4	371	2313	2609	4536	0	2610

TRINITY_DN5646_c0_g1_i1	Glyma.19G135300.1	100	153	11	969	2907	1780	3694	0	2501

TRINITY_DN5646_c0_g1_i2	Glyma.19G135300.1	95.45454545	153	11	969	2907	1780	3694	0	2501

TRINITY_DN6551_c0_g1_i1	Glyma.09G104000.2	98.82352941	151	4	371	2313	2609	4524	0	2543

TRINITY_DN13288_c0_g1_i1	Glyma.19G196800.1	100	142	16	259	2177	5	1931	0	2510

TRINITY_DN12226_c0_g2_i1	Glyma.19G033700.2	98.93617021	136	8	516	2454	1941	1	0	2763

TRINITY_DN12319_c0_g1_i3	Glyma.03G227100.1	98.80952381	130	15	155	2082	215	2157	0	2737

TRINITY_DN15981_c0_g1_i12	Glyma.01G193600.2	100	124	1	1635	3577	2177	232	0	2887

TRINITY_DN15981_c0_g1_i12	Glyma.01G193600.1	100	124	1	1635	3577	2177	232	0	2887

TRINITY_DN15981_c0_g1_i2	Glyma.01G193600.2	100	124	1	1417	3359	2177	232	0	2887

TRINITY_DN15981_c0_g1_i2	Glyma.01G193600.1	98.85057471	124	1	1417	3359	2177	232	0	2887

TRINITY_DN14779_c0_g1_i1	Glyma.17G246600.2	98.84393064	80	4	745	2680	2150	206	0	3079

TRINITY_DN14779_c0_g1_i4	Glyma.17G246600.2	98.27586207	80	4	644	2579	2150	206	0	3079

TRINITY_DN14005_c0_g1_i1	Glyma.11G125500.1	100	349	28	154	2079	61	1990	0	1439

TRINITY_DN16432_c1_g2_i6	Glyma.20G204400.2	98.7755102	325	13	111	2043	54	1992	0	1676

TRINITY_DN14151_c0_g1_i1	Glyma.09G253700.3	98.07692308	324	24	632	2547	1	1923	0	1467

TRINITY_DN16432_c1_g2_i1	Glyma.20G204400.1	96.069869	241	5	284	2216	54	1998	0	2180

TRINITY_DN13842_c0_g1_i2	Glyma.08G186400.3	98.98989899	221	10	1	1932	370	2310	0	2257

TRINITY_DN13842_c0_g1_i2	Glyma.08G186400.2	96.7032967	221	10	1	1932	370	2310	0	2257

TRINITY_DN13842_c0_g1_i2	Glyma.08G186400.1	99.23076923	221	10	1	1932	370	2310	0	2257

TRINITY_DN16230_c0_g1_i2	Glyma.17G233900.4	97.60081674	218	21	80	1968	1927	1	0	1906

TRINITY_DN13713_c0_g1_i2	Glyma.15G042700.7	96.61016949	217	10	326	2254	19	1953	0	2222

TRINITY_DN43422_c0_g1_i1	Glyma.03G151200.1	100	204	1	86	2027	2601	657	0	2442

TRINITY_DN8957_c0_g1_i4	Glyma.05G024500.2	98.4375	202	17	100	2027	2123	211	0	2156

TRINITY_DN8957_c0_g1_i4	Glyma.05G024500.5	98.46153846	202	17	100	2027	2123	211	0	2156

TRINITY_DN8957_c0_g1_i4	Glyma.05G024500.3	98.46153846	202	17	100	2027	2123	211	0	2156

TRINITY_DN8957_c0_g1_i4	Glyma.05G024500.4	98.45201238	202	17	100	2027	2123	211	0	2156

TRINITY_DN8957_c0_g1_i4	Glyma.05G024500.1	98.4375	202	17	100	2027	2123	211	0	2156

TRINITY_DN14850_c0_g1_i1	Glyma.05G056300.1	97.57575758	190	13	35	1963	1	1938	0	2390

TRINITY_DN7470_c0_g1_i2	Glyma.06G318500.1	97.5	189	8	71	2006	1	1939	0	2447

TRINITY_DN14850_c0_g1_i1	Glyma.17G138500.1	95.59322034	188	14	35	1963	1	1938	0	2401

TRINITY_DN13842_c0_g1_i2	Glyma.05G169400.3	100	185	10	1	1923	367	2307	0	2399

TRINITY_DN13842_c0_g1_i2	Glyma.05G169400.1	100	185	10	1	1923	367	2307	0	2399

TRINITY_DN13842_c0_g1_i2	Glyma.05G169400.2	98.98989899	185	10	1	1923	367	2307	0	2399

TRINITY_DN16082_c0_g1_i14	Glyma.08G202000.5	100	181	2	581	2504	2128	184	0	2453

TRINITY_DN16382_c0_g1_i1	Glyma.09G050500.1	95.34883721	170	8	408	2351	1	1929	0	2540

TRINITY_DN16382_c0_g1_i10	Glyma.09G050500.1	100	170	8	408	2351	1	1929	0	2540

TRINITY_DN16382_c0_g1_i3	Glyma.09G050500.1	97.59036145	170	8	313	2256	1	1929	0	2540

TRINITY_DN16382_c0_g1_i6	Glyma.09G050500.1	98.91304348	170	8	313	2256	1	1929	0	2540

TRINITY_DN36582_c0_g1_i1	Glyma.06G180700.1	98.73417722	167	1	415	2356	1945	1	0	2647

TRINITY_DN10472_c0_g1_i1	Glyma.11G051400.2	96.2962963	164	9	127	2063	13	1938	0	2508

TRINITY_DN13288_c0_g1_i1	Glyma.03G199500.1	95.71984436	144	16	266	2177	18	1946	0	2477

TRINITY_DN12226_c0_g2_i3	Glyma.19G033700.2	98.93617021	137	7	660	2597	1941	1	0	2761

TRINITY_DN14314_c0_g1_i2	Glyma.08G246000.1	95.45454545	294	23	241	2169	1	1896	0	1554

TRINITY_DN9547_c0_g1_i1	Glyma.09G199500.1	98.7654321	218	13	21	1949	1	1938	0	2246

TRINITY_DN16230_c0_g1_i5	Glyma.17G233900.4	99.21875	217	18	80	1968	1927	1	0	1923

TRINITY_DN8957_c0_g1_i3	Glyma.05G024500.2	98.46153846	181	12	100	2036	2123	211	0	2342

TRINITY_DN8957_c0_g1_i3	Glyma.05G024500.5	98.46153846	181	12	100	2036	2123	211	0	2342

TRINITY_DN8957_c0_g1_i3	Glyma.05G024500.3	98.45360825	181	12	100	2036	2123	211	0	2342

TRINITY_DN8957_c0_g1_i3	Glyma.05G024500.4	98.4375	181	12	100	2036	2123	211	0	2342

TRINITY_DN8957_c0_g1_i3	Glyma.05G024500.1	98.4375	181	12	100	2036	2123	211	0	2342

TRINITY_DN11081_c0_g2_i1	Glyma.05G021400.1	98.68421053	162	2	169	2112	29	1969	0	2673

TRINITY_DN12758_c0_g1_i1	Glyma.15G011400.1	95.59322034	161	5	388	2325	1940	6	0	2601

TRINITY_DN5991_c0_g2_i1	Glyma.06G174400.1	98.61111111	154	5	68	1996	1	1941	0	2621

TRINITY_DN13640_c1_g2_i1	Glyma.09G255400.2	96.55172414	148	13	441	2335	2931	995	0	2409

TRINITY_DN13640_c1_g2_i2	Glyma.09G255400.2	98.64864865	148	13	441	2335	2931	995	0	2409

TRINITY_DN13640_c1_g2_i3	Glyma.09G255400.2	97.98387097	148	13	441	2335	2931	995	0	2409

TRINITY_DN13640_c1_g2_i4	Glyma.09G255400.2	95.23809524	148	13	441	2335	2931	995	0	2409

TRINITY_DN13640_c1_g2_i5	Glyma.09G255400.2	100	148	13	441	2335	2931	995	0	2409

TRINITY_DN13640_c1_g2_i6	Glyma.09G255400.2	95.45454545	148	13	441	2335	2931	995	0	2409

TRINITY_DN13640_c1_g2_i7	Glyma.09G255400.2	97.46835443	148	13	441	2335	2931	995	0	2409

TRINITY_DN5991_c0_g2_i1	Glyma.04G191400.1	98.61386139	148	13	68	1996	1	1941	0	2654

TRINITY_DN10664_c0_g1_i1	Glyma.18G212600.1	100	143	3	170	2109	1	1943	0	2765

TRINITY_DN13413_c0_g1_i1	Glyma.06G075300.1	96.0502693	138	7	305	2245	1932	1	0	2728

TRINITY_DN10664_c0_g1_i1	Glyma.03G131800.1	95.56650246	136	3	170	2109	1	1943	0	2804

TRINITY_DN15826_c0_g1_i10	Glyma.04G086800.1	99.37106918	122	4	162	2096	1	1944	0	2856

TRINITY_DN15826_c0_g1_i2	Glyma.04G086800.1	95.23809524	122	4	162	2096	1	1944	0	2856

TRINITY_DN15826_c0_g1_i6	Glyma.04G086800.1	98.36065574	122	4	162	2096	1	1944	0	2856

TRINITY_DN15826_c0_g1_i9	Glyma.04G086800.1	99.59266802	122	4	162	2096	1	1944	0	2856

TRINITY_DN16043_c0_g2_i1	Glyma.08G293900.1	100	116	3	56	1998	1748	3684	0	2896

TRINITY_DN13526_c0_g1_i1	Glyma.01G214700.2	97.61904762	115	14	157	2087	49	1964	0	2687

TRINITY_DN38228_c0_g1_i1	Glyma.16G018400.9	98.24561404	111	3	1	1943	1653	3592	0	2942

TRINITY_DN38228_c0_g1_i1	Glyma.16G018400.1	100	111	3	1	1943	1653	3592	0	2942

TRINITY_DN38228_c0_g1_i1	Glyma.16G018400.7	98.24561404	111	3	1	1943	318	2257	0	2942

TRINITY_DN38228_c0_g1_i1	Glyma.16G018400.6	98.27586207	111	3	1	1943	267	2206	0	2942

TRINITY_DN38228_c0_g1_i1	Glyma.16G018400.8	99	110	5	1	1943	1653	3577	0	2852

TRINITY_DN38228_c0_g1_i1	Glyma.16G018400.5	100	110	5	1	1943	267	2191	0	2852

TRINITY_DN16043_c0_g2_i1	Glyma.18G129200.1	100	102	3	56	1998	896	2832	0	2974

TRINITY_DN15539_c0_g1_i3	Glyma.11G079200.1	98.21428571	98	0	140	2083	1	1944	0	3048

TRINITY_DN15539_c0_g1_i3	Glyma.01G164100.1	97.58241758	96	0	140	2083	1	1944	0	3059

TRINITY_DN16245_c0_g3_i1	Glyma.16G099700.3	98.18181818	173	1	192	2134	2778	840	0	2604

TRINITY_DN15925_c0_g1_i2	Glyma.16G054200.1	99.05660377	146	1	292	2234	89	2025	0	2741

TRINITY_DN16245_c0_g3_i3	Glyma.16G099700.3	97.4	120	1	404	2346	2778	840	0	2898

TRINITY_DN16245_c0_g3_i6	Glyma.16G099700.3	100	120	1	1090	3032	2778	840	0	2898

TRINITY_DN38228_c0_g1_i1	Glyma.07G049700.1	95.74468085	109	0	1	1943	1656	3598	0	2985

TRINITY_DN13116_c0_g1_i1	Glyma.13G109600.9	97.86324786	292	10	436	2370	1932	1	0	1858

TRINITY_DN13116_c0_g1_i2	Glyma.13G109600.9	97.86476868	292	10	198	2132	1932	1	0	1858

TRINITY_DN8781_c0_g1_i1	Glyma.05G170700.2	95.34883721	273	6	533	2471	2485	547	0	2036

TRINITY_DN13713_c0_g1_i2	Glyma.15G042700.6	100	217	9	326	2254	19	1950	0	2235

TRINITY_DN36582_c0_g1_i1	Glyma.06G180700.2	98.73417722	167	0	415	2356	1942	1	0	2662

TRINITY_DN14245_c0_g2_i3	Glyma.13G181700.2	95.65217391	140	7	446	2384	94	2020	0	2695

TRINITY_DN16414_c0_g1_i11	Glyma.01G225200.1	98.77300613	137	2	382	2322	1941	1	0	2815

TRINITY_DN16414_c0_g1_i3	Glyma.01G225200.1	96.23655914	136	2	190	2130	1941	1	0	2820

TRINITY_DN16414_c0_g1_i4	Glyma.01G225200.1	96.25	136	2	190	2130	1941	1	0	2820

TRINITY_DN16414_c0_g1_i10	Glyma.01G225200.1	97.87234043	130	2	382	2322	1941	1	0	2854

TRINITY_DN13413_c0_g1_i1	Glyma.04G074200.1	96.0502693	129	4	305	2245	1932	1	0	2800

TRINITY_DN15235_c0_g1_i1	Glyma.16G172600.1	96.2962963	360	20	34	1964	448	2377	0	1454

TRINITY_DN16352_c0_g2_i3	Glyma.08G194700.2	100	359	36	2340	4258	1961	40	0	1330

TRINITY_DN13097_c0_g1_i2	Glyma.08G319100.1	97.56097561	319	29	194	2093	2100	180	0	1423

TRINITY_DN13097_c0_g1_i3	Glyma.08G319100.1	95.50561798	319	29	194	2093	2100	180	0	1423

TRINITY_DN14473_c0_g2_i1	Glyma.10G038000.1	98.66444073	282	8	211	2145	133	2070	0	1964

TRINITY_DN16432_c1_g2_i5	Glyma.20G204400.2	100	273	7	284	2216	54	1992	0	2008

TRINITY_DN13714_c0_g1_i2	Glyma.06G112900.1	98.16513761	270	26	2	1918	157	2085	0	1857

TRINITY_DN18757_c0_g1_i1	Glyma.04G018000.3	97.05882353	193	13	680	2610	2043	122	0	2327

TRINITY_DN18757_c0_g1_i1	Glyma.04G018000.2	97.05882353	193	13	680	2610	2043	122	0	2327

TRINITY_DN15075_c0_g1_i1	Glyma.02G123700.1	100	182	16	489	2399	1932	1	0	2324

TRINITY_DN15075_c0_g1_i2	Glyma.02G123700.1	98.61111111	182	16	674	2584	1932	1	0	2324

TRINITY_DN15075_c0_g1_i3	Glyma.02G123700.1	100	182	16	489	2399	1932	1	0	2324

TRINITY_DN16237_c0_g1_i3	Glyma.08G008100.1	96.34146341	164	12	923	2848	549	2477	0	2501

TRINITY_DN16237_c0_g1_i3	Glyma.05G200800.4	98.21428571	163	11	923	2848	567	2498	0	2527

TRINITY_DN16237_c0_g1_i3	Glyma.05G200800.1	95.60878244	163	11	923	2848	567	2498	0	2527

TRINITY_DN12758_c0_g1_i1	Glyma.13G362400.1	100	158	2	387	2324	1941	10	0	2632

TRINITY_DN15925_c0_g1_i2	Glyma.19G094800.1	99.45054945	144	1	294	2234	91	2025	0	2748

TRINITY_DN11887_c0_g2_i7	Glyma.18G057100.1	99.81981982	139	5	138	2063	1	1941	0	2717

TRINITY_DN11677_c0_g1_i1	Glyma.06G015600.1	96.18320611	138	2	163	2097	1	1941	0	2782

TRINITY_DN11887_c0_g2_i7	Glyma.11G163900.1	99.82269504	137	5	138	2063	1	1941	0	2728

TRINITY_DN15826_c0_g1_i10	Glyma.06G088500.6	96.36363636	125	4	162	2096	1	1941	0	2854

TRINITY_DN15826_c0_g1_i10	Glyma.06G088500.7	98.67109635	125	4	162	2096	1	1941	0	2854

TRINITY_DN15826_c0_g1_i10	Glyma.06G088500.3	96.20253165	125	4	162	2096	1	1941	0	2854

TRINITY_DN15826_c0_g1_i10	Glyma.06G088500.4	96.20253165	125	4	162	2096	1	1941	0	2854

TRINITY_DN15826_c0_g1_i10	Glyma.06G088500.5	98.26589595	125	4	162	2096	1	1941	0	2854

TRINITY_DN15826_c0_g1_i10	Glyma.06G088500.2	95.79831933	125	4	162	2096	1	1941	0	2854

TRINITY_DN15826_c0_g1_i10	Glyma.06G088500.1	98.01980198	125	4	162	2096	1	1941	0	2854

TRINITY_DN15826_c0_g1_i2	Glyma.06G088500.6	97.77777778	125	4	162	2096	1	1941	0	2854

TRINITY_DN15826_c0_g1_i2	Glyma.06G088500.7	98.6440678	125	4	162	2096	1	1941	0	2854

TRINITY_DN15826_c0_g1_i2	Glyma.06G088500.3	99.06542056	125	4	162	2096	1	1941	0	2854

TRINITY_DN15826_c0_g1_i2	Glyma.06G088500.4	98.05825243	125	4	162	2096	1	1941	0	2854

TRINITY_DN15826_c0_g1_i2	Glyma.06G088500.5	100	125	4	162	2096	1	1941	0	2854

TRINITY_DN15826_c0_g1_i2	Glyma.06G088500.2	99.0521327	125	4	162	2096	1	1941	0	2854

TRINITY_DN15826_c0_g1_i2	Glyma.06G088500.1	96.11111111	125	4	162	2096	1	1941	0	2854

TRINITY_DN15826_c0_g1_i3	Glyma.06G088500.8	100	125	4	162	2096	1	1941	0	2854

TRINITY_DN15826_c0_g1_i3	Glyma.06G088500.9	96.83544304	125	4	162	2096	1	1941	0	2854

TRINITY_DN15826_c0_g1_i6	Glyma.06G088500.6	97.95918367	125	4	162	2096	1	1941	0	2854

TRINITY_DN15826_c0_g1_i6	Glyma.06G088500.7	98.98989899	125	4	162	2096	1	1941	0	2854

TRINITY_DN15826_c0_g1_i6	Glyma.06G088500.3	98.98477157	125	4	162	2096	1	1941	0	2854

TRINITY_DN15826_c0_g1_i6	Glyma.06G088500.4	100	125	4	162	2096	1	1941	0	2854

TRINITY_DN15826_c0_g1_i6	Glyma.06G088500.5	97.98657718	125	4	162	2096	1	1941	0	2854

TRINITY_DN15826_c0_g1_i6	Glyma.06G088500.2	100	125	4	162	2096	1	1941	0	2854

TRINITY_DN15826_c0_g1_i6	Glyma.06G088500.1	100	125	4	162	2096	1	1941	0	2854

TRINITY_DN15826_c0_g1_i9	Glyma.06G088500.6	98.54014599	125	4	162	2096	1	1941	0	2854

TRINITY_DN15826_c0_g1_i9	Glyma.06G088500.7	98.13664596	125	4	162	2096	1	1941	0	2854

TRINITY_DN15826_c0_g1_i9	Glyma.06G088500.3	96.2962963	125	4	162	2096	1	1941	0	2854

TRINITY_DN15826_c0_g1_i9	Glyma.06G088500.4	98.86792453	125	4	162	2096	1	1941	0	2854

TRINITY_DN15826_c0_g1_i9	Glyma.06G088500.5	100	125	4	162	2096	1	1941	0	2854

TRINITY_DN15826_c0_g1_i9	Glyma.06G088500.2	96.49122807	125	4	162	2096	1	1941	0	2854

TRINITY_DN15826_c0_g1_i9	Glyma.06G088500.1	100	125	4	162	2096	1	1941	0	2854

TRINITY_DN6157_c0_g1_i1	Glyma.10G154700.1	97.82608696	123	0	374	2314	1941	1	0	2904

TRINITY_DN6157_c0_g1_i1	Glyma.20G233700.2	100	109	0	374	2314	1941	1	0	2981

TRINITY_DN6157_c0_g1_i1	Glyma.20G233700.1	97.11538462	109	0	374	2314	1941	1	0	2981

TRINITY_DN16086_c0_g3_i2	Glyma.15G162600.1	96.1038961	357	9	1	1935	934	2868	0	1541

TRINITY_DN14021_c0_g2_i1	Glyma.05G214900.1	98.14814815	296	7	907	2842	2518	582	0	1897

TRINITY_DN16432_c1_g2_i11	Glyma.20G204400.2	97.85714286	278	5	111	2043	54	1992	0	1991

TRINITY_DN16016_c0_g1_i7	Glyma.16G163200.1	100	273	19	708	2642	3231	1312	0	1908

TRINITY_DN16016_c0_g1_i8	Glyma.16G163200.1	96.49122807	273	19	708	2642	3231	1312	0	1908

TRINITY_DN12673_c0_g4_i1	Glyma.03G003900.1	98.87640449	273	21	86	2010	8	1932	0	1877

TRINITY_DN16432_c1_g2_i1	Glyma.10G185800.1	100	250	5	284	2216	54	1992	0	2146

TRINITY_DN13714_c0_g1_i3	Glyma.06G112900.1	96.25	248	24	2	1918	157	2085	0	1989

TRINITY_DN43422_c0_g1_i1	Glyma.19G153700.1	98	176	1	86	2022	2598	659	0	2588

TRINITY_DN8854_c0_g1_i2	Glyma.04G191600.1	99.34640523	126	7	116	2046	1935	5	0	2769

TRINITY_DN8854_c0_g1_i2	Glyma.04G191600.4	95.78947368	126	7	116	2046	1935	5	0	2769

TRINITY_DN8854_c0_g1_i2	Glyma.04G191600.3	95.78947368	126	7	116	2046	1935	5	0	2769

TRINITY_DN15812_c0_g1_i2	Glyma.10G242800.2	95	454	22	600	2526	2793	867	0	911

TRINITY_DN15812_c0_g1_i2	Glyma.10G242800.1	96.66666667	454	22	600	2526	2793	867	0	911

TRINITY_DN15812_c0_g1_i2	Glyma.10G242800.3	95.60067682	454	22	600	2526	2793	867	0	911

TRINITY_DN15812_c0_g1_i7	Glyma.10G242800.2	97.6744186	454	22	683	2609	2793	867	0	911

TRINITY_DN15812_c0_g1_i7	Glyma.10G242800.1	100	454	22	683	2609	2793	867	0	911

TRINITY_DN15812_c0_g1_i7	Glyma.10G242800.3	100	454	22	683	2609	2793	867	0	911

TRINITY_DN15812_c0_g1_i3	Glyma.10G242800.2	95.83333333	451	20	683	2609	2793	867	0	928

TRINITY_DN15812_c0_g1_i3	Glyma.10G242800.1	97.56097561	451	20	683	2609	2793	867	0	928

TRINITY_DN15812_c0_g1_i3	Glyma.10G242800.3	98.48484848	451	20	683	2609	2793	867	0	928

TRINITY_DN15812_c0_g1_i8	Glyma.10G242800.2	97.14285714	451	20	600	2526	2793	867	0	928

TRINITY_DN15812_c0_g1_i8	Glyma.10G242800.1	97.14285714	451	20	600	2526	2793	867	0	928

TRINITY_DN15812_c0_g1_i8	Glyma.10G242800.3	97.14285714	451	20	600	2526	2793	867	0	928

TRINITY_DN16086_c0_g3_i2	Glyma.09G056300.1	100	365	7	1	1935	1120	3054	0	1507

TRINITY_DN16086_c0_g3_i2	Glyma.09G056300.2	100	365	7	1	1935	934	2868	0	1507

TRINITY_DN14151_c0_g1_i1	Glyma.09G253700.2	97.77777778	325	21	632	2547	1	1917	0	1489

TRINITY_DN16432_c1_g2_i1	Glyma.20G204400.2	95.16616314	242	3	284	2216	54	1992	0	2202

TRINITY_DN1827_c0_g1_i1	Glyma.17G122300.1	100	230	22	183	2082	137	2073	0	2041

TRINITY_DN1827_c0_g1_i1	Glyma.17G122300.2	100	230	22	183	2082	137	2073	0	2041

TRINITY_DN12878_c0_g1_i1	Glyma.11G231700.2	100	174	24	33	1950	1	1915	0	2326

TRINITY_DN12878_c0_g1_i3	Glyma.11G231700.2	100	174	24	33	1950	1	1915	0	2326

TRINITY_DN11746_c0_g1_i1	Glyma.17G030700.1	100	173	4	427	2364	1935	1	0	2590

TRINITY_DN15028_c0_g1_i3	Glyma.04G203600.3	97.12230216	173	6	1304	3239	1933	1	0	2564

TRINITY_DN15028_c0_g1_i3	Glyma.04G203600.2	98.1981982	173	6	1304	3239	1933	1	0	2564

TRINITY_DN6009_c0_g1_i1	Glyma.16G059500.1	97.96954315	170	2	309	2244	3132	1197	0	2601

TRINITY_DN6009_c0_g1_i1	Glyma.16G059500.2	97.97979798	170	2	309	2244	3132	1197	0	2601

TRINITY_DN16303_c0_g1_i2	Glyma.12G201100.5	98.79518072	116	5	152	2090	1	1912	0	2763

TRINITY_DN16303_c0_g1_i3	Glyma.12G201100.5	100	116	5	152	2090	1	1912	0	2763

TRINITY_DN16303_c0_g1_i4	Glyma.12G201100.5	100	116	5	152	2090	1	1912	0	2763

TRINITY_DN16303_c0_g1_i5	Glyma.12G201100.5	100	116	5	152	2090	1	1912	0	2763

TRINITY_DN16307_c0_g1_i1	Glyma.06G116200.1	95.16129032	297	30	292	2198	2101	198	0	1513

TRINITY_DN12008_c0_g1_i1	Glyma.07G042500.1	99.51456311	201	4	174	2109	89	2024	0	2440

TRINITY_DN16082_c0_g1_i4	Glyma.08G202000.4	99.3993994	198	8	552	2475	2119	184	0	2379

TRINITY_DN16082_c0_g1_i14	Glyma.08G202000.4	96.83098592	177	8	581	2504	2119	184	0	2495

TRINITY_DN16206_c0_g1_i3	Glyma.20G130400.4	96.94280079	165	1	676	2613	1	1932	0	2627

TRINITY_DN16206_c0_g1_i3	Glyma.20G130400.3	99.31506849	165	1	676	2613	1	1932	0	2627

TRINITY_DN16206_c0_g1_i1	Glyma.20G130400.4	100	160	1	676	2613	1	1932	0	2654

TRINITY_DN16206_c0_g1_i1	Glyma.20G130400.3	96.03174603	160	1	676	2613	1	1932	0	2654

TRINITY_DN15736_c0_g1_i2	Glyma.04G203200.2	100	160	14	293	2216	173	2097	0	2518

TRINITY_DN15736_c0_g1_i3	Glyma.04G203200.2	98.75	160	14	293	2216	173	2097	0	2518

TRINITY_DN12243_c0_g2_i1	Glyma.08G188900.1	98.1595092	132	8	181	2118	1	1914	0	2693

TRINITY_DN2142_c0_g1_i1	Glyma.07G073500.5	98.71794872	131	12	213	2129	1	1935	0	2699

TRINITY_DN2142_c0_g1_i1	Glyma.07G073500.6	98.71794872	131	12	213	2129	1	1935	0	2699

TRINITY_DN2142_c0_g1_i1	Glyma.07G073500.7	98.7654321	131	12	213	2129	1	1935	0	2699

TRINITY_DN2142_c0_g1_i1	Glyma.07G073500.1	98.67256637	131	12	213	2129	1	1935	0	2699

TRINITY_DN2142_c0_g1_i1	Glyma.07G073500.4	98.67256637	131	12	213	2129	1	1935	0	2699

TRINITY_DN2142_c0_g1_i1	Glyma.07G073500.2	98.67549669	131	12	213	2129	1	1935	0	2699

TRINITY_DN2142_c0_g1_i1	Glyma.07G073500.3	98.72204473	131	12	213	2129	1	1935	0	2699

TRINITY_DN12243_c0_g2_i1	Glyma.12G168800.1	98.1595092	121	7	181	2118	1	1911	0	2734

TRINITY_DN7839_c0_g1_i1	Glyma.05G219500.4	100	112	0	398	2335	1	1938	0	2959

TRINITY_DN11768_c0_g1_i1	Glyma.08G253800.2	98.05825243	414	31	646	2566	3046	1126	0	1077

TRINITY_DN11768_c0_g1_i1	Glyma.08G253800.3	100	407	34	646	2566	3040	1126	0	1077

TRINITY_DN15235_c0_g1_i1	Glyma.16G172400.1	98.7804878	355	13	34	1964	517	2443	0	1507

TRINITY_DN15235_c0_g1_i1	Glyma.16G172400.4	96.91119691	355	13	34	1964	475	2401	0	1507

TRINITY_DN15235_c0_g1_i1	Glyma.16G172400.3	96.90721649	355	13	34	1964	448	2374	0	1507

TRINITY_DN5901_c0_g2_i1	Glyma.02G087000.5	99.42196532	270	15	1062	2996	1	1911	0	1901

TRINITY_DN5901_c0_g1_i1	Glyma.02G087000.5	100	243	15	1062	2996	1	1911	0	2050

TRINITY_DN16086_c0_g3_i2	Glyma.06G076100.1	100	212	4	1	1935	922	2856	0	2377

TRINITY_DN16086_c0_g3_i2	Glyma.04G075100.1	100	205	4	1	1935	922	2856	0	2416

TRINITY_DN2721_c0_g1_i1	Glyma.01G117800.1	100	173	4	417	2350	2252	322	0	2560

TRINITY_DN10472_c0_g1_i3	Glyma.11G051400.1	100	164	9	127	2046	13	1947	0	2545

TRINITY_DN13832_c0_g2_i1	Glyma.10G066000.1	100	147	11	1005	2931	1923	3	0	2595

TRINITY_DN9742_c0_g1_i1	Glyma.03G161300.7	100	143	15	1038	2967	2244	336	0	2558

TRINITY_DN8854_c0_g1_i2	Glyma.06G174300.1	96.79487179	141	7	116	2046	1932	5	0	2699

TRINITY_DN2628_c0_g1_i1	Glyma.06G100600.1	98.41269841	117	4	216	2149	6	1935	0	2865

TRINITY_DN9262_c0_g1_i2	Glyma.08G018400.1	100	106	3	1461	3396	2050	3984	0	2970

TRINITY_DN16951_c0_g1_i1	Glyma.03G139000.1	98	104	0	47	1983	11	1947	0	3001

TRINITY_DN16951_c0_g1_i1	Glyma.19G142100.1	98.01980198	93	0	47	1983	11	1947	0	3062

TRINITY_DN15812_c0_g1_i2	Glyma.20G151400.1	100	445	18	600	2525	2793	868	0	981

TRINITY_DN15812_c0_g1_i2	Glyma.20G151400.3	95	445	18	600	2525	2793	868	0	981

TRINITY_DN15812_c0_g1_i7	Glyma.20G151400.1	98.7804878	445	18	683	2608	2793	868	0	981

TRINITY_DN15812_c0_g1_i7	Glyma.20G151400.3	98.7804878	445	18	683	2608	2793	868	0	981

TRINITY_DN15812_c0_g1_i2	Glyma.20G151400.2	100	445	18	600	2525	2790	865	0	981

TRINITY_DN15812_c0_g1_i7	Glyma.20G151400.2	95.84295612	445	18	683	2608	2790	865	0	981

TRINITY_DN15812_c0_g1_i3	Glyma.20G151400.1	96.22641509	444	18	683	2608	2793	868	0	987

TRINITY_DN15812_c0_g1_i3	Glyma.20G151400.3	95.83333333	444	18	683	2608	2793	868	0	987

TRINITY_DN15812_c0_g1_i8	Glyma.20G151400.1	97.14285714	444	18	600	2525	2793	868	0	987

TRINITY_DN15812_c0_g1_i8	Glyma.20G151400.3	97.14285714	444	18	600	2525	2793	868	0	987

TRINITY_DN15812_c0_g1_i3	Glyma.20G151400.2	97.52066116	444	18	683	2608	2790	865	0	987

TRINITY_DN15812_c0_g1_i8	Glyma.20G151400.2	97.14285714	444	18	600	2525	2790	865	0	987

TRINITY_DN4747_c0_g1_i1	Glyma.08G207600.1	95.34883721	302	39	99	2006	2064	169	0	1463

TRINITY_DN16385_c0_g1_i7	Glyma.19G091300.1	98.26086957	211	14	589	2506	3423	1509	0	2154

TRINITY_DN15361_c1_g1_i1	Glyma.13G166200.1	100	208	10	189	2117	1	1920	0	2274

TRINITY_DN11169_c0_g1_i1	Glyma.20G139900.1	97.65625	201	19	318	2238	66	1980	0	2230

TRINITY_DN16089_c0_g1_i1	Glyma.14G199200.1	100	184	10	186	2096	1	1935	0	2388

TRINITY_DN9601_c0_g2_i1	Glyma.07G071000.1	97.58064516	173	7	330	2257	1948	18	0	2532

TRINITY_DN9601_c0_g2_i1	Glyma.07G071000.2	97.5862069	171	8	330	2257	1933	18	0	2447

TRINITY_DN12951_c0_g1_i1	Glyma.14G116800.1	95.4248366	157	4	894	2825	1935	1	0	2673

TRINITY_DN13832_c0_g2_i1	Glyma.13G151100.1	98.06451613	138	9	1005	2931	1923	3	0	2656

TRINITY_DN11214_c0_g1_i1	Glyma.13G048800.1	98.9010989	354	29	38	1953	2163	245	0	1386

TRINITY_DN14726_c0_g2_i1	Glyma.04G028600.1	100	282	15	551	2474	2174	251	0	1869

TRINITY_DN15361_c1_g1_i1	Glyma.17G113900.2	97.48858447	219	13	189	2117	1	1920	0	2224

TRINITY_DN15361_c1_g1_i1	Glyma.17G113900.1	95.3125	219	13	189	2117	1	1920	0	2224

TRINITY_DN9846_c0_g1_i1	Glyma.01G241000.3	100	211	7	112	2035	90	2010	0	2242

TRINITY_DN9846_c0_g1_i1	Glyma.01G241000.1	98.86363636	211	7	112	2035	90	2010	0	2242

TRINITY_DN5400_c0_g1_i1	Glyma.02G238200.1	97.88235294	146	0	190	2124	1935	1	0	2765

TRINITY_DN2134_c0_g1_i1	Glyma.16G036300.1	98.68421053	133	12	170	2080	1	1923	0	2604

TRINITY_DN15332_c0_g2_i1	Glyma.19G243900.4	100	130	2	578	2509	1929	1	0	2795

TRINITY_DN15332_c0_g2_i2	Glyma.19G243900.4	100	130	2	676	2607	1929	1	0	2795

TRINITY_DN15332_c0_g2_i3	Glyma.19G243900.4	96.42857143	130	2	439	2370	1929	1	0	2795

TRINITY_DN15332_c0_g2_i1	Glyma.03G246500.5	100	127	2	578	2509	1929	1	0	2811

TRINITY_DN15332_c0_g2_i2	Glyma.03G246500.5	98.93617021	127	2	676	2607	1929	1	0	2811

TRINITY_DN15332_c0_g2_i3	Glyma.03G246500.5	99.66442953	127	2	439	2370	1929	1	0	2811

TRINITY_DN5056_c0_g1_i1	Glyma.09G147400.1	95.55555556	127	2	1	1934	200	2133	0	2857

TRINITY_DN16086_c0_g3_i2	Glyma.17G199200.1	96.79420889	92	0	1	1935	922	2856	0	3064

TRINITY_DN16086_c0_g3_i2	Glyma.14G134100.1	97.54098361	86	0	1	1935	922	2856	0	3097

TRINITY_DN12617_c0_g1_i2	Glyma.13G104300.5	100	297	12	2303	4222	1362	3293	0	1823

TRINITY_DN12617_c0_g1_i2	Glyma.13G104300.13	100	297	12	2303	4222	1356	3287	0	1823

TRINITY_DN12617_c0_g1_i2	Glyma.13G104300.12	100	297	12	2303	4222	1338	3269	0	1823

TRINITY_DN13116_c0_g1_i1	Glyma.13G109600.20	97.87234043	285	10	301	2227	1950	27	0	1882

TRINITY_DN15736_c0_g1_i1	Glyma.08G005900.2	97.21115538	201	5	296	2226	265	2195	0	2420

TRINITY_DN15736_c0_g1_i1	Glyma.08G005900.3	97.43589744	201	5	296	2226	265	2195	0	2420

TRINITY_DN15736_c0_g1_i1	Glyma.08G005900.1	95.92760181	201	5	296	2226	265	2195	0	2420

TRINITY_DN15736_c0_g1_i4	Glyma.08G005900.2	96.57142857	201	5	296	2226	265	2195	0	2420

TRINITY_DN15736_c0_g1_i4	Glyma.08G005900.3	96.57142857	201	5	296	2226	265	2195	0	2420

TRINITY_DN15736_c0_g1_i4	Glyma.08G005900.1	99.58506224	201	5	296	2226	265	2195	0	2420

TRINITY_DN18757_c0_g1_i1	Glyma.06G018300.2	97.05882353	187	2	680	2610	2091	158	0	2516

TRINITY_DN18757_c0_g1_i1	Glyma.06G018300.1	97.05882353	187	2	680	2610	2091	158	0	2516

TRINITY_DN14821_c1_g1_i1	Glyma.06G314900.1	95.93495935	179	10	336	2256	2145	228	0	2392

TRINITY_DN14821_c1_g1_i1	Glyma.04G128800.1	97.41176471	177	14	336	2256	2136	219	0	2403

TRINITY_DN11956_c0_g1_i1	Glyma.07G268900.1	95.47169811	151	10	364	2292	2106	181	0	2651

TRINITY_DN15841_c0_g1_i5	Glyma.U033300.2	96.11650485	138	3	569	2502	273	2194	0	2730

TRINITY_DN15841_c0_g1_i6	Glyma.U033300.2	95.61403509	138	3	569	2502	273	2194	0	2730

TRINITY_DN8854_c0_g1_i2	Glyma.04G191600.2	96.28378378	131	11	116	2046	1914	5	0	2671

TRINITY_DN15332_c0_g2_i1	Glyma.19G243900.3	96.52173913	130	2	579	2509	1928	1	0	2793

TRINITY_DN15332_c0_g2_i2	Glyma.19G243900.3	100	130	2	677	2607	1928	1	0	2793

TRINITY_DN15332_c0_g2_i3	Glyma.19G243900.3	98.11320755	130	2	440	2370	1928	1	0	2793

TRINITY_DN15332_c0_g2_i1	Glyma.03G246500.3	98.88888889	127	2	579	2509	1928	1	0	2809

TRINITY_DN15332_c0_g2_i1	Glyma.03G246500.4	98.88888889	127	2	579	2509	1928	1	0	2809

TRINITY_DN15332_c0_g2_i2	Glyma.03G246500.3	100	127	2	677	2607	1928	1	0	2809

TRINITY_DN15332_c0_g2_i2	Glyma.03G246500.4	100	127	2	677	2607	1928	1	0	2809

TRINITY_DN15332_c0_g2_i3	Glyma.03G246500.3	98.13084112	127	2	440	2370	1928	1	0	2809

TRINITY_DN15332_c0_g2_i3	Glyma.03G246500.4	97.69230769	127	2	440	2370	1928	1	0	2809

TRINITY_DN14644_c0_g2_i1	Glyma.20G118200.1	100	311	29	366	2267	2007	95	0	1517

TRINITY_DN16230_c0_g1_i3	Glyma.17G233900.3	98.61111111	223	20	80	1955	1930	1	0	1947

TRINITY_DN18789_c0_g1_i1	Glyma.16G008900.1	100	201	5	343	2271	1932	1	0	2423

TRINITY_DN15820_c0_g2_i2	Glyma.20G059000.1	100	186	4	436	2366	1974	44	0	2514

TRINITY_DN15820_c0_g2_i2	Glyma.20G059000.2	97.05882353	186	4	436	2366	1974	44	0	2514

TRINITY_DN13353_c0_g1_i1	Glyma.19G241200.1	97.61904762	149	2	265	2196	145	2076	0	2732

TRINITY_DN13511_c0_g1_i2	Glyma.06G016500.1	96.21621622	140	2	11	1942	1932	1	0	2782

TRINITY_DN14980_c0_g1_i1	Glyma.15G193300.1	95.3125	134	4	339	2267	1	1932	0	2795

TRINITY_DN14980_c0_g1_i1	Glyma.09G085200.1	95.30516432	131	5	339	2267	1	1932	0	2811

TRINITY_DN13778_c1_g3_i3	Glyma.18G199000.1	96.62921348	369	39	199	2105	3304	1395	0	1219

TRINITY_DN6249_c0_g1_i1	Glyma.17G135000.1	100	329	29	1	1921	141	2046	0	1506

TRINITY_DN12617_c0_g1_i2	Glyma.13G104300.4	100	298	14	2303	4222	1362	3284	0	1781

TRINITY_DN12617_c0_g1_i2	Glyma.13G104300.11	100	298	14	2303	4222	1338	3260	0	1781

TRINITY_DN16230_c0_g1_i1	Glyma.17G233900.3	100	221	17	80	1955	1930	1	0	1969

TRINITY_DN15075_c0_g1_i1	Glyma.01G067000.1	97.70114943	180	10	489	2399	1920	1	0	2357

TRINITY_DN15075_c0_g1_i2	Glyma.01G067000.1	97.82608696	180	10	674	2584	1920	1	0	2357

TRINITY_DN15075_c0_g1_i3	Glyma.01G067000.1	100	180	10	489	2399	1920	1	0	2357

TRINITY_DN13714_c0_g1_i3	Glyma.13G079900.1	97.49103943	170	11	2	1918	163	2091	0	2510

TRINITY_DN11746_c0_g1_i1	Glyma.07G243200.1	96.82539683	166	5	436	2364	1929	1	0	2610

TRINITY_DN16318_c0_g1_i3	Glyma.15G133600.1	95.7537155	160	2	521	2446	1932	1	0	2643

TRINITY_DN16318_c0_g1_i6	Glyma.15G133600.1	95.74468085	160	2	521	2446	1932	1	0	2643

TRINITY_DN13511_c0_g1_i2	Glyma.04G016500.1	100	138	2	11	1942	1929	1	0	2784

TRINITY_DN13511_c0_g1_i2	Glyma.04G016500.2	100	138	2	11	1942	1929	1	0	2784

TRINITY_DN8088_c0_g1_i1	Glyma.16G000200.1	97.87234043	122	3	354	2276	1345	3276	0	2833

TRINITY_DN15303_c2_g1_i1	Glyma.15G173200.1	96.2962963	108	4	830	2760	2254	333	0	2898

TRINITY_DN15303_c2_g1_i1	Glyma.15G173200.2	100	108	4	830	2760	2254	333	0	2898

TRINITY_DN15303_c2_g1_i2	Glyma.15G173200.1	97.14285714	108	4	1061	2991	2254	333	0	2898

TRINITY_DN15303_c2_g1_i2	Glyma.15G173200.2	99.2481203	108	4	1061	2991	2254	333	0	2898

TRINITY_DN15303_c2_g1_i3	Glyma.15G173200.1	100	108	4	1155	3085	2254	333	0	2898

TRINITY_DN15303_c2_g1_i3	Glyma.15G173200.2	99.07407407	108	4	1155	3085	2254	333	0	2898

TRINITY_DN19869_c0_g1_i1	Glyma.17G148200.1	97.14285714	102	0	441	2372	5532	3601	0	3003

TRINITY_DN16054_c1_g1_i4	Glyma.10G168700.2	100	93	0	756	2687	2586	655	0	3053

TRINITY_DN16054_c1_g1_i4	Glyma.20G221000.2	100	86	0	756	2687	2586	655	0	3092

TRINITY_DN14821_c1_g2_i1	Glyma.08G227300.1	100	266	35	254	2142	2154	251	0	1646

TRINITY_DN15529_c1_g1_i3	Glyma.09G188000.3	100	263	12	599	2523	1922	1	0	2012

TRINITY_DN15529_c1_g1_i3	Glyma.09G188000.4	100	263	12	599	2523	1922	1	0	2012

TRINITY_DN15529_c1_g1_i3	Glyma.09G188000.1	96.42857143	263	12	599	2523	1922	1	0	2012

TRINITY_DN15529_c1_g1_i3	Glyma.09G188000.2	96.15384615	263	12	599	2523	1922	1	0	2012

TRINITY_DN15529_c1_g1_i3	Glyma.09G188000.5	98.52941176	263	12	599	2523	1922	1	0	2012

TRINITY_DN14410_c0_g2_i2	Glyma.17G119800.1	97.18309859	249	13	271	2195	32	1953	0	2089

TRINITY_DN15276_c0_g1_i4	Glyma.06G154800.2	95.72192513	239	12	887	2809	497	2421	0	2152

TRINITY_DN15276_c0_g1_i4	Glyma.06G154800.1	96.35036496	239	12	887	2809	497	2421	0	2152

TRINITY_DN10825_c0_g1_i1	Glyma.11G089700.1	95.04132231	220	29	797	2701	1957	58	0	1978

TRINITY_DN16230_c0_g1_i4	Glyma.17G233900.3	96	219	17	80	1952	1930	1	0	1971

TRINITY_DN14821_c1_g1_i1	Glyma.08G227300.1	98.61111111	217	24	336	2254	2154	251	0	2111

TRINITY_DN15529_c1_g1_i1	Glyma.09G188000.3	100	206	12	599	2523	1922	1	0	2327

TRINITY_DN15529_c1_g1_i1	Glyma.09G188000.4	99.54545455	206	12	599	2523	1922	1	0	2327

TRINITY_DN15529_c1_g1_i1	Glyma.09G188000.1	95.04761905	206	12	599	2523	1922	1	0	2327

TRINITY_DN15529_c1_g1_i1	Glyma.09G188000.2	99.15878023	206	12	599	2523	1922	1	0	2327

TRINITY_DN15529_c1_g1_i1	Glyma.09G188000.5	96.8	206	12	599	2523	1922	1	0	2327

TRINITY_DN13224_c1_g3_i1	Glyma.20G112800.2	100	184	9	1	1919	121	2048	0	2449

TRINITY_DN13224_c1_g3_i1	Glyma.20G112800.1	100	184	9	1	1919	121	2048	0	2449

TRINITY_DN13224_c1_g3_i1	Glyma.10G276900.1	100	173	9	1	1919	121	2045	0	2492

TRINITY_DN15954_c0_g1_i4	Glyma.16G038300.2	97.59036145	168	4	287	2215	1	1929	0	2610

TRINITY_DN15954_c0_g1_i8	Glyma.16G038300.2	98.88888889	168	4	147	2075	1	1929	0	2610

TRINITY_DN16256_c1_g6_i2	Glyma.10G196100.2	100	148	13	568	2480	1916	1	0	2532

TRINITY_DN16256_c1_g6_i2	Glyma.10G196100.1	100	147	16	568	2480	1907	1	0	2481

TRINITY_DN13371_c0_g1_i3	Glyma.13G089700.1	97.6744186	142	1	914	2841	1931	1	0	2760

TRINITY_DN13371_c0_g1_i3	Glyma.13G089700.2	97.6744186	142	1	914	2841	1931	1	0	2760

TRINITY_DN15954_c0_g1_i5	Glyma.16G038300.2	99.46524064	126	4	147	2075	1	1929	0	2843

TRINITY_DN15954_c0_g1_i9	Glyma.16G038300.2	100	126	4	287	2215	1	1929	0	2843

TRINITY_DN16274_c0_g2_i1	Glyma.12G189500.1	100	94	9	1	1913	1100	3024	0	2891

TRINITY_DN15812_c0_g1_i2	Glyma.02G002500.3	95.80838323	463	19	607	2526	2786	867	0	870

TRINITY_DN15812_c0_g1_i2	Glyma.02G002500.1	100	463	19	607	2526	2786	867	0	870

TRINITY_DN15812_c0_g1_i7	Glyma.02G002500.3	100	463	19	690	2609	2786	867	0	870

TRINITY_DN15812_c0_g1_i7	Glyma.02G002500.1	100	463	19	690	2609	2786	867	0	870

TRINITY_DN15812_c0_g1_i2	Glyma.02G002500.2	95.80838323	463	19	607	2526	2261	342	0	870

TRINITY_DN15812_c0_g1_i7	Glyma.02G002500.2	96.66666667	463	19	690	2609	2261	342	0	870

TRINITY_DN15276_c0_g1_i2	Glyma.06G154800.2	96.2962963	281	10	887	2809	497	2421	0	1930

TRINITY_DN15276_c0_g1_i2	Glyma.06G154800.1	96.2962963	281	10	887	2809	497	2421	0	1930

TRINITY_DN16230_c0_g1_i3	Glyma.17G233900.4	98.61111111	223	19	80	1955	1927	1	0	1960

TRINITY_DN11188_c0_g1_i1	Glyma.09G181400.1	96.73469388	218	13	337	2235	1929	1	0	2150

TRINITY_DN12874_c0_g1_i2	Glyma.06G210800.1	95.83333333	143	5	519	2427	1	1930	0	2636

TRINITY_DN12874_c0_g1_i3	Glyma.06G210800.1	95.83843329	143	5	519	2427	1	1930	0	2636

TRINITY_DN14821_c1_g2_i1	Glyma.06G314900.1	100	139	9	254	2144	2145	228	0	2464

TRINITY_DN14821_c1_g2_i1	Glyma.04G128800.1	100	137	12	254	2144	2136	219	0	2475

TRINITY_DN13758_c0_g1_i1	Glyma.14G219700.2	100	126	6	331	2242	1	1930	0	2750

TRINITY_DN15812_c0_g1_i3	Glyma.02G002500.3	100	458	17	690	2609	2786	867	0	909

TRINITY_DN15812_c0_g1_i3	Glyma.02G002500.1	100	458	17	690	2609	2786	867	0	909

TRINITY_DN15812_c0_g1_i8	Glyma.02G002500.3	97.14285714	458	17	607	2526	2786	867	0	909

TRINITY_DN15812_c0_g1_i8	Glyma.02G002500.1	97.14285714	458	17	607	2526	2786	867	0	909

TRINITY_DN15812_c0_g1_i3	Glyma.02G002500.2	100	458	17	690	2609	2261	342	0	909

TRINITY_DN15812_c0_g1_i8	Glyma.02G002500.2	97.14285714	458	17	607	2526	2261	342	0	909

TRINITY_DN15759_c0_g2_i2	Glyma.17G191300.1	100	310	23	541	2451	2017	101	0	1652

TRINITY_DN15759_c0_g2_i5	Glyma.17G191300.1	100	310	23	541	2451	2017	101	0	1652

TRINITY_DN16230_c0_g1_i1	Glyma.17G233900.4	98.24561404	221	16	80	1955	1927	1	0	1982

TRINITY_DN12082_c0_g1_i1	Glyma.05G089200.1	100	176	2	115	2043	1	1923	0	2549

TRINITY_DN12082_c0_g1_i2	Glyma.05G089200.1	100	176	2	281	2209	1	1923	0	2549

TRINITY_DN13714_c0_g1_i2	Glyma.13G079900.1	100	151	6	2	1918	163	2091	0	2649

TRINITY_DN38879_c0_g1_i1	Glyma.04G176400.1	98.57142857	141	5	258	2180	94	2019	0	2723

TRINITY_DN10724_c0_g1_i1	Glyma.15G152700.1	96.77419355	137	5	214	2136	1923	1	0	2726

TRINITY_DN8088_c0_g1_i1	Glyma.16G000200.2	97.88235294	123	1	354	2276	1345	3273	0	2843

TRINITY_DN8088_c0_g1_i1	Glyma.12G163700.1	97.89473684	122	1	354	2276	1345	3273	0	2848

TRINITY_DN10732_c0_g1_i1	Glyma.15G034000.1	100	110	1	643	2559	3639	1711	0	2876

TRINITY_DN35829_c0_g1_i1	Glyma.08G028400.5	100	109	0	435	2363	1929	1	0	2959

TRINITY_DN16256_c1_g6_i3	Glyma.10G196100.2	98.63013699	106	5	568	2492	1916	1	0	2865

TRINITY_DN16256_c1_g6_i3	Glyma.10G196100.1	98.63013699	105	7	568	2492	1907	1	0	2813

TRINITY_DN13713_c0_g1_i2	Glyma.U033400.3	96.09756098	78	2	326	2254	19	1944	0	3110

TRINITY_DN13713_c0_g1_i2	Glyma.U033400.7	100	78	2	326	2254	19	1944	0	3110

TRINITY_DN11214_c0_g1_i1	Glyma.08G245600.1	100	462	46	47	1953	1956	68	2.72E-174	614

TRINITY_DN16431_c0_g1_i4	Glyma.01G127700.1	99.21259843	357	34	3419	5296	1959	46	0	1170

TRINITY_DN16431_c0_g1_i5	Glyma.01G127700.1	99.2	357	34	3399	5276	1959	46	0	1170

TRINITY_DN16431_c0_g1_i6	Glyma.01G127700.1	98.82352941	357	34	3062	4939	1959	46	0	1170

TRINITY_DN16431_c0_g1_i7	Glyma.01G127700.1	98.7654321	357	34	3042	4919	1959	46	0	1170

TRINITY_DN12673_c0_g4_i1	Glyma.19G122100.1	98.88888889	270	16	86	2001	8	1923	0	1910

TRINITY_DN14821_c1_g2_i1	Glyma.08G227300.2	100	267	34	254	2142	2151	251	0	1655

TRINITY_DN9056_c0_g2_i1	Glyma.14G214400.1	100	221	16	321	2227	1964	52	0	2104

TRINITY_DN16230_c0_g1_i4	Glyma.17G233900.4	99.78540773	219	16	80	1952	1927	1	0	1986

TRINITY_DN14821_c1_g1_i1	Glyma.08G227300.2	100	218	23	336	2254	2151	251	0	2121

TRINITY_DN7561_c0_g2_i1	Glyma.01G022600.1	100	206	6	1	1916	2106	179	0	2342

TRINITY_DN10729_c0_g1_i1	Glyma.10G019000.2	97.22222222	182	9	286	2211	23	1936	0	2449

TRINITY_DN10729_c0_g1_i1	Glyma.10G019000.1	97.22222222	182	9	286	2211	23	1936	0	2449

TRINITY_DN10729_c0_g1_i2	Glyma.10G019000.2	97.22222222	182	9	274	2199	23	1936	0	2449

TRINITY_DN10729_c0_g1_i2	Glyma.10G019000.1	97.22222222	182	9	274	2199	23	1936	0	2449

TRINITY_DN39155_c0_g1_i1	Glyma.01G215700.2	97.05882353	167	3	1	1922	2506	579	0	2597

TRINITY_DN39155_c0_g1_i1	Glyma.01G215700.3	98.18688982	167	3	1	1922	2506	579	0	2597

TRINITY_DN39155_c0_g1_i1	Glyma.01G215700.1	95	167	3	1	1922	2506	579	0	2597

TRINITY_DN10472_c0_g1_i3	Glyma.11G051400.2	100	165	7	127	2046	13	1938	0	2582

TRINITY_DN16049_c1_g2_i2	Glyma.08G102600.1	98.95833333	157	12	1048	2961	1920	1	0	2549

TRINITY_DN39155_c0_g1_i1	Glyma.11G026700.2	97.05882353	156	2	1	1922	2506	579	0	2658

TRINITY_DN39155_c0_g1_i1	Glyma.11G026700.1	97.05882353	156	2	1	1922	2506	579	0	2658

TRINITY_DN39155_c0_g1_i1	Glyma.11G026700.3	97.03703704	156	2	1	1922	2275	348	0	2658

TRINITY_DN7895_c0_g2_i1	Glyma.16G082400.2	100	150	7	1	1918	245	2157	0	2567

TRINITY_DN14598_c0_g2_i1	Glyma.17G261600.7	95.36423841	148	11	1329	3247	1916	1	0	2604

TRINITY_DN14598_c0_g2_i1	Glyma.17G261600.6	98.4375	148	11	1329	3247	1916	1	0	2604

TRINITY_DN14598_c0_g2_i1	Glyma.17G261600.1	98.4375	148	11	1329	3247	1916	1	0	2604

TRINITY_DN14598_c0_g2_i1	Glyma.17G261600.4	96.42857143	148	11	1329	3247	1916	1	0	2604

TRINITY_DN14598_c0_g2_i1	Glyma.17G261600.5	100	147	12	1329	3247	1904	1	0	2532

TRINITY_DN14598_c0_g2_i1	Glyma.17G261600.2	100	147	12	1329	3247	1904	1	0	2532

TRINITY_DN14598_c0_g2_i1	Glyma.17G261600.3	100	147	12	1329	3247	1904	1	0	2532

TRINITY_DN13371_c0_g1_i3	Glyma.14G174500.2	100	130	0	914	2841	1928	1	0	2841

TRINITY_DN13371_c0_g1_i3	Glyma.14G174500.1	95.32554257	130	0	914	2841	1928	1	0	2841

TRINITY_DN14740_c0_g1_i3	Glyma.02G248400.1	98.99497487	355	21	226	2138	1526	3432	0	1373

TRINITY_DN14740_c0_g1_i3	Glyma.02G248400.2	98.99497487	355	21	226	2138	1526	3432	0	1373

TRINITY_DN14740_c0_g1_i4	Glyma.02G248400.1	96.875	355	21	226	2138	1526	3432	0	1373

TRINITY_DN14740_c0_g1_i4	Glyma.02G248400.2	96.93593315	355	21	226	2138	1526	3432	0	1373

TRINITY_DN14740_c0_g1_i5	Glyma.02G248400.1	99.05660377	355	21	226	2138	1526	3432	0	1373

TRINITY_DN14740_c0_g1_i5	Glyma.02G248400.2	99.21259843	355	21	226	2138	1526	3432	0	1373

TRINITY_DN15235_c0_g1_i1	Glyma.10G227300.5	98.10246679	294	0	34	1960	454	2380	0	1930

TRINITY_DN15235_c0_g1_i1	Glyma.10G227300.4	98.57142857	294	0	34	1960	454	2380	0	1930

TRINITY_DN15235_c0_g1_i1	Glyma.10G227300.3	95.12195122	294	0	34	1960	454	2380	0	1930

TRINITY_DN15235_c0_g1_i1	Glyma.10G227300.1	100	294	0	34	1960	454	2380	0	1930

TRINITY_DN15235_c0_g1_i1	Glyma.10G227300.2	96.19047619	294	0	34	1960	454	2380	0	1930

TRINITY_DN13448_c0_g1_i2	Glyma.11G246200.4	99.00990099	268	0	128	2054	1	1927	0	2074

TRINITY_DN13448_c0_g1_i2	Glyma.11G246200.5	96.42857143	268	0	128	2054	1	1927	0	2074

TRINITY_DN395_c0_g1_i1	Glyma.05G041300.1	95.12195122	227	5	355	2281	1981	64	0	2242

TRINITY_DN14740_c0_g1_i3	Glyma.08G269000.3	96.66666667	212	4	246	2168	1	1926	0	2351

TRINITY_DN14740_c0_g1_i3	Glyma.08G269000.7	96.72131148	212	4	246	2168	1	1926	0	2351

TRINITY_DN14740_c0_g1_i3	Glyma.08G269000.6	99.03846154	212	4	246	2168	1	1926	0	2351

TRINITY_DN14740_c0_g1_i3	Glyma.08G269000.2	99.03846154	212	4	246	2168	1	1926	0	2351

TRINITY_DN14740_c0_g1_i3	Glyma.08G269000.4	99.02912621	212	4	246	2168	1	1926	0	2351

TRINITY_DN14740_c0_g1_i3	Glyma.08G269000.1	98.98989899	212	4	246	2168	1	1926	0	2351

TRINITY_DN14740_c0_g1_i3	Glyma.08G269000.5	98.99280576	212	4	246	2168	1	1926	0	2351

TRINITY_DN14740_c0_g1_i4	Glyma.08G269000.3	99.03846154	212	4	246	2168	1	1926	0	2351

TRINITY_DN14740_c0_g1_i4	Glyma.08G269000.7	99.03846154	212	4	246	2168	1	1926	0	2351

TRINITY_DN14740_c0_g1_i4	Glyma.08G269000.6	99.03846154	212	4	246	2168	1	1926	0	2351

TRINITY_DN14740_c0_g1_i4	Glyma.08G269000.2	99	212	4	246	2168	1	1926	0	2351

TRINITY_DN14740_c0_g1_i4	Glyma.08G269000.4	99	212	4	246	2168	1	1926	0	2351

TRINITY_DN14740_c0_g1_i4	Glyma.08G269000.1	96.90721649	212	4	246	2168	1	1926	0	2351

TRINITY_DN14740_c0_g1_i4	Glyma.08G269000.5	96.94656489	212	4	246	2168	1	1926	0	2351

TRINITY_DN14740_c0_g1_i5	Glyma.08G269000.3	96.875	212	4	246	2168	1	1926	0	2351

TRINITY_DN14740_c0_g1_i5	Glyma.08G269000.7	96.875	212	4	246	2168	1	1926	0	2351

TRINITY_DN14740_c0_g1_i5	Glyma.08G269000.6	96.875	212	4	246	2168	1	1926	0	2351

TRINITY_DN14740_c0_g1_i5	Glyma.08G269000.2	96.875	212	4	246	2168	1	1926	0	2351

TRINITY_DN14740_c0_g1_i5	Glyma.08G269000.4	96.93877551	212	4	246	2168	1	1926	0	2351

TRINITY_DN14740_c0_g1_i5	Glyma.08G269000.1	96.92307692	212	4	246	2168	1	1926	0	2351

TRINITY_DN14740_c0_g1_i5	Glyma.08G269000.5	96.9348659	212	4	246	2168	1	1926	0	2351

TRINITY_DN14913_c0_g2_i1	Glyma.05G117500.2	97.93814433	210	9	757	2674	6	1929	0	2318

TRINITY_DN14297_c0_g1_i1	Glyma.20G164300.1	98.64864865	158	8	348	2260	1926	1	0	2586

TRINITY_DN14297_c0_g1_i1	Glyma.10G228700.1	98.65470852	156	11	348	2256	1932	8	0	2566

TRINITY_DN16027_c1_g3_i1	Glyma.15G027500.1	99.2481203	153	6	502	2392	1927	1	0	2479

TRINITY_DN16027_c1_g3_i4	Glyma.15G027500.1	99.34640523	153	6	502	2392	1927	1	0	2479

TRINITY_DN13900_c0_g1_i1	Glyma.03G262100.1	100	90	0	3341	5267	3918	5844	0	3061

TRINITY_DN13900_c0_g1_i1	Glyma.03G262100.5	98.11827957	90	0	3341	5267	3918	5844	0	3061

TRINITY_DN13900_c0_g1_i1	Glyma.03G262100.6	98.5472155	90	0	3341	5267	3918	5844	0	3061

TRINITY_DN13900_c0_g1_i1	Glyma.03G262100.4	96.55172414	90	0	3341	5267	3192	5118	0	3061

TRINITY_DN13900_c0_g1_i1	Glyma.03G262100.2	98.91304348	90	0	3341	5267	2811	4737	0	3061

TRINITY_DN13900_c0_g1_i1	Glyma.03G262100.3	98.91304348	90	0	3341	5267	2811	4737	0	3061

TRINITY_DN13900_c0_g1_i1	Glyma.19G261200.1	95.41284404	86	0	3341	5267	3912	5838	0	3083

TRINITY_DN13900_c0_g1_i1	Glyma.19G261200.4	98.55072464	86	0	3341	5267	3912	5838	0	3083

TRINITY_DN13900_c0_g1_i1	Glyma.19G261200.2	97.75280899	86	0	3341	5267	3912	5838	0	3083

TRINITY_DN13900_c0_g1_i1	Glyma.19G261200.3	100	86	0	3341	5267	3912	5838	0	3083

TRINITY_DN15403_c0_g2_i4	Glyma.09G153500.4	96.66666667	269	18	839	2744	19	1928	0	1834

TRINITY_DN15403_c0_g2_i4	Glyma.09G153500.1	97.79411765	269	18	839	2744	19	1928	0	1834

TRINITY_DN16082_c0_g1_i4	Glyma.07G016700.2	97.42857143	201	3	552	2475	2101	178	0	2418

TRINITY_DN16082_c0_g1_i4	Glyma.08G202000.2	95.35714286	199	3	552	2475	2107	184	0	2429

TRINITY_DN16274_c0_g2_i1	Glyma.12G095300.1	96.11111111	193	8	1	1913	1103	3018	0	2338

TRINITY_DN16274_c0_g2_i1	Glyma.U010500.1	96.20689655	188	9	1	1913	1127	3045	0	2386

TRINITY_DN12786_c0_g1_i1	Glyma.07G272700.1	95.09803922	164	0	145	2070	92	2017	0	2649

TRINITY_DN15619_c0_g2_i3	Glyma.17G088800.3	99.00990099	139	4	951	2867	3	1927	0	2723

TRINITY_DN14830_c0_g1_i1	Glyma.19G004600.2	95.74468085	134	0	337	2262	1	1926	0	2815

TRINITY_DN1309_c0_g1_i1	Glyma.13G026700.2	100	129	13	11	1905	1	1917	0	2584

TRINITY_DN1309_c0_g1_i1	Glyma.13G026700.1	95.85798817	129	13	11	1905	1	1917	0	2584

TRINITY_DN14677_c0_g1_i5	Glyma.07G252600.4	97.64705882	116	3	1135	3060	2052	130	0	2894

TRINITY_DN6075_c0_g1_i1	Glyma.08G266300.4	98.0952381	116	4	249	2165	1926	1	0	2856

TRINITY_DN15790_c3_g4_i1	Glyma.04G065500.1	98.97260274	97	1	1817	3739	2938	4863	0	3000

TRINITY_DN15790_c3_g4_i3	Glyma.04G065500.1	99.08536585	97	1	1915	3837	2938	4863	0	3000

TRINITY_DN15565_c0_g1_i3	Glyma.09G044300.3	95.66395664	225	16	726	2638	9	1912	0	2095

TRINITY_DN15565_c0_g1_i4	Glyma.09G044300.3	96.66666667	225	16	739	2651	9	1912	0	2095

TRINITY_DN11188_c0_g1_i1	Glyma.07G096100.6	96.92307692	209	11	337	2259	1917	6	0	2300

TRINITY_DN11188_c0_g1_i1	Glyma.07G096100.1	100	209	11	337	2259	1917	6	0	2300

TRINITY_DN7561_c0_g2_i1	Glyma.09G199700.1	100	201	7	2	1914	2105	181	0	2364

TRINITY_DN7561_c0_g2_i1	Glyma.09G199700.4	100	201	7	2	1914	2105	181	0	2364

TRINITY_DN7561_c0_g2_i1	Glyma.09G199700.5	97.91666667	201	7	2	1914	2000	76	0	2364

TRINITY_DN11318_c0_g1_i1	Glyma.01G222500.1	97.89473684	191	12	1283	3162	1952	31	0	2187

TRINITY_DN5343_c0_g1_i1	Glyma.02G099800.1	99.23076923	190	14	224	2134	1	1911	0	2322

TRINITY_DN14740_c0_g1_i3	Glyma.11G219200.1	96.66666667	146	4	246	2168	1	1923	0	2721

TRINITY_DN14740_c0_g1_i4	Glyma.11G219200.1	98.98989899	146	4	246	2168	1	1923	0	2721

TRINITY_DN14740_c0_g1_i5	Glyma.11G219200.1	96.94656489	146	4	246	2168	1	1923	0	2721

TRINITY_DN14740_c0_g1_i3	Glyma.18G038300.1	96.66666667	139	16	246	2168	1	1896	0	2584

TRINITY_DN14740_c0_g1_i4	Glyma.18G038300.1	99.02912621	139	16	246	2168	1	1896	0	2584

TRINITY_DN14740_c0_g1_i5	Glyma.18G038300.1	96.89265537	139	16	246	2168	1	1896	0	2584

TRINITY_DN10502_c0_g2_i1	Glyma.17G038100.4	98.86363636	133	10	326	2241	1	1916	0	2702

TRINITY_DN10502_c0_g2_i1	Glyma.17G038100.6	98.87640449	133	10	326	2241	1	1916	0	2702

TRINITY_DN14913_c0_g1_i1	Glyma.05G117500.2	95.91836735	132	5	757	2674	6	1929	0	2772

TRINITY_DN14578_c0_g1_i2	Glyma.12G001200.2	97.89473684	104	4	154	2073	2721	799	0	2933

TRINITY_DN6136_c0_g1_i1	Glyma.10G041300.3	99.28057554	296	34	258	2162	1893	1	0	1591

TRINITY_DN6136_c0_g2_i1	Glyma.10G041300.3	100	296	34	257	2161	1893	1	0	1591

TRINITY_DN15565_c0_g1_i3	Glyma.09G044300.2	97.08737864	225	16	727	2638	40	1942	0	2093

TRINITY_DN15565_c0_g1_i4	Glyma.09G044300.2	97.85407725	225	16	740	2651	40	1942	0	2093

TRINITY_DN16396_c1_g1_i1	Glyma.15G037100.1	98.34254144	219	25	221	2126	185	2067	0	1958

TRINITY_DN16396_c1_g1_i2	Glyma.15G037100.1	98.33679834	219	25	221	2126	185	2067	0	1958

TRINITY_DN16089_c0_g1_i1	Glyma.02G232100.1	100	198	5	186	2096	1	1923	0	2366

TRINITY_DN16082_c0_g1_i14	Glyma.07G016700.2	98.4962406	183	0	581	2504	2101	178	0	2540

TRINITY_DN16082_c0_g1_i14	Glyma.08G202000.2	100	182	0	581	2504	2107	184	0	2545

TRINITY_DN16049_c1_g2_i2	Glyma.05G145800.1	98.11715481	166	5	1048	2961	1923	1	0	2562

TRINITY_DN16049_c1_g2_i2	Glyma.05G145800.2	100	166	5	1048	2961	1923	1	0	2562

TRINITY_DN6110_c0_g1_i1	Glyma.01G014600.6	99.09638554	159	18	681	2588	1915	2	0	2505

TRINITY_DN277_c0_g1_i1	Glyma.07G249800.4	95.19230769	132	1	375	2292	3486	1563	0	2784

TRINITY_DN277_c0_g1_i1	Glyma.07G249800.5	99.82300885	132	1	375	2292	3486	1563	0	2784

TRINITY_DN14578_c0_g1_i2	Glyma.08G238800.2	96.07843137	107	3	154	2073	2721	799	0	2928

TRINITY_DN14740_c0_g1_i3	Glyma.14G068100.2	98.99497487	357	13	226	2138	1529	3435	0	1406

TRINITY_DN14740_c0_g1_i3	Glyma.14G068100.1	98.99497487	357	13	226	2138	1529	3435	0	1406

TRINITY_DN14740_c0_g1_i4	Glyma.14G068100.2	96.94656489	357	13	226	2138	1529	3435	0	1406

TRINITY_DN14740_c0_g1_i4	Glyma.14G068100.1	96.875	357	13	226	2138	1529	3435	0	1406

TRINITY_DN14740_c0_g1_i5	Glyma.14G068100.2	99.20634921	357	13	226	2138	1529	3435	0	1406

TRINITY_DN14740_c0_g1_i5	Glyma.14G068100.1	99.04761905	357	13	226	2138	1529	3435	0	1406

TRINITY_DN15759_c0_g2_i2	Glyma.17G191300.2	97.10144928	309	24	541	2451	2005	101	0	1646

TRINITY_DN15759_c0_g2_i5	Glyma.17G191300.2	100	309	24	541	2451	2005	101	0	1646

TRINITY_DN36871_c0_g1_i1	Glyma.08G037600.1	97.71573604	165	8	2	1918	274	2178	0	2483

TRINITY_DN15521_c0_g1_i5	Glyma.06G226700.2	97.86324786	159	5	672	2589	428	2348	0	2625

TRINITY_DN16368_c0_g2_i2	Glyma.10G028900.1	100	157	2	5226	7147	1974	53	0	2669

TRINITY_DN10472_c0_g1_i1	Glyma.01G190800.1	96.2962963	151	11	151	2063	37	1938	0	2514

TRINITY_DN11438_c0_g2_i2	Glyma.19G231200.1	100	141	2	234	2155	1926	5	0	2758

TRINITY_DN2134_c0_g2_i1	Glyma.16G036300.1	97.84946237	135	8	170	2068	1	1923	0	2649

TRINITY_DN38193_c0_g1_i1	Glyma.08G227500.9	100	134	2	1	1920	243	2164	0	2784

TRINITY_DN38193_c0_g1_i1	Glyma.08G227500.10	100	134	2	1	1920	243	2164	0	2784

TRINITY_DN5787_c0_g1_i1	Glyma.17G029600.1	98.10606061	133	5	328	2241	1914	1	0	2699

TRINITY_DN2134_c0_g1_i1	Glyma.19G116400.1	98.68421053	133	8	170	2080	1	1911	0	2660

TRINITY_DN11833_c0_g1_i4	Glyma.20G241400.1	99.72375691	129	2	177	2096	1	1899	0	2662

TRINITY_DN16191_c1_g2_i1	Glyma.08G193400.1	99.34640523	105	0	95	2017	1	1923	0	2970

TRINITY_DN16191_c1_g2_i1	Glyma.08G193400.2	98.38709677	105	0	95	2017	1	1923	0	2970

TRINITY_DN16191_c1_g2_i1	Glyma.07G010300.1	97.98657718	99	0	95	2017	1	1923	0	3003

TRINITY_DN13317_c0_g1_i1	Glyma.01G136100.1	95.41284404	86	0	514	2436	1923	1	0	3075

TRINITY_DN11214_c0_g1_i1	Glyma.18G267100.1	96.2962963	447	36	47	1954	1965	76	0	776

TRINITY_DN11214_c0_g1_i1	Glyma.08G246300.1	100	446	36	47	1954	1959	70	0	782

TRINITY_DN15510_c0_g1_i5	Glyma.09G168400.2	98.8372093	307	19	137	2031	6	1921	0	1635

TRINITY_DN15159_c0_g1_i1	Glyma.02G237000.3	98.88888889	305	23	317	2222	1925	19	0	1659

TRINITY_DN15159_c0_g1_i1	Glyma.02G237000.1	100	305	23	317	2222	1925	19	0	1659

TRINITY_DN15159_c0_g1_i1	Glyma.02G237000.2	97.6744186	305	23	317	2222	1925	19	0	1659

TRINITY_DN15867_c0_g3_i5	Glyma.13G106700.16	95	233	5	189	2095	1	1922	0	2161

TRINITY_DN15867_c0_g3_i5	Glyma.13G106700.11	99.19354839	233	5	189	2095	1	1922	0	2161

TRINITY_DN15867_c0_g3_i5	Glyma.13G106700.12	97.2972973	233	5	189	2095	1	1922	0	2161

TRINITY_DN15867_c0_g3_i5	Glyma.13G106700.13	96.15384615	233	5	189	2095	1	1922	0	2161

TRINITY_DN15867_c0_g3_i5	Glyma.13G106700.14	98.82352941	233	5	189	2095	1	1922	0	2161

TRINITY_DN15867_c0_g3_i5	Glyma.13G106700.7	96.875	233	5	189	2095	1	1922	0	2161

TRINITY_DN15867_c0_g3_i5	Glyma.13G106700.1	98.61111111	233	5	189	2095	1	1922	0	2161

TRINITY_DN15867_c0_g3_i5	Glyma.13G106700.2	96.71717172	233	5	189	2095	1	1922	0	2161

TRINITY_DN15867_c0_g3_i5	Glyma.13G106700.3	98.4375	233	5	189	2095	1	1922	0	2161

TRINITY_DN15867_c0_g3_i5	Glyma.13G106700.4	95.56962025	233	5	189	2095	1	1922	0	2161

TRINITY_DN15867_c0_g3_i5	Glyma.13G106700.17	100	233	5	189	2095	1	1922	0	2161

TRINITY_DN15867_c0_g3_i5	Glyma.17G052600.1	98.87640449	217	5	189	2095	1	1922	0	2250

TRINITY_DN1827_c0_g1_i1	Glyma.05G014000.1	98.57142857	214	17	193	2082	138	2058	0	2150

TRINITY_DN16368_c0_g2_i2	Glyma.02G145100.1	100	167	0	5226	7147	1974	53	0	2625

TRINITY_DN12292_c0_g5_i1	Glyma.17G128500.1	100	165	14	1	1893	694	2613	0	2435

TRINITY_DN12292_c0_g5_i1	Glyma.17G128500.3	100	165	14	1	1893	433	2352	0	2435

TRINITY_DN12292_c0_g5_i1	Glyma.17G128500.11	100	165	14	1	1893	271	2190	0	2435

TRINITY_DN11438_c0_g2_i2	Glyma.03G234000.1	100	149	0	234	2155	1926	5	0	2724

TRINITY_DN14268_c0_g1_i1	Glyma.19G007300.4	100	133	2	681	2599	1	1919	0	2774

TRINITY_DN14268_c0_g1_i2	Glyma.19G007300.4	100	133	2	670	2588	1	1919	0	2774

TRINITY_DN14605_c0_g1_i2	Glyma.18G275600.4	99.21875	120	4	473	2392	1	1917	0	2839

TRINITY_DN14605_c0_g1_i2	Glyma.18G275600.3	97.85407725	120	4	473	2392	1	1917	0	2839

TRINITY_DN14605_c0_g1_i2	Glyma.18G275600.2	96.06557377	120	4	473	2392	1	1917	0	2839

TRINITY_DN14605_c0_g1_i2	Glyma.18G275600.1	96.06741573	120	4	473	2392	1	1917	0	2839

TRINITY_DN14605_c0_g1_i3	Glyma.18G275600.4	96.46017699	120	4	332	2251	1	1917	0	2839

TRINITY_DN14605_c0_g1_i3	Glyma.18G275600.3	100	120	4	332	2251	1	1917	0	2839

TRINITY_DN14605_c0_g1_i3	Glyma.18G275600.2	100	120	4	332	2251	1	1917	0	2839

TRINITY_DN14605_c0_g1_i3	Glyma.18G275600.1	95.95959596	120	4	332	2251	1	1917	0	2839

TRINITY_DN12257_c0_g1_i1	Glyma.15G127700.2	97.49478079	110	3	118	2039	148	2055	0	2850

TRINITY_DN13117_c0_g1_i1	Glyma.04G069500.2	100	300	25	286	2190	1917	10	0	1698

TRINITY_DN13117_c0_g1_i1	Glyma.04G069500.3	100	300	25	286	2190	1917	10	0	1698

TRINITY_DN13117_c0_g1_i1	Glyma.04G069500.1	99.51690821	300	25	286	2190	1917	10	0	1698

TRINITY_DN13117_c0_g1_i2	Glyma.04G069500.2	99.51923077	300	25	722	2626	1917	10	0	1698

TRINITY_DN13117_c0_g1_i2	Glyma.04G069500.3	99.51923077	300	25	722	2626	1917	10	0	1698

TRINITY_DN13117_c0_g1_i2	Glyma.04G069500.1	99.51923077	300	25	722	2626	1917	10	0	1698

TRINITY_DN12815_c1_g1_i3	Glyma.18G095200.2	96.98492462	280	27	714	2609	1881	1	0	1576

TRINITY_DN12290_c0_g1_i1	Glyma.01G219000.1	100	250	18	1829	3729	1907	1	0	1943

TRINITY_DN14726_c0_g3_i1	Glyma.17G254100.1	95.08196721	247	9	396	2312	2160	247	0	2108

TRINITY_DN16332_c0_g3_i4	Glyma.17G239500.5	100	206	8	869	2782	2877	961	0	2335

TRINITY_DN16332_c0_g3_i4	Glyma.17G239500.1	100	206	8	869	2782	2877	961	0	2335

TRINITY_DN15867_c0_g3_i5	Glyma.15G172400.7	99.82300885	191	6	189	2096	1	1917	0	2379

TRINITY_DN15867_c0_g3_i5	Glyma.15G172400.2	95.22357724	191	6	189	2096	1	1917	0	2379

TRINITY_DN15867_c0_g3_i5	Glyma.15G172400.3	97.93814433	191	6	189	2096	1	1917	0	2379

TRINITY_DN15867_c0_g3_i5	Glyma.15G172400.6	97.4789916	191	6	189	2096	1	1917	0	2379

TRINITY_DN15867_c0_g3_i5	Glyma.15G172400.1	100	191	6	189	2096	1	1917	0	2379

TRINITY_DN11081_c0_g2_i1	Glyma.17G078000.2	98.68421053	182	0	192	2112	37	1957	0	2540

TRINITY_DN3906_c0_g2_i1	Glyma.14G007100.1	96.1038961	178	2	1	1921	1653	3567	0	2523

TRINITY_DN3906_c0_g2_i1	Glyma.14G007100.2	96.1038961	178	2	1	1921	1653	3567	0	2523

TRINITY_DN3906_c0_g2_i1	Glyma.02G305900.2	96.1038961	168	3	1	1921	1650	3555	0	2519

TRINITY_DN3906_c0_g2_i1	Glyma.02G305900.3	96.1038961	168	3	1	1921	1650	3555	0	2519

TRINITY_DN3906_c0_g2_i1	Glyma.02G305900.1	96.1038961	168	3	1	1921	1650	3555	0	2519

TRINITY_DN9739_c0_g1_i1	Glyma.11G157700.1	95.83333333	156	40	35	1943	31	1872	0	2095

TRINITY_DN10373_c0_g1_i1	Glyma.09G283700.1	98.5915493	154	12	257	2172	1148	3042	0	2494

TRINITY_DN16027_c1_g3_i1	Glyma.13G346900.1	98.61111111	151	8	502	2392	1921	1	0	2518

TRINITY_DN16027_c1_g3_i1	Glyma.13G346900.2	98.11320755	151	8	502	2392	1921	1	0	2518

TRINITY_DN16027_c1_g3_i4	Glyma.13G346900.1	95.0310559	151	8	502	2392	1921	1	0	2518

TRINITY_DN16027_c1_g3_i4	Glyma.13G346900.2	95.03311258	151	8	502	2392	1921	1	0	2518

TRINITY_DN10373_c0_g1_i1	Glyma.19G111600.1	98.6013986	142	11	257	2172	1175	3069	0	2560

TRINITY_DN11359_c0_g2_i1	Glyma.08G074900.1	100	139	2	556	2475	1	1920	0	2765

TRINITY_DN11359_c0_g2_i2	Glyma.08G074900.1	96.55172414	139	2	673	2592	1	1920	0	2765

TRINITY_DN9361_c0_g1_i1	Glyma.12G070100.2	98.98989899	135	4	780	2697	1918	1	0	2761

TRINITY_DN15912_c1_g1_i2	Glyma.13G357100.4	97.68683274	112	16	771	2669	1908	1	0	2700

TRINITY_DN16368_c0_g2_i2	Glyma.03G156700.1	100	283	6	5231	7147	1966	50	0	1940

TRINITY_DN6726_c0_g1_i1	Glyma.13G277400.2	95.15669516	221	9	288	2198	123	2033	0	2206

TRINITY_DN12815_c1_g1_i4	Glyma.18G095200.2	98.38709677	221	25	714	2609	1881	1	0	1914

TRINITY_DN13361_c0_g1_i1	Glyma.13G189200.1	100	204	10	102	1997	1	1911	0	2202

TRINITY_DN15867_c0_g3_i5	Glyma.09G066400.1	100	203	6	189	2096	1	1911	0	2285

TRINITY_DN15159_c0_g1_i1	Glyma.18G051100.4	95.77167019	182	9	292	2201	1920	5	0	2447

TRINITY_DN15028_c0_g1_i3	Glyma.06G161700.3	97.36842105	176	3	1320	3239	1917	1	0	2551

TRINITY_DN15028_c0_g1_i3	Glyma.06G161700.1	100	176	3	1320	3239	1917	1	0	2551

TRINITY_DN15028_c0_g1_i3	Glyma.06G161700.2	100	176	3	1320	3239	1917	1	0	2551

TRINITY_DN15028_c0_g1_i3	Glyma.06G161700.4	96.2406015	176	3	1320	3239	1917	1	0	2551

TRINITY_DN36871_c0_g1_i1	Glyma.08G037600.2	95.56074766	165	7	2	1918	274	2175	0	2495

TRINITY_DN11359_c0_g2_i1	Glyma.05G120100.1	100	147	0	556	2475	1	1920	0	2732

TRINITY_DN11359_c0_g2_i1	Glyma.05G120100.2	100	147	0	556	2475	1	1920	0	2732

TRINITY_DN11359_c0_g2_i1	Glyma.05G120100.3	99.06542056	147	0	556	2475	1	1920	0	2732

TRINITY_DN11359_c0_g2_i2	Glyma.05G120100.1	96.55172414	147	0	673	2592	1	1920	0	2732

TRINITY_DN11359_c0_g2_i2	Glyma.05G120100.2	97.93814433	147	0	673	2592	1	1920	0	2732

TRINITY_DN11359_c0_g2_i2	Glyma.05G120100.3	95.04950495	147	0	673	2592	1	1920	0	2732

TRINITY_DN11882_c0_g1_i1	Glyma.04G163400.1	95.83333333	142	19	176	2086	1	1890	0	2507

TRINITY_DN11882_c0_g1_i3	Glyma.04G163400.1	95.83333333	142	19	176	2086	1	1890	0	2507

TRINITY_DN9773_c0_g1_i1	Glyma.17G107500.1	100	134	9	312	2225	1902	1	0	2649

TRINITY_DN14605_c0_g1_i2	Glyma.08G253000.1	97.72727273	116	1	473	2392	1	1917	0	2883

TRINITY_DN14605_c0_g1_i2	Glyma.08G253000.4	99.38650307	116	1	473	2392	1	1917	0	2883

TRINITY_DN14605_c0_g1_i2	Glyma.08G253000.2	99.38650307	116	1	473	2392	1	1917	0	2883

TRINITY_DN14605_c0_g1_i2	Glyma.08G253000.3	99.21875	116	1	473	2392	1	1917	0	2883

TRINITY_DN14605_c0_g1_i3	Glyma.08G253000.1	96.39889197	116	1	332	2251	1	1917	0	2883

TRINITY_DN14605_c0_g1_i3	Glyma.08G253000.4	96.40102828	116	1	332	2251	1	1917	0	2883

TRINITY_DN14605_c0_g1_i3	Glyma.08G253000.2	95.75757576	116	1	332	2251	1	1917	0	2883

TRINITY_DN14605_c0_g1_i3	Glyma.08G253000.3	96.46017699	116	1	332	2251	1	1917	0	2883

TRINITY_DN15992_c0_g1_i1	Glyma.17G092200.1	97	376	31	480	2381	4268	2382	0	1144

TRINITY_DN15992_c0_g1_i2	Glyma.17G092200.1	96.77419355	376	31	348	2249	4268	2382	0	1144

TRINITY_DN15992_c0_g1_i3	Glyma.17G092200.1	100	376	31	281	2182	4268	2382	0	1144

TRINITY_DN3110_c0_g1_i1	Glyma.08G093800.1	96.28180039	333	30	447	2330	2009	111	0	1343

TRINITY_DN13117_c0_g1_i1	Glyma.06G071200.1	99.51690821	305	23	286	2190	1917	10	0	1692

TRINITY_DN13117_c0_g1_i2	Glyma.06G071200.1	99.51923077	305	23	722	2626	1917	10	0	1692

TRINITY_DN5302_c0_g1_i1	Glyma.14G070700.1	98.88888889	166	19	331	2211	1911	1	0	2327

TRINITY_DN5302_c0_g1_i1	Glyma.14G070700.2	98.88888889	166	19	331	2211	1911	1	0	2327

TRINITY_DN12292_c0_g5_i1	Glyma.17G128500.4	100	165	13	1	1893	694	2610	0	2449

TRINITY_DN12292_c0_g5_i1	Glyma.17G128500.2	100	165	13	1	1893	433	2349	0	2449

TRINITY_DN12292_c0_g5_i1	Glyma.17G128500.10	100	165	13	1	1893	271	2187	0	2449

TRINITY_DN16274_c0_g2_i1	Glyma.12G189500.2	100	94	3	1	1913	1100	3012	0	2946

TRINITY_DN6249_c0_g1_i1	Glyma.05G052700.1	100	343	23	1	1909	138	2031	0	1428

TRINITY_DN11875_c0_g1_i1	Glyma.08G105200.1	95.3125	340	33	1076	2970	960	2851	0	1341

TRINITY_DN13097_c0_g1_i2	Glyma.18G094400.2	100	307	31	236	2116	2061	169	0	1441

TRINITY_DN16368_c0_g2_i2	Glyma.19G159000.1	100	290	2	5231	7147	1966	50	0	1923

TRINITY_DN16332_c0_g3_i4	Glyma.17G239500.3	100	207	6	869	2782	2874	961	0	2344

TRINITY_DN16332_c0_g3_i4	Glyma.17G239500.4	100	207	6	869	2782	2874	961	0	2344

TRINITY_DN16149_c1_g1_i1	Glyma.04G081100.4	100	179	12	407	2314	1928	30	0	2362

TRINITY_DN14204_c0_g1_i2	Glyma.11G067800.1	100	169	6	145	2061	1	1893	0	2438

TRINITY_DN16027_c1_g3_i1	Glyma.15G027500.2	100	154	5	502	2392	1918	1	0	2514

TRINITY_DN16027_c1_g3_i1	Glyma.15G027500.3	97.34513274	154	5	502	2392	1918	1	0	2514

TRINITY_DN16027_c1_g3_i4	Glyma.15G027500.2	100	154	5	502	2392	1918	1	0	2514

TRINITY_DN16027_c1_g3_i4	Glyma.15G027500.3	97.98657718	154	5	502	2392	1918	1	0	2514

TRINITY_DN14204_c0_g1_i1	Glyma.11G067800.1	100	140	6	145	2061	1	1893	0	2599

TRINITY_DN12978_c0_g1_i1	Glyma.16G039400.1	100	134	5	91	2008	3	1911	0	2741

TRINITY_DN12978_c0_g1_i1	Glyma.16G039400.2	100	134	5	91	2008	3	1911	0	2741

TRINITY_DN12978_c0_g1_i2	Glyma.16G039400.1	100	134	5	87	2004	3	1911	0	2741

TRINITY_DN12978_c0_g1_i2	Glyma.16G039400.2	97.77777778	134	5	87	2004	3	1911	0	2741

TRINITY_DN13088_c0_g1_i2	Glyma.10G238100.1	97.5	106	3	564	2475	1	1918	0	2916

TRINITY_DN15778_c0_g1_i1	Glyma.01G111200.4	97.12230216	98	0	998	2915	1923	6	0	3000

TRINITY_DN16401_c1_g2_i14	Glyma.20G048800.1	98.79518072	289	25	305	2185	3338	1429	0	1661

TRINITY_DN16401_c1_g2_i15	Glyma.20G048800.1	97.27047146	275	27	305	2182	3338	1429	0	1720

TRINITY_DN15970_c0_g2_i10	Glyma.18G188000.1	100	258	12	230	2124	31	1943	0	1943

TRINITY_DN15970_c0_g2_i4	Glyma.18G188000.1	95.23809524	258	12	230	2124	31	1943	0	1943

TRINITY_DN15020_c0_g1_i3	Glyma.05G196700.1	99.07407407	252	51	462	2315	1872	1	0	1447

TRINITY_DN13020_c0_g1_i1	Glyma.04G162400.1	100	215	10	239	2149	1	1893	0	2156

TRINITY_DN9258_c1_g1_i1	Glyma.19G115800.4	99.21875	198	2	333	2248	1916	1	0	2431

TRINITY_DN5648_c0_g1_i1	Glyma.20G110300.5	98.7804878	174	16	517	2402	1964	3837	0	2098

TRINITY_DN15918_c0_g1_i7	Glyma.13G055700.1	100	174	18	807	2701	1	1905	0	2357

TRINITY_DN14204_c0_g1_i2	Glyma.01G175600.1	100	165	9	145	2061	1	1893	0	2471

TRINITY_DN15876_c0_g1_i1	Glyma.06G040600.3	97.84946237	163	5	398	2307	228	2143	0	2586

TRINITY_DN15876_c0_g1_i1	Glyma.06G040600.4	97.84946237	163	5	398	2307	228	2143	0	2586

TRINITY_DN12926_c0_g1_i1	Glyma.13G124600.2	95.96491228	161	5	17	1929	1	1910	0	2577

TRINITY_DN15560_c0_g1_i1	Glyma.10G037500.2	99.34640523	161	6	291	2205	1	1905	0	2558

TRINITY_DN15560_c0_g1_i2	Glyma.10G037500.2	99.27007299	161	6	359	2273	1	1905	0	2558

TRINITY_DN10035_c0_g1_i1	Glyma.15G142600.1	97.91666667	158	14	2503	4389	3448	5361	0	2451

TRINITY_DN10035_c0_g1_i1	Glyma.15G142600.3	100	158	14	2503	4389	3403	5316	0	2451

TRINITY_DN10035_c0_g1_i1	Glyma.15G142600.2	100	158	14	2503	4389	3397	5310	0	2451

TRINITY_DN10477_c0_g2_i3	Glyma.05G153400.2	97.21115538	138	11	240	2137	175	2090	0	2647

TRINITY_DN14204_c0_g1_i1	Glyma.01G175600.1	100	132	9	145	2061	1	1893	0	2654

TRINITY_DN16413_c0_g1_i3	Glyma.17G188300.2	100	121	0	1750	3666	1917	1	0	2870

TRINITY_DN16413_c0_g1_i3	Glyma.17G188300.1	98.52216749	121	0	1750	3666	1917	1	0	2870

TRINITY_DN15252_c0_g1_i1	Glyma.17G143300.3	100	117	4	854	2758	1911	1	0	2776

TRINITY_DN16413_c0_g1_i3	Glyma.14G139800.1	97.6744186	114	0	1750	3666	1917	1	0	2909

TRINITY_DN14149_c0_g1_i2	Glyma.08G196200.1	100	102	1	446	2362	1	1914	0	2955

TRINITY_DN14149_c0_g1_i2	Glyma.07G012500.1	100	102	1	446	2362	1	1914	0	2955

TRINITY_DN11254_c0_g1_i1	Glyma.13G347600.1	98.36065574	404	39	668	2559	1990	99	0	990

TRINITY_DN15048_c0_g1_i2	Glyma.15G145200.4	100	309	29	961	2859	1947	61	0	1530

TRINITY_DN15048_c0_g1_i2	Glyma.15G145200.3	100	309	29	961	2859	1947	61	0	1530

TRINITY_DN15048_c0_g1_i2	Glyma.15G145200.2	96.49595687	309	29	961	2859	1947	61	0	1530

TRINITY_DN5703_c0_g1_i1	Glyma.07G255300.1	100	297	8	240	2151	2364	453	0	1842

TRINITY_DN12815_c1_g1_i2	Glyma.18G095200.2	96.99074074	262	24	714	2606	1881	1	0	1712

TRINITY_DN15510_c0_g1_i3	Glyma.09G168600.2	100	251	27	450	2342	8	1903	0	1869

TRINITY_DN15510_c0_g1_i6	Glyma.09G168600.2	100	251	27	424	2316	8	1903	0	1869

TRINITY_DN16332_c0_g3_i4	Glyma.14G085200.1	100	217	6	871	2782	2872	961	0	2285

TRINITY_DN16332_c0_g3_i4	Glyma.14G085200.2	100	217	6	871	2782	2683	772	0	2285

TRINITY_DN6249_c0_g1_i1	Glyma.11G030000.1	100	194	2	2	1916	151	2065	0	2451

TRINITY_DN15820_c0_g2_i2	Glyma.13G026100.1	99.45945946	193	2	436	2350	1974	60	0	2457

TRINITY_DN15918_c0_g1_i7	Glyma.19G031100.1	98.48053181	190	15	807	2701	1	1905	0	2279

TRINITY_DN13508_c0_g1_i3	Glyma.18G210100.2	100	176	40	215	2036	14	1904	0	1794

TRINITY_DN9258_c1_g1_i2	Glyma.19G115800.4	96.64804469	134	0	589	2504	1916	1	0	2796

TRINITY_DN5259_c0_g3_i1	Glyma.03G090000.3	96.51162791	104	0	183	2098	17	1932	0	2963

TRINITY_DN16379_c0_g2_i1	Glyma.04G186000.1	98.55072464	397	16	364	2271	2364	460	0	1227

TRINITY_DN17221_c0_g1_i1	Glyma.08G281700.1	96.05263158	319	20	423	2318	1905	1	0	1581

TRINITY_DN14245_c0_g2_i1	Glyma.13G241400.7	95.65217391	287	29	1269	3153	1	1897	0	1637

TRINITY_DN14245_c0_g2_i3	Glyma.13G241400.7	97.32620321	287	29	1214	3098	1	1897	0	1637

TRINITY_DN14245_c0_g2_i2	Glyma.13G241400.7	95.66003617	286	29	529	2413	1	1897	0	1642

TRINITY_DN15970_c0_g2_i10	Glyma.07G137300.1	97.10144928	258	8	230	2124	166	2078	0	1965

TRINITY_DN15970_c0_g2_i4	Glyma.07G137300.1	100	258	8	230	2124	166	2078	0	1965

TRINITY_DN15020_c0_g1_i3	Glyma.08G004300.1	99.10802775	252	50	462	2315	1869	1	0	1448

TRINITY_DN12886_c0_g1_i2	Glyma.04G194900.1	96.07843137	227	11	846	2747	3426	1519	0	2150

TRINITY_DN12886_c0_g1_i2	Glyma.04G194900.2	96.09375	227	11	846	2747	3327	1420	0	2150

TRINITY_DN12886_c0_g1_i2	Glyma.04G194900.3	96.08433735	227	11	846	2747	3327	1420	0	2150

TRINITY_DN16191_c2_g1_i2	Glyma.14G102000.1	98.26839827	210	3	77	1987	1914	1	0	2340

TRINITY_DN16191_c2_g1_i2	Glyma.17G223200.1	96.17346939	206	3	77	1987	1914	1	0	2362

TRINITY_DN12815_c1_g1_i1	Glyma.18G095200.2	96.98857736	203	22	714	2606	1881	1	0	2050

TRINITY_DN13985_c0_g1_i1	Glyma.12G012000.2	95.77464789	200	15	268	2167	1	1906	0	2274

TRINITY_DN11819_c0_g3_i3	Glyma.19G103000.3	96.76549865	177	7	53	1958	1	1915	0	2497

TRINITY_DN16191_c2_g1_i1	Glyma.04G060700.1	100	174	5	496	2403	1914	1	0	2521

TRINITY_DN16191_c2_g1_i1	Glyma.06G061300.1	97.39776952	167	6	496	2403	1914	1	0	2560

TRINITY_DN15668_c0_g1_i1	Glyma.15G174100.1	95.83333333	141	13	317	2206	1914	1	0	2588

TRINITY_DN16191_c2_g1_i1	Glyma.17G223200.1	96.19047619	139	4	496	2403	1914	1	0	2715

TRINITY_DN11882_c0_g1_i1	Glyma.06G202100.2	95.83333333	138	19	179	2082	7	1898	0	2553

TRINITY_DN11882_c0_g1_i3	Glyma.06G202100.2	95.83333333	138	19	179	2082	7	1898	0	2553

TRINITY_DN16191_c2_g1_i1	Glyma.14G102000.1	96.19047619	135	4	496	2403	1914	1	0	2737

TRINITY_DN11677_c0_g1_i1	Glyma.04G015600.1	100	130	4	163	2071	1	1897	0	2662

TRINITY_DN5893_c0_g1_i1	Glyma.05G001800.1	99.13043478	105	8	331	2236	96	1989	0	2761

TRINITY_DN12659_c1_g1_i1	Glyma.01G140600.6	100	100	3	235	2145	1914	1	0	2950

TRINITY_DN12659_c1_g1_i1	Glyma.01G140600.9	98.11320755	100	3	235	2145	1914	1	0	2950

TRINITY_DN12659_c1_g1_i1	Glyma.01G140600.8	98.10126582	100	3	235	2145	1914	1	0	2950

TRINITY_DN16191_c2_g1_i2	Glyma.06G061300.1	96.1722488	95	3	77	1987	1914	1	0	2977

TRINITY_DN16379_c0_g2_i1	Glyma.06G180000.1	98.55072464	396	14	364	2271	2364	460	0	1243

TRINITY_DN16379_c0_g2_i6	Glyma.04G186000.1	97.82608696	393	14	403	2310	2364	460	0	1260

TRINITY_DN37437_c0_g1_i1	Glyma.04G211800.1	96.08801956	268	32	186	2081	16	1904	0	1772

TRINITY_DN13471_c0_g2_i1	Glyma.16G022600.6	99.18699187	235	7	205	2115	1897	1	0	2104

TRINITY_DN13471_c0_g2_i1	Glyma.16G022600.4	97.22222222	235	8	205	2115	1894	1	0	2084

TRINITY_DN13020_c0_g1_i1	Glyma.06G203900.1	97.6744186	227	16	239	2128	1	1890	0	1967

TRINITY_DN9949_c0_g2_i1	Glyma.06G085300.2	96.12403101	202	23	214	2097	1	1896	0	2106

TRINITY_DN9949_c0_g2_i1	Glyma.06G085300.1	99.45945946	202	23	214	2097	1	1896	0	2106

TRINITY_DN16274_c0_g2_i1	Glyma.12G095300.4	96.20535714	194	4	1	1913	1103	3006	0	2388

TRINITY_DN9949_c0_g2_i1	Glyma.06G085200.4	99.46236559	193	9	214	2103	1	1914	0	2311

TRINITY_DN9949_c0_g2_i1	Glyma.06G085200.1	99.45945946	193	9	214	2103	1	1914	0	2311

TRINITY_DN9949_c0_g2_i1	Glyma.06G085200.2	99.44444444	193	9	214	2103	1	1914	0	2311

TRINITY_DN9949_c0_g2_i1	Glyma.06G085200.3	99.45355191	193	9	214	2103	1	1914	0	2311

TRINITY_DN16274_c0_g2_i1	Glyma.U010500.2	95.4887218	189	3	1	1913	1127	3033	0	2436

TRINITY_DN10014_c0_g1_i1	Glyma.04G248100.1	98.88888889	179	23	547	2421	2514	604	0	2272

TRINITY_DN15533_c0_g1_i2	Glyma.13G092700.1	95.23809524	153	12	1852	3737	1211	3111	0	2422

TRINITY_DN15533_c0_g1_i3	Glyma.13G092700.1	100	153	12	3192	5077	1211	3111	0	2422

TRINITY_DN7895_c0_g1_i1	Glyma.16G082400.2	100	151	4	1	1904	245	2157	0	2627

TRINITY_DN12123_c0_g1_i1	Glyma.17G037100.1	95.5801105	149	1	320	2230	1914	1	0	2689

TRINITY_DN12123_c0_g1_i7	Glyma.17G037100.1	95.23809524	149	1	488	2398	1914	1	0	2689

TRINITY_DN14499_c1_g1_i2	Glyma.01G048500.4	97.37903226	137	7	488	2397	1	1910	0	2724

TRINITY_DN14499_c1_g1_i2	Glyma.01G048500.3	100	137	7	488	2397	1	1910	0	2724

TRINITY_DN14499_c1_g1_i2	Glyma.01G048500.2	100	137	7	488	2397	1	1910	0	2724

TRINITY_DN14499_c1_g1_i2	Glyma.01G048500.1	98.14814815	137	7	488	2397	1	1910	0	2724

TRINITY_DN12978_c0_g1_i1	Glyma.19G113500.2	97.31051345	130	5	91	2004	3	1907	0	2756

TRINITY_DN12978_c0_g1_i1	Glyma.19G113500.1	96.66666667	130	5	91	2004	3	1907	0	2756

TRINITY_DN12978_c0_g1_i2	Glyma.19G113500.2	98.8372093	130	5	87	2000	3	1907	0	2756

TRINITY_DN12978_c0_g1_i2	Glyma.19G113500.1	100	130	5	87	2000	3	1907	0	2756

TRINITY_DN18374_c0_g1_i1	Glyma.12G180400.1	97.93814433	129	4	255	2162	1	1908	0	2743

TRINITY_DN16398_c1_g1_i2	Glyma.08G256300.2	100	126	0	666	2579	13	1926	0	2837

TRINITY_DN16332_c0_g3_i4	Glyma.06G042400.1	100	116	2	869	2782	2856	958	0	2795

TRINITY_DN16332_c0_g3_i4	Glyma.06G042400.2	100	116	2	869	2782	2670	772	0	2795

TRINITY_DN9393_c0_g1_i2	Glyma.07G106100.11	100	115	1	255	2165	1914	1	0	2878

TRINITY_DN16332_c0_g3_i4	Glyma.04G041300.1	100	109	1	869	2782	2868	958	0	2911

TRINITY_DN16332_c0_g3_i4	Glyma.04G041300.2	100	109	1	869	2782	2682	772	0	2911

TRINITY_DN16191_c2_g1_i2	Glyma.04G060700.1	100	96	1	77	1987	1914	1	0	2983

TRINITY_DN16360_c0_g4_i7	Glyma.13G104000.1	100	435	36	449	2343	121	2009	0	852

TRINITY_DN16379_c0_g2_i6	Glyma.06G180000.1	97.81420765	396	12	403	2310	2364	460	0	1254

TRINITY_DN16379_c0_g2_i1	Glyma.12G177100.1	98.56115108	340	10	365	2271	2342	436	0	1572

TRINITY_DN15510_c0_g1_i3	Glyma.09G168400.2	98.75	279	21	459	2339	17	1921	0	1729

TRINITY_DN15510_c0_g1_i6	Glyma.09G168400.2	98.11320755	279	21	433	2313	17	1921	0	1729

TRINITY_DN5434_c0_g1_i1	Glyma.17G206100.1	96.875	241	2	616	2527	1	1912	0	2185

TRINITY_DN14439_c1_g2_i1	Glyma.17G104400.1	97.03703704	232	3	262	2171	2049	140	0	2209

TRINITY_DN14439_c1_g2_i1	Glyma.13G156800.1	100	217	2	262	2171	2049	140	0	2292

TRINITY_DN15533_c0_g1_i2	Glyma.17G067700.1	95.23809524	173	9	1852	3737	1202	3105	0	2342

TRINITY_DN15533_c0_g1_i3	Glyma.17G067700.1	97.84836066	173	9	3192	5077	1202	3105	0	2342

TRINITY_DN15136_c0_g1_i2	Glyma.13G201500.1	98.80952381	136	5	313	2220	1917	10	0	2715

TRINITY_DN8945_c0_g1_i1	Glyma.16G116100.2	97.02970297	286	54	1221	3103	749	2565	0	1146

TRINITY_DN8945_c0_g1_i1	Glyma.16G116100.1	97.01492537	286	54	1221	3103	749	2565	0	1146

TRINITY_DN13417_c0_g1_i1	Glyma.08G027400.1	97.01492537	263	30	88	1974	1	1899	0	1829

TRINITY_DN12886_c0_g1_i2	Glyma.06G171000.1	96.1038961	245	10	846	2747	3438	1534	0	2063

TRINITY_DN12886_c0_g1_i2	Glyma.06G171000.2	96.07843137	245	10	846	2747	3339	1435	0	2063

TRINITY_DN16027_c1_g3_i5	Glyma.07G036300.8	97.45762712	198	24	385	2244	1899	1	0	2013

TRINITY_DN16027_c1_g3_i5	Glyma.07G036300.1	97.45762712	198	24	385	2244	1899	1	0	2013

TRINITY_DN16027_c1_g3_i7	Glyma.07G036300.8	97.97843666	198	24	385	2244	1899	1	0	2013

TRINITY_DN16027_c1_g3_i7	Glyma.07G036300.1	97.97979798	198	24	385	2244	1899	1	0	2013

TRINITY_DN2883_c0_g1_i1	Glyma.19G188700.3	99.53051643	163	10	82	1983	1	1899	0	2479

TRINITY_DN11708_c0_g1_i1	Glyma.06G141100.1	98.92473118	157	3	277	2181	1911	1	0	2610

TRINITY_DN11708_c0_g1_i1	Glyma.06G141100.2	96.89578714	157	3	277	2181	1911	1	0	2610

TRINITY_DN11708_c0_g1_i1	Glyma.06G141100.3	96.89621726	157	3	277	2181	1911	1	0	2610

TRINITY_DN11708_c0_g1_i2	Glyma.06G141100.1	100	157	3	277	2181	1911	1	0	2610

TRINITY_DN11708_c0_g1_i2	Glyma.06G141100.2	95.84415584	157	3	277	2181	1911	1	0	2610

TRINITY_DN11708_c0_g1_i2	Glyma.06G141100.3	95.84462511	157	3	277	2181	1911	1	0	2610

TRINITY_DN11708_c0_g1_i3	Glyma.06G141100.1	98.820059	157	3	277	2181	1911	1	0	2610

TRINITY_DN11708_c0_g1_i3	Glyma.06G141100.2	98.82352941	157	3	277	2181	1911	1	0	2610

TRINITY_DN11708_c0_g1_i3	Glyma.06G141100.3	98.4375	157	3	277	2181	1911	1	0	2610

TRINITY_DN2629_c0_g1_i1	Glyma.07G004800.1	95.97315436	146	4	263	2173	3417	1522	0	2612

TRINITY_DN12978_c0_g1_i2	Glyma.19G113600.1	95	138	3	86	1997	20	1922	0	2708

TRINITY_DN12978_c0_g1_i1	Glyma.19G113600.1	100	136	3	90	2001	20	1922	0	2719

TRINITY_DN12892_c0_g4_i1	Glyma.12G028100.1	100	114	3	889	2794	564	2475	0	2861

TRINITY_DN5259_c0_g3_i1	Glyma.16G083100.3	100	112	2	188	2098	1	1911	0	2898

TRINITY_DN15782_c0_g1_i1	Glyma.04G087300.1	96.36363636	101	2	174	2084	2434	4344	0	2959

TRINITY_DN15782_c0_g1_i1	Glyma.06G089000.2	97.39292365	92	3	174	2084	2437	4344	0	2988

TRINITY_DN15782_c0_g1_i1	Glyma.06G089000.1	98.93617021	92	3	174	2084	2437	4344	0	2988

TRINITY_DN5703_c0_g1_i1	Glyma.17G019000.1	100	291	10	246	2151	2358	453	0	1853

TRINITY_DN14245_c0_g2_i1	Glyma.13G241400.8	95.65217391	266	33	451	2323	14	1901	0	1661

TRINITY_DN14245_c0_g2_i1	Glyma.13G241400.5	95.65217391	266	33	451	2323	14	1901	0	1661

TRINITY_DN14410_c0_g1_i1	Glyma.05G247300.1	99.31034483	212	19	140	2037	77	1971	0	2167

TRINITY_DN9258_c1_g1_i1	Glyma.16G037500.4	99.21875	203	5	335	2240	1917	9	0	2359

TRINITY_DN35781_c0_g1_i1	Glyma.01G170800.1	98.7804878	198	18	351	2252	2385	499	0	2218

TRINITY_DN2796_c0_g1_i1	Glyma.19G228200.1	100	179	16	241	2130	2154	259	0	2305

TRINITY_DN2883_c0_g1_i1	Glyma.19G188700.1	95.76271186	170	3	82	1983	1	1911	0	2529

TRINITY_DN15317_c0_g1_i1	Glyma.02G081000.5	98.11764706	143	6	355	2265	1	1896	0	2639

TRINITY_DN15317_c0_g1_i1	Glyma.02G081000.1	96.50924025	143	6	355	2265	1	1896	0	2639

TRINITY_DN15317_c0_g1_i6	Glyma.02G081000.5	98.7012987	143	6	139	2049	1	1896	0	2639

TRINITY_DN15317_c0_g1_i6	Glyma.02G081000.1	98.7012987	143	6	139	2049	1	1896	0	2639

TRINITY_DN6660_c0_g2_i2	Glyma.10G289000.2	97.33840304	143	9	422	2324	4	1912	0	2673

TRINITY_DN41079_c0_g1_i1	Glyma.15G193000.1	99.07407407	142	0	111	2021	787	2697	0	2743

TRINITY_DN2134_c0_g2_i1	Glyma.19G116400.1	98.75	133	6	170	2068	1	1911	0	2715

TRINITY_DN15441_c0_g1_i2	Glyma.19G213300.2	97.43589744	130	0	134	2044	1	1911	0	2809

TRINITY_DN15441_c0_g1_i2	Glyma.19G213300.1	97.43589744	130	0	134	2044	1	1911	0	2809

TRINITY_DN41079_c0_g1_i1	Glyma.09G084600.1	99.1091314	128	0	111	2021	805	2715	0	2820

TRINITY_DN15441_c0_g1_i2	Glyma.03G216700.1	97.43589744	128	0	134	2044	1	1911	0	2820

TRINITY_DN14238_c0_g2_i3	Glyma.13G247300.3	97.63593381	127	4	278	2183	1	1909	0	2780

TRINITY_DN14238_c0_g2_i3	Glyma.13G247300.1	97.61904762	127	4	278	2183	1	1909	0	2780

TRINITY_DN9393_c0_g1_i2	Glyma.07G106100.10	96.21621622	115	0	255	2165	1911	1	0	2892

TRINITY_DN12659_c1_g1_i1	Glyma.03G026900.4	100	100	0	235	2145	1911	1	0	2976

TRINITY_DN15683_c0_g2_i2	Glyma.15G015300.1	98.97260274	96	0	327	2237	195	2105	0	2998

TRINITY_DN3274_c0_g1_i1	Glyma.17G000600.2	97.93814433	421	50	712	2576	3092	1207	0	749

TRINITY_DN15137_c0_g1_i11	Glyma.14G222700.2	96	227	8	1275	3176	1976	69	0	2206

TRINITY_DN15137_c0_g1_i11	Glyma.14G222700.1	100	227	8	1275	3176	1976	69	0	2206

TRINITY_DN15137_c0_g1_i7	Glyma.14G222700.2	99.11504425	227	8	1388	3289	1976	69	0	2206

TRINITY_DN15137_c0_g1_i7	Glyma.14G222700.1	96.41025641	227	8	1388	3289	1976	69	0	2206

TRINITY_DN15020_c0_g1_i4	Glyma.05G196700.1	97.5308642	222	50	462	2330	1872	1	0	1786

TRINITY_DN14864_c0_g1_i3	Glyma.08G021400.1	95.14285714	191	9	193	2079	4236	2329	0	2307

TRINITY_DN16415_c1_g1_i15	Glyma.U018900.2	97.22222222	168	5	1692	3601	1901	1	0	2538

TRINITY_DN16415_c1_g1_i4	Glyma.U018900.2	100	168	5	1796	3705	1901	1	0	2538

TRINITY_DN11440_c0_g7_i1	Glyma.08G061800.1	99.15966387	168	18	230	2126	1885	1	0	2351

TRINITY_DN16415_c1_g1_i15	Glyma.11G147900.3	100	143	0	1692	3601	2057	148	0	2736

TRINITY_DN16415_c1_g1_i4	Glyma.11G147900.3	100	143	0	1796	3705	2057	148	0	2736

TRINITY_DN16415_c1_g1_i15	Glyma.11G147900.2	98.88888889	143	0	1692	3601	1910	1	0	2736

TRINITY_DN16415_c1_g1_i4	Glyma.11G147900.2	100	143	0	1796	3705	1910	1	0	2736

TRINITY_DN9258_c1_g1_i2	Glyma.16G037500.4	100	139	3	591	2496	1917	9	0	2724

TRINITY_DN15778_c0_g1_i1	Glyma.03G076800.3	100	106	0	1140	3049	1910	1	0	2940

TRINITY_DN16254_c0_g1_i7	Glyma.07G004300.1	99.41860465	440	39	224	2109	2828	946	0	771

TRINITY_DN16254_c0_g1_i7	Glyma.07G004300.2	99.41348974	440	39	224	2109	2726	844	0	771

TRINITY_DN16254_c0_g1_i7	Glyma.07G004300.3	99.41860465	440	39	224	2109	2411	529	0	771

TRINITY_DN13417_c0_g1_i1	Glyma.05G220900.1	100	263	22	88	1974	1	1899	0	1862

TRINITY_DN35835_c0_g1_i1	Glyma.17G207900.1	98.98989899	259	10	154	2050	2596	691	0	1993

TRINITY_DN2796_c0_g1_i1	Glyma.03G231000.1	99.10714286	184	9	241	2130	1905	1	0	2357

TRINITY_DN11440_c0_g7_i1	Glyma.08G061800.2	99.39393939	164	22	230	2126	1864	1	0	2248

TRINITY_DN16245_c0_g3_i2	Glyma.04G166100.1	97.54098361	160	6	375	2280	3500	1598	0	2580

TRINITY_DN5388_c0_g2_i1	Glyma.17G070300.1	97.4025974	158	11	66	1958	28	1935	0	2540

TRINITY_DN2821_c0_g1_i1	Glyma.13G118200.5	97.97979798	118	12	217	2124	183	2062	0	2678

TRINITY_DN2821_c0_g1_i1	Glyma.13G118200.1	97.97979798	118	12	217	2124	183	2062	0	2678

TRINITY_DN15683_c0_g2_i2	Glyma.13G358700.1	96.18138425	108	0	328	2236	196	2104	0	2928

TRINITY_DN16382_c0_g3_i3	Glyma.18G060600.2	96.66666667	102	7	489	2397	184	2070	0	2819

TRINITY_DN10067_c0_g4_i1	Glyma.15G163700.5	96.42857143	89	0	413	2321	1	1909	0	3033

TRINITY_DN10067_c0_g4_i2	Glyma.15G163700.5	95.87628866	89	0	413	2321	1	1909	0	3033

TRINITY_DN16254_c0_g1_i7	Glyma.08G222200.1	98.90710383	438	36	224	2109	2834	949	0	813

TRINITY_DN9125_c0_g1_i2	Glyma.10G195700.1	95.23809524	382	54	300	2188	3238	1380	0	968

TRINITY_DN9125_c0_g1_i2	Glyma.10G195700.2	95.23809524	382	54	300	2188	2308	450	0	968

TRINITY_DN15228_c0_g1_i4	Glyma.19G260400.5	98.8372093	359	60	424	2287	120	1980	0	946

TRINITY_DN11260_c0_g1_i1	Glyma.03G082800.1	100	268	32	839	2695	469	2361	0	1613

TRINITY_DN11099_c0_g2_i1	Glyma.10G172900.1	100	258	17	150	2045	271	2172	0	1978

TRINITY_DN11099_c0_g2_i1	Glyma.10G172900.2	100	258	17	150	2045	118	2019	0	1978

TRINITY_DN13471_c0_g2_i1	Glyma.07G053500.7	97.93814433	232	11	211	2115	1894	1	0	2128

TRINITY_DN13471_c0_g2_i1	Glyma.07G053500.5	97.93814433	232	11	211	2115	1894	1	0	2128

TRINITY_DN13471_c0_g2_i1	Glyma.07G053500.6	98.73417722	232	12	211	2115	1891	1	0	2109

TRINITY_DN13471_c0_g2_i1	Glyma.07G053500.4	100	232	12	211	2115	1891	1	0	2109

TRINITY_DN15020_c0_g1_i4	Glyma.08G004300.1	96.73913043	221	47	462	2330	1869	1	0	1796

TRINITY_DN16215_c0_g1_i2	Glyma.19G234300.1	100	220	14	313	2190	3228	1322	0	2104

TRINITY_DN16215_c0_g1_i4	Glyma.19G234300.1	100	216	14	537	2417	3228	1322	0	2146

TRINITY_DN2883_c0_g1_i1	Glyma.03G188500.1	95.76271186	156	4	82	1983	1	1899	0	2562

TRINITY_DN8298_c0_g1_i1	Glyma.12G092200.1	98.14814815	148	23	364	2231	1941	41	0	2399

TRINITY_DN16245_c0_g3_i2	Glyma.06G196400.1	99.25925926	146	10	378	2278	2993	1093	0	2625

TRINITY_DN18374_c0_g1_i1	Glyma.13G320400.1	99.53271028	142	2	255	2162	1	1902	0	2699

TRINITY_DN12123_c0_g1_i1	Glyma.07G236300.1	100	139	1	326	2230	1908	1	0	2734

TRINITY_DN12123_c0_g1_i7	Glyma.07G236300.1	96.47964796	139	1	494	2398	1908	1	0	2734

TRINITY_DN13514_c1_g1_i1	Glyma.05G012400.1	96	101	7	338	2242	7770	5872	0	2887

TRINITY_DN15992_c0_g1_i1	Glyma.05G035100.1	99.44134078	353	43	480	2360	4271	2400	0	1171

TRINITY_DN15992_c0_g1_i2	Glyma.05G035100.1	95.54140127	353	43	348	2228	4271	2400	0	1171

TRINITY_DN15992_c0_g1_i3	Glyma.05G035100.1	100	353	43	281	2161	4271	2400	0	1171

TRINITY_DN6849_c0_g3_i1	Glyma.02G116500.1	97.65258216	297	16	131	2034	2091	209	0	1701

TRINITY_DN16257_c1_g1_i10	Glyma.12G192100.1	100	278	15	103	1999	2241	342	0	1871

TRINITY_DN16257_c1_g1_i9	Glyma.12G192100.1	97.08029197	271	19	184	2073	2231	342	0	1801

TRINITY_DN11099_c0_g2_i1	Glyma.20G217400.3	100	261	18	150	2045	112	2010	0	1953

TRINITY_DN11099_c0_g2_i1	Glyma.20G217400.2	100	261	18	150	2045	112	2010	0	1953

TRINITY_DN11099_c0_g2_i1	Glyma.20G217400.1	95.59748428	261	18	150	2045	112	2010	0	1953

TRINITY_DN35835_c0_g1_i1	Glyma.14G126500.1	100	253	9	154	2050	2623	718	0	2049

TRINITY_DN9258_c1_g1_i1	Glyma.17G187200.2	97.56097561	243	10	326	2227	1921	22	0	2098

TRINITY_DN9258_c1_g1_i1	Glyma.17G187200.1	96.66666667	243	10	326	2227	1921	22	0	2098

TRINITY_DN9258_c1_g1_i1	Glyma.17G187200.3	96.58536585	243	10	326	2227	1921	22	0	2098

TRINITY_DN6758_c0_g1_i1	Glyma.13G339600.2	98.24561404	206	8	1	1897	1903	1	0	2290

TRINITY_DN6758_c0_g1_i1	Glyma.13G339600.1	98.24561404	206	8	1	1897	1903	1	0	2290

TRINITY_DN6758_c0_g1_i1	Glyma.13G339600.3	98.24561404	206	8	1	1897	1903	1	0	2290

TRINITY_DN16173_c1_g1_i5	Glyma.20G051500.2	95.91836735	196	30	334	2205	1875	1	0	2002

TRINITY_DN16173_c1_g1_i5	Glyma.20G051500.1	100	196	30	334	2205	1875	1	0	2002

TRINITY_DN10014_c0_g1_i1	Glyma.06G114600.1	98.125	171	20	564	2427	2512	610	0	2270

TRINITY_DN10014_c0_g1_i1	Glyma.06G114600.2	97.96954315	171	20	564	2427	2512	610	0	2270

TRINITY_DN37204_c0_g1_i1	Glyma.04G036300.2	99.03518729	152	13	120	1996	34	1931	0	2427

TRINITY_DN8457_c0_g1_i1	Glyma.07G102300.1	96.61835749	138	2	1	1907	209	2112	0	2737

TRINITY_DN8457_c0_g1_i1	Glyma.07G102300.2	96.62162162	138	2	1	1907	209	2112	0	2737

TRINITY_DN8457_c0_g1_i1	Glyma.07G102300.3	96.61016949	138	2	1	1907	209	2112	0	2737

TRINITY_DN12711_c0_g1_i1	Glyma.10G180600.3	95.34883721	130	3	986	2890	1	1905	0	2776

TRINITY_DN12711_c0_g1_i1	Glyma.10G180600.2	100	130	3	986	2890	1	1905	0	2776

TRINITY_DN10529_c0_g2_i2	Glyma.15G047100.1	99.68152866	125	1	837	2743	551	2454	0	2809

TRINITY_DN12717_c0_g1_i1	Glyma.03G244500.14	97.26027397	118	6	100	2006	1	1886	0	2732

TRINITY_DN10529_c0_g2_i2	Glyma.08G185700.1	99.70760234	116	0	837	2743	557	2463	0	2880

TRINITY_DN10529_c0_g2_i2	Glyma.08G185700.7	99.70760234	116	0	837	2743	347	2253	0	2880

TRINITY_DN10529_c0_g2_i2	Glyma.08G185700.2	99.73190349	116	0	837	2743	146	2052	0	2880

TRINITY_DN16138_c1_g1_i22	Glyma.08G183400.1	99.70760234	331	6	796	2697	525	2426	0	1635

TRINITY_DN16138_c1_g1_i22	Glyma.08G183400.10	97.08222812	331	6	796	2697	525	2426	0	1635

TRINITY_DN16138_c1_g1_i21	Glyma.08G183400.1	95.01039501	328	10	796	2685	525	2426	0	1574

TRINITY_DN16138_c1_g1_i21	Glyma.08G183400.10	100	328	10	796	2685	525	2426	0	1574

TRINITY_DN16138_c1_g1_i18	Glyma.08G183400.1	100	319	9	785	2678	525	2426	0	1650

TRINITY_DN16138_c1_g1_i18	Glyma.08G183400.10	100	319	9	785	2678	525	2426	0	1650

TRINITY_DN16138_c1_g1_i5	Glyma.08G183400.1	99.4011976	319	9	1051	2944	525	2426	0	1650

TRINITY_DN16138_c1_g1_i5	Glyma.08G183400.10	98.42519685	319	9	1051	2944	525	2426	0	1650

TRINITY_DN16138_c1_g1_i20	Glyma.08G183400.1	100	316	13	785	2666	525	2426	0	1589

TRINITY_DN16138_c1_g1_i20	Glyma.08G183400.10	100	316	13	785	2666	525	2426	0	1589

TRINITY_DN16138_c1_g1_i7	Glyma.08G183400.1	100	316	13	1051	2932	525	2426	0	1589

TRINITY_DN16138_c1_g1_i7	Glyma.08G183400.10	100	316	13	1051	2932	525	2426	0	1589

TRINITY_DN16138_c1_g1_i11	Glyma.08G183400.1	97.12389381	287	6	796	2697	525	2426	0	1879

TRINITY_DN16138_c1_g1_i11	Glyma.08G183400.10	98.18181818	287	6	796	2697	525	2426	0	1879

TRINITY_DN16173_c1_g1_i1	Glyma.20G051500.2	100	210	28	334	2205	1875	1	0	1936

TRINITY_DN16173_c1_g1_i1	Glyma.20G051500.1	100	210	28	334	2205	1875	1	0	1936

TRINITY_DN16173_c1_g1_i2	Glyma.20G051500.2	95.12195122	192	27	334	2208	1875	1	0	2056

TRINITY_DN16173_c1_g1_i2	Glyma.20G051500.1	95.12195122	192	27	334	2208	1875	1	0	2056

TRINITY_DN16274_c0_g2_i1	Glyma.12G095300.3	97.66081871	191	8	1	1893	1103	2998	0	2313

TRINITY_DN15940_c0_g1_i1	Glyma.18G263300.1	100	184	8	498	2397	120	2016	0	2403

TRINITY_DN16093_c0_g2_i1	Glyma.12G122100.1	95.07042254	183	8	1339	3235	723	2625	0	2429

TRINITY_DN16093_c0_g2_i2	Glyma.12G122100.1	96.59090909	183	8	1339	3235	723	2625	0	2429

TRINITY_DN15940_c0_g1_i1	Glyma.18G263300.2	100	182	14	498	2397	120	2007	0	2357

TRINITY_DN11440_c0_g7_i1	Glyma.07G187300.1	99.39393939	181	12	230	2126	1885	1	0	2324

TRINITY_DN5572_c0_g1_i1	Glyma.19G165500.4	100	178	7	675	2577	2188	300	0	2405

TRINITY_DN5572_c0_g1_i1	Glyma.19G165500.1	98.47826087	178	7	675	2577	2188	300	0	2405

TRINITY_DN5572_c0_g1_i1	Glyma.19G165500.3	98.50746269	178	7	675	2577	2188	300	0	2405

TRINITY_DN5572_c0_g1_i1	Glyma.19G165500.6	100	178	7	675	2577	2188	300	0	2405

TRINITY_DN8130_c0_g2_i1	Glyma.17G029800.1	95.79124579	176	23	108	1976	1	1881	0	2145

TRINITY_DN8130_c0_g2_i1	Glyma.07G244100.1	98.72881356	174	24	108	1976	1	1881	0	2156

TRINITY_DN13391_c0_g1_i2	Glyma.17G033400.1	100	164	7	45	1937	1	1902	0	2501

TRINITY_DN12444_c0_g1_i2	Glyma.02G288600.1	100	157	2	1	1906	1172	3074	0	2630

TRINITY_DN12444_c0_g1_i2	Glyma.02G288600.2	100	157	2	1	1906	914	2816	0	2630

TRINITY_DN12444_c0_g1_i2	Glyma.14G026300.1	97.02380952	155	2	1	1906	1172	3074	0	2641

TRINITY_DN10472_c0_g1_i3	Glyma.01G190800.1	100	152	9	151	2046	37	1938	0	2588

TRINITY_DN15396_c0_g1_i3	Glyma.10G086800.1	100	144	4	104	2009	1888	1	0	2606

TRINITY_DN15396_c0_g1_i3	Glyma.10G086800.3	98.38709677	144	4	104	2009	1888	1	0	2606

TRINITY_DN15396_c0_g1_i3	Glyma.02G188300.1	99.73890339	143	2	104	2009	1903	1	0	2708

TRINITY_DN15510_c0_g1_i5	Glyma.09G168600.2	97.33333333	124	2	132	2034	1	1903	0	2795

TRINITY_DN14410_c0_g2_i2	Glyma.06G211600.1	97.91666667	398	36	300	2179	67	1946	0	990

TRINITY_DN14245_c0_g2_i3	Glyma.13G241400.8	95.65217391	252	26	393	2268	14	1901	0	1825

TRINITY_DN14245_c0_g2_i3	Glyma.13G241400.5	97.33333333	252	26	393	2268	14	1901	0	1825

TRINITY_DN15918_c0_g1_i2	Glyma.18G268900.2	97.69736842	232	4	506	2395	6	1904	0	2097

TRINITY_DN15918_c0_g1_i3	Glyma.18G268900.2	96.70781893	232	4	787	2676	6	1904	0	2097

TRINITY_DN11081_c0_g2_i1	Glyma.17G078000.1	98.68421053	181	0	192	2096	37	1941	0	2516

TRINITY_DN9499_c0_g2_i1	Glyma.13G217900.1	100	163	1	388	2289	1905	1	0	2595

TRINITY_DN11811_c0_g1_i1	Glyma.01G068800.1	97.10144928	149	5	159	2051	1	1889	0	2512

TRINITY_DN16245_c0_g3_i2	Glyma.06G196400.3	98.7804878	147	8	378	2278	2990	1093	0	2632

TRINITY_DN15565_c0_g1_i1	Glyma.09G044300.4	98.7012987	146	3	714	2612	77	1969	0	2593

TRINITY_DN11811_c0_g1_i1	Glyma.02G125400.11	97.75280899	141	5	159	2051	1	1889	0	2556

TRINITY_DN11811_c0_g1_i1	Glyma.02G125400.5	97.72727273	141	5	159	2051	1	1889	0	2556

TRINITY_DN14238_c0_g2_i3	Glyma.15G066700.6	97.61904762	139	4	284	2183	1	1903	0	2702

TRINITY_DN14238_c0_g2_i3	Glyma.15G066700.1	97.61904762	139	4	284	2183	1	1903	0	2702

TRINITY_DN12711_c0_g1_i1	Glyma.20G209900.1	95.34883721	125	0	986	2890	1	1905	0	2826

TRINITY_DN11433_c0_g1_i2	Glyma.05G163800.1	98.47764034	122	3	484	2376	477	2381	0	2765

TRINITY_DN11433_c0_g1_i2	Glyma.05G163800.6	100	122	3	484	2376	474	2378	0	2765

TRINITY_DN11433_c0_g1_i2	Glyma.05G163800.7	98.42519685	122	3	484	2376	474	2378	0	2765

TRINITY_DN13645_c0_g1_i1	Glyma.19G225800.2	99.4047619	115	4	1466	3370	1899	1	0	2843

TRINITY_DN13645_c0_g1_i1	Glyma.19G225800.1	99.4047619	115	4	1466	3370	1899	1	0	2843

TRINITY_DN15327_c1_g1_i2	Glyma.02G272100.1	97.18670077	236	2	236	2138	1951	49	0	2196

TRINITY_DN15327_c1_g1_i4	Glyma.02G272100.1	100	210	2	236	2138	1951	49	0	2340

TRINITY_DN18954_c0_g1_i1	Glyma.15G164700.1	100	159	1	2	1905	1295	3195	0	2615

TRINITY_DN18954_c0_g1_i1	Glyma.15G164700.2	100	159	1	2	1905	1295	3195	0	2615

TRINITY_DN36295_c0_g1_i1	Glyma.16G208400.11	100	142	6	541	2436	1	1902	0	2665

TRINITY_DN36295_c0_g1_i1	Glyma.16G208400.13	96.96428571	142	6	541	2436	1	1902	0	2665

TRINITY_DN11383_c0_g1_i1	Glyma.02G133500.1	96.996997	133	4	248	2149	1902	1	0	2754

TRINITY_DN7659_c0_g1_i1	Glyma.19G191200.1	95	111	3	288	2185	1959	68	0	2785

TRINITY_DN142_c0_g1_i1	Glyma.09G249100.1	96.73202614	99	4	296	2195	131	2033	0	2935

TRINITY_DN12886_c0_g1_i2	Glyma.17G129200.1	96.08695652	97	2	846	2749	3396	1496	0	2959

TRINITY_DN12886_c0_g1_i2	Glyma.05G047400.1	96.09375	96	2	846	2749	3396	1496	0	2964

TRINITY_DN12886_c0_g1_i2	Glyma.05G047400.2	97	96	2	846	2749	3312	1412	0	2964

TRINITY_DN12519_c0_g1_i2	Glyma.20G017000.4	96.92832765	96	3	161	2058	1	1904	0	2946

TRINITY_DN7765_c0_g1_i1	Glyma.20G243700.1	99.49748744	95	0	103	2006	8	1911	0	2990

TRINITY_DN12519_c0_g1_i1	Glyma.20G017000.4	97.65013055	95	4	161	2042	1	1904	0	2848

TRINITY_DN14001_c0_g1_i1	Glyma.03G189800.1	98.85714286	230	34	186	2040	1922	38	0	1814

TRINITY_DN2833_c0_g1_i1	Glyma.11G244100.1	96.875	166	5	153	2043	1894	1	0	2459

TRINITY_DN15136_c0_g1_i2	Glyma.12G236000.1	100	138	5	313	2210	1911	14	0	2686

TRINITY_DN9142_c0_g1_i1	Glyma.12G018300.1	95.61403509	137	0	620	2522	1903	1	0	2756

TRINITY_DN6197_c0_g1_i1	Glyma.17G094300.1	100	129	1	1	1894	678	2580	0	2741

TRINITY_DN14359_c2_g1_i1	Glyma.18G301600.2	98.10606061	118	2	342	2244	1894	1	0	2802

TRINITY_DN14359_c2_g1_i1	Glyma.18G301600.3	100	118	2	342	2244	1894	1	0	2802

TRINITY_DN14359_c2_g1_i1	Glyma.18G301600.1	95.59082892	118	2	342	2244	1894	1	0	2802

TRINITY_DN14359_c2_g1_i2	Glyma.18G301600.2	98.6013986	118	2	294	2196	1894	1	0	2802

TRINITY_DN14359_c2_g1_i2	Glyma.18G301600.3	98.6013986	118	2	294	2196	1894	1	0	2802

TRINITY_DN14359_c2_g1_i2	Glyma.18G301600.1	100	118	2	294	2196	1894	1	0	2802

TRINITY_DN6285_c0_g1_i1	Glyma.12G236600.1	97.61904762	107	1	2	1904	1632	3531	0	2902

TRINITY_DN15659_c0_g2_i1	Glyma.07G184800.2	99.18032787	107	5	192	2086	2	1902	0	2857

TRINITY_DN15659_c0_g2_i1	Glyma.07G184800.1	99.18032787	107	5	192	2086	2	1902	0	2857

TRINITY_DN15659_c0_g2_i1	Glyma.07G184800.3	99.18032787	107	5	192	2086	2	1902	0	2857

TRINITY_DN15659_c0_g2_i3	Glyma.07G184800.2	98.39572193	107	5	115	2009	2	1902	0	2857

TRINITY_DN15659_c0_g2_i3	Glyma.07G184800.1	99.55947137	107	5	115	2009	2	1902	0	2857

TRINITY_DN15659_c0_g2_i3	Glyma.07G184800.3	95.92760181	107	5	115	2009	2	1902	0	2857

TRINITY_DN15659_c0_g2_i4	Glyma.07G184800.2	98.48484848	107	5	115	2009	2	1902	0	2857

TRINITY_DN15659_c0_g2_i7	Glyma.07G184800.2	95.59193955	107	5	192	2086	2	1902	0	2857

TRINITY_DN16382_c0_g3_i4	Glyma.18G060600.2	96.66666667	101	4	489	2391	184	2070	0	2852

TRINITY_DN12717_c0_g1_i1	Glyma.10G159000.2	100	266	17	100	1992	1	1869	0	1768

TRINITY_DN12717_c0_g1_i1	Glyma.10G159000.1	96.875	266	17	100	1992	1	1869	0	1768

TRINITY_DN16444_c0_g1_i7	Glyma.16G204200.1	98.46153846	178	6	339	2235	651	2549	0	2475

TRINITY_DN16444_c0_g1_i7	Glyma.16G204200.2	98.9010989	178	6	339	2235	558	2456	0	2475

TRINITY_DN16444_c0_g1_i7	Glyma.16G204200.3	97.91666667	178	6	339	2235	21	1919	0	2475

TRINITY_DN7177_c0_g1_i1	Glyma.20G226300.6	99.31034483	157	2	376	2276	1901	1	0	2630

TRINITY_DN7177_c0_g1_i1	Glyma.20G226300.1	99.31506849	157	2	376	2276	1901	1	0	2630

TRINITY_DN7177_c0_g1_i1	Glyma.20G226300.7	95.34883721	157	2	376	2276	1901	1	0	2630

TRINITY_DN7177_c0_g1_i1	Glyma.20G226300.3	100	157	2	376	2276	1901	1	0	2630

TRINITY_DN7177_c0_g1_i1	Glyma.20G226300.2	100	157	2	376	2276	1901	1	0	2630

TRINITY_DN7177_c0_g1_i1	Glyma.20G226300.5	98.21428571	157	2	376	2276	1901	1	0	2630

TRINITY_DN9499_c0_g2_i1	Glyma.15G094500.1	100	149	0	388	2289	1902	1	0	2687

TRINITY_DN6197_c0_g1_i1	Glyma.05G032300.1	100	140	3	5	1894	676	2577	0	2660

TRINITY_DN16115_c0_g2_i7	Glyma.12G074300.1	99.20634921	133	2	789	2681	2646	745	0	2717

TRINITY_DN12172_c0_g1_i1	Glyma.12G215200.3	95.11400651	123	1	589	2490	1899	1	0	2811

TRINITY_DN40621_c0_g1_i1	Glyma.03G090100.2	100	121	1	184	2085	1899	1	0	2822

TRINITY_DN15750_c1_g1_i6	Glyma.17G055100.2	99.06542056	286	9	550	2445	1944	49	0	1862

TRINITY_DN16231_c1_g2_i1	Glyma.17G045800.3	99.15966387	258	6	77	1974	1	1898	0	2043

TRINITY_DN16231_c1_g2_i1	Glyma.17G045800.2	99.15966387	258	6	77	1974	1	1898	0	2043

TRINITY_DN16231_c1_g2_i1	Glyma.17G045800.1	99.15966387	258	6	77	1974	1	1898	0	2043

TRINITY_DN12515_c0_g1_i1	Glyma.06G183400.1	100	252	18	166	2059	1890	12	0	1927

TRINITY_DN15750_c1_g1_i7	Glyma.17G055100.2	100	240	9	550	2445	1944	49	0	2117

TRINITY_DN13361_c0_g1_i1	Glyma.15G227100.1	98.48484848	209	14	102	1977	1	1891	0	2126

TRINITY_DN12830_c0_g2_i1	Glyma.08G071100.1	98.75	176	2	243	2140	190	2090	0	2516

TRINITY_DN7177_c0_g1_i1	Glyma.10G163700.1	100	146	0	376	2276	1901	1	0	2702

TRINITY_DN7177_c0_g1_i1	Glyma.10G163700.3	95.63636364	146	0	376	2276	1901	1	0	2702

TRINITY_DN7177_c0_g1_i1	Glyma.10G163700.2	97.76119403	146	0	376	2276	1901	1	0	2702

TRINITY_DN7177_c0_g1_i1	Glyma.10G163700.4	97.76119403	146	0	376	2276	1901	1	0	2702

TRINITY_DN14592_c0_g1_i1	Glyma.03G175300.1	95.56840077	416	43	486	2353	330	2201	0	817

TRINITY_DN9258_c1_g1_i2	Glyma.17G187200.2	100	295	11	589	2483	1913	22	0	1790

TRINITY_DN9258_c1_g1_i2	Glyma.17G187200.1	98.7654321	295	11	589	2483	1913	22	0	1790

TRINITY_DN9258_c1_g1_i2	Glyma.17G187200.3	96.66666667	295	11	589	2483	1913	22	0	1790

TRINITY_DN15365_c0_g1_i1	Glyma.16G109700.1	100	245	20	368	2241	1915	18	0	1971

TRINITY_DN15759_c0_g2_i1	Glyma.17G191300.1	97.6744186	242	8	1	1896	1990	101	0	2078

TRINITY_DN15759_c0_g2_i4	Glyma.17G191300.1	99.26470588	242	8	1	1896	1990	101	0	2078

TRINITY_DN15759_c0_g2_i1	Glyma.17G191300.2	98.79032258	239	15	1	1896	1978	101	0	2017

TRINITY_DN15759_c0_g2_i4	Glyma.17G191300.2	96.2585034	239	15	1	1896	1978	101	0	2017

TRINITY_DN13071_c1_g1_i2	Glyma.08G002100.1	97.48953975	220	9	63	1956	6	1902	0	2231

TRINITY_DN6758_c0_g1_i1	Glyma.15G034800.3	100	215	3	1	1897	1897	1	0	2279

TRINITY_DN6758_c0_g1_i1	Glyma.15G034800.2	99.59266802	215	3	1	1897	1897	1	0	2279

TRINITY_DN6758_c0_g1_i1	Glyma.15G034800.1	99.59349593	215	3	1	1897	1897	1	0	2279

TRINITY_DN16173_c1_g1_i3	Glyma.20G051500.2	99.06542056	211	20	408	2273	1875	1	0	1958

TRINITY_DN16173_c1_g1_i3	Glyma.20G051500.1	98.00995025	211	20	408	2273	1875	1	0	1958

TRINITY_DN12442_c0_g1_i1	Glyma.14G141800.3	96.33027523	169	4	216	2103	1	1897	0	2475

TRINITY_DN12442_c0_g1_i1	Glyma.14G141800.1	95.91836735	169	4	216	2103	1	1897	0	2475

TRINITY_DN12442_c0_g1_i1	Glyma.14G141800.2	95.91836735	169	4	216	2103	1	1897	0	2475

TRINITY_DN14457_c1_g1_i1	Glyma.03G123200.1	97.36842105	166	16	620	2471	433	2317	0	2182

TRINITY_DN13391_c0_g1_i2	Glyma.07G240000.1	97.10144928	165	9	45	1937	1	1893	0	2505

TRINITY_DN15781_c0_g1_i2	Glyma.06G188600.1	100	156	27	1	1851	317	2194	0	2185

TRINITY_DN17643_c0_g1_i1	Glyma.10G095200.1	95.95375723	143	5	1	1896	2331	433	0	2684

TRINITY_DN11665_c0_g3_i1	Glyma.06G092800.1	95.61170213	118	2	182	2080	1899	1	0	2843

TRINITY_DN11665_c0_g3_i1	Glyma.06G092800.2	98.9010989	118	2	182	2080	1899	1	0	2843

TRINITY_DN7765_c0_g1_i1	Glyma.10G293800.1	98.50746269	104	0	107	2006	12	1911	0	2933

TRINITY_DN13037_c0_g1_i1	Glyma.13G150000.1	98.41269841	267	12	1	1882	171	2067	0	1905

TRINITY_DN15970_c0_g2_i10	Glyma.18G188000.2	97.82608696	259	8	230	2124	31	1925	0	2021

TRINITY_DN15970_c0_g2_i4	Glyma.18G188000.2	100	259	8	230	2124	31	1925	0	2021

TRINITY_DN16173_c1_g1_i4	Glyma.20G051500.2	97.73869347	225	18	408	2273	1875	1	0	1892

TRINITY_DN16173_c1_g1_i4	Glyma.20G051500.1	97.74011299	225	18	408	2273	1875	1	0	1892

TRINITY_DN16173_c1_g1_i6	Glyma.20G051500.2	100	207	17	408	2276	1875	1	0	2012

TRINITY_DN16173_c1_g1_i6	Glyma.20G051500.1	100	207	17	408	2276	1875	1	0	2012

TRINITY_DN13985_c0_g1_i1	Glyma.09G225000.1	95.77464789	192	14	283	2167	31	1921	0	2302

TRINITY_DN6249_c0_g1_i1	Glyma.01G211900.1	100	186	2	1	1898	150	2047	0	2464

TRINITY_DN6249_c0_g1_i1	Glyma.01G211900.2	96.42857143	186	2	1	1898	48	1945	0	2464

TRINITY_DN12830_c0_g2_i1	Glyma.13G043600.1	98.75	186	5	243	2140	175	2060	0	2386

TRINITY_DN14801_c0_g1_i1	Glyma.06G115600.1	100	177	8	171	2057	1896	1	0	2429

TRINITY_DN37356_c0_g1_i1	Glyma.16G131900.1	98.71794872	171	7	35	1923	1	1886	0	2410

TRINITY_DN15687_c0_g1_i1	Glyma.15G235600.1	97.73462783	142	8	1672	3569	515	2400	0	2630

TRINITY_DN6197_c0_g1_i1	Glyma.05G032300.2	98.66666667	141	1	5	1894	676	2574	0	2667

TRINITY_DN15143_c0_g2_i2	Glyma.09G187400.3	95.86614173	134	6	402	2300	1	1884	0	2667

TRINITY_DN15143_c0_g2_i4	Glyma.09G187400.3	99.40828402	134	6	444	2342	1	1884	0	2667

TRINITY_DN11833_c0_g1_i2	Glyma.20G241400.1	95.57522124	129	1	177	2072	1	1899	0	2772

TRINITY_DN6285_c0_g1_i1	Glyma.13G202100.1	97.69911504	106	1	2	1900	1632	3527	0	2900

TRINITY_DN14245_c0_g2_i1	Glyma.15G072000.2	95.65217391	275	16	451	2323	14	1898	0	1736

TRINITY_DN16257_c1_g1_i10	Glyma.12G192000.1	95.32467532	246	5	102	1997	2239	345	0	2109

TRINITY_DN15450_c1_g1_i1	Glyma.01G136500.1	99.59677419	246	6	280	2174	2174	280	0	2104

TRINITY_DN15450_c1_g1_i3	Glyma.01G136500.1	100	246	6	227	2121	2174	280	0	2104

TRINITY_DN15450_c1_g1_i1	Glyma.03G031700.1	100	243	6	280	2174	2162	268	0	2121

TRINITY_DN15450_c1_g1_i3	Glyma.03G031700.1	100	243	6	227	2121	2162	268	0	2121

TRINITY_DN15365_c0_g2_i1	Glyma.16G109700.1	99.81238274	175	21	333	2204	1913	18	0	2355

TRINITY_DN13645_c0_g1_i1	Glyma.03G228500.1	95.54973822	159	9	1473	3370	1886	1	0	2547

TRINITY_DN13056_c1_g1_i1	Glyma.20G024500.1	96.25	159	13	96	1963	18	1912	0	2410

TRINITY_DN13056_c1_g1_i1	Glyma.20G024500.2	96.25	159	13	96	1963	18	1912	0	2410

TRINITY_DN13056_c1_g1_i3	Glyma.20G024500.1	96.25	159	13	90	1957	18	1912	0	2410

TRINITY_DN13056_c1_g1_i3	Glyma.20G024500.2	96.25	159	13	90	1957	18	1912	0	2410

TRINITY_DN13353_c0_g1_i1	Glyma.03G243700.1	96	157	1	299	2196	219	2115	0	2628

TRINITY_DN16268_c1_g2_i5	Glyma.17G161500.4	95.07874016	157	13	559	2433	1890	1	0	2435

TRINITY_DN16268_c1_g2_i5	Glyma.17G161500.2	97.87234043	157	13	559	2433	1890	1	0	2435

TRINITY_DN16268_c1_g2_i5	Glyma.17G161500.3	97.87234043	157	13	559	2433	1890	1	0	2435

TRINITY_DN16268_c1_g2_i5	Glyma.17G161500.1	95.87628866	157	13	559	2433	1890	1	0	2435

TRINITY_DN16268_c1_g2_i3	Glyma.17G161500.4	98.86363636	151	20	559	2427	1890	1	0	2429

TRINITY_DN16268_c1_g2_i3	Glyma.17G161500.2	98.86363636	151	20	559	2427	1890	1	0	2429

TRINITY_DN16268_c1_g2_i3	Glyma.17G161500.3	98.86363636	151	20	559	2427	1890	1	0	2429

TRINITY_DN16268_c1_g2_i3	Glyma.17G161500.1	98.86363636	151	20	559	2427	1890	1	0	2429

TRINITY_DN16231_c1_g2_i1	Glyma.09G073600.4	95.89442815	138	0	77	1974	1	1898	0	2741

TRINITY_DN16231_c1_g2_i1	Glyma.09G073600.3	100	138	0	77	1974	1	1898	0	2741

TRINITY_DN16231_c1_g2_i1	Glyma.09G073600.5	96.84210526	138	0	77	1974	1	1898	0	2741

TRINITY_DN16231_c1_g2_i1	Glyma.09G073600.2	95.12195122	138	0	77	1974	1	1898	0	2741

TRINITY_DN16231_c1_g2_i1	Glyma.09G073600.1	95.18304432	138	0	77	1974	1	1898	0	2741

TRINITY_DN12978_c0_g1_i2	Glyma.19G113600.3	95.41420118	136	3	100	1997	1	1889	0	2693

TRINITY_DN12978_c0_g1_i2	Glyma.19G113600.2	100	136	3	100	1997	1	1889	0	2693

TRINITY_DN12036_c0_g1_i4	Glyma.12G010000.1	98.96907216	135	5	141	2038	4	1889	0	2680

TRINITY_DN16231_c1_g2_i1	Glyma.15G182600.2	95.93495935	134	0	77	1974	1	1898	0	2763

TRINITY_DN16231_c1_g2_i1	Glyma.15G182600.1	96.53061224	134	0	77	1974	1	1898	0	2763

TRINITY_DN12978_c0_g1_i1	Glyma.19G113600.3	100	134	3	104	2001	1	1889	0	2704

TRINITY_DN12978_c0_g1_i1	Glyma.19G113600.2	95.97069597	134	3	104	2001	1	1889	0	2704

TRINITY_DN4033_c0_g1_i1	Glyma.11G123500.1	97.58064516	117	3	808	2705	11283	9395	0	2798

TRINITY_DN4033_c0_g1_i1	Glyma.11G123500.2	97.92899408	117	3	808	2705	11283	9395	0	2798

TRINITY_DN9308_c0_g1_i1	Glyma.01G159900.1	96.12403101	112	0	77	1974	1898	1	0	2885

TRINITY_DN2996_c0_g1_i1	Glyma.12G057600.1	96.34146341	109	5	1	1894	6	1899	0	2850

TRINITY_DN15594_c0_g1_i3	Glyma.01G203100.1	100	106	1	558	2452	2670	773	0	2898

TRINITY_DN15594_c0_g1_i3	Glyma.01G203100.2	95.57195572	106	1	558	2452	2670	773	0	2898

TRINITY_DN13088_c0_g1_i2	Glyma.09G143700.1	100	329	22	595	2466	89	1978	0	1474

TRINITY_DN15028_c0_g1_i3	Glyma.05G198500.1	96.2406015	304	36	1328	3183	2032	153	0	1445

TRINITY_DN15970_c0_g2_i10	Glyma.07G137300.2	98.30508475	259	4	230	2124	166	2060	0	2043

TRINITY_DN15970_c0_g2_i4	Glyma.07G137300.2	97.07317073	259	4	230	2124	166	2060	0	2043

TRINITY_DN15365_c0_g1_i1	Glyma.16G076700.1	100	250	22	370	2241	1910	18	0	1930

TRINITY_DN14031_c0_g1_i1	Glyma.07G168900.2	95.44159544	236	6	62	1954	128	2019	0	2137

TRINITY_DN16509_c0_g1_i1	Glyma.02G022700.1	100	230	11	175	2064	1884	1	0	2100

TRINITY_DN9389_c0_g1_i2	Glyma.14G083400.1	98.31460674	203	5	112	2004	257	2150	0	2333

TRINITY_DN9389_c0_g1_i1	Glyma.14G083400.1	100	202	13	112	1986	257	2150	0	2222

TRINITY_DN38438_c0_g1_i1	Glyma.18G074700.1	100	187	11	209	2098	96	1986	0	2383

TRINITY_DN15365_c0_g2_i1	Glyma.16G076700.1	99.81238274	183	23	333	2204	1910	18	0	2302

TRINITY_DN14740_c0_g1_i3	Glyma.16G133400.1	96.66666667	175	3	246	2139	1	1894	0	2495

TRINITY_DN14740_c0_g1_i4	Glyma.16G133400.1	99.03846154	175	3	246	2139	1	1894	0	2495

TRINITY_DN14740_c0_g1_i5	Glyma.16G133400.1	96.95121951	175	3	246	2139	1	1894	0	2495

TRINITY_DN16268_c1_g2_i5	Glyma.17G161600.1	97.43589744	166	11	559	2433	1893	1	0	2416

TRINITY_DN14801_c0_g1_i1	Glyma.04G247200.1	100	160	7	171	2057	1896	1	0	2545

TRINITY_DN12711_c0_g1_i1	Glyma.10G180600.1	100	130	3	986	2880	1	1895	0	2758

TRINITY_DN2716_c0_g1_i1	Glyma.13G094800.1	100	120	10	450	2342	1896	19	0	2689

TRINITY_DN12073_c0_g2_i2	Glyma.16G012300.1	97.01492537	103	13	346	2234	2439	563	0	2752

TRINITY_DN14369_c1_g3_i1	Glyma.20G133800.1	97.94520548	102	3	333	2225	1	1896	0	2905

TRINITY_DN13088_c0_g1_i2	Glyma.16G196700.1	100	324	21	596	2466	90	1972	0	1461

TRINITY_DN16308_c1_g3_i2	Glyma.20G046200.1	100	304	16	132	2019	1	1885	0	1694

TRINITY_DN13037_c0_g1_i1	Glyma.10G065200.1	100	272	16	1	1882	324	2208	0	1832

TRINITY_DN12077_c1_g1_i1	Glyma.20G183100.1	97.02970297	248	11	94	1983	1887	1	0	2030

TRINITY_DN12077_c1_g1_i2	Glyma.20G183100.1	97.00598802	248	11	430	2319	1887	1	0	2030

TRINITY_DN6205_c0_g2_i1	Glyma.03G203900.3	95.18072289	214	36	70	1929	1854	1	0	1812

TRINITY_DN6205_c0_g2_i1	Glyma.03G203900.1	95.19230769	214	36	70	1929	1854	1	0	1812

TRINITY_DN6205_c0_g2_i1	Glyma.03G203900.2	95.19230769	214	36	70	1929	1854	1	0	1812

TRINITY_DN6205_c0_g1_i1	Glyma.03G203900.3	95.27896996	213	36	70	1929	1854	1	0	1818

TRINITY_DN6205_c0_g1_i1	Glyma.03G203900.1	95.23809524	213	36	70	1929	1854	1	0	1818

TRINITY_DN6205_c0_g1_i1	Glyma.03G203900.2	95.27559055	213	36	70	1929	1854	1	0	1818

TRINITY_DN4296_c0_g1_i1	Glyma.20G181000.1	100	199	7	1553	3440	3549	1658	0	2322

TRINITY_DN16181_c0_g1_i3	Glyma.17G248400.5	96.2962963	167	2	890	2784	2008	114	0	2564

TRINITY_DN9205_c0_g1_i2	Glyma.07G148100.23	97.33840304	167	6	830	2709	1895	1	0	2466

TRINITY_DN9205_c0_g1_i2	Glyma.07G148100.3	95.625	167	6	830	2709	1895	1	0	2466

TRINITY_DN9205_c0_g1_i3	Glyma.07G148100.23	96.21212121	167	6	973	2852	1895	1	0	2466

TRINITY_DN9205_c0_g1_i3	Glyma.07G148100.3	100	167	6	973	2852	1895	1	0	2466

TRINITY_DN9205_c0_g1_i4	Glyma.07G148100.23	100	167	6	734	2613	1895	1	0	2466

TRINITY_DN9205_c0_g1_i4	Glyma.07G148100.3	100	167	6	734	2613	1895	1	0	2466

TRINITY_DN13868_c0_g1_i1	Glyma.15G076900.1	100	165	0	171	2066	1	1896	0	2588

TRINITY_DN13868_c0_g1_i1	Glyma.15G076900.3	100	165	0	171	2066	1	1896	0	2588

TRINITY_DN13868_c0_g1_i3	Glyma.15G076900.1	97.07446809	165	0	171	2066	1	1896	0	2588

TRINITY_DN13868_c0_g1_i3	Glyma.15G076900.3	99.20634921	165	0	171	2066	1	1896	0	2588

TRINITY_DN13868_c0_g1_i6	Glyma.15G076900.1	97.36842105	165	0	171	2066	1	1896	0	2588

TRINITY_DN13868_c0_g1_i6	Glyma.15G076900.3	97.36842105	165	0	171	2066	1	1896	0	2588

TRINITY_DN13868_c0_g1_i8	Glyma.15G076900.1	97.86476868	165	0	171	2066	1	1896	0	2588

TRINITY_DN13868_c0_g1_i8	Glyma.15G076900.3	98.39816934	165	0	171	2066	1	1896	0	2588

TRINITY_DN9205_c0_g1_i2	Glyma.07G148100.22	95.93495935	165	12	830	2709	1871	1	0	2322

TRINITY_DN9205_c0_g1_i2	Glyma.07G148100.2	98.82352941	165	12	830	2709	1871	1	0	2322

TRINITY_DN9205_c0_g1_i3	Glyma.07G148100.22	96.73590504	165	12	973	2852	1871	1	0	2322

TRINITY_DN9205_c0_g1_i3	Glyma.07G148100.2	100	165	12	973	2852	1871	1	0	2322

TRINITY_DN9205_c0_g1_i4	Glyma.07G148100.22	97.27891156	165	12	734	2613	1871	1	0	2322

TRINITY_DN9205_c0_g1_i4	Glyma.07G148100.2	99.33554817	165	12	734	2613	1871	1	0	2322

TRINITY_DN14457_c1_g1_i1	Glyma.19G126800.1	97.36842105	162	15	618	2471	464	2344	0	2235

TRINITY_DN20587_c0_g1_i1	Glyma.11G212300.1	95.70200573	160	13	209	2086	3225	1342	0	2422

TRINITY_DN16268_c1_g2_i5	Glyma.05G105000.3	97.76357827	160	15	559	2433	1875	1	0	2344

TRINITY_DN16268_c1_g2_i5	Glyma.05G105000.1	99.44751381	160	15	559	2433	1875	1	0	2344

TRINITY_DN16268_c1_g2_i5	Glyma.05G105000.2	98.97959184	160	15	559	2433	1875	1	0	2344

TRINITY_DN16268_c1_g2_i3	Glyma.05G105000.3	98.86363636	157	18	559	2427	1875	1	0	2322

TRINITY_DN16268_c1_g2_i3	Glyma.05G105000.1	98.86363636	157	18	559	2427	1875	1	0	2322

TRINITY_DN16268_c1_g2_i3	Glyma.05G105000.2	98.86363636	157	18	559	2427	1875	1	0	2322

TRINITY_DN16268_c1_g2_i3	Glyma.17G161600.1	98.86363636	155	11	559	2427	1893	1	0	2449

TRINITY_DN12435_c0_g1_i1	Glyma.05G208300.1	99.62264151	154	1	191	2080	1	1896	0	2610

TRINITY_DN12659_c0_g1_i1	Glyma.06G238000.10	98.49435383	144	3	164	2055	1	1895	0	2671

TRINITY_DN12659_c0_g1_i1	Glyma.06G238000.9	98.50187266	144	3	164	2055	1	1895	0	2671

TRINITY_DN12659_c0_g1_i1	Glyma.06G238000.14	98.4962406	144	3	164	2055	1	1895	0	2671

TRINITY_DN12659_c0_g1_i1	Glyma.06G238000.5	98.48771267	144	3	164	2055	1	1895	0	2671

TRINITY_DN3342_c0_g1_i1	Glyma.16G015800.2	97.01492537	137	2	111	1994	1	1893	0	2645

TRINITY_DN3342_c0_g1_i1	Glyma.16G015800.3	97.95918367	137	2	111	1994	1	1893	0	2645

TRINITY_DN3342_c0_g1_i1	Glyma.16G015800.1	95.4954955	137	2	111	1994	1	1893	0	2645

TRINITY_DN13868_c0_g1_i10	Glyma.15G076900.1	95.02840909	126	0	171	2066	1	1896	0	2804

TRINITY_DN13868_c0_g1_i10	Glyma.15G076900.3	96.86985173	126	0	171	2066	1	1896	0	2804

TRINITY_DN13868_c0_g1_i4	Glyma.15G076900.1	97.61904762	126	0	171	2066	1	1896	0	2804

TRINITY_DN13868_c0_g1_i4	Glyma.15G076900.3	100	126	0	171	2066	1	1896	0	2804

TRINITY_DN13868_c0_g1_i5	Glyma.15G076900.1	99.609375	126	0	171	2066	1	1896	0	2804

TRINITY_DN13868_c0_g1_i5	Glyma.15G076900.3	100	126	0	171	2066	1	1896	0	2804

TRINITY_DN13868_c0_g1_i9	Glyma.15G076900.1	97.43589744	126	0	171	2066	1	1896	0	2804

TRINITY_DN13868_c0_g1_i9	Glyma.15G076900.3	97.78481013	126	0	171	2066	1	1896	0	2804

TRINITY_DN5503_c0_g1_i4	Glyma.20G182600.1	100	125	1	305	2197	1	1896	0	2789

TRINITY_DN3800_c0_g1_i1	Glyma.13G160800.1	96.25360231	124	14	76	1956	24	1892	0	2543

TRINITY_DN11099_c0_g1_i1	Glyma.13G001500.4	97.88359788	366	13	3	1892	127	2007	0	1349

TRINITY_DN11099_c0_g1_i1	Glyma.13G001500.1	97.99270073	366	13	3	1892	127	2007	0	1349

TRINITY_DN14680_c0_g1_i1	Glyma.19G175600.4	97.36842105	337	30	3	1864	549	2429	0	1328

TRINITY_DN14680_c0_g1_i1	Glyma.19G175600.3	96.72131148	337	30	3	1864	549	2429	0	1328

TRINITY_DN12077_c1_g1_i1	Glyma.10G207700.1	97.00598802	259	11	94	1983	1887	1	0	1980

TRINITY_DN12077_c1_g1_i2	Glyma.10G207700.1	97.01492537	259	11	430	2319	1887	1	0	1980

TRINITY_DN16027_c1_g3_i5	Glyma.16G005700.1	100	211	15	385	2244	1887	1	0	2052

TRINITY_DN16027_c1_g3_i7	Glyma.16G005700.1	97.9757085	211	15	385	2244	1887	1	0	2052

TRINITY_DN15048_c0_g2_i2	Glyma.15G145200.4	95.53571429	208	8	647	2539	1947	61	0	2283

TRINITY_DN15048_c0_g2_i2	Glyma.15G145200.3	95.53571429	208	8	647	2539	1947	61	0	2283

TRINITY_DN15048_c0_g2_i2	Glyma.15G145200.2	98.41269841	208	8	647	2539	1947	61	0	2283

TRINITY_DN15968_c0_g1_i1	Glyma.16G051300.1	100	201	7	266	2152	143	2032	0	2302

TRINITY_DN11811_c0_g1_i1	Glyma.02G125400.1	97.10144928	143	5	159	2040	1	1883	0	2545

TRINITY_DN11811_c0_g1_i1	Glyma.02G125400.9	97.11538462	143	5	159	2040	1	1883	0	2545

TRINITY_DN11811_c0_g2_i1	Glyma.02G125400.1	97.10144928	140	5	159	2040	1	1883	0	2562

TRINITY_DN11811_c0_g2_i1	Glyma.02G125400.9	97.11286089	140	5	159	2040	1	1883	0	2562

TRINITY_DN11811_c0_g2_i2	Glyma.02G125400.1	97.11538462	140	5	159	2040	1	1883	0	2562

TRINITY_DN11811_c0_g2_i2	Glyma.02G125400.9	97.10144928	140	5	159	2040	1	1883	0	2562

TRINITY_DN14369_c1_g1_i1	Glyma.01G242800.1	97.22222222	140	8	71	1962	47	1932	0	2647

TRINITY_DN16181_c0_g1_i1	Glyma.17G248400.5	95.38188277	133	0	890	2784	2008	114	0	2763

TRINITY_DN15970_c0_g2_i10	Glyma.01G099800.2	97.2972973	125	0	230	2124	31	1925	0	2808

TRINITY_DN15970_c0_g2_i4	Glyma.01G099800.2	100	125	0	230	2124	31	1925	0	2808

TRINITY_DN36159_c0_g1_i1	Glyma.15G106800.1	100	115	2	356	2249	1	1894	0	2850

TRINITY_DN12073_c0_g2_i2	Glyma.07G043900.1	97.01492537	112	8	346	2234	2358	482	0	2724

TRINITY_DN36159_c0_g1_i1	Glyma.13G205700.1	100	110	2	356	2249	1	1894	0	2878

TRINITY_DN8360_c0_g2_i1	Glyma.08G324500.1	96.80851064	451	51	355	2206	123	1992	6.16E-162	573

TRINITY_DN11099_c0_g1_i1	Glyma.13G001500.2	97.84482759	367	14	3	1892	127	2004	0	1336

TRINITY_DN11099_c0_g1_i1	Glyma.13G001500.3	99.07407407	367	14	3	1892	127	2004	0	1336

TRINITY_DN14238_c0_g2_i1	Glyma.15G066700.6	97.61904762	271	22	189	2062	21	1903	0	1796

TRINITY_DN14238_c0_g2_i1	Glyma.15G066700.1	98.7012987	271	22	189	2062	21	1903	0	1796

TRINITY_DN16027_c1_g3_i5	Glyma.16G005700.3	95.45454545	210	17	385	2244	1881	1	0	2030

TRINITY_DN16027_c1_g3_i5	Glyma.16G005700.8	95.45454545	210	17	385	2244	1881	1	0	2030

TRINITY_DN16027_c1_g3_i7	Glyma.16G005700.3	97.97752809	210	17	385	2244	1881	1	0	2030

TRINITY_DN16027_c1_g3_i7	Glyma.16G005700.8	97.97752809	210	17	385	2244	1881	1	0	2030

TRINITY_DN15594_c0_g1_i3	Glyma.17G151100.1	98.71794872	204	1	562	2452	2657	764	0	2348

TRINITY_DN15594_c0_g1_i3	Glyma.05G068600.1	95	201	1	562	2452	2654	761	0	2364

TRINITY_DN4296_c0_g1_i1	Glyma.10G209600.2	100	193	6	1553	3440	3546	1655	0	2377

TRINITY_DN4296_c0_g1_i1	Glyma.10G209600.1	96.95740365	193	6	1553	3440	3546	1655	0	2377

TRINITY_DN16274_c0_g2_i1	Glyma.12G095300.2	96.20689655	192	4	1	1893	1103	2986	0	2362

TRINITY_DN14204_c0_g1_i4	Glyma.11G067800.1	100	159	2	145	2037	1	1893	0	2604

TRINITY_DN16268_c1_g2_i2	Glyma.17G161500.4	96.52406417	147	11	559	2430	1890	1	0	2516

TRINITY_DN16268_c1_g2_i2	Glyma.17G161500.2	100	147	11	559	2430	1890	1	0	2516

TRINITY_DN16268_c1_g2_i2	Glyma.17G161500.3	100	147	11	559	2430	1890	1	0	2516

TRINITY_DN16268_c1_g2_i2	Glyma.17G161500.1	100	147	11	559	2430	1890	1	0	2516

TRINITY_DN16268_c1_g2_i10	Glyma.17G161500.4	98.26589595	141	18	559	2424	1890	1	0	2510

TRINITY_DN16268_c1_g2_i10	Glyma.17G161500.2	98.10246679	141	18	559	2424	1890	1	0	2510

TRINITY_DN16268_c1_g2_i10	Glyma.17G161500.3	97.05882353	141	18	559	2424	1890	1	0	2510

TRINITY_DN16268_c1_g2_i10	Glyma.17G161500.1	97.39776952	141	18	559	2424	1890	1	0	2510

TRINITY_DN11855_c0_g1_i1	Glyma.17G063500.1	97.56097561	137	4	224	2113	1	1893	0	2706

TRINITY_DN2636_c0_g1_i1	Glyma.02G032800.1	95.98662207	133	9	1	1888	1922	38	0	2663

TRINITY_DN14204_c0_g1_i3	Glyma.11G067800.1	100	130	2	145	2037	1	1893	0	2765

TRINITY_DN3800_c0_g1_i1	Glyma.17G110600.1	98.7012987	113	4	76	1957	24	1916	0	2787

TRINITY_DN12073_c0_g2_i2	Glyma.16G012300.2	97.01492537	104	11	346	2234	2436	563	0	2760

TRINITY_DN14369_c1_g3_i1	Glyma.10G257300.1	97.94520548	103	1	333	2223	1	1894	0	2907

TRINITY_DN11099_c0_g1_i1	Glyma.20G068300.1	97.02970297	356	10	3	1892	130	2010	0	1426

TRINITY_DN13054_c0_g1_i1	Glyma.15G067700.2	98.21428571	272	12	594	2466	1903	19	0	1808

TRINITY_DN13054_c0_g1_i1	Glyma.15G067700.1	97.5	272	12	594	2466	1903	19	0	1808

TRINITY_DN14238_c0_g2_i1	Glyma.13G247300.3	97.61904762	272	20	189	2062	27	1909	0	1801

TRINITY_DN14238_c0_g2_i1	Glyma.13G247300.1	97.61904762	272	20	189	2062	27	1909	0	1801

TRINITY_DN2154_c0_g1_i1	Glyma.03G121500.1	100	203	0	198	2090	1893	1	0	2372

TRINITY_DN13377_c0_g1_i4	Glyma.15G100500.6	95.83333333	179	21	591	2453	155	2035	0	2233

TRINITY_DN13377_c0_g1_i5	Glyma.15G100500.6	97.82608696	179	21	386	2248	155	2035	0	2233

TRINITY_DN3991_c0_g1_i1	Glyma.18G030600.2	99.5951417	169	1	113	2005	1	1890	0	2540

TRINITY_DN3991_c0_g1_i1	Glyma.18G030600.1	100	169	1	113	2005	1	1890	0	2540

TRINITY_DN3991_c0_g1_i1	Glyma.11G226600.2	96.21212121	165	1	113	2005	1	1890	0	2562

TRINITY_DN3991_c0_g1_i1	Glyma.11G226600.1	98.53658537	165	1	113	2005	1	1890	0	2562

TRINITY_DN16268_c1_g2_i2	Glyma.17G161600.1	100	160	7	559	2430	1893	1	0	2473

TRINITY_DN14204_c0_g1_i4	Glyma.01G175600.1	95.12195122	155	0	145	2037	1	1893	0	2638

TRINITY_DN12435_c0_g1_i1	Glyma.08G014900.1	99.62264151	153	1	191	2080	1	1893	0	2628

TRINITY_DN11811_c0_g1_i1	Glyma.01G068800.2	97.11538462	149	4	159	2039	1	1881	0	2516

TRINITY_DN16268_c1_g2_i10	Glyma.17G161600.1	97.4025974	147	9	559	2424	1893	1	0	2507

TRINITY_DN11811_c0_g2_i1	Glyma.01G068800.2	95.55555556	146	4	159	2039	1	1881	0	2532

TRINITY_DN11811_c0_g2_i2	Glyma.01G068800.2	97.10144928	146	4	159	2039	1	1881	0	2532

TRINITY_DN12659_c0_g1_i1	Glyma.06G238000.8	98.49246231	144	2	164	2055	1	1892	0	2686

TRINITY_DN12659_c0_g1_i1	Glyma.06G238000.4	98.50746269	144	2	164	2055	1	1892	0	2686

TRINITY_DN12659_c0_g1_i1	Glyma.06G238000.13	98.49056604	144	2	164	2055	1	1892	0	2686

TRINITY_DN11811_c0_g1_i1	Glyma.02G125400.7	97.75280899	141	4	159	2039	1	1881	0	2560

TRINITY_DN11811_c0_g1_i1	Glyma.02G125400.2	97.72727273	141	4	159	2039	1	1881	0	2560

TRINITY_DN11811_c0_g1_i1	Glyma.02G125400.3	97.74774775	141	4	159	2039	1	1881	0	2560

TRINITY_DN11855_c0_g1_i1	Glyma.20G083600.1	97.56097561	140	2	224	2113	1	1893	0	2700

TRINITY_DN11811_c0_g2_i1	Glyma.02G125400.7	97.10234279	138	4	159	2039	1	1881	0	2577

TRINITY_DN11811_c0_g2_i1	Glyma.02G125400.2	97.11538462	138	4	159	2039	1	1881	0	2577

TRINITY_DN11811_c0_g2_i1	Glyma.02G125400.3	97.10144928	138	4	159	2039	1	1881	0	2577

TRINITY_DN11811_c0_g2_i2	Glyma.02G125400.7	97.11538462	138	4	159	2039	1	1881	0	2577

TRINITY_DN11811_c0_g2_i2	Glyma.02G125400.2	97.11538462	138	4	159	2039	1	1881	0	2577

TRINITY_DN11811_c0_g2_i2	Glyma.02G125400.3	97.10144928	138	4	159	2039	1	1881	0	2577

TRINITY_DN11433_c0_g1_i2	Glyma.08G121100.6	98.47908745	137	1	484	2376	474	2363	0	2717

TRINITY_DN11433_c0_g1_i2	Glyma.08G121100.1	98.7654321	137	1	484	2376	474	2363	0	2717

TRINITY_DN11433_c0_g1_i2	Glyma.08G121100.3	98.47826087	137	1	484	2376	294	2183	0	2717

TRINITY_DN16115_c0_g2_i7	Glyma.U033900.2	95.11400651	131	0	789	2681	2667	775	0	2771

TRINITY_DN16115_c0_g2_i7	Glyma.U033900.3	96.22641509	131	0	789	2681	2667	775	0	2771

TRINITY_DN16115_c0_g2_i7	Glyma.U033900.1	100	131	0	789	2681	2667	775	0	2771

TRINITY_DN14204_c0_g1_i3	Glyma.01G175600.1	95.09202454	122	0	145	2037	1	1893	0	2820

TRINITY_DN2716_c0_g1_i1	Glyma.17G065500.1	100	109	13	454	2342	1880	19	0	2665

TRINITY_DN2716_c0_g1_i1	Glyma.17G065500.2	96.50655022	109	13	454	2342	1880	19	0	2665

TRINITY_DN11393_c0_g1_i1	Glyma.17G129100.4	95.21126761	108	0	719	2611	1893	1	0	2898

TRINITY_DN9308_c0_g1_i1	Glyma.11G084200.1	96.12590799	108	1	82	1974	1890	1	0	2878

TRINITY_DN15748_c0_g1_i2	Glyma.08G283700.5	99.43661972	102	1	2006	3898	2717	828	0	2911

TRINITY_DN15748_c0_g1_i3	Glyma.08G283700.5	95.55555556	102	1	1363	3255	2717	828	0	2911

TRINITY_DN16444_c0_g1_i7	Glyma.13G159800.1	100	326	26	359	2234	821	2686	0	1417

TRINITY_DN12515_c0_g1_i1	Glyma.04G182300.1	100	273	15	173	2056	1889	12	0	1840

TRINITY_DN15878_c0_g2_i1	Glyma.19G260900.3	98.80239521	267	39	60	1926	1855	1	0	1615

TRINITY_DN15878_c0_g2_i1	Glyma.19G260900.2	100	267	39	60	1926	1855	1	0	1615

TRINITY_DN14245_c0_g2_i3	Glyma.15G072000.2	95.65217391	264	11	393	2268	14	1898	0	1882

TRINITY_DN5901_c0_g2_i1	Glyma.02G087000.6	99.43181818	262	15	1062	2951	1	1866	0	1862

TRINITY_DN14238_c0_g2_i3	Glyma.08G060100.1	97.62470309	256	17	303	2185	257	2133	0	1921

TRINITY_DN16257_c1_g1_i9	Glyma.12G192000.1	98.93617021	235	15	192	2071	2220	345	0	2012

TRINITY_DN5901_c0_g1_i1	Glyma.02G087000.6	100	235	15	1062	2951	1	1866	0	2012

TRINITY_DN13377_c0_g1_i1	Glyma.15G100500.7	95.82089552	179	21	552	2413	155	2034	0	2231

TRINITY_DN13377_c0_g1_i2	Glyma.15G100500.7	95.32163743	179	21	591	2452	155	2034	0	2231

TRINITY_DN13377_c0_g1_i3	Glyma.15G100500.7	96.1352657	179	21	386	2247	155	2034	0	2231

TRINITY_DN13377_c0_g1_i4	Glyma.15G100500.7	98.03921569	179	21	591	2452	155	2034	0	2231

TRINITY_DN13377_c0_g1_i5	Glyma.15G100500.7	96.01677149	179	21	386	2247	155	2034	0	2231

TRINITY_DN6085_c0_g1_i1	Glyma.08G217500.1	97.33333333	160	4	225	2114	1890	1	0	2582

TRINITY_DN6085_c0_g1_i1	Glyma.08G217500.3	97.33009709	160	4	225	2114	1890	1	0	2582

TRINITY_DN6085_c0_g1_i1	Glyma.08G217500.2	97.34513274	160	4	225	2114	1890	1	0	2582

TRINITY_DN13039_c0_g2_i1	Glyma.02G303300.1	100	152	1	139	2024	1	1892	0	2614

TRINITY_DN5733_c0_g1_i1	Glyma.16G143200.1	98.55072464	152	5	335	2211	2055	164	0	2555

TRINITY_DN6085_c0_g1_i1	Glyma.07G024100.3	97.32142857	149	4	225	2114	1890	1	0	2643

TRINITY_DN6085_c0_g1_i1	Glyma.07G024100.1	97.33487833	149	4	225	2114	1890	1	0	2643

TRINITY_DN6085_c0_g1_i1	Glyma.07G024100.2	97.32540862	149	4	225	2114	1890	1	0	2643

TRINITY_DN13039_c0_g2_i1	Glyma.14G010500.1	96	131	2	139	2024	1	1892	0	2730

TRINITY_DN15659_c0_g2_i1	Glyma.08G064600.1	97.2972973	123	4	202	2086	12	1902	0	2761

TRINITY_DN15659_c0_g2_i3	Glyma.08G064600.1	96.20253165	123	4	125	2009	12	1902	0	2761

TRINITY_DN15659_c0_g2_i4	Glyma.08G064600.3	98.48484848	123	4	125	2009	12	1902	0	2761

TRINITY_DN15659_c0_g2_i4	Glyma.08G064600.2	97.81021898	123	4	125	2009	12	1902	0	2761

TRINITY_DN15659_c0_g2_i7	Glyma.08G064600.3	96.18320611	123	4	202	2086	12	1902	0	2761

TRINITY_DN15659_c0_g2_i7	Glyma.08G064600.2	100	123	4	202	2086	12	1902	0	2761

TRINITY_DN12073_c0_g2_i2	Glyma.07G043900.4	97.01492537	112	7	346	2234	2355	482	0	2737

TRINITY_DN12077_c1_g1_i1	Glyma.02G165500.1	97.01492537	106	3	94	1983	1890	1	0	2881

TRINITY_DN12077_c1_g1_i2	Glyma.02G165500.1	97.01149425	106	3	430	2319	1890	1	0	2881

TRINITY_DN9234_c0_g1_i1	Glyma.10G197000.1	96.2962963	101	0	1	1892	965	2856	0	2935

TRINITY_DN9234_c0_g2_i1	Glyma.10G197000.1	96.2962963	101	1	1	1882	965	2856	0	2870

TRINITY_DN14522_c0_g1_i1	Glyma.08G287500.1	100	286	24	355	2215	1882	7	0	1616

TRINITY_DN13560_c0_g1_i2	Glyma.02G309100.1	96.61016949	284	11	231	2114	1881	1	0	1808

TRINITY_DN15663_c0_g1_i1	Glyma.02G034700.1	98.46153846	267	12	689	2566	1887	1	0	1903

TRINITY_DN15663_c0_g1_i1	Glyma.02G034700.2	96.67774086	267	12	689	2566	1887	1	0	1903

TRINITY_DN15663_c0_g1_i3	Glyma.02G034700.1	97.18309859	267	12	689	2566	1887	1	0	1903

TRINITY_DN15663_c0_g1_i3	Glyma.02G034700.2	96.57258065	267	12	689	2566	1887	1	0	1903

TRINITY_DN15944_c2_g2_i1	Glyma.13G083300.1	98.66666667	253	7	269	2152	2074	188	0	2019

TRINITY_DN15944_c2_g2_i1	Glyma.13G083300.3	100	253	7	269	2152	2074	188	0	2019

TRINITY_DN15944_c2_g2_i1	Glyma.13G083300.2	100	253	8	269	2152	2071	188	0	2001

TRINITY_DN15944_c2_g2_i1	Glyma.13G083300.4	96.66666667	253	8	269	2152	2071	188	0	2001

TRINITY_DN16231_c1_g2_i1	Glyma.13G114000.4	95.2	252	6	87	1974	11	1898	0	2058

TRINITY_DN16231_c1_g2_i1	Glyma.13G114000.1	96.96132597	252	6	87	1974	11	1898	0	2058

TRINITY_DN16231_c1_g2_i1	Glyma.13G114000.2	100	252	6	87	1974	11	1898	0	2058

TRINITY_DN16231_c1_g2_i1	Glyma.13G114000.3	99.15966387	252	6	87	1974	11	1898	0	2058

TRINITY_DN16257_c1_g1_i9	Glyma.13G310300.1	98.69281046	245	8	183	2064	2235	357	0	1999

TRINITY_DN16217_c2_g1_i3	Glyma.08G140900.1	98.30508475	211	3	523	2409	2274	385	0	2290

TRINITY_DN14983_c0_g1_i1	Glyma.08G026400.1	95.57195572	169	13	237	2108	1	1890	0	2427

TRINITY_DN14983_c0_g1_i1	Glyma.08G026400.2	95.59748428	169	13	237	2108	1	1890	0	2427

TRINITY_DN16217_c2_g1_i7	Glyma.08G140900.1	100	161	3	523	2409	2274	385	0	2567

TRINITY_DN16268_c1_g2_i2	Glyma.05G105000.3	98.86363636	158	10	559	2430	1875	1	0	2390

TRINITY_DN16268_c1_g2_i2	Glyma.05G105000.1	98.86363636	158	10	559	2430	1875	1	0	2390

TRINITY_DN16268_c1_g2_i2	Glyma.05G105000.2	98.86363636	158	10	559	2430	1875	1	0	2390

TRINITY_DN3523_c0_g1_i1	Glyma.14G182300.1	96.46569647	155	6	118	2004	1	1884	0	2562

TRINITY_DN16268_c1_g2_i10	Glyma.05G105000.3	98.66666667	155	13	559	2424	1875	1	0	2368

TRINITY_DN16268_c1_g2_i10	Glyma.05G105000.1	99.03846154	155	13	559	2424	1875	1	0	2368

TRINITY_DN16268_c1_g2_i10	Glyma.05G105000.2	98.41269841	155	13	559	2424	1875	1	0	2368

TRINITY_DN14922_c0_g1_i2	Glyma.20G105000.1	96.2962963	143	2	49	1938	23	1912	0	2687

TRINITY_DN16268_c1_g2_i5	Glyma.05G104900.1	100	142	9	559	2433	1887	1	0	2577

TRINITY_DN16268_c1_g2_i5	Glyma.05G104900.3	97.35560195	142	9	559	2433	1887	1	0	2577

TRINITY_DN16268_c1_g2_i5	Glyma.05G104900.2	100	142	9	559	2433	1887	1	0	2577

TRINITY_DN16268_c1_g2_i5	Glyma.05G104900.4	95.07692308	142	9	559	2433	1887	1	0	2577

TRINITY_DN3342_c0_g1_i1	Glyma.07G047300.1	95.4954955	141	6	111	1994	1	1884	0	2621

TRINITY_DN11810_c0_g1_i2	Glyma.12G049700.3	97.56097561	139	9	831	2721	3261	1392	0	2586

TRINITY_DN11721_c0_g1_i1	Glyma.04G245100.1	100	122	7	438	2327	1902	19	0	2765

TRINITY_DN12827_c0_g1_i1	Glyma.10G049600.5	99.16666667	340	22	416	2272	1881	1	0	1336

TRINITY_DN12827_c0_g1_i1	Glyma.10G049600.1	99.18032787	340	22	416	2272	1881	1	0	1336

TRINITY_DN12827_c0_g1_i1	Glyma.10G049600.2	99.18032787	340	22	416	2272	1881	1	0	1336

TRINITY_DN12827_c0_g1_i1	Glyma.10G049600.4	99.18032787	340	22	416	2272	1881	1	0	1336

TRINITY_DN12827_c0_g1_i3	Glyma.10G049600.5	100	340	22	416	2272	1881	1	0	1336

TRINITY_DN12827_c0_g1_i3	Glyma.10G049600.1	100	340	22	416	2272	1881	1	0	1336

TRINITY_DN12827_c0_g1_i3	Glyma.10G049600.2	100	340	22	416	2272	1881	1	0	1336

TRINITY_DN12827_c0_g1_i3	Glyma.10G049600.4	100	340	22	416	2272	1881	1	0	1336

TRINITY_DN12827_c0_g1_i1	Glyma.10G049600.3	99.17355372	340	23	416	2272	1878	1	0	1315

TRINITY_DN12827_c0_g1_i1	Glyma.10G049600.6	99.16666667	340	23	416	2272	1878	1	0	1315

TRINITY_DN12827_c0_g1_i3	Glyma.10G049600.3	100	340	23	416	2272	1878	1	0	1315

TRINITY_DN12827_c0_g1_i3	Glyma.10G049600.6	100	340	23	416	2272	1878	1	0	1315

TRINITY_DN13543_c1_g2_i1	Glyma.06G108800.1	96.15384615	284	33	537	2411	1848	1	0	1548

TRINITY_DN13560_c0_g1_i2	Glyma.14G003700.1	96.60493827	280	13	231	2114	1875	1	0	1803

TRINITY_DN15937_c0_g1_i4	Glyma.12G155700.3	98.05447471	229	36	544	2398	3	1860	0	1788

TRINITY_DN12195_c0_g1_i3	Glyma.18G299200.1	100	221	2	325	2212	1888	1	0	2241

TRINITY_DN16115_c0_g2_i7	Glyma.13G330600.2	95.45454545	210	5	795	2681	2667	781	0	2289

TRINITY_DN16115_c0_g2_i7	Glyma.13G330600.1	95.45454545	210	5	795	2681	2667	781	0	2289

TRINITY_DN15427_c0_g1_i1	Glyma.12G224800.1	100	189	23	386	2254	1869	1	0	2172

TRINITY_DN5733_c0_g2_i2	Glyma.16G143200.1	97.6744186	175	5	294	2168	2053	164	0	2423

TRINITY_DN12745_c0_g1_i1	Glyma.05G141800.1	96.96969697	160	10	113	1990	1	1866	0	2372

TRINITY_DN20587_c0_g1_i1	Glyma.18G044000.2	95.37037037	156	7	209	2086	3213	1342	0	2433

TRINITY_DN20587_c0_g1_i1	Glyma.18G044000.1	95.38461538	156	7	209	2086	3213	1342	0	2433

TRINITY_DN13808_c0_g1_i1	Glyma.02G048200.1	100	153	9	70	1935	1	1878	0	2410

TRINITY_DN6849_c0_g3_i1	Glyma.12G037500.1	98.61111111	140	14	155	2036	2127	249	0	2591

TRINITY_DN16268_c1_g2_i3	Glyma.05G104900.1	98.86363636	137	9	559	2427	1887	1	0	2577

TRINITY_DN16268_c1_g2_i3	Glyma.05G104900.3	98.86363636	137	9	559	2427	1887	1	0	2577

TRINITY_DN16268_c1_g2_i3	Glyma.05G104900.2	98.86363636	137	9	559	2427	1887	1	0	2577

TRINITY_DN16268_c1_g2_i3	Glyma.05G104900.4	98.86363636	137	9	559	2427	1887	1	0	2577

TRINITY_DN9775_c0_g1_i1	Glyma.08G181200.1	95.87628866	114	1	134	2023	1884	1	0	2820

TRINITY_DN14920_c0_g2_i1	Glyma.04G236000.4	100	113	2	195	2084	1935	49	0	2844

TRINITY_DN12866_c0_g2_i1	Glyma.05G019400.2	97.14285714	110	0	597	2486	1890	1	0	2881

TRINITY_DN12866_c0_g2_i1	Glyma.05G019400.1	97.14285714	110	0	597	2486	1890	1	0	2881

TRINITY_DN12866_c0_g2_i1	Glyma.17G080400.1	97.15302491	95	0	597	2486	1890	1	0	2964

TRINITY_DN15501_c0_g2_i3	Glyma.08G214300.1	95	326	8	608	2492	2040	156	0	1631

TRINITY_DN16444_c0_g1_i7	Glyma.17G111500.1	98.46153846	315	24	364	2234	970	2848	0	1563

TRINITY_DN15663_c0_g1_i1	Glyma.01G030900.2	96.67812142	276	12	697	2566	1882	1	0	1792

TRINITY_DN15663_c0_g1_i1	Glyma.01G030900.1	97.89473684	276	12	697	2566	1882	1	0	1792

TRINITY_DN15663_c0_g1_i3	Glyma.01G030900.2	99.10714286	276	12	697	2566	1882	1	0	1792

TRINITY_DN15663_c0_g1_i3	Glyma.01G030900.1	97.97101449	276	12	697	2566	1882	1	0	1792

TRINITY_DN15878_c0_g2_i1	Glyma.03G261800.5	100	269	35	60	1926	1852	1	0	1616

TRINITY_DN15878_c0_g2_i1	Glyma.03G261800.1	100	269	35	60	1926	1852	1	0	1616

TRINITY_DN15878_c0_g2_i1	Glyma.03G261800.2	100	269	35	60	1926	1852	1	0	1616

TRINITY_DN15878_c0_g2_i1	Glyma.03G261800.3	100	269	35	60	1926	1852	1	0	1616

TRINITY_DN15878_c0_g2_i1	Glyma.03G261800.4	98.80239521	269	35	60	1926	1852	1	0	1616

TRINITY_DN16257_c1_g1_i10	Glyma.13G310300.1	99.12280702	264	0	102	1990	2245	357	0	2026

TRINITY_DN15944_c2_g2_i7	Glyma.04G253000.1	98.20627803	233	7	269	2151	2053	171	0	2121

TRINITY_DN16173_c1_g1_i5	Glyma.13G076700.1	95.91836735	209	16	334	2208	1866	1	0	2091

TRINITY_DN15216_c0_g2_i1	Glyma.08G131400.3	96.15384615	179	10	45	1931	37	1908	0	2374

TRINITY_DN15216_c0_g2_i1	Glyma.08G131400.2	97.82608696	179	10	45	1931	37	1908	0	2374

TRINITY_DN15216_c0_g2_i1	Glyma.08G131400.1	98.54014599	179	10	45	1931	37	1908	0	2374

TRINITY_DN8602_c0_g1_i1	Glyma.03G161800.3	99.55555556	153	11	185	2064	2229	356	0	2486

TRINITY_DN8602_c0_g1_i1	Glyma.03G161800.2	96.36963696	153	11	185	2064	2229	356	0	2486

TRINITY_DN11099_c0_g1_i1	Glyma.20G217400.3	99.7706422	149	1	1	1889	128	2010	0	2625

TRINITY_DN11099_c0_g1_i1	Glyma.20G217400.2	99.7716895	149	1	1	1889	128	2010	0	2625

TRINITY_DN11099_c0_g1_i1	Glyma.20G217400.1	95.05703422	149	1	1	1889	128	2010	0	2625

TRINITY_DN11099_c0_g1_i1	Glyma.10G172900.1	98.5	146	1	1	1889	287	2172	0	2660

TRINITY_DN11099_c0_g1_i1	Glyma.10G172900.2	100	146	1	1	1889	134	2019	0	2660

TRINITY_DN15519_c1_g1_i2	Glyma.07G218800.1	96.55172414	306	4	378	2263	1931	46	0	1766

TRINITY_DN14122_c0_g1_i1	Glyma.16G156100.2	97.68339768	283	21	224	2089	1	1881	0	1731

TRINITY_DN14122_c0_g1_i1	Glyma.16G156100.1	100	283	21	224	2089	1	1881	0	1731

TRINITY_DN14122_c0_g1_i2	Glyma.16G156100.2	97.84172662	283	21	224	2089	1	1881	0	1731

TRINITY_DN14122_c0_g1_i2	Glyma.16G156100.1	96.23188406	283	21	224	2089	1	1881	0	1731

TRINITY_DN13423_c0_g2_i1	Glyma.09G037900.4	96.6442953	279	6	1475	3359	1886	5	0	1882

TRINITY_DN13543_c1_g1_i1	Glyma.14G166100.1	96.17918314	275	36	444	2288	1857	1	0	1485

TRINITY_DN16173_c1_g1_i1	Glyma.13G076700.1	100	221	14	334	2208	1866	1	0	2036

TRINITY_DN15501_c0_g2_i1	Glyma.07G028500.2	99.49238579	205	6	568	2452	2040	156	0	2313

TRINITY_DN16173_c1_g1_i2	Glyma.13G076700.1	95.12195122	203	13	334	2211	1866	1	0	2156

TRINITY_DN15216_c0_g2_i1	Glyma.05G174200.1	96.09375	184	2	45	1931	37	1923	0	2455

TRINITY_DN15216_c0_g2_i1	Glyma.05G174200.2	100	184	2	45	1931	37	1923	0	2455

TRINITY_DN15501_c0_g2_i6	Glyma.07G028500.2	95.55555556	166	6	103	1987	2040	156	0	2529

TRINITY_DN17221_c0_g1_i1	Glyma.02G039400.1	98.79518072	146	7	441	2318	1887	1	0	2606

TRINITY_DN12691_c0_g1_i1	Glyma.11G104500.1	97.33333333	141	3	116	2001	1886	1	0	2680

TRINITY_DN15216_c0_g2_i2	Glyma.08G131400.3	98.86792453	141	8	45	1931	37	1908	0	2595

TRINITY_DN15216_c0_g2_i2	Glyma.08G131400.2	98.46153846	141	8	45	1931	37	1908	0	2595

TRINITY_DN15216_c0_g2_i2	Glyma.08G131400.1	97.6076555	141	8	45	1931	37	1908	0	2595

TRINITY_DN12691_c0_g1_i1	Glyma.12G029500.1	97.33333333	138	3	116	2001	1886	1	0	2697

TRINITY_DN5503_c0_g1_i4	Glyma.10G208200.1	98.58490566	135	2	305	2186	1	1888	0	2700

TRINITY_DN13868_c1_g1_i1	Glyma.11G012300.1	97.59036145	119	5	178	2046	1	1881	0	2660

TRINITY_DN16802_c0_g1_i1	Glyma.20G221100.3	97.72727273	118	2	361	2247	1887	1	0	2820

TRINITY_DN15097_c0_g1_i3	Glyma.02G038800.1	96.42857143	115	10	14	1899	247	2106	0	2656

TRINITY_DN11825_c0_g2_i1	Glyma.05G044200.2	98.55725879	109	1	376	2263	1915	31	0	2863

TRINITY_DN11825_c0_g2_i3	Glyma.05G044200.2	98.56115108	109	1	376	2263	1915	31	0	2863

TRINITY_DN14713_c0_g1_i6	Glyma.04G086300.1	100	106	8	124	1989	1	1869	0	2634

TRINITY_DN14677_c0_g1_i5	Glyma.07G252600.3	100	95	0	986	2873	1892	5	0	2961

TRINITY_DN16257_c1_g1_i10	Glyma.12G191700.1	97.14285714	432	85	154	1980	2208	370	3.94E-108	394

TRINITY_DN13543_c1_g2_i1	Glyma.06G108800.3	96.15384615	284	32	537	2411	1845	1	0	1563

TRINITY_DN13543_c1_g2_i1	Glyma.06G108800.4	96.15384615	284	32	537	2411	1845	1	0	1563

TRINITY_DN13543_c1_g2_i1	Glyma.06G108800.5	96.15384615	284	32	537	2411	1845	1	0	1563

TRINITY_DN13543_c1_g2_i1	Glyma.06G108800.2	96.15384615	284	32	537	2411	1845	1	0	1563

TRINITY_DN15736_c0_g1_i4	Glyma.04G203200.1	98.61111111	256	17	403	2279	426	2302	0	1938

TRINITY_DN16279_c0_g3_i2	Glyma.18G098400.5	96.06299213	243	11	200	2081	31	1909	0	2054

TRINITY_DN15944_c2_g2_i1	Glyma.14G164600.4	97.43589744	241	1	269	2152	2059	173	0	2130

TRINITY_DN15944_c2_g2_i1	Glyma.14G164600.1	95.19650655	241	1	269	2152	2059	173	0	2130

TRINITY_DN15944_c2_g2_i1	Glyma.14G164600.3	97.93814433	241	2	269	2152	2056	173	0	2111

TRINITY_DN15944_c2_g2_i1	Glyma.14G164600.2	97.93814433	241	2	269	2152	2056	173	0	2111

TRINITY_DN14706_c0_g1_i2	Glyma.09G196200.1	96.51898734	204	7	460	2331	1887	1	0	2257

TRINITY_DN14706_c0_g1_i2	Glyma.09G196200.2	96.49122807	204	7	460	2331	1887	1	0	2257

TRINITY_DN16115_c0_g2_i7	Glyma.15G043400.3	100	201	2	796	2681	2666	781	0	2359

TRINITY_DN16115_c0_g2_i7	Glyma.15G043400.2	100	201	2	796	2681	2666	781	0	2359

TRINITY_DN16115_c0_g2_i7	Glyma.15G043400.1	95.45454545	201	2	796	2681	2666	781	0	2359

TRINITY_DN14706_c0_g1_i2	Glyma.16G112800.1	96.47058824	187	6	460	2331	1884	1	0	2333

TRINITY_DN14706_c0_g1_i2	Glyma.16G112800.2	96.47058824	187	6	460	2331	1884	1	0	2333

TRINITY_DN36250_c0_g1_i1	Glyma.13G204900.4	96.32	170	15	1	1872	775	2646	0	2350

TRINITY_DN36250_c0_g1_i1	Glyma.13G204900.3	98.66666667	170	15	1	1872	775	2646	0	2350

TRINITY_DN36250_c0_g1_i1	Glyma.13G204900.1	98.55072464	170	15	1	1872	775	2646	0	2350

TRINITY_DN36250_c0_g1_i1	Glyma.13G204900.2	99.11111111	170	15	1	1872	775	2646	0	2350

TRINITY_DN10824_c0_g1_i1	Glyma.03G159200.1	97.94238683	166	12	254	2131	1878	1	0	2449

TRINITY_DN10824_c0_g1_i1	Glyma.03G159200.2	98.71794872	166	13	254	2131	1872	1	0	2410

TRINITY_DN15541_c0_g1_i1	Glyma.03G263200.1	96.4028777	162	4	370	2250	2041	156	0	2542

TRINITY_DN119_c0_g1_i1	Glyma.19G229200.1	98.88888889	159	9	912	2798	1874	7	0	2481

TRINITY_DN10824_c0_g1_i1	Glyma.19G161500.1	98.4962406	158	11	254	2131	1878	1	0	2494

TRINITY_DN10824_c0_g1_i1	Glyma.19G161500.2	98.4962406	158	12	254	2131	1872	1	0	2455

TRINITY_DN9290_c0_g1_i3	Glyma.13G237200.2	100	148	2	186	2063	1	1887	0	2606

TRINITY_DN14809_c0_g2_i1	Glyma.02G111800.2	95.83333333	148	11	342	2216	1878	1	0	2529

TRINITY_DN14809_c0_g2_i1	Glyma.02G111800.1	98.22485207	148	11	342	2216	1878	1	0	2529

TRINITY_DN15216_c0_g2_i2	Glyma.05G174200.1	98.97959184	146	0	45	1931	37	1923	0	2676

TRINITY_DN15216_c0_g2_i2	Glyma.05G174200.2	100	146	0	45	1931	37	1923	0	2676

TRINITY_DN11811_c0_g1_i1	Glyma.02G125400.10	97.72727273	141	4	159	2033	1	1875	0	2549

TRINITY_DN11811_c0_g1_i1	Glyma.02G125400.6	97.73584906	141	4	159	2033	1	1875	0	2549

TRINITY_DN16268_c1_g2_i2	Glyma.05G104900.1	100	139	5	559	2430	1887	1	0	2617

TRINITY_DN16268_c1_g2_i2	Glyma.05G104900.3	100	139	5	559	2430	1887	1	0	2617

TRINITY_DN16268_c1_g2_i2	Glyma.05G104900.2	100	139	5	559	2430	1887	1	0	2617

TRINITY_DN16268_c1_g2_i2	Glyma.05G104900.4	96.7032967	139	5	559	2430	1887	1	0	2617

TRINITY_DN11811_c0_g2_i1	Glyma.02G125400.10	97.11191336	138	4	159	2033	1	1875	0	2566

TRINITY_DN11811_c0_g2_i1	Glyma.02G125400.6	97.10144928	138	4	159	2033	1	1875	0	2566

TRINITY_DN11811_c0_g2_i2	Glyma.02G125400.10	97.11538462	138	4	159	2033	1	1875	0	2566

TRINITY_DN11811_c0_g2_i2	Glyma.02G125400.6	97.10144928	138	4	159	2033	1	1875	0	2566

TRINITY_DN2125_c0_g1_i1	Glyma.20G153600.1	95.19230769	133	0	74	1960	1	1887	0	2748

TRINITY_DN16268_c1_g2_i10	Glyma.05G104900.1	99.01960784	132	7	559	2424	1887	1	0	2617

TRINITY_DN16268_c1_g2_i10	Glyma.05G104900.3	100	132	7	559	2424	1887	1	0	2617

TRINITY_DN16268_c1_g2_i10	Glyma.05G104900.2	99.29577465	132	7	559	2424	1887	1	0	2617

TRINITY_DN16268_c1_g2_i10	Glyma.05G104900.4	99.29577465	132	7	559	2424	1887	1	0	2617

TRINITY_DN2125_c0_g1_i1	Glyma.10G241000.1	95.19230769	122	0	74	1960	1	1887	0	2809

TRINITY_DN15097_c0_g1_i3	Glyma.01G026000.1	100	115	10	14	1899	247	2106	0	2667

TRINITY_DN11030_c0_g1_i1	Glyma.13G280700.1	100	106	2	206	2092	1	1881	0	2859

TRINITY_DN11030_c0_g1_i1	Glyma.12G220900.2	95.90163934	103	2	206	2092	1	1881	0	2876

TRINITY_DN11030_c0_g1_i1	Glyma.12G220900.1	96.8	103	2	206	2092	1	1881	0	2876

TRINITY_DN16382_c0_g3_i1	Glyma.18G060600.2	100	102	0	489	2375	184	2070	0	2920

TRINITY_DN11768_c0_g1_i1	Glyma.18G276200.1	96.55172414	398	25	646	2518	3139	1267	0	1110

TRINITY_DN13088_c0_g1_i2	Glyma.09G143700.3	99.45355191	328	22	606	2466	1	1879	0	1459

TRINITY_DN13088_c0_g1_i2	Glyma.09G143700.4	99.45355191	328	22	606	2466	1	1879	0	1459

TRINITY_DN13088_c0_g1_i2	Glyma.09G143700.2	99.45355191	328	22	606	2466	1	1879	0	1459

TRINITY_DN13088_c0_g1_i2	Glyma.16G196700.2	99.45652174	323	21	606	2466	1	1873	0	1448

TRINITY_DN13088_c0_g1_i2	Glyma.16G196700.3	99.45652174	323	21	606	2466	1	1873	0	1448

TRINITY_DN13543_c1_g2_i1	Glyma.04G253600.4	96.15384615	290	41	537	2411	1830	1	0	1443

TRINITY_DN13543_c1_g2_i1	Glyma.04G253600.2	96.15384615	290	41	537	2411	1830	1	0	1443

TRINITY_DN13543_c1_g2_i1	Glyma.04G253600.1	96.15384615	290	41	537	2411	1830	1	0	1443

TRINITY_DN15736_c0_g1_i4	Glyma.06G162300.1	99.58506224	254	16	404	2276	457	2335	0	1947

TRINITY_DN15685_c0_g1_i1	Glyma.19G230500.1	95.4954955	229	7	200	2079	3393	1511	0	2156

TRINITY_DN15685_c0_g1_i1	Glyma.03G233400.1	95.4954955	227	6	200	2079	3393	1511	0	2167

TRINITY_DN15685_c0_g1_i1	Glyma.19G230500.3	95.23809524	225	16	200	2079	3369	1511	0	2023

TRINITY_DN11977_c0_g1_i1	Glyma.04G041400.2	100	187	3	281	2165	76	1959	0	2427

TRINITY_DN16324_c0_g1_i1	Glyma.13G091200.1	100	161	0	280	2165	1886	1	0	2591

TRINITY_DN16324_c0_g1_i7	Glyma.13G091200.1	100	161	0	280	2165	1886	1	0	2591

TRINITY_DN40841_c0_g1_i1	Glyma.16G047300.1	99.00990099	159	7	17	1895	1248	3129	0	2531

TRINITY_DN5733_c0_g1_i1	Glyma.16G143200.2	98.55072464	150	5	335	2205	1887	2	0	2555

TRINITY_DN16324_c0_g1_i1	Glyma.17G069100.1	100	148	0	280	2165	1886	1	0	2663

TRINITY_DN16324_c0_g1_i7	Glyma.17G069100.1	100	148	0	280	2165	1886	1	0	2663

TRINITY_DN14809_c0_g2_i1	Glyma.01G053300.1	100	147	10	342	2216	1872	1	0	2507

TRINITY_DN15685_c0_g2_i1	Glyma.19G230500.1	97.68786127	122	3	306	2188	3393	1511	0	2769

TRINITY_DN15685_c0_g2_i1	Glyma.19G230500.3	97.68976898	120	11	306	2188	3369	1511	0	2625

TRINITY_DN15619_c0_g2_i3	Glyma.05G038300.2	99.00990099	117	2	912	2793	1	1886	0	2809

TRINITY_DN15685_c0_g2_i1	Glyma.03G233400.1	98.51973684	112	3	306	2188	3393	1511	0	2824

TRINITY_DN5791_c0_g1_i1	Glyma.18G101400.2	98.48771267	108	6	320	2199	704	2580	0	2787

TRINITY_DN16680_c0_g1_i1	Glyma.20G024700.1	97.14285714	107	3	136	2019	2007	123	0	2870

TRINITY_DN13514_c1_g1_i1	Glyma.17G120500.1	100	101	6	341	2221	7752	5875	0	2839

TRINITY_DN14809_c0_g1_i4	Glyma.02G111800.2	96.50582363	325	14	387	2261	1878	1	0	1570

TRINITY_DN14809_c0_g1_i4	Glyma.02G111800.1	95.94594595	325	14	387	2261	1878	1	0	1570

TRINITY_DN15950_c0_g1_i2	Glyma.13G360400.1	97.84946237	311	58	3	1799	172	2019	0	950

TRINITY_DN15950_c0_g1_i2	Glyma.13G360400.2	97.84946237	311	58	3	1799	172	2019	0	950

TRINITY_DN14809_c0_g1_i5	Glyma.02G111800.2	98.64864865	309	14	387	2261	1878	1	0	1659

TRINITY_DN14809_c0_g1_i5	Glyma.02G111800.1	97.36842105	309	14	387	2261	1878	1	0	1659

TRINITY_DN15944_c1_g1_i4	Glyma.05G056800.3	100	301	6	521	2402	1924	43	0	1775

TRINITY_DN15403_c0_g2_i3	Glyma.09G153500.4	96.77419355	296	18	839	2716	19	1875	0	1615

TRINITY_DN15403_c0_g2_i3	Glyma.09G153500.1	96.49122807	296	18	839	2716	19	1875	0	1615

TRINITY_DN16257_c1_g1_i10	Glyma.12G191800.1	96.99074074	265	2	102	1985	2239	356	0	2001

TRINITY_DN12925_c0_g1_i1	Glyma.01G212200.4	96.71052632	265	12	263	2147	1926	63	0	1877

TRINITY_DN15365_c0_g2_i1	Glyma.01G153600.1	99.81308411	216	2	326	2209	1884	1	0	2272

TRINITY_DN16027_c1_g3_i5	Glyma.07G036300.6	95.32554257	199	13	385	2244	1872	1	0	2134

TRINITY_DN16027_c1_g3_i5	Glyma.07G036300.9	97.5	199	13	385	2244	1872	1	0	2134

TRINITY_DN16027_c1_g3_i5	Glyma.07G036300.7	99.01960784	199	13	385	2244	1872	1	0	2134

TRINITY_DN16027_c1_g3_i7	Glyma.07G036300.6	100	199	13	385	2244	1872	1	0	2134

TRINITY_DN16027_c1_g3_i7	Glyma.07G036300.9	99.58847737	199	13	385	2244	1872	1	0	2134

TRINITY_DN16027_c1_g3_i7	Glyma.07G036300.7	100	199	13	385	2244	1872	1	0	2134

TRINITY_DN14238_c0_g2_i4	Glyma.15G066700.6	97.63033175	137	4	84	1963	21	1903	0	2676

TRINITY_DN14238_c0_g2_i4	Glyma.15G066700.1	97.61904762	137	4	84	1963	21	1903	0	2676

TRINITY_DN7211_c0_g1_i1	Glyma.16G093800.38	100	133	14	1	1876	3387	5229	0	2414

TRINITY_DN7211_c0_g1_i1	Glyma.16G093800.1	100	133	14	1	1876	3387	5229	0	2414

TRINITY_DN7211_c0_g1_i1	Glyma.16G093800.11	97.01810437	133	14	1	1876	3372	5214	0	2414

TRINITY_DN7211_c0_g1_i1	Glyma.16G093800.33	100	133	14	1	1876	3372	5214	0	2414

TRINITY_DN7211_c0_g1_i1	Glyma.16G093800.34	96.34551495	133	14	1	1876	3372	5214	0	2414

TRINITY_DN7211_c0_g1_i1	Glyma.16G093800.7	100	133	14	1	1876	3372	5214	0	2414

TRINITY_DN7211_c0_g1_i1	Glyma.16G093800.10	95.91836735	133	14	1	1876	3369	5211	0	2414

TRINITY_DN7211_c0_g1_i1	Glyma.16G093800.31	98.24561404	133	14	1	1876	3369	5211	0	2414

TRINITY_DN7211_c0_g1_i1	Glyma.16G093800.5	100	133	14	1	1876	3369	5211	0	2414

TRINITY_DN7211_c0_g1_i1	Glyma.16G093800.36	95.91836735	131	18	1	1876	3387	5220	0	2368

TRINITY_DN7211_c0_g1_i1	Glyma.16G093800.15	100	131	18	1	1876	3387	5220	0	2368

TRINITY_DN7211_c0_g1_i1	Glyma.16G093800.28	100	131	18	1	1876	3372	5205	0	2368

TRINITY_DN7211_c0_g1_i1	Glyma.16G093800.29	100	131	18	1	1876	3372	5205	0	2368

TRINITY_DN7211_c0_g1_i1	Glyma.16G093800.13	98.66666667	131	18	1	1876	3372	5205	0	2368

TRINITY_DN7211_c0_g1_i1	Glyma.16G093800.25	95.10135135	131	18	1	1876	3369	5202	0	2368

TRINITY_DN7211_c0_g1_i1	Glyma.16G093800.18	95.10204082	131	18	1	1876	3369	5202	0	2368

TRINITY_DN13868_c1_g1_i1	Glyma.01G229500.1	100	129	3	178	2046	1	1884	0	2656

TRINITY_DN14238_c0_g2_i4	Glyma.13G247300.3	97.61904762	125	4	84	1963	27	1909	0	2743

TRINITY_DN14238_c0_g2_i4	Glyma.13G247300.1	97.61904762	125	4	84	1963	27	1909	0	2743

TRINITY_DN14693_c0_g1_i1	Glyma.10G201000.2	99.25373134	120	7	128	2003	1	1882	0	2739

TRINITY_DN13868_c1_g1_i1	Glyma.11G012300.2	96.15384615	119	4	178	2046	1	1878	0	2673

TRINITY_DN14693_c0_g1_i1	Glyma.20G189500.1	98.83040936	118	6	128	2003	1	1885	0	2769

TRINITY_DN16372_c0_g1_i6	Glyma.07G114200.1	98.30508475	310	10	609	2486	1878	1	0	1685

TRINITY_DN15937_c0_g1_i1	Glyma.06G242100.1	98	287	37	544	2393	3	1855	0	1469

TRINITY_DN15937_c0_g1_i1	Glyma.06G242100.2	95.27559055	287	37	544	2393	3	1855	0	1469

TRINITY_DN15937_c0_g1_i1	Glyma.06G242100.3	95.27559055	287	37	544	2393	3	1855	0	1469

TRINITY_DN15937_c0_g1_i2	Glyma.06G242100.1	95.23809524	287	37	544	2393	3	1855	0	1469

TRINITY_DN15937_c0_g1_i2	Glyma.06G242100.2	99.26470588	287	37	544	2393	3	1855	0	1469

TRINITY_DN15937_c0_g1_i2	Glyma.06G242100.3	100	287	37	544	2393	3	1855	0	1469

TRINITY_DN15937_c0_g1_i3	Glyma.06G242100.1	97.8021978	287	37	544	2393	3	1855	0	1469

TRINITY_DN15937_c0_g1_i3	Glyma.06G242100.2	98.75	287	37	544	2393	3	1855	0	1469

TRINITY_DN15937_c0_g1_i3	Glyma.06G242100.3	100	287	37	544	2393	3	1855	0	1469

TRINITY_DN15937_c0_g1_i5	Glyma.06G242100.1	100	287	37	544	2393	3	1855	0	1469

TRINITY_DN15937_c0_g1_i5	Glyma.06G242100.2	96.34703196	287	37	544	2393	3	1855	0	1469

TRINITY_DN15937_c0_g1_i5	Glyma.06G242100.3	97.95918367	287	37	544	2393	3	1855	0	1469

TRINITY_DN13543_c1_g1_i1	Glyma.13G084300.1	99.26470588	286	32	444	2288	1866	1	0	1526

TRINITY_DN15365_c0_g2_i1	Glyma.11G091000.1	98.55072464	218	0	326	2209	1884	1	0	2272

TRINITY_DN14410_c0_g1_i1	Glyma.08G055700.1	96.2962963	214	13	141	2012	60	1931	0	2139

TRINITY_DN15327_c1_g1_i5	Glyma.10G106600.1	98.65470852	175	0	234	2117	1884	1	0	2510

TRINITY_DN5733_c0_g2_i2	Glyma.16G143200.2	98.95833333	173	5	294	2162	1885	2	0	2423

TRINITY_DN13867_c0_g1_i1	Glyma.07G032500.1	97.50260146	173	12	164	2006	1	1870	0	2165

TRINITY_DN13867_c0_g1_i1	Glyma.07G032500.10	99.41860465	173	12	164	2006	1	1870	0	2165

TRINITY_DN15944_c1_g1_i2	Glyma.05G056800.3	95.52238806	169	2	621	2503	1925	43	0	2531

TRINITY_DN36250_c0_g1_i1	Glyma.15G107800.2	97.18309859	159	9	1	1872	766	2637	0	2444

TRINITY_DN36250_c0_g1_i1	Glyma.15G107800.5	95.08928571	159	9	1	1872	766	2637	0	2444

TRINITY_DN36250_c0_g1_i1	Glyma.15G107800.1	95.08928571	159	9	1	1872	766	2637	0	2444

TRINITY_DN36250_c0_g1_i1	Glyma.15G107800.3	95.08928571	159	9	1	1872	766	2637	0	2444

TRINITY_DN14667_c0_g2_i1	Glyma.08G209100.1	96.42857143	159	22	429	2273	1872	1	0	2268

TRINITY_DN15327_c1_g1_i3	Glyma.10G106600.1	98.93617021	137	0	234	2117	1884	1	0	2721

TRINITY_DN15365_c0_g1_i1	Glyma.11G091000.1	97.43589744	130	0	363	2246	1884	1	0	2760

TRINITY_DN15365_c0_g1_i1	Glyma.01G153600.1	96.96969697	128	0	363	2246	1884	1	0	2771

TRINITY_DN12877_c0_g3_i4	Glyma.10G280500.1	98.37962963	128	4	207	2081	2325	448	0	2673

TRINITY_DN10347_c0_g2_i1	Glyma.05G179000.1	95.55921053	95	0	420	2303	1	1884	0	2953

TRINITY_DN10347_c0_g2_i1	Glyma.08G136500.1	95.55555556	90	0	420	2303	1	1884	0	2981

TRINITY_DN14967_c1_g1_i1	Glyma.11G004300.2	100	86	4	383	2259	244	2126	0	2952

TRINITY_DN14967_c1_g1_i1	Glyma.11G004300.1	100	86	4	383	2259	244	2126	0	2952

TRINITY_DN15369_c0_g2_i4	Glyma.18G289100.1	98.18181818	301	13	7	1882	1876	1	0	1720

TRINITY_DN15369_c0_g1_i1	Glyma.03G171100.1	96.57142857	296	5	383	2262	1880	1	0	1799

TRINITY_DN10335_c1_g2_i1	Glyma.10G023400.1	100	267	27	4	1860	19	1890	0	1759

TRINITY_DN15327_c1_g1_i2	Glyma.02G272100.4	100	235	2	236	2117	1882	1	0	2163

TRINITY_DN15685_c0_g2_i1	Glyma.20G238400.1	100	232	1	306	2188	3393	1514	0	2172

TRINITY_DN15685_c0_g2_i1	Glyma.20G238400.2	100	232	1	306	2188	3213	1334	0	2172

TRINITY_DN15685_c0_g2_i1	Glyma.10G150000.1	100	219	1	306	2188	3357	1478	0	2244

TRINITY_DN15685_c0_g2_i1	Glyma.10G150000.2	95	219	1	306	2188	2508	629	0	2244

TRINITY_DN16245_c0_g3_i2	Glyma.16G099700.1	98.38709677	219	2	399	2280	3473	1592	0	2252

TRINITY_DN16027_c1_g3_i5	Glyma.16G005700.7	99.01960784	213	11	385	2244	1875	1	0	2097

TRINITY_DN16027_c1_g3_i7	Glyma.16G005700.7	100	213	11	385	2244	1875	1	0	2097

TRINITY_DN9237_c0_g1_i2	Glyma.05G151200.2	100	212	12	264	2140	176	2052	0	2226

TRINITY_DN9237_c0_g1_i2	Glyma.05G151200.1	98.9010989	212	12	264	2140	176	2052	0	2226

TRINITY_DN15327_c1_g1_i4	Glyma.02G272100.4	97.56097561	209	2	236	2117	1882	1	0	2307

TRINITY_DN12149_c0_g1_i1	Glyma.14G073700.1	97.36842105	196	26	223	2064	2074	200	0	2074

TRINITY_DN13734_c0_g1_i3	Glyma.01G191100.2	100	182	8	1	1865	661	2537	0	2314

TRINITY_DN13734_c0_g1_i3	Glyma.01G191100.1	100	182	8	1	1865	661	2537	0	2314

TRINITY_DN15008_c0_g1_i1	Glyma.08G013300.1	99.6763754	180	26	85	1926	37	1905	0	2124

TRINITY_DN13867_c0_g1_i1	Glyma.07G032500.4	97.36842105	166	31	164	2006	1	1855	0	2119

TRINITY_DN12751_c0_g1_i3	Glyma.18G180900.10	95.7957958	151	8	165	2041	1920	56	0	2486

TRINITY_DN418_c0_g1_i1	Glyma.18G222100.2	97.87234043	140	13	9	1867	70	1943	0	2488

TRINITY_DN14369_c1_g1_i1	Glyma.11G001500.1	97.22222222	138	4	84	1962	7	1887	0	2675

TRINITY_DN11533_c0_g1_i1	Glyma.12G078500.1	96.98492462	136	9	271	2153	2126	268	0	2569

TRINITY_DN11533_c0_g1_i2	Glyma.12G078500.1	97.17514124	136	9	271	2153	2126	268	0	2569

TRINITY_DN11533_c0_g1_i1	Glyma.12G078500.2	96.51162791	136	9	271	2153	2114	256	0	2569

TRINITY_DN11533_c0_g1_i2	Glyma.12G078500.2	97.17514124	136	9	271	2153	2114	256	0	2569

TRINITY_DN6848_c0_g2_i1	Glyma.04G096900.2	100	257	9	1	1879	4956	3093	0	1916

TRINITY_DN6848_c0_g2_i1	Glyma.04G096900.1	100	257	9	1	1879	4956	3093	0	1916

TRINITY_DN16173_c1_g1_i2	Glyma.13G076800.1	95.12195122	213	19	334	2211	1857	1	0	2108

TRINITY_DN16027_c1_g3_i5	Glyma.16G005700.6	99.01960784	212	13	385	2244	1869	1	0	2074

TRINITY_DN16027_c1_g3_i5	Glyma.16G005700.2	99.01960784	212	13	385	2244	1869	1	0	2074

TRINITY_DN16027_c1_g3_i7	Glyma.16G005700.6	98.57142857	212	13	385	2244	1869	1	0	2074

TRINITY_DN16027_c1_g3_i7	Glyma.16G005700.2	95.74468085	212	13	385	2244	1869	1	0	2074

TRINITY_DN16173_c1_g1_i1	Glyma.13G076800.1	100	204	20	334	2208	1857	1	0	2139

TRINITY_DN12875_c0_g1_i2	Glyma.01G005200.1	99.33993399	193	4	370	2249	1881	2	0	2381

TRINITY_DN16173_c1_g1_i5	Glyma.13G076800.1	98.27586207	193	20	334	2208	1857	1	0	2200

TRINITY_DN12875_c0_g1_i1	Glyma.01G005200.1	99.32885906	189	13	370	2229	1881	2	0	2274

TRINITY_DN14667_c0_g2_i1	Glyma.07G033100.1	100	160	22	429	2273	1869	1	0	2266

TRINITY_DN11533_c0_g1_i1	Glyma.11G195800.2	99.5	159	10	270	2151	2115	258	0	2440

TRINITY_DN11533_c0_g1_i2	Glyma.11G195800.2	98.68852459	159	10	270	2151	2115	258	0	2440

TRINITY_DN13056_c1_g1_i1	Glyma.07G224600.1	96.25	146	12	103	1963	7	1888	0	2531

TRINITY_DN13056_c1_g1_i3	Glyma.07G224600.1	96.25	146	12	97	1957	7	1888	0	2531

TRINITY_DN7211_c0_g1_i1	Glyma.03G081300.5	96.59090909	141	11	1	1876	3360	5214	0	2481

TRINITY_DN7211_c0_g1_i1	Glyma.03G081300.6	98.24561404	141	11	1	1876	3360	5214	0	2481

TRINITY_DN7211_c0_g1_i1	Glyma.03G081300.4	98.24561404	141	11	1	1876	3360	5214	0	2481

TRINITY_DN7211_c0_g1_i1	Glyma.03G081300.3	98.42519685	141	11	1	1876	3360	5214	0	2481

TRINITY_DN7211_c0_g1_i1	Glyma.03G081300.1	98.42519685	141	11	1	1876	3360	5214	0	2481

TRINITY_DN7211_c0_g1_i1	Glyma.16G093800.23	95.53571429	133	13	1	1876	3387	5226	0	2429

TRINITY_DN7211_c0_g1_i1	Glyma.16G093800.37	97.6744186	133	13	1	1876	3387	5226	0	2429

TRINITY_DN7211_c0_g1_i1	Glyma.16G093800.16	100	133	13	1	1876	3387	5226	0	2429

TRINITY_DN7211_c0_g1_i1	Glyma.16G093800.42	98.8372093	133	13	1	1876	3372	5211	0	2429

TRINITY_DN7211_c0_g1_i1	Glyma.16G093800.9	100	133	13	1	1876	3372	5211	0	2429

TRINITY_DN7211_c0_g1_i1	Glyma.16G093800.3	100	133	13	1	1876	3372	5211	0	2429

TRINITY_DN7211_c0_g1_i1	Glyma.16G093800.40	95.53571429	133	13	1	1876	3372	5211	0	2429

TRINITY_DN7211_c0_g1_i1	Glyma.16G093800.32	98.52216749	133	13	1	1876	3372	5211	0	2429

TRINITY_DN7211_c0_g1_i1	Glyma.16G093800.41	99.03225806	133	13	1	1876	3372	5211	0	2429

TRINITY_DN7211_c0_g1_i1	Glyma.16G093800.6	100	133	13	1	1876	3372	5211	0	2429

TRINITY_DN7211_c0_g1_i1	Glyma.16G093800.39	99.14529915	133	13	1	1876	3372	5211	0	2429

TRINITY_DN7211_c0_g1_i1	Glyma.16G093800.2	97.6744186	133	13	1	1876	3372	5211	0	2429

TRINITY_DN7211_c0_g1_i1	Glyma.16G093800.8	98.75	133	13	1	1876	3369	5208	0	2429

TRINITY_DN7211_c0_g1_i1	Glyma.16G093800.30	100	133	13	1	1876	3369	5208	0	2429

TRINITY_DN7211_c0_g1_i1	Glyma.16G093800.4	100	133	13	1	1876	3369	5208	0	2429

TRINITY_DN7211_c0_g1_i1	Glyma.16G093800.22	100	133	13	1	1876	3282	5121	0	2429

TRINITY_DN7211_c0_g1_i1	Glyma.16G093800.20	95.83333333	133	13	1	1876	3282	5121	0	2429

TRINITY_DN7211_c0_g1_i1	Glyma.16G093800.21	97.6744186	133	13	1	1876	3267	5106	0	2429

TRINITY_DN7211_c0_g1_i1	Glyma.16G093800.19	98.41269841	133	13	1	1876	3267	5106	0	2429

TRINITY_DN7211_c0_g1_i1	Glyma.16G093800.35	98.13664596	131	17	1	1876	3387	5217	0	2381

TRINITY_DN7211_c0_g1_i1	Glyma.16G093800.14	98.13664596	131	17	1	1876	3387	5217	0	2381

TRINITY_DN7211_c0_g1_i1	Glyma.16G093800.26	97.16885743	131	17	1	1876	3372	5202	0	2381

TRINITY_DN7211_c0_g1_i1	Glyma.16G093800.27	98.13432836	131	17	1	1876	3372	5202	0	2381

TRINITY_DN7211_c0_g1_i1	Glyma.16G093800.12	98.13432836	131	17	1	1876	3372	5202	0	2381

TRINITY_DN7211_c0_g1_i1	Glyma.16G093800.24	100	131	17	1	1876	3369	5199	0	2381

TRINITY_DN7211_c0_g1_i1	Glyma.16G093800.17	97.2972973	131	17	1	1876	3369	5199	0	2381

TRINITY_DN12709_c0_g1_i3	Glyma.17G041100.1	99.27536232	112	4	152	2029	1	1878	0	2804

TRINITY_DN12709_c0_g1_i3	Glyma.17G041100.2	98.07692308	110	5	152	2029	1	1863	0	2717

TRINITY_DN15369_c0_g2_i1	Glyma.18G289100.1	99	306	9	7	1882	1876	1	0	1714

TRINITY_DN8179_c0_g1_i1	Glyma.02G101100.1	98.22834646	286	32	1	1822	387	2256	0	1437

TRINITY_DN9793_c0_g1_i1	Glyma.11G223200.1	99.23076923	248	13	216	2087	79	1950	0	1984

TRINITY_DN12944_c0_g2_i1	Glyma.13G328900.1	97.6109215	186	30	181	2019	1845	1	0	1940

TRINITY_DN15247_c0_g2_i1	Glyma.07G226100.1	97.54601227	174	8	256	2118	1	1857	0	2239

TRINITY_DN15247_c0_g2_i4	Glyma.07G226100.1	100	174	8	256	2118	1	1857	0	2239

TRINITY_DN13867_c0_g1_i1	Glyma.07G032500.2	97.5	173	11	164	2006	1	1867	0	2180

TRINITY_DN12745_c0_g1_i1	Glyma.08G097700.1	96.96969697	170	4	113	1990	1	1878	0	2494

TRINITY_DN11533_c0_g1_i1	Glyma.11G195800.1	97.74647887	158	10	271	2151	2114	258	0	2444

TRINITY_DN11533_c0_g1_i2	Glyma.11G195800.1	97.17514124	158	10	271	2151	2114	258	0	2444

TRINITY_DN14257_c0_g2_i1	Glyma.18G141500.1	97.14285714	156	1	215	2089	70	1950	0	2571

TRINITY_DN14005_c0_g1_i1	Glyma.15G027900.6	97.11538462	150	6	207	2079	1	1879	0	2579

TRINITY_DN14005_c0_g1_i1	Glyma.15G027900.3	100	150	6	207	2079	1	1879	0	2579

TRINITY_DN14005_c0_g1_i1	Glyma.15G027900.4	98.5915493	150	6	207	2079	1	1879	0	2579

TRINITY_DN14005_c0_g1_i1	Glyma.15G027900.2	95.95375723	150	6	207	2079	1	1879	0	2579

TRINITY_DN14005_c0_g1_i1	Glyma.15G027900.1	95.38461538	150	6	207	2079	1	1879	0	2579

TRINITY_DN14005_c0_g1_i1	Glyma.15G027900.5	95.38461538	150	6	207	2079	1	1879	0	2579

TRINITY_DN12751_c0_g1_i3	Glyma.07G132100.1	100	136	4	159	2036	1929	64	0	2604

TRINITY_DN13832_c0_g2_i2	Glyma.13G151100.2	97.69357496	121	1	313	2193	1878	1	0	2784

TRINITY_DN12709_c0_g1_i3	Glyma.13G119100.1	96.35258359	113	2	152	2029	1	1881	0	2828

TRINITY_DN14378_c0_g2_i1	Glyma.14G082600.1	95.66326531	346	24	759	2617	1040	2910	0	1362

TRINITY_DN14378_c0_g2_i1	Glyma.14G082600.2	97.43589744	346	24	759	2617	1040	2910	0	1362

TRINITY_DN16372_c0_g1_i3	Glyma.03G101800.1	100	326	6	152	2029	1875	1	0	1620

TRINITY_DN16372_c0_g1_i6	Glyma.03G101800.1	98.30508475	326	6	609	2486	1875	1	0	1620

TRINITY_DN15369_c0_g2_i4	Glyma.03G171100.1	100	289	6	6	1882	1877	1	0	1832

TRINITY_DN13371_c0_g1_i3	Glyma.06G102500.6	100	276	17	914	2783	1919	56	0	1775

TRINITY_DN13371_c0_g1_i3	Glyma.06G102500.1	96.66666667	276	17	914	2783	1919	56	0	1775

TRINITY_DN13371_c0_g1_i3	Glyma.06G102500.5	96.66666667	276	17	914	2783	1919	56	0	1775

TRINITY_DN13371_c0_g1_i3	Glyma.06G102500.3	96.66666667	276	17	914	2783	1919	56	0	1775

TRINITY_DN13371_c0_g1_i3	Glyma.06G102500.4	99.10714286	276	17	914	2783	1919	56	0	1775

TRINITY_DN13371_c0_g1_i3	Glyma.06G102500.2	100	276	17	914	2783	1919	56	0	1775

TRINITY_DN15305_c1_g2_i1	Glyma.13G330400.1	97.39130435	254	27	258	2110	2903	1036	0	1812

TRINITY_DN12315_c0_g1_i1	Glyma.10G006100.1	95.50561798	224	10	315	2183	1878	1	0	2146

TRINITY_DN12315_c0_g1_i2	Glyma.10G006100.1	100	224	10	315	2183	1878	1	0	2146

TRINITY_DN12315_c0_g1_i3	Glyma.10G006100.1	97.87234043	224	10	315	2183	1878	1	0	2146

TRINITY_DN15066_c0_g1_i1	Glyma.12G075200.1	98.18181818	200	19	121	1998	1	1845	0	2124

TRINITY_DN16240_c0_g1_i1	Glyma.19G202400.1	96.57534247	189	8	322	2197	1870	1	0	2335

TRINITY_DN16240_c0_g1_i1	Glyma.19G202400.2	100	189	8	322	2197	1870	1	0	2335

TRINITY_DN53_c0_g1_i1	Glyma.07G121500.1	96.15384615	184	5	266	2143	1875	1	0	2407

TRINITY_DN13867_c0_g1_i1	Glyma.07G032500.3	98.39572193	166	29	164	2006	1	1852	0	2132

TRINITY_DN9062_c0_g1_i1	Glyma.04G235200.1	97.56097561	165	18	125	1997	93	1956	0	2409

TRINITY_DN16704_c0_g1_i1	Glyma.15G149000.1	100	163	7	1	1879	2670	807	0	2459

TRINITY_DN15517_c0_g2_i2	Glyma.08G313400.1	97.08737864	159	4	968	2846	2702	827	0	2558

TRINITY_DN15517_c0_g2_i2	Glyma.08G313400.2	96.03960396	159	4	968	2846	2615	740	0	2558

TRINITY_DN15517_c0_g2_i2	Glyma.08G313400.4	96.16613419	159	4	968	2846	2612	737	0	2558

TRINITY_DN15517_c0_g2_i2	Glyma.08G313400.3	96.16613419	159	4	968	2846	2525	650	0	2558

TRINITY_DN4267_c0_g1_i1	Glyma.10G095200.1	100	152	15	1	1865	3098	4956	0	2398

TRINITY_DN12751_c0_g1_i3	Glyma.18G180900.1	95.97315436	151	7	165	2041	1923	62	0	2499

TRINITY_DN12751_c0_g1_i3	Glyma.18G180900.6	95.61403509	151	7	165	2041	1917	56	0	2499

TRINITY_DN15685_c0_g1_i1	Glyma.10G150000.1	95.4954955	99	0	200	2079	3357	1478	0	2924

TRINITY_DN15685_c0_g1_i1	Glyma.10G150000.2	98.13084112	99	0	200	2079	2508	629	0	2924

TRINITY_DN15685_c0_g1_i1	Glyma.20G238400.1	95.78947368	97	0	200	2079	3393	1514	0	2935

TRINITY_DN15685_c0_g1_i1	Glyma.20G238400.2	95.78947368	97	0	200	2079	3213	1334	0	2935

TRINITY_DN14164_c0_g1_i3	Glyma.20G179900.1	99.00990099	92	15	111	1977	1	1836	0	2593

TRINITY_DN12827_c0_g1_i1	Glyma.13G137200.4	99.18032787	351	21	422	2272	1872	1	0	1299

TRINITY_DN12827_c0_g1_i1	Glyma.13G137200.5	99.18032787	351	21	422	2272	1872	1	0	1299

TRINITY_DN12827_c0_g1_i1	Glyma.13G137200.6	99.17355372	351	21	422	2272	1872	1	0	1299

TRINITY_DN12827_c0_g1_i1	Glyma.13G137200.1	99.18032787	351	21	422	2272	1872	1	0	1299

TRINITY_DN12827_c0_g1_i3	Glyma.13G137200.4	100	351	21	422	2272	1872	1	0	1299

TRINITY_DN12827_c0_g1_i3	Glyma.13G137200.5	100	351	21	422	2272	1872	1	0	1299

TRINITY_DN12827_c0_g1_i3	Glyma.13G137200.6	100	351	21	422	2272	1872	1	0	1299

TRINITY_DN12827_c0_g1_i3	Glyma.13G137200.1	100	351	21	422	2272	1872	1	0	1299

TRINITY_DN12827_c0_g1_i1	Glyma.13G137200.3	99.18032787	351	22	422	2272	1869	1	0	1280

TRINITY_DN12827_c0_g1_i1	Glyma.13G137200.2	99.17355372	351	22	422	2272	1869	1	0	1280

TRINITY_DN12827_c0_g1_i3	Glyma.13G137200.3	100	351	22	422	2272	1869	1	0	1280

TRINITY_DN12827_c0_g1_i3	Glyma.13G137200.2	100	351	22	422	2272	1869	1	0	1280

TRINITY_DN16211_c1_g2_i1	Glyma.17G003400.1	96	312	25	155	2016	3	1858	0	1483

TRINITY_DN16211_c1_g2_i1	Glyma.07G270600.1	99.14529915	312	28	155	2016	3	1858	0	1483

TRINITY_DN15369_c0_g2_i4	Glyma.19G172200.1	98.8372093	303	4	6	1882	1877	1	0	1766

TRINITY_DN16372_c0_g1_i1	Glyma.03G101800.1	100	292	7	152	2026	1875	1	0	1801

TRINITY_DN8949_c0_g1_i1	Glyma.11G049800.7	100	284	49	184	2018	1861	16	0	1399

TRINITY_DN15369_c0_g2_i1	Glyma.03G171100.1	100	265	4	6	1882	1877	1	0	1977

TRINITY_DN16173_c1_g1_i3	Glyma.13G076700.1	95.23809524	231	11	408	2276	1866	1	0	2041

TRINITY_DN12315_c0_g1_i1	Glyma.02G002900.2	96.77419355	219	8	315	2183	1878	1	0	2185

TRINITY_DN12315_c0_g1_i1	Glyma.02G002900.1	97.4137931	219	8	315	2183	1878	1	0	2185

TRINITY_DN12315_c0_g1_i2	Glyma.02G002900.2	100	219	8	315	2183	1878	1	0	2185

TRINITY_DN12315_c0_g1_i2	Glyma.02G002900.1	100	219	8	315	2183	1878	1	0	2185

TRINITY_DN12315_c0_g1_i3	Glyma.02G002900.2	98.61591696	219	8	315	2183	1878	1	0	2185

TRINITY_DN12315_c0_g1_i3	Glyma.02G002900.1	98.61751152	219	8	315	2183	1878	1	0	2185

TRINITY_DN15066_c0_g1_i1	Glyma.11G144600.1	100	212	22	121	1998	1	1830	0	1973

TRINITY_DN13713_c0_g1_i2	Glyma.15G042700.1	100	210	10	392	2254	124	1992	0	2139

TRINITY_DN13713_c0_g1_i4	Glyma.15G042700.1	99.15611814	210	10	380	2242	124	1992	0	2139

TRINITY_DN14983_c0_g1_i1	Glyma.05G220100.1	95.58823529	157	9	237	2108	1	1872	0	2510

TRINITY_DN14983_c0_g1_i1	Glyma.05G220100.2	95.58823529	157	9	237	2108	1	1872	0	2510

TRINITY_DN37246_c0_g1_i1	Glyma.18G073600.2	97.56097561	137	0	1	1879	937	2815	0	2712

TRINITY_DN6849_c0_g3_i1	Glyma.11G111200.1	98.61111111	137	10	155	2024	2103	237	0	2575

TRINITY_DN15970_c0_g2_i10	Glyma.01G099800.3	97.2972973	122	0	246	2124	23	1901	0	2795

TRINITY_DN15970_c0_g2_i4	Glyma.01G099800.3	95.23809524	122	0	246	2124	23	1901	0	2795

TRINITY_DN15950_c0_g1_i2	Glyma.15G013500.1	99.0990991	306	56	3	1799	166	2007	0	1016

TRINITY_DN15950_c0_g1_i2	Glyma.15G013500.2	99.0990991	306	56	3	1799	166	2007	0	1016

TRINITY_DN13371_c0_g1_i3	Glyma.04G100700.1	97.08222812	277	14	914	2783	1919	56	0	1792

TRINITY_DN13371_c0_g1_i3	Glyma.04G100700.2	100	277	14	914	2783	1919	56	0	1792

TRINITY_DN15369_c0_g2_i1	Glyma.19G172200.1	100	270	2	6	1882	1877	1	0	1960

TRINITY_DN16415_c1_g1_i13	Glyma.13G327000.2	100	268	7	2350	4222	1873	1	0	1919

TRINITY_DN16415_c1_g1_i6	Glyma.13G327000.2	99.49748744	268	7	2256	4128	1873	1	0	1919

TRINITY_DN16257_c1_g1_i9	Glyma.12G191800.1	98.69281046	254	12	193	2059	2219	356	0	1899

TRINITY_DN16173_c1_g1_i4	Glyma.13G076700.1	99.06542056	243	9	408	2276	1866	1	0	1986

TRINITY_DN16173_c1_g1_i6	Glyma.13G076700.1	100	225	8	408	2279	1866	1	0	2106

TRINITY_DN6018_c0_g2_i1	Glyma.20G171200.1	100	220	14	160	2031	100	1959	0	2095

TRINITY_DN13713_c0_g1_i1	Glyma.15G042700.7	96.09756098	210	10	420	2281	86	1953	0	2137

TRINITY_DN13713_c0_g1_i3	Glyma.15G042700.7	95.65217391	210	10	137	1998	86	1953	0	2137

TRINITY_DN11135_c0_g1_i1	Glyma.20G200700.1	100	171	7	586	2458	1873	1	0	2457

TRINITY_DN11135_c0_g1_i3	Glyma.20G200700.1	100	171	7	586	2458	1873	1	0	2457

TRINITY_DN12827_c0_g1_i1	Glyma.19G178900.8	99.17355372	167	14	416	2272	1872	1	0	2368

TRINITY_DN12827_c0_g1_i1	Glyma.19G178900.4	99.18032787	167	14	416	2272	1872	1	0	2368

TRINITY_DN12827_c0_g1_i1	Glyma.19G178900.5	98.96907216	167	14	416	2272	1872	1	0	2368

TRINITY_DN12827_c0_g1_i1	Glyma.19G178900.3	99.16666667	167	14	416	2272	1872	1	0	2368

TRINITY_DN12827_c0_g1_i1	Glyma.19G178900.6	99.17355372	167	14	416	2272	1872	1	0	2368

TRINITY_DN12827_c0_g1_i1	Glyma.19G178900.1	99.16666667	167	14	416	2272	1872	1	0	2368

TRINITY_DN12827_c0_g1_i1	Glyma.19G178900.7	99.17355372	167	14	416	2272	1872	1	0	2368

TRINITY_DN12827_c0_g1_i1	Glyma.19G178900.9	99.18032787	167	14	416	2272	1872	1	0	2368

TRINITY_DN12827_c0_g1_i1	Glyma.19G178900.2	99.18032787	167	14	416	2272	1872	1	0	2368

TRINITY_DN12827_c0_g1_i3	Glyma.19G178900.8	100	167	14	416	2272	1872	1	0	2368

TRINITY_DN12827_c0_g1_i3	Glyma.19G178900.4	98.98534386	167	14	416	2272	1872	1	0	2368

TRINITY_DN12827_c0_g1_i3	Glyma.19G178900.5	100	167	14	416	2272	1872	1	0	2368

TRINITY_DN12827_c0_g1_i3	Glyma.19G178900.3	100	167	14	416	2272	1872	1	0	2368

TRINITY_DN12827_c0_g1_i3	Glyma.19G178900.6	100	167	14	416	2272	1872	1	0	2368

TRINITY_DN12827_c0_g1_i3	Glyma.19G178900.1	100	167	14	416	2272	1872	1	0	2368

TRINITY_DN12827_c0_g1_i3	Glyma.19G178900.7	100	167	14	416	2272	1872	1	0	2368

TRINITY_DN12827_c0_g1_i3	Glyma.19G178900.9	100	167	14	416	2272	1872	1	0	2368

TRINITY_DN12827_c0_g1_i3	Glyma.19G178900.2	100	167	14	416	2272	1872	1	0	2368

TRINITY_DN16457_c0_g1_i1	Glyma.01G244400.1	95.05208333	143	4	5	1881	1745	3618	0	2643

TRINITY_DN14238_c0_g2_i3	Glyma.15G066700.4	97.61904762	137	4	311	2183	1	1876	0	2663

TRINITY_DN14238_c0_g2_i3	Glyma.15G066700.2	97.61904762	137	4	311	2183	1	1876	0	2663

TRINITY_DN14238_c0_g2_i4	Glyma.15G066700.4	98.65771812	137	4	91	1963	1	1876	0	2663

TRINITY_DN14238_c0_g2_i4	Glyma.15G066700.2	98.64864865	137	4	91	1963	1	1876	0	2663

TRINITY_DN14238_c0_g2_i3	Glyma.13G247300.4	97.63033175	125	4	311	2183	1	1876	0	2730

TRINITY_DN14238_c0_g2_i3	Glyma.13G247300.2	97.61904762	125	4	311	2183	1	1876	0	2730

TRINITY_DN14238_c0_g2_i4	Glyma.13G247300.4	97.61904762	125	4	91	1963	1	1876	0	2730

TRINITY_DN14238_c0_g2_i4	Glyma.13G247300.2	97.61904762	125	4	91	1963	1	1876	0	2730

TRINITY_DN13543_c1_g2_i1	Glyma.14G166100.1	96.15384615	124	11	537	2411	1857	1	0	2627

TRINITY_DN13764_c0_g1_i3	Glyma.08G022300.1	100	122	1	124	1998	1	1878	0	2772

TRINITY_DN11833_c0_g1_i4	Glyma.10G292100.1	99.73474801	122	11	177	2044	1	1853	0	2566

TRINITY_DN12647_c0_g1_i3	Glyma.12G241700.3	100	71	1	180	2057	1	1875	0	3055

TRINITY_DN14522_c0_g1_i1	Glyma.18G138000.1	96.57258065	284	16	357	2218	1865	1	0	1718

TRINITY_DN15772_c0_g1_i2	Glyma.13G050200.1	100	245	7	143	2014	1	1875	0	2063

TRINITY_DN15772_c0_g1_i9	Glyma.13G050200.1	100	245	7	158	2029	1	1875	0	2063

TRINITY_DN38463_c0_g1_i1	Glyma.19G263900.1	95.1048951	178	14	1	1871	2596	4457	0	2344

TRINITY_DN38463_c0_g1_i1	Glyma.02G000100.2	100	173	11	1	1871	2596	4457	0	2372

TRINITY_DN38463_c0_g1_i1	Glyma.02G000100.3	100	173	11	1	1871	2596	4457	0	2372

TRINITY_DN38463_c0_g1_i1	Glyma.02G000100.1	95.1048951	173	11	1	1871	2596	4457	0	2372

TRINITY_DN15710_c0_g1_i1	Glyma.U033500.4	98.77300613	156	8	182	2053	1869	1	0	2518

TRINITY_DN15710_c0_g1_i1	Glyma.U033500.2	98.77300613	156	8	182	2053	1869	1	0	2518

TRINITY_DN4171_c0_g1_i1	Glyma.20G248800.1	96.2962963	140	0	72	1948	29	1905	0	2691

TRINITY_DN4171_c0_g1_i1	Glyma.20G248800.2	97.87234043	134	1	72	1948	29	1884	0	2588

TRINITY_DN15538_c0_g1_i7	Glyma.08G333900.3	97.97979798	123	0	443	2319	1	1877	0	2785

TRINITY_DN15538_c0_g1_i7	Glyma.08G333900.1	95.23809524	123	0	443	2319	1	1877	0	2785

TRINITY_DN15538_c0_g1_i7	Glyma.08G333900.2	99.37888199	123	0	443	2319	1	1877	0	2785

TRINITY_DN15538_c0_g1_i9	Glyma.08G333900.3	100	123	0	443	2319	1	1877	0	2785

TRINITY_DN15538_c0_g1_i9	Glyma.08G333900.1	100	123	0	443	2319	1	1877	0	2785

TRINITY_DN15538_c0_g1_i9	Glyma.08G333900.2	100	123	0	443	2319	1	1877	0	2785

TRINITY_DN15538_c0_g1_i7	Glyma.18G067000.3	100	117	0	443	2319	1	1877	0	2819

TRINITY_DN15538_c0_g1_i7	Glyma.18G067000.2	99.57446809	117	0	443	2319	1	1877	0	2819

TRINITY_DN15538_c0_g1_i7	Glyma.18G067000.1	95.96774194	117	0	443	2319	1	1877	0	2819

TRINITY_DN15538_c0_g1_i9	Glyma.18G067000.3	100	117	0	443	2319	1	1877	0	2819

TRINITY_DN15538_c0_g1_i9	Glyma.18G067000.2	100	117	0	443	2319	1	1877	0	2819

TRINITY_DN15538_c0_g1_i9	Glyma.18G067000.1	100	117	0	443	2319	1	1877	0	2819

TRINITY_DN14967_c1_g1_i1	Glyma.01G239200.1	100	102	0	383	2259	244	2120	0	2902

TRINITY_DN16271_c0_g1_i3	Glyma.08G361500.2	97.5	79	0	511	2387	5393	3517	0	3029

TRINITY_DN15750_c1_g1_i3	Glyma.07G010400.1	98.03921569	432	33	824	2674	2004	148	0	787

TRINITY_DN15772_c0_g1_i5	Glyma.19G039100.1	95.78313253	231	3	122	1993	1	1875	0	2152

TRINITY_DN15565_c0_g1_i3	Glyma.09G044300.4	95.18900344	217	16	775	2638	1	1855	0	2049

TRINITY_DN15565_c0_g1_i4	Glyma.09G044300.4	95.52238806	217	16	788	2651	1	1855	0	2049

TRINITY_DN13713_c0_g1_i2	Glyma.15G042700.8	99.12280702	210	9	392	2254	124	1989	0	2152

TRINITY_DN13713_c0_g1_i4	Glyma.15G042700.8	100	210	9	380	2242	124	1989	0	2152

TRINITY_DN16332_c0_g3_i4	Glyma.17G239500.2	100	201	6	911	2782	2832	961	0	2300

TRINITY_DN16332_c0_g3_i5	Glyma.17G239500.2	100	201	6	556	2427	2832	961	0	2300

TRINITY_DN12827_c0_g1_i1	Glyma.03G178100.1	99.18032787	169	13	417	2272	1865	1	0	2327

TRINITY_DN12827_c0_g1_i3	Glyma.03G178100.1	100	169	13	417	2272	1865	1	0	2327

TRINITY_DN12827_c0_g1_i1	Glyma.03G178100.2	99.18032787	168	15	417	2272	1862	1	0	2314

TRINITY_DN12827_c0_g1_i3	Glyma.03G178100.2	100	168	15	417	2272	1862	1	0	2314

TRINITY_DN14005_c0_g1_i1	Glyma.13G346300.9	95.38461538	167	7	207	2079	1	1864	0	2442

TRINITY_DN14005_c0_g1_i1	Glyma.13G346300.7	97.02380952	167	7	207	2079	1	1864	0	2442

TRINITY_DN14005_c0_g1_i1	Glyma.13G346300.8	98.95833333	167	7	207	2079	1	1864	0	2442

TRINITY_DN14005_c0_g1_i1	Glyma.13G346300.4	97.14285714	167	7	207	2079	1	1864	0	2442

TRINITY_DN14005_c0_g1_i1	Glyma.13G346300.1	100	167	7	207	2079	1	1864	0	2442

TRINITY_DN15878_c0_g2_i1	Glyma.07G048500.2	97.10982659	141	5	60	1926	1867	1	0	2567

TRINITY_DN15878_c0_g2_i1	Glyma.07G048500.1	98.60788863	141	5	60	1926	1867	1	0	2567

TRINITY_DN15878_c0_g2_i1	Glyma.07G048500.3	97.11538462	141	5	60	1926	1867	1	0	2567

TRINITY_DN15878_c0_g2_i1	Glyma.07G048500.5	97.11538462	141	5	60	1926	1867	1	0	2567

TRINITY_DN15878_c0_g2_i1	Glyma.07G048500.6	97.11934156	141	5	60	1926	1867	1	0	2567

TRINITY_DN15878_c0_g2_i1	Glyma.07G048500.7	100	141	5	60	1926	1867	1	0	2567

TRINITY_DN15878_c0_g2_i1	Glyma.07G048500.4	96.71052632	141	5	60	1926	1867	1	0	2567

TRINITY_DN7482_c0_g1_i1	Glyma.20G056500.1	100	139	16	572	2443	1857	1	0	2545

TRINITY_DN7482_c0_g1_i1	Glyma.20G056500.2	96.27329193	139	16	572	2443	1857	1	0	2545

TRINITY_DN7482_c0_g1_i1	Glyma.20G056500.3	100	139	16	572	2443	1857	1	0	2545

TRINITY_DN15878_c0_g2_i1	Glyma.16G017400.5	100	138	7	60	1926	1864	1	0	2564

TRINITY_DN15878_c0_g2_i1	Glyma.16G017400.1	100	138	7	60	1926	1864	1	0	2564

TRINITY_DN15878_c0_g2_i1	Glyma.16G017400.4	95.98108747	138	7	60	1926	1864	1	0	2564

TRINITY_DN15878_c0_g2_i1	Glyma.16G017400.6	97.03703704	138	7	60	1926	1864	1	0	2564

TRINITY_DN15878_c0_g2_i1	Glyma.16G017400.3	96.52173913	138	7	60	1926	1864	1	0	2564

TRINITY_DN15878_c0_g2_i1	Glyma.16G017400.2	96.52173913	138	7	60	1926	1864	1	0	2564

TRINITY_DN12878_c0_g1_i1	Glyma.18G025500.5	100	133	0	752	2627	3	1878	0	2728

TRINITY_DN14238_c0_g2_i1	Glyma.08G060100.1	100	129	0	189	2064	258	2133	0	2750

TRINITY_DN9577_c0_g1_i1	Glyma.20G238800.2	100	123	14	1	1852	455	2321	0	2569

TRINITY_DN9577_c0_g1_i1	Glyma.20G238800.3	100	123	14	1	1852	455	2321	0	2569

TRINITY_DN13764_c0_g1_i3	Glyma.05G216400.1	95.83333333	119	2	124	1998	1	1875	0	2793

TRINITY_DN13088_c0_g1_i2	Glyma.20G156500.3	95.60632689	117	3	606	2475	1	1876	0	2778

TRINITY_DN13088_c0_g1_i2	Glyma.20G156500.4	97.01230228	117	3	606	2475	1	1876	0	2778

TRINITY_DN13088_c0_g1_i2	Glyma.20G156500.2	96.32183908	117	3	606	2475	1	1876	0	2778

TRINITY_DN19670_c0_g1_i1	Glyma.10G258000.3	97.33333333	107	2	286	2160	1875	1	0	2859

TRINITY_DN19670_c0_g1_i1	Glyma.10G258000.2	97.34848485	107	2	286	2160	1875	1	0	2859

TRINITY_DN19670_c0_g1_i1	Glyma.10G258000.1	97.34513274	107	2	286	2160	1875	1	0	2859

TRINITY_DN19670_c0_g1_i1	Glyma.10G258000.4	97.34042553	107	2	286	2160	1875	1	0	2859

TRINITY_DN13088_c0_g1_i2	Glyma.10G238100.2	96.77419355	106	3	606	2475	1	1876	0	2839

TRINITY_DN13088_c0_g1_i2	Glyma.10G238100.3	99.44134078	106	3	606	2475	1	1876	0	2839

TRINITY_DN8931_c0_g1_i1	Glyma.05G185300.1	95.67039106	101	0	1	1876	6723	8598	0	2905

TRINITY_DN8931_c0_g1_i1	Glyma.08G143200.2	95.69892473	100	0	1	1876	7779	9654	0	2911

TRINITY_DN8931_c0_g1_i1	Glyma.08G143200.1	95.66787004	100	0	1	1876	7779	9654	0	2911

TRINITY_DN8931_c0_g1_i1	Glyma.08G143200.4	95.67567568	100	0	1	1876	7779	9654	0	2911

TRINITY_DN12944_c0_g2_i1	Glyma.12G072200.7	100	350	38	181	2019	1848	1	0	1116

TRINITY_DN12944_c0_g2_i1	Glyma.12G072200.5	100	350	38	181	2019	1848	1	0	1116

TRINITY_DN12944_c0_g2_i1	Glyma.12G072200.6	97.265625	350	38	181	2019	1848	1	0	1116

TRINITY_DN12944_c0_g2_i1	Glyma.12G072200.4	95.62043796	350	38	181	2019	1848	1	0	1116

TRINITY_DN12944_c0_g2_i1	Glyma.12G072200.1	100	350	38	181	2019	1848	1	0	1116

TRINITY_DN12944_c0_g2_i1	Glyma.12G072200.2	100	350	38	181	2019	1848	1	0	1116

TRINITY_DN12944_c0_g2_i1	Glyma.12G072200.3	100	350	38	181	2019	1848	1	0	1116

TRINITY_DN15139_c0_g3_i1	Glyma.01G157900.2	97.6744186	301	22	263	2123	2791	927	0	1640

TRINITY_DN15139_c0_g3_i1	Glyma.01G157900.1	95.22012579	301	22	263	2123	2791	927	0	1640

TRINITY_DN16061_c0_g1_i2	Glyma.18G224200.1	100	247	9	548	2417	1993	127	0	2010

TRINITY_DN15772_c0_g1_i5	Glyma.13G050200.1	95.78059072	227	3	122	1993	1	1875	0	2185

TRINITY_DN16251_c0_g3_i7	Glyma.15G167100.1	100	222	13	548	2415	2332	472	0	2097

TRINITY_DN16251_c0_g3_i7	Glyma.15G167100.3	96.70781893	222	13	548	2415	2290	430	0	2097

TRINITY_DN16251_c0_g3_i7	Glyma.15G167100.4	95.32846715	221	14	548	2415	2311	472	0	1967

TRINITY_DN16251_c0_g3_i7	Glyma.15G167100.5	98.38709677	220	15	548	2415	2329	472	0	2089

TRINITY_DN16251_c0_g3_i7	Glyma.15G167100.2	97.8021978	220	15	548	2415	2287	430	0	2089

TRINITY_DN13713_c0_g1_i1	Glyma.15G042700.6	97.43589744	210	9	420	2281	86	1950	0	2150

TRINITY_DN13713_c0_g1_i3	Glyma.15G042700.6	98.14814815	210	9	137	1998	86	1950	0	2150

TRINITY_DN4019_c0_g2_i1	Glyma.14G087900.1	95.82577132	206	37	419	2236	1839	1	0	1720

TRINITY_DN4019_c0_g2_i1	Glyma.14G087900.2	97.95918367	206	37	419	2236	1839	1	0	1720

TRINITY_DN15008_c0_g1_i1	Glyma.05G206400.1	99.6763754	183	25	85	1919	37	1896	0	2093

TRINITY_DN11135_c0_g1_i1	Glyma.10G190100.1	100	172	5	586	2458	1867	1	0	2446

TRINITY_DN11135_c0_g1_i3	Glyma.10G190100.1	97.36842105	172	5	586	2458	1867	1	0	2446

TRINITY_DN11708_c0_g1_i1	Glyma.04G223800.4	96.89655172	159	4	307	2181	1866	1	0	2523

TRINITY_DN11708_c0_g1_i1	Glyma.04G223800.1	97.43589744	159	4	307	2181	1866	1	0	2523

TRINITY_DN11708_c0_g1_i1	Glyma.04G223800.3	95.26315789	159	4	307	2181	1866	1	0	2523

TRINITY_DN11708_c0_g1_i1	Glyma.04G223800.2	95.9446093	159	4	307	2181	1866	1	0	2523

TRINITY_DN11708_c0_g1_i2	Glyma.04G223800.4	100	159	4	307	2181	1866	1	0	2523

TRINITY_DN11708_c0_g1_i2	Glyma.04G223800.1	100	159	4	307	2181	1866	1	0	2523

TRINITY_DN11708_c0_g1_i2	Glyma.04G223800.3	96.06126915	159	4	307	2181	1866	1	0	2523

TRINITY_DN11708_c0_g1_i2	Glyma.04G223800.2	96.06164384	159	4	307	2181	1866	1	0	2523

TRINITY_DN11708_c0_g1_i3	Glyma.04G223800.4	100	159	4	307	2181	1866	1	0	2523

TRINITY_DN11708_c0_g1_i3	Glyma.04G223800.1	95.1986755	159	4	307	2181	1866	1	0	2523

TRINITY_DN11708_c0_g1_i3	Glyma.04G223800.3	95.2	159	4	307	2181	1866	1	0	2523

TRINITY_DN11708_c0_g1_i3	Glyma.04G223800.2	95.2	159	4	307	2181	1866	1	0	2523

TRINITY_DN1095_c0_g2_i1	Glyma.07G166300.2	100	156	10	760	2614	1873	1	0	2457

TRINITY_DN119_c0_g1_i1	Glyma.03G232200.1	98.88888889	154	7	912	2786	1859	1	0	2507

TRINITY_DN1095_c0_g1_i1	Glyma.07G166300.2	100	154	17	760	2598	1873	1	0	2364

TRINITY_DN15950_c0_g3_i1	Glyma.15G013500.1	95.16129032	154	21	3	1856	166	2007	0	2261

TRINITY_DN15950_c0_g3_i1	Glyma.15G013500.2	95.16129032	154	21	3	1856	166	2007	0	2261

TRINITY_DN12469_c0_g1_i1	Glyma.02G236600.4	100	150	4	169	2043	1	1866	0	2573

TRINITY_DN12469_c0_g1_i1	Glyma.02G236600.3	98.7012987	150	4	169	2043	1	1866	0	2573

TRINITY_DN12469_c0_g1_i1	Glyma.02G236600.1	97.99196787	150	4	169	2043	1	1866	0	2573

TRINITY_DN12469_c0_g1_i2	Glyma.02G236600.4	96.3963964	150	4	187	2061	1	1866	0	2573

TRINITY_DN12469_c0_g1_i2	Glyma.02G236600.3	96.3963964	150	4	187	2061	1	1866	0	2573

TRINITY_DN12469_c0_g1_i2	Glyma.02G236600.1	98.5915493	150	4	187	2061	1	1866	0	2573

TRINITY_DN15710_c0_g1_i1	Glyma.12G074600.8	95.85635359	143	2	182	2053	1872	1	0	2632

TRINITY_DN15710_c0_g1_i1	Glyma.12G074600.9	100	143	2	182	2053	1872	1	0	2632

TRINITY_DN9577_c0_g1_i1	Glyma.10G149400.1	100	139	11	1	1851	506	2373	0	2492

TRINITY_DN3810_c0_g1_i1	Glyma.14G021300.1	100	136	3	36	1907	872	2745	0	2684

TRINITY_DN13543_c1_g2_i1	Glyma.13G084300.1	96.15384615	130	2	537	2411	1866	1	0	2684

TRINITY_DN15970_c0_g2_i10	Glyma.01G099800.4	100	121	0	250	2124	3	1877	0	2793

TRINITY_DN15970_c0_g2_i4	Glyma.01G099800.4	100	121	0	250	2124	3	1877	0	2793

TRINITY_DN16091_c0_g1_i1	Glyma.10G218800.1	98.95833333	108	0	480	2354	1	1875	0	2865

TRINITY_DN19670_c0_g1_i1	Glyma.20G133100.2	97.33333333	104	1	286	2160	1872	1	0	2867

TRINITY_DN19670_c0_g1_i1	Glyma.20G133100.4	97.33333333	104	1	286	2160	1872	1	0	2867

TRINITY_DN19670_c0_g1_i1	Glyma.20G133100.3	97.33606557	104	1	286	2160	1872	1	0	2867

TRINITY_DN16091_c0_g1_i1	Glyma.20G173000.1	100	92	0	480	2354	1	1875	0	2953

TRINITY_DN16401_c1_g2_i8	Glyma.13G076200.1	100	339	41	38	1865	1939	80	0	1195

TRINITY_DN16444_c0_g1_i7	Glyma.13G160000.1	99.11504425	325	20	375	2234	1062	2926	0	1511

TRINITY_DN8949_c0_g1_i1	Glyma.11G049800.2	100	284	47	184	2018	1855	16	0	1415

TRINITY_DN16257_c1_g1_i12	Glyma.12G192100.1	100	249	20	184	2039	2231	376	0	1849

TRINITY_DN12569_c0_g1_i3	Glyma.15G061700.1	96.42857143	214	22	372	2214	218	2084	0	2030

TRINITY_DN14326_c0_g3_i1	Glyma.01G169000.1	97.83783784	204	15	256	2113	126	1965	0	2008

TRINITY_DN16173_c1_g1_i6	Glyma.13G076800.1	100	192	10	408	2279	1857	1	0	2274

TRINITY_DN16193_c0_g1_i1	Glyma.13G178100.2	100	191	48	1	1847	1810	6	0	1783

TRINITY_DN16193_c0_g1_i1	Glyma.13G178100.3	96.18320611	191	48	1	1847	1810	6	0	1783

TRINITY_DN16193_c0_g1_i1	Glyma.13G178100.1	99.16839917	191	48	1	1847	1810	6	0	1783

TRINITY_DN16193_c0_g1_i3	Glyma.13G178100.2	97.41935484	191	48	1	1847	1810	6	0	1783

TRINITY_DN16193_c0_g1_i3	Glyma.13G178100.3	99.03846154	191	48	1	1847	1810	6	0	1783

TRINITY_DN16193_c0_g1_i3	Glyma.13G178100.1	96.42857143	191	48	1	1847	1810	6	0	1783

TRINITY_DN16173_c1_g1_i4	Glyma.13G076800.1	95.0877193	183	11	408	2276	1857	1	0	2305

TRINITY_DN12469_c0_g1_i1	Glyma.14G205100.1	97.39776952	175	1	169	2042	1	1871	0	2471

TRINITY_DN12469_c0_g1_i2	Glyma.14G205100.1	98.85057471	175	1	187	2060	1	1871	0	2471

TRINITY_DN16173_c1_g1_i3	Glyma.13G076800.1	100	172	11	408	2276	1857	1	0	2366

TRINITY_DN8984_c0_g1_i1	Glyma.02G098300.1	97.93814433	113	3	102	1975	1868	1	0	2796

TRINITY_DN16059_c0_g3_i1	Glyma.19G170200.1	100	331	15	315	2177	82	1941	0	1476

TRINITY_DN16059_c0_g3_i2	Glyma.19G170200.1	98.6013986	331	15	594	2456	82	1941	0	1476

TRINITY_DN8179_c0_g1_i1	Glyma.01G089300.1	98.00995025	277	31	1	1823	393	2254	0	1530

TRINITY_DN8179_c0_g1_i1	Glyma.01G089300.3	96.44268775	277	31	1	1823	387	2248	0	1530

TRINITY_DN8179_c0_g1_i1	Glyma.01G089300.2	96.44194757	277	31	1	1823	369	2230	0	1530

TRINITY_DN8156_c0_g1_i1	Glyma.13G152900.1	95.23809524	230	43	191	1987	1863	1	0	1629

TRINITY_DN15327_c1_g1_i4	Glyma.10G106600.1	98.64864865	224	0	234	2106	1884	12	0	2218

TRINITY_DN15139_c0_g3_i2	Glyma.01G157900.2	100	200	19	263	2123	2791	927	0	2222

TRINITY_DN15139_c0_g3_i2	Glyma.01G157900.1	95.83333333	200	19	263	2123	2791	927	0	2222

TRINITY_DN9320_c0_g1_i1	Glyma.16G110100.1	96.77419355	197	6	165	2034	87	1956	0	2329

TRINITY_DN9320_c0_g1_i1	Glyma.16G110100.2	100	194	10	165	2034	87	1944	0	2268

TRINITY_DN15327_c1_g1_i2	Glyma.10G106600.1	100	186	0	234	2106	1884	12	0	2429

TRINITY_DN11554_c0_g2_i1	Glyma.13G003700.1	95.83858764	182	4	149	2020	1	1869	0	2418

TRINITY_DN16704_c0_g1_i1	Glyma.09G044100.1	100	164	7	1	1872	2691	835	0	2440

TRINITY_DN13867_c0_g1_i1	Glyma.08G210000.1	100	164	25	164	1994	1	1858	0	2182

TRINITY_DN14432_c0_g1_i2	Glyma.17G233000.1	100	108	14	210	2052	88	1954	0	2628

TRINITY_DN14713_c0_g1_i6	Glyma.04G086300.2	96.04519774	106	7	124	1989	1	1854	0	2704

TRINITY_DN5574_c0_g1_i1	Glyma.08G343500.1	97.72727273	103	10	214	2075	1914	49	0	2772

TRINITY_DN6240_c0_g1_i1	Glyma.03G251100.1	96.50793651	102	0	8	1880	1626	3498	0	2894

TRINITY_DN13596_c0_g1_i2	Glyma.05G016400.1	96.59090909	102	7	2	1871	28	1891	0	2817

TRINITY_DN13596_c0_g1_i2	Glyma.05G016400.2	96.61016949	102	7	2	1871	28	1891	0	2817

TRINITY_DN6240_c0_g1_i1	Glyma.19G248400.1	96.46017699	97	0	8	1880	1626	3498	0	2922

TRINITY_DN6240_c0_g1_i1	Glyma.19G248400.2	96.49122807	97	0	8	1880	1323	3195	0	2922

TRINITY_DN8949_c0_g1_i1	Glyma.01G192300.1	100	294	47	184	2018	1855	16	0	1382

TRINITY_DN15216_c0_g2_i2	Glyma.11G243600.1	100	273	15	71	1933	60	1925	0	1847

TRINITY_DN15216_c0_g2_i2	Glyma.11G243600.3	100	271	16	71	1933	60	1916	0	1801

TRINITY_DN15216_c0_g2_i1	Glyma.11G243600.1	95.6043956	230	15	71	1933	60	1925	0	2085

TRINITY_DN15216_c0_g2_i1	Glyma.11G243600.3	99.72144847	230	16	71	1933	60	1916	0	2028

TRINITY_DN16240_c0_g1_i1	Glyma.03G205000.1	97.97979798	201	9	328	2197	1861	1	0	2259

TRINITY_DN16218_c0_g5_i14	Glyma.10G161400.1	100	197	9	403	2267	1863	1	0	2263

TRINITY_DN16218_c0_g5_i14	Glyma.10G161400.2	95.45454545	197	9	403	2267	1863	1	0	2263

TRINITY_DN9850_c0_g1_i1	Glyma.18G207500.5	100	187	0	199	2070	1	1872	0	2422

TRINITY_DN7559_c0_g1_i1	Glyma.13G141800.4	95.50561798	187	35	477	2280	1867	1	0	1949

TRINITY_DN7559_c0_g1_i1	Glyma.13G141800.1	95.50561798	187	35	477	2280	1867	1	0	1949

TRINITY_DN11573_c0_g1_i1	Glyma.10G295400.2	97.18670077	175	12	413	2260	1863	1	0	2274

TRINITY_DN11573_c0_g1_i1	Glyma.10G295400.1	100	175	12	413	2260	1863	1	0	2274

TRINITY_DN11573_c0_g1_i1	Glyma.10G295400.3	100	175	12	413	2260	1863	1	0	2274

TRINITY_DN15374_c0_g1_i1	Glyma.08G077800.1	98.76033058	142	2	526	2397	1869	1	0	2651

TRINITY_DN15074_c0_g1_i1	Glyma.10G130800.2	98.33333333	140	19	1177	3011	1871	1	0	2436

TRINITY_DN3810_c0_g1_i1	Glyma.14G021300.3	98.27160494	136	1	36	1907	872	2742	0	2697

TRINITY_DN15374_c0_g1_i1	Glyma.05G122800.1	98.76160991	135	2	526	2397	1869	1	0	2689

TRINITY_DN2821_c0_g1_i1	Glyma.17G041700.1	100	135	8	218	2089	177	2020	0	2529

TRINITY_DN2821_c0_g1_i1	Glyma.17G041700.6	99.47368421	135	8	218	2089	177	2020	0	2529

TRINITY_DN2821_c0_g1_i1	Glyma.17G041700.4	95.69892473	135	9	218	2089	177	2017	0	2508

TRINITY_DN2821_c0_g1_i1	Glyma.17G041700.5	96.7032967	135	9	218	2089	177	2017	0	2508

TRINITY_DN15330_c0_g5_i5	Glyma.20G126600.2	98.25581395	128	2	327	2192	1872	1	0	2710

TRINITY_DN15330_c0_g5_i5	Glyma.20G126600.1	98.25581395	128	2	327	2192	1872	1	0	2710

TRINITY_DN11833_c0_g1_i4	Glyma.10G292100.2	99.69040248	122	10	177	2044	1	1847	0	2593

TRINITY_DN15772_c0_g1_i2	Glyma.18G278200.2	100	117	0	143	2014	1	1872	0	2809

TRINITY_DN15772_c0_g1_i2	Glyma.18G278200.1	97.11538462	117	0	143	2014	1	1872	0	2809

TRINITY_DN15772_c0_g1_i2	Glyma.18G278200.3	95.59193955	117	0	143	2014	1	1872	0	2809

TRINITY_DN15772_c0_g1_i9	Glyma.18G278200.2	96.39344262	117	0	158	2029	1	1872	0	2809

TRINITY_DN15772_c0_g1_i9	Glyma.18G278200.1	96.39344262	117	0	158	2029	1	1872	0	2809

TRINITY_DN15772_c0_g1_i9	Glyma.18G278200.3	100	117	0	158	2029	1	1872	0	2809

TRINITY_DN10529_c0_g2_i1	Glyma.08G185700.9	99.6996997	115	0	506	2377	1	1872	0	2820

TRINITY_DN10529_c0_g2_i2	Glyma.08G185700.9	99.73753281	115	0	872	2743	1	1872	0	2820

TRINITY_DN15772_c0_g1_i2	Glyma.08G255300.1	97.43954481	115	0	143	2014	1	1872	0	2820

TRINITY_DN15772_c0_g1_i9	Glyma.08G255300.1	96.83377309	115	0	158	2029	1	1872	0	2820

TRINITY_DN15247_c0_g1_i1	Glyma.02G115600.1	99.69230769	114	1	449	2311	1	1872	0	2767

TRINITY_DN15247_c0_g1_i1	Glyma.01G057500.1	99.25187032	110	1	449	2311	1	1872	0	2789

TRINITY_DN13596_c0_g1_i1	Glyma.05G016400.1	96.61016949	101	6	2	1847	28	1891	0	2678

TRINITY_DN13596_c0_g1_i1	Glyma.05G016400.2	96.59090909	101	6	2	1847	28	1891	0	2678

TRINITY_DN9838_c0_g1_i1	Glyma.07G002300.1	100	83	0	680	2551	1872	1	0	2998

TRINITY_DN9838_c0_g1_i1	Glyma.08G224400.1	100	76	0	680	2551	1872	1	0	3037

TRINITY_DN9294_c0_g1_i1	Glyma.14G129600.1	100	327	49	6	1833	1027	2860	0	1127

TRINITY_DN16059_c0_g3_i1	Glyma.03G168800.1	100	324	24	317	2177	87	1935	0	1454

TRINITY_DN16059_c0_g3_i2	Glyma.03G168800.1	95.4312115	324	24	596	2456	87	1935	0	1454

TRINITY_DN16415_c1_g1_i1	Glyma.13G327000.2	95.76271186	268	7	1810	3675	1866	1	0	1906

TRINITY_DN16415_c1_g1_i10	Glyma.13G327000.2	96.56862745	268	7	1403	3268	1866	1	0	1906

TRINITY_DN16415_c1_g1_i13	Glyma.13G327000.3	97.01492537	268	7	2357	4222	1866	1	0	1906

TRINITY_DN16415_c1_g1_i13	Glyma.13G327000.1	98.80952381	268	7	2357	4222	1866	1	0	1906

TRINITY_DN16415_c1_g1_i15	Glyma.13G327000.2	97.22222222	268	7	1904	3769	1866	1	0	1906

TRINITY_DN16415_c1_g1_i4	Glyma.13G327000.2	95.74468085	268	7	2008	3873	1866	1	0	1906

TRINITY_DN16415_c1_g1_i6	Glyma.13G327000.3	99.49748744	268	7	2263	4128	1866	1	0	1906

TRINITY_DN16415_c1_g1_i6	Glyma.13G327000.1	96.53179191	268	7	2263	4128	1866	1	0	1906

TRINITY_DN16415_c1_g1_i7	Glyma.13G327000.2	100	268	7	1914	3779	1866	1	0	1906

TRINITY_DN15327_c1_g1_i2	Glyma.14G045000.1	97.18670077	211	4	249	2117	1869	1	0	2261

TRINITY_DN12569_c0_g1_i3	Glyma.13G253200.1	96.42857143	206	24	372	2202	221	2084	0	2010

TRINITY_DN15099_c0_g1_i1	Glyma.13G261200.1	99.2	201	12	93	1935	438	2305	0	2141

TRINITY_DN15099_c0_g1_i1	Glyma.13G261200.2	99.2	201	12	93	1935	363	2230	0	2141

TRINITY_DN16061_c0_g1_i1	Glyma.18G224200.1	98.14126394	183	6	332	2197	1995	127	0	2396

TRINITY_DN15428_c0_g1_i2	Glyma.12G235000.1	96.89440994	140	10	874	2728	478	2344	0	2551

TRINITY_DN668_c0_g1_i1	Glyma.02G257600.2	100	139	9	156	1996	1	1868	0	2471

TRINITY_DN11675_c0_g1_i1	Glyma.02G089000.1	100	127	2	2130	3997	1868	1	0	2713

TRINITY_DN11675_c0_g1_i2	Glyma.02G089000.1	97.84946237	127	2	270	2137	1868	1	0	2713

TRINITY_DN11675_c0_g1_i3	Glyma.02G089000.1	100	127	2	207	2074	1868	1	0	2713

TRINITY_DN1464_c0_g1_i1	Glyma.07G086500.2	100	126	0	509	2379	2	1872	0	2758

TRINITY_DN14164_c0_g1_i3	Glyma.20G179900.3	99.57983193	92	14	111	1977	1	1827	0	2636

TRINITY_DN16218_c0_g5_i14	Glyma.19G243600.1	95.06008011	327	15	409	2267	1860	1	0	1506

TRINITY_DN10335_c1_g2_i1	Glyma.02G150600.1	100	256	26	16	1860	19	1878	0	1808

TRINITY_DN15139_c0_g3_i2	Glyma.09G197100.1	95.83333333	247	18	227	2088	2844	989	0	1943

TRINITY_DN15139_c0_g3_i2	Glyma.09G197100.2	95.83333333	247	18	227	2088	2538	683	0	1943

TRINITY_DN9079_c0_g1_i1	Glyma.07G171000.1	100	244	18	247	2106	1	1853	0	1927

TRINITY_DN15369_c0_g2_i4	Glyma.11G140500.1	97.6284585	241	7	17	1882	1866	1	0	2067

TRINITY_DN16251_c0_g3_i6	Glyma.09G060600.1	99.6	219	12	357	2218	2523	659	0	2156

TRINITY_DN16251_c0_g3_i6	Glyma.09G060600.4	96.8503937	219	12	357	2218	2508	644	0	2156

TRINITY_DN16251_c0_g3_i6	Glyma.09G060600.3	100	218	13	357	2218	2502	659	0	2026

TRINITY_DN16251_c0_g3_i6	Glyma.09G060600.2	100	218	13	357	2218	2487	644	0	2026

TRINITY_DN16251_c0_g3_i6	Glyma.09G060600.5	96.66666667	217	14	357	2218	2520	659	0	2148

TRINITY_DN12944_c0_g1_i1	Glyma.U021100.10	100	205	18	181	2040	1848	1	0	2111

TRINITY_DN12944_c0_g1_i1	Glyma.U021100.8	98.78787879	205	18	181	2040	1848	1	0	2111

TRINITY_DN12944_c0_g1_i1	Glyma.U021100.7	98.03921569	205	18	181	2040	1848	1	0	2111

TRINITY_DN12944_c0_g1_i1	Glyma.U021100.5	96.15384615	205	18	181	2040	1848	1	0	2111

TRINITY_DN12944_c0_g1_i1	Glyma.U021100.4	96.05425401	205	18	181	2040	1848	1	0	2111

TRINITY_DN12944_c0_g1_i1	Glyma.U021100.2	96.05425401	205	18	181	2040	1848	1	0	2111

TRINITY_DN12944_c0_g1_i2	Glyma.U021100.10	99.06103286	205	18	181	2040	1848	1	0	2111

TRINITY_DN12944_c0_g1_i2	Glyma.U021100.8	95.52845528	205	18	181	2040	1848	1	0	2111

TRINITY_DN12944_c0_g1_i2	Glyma.U021100.7	95.04950495	205	18	181	2040	1848	1	0	2111

TRINITY_DN12944_c0_g1_i2	Glyma.U021100.5	96.93877551	205	18	181	2040	1848	1	0	2111

TRINITY_DN12944_c0_g1_i2	Glyma.U021100.4	96.93877551	205	18	181	2040	1848	1	0	2111

TRINITY_DN12944_c0_g1_i2	Glyma.U021100.2	95.83333333	205	18	181	2040	1848	1	0	2111

TRINITY_DN12944_c0_g1_i3	Glyma.U021100.10	100	205	18	181	2040	1848	1	0	2111

TRINITY_DN12944_c0_g1_i3	Glyma.U021100.8	100	205	18	181	2040	1848	1	0	2111

TRINITY_DN12944_c0_g1_i3	Glyma.U021100.7	98.27586207	205	18	181	2040	1848	1	0	2111

TRINITY_DN12944_c0_g1_i3	Glyma.U021100.5	99.81238274	205	18	181	2040	1848	1	0	2111

TRINITY_DN12944_c0_g1_i3	Glyma.U021100.4	99.81343284	205	18	181	2040	1848	1	0	2111

TRINITY_DN12944_c0_g1_i3	Glyma.U021100.2	99.81447124	205	18	181	2040	1848	1	0	2111

TRINITY_DN12944_c0_g1_i1	Glyma.12G072200.7	100	204	20	181	2040	1848	1	0	2117

TRINITY_DN12944_c0_g1_i1	Glyma.12G072200.5	97.07865169	204	20	181	2040	1848	1	0	2117

TRINITY_DN12944_c0_g1_i1	Glyma.12G072200.6	95.55353902	204	20	181	2040	1848	1	0	2117

TRINITY_DN12944_c0_g1_i1	Glyma.12G072200.4	96.74556213	204	20	181	2040	1848	1	0	2117

TRINITY_DN12944_c0_g1_i1	Glyma.12G072200.1	96.50793651	204	20	181	2040	1848	1	0	2117

TRINITY_DN12944_c0_g1_i1	Glyma.12G072200.2	100	204	20	181	2040	1848	1	0	2117

TRINITY_DN12944_c0_g1_i1	Glyma.12G072200.3	96.44970414	204	20	181	2040	1848	1	0	2117

TRINITY_DN12944_c0_g1_i2	Glyma.12G072200.7	98.84169884	204	20	181	2040	1848	1	0	2117

TRINITY_DN12944_c0_g1_i2	Glyma.12G072200.5	98.5915493	204	20	181	2040	1848	1	0	2117

TRINITY_DN12944_c0_g1_i2	Glyma.12G072200.6	98.5915493	204	20	181	2040	1848	1	0	2117

TRINITY_DN12944_c0_g1_i2	Glyma.12G072200.4	95.58823529	204	20	181	2040	1848	1	0	2117

TRINITY_DN12944_c0_g1_i2	Glyma.12G072200.1	99.29577465	204	20	181	2040	1848	1	0	2117

TRINITY_DN12944_c0_g1_i2	Glyma.12G072200.2	98.98989899	204	20	181	2040	1848	1	0	2117

TRINITY_DN12944_c0_g1_i2	Glyma.12G072200.3	97.52475248	204	20	181	2040	1848	1	0	2117

TRINITY_DN12944_c0_g1_i3	Glyma.12G072200.7	97.36842105	204	20	181	2040	1848	1	0	2117

TRINITY_DN12944_c0_g1_i3	Glyma.12G072200.5	97.36842105	204	20	181	2040	1848	1	0	2117

TRINITY_DN12944_c0_g1_i3	Glyma.12G072200.6	96.38554217	204	20	181	2040	1848	1	0	2117

TRINITY_DN12944_c0_g1_i3	Glyma.12G072200.4	97.59036145	204	20	181	2040	1848	1	0	2117

TRINITY_DN12944_c0_g1_i3	Glyma.12G072200.1	97.59036145	204	20	181	2040	1848	1	0	2117

TRINITY_DN12944_c0_g1_i3	Glyma.12G072200.2	96.61654135	204	20	181	2040	1848	1	0	2117

TRINITY_DN12944_c0_g1_i3	Glyma.12G072200.3	96.98189135	204	20	181	2040	1848	1	0	2117

TRINITY_DN5343_c0_g1_i1	Glyma.01G087700.1	96.99398798	202	0	265	2134	60	1929	0	2335

TRINITY_DN15327_c1_g1_i4	Glyma.14G045000.1	95	184	2	249	2117	1869	1	0	2422

TRINITY_DN15369_c0_g2_i1	Glyma.18G289600.1	98.83040936	177	5	17	1882	1866	1	0	2422

TRINITY_DN11554_c0_g2_i1	Glyma.20G065900.1	97.37991266	174	6	153	2020	8	1872	0	2444

TRINITY_DN6150_c0_g1_i1	Glyma.05G208600.1	96.08695652	171	7	361	2223	2247	391	0	2377

TRINITY_DN16436_c0_g2_i13	Glyma.11G131600.2	100	169	5	915	2770	1	1868	0	2414

TRINITY_DN16436_c0_g2_i5	Glyma.11G131600.2	97.01492537	162	5	183	2038	1	1868	0	2453

TRINITY_DN11607_c0_g2_i1	Glyma.06G158000.1	97.42647059	125	4	83	1948	1866	1	0	2710

TRINITY_DN2628_c0_g1_i1	Glyma.04G098900.4	100	125	5	179	2044	1	1863	0	2689

TRINITY_DN15034_c0_g1_i1	Glyma.06G292500.3	100	97	2	964	2833	1867	1	0	2896

TRINITY_DN8153_c0_g1_i1	Glyma.08G002000.1	97.6	408	18	274	2136	1863	10	0	1055

TRINITY_DN8153_c0_g1_i1	Glyma.08G002000.2	98.27586207	408	18	274	2136	1863	10	0	1055

TRINITY_DN15369_c0_g2_i4	Glyma.18G289600.1	100	292	3	17	1882	1866	1	0	1796

TRINITY_DN16211_c1_g2_i3	Glyma.15G124600.1	95.18072289	291	17	259	2112	1	1857	0	1664

TRINITY_DN13116_c0_g1_i2	Glyma.13G109600.20	97.86476868	280	10	128	1989	1885	27	0	1790

TRINITY_DN11244_c0_g1_i2	Glyma.16G035400.1	100	250	24	222	2056	1	1865	0	1821

TRINITY_DN16255_c1_g1_i3	Glyma.08G207100.1	99.00990099	224	4	360	2226	1992	126	0	2185

TRINITY_DN16255_c1_g1_i3	Glyma.08G207100.4	99.2	221	5	360	2226	1968	126	0	2047

TRINITY_DN13713_c0_g1_i6	Glyma.15G042700.7	97.24409449	215	12	420	2264	86	1953	0	2098

TRINITY_DN16218_c0_g5_i14	Glyma.20G224100.1	95.45454545	203	7	403	2267	1860	1	0	2242

TRINITY_DN16218_c0_g5_i14	Glyma.20G224100.3	95.45454545	203	7	403	2267	1860	1	0	2242

TRINITY_DN16218_c0_g5_i14	Glyma.20G224100.4	95.45454545	203	7	403	2267	1860	1	0	2242

TRINITY_DN16218_c0_g5_i14	Glyma.20G224100.5	98.63013699	203	7	403	2267	1860	1	0	2242

TRINITY_DN16193_c0_g1_i1	Glyma.08G302400.1	100	187	39	1	1829	1861	33	0	1899

TRINITY_DN16193_c0_g1_i1	Glyma.08G302400.2	100	187	39	1	1829	1861	33	0	1899

TRINITY_DN16193_c0_g1_i3	Glyma.08G302400.1	97	187	39	1	1829	1861	33	0	1899

TRINITY_DN16193_c0_g1_i3	Glyma.08G302400.2	97.41935484	187	39	1	1829	1861	33	0	1899

TRINITY_DN15427_c0_g1_i1	Glyma.13G276500.3	100	183	10	386	2254	1851	1	0	2322

TRINITY_DN15427_c0_g1_i1	Glyma.13G276500.1	100	183	10	386	2254	1851	1	0	2322

TRINITY_DN15427_c0_g1_i1	Glyma.13G276500.4	100	183	10	386	2254	1851	1	0	2322

TRINITY_DN15427_c0_g1_i1	Glyma.13G276500.2	96.61016949	182	19	386	2254	1839	1	0	2250

TRINITY_DN6150_c0_g1_i1	Glyma.08G015200.1	96.08695652	177	7	361	2223	2253	388	0	2412

TRINITY_DN37131_c0_g1_i1	Glyma.07G056400.3	95.45454545	160	6	196	2058	1	1866	0	2507

TRINITY_DN37131_c0_g1_i1	Glyma.07G056400.2	95.45454545	160	6	196	2058	1	1866	0	2507

TRINITY_DN37131_c0_g1_i1	Glyma.07G056400.1	98.21428571	160	6	196	2058	1	1866	0	2507

TRINITY_DN14238_c0_g2_i1	Glyma.07G189200.1	97.61904762	133	0	196	2064	1	1869	0	2715

TRINITY_DN14315_c0_g1_i1	Glyma.01G106600.1	95.34883721	131	0	278	2146	1869	1	0	2726

TRINITY_DN14315_c0_g1_i2	Glyma.01G106600.1	97.5308642	131	0	278	2146	1869	1	0	2726

TRINITY_DN14315_c0_g1_i5	Glyma.01G106600.1	100	131	0	278	2146	1869	1	0	2726

TRINITY_DN15330_c0_g5_i5	Glyma.10G263700.1	98.25528007	130	1	327	2192	1869	1	0	2712

TRINITY_DN14238_c0_g2_i1	Glyma.08G060100.3	100	129	0	196	2064	1	1869	0	2737

TRINITY_DN14238_c0_g2_i1	Glyma.08G060100.2	97.61904762	129	0	196	2064	1	1869	0	2737

TRINITY_DN14315_c0_g1_i1	Glyma.04G108100.1	97.77777778	128	0	278	2146	1869	1	0	2743

TRINITY_DN14315_c0_g1_i2	Glyma.04G108100.1	96.77419355	128	0	278	2146	1869	1	0	2743

TRINITY_DN14315_c0_g1_i5	Glyma.04G108100.1	96.80851064	128	0	278	2146	1869	1	0	2743

TRINITY_DN12467_c0_g1_i1	Glyma.03G102900.1	95	101	0	165	2033	1	1869	0	2892

TRINITY_DN12315_c0_g1_i1	Glyma.10G181400.1	95.85492228	99	1	315	2183	1866	1	0	2883

TRINITY_DN12315_c0_g1_i2	Glyma.10G181400.1	95.50561798	99	1	315	2183	1866	1	0	2883

TRINITY_DN12315_c0_g1_i3	Glyma.10G181400.1	98.69565217	99	1	315	2183	1866	1	0	2883

TRINITY_DN12315_c0_g1_i1	Glyma.20G209000.1	99.31034483	92	3	315	2183	1863	1	0	2904

TRINITY_DN12315_c0_g1_i1	Glyma.20G209000.3	97.05093834	92	3	315	2183	1863	1	0	2904

TRINITY_DN12315_c0_g1_i1	Glyma.20G209000.2	95.8537916	92	3	315	2183	1863	1	0	2904

TRINITY_DN12315_c0_g1_i2	Glyma.20G209000.1	95.50561798	92	3	315	2183	1863	1	0	2904

TRINITY_DN12315_c0_g1_i2	Glyma.20G209000.3	95.50561798	92	3	315	2183	1863	1	0	2904

TRINITY_DN12315_c0_g1_i2	Glyma.20G209000.2	95.50561798	92	3	315	2183	1863	1	0	2904

TRINITY_DN12315_c0_g1_i3	Glyma.20G209000.1	98.69281046	92	3	315	2183	1863	1	0	2904

TRINITY_DN12315_c0_g1_i3	Glyma.20G209000.3	98.69281046	92	3	315	2183	1863	1	0	2904

TRINITY_DN12315_c0_g1_i3	Glyma.20G209000.2	98.69281046	92	3	315	2183	1863	1	0	2904

TRINITY_DN12647_c0_g1_i3	Glyma.09G002600.17	100	80	0	180	2048	1	1869	0	3009

TRINITY_DN12647_c0_g1_i3	Glyma.09G002600.12	100	80	0	180	2048	1	1869	0	3009

TRINITY_DN12647_c0_g1_i3	Glyma.09G002600.11	100	80	0	180	2048	1	1869	0	3009

TRINITY_DN12647_c0_g1_i3	Glyma.09G002600.13	100	80	0	180	2048	1	1869	0	3009

TRINITY_DN12647_c0_g1_i3	Glyma.09G002600.14	100	80	0	180	2048	1	1869	0	3009

TRINITY_DN12647_c0_g1_i3	Glyma.09G002600.1	96.96969697	80	0	180	2048	1	1869	0	3009

TRINITY_DN12647_c0_g1_i3	Glyma.09G002600.5	100	80	0	180	2048	1	1869	0	3009

TRINITY_DN12647_c0_g1_i3	Glyma.09G002600.3	100	80	0	180	2048	1	1869	0	3009

TRINITY_DN12647_c0_g1_i3	Glyma.09G002600.15	100	80	0	180	2048	1	1869	0	3009

TRINITY_DN12647_c0_g1_i3	Glyma.12G241700.2	100	69	1	180	2048	1	1866	0	3049

TRINITY_DN12647_c0_g1_i3	Glyma.12G241700.1	100	69	1	180	2048	1	1866	0	3049

TRINITY_DN12647_c0_g1_i3	Glyma.12G241700.4	100	69	1	180	2048	1	1866	0	3049

TRINITY_DN9294_c0_g1_i1	Glyma.17G204300.2	96.1038961	320	52	6	1833	1774	3604	0	1179

TRINITY_DN9294_c0_g1_i1	Glyma.17G204300.1	97.95918367	320	52	6	1833	1774	3604	0	1179

TRINITY_DN6018_c0_g2_i1	Glyma.16G160800.1	100	319	19	136	1993	55	1906	0	1515

TRINITY_DN14801_c0_g1_i2	Glyma.13G031300.1	95.95375723	310	20	420	2265	2291	438	0	1500

TRINITY_DN3954_c0_g2_i1	Glyma.03G251700.3	96.89265537	296	17	866	2719	4	1860	0	1648

TRINITY_DN3954_c0_g1_i1	Glyma.03G251700.3	98.57142857	295	18	866	2718	4	1860	0	1648

TRINITY_DN15369_c0_g2_i4	Glyma.12G064000.1	100	259	3	17	1882	1866	1	0	1989

TRINITY_DN9079_c0_g1_i1	Glyma.02G085700.1	100	237	15	247	2106	1	1853	0	1988

TRINITY_DN6205_c0_g2_i1	Glyma.19G201300.5	95.27559055	222	13	74	1929	1847	1	0	2006

TRINITY_DN6205_c0_g2_i1	Glyma.19G201300.3	95.18072289	222	13	74	1929	1847	1	0	2006

TRINITY_DN6205_c0_g2_i1	Glyma.19G201300.2	95.256917	222	13	74	1929	1847	1	0	2006

TRINITY_DN6205_c0_g2_i1	Glyma.19G201300.4	100	222	13	74	1929	1847	1	0	2006

TRINITY_DN6205_c0_g2_i1	Glyma.19G201300.1	95.23809524	222	13	74	1929	1847	1	0	2006

TRINITY_DN6205_c0_g2_i1	Glyma.19G201300.6	95.18072289	222	13	74	1929	1847	1	0	2006

TRINITY_DN16255_c1_g1_i3	Glyma.07G006500.1	98.26254826	221	4	360	2225	1947	82	0	2200

TRINITY_DN6205_c0_g1_i1	Glyma.19G201300.5	95.18388792	221	13	74	1929	1847	1	0	2012

TRINITY_DN6205_c0_g1_i1	Glyma.19G201300.3	95.18518519	221	13	74	1929	1847	1	0	2012

TRINITY_DN6205_c0_g1_i1	Glyma.19G201300.2	95.18599562	221	13	74	1929	1847	1	0	2012

TRINITY_DN6205_c0_g1_i1	Glyma.19G201300.4	95.18716578	221	13	74	1929	1847	1	0	2012

TRINITY_DN6205_c0_g1_i1	Glyma.19G201300.1	95.20295203	221	13	74	1929	1847	1	0	2012

TRINITY_DN6205_c0_g1_i1	Glyma.19G201300.6	95.20295203	221	13	74	1929	1847	1	0	2012

TRINITY_DN13054_c0_g1_i3	Glyma.15G067700.2	100	216	8	594	2442	1903	40	0	2104

TRINITY_DN13054_c0_g1_i3	Glyma.15G067700.1	99.35483871	216	8	594	2442	1903	40	0	2104

TRINITY_DN15781_c0_g1_i2	Glyma.05G031300.1	100	205	5	7	1865	284	2148	0	2237

TRINITY_DN16261_c0_g1_i1	Glyma.10G068100.1	98.96907216	193	14	240	2093	3366	1513	0	2200

TRINITY_DN16261_c0_g1_i1	Glyma.10G068100.2	100	193	14	240	2093	2880	1027	0	2200

TRINITY_DN4959_c0_g1_i1	Glyma.09G200800.1	96.58792651	151	3	1	1859	1048	2915	0	2555

TRINITY_DN11593_c0_g1_i2	Glyma.02G146500.1	100	143	12	291	2157	834	2649	0	2314

TRINITY_DN11593_c0_g1_i2	Glyma.02G146500.2	100	143	13	291	2157	834	2646	0	2296

TRINITY_DN16191_c1_g2_i1	Glyma.17G223200.1	98.38709677	437	39	172	2015	69	1912	0	730

TRINITY_DN16401_c1_g2_i10	Glyma.13G076200.1	97.82608696	343	40	38	1858	1939	86	0	1166

TRINITY_DN15139_c0_g3_i1	Glyma.11G087100.3	95.22058824	307	16	268	2123	2785	927	0	1624

TRINITY_DN15139_c0_g3_i1	Glyma.11G087100.1	95.22058824	307	16	268	2123	2785	927	0	1624

TRINITY_DN15139_c0_g3_i1	Glyma.11G087100.2	95.22058824	307	16	268	2123	2785	927	0	1624

TRINITY_DN16257_c1_g1_i12	Glyma.13G310300.1	100	264	12	183	2039	2235	382	0	1836

TRINITY_DN13612_c0_g1_i1	Glyma.01G001900.1	95	261	53	57	1846	565	2393	0	1258

TRINITY_DN13612_c0_g1_i1	Glyma.01G001900.2	95	261	53	57	1846	562	2390	0	1258

TRINITY_DN9244_c0_g1_i2	Glyma.17G078700.1	98.30508475	226	14	253	2085	1860	1	0	1930

TRINITY_DN14835_c0_g1_i1	Glyma.01G032400.1	99.35064935	219	19	2	1845	2519	4359	0	1917

TRINITY_DN14835_c0_g1_i1	Glyma.01G032400.2	98.5915493	219	20	2	1845	2519	4356	0	1899

TRINITY_DN16372_c0_g1_i3	Glyma.03G101200.1	100	218	11	166	2029	1852	1	0	2124

TRINITY_DN16218_c0_g5_i8	Glyma.10G161400.1	96.1038961	211	7	403	2262	1863	1	0	2207

TRINITY_DN16218_c0_g5_i8	Glyma.10G161400.2	96.1038961	211	7	403	2262	1863	1	0	2207

TRINITY_DN16218_c0_g5_i1	Glyma.10G161400.1	95.4954955	198	7	344	2203	1863	1	0	2279

TRINITY_DN16218_c0_g5_i1	Glyma.10G161400.2	95.65217391	198	7	344	2203	1863	1	0	2279

TRINITY_DN16218_c0_g5_i9	Glyma.10G161400.1	97.33333333	198	7	344	2203	1863	1	0	2279

TRINITY_DN16218_c0_g5_i9	Glyma.10G161400.2	97.07207207	198	7	344	2203	1863	1	0	2279

TRINITY_DN13524_c0_g2_i2	Glyma.15G159000.4	97.0845481	193	3	768	2633	1	1860	0	2327

TRINITY_DN13524_c0_g2_i2	Glyma.09G052700.3	97.0845481	193	3	768	2633	1	1860	0	2327

TRINITY_DN11244_c0_g1_i1	Glyma.16G035400.1	95.82309582	167	20	222	2056	1	1865	0	2303

TRINITY_DN8787_c0_g1_i1	Glyma.10G120400.1	97.34693878	162	18	51	1879	1	1859	0	2254

TRINITY_DN14809_c0_g1_i5	Glyma.07G227700.1	95.6937799	159	4	404	2261	1864	1	0	2490

TRINITY_DN15940_c0_g1_i1	Glyma.08G240700.4	97.08029197	151	7	544	2397	160	2022	0	2501

TRINITY_DN15940_c0_g1_i1	Glyma.08G240700.3	100	150	10	544	2397	160	2013	0	2449

TRINITY_DN14809_c0_g1_i4	Glyma.07G227700.1	97.88359788	148	4	404	2261	1864	1	0	2551

TRINITY_DN6539_c0_g1_i3	Glyma.08G038200.8	99.60784314	146	2	417	2282	1866	1	0	2627

TRINITY_DN6539_c0_g1_i3	Glyma.08G038200.5	99.59349593	146	2	417	2282	1866	1	0	2627

TRINITY_DN6539_c0_g1_i3	Glyma.08G038200.1	99.61832061	146	2	417	2282	1866	1	0	2627

TRINITY_DN6539_c0_g1_i3	Glyma.08G038200.9	99.62121212	146	2	417	2282	1866	1	0	2627

TRINITY_DN6539_c0_g1_i3	Glyma.08G038200.6	99.59349593	146	2	417	2282	1866	1	0	2627

TRINITY_DN6539_c0_g1_i3	Glyma.08G038200.7	99.62264151	145	4	417	2282	1854	1	0	2555

TRINITY_DN6539_c0_g1_i3	Glyma.08G038200.2	99.59349593	145	4	417	2282	1854	1	0	2555

TRINITY_DN6539_c0_g1_i3	Glyma.08G038200.3	99.58506224	145	4	417	2282	1854	1	0	2555

TRINITY_DN6539_c0_g1_i3	Glyma.08G038200.4	99.59349593	145	4	417	2282	1854	1	0	2555

TRINITY_DN4541_c0_g2_i1	Glyma.11G091700.1	97.0624235	144	13	6	1856	1	1863	0	2521

TRINITY_DN6539_c0_g1_i3	Glyma.05G230700.2	99.5951417	141	2	417	2282	1866	1	0	2654

TRINITY_DN6539_c0_g1_i3	Glyma.05G230700.3	99.59432049	140	3	417	2282	1851	1	0	2562

TRINITY_DN6539_c0_g1_i3	Glyma.05G230700.4	99.58506224	140	4	417	2282	1854	1	0	2582

TRINITY_DN16218_c0_g5_i8	Glyma.19G243600.1	96.11650485	342	15	409	2262	1860	1	0	1435

TRINITY_DN16023_c0_g1_i3	Glyma.14G193300.1	96.12903226	319	20	370	2224	4323	2469	0	1537

TRINITY_DN16023_c0_g1_i3	Glyma.14G193300.2	98.18181818	319	20	370	2224	4257	2403	0	1537

TRINITY_DN15327_c1_g1_i3	Glyma.02G272100.1	97.93103448	279	2	236	2100	1951	87	0	1888

TRINITY_DN15327_c1_g1_i3	Glyma.02G272100.4	100	279	2	236	2100	1882	18	0	1888

TRINITY_DN15710_c0_g2_i1	Glyma.U033500.4	95.54140127	269	36	36	1877	1863	30	0	1594

TRINITY_DN15710_c0_g2_i1	Glyma.U033500.2	95.54140127	269	36	36	1877	1863	30	0	1594

TRINITY_DN15710_c0_g2_i2	Glyma.U033500.4	99.2481203	269	36	36	1877	1863	30	0	1594

TRINITY_DN15710_c0_g2_i2	Glyma.U033500.2	98.98989899	269	36	36	1877	1863	30	0	1594

TRINITY_DN15327_c1_g1_i5	Glyma.02G272100.1	100	253	2	236	2100	1951	87	0	2032

TRINITY_DN15327_c1_g1_i5	Glyma.02G272100.4	100	253	2	236	2100	1882	18	0	2032

TRINITY_DN13713_c0_g1_i6	Glyma.15G042700.6	97.24409449	215	11	420	2264	86	1950	0	2113

TRINITY_DN9320_c0_g1_i1	Glyma.16G076900.1	97.61904762	201	8	179	2034	108	1971	0	2255

TRINITY_DN15007_c0_g1_i3	Glyma.08G133200.1	100	199	0	539	2404	1866	1	0	2344

TRINITY_DN15007_c0_g1_i9	Glyma.08G133200.1	98.27586207	199	0	539	2404	1866	1	0	2344

TRINITY_DN14967_c1_g1_i1	Glyma.10G250100.2	100	197	6	383	2247	250	2109	0	2309

TRINITY_DN14967_c1_g1_i1	Glyma.10G250100.1	100	197	6	383	2247	250	2109	0	2309

TRINITY_DN15139_c0_g3_i2	Glyma.11G087100.3	95.22058824	197	14	268	2123	2785	927	0	2244

TRINITY_DN15139_c0_g3_i2	Glyma.11G087100.1	95.22091975	197	14	268	2123	2785	927	0	2244

TRINITY_DN15139_c0_g3_i2	Glyma.11G087100.2	95.221843	197	14	268	2123	2785	927	0	2244

TRINITY_DN15007_c0_g1_i3	Glyma.08G133200.2	100	196	3	539	2404	1857	1	0	2302

TRINITY_DN15007_c0_g1_i9	Glyma.08G133200.2	100	196	3	539	2404	1857	1	0	2302

TRINITY_DN11977_c0_g1_i1	Glyma.04G041400.1	100	182	3	281	2145	76	1939	0	2418

TRINITY_DN18011_c0_g1_i1	Glyma.04G250200.4	96.21621622	178	26	1	1839	1681	3513	0	2073

TRINITY_DN14554_c0_g1_i1	Glyma.06G271200.1	97.06601467	176	12	207	2048	2076	211	0	2316

TRINITY_DN13734_c0_g1_i3	Glyma.05G102400.1	100	172	11	1	1863	691	2541	0	2377

TRINITY_DN15706_c0_g1_i5	Glyma.08G180900.2	100	164	0	640	2505	1	1866	0	2538

TRINITY_DN7983_c0_g1_i1	Glyma.13G184200.4	95.77836412	156	8	414	2276	1	1857	0	2505

TRINITY_DN16365_c0_g1_i8	Glyma.08G113000.1	97.5	156	12	303	2147	1	1860	0	2407

TRINITY_DN16365_c0_g1_i9	Glyma.08G113000.1	98.63945578	154	11	253	2100	1	1860	0	2438

TRINITY_DN15940_c0_g1_i1	Glyma.08G240700.1	98.56115108	151	7	544	2396	160	2021	0	2499

TRINITY_DN12139_c0_g1_i2	Glyma.18G075000.1	98.5	151	13	90	1925	1	1863	0	2396

TRINITY_DN15940_c0_g1_i1	Glyma.08G240700.2	100	150	10	544	2396	160	2012	0	2447

TRINITY_DN13524_c0_g2_i1	Glyma.09G052700.3	100	146	1	768	2633	1	1860	0	2599

TRINITY_DN9201_c0_g1_i1	Glyma.17G019900.1	95.79288026	144	19	14	1856	24	1880	0	2442

TRINITY_DN13524_c0_g2_i1	Glyma.15G159000.4	100	143	1	768	2633	1	1860	0	2615

TRINITY_DN11593_c0_g1_i1	Glyma.02G146500.1	100	143	12	291	2155	834	2647	0	2311

TRINITY_DN11593_c0_g1_i1	Glyma.02G146500.2	100	143	13	291	2155	834	2644	0	2292

TRINITY_DN15007_c0_g1_i4	Glyma.08G133200.1	96.69811321	139	0	539	2404	1866	1	0	2676

TRINITY_DN15007_c0_g1_i5	Glyma.08G133200.1	97.4025974	139	0	539	2404	1866	1	0	2676

TRINITY_DN15007_c0_g1_i4	Glyma.08G133200.2	99.29824561	138	2	539	2404	1857	1	0	2623

TRINITY_DN15007_c0_g1_i5	Glyma.08G133200.2	99.25373134	138	2	539	2404	1857	1	0	2623

TRINITY_DN15706_c0_g1_i3	Glyma.08G180900.2	100	133	0	640	2505	1	1866	0	2710

TRINITY_DN11607_c0_g2_i1	Glyma.04G207800.1	97.72727273	122	2	83	1948	1863	1	0	2750

TRINITY_DN16365_c0_g1_i3	Glyma.08G113000.1	97.60479042	119	9	253	2103	1	1860	0	2651

TRINITY_DN16365_c0_g1_i5	Glyma.08G113000.1	97.50297265	118	9	303	2153	1	1860	0	2656

TRINITY_DN16218_c0_g5_i11	Glyma.10G161400.1	100	117	4	403	2262	1863	1	0	2739

TRINITY_DN16218_c0_g5_i11	Glyma.10G161400.2	100	117	4	403	2262	1863	1	0	2739

TRINITY_DN16218_c0_g5_i13	Glyma.10G161400.1	96.13636364	117	4	403	2262	1863	1	0	2739

TRINITY_DN16218_c0_g5_i13	Glyma.10G161400.2	95.07186858	117	4	403	2262	1863	1	0	2739

TRINITY_DN12251_c0_g1_i1	Glyma.09G018300.2	100	88	0	170	2035	1	1866	0	2959

TRINITY_DN16218_c0_g5_i1	Glyma.19G243600.1	95.28795812	328	13	350	2203	1860	1	0	1524

TRINITY_DN16218_c0_g5_i9	Glyma.19G243600.1	95.5	328	13	350	2203	1860	1	0	1524

TRINITY_DN16372_c0_g1_i3	Glyma.07G114200.1	100	307	8	168	2029	1858	1	0	1679

TRINITY_DN16218_c0_g5_i11	Glyma.19G243600.1	96.42857143	269	13	409	2262	1860	1	0	1851

TRINITY_DN16218_c0_g5_i13	Glyma.19G243600.1	96.26556017	269	13	409	2262	1860	1	0	1851

TRINITY_DN16012_c0_g1_i7	Glyma.20G066800.1	100	228	15	166	2022	469	2325	0	2078

TRINITY_DN16012_c0_g1_i7	Glyma.13G002200.1	100	227	14	166	2022	469	2325	0	2084

TRINITY_DN9244_c0_g1_i2	Glyma.05G020600.4	100	214	14	253	2085	1863	1	0	2039

TRINITY_DN9244_c0_g1_i2	Glyma.05G020600.3	100	214	14	253	2085	1863	1	0	2039

TRINITY_DN9244_c0_g1_i2	Glyma.05G020600.1	95.71428571	214	14	253	2085	1863	1	0	2039

TRINITY_DN9244_c0_g1_i2	Glyma.05G020600.2	98.63945578	214	14	253	2085	1863	1	0	2039

TRINITY_DN13734_c0_g1_i3	Glyma.17G164600.1	98.9010989	167	9	1	1863	667	2517	0	2416

TRINITY_DN5388_c0_g2_i1	Glyma.02G207400.1	95.52238806	153	1	97	1958	53	1917	0	2577

TRINITY_DN16415_c1_g1_i13	Glyma.11G147900.1	99.2248062	144	4	2357	4220	1866	5	0	2621

TRINITY_DN16415_c1_g1_i6	Glyma.11G147900.1	98.92473118	144	4	2263	4126	1866	5	0	2621

TRINITY_DN4959_c0_g1_i1	Glyma.01G021200.1	96.59685864	139	2	1	1859	1048	2912	0	2636

TRINITY_DN16012_c0_g1_i7	Glyma.17G010500.1	96.50793651	133	9	166	2022	469	2328	0	2623

TRINITY_DN16012_c0_g1_i7	Glyma.17G010500.2	96.50872818	133	9	166	2022	469	2328	0	2623

TRINITY_DN13054_c0_g1_i1	Glyma.07G190700.1	97.14285714	128	6	589	2444	1872	17	0	2619

TRINITY_DN14312_c0_g1_i4	Glyma.01G229200.3	97.40802676	108	1	563	2427	2502	641	0	2826

TRINITY_DN3215_c0_g1_i2	Glyma.02G017400.1	98.29059829	94	0	356	2220	1865	1	0	2924

TRINITY_DN3215_c0_g1_i2	Glyma.10G017800.1	98.28473413	91	0	356	2220	1865	1	0	2940

TRINITY_DN16255_c1_g1_i2	Glyma.07G006500.1	98.30508475	270	8	213	2072	1941	82	0	1895

TRINITY_DN12151_c0_g1_i1	Glyma.16G083700.1	96.59090909	267	1	1	1861	557	2420	0	1943

TRINITY_DN15619_c0_g2_i4	Glyma.06G181900.1	97.82608696	240	7	912	2769	1	1861	0	2054

TRINITY_DN5733_c0_g2_i2	Glyma.01G180200.1	96.27118644	238	26	292	2131	1893	51	0	1832

TRINITY_DN16218_c0_g5_i8	Glyma.20G224100.1	96.10182975	216	5	403	2262	1860	1	0	2194

TRINITY_DN16218_c0_g5_i8	Glyma.20G224100.3	96.1038961	216	5	403	2262	1860	1	0	2194

TRINITY_DN16218_c0_g5_i8	Glyma.20G224100.4	99.43181818	216	5	403	2262	1860	1	0	2194

TRINITY_DN16218_c0_g5_i8	Glyma.20G224100.5	100	216	5	403	2262	1860	1	0	2194

TRINITY_DN38924_c0_g1_i1	Glyma.10G084300.3	98.24561404	213	8	234	2089	1	1859	0	2178

TRINITY_DN16218_c0_g5_i1	Glyma.20G224100.1	100	204	5	344	2203	1860	1	0	2261

TRINITY_DN16218_c0_g5_i1	Glyma.20G224100.3	97.48427673	204	5	344	2203	1860	1	0	2261

TRINITY_DN16218_c0_g5_i1	Glyma.20G224100.4	95.45454545	204	5	344	2203	1860	1	0	2261

TRINITY_DN16218_c0_g5_i1	Glyma.20G224100.5	95.83333333	204	5	344	2203	1860	1	0	2261

TRINITY_DN16218_c0_g5_i9	Glyma.20G224100.1	95.87426326	204	5	344	2203	1860	1	0	2261

TRINITY_DN16218_c0_g5_i9	Glyma.20G224100.3	95.87482219	204	5	344	2203	1860	1	0	2261

TRINITY_DN16218_c0_g5_i9	Glyma.20G224100.4	100	204	5	344	2203	1860	1	0	2261

TRINITY_DN16218_c0_g5_i9	Glyma.20G224100.5	95.87628866	204	5	344	2203	1860	1	0	2261

TRINITY_DN11169_c0_g1_i1	Glyma.20G139900.2	100	198	13	318	2166	66	1923	0	2209

TRINITY_DN16251_c0_g3_i8	Glyma.15G167100.1	100	191	7	548	2405	2332	472	0	2326

TRINITY_DN16251_c0_g3_i8	Glyma.15G167100.3	97.38372093	191	7	548	2405	2290	430	0	2326

TRINITY_DN16251_c0_g3_i8	Glyma.15G167100.4	96.55172414	190	8	548	2405	2311	472	0	2196

TRINITY_DN16251_c0_g3_i8	Glyma.15G167100.5	100	189	9	548	2405	2329	472	0	2318

TRINITY_DN16251_c0_g3_i8	Glyma.15G167100.2	100	189	9	548	2405	2287	430	0	2318

TRINITY_DN8787_c0_g1_i1	Glyma.20G070800.1	96.33699634	176	10	51	1886	1	1857	0	2241

TRINITY_DN36201_c0_g1_i1	Glyma.16G099100.3	99.39393939	170	4	968	2829	2	1860	0	2455

TRINITY_DN15950_c0_g3_i1	Glyma.13G360400.1	97.18969555	169	15	3	1856	172	2019	0	2338

TRINITY_DN15950_c0_g3_i1	Glyma.13G360400.2	95.16129032	169	15	3	1856	172	2019	0	2338

TRINITY_DN12017_c0_g1_i1	Glyma.11G228500.1	98.10126582	168	14	203	2049	1860	17	0	2272

TRINITY_DN12944_c0_g2_i1	Glyma.15G044900.2	95.83333333	168	31	181	2008	1848	21	0	2047

TRINITY_DN12944_c0_g2_i1	Glyma.15G044900.1	95.83333333	168	31	181	2008	1848	21	0	2047

TRINITY_DN8787_c0_g1_i1	Glyma.10G120400.2	98.10606061	163	17	51	1879	1	1856	0	2261

TRINITY_DN39905_c0_g1_i2	Glyma.15G128500.1	98.06362379	113	2	1	1855	201	2064	0	2758

TRINITY_DN39905_c0_g1_i5	Glyma.15G128500.1	98.05825243	113	2	1	1855	201	2064	0	2758

TRINITY_DN39905_c0_g1_i6	Glyma.15G128500.1	98.04469274	113	2	1	1855	201	2064	0	2758

TRINITY_DN6860_c0_g1_i2	Glyma.14G049700.1	99.23076923	92	4	725	2582	1858	1	0	2856

TRINITY_DN9165_c0_g1_i1	Glyma.18G033500.1	95.28985507	355	37	120	1968	94	1914	0	1112

TRINITY_DN9165_c0_g1_i1	Glyma.18G033500.2	98.63013699	355	37	120	1968	91	1911	0	1112

TRINITY_DN9294_c0_g1_i1	Glyma.17G204700.2	100	322	50	15	1833	1774	3595	0	1107

TRINITY_DN9294_c0_g1_i1	Glyma.17G204700.1	100	322	50	15	1833	1774	3595	0	1107

TRINITY_DN14364_c0_g2_i1	Glyma.17G031900.1	99.34210526	315	18	302	2145	2754	902	0	1507

TRINITY_DN14364_c0_g2_i6	Glyma.17G031900.1	99.45652174	315	18	302	2145	2754	902	0	1507

TRINITY_DN16372_c0_g1_i1	Glyma.03G101200.1	100	305	8	166	2026	1852	1	0	1666

TRINITY_DN14364_c0_g2_i1	Glyma.07G242000.1	95.19650655	304	16	302	2145	2757	902	0	1589

TRINITY_DN14364_c0_g2_i6	Glyma.07G242000.1	100	304	16	302	2145	2757	902	0	1589

TRINITY_DN14364_c0_g2_i1	Glyma.07G242000.2	95.19650655	304	16	302	2145	2706	851	0	1589

TRINITY_DN14364_c0_g2_i6	Glyma.07G242000.2	97.45762712	304	16	302	2145	2706	851	0	1589

TRINITY_DN15937_c0_g1_i1	Glyma.12G155700.1	99.11504425	292	31	544	2398	3	1812	0	1428

TRINITY_DN15937_c0_g1_i2	Glyma.12G155700.1	97.92060491	292	31	544	2398	3	1812	0	1428

TRINITY_DN15937_c0_g1_i3	Glyma.12G155700.1	95.27027027	292	31	544	2398	3	1812	0	1428

TRINITY_DN15937_c0_g1_i5	Glyma.12G155700.1	100	292	31	544	2398	3	1812	0	1428

TRINITY_DN16255_c1_g1_i2	Glyma.08G207100.1	95.21276596	283	4	213	2073	1986	126	0	1847

TRINITY_DN16255_c1_g1_i2	Glyma.08G207100.4	95.21276596	276	10	213	2073	1962	126	0	1731

TRINITY_DN16372_c0_g1_i1	Glyma.07G114200.1	100	270	8	168	2026	1858	1	0	1886

TRINITY_DN15710_c0_g2_i1	Glyma.12G074600.8	100	263	34	36	1877	1866	30	0	1679

TRINITY_DN15710_c0_g2_i1	Glyma.12G074600.9	95.45454545	263	34	36	1877	1866	30	0	1679

TRINITY_DN15710_c0_g2_i2	Glyma.12G074600.8	100	263	34	36	1877	1866	30	0	1679

TRINITY_DN15710_c0_g2_i2	Glyma.12G074600.9	100	263	34	36	1877	1866	30	0	1679

TRINITY_DN16211_c1_g2_i3	Glyma.07G270600.1	100	197	9	253	2110	1	1858	0	2285

TRINITY_DN13985_c0_g1_i1	Glyma.12G012000.3	95.77464789	194	15	268	2115	1	1854	0	2211

TRINITY_DN13985_c0_g1_i1	Glyma.12G012000.1	99.29078014	194	15	268	2115	1	1854	0	2211

TRINITY_DN2619_c0_g1_i1	Glyma.13G348300.3	98.98989899	162	4	437	2298	153	2012	0	2518

TRINITY_DN16365_c0_g1_i8	Glyma.05G155100.1	97.5	162	17	303	2147	1	1845	0	2311

TRINITY_DN16365_c0_g1_i8	Glyma.05G155100.2	97.5	162	17	303	2147	1	1845	0	2311

TRINITY_DN16365_c0_g1_i9	Glyma.05G155100.1	98.63013699	161	14	253	2100	1	1845	0	2335

TRINITY_DN16365_c0_g1_i9	Glyma.05G155100.2	98.63481229	161	14	253	2100	1	1845	0	2335

TRINITY_DN9638_c0_g1_i2	Glyma.10G233400.2	100	150	9	525	2376	1861	1	0	2525

TRINITY_DN9638_c0_g1_i2	Glyma.10G233400.1	100	150	9	525	2376	1861	1	0	2525

TRINITY_DN14265_c0_g1_i1	Glyma.06G322200.1	95	133	2	417	2273	1863	1	0	2665

TRINITY_DN14265_c0_g1_i1	Glyma.06G322200.2	100	133	2	417	2273	1863	1	0	2665

TRINITY_DN16365_c0_g1_i3	Glyma.05G155100.1	97.59036145	128	12	253	2103	1	1845	0	2538

TRINITY_DN16365_c0_g1_i3	Glyma.05G155100.2	97.59615385	128	12	253	2103	1	1845	0	2538

TRINITY_DN16365_c0_g1_i5	Glyma.05G155100.1	97.51552795	127	12	303	2153	1	1845	0	2543

TRINITY_DN16365_c0_g1_i5	Glyma.05G155100.2	97.52066116	127	12	303	2153	1	1845	0	2543

TRINITY_DN14423_c0_g1_i2	Glyma.10G272800.2	96.2962963	124	0	181	2043	1	1863	0	2754

TRINITY_DN14423_c0_g1_i2	Glyma.10G272800.1	96.1038961	124	0	181	2043	1	1863	0	2754

TRINITY_DN14265_c0_g1_i1	Glyma.04G112300.1	95	123	2	417	2273	1863	1	0	2721

TRINITY_DN14265_c0_g1_i1	Glyma.04G112300.2	100	123	2	417	2273	1863	1	0	2721

TRINITY_DN11150_c0_g1_i1	Glyma.19G208100.2	95.97315436	117	2	275	2136	24	1885	0	2780

TRINITY_DN11150_c0_g1_i2	Glyma.19G208100.2	97.05882353	117	2	275	2136	24	1885	0	2780

TRINITY_DN15097_c0_g1_i4	Glyma.02G038800.1	100	115	5	14	1874	247	2106	0	2771

TRINITY_DN16218_c0_g5_i11	Glyma.20G224100.1	98	114	3	403	2262	1860	1	0	2771

TRINITY_DN16218_c0_g5_i11	Glyma.20G224100.3	99.18699187	114	3	403	2262	1860	1	0	2771

TRINITY_DN16218_c0_g5_i11	Glyma.20G224100.4	97.6	114	3	403	2262	1860	1	0	2771

TRINITY_DN16218_c0_g5_i11	Glyma.20G224100.5	98.65771812	114	3	403	2262	1860	1	0	2771

TRINITY_DN16218_c0_g5_i13	Glyma.20G224100.1	100	114	3	403	2262	1860	1	0	2771

TRINITY_DN16218_c0_g5_i13	Glyma.20G224100.3	100	114	3	403	2262	1860	1	0	2771

TRINITY_DN16218_c0_g5_i13	Glyma.20G224100.4	100	114	3	403	2262	1860	1	0	2771

TRINITY_DN16218_c0_g5_i13	Glyma.20G224100.5	100	114	3	403	2262	1860	1	0	2771

TRINITY_DN16401_c1_g2_i8	Glyma.20G048800.14	97.21115538	345	51	51	1865	1908	68	0	1088

TRINITY_DN16401_c1_g2_i8	Glyma.20G048800.6	98.57142857	345	51	51	1865	1908	68	0	1088

TRINITY_DN16401_c1_g2_i8	Glyma.20G048800.7	100	345	51	51	1865	1908	68	0	1088

TRINITY_DN16401_c1_g2_i8	Glyma.20G048800.15	100	345	51	51	1865	1908	68	0	1088

TRINITY_DN16401_c1_g2_i8	Glyma.20G048800.16	100	345	51	51	1865	1908	68	0	1088

TRINITY_DN16401_c1_g2_i8	Glyma.20G048800.17	98.9010989	345	51	51	1865	1908	68	0	1088

TRINITY_DN16401_c1_g2_i8	Glyma.20G048800.12	97.95918367	345	51	51	1865	1908	68	0	1088

TRINITY_DN16401_c1_g2_i8	Glyma.20G048800.8	96.46464646	345	51	51	1865	1908	68	0	1088

TRINITY_DN16401_c1_g2_i8	Glyma.20G048800.9	100	345	51	51	1865	1908	68	0	1088

TRINITY_DN16401_c1_g2_i8	Glyma.20G048800.2	100	345	51	51	1865	1908	68	0	1088

TRINITY_DN16401_c1_g2_i8	Glyma.20G048800.13	100	345	51	51	1865	1908	68	0	1088

TRINITY_DN16401_c1_g2_i8	Glyma.20G048800.10	100	345	51	51	1865	1908	68	0	1088

TRINITY_DN16401_c1_g2_i8	Glyma.20G048800.11	95.23809524	345	51	51	1865	1908	68	0	1088

TRINITY_DN16401_c1_g2_i8	Glyma.20G048800.3	100	345	51	51	1865	1908	68	0	1088

TRINITY_DN16401_c1_g2_i8	Glyma.20G048800.1	99.6763754	345	51	51	1865	1908	68	0	1088

TRINITY_DN15137_c0_g1_i1	Glyma.14G222700.2	98.58490566	298	9	1361	3213	1976	118	0	1711

TRINITY_DN15137_c0_g1_i1	Glyma.14G222700.1	100	298	9	1361	3213	1976	118	0	1711

TRINITY_DN15137_c0_g1_i13	Glyma.14G222700.2	100	298	9	1275	3127	1976	118	0	1711

TRINITY_DN15137_c0_g1_i13	Glyma.14G222700.1	100	298	9	1275	3127	1976	118	0	1711

TRINITY_DN15137_c0_g1_i6	Glyma.14G222700.2	97.43589744	298	9	1388	3240	1976	118	0	1711

TRINITY_DN15137_c0_g1_i6	Glyma.14G222700.1	99.11504425	298	9	1388	3240	1976	118	0	1711

TRINITY_DN15937_c0_g1_i1	Glyma.12G155700.3	100	291	31	544	2397	3	1811	0	1432

TRINITY_DN15937_c0_g1_i2	Glyma.12G155700.3	98.64253394	291	31	544	2397	3	1811	0	1432

TRINITY_DN15937_c0_g1_i3	Glyma.12G155700.3	100	291	31	544	2397	3	1811	0	1432

TRINITY_DN15937_c0_g1_i5	Glyma.12G155700.3	95.71428571	291	31	544	2397	3	1811	0	1432

TRINITY_DN16211_c1_g2_i3	Glyma.09G018500.1	100	286	21	268	2112	10	1857	0	1653

TRINITY_DN5733_c0_g1_i1	Glyma.01G180200.1	98.6013986	267	26	337	2174	1891	51	0	1668

TRINITY_DN11244_c0_g1_i2	Glyma.19G117700.1	97.87234043	244	15	222	2056	1	1856	0	1873

TRINITY_DN11244_c0_g1_i2	Glyma.19G117700.3	100	244	16	222	2056	1	1850	0	1834

TRINITY_DN11244_c0_g1_i2	Glyma.19G117700.2	100	244	16	222	2056	1	1850	0	1834

TRINITY_DN8977_c0_g1_i1	Glyma.04G092200.1	98.36065574	145	15	1	1846	492	2337	0	2429

TRINITY_DN10580_c0_g1_i1	Glyma.15G173100.1	100	142	10	359	2209	3354	1503	0	2516

TRINITY_DN8977_c0_g1_i1	Glyma.06G094000.1	98.36065574	138	19	1	1847	489	2317	0	2364

TRINITY_DN16365_c0_g1_i1	Glyma.08G113000.1	97.6076555	122	9	253	2099	1	1856	0	2627

TRINITY_DN16365_c0_g1_i6	Glyma.08G113000.1	97.5088968	121	9	303	2149	1	1856	0	2632

TRINITY_DN15139_c0_g3_i1	Glyma.09G197100.1	100	120	2	227	2088	2844	989	0	2736

TRINITY_DN15139_c0_g3_i1	Glyma.09G197100.2	100	120	2	227	2088	2538	683	0	2736

TRINITY_DN15619_c0_g2_i4	Glyma.05G038300.2	100	115	0	912	2773	1	1862	0	2802

TRINITY_DN15097_c0_g1_i4	Glyma.01G026000.1	98.98477157	115	3	14	1874	247	2106	0	2782

TRINITY_DN527_c0_g1_i1	Glyma.02G312200.1	97.20670391	94	4	1	1858	3309	5166	0	2867

TRINITY_DN527_c0_g1_i1	Glyma.02G312200.2	97.20670391	94	4	1	1858	1983	3840	0	2867

TRINITY_DN13713_c0_g1_i2	Glyma.U033400.8	100	74	2	393	2254	125	1983	0	3009

TRINITY_DN13713_c0_g1_i2	Glyma.U033400.1	99.2481203	74	2	393	2254	125	1983	0	3009

TRINITY_DN13713_c0_g1_i4	Glyma.U033400.8	98.11320755	74	2	381	2242	125	1983	0	3009

TRINITY_DN13713_c0_g1_i4	Glyma.U033400.1	95.71428571	74	2	381	2242	125	1983	0	3009

TRINITY_DN13713_c0_g1_i1	Glyma.U033400.3	100	74	2	420	2281	86	1944	0	3009

TRINITY_DN13713_c0_g1_i1	Glyma.U033400.7	98.57142857	74	2	420	2281	86	1944	0	3009

TRINITY_DN13713_c0_g1_i3	Glyma.U033400.3	99.27007299	74	2	137	1998	86	1944	0	3009

TRINITY_DN13713_c0_g1_i3	Glyma.U033400.7	95.1417004	74	2	137	1998	86	1944	0	3009

TRINITY_DN13713_c0_g1_i6	Glyma.U033400.3	100	72	4	420	2264	86	1944	0	2911

TRINITY_DN13713_c0_g1_i6	Glyma.U033400.7	100	72	4	420	2264	86	1944	0	2911

TRINITY_DN13713_c0_g1_i2	Glyma.12G074700.8	99.15254237	64	3	393	2254	125	1977	0	3025

TRINITY_DN13713_c0_g1_i2	Glyma.12G074700.1	100	64	3	393	2254	125	1977	0	3025

TRINITY_DN13713_c0_g1_i4	Glyma.12G074700.8	100	64	3	381	2242	125	1977	0	3025

TRINITY_DN13713_c0_g1_i4	Glyma.12G074700.1	98.85057471	64	3	381	2242	125	1977	0	3025

TRINITY_DN9320_c0_g2_i1	Glyma.16G076900.1	97.08029197	300	11	231	2075	115	1971	0	1646

TRINITY_DN14936_c0_g2_i1	Glyma.04G060800.2	96.2962963	296	12	374	2218	1	1857	0	1668

TRINITY_DN3648_c0_g1_i1	Glyma.06G187200.1	100	296	28	60	1882	11	1863	0	1500

TRINITY_DN14936_c0_g2_i1	Glyma.06G061400.1	96.2962963	285	28	374	2218	1	1836	0	1592

TRINITY_DN14936_c0_g2_i2	Glyma.04G060800.2	96.2962963	258	12	75	1919	1	1857	0	1879

TRINITY_DN15619_c0_g2_i4	Glyma.06G181900.2	100	240	5	912	2769	1	1858	0	2069

TRINITY_DN16257_c1_g1_i12	Glyma.12G192000.1	100	239	19	192	2039	2220	376	0	1925

TRINITY_DN16211_c1_g2_i3	Glyma.17G003400.1	100	200	6	253	2110	1	1858	0	2290

TRINITY_DN13054_c0_g1_i3	Glyma.08G058600.1	100	192	8	589	2439	1875	19	0	2283

TRINITY_DN13054_c0_g1_i3	Glyma.07G190700.1	95.18072289	174	8	589	2439	1872	19	0	2362

TRINITY_DN9428_c0_g2_i1	Glyma.16G067700.1	95.40636042	166	4	94	1951	1	1860	0	2492

TRINITY_DN8897_c0_g2_i1	Glyma.09G276300.1	96.35499208	155	21	356	2195	1846	1	0	2346

TRINITY_DN13193_c0_g1_i1	Glyma.07G049900.1	95.77464789	151	7	97	1947	1	1860	0	2529

TRINITY_DN13193_c0_g1_i1	Glyma.07G049900.2	95.77464789	151	7	97	1947	1	1860	0	2529

TRINITY_DN13193_c0_g1_i1	Glyma.16G018500.2	96.80851064	147	7	97	1947	1	1860	0	2551

TRINITY_DN13193_c0_g1_i1	Glyma.16G018500.1	96.81528662	147	7	97	1947	1	1860	0	2551

TRINITY_DN9201_c0_g1_i2	Glyma.17G019900.1	98.72881356	136	6	14	1867	24	1880	0	2612

TRINITY_DN14827_c0_g1_i2	Glyma.03G188600.1	100	133	2	106	1966	159	2013	0	2662

TRINITY_DN14827_c0_g1_i6	Glyma.03G188600.1	97.36842105	133	2	106	1966	159	2013	0	2662

TRINITY_DN14827_c0_g1_i7	Glyma.03G188600.1	95.23809524	133	2	106	1966	159	2013	0	2662

TRINITY_DN13484_c0_g3_i2	Glyma.06G094300.1	95.23809524	130	15	161	2005	1	1848	0	2529

TRINITY_DN15521_c0_g1_i3	Glyma.06G226700.1	95.45454545	128	2	1355	3214	1179	3038	0	2715

TRINITY_DN15521_c0_g1_i3	Glyma.06G226700.2	96.62447257	128	2	1355	3214	1035	2894	0	2715

TRINITY_DN527_c0_g1_i1	Glyma.14G000300.1	96	101	2	1	1858	3288	5145	0	2839

TRINITY_DN527_c0_g1_i1	Glyma.14G000300.2	96	101	2	1	1858	2190	4047	0	2839

TRINITY_DN14364_c0_g2_i4	Glyma.17G031900.1	98.75776398	258	11	302	2145	2754	902	0	1857

TRINITY_DN14364_c0_g2_i5	Glyma.17G031900.1	100	258	11	302	2145	2754	902	0	1857

TRINITY_DN15327_c1_g1_i3	Glyma.14G045000.1	100	253	4	249	2106	1869	12	0	2008

TRINITY_DN14936_c0_g2_i2	Glyma.06G061400.1	96.2962963	246	26	75	1919	1	1836	0	1820

TRINITY_DN11113_c0_g1_i1	Glyma.16G095000.1	98.57142857	242	24	264	2112	15	1848	0	1855

TRINITY_DN11113_c0_g1_i1	Glyma.16G095000.2	98.57142857	242	24	264	2112	15	1848	0	1855

TRINITY_DN11113_c0_g1_i2	Glyma.16G095000.1	95.6043956	242	24	234	2082	15	1848	0	1855

TRINITY_DN11113_c0_g1_i2	Glyma.16G095000.2	95.6043956	242	24	234	2082	15	1848	0	1855

TRINITY_DN15619_c0_g2_i4	Glyma.04G184000.1	95.8677686	228	3	912	2769	1	1858	0	2146

TRINITY_DN15619_c0_g2_i4	Glyma.04G184000.3	100	228	3	912	2769	1	1858	0	2146

TRINITY_DN16509_c0_g1_i1	Glyma.01G041400.1	100	224	7	211	2064	1854	1	0	2117

TRINITY_DN3062_c0_g1_i1	Glyma.04G146900.2	98.70967742	206	19	152	2002	1836	1	0	2080

TRINITY_DN16012_c0_g1_i10	Glyma.17G010500.1	98.27586207	202	4	166	2019	469	2328	0	2278

TRINITY_DN16012_c0_g1_i10	Glyma.17G010500.2	99.14529915	202	4	166	2019	469	2328	0	2278

TRINITY_DN37131_c0_g1_i1	Glyma.16G025400.1	97.70992366	188	6	196	2050	1	1855	0	2329

TRINITY_DN14554_c0_g1_i1	Glyma.12G133700.1	99.11111111	179	7	207	2048	2073	214	0	2327

TRINITY_DN15216_c0_g2_i1	Glyma.08G131400.4	100	177	8	73	1931	11	1854	0	2344

TRINITY_DN15068_c0_g1_i1	Glyma.06G082300.1	99.18699187	175	14	1222	3067	1863	15	0	2303

TRINITY_DN16012_c0_g1_i10	Glyma.20G066800.1	100	171	9	166	2019	469	2325	0	2429

TRINITY_DN16012_c0_g1_i10	Glyma.13G002200.1	100	168	9	166	2019	469	2325	0	2446

TRINITY_DN9638_c0_g1_i2	Glyma.20G161000.1	100	166	11	525	2376	1840	1	0	2335

TRINITY_DN14809_c0_g1_i5	Glyma.20G030100.1	95.8	165	2	405	2261	1860	1	0	2501

TRINITY_DN14809_c0_g1_i4	Glyma.20G030100.1	97.88359788	159	2	405	2261	1860	1	0	2534

TRINITY_DN11244_c0_g1_i1	Glyma.19G117700.1	99.07407407	159	11	222	2056	1	1856	0	2366

TRINITY_DN11244_c0_g1_i1	Glyma.19G117700.3	100	159	12	222	2056	1	1850	0	2327

TRINITY_DN11244_c0_g1_i1	Glyma.19G117700.2	97.99196787	159	12	222	2056	1	1850	0	2327

TRINITY_DN15148_c0_g1_i1	Glyma.16G012100.2	98.93617021	155	19	1212	3050	2955	1111	0	2344

TRINITY_DN15148_c0_g1_i2	Glyma.16G012100.2	95.37037037	155	19	1336	3174	2955	1111	0	2344

TRINITY_DN15217_c0_g1_i2	Glyma.20G166300.1	99.01477833	154	9	236	2086	1	1839	0	2388

TRINITY_DN16211_c1_g2_i1	Glyma.15G124600.1	96.13259669	148	3	159	2018	1	1857	0	2595

TRINITY_DN15619_c0_g2_i1	Glyma.05G038300.1	97.21115538	137	0	1188	3047	182	2041	0	2676

TRINITY_DN15217_c0_g1_i2	Glyma.10G225500.1	99.01477833	137	10	236	2086	1	1839	0	2483

TRINITY_DN4541_c0_g2_i1	Glyma.12G017700.1	98.97959184	131	9	6	1856	1	1854	0	2612

TRINITY_DN15521_c0_g1_i3	Glyma.12G155900.1	97.37903226	115	0	1355	3214	1176	3035	0	2798

TRINITY_DN9079_c0_g1_i1	Glyma.08G046400.1	100	93	3	247	2106	1	1854	0	2881

TRINITY_DN9079_c0_g1_i1	Glyma.05G239300.1	100	88	3	247	2106	1	1854	0	2909

TRINITY_DN14554_c0_g1_i1	Glyma.12G219000.1	95.51282051	325	23	207	2053	2052	209	0	1458

TRINITY_DN9320_c0_g2_i1	Glyma.16G110100.1	97.24770642	302	5	223	2075	99	1956	0	1714

TRINITY_DN14364_c0_g2_i4	Glyma.07G242000.1	98.66071429	248	8	302	2145	2757	902	0	1943

TRINITY_DN14364_c0_g2_i5	Glyma.07G242000.1	100	248	8	302	2145	2757	902	0	1943

TRINITY_DN14364_c0_g2_i4	Glyma.07G242000.2	99.57446809	248	8	302	2145	2706	851	0	1943

TRINITY_DN14364_c0_g2_i5	Glyma.07G242000.2	98.08917197	248	8	302	2145	2706	851	0	1943

TRINITY_DN5733_c0_g2_i2	Glyma.11G062000.2	98.24561404	245	20	292	2131	1893	51	0	1849

TRINITY_DN5733_c0_g2_i2	Glyma.11G062000.1	98.95615866	245	20	292	2131	1893	51	0	1849

TRINITY_DN15327_c1_g1_i5	Glyma.14G045000.1	95.22357724	226	2	249	2106	1869	12	0	2169

TRINITY_DN13349_c1_g3_i2	Glyma.08G309000.2	99.15966387	222	17	716	2550	1859	19	0	1932

TRINITY_DN14967_c1_g1_i1	Glyma.20G143500.1	100	199	5	383	2241	250	2102	0	2292

TRINITY_DN14244_c0_g4_i2	Glyma.12G072700.3	96.20253165	197	7	186	2042	1875	22	0	2296

TRINITY_DN12875_c0_g1_i2	Glyma.07G126200.1	99.33333333	195	4	370	2226	1857	1	0	2327

TRINITY_DN12875_c0_g1_i1	Glyma.07G126200.1	99.33774834	190	13	370	2206	1857	1	0	2226

TRINITY_DN15216_c0_g2_i1	Glyma.05G174200.4	100	181	2	74	1931	51	1908	0	2418

TRINITY_DN20853_c0_g1_i1	Glyma.08G121900.1	100	149	7	182	2030	114	1971	0	2536

TRINITY_DN15216_c0_g2_i2	Glyma.08G131400.4	96.68508287	139	6	73	1931	11	1854	0	2566

TRINITY_DN16365_c0_g1_i1	Glyma.05G155100.1	97.61904762	131	12	253	2099	1	1841	0	2514

TRINITY_DN16365_c0_g1_i1	Glyma.05G155100.2	97.59615385	131	12	253	2099	1	1841	0	2514

TRINITY_DN16365_c0_g1_i6	Glyma.05G155100.1	97.5	130	12	303	2149	1	1841	0	2519

TRINITY_DN16365_c0_g1_i6	Glyma.05G155100.2	97.5	130	12	303	2149	1	1841	0	2519

TRINITY_DN15736_c0_g1_i2	Glyma.05G198700.1	98.98648649	295	9	556	2408	388	2240	0	1733

TRINITY_DN16237_c0_g1_i2	Glyma.06G164900.2	97.33333333	261	29	923	2759	513	2346	0	1694

TRINITY_DN15321_c1_g3_i1	Glyma.11G240900.2	97.22222222	258	16	189	2032	36	1878	0	1814

TRINITY_DN15321_c1_g3_i1	Glyma.11G240900.4	100	258	16	189	2032	36	1878	0	1814

TRINITY_DN15321_c1_g3_i1	Glyma.11G240900.3	99.27007299	258	16	189	2032	36	1878	0	1814

TRINITY_DN11718_c0_g1_i2	Glyma.09G204600.2	97.18309859	249	42	252	2071	2106	287	0	1561

TRINITY_DN16257_c1_g1_i12	Glyma.12G191800.1	98.41269841	240	15	193	2039	2219	376	0	1940

TRINITY_DN16547_c0_g1_i1	Glyma.06G141400.1	99.7382199	184	11	1	1814	1099	2946	0	2063

TRINITY_DN15068_c0_g1_i1	Glyma.04G080600.1	95.65217391	178	14	1222	3067	1854	15	0	2252

TRINITY_DN15936_c0_g2_i2	Glyma.06G285400.1	98.07692308	151	4	28	1884	175	2028	0	2562

TRINITY_DN15936_c0_g2_i2	Glyma.12G120300.1	98.70967742	151	4	28	1884	175	2028	0	2562

TRINITY_DN13543_c1_g1_i1	Glyma.06G108800.1	99.28315412	151	12	444	2288	1848	1	0	2446

TRINITY_DN15216_c0_g2_i2	Glyma.05G174200.4	100	143	0	74	1931	51	1908	0	2639

TRINITY_DN14432_c0_g1_i2	Glyma.U029000.4	100	123	8	210	2052	88	1942	0	2634

TRINITY_DN14432_c0_g1_i2	Glyma.U029000.3	98.75	123	8	210	2052	88	1942	0	2634

TRINITY_DN14432_c0_g1_i2	Glyma.U029000.1	100	123	8	210	2052	88	1942	0	2634

TRINITY_DN14432_c0_g1_i2	Glyma.U029000.2	100	123	8	210	2052	88	1942	0	2634

TRINITY_DN35745_c0_g1_i1	Glyma.02G071700.1	96.07142857	338	56	150	1925	1839	1	0	917

TRINITY_DN35745_c0_g1_i1	Glyma.02G071700.2	96.07250755	338	56	150	1925	1839	1	0	917

TRINITY_DN5733_c0_g1_i1	Glyma.11G062000.2	100	274	20	337	2174	1891	51	0	1685

TRINITY_DN5733_c0_g1_i1	Glyma.11G062000.1	97.5	274	20	337	2174	1891	51	0	1685

TRINITY_DN12981_c0_g2_i5	Glyma.03G102800.1	100	251	8	151	1999	1	1855	0	1975

TRINITY_DN8156_c0_g1_i1	Glyma.18G044900.1	96.55172414	230	36	191	1987	1848	1	0	1709

TRINITY_DN4954_c0_g1_i1	Glyma.12G128600.1	97.4789916	203	8	189	2041	2136	284	0	2254

TRINITY_DN53_c0_g1_i1	Glyma.03G101700.1	98.4375	173	6	290	2143	1854	1	0	2433

TRINITY_DN15750_c1_g1_i2	Glyma.09G062900.1	97.40932642	170	1	1047	2903	1854	1	0	2468

TRINITY_DN15750_c1_g1_i2	Glyma.09G062900.2	97.4137931	170	1	1047	2903	1854	1	0	2468

TRINITY_DN15750_c1_g1_i5	Glyma.09G062900.1	99.28057554	170	1	962	2818	1854	1	0	2468

TRINITY_DN15750_c1_g1_i5	Glyma.09G062900.2	97.2826087	170	1	962	2818	1854	1	0	2468

TRINITY_DN15750_c1_g1_i2	Glyma.15G169500.2	97.08737864	159	1	1047	2903	1854	1	0	2529

TRINITY_DN15750_c1_g1_i2	Glyma.15G169500.1	96.36363636	159	1	1047	2903	1854	1	0	2529

TRINITY_DN15750_c1_g1_i5	Glyma.15G169500.2	95.68965517	159	1	962	2818	1854	1	0	2529

TRINITY_DN15750_c1_g1_i5	Glyma.15G169500.1	98.48484848	159	1	962	2818	1854	1	0	2529

TRINITY_DN12981_c0_g2_i4	Glyma.08G340200.1	99.18699187	155	6	150	2005	3	1849	0	2499

TRINITY_DN16386_c0_g1_i3	Glyma.16G198200.1	97.74011299	147	7	1	1853	764	2610	0	2525

TRINITY_DN11072_c0_g1_i1	Glyma.18G245200.2	99.43820225	134	2	744	2597	1857	1	0	2667

TRINITY_DN12452_c0_g1_i1	Glyma.17G145700.1	96.12068966	126	2	141	1991	1	1857	0	2693

TRINITY_DN15619_c0_g2_i4	Glyma.17G088800.1	97.82608696	126	2	912	2767	1	1856	0	2719

TRINITY_DN15697_c0_g4_i2	Glyma.08G309500.1	100	113	9	641	2491	1848	1	0	2706

TRINITY_DN15697_c0_g4_i2	Glyma.18G106800.1	97.6744186	107	5	641	2491	1845	1	0	2721

TRINITY_DN9294_c0_g1_i1	Glyma.14G129700.1	97.95918367	320	44	21	1833	1792	3616	0	1177

TRINITY_DN16218_c0_g5_i14	Glyma.03G246100.4	95.0617284	313	22	424	2267	1830	1	0	1448

TRINITY_DN16218_c0_g5_i14	Glyma.03G246100.2	95.0617284	313	22	424	2267	1830	1	0	1448

TRINITY_DN16218_c0_g5_i14	Glyma.03G246100.1	95.0617284	313	22	424	2267	1830	1	0	1448

TRINITY_DN9320_c0_g2_i1	Glyma.16G110100.2	98.75776398	299	6	223	2075	99	1944	0	1687

TRINITY_DN16372_c0_g1_i6	Glyma.03G101200.1	98.30508475	210	9	633	2486	1842	1	0	2161

TRINITY_DN14326_c0_g3_i1	Glyma.11G074400.1	96.47887324	207	18	276	2113	146	1968	0	1951

TRINITY_DN12981_c0_g2_i4	Glyma.03G102800.1	100	177	2	151	2005	1	1855	0	2435

TRINITY_DN10463_c0_g1_i1	Glyma.19G235100.3	96.03960396	161	9	618	2471	1845	1	0	2451

TRINITY_DN16237_c0_g1_i11	Glyma.08G008100.2	98.7654321	159	12	904	2744	31	1874	0	2372

TRINITY_DN16237_c0_g1_i3	Glyma.08G008100.2	100	159	12	1008	2848	31	1874	0	2372

TRINITY_DN16237_c0_g1_i5	Glyma.08G008100.2	98.73417722	159	12	904	2744	31	1874	0	2372

TRINITY_DN14452_c0_g1_i2	Glyma.07G015500.2	100	143	12	266	2100	1	1844	0	2422

TRINITY_DN19231_c0_g1_i1	Glyma.17G250400.1	96.66666667	132	12	3	1844	91	1941	0	2573

TRINITY_DN15619_c0_g2_i4	Glyma.05G038300.1	100	114	0	912	2767	1	1856	0	2796

TRINITY_DN19780_c0_g1_i1	Glyma.19G116700.2	97.64705882	101	5	832	2685	1883	31	0	2835

TRINITY_DN9378_c0_g1_i1	Glyma.09G191400.3	96.15384615	84	2	835	2687	1856	1	0	2942

TRINITY_DN15034_c0_g1_i1	Glyma.15G074000.5	100	377	33	3500	5336	2265	429	0	1105

TRINITY_DN15034_c0_g1_i1	Glyma.15G074000.6	95.40229885	377	33	3500	5336	2265	429	0	1105

TRINITY_DN15034_c0_g1_i1	Glyma.15G074000.2	95.40229885	377	33	3500	5336	2265	429	0	1105

TRINITY_DN15034_c0_g1_i1	Glyma.15G074000.1	98.36065574	377	33	3500	5336	2265	429	0	1105

TRINITY_DN15034_c0_g1_i1	Glyma.15G074000.4	99.32885906	377	33	3500	5336	2265	429	0	1105

TRINITY_DN15034_c0_g1_i1	Glyma.15G074000.3	96.09375	377	33	3500	5336	2265	429	0	1105

TRINITY_DN16237_c0_g1_i2	Glyma.04G200600.2	100	266	23	923	2759	513	2346	0	1700

TRINITY_DN16237_c0_g1_i2	Glyma.06G164900.1	98.58490566	261	29	923	2756	513	2343	0	1688

TRINITY_DN16237_c0_g1_i3	Glyma.06G164900.2	100	261	29	923	2756	513	2343	0	1688

TRINITY_DN15697_c0_g4_i2	Glyma.14G028100.1	100	260	11	641	2491	1836	1	0	1836

TRINITY_DN15697_c0_g4_i2	Glyma.02G286800.4	95.83333333	256	14	641	2491	1836	1	0	1858

TRINITY_DN12981_c0_g2_i5	Glyma.07G120900.1	99.45652174	241	5	151	1999	1	1855	0	2052

TRINITY_DN12944_c0_g1_i1	Glyma.U021100.11	96.05633803	202	18	181	2025	1848	16	0	2100

TRINITY_DN12944_c0_g1_i1	Glyma.U021100.9	100	202	18	181	2025	1848	16	0	2100

TRINITY_DN12944_c0_g1_i1	Glyma.U021100.6	97.64705882	202	18	181	2025	1848	16	0	2100

TRINITY_DN12944_c0_g1_i1	Glyma.U021100.3	96.76806084	202	18	181	2025	1848	16	0	2100

TRINITY_DN12944_c0_g1_i2	Glyma.U021100.11	97.5	202	18	181	2025	1848	16	0	2100

TRINITY_DN12944_c0_g1_i2	Glyma.U021100.9	100	202	18	181	2025	1848	16	0	2100

TRINITY_DN12944_c0_g1_i2	Glyma.U021100.6	98.98989899	202	18	181	2025	1848	16	0	2100

TRINITY_DN12944_c0_g1_i2	Glyma.U021100.3	96.66666667	202	18	181	2025	1848	16	0	2100

TRINITY_DN12944_c0_g1_i3	Glyma.U021100.11	97.08029197	202	18	181	2025	1848	16	0	2100

TRINITY_DN12944_c0_g1_i3	Glyma.U021100.9	98.9010989	202	18	181	2025	1848	16	0	2100

TRINITY_DN12944_c0_g1_i3	Glyma.U021100.6	100	202	18	181	2025	1848	16	0	2100

TRINITY_DN12944_c0_g1_i3	Glyma.U021100.3	95.65217391	202	18	181	2025	1848	16	0	2100

TRINITY_DN12944_c0_g1_i1	Glyma.12G072200.8	96.05568445	201	20	181	2025	1848	16	0	2106

TRINITY_DN12944_c0_g1_i2	Glyma.12G072200.8	96	201	20	181	2025	1848	16	0	2106

TRINITY_DN12944_c0_g1_i3	Glyma.12G072200.8	97.05882353	201	20	181	2025	1848	16	0	2106

TRINITY_DN12017_c0_g1_i1	Glyma.18G028600.1	98.11320755	175	12	203	2041	1839	1	0	2250

TRINITY_DN12981_c0_g2_i4	Glyma.07G120900.1	100	155	0	151	2005	1	1855	0	2567

TRINITY_DN13543_c1_g1_i1	Glyma.06G108800.3	99.2970123	151	11	444	2288	1845	1	0	2460

TRINITY_DN13543_c1_g1_i1	Glyma.06G108800.4	99.28571429	151	11	444	2288	1845	1	0	2460

TRINITY_DN13543_c1_g1_i1	Glyma.06G108800.5	99.29245283	151	11	444	2288	1845	1	0	2460

TRINITY_DN13543_c1_g1_i1	Glyma.06G108800.2	96.2962963	151	11	444	2288	1845	1	0	2460

TRINITY_DN10000_c0_g1_i2	Glyma.12G007800.3	97.05882353	148	20	2368	4196	1896	70	0	2257

TRINITY_DN10000_c0_g1_i2	Glyma.12G007800.2	96.80511182	148	20	2368	4196	1896	70	0	2257

TRINITY_DN10000_c0_g1_i2	Glyma.12G007800.1	95.74909279	148	20	2368	4196	1896	70	0	2257

TRINITY_DN10000_c0_g1_i2	Glyma.12G007800.6	99.3006993	148	20	2368	4196	1896	70	0	2257

TRINITY_DN10000_c0_g1_i3	Glyma.12G007800.3	96.86098655	148	20	2638	4466	1896	70	0	2257

TRINITY_DN10000_c0_g1_i3	Glyma.12G007800.2	96.64804469	148	20	2638	4466	1896	70	0	2257

TRINITY_DN10000_c0_g1_i3	Glyma.12G007800.1	100	148	20	2638	4466	1896	70	0	2257

TRINITY_DN10000_c0_g1_i3	Glyma.12G007800.6	97.01986755	148	20	2638	4466	1896	70	0	2257

TRINITY_DN12811_c0_g1_i7	Glyma.09G183900.1	97.72727273	132	16	1	1849	480	2313	0	2519

TRINITY_DN12811_c0_g1_i8	Glyma.09G183900.1	97.10144928	132	16	1	1849	480	2313	0	2519

TRINITY_DN12365_c0_g1_i1	Glyma.03G137100.2	96.84813754	116	0	191	2045	1	1855	0	2784

TRINITY_DN16081_c0_g1_i1	Glyma.13G097600.1	95.77464789	108	11	164	2002	1	1854	0	2717

TRINITY_DN16345_c0_g1_i12	Glyma.07G093700.2	99.2248062	102	6	3608	5459	1849	1	0	2802

TRINITY_DN16345_c0_g1_i12	Glyma.07G093700.1	99.21875	102	6	3608	5459	1849	1	0	2802

TRINITY_DN16345_c0_g1_i4	Glyma.07G093700.1	98.4496124	102	6	2395	4246	1849	1	0	2802

TRINITY_DN16345_c0_g1_i4	Glyma.07G093700.2	98.45360825	102	6	2395	4246	1849	1	0	2802

TRINITY_DN15034_c0_g1_i1	Glyma.13G239300.1	100	374	32	3500	5336	2193	357	0	1133

TRINITY_DN10651_c0_g2_i1	Glyma.05G149700.1	99.00990099	335	21	379	2222	3338	1507	0	1362

TRINITY_DN14809_c0_g2_i1	Glyma.07G227700.1	100	318	32	387	2216	1833	1	0	1371

TRINITY_DN15937_c0_g1_i1	Glyma.12G155700.2	95.14563107	290	31	544	2389	3	1803	0	1423

TRINITY_DN15937_c0_g1_i2	Glyma.12G155700.2	96.875	290	31	544	2389	3	1803	0	1423

TRINITY_DN15937_c0_g1_i3	Glyma.12G155700.2	99.76415094	290	31	544	2389	3	1803	0	1423

TRINITY_DN15937_c0_g1_i5	Glyma.12G155700.2	96.37462236	290	31	544	2389	3	1803	0	1423

TRINITY_DN13544_c0_g1_i3	Glyma.02G120600.1	98.03921569	250	29	24	1856	1833	1	0	1768

TRINITY_DN15195_c0_g1_i1	Glyma.06G001900.1	100	200	9	91	1924	1	1852	0	2174

TRINITY_DN15195_c0_g1_i2	Glyma.06G001900.1	96.2962963	200	9	91	1924	1	1852	0	2174

TRINITY_DN15195_c0_g1_i3	Glyma.06G001900.1	96.51162791	200	9	91	1924	1	1852	0	2174

TRINITY_DN16211_c1_g2_i1	Glyma.09G018500.1	98.61751152	151	6	167	2018	9	1857	0	2542

TRINITY_DN14624_c0_g1_i1	Glyma.05G239100.1	95.63218391	149	3	499	2352	1851	1	0	2579

TRINITY_DN5720_c0_g1_i1	Glyma.20G189900.2	99.5412844	142	0	350	2203	2163	310	0	2638

TRINITY_DN14624_c0_g1_i1	Glyma.08G046200.1	99.54441913	140	3	499	2352	1851	1	0	2628

TRINITY_DN14254_c0_g2_i1	Glyma.11G047200.1	99.0990991	131	15	166	2004	1824	1	0	2407

TRINITY_DN11833_c0_g1_i2	Glyma.10G292100.1	99.73404255	123	4	177	2020	1	1853	0	2671

TRINITY_DN16081_c0_g1_i1	Glyma.17G062000.1	100	118	9	164	2002	1	1854	0	2673

TRINITY_DN14517_c0_g1_i1	Glyma.12G027500.1	100	113	0	1398	3251	1267	3120	0	2798

TRINITY_DN14517_c0_g1_i1	Glyma.11G101600.1	99.82517483	102	0	1398	3251	1264	3117	0	2859

TRINITY_DN16054_c1_g1_i2	Glyma.10G168700.3	100	89	0	893	2746	1854	1	0	2931

TRINITY_DN15369_c0_g2_i1	Glyma.17G072400.1	100	389	23	18	1858	1862	22	0	1112

TRINITY_DN15369_c0_g1_i1	Glyma.02G205600.1	98.27586207	373	21	397	2238	1863	22	0	1214

TRINITY_DN8982_c0_g2_i1	Glyma.15G017000.1	95	316	21	380	2212	1847	1	0	1504

TRINITY_DN8982_c0_g1_i1	Glyma.15G017000.1	100	315	21	380	2212	1847	1	0	1509

TRINITY_DN15235_c0_g1_i7	Glyma.16G172500.1	99.68253968	217	10	747	2592	1211	3055	0	2122

TRINITY_DN16311_c0_g1_i4	Glyma.06G182100.3	97.36842105	193	5	855	2702	889	2739	0	2307

TRINITY_DN16311_c0_g1_i4	Glyma.06G182100.2	96.55172414	193	5	855	2702	889	2739	0	2307

TRINITY_DN16311_c0_g1_i4	Glyma.06G182100.1	96.55543595	193	5	855	2702	889	2739	0	2307

TRINITY_DN11337_c0_g1_i1	Glyma.13G353200.9	97.84102061	186	17	166	2001	1836	1	0	2172

TRINITY_DN11337_c0_g1_i1	Glyma.13G353200.8	97.84102061	186	17	166	2001	1836	1	0	2172

TRINITY_DN11337_c0_g1_i1	Glyma.13G353200.11	98.68421053	186	17	166	2001	1836	1	0	2172

TRINITY_DN11337_c0_g1_i1	Glyma.13G353200.10	98.68421053	186	17	166	2001	1836	1	0	2172

TRINITY_DN11337_c0_g1_i1	Glyma.13G353200.1	95.47738693	186	17	166	2001	1836	1	0	2172

TRINITY_DN14936_c0_g2_i2	Glyma.17G223000.1	96.2962963	179	9	75	1919	1	1851	0	2366

TRINITY_DN15417_c0_g2_i1	Glyma.06G156500.1	100	175	7	1094	2930	2178	342	0	2246

TRINITY_DN14936_c0_g2_i1	Glyma.17G223000.1	96.2962963	151	9	374	2218	1	1851	0	2521

TRINITY_DN10212_c0_g1_i1	Glyma.15G192300.1	100	137	2	1	1852	2234	383	0	2651

TRINITY_DN14517_c0_g1_i3	Glyma.12G027500.2	95.41984733	113	0	1473	3325	1202	3054	0	2796

TRINITY_DN14517_c0_g1_i3	Glyma.11G101600.2	95.41984733	102	0	1473	3325	1199	3051	0	2857

TRINITY_DN14973_c1_g2_i7	Glyma.09G031400.2	100	95	1	1141	2993	1852	1	0	2889

TRINITY_DN14973_c1_g2_i7	Glyma.09G031400.3	100	95	1	1141	2993	1852	1	0	2889

TRINITY_DN14973_c1_g2_i7	Glyma.09G031400.1	100	95	1	1141	2993	1852	1	0	2889

TRINITY_DN14973_c1_g2_i9	Glyma.09G031400.2	100	95	1	1141	2993	1852	1	0	2889

TRINITY_DN14973_c1_g2_i9	Glyma.09G031400.3	100	95	1	1141	2993	1852	1	0	2889

TRINITY_DN14973_c1_g2_i9	Glyma.09G031400.1	100	95	1	1141	2993	1852	1	0	2889

TRINITY_DN16218_c0_g5_i8	Glyma.03G246100.4	96.11650485	332	22	424	2262	1830	1	0	1354

TRINITY_DN16218_c0_g5_i8	Glyma.03G246100.2	96.55172414	332	22	424	2262	1830	1	0	1354

TRINITY_DN16218_c0_g5_i8	Glyma.03G246100.1	98.91304348	332	22	424	2262	1830	1	0	1354

TRINITY_DN3110_c0_g1_i1	Glyma.05G138600.1	95.83333333	319	25	517	2330	1941	99	0	1349

TRINITY_DN3772_c0_g1_i1	Glyma.11G155500.1	97.6744186	294	33	9	1819	428	2265	0	1435

TRINITY_DN10095_c0_g1_i1	Glyma.06G187400.2	100	289	29	144	1971	23	1847	0	1489

TRINITY_DN10095_c0_g1_i1	Glyma.06G187400.1	96.55172414	289	29	144	1971	23	1847	0	1489

TRINITY_DN16237_c0_g1_i2	Glyma.04G200600.1	96.81908549	266	23	923	2756	513	2343	0	1694

TRINITY_DN16237_c0_g1_i3	Glyma.04G200600.2	98.25327511	266	23	923	2756	513	2343	0	1694

TRINITY_DN15521_c0_g1_i3	Glyma.13G266300.1	95.83333333	260	25	1361	3198	1200	3037	0	1799

TRINITY_DN15321_c1_g3_i1	Glyma.11G240900.1	96.875	257	16	195	2032	66	1902	0	1808

TRINITY_DN10931_c0_g1_i1	Glyma.04G035100.1	100	247	21	201	2030	2139	307	0	1786

TRINITY_DN10931_c0_g1_i1	Glyma.04G035100.2	97.91666667	246	22	201	2030	2136	307	0	1773

TRINITY_DN10931_c0_g1_i1	Glyma.06G035000.1	98.4375	246	25	201	2030	2130	304	0	1753

TRINITY_DN16138_c1_g1_i13	Glyma.08G077100.3	98.07692308	197	7	566	2392	1	1850	0	2154

TRINITY_DN16311_c0_g1_i4	Glyma.13G146900.2	96.55172414	197	11	855	2702	901	2733	0	2180

TRINITY_DN16311_c0_g1_i3	Glyma.13G146900.2	96.55364091	168	4	855	2702	901	2733	0	2340

TRINITY_DN16311_c0_g1_i3	Glyma.06G182100.3	96.55172414	162	3	855	2702	889	2739	0	2490

TRINITY_DN16311_c0_g1_i3	Glyma.06G182100.2	96.55172414	162	3	855	2702	889	2739	0	2490

TRINITY_DN16311_c0_g1_i3	Glyma.06G182100.1	96.55172414	162	3	855	2702	889	2739	0	2490

TRINITY_DN13543_c1_g1_i1	Glyma.04G253600.4	99.26470588	162	9	444	2288	1830	1	0	2335

TRINITY_DN13543_c1_g1_i1	Glyma.04G253600.2	99.26470588	162	9	444	2288	1830	1	0	2335

TRINITY_DN13543_c1_g1_i1	Glyma.04G253600.1	99.28571429	162	9	444	2288	1830	1	0	2335

TRINITY_DN12139_c0_g1_i2	Glyma.08G332000.1	98.50187266	158	13	90	1925	1	1845	0	2396

TRINITY_DN15683_c0_g2_i4	Glyma.07G005200.3	96.11940299	143	12	257	2087	17	1865	0	2473

TRINITY_DN6062_c0_g1_i2	Glyma.10G159100.3	97.27272727	136	9	299	2138	98	1935	0	2499

TRINITY_DN6062_c0_g1_i2	Glyma.10G159100.1	97.28506787	136	9	299	2138	98	1935	0	2499

TRINITY_DN6062_c0_g1_i2	Glyma.10G159100.2	97.2972973	136	9	299	2138	98	1935	0	2499

TRINITY_DN16345_c0_g1_i12	Glyma.09G182600.1	99.23076923	103	4	3608	5459	1843	1	0	2791

TRINITY_DN16345_c0_g1_i4	Glyma.09G182600.1	98.4375	103	4	2395	4246	1843	1	0	2791

TRINITY_DN12229_c0_g2_i1	Glyma.06G069600.1	96.15384615	83	0	1	1852	1404	3255	0	2961

TRINITY_DN12229_c0_g2_i1	Glyma.06G069600.2	96.15384615	83	0	1	1852	945	2796	0	2961

TRINITY_DN12229_c0_g2_i1	Glyma.04G067900.1	96.15384615	72	0	1	1852	1404	3255	0	3022

TRINITY_DN15369_c0_g6_i1	Glyma.02G205600.1	100	409	20	601	2440	1861	22	0	1011

TRINITY_DN8153_c0_g1_i1	Glyma.05G194000.2	98.71794872	398	31	274	2110	1863	35	0	981

TRINITY_DN8153_c0_g1_i1	Glyma.05G194000.1	96.67194929	398	31	274	2110	1863	35	0	981

TRINITY_DN16218_c0_g5_i1	Glyma.03G246100.4	98.97959184	314	20	365	2203	1830	1	0	1465

TRINITY_DN16218_c0_g5_i1	Glyma.03G246100.2	97.01492537	314	20	365	2203	1830	1	0	1465

TRINITY_DN16218_c0_g5_i1	Glyma.03G246100.1	97.77777778	314	20	365	2203	1830	1	0	1465

TRINITY_DN16218_c0_g5_i9	Glyma.03G246100.4	97.82608696	314	20	365	2203	1830	1	0	1465

TRINITY_DN16218_c0_g5_i9	Glyma.03G246100.2	97.82608696	314	20	365	2203	1830	1	0	1465

TRINITY_DN16218_c0_g5_i9	Glyma.03G246100.1	98.06451613	314	20	365	2203	1830	1	0	1465

TRINITY_DN15195_c0_g1_i1	Glyma.06G001900.3	98.7012987	200	8	91	1924	1	1849	0	2187

TRINITY_DN15195_c0_g1_i2	Glyma.06G001900.3	98.64864865	200	8	91	1924	1	1849	0	2187

TRINITY_DN15195_c0_g1_i3	Glyma.06G001900.3	99.67948718	200	8	91	1924	1	1849	0	2187

TRINITY_DN16138_c1_g1_i10	Glyma.08G077100.3	97.08879185	197	6	285	2122	1	1849	0	2230

TRINITY_DN16138_c1_g1_i18	Glyma.08G077100.3	98.125	197	6	300	2137	1	1849	0	2230

TRINITY_DN16138_c1_g1_i5	Glyma.08G077100.3	100	197	6	566	2403	1	1849	0	2230

TRINITY_DN16138_c1_g1_i13	Glyma.08G077100.4	97.5308642	197	7	566	2391	1	1849	0	2152

TRINITY_DN16138_c1_g1_i13	Glyma.08G077100.1	95	197	7	566	2391	1	1849	0	2152

TRINITY_DN16138_c1_g1_i2	Glyma.08G077100.3	99.10714286	197	7	300	2125	1	1849	0	2152

TRINITY_DN16138_c1_g1_i20	Glyma.08G077100.3	98.7704918	197	7	300	2125	1	1849	0	2152

TRINITY_DN16138_c1_g1_i3	Glyma.08G077100.3	97.79411765	197	7	566	2391	1	1849	0	2152

TRINITY_DN16138_c1_g1_i7	Glyma.08G077100.3	96.42857143	197	7	566	2391	1	1849	0	2152

TRINITY_DN16138_c1_g1_i8	Glyma.08G077100.3	97.56097561	197	7	300	2125	1	1849	0	2152

TRINITY_DN16138_c1_g1_i9	Glyma.08G077100.3	95.49295775	197	7	285	2110	1	1849	0	2152

TRINITY_DN14936_c0_g2_i2	Glyma.14G102500.1	96.2962963	178	6	75	1919	1	1848	0	2374

TRINITY_DN14617_c0_g1_i1	Glyma.17G232600.1	99.20318725	177	62	259	2007	1	1803	0	1469

TRINITY_DN16237_c0_g1_i2	Glyma.05G200800.2	98.16513761	158	9	923	2759	567	2409	0	2401

TRINITY_DN16237_c0_g1_i2	Glyma.05G200800.3	95.79100145	158	9	923	2759	567	2409	0	2401

TRINITY_DN14667_c0_g1_i1	Glyma.15G011500.1	100	152	16	225	2055	1843	1	0	2396

TRINITY_DN14936_c0_g2_i1	Glyma.14G102500.1	96.2962963	148	6	374	2218	1	1848	0	2540

TRINITY_DN14953_c0_g2_i1	Glyma.04G123900.1	98.07692308	134	8	530	2373	1895	49	0	2604

TRINITY_DN12811_c0_g1_i7	Glyma.07G092300.1	97.72727273	133	17	1	1845	477	2309	0	2527

TRINITY_DN12811_c0_g1_i8	Glyma.07G092300.1	97.73584906	133	17	1	1845	477	2309	0	2527

TRINITY_DN15220_c0_g1_i4	Glyma.16G008400.1	95.38461538	129	13	509	2338	1	1839	0	2490

TRINITY_DN14492_c1_g1_i2	Glyma.03G192300.4	97.9020979	125	1	537	2384	1	1851	0	2706

TRINITY_DN14492_c1_g1_i4	Glyma.03G192300.4	95.65217391	125	1	537	2384	1	1851	0	2706

TRINITY_DN14827_c0_g1_i2	Glyma.19G188800.1	98.42519685	123	1	116	1966	175	2022	0	2717

TRINITY_DN14827_c0_g1_i6	Glyma.19G188800.1	100	123	1	116	1966	175	2022	0	2717

TRINITY_DN14827_c0_g1_i7	Glyma.19G188800.1	96.61458333	123	1	116	1966	175	2022	0	2717

TRINITY_DN10974_c0_g1_i1	Glyma.05G047900.1	96.64082687	114	0	222	2072	1875	25	0	2787

TRINITY_DN11476_c0_g1_i1	Glyma.08G168800.1	100	114	10	368	2203	1	1836	0	2593

TRINITY_DN14624_c0_g1_i3	Glyma.05G239100.1	95.48387097	91	0	499	2349	1851	1	0	2915

TRINITY_DN14624_c0_g1_i3	Glyma.08G046200.1	95.65217391	86	0	499	2349	1851	1	0	2942

TRINITY_DN16352_c0_g2_i1	Glyma.08G194700.2	100	344	36	2521	4348	1870	40	0	1245

TRINITY_DN15235_c0_g1_i3	Glyma.16G172500.1	95.23809524	211	6	838	2683	1211	3055	0	2189

TRINITY_DN9339_c0_g1_i1	Glyma.03G089300.1	100	202	17	128	1966	82	1920	0	2156

TRINITY_DN12944_c0_g1_i1	Glyma.U021100.1	95.58823529	202	18	181	2020	1854	27	0	2091

TRINITY_DN12944_c0_g1_i2	Glyma.U021100.1	96.60056657	202	18	181	2020	1854	27	0	2091

TRINITY_DN12944_c0_g1_i3	Glyma.U021100.1	100	202	18	181	2020	1854	27	0	2091

TRINITY_DN9339_c0_g1_i1	Glyma.03G089300.2	96.2962963	199	19	128	1966	82	1908	0	2095

TRINITY_DN11898_c0_g2_i3	Glyma.14G181100.1	98.75	193	21	66	1896	3138	1332	0	1947

TRINITY_DN16138_c1_g1_i11	Glyma.08G077100.3	96.35258359	184	3	311	2156	1	1849	0	2364

TRINITY_DN16386_c0_g1_i2	Glyma.16G198300.1	98.25581395	164	13	23	1868	816	2640	0	2320

TRINITY_DN16386_c0_g1_i2	Glyma.16G198300.2	98.25436409	164	13	23	1868	816	2640	0	2320

TRINITY_DN10216_c0_g1_i1	Glyma.03G229400.1	97.2972973	161	7	82	1925	1838	1	0	2409

TRINITY_DN16203_c0_g1_i1	Glyma.01G089900.1	97.02048417	143	24	85	1896	1	1836	0	2289

TRINITY_DN16138_c1_g1_i6	Glyma.08G077100.3	96	139	10	311	2145	1	1850	0	2549

TRINITY_DN14953_c0_g2_i1	Glyma.04G123900.2	98.07692308	135	8	530	2373	1892	49	0	2591

TRINITY_DN15683_c0_g2_i3	Glyma.07G005200.3	100	101	6	257	2088	17	1866	0	2741

TRINITY_DN12981_c0_g2_i5	Glyma.08G340200.1	99.45652174	80	1	150	1999	3	1849	0	2953

TRINITY_DN15369_c0_g6_i2	Glyma.02G205600.1	95.73459716	410	17	601	2440	1861	22	0	1027

TRINITY_DN119_c0_g1_i1	Glyma.20G239400.1	98.88888889	377	51	950	2757	1848	24	0	905

TRINITY_DN16218_c0_g5_i11	Glyma.03G246100.4	98.65513929	261	18	424	2262	1830	1	0	1781

TRINITY_DN16218_c0_g5_i11	Glyma.03G246100.2	100	261	18	424	2262	1830	1	0	1781

TRINITY_DN16218_c0_g5_i11	Glyma.03G246100.1	96.23059867	261	18	424	2262	1830	1	0	1781

TRINITY_DN16218_c0_g5_i13	Glyma.03G246100.4	96.26556017	261	18	424	2262	1830	1	0	1781

TRINITY_DN16218_c0_g5_i13	Glyma.03G246100.2	97.95918367	261	18	424	2262	1830	1	0	1781

TRINITY_DN16218_c0_g5_i13	Glyma.03G246100.1	96.62921348	261	18	424	2262	1830	1	0	1781

TRINITY_DN15867_c0_g3_i4	Glyma.17G052600.2	96.67458432	215	6	615	2448	1	1846	0	2108

TRINITY_DN16091_c0_g1_i1	Glyma.02G076100.1	98.02955665	189	0	506	2354	27	1875	0	2368

TRINITY_DN15774_c0_g1_i2	Glyma.04G085900.2	97.39130435	181	5	1424	3268	1992	146	0	2374

TRINITY_DN15774_c0_g1_i3	Glyma.04G085900.2	95.57522124	181	5	2269	4113	1992	146	0	2374

TRINITY_DN16438_c0_g1_i3	Glyma.02G101800.1	100	149	13	134	1978	1	1834	0	2466

TRINITY_DN6062_c0_g1_i2	Glyma.20G229600.1	97.27722772	143	8	299	2138	92	1926	0	2473

TRINITY_DN6062_c0_g1_i2	Glyma.20G229600.2	97.2972973	143	8	299	2138	92	1926	0	2473

TRINITY_DN16138_c1_g1_i22	Glyma.08G077100.3	98.82352941	139	1	311	2156	1	1849	0	2625

TRINITY_DN16138_c1_g1_i17	Glyma.08G077100.3	96.63865546	139	10	311	2144	1	1849	0	2547

TRINITY_DN16138_c1_g1_i19	Glyma.08G077100.3	98.55072464	139	10	311	2144	1	1849	0	2547

TRINITY_DN16138_c1_g1_i21	Glyma.08G077100.3	100	139	10	311	2144	1	1849	0	2547

TRINITY_DN16138_c1_g1_i6	Glyma.08G077100.4	99.24098672	139	10	311	2144	1	1849	0	2547

TRINITY_DN16138_c1_g1_i6	Glyma.08G077100.1	97.36842105	139	10	311	2144	1	1849	0	2547

TRINITY_DN15323_c0_g3_i1	Glyma.03G140100.1	97.4025974	137	6	410	2242	1	1845	0	2527

TRINITY_DN15220_c0_g1_i4	Glyma.07G039600.1	95.07042254	135	12	509	2338	1	1836	0	2460

TRINITY_DN13484_c0_g3_i1	Glyma.06G094300.1	100	131	11	161	1993	1	1848	0	2579

TRINITY_DN14164_c0_g1_i4	Glyma.20G179900.1	98.92473118	92	6	111	1946	1	1836	0	2737

TRINITY_DN15369_c0_g1_i1	Glyma.17G072400.1	95.79349904	383	14	399	2238	1861	22	0	1188

TRINITY_DN12944_c0_g2_i1	Glyma.12G072200.8	100	343	38	181	1992	1848	28	0	1105

TRINITY_DN15789_c0_g1_i1	Glyma.04G032700.1	96.22641509	327	28	146	1969	870	2699	0	1330

TRINITY_DN15789_c0_g1_i1	Glyma.04G032700.4	98.57142857	327	28	146	1969	594	2423	0	1330

TRINITY_DN15789_c0_g1_i1	Glyma.04G032700.3	98.37398374	327	28	146	1969	594	2423	0	1330

TRINITY_DN15789_c0_g1_i1	Glyma.04G032700.2	98.46153846	327	28	146	1969	594	2423	0	1330

TRINITY_DN15062_c0_g1_i1	Glyma.11G075100.5	100	303	18	926	2763	1826	1	0	1528

TRINITY_DN15137_c0_g1_i11	Glyma.04G019700.1	100	284	8	1278	3121	1868	25	0	1788

TRINITY_DN15137_c0_g1_i7	Glyma.04G019700.1	99.16666667	284	8	1391	3234	1868	25	0	1788

TRINITY_DN16143_c0_g1_i1	Glyma.18G186200.1	100	279	29	1472	3275	121	1936	0	1376

TRINITY_DN9794_c0_g2_i1	Glyma.04G045800.3	95.62624254	257	17	550	2376	1	1839	0	1796

TRINITY_DN15970_c0_g2_i1	Glyma.18G188000.1	100	249	12	230	2055	31	1874	0	1866

TRINITY_DN15970_c0_g2_i9	Glyma.18G188000.1	95.14563107	249	12	230	2055	31	1874	0	1866

TRINITY_DN13448_c0_g1_i7	Glyma.08G100800.3	100	204	9	1	1840	27	1866	0	2180

TRINITY_DN13448_c0_g1_i7	Glyma.08G100800.2	100	204	9	1	1840	27	1866	0	2180

TRINITY_DN13448_c0_g1_i7	Glyma.08G100800.1	97.77777778	204	9	1	1840	27	1866	0	2180

TRINITY_DN12569_c0_g1_i5	Glyma.13G253200.1	98.27586207	198	11	69	1900	1	1844	0	2187

TRINITY_DN18011_c0_g1_i1	Glyma.04G250200.3	96.21621622	177	26	19	1839	1693	3507	0	2045

TRINITY_DN16237_c0_g1_i2	Glyma.08G008100.1	97.25557461	159	10	923	2756	549	2385	0	2370

TRINITY_DN16237_c0_g1_i2	Glyma.05G200800.4	96.22905028	158	9	923	2756	567	2406	0	2396

TRINITY_DN16237_c0_g1_i2	Glyma.05G200800.1	98.85057471	158	9	923	2756	567	2406	0	2396

TRINITY_DN16237_c0_g1_i3	Glyma.05G200800.2	96.34146341	158	9	923	2756	567	2406	0	2396

TRINITY_DN16237_c0_g1_i3	Glyma.05G200800.3	98.41269841	158	9	923	2756	567	2406	0	2396

TRINITY_DN3207_c0_g1_i1	Glyma.06G063500.1	99.41860465	154	24	1	1793	219	2060	0	2165

TRINITY_DN15323_c0_g3_i1	Glyma.19G143000.1	98.90710383	125	7	410	2242	1	1845	0	2604

TRINITY_DN15323_c0_g3_i1	Glyma.19G143000.2	97.4025974	125	7	410	2242	1	1845	0	2604

TRINITY_DN11833_c0_g1_i2	Glyma.10G292100.2	99.72144847	123	3	177	2020	1	1847	0	2699

TRINITY_DN1922_c0_g1_i1	Glyma.07G160500.1	100	122	0	161	2008	1	1848	0	2737

TRINITY_DN11254_c0_g1_i1	Glyma.07G034800.3	99.26470588	353	33	895	2720	1844	22	0	1164

TRINITY_DN6323_c0_g2_i2	Glyma.17G047900.1	98.48484848	286	27	66	1889	1	1830	0	1568

TRINITY_DN6323_c0_g2_i1	Glyma.17G047900.1	95.94594595	285	28	66	1886	1	1830	0	1554

TRINITY_DN36428_c0_g1_i1	Glyma.17G044700.1	95.79500657	250	30	287	2122	1812	1	0	1729

TRINITY_DN8153_c0_g1_i1	Glyma.06G155700.1	100	198	4	275	2115	1847	1	0	2276

TRINITY_DN16415_c1_g1_i13	Glyma.U018900.1	100	163	5	2357	4203	1854	17	0	2449

TRINITY_DN16415_c1_g1_i6	Glyma.U018900.1	96.32768362	163	5	2263	4109	1854	17	0	2449

TRINITY_DN2205_c0_g1_i1	Glyma.20G181400.2	98.80952381	147	4	5	1846	1853	11	0	2538

TRINITY_DN16203_c0_g1_i1	Glyma.01G089900.2	100	143	23	85	1896	1	1833	0	2302

TRINITY_DN14667_c0_g1_i1	Glyma.13G362300.1	96.73913043	138	16	225	2055	1837	1	0	2479

TRINITY_DN9702_c0_g2_i1	Glyma.04G069600.5	95	124	1	482	2313	2	1848	0	2627

TRINITY_DN13868_c0_g1_i10	Glyma.13G236700.3	100	124	2	171	2016	1	1846	0	2712

TRINITY_DN13868_c0_g1_i10	Glyma.13G236700.1	100	124	2	171	2016	1	1846	0	2712

TRINITY_DN13868_c0_g1_i4	Glyma.13G236700.3	100	124	2	171	2016	1	1846	0	2712

TRINITY_DN13868_c0_g1_i4	Glyma.13G236700.1	100	124	2	171	2016	1	1846	0	2712

TRINITY_DN13868_c0_g1_i5	Glyma.13G236700.3	98.9010989	124	2	171	2016	1	1846	0	2712

TRINITY_DN13868_c0_g1_i5	Glyma.13G236700.1	98.9010989	124	2	171	2016	1	1846	0	2712

TRINITY_DN13868_c0_g1_i9	Glyma.13G236700.3	99.48051948	124	2	171	2016	1	1846	0	2712

TRINITY_DN13868_c0_g1_i9	Glyma.13G236700.1	96.50059312	124	2	171	2016	1	1846	0	2712

TRINITY_DN2211_c0_g1_i1	Glyma.03G264400.5	100	82	1	1193	3036	1031	2877	0	2937

TRINITY_DN16004_c1_g1_i5	Glyma.03G113400.1	100	436	42	106	1930	367	2188	0	702

TRINITY_DN14809_c0_g2_i1	Glyma.20G030100.1	95.38461538	328	24	387	2216	1829	1	0	1378

TRINITY_DN12490_c0_g1_i1	Glyma.13G288300.1	98.36065574	297	3	381	2223	1997	152	0	1744

TRINITY_DN12490_c0_g1_i1	Glyma.12G213400.1	96.2962963	296	3	381	2223	2012	167	0	1749

TRINITY_DN15970_c0_g2_i1	Glyma.07G137300.1	100	249	8	230	2055	166	2009	0	1888

TRINITY_DN15970_c0_g2_i9	Glyma.07G137300.1	97.36842105	249	8	230	2055	166	2009	0	1888

TRINITY_DN12670_c0_g1_i4	Glyma.14G159600.1	98.36065574	220	10	270	2099	1	1845	0	2080

TRINITY_DN8982_c0_g1_i1	Glyma.08G204000.1	97.05882353	209	7	380	2225	1824	3	0	2097

TRINITY_DN4954_c0_g1_i1	Glyma.06G277000.1	97.4789916	208	12	189	2024	2157	316	0	2167

TRINITY_DN13987_c0_g1_i3	Glyma.20G244100.1	100	206	20	289	2112	1833	1	0	2041

TRINITY_DN8982_c0_g2_i1	Glyma.08G204000.1	100	187	7	380	2225	1824	3	0	2218

TRINITY_DN15606_c0_g1_i1	Glyma.15G070700.1	100	184	11	983	2818	973	2808	0	2261

TRINITY_DN15606_c0_g1_i2	Glyma.15G070700.1	96	184	11	1048	2883	973	2808	0	2261

TRINITY_DN15606_c0_g1_i1	Glyma.15G070700.2	95.3125	184	11	983	2818	628	2463	0	2261

TRINITY_DN15606_c0_g1_i2	Glyma.15G070700.2	100	184	11	1048	2883	628	2463	0	2261

TRINITY_DN13868_c0_g1_i1	Glyma.13G236700.3	96.875	162	0	171	2016	1	1846	0	2512

TRINITY_DN13868_c0_g1_i1	Glyma.13G236700.1	96.875	162	0	171	2016	1	1846	0	2512

TRINITY_DN13868_c0_g1_i3	Glyma.13G236700.3	99.20634921	162	0	171	2016	1	1846	0	2512

TRINITY_DN13868_c0_g1_i3	Glyma.13G236700.1	98.97959184	162	0	171	2016	1	1846	0	2512

TRINITY_DN13868_c0_g1_i6	Glyma.13G236700.3	97.36842105	162	0	171	2016	1	1846	0	2512

TRINITY_DN13868_c0_g1_i6	Glyma.13G236700.1	99.52606635	162	0	171	2016	1	1846	0	2512

TRINITY_DN13868_c0_g1_i8	Glyma.13G236700.3	100	162	0	171	2016	1	1846	0	2512

TRINITY_DN13868_c0_g1_i8	Glyma.13G236700.1	95.62682216	162	0	171	2016	1	1846	0	2512

TRINITY_DN13484_c0_g3_i2	Glyma.04G092400.2	95.23809524	142	9	161	1991	1	1834	0	2447

TRINITY_DN14745_c0_g1_i2	Glyma.17G019100.1	100	133	16	371	2203	1827	1	0	2466

TRINITY_DN9142_c0_g1_i1	Glyma.12G018300.2	99.73474801	129	0	677	2522	1846	1	0	2695

TRINITY_DN12896_c0_g2_i2	Glyma.08G337200.2	95.83333333	127	2	1	1845	452	2296	0	2693

TRINITY_DN12896_c0_g2_i2	Glyma.08G337200.1	95.83333333	127	2	1	1845	452	2296	0	2693

TRINITY_DN11476_c0_g1_i1	Glyma.15G258300.1	100	119	14	368	2203	1	1821	0	2523

TRINITY_DN2636_c0_g1_i1	Glyma.01G032500.1	95.99406528	116	2	1	1845	1943	99	0	2754

TRINITY_DN15678_c0_g1_i4	Glyma.07G241600.1	100	93	5	139	1972	1	1843	0	2796

TRINITY_DN12830_c0_g2_i1	Glyma.13G043800.1	98.75	304	15	222	2062	151	1976	0	1574

TRINITY_DN9320_c0_g1_i1	Glyma.01G154400.1	97.61904762	239	6	193	2034	141	1980	0	2032

TRINITY_DN9320_c0_g1_i1	Glyma.11G090200.1	97.61904762	232	8	194	2034	145	1983	0	2058

TRINITY_DN9142_c0_g1_i1	Glyma.13G000200.1	100	167	1	678	2522	1842	1	0	2462

TRINITY_DN10786_c0_g1_i1	Glyma.13G123700.1	100	153	2	375	2218	3702	1859	0	2547

TRINITY_DN8729_c0_g1_i1	Glyma.03G192900.1	99.43502825	153	10	355	2174	2940	1100	0	2372

TRINITY_DN3589_c0_g1_i1	Glyma.04G121300.1	95.53846154	125	0	157	2001	1	1845	0	2715

TRINITY_DN3589_c0_g1_i1	Glyma.06G317600.1	95.53903346	121	5	157	2001	1	1839	0	2699

TRINITY_DN14312_c0_g1_i4	Glyma.11G012000.4	100	117	1	563	2407	1842	1	0	2739

TRINITY_DN16081_c0_g1_i2	Glyma.17G062000.1	100	87	9	164	1993	1	1845	0	2828

TRINITY_DN11190_c0_g1_i1	Glyma.04G184000.1	99.21259843	428	33	692	2515	1824	1	0	776

TRINITY_DN11190_c0_g1_i1	Glyma.04G184000.3	97.09193246	428	33	692	2515	1824	1	0	776

TRINITY_DN14033_c0_g2_i1	Glyma.07G159600.1	98.4375	187	11	81	1914	2058	3891	0	2241

TRINITY_DN15648_c0_g2_i1	Glyma.10G153800.12	97.2027972	176	7	920	2757	2685	844	0	2379

TRINITY_DN15648_c0_g2_i1	Glyma.10G153800.10	98.93048128	176	7	920	2757	2685	844	0	2379

TRINITY_DN15648_c0_g2_i1	Glyma.10G153800.9	98.71794872	176	7	920	2757	2685	844	0	2379

TRINITY_DN15648_c0_g2_i1	Glyma.10G153800.8	100	176	7	920	2757	2685	844	0	2379

TRINITY_DN15648_c0_g2_i1	Glyma.10G153800.7	100	176	7	920	2757	2685	844	0	2379

TRINITY_DN15648_c0_g2_i1	Glyma.10G153800.6	95.23809524	176	7	920	2757	2685	844	0	2379

TRINITY_DN15648_c0_g2_i2	Glyma.10G153800.12	97.20149254	176	7	515	2352	2685	844	0	2379

TRINITY_DN15648_c0_g2_i2	Glyma.10G153800.10	95.73590096	176	7	515	2352	2685	844	0	2379

TRINITY_DN15648_c0_g2_i2	Glyma.10G153800.9	98.24561404	176	7	515	2352	2685	844	0	2379

TRINITY_DN15648_c0_g2_i2	Glyma.10G153800.8	100	176	7	515	2352	2685	844	0	2379

TRINITY_DN15648_c0_g2_i2	Glyma.10G153800.7	97.52066116	176	7	515	2352	2685	844	0	2379

TRINITY_DN15648_c0_g2_i2	Glyma.10G153800.6	95.94594595	176	7	515	2352	2685	844	0	2379

TRINITY_DN15648_c0_g2_i1	Glyma.10G153800.22	100	176	7	920	2757	2682	841	0	2379

TRINITY_DN15648_c0_g2_i1	Glyma.10G153800.21	100	176	7	920	2757	2682	841	0	2379

TRINITY_DN15648_c0_g2_i1	Glyma.10G153800.20	99.72451791	176	7	920	2757	2682	841	0	2379

TRINITY_DN15648_c0_g2_i1	Glyma.10G153800.19	100	176	7	920	2757	2682	841	0	2379

TRINITY_DN15648_c0_g2_i1	Glyma.10G153800.18	96.58119658	176	7	920	2757	2682	841	0	2379

TRINITY_DN15648_c0_g2_i2	Glyma.10G153800.22	100	176	7	515	2352	2682	841	0	2379

TRINITY_DN15648_c0_g2_i2	Glyma.10G153800.21	98	176	7	515	2352	2682	841	0	2379

TRINITY_DN15648_c0_g2_i2	Glyma.10G153800.20	96	176	7	515	2352	2682	841	0	2379

TRINITY_DN15648_c0_g2_i2	Glyma.10G153800.19	97.32142857	176	7	515	2352	2682	841	0	2379

TRINITY_DN15648_c0_g2_i2	Glyma.10G153800.18	98.85057471	176	7	515	2352	2682	841	0	2379

TRINITY_DN8179_c0_g1_i1	Glyma.20G185300.1	98.58757062	168	5	1	1823	414	2257	0	2338

TRINITY_DN16167_c0_g1_i10	Glyma.04G141400.6	100	148	6	692	2523	1805	1	0	2255

TRINITY_DN16167_c0_g1_i10	Glyma.04G141400.1	96.55172414	148	6	692	2523	1805	1	0	2255

TRINITY_DN16167_c0_g1_i10	Glyma.04G141400.7	97.10144928	148	6	692	2523	1805	1	0	2255

TRINITY_DN16167_c0_g1_i9	Glyma.04G141400.6	98.5915493	148	6	593	2424	1805	1	0	2255

TRINITY_DN16167_c0_g1_i9	Glyma.04G141400.1	98.5915493	148	6	593	2424	1805	1	0	2255

TRINITY_DN16167_c0_g1_i9	Glyma.04G141400.7	96.13402062	148	6	593	2424	1805	1	0	2255

TRINITY_DN16167_c0_g1_i10	Glyma.11G154700.1	100	141	10	692	2523	1817	1	0	2372

TRINITY_DN16167_c0_g1_i10	Glyma.11G154700.6	100	141	10	692	2523	1817	1	0	2372

TRINITY_DN16167_c0_g1_i10	Glyma.11G154700.7	100	141	10	692	2523	1817	1	0	2372

TRINITY_DN16167_c0_g1_i10	Glyma.11G154700.8	100	141	10	692	2523	1817	1	0	2372

TRINITY_DN16167_c0_g1_i9	Glyma.11G154700.1	96.59574468	141	10	593	2424	1817	1	0	2372

TRINITY_DN16167_c0_g1_i9	Glyma.11G154700.6	96.59574468	141	10	593	2424	1817	1	0	2372

TRINITY_DN16167_c0_g1_i9	Glyma.11G154700.7	96.59574468	141	10	593	2424	1817	1	0	2372

TRINITY_DN16167_c0_g1_i9	Glyma.11G154700.8	95.90909091	141	10	593	2424	1817	1	0	2372

TRINITY_DN15137_c0_g1_i1	Glyma.04G019700.1	100	129	1	1364	3204	1868	25	0	2671

TRINITY_DN15137_c0_g1_i13	Glyma.04G019700.1	100	129	1	1278	3118	1868	25	0	2671

TRINITY_DN15137_c0_g1_i6	Glyma.04G019700.1	97.43589744	129	1	1391	3231	1868	25	0	2671

TRINITY_DN36112_c0_g1_i1	Glyma.10G104900.1	100	118	9	3	1843	862	2690	0	2636

TRINITY_DN6058_c0_g1_i1	Glyma.02G151400.5	97.33840304	116	2	1204	3047	1829	1	0	2665

TRINITY_DN16245_c0_g3_i4	Glyma.17G123800.1	98.5	412	47	582	2398	3254	1441	0	765

TRINITY_DN16401_c1_g2_i8	Glyma.20G048800.4	98.71794872	342	51	70	1865	1889	68	0	1070

TRINITY_DN16401_c1_g2_i8	Glyma.20G048800.5	100	342	51	70	1865	1889	68	0	1070

TRINITY_DN16401_c1_g2_i7	Glyma.20G048800.4	95.22900763	341	49	306	2102	1890	68	0	1088

TRINITY_DN16401_c1_g2_i7	Glyma.20G048800.5	100	341	49	306	2102	1890	68	0	1088

TRINITY_DN16401_c1_g2_i7	Glyma.20G048800.14	100	341	49	306	2102	1890	68	0	1088

TRINITY_DN16401_c1_g2_i7	Glyma.20G048800.6	97.47191011	341	49	306	2102	1890	68	0	1088

TRINITY_DN16401_c1_g2_i7	Glyma.20G048800.7	100	341	49	306	2102	1890	68	0	1088

TRINITY_DN16401_c1_g2_i7	Glyma.20G048800.15	98.33333333	341	49	306	2102	1890	68	0	1088

TRINITY_DN16401_c1_g2_i7	Glyma.20G048800.16	96.94117647	341	49	306	2102	1890	68	0	1088

TRINITY_DN16401_c1_g2_i7	Glyma.20G048800.17	96.94189602	341	49	306	2102	1890	68	0	1088

TRINITY_DN16401_c1_g2_i7	Glyma.20G048800.12	96.66666667	341	49	306	2102	1890	68	0	1088

TRINITY_DN16401_c1_g2_i7	Glyma.20G048800.8	99.23076923	341	49	306	2102	1890	68	0	1088

TRINITY_DN16401_c1_g2_i7	Glyma.20G048800.9	100	341	49	306	2102	1890	68	0	1088

TRINITY_DN16401_c1_g2_i7	Glyma.20G048800.2	100	341	49	306	2102	1890	68	0	1088

TRINITY_DN16401_c1_g2_i7	Glyma.20G048800.13	100	341	49	306	2102	1890	68	0	1088

TRINITY_DN16401_c1_g2_i7	Glyma.20G048800.10	95.55555556	341	49	306	2102	1890	68	0	1088

TRINITY_DN16401_c1_g2_i7	Glyma.20G048800.11	100	341	49	306	2102	1890	68	0	1088

TRINITY_DN16401_c1_g2_i7	Glyma.20G048800.3	99.26470588	341	49	306	2102	1890	68	0	1088

TRINITY_DN16401_c1_g2_i7	Glyma.20G048800.1	97.25274725	341	49	306	2102	1890	68	0	1088

TRINITY_DN11875_c0_g2_i2	Glyma.11G251900.1	95.29411765	331	34	1283	3083	867	2691	0	1182

TRINITY_DN16401_c1_g2_i7	Glyma.13G076200.1	98.74213836	331	41	306	2102	1908	80	0	1182

TRINITY_DN15619_c0_g2_i1	Glyma.04G184000.2	97.41100324	260	10	1310	3138	1	1840	0	1853

TRINITY_DN15619_c0_g2_i3	Glyma.04G184000.2	100	260	10	1215	3043	1	1840	0	1853

TRINITY_DN1384_c0_g1_i1	Glyma.18G001400.1	96.47058824	258	15	758	2588	2191	361	0	1820

TRINITY_DN14801_c0_g2_i1	Glyma.14G153000.2	95.57522124	172	8	259	2097	1833	1	0	2361

TRINITY_DN8762_c0_g1_i1	Glyma.10G188000.1	98.71794872	169	15	497	2303	1843	1	0	2235

TRINITY_DN16081_c0_g1_i3	Glyma.09G056100.2	96	135	7	164	1994	1	1840	0	2558

TRINITY_DN16081_c0_g1_i3	Glyma.09G056100.1	98.7012987	135	7	164	1994	1	1840	0	2558

TRINITY_DN15271_c0_g1_i1	Glyma.05G070000.1	98.21428571	117	2	225	2066	1	1842	0	2743

TRINITY_DN15271_c0_g1_i1	Glyma.05G070000.2	97.36842105	117	2	225	2066	1	1842	0	2743

TRINITY_DN5747_c0_g2_i1	Glyma.13G235600.2	95.40229885	85	0	283	2125	677	2519	0	2933

TRINITY_DN6018_c0_g2_i2	Glyma.16G160800.1	100	345	18	303	2133	79	1906	0	1328

TRINITY_DN12379_c0_g1_i1	Glyma.04G121700.1	95.34883721	282	37	100	1918	1	1804	0	1445

TRINITY_DN16325_c0_g1_i2	Glyma.19G016600.4	99.50980392	233	9	1362	3199	2785	951	0	2039

TRINITY_DN16325_c0_g1_i2	Glyma.19G016600.2	99.50980392	233	9	1362	3199	2782	948	0	2039

TRINITY_DN16325_c0_g1_i2	Glyma.19G016600.3	100	233	9	1362	3199	2758	924	0	2039

TRINITY_DN16325_c0_g1_i3	Glyma.19G016600.4	100	230	9	1179	3016	2785	951	0	2056

TRINITY_DN16325_c0_g1_i3	Glyma.19G016600.2	100	230	9	1179	3016	2782	948	0	2056

TRINITY_DN16325_c0_g1_i3	Glyma.19G016600.3	100	230	9	1179	3016	2758	924	0	2056

TRINITY_DN9381_c0_g1_i1	Glyma.20G066900.1	100	198	6	562	2398	23	1857	0	2228

TRINITY_DN15648_c0_g2_i1	Glyma.20G234500.1	96.15384615	166	7	920	2757	2769	931	0	2436

TRINITY_DN15648_c0_g2_i2	Glyma.20G234500.1	96.1038961	166	7	515	2352	2769	931	0	2436

TRINITY_DN15648_c0_g2_i1	Glyma.20G234500.5	100	166	7	920	2757	2724	886	0	2436

TRINITY_DN15648_c0_g2_i2	Glyma.20G234500.5	96.1038961	166	7	515	2352	2724	886	0	2436

TRINITY_DN15648_c0_g2_i1	Glyma.20G234500.3	96.46990741	166	7	920	2757	2682	844	0	2436

TRINITY_DN15648_c0_g2_i2	Glyma.20G234500.3	97.06390328	166	7	515	2352	2682	844	0	2436

TRINITY_DN8729_c0_g1_i1	Glyma.03G192900.2	99.16666667	153	9	355	2174	2937	1100	0	2386

TRINITY_DN12469_c0_g1_i1	Glyma.02G236600.2	97.99196787	148	4	202	2043	1	1833	0	2523

TRINITY_DN12469_c0_g1_i2	Glyma.02G236600.2	98.5915493	148	4	220	2061	1	1833	0	2523

TRINITY_DN37204_c0_g1_i1	Glyma.04G036300.1	99.43181818	146	13	120	1931	34	1866	0	2340

TRINITY_DN15271_c0_g1_i1	Glyma.17G152700.4	97.36842105	132	0	225	2066	1	1842	0	2671

TRINITY_DN15271_c0_g1_i1	Glyma.17G152700.1	97.01492537	132	0	225	2066	1	1842	0	2671

TRINITY_DN15271_c0_g1_i1	Glyma.17G152700.3	97.90026247	132	0	225	2066	1	1842	0	2671

TRINITY_DN15271_c0_g1_i1	Glyma.17G152700.2	98.66666667	132	0	225	2066	1	1842	0	2671

TRINITY_DN14265_c0_g1_i1	Glyma.06G322200.3	95	131	2	438	2273	1842	1	0	2638

TRINITY_DN14265_c0_g1_i1	Glyma.06G322200.4	100	131	2	438	2273	1842	1	0	2638

TRINITY_DN16311_c0_g1_i3	Glyma.17G089200.1	96.55172414	131	2	855	2695	862	2702	0	2663

TRINITY_DN16311_c0_g1_i3	Glyma.17G089200.2	96.55172414	131	2	855	2695	859	2699	0	2663

TRINITY_DN14149_c0_g1_i2	Glyma.07G012500.2	100	96	1	446	2287	1	1839	0	2850

TRINITY_DN11190_c0_g1_i1	Glyma.06G181900.1	96.12590799	436	26	692	2515	1827	1	0	784

TRINITY_DN11190_c0_g1_i1	Glyma.06G181900.2	99.21259843	434	28	692	2515	1824	1	0	776

TRINITY_DN16254_c0_g1_i8	Glyma.08G222200.1	100	430	34	866	2686	2834	1015	0	752

TRINITY_DN7069_c0_g1_i1	Glyma.02G095900.8	100	244	45	601	2390	401	2199	0	1447

TRINITY_DN7069_c0_g1_i1	Glyma.02G095900.5	98.57142857	244	45	601	2390	401	2199	0	1447

TRINITY_DN7069_c0_g1_i1	Glyma.02G095900.2	98.57142857	244	45	601	2390	401	2199	0	1447

TRINITY_DN7069_c0_g1_i1	Glyma.02G095900.1	98.57142857	244	45	601	2390	401	2199	0	1447

TRINITY_DN7069_c0_g1_i1	Glyma.02G095900.6	98.57142857	244	45	601	2390	401	2199	0	1447

TRINITY_DN7069_c0_g1_i1	Glyma.02G095900.3	98.57142857	244	45	601	2390	401	2199	0	1447

TRINITY_DN7069_c0_g1_i1	Glyma.02G095900.7	98.61111111	244	45	601	2390	401	2199	0	1447

TRINITY_DN7069_c0_g1_i1	Glyma.02G095900.4	97.72727273	244	45	601	2390	401	2199	0	1447

TRINITY_DN7069_c0_g1_i1	Glyma.02G095900.9	97.72727273	244	45	601	2390	401	2199	0	1447

TRINITY_DN16086_c0_g3_i2	Glyma.04G075100.2	97.05882353	194	4	1	1839	922	2760	0	2300

TRINITY_DN16325_c1_g1_i1	Glyma.19G016400.1	97.89915966	173	21	1	1831	3290	1489	0	2124

TRINITY_DN1636_c0_g1_i1	Glyma.20G198400.1	96.62921348	170	13	940	2758	1828	1	0	2231

TRINITY_DN1636_c0_g2_i1	Glyma.20G198400.1	96.36363636	170	13	940	2758	1828	1	0	2231

TRINITY_DN10262_c0_g2_i1	Glyma.08G295700.1	100	166	17	193	2005	1860	36	0	2196

TRINITY_DN15501_c0_g2_i6	Glyma.08G214300.2	100	152	5	1	1839	1838	1	0	2525

TRINITY_DN9794_c0_g2_i1	Glyma.17G235000.4	95.62937063	151	3	550	2388	1	1839	0	2538

TRINITY_DN16167_c0_g1_i10	Glyma.04G141400.4	100	148	5	692	2523	1802	1	0	2270

TRINITY_DN16167_c0_g1_i10	Glyma.04G141400.5	100	148	5	692	2523	1802	1	0	2270

TRINITY_DN16167_c0_g1_i9	Glyma.04G141400.4	98.73417722	148	5	593	2424	1802	1	0	2270

TRINITY_DN16167_c0_g1_i9	Glyma.04G141400.5	95.16129032	148	5	593	2424	1802	1	0	2270

TRINITY_DN16167_c0_g1_i10	Glyma.11G154700.2	100	141	9	692	2523	1814	1	0	2386

TRINITY_DN16167_c0_g1_i10	Glyma.11G154700.4	100	141	9	692	2523	1814	1	0	2386

TRINITY_DN16167_c0_g1_i10	Glyma.11G154700.3	100	141	9	692	2523	1814	1	0	2386

TRINITY_DN16167_c0_g1_i10	Glyma.11G154700.5	100	141	9	692	2523	1814	1	0	2386

TRINITY_DN16167_c0_g1_i9	Glyma.11G154700.2	95.25547445	141	9	593	2424	1814	1	0	2386

TRINITY_DN16167_c0_g1_i9	Glyma.11G154700.4	95.25547445	141	9	593	2424	1814	1	0	2386

TRINITY_DN16167_c0_g1_i9	Glyma.11G154700.3	100	141	9	593	2424	1814	1	0	2386

TRINITY_DN16167_c0_g1_i9	Glyma.11G154700.5	100	141	9	593	2424	1814	1	0	2386

TRINITY_DN8988_c0_g1_i2	Glyma.09G049700.2	96.6442953	130	3	83	1920	1875	38	0	2641

TRINITY_DN8988_c0_g1_i2	Glyma.09G049700.1	96.65427509	130	3	83	1920	1875	38	0	2641

TRINITY_DN8988_c0_g1_i3	Glyma.09G049700.2	96.64634146	130	3	496	2333	1875	38	0	2641

TRINITY_DN8988_c0_g1_i3	Glyma.09G049700.1	96.66666667	130	3	496	2333	1875	38	0	2641

TRINITY_DN5787_c0_g1_i1	Glyma.07G244300.1	95.5801105	126	3	328	2159	1905	65	0	2643

TRINITY_DN13783_c0_g1_i3	Glyma.12G241900.1	97.72727273	117	1	1450	3287	1841	1	0	2732

TRINITY_DN13783_c0_g1_i3	Glyma.09G002400.1	96.12590799	115	1	1450	3287	1841	1	0	2743

TRINITY_DN13783_c0_g1_i3	Glyma.09G002400.2	100	115	1	1450	3287	1841	1	0	2743

TRINITY_DN16311_c0_g1_i4	Glyma.17G089200.1	96.55172414	100	0	855	2695	862	2702	0	2846

TRINITY_DN16311_c0_g1_i4	Glyma.17G089200.2	96.55172414	100	0	855	2695	859	2699	0	2846

TRINITY_DN16138_c1_g1_i6	Glyma.15G049300.1	97.70114943	289	22	311	2130	1	1829	0	1596

TRINITY_DN15515_c0_g1_i1	Glyma.09G109000.1	100	230	14	292	2123	43	1862	0	1943

TRINITY_DN15515_c0_g1_i2	Glyma.09G109000.1	95.12195122	230	14	319	2150	43	1862	0	1943

TRINITY_DN13322_c0_g1_i1	Glyma.19G129900.1	99.02439024	224	11	277	2106	1833	1	0	2047

TRINITY_DN13448_c0_g1_i7	Glyma.05G144400.4	97.8021978	206	3	1	1839	27	1859	0	2206

TRINITY_DN13448_c0_g1_i7	Glyma.05G144400.3	97.20670391	206	3	1	1839	27	1859	0	2206

TRINITY_DN13448_c0_g1_i7	Glyma.05G144400.1	97.77777778	206	3	1	1839	27	1859	0	2206

TRINITY_DN35755_c0_g1_i1	Glyma.06G128900.2	98.1595092	179	8	2	1837	47	1876	0	2316

TRINITY_DN35755_c0_g1_i1	Glyma.06G128900.1	96.5625	179	8	2	1837	47	1876	0	2316

TRINITY_DN16081_c0_g1_i4	Glyma.09G056100.2	96.03399433	162	7	164	1990	1	1836	0	2390

TRINITY_DN16081_c0_g1_i4	Glyma.09G056100.1	96.03399433	162	7	164	1990	1	1836	0	2390

TRINITY_DN16081_c0_g1_i4	Glyma.15G162300.1	96.03174603	144	6	164	1998	1	1835	0	2536

TRINITY_DN16081_c0_g1_i4	Glyma.15G162300.3	96.03340292	144	6	164	1998	1	1835	0	2536

TRINITY_DN14745_c0_g1_i2	Glyma.17G019100.2	100	133	15	371	2203	1821	1	0	2494

TRINITY_DN6670_c0_g1_i1	Glyma.01G038100.1	97.86585366	124	0	257	2096	3489	1650	0	2712

TRINITY_DN7491_c0_g1_i1	Glyma.07G089600.3	98.18181818	94	0	466	2305	1840	1	0	2878

TRINITY_DN7491_c0_g1_i1	Glyma.07G089600.2	96.68508287	94	0	466	2305	1840	1	0	2878

TRINITY_DN14164_c0_g1_i4	Glyma.20G179900.3	96.54218534	92	5	111	1946	1	1827	0	2778

TRINITY_DN6323_c0_g2_i2	Glyma.13G111800.1	100	312	25	66	1889	1	1821	0	1454

TRINITY_DN16149_c1_g1_i1	Glyma.17G191000.2	97.86324786	249	37	407	2193	1796	1	0	1402

TRINITY_DN14726_c0_g3_i1	Glyma.17G254100.3	95.08196721	235	9	478	2312	2078	247	0	2023

TRINITY_DN15007_c0_g1_i10	Glyma.08G133200.1	95.55288462	231	2	985	2822	1838	1	0	2104

TRINITY_DN15007_c0_g1_i7	Glyma.08G133200.1	100	231	2	985	2822	1838	1	0	2104

TRINITY_DN15007_c0_g1_i10	Glyma.08G133200.2	96.91119691	228	5	985	2822	1829	1	0	2061

TRINITY_DN15007_c0_g1_i7	Glyma.08G133200.2	100	228	5	985	2822	1829	1	0	2061

TRINITY_DN11254_c0_g1_i1	Glyma.07G007000.2	98.85057471	215	5	895	2729	1841	7	0	2154

TRINITY_DN12569_c0_g1_i5	Glyma.15G061700.1	95.97585513	199	9	69	1897	1	1835	0	2204

TRINITY_DN10825_c0_g1_i1	Glyma.01G154700.1	100	197	23	797	2618	1942	130	0	2026

TRINITY_DN14033_c0_g2_i1	Glyma.20G006500.1	97.20394737	188	9	81	1914	2676	4506	0	2270

TRINITY_DN14239_c0_g1_i1	Glyma.13G186800.1	98.52941176	175	18	513	2312	2022	196	0	2097

TRINITY_DN14239_c0_g1_i1	Glyma.13G186800.2	98.52941176	175	18	513	2312	2022	196	0	2097

TRINITY_DN14239_c0_g1_i1	Glyma.13G186800.4	98.52941176	175	18	513	2312	2022	196	0	2097

TRINITY_DN14239_c0_g1_i1	Glyma.15G224400.1	100	172	17	513	2312	2022	196	0	2113

TRINITY_DN14239_c0_g1_i1	Glyma.15G224400.13	95.57823129	172	17	513	2312	2022	196	0	2113

TRINITY_DN14239_c0_g1_i1	Glyma.15G224400.9	100	172	17	513	2312	2022	196	0	2113

TRINITY_DN14239_c0_g1_i1	Glyma.15G224400.8	100	172	17	513	2312	2022	196	0	2113

TRINITY_DN14239_c0_g1_i1	Glyma.15G224400.10	100	172	17	513	2312	2022	196	0	2113

TRINITY_DN14239_c0_g1_i1	Glyma.15G224400.11	100	172	17	513	2312	2022	196	0	2113

TRINITY_DN14239_c0_g1_i1	Glyma.15G224400.12	100	172	17	513	2312	2022	196	0	2113

TRINITY_DN13054_c0_g1_i1	Glyma.08G058600.1	97.14285714	140	4	589	2415	1875	40	0	2523

TRINITY_DN14745_c0_g1_i2	Glyma.07G255200.1	100	130	14	371	2203	1821	1	0	2521

TRINITY_DN11599_c0_g1_i3	Glyma.20G215200.2	96.68246445	128	8	887	2719	1818	1	0	2512

TRINITY_DN11599_c0_g1_i5	Glyma.20G215200.2	98.86792453	128	8	887	2719	1818	1	0	2512

TRINITY_DN11599_c0_g1_i6	Glyma.20G215200.2	100	128	8	887	2719	1818	1	0	2512

TRINITY_DN15332_c0_g2_i1	Glyma.03G246500.2	96.77419355	120	3	675	2509	1833	1	0	2667

TRINITY_DN15332_c0_g2_i2	Glyma.03G246500.2	96.42857143	120	3	773	2607	1833	1	0	2667

TRINITY_DN15332_c0_g2_i3	Glyma.03G246500.2	98.41269841	120	3	536	2370	1833	1	0	2667

TRINITY_DN16081_c0_g1_i3	Glyma.15G162300.1	100	113	4	164	1998	1	1835	0	2719

TRINITY_DN16081_c0_g1_i3	Glyma.15G162300.3	98.30508475	113	4	164	1998	1	1835	0	2719

TRINITY_DN15937_c0_g1_i4	Glyma.12G155700.1	100	222	34	544	2346	3	1811	0	1751

TRINITY_DN5259_c0_g2_i1	Glyma.08G344100.1	97.83549784	215	6	98	1934	1	1833	0	2165

TRINITY_DN14896_c0_g1_i1	Glyma.01G225900.1	100	201	10	189	2021	1833	1	0	2217

TRINITY_DN14033_c0_g2_i1	Glyma.20G006500.2	98.48484848	190	8	81	1914	2676	4497	0	2213

TRINITY_DN16386_c0_g1_i3	Glyma.16G198300.1	97.7443609	171	10	23	1853	819	2640	0	2298

TRINITY_DN16386_c0_g1_i3	Glyma.16G198300.2	98.27586207	171	10	23	1853	819	2640	0	2298

TRINITY_DN18058_c0_g1_i1	Glyma.03G252300.1	95.07772021	148	0	1	1838	2395	558	0	2575

TRINITY_DN18058_c0_g1_i1	Glyma.19G249900.1	100	136	0	1	1838	2395	558	0	2641

TRINITY_DN5259_c0_g2_i1	Glyma.16G083100.1	95.28301887	105	3	98	1934	1	1836	0	2793

TRINITY_DN5259_c0_g2_i1	Glyma.16G083100.2	97.36842105	105	3	98	1934	1	1836	0	2793

TRINITY_DN14688_c0_g2_i1	Glyma.13G325000.2	99.609375	104	2	241	2072	1842	5	0	2780

TRINITY_DN16138_c1_g1_i6	Glyma.08G183400.7	96.61458333	306	14	311	2130	1	1829	0	1535

TRINITY_DN16138_c1_g1_i6	Glyma.08G183400.8	95.23809524	306	14	311	2130	1	1829	0	1535

TRINITY_DN16138_c1_g1_i6	Glyma.08G183400.9	96.63299663	306	14	311	2130	1	1829	0	1535

TRINITY_DN6323_c0_g2_i2	Glyma.17G047900.2	98.61751152	285	27	66	1879	1	1820	0	1555

TRINITY_DN6323_c0_g2_i1	Glyma.17G047900.2	98.13664596	284	28	66	1876	1	1820	0	1541

TRINITY_DN15521_c0_g1_i3	Glyma.12G231800.1	100	276	12	1361	3190	1194	3023	0	1773

TRINITY_DN15521_c0_g1_i3	Glyma.12G233000.1	97.13261649	274	12	1361	3190	1191	3020	0	1784

TRINITY_DN15521_c0_g1_i3	Glyma.12G233000.2	95.84245077	274	12	1361	3190	1119	2948	0	1784

TRINITY_DN15888_c0_g3_i5	Glyma.14G048300.1	100	227	25	833	2655	1477	3287	0	1877

TRINITY_DN11959_c0_g1_i1	Glyma.18G228000.1	100	173	12	135	1958	1	1821	0	2246

TRINITY_DN11959_c0_g1_i1	Glyma.18G228000.2	100	173	15	135	1958	1	1818	0	2228

TRINITY_DN8825_c0_g1_i1	Glyma.02G000600.1	98.7012987	165	19	100	1908	1	1830	0	2252

TRINITY_DN15664_c0_g2_i3	Glyma.18G266400.1	96.71772429	159	2	1	1831	1837	1	0	2473

TRINITY_DN10964_c0_g1_i1	Glyma.09G001900.1	96.11872146	152	2	98	1933	1	1836	0	2538

TRINITY_DN14896_c0_g1_i2	Glyma.01G225900.1	100	136	8	189	2021	1833	1	0	2588

TRINITY_DN16405_c0_g1_i1	Glyma.04G015300.1	95.95959596	127	1	155	1991	27	1860	0	2669

TRINITY_DN12479_c0_g1_i7	Glyma.17G085300.1	98.8372093	122	18	243	2075	1809	1	0	2510

TRINITY_DN16012_c0_g1_i7	Glyma.07G263300.1	100	121	9	166	1995	469	2301	0	2651

TRINITY_DN5259_c0_g2_i1	Glyma.03G090000.1	97.5308642	102	1	98	1934	1	1836	0	2820

TRINITY_DN5259_c0_g2_i1	Glyma.03G090000.2	97.77777778	102	1	98	1934	1	1836	0	2820

TRINITY_DN14164_c0_g1_i4	Glyma.10G210700.1	98.74715262	95	8	114	1946	7	1833	0	2776

TRINITY_DN10347_c0_g2_i1	Glyma.05G179000.2	95.56313993	94	0	420	2256	1	1837	0	2872

TRINITY_DN16081_c0_g1_i2	Glyma.13G097600.1	100	77	10	164	1985	1	1836	0	2863

TRINITY_DN6323_c0_g2_i1	Glyma.13G111800.1	96.15384615	313	23	66	1886	1	1821	0	1463

TRINITY_DN15510_c0_g1_i1	Glyma.09G168400.3	96.32768362	278	23	270	2073	7	1831	0	1572

TRINITY_DN15510_c0_g1_i3	Glyma.09G168400.3	96.55172414	278	23	296	2099	7	1831	0	1572

TRINITY_DN15510_c0_g1_i4	Glyma.09G168400.3	98.8700565	278	23	296	2099	7	1831	0	1572

TRINITY_DN15510_c0_g1_i6	Glyma.09G168400.3	96.9924812	278	23	270	2073	7	1831	0	1572

TRINITY_DN16138_c1_g1_i13	Glyma.15G049300.1	95.64428312	243	19	566	2377	1	1829	0	1844

TRINITY_DN14896_c0_g1_i1	Glyma.11G017000.1	100	204	6	189	2021	1833	1	0	2222

TRINITY_DN11281_c0_g1_i1	Glyma.09G252300.1	100	163	5	481	2312	1839	8	0	2436

TRINITY_DN10964_c0_g1_i1	Glyma.12G241600.1	100	157	0	98	1933	1	1836	0	2521

TRINITY_DN14326_c0_g4_i1	Glyma.01G168900.1	96	144	9	453	2282	107	1918	0	2399

TRINITY_DN6345_c0_g1_i1	Glyma.11G073400.1	98.5915493	141	0	102	1937	1	1836	0	2610

TRINITY_DN12479_c0_g1_i7	Glyma.05G041000.2	98.8372093	135	8	243	2075	1818	1	0	2507

TRINITY_DN14326_c0_g4_i1	Glyma.11G074600.1	98.66666667	135	10	453	2282	131	1942	0	2449

TRINITY_DN15464_c0_g1_i3	Glyma.01G070300.1	96.45390071	389	43	230	2040	47	1822	0	697

TRINITY_DN15464_c0_g1_i3	Glyma.01G070300.2	100	389	43	230	2040	47	1822	0	697

TRINITY_DN15464_c0_g1_i3	Glyma.01G070300.3	100	389	43	230	2040	47	1822	0	697

TRINITY_DN14809_c0_g1_i4	Glyma.01G053300.1	99.52380952	308	11	435	2261	1827	1	0	1580

TRINITY_DN14809_c0_g1_i5	Glyma.01G053300.1	96.96969697	294	11	435	2261	1827	1	0	1657

TRINITY_DN16138_c1_g1_i13	Glyma.08G183400.7	99.18032787	258	20	566	2377	1	1829	0	1772

TRINITY_DN16138_c1_g1_i13	Glyma.08G183400.8	99.18032787	258	20	566	2377	1	1829	0	1772

TRINITY_DN16138_c1_g1_i13	Glyma.08G183400.9	99.18032787	258	20	566	2377	1	1829	0	1772

TRINITY_DN15772_c0_g1_i2	Glyma.19G039100.1	100	244	4	182	2014	43	1875	0	2012

TRINITY_DN15772_c0_g1_i9	Glyma.19G039100.1	98.73417722	244	4	197	2029	43	1875	0	2012

TRINITY_DN8982_c0_g1_i1	Glyma.07G018800.1	100	187	4	380	2212	1821	1	0	2250

TRINITY_DN15160_c0_g1_i1	Glyma.02G173300.1	100	174	5	546	2365	1864	30	0	2327

TRINITY_DN14830_c0_g3_i1	Glyma.05G004200.1	100	173	7	286	2101	50	1881	0	2289

TRINITY_DN11959_c0_g1_i1	Glyma.09G264000.1	98.63013699	171	15	135	1958	1	1824	0	2300

TRINITY_DN15648_c0_g2_i3	Glyma.20G234500.6	98.18181818	165	6	893	2724	2717	886	0	2436

TRINITY_DN15648_c0_g2_i4	Glyma.20G234500.6	96.36363636	165	6	488	2319	2717	886	0	2436

TRINITY_DN15648_c0_g2_i3	Glyma.20G234500.4	100	165	6	893	2724	2675	844	0	2436

TRINITY_DN15648_c0_g2_i4	Glyma.20G234500.4	98.99280576	165	6	488	2319	2675	844	0	2436

TRINITY_DN8982_c0_g2_i1	Glyma.07G018800.1	100	161	4	380	2212	1821	1	0	2394

TRINITY_DN15828_c0_g1_i4	Glyma.09G015400.1	95.52238806	150	11	572	2386	1827	1	0	2377

TRINITY_DN15828_c0_g1_i4	Glyma.09G015400.2	96.95121951	150	11	572	2386	1827	1	0	2377

TRINITY_DN15828_c0_g1_i4	Glyma.09G015400.3	98.36065574	150	11	572	2386	1827	1	0	2377

TRINITY_DN15828_c0_g1_i5	Glyma.09G015400.1	95.37037037	150	11	572	2386	1827	1	0	2377

TRINITY_DN15828_c0_g1_i5	Glyma.09G015400.2	95.37037037	150	11	572	2386	1827	1	0	2377

TRINITY_DN15828_c0_g1_i5	Glyma.09G015400.3	100	150	11	572	2386	1827	1	0	2377

TRINITY_DN14896_c0_g1_i2	Glyma.11G017000.1	100	148	4	189	2021	1833	1	0	2543

TRINITY_DN13902_c0_g1_i4	Glyma.11G219700.1	100	145	3	115	1943	341	2175	0	2547

TRINITY_DN6345_c0_g1_i1	Glyma.01G169900.1	98.5915493	140	0	102	1936	1	1835	0	2614

TRINITY_DN16122_c0_g1_i2	Glyma.14G033400.1	97.73462783	140	27	620	2420	1814	1	0	2257

TRINITY_DN36355_c0_g1_i1	Glyma.13G193200.1	100	134	11	80	1902	917	2742	0	2510

TRINITY_DN36355_c0_g1_i1	Glyma.13G193200.3	100	134	11	80	1902	884	2709	0	2510

TRINITY_DN36355_c0_g1_i1	Glyma.13G193200.2	95.52845528	134	11	80	1902	884	2709	0	2510

TRINITY_DN9702_c0_g2_i1	Glyma.06G071300.5	95	119	3	482	2313	2	1833	0	2691

TRINITY_DN9702_c0_g2_i1	Glyma.06G071300.2	95	119	3	482	2313	2	1833	0	2691

TRINITY_DN9464_c0_g2_i1	Glyma.01G036200.1	100	107	5	177	2009	1	1821	0	2693

TRINITY_DN9464_c0_g2_i1	Glyma.01G036200.3	100	106	6	177	2009	1	1818	0	2678

TRINITY_DN15464_c0_g1_i3	Glyma.02G127400.1	98.83040936	385	45	230	2040	47	1822	0	730

TRINITY_DN15464_c0_g1_i3	Glyma.02G127400.2	96.09375	385	45	230	2040	47	1822	0	730

TRINITY_DN15751_c0_g1_i8	Glyma.05G045700.1	100	323	33	1420	3235	420	2220	0	1267

TRINITY_DN2865_c0_g1_i1	Glyma.11G094600.1	98.82352941	204	13	13	1824	31	1860	0	2089

TRINITY_DN9339_c0_g1_i1	Glyma.16G083800.1	100	193	16	127	1957	51	1866	0	2182

TRINITY_DN9339_c0_g1_i1	Glyma.16G083800.2	98.7012987	190	18	127	1957	51	1854	0	2121

TRINITY_DN15160_c0_g1_i1	Glyma.02G001200.1	100	175	5	546	2364	1849	16	0	2320

TRINITY_DN14896_c0_g1_i2	Glyma.08G023300.1	100	170	2	189	2021	1833	1	0	2433

TRINITY_DN14896_c0_g1_i2	Glyma.05G217300.1	100	163	2	189	2021	1833	1	0	2471

TRINITY_DN9028_c0_g1_i1	Glyma.07G186700.1	96.69811321	156	18	24	1846	554	2364	0	2303

TRINITY_DN4072_c0_g1_i1	Glyma.07G233200.1	97.56097561	149	5	576	2399	253	2085	0	2490

TRINITY_DN10079_c0_g2_i1	Glyma.07G264300.1	100	145	5	1	1822	480	2310	0	2486

TRINITY_DN13484_c0_g3_i1	Glyma.04G092400.2	99.12280702	142	5	161	1979	1	1834	0	2503

TRINITY_DN15589_c0_g1_i1	Glyma.13G072500.1	97.75784753	129	0	708	2541	1837	4	0	2673

TRINITY_DN14801_c0_g1_i2	Glyma.14G153000.2	95.9553696	338	10	395	2215	1827	1	0	1397

TRINITY_DN16444_c0_g1_i1	Glyma.17G111500.1	98.73417722	305	19	2	1817	1024	2848	0	1533

TRINITY_DN15937_c0_g1_i4	Glyma.12G155700.2	100	221	34	544	2341	3	1806	0	1748

TRINITY_DN10795_c0_g2_i1	Glyma.02G148900.1	100	212	22	144	1950	290	2099	0	1893

TRINITY_DN16012_c0_g1_i10	Glyma.07G263300.1	100	191	3	166	1993	469	2301	0	2294

TRINITY_DN9062_c0_g1_i1	Glyma.06G129400.1	97.54901961	164	14	125	1948	81	1898	0	2322

TRINITY_DN15626_c0_g3_i3	Glyma.08G161000.1	95.72649573	136	7	330	2150	1	1833	0	2555

TRINITY_DN14280_c0_g2_i2	Glyma.05G090400.1	97.83549784	129	7	269	2088	1829	1	0	2560

TRINITY_DN15772_c0_g1_i5	Glyma.18G278200.2	96.25	126	0	161	1993	40	1872	0	2687

TRINITY_DN15772_c0_g1_i5	Glyma.18G278200.1	98.11676083	126	0	161	1993	40	1872	0	2687

TRINITY_DN15772_c0_g1_i5	Glyma.18G278200.3	95.78059072	126	0	161	1993	40	1872	0	2687

TRINITY_DN1922_c0_g1_i1	Glyma.20G007400.1	100	122	0	161	1993	1	1833	0	2710

TRINITY_DN14280_c0_g2_i2	Glyma.17G176400.1	97.83783784	121	8	269	2088	1826	1	0	2586

TRINITY_DN15321_c1_g3_i1	Glyma.08G134600.4	96.41791045	115	0	200	2032	1	1833	0	2748

TRINITY_DN15321_c1_g3_i1	Glyma.08G134600.3	100	115	0	200	2032	1	1833	0	2748

TRINITY_DN15321_c1_g3_i1	Glyma.08G134600.2	95.77464789	115	0	200	2032	1	1833	0	2748

TRINITY_DN15321_c1_g3_i1	Glyma.08G134600.1	100	115	0	200	2032	1	1833	0	2748

TRINITY_DN15321_c1_g3_i1	Glyma.08G134600.5	100	115	0	200	2032	1	1833	0	2748

TRINITY_DN2628_c0_g1_i1	Glyma.06G100600.2	100	115	4	216	2045	6	1831	0	2684

TRINITY_DN9464_c0_g2_i1	Glyma.02G029800.1	95.65217391	112	1	177	2009	1	1821	0	2687

TRINITY_DN15321_c1_g3_i1	Glyma.05G177500.1	98.73417722	106	0	200	2032	1	1833	0	2798

TRINITY_DN15321_c1_g3_i1	Glyma.05G177500.5	99.65870307	106	0	200	2032	1	1833	0	2798

TRINITY_DN15321_c1_g3_i1	Glyma.05G177500.2	96.58634538	106	0	200	2032	1	1833	0	2798

TRINITY_DN15321_c1_g3_i1	Glyma.05G177500.3	96.44268775	106	0	200	2032	1	1833	0	2798

TRINITY_DN15321_c1_g3_i1	Glyma.05G177500.4	95.03311258	106	0	200	2032	1	1833	0	2798

TRINITY_DN12169_c0_g1_i1	Glyma.08G051900.1	100	103	0	591	2423	1833	1	0	2815

TRINITY_DN4986_c0_g1_i1	Glyma.08G050200.1	97.5308642	97	2	1	1824	1702	3534	0	2789

TRINITY_DN15626_c0_g3_i2	Glyma.08G161000.1	96.2962963	96	7	425	2245	1	1833	0	2776

TRINITY_DN14896_c0_g1_i1	Glyma.08G023300.1	100	94	0	189	2021	1833	1	0	2865

TRINITY_DN10284_c0_g1_i1	Glyma.03G254000.3	97.61904762	90	1	184	2013	1833	1	0	2867

TRINITY_DN10284_c0_g1_i1	Glyma.03G254000.1	97.61904762	90	1	184	2013	1833	1	0	2867

TRINITY_DN10284_c0_g1_i1	Glyma.03G254000.4	97.61904762	90	1	184	2013	1833	1	0	2867

TRINITY_DN10284_c0_g1_i1	Glyma.03G254000.2	97.62470309	90	1	184	2013	1833	1	0	2867

TRINITY_DN10324_c0_g2_i2	Glyma.07G273900.2	98.85057471	88	1	761	2593	1	1818	0	2800

TRINITY_DN10324_c0_g2_i2	Glyma.07G273900.1	95.77464789	88	1	761	2593	1	1818	0	2800

TRINITY_DN14896_c0_g1_i1	Glyma.05G217300.1	99.18032787	85	0	189	2021	1833	1	0	2915

TRINITY_DN10324_c0_g2_i2	Glyma.17G000500.1	98.85057471	84	1	761	2593	1	1818	0	2822

TRINITY_DN10324_c0_g2_i2	Glyma.17G000500.2	98.85057471	84	1	761	2593	1	1818	0	2822

TRINITY_DN4986_c0_g1_i1	Glyma.05G242800.1	97.54901961	83	5	1	1824	1711	3543	0	2867

TRINITY_DN4986_c0_g1_i1	Glyma.05G242800.2	97.5308642	83	5	1	1824	679	2511	0	2867

TRINITY_DN15235_c0_g1_i2	Glyma.16G172500.1	98.8372093	319	10	838	2666	1211	3035	0	1552

TRINITY_DN15235_c0_g1_i4	Glyma.16G172500.1	99.09090909	319	10	838	2666	1211	3035	0	1552

TRINITY_DN15333_c1_g1_i1	Glyma.14G150900.1	96.7611336	232	7	500	2322	1	1826	0	2001

TRINITY_DN15333_c1_g1_i1	Glyma.14G150900.2	96.77419355	232	7	500	2322	1	1826	0	2001

TRINITY_DN5817_c0_g1_i1	Glyma.14G084400.1	100	210	27	370	2170	1812	27	0	1722

TRINITY_DN9350_c0_g1_i1	Glyma.10G019200.9	96.40883978	195	7	154	1983	1	1821	0	2218

TRINITY_DN18757_c0_g1_i1	Glyma.04G018000.1	98.57142857	187	13	789	2610	1934	122	0	2159

TRINITY_DN13181_c0_g1_i2	Glyma.19G226000.1	97.77777778	182	10	69	1891	86	1911	0	2278

TRINITY_DN16268_c1_g2_i5	Glyma.17G161600.2	96.66666667	166	8	559	2372	1845	17	0	2327

TRINITY_DN16268_c1_g2_i3	Glyma.17G161600.2	98.86363636	153	10	559	2366	1845	17	0	2361

TRINITY_DN13902_c0_g1_i4	Glyma.18G037700.1	95.68733154	141	1	115	1943	341	2172	0	2582

TRINITY_DN14726_c0_g2_i1	Glyma.17G254100.3	100	137	1	644	2472	2078	247	0	2604

TRINITY_DN1744_c0_g1_i1	Glyma.15G032800.2	98.11827957	130	2	1	1831	273	2103	0	2651

TRINITY_DN2139_c0_g1_i1	Glyma.05G149200.1	98.97959184	129	4	392	2221	1	1830	0	2643

TRINITY_DN16405_c0_g1_i1	Glyma.06G015400.1	100	126	0	160	1991	11	1842	0	2686

TRINITY_DN9702_c0_g2_i1	Glyma.04G069600.4	95	124	0	482	2313	2	1833	0	2697

TRINITY_DN13902_c0_g1_i2	Glyma.02G248700.2	95.65217391	115	1	115	1946	341	2169	0	2726

TRINITY_DN13902_c0_g1_i2	Glyma.02G248700.1	95.65217391	115	1	115	1946	341	2169	0	2726

TRINITY_DN2628_c0_g1_i1	Glyma.06G100600.3	100	115	4	216	2044	6	1830	0	2682

TRINITY_DN14650_c0_g2_i2	Glyma.06G031900.4	100	104	4	424	2253	1926	97	0	2782

TRINITY_DN7069_c0_g1_i1	Glyma.18G285600.1	96.875	230	50	611	2390	411	2199	0	1506

TRINITY_DN15648_c0_g2_i3	Glyma.10G153800.5	96.85658153	173	6	899	2724	2672	844	0	2377

TRINITY_DN15648_c0_g2_i3	Glyma.10G153800.1	98.83449883	173	6	899	2724	2672	844	0	2377

TRINITY_DN15648_c0_g2_i3	Glyma.10G153800.4	95.37037037	173	6	899	2724	2672	844	0	2377

TRINITY_DN15648_c0_g2_i3	Glyma.10G153800.3	95.38461538	173	6	899	2724	2672	844	0	2377

TRINITY_DN15648_c0_g2_i3	Glyma.10G153800.2	95.17241379	173	6	899	2724	2672	844	0	2377

TRINITY_DN15648_c0_g2_i3	Glyma.10G153800.11	100	173	6	899	2724	2672	844	0	2377

TRINITY_DN15648_c0_g2_i4	Glyma.10G153800.5	98.87640449	173	6	494	2319	2672	844	0	2377

TRINITY_DN15648_c0_g2_i4	Glyma.10G153800.1	100	173	6	494	2319	2672	844	0	2377

TRINITY_DN15648_c0_g2_i4	Glyma.10G153800.4	98.24561404	173	6	494	2319	2672	844	0	2377

TRINITY_DN15648_c0_g2_i4	Glyma.10G153800.3	98.24561404	173	6	494	2319	2672	844	0	2377

TRINITY_DN15648_c0_g2_i4	Glyma.10G153800.2	96.07843137	173	6	494	2319	2672	844	0	2377

TRINITY_DN15648_c0_g2_i4	Glyma.10G153800.11	96.875	173	6	494	2319	2672	844	0	2377

TRINITY_DN15648_c0_g2_i3	Glyma.10G153800.17	97.54098361	173	6	899	2724	2669	841	0	2377

TRINITY_DN15648_c0_g2_i3	Glyma.10G153800.16	96.62921348	173	6	899	2724	2669	841	0	2377

TRINITY_DN15648_c0_g2_i3	Glyma.10G153800.15	97.5308642	173	6	899	2724	2669	841	0	2377

TRINITY_DN15648_c0_g2_i3	Glyma.10G153800.14	98.26086957	173	6	899	2724	2669	841	0	2377

TRINITY_DN15648_c0_g2_i3	Glyma.10G153800.13	100	173	6	899	2724	2669	841	0	2377

TRINITY_DN15648_c0_g2_i4	Glyma.10G153800.17	97.43589744	173	6	494	2319	2669	841	0	2377

TRINITY_DN15648_c0_g2_i4	Glyma.10G153800.16	96.77419355	173	6	494	2319	2669	841	0	2377

TRINITY_DN15648_c0_g2_i4	Glyma.10G153800.15	97.18309859	173	6	494	2319	2669	841	0	2377

TRINITY_DN15648_c0_g2_i4	Glyma.10G153800.14	99.81238274	173	6	494	2319	2669	841	0	2377

TRINITY_DN15648_c0_g2_i4	Glyma.10G153800.13	99.13793103	173	6	494	2319	2669	841	0	2377

TRINITY_DN16268_c1_g2_i5	Glyma.17G161600.3	96.11650485	166	8	559	2371	1842	15	0	2326

TRINITY_DN16268_c1_g2_i5	Glyma.17G161600.4	96.94117647	166	8	559	2371	1830	3	0	2326

TRINITY_DN12888_c0_g1_i2	Glyma.18G196000.1	97.10144928	161	5	374	2193	1	1829	0	2405

TRINITY_DN12888_c0_g1_i2	Glyma.18G195900.1	97.11538462	158	6	374	2193	1	1829	0	2422

TRINITY_DN9028_c0_g1_i1	Glyma.07G186700.2	96.69117647	156	17	24	1846	554	2361	0	2316

TRINITY_DN16268_c1_g2_i3	Glyma.17G161600.3	98.86363636	153	10	559	2365	1842	15	0	2359

TRINITY_DN16268_c1_g2_i3	Glyma.17G161600.4	98.86363636	153	10	559	2365	1830	3	0	2359

TRINITY_DN9028_c0_g1_i1	Glyma.08G062500.1	96.69051878	153	18	24	1846	566	2373	0	2333

TRINITY_DN10079_c0_g2_i1	Glyma.17G009700.1	98.52941176	145	3	1	1822	504	2331	0	2501

TRINITY_DN177_c0_g1_i1	Glyma.18G216600.1	100	120	3	732	2561	1827	1	0	2684

TRINITY_DN177_c0_g1_i1	Glyma.09G272500.1	100	110	3	732	2561	1827	1	0	2739

TRINITY_DN14896_c0_g1_i2	Glyma.07G055600.1	100	410	15	198	2021	1821	1	0	1011

TRINITY_DN14896_c0_g1_i1	Glyma.07G055600.1	100	393	15	198	2021	1821	1	0	1105

TRINITY_DN14337_c0_g2_i1	Glyma.15G208700.2	100	284	18	126	1937	2133	319	0	1592

TRINITY_DN15736_c0_g1_i2	Glyma.08G005900.2	100	281	12	556	2378	373	2195	0	1733

TRINITY_DN15736_c0_g1_i2	Glyma.08G005900.3	100	281	12	556	2378	373	2195	0	1733

TRINITY_DN15736_c0_g1_i2	Glyma.08G005900.1	100	281	12	556	2378	373	2195	0	1733

TRINITY_DN15736_c0_g1_i3	Glyma.08G005900.2	100	281	12	556	2378	373	2195	0	1733

TRINITY_DN15736_c0_g1_i3	Glyma.08G005900.3	97.14285714	281	12	556	2378	373	2195	0	1733

TRINITY_DN15736_c0_g1_i3	Glyma.08G005900.1	95.42253521	281	12	556	2378	373	2195	0	1733

TRINITY_DN14175_c1_g1_i3	Glyma.11G061200.1	98.71794872	266	50	265	2079	1743	1	0	1247

TRINITY_DN15697_c0_g4_i2	Glyma.02G286800.8	96.66666667	253	14	641	2466	1842	32	0	1829

TRINITY_DN15697_c0_g4_i2	Glyma.02G286800.10	96.3963964	253	14	641	2466	1815	5	0	1829

TRINITY_DN15970_c0_g2_i1	Glyma.18G188000.2	100	250	8	230	2055	31	1856	0	1943

TRINITY_DN15970_c0_g2_i9	Glyma.18G188000.2	97.36842105	250	8	230	2055	31	1856	0	1943

TRINITY_DN36824_c0_g1_i1	Glyma.04G027100.2	97.84946237	183	30	741	2545	2	1791	0	1945

TRINITY_DN16327_c0_g1_i2	Glyma.03G142600.1	100	149	14	509	2314	1827	1	0	2379

TRINITY_DN2355_c0_g1_i1	Glyma.10G086700.1	100	129	4	191	2011	1830	1	0	2606

TRINITY_DN2355_c0_g2_i1	Glyma.10G086700.1	95.45454545	129	4	198	2018	1830	1	0	2606

TRINITY_DN2355_c0_g3_i1	Glyma.10G086700.1	96.98492462	129	4	200	2020	1830	1	0	2606

TRINITY_DN15626_c0_g3_i3	Glyma.15G266600.1	97.05882353	126	4	330	2150	1	1830	0	2623

TRINITY_DN15772_c0_g1_i5	Glyma.08G255300.1	96.25	123	0	164	1993	43	1872	0	2699

TRINITY_DN2139_c0_g1_i1	Glyma.08G105800.1	98.97959184	123	1	392	2221	1	1827	0	2678

TRINITY_DN14280_c0_g2_i2	Glyma.17G176400.2	97.84313725	121	7	269	2088	1823	1	0	2599

TRINITY_DN14280_c0_g2_i2	Glyma.17G176400.4	97.84946237	121	7	269	2088	1823	1	0	2599

TRINITY_DN15912_c1_g1_i2	Glyma.13G357100.2	96.41255605	106	16	862	2669	1817	1	0	2566

TRINITY_DN15912_c1_g1_i2	Glyma.13G357100.3	98.96907216	106	16	862	2669	1817	1	0	2566

TRINITY_DN15626_c0_g3_i2	Glyma.15G266600.1	95.04132231	87	4	425	2245	1	1830	0	2839

TRINITY_DN6599_c0_g1_i1	Glyma.13G002200.3	98.82352941	418	46	244	2040	16	1818	0	688

TRINITY_DN15353_c0_g1_i1	Glyma.07G070300.1	98.93617021	274	24	292	2089	2649	828	0	1615

TRINITY_DN15353_c0_g1_i2	Glyma.07G070300.1	98.38709677	274	24	292	2089	2649	828	0	1615

TRINITY_DN14672_c0_g3_i3	Glyma.16G131000.1	97.32142857	265	18	389	2210	202	2011	0	1742

TRINITY_DN13902_c0_g1_i4	Glyma.02G248700.2	100	230	0	115	1943	341	2169	0	2104

TRINITY_DN13902_c0_g1_i4	Glyma.02G248700.1	100	230	0	115	1943	341	2169	0	2104

TRINITY_DN16353_c0_g1_i6	Glyma.19G068800.1	99.36102236	193	7	94	1905	1044	2870	0	2185

TRINITY_DN5722_c0_g1_i1	Glyma.17G258000.1	97.22222222	186	15	1512	3312	1927	115	0	2063

TRINITY_DN5722_c0_g2_i1	Glyma.17G258000.1	100	186	16	1512	3313	1927	115	0	2069

TRINITY_DN12888_c0_g1_i2	Glyma.18G195700.1	97.11538462	164	2	374	2193	1	1829	0	2410

TRINITY_DN12888_c0_g1_i2	Glyma.18G195700.2	97.11538462	164	2	374	2193	1	1829	0	2410

TRINITY_DN16268_c1_g2_i2	Glyma.17G161600.2	98.86363636	158	6	559	2369	1845	17	0	2386

TRINITY_DN13081_c0_g1_i1	Glyma.06G260800.1	100	157	3	173	1998	1	1826	0	2470

TRINITY_DN12951_c0_g1_i1	Glyma.17G211000.1	96.41693811	149	3	1002	2825	1828	1	0	2514

TRINITY_DN16268_c1_g2_i10	Glyma.17G161600.2	96.38554217	145	8	559	2363	1845	17	0	2420

TRINITY_DN16327_c0_g1_i6	Glyma.03G142600.1	95.64428312	144	11	617	2423	1827	1	0	2425

TRINITY_DN15127_c0_g1_i1	Glyma.16G003700.2	97.2972973	140	4	188	2012	67	1894	0	2569

TRINITY_DN7895_c0_g2_i1	Glyma.03G091800.2	100	139	8	1	1813	245	2058	0	2407

TRINITY_DN15127_c0_g1_i1	Glyma.12G167800.2	95.37037037	136	7	188	2012	76	1888	0	2495

TRINITY_DN14953_c0_g1_i1	Glyma.04G123900.1	98.07692308	134	8	1	1822	1873	49	0	2564

TRINITY_DN3772_c0_g1_i1	Glyma.13G196700.1	95.45454545	132	4	1	1822	432	2259	0	2595

TRINITY_DN15424_c0_g1_i1	Glyma.06G255000.3	100	129	0	1043	2871	1829	1	0	2663

TRINITY_DN15424_c0_g1_i2	Glyma.06G255000.3	100	126	1	1043	2846	1829	1	0	2518

TRINITY_DN4194_c0_g1_i1	Glyma.08G041800.2	98.18181818	117	7	171	1992	4	1828	0	2658

TRINITY_DN4194_c0_g2_i1	Glyma.08G041800.2	100	117	7	171	1992	4	1828	0	2658

TRINITY_DN14005_c0_g1_i1	Glyma.11G125500.3	98.5915493	327	20	264	2079	58	1873	0	1410

TRINITY_DN14005_c0_g1_i1	Glyma.11G125500.2	99.38271605	327	20	264	2079	58	1873	0	1410

TRINITY_DN15750_c1_g1_i2	Glyma.13G104000.1	99.04534606	260	9	1047	2869	1875	53	0	1871

TRINITY_DN15750_c1_g1_i5	Glyma.13G104000.1	97.64705882	260	9	962	2784	1875	53	0	1871

TRINITY_DN15970_c0_g2_i1	Glyma.07G137300.2	97.14828897	250	4	230	2055	166	1991	0	1965

TRINITY_DN15970_c0_g2_i9	Glyma.07G137300.2	98.75	250	4	230	2055	166	1991	0	1965

TRINITY_DN35755_c0_g1_i1	Glyma.04G235800.2	98.15668203	166	10	12	1837	57	1873	0	2372

TRINITY_DN35755_c0_g1_i1	Glyma.04G235800.1	98.15668203	166	10	12	1837	57	1873	0	2372

TRINITY_DN14151_c0_g1_i1	Glyma.16G030600.2	96	161	3	727	2545	1	1825	0	2407

TRINITY_DN16268_c1_g2_i2	Glyma.17G161600.3	98.86363636	158	6	559	2368	1842	15	0	2385

TRINITY_DN16268_c1_g2_i2	Glyma.17G161600.4	98.86363636	158	6	559	2368	1830	3	0	2385

TRINITY_DN14146_c1_g1_i2	Glyma.03G245000.1	96.21621622	152	17	1	1810	1813	1	0	2320

TRINITY_DN16386_c0_g1_i2	Glyma.16G198200.1	98.27586207	148	5	45	1868	787	2610	0	2505

TRINITY_DN14146_c1_g1_i2	Glyma.19G242400.1	100	146	17	1	1810	1807	1	0	2314

TRINITY_DN16268_c1_g2_i10	Glyma.17G161600.3	98.64864865	145	8	559	2362	1842	15	0	2418

TRINITY_DN16268_c1_g2_i10	Glyma.17G161600.4	98.64864865	145	8	559	2362	1830	3	0	2418

TRINITY_DN13622_c0_g1_i1	Glyma.09G136800.1	95.2	138	2	401	2227	2649	823	0	2599

TRINITY_DN13622_c0_g1_i1	Glyma.09G136800.2	100	138	2	401	2227	2382	556	0	2599

TRINITY_DN13622_c0_g1_i1	Glyma.09G136800.6	96.2962963	138	2	401	2227	2295	469	0	2599

TRINITY_DN13622_c0_g1_i1	Glyma.09G136800.5	96.95945946	138	2	401	2227	2028	202	0	2599

TRINITY_DN9794_c0_g2_i1	Glyma.17G235000.2	95.65217391	137	4	256	2078	1	1826	0	2571

TRINITY_DN9794_c0_g2_i1	Glyma.17G235000.3	95.62043796	137	4	256	2078	1	1826	0	2571

TRINITY_DN14953_c0_g1_i1	Glyma.04G123900.2	98.07692308	135	8	1	1822	1870	49	0	2551

TRINITY_DN15850_c3_g2_i5	Glyma.02G235700.1	100	131	20	724	2507	3	1825	0	2333

TRINITY_DN16216_c0_g3_i2	Glyma.20G241900.1	97.56097561	130	7	436	2257	594	2406	0	2519

TRINITY_DN5392_c0_g1_i1	Glyma.04G237100.1	100	128	4	471	2294	1824	1	0	2615

TRINITY_DN5392_c0_g2_i1	Glyma.04G237100.1	95.34883721	128	4	471	2294	1824	1	0	2615

TRINITY_DN13963_c0_g1_i1	Glyma.04G053300.4	96.90721649	123	0	127	1954	1	1828	0	2695

TRINITY_DN13963_c0_g1_i1	Glyma.04G053300.1	97.00598802	123	0	127	1954	1	1828	0	2695

TRINITY_DN1464_c0_g1_i1	Glyma.09G190500.2	96.8847352	119	0	552	2379	207	2034	0	2717

TRINITY_DN1464_c0_g1_i1	Glyma.09G190500.1	100	119	0	552	2379	207	2034	0	2717

TRINITY_DN14693_c0_g1_i1	Glyma.10G201000.1	99.35483871	118	7	128	1946	1	1825	0	2645

TRINITY_DN3990_c0_g1_i1	Glyma.15G011200.1	100	109	2	1	1822	183	2010	0	2734

TRINITY_DN12008_c0_g1_i1	Glyma.19G263100.1	98.55072464	367	12	175	1993	120	1941	0	1256

TRINITY_DN10974_c0_g1_i1	Glyma.06G170300.1	95.08196721	334	21	222	2035	1872	56	0	1375

TRINITY_DN15751_c0_g1_i3	Glyma.05G045700.1	100	286	30	1420	3235	420	2214	0	1511

TRINITY_DN15751_c0_g1_i7	Glyma.05G045700.1	96.03174603	286	30	1420	3235	420	2214	0	1511

TRINITY_DN15750_c1_g1_i2	Glyma.17G055100.1	96.85534591	257	7	1047	2869	1875	53	0	1899

TRINITY_DN15750_c1_g1_i5	Glyma.17G055100.1	98.95833333	257	7	962	2784	1875	53	0	1899

TRINITY_DN15924_c0_g1_i4	Glyma.08G300700.2	96.49122807	229	2	386	2209	1827	1	0	2085

TRINITY_DN15924_c0_g1_i4	Glyma.08G300700.1	100	229	2	386	2209	1827	1	0	2085

TRINITY_DN15924_c0_g1_i4	Glyma.08G300700.3	100	229	2	386	2209	1827	1	0	2085

TRINITY_DN15924_c0_g1_i4	Glyma.18G119600.3	96.61016949	226	1	386	2209	1827	1	0	2102

TRINITY_DN15924_c0_g1_i4	Glyma.18G119600.2	99.12280702	226	1	386	2209	1827	1	0	2102

TRINITY_DN15924_c0_g1_i4	Glyma.18G119600.1	100	226	1	386	2209	1827	1	0	2102

TRINITY_DN16353_c0_g1_i4	Glyma.10G284500.1	96.34703196	222	15	389	2200	1006	2817	0	1951

TRINITY_DN16353_c0_g1_i5	Glyma.10G284500.1	100	222	15	471	2282	1006	2817	0	1951

TRINITY_DN11617_c0_g1_i1	Glyma.06G075900.1	95.95959596	195	29	200	1986	23	1809	0	1777

TRINITY_DN8825_c0_g1_i1	Glyma.10G000600.1	100	191	31	100	1908	1	1803	0	2045

TRINITY_DN16032_c0_g1_i5	Glyma.15G140300.1	97.56097561	182	0	263	2089	1	1827	0	2366

TRINITY_DN16032_c0_g1_i7	Glyma.15G140300.1	97.98657718	182	0	110	1936	1	1827	0	2366

TRINITY_DN8237_c0_g1_i1	Glyma.03G028100.1	97.71167048	174	9	320	2130	1944	119	0	2300

TRINITY_DN16032_c0_g1_i1	Glyma.17G033600.1	99.09090909	173	0	212	2038	1	1827	0	2416

TRINITY_DN16032_c0_g1_i1	Glyma.17G033600.2	99.09090909	173	0	212	2038	1	1827	0	2416

TRINITY_DN16032_c0_g1_i1	Glyma.17G033600.3	98.92473118	173	0	212	2038	1	1827	0	2416

TRINITY_DN16032_c0_g1_i3	Glyma.17G033600.1	100	173	0	161	1987	1	1827	0	2416

TRINITY_DN16032_c0_g1_i3	Glyma.17G033600.2	97.54601227	173	0	161	1987	1	1827	0	2416

TRINITY_DN16032_c0_g1_i3	Glyma.17G033600.3	97.54689755	173	0	161	1987	1	1827	0	2416

TRINITY_DN16032_c0_g1_i5	Glyma.09G035600.1	95.8073149	173	0	263	2089	1	1827	0	2416

TRINITY_DN16032_c0_g1_i5	Glyma.09G035600.2	99.2	173	0	263	2089	1	1827	0	2416

TRINITY_DN16032_c0_g1_i7	Glyma.09G035600.1	100	173	0	110	1936	1	1827	0	2416

TRINITY_DN16032_c0_g1_i7	Glyma.09G035600.2	97.98657718	173	0	110	1936	1	1827	0	2416

TRINITY_DN18917_c0_g1_i1	Glyma.06G131400.1	98.33333333	172	14	494	2311	295	2106	0	2266

TRINITY_DN18917_c0_g1_i1	Glyma.06G131400.2	98.31932773	172	14	494	2311	295	2106	0	2266

TRINITY_DN16032_c0_g1_i1	Glyma.07G239700.3	95.8677686	165	0	212	2038	1	1827	0	2460

TRINITY_DN16032_c0_g1_i1	Glyma.07G239700.4	99.09090909	165	0	212	2038	1	1827	0	2460

TRINITY_DN16032_c0_g1_i1	Glyma.07G239700.1	99.09090909	165	0	212	2038	1	1827	0	2460

TRINITY_DN16032_c0_g1_i3	Glyma.07G239700.3	100	165	0	161	1987	1	1827	0	2460

TRINITY_DN16032_c0_g1_i3	Glyma.07G239700.4	98.52216749	165	0	161	1987	1	1827	0	2460

TRINITY_DN16032_c0_g1_i3	Glyma.07G239700.1	95	165	0	161	1987	1	1827	0	2460

TRINITY_DN16032_c0_g1_i2	Glyma.15G140300.1	98.5915493	159	0	212	2038	1	1827	0	2494

TRINITY_DN16032_c0_g1_i2	Glyma.09G035600.1	97.77777778	152	0	212	2038	1	1827	0	2532

TRINITY_DN16032_c0_g1_i2	Glyma.09G035600.2	98.5915493	152	0	212	2038	1	1827	0	2532

TRINITY_DN16203_c0_g1_i1	Glyma.02G101700.1	99.52830189	149	18	85	1871	1	1826	0	2283

TRINITY_DN16032_c0_g1_i4	Glyma.17G033600.1	95.55555556	148	0	263	2089	1	1827	0	2555

TRINITY_DN16032_c0_g1_i4	Glyma.17G033600.2	97.64705882	148	0	263	2089	1	1827	0	2555

TRINITY_DN16032_c0_g1_i4	Glyma.17G033600.3	97.05882353	148	0	263	2089	1	1827	0	2555

TRINITY_DN101_c0_g1_i2	Glyma.10G008200.1	100	144	17	496	2295	1809	1	0	2285

TRINITY_DN13622_c0_g1_i1	Glyma.16G182200.4	96.07843137	143	0	401	2227	2637	811	0	2582

TRINITY_DN13622_c0_g1_i1	Glyma.16G182200.3	98.80952381	143	0	401	2227	2295	469	0	2582

TRINITY_DN3954_c0_g2_i1	Glyma.20G221800.2	95.14563107	143	1	893	2719	1	1824	0	2562

TRINITY_DN3954_c0_g1_i1	Glyma.20G221800.2	100	143	2	893	2718	1	1824	0	2556

TRINITY_DN15127_c0_g1_i1	Glyma.16G003700.3	99.23076923	140	4	188	2010	67	1892	0	2566

TRINITY_DN16032_c0_g1_i4	Glyma.07G239700.3	98.77300613	138	0	263	2089	1	1827	0	2610

TRINITY_DN16032_c0_g1_i4	Glyma.07G239700.4	99.12280702	138	0	263	2089	1	1827	0	2610

TRINITY_DN16032_c0_g1_i4	Glyma.07G239700.1	95.78454333	138	0	263	2089	1	1827	0	2610

TRINITY_DN12776_c0_g2_i2	Glyma.07G037100.2	100	138	22	289	2099	1	1790	0	2266

TRINITY_DN11259_c0_g1_i1	Glyma.08G187600.1	98.52941176	130	2	162	1984	679	2505	0	2628

TRINITY_DN2355_c0_g1_i1	Glyma.02G188200.1	100	124	4	191	2011	1827	1	0	2649

TRINITY_DN2355_c0_g2_i1	Glyma.02G188200.1	96.21621622	124	4	198	2018	1827	1	0	2649

TRINITY_DN2355_c0_g3_i1	Glyma.02G188200.1	96.98492462	124	4	200	2020	1827	1	0	2649

TRINITY_DN16032_c0_g1_i2	Glyma.17G033600.1	99.34210526	119	0	212	2038	1	1827	0	2715

TRINITY_DN16032_c0_g1_i2	Glyma.17G033600.2	96.59090909	119	0	212	2038	1	1827	0	2715

TRINITY_DN16032_c0_g1_i2	Glyma.17G033600.3	97.91666667	119	0	212	2038	1	1827	0	2715

TRINITY_DN11433_c0_g1_i1	Glyma.05G163800.1	100	115	3	484	2298	555	2381	0	2660

TRINITY_DN11433_c0_g1_i1	Glyma.05G163800.6	100	115	3	484	2298	552	2378	0	2660

TRINITY_DN11433_c0_g1_i1	Glyma.05G163800.7	100	115	3	484	2298	552	2378	0	2660

TRINITY_DN11433_c0_g1_i1	Glyma.05G163800.4	95.46351085	115	3	484	2298	336	2162	0	2660

TRINITY_DN11433_c0_g1_i1	Glyma.05G163800.5	96.55172414	115	3	484	2298	336	2162	0	2660

TRINITY_DN11433_c0_g1_i1	Glyma.05G163800.2	96.77419355	115	3	484	2298	333	2159	0	2660

TRINITY_DN11433_c0_g1_i1	Glyma.05G163800.3	97.5877193	115	3	484	2298	333	2159	0	2660

TRINITY_DN16032_c0_g1_i2	Glyma.07G239700.3	99.28571429	113	0	212	2038	1	1827	0	2748

TRINITY_DN16032_c0_g1_i2	Glyma.07G239700.4	98.39816934	113	0	212	2038	1	1827	0	2748

TRINITY_DN16032_c0_g1_i2	Glyma.07G239700.1	100	113	0	212	2038	1	1827	0	2748

TRINITY_DN16032_c0_g1_i4	Glyma.15G140300.1	95	113	0	263	2089	1	1827	0	2748

TRINITY_DN12169_c0_g1_i1	Glyma.05G243900.1	100	105	0	597	2423	1827	1	0	2793

TRINITY_DN16032_c0_g1_i4	Glyma.09G035600.1	98.26086957	103	0	263	2089	1	1827	0	2804

TRINITY_DN16032_c0_g1_i4	Glyma.09G035600.2	97.40802676	103	0	263	2089	1	1827	0	2804

TRINITY_DN15621_c0_g1_i2	Glyma.19G089800.3	100	98	0	530	2356	1	1827	0	2832

TRINITY_DN15621_c0_g1_i3	Glyma.19G089800.3	100	98	0	530	2356	1	1827	0	2832

TRINITY_DN16032_c0_g1_i5	Glyma.17G033600.1	100	94	0	263	2089	1	1827	0	2854

TRINITY_DN16032_c0_g1_i5	Glyma.17G033600.2	100	94	0	263	2089	1	1827	0	2854

TRINITY_DN16032_c0_g1_i5	Glyma.17G033600.3	100	94	0	263	2089	1	1827	0	2854

TRINITY_DN16032_c0_g1_i7	Glyma.17G033600.1	97.01492537	94	0	110	1936	1	1827	0	2854

TRINITY_DN16032_c0_g1_i7	Glyma.17G033600.2	100	94	0	110	1936	1	1827	0	2854

TRINITY_DN16032_c0_g1_i7	Glyma.17G033600.3	100	94	0	110	1936	1	1827	0	2854

TRINITY_DN16032_c0_g1_i1	Glyma.15G140300.1	95.8677686	90	0	212	2038	1	1827	0	2876

TRINITY_DN16032_c0_g1_i3	Glyma.15G140300.1	95.6043956	90	0	161	1987	1	1827	0	2876

TRINITY_DN16032_c0_g1_i5	Glyma.07G239700.3	95.27559055	86	0	263	2089	1	1827	0	2898

TRINITY_DN16032_c0_g1_i5	Glyma.07G239700.4	100	86	0	263	2089	1	1827	0	2898

TRINITY_DN16032_c0_g1_i5	Glyma.07G239700.1	100	86	0	263	2089	1	1827	0	2898

TRINITY_DN16032_c0_g1_i7	Glyma.07G239700.3	95.71428571	86	0	110	1936	1	1827	0	2898

TRINITY_DN16032_c0_g1_i7	Glyma.07G239700.4	96.13402062	86	0	110	1936	1	1827	0	2898

TRINITY_DN16032_c0_g1_i7	Glyma.07G239700.1	97.72727273	86	0	110	1936	1	1827	0	2898

TRINITY_DN16032_c0_g1_i1	Glyma.09G035600.1	96.82539683	82	0	212	2038	1	1827	0	2920

TRINITY_DN16032_c0_g1_i1	Glyma.09G035600.2	99.02912621	82	0	212	2038	1	1827	0	2920

TRINITY_DN16032_c0_g1_i3	Glyma.09G035600.1	97.39776952	82	0	161	1987	1	1827	0	2920

TRINITY_DN16032_c0_g1_i3	Glyma.09G035600.2	97.39776952	82	0	161	1987	1	1827	0	2920

TRINITY_DN12082_c0_g1_i1	Glyma.19G116500.1	100	342	15	164	1985	45	1853	0	1341

TRINITY_DN12082_c0_g1_i2	Glyma.19G116500.1	100	342	15	330	2151	45	1853	0	1341

TRINITY_DN12830_c0_g2_i1	Glyma.08G071200.2	98.74213836	298	10	241	2062	176	1985	0	1592

TRINITY_DN12830_c0_g2_i1	Glyma.08G071200.3	98.74213836	298	10	241	2062	176	1985	0	1592

TRINITY_DN12830_c0_g2_i1	Glyma.08G071200.1	98.75	298	10	241	2062	176	1985	0	1592

TRINITY_DN14672_c0_g3_i3	Glyma.02G050700.1	97.59036145	284	15	389	2209	208	2016	0	1657

TRINITY_DN15353_c0_g1_i1	Glyma.07G070300.2	98.93617021	274	23	292	2089	2646	828	0	1628

TRINITY_DN15353_c0_g1_i2	Glyma.07G070300.2	98.7654321	274	23	292	2089	2646	828	0	1628

TRINITY_DN15663_c0_g1_i2	Glyma.02G034700.1	95.6043956	257	14	689	2501	1887	66	0	1838

TRINITY_DN15663_c0_g1_i2	Glyma.02G034700.2	96.27071823	257	14	689	2501	1887	66	0	1838

TRINITY_DN14372_c0_g2_i5	Glyma.20G237900.1	99.12280702	189	13	180	1993	1	1812	0	2158

TRINITY_DN12526_c0_g1_i1	Glyma.13G238000.3	100	172	10	1135	2955	1113	2924	0	2296

TRINITY_DN12526_c0_g1_i1	Glyma.13G238000.4	99.15254237	172	10	1135	2955	1113	2924	0	2296

TRINITY_DN14364_c0_g2_i3	Glyma.15G143400.1	95.94594595	148	1	1432	3257	1824	2	0	2532

TRINITY_DN17258_c0_g1_i1	Glyma.01G023700.1	97.36842105	141	4	1	1819	1155	2979	0	2540

TRINITY_DN15850_c3_g2_i5	Glyma.14G203700.1	98.80952381	137	14	724	2507	3	1822	0	2303

TRINITY_DN14870_c0_g1_i5	Glyma.07G019100.1	99.12280702	134	16	273	2072	1821	1	0	2429

TRINITY_DN15970_c0_g2_i1	Glyma.01G099800.2	96.72131148	123	0	230	2055	31	1856	0	2691

TRINITY_DN15970_c0_g2_i9	Glyma.01G099800.2	95.95959596	123	0	230	2055	31	1856	0	2691

TRINITY_DN8623_c0_g1_i1	Glyma.11G248700.3	96.99570815	115	2	696	2519	1922	99	0	2710

TRINITY_DN15981_c0_g1_i1	Glyma.11G048300.3	100	115	3	1370	3189	1826	1	0	2697

TRINITY_DN15981_c0_g1_i13	Glyma.11G048300.3	100	115	3	1354	3173	1826	1	0	2697

TRINITY_DN15981_c0_g1_i18	Glyma.11G048300.3	98.64864865	115	3	1244	3063	1826	1	0	2697

TRINITY_DN15981_c0_g1_i23	Glyma.11G048300.3	96.2962963	115	3	1480	3299	1826	1	0	2697

TRINITY_DN15981_c0_g1_i4	Glyma.11G048300.3	98.27586207	115	3	1244	3063	1826	1	0	2697

TRINITY_DN15981_c0_g1_i7	Glyma.11G048300.3	99.2248062	115	3	1354	3173	1826	1	0	2697

TRINITY_DN15981_c0_g1_i9	Glyma.11G048300.3	97.14285714	115	3	1480	3299	1826	1	0	2697

TRINITY_DN10873_c0_g2_i1	Glyma.04G017700.1	97.18309859	101	4	26	1849	1821	1	0	2767

TRINITY_DN14778_c0_g1_i1	Glyma.14G035400.1	95.23809524	100	2	74	1896	788	2604	0	2741

TRINITY_DN14778_c0_g1_i1	Glyma.02G279300.1	95.23809524	97	1	74	1896	788	2613	0	2815

TRINITY_DN7878_c0_g1_i1	Glyma.01G011200.4	99.03846154	93	8	1147	2971	1816	1	0	2785

TRINITY_DN7878_c0_g3_i1	Glyma.01G011200.4	99.02912621	93	8	1147	2971	1816	1	0	2785

TRINITY_DN16360_c0_g4_i1	Glyma.13G104000.2	95.00805153	431	52	1021	2820	549	2333	1.69E-158	562

TRINITY_DN14005_c0_g1_i1	Glyma.12G050100.2	99.38271605	334	14	264	2079	58	1873	0	1404

TRINITY_DN14005_c0_g1_i1	Glyma.12G050100.3	99.38271605	334	14	264	2079	58	1873	0	1404

TRINITY_DN14005_c0_g1_i1	Glyma.12G050100.4	99.38461538	334	14	264	2079	58	1873	0	1404

TRINITY_DN14005_c0_g1_i1	Glyma.12G050100.1	96.91358025	334	14	264	2079	58	1873	0	1404

TRINITY_DN15910_c0_g1_i5	Glyma.10G012000.1	100	290	2	584	2407	271	2094	0	1751

TRINITY_DN9650_c0_g1_i1	Glyma.13G211600.1	96.72131148	213	13	350	2152	1824	1	0	2041

TRINITY_DN10795_c0_g2_i1	Glyma.02G148900.5	100	212	22	152	1950	1	1802	0	1879

TRINITY_DN10795_c0_g2_i1	Glyma.02G148900.3	100	212	22	152	1950	1	1802	0	1879

TRINITY_DN13448_c0_g1_i7	Glyma.18G011000.1	96.46017699	192	2	17	1840	43	1866	0	2294

TRINITY_DN16290_c0_g1_i9	Glyma.13G254200.2	100	153	6	284	2080	1	1824	0	2335

TRINITY_DN16290_c0_g1_i9	Glyma.13G254200.1	100	153	6	284	2080	1	1824	0	2335

TRINITY_DN16290_c0_g1_i3	Glyma.13G254200.2	100	151	2	187	2010	1	1824	0	2521

TRINITY_DN16290_c0_g1_i3	Glyma.13G254200.1	97.98657718	151	2	187	2010	1	1824	0	2521

TRINITY_DN16290_c0_g1_i7	Glyma.13G254200.2	97.39130435	151	2	284	2107	1	1824	0	2521

TRINITY_DN16290_c0_g1_i7	Glyma.13G254200.1	97.39130435	151	2	284	2107	1	1824	0	2521

TRINITY_DN14364_c0_g2_i3	Glyma.09G038300.2	96.77419355	148	1	1433	3257	1826	5	0	2531

TRINITY_DN14364_c0_g2_i3	Glyma.09G038300.1	98.33333333	148	1	1433	3257	1826	5	0	2531

TRINITY_DN15549_c0_g1_i4	Glyma.08G055400.2	99.20634921	144	7	1048	2842	2642	821	0	2359

TRINITY_DN15549_c0_g1_i7	Glyma.08G055400.2	97.69230769	144	7	1012	2806	2642	821	0	2359

TRINITY_DN5392_c0_g1_i1	Glyma.06G127100.2	95.03311258	136	2	473	2294	1825	1	0	2597

TRINITY_DN5392_c0_g1_i1	Glyma.06G127100.3	100	136	2	473	2294	1825	1	0	2597

TRINITY_DN5392_c0_g1_i1	Glyma.06G127100.1	100	136	2	473	2294	1825	1	0	2597

TRINITY_DN5392_c0_g2_i1	Glyma.06G127100.2	99.13793103	136	2	473	2294	1825	1	0	2597

TRINITY_DN5392_c0_g2_i1	Glyma.06G127100.3	96.80696662	136	2	473	2294	1825	1	0	2597

TRINITY_DN5392_c0_g2_i1	Glyma.06G127100.1	95.65217391	136	2	473	2294	1825	1	0	2597

TRINITY_DN3772_c0_g1_i1	Glyma.15G234800.1	95.45454545	131	1	1	1822	432	2256	0	2625

TRINITY_DN15619_c0_g2_i4	Glyma.17G088800.3	98.75	124	3	951	2767	3	1826	0	2625

TRINITY_DN15624_c0_g1_i3	Glyma.14G169300.4	98.73015873	116	4	181	2004	1	1821	0	2695

TRINITY_DN15624_c0_g1_i5	Glyma.14G169300.4	95.96412556	116	4	181	2004	1	1821	0	2695

TRINITY_DN14713_c0_g1_i5	Glyma.06G087800.4	100	115	12	128	1941	40	1857	0	2617

TRINITY_DN15624_c0_g1_i2	Glyma.14G169300.4	99.38461538	114	8	181	1991	1	1821	0	2623

TRINITY_DN10529_c0_g2_i1	Glyma.08G185700.6	99.68944099	111	0	1	1825	87	1911	0	2756

TRINITY_DN3197_c0_g1_i1	Glyma.17G001500.1	100	68	0	1	1825	33	1857	0	2994

TRINITY_DN3197_c0_g1_i1	Glyma.07G273100.1	100	67	0	1	1825	33	1857	0	3000

TRINITY_DN15663_c0_g1_i2	Glyma.01G030900.2	96.1038961	269	13	697	2501	1882	63	0	1729

TRINITY_DN15663_c0_g1_i2	Glyma.01G030900.1	100	269	13	697	2501	1882	63	0	1729

TRINITY_DN12379_c0_g1_i1	Glyma.15G071200.1	95.85492228	226	15	100	1918	1	1807	0	1975

TRINITY_DN4598_c0_g1_i1	Glyma.11G202800.1	95.20958084	213	19	472	2259	388	2199	0	1879

TRINITY_DN13448_c0_g1_i7	Glyma.11G246200.8	96.46017699	201	0	17	1840	43	1866	0	2255

TRINITY_DN13448_c0_g1_i7	Glyma.11G246200.6	96.46017699	201	0	17	1840	43	1866	0	2255

TRINITY_DN13448_c0_g1_i7	Glyma.11G246200.9	97.79411765	201	0	17	1840	43	1866	0	2255

TRINITY_DN13448_c0_g1_i7	Glyma.11G246200.1	97.75280899	201	0	17	1840	43	1866	0	2255

TRINITY_DN15815_c0_g2_i5	Glyma.12G147000.2	97.91666667	194	7	2470	4292	1803	1	0	2152

TRINITY_DN15815_c0_g2_i6	Glyma.12G147000.2	99.20634921	194	7	1909	3731	1803	1	0	2152

TRINITY_DN13081_c0_g1_i1	Glyma.12G141000.1	96.42857143	157	0	173	1996	1	1824	0	2499

TRINITY_DN12292_c0_g5_i1	Glyma.17G128500.8	100	157	9	1	1802	694	2516	0	2350

TRINITY_DN12292_c0_g5_i1	Glyma.17G128500.12	100	157	9	1	1802	271	2093	0	2350

TRINITY_DN16290_c0_g1_i5	Glyma.13G254200.2	98.24561404	156	4	184	1980	1	1824	0	2329

TRINITY_DN16290_c0_g1_i5	Glyma.13G254200.1	98.1595092	156	4	184	1980	1	1824	0	2329

TRINITY_DN18172_c0_g1_i1	Glyma.01G011700.1	100	152	1	122	1945	1818	1	0	2488

TRINITY_DN16290_c0_g1_i5	Glyma.15G060700.1	98.63945578	149	11	184	1980	1	1824	0	2368

TRINITY_DN16290_c0_g1_i5	Glyma.15G060700.2	98.63945578	149	11	184	1980	1	1824	0	2368

TRINITY_DN16290_c0_g1_i9	Glyma.15G060700.1	98.54014599	149	11	284	2080	1	1824	0	2368

TRINITY_DN16290_c0_g1_i9	Glyma.15G060700.2	99.28571429	149	11	284	2080	1	1824	0	2368

TRINITY_DN18172_c0_g1_i1	Glyma.01G011300.1	100	147	1	122	1945	1818	1	0	2516

TRINITY_DN16290_c0_g1_i3	Glyma.15G060700.1	95	145	0	187	2010	1	1824	0	2566

TRINITY_DN16290_c0_g1_i3	Glyma.15G060700.2	97.5	145	0	187	2010	1	1824	0	2566

TRINITY_DN16290_c0_g1_i7	Glyma.15G060700.1	96.48241206	145	0	284	2107	1	1824	0	2566

TRINITY_DN16290_c0_g1_i7	Glyma.15G060700.2	97.64705882	145	0	284	2107	1	1824	0	2566

TRINITY_DN8977_c0_g1_i1	Glyma.06G094000.6	98.35526316	138	19	39	1847	476	2266	0	2294

TRINITY_DN14450_c1_g1_i2	Glyma.11G110800.5	99.38461538	129	15	330	2128	2678	868	0	2409

TRINITY_DN14450_c1_g1_i2	Glyma.11G110800.7	99.39209726	129	15	330	2128	2678	868	0	2409

TRINITY_DN14450_c1_g1_i2	Glyma.11G110800.3	99.3902439	129	15	330	2128	2678	868	0	2409

TRINITY_DN15697_c0_g1_i3	Glyma.15G240700.1	97.22222222	116	2	449	2269	1824	1	0	2706

TRINITY_DN15924_c0_g1_i4	Glyma.02G106900.1	100	113	0	386	2209	1824	1	0	2743

TRINITY_DN14650_c0_g2_i2	Glyma.06G031900.1	95.58823529	102	4	432	2253	1918	97	0	2778

TRINITY_DN13573_c0_g1_i1	Glyma.18G059700.1	97.48822606	453	35	740	2542	2693	891	1.83E-169	599

TRINITY_DN14604_c0_g1_i1	Glyma.16G116000.2	97.52650177	313	10	701	2517	1	1805	0	1478

TRINITY_DN14547_c0_g1_i2	Glyma.16G079200.1	97.5	295	15	254	2065	1845	35	0	1583

TRINITY_DN14547_c0_g1_i3	Glyma.16G079200.1	100	295	15	254	2065	1845	35	0	1583

TRINITY_DN12379_c0_g1_i1	Glyma.13G242300.1	100	238	13	100	1918	1	1804	0	1899

TRINITY_DN13902_c0_g1_i2	Glyma.11G219700.1	97.05882353	227	8	115	1928	341	2157	0	2012

TRINITY_DN16309_c0_g1_i5	Glyma.11G033600.2	99.31034483	217	30	3	1789	3019	4823	0	1816

TRINITY_DN16309_c0_g1_i5	Glyma.11G033600.1	100	217	30	3	1789	3019	4823	0	1816

TRINITY_DN16309_c0_g1_i5	Glyma.11G033600.3	100	217	30	3	1789	3019	4823	0	1816

TRINITY_DN16309_c0_g1_i5	Glyma.11G033600.4	100	217	30	3	1789	3019	4823	0	1816

TRINITY_DN10517_c0_g1_i1	Glyma.02G026500.1	98.85714286	173	14	123	1925	1	1818	0	2246

TRINITY_DN7724_c0_g1_i1	Glyma.18G013200.1	95.14563107	157	26	168	1927	23	1836	0	2032

TRINITY_DN7724_c0_g1_i2	Glyma.18G013200.1	100	157	26	168	1927	23	1836	0	2032

TRINITY_DN16130_c2_g6_i10	Glyma.19G196000.2	100	154	1	337	2158	383	2205	0	2507

TRINITY_DN16130_c2_g6_i7	Glyma.19G196000.2	100	154	1	337	2158	383	2205	0	2507

TRINITY_DN11634_c0_g1_i4	Glyma.10G083500.1	97.60609358	153	10	710	2521	495	2310	0	2403

TRINITY_DN6539_c0_g1_i2	Glyma.08G038200.8	100	142	6	417	2234	1866	45	0	2542

TRINITY_DN6539_c0_g1_i2	Glyma.08G038200.5	100	142	6	417	2234	1866	45	0	2542

TRINITY_DN6539_c0_g1_i2	Glyma.08G038200.1	100	142	6	417	2234	1866	45	0	2542

TRINITY_DN6539_c0_g1_i2	Glyma.08G038200.9	99.62264151	142	6	417	2234	1866	45	0	2542

TRINITY_DN6539_c0_g1_i2	Glyma.08G038200.6	99.6	142	6	417	2234	1866	45	0	2542

TRINITY_DN6539_c0_g1_i2	Glyma.05G230700.2	100	137	6	417	2234	1866	45	0	2569

TRINITY_DN13307_c0_g1_i1	Glyma.08G327900.1	95.65217391	128	4	51	1864	1	1823	0	2599

TRINITY_DN13307_c0_g1_i3	Glyma.08G327900.1	100	128	4	232	2045	1	1823	0	2599

TRINITY_DN13307_c0_g1_i1	Glyma.18G079400.1	100	126	4	51	1864	1	1823	0	2610

TRINITY_DN13307_c0_g1_i1	Glyma.18G079400.5	99.00990099	126	4	51	1864	1	1823	0	2610

TRINITY_DN13307_c0_g1_i1	Glyma.18G079400.2	99.67741935	126	4	51	1864	1	1823	0	2610

TRINITY_DN13307_c0_g1_i1	Glyma.18G079400.3	98.71794872	126	4	51	1864	1	1823	0	2610

TRINITY_DN13307_c0_g1_i1	Glyma.18G079400.4	97.01492537	126	4	51	1864	1	1823	0	2610

TRINITY_DN13307_c0_g1_i3	Glyma.18G079400.1	96.47058824	126	4	232	2045	1	1823	0	2610

TRINITY_DN13307_c0_g1_i3	Glyma.18G079400.5	96.78571429	126	4	232	2045	1	1823	0	2610

TRINITY_DN13307_c0_g1_i3	Glyma.18G079400.2	99.49494949	126	4	232	2045	1	1823	0	2610

TRINITY_DN13307_c0_g1_i3	Glyma.18G079400.3	97.20496894	126	4	232	2045	1	1823	0	2610

TRINITY_DN13307_c0_g1_i3	Glyma.18G079400.4	96.15384615	126	4	232	2045	1	1823	0	2610

TRINITY_DN16130_c2_g6_i6	Glyma.19G196000.2	97.95918367	106	1	337	2158	383	2205	0	2772

TRINITY_DN14778_c0_g1_i1	Glyma.14G035400.2	95.23809524	100	1	74	1896	788	2601	0	2754

TRINITY_DN14778_c0_g1_i1	Glyma.02G279300.2	95.23809524	97	0	74	1896	788	2610	0	2830

TRINITY_DN14526_c0_g1_i1	Glyma.01G226700.2	100	86	1	291	2113	1848	27	0	2883

TRINITY_DN6599_c0_g1_i1	Glyma.02G271300.2	98.82352941	78	2	223	2044	1	1822	0	2922

TRINITY_DN16436_c0_g2_i11	Glyma.15G033000.2	96.9581749	287	19	709	2510	1	1812	0	1581

TRINITY_DN10974_c0_g2_i1	Glyma.05G047900.1	98.79518072	277	21	1685	3495	1835	25	0	1688

TRINITY_DN10974_c0_g2_i4	Glyma.05G047900.1	96.62921348	277	21	1813	3623	1835	25	0	1688

TRINITY_DN14238_c0_g2_i3	Glyma.07G189200.1	97.63313609	228	12	367	2185	63	1869	0	1986

TRINITY_DN14238_c0_g2_i4	Glyma.07G189200.1	98.65771812	228	12	147	1965	63	1869	0	1986

TRINITY_DN15946_c0_g2_i3	Glyma.11G087500.1	95.87155963	189	36	283	2062	6	1803	0	1892

TRINITY_DN1479_c0_g1_i2	Glyma.06G017300.1	100	151	11	142	1942	1998	186	0	2335

TRINITY_DN19231_c0_g1_i1	Glyma.14G075000.1	96.67832168	149	8	30	1844	112	1929	0	2468

TRINITY_DN15924_c0_g1_i4	Glyma.01G046700.2	98.03921569	117	0	388	2209	1822	1	0	2717

TRINITY_DN15924_c0_g1_i4	Glyma.01G046700.1	100	117	0	388	2209	1822	1	0	2717

TRINITY_DN16245_c0_g3_i4	Glyma.06G208900.1	100	409	38	599	2398	3243	1447	0	806

TRINITY_DN16245_c0_g3_i4	Glyma.06G208900.4	98.4984985	409	38	599	2398	3243	1447	0	806

TRINITY_DN16245_c0_g3_i4	Glyma.06G208900.2	98.31223629	409	38	599	2398	2940	1144	0	806

TRINITY_DN16245_c0_g3_i4	Glyma.06G208900.3	98.31223629	409	38	599	2398	2940	1144	0	806

TRINITY_DN11607_c0_g2_i1	Glyma.08G003100.2	98.42105263	270	7	131	1948	1815	1	0	1808

TRINITY_DN11607_c0_g2_i1	Glyma.08G003100.1	96.92307692	270	7	131	1948	1815	1	0	1808

TRINITY_DN16309_c0_g1_i5	Glyma.01G208600.1	99.31034483	215	26	3	1789	3025	4829	0	1849

TRINITY_DN16353_c0_g1_i6	Glyma.10G284500.1	99.36708861	201	13	91	1905	1679	3491	0	2159

TRINITY_DN11617_c0_g1_i1	Glyma.06G075900.3	97.07602339	195	28	200	1986	23	1803	0	1805

TRINITY_DN11617_c0_g1_i1	Glyma.06G075900.2	99.56331878	194	29	200	1986	23	1794	0	1751

TRINITY_DN13991_c0_g1_i2	Glyma.13G111500.3	95.12195122	192	10	739	2547	1818	1	0	2202

TRINITY_DN5722_c0_g2_i1	Glyma.17G258000.5	100	186	15	1512	3313	1918	115	0	2100

TRINITY_DN5722_c0_g2_i1	Glyma.17G258000.4	100	186	15	1512	3313	1915	112	0	2100

TRINITY_DN15774_c0_g1_i2	Glyma.06G087500.1	100	166	2	1424	3241	1821	1	0	2423

TRINITY_DN15774_c0_g1_i3	Glyma.06G087500.1	98.94736842	166	2	2269	4086	1821	1	0	2423

TRINITY_DN2620_c0_g1_i1	Glyma.11G102000.2	100	164	7	969	2771	1863	43	0	2338

TRINITY_DN101_c0_g1_i2	Glyma.20G087700.1	100	143	17	496	2295	1812	1	0	2377

TRINITY_DN15697_c0_g4_i2	Glyma.08G309500.2	100	112	9	641	2455	1812	1	0	2645

TRINITY_DN15697_c0_g4_i2	Glyma.18G106800.2	100	106	5	641	2455	1809	1	0	2660

TRINITY_DN208_c0_g1_i1	Glyma.11G090900.1	100	98	0	527	2347	1821	1	0	2820

TRINITY_DN16444_c0_g1_i1	Glyma.13G160000.1	98.7654321	313	17	12	1817	1115	2926	0	1485

TRINITY_DN13723_c0_g1_i1	Glyma.16G074800.13	100	310	21	187	1993	1	1812	0	1507

TRINITY_DN13723_c0_g1_i1	Glyma.16G074800.11	95.86206897	310	21	187	1993	1	1812	0	1507

TRINITY_DN13723_c0_g1_i1	Glyma.16G074800.9	98.57142857	310	21	187	1993	1	1812	0	1507

TRINITY_DN13723_c0_g1_i1	Glyma.16G074800.7	100	310	21	187	1993	1	1812	0	1507

TRINITY_DN13723_c0_g1_i1	Glyma.16G074800.4	99.40828402	310	21	187	1993	1	1812	0	1507

TRINITY_DN13723_c0_g1_i1	Glyma.16G074800.2	99.40828402	310	21	187	1993	1	1812	0	1507

TRINITY_DN13723_c0_g1_i1	Glyma.16G074800.1	95.40229885	310	21	187	1993	1	1812	0	1507

TRINITY_DN13723_c0_g1_i4	Glyma.16G074800.13	98.57142857	310	21	569	2375	1	1812	0	1507

TRINITY_DN13723_c0_g1_i4	Glyma.16G074800.11	98.57142857	310	21	569	2375	1	1812	0	1507

TRINITY_DN13723_c0_g1_i4	Glyma.16G074800.9	98.57142857	310	21	569	2375	1	1812	0	1507

TRINITY_DN13723_c0_g1_i4	Glyma.16G074800.7	98.57142857	310	21	569	2375	1	1812	0	1507

TRINITY_DN13723_c0_g1_i4	Glyma.16G074800.4	100	310	21	569	2375	1	1812	0	1507

TRINITY_DN13723_c0_g1_i4	Glyma.16G074800.2	100	310	21	569	2375	1	1812	0	1507

TRINITY_DN13723_c0_g1_i4	Glyma.16G074800.1	100	310	21	569	2375	1	1812	0	1507

TRINITY_DN15848_c1_g4_i1	Glyma.20G007900.3	100	211	8	247	2061	1	1815	0	2128

TRINITY_DN5722_c0_g1_i1	Glyma.17G258000.5	100	186	14	1512	3312	1918	115	0	2104

TRINITY_DN5722_c0_g1_i1	Glyma.17G258000.4	100	186	14	1512	3312	1915	112	0	2104

TRINITY_DN4211_c0_g1_i1	Glyma.18G259000.2	100	186	32	328	2101	1	1810	0	1969

TRINITY_DN9401_c0_g1_i1	Glyma.09G012600.1	95.45454545	179	7	440	2253	2097	296	0	2215

TRINITY_DN11281_c0_g1_i1	Glyma.18G240300.1	99.28571429	163	3	497	2312	1826	8	0	2425

TRINITY_DN14225_c0_g1_i3	Glyma.04G182000.1	98.52507375	148	10	260	2058	86	1899	0	2366

TRINITY_DN14225_c0_g1_i3	Glyma.06G183100.1	98.51851852	146	8	260	2058	89	1905	0	2398

TRINITY_DN15533_c0_g1_i4	Glyma.18G019700.1	98.7012987	132	12	3	1802	313	2127	0	2468

TRINITY_DN101_c0_g1_i1	Glyma.10G008200.1	96.73076923	129	17	496	2289	1809	1	0	2407

TRINITY_DN16216_c0_g3_i2	Glyma.11G012500.1	97.25685786	125	0	436	2255	825	2644	0	2669

TRINITY_DN16216_c0_g3_i2	Glyma.11G012500.2	97.25685786	125	0	436	2255	825	2644	0	2669

TRINITY_DN14450_c1_g1_i2	Glyma.12G037100.1	100	123	12	330	2128	2669	865	0	2447

TRINITY_DN14450_c1_g1_i2	Glyma.12G037100.5	100	123	12	330	2128	2669	865	0	2447

TRINITY_DN16216_c0_g3_i2	Glyma.01G229700.1	97.85407725	113	0	436	2255	678	2497	0	2736

TRINITY_DN16054_c1_g1_i3	Glyma.10G168700.1	95.75757576	109	0	375	2194	2967	1148	0	2758

TRINITY_DN16054_c1_g1_i3	Glyma.10G168700.3	95.30201342	109	0	375	2194	2235	416	0	2758

TRINITY_DN16054_c1_g1_i3	Glyma.20G221000.1	96.42857143	97	0	375	2194	2967	1148	0	2824

TRINITY_DN6625_c0_g2_i1	Glyma.10G019700.1	99.4011976	182	7	285	2091	1819	1	0	2274

TRINITY_DN13808_c0_g1_i2	Glyma.02G048200.1	97.59299781	148	9	70	1864	1	1807	0	2307

TRINITY_DN8746_c0_g1_i2	Glyma.13G104400.1	96.25	144	6	699	2517	1804	1	0	2464

TRINITY_DN8746_c0_g1_i2	Glyma.13G104400.2	100	144	6	699	2517	1804	1	0	2464

TRINITY_DN38228_c0_g1_i1	Glyma.16G018400.3	100	102	1	125	1943	1	1816	0	2774

TRINITY_DN38228_c0_g1_i1	Glyma.16G018400.4	96	102	1	125	1943	1	1816	0	2774

TRINITY_DN38228_c0_g1_i1	Glyma.16G018400.2	95.79831933	101	3	125	1943	1	1801	0	2684

TRINITY_DN14526_c0_g1_i1	Glyma.11G016000.2	100	82	0	291	2109	1866	48	0	2905

TRINITY_DN14337_c0_g2_i3	Glyma.15G208700.2	100	284	18	260	2059	2121	319	0	1570

TRINITY_DN15888_c0_g3_i1	Glyma.14G048300.1	96.11650485	224	15	1263	3072	1490	3287	0	1936

TRINITY_DN15818_c1_g2_i4	Glyma.03G136500.2	96.40831758	214	2	1	1817	1817	1	0	2159

TRINITY_DN15818_c1_g2_i4	Glyma.03G136500.1	96.40883978	214	2	1	1817	1817	1	0	2159

TRINITY_DN11617_c0_g1_i1	Glyma.04G074900.1	98	196	17	200	1986	23	1821	0	1949

TRINITY_DN11617_c0_g1_i1	Glyma.04G074900.3	97.37609329	195	18	200	1986	23	1812	0	1895

TRINITY_DN15818_c1_g2_i3	Glyma.19G138700.1	99.62546816	194	2	1	1817	1817	1	0	2270

TRINITY_DN15818_c1_g2_i3	Glyma.19G138700.2	96.55172414	194	2	1	1817	1817	1	0	2270

TRINITY_DN15818_c1_g2_i3	Glyma.02G184600.1	99.13043478	183	2	1	1817	1817	1	0	2331

TRINITY_DN15818_c1_g2_i3	Glyma.10G099200.3	99.13043478	183	2	1	1817	1817	1	0	2331

TRINITY_DN15818_c1_g2_i3	Glyma.10G099200.2	100	183	2	1	1817	1817	1	0	2331

TRINITY_DN15818_c1_g2_i3	Glyma.10G099200.1	98.85057471	183	2	1	1817	1817	1	0	2331

TRINITY_DN15818_c1_g2_i3	Glyma.03G136500.2	95.12195122	182	2	1	1817	1817	1	0	2337

TRINITY_DN15818_c1_g2_i3	Glyma.03G136500.1	95.12195122	182	2	1	1817	1817	1	0	2337

TRINITY_DN9156_c0_g1_i1	Glyma.18G109800.2	96.24413146	169	3	63	1879	1	1804	0	2324

TRINITY_DN10517_c0_g1_i1	Glyma.01G038500.1	98.86363636	166	9	123	1925	1	1818	0	2340

TRINITY_DN4472_c0_g1_i1	Glyma.20G212000.1	100	164	4	297	2108	1818	1	0	2410

TRINITY_DN4472_c0_g1_i2	Glyma.20G212000.1	100	164	4	297	2108	1818	1	0	2410

TRINITY_DN14547_c0_g1_i2	Glyma.18G246400.1	96.9151671	161	2	254	2068	1857	41	0	2440

TRINITY_DN14547_c0_g1_i3	Glyma.18G246400.1	100	161	2	254	2068	1857	41	0	2440

TRINITY_DN15818_c1_g2_i4	Glyma.10G099200.3	100	157	2	1	1817	1817	1	0	2475

TRINITY_DN15818_c1_g2_i4	Glyma.10G099200.2	99.01960784	157	2	1	1817	1817	1	0	2475

TRINITY_DN15818_c1_g2_i4	Glyma.10G099200.1	98.61111111	157	2	1	1817	1817	1	0	2475

TRINITY_DN16012_c0_g1_i2	Glyma.17G010500.1	95.79158317	155	2	287	2101	1	1818	0	2479

TRINITY_DN16012_c0_g1_i2	Glyma.17G010500.2	100	155	2	287	2101	1	1818	0	2479

TRINITY_DN15818_c1_g2_i4	Glyma.02G184600.1	99.04761905	151	2	1	1817	1817	1	0	2508

TRINITY_DN16012_c0_g1_i2	Glyma.07G263300.1	97.76119403	149	2	287	2101	1	1818	0	2512

TRINITY_DN11634_c0_g1_i4	Glyma.02G193700.1	100	143	5	713	2521	496	2310	0	2488

TRINITY_DN15533_c0_g1_i4	Glyma.11G237500.3	100	143	9	1	1802	311	2118	0	2398

TRINITY_DN15533_c0_g1_i4	Glyma.11G237500.1	98.27586207	143	9	1	1802	311	2118	0	2398

TRINITY_DN15533_c0_g1_i4	Glyma.11G237500.2	99.42528736	142	12	1	1802	311	2115	0	2383

TRINITY_DN11259_c0_g1_i1	Glyma.12G170500.1	97.10144928	140	1	162	1979	682	2496	0	2562

TRINITY_DN11259_c0_g1_i1	Glyma.12G170500.3	97.10144928	140	1	162	1979	196	2010	0	2562

TRINITY_DN15572_c0_g1_i2	Glyma.02G137100.1	95.66115702	139	19	701	2507	360	2151	0	2348

TRINITY_DN15572_c0_g1_i2	Glyma.07G208300.1	96.52509653	137	12	702	2507	208	2007	0	2405

TRINITY_DN15848_c1_g4_i8	Glyma.20G007900.3	97.97160243	124	1	247	2064	1	1815	0	2651

TRINITY_DN16197_c0_g1_i1	Glyma.06G324000.1	95.1171875	113	3	64	1872	1	1818	0	2673

TRINITY_DN15464_c0_g1_i5	Glyma.01G070300.1	98.71794872	390	37	230	2022	47	1822	0	774

TRINITY_DN15464_c0_g1_i5	Glyma.01G070300.2	98.71794872	390	37	230	2022	47	1822	0	774

TRINITY_DN15464_c0_g1_i5	Glyma.01G070300.3	98.71794872	390	37	230	2022	47	1822	0	774

TRINITY_DN16436_c0_g2_i1	Glyma.15G033000.2	96	284	21	709	2505	1	1807	0	1589

TRINITY_DN16436_c0_g2_i10	Glyma.15G033000.2	100	284	23	709	2505	1	1807	0	1589

TRINITY_DN15910_c0_g1_i5	Glyma.02G011400.1	96.55172414	274	4	593	2407	274	2088	0	1812

TRINITY_DN15910_c0_g1_i5	Glyma.02G011400.2	100	274	4	593	2407	274	2088	0	1812

TRINITY_DN13902_c0_g1_i2	Glyma.18G037700.1	95.65217391	233	2	115	1928	341	2154	0	2026

TRINITY_DN14369_c1_g1_i1	Glyma.20G133800.1	97.22222222	233	3	150	1962	81	1896	0	2032

TRINITY_DN15818_c1_g2_i4	Glyma.19G138700.1	99.513382	221	0	1	1817	1817	1	0	2132

TRINITY_DN15818_c1_g2_i4	Glyma.19G138700.2	97.22222222	221	0	1	1817	1817	1	0	2132

TRINITY_DN18917_c0_g1_i1	Glyma.04G233400.2	98.33333333	190	5	494	2303	289	2104	0	2252

TRINITY_DN18917_c0_g1_i1	Glyma.04G233400.1	98.33333333	190	5	494	2303	289	2104	0	2252

TRINITY_DN18917_c0_g1_i1	Glyma.04G233400.3	98.33333333	190	5	494	2303	289	2104	0	2252

TRINITY_DN16251_c0_g3_i10	Glyma.15G167100.1	95.41984733	190	7	548	2358	2332	519	0	2244

TRINITY_DN16251_c0_g3_i10	Glyma.15G167100.3	95.41832669	190	7	548	2358	2290	477	0	2244

TRINITY_DN14624_c0_g1_i3	Glyma.02G085500.1	100	190	13	499	2300	1839	27	0	2180

TRINITY_DN16251_c0_g3_i10	Glyma.15G167100.4	95.13888889	189	8	548	2358	2311	519	0	2115

TRINITY_DN16251_c0_g3_i10	Glyma.15G167100.5	100	188	9	548	2358	2329	519	0	2237

TRINITY_DN16251_c0_g3_i10	Glyma.15G167100.2	95.23809524	188	9	548	2358	2287	477	0	2237

TRINITY_DN14313_c2_g1_i1	Glyma.06G296500.1	100	181	10	135	1936	1811	1	0	2217

TRINITY_DN36978_c0_g1_i1	Glyma.09G023300.1	95.12195122	165	4	10	1825	311	2123	0	2409

TRINITY_DN36978_c0_g1_i1	Glyma.09G023300.2	95.12195122	165	4	10	1825	311	2123	0	2409

TRINITY_DN10790_c0_g1_i1	Glyma.17G072000.6	95.3125	161	3	248	2061	1	1814	0	2425

TRINITY_DN10790_c0_g1_i1	Glyma.17G072000.5	95.30685921	161	3	248	2061	1	1814	0	2425

TRINITY_DN10790_c0_g1_i1	Glyma.17G072000.4	95.30685921	161	3	248	2061	1	1814	0	2425

TRINITY_DN10790_c0_g1_i1	Glyma.17G072000.3	95.29411765	161	3	248	2061	1	1814	0	2425

TRINITY_DN10790_c0_g1_i1	Glyma.17G072000.2	95.29652352	161	3	248	2061	1	1814	0	2425

TRINITY_DN12154_c0_g1_i2	Glyma.02G245500.1	100	147	30	106	1902	1	1779	0	2167

TRINITY_DN38723_c0_g1_i1	Glyma.10G292800.4	99.13793103	137	5	107	1911	1	1817	0	2519

TRINITY_DN38723_c0_g1_i1	Glyma.10G292800.1	100	137	5	107	1911	1	1817	0	2519

TRINITY_DN38723_c0_g1_i1	Glyma.10G292800.3	99.33333333	137	5	107	1911	1	1817	0	2519

TRINITY_DN14243_c0_g1_i1	Glyma.19G077600.2	96.20689655	119	3	611	2421	80	1890	0	2619

TRINITY_DN14548_c0_g1_i2	Glyma.18G239900.1	100	119	6	303	2119	107	1914	0	2638

TRINITY_DN16257_c0_g1_i1	Glyma.12G192100.1	100	425	55	293	2068	2055	268	3.92E-158	560

TRINITY_DN15464_c0_g1_i5	Glyma.02G127400.1	99.4011976	386	39	230	2022	47	1822	0	808

TRINITY_DN15464_c0_g1_i5	Glyma.02G127400.2	98.71794872	386	39	230	2022	47	1822	0	808

TRINITY_DN15369_c0_g2_i4	Glyma.17G072400.1	95.23809524	383	15	18	1825	1862	55	0	1129

TRINITY_DN7482_c0_g1_i1	Glyma.02G270100.1	100	294	34	1825	3607	1804	1	0	1434

TRINITY_DN13529_c0_g1_i3	Glyma.15G121700.1	98.4375	272	35	527	2305	1	1801	0	1511

TRINITY_DN13529_c0_g1_i3	Glyma.15G121700.3	98.42767296	272	35	527	2305	1	1801	0	1511

TRINITY_DN13529_c0_g1_i3	Glyma.15G121700.2	98.4375	272	35	527	2305	1	1801	0	1511

TRINITY_DN16407_c0_g3_i2	Glyma.03G207300.2	95.34883721	223	9	742	2547	1809	1	0	2008

TRINITY_DN16407_c0_g3_i2	Glyma.03G207300.1	100	223	9	742	2547	1809	1	0	2008

TRINITY_DN16407_c0_g3_i4	Glyma.03G207300.2	96.89922481	223	9	1576	3381	1809	1	0	2008

TRINITY_DN16407_c0_g3_i4	Glyma.03G207300.1	96.89922481	223	9	1576	3381	1809	1	0	2008

TRINITY_DN16407_c0_g3_i6	Glyma.03G207300.2	100	223	9	592	2397	1809	1	0	2008

TRINITY_DN16407_c0_g3_i6	Glyma.03G207300.1	100	223	9	592	2397	1809	1	0	2008

TRINITY_DN16407_c0_g3_i8	Glyma.03G207300.2	95.14563107	223	9	1173	2978	1809	1	0	2008

TRINITY_DN16407_c0_g3_i8	Glyma.03G207300.1	96.42521167	223	9	1173	2978	1809	1	0	2008

TRINITY_DN16407_c0_g3_i7	Glyma.03G207300.2	97.21254355	220	9	591	2396	1809	1	0	2025

TRINITY_DN16407_c0_g3_i7	Glyma.03G207300.1	97.60479042	220	9	591	2396	1809	1	0	2025

TRINITY_DN16407_c0_g3_i1	Glyma.03G207300.2	95.16129032	217	7	591	2396	1809	1	0	2041

TRINITY_DN16407_c0_g3_i1	Glyma.03G207300.1	97.7443609	217	7	591	2396	1809	1	0	2041

TRINITY_DN16353_c0_g1_i6	Glyma.19G064000.1	99.36708861	174	1	91	1905	722	2537	0	2383

TRINITY_DN7256_c0_g1_i3	Glyma.01G010800.1	95.87155963	166	15	310	2103	2045	248	0	2176

TRINITY_DN16255_c1_g1_i5	Glyma.15G014000.1	100	150	4	446	2260	1812	1	0	2490

TRINITY_DN15963_c1_g1_i1	Glyma.07G074700.1	99.54545455	145	7	104	1909	1	1815	0	2479

TRINITY_DN14624_c0_g1_i1	Glyma.02G085500.1	100	145	11	499	2303	1839	27	0	2460

TRINITY_DN14027_c0_g1_i1	Glyma.10G173000.5	98.01980198	144	3	851	2666	1812	10	0	2471

TRINITY_DN15963_c1_g1_i6	Glyma.07G074700.1	97.84946237	117	7	104	1909	1	1815	0	2634

TRINITY_DN15963_c1_g1_i5	Glyma.07G074700.1	95.81151832	116	2	104	1918	1	1815	0	2699

TRINITY_DN15963_c1_g1_i4	Glyma.07G074700.1	99.01234568	88	2	104	1918	1	1815	0	2854

TRINITY_DN12830_c0_g2_i1	Glyma.08G070900.1	98.75	424	31	242	2043	159	1942	0	719

TRINITY_DN16410_c0_g1_i3	Glyma.08G326600.1	98.1981982	402	43	15	1803	2689	4477	0	789

TRINITY_DN16431_c0_g1_i4	Glyma.03G039500.1	100	312	28	2771	4549	1800	1	0	1293

TRINITY_DN16431_c0_g1_i5	Glyma.03G039500.1	95.45454545	312	28	2751	4529	1800	1	0	1293

TRINITY_DN16431_c0_g1_i6	Glyma.03G039500.1	96.52509653	312	28	2414	4192	1800	1	0	1293

TRINITY_DN16431_c0_g1_i7	Glyma.03G039500.1	100	312	28	2394	4172	1800	1	0	1293

TRINITY_DN15305_c1_g1_i1	Glyma.15G043600.1	98.79518072	179	32	1214	2973	1805	1	0	1940

TRINITY_DN15305_c1_g1_i2	Glyma.15G043600.1	98.73417722	179	32	1353	3112	1805	1	0	1940

TRINITY_DN14313_c2_g1_i1	Glyma.12G108900.1	100	169	7	135	1936	1814	1	0	2326

TRINITY_DN14547_c0_g1_i2	Glyma.09G246600.2	96.91358025	160	1	254	2068	1854	41	0	2459

TRINITY_DN14547_c0_g1_i2	Glyma.09G246600.1	96.91358025	160	1	254	2068	1854	41	0	2459

TRINITY_DN14547_c0_g1_i3	Glyma.09G246600.2	97.01492537	160	1	254	2068	1854	41	0	2459

TRINITY_DN14547_c0_g1_i3	Glyma.09G246600.1	100	160	1	254	2068	1854	41	0	2459

TRINITY_DN13519_c0_g1_i1	Glyma.03G157900.2	96.66666667	154	15	388	2169	1	1794	0	2150

TRINITY_DN16255_c1_g1_i5	Glyma.13G360000.1	100	149	8	446	2260	1803	1	0	2449

TRINITY_DN7895_c0_g1_i1	Glyma.03G091800.2	100	139	7	1	1799	245	2058	0	2471

TRINITY_DN12776_c0_g2_i1	Glyma.07G037100.2	95.45454545	139	19	289	2087	1	1790	0	2316

TRINITY_DN6539_c0_g1_i3	Glyma.05G230700.1	99.60159363	138	2	469	2282	1814	1	0	2575

TRINITY_DN6539_c0_g1_i3	Glyma.05G230700.5	99.61538462	137	4	469	2282	1802	1	0	2503

TRINITY_DN15572_c0_g1_i2	Glyma.07G208300.3	96.52406417	137	11	702	2507	208	2004	0	2418

TRINITY_DN15572_c0_g1_i2	Glyma.07G208300.2	95.66003617	137	13	702	2507	208	2001	0	2399

TRINITY_DN11433_c0_g1_i1	Glyma.08G121100.6	100	128	1	484	2298	552	2363	0	2623

TRINITY_DN11433_c0_g1_i1	Glyma.08G121100.1	99.53488372	128	1	484	2298	552	2363	0	2623

TRINITY_DN11433_c0_g1_i1	Glyma.08G121100.3	100	128	1	484	2298	372	2183	0	2623

TRINITY_DN11433_c0_g1_i1	Glyma.08G121100.5	100	128	1	484	2298	333	2144	0	2623

TRINITY_DN11433_c0_g1_i1	Glyma.08G121100.4	100	128	1	484	2298	333	2144	0	2623

TRINITY_DN11433_c0_g1_i1	Glyma.08G121100.2	100	128	1	484	2298	153	1964	0	2623

TRINITY_DN13690_c0_g1_i1	Glyma.05G175900.1	98.08306709	128	3	190	1998	1	1815	0	2604

TRINITY_DN13690_c0_g1_i1	Glyma.08G133400.1	100	126	2	190	1998	1	1815	0	2615

TRINITY_DN8988_c0_g1_i2	Glyma.15G156400.1	96.66666667	118	1	83	1894	1887	73	0	2678

TRINITY_DN8988_c0_g1_i3	Glyma.15G156400.1	96.63865546	118	1	496	2307	1887	73	0	2678

TRINITY_DN10747_c0_g1_i1	Glyma.08G027700.2	100	99	0	1	1815	914	2728	0	2804

TRINITY_DN12949_c0_g1_i1	Glyma.15G151000.4	100	411	21	362	2162	2355	555	0	905

TRINITY_DN16410_c0_g1_i2	Glyma.08G326600.1	98.82352941	403	41	15	1803	2689	4477	0	795

TRINITY_DN13566_c0_g1_i2	Glyma.14G048000.1	96.1892247	269	22	18	1812	1798	1	0	1633

TRINITY_DN16177_c0_g1_i1	Glyma.05G040800.1	97.73755656	222	10	51	1854	9	1809	0	1971

TRINITY_DN16177_c0_g1_i2	Glyma.05G040800.1	100	222	10	51	1854	9	1809	0	1971

TRINITY_DN16177_c0_g1_i3	Glyma.05G040800.1	95.65217391	222	10	51	1854	9	1809	0	1971

TRINITY_DN12154_c0_g1_i2	Glyma.14G215700.1	96.0199005	161	24	106	1902	1	1767	0	2045

TRINITY_DN12154_c0_g1_i2	Glyma.14G215700.2	96.0199005	159	28	106	1902	1	1749	0	1940

TRINITY_DN15648_c0_g2_i3	Glyma.20G234500.2	97.46835443	156	2	223	2035	3294	1482	0	2473

TRINITY_DN101_c0_g1_i1	Glyma.20G087700.1	96.72131148	126	13	496	2289	1812	1	0	2510

TRINITY_DN14244_c0_g4_i2	Glyma.13G329100.3	96.19047619	98	0	186	1999	1908	95	0	2808

TRINITY_DN15396_c0_g1_i3	Glyma.03G204800.1	99.75186104	260	23	104	1899	1894	99	0	1688

TRINITY_DN13566_c0_g1_i2	Glyma.14G048200.1	97.65625	256	17	18	1813	1795	3	0	1690

TRINITY_DN6625_c0_g2_i1	Glyma.02G154600.1	99.4011976	177	4	285	2091	1813	1	0	2329

TRINITY_DN15963_c1_g1_i1	Glyma.03G014600.1	99.18032787	149	5	104	1907	1	1813	0	2464

TRINITY_DN14294_c0_g1_i1	Glyma.13G207900.1	96.21212121	128	11	315	2120	1806	1	0	2549

TRINITY_DN12811_c0_g1_i5	Glyma.09G183900.1	97.72727273	128	16	1	1807	480	2271	0	2464

TRINITY_DN12811_c0_g1_i5	Glyma.07G092300.1	97.73462783	128	17	1	1807	477	2271	0	2484

TRINITY_DN9450_c0_g2_i3	Glyma.04G184600.1	97.2972973	123	8	1443	3227	1276	3084	0	2460

TRINITY_DN15963_c1_g1_i6	Glyma.03G014600.1	97.85002622	120	5	104	1907	1	1813	0	2625

TRINITY_DN15963_c1_g1_i5	Glyma.03G014600.1	97.77777778	118	0	104	1916	1	1813	0	2695

TRINITY_DN19670_c0_g1_i1	Glyma.20G133100.1	97.33333333	103	1	286	2098	1893	84	0	2758

TRINITY_DN15963_c1_g1_i4	Glyma.03G014600.1	96.42857143	89	0	104	1916	1	1813	0	2856

TRINITY_DN14238_c0_g2_i1	Glyma.15G066700.4	97.62931034	250	19	270	2062	72	1876	0	1794

TRINITY_DN14238_c0_g2_i1	Glyma.15G066700.2	97.61904762	250	19	270	2062	72	1876	0	1794

TRINITY_DN13471_c0_g2_i1	Glyma.16G022600.3	97.89473684	225	6	205	2013	1798	2	0	1984

TRINITY_DN13471_c0_g2_i1	Glyma.16G022600.2	95.16806723	225	7	205	2013	1795	2	0	1964

TRINITY_DN38654_c0_g1_i1	Glyma.11G235800.1	98.88888889	224	4	20	1827	1837	30	0	2054

TRINITY_DN9899_c0_g1_i4	Glyma.19G254200.1	100	223	31	13	1795	472	2254	0	1736

TRINITY_DN13868_c0_g1_i1	Glyma.13G176900.1	98.6013986	221	9	258	2060	73	1878	0	2025

TRINITY_DN13868_c0_g1_i3	Glyma.13G176900.1	96.47058824	221	9	258	2060	73	1878	0	2025

TRINITY_DN13868_c0_g1_i6	Glyma.13G176900.1	99.0881459	221	9	258	2060	73	1878	0	2025

TRINITY_DN13868_c0_g1_i8	Glyma.13G176900.1	95.77464789	221	9	258	2060	73	1878	0	2025

TRINITY_DN11617_c0_g1_i1	Glyma.04G074900.2	97.37609329	195	17	200	1986	23	1806	0	1923

TRINITY_DN10786_c0_g1_i1	Glyma.13G123700.1	100	184	2	2218	4028	1811	1	0	2314

TRINITY_DN36978_c0_g1_i1	Glyma.15G129400.2	96.25	163	8	21	1825	331	2135	0	2353

TRINITY_DN36978_c0_g1_i1	Glyma.15G129400.1	95.08196721	163	8	21	1825	331	2135	0	2353

TRINITY_DN14204_c0_g1_i1	Glyma.02G057400.1	100	146	1	249	2060	117	1904	0	2383

TRINITY_DN16194_c2_g1_i1	Glyma.19G232500.1	100	146	3	209	2014	1	1812	0	2499

TRINITY_DN16194_c2_g1_i5	Glyma.19G232500.1	100	146	3	209	2014	1	1812	0	2499

TRINITY_DN16194_c2_g1_i2	Glyma.19G232500.1	98.05194805	146	7	209	2003	1	1812	0	2427

TRINITY_DN16194_c2_g1_i3	Glyma.19G232500.1	100	145	1	209	2014	1	1812	0	2505

TRINITY_DN16194_c2_g1_i6	Glyma.19G232500.1	97.39130435	145	1	209	2014	1	1812	0	2505

TRINITY_DN14450_c1_g1_i2	Glyma.11G110800.6	100	129	9	330	2128	2663	868	0	2444

TRINITY_DN14450_c1_g1_i2	Glyma.11G110800.4	100	129	9	330	2128	2663	868	0	2444

TRINITY_DN14450_c1_g1_i2	Glyma.11G110800.2	100	129	9	330	2128	2663	868	0	2444

TRINITY_DN1309_c0_g1_i1	Glyma.13G026700.3	98.51851852	124	13	125	1905	1	1803	0	2401

TRINITY_DN14204_c0_g1_i2	Glyma.02G057400.1	100	123	1	249	2060	117	1904	0	2510

TRINITY_DN14823_c0_g1_i4	Glyma.16G096500.1	95.5801105	118	9	389	2191	1806	1	0	2595

TRINITY_DN9060_c0_g1_i1	Glyma.05G200600.1	97.56097561	113	0	219	2030	1812	1	0	2721

TRINITY_DN13529_c0_g1_i3	Glyma.07G269100.1	100	108	25	527	2311	1	1779	0	2361

TRINITY_DN15539_c0_g1_i1	Glyma.17G259200.1	100	378	31	804	2591	595	2385	0	972

TRINITY_DN14604_c0_g1_i5	Glyma.16G116000.2	95.65217391	313	10	701	2505	1	1805	0	1533

TRINITY_DN13529_c0_g1_i3	Glyma.09G015900.1	98.43342037	275	21	527	2314	1	1798	0	1589

TRINITY_DN13529_c0_g1_i3	Glyma.09G015900.3	98.44617092	275	21	527	2314	1	1798	0	1589

TRINITY_DN13529_c0_g1_i3	Glyma.09G015900.2	98.47328244	275	21	527	2314	1	1798	0	1589

TRINITY_DN13529_c0_g1_i3	Glyma.09G015900.4	98.4375	275	21	527	2314	1	1798	0	1589

TRINITY_DN6769_c0_g1_i3	Glyma.15G160700.1	96.55172414	272	34	252	2039	68	1835	0	1411

TRINITY_DN15888_c0_g3_i2	Glyma.14G048300.1	98.75	263	31	833	2628	1477	3257	0	1596

TRINITY_DN38654_c0_g1_i1	Glyma.18G021100.1	98.87640449	206	4	20	1827	1870	66	0	2145

TRINITY_DN5722_c0_g2_i1	Glyma.14G219000.1	96.49122807	185	16	1512	3313	1906	136	0	2002

TRINITY_DN5722_c0_g2_i1	Glyma.14G219000.2	98.18181818	185	16	1512	3313	1906	136	0	2002

TRINITY_DN5722_c0_g2_i1	Glyma.14G219000.4	96.2962963	185	16	1512	3313	1906	136	0	2002

TRINITY_DN5722_c0_g2_i1	Glyma.14G219000.3	98.48484848	184	17	1512	3313	1903	136	0	1989

TRINITY_DN135_c0_g2_i1	Glyma.08G004200.1	100	141	2	178	1987	39	1848	0	2551

TRINITY_DN14548_c0_g1_i2	Glyma.09G252800.1	100	119	6	315	2125	101	1902	0	2627

TRINITY_DN12290_c0_g1_i1	Glyma.19G140000.1	100	383	23	1825	3615	1944	145	0	1033

TRINITY_DN10335_c1_g1_i1	Glyma.10G023400.1	100	298	31	4	1781	19	1814	0	1395

TRINITY_DN15510_c0_g1_i5	Glyma.16G218100.1	99.08256881	266	23	234	2032	185	1973	0	1663

TRINITY_DN15510_c0_g1_i5	Glyma.16G218100.3	98.92473118	266	23	234	2032	185	1973	0	1663

TRINITY_DN15910_c0_g1_i4	Glyma.19G217300.1	99.07407407	258	12	518	2319	362	2166	0	1829

TRINITY_DN15910_c0_g1_i4	Glyma.03G220500.1	95.65217391	254	10	517	2319	415	2220	0	1864

TRINITY_DN15910_c0_g1_i4	Glyma.03G220500.3	100	254	10	517	2319	358	2163	0	1864

TRINITY_DN15910_c0_g1_i4	Glyma.03G220500.2	100	254	10	517	2319	283	2088	0	1864

TRINITY_DN16407_c0_g3_i2	Glyma.19G204800.2	95.34883721	222	7	742	2547	1803	1	0	2041

TRINITY_DN16407_c0_g3_i2	Glyma.19G204800.1	95.34883721	222	7	742	2547	1803	1	0	2041

TRINITY_DN16407_c0_g3_i4	Glyma.19G204800.2	96.96969697	222	7	1576	3381	1803	1	0	2041

TRINITY_DN16407_c0_g3_i4	Glyma.19G204800.1	95.87628866	222	7	1576	3381	1803	1	0	2041

TRINITY_DN16407_c0_g3_i6	Glyma.19G204800.2	100	222	7	592	2397	1803	1	0	2041

TRINITY_DN16407_c0_g3_i6	Glyma.19G204800.1	100	222	7	592	2397	1803	1	0	2041

TRINITY_DN16407_c0_g3_i8	Glyma.19G204800.2	96.06557377	222	7	1173	2978	1803	1	0	2041

TRINITY_DN16407_c0_g3_i8	Glyma.19G204800.1	100	222	7	1173	2978	1803	1	0	2041

TRINITY_DN16407_c0_g3_i1	Glyma.19G204800.2	95.16129032	221	6	591	2396	1803	1	0	2047

TRINITY_DN16407_c0_g3_i1	Glyma.19G204800.1	95.16129032	221	6	591	2396	1803	1	0	2047

TRINITY_DN16407_c0_g3_i7	Glyma.19G204800.2	100	219	7	591	2396	1803	1	0	2058

TRINITY_DN16407_c0_g3_i7	Glyma.19G204800.1	98.00995025	219	7	591	2396	1803	1	0	2058

TRINITY_DN13529_c0_g1_i2	Glyma.15G121700.1	100	208	27	527	2302	1	1801	0	1912

TRINITY_DN13529_c0_g1_i2	Glyma.15G121700.3	100	208	27	527	2302	1	1801	0	1912

TRINITY_DN13529_c0_g1_i2	Glyma.15G121700.2	98.42519685	208	27	527	2302	1	1801	0	1912

TRINITY_DN16257_c1_g1_i2	Glyma.12G192100.1	100	196	14	81	1882	2241	443	0	2134

TRINITY_DN5722_c0_g1_i1	Glyma.14G219000.1	99.2248062	185	15	1512	3312	1906	136	0	2008

TRINITY_DN5722_c0_g1_i1	Glyma.14G219000.2	96.35258359	185	15	1512	3312	1906	136	0	2008

TRINITY_DN5722_c0_g1_i1	Glyma.14G219000.4	96.35416667	185	15	1512	3312	1906	136	0	2008

TRINITY_DN5722_c0_g1_i1	Glyma.14G219000.3	96.35416667	184	16	1512	3312	1903	136	0	1993

TRINITY_DN12739_c0_g1_i1	Glyma.13G220900.1	99.43502825	153	11	308	2099	1	1801	0	2320

TRINITY_DN14146_c1_g1_i2	Glyma.20G228700.3	98.41427156	144	11	1	1810	1792	1	0	2429

TRINITY_DN14146_c1_g1_i2	Glyma.20G228700.2	97.95918367	144	11	1	1810	1792	1	0	2429

TRINITY_DN14146_c1_g1_i2	Glyma.20G228700.1	98.9010989	144	11	1	1810	1792	1	0	2429

TRINITY_DN14146_c1_g1_i2	Glyma.20G228700.4	96.21621622	144	11	1	1810	1792	1	0	2429

TRINITY_DN15970_c0_g2_i1	Glyma.01G099800.3	96.875	120	0	246	2055	23	1832	0	2678

TRINITY_DN15970_c0_g2_i9	Glyma.01G099800.3	95.37572254	120	0	246	2055	23	1832	0	2678

TRINITY_DN13596_c0_g1_i2	Glyma.17G083700.1	96.59090909	100	0	62	1871	1	1810	0	2789

TRINITY_DN13596_c0_g1_i1	Glyma.17G083700.1	96.61016949	100	7	62	1847	1	1810	0	2634

TRINITY_DN15464_c0_g1_i4	Glyma.01G070300.1	95.23809524	392	37	230	2014	47	1822	0	800

TRINITY_DN15464_c0_g1_i4	Glyma.01G070300.2	100	392	37	230	2014	47	1822	0	800

TRINITY_DN15464_c0_g1_i4	Glyma.01G070300.3	96	392	37	230	2014	47	1822	0	800

TRINITY_DN13723_c0_g1_i1	Glyma.16G074700.3	98.1981982	284	24	187	1980	1	1797	0	1592

TRINITY_DN13723_c0_g1_i1	Glyma.16G074700.1	95.23809524	284	24	187	1980	1	1797	0	1592

TRINITY_DN13723_c0_g1_i1	Glyma.16G074700.2	97.00598802	284	24	187	1980	1	1797	0	1592

TRINITY_DN13723_c0_g1_i4	Glyma.16G074700.3	98.02631579	284	24	569	2362	1	1797	0	1592

TRINITY_DN13723_c0_g1_i4	Glyma.16G074700.1	95.28301887	284	24	569	2362	1	1797	0	1592

TRINITY_DN13723_c0_g1_i4	Glyma.16G074700.2	97.61904762	284	24	569	2362	1	1797	0	1592

TRINITY_DN3545_c0_g2_i1	Glyma.13G137000.1	95	264	15	17	1795	535	2337	0	1646

TRINITY_DN11607_c0_g2_i1	Glyma.05G195500.1	100	257	8	124	1930	1819	19	0	1853

TRINITY_DN15175_c0_g1_i3	Glyma.01G006100.1	98.07280514	257	10	80	1883	151	1954	0	1853

TRINITY_DN14759_c0_g1_i7	Glyma.01G097400.3	100	247	9	293	2091	1881	77	0	1882

TRINITY_DN14759_c0_g1_i7	Glyma.01G097400.1	100	247	9	293	2091	1881	77	0	1882

TRINITY_DN14759_c0_g1_i7	Glyma.01G097400.2	100	247	9	293	2091	1881	77	0	1882

TRINITY_DN15772_c0_g1_i5	Glyma.19G039100.2	98.39572193	224	3	122	1926	1	1808	0	2067

TRINITY_DN16257_c1_g1_i6	Glyma.12G192100.1	97.75280899	212	12	103	1904	2241	443	0	2056

TRINITY_DN16022_c0_g2_i2	Glyma.01G118400.1	99.08256881	188	14	1230	3026	79	1872	0	2124

TRINITY_DN16022_c0_g2_i2	Glyma.01G118400.2	100	188	14	1230	3026	16	1809	0	2124

TRINITY_DN13529_c0_g1_i2	Glyma.07G269100.1	100	184	25	527	2308	1	1779	0	1953

TRINITY_DN10035_c0_g1_i1	Glyma.09G037300.1	96.42857143	166	4	2590	4389	3565	5373	0	2362

TRINITY_DN10035_c0_g1_i1	Glyma.09G037300.2	95.74468085	166	4	2590	4389	3565	5373	0	2362

TRINITY_DN10790_c0_g1_i1	Glyma.17G072000.1	100	161	3	248	2053	1	1806	0	2410

TRINITY_DN10790_c0_g1_i1	Glyma.17G072000.7	100	161	3	248	2053	1	1806	0	2410

TRINITY_DN13878_c0_g1_i2	Glyma.04G188800.1	97.01492537	157	24	693	2486	1779	1	0	2180

TRINITY_DN14621_c1_g1_i4	Glyma.05G072300.2	96.2962963	152	3	122	1924	1	1803	0	2422

TRINITY_DN9851_c0_g1_i1	Glyma.18G041000.2	100	151	0	1	1809	907	2715	0	2505

TRINITY_DN9851_c0_g1_i2	Glyma.18G041000.2	98.57142857	151	0	1	1809	907	2715	0	2505

TRINITY_DN5720_c0_g1_i1	Glyma.20G189900.3	100	139	0	350	2158	1809	1	0	2571

TRINITY_DN14450_c1_g1_i2	Glyma.12G037100.2	100	122	9	330	2128	2654	865	0	2477

TRINITY_DN14450_c1_g1_i2	Glyma.12G037100.4	96	122	9	330	2128	2654	865	0	2477

TRINITY_DN14450_c1_g1_i2	Glyma.12G037100.3	100	122	10	330	2128	2651	865	0	2459

TRINITY_DN14621_c1_g1_i2	Glyma.05G072300.2	100	112	3	122	1924	1	1803	0	2643

TRINITY_DN16197_c0_g1_i1	Glyma.04G255700.1	97.91666667	110	1	64	1872	1	1803	0	2693

TRINITY_DN16197_c0_g1_i1	Glyma.04G255700.2	100	108	3	64	1872	1	1797	0	2665

TRINITY_DN14337_c0_g2_i2	Glyma.13G156600.2	100	105	8	607	2394	1809	1	0	2623

TRINITY_DN14337_c0_g2_i2	Glyma.13G156600.3	95.32258065	105	8	607	2394	1809	1	0	2623

TRINITY_DN14337_c0_g2_i2	Glyma.13G156600.1	100	105	8	607	2394	1809	1	0	2623

TRINITY_DN14323_c0_g1_i3	Glyma.11G085100.1	100	105	16	380	2164	1803	1	0	2566

TRINITY_DN5219_c0_g2_i1	Glyma.15G255300.1	100	96	0	419	2227	1809	1	0	2809

TRINITY_DN5219_c0_g2_i1	Glyma.08G171900.1	96.2406015	94	0	419	2227	1809	1	0	2820

TRINITY_DN10975_c0_g1_i1	Glyma.08G225500.2	95.51820728	81	0	1043	2851	598	2406	0	2892

TRINITY_DN10975_c0_g1_i1	Glyma.08G225500.1	96.72131148	81	0	1043	2851	598	2406	0	2892

TRINITY_DN10975_c0_g1_i1	Glyma.07G001500.2	95.0617284	77	0	1043	2851	598	2406	0	2915

TRINITY_DN10975_c0_g1_i1	Glyma.07G001500.1	100	77	0	1043	2851	598	2406	0	2915

TRINITY_DN13713_c0_g1_i1	Glyma.12G074700.4	95.29411765	63	3	473	2281	1	1800	0	2933

TRINITY_DN13713_c0_g1_i2	Glyma.12G074700.4	99.11504425	63	3	446	2254	1	1800	0	2933

TRINITY_DN13713_c0_g1_i3	Glyma.12G074700.4	98.16513761	63	3	190	1998	1	1800	0	2933

TRINITY_DN13713_c0_g1_i4	Glyma.12G074700.4	99.26470588	63	3	434	2242	1	1800	0	2933

TRINITY_DN13713_c0_g1_i6	Glyma.12G074700.4	100	61	5	473	2264	1	1800	0	2835

TRINITY_DN15464_c0_g1_i4	Glyma.02G127400.1	100	389	36	230	2014	47	1822	0	828

TRINITY_DN15464_c0_g1_i4	Glyma.02G127400.2	98.63013699	389	36	230	2014	47	1822	0	828

TRINITY_DN15539_c0_g1_i1	Glyma.14G220300.1	95.29411765	383	31	804	2591	595	2382	0	959

TRINITY_DN3545_c0_g1_i1	Glyma.13G137000.1	100	266	13	17	1795	535	2337	0	1646

TRINITY_DN16257_c1_g1_i6	Glyma.13G310300.1	98.5840708	252	16	102	1902	2245	451	0	1814

TRINITY_DN16257_c1_g1_i2	Glyma.13G310300.1	100	250	16	80	1880	2245	451	0	1825

TRINITY_DN16257_c1_g1_i6	Glyma.12G192000.1	95.0310559	232	14	102	1904	2239	443	0	1951

TRINITY_DN15924_c0_g1_i3	Glyma.01G046700.2	96.2962963	228	11	388	2190	1800	1	0	1991

TRINITY_DN15924_c0_g1_i3	Glyma.01G046700.1	100	228	11	388	2190	1800	1	0	1991

TRINITY_DN15924_c0_g1_i3	Glyma.02G106900.1	100	226	11	388	2190	1800	1	0	2002

TRINITY_DN16257_c1_g1_i2	Glyma.12G192000.1	100	226	14	80	1882	2239	443	0	1984

TRINITY_DN15186_c0_g1_i3	Glyma.17G211700.1	98.83040936	213	3	395	2198	1820	14	0	2126

TRINITY_DN12569_c0_g1_i3	Glyma.15G061700.3	99.12280702	209	23	438	2214	116	1916	0	1936

TRINITY_DN12569_c0_g1_i6	Glyma.15G061700.3	100	209	23	352	2128	116	1916	0	1936

TRINITY_DN36827_c0_g1_i1	Glyma.16G017300.1	100	161	3	295	2099	2088	290	0	2370

TRINITY_DN11772_c0_g1_i2	Glyma.08G298500.1	96.34408602	138	10	142	1931	2	1809	0	2459

TRINITY_DN11772_c0_g1_i4	Glyma.08G298500.1	100	138	11	142	1905	2	1809	0	2290

TRINITY_DN12479_c0_g1_i7	Glyma.05G041000.1	100	133	8	271	2075	1790	1	0	2466

TRINITY_DN12299_c0_g1_i1	Glyma.07G099400.2	99.31506849	121	3	727	2530	1807	1	0	2636

TRINITY_DN12299_c0_g1_i2	Glyma.07G099400.2	99.31506849	121	3	727	2530	1807	1	0	2636

TRINITY_DN15912_c1_g1_i2	Glyma.15G016500.2	96.41255605	111	6	862	2669	1784	1	0	2569

TRINITY_DN15850_c3_g2_i5	Glyma.18G054100.1	99.3485342	331	14	710	2506	22	1818	0	1375

TRINITY_DN15164_c0_g2_i6	Glyma.01G228900.1	98.79518072	183	9	235	2040	1	1788	0	2194

TRINITY_DN2451_c0_g1_i1	Glyma.01G077200.3	99	182	14	1135	2928	4440	2656	0	2102

TRINITY_DN15164_c0_g2_i6	Glyma.11G011700.1	98.79518072	181	11	235	2040	1	1788	0	2206

TRINITY_DN12692_c0_g1_i1	Glyma.05G160000.1	100	177	9	745	2548	3686	1889	0	2279

TRINITY_DN35786_c0_g1_i1	Glyma.19G220100.1	97.5	160	9	1	1806	2372	4159	0	2322

TRINITY_DN12797_c0_g1_i1	Glyma.02G261800.3	97.08994709	152	9	289	2091	3933	2134	0	2423

TRINITY_DN12797_c0_g1_i1	Glyma.02G261800.2	97.09090909	152	9	289	2091	3933	2134	0	2423

TRINITY_DN12797_c0_g1_i1	Glyma.02G261800.1	99.21259843	152	9	289	2091	3933	2134	0	2423

TRINITY_DN6312_c0_g1_i1	Glyma.20G189200.4	97.36842105	148	18	390	2174	1803	1	0	2350

TRINITY_DN6312_c0_g1_i1	Glyma.20G189200.3	97.36842105	148	18	390	2174	1803	1	0	2350

TRINITY_DN6312_c0_g1_i1	Glyma.20G189200.1	97.36842105	148	18	390	2174	1803	1	0	2350

TRINITY_DN6312_c0_g1_i1	Glyma.20G189200.2	96.72131148	148	18	390	2174	1803	1	0	2350

TRINITY_DN6312_c0_g1_i2	Glyma.20G189200.4	99.36305732	148	18	390	2174	1803	1	0	2350

TRINITY_DN6312_c0_g1_i2	Glyma.20G189200.3	99.36305732	148	18	390	2174	1803	1	0	2350

TRINITY_DN6312_c0_g1_i2	Glyma.20G189200.1	100	148	18	390	2174	1803	1	0	2350

TRINITY_DN6312_c0_g1_i2	Glyma.20G189200.2	100	148	18	390	2174	1803	1	0	2350

TRINITY_DN3027_c0_g1_i1	Glyma.10G179900.1	97.64705882	131	5	34	1827	55	1860	0	2521

TRINITY_DN16407_c0_g3_i2	Glyma.09G105600.1	95	120	3	742	2547	1803	1	0	2639

TRINITY_DN16407_c0_g3_i2	Glyma.09G105600.2	95.23809524	120	3	742	2547	1803	1	0	2639

TRINITY_DN16407_c0_g3_i4	Glyma.09G105600.1	97.36842105	120	3	1576	3381	1803	1	0	2639

TRINITY_DN16407_c0_g3_i4	Glyma.09G105600.2	97.36842105	120	3	1576	3381	1803	1	0	2639

TRINITY_DN16407_c0_g3_i6	Glyma.09G105600.1	100	120	3	592	2397	1803	1	0	2639

TRINITY_DN16407_c0_g3_i6	Glyma.09G105600.2	100	120	3	592	2397	1803	1	0	2639

TRINITY_DN16407_c0_g3_i8	Glyma.09G105600.1	96.06440072	120	3	1173	2978	1803	1	0	2639

TRINITY_DN16407_c0_g3_i8	Glyma.09G105600.2	96.06481481	120	3	1173	2978	1803	1	0	2639

TRINITY_DN16407_c0_g3_i1	Glyma.09G105600.1	95.16129032	117	3	591	2396	1803	1	0	2656

TRINITY_DN16407_c0_g3_i1	Glyma.09G105600.2	95.16129032	117	3	591	2396	1803	1	0	2656

TRINITY_DN16407_c0_g3_i7	Glyma.09G105600.1	95.23809524	110	3	591	2396	1803	1	0	2695

TRINITY_DN16407_c0_g3_i7	Glyma.09G105600.2	98.68421053	110	3	591	2396	1803	1	0	2695

TRINITY_DN13723_c0_g1_i1	Glyma.16G105300.1	100	287	18	187	1980	1	1797	0	1609

TRINITY_DN13723_c0_g1_i1	Glyma.16G105300.2	98.1981982	287	18	187	1980	1	1797	0	1609

TRINITY_DN13723_c0_g1_i4	Glyma.16G105300.1	95	287	18	569	2362	1	1797	0	1609

TRINITY_DN13723_c0_g1_i4	Glyma.16G105300.2	99.27007299	287	18	569	2362	1	1797	0	1609

TRINITY_DN13868_c0_g1_i10	Glyma.07G199500.1	100	275	7	258	2060	73	1872	0	1753

TRINITY_DN13868_c0_g1_i4	Glyma.07G199500.1	98.79032258	275	7	258	2060	73	1872	0	1753

TRINITY_DN13868_c0_g1_i5	Glyma.07G199500.1	100	275	7	258	2060	73	1872	0	1753

TRINITY_DN13868_c0_g1_i9	Glyma.07G199500.1	100	275	7	258	2060	73	1872	0	1753

TRINITY_DN16257_c1_g1_i6	Glyma.12G191800.1	98.58429858	262	10	102	1904	2239	443	0	1807

TRINITY_DN16415_c1_g1_i13	Glyma.12G173700.1	98.87640449	259	5	2357	4157	1866	63	0	1855

TRINITY_DN16415_c1_g1_i6	Glyma.12G173700.1	98.38709677	259	5	2263	4063	1866	63	0	1855

TRINITY_DN16257_c1_g1_i2	Glyma.12G191800.1	96.51162791	259	10	80	1882	2239	443	0	1823

TRINITY_DN13868_c0_g1_i1	Glyma.07G199500.1	95	235	7	258	2060	73	1872	0	1975

TRINITY_DN13868_c0_g1_i3	Glyma.07G199500.1	96.47058824	235	7	258	2060	73	1872	0	1975

TRINITY_DN13868_c0_g1_i6	Glyma.07G199500.1	99.09090909	235	7	258	2060	73	1872	0	1975

TRINITY_DN13868_c0_g1_i8	Glyma.07G199500.1	95.91836735	235	7	258	2060	73	1872	0	1975

TRINITY_DN6312_c0_g1_i1	Glyma.10G201200.2	100	160	12	390	2174	1803	1	0	2294

TRINITY_DN6312_c0_g1_i1	Glyma.10G201200.1	99.36305732	160	12	390	2174	1803	1	0	2294

TRINITY_DN6312_c0_g1_i2	Glyma.10G201200.2	99.11504425	160	12	390	2174	1803	1	0	2294

TRINITY_DN6312_c0_g1_i2	Glyma.10G201200.1	100	160	12	390	2174	1803	1	0	2294

TRINITY_DN12797_c0_g1_i1	Glyma.U027200.1	97.08879185	151	7	289	2091	3924	2125	0	2440

TRINITY_DN12797_c0_g1_i1	Glyma.U027200.2	97.08994709	151	7	289	2091	3924	2125	0	2440

TRINITY_DN11256_c0_g1_i1	Glyma.18G028700.1	97.33333333	151	11	447	2234	1	1806	0	2383

TRINITY_DN14057_c0_g1_i3	Glyma.07G212300.1	98.29059829	148	25	128	1923	56	1824	0	2211

TRINITY_DN10216_c0_g1_i1	Glyma.19G226700.1	97.29344729	141	10	81	1880	1815	25	0	2418

TRINITY_DN16410_c0_g1_i3	Glyma.08G326200.1	100	137	2	7	1811	2678	4482	0	2564

TRINITY_DN9376_c0_g1_i1	Glyma.02G010300.6	100	136	1	531	2335	1806	1	0	2575

TRINITY_DN9376_c0_g1_i2	Glyma.02G010300.6	100	136	1	531	2335	1806	1	0	2575

TRINITY_DN16410_c0_g1_i2	Glyma.08G326200.1	95.96774194	136	2	7	1811	2678	4482	0	2569

TRINITY_DN16410_c0_g1_i3	Glyma.18G080900.3	99.04761905	131	0	6	1811	2101	3906	0	2610

TRINITY_DN16410_c0_g1_i3	Glyma.18G080900.1	99.04761905	131	0	6	1811	2101	3906	0	2610

TRINITY_DN16410_c0_g1_i3	Glyma.18G080900.4	97.24770642	131	0	6	1811	1564	3369	0	2610

TRINITY_DN16410_c0_g1_i2	Glyma.18G080900.3	95.96774194	130	0	6	1811	2101	3906	0	2615

TRINITY_DN16410_c0_g1_i2	Glyma.18G080900.1	95.96774194	130	0	6	1811	2101	3906	0	2615

TRINITY_DN16410_c0_g1_i2	Glyma.18G080900.4	95.96774194	130	0	6	1811	1564	3369	0	2615

TRINITY_DN12923_c0_g1_i1	Glyma.20G001000.1	98.68421053	129	0	172	1977	1	1806	0	2621

TRINITY_DN5651_c0_g1_i1	Glyma.06G308700.3	98.33333333	128	0	1	1806	479	2284	0	2627

TRINITY_DN12923_c0_g1_i1	Glyma.09G285100.1	99.0920882	128	0	172	1977	1	1806	0	2627

TRINITY_DN15924_c0_g1_i3	Glyma.08G300700.2	100	122	0	385	2190	1806	1	0	2660

TRINITY_DN15924_c0_g1_i3	Glyma.08G300700.1	95.53264605	122	0	385	2190	1806	1	0	2660

TRINITY_DN15924_c0_g1_i3	Glyma.08G300700.3	97.45762712	122	0	385	2190	1806	1	0	2660

TRINITY_DN15970_c0_g2_i1	Glyma.01G099800.4	96	119	0	250	2055	3	1808	0	2676

TRINITY_DN15970_c0_g2_i9	Glyma.01G099800.4	96.40102828	119	0	250	2055	3	1808	0	2676

TRINITY_DN15924_c0_g1_i3	Glyma.18G119600.3	95	115	0	385	2190	1806	1	0	2699

TRINITY_DN15924_c0_g1_i3	Glyma.18G119600.2	96.22641509	115	0	385	2190	1806	1	0	2699

TRINITY_DN15924_c0_g1_i3	Glyma.18G119600.1	95.98853868	115	0	385	2190	1806	1	0	2699

TRINITY_DN15897_c0_g2_i1	Glyma.14G029100.1	100	115	12	155	1947	3192	1414	0	2440

TRINITY_DN15019_c0_g1_i4	Glyma.18G284600.1	95.77464789	109	18	485	2275	1804	8	0	2577

TRINITY_DN15164_c0_g2_i6	Glyma.11G092200.4	95.08196721	106	0	235	2040	1	1806	0	2748

TRINITY_DN15164_c0_g2_i6	Glyma.11G092200.5	96.96969697	106	0	235	2040	1	1806	0	2748

TRINITY_DN15164_c0_g2_i6	Glyma.11G092200.1	96.43493761	106	0	235	2040	1	1806	0	2748

TRINITY_DN15174_c0_g1_i1	Glyma.07G222200.1	95.34883721	90	2	313	2118	1	1803	0	2817

TRINITY_DN15174_c0_g1_i1	Glyma.07G222200.3	100	90	2	313	2118	1	1803	0	2817

TRINITY_DN16323_c0_g2_i1	Glyma.09G243700.2	100	315	16	855	2650	1820	28	0	1452

TRINITY_DN13529_c0_g1_i2	Glyma.09G015900.1	98.82352941	205	13	527	2311	1	1798	0	2023

TRINITY_DN13529_c0_g1_i2	Glyma.09G015900.3	98.79518072	205	13	527	2311	1	1798	0	2023

TRINITY_DN13529_c0_g1_i2	Glyma.09G015900.2	98.82352941	205	13	527	2311	1	1798	0	2023

TRINITY_DN13529_c0_g1_i2	Glyma.09G015900.4	98.8372093	205	13	527	2311	1	1798	0	2023

TRINITY_DN10695_c0_g1_i1	Glyma.20G054500.1	97.87234043	179	14	136	1931	61	1856	0	2226

TRINITY_DN14364_c0_g2_i2	Glyma.15G143400.1	96.80365297	175	3	1432	3235	1824	24	0	2331

TRINITY_DN9156_c0_g1_i2	Glyma.18G109800.2	99.23076923	169	2	63	1866	1	1804	0	2385

TRINITY_DN10177_c0_g1_i1	Glyma.07G177100.1	98.13084112	157	12	121	1898	1805	7	0	2250

TRINITY_DN41362_c0_g1_i1	Glyma.08G225900.1	100	156	12	2	1778	1811	11	0	2263

TRINITY_DN16151_c0_g2_i2	Glyma.01G038300.1	95.38461538	147	8	1534	3323	1321	3122	0	2403

TRINITY_DN16151_c0_g2_i6	Glyma.01G038300.1	97.36842105	147	8	1273	3062	1321	3122	0	2403

TRINITY_DN16189_c1_g3_i1	Glyma.05G201100.1	95.45454545	135	4	2	1804	958	2760	0	2560

TRINITY_DN19533_c0_g1_i1	Glyma.11G009000.2	96.83257919	122	2	2	1803	2890	1087	0	2632

TRINITY_DN19533_c0_g1_i1	Glyma.11G009000.1	100	122	2	2	1803	2890	1087	0	2632

TRINITY_DN16189_c1_g3_i1	Glyma.06G165200.2	100	117	2	1	1804	966	2769	0	2673

TRINITY_DN16189_c1_g3_i1	Glyma.06G165200.1	98.96551724	117	2	1	1804	966	2769	0	2673

TRINITY_DN15164_c0_g2_i6	Glyma.12G018500.2	98.11827957	112	0	235	2039	1	1805	0	2713

TRINITY_DN15164_c0_g2_i6	Glyma.12G018500.3	98.11827957	112	0	235	2039	1	1805	0	2713

TRINITY_DN15164_c0_g2_i6	Glyma.12G018500.1	98.11912226	112	0	235	2039	1	1805	0	2713

TRINITY_DN13991_c0_g1_i1	Glyma.13G111500.2	100	317	19	133	1931	1812	38	0	1356

TRINITY_DN13991_c0_g1_i3	Glyma.13G111500.2	100	317	19	133	1931	1812	38	0	1356

TRINITY_DN15396_c0_g1_i3	Glyma.19G202200.1	99.80916031	268	16	104	1899	1882	93	0	1705

TRINITY_DN12184_c0_g1_i1	Glyma.17G071600.1	97.16981132	242	9	1	1798	1056	2856	0	1932

TRINITY_DN16189_c1_g3_i2	Glyma.05G201100.1	98.34710744	181	2	2	1804	958	2760	0	2316

TRINITY_DN11337_c0_g1_i1	Glyma.13G353200.7	95.6097561	179	17	215	2001	1787	1	0	2121

TRINITY_DN11337_c0_g1_i1	Glyma.13G353200.4	100	179	17	215	2001	1787	1	0	2121

TRINITY_DN14364_c0_g2_i2	Glyma.09G038300.2	97.84946237	176	3	1433	3235	1826	27	0	2324

TRINITY_DN14364_c0_g2_i2	Glyma.09G038300.1	97.24770642	176	3	1433	3235	1826	27	0	2324

TRINITY_DN35745_c0_g1_i1	Glyma.16G041800.1	99.53488372	173	29	150	1925	1773	1	0	1991

TRINITY_DN5833_c0_g1_i1	Glyma.17G084400.1	98.92473118	168	4	344	2140	1862	60	0	2350

TRINITY_DN15418_c0_g2_i1	Glyma.08G095200.1	95.52238806	156	10	123	1910	1	1800	0	2338

TRINITY_DN16386_c0_g1_i4	Glyma.16G198200.1	98.26589595	152	7	1	1796	812	2610	0	2405

TRINITY_DN15910_c0_g1_i4	Glyma.02G011400.1	95.3271028	144	0	516	2319	285	2088	0	2534

TRINITY_DN15910_c0_g1_i4	Glyma.02G011400.2	95.3271028	144	0	516	2319	285	2088	0	2534

TRINITY_DN15910_c0_g1_i4	Glyma.10G012000.1	96	135	0	516	2319	291	2094	0	2584

TRINITY_DN16189_c1_g3_i1	Glyma.04G200400.1	98.96640827	124	0	1	1804	966	2769	0	2645

TRINITY_DN16189_c1_g3_i1	Glyma.04G200400.2	100	124	0	1	1804	966	2769	0	2645

TRINITY_DN35728_c0_g1_i1	Glyma.09G063000.1	96.90721649	111	45	185	1885	1803	1	0	2045

TRINITY_DN15382_c0_g1_i1	Glyma.04G133800.1	100	98	1	91	1888	1	1804	0	2750

TRINITY_DN15382_c0_g1_i2	Glyma.04G133800.1	98.33333333	98	1	91	1888	1	1804	0	2750

TRINITY_DN16189_c1_g3_i2	Glyma.04G200400.1	96.37681159	91	0	1	1804	966	2769	0	2828

TRINITY_DN16189_c1_g3_i2	Glyma.04G200400.2	98.50746269	91	0	1	1804	966	2769	0	2828

TRINITY_DN16189_c1_g3_i2	Glyma.06G165200.2	98.57549858	89	0	1	1804	966	2769	0	2839

TRINITY_DN16189_c1_g3_i2	Glyma.06G165200.1	96.37681159	89	0	1	1804	966	2769	0	2839

TRINITY_DN13967_c3_g1_i1	Glyma.07G046000.1	98.50746269	313	17	202	1988	1	1790	0	1408

TRINITY_DN15624_c0_g1_i3	Glyma.06G106200.1	100	235	9	334	2133	157	1947	0	1930

TRINITY_DN15624_c0_g1_i5	Glyma.06G106200.1	96.98492462	235	9	334	2133	157	1947	0	1930

TRINITY_DN15624_c0_g1_i2	Glyma.06G106200.1	98.63013699	234	15	334	2120	157	1947	0	1853

TRINITY_DN11657_c1_g1_i1	Glyma.13G058900.1	97.4137931	182	14	352	2133	1	1800	0	2167

TRINITY_DN16444_c0_g1_i1	Glyma.16G204200.1	98.73417722	166	3	20	1818	748	2549	0	2377

TRINITY_DN16444_c0_g1_i1	Glyma.16G204200.2	98.73417722	166	3	20	1818	655	2456	0	2377

TRINITY_DN16444_c0_g1_i1	Glyma.16G204200.3	98.1595092	166	3	20	1818	118	1919	0	2377

TRINITY_DN13483_c0_g1_i6	Glyma.20G095800.1	95.08928571	159	1	58	1860	16	1815	0	2429

TRINITY_DN13483_c0_g1_i6	Glyma.20G095800.2	98.34710744	158	6	58	1860	16	1797	0	2318

TRINITY_DN10790_c0_g1_i1	Glyma.02G206000.3	100	155	8	248	2044	1	1794	0	2374

TRINITY_DN10790_c0_g1_i1	Glyma.02G206000.4	100	155	8	248	2044	1	1794	0	2374

TRINITY_DN10790_c0_g1_i1	Glyma.02G206000.2	100	155	8	248	2044	1	1794	0	2374

TRINITY_DN10790_c0_g1_i1	Glyma.02G206000.1	100	155	8	248	2044	1	1794	0	2374

TRINITY_DN12347_c0_g1_i1	Glyma.14G215500.1	100	146	8	50	1819	4	1803	0	2289

TRINITY_DN3371_c0_g1_i1	Glyma.11G176400.1	95.10204082	143	2	379	2180	1893	92	0	2525

TRINITY_DN3371_c0_g1_i1	Glyma.11G176400.2	100	143	2	379	2180	1806	5	0	2525

TRINITY_DN3523_c0_g1_i1	Glyma.14G182300.2	96.4664311	141	6	206	2004	2	1797	0	2477

TRINITY_DN16149_c1_g1_i2	Glyma.17G191000.2	95.40229885	126	10	482	2245	1797	1	0	2340

TRINITY_DN11721_c0_g1_i1	Glyma.06G118100.1	98.57142857	124	0	438	2240	1803	1	0	2643

TRINITY_DN11721_c0_g1_i1	Glyma.06G118100.3	95.8677686	124	0	438	2240	1803	1	0	2643

TRINITY_DN11450_c0_g1_i1	Glyma.09G258100.3	95.91836735	116	3	59	1851	1	1803	0	2623

TRINITY_DN11450_c0_g1_i1	Glyma.09G258100.6	100	116	3	59	1851	1	1803	0	2623

TRINITY_DN11450_c0_g1_i1	Glyma.09G258100.5	99.46524064	116	3	59	1851	1	1803	0	2623

TRINITY_DN11450_c0_g2_i1	Glyma.09G258100.3	97.91666667	116	3	59	1851	1	1803	0	2623

TRINITY_DN11450_c0_g2_i1	Glyma.09G258100.6	100	116	3	59	1851	1	1803	0	2623

TRINITY_DN11450_c0_g2_i1	Glyma.09G258100.5	99.00990099	116	3	59	1851	1	1803	0	2623

TRINITY_DN14734_c0_g1_i1	Glyma.15G010000.2	99.02439024	90	0	990	2792	1824	22	0	2832

TRINITY_DN13991_c0_g1_i1	Glyma.17G048100.2	100	320	15	133	1931	1812	38	0	1362

TRINITY_DN13991_c0_g1_i3	Glyma.17G048100.2	100	320	15	133	1931	1812	38	0	1362

TRINITY_DN15950_c0_g1_i1	Glyma.15G013500.1	97.61904762	296	54	3	1729	166	1931	0	970

TRINITY_DN15950_c0_g1_i1	Glyma.15G013500.2	99.29577465	296	54	3	1729	166	1931	0	970

TRINITY_DN13157_c0_g1_i3	Glyma.08G052700.2	95.61403509	237	19	92	1853	1	1796	0	1718

TRINITY_DN13157_c0_g1_i3	Glyma.08G052700.1	95.6043956	237	19	92	1853	1	1796	0	1718

TRINITY_DN16386_c0_g1_i4	Glyma.16G198300.1	98.27586207	180	9	1	1796	854	2640	0	2194

TRINITY_DN16386_c0_g1_i4	Glyma.16G198300.2	98.26086957	180	9	1	1796	854	2640	0	2194

TRINITY_DN7256_c0_g1_i2	Glyma.01G010800.1	100	167	12	310	2089	2045	248	0	2235

TRINITY_DN7724_c0_g1_i3	Glyma.18G013200.1	97.84482759	157	25	168	1909	23	1815	0	2012

TRINITY_DN7724_c0_g1_i4	Glyma.18G013200.1	95.59082892	157	25	168	1909	23	1815	0	2012

TRINITY_DN12691_c0_g1_i2	Glyma.12G029500.3	97.33333333	148	5	226	2022	1797	1	0	2444

TRINITY_DN12691_c0_g1_i2	Glyma.11G104500.6	97.32824427	145	5	226	2022	1797	1	0	2460

TRINITY_DN12691_c0_g1_i2	Glyma.11G104500.5	97.34513274	145	5	226	2022	1797	1	0	2460

TRINITY_DN16628_c0_g1_i1	Glyma.20G092500.3	97.14285714	115	5	569	2369	1798	1	0	2658

TRINITY_DN14280_c0_g2_i2	Glyma.01G184600.1	97.83393502	262	17	269	2055	1781	1	0	1655

TRINITY_DN14280_c0_g2_i2	Glyma.01G184600.10	97.84172662	262	17	269	2055	1781	1	0	1655

TRINITY_DN14280_c0_g2_i2	Glyma.01G184600.3	97.84656797	262	17	269	2055	1781	1	0	1655

TRINITY_DN9420_c0_g1_i1	Glyma.10G152900.1	100	154	6	210	2004	1795	1	0	2396

TRINITY_DN13478_c0_g3_i1	Glyma.13G342500.1	96.19377163	145	21	551	2335	1776	1	0	2257

TRINITY_DN13478_c0_g3_i2	Glyma.13G342500.1	100	145	21	456	2240	1776	1	0	2257

TRINITY_DN13478_c0_g3_i1	Glyma.15G031800.1	96.19377163	138	23	551	2335	1773	1	0	2278

TRINITY_DN13478_c0_g3_i2	Glyma.15G031800.1	100	138	23	456	2240	1773	1	0	2278

TRINITY_DN8284_c0_g1_i1	Glyma.05G196800.1	98.88888889	126	6	185	1983	3	1789	0	2525

TRINITY_DN14542_c0_g2_i2	Glyma.08G187100.2	97.22222222	124	0	189	1989	1	1801	0	2639

TRINITY_DN14542_c0_g2_i2	Glyma.15G045900.1	97.02970297	119	0	189	1989	1	1801	0	2667

TRINITY_DN15915_c0_g2_i14	Glyma.06G285200.1	100	113	0	360	2160	2013	213	0	2700

TRINITY_DN15915_c0_g2_i14	Glyma.12G120500.1	98.47328244	109	0	360	2160	2013	213	0	2723

TRINITY_DN15778_c0_g1_i1	Glyma.19G154500.1	95.25862069	427	48	1046	2820	1858	92	3.66E-165	584

TRINITY_DN13991_c0_g1_i1	Glyma.13G111500.3	98.71794872	347	11	628	2424	1788	1	0	1304

TRINITY_DN13991_c0_g1_i3	Glyma.13G111500.3	99.61832061	342	18	628	2411	1788	1	0	1249

TRINITY_DN13081_c0_g1_i1	Glyma.13G284600.1	95	319	11	206	1996	46	1836	0	1441

TRINITY_DN14936_c0_g2_i1	Glyma.04G060800.1	96.2962963	291	12	435	2218	101	1896	0	1583

TRINITY_DN14936_c0_g2_i1	Glyma.06G061400.2	96.2962963	280	28	435	2218	5	1779	0	1507

TRINITY_DN14936_c0_g2_i2	Glyma.04G060800.1	96.2962963	256	12	136	1919	101	1896	0	1777

TRINITY_DN15348_c0_g1_i1	Glyma.13G241100.1	95.91836735	241	12	1	1778	1885	3680	0	1821

TRINITY_DN15348_c0_g1_i3	Glyma.13G241100.1	96.84684685	241	12	1	1778	1885	3680	0	1821

TRINITY_DN13642_c0_g1_i1	Glyma.05G120800.1	97.78481013	210	26	288	2066	1758	1	0	1753

TRINITY_DN16012_c0_g1_i2	Glyma.13G002200.3	95.72649573	194	8	312	2105	26	1822	0	2191

TRINITY_DN7256_c0_g1_i3	Glyma.01G010800.2	95.78651685	172	6	310	2103	2030	248	0	2222

TRINITY_DN13868_c0_g1_i1	Glyma.15G076900.5	96.875	154	0	267	2066	1	1800	0	2471

TRINITY_DN13868_c0_g1_i3	Glyma.15G076900.5	95.23809524	154	0	267	2066	1	1800	0	2471

TRINITY_DN13868_c0_g1_i6	Glyma.15G076900.5	96.66136725	154	0	267	2066	1	1800	0	2471

TRINITY_DN13868_c0_g1_i8	Glyma.15G076900.5	97.36842105	154	0	267	2066	1	1800	0	2471

TRINITY_DN9450_c0_g2_i3	Glyma.06G181100.1	98.48484848	136	8	1443	3218	1219	2997	0	2279

TRINITY_DN8284_c0_g1_i1	Glyma.08G004400.1	97.75280899	125	6	186	1983	1	1792	0	2567

TRINITY_DN14727_c0_g1_i1	Glyma.19G098100.1	98.33333333	116	6	662	2461	2013	223	0	2623

TRINITY_DN13868_c0_g1_i10	Glyma.15G076900.5	100	115	0	267	2066	1	1800	0	2687

TRINITY_DN13868_c0_g1_i4	Glyma.15G076900.5	100	115	0	267	2066	1	1800	0	2687

TRINITY_DN13868_c0_g1_i5	Glyma.15G076900.5	98.9010989	115	0	267	2066	1	1800	0	2687

TRINITY_DN13868_c0_g1_i9	Glyma.15G076900.5	95	115	0	267	2066	1	1800	0	2687

TRINITY_DN11806_c0_g2_i1	Glyma.11G210700.1	100	108	4	255	2042	1800	1	0	2649

TRINITY_DN11806_c0_g2_i2	Glyma.11G210700.1	100	108	4	398	2185	1800	1	0	2649

TRINITY_DN15539_c0_g1_i1	Glyma.01G164100.1	100	89	0	804	2603	586	2385	0	2832

TRINITY_DN10284_c0_g1_i1	Glyma.19G251400.1	97.63313609	86	1	184	1980	1800	1	0	2828

TRINITY_DN16141_c0_g1_i1	Glyma.06G003000.12	100	70	2	1594	3381	2262	463	0	2859

TRINITY_DN16141_c0_g1_i3	Glyma.06G003000.12	98.48484848	70	2	1594	3381	2262	463	0	2859

TRINITY_DN10974_c0_g2_i1	Glyma.06G170300.1	96.46017699	391	43	1685	3458	1832	56	0	852

TRINITY_DN10974_c0_g2_i4	Glyma.06G170300.1	95.09202454	391	43	1813	3586	1832	56	0	852

TRINITY_DN2139_c0_g1_i1	Glyma.11G252700.1	98.9010989	277	18	429	2211	39	1811	0	1517

TRINITY_DN15750_c1_g1_i3	Glyma.17G055100.2	100	271	7	736	2530	1843	49	0	1770

TRINITY_DN16452_c0_g1_i1	Glyma.08G349400.1	100	258	22	285	2057	1779	1	0	1596

TRINITY_DN14936_c0_g2_i2	Glyma.06G061400.2	96.53846154	244	26	136	1919	5	1779	0	1718

TRINITY_DN16251_c0_g3_i7	Glyma.09G060600.6	95.3271028	214	12	624	2415	2256	472	0	2001

TRINITY_DN16353_c0_g1_i2	Glyma.19G064000.1	100	212	10	667	2458	330	2121	0	2058

TRINITY_DN16353_c0_g1_i7	Glyma.19G064000.1	99.3495935	212	10	749	2540	330	2121	0	2058

TRINITY_DN16251_c0_g3_i7	Glyma.15G167100.7	100	207	13	624	2415	2256	472	0	2039

TRINITY_DN16251_c0_g3_i7	Glyma.15G167100.8	98.5915493	207	13	624	2415	2256	472	0	2039

TRINITY_DN16251_c0_g3_i7	Glyma.15G167100.6	100	205	15	624	2415	2253	472	0	2032

TRINITY_DN9381_c0_g1_i1	Glyma.13G002100.1	95	190	1	548	2343	9	1807	0	2250

TRINITY_DN16022_c0_g2_i2	Glyma.01G118400.4	95.63218391	185	14	1240	3026	5	1788	0	2122

TRINITY_DN16022_c0_g2_i2	Glyma.01G118400.3	100	185	14	1240	3026	5	1788	0	2122

TRINITY_DN15768_c0_g1_i2	Glyma.19G178400.2	99.09090909	149	6	1	1796	532	2327	0	2459

TRINITY_DN15768_c0_g1_i2	Glyma.19G178400.1	99.02912621	149	6	1	1796	532	2327	0	2459

TRINITY_DN13808_c0_g1_i3	Glyma.02G048200.1	95.23809524	147	14	245	2019	106	1878	0	2185

TRINITY_DN7767_c0_g1_i3	Glyma.10G279100.1	97.43589744	125	1	310	2102	3801	2003	0	2591

TRINITY_DN7767_c0_g1_i3	Glyma.20G110600.1	96.47058824	121	1	310	2102	3804	2006	0	2614

TRINITY_DN10603_c0_g1_i2	Glyma.10G031400.1	98.98989899	92	6	570	2361	1806	15	0	2723

TRINITY_DN15768_c0_g1_i2	Glyma.10G048800.1	100	252	4	1	1796	523	2318	0	1899

TRINITY_DN15624_c0_g1_i1	Glyma.13G087100.1	97.5	249	10	346	2129	151	1944	0	1825

TRINITY_DN14369_c1_g3_i1	Glyma.11G001500.1	98.9010989	241	6	410	2205	75	1867	0	1940

TRINITY_DN14369_c1_g3_i1	Glyma.01G242800.1	100	231	6	410	2205	120	1912	0	1995

TRINITY_DN13157_c0_g1_i3	Glyma.05G245300.8	95.60185185	229	17	92	1853	1	1793	0	1786

TRINITY_DN13157_c0_g1_i3	Glyma.05G245300.6	95.625	229	17	92	1853	1	1793	0	1786

TRINITY_DN13157_c0_g1_i3	Glyma.05G245300.9	95.61403509	229	17	92	1853	1	1793	0	1786

TRINITY_DN13157_c0_g1_i3	Glyma.05G245300.4	95.62043796	229	17	92	1853	1	1793	0	1786

TRINITY_DN13157_c0_g1_i3	Glyma.05G245300.1	95.6043956	229	17	92	1853	1	1793	0	1786

TRINITY_DN13018_c0_g1_i1	Glyma.03G081600.2	99.55357143	198	28	549	2270	1807	11	0	1725

TRINITY_DN13018_c0_g1_i1	Glyma.03G081600.3	96.42857143	198	28	549	2270	1807	11	0	1725

TRINITY_DN13018_c0_g1_i1	Glyma.03G081600.1	96.42857143	198	28	549	2270	1807	11	0	1725

TRINITY_DN6029_c0_g1_i1	Glyma.17G098300.1	98.08219178	183	14	28	1800	307	2094	0	2080

TRINITY_DN10814_c0_g1_i1	Glyma.11G139700.2	96.55172414	178	0	735	2532	481	2278	0	2335

TRINITY_DN16138_c1_g1_i8	Glyma.08G077100.2	95.74468085	161	5	608	2383	3	1798	0	2274

TRINITY_DN16138_c1_g1_i9	Glyma.08G077100.2	99.78902954	161	5	593	2368	3	1798	0	2274

TRINITY_DN3420_c0_g2_i1	Glyma.05G049200.1	100	161	9	168	1944	1	1795	0	2274

TRINITY_DN15418_c0_g2_i1	Glyma.05G139900.1	95.52238806	154	9	123	1910	1	1794	0	2377

TRINITY_DN12739_c0_g1_i1	Glyma.15G091600.1	99.43502825	152	1	308	2099	1	1798	0	2440

TRINITY_DN15768_c0_g1_i2	Glyma.03G177600.2	97.78699862	143	4	1	1796	535	2330	0	2503

TRINITY_DN15768_c0_g1_i2	Glyma.03G177600.1	100	143	4	1	1796	535	2330	0	2503

TRINITY_DN16429_c0_g2_i2	Glyma.17G228100.2	99.09090909	137	17	514	2296	75	1857	0	2368

TRINITY_DN10263_c0_g2_i2	Glyma.02G191500.1	100	127	2	209	2005	97	1893	0	2604

TRINITY_DN12309_c0_g1_i1	Glyma.10G012600.1	98.21428571	121	2	507	2301	2613	816	0	2630

TRINITY_DN12309_c0_g1_i4	Glyma.10G012600.1	98.29059829	120	3	507	2289	2613	816	0	2558

TRINITY_DN15019_c0_g1_i4	Glyma.02G096800.1	95.75289575	114	12	487	2272	1805	11	0	2591

TRINITY_DN15051_c0_g1_i1	Glyma.18G217100.1	98.64253394	111	2	436	2232	1	1797	0	2693

TRINITY_DN4033_c0_g1_i1	Glyma.11G123500.3	97.45098039	111	3	908	2705	11183	9395	0	2647

TRINITY_DN14650_c0_g2_i2	Glyma.04G031800.1	99.45054945	108	3	424	2217	1797	1	0	2689

TRINITY_DN15174_c0_g1_i1	Glyma.20G020400.1	100	100	2	313	2110	1	1795	0	2747

TRINITY_DN12184_c0_g1_i1	Glyma.06G217400.1	98.42519685	92	0	1	1798	1125	2922	0	2811

TRINITY_DN10603_c0_g1_i2	Glyma.02G143000.1	96.12403101	87	7	570	2361	1806	15	0	2761

TRINITY_DN10603_c0_g1_i2	Glyma.02G143000.2	100	87	7	570	2361	1806	15	0	2761

TRINITY_DN15539_c0_g1_i1	Glyma.11G079200.1	100	85	0	804	2601	586	2383	0	2850

TRINITY_DN16004_c1_g1_i5	Glyma.17G096100.1	100	410	42	106	1880	421	2195	0	763

TRINITY_DN9028_c0_g1_i1	Glyma.13G251100.1	96.69811321	252	15	53	1837	604	2391	0	1786

TRINITY_DN14238_c0_g2_i1	Glyma.13G247300.4	97.61904762	245	17	285	2062	87	1876	0	1794

TRINITY_DN14238_c0_g2_i1	Glyma.13G247300.2	97.61904762	245	17	285	2062	87	1876	0	1794

TRINITY_DN15768_c0_g1_i2	Glyma.13G136700.1	96.49122807	238	2	1	1796	604	2399	0	1988

TRINITY_DN15768_c0_g1_i2	Glyma.13G136700.2	97.1659919	238	2	1	1796	529	2324	0	1988

TRINITY_DN15768_c0_g1_i2	Glyma.13G136700.3	97.05882353	238	2	1	1796	529	2324	0	1988

TRINITY_DN9512_c0_g1_i1	Glyma.06G228800.1	96.15384615	225	24	51	1825	31	1815	0	1853

TRINITY_DN16410_c0_g1_i2	Glyma.18G263200.1	98.57142857	223	0	15	1811	2125	3921	0	2084

TRINITY_DN16410_c0_g1_i3	Glyma.18G263200.1	97.97979798	223	0	15	1811	2125	3921	0	2084

TRINITY_DN9899_c0_g1_i4	Glyma.03G256300.2	98.06763285	209	36	27	1795	473	2233	0	1748

TRINITY_DN9899_c0_g1_i4	Glyma.03G256300.1	100	209	36	27	1795	473	2233	0	1748

TRINITY_DN6449_c0_g1_i1	Glyma.15G088000.1	96.2962963	191	7	59	1844	112	1906	0	2176

TRINITY_DN16149_c1_g1_i1	Glyma.06G082800.2	97.56097561	175	7	407	2193	1778	1	0	2161

TRINITY_DN11819_c0_g3_i3	Glyma.19G103000.2	96.75925926	168	7	53	1840	1	1797	0	2329

TRINITY_DN1784_c0_g1_i1	Glyma.01G245400.1	97.5	162	14	451	2239	414	2199	0	2298

TRINITY_DN2358_c0_g1_i1	Glyma.06G080900.1	96.875	158	27	245	2019	2134	363	0	2139

TRINITY_DN11473_c0_g1_i2	Glyma.18G288300.1	95.40229885	157	4	193	1988	3243	5033	0	2403

TRINITY_DN15519_c1_g1_i2	Glyma.20G017100.2	96.54320988	156	1	510	2306	1800	12	0	2403

TRINITY_DN6023_c0_g1_i1	Glyma.20G135800.4	98.07692308	128	9	162	1945	1	1796	0	2519

TRINITY_DN15051_c0_g1_i1	Glyma.09G271900.1	100	127	0	436	2232	1	1797	0	2615

TRINITY_DN14337_c0_g2_i2	Glyma.17G104200.1	100	114	5	607	2394	1797	1	0	2628

TRINITY_DN13529_c0_g1_i3	Glyma.07G269100.5	98.4	109	17	527	2311	1	1764	0	2423

TRINITY_DN13529_c0_g1_i3	Glyma.07G269100.3	100	109	17	527	2311	1	1764	0	2423

TRINITY_DN16141_c0_g1_i1	Glyma.06G003000.11	97.95321637	70	1	1594	3381	2259	463	0	2872

TRINITY_DN16141_c0_g1_i3	Glyma.06G003000.11	100	70	1	1594	3381	2259	463	0	2872

TRINITY_DN16004_c1_g1_i2	Glyma.13G369300.1	95.68965517	384	32	1367	3132	1060	2843	0	918

TRINITY_DN16323_c0_g2_i1	Glyma.18G250700.1	99.05660377	272	8	327	2109	1799	11	0	1681

TRINITY_DN9028_c0_g1_i1	Glyma.15G063200.1	96.68874172	256	10	53	1837	607	2394	0	1775

TRINITY_DN15626_c0_g3_i2	Glyma.15G217500.1	98.83040936	221	12	425	2208	1	1781	0	1917

TRINITY_DN15626_c0_g3_i2	Glyma.15G217500.2	98.83040936	221	12	425	2208	1	1781	0	1917

TRINITY_DN16012_c0_g1_i3	Glyma.13G002200.3	98.5915493	214	8	406	2195	26	1818	0	2073

TRINITY_DN16012_c0_g1_i5	Glyma.13G002200.3	100	214	8	312	2101	26	1818	0	2073

TRINITY_DN16012_c0_g1_i2	Glyma.20G066800.1	97.10982659	197	9	312	2101	26	1818	0	2167

TRINITY_DN14244_c0_g4_i2	Glyma.12G072700.2	96.19377163	194	7	186	1979	1791	1	0	2196

TRINITY_DN16012_c0_g1_i2	Glyma.13G002200.1	98.55072464	194	8	312	2101	26	1818	0	2183

TRINITY_DN16012_c0_g1_i4	Glyma.13G002200.3	99.18032787	194	8	406	2195	26	1818	0	2183

TRINITY_DN16012_c0_g1_i9	Glyma.13G002200.3	100	194	8	312	2101	26	1818	0	2183

TRINITY_DN15626_c0_g3_i3	Glyma.15G217500.1	100	177	12	330	2113	1	1781	0	2161

TRINITY_DN15626_c0_g3_i3	Glyma.15G217500.2	99.36305732	177	12	330	2113	1	1781	0	2161

TRINITY_DN15876_c0_g1_i2	Glyma.04G039400.2	98.83495146	163	0	107	1902	1	1796	0	2414

TRINITY_DN16004_c1_g1_i2	Glyma.17G103700.1	98.7012987	158	2	1347	3139	1049	2844	0	2422

TRINITY_DN16004_c1_g1_i2	Glyma.13G156000.1	98.96907216	140	2	1347	3139	1049	2844	0	2521

TRINITY_DN16138_c1_g1_i19	Glyma.08G077100.2	97.82608696	135	1	619	2402	3	1798	0	2492

TRINITY_DN12309_c0_g1_i1	Glyma.02G012100.1	99.35897436	134	3	507	2301	2607	816	0	2542

TRINITY_DN12309_c0_g1_i1	Glyma.02G012100.2	96.05263158	134	3	507	2301	2589	798	0	2542

TRINITY_DN12309_c0_g1_i4	Glyma.02G012100.1	98.29059829	131	4	507	2289	2607	816	0	2481

TRINITY_DN12309_c0_g1_i4	Glyma.02G012100.2	98.29059829	131	4	507	2289	2589	798	0	2481

TRINITY_DN11049_c0_g3_i1	Glyma.10G045500.3	97.76536313	111	0	1098	2893	2286	491	0	2702

TRINITY_DN11049_c0_g3_i1	Glyma.10G045500.4	97.40820734	111	0	1098	2893	1842	47	0	2702

TRINITY_DN14526_c0_g1_i1	Glyma.11G016000.3	100	99	2	673	2462	1871	76	0	2730

TRINITY_DN14323_c0_g1_i3	Glyma.01G159400.1	96.52928416	95	5	380	2167	1794	1	0	2726

TRINITY_DN16004_c1_g1_i2	Glyma.15G004300.1	96.10894942	397	30	1367	3132	1060	2843	0	857

TRINITY_DN14369_c1_g1_i1	Glyma.10G257300.1	97.22222222	224	3	150	1940	81	1874	0	2041

TRINITY_DN8285_c0_g1_i1	Glyma.02G107200.2	95.29411765	198	31	587	2300	32	1811	0	1598

TRINITY_DN11825_c0_g2_i2	Glyma.04G191500.2	98.57142857	194	2	373	2166	2116	323	0	2228

TRINITY_DN11825_c0_g2_i2	Glyma.04G191500.1	98.5645933	194	2	373	2166	2116	323	0	2228

TRINITY_DN36336_c0_g2_i1	Glyma.03G185100.1	100	169	14	389	2158	1851	57	0	2217

TRINITY_DN3420_c0_g2_i1	Glyma.17G131100.1	100	168	8	168	1944	1	1792	0	2248

TRINITY_DN14265_c0_g2_i1	Glyma.16G102400.1	95	166	6	320	2112	1852	66	0	2331

TRINITY_DN15175_c0_g1_i3	Glyma.07G125400.1	99.58506224	161	7	95	1883	1	1792	0	2364

TRINITY_DN10263_c0_g2_i2	Glyma.10G105400.1	100	150	0	211	2005	63	1857	0	2484

TRINITY_DN15711_c0_g1_i3	Glyma.01G119500.3	100	132	1	1	1792	214	2008	0	2564

TRINITY_DN15711_c0_g1_i3	Glyma.01G119500.1	97.6635514	132	1	1	1792	214	2008	0	2564

TRINITY_DN15711_c0_g1_i3	Glyma.03G055900.1	100	129	1	1	1792	217	2011	0	2580

TRINITY_DN12309_c0_g1_i1	Glyma.10G012600.2	97.05882353	122	0	507	2301	2610	816	0	2639

TRINITY_DN12309_c0_g1_i1	Glyma.10G012600.3	96.22641509	122	0	507	2301	2595	801	0	2639

TRINITY_DN12309_c0_g1_i4	Glyma.10G012600.2	97.84560144	121	1	507	2289	2610	816	0	2567

TRINITY_DN12309_c0_g1_i4	Glyma.10G012600.3	95.215311	121	1	507	2289	2595	801	0	2567

TRINITY_DN14432_c0_g1_i1	Glyma.U029000.5	100	105	3	798	2583	1	1795	0	2675

TRINITY_DN12701_c0_g2_i1	Glyma.05G220300.1	96.07843137	96	1	1	1795	163	1954	0	2763

TRINITY_DN12701_c0_g2_i1	Glyma.08G026700.1	96.07843137	85	1	1	1795	163	1954	0	2824

TRINITY_DN15634_c2_g1_i2	Glyma.03G192600.1	98.82352941	446	53	2314	4078	2309	544	1.19E-131	473

TRINITY_DN15634_c2_g1_i3	Glyma.03G192600.1	100	446	53	2425	4189	2309	544	1.22E-131	473

TRINITY_DN12797_c0_g1_i1	Glyma.18G023900.1	99.21414538	322	19	305	2085	3698	1918	0	1362

TRINITY_DN15699_c0_g1_i12	Glyma.19G056700.1	100	228	6	105	1894	405	2194	0	1999

TRINITY_DN13529_c0_g1_i2	Glyma.07G269100.5	100	187	16	527	2308	1	1764	0	2006

TRINITY_DN13529_c0_g1_i2	Glyma.07G269100.3	100	187	16	527	2308	1	1764	0	2006

TRINITY_DN5299_c0_g2_i1	Glyma.05G030600.1	95.97585513	170	4	110	1891	1	1794	0	2294

TRINITY_DN7614_c0_g1_i1	Glyma.02G089100.4	100	169	4	387	2174	1863	72	0	2326

TRINITY_DN13769_c0_g1_i1	Glyma.07G055500.1	96.15384615	152	4	545	2335	1	1785	0	2394

TRINITY_DN13769_c0_g1_i1	Glyma.07G055500.2	100	152	4	545	2335	1	1785	0	2394

TRINITY_DN13723_c0_g1_i1	Glyma.09G213100.1	98.07228916	149	0	187	1980	1	1794	0	2488

TRINITY_DN13723_c0_g1_i1	Glyma.09G213100.2	98.33333333	149	0	187	1980	1	1794	0	2488

TRINITY_DN13723_c0_g1_i4	Glyma.09G213100.1	97.75280899	149	0	569	2362	1	1794	0	2488

TRINITY_DN13723_c0_g1_i4	Glyma.09G213100.2	98.2832618	149	0	569	2362	1	1794	0	2488

TRINITY_DN7983_c0_g1_i2	Glyma.13G184200.3	95.78059072	148	8	414	2204	1	1785	0	2416

TRINITY_DN8994_c0_g2_i1	Glyma.12G227800.1	100	135	17	48	1823	7	1788	0	2372

TRINITY_DN13723_c0_g1_i1	Glyma.01G149300.1	99.10714286	129	0	187	1980	1	1794	0	2599

TRINITY_DN13723_c0_g1_i1	Glyma.01G149300.2	97.6744186	129	0	187	1980	1	1794	0	2599

TRINITY_DN13723_c0_g1_i1	Glyma.01G149300.3	96.59090909	129	0	187	1980	1	1794	0	2599

TRINITY_DN13723_c0_g1_i4	Glyma.01G149300.1	98.55072464	129	0	569	2362	1	1794	0	2599

TRINITY_DN13723_c0_g1_i4	Glyma.01G149300.2	100	129	0	569	2362	1	1794	0	2599

TRINITY_DN13723_c0_g1_i4	Glyma.01G149300.3	98.75	129	0	569	2362	1	1794	0	2599

TRINITY_DN35698_c0_g1_i1	Glyma.11G047800.1	98.25581395	100	3	222	2012	1	1794	0	2739

TRINITY_DN35698_c0_g1_i1	Glyma.01G194100.1	98.27586207	98	3	222	2012	1	1794	0	2750

TRINITY_DN14704_c0_g1_i1	Glyma.06G081600.2	95.16129032	78	1	192	1982	1	1794	0	2861

TRINITY_DN15884_c0_g1_i6	Glyma.12G177500.1	97.58064516	217	13	391	2172	1788	1	0	2006

TRINITY_DN15884_c0_g1_i6	Glyma.12G177500.2	97.58064516	217	13	391	2172	1788	1	0	2006

TRINITY_DN15302_c0_g1_i2	Glyma.13G092500.1	98.37398374	198	5	839	2627	1	1789	0	2163

TRINITY_DN15175_c0_g1_i3	Glyma.01G006400.1	99.58506224	165	3	95	1883	1	1792	0	2364

TRINITY_DN14969_c0_g1_i1	Glyma.02G220700.1	100	144	7	542	2326	1	1785	0	2410

TRINITY_DN14969_c0_g1_i2	Glyma.02G220700.1	100	144	7	464	2248	1	1785	0	2410

TRINITY_DN12072_c0_g1_i2	Glyma.03G086800.1	100	136	28	316	2058	1	1785	0	2183

TRINITY_DN13758_c0_g1_i3	Glyma.14G219700.4	100	121	8	64	1838	1	1793	0	2525

TRINITY_DN13758_c0_g1_i3	Glyma.14G219700.1	95.12195122	121	8	64	1838	1	1793	0	2525

TRINITY_DN13758_c0_g1_i3	Glyma.14G219700.3	96.37526652	120	9	64	1838	1	1790	0	2510

TRINITY_DN14728_c0_g2_i1	Glyma.15G140900.1	96.03174603	119	2	109	1898	1	1792	0	2627

TRINITY_DN13758_c0_g1_i3	Glyma.17G258700.1	100	116	8	64	1838	1	1793	0	2553

TRINITY_DN13758_c0_g1_i3	Glyma.17G258700.2	100	114	10	64	1838	1	1790	0	2543

TRINITY_DN15699_c0_g1_i12	Glyma.19G003200.1	96.90721649	91	0	102	1894	408	2200	0	2808

TRINITY_DN15886_c3_g1_i1	Glyma.07G234200.2	96.69421488	270	24	1715	3497	1241	3009	0	1607

TRINITY_DN15886_c3_g1_i3	Glyma.07G234200.2	95.38461538	270	24	1924	3706	1241	3009	0	1607

TRINITY_DN15624_c0_g1_i1	Glyma.14G169300.1	98.05194805	265	9	346	2125	151	1940	0	1751

TRINITY_DN9121_c0_g1_i1	Glyma.06G169400.1	97.56097561	208	24	126	1909	1	1766	0	1938

TRINITY_DN14645_c0_g1_i2	Glyma.09G059000.3	98.27586207	203	15	951	2720	1791	1	0	2036

TRINITY_DN14645_c0_g1_i3	Glyma.09G059000.3	97.91666667	203	15	327	2096	1791	1	0	2036

TRINITY_DN14645_c0_g1_i1	Glyma.09G059000.3	100	174	15	951	2720	1791	1	0	2196

TRINITY_DN13308_c0_g1_i1	Glyma.01G200100.1	98.45201238	151	2	104	1894	1	1791	0	2460

TRINITY_DN15702_c2_g1_i1	Glyma.12G096600.1	100	133	15	406	2193	1749	1	0	2268

TRINITY_DN15702_c2_g1_i1	Glyma.06G308200.1	96.88385269	125	12	406	2193	1746	1	0	2294

TRINITY_DN37056_c0_g1_i1	Glyma.18G048900.1	100	116	2	1	1791	101	1891	0	2654

TRINITY_DN2927_c0_g1_i1	Glyma.14G190200.1	97.77777778	109	1	1	1783	1170	2961	0	2647

TRINITY_DN16078_c0_g2_i2	Glyma.10G208100.1	98.36065574	96	2	103	1893	1	1791	0	2765

TRINITY_DN15408_c0_g1_i6	Glyma.07G069600.1	96.2962963	91	13	75	1832	1	1779	0	2501

TRINITY_DN15464_c0_g1_i2	Glyma.01G070300.1	97.38219895	393	31	230	1996	47	1822	0	878

TRINITY_DN15464_c0_g1_i2	Glyma.01G070300.2	95.95959596	393	31	230	1996	47	1822	0	878

TRINITY_DN15464_c0_g1_i2	Glyma.01G070300.3	95.95959596	393	31	230	1996	47	1822	0	878

TRINITY_DN36917_c0_g1_i1	Glyma.02G237300.2	100	347	34	9	1770	2183	410	0	1088

TRINITY_DN2139_c0_g1_i1	Glyma.18G004200.1	98.98989899	281	17	428	2211	73	1835	0	1524

TRINITY_DN15888_c0_g3_i4	Glyma.14G048300.1	95.14563107	262	22	1263	3045	1490	3257	0	1655

TRINITY_DN15897_c0_g2_i1	Glyma.18G108100.1	96.04130809	242	29	164	1947	3152	1405	0	1644

TRINITY_DN14238_c0_g2_i4	Glyma.08G060100.1	98.64864865	234	9	176	1965	356	2133	0	1921

TRINITY_DN14238_c0_g2_i3	Glyma.08G060100.3	97.61904762	234	9	396	2185	92	1869	0	1921

TRINITY_DN14238_c0_g2_i3	Glyma.08G060100.2	97.61904762	234	9	396	2185	92	1869	0	1921

TRINITY_DN14238_c0_g2_i4	Glyma.08G060100.3	98.66444073	234	9	176	1965	92	1869	0	1921

TRINITY_DN14238_c0_g2_i4	Glyma.08G060100.2	98.64864865	234	9	176	1965	92	1869	0	1921

TRINITY_DN13631_c1_g1_i1	Glyma.16G127400.1	95.4954955	212	17	137	1920	2007	251	0	1868

TRINITY_DN6307_c0_g1_i1	Glyma.05G030400.1	95.68965517	199	4	129	1918	7	1790	0	2154

TRINITY_DN35745_c0_g1_i1	Glyma.19G110600.1	99.53379953	178	15	150	1925	1767	1	0	2069

TRINITY_DN6029_c0_g1_i1	Glyma.05G028500.1	98.08259587	172	17	28	1800	295	2079	0	2200

TRINITY_DN13308_c0_g1_i1	Glyma.11G042000.1	98.45360825	159	0	104	1894	1	1791	0	2427

TRINITY_DN135_c0_g2_i1	Glyma.05G196400.1	98.7804878	141	2	200	1987	1	1791	0	2507

TRINITY_DN135_c0_g2_i1	Glyma.05G196400.3	98.77675841	141	2	200	1987	1	1791	0	2507

TRINITY_DN135_c0_g2_i1	Glyma.05G196400.2	98.7804878	136	8	200	1987	1	1776	0	2438

TRINITY_DN6539_c0_g1_i2	Glyma.05G230700.4	100	135	2	417	2206	1854	65	0	2547

TRINITY_DN37056_c0_g1_i1	Glyma.11G201900.1	98.93617021	126	0	1	1791	101	1891	0	2610

TRINITY_DN11806_c0_g2_i1	Glyma.10G069800.1	100	118	1	255	2042	1791	1	0	2634

TRINITY_DN11806_c0_g2_i2	Glyma.10G069800.1	100	118	1	398	2185	1791	1	0	2634

TRINITY_DN16811_c0_g1_i1	Glyma.10G159200.1	96.36363636	112	12	1742	3520	1782	1	0	2551

TRINITY_DN13529_c0_g1_i3	Glyma.07G269100.6	100	109	16	527	2311	1	1758	0	2451

TRINITY_DN13529_c0_g1_i3	Glyma.07G269100.4	100	109	16	527	2311	1	1758	0	2451

TRINITY_DN13529_c0_g1_i3	Glyma.07G269100.2	100	109	16	527	2311	1	1758	0	2451

TRINITY_DN15408_c0_g1_i2	Glyma.03G008200.1	100	105	14	75	1832	1	1782	0	2455

TRINITY_DN15408_c0_g1_i5	Glyma.03G008200.1	95.87155963	105	14	75	1832	1	1782	0	2455

TRINITY_DN11825_c0_g2_i2	Glyma.05G044200.1	98.57142857	100	1	376	2166	2101	314	0	2734

TRINITY_DN11825_c0_g2_i2	Glyma.05G044200.2	98.58156028	100	1	376	2166	1915	128	0	2734

TRINITY_DN3861_c0_g1_i1	Glyma.10G091000.1	96.03174603	99	1	1	1791	635	2424	0	2752

TRINITY_DN11825_c0_g2_i2	Glyma.17G126700.1	98.57142857	98	1	376	2166	2038	251	0	2745

TRINITY_DN16141_c0_g1_i1	Glyma.06G003000.10	95.06398537	70	1	1594	3381	2253	463	0	2900

TRINITY_DN16141_c0_g1_i3	Glyma.06G003000.10	99.41348974	70	1	1594	3381	2253	463	0	2900

TRINITY_DN15464_c0_g1_i2	Glyma.02G127400.1	97.38219895	390	30	230	1996	47	1822	0	905

TRINITY_DN15464_c0_g1_i2	Glyma.02G127400.2	97.38219895	390	30	230	1996	47	1822	0	905

TRINITY_DN14680_c0_g2_i1	Glyma.13G134500.2	96.59090909	351	42	43	1804	353	2102	0	922

TRINITY_DN14680_c0_g2_i2	Glyma.13G134500.2	100	351	42	43	1804	353	2102	0	922

TRINITY_DN14680_c0_g2_i3	Glyma.13G134500.2	95.74468085	351	42	43	1804	353	2102	0	922

TRINITY_DN14680_c0_g2_i4	Glyma.13G134500.2	95.4248366	351	42	43	1804	353	2102	0	922

TRINITY_DN14680_c0_g2_i5	Glyma.13G134500.2	98.75776398	351	42	43	1804	353	2102	0	922

TRINITY_DN14680_c0_g2_i6	Glyma.13G134500.2	96.35535308	351	42	43	1804	353	2102	0	922

TRINITY_DN6769_c0_g1_i3	Glyma.15G160700.3	99.00990099	274	26	252	2039	68	1814	0	1496

TRINITY_DN6769_c0_g1_i3	Glyma.15G160700.4	99.00990099	274	26	252	2039	68	1814	0	1496

TRINITY_DN6769_c0_g1_i3	Glyma.15G160700.2	97.25274725	274	26	252	2039	68	1814	0	1496

TRINITY_DN15884_c0_g1_i6	Glyma.13G323200.1	96.9924812	221	12	391	2172	1782	1	0	1978

TRINITY_DN16177_c0_g1_i1	Glyma.17G085500.1	99.80314961	209	10	78	1854	2151	3927	0	1980

TRINITY_DN16177_c0_g1_i2	Glyma.17G085500.1	99.6763754	209	10	78	1854	2151	3927	0	1980

TRINITY_DN16177_c0_g1_i3	Glyma.17G085500.1	100	209	10	78	1854	2151	3927	0	1980

TRINITY_DN15961_c0_g1_i6	Glyma.18G109100.1	97.54098361	171	8	1	1774	4089	5877	0	2248

TRINITY_DN15961_c0_g1_i6	Glyma.18G109100.2	95.36423841	171	8	1	1774	4056	5844	0	2248

TRINITY_DN15961_c0_g1_i6	Glyma.18G109100.3	98	171	8	1	1774	3981	5769	0	2248

TRINITY_DN15961_c0_g1_i6	Glyma.18G109100.4	98	171	8	1	1774	2466	4254	0	2248

TRINITY_DN14540_c0_g1_i3	Glyma.19G067400.1	95.12195122	162	10	790	2561	1775	1	0	2194

TRINITY_DN12735_c0_g2_i1	Glyma.08G352800.1	97.64705882	138	6	388	2172	1785	1	0	2477

TRINITY_DN12735_c0_g2_i1	Glyma.08G352800.7	100	138	6	388	2172	1785	1	0	2477

TRINITY_DN12735_c0_g2_i1	Glyma.08G352800.8	100	138	6	388	2172	1785	1	0	2477

TRINITY_DN12735_c0_g2_i1	Glyma.08G352800.10	100	138	6	388	2172	1785	1	0	2477

TRINITY_DN12735_c0_g2_i4	Glyma.08G352800.1	95.95959596	138	6	388	2172	1785	1	0	2477

TRINITY_DN12735_c0_g2_i4	Glyma.08G352800.7	95.95959596	138	6	388	2172	1785	1	0	2477

TRINITY_DN12735_c0_g2_i4	Glyma.08G352800.8	95.60723514	138	6	388	2172	1785	1	0	2477

TRINITY_DN12735_c0_g2_i4	Glyma.08G352800.10	95.60810811	138	6	388	2172	1785	1	0	2477

TRINITY_DN8994_c0_g2_i1	Glyma.13G272300.1	100	138	14	48	1823	7	1791	0	2418

TRINITY_DN6539_c0_g1_i2	Glyma.05G230700.3	100	135	2	417	2205	1851	63	0	2545

TRINITY_DN15961_c0_g1_i5	Glyma.18G109100.1	99.0990991	135	4	1	1780	4089	5877	0	2486

TRINITY_DN15961_c0_g1_i5	Glyma.18G109100.2	100	135	4	1	1780	4056	5844	0	2486

TRINITY_DN15961_c0_g1_i5	Glyma.18G109100.3	97.41935484	135	4	1	1780	3981	5769	0	2486

TRINITY_DN15961_c0_g1_i5	Glyma.18G109100.4	98.91067538	135	4	1	1780	2466	4254	0	2486

TRINITY_DN10749_c0_g1_i1	Glyma.06G130700.1	95.83333333	133	10	1	1783	162	1944	0	2479

TRINITY_DN12735_c0_g2_i2	Glyma.08G352800.1	98.31932773	132	15	388	2145	1785	1	0	2335

TRINITY_DN12735_c0_g2_i2	Glyma.08G352800.7	98.31932773	132	15	388	2145	1785	1	0	2335

TRINITY_DN12735_c0_g2_i2	Glyma.08G352800.8	98.31932773	132	15	388	2145	1785	1	0	2335

TRINITY_DN12735_c0_g2_i2	Glyma.08G352800.10	97.25685786	132	15	388	2145	1785	1	0	2335

TRINITY_DN12735_c0_g2_i3	Glyma.08G352800.1	99.78070175	132	15	388	2145	1785	1	0	2335

TRINITY_DN12735_c0_g2_i3	Glyma.08G352800.7	100	132	15	388	2145	1785	1	0	2335

TRINITY_DN12735_c0_g2_i3	Glyma.08G352800.8	99.50738916	132	15	388	2145	1785	1	0	2335

TRINITY_DN12735_c0_g2_i3	Glyma.08G352800.10	100	132	15	388	2145	1785	1	0	2335

TRINITY_DN15136_c0_g1_i2	Glyma.12G236000.2	100	129	5	313	2097	1785	1	0	2527

TRINITY_DN14323_c0_g1_i3	Glyma.01G159400.2	97.87234043	95	4	380	2167	1788	1	0	2754

TRINITY_DN16452_c0_g1_i1	Glyma.18G167000.1	96.6442953	255	21	285	2057	1770	1	0	1664

TRINITY_DN15848_c1_g4_i8	Glyma.12G047400.3	100	250	1	430	2218	1	1786	0	1899

TRINITY_DN15848_c1_g4_i8	Glyma.12G047400.2	99.06542056	250	1	430	2218	1	1786	0	1899

TRINITY_DN4570_c0_g1_i1	Glyma.09G154100.1	100	230	16	47	1828	520	2292	0	1881

TRINITY_DN15606_c0_g1_i1	Glyma.13G242800.1	99.04761905	180	14	983	2761	976	2727	0	2002

TRINITY_DN15606_c0_g1_i2	Glyma.13G242800.1	100	180	14	1048	2826	976	2727	0	2002

TRINITY_DN15961_c0_g1_i4	Glyma.18G109100.1	96.84210526	157	4	1	1779	4090	5877	0	2362

TRINITY_DN15961_c0_g1_i4	Glyma.18G109100.2	95.77464789	157	4	1	1779	4057	5844	0	2362

TRINITY_DN15961_c0_g1_i4	Glyma.18G109100.3	99.07407407	157	4	1	1779	3982	5769	0	2362

TRINITY_DN15961_c0_g1_i4	Glyma.18G109100.4	99.07407407	157	4	1	1779	2467	4254	0	2362

TRINITY_DN16429_c0_g2_i2	Glyma.14G096100.2	96.16935484	148	10	514	2296	99	1878	0	2386

TRINITY_DN6539_c0_g1_i2	Glyma.08G038200.7	99.6124031	139	2	417	2204	1854	67	0	2521

TRINITY_DN6539_c0_g1_i2	Glyma.08G038200.2	99.62216625	139	2	417	2204	1854	67	0	2521

TRINITY_DN6539_c0_g1_i2	Glyma.08G038200.3	99.58677686	139	2	417	2204	1854	67	0	2521

TRINITY_DN6539_c0_g1_i2	Glyma.08G038200.4	99.6031746	139	2	417	2204	1854	67	0	2521

TRINITY_DN135_c0_g2_i1	Glyma.08G004200.3	100	139	2	200	1987	1	1788	0	2521

TRINITY_DN15279_c0_g1_i1	Glyma.04G133100.1	97.01492537	117	9	81	1866	48	1818	0	2519

TRINITY_DN16047_c1_g1_i1	Glyma.06G016700.1	100	117	16	265	2042	2693	934	0	2398

TRINITY_DN15408_c0_g1_i6	Glyma.03G008200.1	96.2962963	99	14	75	1830	1	1780	0	2484

TRINITY_DN10790_c0_g1_i1	Glyma.04G155000.1	100	299	39	291	2051	44	1801	0	1277

TRINITY_DN10790_c0_g1_i1	Glyma.04G155000.7	100	299	39	291	2051	44	1801	0	1277

TRINITY_DN10790_c0_g1_i1	Glyma.04G155000.8	100	299	39	291	2051	44	1801	0	1277

TRINITY_DN10790_c0_g1_i1	Glyma.04G155000.2	100	299	39	291	2051	44	1801	0	1277

TRINITY_DN10790_c0_g1_i1	Glyma.04G155000.3	100	299	39	291	2051	44	1801	0	1277

TRINITY_DN10790_c0_g1_i1	Glyma.04G155000.4	100	299	39	291	2051	44	1801	0	1277

TRINITY_DN10790_c0_g1_i1	Glyma.04G155000.5	100	299	39	291	2051	44	1801	0	1277

TRINITY_DN10790_c0_g1_i1	Glyma.04G155000.6	100	299	39	291	2051	44	1801	0	1277

TRINITY_DN10335_c1_g1_i1	Glyma.02G150600.1	100	293	25	16	1781	19	1796	0	1472

TRINITY_DN6769_c0_g1_i2	Glyma.15G160700.1	98.87640449	277	23	252	2016	68	1835	0	1489

TRINITY_DN13529_c0_g1_i2	Glyma.07G269100.6	98.8372093	187	15	527	2308	1	1758	0	2034

TRINITY_DN13529_c0_g1_i2	Glyma.07G269100.4	98.81656805	187	15	527	2308	1	1758	0	2034

TRINITY_DN13529_c0_g1_i2	Glyma.07G269100.2	98.41269841	187	15	527	2308	1	1758	0	2034

TRINITY_DN16251_c0_g3_i8	Glyma.09G060600.6	96.38554217	186	8	624	2405	2256	472	0	2213

TRINITY_DN16251_c0_g3_i8	Glyma.15G167100.7	100	176	7	624	2405	2256	472	0	2268

TRINITY_DN16251_c0_g3_i8	Glyma.15G167100.8	96	176	7	624	2405	2256	472	0	2268

TRINITY_DN16251_c0_g3_i8	Glyma.15G167100.6	97.10144928	174	9	624	2405	2253	472	0	2261

TRINITY_DN14204_c0_g1_i3	Glyma.02G057400.1	100	170	0	249	2036	117	1904	0	2361

TRINITY_DN8949_c0_g1_i1	Glyma.05G104000.1	100	156	16	182	1945	1773	1	0	2185

TRINITY_DN16429_c0_g2_i4	Glyma.17G228100.2	97.34693878	148	12	514	2286	75	1856	0	2346

TRINITY_DN14204_c0_g1_i4	Glyma.02G057400.1	100	147	0	249	2036	117	1904	0	2488

TRINITY_DN10000_c0_g1_i1	Glyma.12G007800.3	97.21254355	137	15	2368	4136	1896	137	0	2239

TRINITY_DN10000_c0_g1_i1	Glyma.12G007800.2	99.57446809	137	15	2368	4136	1896	137	0	2239

TRINITY_DN10000_c0_g1_i1	Glyma.12G007800.1	96.06299213	137	15	2368	4136	1896	137	0	2239

TRINITY_DN10000_c0_g1_i1	Glyma.12G007800.6	96.06481481	137	15	2368	4136	1896	137	0	2239

TRINITY_DN10000_c0_g1_i4	Glyma.12G007800.3	97.22222222	137	15	2638	4406	1896	137	0	2239

TRINITY_DN10000_c0_g1_i4	Glyma.12G007800.2	98.57142857	137	15	2638	4406	1896	137	0	2239

TRINITY_DN10000_c0_g1_i4	Glyma.12G007800.1	97.39130435	137	15	2638	4406	1896	137	0	2239

TRINITY_DN10000_c0_g1_i4	Glyma.12G007800.6	100	137	15	2638	4406	1896	137	0	2239

TRINITY_DN14969_c0_g1_i1	Glyma.14G187000.1	100	134	4	542	2326	1	1779	0	2483

TRINITY_DN14969_c0_g1_i1	Glyma.14G187000.2	100	134	4	542	2326	1	1779	0	2483

TRINITY_DN14969_c0_g1_i2	Glyma.14G187000.1	100	134	4	464	2248	1	1779	0	2483

TRINITY_DN14969_c0_g1_i2	Glyma.14G187000.2	100	134	4	464	2248	1	1779	0	2483

TRINITY_DN12735_c0_g2_i1	Glyma.18G169800.1	99.74747475	129	2	388	2172	1785	1	0	2549

TRINITY_DN12735_c0_g2_i1	Glyma.18G169800.10	100	129	2	388	2172	1785	1	0	2549

TRINITY_DN12735_c0_g2_i4	Glyma.18G169800.1	100	129	2	388	2172	1785	1	0	2549

TRINITY_DN12735_c0_g2_i4	Glyma.18G169800.10	96.92307692	129	2	388	2172	1785	1	0	2549

TRINITY_DN12735_c0_g2_i2	Glyma.18G169800.1	99.0990991	125	10	388	2145	1785	1	0	2396

TRINITY_DN12735_c0_g2_i2	Glyma.18G169800.10	99.0990991	125	10	388	2145	1785	1	0	2396

TRINITY_DN12735_c0_g2_i3	Glyma.18G169800.1	95.91836735	125	10	388	2145	1785	1	0	2396

TRINITY_DN12735_c0_g2_i3	Glyma.18G169800.10	98.46153846	125	10	388	2145	1785	1	0	2396

TRINITY_DN15884_c0_g1_i6	Glyma.11G194700.1	98.1981982	124	5	391	2172	1788	1	0	2577

TRINITY_DN12717_c0_g1_i1	Glyma.19G241800.3	97.26027397	114	0	607	2394	1	1788	0	2671

TRINITY_DN12717_c0_g1_i2	Glyma.19G241800.3	96.60056657	114	0	363	2150	1	1788	0	2671

TRINITY_DN15408_c0_g1_i2	Glyma.07G069600.1	96.27071823	96	13	75	1828	1	1775	0	2466

TRINITY_DN15408_c0_g1_i5	Glyma.07G069600.1	95.87155963	96	13	75	1828	1	1775	0	2466

TRINITY_DN9279_c0_g1_i1	Glyma.09G215700.1	100	93	1	448	2232	1	1788	0	2767

TRINITY_DN9279_c0_g1_i1	Glyma.09G215700.2	98.83449883	93	1	448	2232	1	1788	0	2767

TRINITY_DN9279_c0_g1_i1	Glyma.01G150700.3	96.69421488	90	1	448	2232	1	1788	0	2784

TRINITY_DN9279_c0_g1_i1	Glyma.01G150700.1	100	90	1	448	2232	1	1788	0	2784

TRINITY_DN16141_c0_g1_i1	Glyma.06G003000.9	100	70	0	1594	3381	2250	463	0	2915

TRINITY_DN16141_c0_g1_i3	Glyma.06G003000.9	95.6043956	70	0	1594	3381	2250	463	0	2915

TRINITY_DN13778_c1_g3_i3	Glyma.16G065200.1	98.95287958	286	15	689	2463	3046	1271	0	1567

TRINITY_DN12711_c0_g1_i1	Glyma.02G005700.1	100	256	29	986	2750	1	1756	0	1539

TRINITY_DN13212_c0_g1_i1	Glyma.01G097900.1	96.9581749	242	38	282	2018	223	1947	0	1236

TRINITY_DN9512_c0_g1_i1	Glyma.12G157500.1	100	205	26	57	1825	26	1800	0	1971

TRINITY_DN12768_c0_g1_i1	Glyma.12G116700.1	99.54545455	145	6	322	2102	2406	629	0	2399

TRINITY_DN9519_c0_g1_i2	Glyma.18G075600.1	99.33333333	133	2	312	2089	1962	176	0	2505

TRINITY_DN9519_c0_g1_i2	Glyma.08G330800.2	95.60810811	133	5	312	2089	1962	176	0	2505

TRINITY_DN9519_c0_g1_i2	Glyma.08G330800.1	96.4028777	133	5	312	2089	1962	176	0	2505

TRINITY_DN15884_c0_g1_i6	Glyma.12G079400.1	97.58064516	131	3	391	2172	1785	1	0	2529

TRINITY_DN10749_c0_g1_i1	Glyma.04G234100.1	95.83333333	129	7	1	1783	162	1935	0	2475

TRINITY_DN16047_c1_g1_i1	Glyma.04G016600.2	96.28647215	123	18	265	2041	2711	953	0	2374

TRINITY_DN16047_c1_g1_i1	Glyma.04G016600.1	100	123	18	265	2041	2711	953	0	2374

TRINITY_DN37821_c0_g1_i1	Glyma.19G069800.1	95.3125	121	15	332	2097	1770	1	0	2385

TRINITY_DN11291_c0_g2_i1	Glyma.04G061600.2	100	103	6	536	2310	1	1784	0	2632

TRINITY_DN13707_c0_g1_i1	Glyma.20G230900.2	100	99	1	579	2362	175	1961	0	2732

TRINITY_DN15516_c0_g1_i1	Glyma.12G198600.2	100	336	23	862	2630	1	1775	0	1256

TRINITY_DN15516_c0_g1_i1	Glyma.12G198600.3	97.95918367	336	23	862	2630	1	1775	0	1256

TRINITY_DN15450_c1_g1_i1	Glyma.01G136500.2	100	234	6	392	2174	2062	280	0	1964

TRINITY_DN15450_c1_g1_i3	Glyma.01G136500.2	97.44897959	234	6	339	2121	2062	280	0	1964

TRINITY_DN7256_c0_g1_i2	Glyma.01G010800.2	100	173	3	310	2089	2030	248	0	2281

TRINITY_DN14239_c0_g1_i1	Glyma.15G224400.2	100	167	14	513	2259	1818	36	0	2102

TRINITY_DN14239_c0_g1_i1	Glyma.15G224400.3	100	167	14	513	2259	1818	36	0	2102

TRINITY_DN14239_c0_g1_i1	Glyma.15G224400.5	100	167	14	513	2259	1818	36	0	2102

TRINITY_DN14239_c0_g1_i1	Glyma.15G224400.4	100	167	14	513	2259	1818	36	0	2102

TRINITY_DN14239_c0_g1_i1	Glyma.15G224400.7	100	167	14	513	2259	1818	36	0	2102

TRINITY_DN14239_c0_g1_i1	Glyma.15G224400.6	100	167	14	513	2259	1818	36	0	2102

TRINITY_DN9899_c0_g1_i3	Glyma.19G254200.1	97.77777778	162	2	13	1795	472	2254	0	2362

TRINITY_DN15848_c1_g4_i1	Glyma.12G047400.3	100	154	0	430	2215	1	1786	0	2446

TRINITY_DN15848_c1_g4_i1	Glyma.12G047400.2	100	154	0	430	2215	1	1786	0	2446

TRINITY_DN15866_c0_g1_i5	Glyma.08G179400.1	100	149	7	1759	3541	1	1770	0	2350

TRINITY_DN15866_c0_g1_i5	Glyma.08G179400.4	100	149	7	1759	3541	1	1770	0	2350

TRINITY_DN15866_c0_g1_i5	Glyma.08G179400.3	98.57142857	149	7	1759	3541	1	1770	0	2350

TRINITY_DN12659_c1_g1_i1	Glyma.01G140600.5	98.1042654	94	1	723	2505	1786	1	0	2758

TRINITY_DN3744_c0_g1_i1	Glyma.11G029400.1	95.54140127	360	21	229	2003	2189	424	0	1114

TRINITY_DN10699_c0_g1_i1	Glyma.05G116700.1	99.07407407	307	22	787	2551	1810	34	0	1415

TRINITY_DN15282_c0_g1_i1	Glyma.05G150200.1	96.26769627	239	25	1251	2996	2474	703	0	1637

TRINITY_DN15282_c0_g1_i2	Glyma.05G150200.1	96.52777778	239	25	1320	3065	2474	703	0	1637

TRINITY_DN15282_c0_g1_i6	Glyma.05G150200.1	98.1042654	239	25	706	2451	2474	703	0	1637

TRINITY_DN15282_c0_g1_i1	Glyma.05G150200.2	95.14563107	239	25	1251	2996	2468	697	0	1637

TRINITY_DN15282_c0_g1_i2	Glyma.05G150200.2	97.58241758	239	25	1320	3065	2468	697	0	1637

TRINITY_DN15282_c0_g1_i6	Glyma.05G150200.2	99.08466819	239	25	706	2451	2468	697	0	1637

TRINITY_DN16429_c0_g2_i4	Glyma.14G096100.2	97.34693878	154	9	514	2286	99	1877	0	2327

TRINITY_DN13808_c0_g1_i6	Glyma.02G048200.1	96.89440994	149	9	245	2005	106	1878	0	2239

TRINITY_DN15042_c0_g1_i1	Glyma.11G052700.1	96.23529412	140	5	744	2519	1824	55	0	2366

TRINITY_DN15042_c0_g1_i2	Glyma.11G052700.1	100	140	5	744	2519	1824	55	0	2366

TRINITY_DN14767_c0_g1_i1	Glyma.13G345300.1	100	139	20	436	2144	2835	1055	0	2010

TRINITY_DN14767_c0_g1_i1	Glyma.13G345300.3	100	139	20	436	2144	2835	1055	0	2010

TRINITY_DN14767_c0_g1_i3	Glyma.13G345300.1	100	139	20	971	2679	2835	1055	0	2010

TRINITY_DN14767_c0_g1_i3	Glyma.13G345300.3	100	139	20	971	2679	2835	1055	0	2010

TRINITY_DN10000_c0_g1_i1	Glyma.09G228800.2	100	137	16	2368	4128	1758	1	0	2207

TRINITY_DN10000_c0_g1_i1	Glyma.09G228800.1	100	137	16	2368	4128	1758	1	0	2207

TRINITY_DN10000_c0_g1_i2	Glyma.09G228800.2	100	137	16	2368	4128	1758	1	0	2207

TRINITY_DN10000_c0_g1_i2	Glyma.09G228800.1	96.42521167	137	16	2368	4128	1758	1	0	2207

TRINITY_DN10000_c0_g1_i3	Glyma.09G228800.2	100	137	16	2638	4398	1758	1	0	2207

TRINITY_DN10000_c0_g1_i3	Glyma.09G228800.1	97.63313609	137	16	2638	4398	1758	1	0	2207

TRINITY_DN10000_c0_g1_i4	Glyma.09G228800.2	98.66666667	137	16	2638	4398	1758	1	0	2207

TRINITY_DN10000_c0_g1_i4	Glyma.09G228800.1	100	137	16	2638	4398	1758	1	0	2207

TRINITY_DN13032_c0_g2_i1	Glyma.08G019600.3	97.90575916	135	1	537	2318	2325	541	0	2529

TRINITY_DN5734_c0_g1_i1	Glyma.14G135100.1	100	133	9	1	1782	238	2010	0	2462

TRINITY_DN13979_c0_g2_i1	Glyma.10G005300.1	98.06763285	132	2	76	1860	1	1773	0	2488

TRINITY_DN11259_c0_g1_i1	Glyma.08G187600.3	96.28378378	128	2	204	1984	1	1785	0	2562

TRINITY_DN11259_c0_g1_i1	Glyma.08G187600.2	99.08536585	128	2	204	1984	1	1785	0	2562

TRINITY_DN11259_c0_g1_i2	Glyma.08G187600.3	97.77777778	128	2	802	2582	1	1785	0	2562

TRINITY_DN11259_c0_g1_i2	Glyma.08G187600.2	100	128	2	802	2582	1	1785	0	2562

TRINITY_DN11259_c0_g1_i3	Glyma.08G187600.3	98.06763285	128	2	368	2148	1	1785	0	2562

TRINITY_DN11259_c0_g1_i3	Glyma.08G187600.2	100	128	2	368	2148	1	1785	0	2562

TRINITY_DN14563_c0_g1_i2	Glyma.18G218900.1	98.06763285	128	3	349	2127	3999	2215	0	2549

TRINITY_DN14563_c0_g1_i4	Glyma.18G218900.1	95.12195122	128	3	349	2127	3999	2215	0	2549

TRINITY_DN11882_c0_g1_i1	Glyma.06G202100.1	95.83333333	123	19	179	1952	7	1768	0	2396

TRINITY_DN11882_c0_g1_i3	Glyma.06G202100.1	95.83333333	123	19	179	1952	7	1768	0	2396

TRINITY_DN13979_c0_g1_i1	Glyma.10G183900.1	99.01477833	111	5	71	1846	1	1785	0	2623

TRINITY_DN13979_c0_g1_i1	Glyma.20G206300.1	99.01477833	103	5	71	1846	1	1785	0	2667

TRINITY_DN9279_c0_g1_i1	Glyma.09G215700.4	98.83495146	95	7	448	2232	1	1773	0	2693

TRINITY_DN9279_c0_g1_i1	Glyma.09G215700.3	98.8372093	95	7	448	2232	1	1773	0	2693

TRINITY_DN12659_c1_g1_i1	Glyma.01G140600.3	98.11320755	94	1	724	2505	1785	1	0	2756

TRINITY_DN16352_c0_g2_i1	Glyma.08G194700.1	100	298	10	441	2218	4497	2719	0	1572

TRINITY_DN16352_c0_g2_i3	Glyma.08G194700.1	100	298	10	441	2218	4497	2719	0	1572

TRINITY_DN16352_c0_g2_i1	Glyma.08G194700.2	100	298	10	441	2218	3873	2095	0	1572

TRINITY_DN16352_c0_g2_i3	Glyma.08G194700.2	100	298	10	441	2218	3873	2095	0	1572

TRINITY_DN15657_c0_g1_i1	Glyma.09G259400.1	100	297	26	3936	5703	5022	6780	0	1384

TRINITY_DN15657_c0_g1_i1	Glyma.09G259400.2	100	297	26	3936	5703	5022	6780	0	1384

TRINITY_DN15657_c0_g1_i1	Glyma.09G259400.3	96.82539683	297	26	3936	5703	5022	6780	0	1384

TRINITY_DN15657_c0_g1_i3	Glyma.09G259400.1	96.1130742	297	26	3231	4998	5022	6780	0	1384

TRINITY_DN15657_c0_g1_i3	Glyma.09G259400.2	96.11650485	297	26	3231	4998	5022	6780	0	1384

TRINITY_DN15657_c0_g1_i3	Glyma.09G259400.3	98.38709677	297	26	3231	4998	5022	6780	0	1384

TRINITY_DN14389_c0_g1_i4	Glyma.11G224500.1	98.42767296	192	19	97	1872	1	1755	0	1991

TRINITY_DN13979_c0_g2_i1	Glyma.02G003400.1	100	131	3	76	1859	1	1772	0	2492

TRINITY_DN2927_c0_g1_i1	Glyma.02G223400.1	97.01492537	121	2	1	1783	1170	2952	0	2612

TRINITY_DN2927_c0_g1_i1	Glyma.02G223400.5	98.92473118	121	2	1	1783	1128	2910	0	2612

TRINITY_DN2927_c0_g1_i1	Glyma.02G223400.3	97.05882353	121	2	1	1783	861	2643	0	2612

TRINITY_DN2927_c0_g1_i1	Glyma.02G223400.4	97.05882353	121	2	1	1783	861	2643	0	2612

TRINITY_DN2927_c0_g1_i1	Glyma.02G223400.8	98.92473118	121	2	1	1783	861	2643	0	2612

TRINITY_DN2927_c0_g1_i1	Glyma.02G223400.7	98.88888889	121	2	1	1783	627	2409	0	2612

TRINITY_DN2927_c0_g1_i1	Glyma.02G223400.2	98.39816934	121	2	1	1783	627	2409	0	2612

TRINITY_DN12479_c0_g1_i3	Glyma.17G085300.1	96.36363636	120	18	243	2022	1809	54	0	2423

TRINITY_DN1744_c0_g1_i1	Glyma.13G341600.2	98.125	114	2	1	1783	273	2055	0	2651

TRINITY_DN18214_c0_g1_i1	Glyma.10G196300.1	100	110	32	1	1711	831	2598	0	2109

TRINITY_DN16307_c0_g1_i3	Glyma.06G116200.1	96.11650485	287	18	292	2059	2101	352	0	1393

TRINITY_DN17100_c0_g1_i1	Glyma.17G088000.1	97.18309859	250	22	720	2479	1819	45	0	1707

TRINITY_DN14389_c0_g1_i4	Glyma.18G033800.1	100	202	5	97	1872	1	1782	0	2122

TRINITY_DN15082_c0_g1_i3	Glyma.01G211200.1	98.85714286	166	16	185	1927	1	1782	0	2108

TRINITY_DN15337_c0_g2_i1	Glyma.11G219500.1	95.47038328	158	10	1309	3079	1	1771	0	2263

TRINITY_DN12768_c0_g1_i1	Glyma.06G290300.1	99.43502825	155	9	322	2097	2286	511	0	2344

TRINITY_DN12768_c0_g1_i1	Glyma.06G290300.2	99.54751131	155	9	322	2097	2283	508	0	2344

TRINITY_DN12768_c0_g1_i1	Glyma.06G290300.3	98.95833333	153	11	322	2097	2280	508	0	2335

TRINITY_DN15866_c0_g1_i5	Glyma.15G053000.6	95.08196721	145	9	1759	3538	1	1767	0	2366

TRINITY_DN15866_c0_g1_i5	Glyma.15G053000.1	100	145	9	1759	3538	1	1767	0	2366

TRINITY_DN15866_c0_g1_i5	Glyma.15G053000.7	100	145	9	1759	3538	1	1767	0	2366

TRINITY_DN15866_c0_g1_i5	Glyma.15G053000.3	98.79518072	145	10	1759	3538	1	1761	0	2327

TRINITY_DN16327_c0_g1_i3	Glyma.03G142600.1	100	145	14	509	2267	1827	48	0	2314

TRINITY_DN39157_c0_g1_i1	Glyma.19G086700.3	100	142	3	167	1945	1782	1	0	2473

TRINITY_DN39157_c0_g1_i1	Glyma.19G086700.2	100	142	3	167	1945	1782	1	0	2473

TRINITY_DN5734_c0_g1_i1	Glyma.17G197600.2	100	132	10	1	1782	238	2007	0	2471

TRINITY_DN5734_c0_g1_i1	Glyma.17G197600.1	95.91836735	132	10	1	1782	238	2007	0	2471

TRINITY_DN15042_c0_g1_i1	Glyma.01G189500.1	96.3562753	129	8	744	2519	1821	55	0	2429

TRINITY_DN15042_c0_g1_i2	Glyma.01G189500.1	100	129	8	744	2519	1821	55	0	2429

TRINITY_DN8240_c0_g1_i1	Glyma.01G224300.2	96.875	128	4	101	1881	1	1781	0	2558

TRINITY_DN11990_c0_g1_i1	Glyma.05G005500.1	97.5	128	9	193	1968	1	1773	0	2473

TRINITY_DN12085_c0_g1_i1	Glyma.06G041000.2	100	126	6	1	1777	1774	1	0	2497

TRINITY_DN12085_c0_g1_i1	Glyma.06G041000.3	100	126	6	1	1777	1774	1	0	2497

TRINITY_DN12085_c0_g1_i1	Glyma.06G041000.1	100	126	6	1	1777	1774	1	0	2497

TRINITY_DN12085_c0_g1_i1	Glyma.06G041000.4	100	126	6	1	1777	1774	1	0	2497

TRINITY_DN12085_c0_g1_i1	Glyma.06G041000.5	100	126	6	1	1777	1774	1	0	2497

TRINITY_DN12659_c1_g1_i1	Glyma.01G140600.4	98.11320755	94	0	723	2505	1783	1	0	2772

TRINITY_DN3370_c0_g1_i1	Glyma.08G127400.1	98.32635983	391	52	836	2595	1907	175	0	665

TRINITY_DN15186_c0_g1_i3	Glyma.04G065900.1	97.5	308	19	404	2174	1775	8	0	1423

TRINITY_DN15186_c0_g1_i3	Glyma.04G065900.4	99.10714286	308	19	404	2174	1775	8	0	1423

TRINITY_DN12961_c0_g1_i1	Glyma.10G026200.1	97.5	286	8	1	1778	337	2114	0	1655

TRINITY_DN15396_c0_g1_i2	Glyma.03G204800.2	99.7706422	256	12	399	2172	1816	43	0	1770

TRINITY_DN12961_c0_g1_i2	Glyma.10G026200.1	100	255	8	1	1778	337	2114	0	1827

TRINITY_DN15963_c1_g1_i4	Glyma.18G229300.1	96.91358025	219	6	140	1918	25	1803	0	2039

TRINITY_DN15963_c1_g1_i6	Glyma.18G229300.1	98.48484848	192	10	140	1909	25	1803	0	2130

TRINITY_DN39157_c0_g1_i1	Glyma.16G060400.2	100	171	2	167	1945	1782	1	0	2324

TRINITY_DN16169_c0_g1_i1	Glyma.10G292400.2	100	170	6	1179	2957	3459	1690	0	2252

TRINITY_DN12085_c0_g1_i1	Glyma.04G039800.2	100	153	9	1	1777	1771	1	0	2340

TRINITY_DN12085_c0_g1_i1	Glyma.04G039800.3	100	153	9	1	1777	1771	1	0	2340

TRINITY_DN12085_c0_g1_i1	Glyma.04G039800.4	100	153	9	1	1777	1771	1	0	2340

TRINITY_DN16327_c0_g1_i5	Glyma.03G142600.1	100	140	11	617	2376	1827	48	0	2361

TRINITY_DN11990_c0_g1_i1	Glyma.19G006000.1	99.11504425	134	5	193	1970	1	1778	0	2497

TRINITY_DN11990_c0_g1_i1	Glyma.19G006000.4	98.7012987	134	5	193	1970	1	1778	0	2497

TRINITY_DN12479_c0_g1_i3	Glyma.05G041000.2	96.36363636	128	8	243	2021	1818	55	0	2446

TRINITY_DN12226_c0_g2_i3	Glyma.13G053100.3	98.93111639	118	0	624	2405	1782	1	0	2638

TRINITY_DN12226_c0_g2_i3	Glyma.13G053100.1	98.93238434	118	0	624	2405	1782	1	0	2638

TRINITY_DN13455_c0_g1_i2	Glyma.17G209600.1	99.03846154	113	1	412	2181	1782	1	0	2588

TRINITY_DN13455_c0_g1_i4	Glyma.17G209600.1	100	113	1	337	2106	1782	1	0	2588

TRINITY_DN13455_c0_g1_i2	Glyma.17G209600.3	97.2027972	111	5	412	2181	1770	1	0	2521

TRINITY_DN13455_c0_g1_i4	Glyma.17G209600.3	97.56097561	111	5	337	2106	1770	1	0	2521

TRINITY_DN14047_c0_g1_i3	Glyma.11G169900.4	97.8021978	107	1	861	2639	1	1782	0	2678

TRINITY_DN12702_c0_g1_i1	Glyma.04G033200.2	100	98	0	252	2033	1782	1	0	2748

TRINITY_DN12702_c0_g1_i1	Glyma.06G033200.3	97.14285714	92	0	252	2033	1782	1	0	2782

TRINITY_DN14887_c0_g1_i1	Glyma.11G004600.3	100	74	3	228	1998	1893	112	0	2809

TRINITY_DN9805_c0_g1_i1	Glyma.18G085400.1	99.44134078	336	32	493	2248	2238	477	0	1144

TRINITY_DN12961_c0_g1_i2	Glyma.19G155700.1	97.38372093	161	2	1	1780	61	1840	0	2385

TRINITY_DN12961_c0_g1_i2	Glyma.03G153300.1	99.20634921	158	2	1	1780	61	1840	0	2401

TRINITY_DN9735_c0_g2_i1	Glyma.10G165600.7	95.33678756	153	6	308	2080	1	1779	0	2377

TRINITY_DN9735_c0_g2_i1	Glyma.10G165600.1	95.32967033	153	6	308	2080	1	1779	0	2377

TRINITY_DN9735_c0_g2_i1	Glyma.10G165600.6	95.34883721	153	6	308	2080	1	1779	0	2377

TRINITY_DN9735_c0_g2_i1	Glyma.10G165600.5	95.3271028	153	6	308	2080	1	1779	0	2377

TRINITY_DN9735_c0_g2_i1	Glyma.10G165600.3	95.3271028	153	6	308	2080	1	1779	0	2377

TRINITY_DN9735_c0_g2_i1	Glyma.10G165600.2	95.3271028	153	6	308	2080	1	1779	0	2377

TRINITY_DN9735_c0_g2_i1	Glyma.10G165600.4	97.24137931	153	6	308	2080	1	1779	0	2377

TRINITY_DN15961_c0_g1_i6	Glyma.08G308700.2	98.5915493	133	4	1	1774	3987	5766	0	2501

TRINITY_DN15961_c0_g1_i6	Glyma.08G308700.1	100	133	4	1	1774	3987	5766	0	2501

TRINITY_DN15961_c0_g1_i6	Glyma.08G308700.3	100	133	4	1	1774	3810	5589	0	2501

TRINITY_DN15961_c0_g1_i6	Glyma.18G107900.3	97.22222222	131	4	1	1774	3978	5757	0	2512

TRINITY_DN15961_c0_g1_i6	Glyma.18G107900.1	100	131	4	1	1774	3978	5757	0	2512

TRINITY_DN15961_c0_g1_i6	Glyma.18G107900.2	98.9010989	131	4	1	1774	3978	5757	0	2512

TRINITY_DN12961_c0_g1_i1	Glyma.19G155700.1	99.25925926	127	2	1	1780	61	1840	0	2573

TRINITY_DN12961_c0_g1_i1	Glyma.03G153300.1	97.68637532	123	2	1	1780	61	1840	0	2595

TRINITY_DN37821_c0_g1_i1	Glyma.16G073500.1	95.3125	123	15	332	2097	1758	1	0	2362

TRINITY_DN10885_c0_g1_i1	Glyma.05G174600.1	97.68421053	117	9	121	1892	60	1833	0	2538

TRINITY_DN12261_c0_g1_i2	Glyma.05G161000.1	100	116	4	37	1814	32	1806	0	2588

TRINITY_DN15910_c0_g1_i1	Glyma.19G217300.1	95.12195122	97	3	516	2293	386	2166	0	2732

TRINITY_DN15910_c0_g1_i5	Glyma.19G217300.2	96.44970414	97	3	630	2407	269	2049	0	2732

TRINITY_DN15910_c0_g1_i1	Glyma.03G220500.1	100	95	3	516	2293	440	2220	0	2743

TRINITY_DN15910_c0_g1_i1	Glyma.03G220500.3	100	95	3	516	2293	383	2163	0	2743

TRINITY_DN15910_c0_g1_i1	Glyma.03G220500.2	100	95	3	516	2293	308	2088	0	2743

TRINITY_DN15961_c0_g1_i5	Glyma.08G308700.2	97.60191847	84	2	1	1780	3987	5766	0	2811

TRINITY_DN15961_c0_g1_i5	Glyma.08G308700.1	97.60191847	84	2	1	1780	3987	5766	0	2811

TRINITY_DN15961_c0_g1_i5	Glyma.08G308700.3	98.87640449	84	2	1	1780	3810	5589	0	2811

TRINITY_DN15961_c0_g1_i5	Glyma.18G107900.3	99.24242424	82	2	1	1780	3978	5757	0	2822

TRINITY_DN15961_c0_g1_i5	Glyma.18G107900.1	97.56097561	82	2	1	1780	3978	5757	0	2822

TRINITY_DN15961_c0_g1_i5	Glyma.18G107900.2	97.39130435	82	2	1	1780	3978	5757	0	2822

TRINITY_DN14313_c2_g1_i1	Glyma.13G299700.1	95.12195122	307	16	127	1894	1795	22	0	1471

TRINITY_DN14088_c0_g1_i3	Glyma.13G037100.1	96.38554217	214	15	347	2098	1776	1	0	1895

TRINITY_DN14088_c0_g1_i4	Glyma.13G037100.1	97.05882353	214	15	347	2098	1776	1	0	1895

TRINITY_DN12303_c0_g2_i3	Glyma.14G189300.1	96.89655172	198	30	417	2138	1773	1	0	1770

TRINITY_DN12303_c0_g2_i3	Glyma.02G222300.1	96.49122807	196	29	417	2138	1770	1	0	1762

TRINITY_DN15963_c1_g1_i5	Glyma.18G229300.1	100	191	2	140	1918	25	1803	0	2217

TRINITY_DN9632_c0_g1_i2	Glyma.02G277200.1	98.77675841	178	12	36	1796	1	1770	0	2113

TRINITY_DN17368_c0_g1_i1	Glyma.06G322500.1	100	176	2	3	1781	166	1944	0	2300

TRINITY_DN15963_c1_g1_i1	Glyma.18G229300.1	99.18032787	164	6	140	1909	25	1803	0	2307

TRINITY_DN15519_c1_g1_i4	Glyma.07G218900.1	98.49435383	136	2	622	2398	1791	12	0	2514

TRINITY_DN9202_c0_g1_i1	Glyma.05G005600.2	95.52238806	136	10	181	1950	1	1773	0	2423

TRINITY_DN9202_c0_g1_i1	Glyma.05G005600.1	97.01492537	136	10	181	1950	1	1773	0	2423

TRINITY_DN10000_c0_g1_i1	Glyma.12G007800.5	95.23809524	136	15	2368	4128	1752	1	0	2230

TRINITY_DN10000_c0_g1_i1	Glyma.12G007800.4	100	136	15	2368	4128	1752	1	0	2230

TRINITY_DN10000_c0_g1_i2	Glyma.12G007800.5	95.83333333	136	15	2368	4128	1752	1	0	2230

TRINITY_DN10000_c0_g1_i2	Glyma.12G007800.4	97.8021978	136	15	2368	4128	1752	1	0	2230

TRINITY_DN10000_c0_g1_i3	Glyma.12G007800.5	100	136	15	2638	4398	1752	1	0	2230

TRINITY_DN10000_c0_g1_i3	Glyma.12G007800.4	95.02164502	136	15	2638	4398	1752	1	0	2230

TRINITY_DN10000_c0_g1_i4	Glyma.12G007800.5	100	136	15	2638	4398	1752	1	0	2230

TRINITY_DN10000_c0_g1_i4	Glyma.12G007800.4	98.80952381	136	15	2638	4398	1752	1	0	2230

TRINITY_DN10865_c0_g1_i4	Glyma.14G196600.1	100	132	19	200	1942	1773	1	0	2272

TRINITY_DN11990_c0_g1_i1	Glyma.05G005500.2	100	129	7	193	1968	1	1770	0	2483

TRINITY_DN15961_c0_g1_i4	Glyma.08G308700.2	97.48427673	120	2	1	1779	3988	5766	0	2610

TRINITY_DN15961_c0_g1_i4	Glyma.08G308700.1	98.71794872	120	2	1	1779	3988	5766	0	2610

TRINITY_DN15961_c0_g1_i4	Glyma.08G308700.3	97.19202899	120	2	1	1779	3811	5589	0	2610

TRINITY_DN15961_c0_g1_i4	Glyma.18G107900.3	100	116	2	1	1779	3979	5757	0	2632

TRINITY_DN15961_c0_g1_i4	Glyma.18G107900.1	96.40933573	116	2	1	1779	3979	5757	0	2632

TRINITY_DN15961_c0_g1_i4	Glyma.18G107900.2	98.71794872	116	2	1	1779	3979	5757	0	2632

TRINITY_DN3417_c0_g1_i1	Glyma.15G252800.2	99.23664122	109	7	1341	3114	1176	2952	0	2625

TRINITY_DN6860_c0_g1_i2	Glyma.02G267800.1	99.24242424	97	0	725	2504	1840	61	0	2750

TRINITY_DN16415_c1_g1_i2	Glyma.13G327000.2	99.61538462	252	7	2614	4387	1774	1	0	1825

TRINITY_DN16415_c1_g1_i2	Glyma.13G327000.3	99.61685824	252	7	2614	4387	1774	1	0	1825

TRINITY_DN16415_c1_g1_i2	Glyma.13G327000.1	98.87640449	252	7	2614	4387	1774	1	0	1825

TRINITY_DN16415_c1_g1_i9	Glyma.13G327000.2	97.77777778	252	7	2520	4293	1774	1	0	1825

TRINITY_DN16415_c1_g1_i9	Glyma.13G327000.3	95.08196721	252	7	2520	4293	1774	1	0	1825

TRINITY_DN16415_c1_g1_i9	Glyma.13G327000.1	97.6744186	252	7	2520	4293	1774	1	0	1825

TRINITY_DN14088_c0_g1_i3	Glyma.14G119100.1	96.38554217	228	12	347	2098	1776	1	0	1829

TRINITY_DN14088_c0_g1_i4	Glyma.14G119100.1	97.05882353	228	12	347	2098	1776	1	0	1829

TRINITY_DN15963_c1_g1_i4	Glyma.09G262900.1	98.42519685	217	4	140	1916	28	1804	0	2058

TRINITY_DN14503_c0_g1_i1	Glyma.18G300100.2	100	197	8	623	2397	1772	1	0	2122

TRINITY_DN15989_c0_g1_i3	Glyma.05G227400.1	98.7654321	196	4	273	2049	1902	126	0	2174

TRINITY_DN15963_c1_g1_i6	Glyma.09G262900.1	100	194	9	140	1907	28	1804	0	2126

TRINITY_DN11964_c0_g1_i1	Glyma.11G173800.1	98.46153846	165	4	69	1844	1	1770	0	2294

TRINITY_DN12139_c0_g1_i2	Glyma.08G332000.2	100	149	13	90	1852	1	1772	0	2311

TRINITY_DN14473_c0_g2_i2	Glyma.03G164400.2	98.65771812	145	1	79	1857	1	1776	0	2462

TRINITY_DN14473_c0_g2_i2	Glyma.03G164400.3	98.67549669	145	1	79	1857	1	1776	0	2462

TRINITY_DN14473_c0_g2_i2	Glyma.03G164400.1	98.6784141	145	1	79	1857	1	1776	0	2462

TRINITY_DN11964_c0_g1_i1	Glyma.18G061800.1	96.15384615	144	4	69	1844	1	1770	0	2410

TRINITY_DN14473_c0_g2_i2	Glyma.19G165800.1	98.65771812	143	0	79	1857	1	1779	0	2494

TRINITY_DN5999_c0_g1_i1	Glyma.08G132600.1	100	140	0	402	2180	2259	481	0	2510

TRINITY_DN5999_c0_g1_i1	Glyma.08G132600.2	100	140	0	402	2180	2259	481	0	2510

TRINITY_DN16187_c1_g2_i1	Glyma.07G107100.1	95.55555556	140	0	107	1885	1	1779	0	2510

TRINITY_DN16187_c1_g2_i1	Glyma.09G170600.2	100	138	0	107	1885	1	1779	0	2521

TRINITY_DN14767_c0_g1_i1	Glyma.15G029000.6	100	137	30	436	2144	2829	1055	0	2049

TRINITY_DN14767_c0_g1_i1	Glyma.15G029000.7	100	137	30	436	2144	2829	1055	0	2049

TRINITY_DN14767_c0_g1_i1	Glyma.15G029000.1	100	137	30	436	2144	2829	1055	0	2049

TRINITY_DN14767_c0_g1_i1	Glyma.15G029000.3	100	137	30	436	2144	2829	1055	0	2049

TRINITY_DN14767_c0_g1_i3	Glyma.15G029000.6	100	137	30	971	2679	2829	1055	0	2049

TRINITY_DN14767_c0_g1_i3	Glyma.15G029000.7	100	137	30	971	2679	2829	1055	0	2049

TRINITY_DN14767_c0_g1_i3	Glyma.15G029000.1	100	137	30	971	2679	2829	1055	0	2049

TRINITY_DN14767_c0_g1_i3	Glyma.15G029000.3	100	137	30	971	2679	2829	1055	0	2049

TRINITY_DN14767_c0_g1_i1	Glyma.15G029000.4	100	137	30	436	2144	2787	1013	0	2049

TRINITY_DN14767_c0_g1_i1	Glyma.15G029000.5	99.0521327	137	30	436	2144	2787	1013	0	2049

TRINITY_DN14767_c0_g1_i1	Glyma.15G029000.2	100	137	30	436	2144	2787	1013	0	2049

TRINITY_DN14767_c0_g1_i3	Glyma.15G029000.4	100	137	30	971	2679	2787	1013	0	2049

TRINITY_DN14767_c0_g1_i3	Glyma.15G029000.5	100	137	30	971	2679	2787	1013	0	2049

TRINITY_DN14767_c0_g1_i3	Glyma.15G029000.2	100	137	30	971	2679	2787	1013	0	2049

TRINITY_DN15348_c0_g1_i1	Glyma.13G181200.1	95.91836735	120	2	1	1779	1966	3735	0	2562

TRINITY_DN15348_c0_g1_i3	Glyma.13G181200.1	98.4063745	120	2	1	1779	1966	3735	0	2562

TRINITY_DN9773_c0_g1_i1	Glyma.13G163400.1	100	114	7	312	2088	1899	129	0	2590

TRINITY_DN14432_c0_g1_i1	Glyma.17G233000.2	100	101	8	829	2583	587	2365	0	2571

TRINITY_DN8292_c0_g1_i2	Glyma.10G212000.2	100	100	0	897	2675	31	1809	0	2732

TRINITY_DN9404_c0_g1_i1	Glyma.08G257600.2	95.83333333	69	0	1094	2872	1779	1	0	2904

TRINITY_DN6769_c0_g1_i3	Glyma.09G054800.1	96.55172414	277	24	252	2025	71	1806	0	1452

TRINITY_DN6769_c0_g1_i3	Glyma.09G054800.3	97.06666667	277	24	252	2025	2	1737	0	1452

TRINITY_DN15399_c0_g1_i1	Glyma.01G144500.3	98.30985915	224	9	1	1765	5	1781	0	1953

TRINITY_DN15399_c0_g1_i1	Glyma.01G144500.2	98.33333333	224	9	1	1765	5	1781	0	1953

TRINITY_DN14728_c0_g1_i1	Glyma.15G140900.1	98.85057471	204	5	178	1952	1	1775	0	2115

TRINITY_DN14503_c0_g1_i1	Glyma.08G361700.3	96.42857143	199	8	623	2397	1772	1	0	2122

TRINITY_DN15989_c0_g1_i3	Glyma.05G227400.2	96.53179191	196	4	273	2048	1776	1	0	2172

TRINITY_DN15989_c0_g1_i3	Glyma.05G227400.3	100	196	4	273	2048	1776	1	0	2172

TRINITY_DN15963_c1_g1_i5	Glyma.09G262900.1	100	192	2	140	1916	28	1804	0	2207

TRINITY_DN15399_c0_g1_i2	Glyma.01G144500.3	98.31932773	188	8	1	1768	5	1781	0	2170

TRINITY_DN15399_c0_g1_i2	Glyma.01G144500.2	98.31932773	188	8	1	1768	5	1781	0	2170

TRINITY_DN5299_c0_g2_i1	Glyma.17G096200.1	97.59036145	187	1	120	1891	2	1779	0	2209

TRINITY_DN13991_c0_g1_i2	Glyma.13G111500.2	96.72131148	180	2	294	2070	1810	34	0	2274

TRINITY_DN15399_c0_g1_i4	Glyma.01G144500.3	99.51923077	174	9	1	1765	5	1781	0	2230

TRINITY_DN15399_c0_g1_i4	Glyma.01G144500.2	99.50738916	174	9	1	1765	5	1781	0	2230

TRINITY_DN15963_c1_g1_i1	Glyma.09G262900.1	99.18367347	169	7	140	1907	28	1804	0	2276

TRINITY_DN15940_c0_g1_i2	Glyma.18G263300.1	98.82352941	165	5	498	2272	120	1891	0	2311

TRINITY_DN13991_c0_g1_i2	Glyma.17G048100.2	96.72131148	164	2	294	2070	1810	34	0	2362

TRINITY_DN15940_c0_g1_i2	Glyma.18G263300.2	97.48953975	163	11	498	2272	120	1882	0	2265

TRINITY_DN16038_c0_g1_i1	Glyma.10G266600.2	98.95833333	144	0	231	2008	8	1785	0	2486

TRINITY_DN15399_c0_g1_i3	Glyma.01G144500.3	98.36065574	138	8	1	1768	5	1781	0	2447

TRINITY_DN15399_c0_g1_i3	Glyma.01G144500.2	98.32402235	138	8	1	1768	5	1781	0	2447

TRINITY_DN14038_c0_g1_i1	Glyma.10G188100.1	96.90082645	128	5	257	2030	1	1777	0	2542

TRINITY_DN11061_c0_g1_i1	Glyma.17G065600.1	100	117	3	133	1905	11	1788	0	2603

TRINITY_DN14432_c0_g1_i4	Glyma.U029000.7	97.47191011	95	1	1425	3202	98	1869	0	2719

TRINITY_DN14432_c0_g1_i5	Glyma.U029000.7	97.4789916	95	1	1348	3125	98	1869	0	2719

TRINITY_DN3954_c0_g2_i1	Glyma.19G249100.2	100	300	11	866	2633	4	1773	0	1517

TRINITY_DN3954_c0_g2_i1	Glyma.19G249100.3	95.54317549	300	11	866	2633	4	1773	0	1517

TRINITY_DN3954_c0_g1_i1	Glyma.19G249100.2	98.79518072	299	12	866	2632	4	1773	0	1515

TRINITY_DN3954_c0_g1_i1	Glyma.19G249100.3	98.79518072	299	12	866	2632	4	1773	0	1515

TRINITY_DN14238_c0_g2_i2	Glyma.15G066700.6	97.61904762	259	22	189	1945	21	1786	0	1646

TRINITY_DN14238_c0_g2_i2	Glyma.15G066700.1	97.63313609	259	22	189	1945	21	1786	0	1646

TRINITY_DN14088_c0_g1_i1	Glyma.13G037100.1	98.48484848	224	15	158	1906	1773	1	0	1834

TRINITY_DN14088_c0_g1_i2	Glyma.13G037100.1	95.38461538	224	15	158	1906	1773	1	0	1834

TRINITY_DN14637_c0_g1_i1	Glyma.02G015500.1	95.3125	212	21	422	2181	889	2653	0	1919

TRINITY_DN15989_c0_g1_i1	Glyma.05G227400.1	97.14889124	166	0	273	2049	1902	126	0	2362

TRINITY_DN15519_c1_g1_i4	Glyma.20G017100.1	98.4962406	157	0	622	2398	1788	12	0	2412

TRINITY_DN15989_c0_g1_i1	Glyma.01G008500.1	97.14828897	153	2	273	2048	1776	1	0	2422

TRINITY_DN15989_c0_g1_i1	Glyma.16G077800.1	100	142	2	273	2048	1776	1	0	2483

TRINITY_DN15619_c0_g2_i1	Glyma.17G088800.1	99.18699187	134	3	1188	2962	182	1957	0	2519

TRINITY_DN15619_c0_g2_i1	Glyma.17G088800.3	97.21254355	134	3	1188	2962	152	1927	0	2519

TRINITY_DN12072_c0_g1_i2	Glyma.16G087300.1	100	133	21	316	2058	1	1770	0	2279

TRINITY_DN12735_c0_g2_i1	Glyma.18G169800.9	100	129	2	388	2161	1779	6	0	2529

TRINITY_DN12735_c0_g2_i1	Glyma.18G169800.6	100	129	2	388	2161	1779	6	0	2529

TRINITY_DN12735_c0_g2_i4	Glyma.18G169800.9	97.46835443	129	2	388	2161	1779	6	0	2529

TRINITY_DN12735_c0_g2_i4	Glyma.18G169800.6	100	129	2	388	2161	1779	6	0	2529

TRINITY_DN12735_c0_g2_i2	Glyma.18G169800.9	96.25	125	10	388	2134	1779	6	0	2375

TRINITY_DN12735_c0_g2_i2	Glyma.18G169800.6	96.25	125	10	388	2134	1779	6	0	2375

TRINITY_DN12735_c0_g2_i3	Glyma.18G169800.9	97.5	125	10	388	2134	1779	6	0	2375

TRINITY_DN12735_c0_g2_i3	Glyma.18G169800.6	96.94656489	125	10	388	2134	1779	6	0	2375

TRINITY_DN15989_c0_g1_i3	Glyma.01G008500.1	95.65217391	117	2	273	2048	1776	1	0	2621

TRINITY_DN11643_c0_g1_i1	Glyma.17G138300.1	96.53846154	111	3	157	1930	1	1777	0	2647

TRINITY_DN11643_c0_g1_i1	Glyma.17G138300.2	96.53979239	111	3	157	1930	1	1777	0	2647

TRINITY_DN11643_c0_g1_i1	Glyma.17G138300.3	96.34146341	111	3	157	1930	1	1777	0	2647

TRINITY_DN11643_c0_g1_i2	Glyma.17G138300.1	97.77777778	111	3	207	1980	1	1777	0	2647

TRINITY_DN11643_c0_g1_i2	Glyma.17G138300.2	98.8372093	111	3	207	1980	1	1777	0	2647

TRINITY_DN11643_c0_g1_i2	Glyma.17G138300.3	97.0624235	111	3	207	1980	1	1777	0	2647

TRINITY_DN11643_c0_g1_i3	Glyma.17G138300.1	96.54255319	111	3	157	1930	1	1777	0	2647

TRINITY_DN11643_c0_g1_i3	Glyma.17G138300.2	100	111	3	157	1930	1	1777	0	2647

TRINITY_DN11643_c0_g1_i3	Glyma.17G138300.3	100	111	3	157	1930	1	1777	0	2647

TRINITY_DN11643_c0_g1_i4	Glyma.17G138300.1	100	111	3	207	1980	1	1777	0	2647

TRINITY_DN11643_c0_g1_i4	Glyma.17G138300.2	100	111	3	207	1980	1	1777	0	2647

TRINITY_DN11643_c0_g1_i4	Glyma.17G138300.3	100	111	3	207	1980	1	1777	0	2647

TRINITY_DN15989_c0_g1_i3	Glyma.16G077800.1	98.22616408	104	2	273	2048	1776	1	0	2693

TRINITY_DN14728_c0_g1_i1	Glyma.17G033100.1	98.85057471	101	1	178	1954	1	1774	0	2702

TRINITY_DN14728_c0_g1_i1	Glyma.07G240300.1	98.85057471	101	2	178	1954	1	1774	0	2702

TRINITY_DN16360_c0_g4_i4	Glyma.07G010400.1	95.0310559	83	3	329	2101	1	1776	0	2789

TRINITY_DN8031_c0_g2_i1	Glyma.17G165300.1	95.3307393	338	26	475	2231	1782	17	0	1219

TRINITY_DN16076_c2_g3_i1	Glyma.17G180000.1	95.75757576	302	7	352	2121	1986	214	0	1548

TRINITY_DN16076_c2_g3_i2	Glyma.17G180000.1	100	302	7	352	2121	1986	214	0	1548

TRINITY_DN14238_c0_g2_i2	Glyma.13G247300.3	97.61904762	260	20	189	1945	27	1792	0	1652

TRINITY_DN14238_c0_g2_i2	Glyma.13G247300.1	97.62470309	260	20	189	1945	27	1792	0	1652

TRINITY_DN37493_c0_g1_i1	Glyma.16G015000.1	97.76119403	256	7	3	1768	1413	3184	0	1772

TRINITY_DN15910_c0_g1_i4	Glyma.19G217300.2	95.3125	247	13	553	2319	279	2049	0	1821

TRINITY_DN14088_c0_g1_i1	Glyma.14G119100.1	100	238	12	158	1906	1773	1	0	1768

TRINITY_DN14088_c0_g1_i2	Glyma.14G119100.1	95.23809524	238	12	158	1906	1773	1	0	1768

TRINITY_DN14637_c0_g1_i1	Glyma.10G016000.1	95.3125	202	21	422	2174	901	2665	0	1941

TRINITY_DN14637_c0_g1_i1	Glyma.10G016000.2	95.12195122	202	21	422	2174	889	2653	0	1941

TRINITY_DN5792_c0_g1_i1	Glyma.11G237700.1	100	195	4	270	2030	6603	4831	0	2084

TRINITY_DN16132_c0_g1_i3	Glyma.06G056400.2	100	193	4	721	2495	84	1855	0	2178

TRINITY_DN15989_c0_g1_i1	Glyma.05G227400.2	100	166	0	273	2048	1776	1	0	2361

TRINITY_DN15989_c0_g1_i1	Glyma.05G227400.3	95.83333333	166	0	273	2048	1776	1	0	2361

TRINITY_DN15133_c0_g1_i1	Glyma.02G293200.1	96.3190184	164	14	1562	3326	399	2145	0	2113

TRINITY_DN15133_c0_g1_i3	Glyma.02G293200.1	95.16129032	164	14	1701	3465	399	2145	0	2113

TRINITY_DN15133_c0_g1_i5	Glyma.02G293200.1	98.1981982	164	14	982	2746	399	2145	0	2113

TRINITY_DN15133_c0_g1_i8	Glyma.02G293200.1	98.41269841	164	14	832	2596	399	2145	0	2113

TRINITY_DN15133_c0_g1_i9	Glyma.02G293200.1	97.90794979	164	14	1551	3315	399	2145	0	2113

TRINITY_DN15133_c0_g1_i1	Glyma.02G293200.2	100	164	14	1562	3326	174	1920	0	2113

TRINITY_DN15133_c0_g1_i3	Glyma.02G293200.2	95.95959596	164	14	1701	3465	174	1920	0	2113

TRINITY_DN15133_c0_g1_i5	Glyma.02G293200.2	96.2962963	164	14	982	2746	174	1920	0	2113

TRINITY_DN15133_c0_g1_i8	Glyma.02G293200.2	100	164	14	832	2596	174	1920	0	2113

TRINITY_DN15133_c0_g1_i9	Glyma.02G293200.2	97.90794979	164	14	1551	3315	174	1920	0	2113

TRINITY_DN15866_c0_g1_i11	Glyma.08G179400.1	95.89041096	151	3	1284	3056	1	1770	0	2385

TRINITY_DN15866_c0_g1_i11	Glyma.08G179400.4	96.20938628	151	3	1284	3056	1	1770	0	2385

TRINITY_DN15866_c0_g1_i11	Glyma.08G179400.3	96.20991254	151	3	1284	3056	1	1770	0	2385

TRINITY_DN15866_c0_g1_i2	Glyma.08G179400.1	96.94656489	151	3	1193	2965	1	1770	0	2385

TRINITY_DN15866_c0_g1_i2	Glyma.08G179400.4	100	151	3	1193	2965	1	1770	0	2385

TRINITY_DN15866_c0_g1_i2	Glyma.08G179400.3	96.875	151	3	1193	2965	1	1770	0	2385

TRINITY_DN15866_c0_g1_i8	Glyma.08G179400.1	99.57805907	151	3	1333	3105	1	1770	0	2385

TRINITY_DN15866_c0_g1_i8	Glyma.08G179400.4	96.66666667	151	3	1333	3105	1	1770	0	2385

TRINITY_DN15866_c0_g1_i8	Glyma.08G179400.3	100	151	3	1333	3105	1	1770	0	2385

TRINITY_DN15866_c0_g1_i9	Glyma.08G179400.1	98.19277108	151	3	1424	3196	1	1770	0	2385

TRINITY_DN15866_c0_g1_i9	Glyma.08G179400.4	98.19277108	151	3	1424	3196	1	1770	0	2385

TRINITY_DN15866_c0_g1_i9	Glyma.08G179400.3	95.45454545	151	3	1424	3196	1	1770	0	2385

TRINITY_DN13058_c0_g1_i2	Glyma.09G193300.1	100	150	3	51	1819	3226	1452	0	2398

TRINITY_DN9735_c0_g2_i1	Glyma.20G223600.1	95.3271028	145	1	308	2080	1	1776	0	2457

TRINITY_DN9735_c0_g2_i1	Glyma.20G223600.4	95.34050179	145	1	308	2080	1	1776	0	2457

TRINITY_DN9735_c0_g2_i1	Glyma.20G223600.6	95.3271028	145	1	308	2080	1	1776	0	2457

TRINITY_DN12437_c1_g2_i1	Glyma.04G173000.4	96.34146341	145	3	1	1775	856	2627	0	2444

TRINITY_DN12437_c1_g2_i2	Glyma.04G173000.4	100	143	3	1	1775	856	2627	0	2455

TRINITY_DN11259_c0_g1_i1	Glyma.12G170500.2	99.08536585	139	1	204	1979	1	1773	0	2490

TRINITY_DN11259_c0_g1_i2	Glyma.12G170500.2	100	139	1	802	2577	1	1773	0	2490

TRINITY_DN11259_c0_g1_i3	Glyma.12G170500.2	100	139	1	368	2143	1	1773	0	2490

TRINITY_DN14210_c0_g2_i1	Glyma.09G272800.1	98.98989899	139	20	320	2053	1	1761	0	2141

TRINITY_DN14637_c0_g1_i1	Glyma.19G213600.4	96	123	11	423	2174	890	2659	0	2405

TRINITY_DN14637_c0_g1_i1	Glyma.19G213600.5	100	123	11	423	2174	890	2659	0	2405

TRINITY_DN14637_c0_g1_i1	Glyma.19G213600.1	95.65217391	123	11	423	2174	890	2659	0	2405

TRINITY_DN14637_c0_g1_i1	Glyma.19G213600.3	100	123	11	423	2174	827	2596	0	2405

TRINITY_DN35829_c0_g1_i1	Glyma.08G028400.4	97.20670391	97	0	435	2210	1776	1	0	2743

TRINITY_DN35829_c0_g1_i1	Glyma.08G028400.2	99.03846154	97	0	435	2210	1776	1	0	2743

TRINITY_DN18060_c0_g1_i1	Glyma.18G221200.1	95.53264605	442	62	1191	2933	1834	3575	2.09E-112	409

TRINITY_DN18060_c0_g1_i1	Glyma.01G144200.1	97.85714286	441	62	1191	2933	1912	3653	4.49E-114	414

TRINITY_DN18060_c0_g1_i1	Glyma.01G144200.3	97.85714286	441	62	1191	2933	1912	3653	4.49E-114	414

TRINITY_DN18060_c0_g1_i1	Glyma.01G144200.2	97.36842105	441	62	1191	2933	757	2498	4.49E-114	414

TRINITY_DN13326_c0_g2_i1	Glyma.02G178300.1	98.42519685	329	34	126	1877	1749	1	0	1138

TRINITY_DN13326_c0_g2_i1	Glyma.02G178300.2	97.93103448	329	34	126	1877	1749	1	0	1138

TRINITY_DN14313_c2_g1_i1	Glyma.05G034000.1	96.15384615	305	15	127	1889	1957	189	0	1472

TRINITY_DN14371_c0_g3_i1	Glyma.04G173100.1	95.34883721	301	28	258	2012	1	1752	0	1332

TRINITY_DN14371_c0_g3_i1	Glyma.04G173100.2	100	301	28	258	2012	1	1752	0	1332

TRINITY_DN5833_c0_g1_i1	Glyma.17G124300.1	99.45945946	296	27	364	2127	1845	91	0	1437

TRINITY_DN15886_c3_g1_i4	Glyma.07G234200.1	100	233	22	1	1770	1369	3120	0	1807

TRINITY_DN15886_c3_g1_i4	Glyma.07G234200.3	95.23809524	233	22	1	1770	1369	3120	0	1807

TRINITY_DN15886_c3_g1_i4	Glyma.07G234200.2	99.28571429	233	22	1	1770	1258	3009	0	1807

TRINITY_DN14540_c0_g1_i4	Glyma.19G067400.1	100	165	8	790	2546	1775	1	0	2248

TRINITY_DN39731_c0_g1_i1	Glyma.10G177600.1	100	161	11	1	1751	1813	39	0	2231

TRINITY_DN6226_c0_g1_i2	Glyma.01G007800.1	95.93495935	156	15	771	2524	845	2619	0	2278

TRINITY_DN10350_c0_g1_i1	Glyma.08G092100.1	96.63865546	107	1	324	2095	1815	41	0	2665

TRINITY_DN10873_c0_g2_i1	Glyma.04G017700.2	100	98	4	26	1796	1776	2	0	2710

TRINITY_DN15139_c0_g3_i2	Glyma.16G111700.5	95.83333333	241	14	321	2088	2750	989	0	1825

TRINITY_DN15139_c0_g3_i2	Glyma.16G111700.3	96.15384615	241	14	321	2088	2750	989	0	1825

TRINITY_DN15139_c0_g3_i2	Glyma.16G111700.2	100	241	14	321	2088	2750	989	0	1825

TRINITY_DN15139_c0_g3_i2	Glyma.16G111700.4	99.06542056	241	14	321	2088	2750	989	0	1825

TRINITY_DN15139_c0_g3_i2	Glyma.16G111700.1	99.06542056	241	14	321	2088	2750	989	0	1825

TRINITY_DN14897_c0_g1_i1	Glyma.08G263800.1	100	235	21	418	2184	304	2046	0	1729

TRINITY_DN15886_c3_g1_i4	Glyma.15G190200.1	97.17868339	194	7	1	1770	1336	3099	0	2111

TRINITY_DN15886_c3_g1_i4	Glyma.15G190200.2	100	194	7	1	1770	1336	3099	0	2111

TRINITY_DN15886_c3_g1_i4	Glyma.15G190200.3	99.09090909	194	7	1	1770	643	2406	0	2111

TRINITY_DN15886_c3_g1_i4	Glyma.15G190200.4	100	194	7	1	1770	553	2316	0	2111

TRINITY_DN11699_c0_g2_i1	Glyma.09G208200.1	100	193	13	251	2014	1603	3354	0	2001

TRINITY_DN11699_c0_g2_i1	Glyma.09G208200.2	99.15254237	193	13	251	2014	1603	3354	0	2001

TRINITY_DN16049_c1_g1_i1	Glyma.17G128200.1	95.11201629	191	32	938	2703	1718	1	0	1805

TRINITY_DN16049_c1_g1_i1	Glyma.17G128200.3	95.11278195	191	32	938	2703	1718	1	0	1805

TRINITY_DN16049_c1_g1_i1	Glyma.17G128200.5	95.11278195	191	32	938	2703	1718	1	0	1805

TRINITY_DN16049_c1_g1_i1	Glyma.17G128200.4	96.19047619	191	32	938	2703	1718	1	0	1805

TRINITY_DN16049_c1_g1_i1	Glyma.05G046000.1	100	187	27	938	2703	1730	1	0	1905

TRINITY_DN16049_c1_g1_i1	Glyma.05G046000.3	98.91304348	187	27	938	2703	1730	1	0	1905

TRINITY_DN16049_c1_g1_i1	Glyma.05G046000.4	95.11201629	187	27	938	2703	1730	1	0	1905

TRINITY_DN13097_c0_g1_i2	Glyma.02G292300.4	99.00990099	167	5	194	1966	1770	1	0	2318

TRINITY_DN13097_c0_g1_i3	Glyma.02G292300.4	97.98657718	167	5	194	1966	1770	1	0	2318

TRINITY_DN8949_c0_g1_i1	Glyma.17G163500.1	100	167	23	182	1945	1752	1	0	2145

TRINITY_DN8949_c0_g1_i1	Glyma.17G163500.2	100	167	23	182	1945	1752	1	0	2145

TRINITY_DN15337_c0_g2_i1	Glyma.11G219500.2	100	159	8	1309	3079	1	1762	0	2298

TRINITY_DN15742_c0_g1_i2	Glyma.14G031300.1	98.7654321	158	7	746	2513	1	1769	0	2329

TRINITY_DN15403_c0_g4_i1	Glyma.14G018300.1	97.95918367	153	5	143	1904	3	1773	0	2331

TRINITY_DN15337_c0_g2_i1	Glyma.18G037900.3	96.31675875	147	8	1309	3079	1	1756	0	2326

TRINITY_DN15337_c0_g2_i1	Glyma.18G037900.1	95.4887218	147	8	1309	3079	1	1756	0	2326

TRINITY_DN15337_c0_g2_i1	Glyma.18G037900.6	98.55072464	147	8	1309	3079	1	1756	0	2326

TRINITY_DN14204_c0_g1_i2	Glyma.01G175600.3	100	143	9	145	1918	1	1750	0	2329

TRINITY_DN10865_c0_g1_i4	Glyma.02G229900.1	97.83693844	138	23	200	1942	1767	1	0	2266

TRINITY_DN12786_c0_g1_i1	Glyma.07G272700.3	99.20634921	137	0	386	2159	3	1776	0	2518

TRINITY_DN12786_c0_g1_i1	Glyma.07G272700.2	99.20634921	137	0	386	2159	3	1776	0	2518

TRINITY_DN14038_c0_g1_i1	Glyma.20G202700.1	100	132	2	257	2030	1	1768	0	2507

TRINITY_DN14038_c0_g1_i1	Glyma.20G202700.4	96.13733906	132	3	257	2030	1	1765	0	2486

TRINITY_DN12261_c0_g1_i2	Glyma.08G118400.1	100	126	4	42	1814	37	1803	0	2527

TRINITY_DN14204_c0_g1_i1	Glyma.01G175600.3	100	110	9	145	1918	1	1750	0	2512

TRINITY_DN15403_c0_g1_i1	Glyma.14G018300.1	95.25862069	97	3	143	1916	3	1773	0	2719

TRINITY_DN16360_c0_g4_i4	Glyma.08G193500.1	95	94	4	329	2101	1	1770	0	2723

TRINITY_DN10472_c0_g1_i1	Glyma.05G101900.1	97.86729858	277	19	299	2063	311	2058	0	1526

TRINITY_DN11113_c0_g1_i1	Glyma.08G266500.2	100	263	22	350	2112	11	1761	0	1611

TRINITY_DN11113_c0_g1_i1	Glyma.08G266500.1	98.73417722	263	22	350	2112	11	1761	0	1611

TRINITY_DN11113_c0_g1_i2	Glyma.08G266500.2	95	263	22	320	2082	11	1761	0	1611

TRINITY_DN11113_c0_g1_i2	Glyma.08G266500.1	100	263	22	320	2082	11	1761	0	1611

TRINITY_DN12617_c0_g1_i2	Glyma.13G104300.2	100	257	8	2462	4222	1347	3119	0	1773

TRINITY_DN12617_c0_g1_i2	Glyma.13G104300.10	100	257	8	2462	4222	1323	3095	0	1773

TRINITY_DN12617_c0_g1_i2	Glyma.13G104300.8	100	257	8	2462	4222	1221	2993	0	1773

TRINITY_DN16132_c0_g1_i4	Glyma.06G056400.2	96.21212121	230	2	721	2492	84	1855	0	1988

TRINITY_DN11323_c0_g1_i2	Glyma.17G139400.1	98.40425532	230	3	249	2017	1772	1	0	1967

TRINITY_DN11419_c0_g1_i1	Glyma.04G222000.1	100	194	0	358	2130	1773	1	0	2200

TRINITY_DN15133_c0_g1_i10	Glyma.02G293200.1	97.08737864	163	14	579	2340	402	2145	0	2113

TRINITY_DN15133_c0_g1_i2	Glyma.02G293200.1	97.85714286	163	14	1448	3209	402	2145	0	2113

TRINITY_DN15133_c0_g1_i4	Glyma.02G293200.1	96.66666667	163	14	1309	3070	402	2145	0	2113

TRINITY_DN15133_c0_g1_i6	Glyma.02G293200.1	98.39228296	163	14	729	2490	402	2145	0	2113

TRINITY_DN15133_c0_g1_i7	Glyma.02G293200.1	100	163	14	1298	3059	402	2145	0	2113

TRINITY_DN15133_c0_g1_i10	Glyma.02G293200.2	97.08737864	163	14	579	2340	177	1920	0	2113

TRINITY_DN15133_c0_g1_i2	Glyma.02G293200.2	97.85407725	163	14	1448	3209	177	1920	0	2113

TRINITY_DN15133_c0_g1_i4	Glyma.02G293200.2	99.45652174	163	14	1309	3070	177	1920	0	2113

TRINITY_DN15133_c0_g1_i6	Glyma.02G293200.2	100	163	14	729	2490	177	1920	0	2113

TRINITY_DN15133_c0_g1_i7	Glyma.02G293200.2	100	163	14	1298	3059	177	1920	0	2113

TRINITY_DN15742_c0_g1_i2	Glyma.02G283300.3	96.17834395	160	5	746	2513	1	1769	0	2329

TRINITY_DN15742_c0_g1_i2	Glyma.02G283300.2	95.54565702	160	5	746	2513	1	1769	0	2329

TRINITY_DN15742_c0_g1_i2	Glyma.02G283300.1	100	160	5	746	2513	1	1769	0	2329

TRINITY_DN13058_c0_g1_i2	Glyma.01G145400.1	100	153	1	47	1819	3224	1455	0	2407

TRINITY_DN15866_c0_g1_i11	Glyma.15G053000.6	98.92473118	147	5	1284	3053	1	1767	0	2401

TRINITY_DN15866_c0_g1_i11	Glyma.15G053000.1	97.86476868	147	5	1284	3053	1	1767	0	2401

TRINITY_DN15866_c0_g1_i11	Glyma.15G053000.7	100	147	5	1284	3053	1	1767	0	2401

TRINITY_DN15866_c0_g1_i2	Glyma.15G053000.6	99.28741093	147	5	1193	2962	1	1767	0	2401

TRINITY_DN15866_c0_g1_i2	Glyma.15G053000.1	99.28741093	147	5	1193	2962	1	1767	0	2401

TRINITY_DN15866_c0_g1_i2	Glyma.15G053000.7	96.94656489	147	5	1193	2962	1	1767	0	2401

TRINITY_DN15866_c0_g1_i8	Glyma.15G053000.6	99.57627119	147	5	1333	3102	1	1767	0	2401

TRINITY_DN15866_c0_g1_i8	Glyma.15G053000.1	99.57754979	147	5	1333	3102	1	1767	0	2401

TRINITY_DN15866_c0_g1_i8	Glyma.15G053000.7	99.57754979	147	5	1333	3102	1	1767	0	2401

TRINITY_DN15866_c0_g1_i9	Glyma.15G053000.6	100	147	5	1424	3193	1	1767	0	2401

TRINITY_DN15866_c0_g1_i9	Glyma.15G053000.1	100	147	5	1424	3193	1	1767	0	2401

TRINITY_DN15866_c0_g1_i9	Glyma.15G053000.7	100	147	5	1424	3193	1	1767	0	2401

TRINITY_DN15866_c0_g1_i11	Glyma.15G053000.3	96.21212121	147	6	1284	3053	1	1761	0	2362

TRINITY_DN15866_c0_g1_i2	Glyma.15G053000.3	100	147	6	1193	2962	1	1761	0	2362

TRINITY_DN15866_c0_g1_i8	Glyma.15G053000.3	97.36842105	147	6	1333	3102	1	1761	0	2362

TRINITY_DN15866_c0_g1_i9	Glyma.15G053000.3	97.83783784	147	6	1424	3193	1	1761	0	2362

TRINITY_DN16324_c0_g1_i2	Glyma.17G069100.2	100	143	4	203	1969	1773	1	0	2444

TRINITY_DN16324_c0_g1_i4	Glyma.17G069100.2	100	143	4	203	1969	1773	1	0	2444

TRINITY_DN14210_c0_g2_i1	Glyma.18G216300.1	99	135	22	320	2053	1	1758	0	2178

TRINITY_DN39905_c0_g1_i2	Glyma.09G022200.1	98.05996473	106	2	1	1772	201	1972	0	2675

TRINITY_DN39905_c0_g1_i5	Glyma.09G022200.1	98.05825243	106	2	1	1772	201	1972	0	2675

TRINITY_DN39905_c0_g1_i6	Glyma.09G022200.1	98.05825243	106	2	1	1772	201	1972	0	2675

TRINITY_DN15321_c1_g3_i1	Glyma.18G016400.1	98.63636364	263	7	265	2032	63	1821	0	1705

TRINITY_DN15282_c0_g1_i1	Glyma.08G107100.2	95.23809524	234	13	1251	2991	2447	686	0	1711

TRINITY_DN15282_c0_g1_i1	Glyma.08G107100.3	97.13114754	234	13	1251	2991	2447	686	0	1711

TRINITY_DN15282_c0_g1_i1	Glyma.08G107100.1	97.13114754	234	13	1251	2991	2447	686	0	1711

TRINITY_DN15282_c0_g1_i2	Glyma.08G107100.2	96.26865672	234	13	1320	3060	2447	686	0	1711

TRINITY_DN15282_c0_g1_i2	Glyma.08G107100.3	97.22222222	234	13	1320	3060	2447	686	0	1711

TRINITY_DN15282_c0_g1_i2	Glyma.08G107100.1	97.2972973	234	13	1320	3060	2447	686	0	1711

TRINITY_DN15282_c0_g1_i6	Glyma.08G107100.2	97.23756906	234	13	706	2446	2447	686	0	1711

TRINITY_DN15282_c0_g1_i6	Glyma.08G107100.3	97.05882353	234	13	706	2446	2447	686	0	1711

TRINITY_DN15282_c0_g1_i6	Glyma.08G107100.1	100	234	13	706	2446	2447	686	0	1711

TRINITY_DN7614_c0_g1_i1	Glyma.18G294100.1	100	160	2	406	2174	1843	72	0	2366

TRINITY_DN12085_c0_g1_i1	Glyma.04G039800.8	100	153	10	12	1777	1761	1	0	2314

TRINITY_DN12085_c0_g1_i1	Glyma.04G039800.6	100	153	10	12	1777	1761	1	0	2314

TRINITY_DN12085_c0_g1_i1	Glyma.04G039800.5	100	153	10	12	1777	1761	1	0	2314

TRINITY_DN12085_c0_g1_i1	Glyma.04G039800.7	100	153	10	12	1777	1761	1	0	2314

TRINITY_DN12085_c0_g1_i1	Glyma.04G039800.1	100	153	10	12	1777	1761	1	0	2314

TRINITY_DN11696_c0_g2_i1	Glyma.05G089300.3	96.34146341	149	7	115	1879	1	1765	0	2357

TRINITY_DN15100_c0_g1_i2	Glyma.06G321400.1	98.63013699	148	11	238	2001	1764	1	0	2350

TRINITY_DN16438_c0_g1_i7	Glyma.02G101800.1	97.74774775	147	4	132	1899	63	1834	0	2433

TRINITY_DN6730_c0_g1_i1	Glyma.01G039000.1	100	147	5	428	2199	3768	2018	0	2322

TRINITY_DN15626_c0_g4_i1	Glyma.01G021400.1	97.75280899	126	2	193	1961	1851	87	0	2510

TRINITY_DN8790_c0_g2_i1	Glyma.04G087200.1	100	124	4	315	2086	5904	4139	0	2547

TRINITY_DN1320_c0_g1_i1	Glyma.16G101100.1	97.64705882	121	5	1113	2877	243	2013	0	2551

TRINITY_DN8031_c0_g2_i1	Glyma.05G101300.1	99.47368421	343	23	475	2226	1785	25	0	1182

TRINITY_DN12617_c0_g1_i2	Glyma.13G104300.3	100	258	10	2462	4222	1347	3110	0	1731

TRINITY_DN12617_c0_g1_i2	Glyma.13G104300.9	100	258	10	2462	4222	1323	3086	0	1731

TRINITY_DN12617_c0_g1_i2	Glyma.13G104300.7	100	258	10	2462	4222	1221	2984	0	1731

TRINITY_DN2648_c0_g2_i1	Glyma.13G214200.2	100	220	19	237	1988	219	1973	0	1825

TRINITY_DN2648_c0_g2_i1	Glyma.13G214200.1	100	220	19	237	1988	219	1973	0	1825

TRINITY_DN9900_c0_g1_i1	Glyma.08G044300.1	98.33333333	205	17	233	1981	1897	143	0	1890

TRINITY_DN7614_c0_g1_i1	Glyma.18G294100.2	100	160	2	406	2173	1840	70	0	2364

TRINITY_DN36911_c0_g1_i1	Glyma.11G111800.1	96.72131148	160	4	2	1744	550	2320	0	2204

TRINITY_DN42531_c0_g1_i1	Glyma.13G354300.1	100	148	2	453	2222	36	1805	0	2438

TRINITY_DN9899_c0_g1_i3	Glyma.03G256300.2	99.08536585	145	5	27	1795	473	2233	0	2390

TRINITY_DN9899_c0_g1_i3	Glyma.03G256300.1	100	145	5	27	1795	473	2233	0	2390

TRINITY_DN10699_c0_g1_i1	Glyma.08G178700.1	97.39130435	139	7	796	2561	1786	18	0	2455

TRINITY_DN6539_c0_g1_i2	Glyma.05G230700.1	99.62121212	134	6	469	2234	1814	45	0	2490

TRINITY_DN13962_c1_g2_i1	Glyma.03G220300.1	97.41935484	122	5	477	2246	1764	1	0	2543

TRINITY_DN177_c0_g2_i1	Glyma.18G216600.1	98.86363636	115	3	670	2439	1767	1	0	2601

TRINITY_DN177_c0_g2_i1	Glyma.09G272500.1	98.86363636	106	3	670	2439	1767	1	0	2651

TRINITY_DN14823_c0_g1_i4	Glyma.08G264800.1	100	104	0	421	2191	1771	1	0	2695

TRINITY_DN6151_c0_g1_i1	Glyma.04G039800.8	95.87628866	330	36	354	2079	1	1750	0	1027

TRINITY_DN6151_c0_g1_i1	Glyma.04G039800.6	98.38709677	330	36	354	2079	1	1750	0	1027

TRINITY_DN6151_c0_g1_i1	Glyma.04G039800.5	100	330	36	354	2079	1	1750	0	1027

TRINITY_DN6151_c0_g1_i1	Glyma.04G039800.7	100	330	36	354	2079	1	1750	0	1027

TRINITY_DN6151_c0_g1_i1	Glyma.04G039800.1	100	330	36	354	2079	1	1750	0	1027

TRINITY_DN14371_c0_g3_i1	Glyma.06G191200.1	100	289	23	258	2010	1	1753	0	1448

TRINITY_DN14371_c0_g3_i1	Glyma.06G191200.3	100	289	23	258	2010	1	1753	0	1448

TRINITY_DN14371_c0_g3_i1	Glyma.06G191200.2	95.34883721	289	23	258	2010	1	1753	0	1448

TRINITY_DN14371_c0_g3_i1	Glyma.06G191200.4	95.34883721	289	23	258	2010	1	1753	0	1448

TRINITY_DN14265_c0_g1_i1	Glyma.16G102400.1	95	285	26	456	2210	1779	37	0	1419

TRINITY_DN16353_c0_g1_i2	Glyma.19G068800.1	96.34489222	199	12	714	2463	695	2459	0	2004

TRINITY_DN16353_c0_g1_i7	Glyma.19G068800.1	99.36305732	199	12	796	2545	695	2459	0	2004

TRINITY_DN15886_c3_g1_i4	Glyma.09G082000.1	96.4028777	181	2	1	1770	1336	3099	0	2228

TRINITY_DN15886_c3_g1_i4	Glyma.09G082000.2	97.2972973	181	2	1	1770	1336	3099	0	2228

TRINITY_DN3280_c0_g1_i1	Glyma.02G164400.1	97.2972973	169	0	146	1915	1770	1	0	2333

TRINITY_DN10699_c0_g1_i1	Glyma.15G053800.1	98.24561404	150	5	796	2561	1786	18	0	2405

TRINITY_DN13769_c0_g1_i1	Glyma.16G024200.1	96.34146341	139	2	545	2311	1	1770	0	2479

TRINITY_DN5400_c0_g1_i1	Glyma.14G122600.1	98.24561404	135	1	355	2124	1767	1	0	2501

TRINITY_DN4106_c0_g1_i1	Glyma.11G232900.4	97.10982659	133	0	69	1838	1	1770	0	2532

TRINITY_DN15726_c0_g1_i1	Glyma.15G127200.1	100	129	2	270	2033	1770	1	0	2516

TRINITY_DN15726_c0_g1_i3	Glyma.15G127200.1	100	129	2	270	2033	1770	1	0	2516

TRINITY_DN15726_c0_g1_i5	Glyma.15G127200.1	99.19354839	129	2	270	2033	1770	1	0	2516

TRINITY_DN15726_c0_g1_i7	Glyma.15G127200.1	98	129	2	270	2033	1770	1	0	2516

TRINITY_DN13962_c1_g2_i1	Glyma.19G217200.1	97.41935484	127	2	477	2246	1767	1	0	2545

TRINITY_DN15726_c0_g1_i1	Glyma.09G020800.1	100	126	2	270	2033	1770	1	0	2532

TRINITY_DN15726_c0_g1_i3	Glyma.09G020800.1	100	126	2	270	2033	1770	1	0	2532

TRINITY_DN15726_c0_g1_i5	Glyma.09G020800.1	96.15384615	126	2	270	2033	1770	1	0	2532

TRINITY_DN15726_c0_g1_i7	Glyma.09G020800.1	100	126	2	270	2033	1770	1	0	2532

TRINITY_DN13413_c0_g1_i1	Glyma.04G074200.2	96.36363636	118	5	478	2245	1760	1	0	2538

TRINITY_DN13702_c0_g1_i1	Glyma.08G043900.1	100	115	8	323	2090	1	1762	0	2567

TRINITY_DN4106_c0_g1_i1	Glyma.18G024200.1	98.68421053	107	0	69	1838	1	1770	0	2676

TRINITY_DN7905_c0_g1_i1	Glyma.04G144600.2	95.43147208	103	3	714	2468	1	1770	0	2601

TRINITY_DN7905_c0_g1_i2	Glyma.04G144600.2	95.45454545	103	3	613	2367	1	1770	0	2601

TRINITY_DN10350_c0_g1_i1	Glyma.05G136800.1	96.6442953	102	0	324	2093	1806	37	0	2704

TRINITY_DN14167_c0_g1_i1	Glyma.04G203800.1	95.53264605	102	5	396	2165	1755	1	0	2606

TRINITY_DN11746_c0_g1_i1	Glyma.09G039900.1	100	424	21	517	2274	1860	106	0	756

TRINITY_DN15910_c0_g1_i1	Glyma.02G011400.1	100	260	4	527	2293	322	2088	0	1801

TRINITY_DN15910_c0_g1_i1	Glyma.02G011400.2	98.26839827	260	4	527	2293	322	2088	0	1801

TRINITY_DN10865_c0_g1_i4	Glyma.11G166000.1	100	251	30	201	1942	1748	1	0	1567

TRINITY_DN15765_c0_g1_i2	Glyma.11G055700.1	95.42857143	217	11	339	2100	243	2004	0	1975

TRINITY_DN11672_c0_g1_i1	Glyma.01G180100.1	100	197	14	1	1755	2303	4066	0	2052

TRINITY_DN11672_c0_g1_i1	Glyma.01G180100.2	99.08256881	197	14	1	1755	2303	4066	0	2052

TRINITY_DN11672_c0_g1_i1	Glyma.01G180100.3	96.64570231	197	14	1	1755	2303	4066	0	2052

TRINITY_DN11672_c0_g1_i1	Glyma.01G180100.4	100	197	14	1	1755	1346	3109	0	2052

TRINITY_DN14389_c0_g1_i4	Glyma.18G033700.1	100	189	6	111	1872	3	1770	0	2169

TRINITY_DN11672_c0_g1_i1	Glyma.11G062100.3	98.73417722	182	14	3	1755	2164	3925	0	2109

TRINITY_DN11672_c0_g1_i1	Glyma.11G062100.1	98.63013699	182	14	3	1755	2164	3925	0	2109

TRINITY_DN11672_c0_g1_i1	Glyma.11G062100.6	96.31578947	182	14	3	1755	2062	3823	0	2109

TRINITY_DN11672_c0_g1_i1	Glyma.11G062100.4	95.15418502	182	14	3	1755	2062	3823	0	2109

TRINITY_DN11672_c0_g1_i1	Glyma.11G062100.5	96.82539683	182	14	3	1755	2059	3820	0	2109

TRINITY_DN11672_c0_g1_i1	Glyma.11G062100.2	98.30508475	182	14	3	1755	2059	3820	0	2109

TRINITY_DN9374_c0_g1_i1	Glyma.08G156900.1	96.875	142	24	164	1903	42	1789	0	2158

TRINITY_DN177_c0_g1_i1	Glyma.18G216600.2	100	115	3	794	2561	1765	1	0	2597

TRINITY_DN177_c0_g2_i1	Glyma.18G216600.2	98.86363636	115	3	672	2439	1765	1	0	2597

TRINITY_DN177_c0_g1_i1	Glyma.09G272500.2	100	106	3	794	2561	1765	1	0	2647

TRINITY_DN177_c0_g2_i1	Glyma.09G272500.2	98.85550787	106	3	672	2439	1765	1	0	2647

TRINITY_DN16132_c0_g1_i1	Glyma.14G098600.2	96.07843137	105	0	1174	2942	432	2200	0	2686

TRINITY_DN38438_c0_g1_i1	Glyma.02G302300.1	100	362	27	328	2076	111	1847	0	937

TRINITY_DN10472_c0_g1_i1	Glyma.17G165000.1	97.66536965	279	11	299	2063	161	1905	0	1552

TRINITY_DN15288_c0_g1_i1	Glyma.06G017400.1	98.13664596	279	42	35	1746	1	1751	0	1247

TRINITY_DN15910_c0_g1_i1	Glyma.10G012000.1	100	274	2	527	2293	328	2094	0	1735

TRINITY_DN15772_c0_g1_i2	Glyma.19G039100.2	97.43589744	237	4	182	1947	43	1808	0	1927

TRINITY_DN15772_c0_g1_i9	Glyma.19G039100.2	96.05263158	237	4	197	1962	43	1808	0	1927

TRINITY_DN15642_c0_g1_i6	Glyma.12G017600.1	100	217	6	179	1945	1669	3426	0	1991

TRINITY_DN15642_c0_g1_i6	Glyma.12G017600.2	98.33333333	217	6	179	1945	1669	3426	0	1991

TRINITY_DN2233_c0_g1_i1	Glyma.19G092100.1	95.85798817	179	7	397	2160	1	1764	0	2222

TRINITY_DN15893_c0_g4_i11	Glyma.09G225700.5	100	166	16	733	2473	344	2096	0	2074

TRINITY_DN15893_c0_g4_i11	Glyma.09G225700.6	100	166	16	733	2473	344	2096	0	2074

TRINITY_DN15893_c0_g4_i11	Glyma.09G225700.1	98.70967742	166	16	733	2473	344	2096	0	2074

TRINITY_DN15893_c0_g4_i3	Glyma.09G225700.5	95.03546099	166	16	733	2473	344	2096	0	2074

TRINITY_DN15893_c0_g4_i3	Glyma.09G225700.6	98.55072464	166	16	733	2473	344	2096	0	2074

TRINITY_DN15893_c0_g4_i3	Glyma.09G225700.1	96.37681159	166	16	733	2473	344	2096	0	2074

TRINITY_DN15893_c0_g4_i4	Glyma.09G225700.5	100	166	16	1168	2908	344	2096	0	2074

TRINITY_DN15893_c0_g4_i4	Glyma.09G225700.6	95.54140127	166	16	1168	2908	344	2096	0	2074

TRINITY_DN15893_c0_g4_i4	Glyma.09G225700.1	100	166	16	1168	2908	344	2096	0	2074

TRINITY_DN16189_c1_g3_i1	Glyma.08G008400.1	99.05660377	146	2	1	1767	957	2723	0	2444

TRINITY_DN16189_c1_g3_i1	Glyma.08G008400.2	100	146	2	1	1767	957	2723	0	2444

TRINITY_DN37476_c0_g1_i1	Glyma.15G224500.1	96.1038961	134	2	180	1946	568	2334	0	2510

TRINITY_DN1320_c0_g1_i1	Glyma.03G064400.1	100	134	4	1113	2877	357	2115	0	2446

TRINITY_DN13832_c0_g3_i1	Glyma.19G191600.1	96.2962963	127	6	316	2078	2412	650	0	2497

TRINITY_DN13832_c0_g3_i1	Glyma.19G191600.2	100	127	6	316	2078	2412	650	0	2497

TRINITY_DN13832_c0_g3_i1	Glyma.03G191000.2	100	126	5	316	2078	2418	653	0	2521

TRINITY_DN13832_c0_g3_i1	Glyma.03G191000.1	96.2962963	126	5	316	2078	2418	653	0	2521

TRINITY_DN15139_c0_g3_i1	Glyma.16G111700.5	100	116	2	321	2088	2750	989	0	2584

TRINITY_DN15139_c0_g3_i1	Glyma.16G111700.3	99.09090909	116	2	321	2088	2750	989	0	2584

TRINITY_DN15139_c0_g3_i1	Glyma.16G111700.2	98.25581395	116	2	321	2088	2750	989	0	2584

TRINITY_DN15139_c0_g3_i1	Glyma.16G111700.4	95.35864979	116	2	321	2088	2750	989	0	2584

TRINITY_DN15139_c0_g3_i1	Glyma.16G111700.1	100	116	2	321	2088	2750	989	0	2584

TRINITY_DN14088_c0_g1_i1	Glyma.04G242100.1	100	328	24	146	1872	1791	35	0	1123

TRINITY_DN14088_c0_g1_i1	Glyma.04G242100.2	100	328	24	146	1872	1791	35	0	1123

TRINITY_DN14088_c0_g1_i2	Glyma.04G242100.1	95.23809524	328	24	146	1872	1791	35	0	1123

TRINITY_DN14088_c0_g1_i2	Glyma.04G242100.2	95.23809524	328	24	146	1872	1791	35	0	1123

TRINITY_DN6769_c0_g1_i2	Glyma.15G160700.3	98.88888889	279	15	252	2016	68	1814	0	1576

TRINITY_DN6769_c0_g1_i2	Glyma.15G160700.4	98.88888889	279	15	252	2016	68	1814	0	1576

TRINITY_DN6769_c0_g1_i2	Glyma.15G160700.2	98.88888889	279	15	252	2016	68	1814	0	1576

TRINITY_DN14702_c0_g1_i1	Glyma.01G224000.1	99.00990099	247	20	282	2018	1902	136	0	1701

TRINITY_DN14702_c0_g1_i1	Glyma.01G224000.2	96.3060686	247	20	282	2018	1770	4	0	1701

TRINITY_DN15681_c0_g2_i1	Glyma.09G035400.1	95.75289575	237	11	627	2385	206	1958	0	1808

TRINITY_DN13349_c1_g3_i2	Glyma.08G309000.3	99.15966387	208	17	716	2458	1772	24	0	1840

TRINITY_DN16379_c0_g2_i3	Glyma.03G182800.1	97.36842105	198	0	315	2081	1767	1	0	2167

TRINITY_DN16379_c0_g2_i3	Glyma.19G183400.1	98.55072464	194	0	315	2081	1767	1	0	2189

TRINITY_DN2233_c0_g1_i1	Glyma.16G055800.1	96.15384615	194	4	397	2160	1	1764	0	2150

TRINITY_DN16189_c1_g3_i2	Glyma.08G008400.1	96.93877551	186	0	1	1767	957	2723	0	2233

TRINITY_DN16189_c1_g3_i2	Glyma.08G008400.2	95.30201342	186	0	1	1767	957	2723	0	2233

TRINITY_DN13080_c0_g1_i1	Glyma.13G137200.4	95.38461538	153	8	276	2027	111	1873	0	2292

TRINITY_DN13080_c0_g1_i1	Glyma.13G137200.5	95.38461538	153	8	276	2027	111	1873	0	2292

TRINITY_DN13080_c0_g1_i1	Glyma.13G137200.6	95.3038674	153	8	276	2027	111	1873	0	2292

TRINITY_DN13080_c0_g1_i1	Glyma.13G137200.1	97.8021978	153	8	276	2027	111	1873	0	2292

TRINITY_DN13080_c0_g1_i2	Glyma.13G137200.4	100	153	8	447	2198	111	1873	0	2292

TRINITY_DN13080_c0_g1_i2	Glyma.13G137200.5	97.39583333	153	8	447	2198	111	1873	0	2292

TRINITY_DN13080_c0_g1_i2	Glyma.13G137200.6	97.05882353	153	8	447	2198	111	1873	0	2292

TRINITY_DN13080_c0_g1_i2	Glyma.13G137200.1	96.21212121	153	8	447	2198	111	1873	0	2292

TRINITY_DN13080_c0_g1_i1	Glyma.13G137200.3	97.8021978	153	9	276	2027	111	1870	0	2274

TRINITY_DN13080_c0_g1_i1	Glyma.13G137200.2	97.8021978	153	9	276	2027	111	1870	0	2274

TRINITY_DN13080_c0_g1_i2	Glyma.13G137200.3	96.73590504	153	9	447	2198	111	1870	0	2274

TRINITY_DN13080_c0_g1_i2	Glyma.13G137200.2	100	153	9	447	2198	111	1870	0	2274

TRINITY_DN14371_c0_g3_i1	Glyma.17G098200.1	98.92473118	143	5	258	2012	1	1767	0	2394

TRINITY_DN6114_c0_g1_i1	Glyma.10G017700.1	98.42519685	138	9	79	1830	1	1755	0	2324

TRINITY_DN16379_c0_g2_i3	Glyma.13G143600.1	98.55072464	136	0	315	2081	1767	1	0	2510

TRINITY_DN37476_c0_g1_i1	Glyma.13G187000.1	96.07843137	129	0	180	1946	571	2337	0	2549

TRINITY_DN12159_c0_g1_i1	Glyma.02G166300.1	100	129	10	109	1866	1	1755	0	2412

TRINITY_DN12159_c0_g1_i2	Glyma.02G166300.1	95.12195122	129	10	109	1866	1	1755	0	2412

TRINITY_DN12878_c0_g1_i3	Glyma.18G025500.5	100	128	0	752	2518	3	1769	0	2555

TRINITY_DN15642_c0_g1_i6	Glyma.01G232500.2	96	126	0	179	1945	1666	3432	0	2566

TRINITY_DN15642_c0_g1_i6	Glyma.01G232500.1	95.7278481	126	0	179	1945	1666	3432	0	2566

TRINITY_DN15642_c0_g1_i6	Glyma.11G010400.1	98.8372093	118	0	179	1945	1666	3432	0	2610

TRINITY_DN11644_c0_g2_i1	Glyma.13G135900.5	100	112	1	313	2076	1767	1	0	2623

TRINITY_DN11644_c0_g2_i2	Glyma.13G135900.5	98.61111111	112	1	313	2076	1767	1	0	2623

TRINITY_DN16382_c0_g3_i2	Glyma.11G169700.1	96.6442953	106	0	177	1943	1	1767	0	2676

TRINITY_DN16382_c0_g3_i2	Glyma.11G169700.2	96.64082687	105	1	177	1943	1	1758	0	2623

TRINITY_DN16382_c0_g3_i2	Glyma.18G060600.3	98.42519685	103	0	177	1943	1	1767	0	2693

TRINITY_DN16382_c0_g3_i2	Glyma.18G060600.1	98.42519685	103	0	177	1943	1	1767	0	2693

TRINITY_DN15938_c0_g1_i1	Glyma.19G082800.3	95.54140127	102	0	171	1937	1	1767	0	2699

TRINITY_DN15938_c0_g1_i1	Glyma.19G082800.1	98.17518248	102	0	171	1937	1	1767	0	2699

TRINITY_DN15938_c0_g1_i1	Glyma.19G082800.2	98.17629179	102	0	171	1937	1	1767	0	2699

TRINITY_DN14874_c0_g2_i1	Glyma.07G046000.1	100	211	8	293	2050	3558	1795	0	2028

TRINITY_DN15493_c0_g1_i2	Glyma.14G047200.1	96.25	139	2	857	2622	1763	1	0	2471

TRINITY_DN6596_c0_g1_i1	Glyma.10G146600.1	100	137	13	250	1998	1	1764	0	2379

TRINITY_DN6596_c0_g1_i1	Glyma.10G146600.3	100	137	13	250	1998	1	1764	0	2379

TRINITY_DN16415_c1_g1_i2	Glyma.11G147900.1	98.14814815	135	4	2621	4385	1767	5	0	2488

TRINITY_DN16415_c1_g1_i9	Glyma.11G147900.1	98.98477157	135	4	2527	4291	1767	5	0	2488

TRINITY_DN9940_c0_g1_i1	Glyma.04G115500.2	95.94594595	134	13	355	2092	1	1759	0	2292

TRINITY_DN9940_c0_g1_i1	Glyma.04G115500.1	99.07407407	134	13	355	2092	1	1759	0	2292

TRINITY_DN9940_c0_g1_i2	Glyma.04G115500.2	95.95959596	134	13	572	2309	1	1759	0	2292

TRINITY_DN9940_c0_g1_i2	Glyma.04G115500.1	95.96412556	134	13	572	2309	1	1759	0	2292

TRINITY_DN9940_c0_g1_i3	Glyma.04G115500.2	95.9553696	134	13	337	2074	1	1759	0	2292

TRINITY_DN9940_c0_g1_i3	Glyma.04G115500.1	95.94594595	134	13	337	2074	1	1759	0	2292

TRINITY_DN15493_c0_g1_i2	Glyma.02G269700.1	96.15384615	133	0	857	2622	1766	1	0	2525

TRINITY_DN2513_c0_g1_i1	Glyma.01G149800.2	98.91304348	128	1	219	1981	1	1766	0	2532

TRINITY_DN2513_c0_g1_i1	Glyma.01G149800.3	98.91304348	128	1	219	1981	1	1766	0	2532

TRINITY_DN2513_c0_g2_i1	Glyma.01G149800.2	100	128	1	219	1981	1	1766	0	2532

TRINITY_DN2513_c0_g2_i1	Glyma.01G149800.3	98.55072464	128	1	219	1981	1	1766	0	2532

TRINITY_DN11806_c0_g2_i1	Glyma.17G028300.1	100	126	4	255	2015	1809	46	0	2518

TRINITY_DN11806_c0_g2_i2	Glyma.17G028300.1	100	126	4	398	2158	1809	46	0	2518

TRINITY_DN14294_c0_g1_i2	Glyma.13G207900.1	96.20253165	124	12	36	1794	1758	1	0	2477

TRINITY_DN10865_c0_g1_i3	Glyma.14G196600.1	96.26168224	109	17	163	1892	1760	1	0	2386

TRINITY_DN9782_c0_g1_i1	Glyma.18G149700.1	98.30508475	97	2	278	2043	1782	23	0	2686

TRINITY_DN15464_c0_g1_i3	Glyma.05G016700.2	99.18032787	92	4	448	2210	1	1722	0	2447

TRINITY_DN15464_c0_g1_i3	Glyma.05G016700.3	99.18032787	92	4	448	2210	1	1722	0	2447

TRINITY_DN15464_c0_g1_i3	Glyma.05G016700.4	100	92	4	448	2210	1	1722	0	2447

TRINITY_DN15464_c0_g1_i3	Glyma.05G016700.5	100	92	4	448	2210	1	1722	0	2447

TRINITY_DN16345_c0_g1_i6	Glyma.09G115100.3	95.44513458	398	47	477	2218	3695	1963	0	699

TRINITY_DN6691_c0_g2_i1	Glyma.11G069300.2	98.75	387	30	400	2147	32	1773	0	857

TRINITY_DN6691_c0_g2_i1	Glyma.11G069300.4	99.13043478	387	30	400	2147	32	1773	0	857

TRINITY_DN6691_c0_g2_i1	Glyma.11G069300.3	100	387	30	400	2147	32	1773	0	857

TRINITY_DN13326_c0_g2_i1	Glyma.09G054200.1	98.42519685	280	12	126	1877	1758	1	0	1580

TRINITY_DN14874_c0_g2_i1	Glyma.16G014000.1	99.58677686	210	9	293	2050	1761	1	0	2025

TRINITY_DN9121_c0_g1_i1	Glyma.04G196400.1	97.19626168	187	18	147	1909	10	1745	0	2023

TRINITY_DN11419_c0_g1_i1	Glyma.06G143400.1	97.65100671	182	2	367	2130	1764	1	0	2239

TRINITY_DN15100_c0_g1_i2	Glyma.04G113700.1	95.52845528	154	5	238	2001	1758	1	0	2355

TRINITY_DN5501_c0_g1_i1	Glyma.20G197500.1	95.33527697	130	5	300	2054	1	1764	0	2468

TRINITY_DN5501_c0_g1_i1	Glyma.10G193000.1	98.6970684	128	5	300	2054	1	1764	0	2479

TRINITY_DN5501_c0_g1_i1	Glyma.10G193000.3	98.7012987	128	5	300	2054	1	1764	0	2479

TRINITY_DN12347_c0_g1_i1	Glyma.02G245400.1	100	127	28	50	1787	4	1744	0	2226

TRINITY_DN5679_c0_g1_i1	Glyma.02G157700.1	100	110	5	1	1747	2189	3949	0	2508

TRINITY_DN9782_c0_g1_i1	Glyma.03G083100.1	98.30985915	101	4	278	2042	1779	24	0	2641

TRINITY_DN13347_c0_g1_i1	Glyma.08G270300.1	97.95918367	252	9	423	2175	41	1802	0	1777

TRINITY_DN13347_c0_g1_i2	Glyma.08G270300.1	98.21428571	252	9	423	2175	41	1802	0	1777

TRINITY_DN13347_c0_g1_i3	Glyma.08G270300.1	95.72192513	252	9	423	2175	41	1802	0	1777

TRINITY_DN16188_c2_g1_i4	Glyma.14G138200.1	95.89041096	245	6	31	1794	4	1758	0	1842

TRINITY_DN16756_c0_g1_i1	Glyma.06G088400.1	96.16724739	231	6	1	1752	1306	3066	0	1881

TRINITY_DN11699_c0_g2_i1	Glyma.01G014000.2	100	199	2	251	2014	1597	3357	0	2135

TRINITY_DN11699_c0_g2_i1	Glyma.01G014000.3	97.14285714	199	2	251	2014	1597	3357	0	2135

TRINITY_DN11699_c0_g2_i1	Glyma.01G014000.1	95.56962025	199	2	251	2014	1597	3357	0	2135

TRINITY_DN11699_c0_g2_i1	Glyma.01G014000.4	96.20253165	199	2	251	2014	1597	3357	0	2135

TRINITY_DN13714_c0_g1_i3	Glyma.13G079900.2	95.09803922	158	11	2	1750	163	1923	0	2266

TRINITY_DN15519_c1_g1_i4	Glyma.20G017100.2	97.86096257	155	0	635	2398	1775	12	0	2399

TRINITY_DN14807_c0_g1_i2	Glyma.20G058500.2	99.13793103	155	12	315	2073	1751	1	0	2283

TRINITY_DN13080_c0_g1_i1	Glyma.10G049600.5	99.07407407	148	3	276	2021	111	1873	0	2314

TRINITY_DN13080_c0_g1_i1	Glyma.10G049600.1	99.07407407	148	3	276	2021	111	1873	0	2314

TRINITY_DN13080_c0_g1_i1	Glyma.10G049600.2	100	148	3	276	2021	111	1873	0	2314

TRINITY_DN13080_c0_g1_i1	Glyma.10G049600.4	97.33840304	148	3	276	2021	111	1873	0	2314

TRINITY_DN13080_c0_g1_i2	Glyma.10G049600.5	100	148	3	447	2192	111	1873	0	2314

TRINITY_DN13080_c0_g1_i2	Glyma.10G049600.1	98.18181818	148	3	447	2192	111	1873	0	2314

TRINITY_DN13080_c0_g1_i2	Glyma.10G049600.2	100	148	3	447	2192	111	1873	0	2314

TRINITY_DN13080_c0_g1_i2	Glyma.10G049600.4	100	148	3	447	2192	111	1873	0	2314

TRINITY_DN13080_c0_g1_i1	Glyma.10G049600.3	95.93495935	147	5	276	2021	111	1870	0	2302

TRINITY_DN13080_c0_g1_i1	Glyma.10G049600.6	98.82352941	147	5	276	2021	111	1870	0	2302

TRINITY_DN13080_c0_g1_i2	Glyma.10G049600.3	98.87640449	147	5	447	2192	111	1870	0	2302

TRINITY_DN13080_c0_g1_i2	Glyma.10G049600.6	98.97959184	147	5	447	2192	111	1870	0	2302

TRINITY_DN14371_c0_g3_i1	Glyma.17G098200.3	99.05660377	143	4	258	2012	1	1764	0	2407

TRINITY_DN14371_c0_g3_i1	Glyma.17G098200.2	97.46835443	143	4	258	2012	1	1764	0	2407

TRINITY_DN15153_c0_g1_i3	Glyma.18G166600.1	99.44134078	130	6	459	2215	1764	5	0	2466

TRINITY_DN14371_c0_g3_i1	Glyma.05G028600.4	96	128	4	258	2012	1	1761	0	2471

TRINITY_DN14371_c0_g3_i1	Glyma.05G028600.1	99.15492958	128	4	258	2012	1	1761	0	2471

TRINITY_DN14371_c0_g3_i1	Glyma.05G028600.3	100	128	6	258	2012	1	1758	0	2451

TRINITY_DN14371_c0_g3_i1	Glyma.05G028600.2	100	128	6	258	2012	1	1758	0	2451

TRINITY_DN6859_c0_g1_i2	Glyma.18G010200.2	97.64918626	115	2	99	1861	1764	2	0	2608

TRINITY_DN14909_c0_g1_i1	Glyma.19G206800.1	95.92944369	115	3	74	1833	2	1764	0	2588

TRINITY_DN2699_c0_g2_i2	Glyma.19G130100.1	98.42519685	107	0	187	1950	1	1764	0	2665

TRINITY_DN15789_c0_g1_i1	Glyma.06G032600.1	98.51851852	314	23	146	1876	870	2615	0	1199

TRINITY_DN15789_c0_g1_i1	Glyma.06G032600.4	100	314	23	146	1876	810	2555	0	1199

TRINITY_DN15789_c0_g1_i1	Glyma.06G032600.2	100	314	23	146	1876	810	2555	0	1199

TRINITY_DN15789_c0_g1_i1	Glyma.06G032600.3	100	314	23	146	1876	594	2339	0	1199

TRINITY_DN9863_c0_g3_i1	Glyma.08G008600.1	96.27959414	267	17	1	1756	1014	2754	0	1589

TRINITY_DN9863_c0_g3_i1	Glyma.08G008600.2	98.30508475	267	17	1	1756	1008	2748	0	1589

TRINITY_DN9863_c0_g3_i1	Glyma.08G008600.3	99.74683544	267	17	1	1756	1008	2748	0	1589

TRINITY_DN14525_c0_g2_i1	Glyma.16G137800.1	95.93495935	209	16	1	1744	658	2404	0	1871

TRINITY_DN16237_c0_g1_i15	Glyma.08G008100.2	96.61971831	154	10	904	2652	31	1782	0	2241

TRINITY_DN16237_c0_g1_i2	Glyma.08G008100.2	100	154	10	1008	2756	31	1782	0	2241

TRINITY_DN15893_c0_g4_i5	Glyma.09G225700.5	100	151	14	733	2468	344	2097	0	2187

TRINITY_DN15893_c0_g4_i5	Glyma.09G225700.6	100	151	14	733	2468	344	2097	0	2187

TRINITY_DN15893_c0_g4_i5	Glyma.09G225700.1	96.76549865	151	14	733	2468	344	2097	0	2187

TRINITY_DN15893_c0_g4_i8	Glyma.09G225700.5	99.05660377	151	14	1168	2903	344	2097	0	2187

TRINITY_DN15893_c0_g4_i8	Glyma.09G225700.6	96.15384615	151	14	1168	2903	344	2097	0	2187

TRINITY_DN15893_c0_g4_i8	Glyma.09G225700.1	99.15492958	151	14	1168	2903	344	2097	0	2187

TRINITY_DN11806_c0_g2_i1	Glyma.17G028300.2	100	125	7	255	2015	1797	46	0	2479

TRINITY_DN11806_c0_g2_i2	Glyma.17G028300.2	100	125	7	398	2158	1797	46	0	2479

TRINITY_DN15850_c3_g2_i5	Glyma.02G235700.2	97.84366577	124	20	724	2442	3	1760	0	2252

TRINITY_DN10792_c0_g1_i1	Glyma.18G132200.1	99.82269504	122	19	526	2277	1740	1	0	2361

TRINITY_DN18214_c0_g1_i1	Glyma.20G193900.1	100	115	22	1	1711	813	2559	0	2180

TRINITY_DN11036_c0_g2_i1	Glyma.05G222500.1	100	108	3	507	2267	1761	1	0	2632

TRINITY_DN11036_c0_g2_i1	Glyma.08G029300.3	100	99	3	507	2267	1761	1	0	2682

TRINITY_DN11036_c0_g2_i1	Glyma.08G029300.2	100	99	3	507	2267	1761	1	0	2682

TRINITY_DN11036_c0_g2_i1	Glyma.08G029300.1	100	99	3	507	2267	1761	1	0	2682

TRINITY_DN2628_c0_g1_i1	Glyma.04G098900.2	100	95	1	387	2149	11	1767	0	2691

TRINITY_DN12978_c0_g1_i1	Glyma.17G188200.1	97.05882353	366	15	228	1980	119	1871	0	1110

TRINITY_DN12978_c0_g1_i2	Glyma.17G188200.1	95.23809524	366	15	224	1976	119	1871	0	1110

TRINITY_DN36438_c0_g2_i1	Glyma.06G204800.1	99.61977186	311	37	506	2239	1809	73	0	1188

TRINITY_DN2139_c0_g1_i1	Glyma.11G252700.4	98.97959184	273	18	429	2174	39	1774	0	1471

TRINITY_DN14389_c0_g1_i4	Glyma.18G034000.1	97.19626168	268	9	89	1831	86	1846	0	1640

TRINITY_DN15828_c0_g1_i1	Glyma.09G015400.1	100	217	18	572	2327	1827	96	0	1820

TRINITY_DN15828_c0_g1_i1	Glyma.09G015400.2	100	217	18	572	2327	1827	96	0	1820

TRINITY_DN15828_c0_g1_i1	Glyma.09G015400.3	97.12230216	217	18	572	2327	1827	96	0	1820

TRINITY_DN15828_c0_g1_i2	Glyma.09G015400.1	98.42519685	217	18	572	2327	1827	96	0	1820

TRINITY_DN15828_c0_g1_i2	Glyma.09G015400.2	98.4962406	217	18	572	2327	1827	96	0	1820

TRINITY_DN15828_c0_g1_i2	Glyma.09G015400.3	99.13043478	217	18	572	2327	1827	96	0	1820

TRINITY_DN15828_c0_g1_i7	Glyma.09G015400.1	95.43378995	217	18	572	2327	1827	96	0	1820

TRINITY_DN15828_c0_g1_i7	Glyma.09G015400.2	97.52066116	217	18	572	2327	1827	96	0	1820

TRINITY_DN15828_c0_g1_i7	Glyma.09G015400.3	100	217	18	572	2327	1827	96	0	1820

TRINITY_DN15828_c0_g1_i8	Glyma.09G015400.1	95	217	18	572	2327	1827	96	0	1820

TRINITY_DN15828_c0_g1_i8	Glyma.09G015400.2	96.11111111	217	18	572	2327	1827	96	0	1820

TRINITY_DN15828_c0_g1_i8	Glyma.09G015400.3	96.11111111	217	18	572	2327	1827	96	0	1820

TRINITY_DN8031_c0_g2_i1	Glyma.11G051600.1	95.3271028	209	1	475	2235	1782	21	0	2089

TRINITY_DN15430_c0_g1_i1	Glyma.02G097600.1	97.90575916	190	2	284	2044	1764	4	0	2189

TRINITY_DN15430_c0_g1_i1	Glyma.02G097600.2	97.90575916	184	10	284	2044	1743	4	0	2085

TRINITY_DN13157_c0_g1_i2	Glyma.05G245300.1	97.91666667	183	20	1305	3025	1266	3016	0	1905

TRINITY_DN13157_c0_g1_i2	Glyma.05G245300.3	97.91666667	183	20	1305	3025	732	2482	0	1905

TRINITY_DN15893_c0_g4_i11	Glyma.09G225700.2	96.10778443	166	15	733	2473	344	2090	0	2102

TRINITY_DN15893_c0_g4_i11	Glyma.09G225700.4	96.89320388	166	15	733	2473	344	2090	0	2102

TRINITY_DN15893_c0_g4_i11	Glyma.09G225700.3	100	166	15	733	2473	344	2090	0	2102

TRINITY_DN15893_c0_g4_i3	Glyma.09G225700.2	95.41284404	166	15	733	2473	344	2090	0	2102

TRINITY_DN15893_c0_g4_i3	Glyma.09G225700.4	96.20253165	166	15	733	2473	344	2090	0	2102

TRINITY_DN15893_c0_g4_i3	Glyma.09G225700.3	99.52	166	15	733	2473	344	2090	0	2102

TRINITY_DN15893_c0_g4_i4	Glyma.09G225700.2	99.57264957	166	15	1168	2908	344	2090	0	2102

TRINITY_DN15893_c0_g4_i4	Glyma.09G225700.4	95.11201629	166	15	1168	2908	344	2090	0	2102

TRINITY_DN15893_c0_g4_i4	Glyma.09G225700.3	98.6440678	166	15	1168	2908	344	2090	0	2102

TRINITY_DN1607_c0_g2_i1	Glyma.20G187800.1	95.43378995	147	9	138	1893	1	1747	0	2303

TRINITY_DN15664_c0_g2_i8	Glyma.18G266400.2	96.48093842	142	1	1840	3598	1762	1	0	2447

TRINITY_DN15430_c0_g1_i4	Glyma.02G097600.1	100	142	2	284	2044	1764	4	0	2455

TRINITY_DN15430_c0_g1_i4	Glyma.02G097600.2	96.98630137	140	3	284	2044	1743	4	0	2329

TRINITY_DN10644_c0_g1_i1	Glyma.12G078100.1	100	137	4	351	2111	1755	1	0	2444

TRINITY_DN12691_c0_g1_i1	Glyma.11G104500.3	97.32620321	132	3	116	1875	1760	1	0	2497

TRINITY_DN12691_c0_g1_i1	Glyma.11G104500.2	97.32540862	132	3	116	1875	1760	1	0	2497

TRINITY_DN12691_c0_g1_i1	Glyma.12G029500.2	97.32540862	131	3	116	1875	1760	1	0	2503

TRINITY_DN8191_c0_g2_i2	Glyma.03G086000.4	98.7012987	117	1	1	1759	103	1864	0	2586

TRINITY_DN5679_c0_g1_i1	Glyma.02G157700.2	96.2962963	110	3	1	1747	2189	3946	0	2521

TRINITY_DN5679_c0_g1_i1	Glyma.02G157700.3	97.04142012	110	7	1	1747	2189	3937	0	2464

TRINITY_DN13326_c0_g2_i1	Glyma.15G160400.1	95.83333333	266	9	126	1877	1755	1	0	1681

TRINITY_DN13326_c0_g2_i1	Glyma.15G160400.2	98.05825243	266	9	126	1877	1755	1	0	1681

TRINITY_DN18661_c0_g1_i1	Glyma.09G246200.1	98.93048128	252	59	6	1675	1735	9	0	1048

TRINITY_DN14955_c0_g1_i2	Glyma.08G159500.1	96.55172414	235	9	122	1870	1758	1	0	1853

TRINITY_DN15655_c0_g1_i2	Glyma.07G272800.1	99.33333333	197	9	1058	2801	2015	257	0	2037

TRINITY_DN16055_c0_g1_i2	Glyma.05G233400.1	98.41269841	186	42	220	1916	115	1869	0	1770

TRINITY_DN16055_c0_g1_i2	Glyma.05G233400.2	96.42857143	186	42	220	1916	115	1869	0	1770

TRINITY_DN11898_c0_g2_i3	Glyma.02G213400.1	98.75	176	22	147	1896	3051	1329	0	1960

TRINITY_DN5356_c0_g1_i1	Glyma.20G132700.1	96.80851064	162	7	133	1892	128	1878	0	2283

TRINITY_DN13714_c0_g1_i2	Glyma.13G079900.2	100	139	6	2	1750	163	1923	0	2405

TRINITY_DN14348_c0_g2_i1	Glyma.06G316600.1	98.1981982	132	1	1955	3712	1	1761	0	2501

TRINITY_DN14348_c0_g2_i2	Glyma.06G316600.1	95.39473684	132	1	1486	3243	1	1761	0	2501

TRINITY_DN14348_c0_g2_i3	Glyma.06G316600.1	99.09090909	132	1	1938	3695	1	1761	0	2501

TRINITY_DN14348_c0_g2_i4	Glyma.06G316600.1	98	132	1	2667	4424	1	1761	0	2501

TRINITY_DN12717_c0_g1_i2	Glyma.03G244500.14	96.66666667	125	13	3	1762	163	1886	0	2314

TRINITY_DN7798_c0_g1_i1	Glyma.10G281800.1	98.63013699	125	46	79	1758	1716	1	0	1746

TRINITY_DN14909_c0_g1_i1	Glyma.03G209400.1	95.91836735	120	2	74	1833	2	1761	0	2575

TRINITY_DN11552_c0_g1_i1	Glyma.04G256100.1	96.875	113	1	186	1943	31	1791	0	2606

TRINITY_DN11552_c0_g1_i2	Glyma.04G256100.1	100	113	1	223	1980	31	1791	0	2606

TRINITY_DN15748_c0_g1_i1	Glyma.08G283700.1	100	109	2	343	2103	4380	2622	0	2636

TRINITY_DN15938_c0_g1_i1	Glyma.03G236700.3	99.75609756	99	0	177	1937	4	1764	0	2704

TRINITY_DN15938_c0_g1_i1	Glyma.03G236700.1	96.47058824	99	0	177	1937	4	1764	0	2704

TRINITY_DN15938_c0_g1_i1	Glyma.03G236700.2	95.83333333	99	0	177	1937	4	1764	0	2704

TRINITY_DN16098_c0_g1_i2	Glyma.08G126900.1	95.52238806	86	0	530	2290	1773	13	0	2776

TRINITY_DN16098_c0_g1_i2	Glyma.05G168500.1	95.52238806	81	0	530	2290	1773	13	0	2804

TRINITY_DN11713_c0_g1_i1	Glyma.17G045100.1	100	77	5	567	2326	2100	346	0	2780

TRINITY_DN16345_c0_g1_i6	Glyma.09G115100.1	98.31932773	397	47	482	2218	3690	1963	0	695

TRINITY_DN12204_c0_g1_i2	Glyma.06G323600.1	97.74647887	308	34	229	1969	84	1818	0	1260

TRINITY_DN12204_c0_g1_i2	Glyma.06G323600.4	96.42857143	308	34	229	1969	84	1818	0	1260

TRINITY_DN12204_c0_g1_i2	Glyma.06G323600.3	96.42857143	308	34	229	1969	84	1818	0	1260

TRINITY_DN12204_c0_g1_i2	Glyma.06G323600.5	96.42857143	308	34	229	1969	84	1818	0	1260

TRINITY_DN12204_c0_g1_i3	Glyma.06G323600.1	95	308	34	182	1922	84	1818	0	1260

TRINITY_DN12204_c0_g1_i3	Glyma.06G323600.4	95	308	34	182	1922	84	1818	0	1260

TRINITY_DN12204_c0_g1_i3	Glyma.06G323600.3	95	308	34	182	1922	84	1818	0	1260

TRINITY_DN12204_c0_g1_i3	Glyma.06G323600.5	95	308	34	182	1922	84	1818	0	1260

TRINITY_DN18661_c0_g1_i1	Glyma.18G246800.1	96.16182573	243	64	6	1675	1720	9	0	1013

TRINITY_DN10790_c0_g1_i2	Glyma.02G206000.3	100	154	8	248	2001	1	1751	0	2300

TRINITY_DN10790_c0_g1_i2	Glyma.02G206000.4	100	154	8	248	2001	1	1751	0	2300

TRINITY_DN10790_c0_g1_i2	Glyma.02G206000.2	100	154	8	248	2001	1	1751	0	2300

TRINITY_DN10790_c0_g1_i2	Glyma.02G206000.1	100	154	8	248	2001	1	1751	0	2300

TRINITY_DN36295_c0_g1_i1	Glyma.09G157900.2	96.76258993	144	1	680	2436	545	2304	0	2433

TRINITY_DN9117_c0_g1_i1	Glyma.07G212800.2	97.66081871	132	12	403	2140	1765	7	0	2370

TRINITY_DN10644_c0_g1_i1	Glyma.11G195900.3	98.93238434	128	4	353	2111	1753	1	0	2490

TRINITY_DN10644_c0_g1_i1	Glyma.11G195900.2	95.83333333	128	4	353	2111	1753	1	0	2490

TRINITY_DN9940_c0_g1_i1	Glyma.06G320700.1	95.95070423	122	13	355	2092	1	1750	0	2368

TRINITY_DN9940_c0_g1_i2	Glyma.06G320700.1	95.94594595	122	13	572	2309	1	1750	0	2368

TRINITY_DN9940_c0_g1_i3	Glyma.06G320700.1	95.95959596	122	13	337	2074	1	1750	0	2368

TRINITY_DN10865_c0_g1_i3	Glyma.02G229900.1	98.66666667	113	21	163	1892	1754	1	0	2392

TRINITY_DN15285_c1_g1_i1	Glyma.06G152500.1	97.39130435	105	3	68	1821	44	1797	0	2591

TRINITY_DN15285_c1_g1_i1	Glyma.06G152500.2	95.23809524	105	3	68	1821	44	1797	0	2591

TRINITY_DN15748_c0_g1_i1	Glyma.18G142500.1	98.97959184	101	2	344	2103	4379	2622	0	2678

TRINITY_DN15748_c0_g1_i1	Glyma.18G142500.2	97.79507134	101	2	344	2103	4379	2622	0	2678

TRINITY_DN13071_c1_g1_i2	Glyma.04G209700.1	98.18181818	323	26	217	1956	160	1902	0	1232

TRINITY_DN16188_c2_g1_i3	Glyma.14G138200.1	100	235	7	29	1783	4	1758	0	1895

TRINITY_DN15082_c0_g1_i3	Glyma.01G211200.3	98.73417722	168	9	185	1927	1	1758	0	2207

TRINITY_DN14628_c0_g1_i1	Glyma.08G093800.1	97.14285714	163	7	522	2274	1835	83	0	2268

TRINITY_DN14628_c0_g1_i3	Glyma.08G093800.1	99.18699187	163	7	522	2274	1835	83	0	2268

TRINITY_DN14628_c0_g1_i4	Glyma.08G093800.1	99.21259843	163	7	522	2274	1835	83	0	2268

TRINITY_DN14628_c0_g1_i5	Glyma.08G093800.1	97.41935484	163	7	522	2274	1835	83	0	2268

TRINITY_DN14628_c0_g1_i6	Glyma.08G093800.1	100	163	7	522	2274	1835	83	0	2268

TRINITY_DN14628_c0_g1_i2	Glyma.08G093800.1	97.43589744	163	9	522	2262	1835	83	0	2191

TRINITY_DN15430_c0_g1_i4	Glyma.10G275800.1	100	130	2	284	2041	1758	1	0	2516

TRINITY_DN11552_c0_g1_i1	Glyma.06G325300.1	96.15384615	104	0	185	1943	30	1788	0	2673

TRINITY_DN11552_c0_g1_i2	Glyma.06G325300.1	98.00664452	104	0	222	1980	30	1788	0	2673

TRINITY_DN9476_c0_g1_i4	Glyma.07G263100.3	100	102	1	709	2467	2544	806	0	2555

TRINITY_DN16098_c0_g1_i1	Glyma.08G188400.1	100	95	2	459	2216	1758	1	0	2710

TRINITY_DN2732_c0_g1_i1	Glyma.02G144500.5	98.7804878	94	4	110	1864	2637	880	0	2695

TRINITY_DN2732_c0_g1_i1	Glyma.02G144500.1	98.13084112	94	4	110	1864	2637	880	0	2695

TRINITY_DN2732_c0_g1_i1	Glyma.02G144500.4	98.33679834	94	4	110	1864	2637	880	0	2695

TRINITY_DN15778_c0_g1_i1	Glyma.03G152000.1	96.18320611	402	61	1055	2776	1854	136	3.74E-150	534

TRINITY_DN10095_c0_g1_i1	Glyma.06G187400.3	96.55172414	273	23	229	1971	87	1820	0	1482

TRINITY_DN15681_c0_g2_i5	Glyma.09G035400.1	100	271	9	627	2370	206	1958	0	1622

TRINITY_DN9643_c0_g1_i1	Glyma.17G059500.1	100	187	17	64	1815	1	1725	0	1958

TRINITY_DN9643_c0_g1_i1	Glyma.13G100400.1	100	178	15	64	1815	1	1725	0	2008

TRINITY_DN15082_c0_g1_i3	Glyma.11G030500.2	96.65271967	163	6	185	1927	1	1758	0	2246

TRINITY_DN15082_c0_g1_i3	Glyma.11G030500.1	98.6013986	163	6	185	1927	1	1758	0	2246

TRINITY_DN15774_c0_g1_i1	Glyma.04G085900.2	97.69230769	156	5	4	1757	1901	146	0	2344

TRINITY_DN15430_c0_g1_i4	Glyma.20G114000.1	100	138	0	284	2041	1758	1	0	2483

TRINITY_DN10757_c0_g2_i1	Glyma.03G035900.1	95.83333333	128	3	419	2176	1	1746	0	2460

TRINITY_DN15845_c0_g1_i3	Glyma.01G236300.2	100	123	23	678	2387	1	1731	0	2080

TRINITY_DN4713_c0_g1_i1	Glyma.17G115300.1	97.85407725	122	3	72	1817	1	1758	0	2494

TRINITY_DN4460_c0_g1_i1	Glyma.10G284400.2	98.94736842	119	3	188	1936	1758	1	0	2529

TRINITY_DN4460_c0_g1_i1	Glyma.10G284400.1	98.95833333	119	3	188	1936	1758	1	0	2529

TRINITY_DN10757_c0_g2_i1	Glyma.01G132400.1	95.83333333	119	5	419	2176	1	1746	0	2510

TRINITY_DN11552_c0_g1_i1	Glyma.04G256100.2	95.29616725	113	0	186	1943	31	1788	0	2621

TRINITY_DN11552_c0_g1_i1	Glyma.04G256100.3	96.875	113	0	186	1943	31	1788	0	2621

TRINITY_DN11552_c0_g1_i2	Glyma.04G256100.2	98.93617021	113	0	223	1980	31	1788	0	2621

TRINITY_DN11552_c0_g1_i2	Glyma.04G256100.3	97.05882353	113	0	223	1980	31	1788	0	2621

TRINITY_DN9373_c0_g1_i1	Glyma.02G100000.1	99.11504425	107	15	116	1846	4	1746	0	2383

TRINITY_DN9373_c0_g1_i2	Glyma.02G100000.1	95.92326139	107	15	116	1846	4	1746	0	2383

TRINITY_DN15655_c0_g1_i2	Glyma.16G000300.1	99.04534606	106	3	1050	2801	1996	239	0	2621

TRINITY_DN15655_c0_g1_i2	Glyma.12G164100.1	100	96	3	1050	2801	1996	239	0	2676

TRINITY_DN2732_c0_g1_i1	Glyma.02G144500.3	96.34146341	94	4	111	1864	2636	880	0	2693

TRINITY_DN2732_c0_g1_i1	Glyma.02G144500.2	100	94	4	111	1864	2636	880	0	2693

TRINITY_DN15430_c0_g1_i1	Glyma.20G114000.1	97.90419162	80	0	284	2041	1758	1	0	2804

TRINITY_DN15430_c0_g1_i1	Glyma.10G275800.1	97.90356394	75	0	284	2041	1758	1	0	2832

TRINITY_DN12204_c0_g1_i2	Glyma.04G110900.3	96.42857143	321	29	229	1969	78	1812	0	1221

TRINITY_DN12204_c0_g1_i2	Glyma.04G110900.7	96.42857143	321	29	229	1969	78	1812	0	1221

TRINITY_DN12204_c0_g1_i2	Glyma.04G110900.1	96.42857143	321	29	229	1969	78	1812	0	1221

TRINITY_DN12204_c0_g1_i2	Glyma.04G110900.6	96.42857143	321	29	229	1969	78	1812	0	1221

TRINITY_DN12204_c0_g1_i3	Glyma.04G110900.3	95	321	29	182	1922	78	1812	0	1221

TRINITY_DN12204_c0_g1_i3	Glyma.04G110900.7	95.01915709	321	29	182	1922	78	1812	0	1221

TRINITY_DN12204_c0_g1_i3	Glyma.04G110900.1	95	321	29	182	1922	78	1812	0	1221

TRINITY_DN12204_c0_g1_i3	Glyma.04G110900.6	95	321	29	182	1922	78	1812	0	1221

TRINITY_DN9863_c0_g3_i1	Glyma.05G201200.1	97.33333333	265	21	1	1750	1023	2754	0	1570

TRINITY_DN9863_c0_g3_i1	Glyma.05G201200.2	97.33333333	265	21	1	1750	1017	2748	0	1570

TRINITY_DN9863_c0_g3_i1	Glyma.05G201200.3	97.33333333	265	21	1	1750	672	2403	0	1570

TRINITY_DN10865_c0_g1_i3	Glyma.11G166000.1	95.83333333	265	30	163	1892	1736	1	0	1467

TRINITY_DN11672_c0_g1_i1	Glyma.16G143100.2	99.69325153	218	8	2	1753	2328	4076	0	1953

TRINITY_DN11672_c0_g1_i1	Glyma.16G143100.6	98.55072464	218	8	2	1753	2328	4076	0	1953

TRINITY_DN11672_c0_g1_i1	Glyma.16G143100.3	97.88359788	218	8	2	1753	2328	4076	0	1953

TRINITY_DN11672_c0_g1_i1	Glyma.16G143100.7	100	218	8	2	1753	2328	4076	0	1953

TRINITY_DN11672_c0_g1_i1	Glyma.16G143100.4	100	218	8	2	1753	2328	4076	0	1953

TRINITY_DN11672_c0_g1_i1	Glyma.16G143100.5	100	218	8	2	1753	2328	4076	0	1953

TRINITY_DN11672_c0_g1_i1	Glyma.16G143100.1	97.19626168	218	8	2	1753	2328	4076	0	1953

TRINITY_DN15619_c0_g2_i4	Glyma.04G184000.4	99.33110368	216	3	1015	2769	41	1795	0	2023

TRINITY_DN9977_c0_g2_i1	Glyma.18G192400.2	100	163	5	419	2168	2832	1080	0	2270

TRINITY_DN9977_c0_g2_i1	Glyma.18G192400.1	100	163	5	419	2168	2832	1080	0	2270

TRINITY_DN15500_c0_g1_i2	Glyma.09G207200.1	100	160	6	369	2123	1752	1	0	2313

TRINITY_DN15500_c0_g1_i3	Glyma.09G207200.1	96.24413146	160	6	369	2123	1752	1	0	2313

TRINITY_DN10790_c0_g1_i2	Glyma.17G072000.6	100	159	3	248	2001	1	1754	0	2326

TRINITY_DN10790_c0_g1_i2	Glyma.17G072000.5	100	159	3	248	2001	1	1754	0	2326

TRINITY_DN10790_c0_g1_i2	Glyma.17G072000.4	100	159	3	248	2001	1	1754	0	2326

TRINITY_DN10790_c0_g1_i2	Glyma.17G072000.3	100	159	3	248	2001	1	1754	0	2326

TRINITY_DN10790_c0_g1_i2	Glyma.17G072000.2	100	159	3	248	2001	1	1754	0	2326

TRINITY_DN10790_c0_g1_i2	Glyma.17G072000.1	100	159	3	248	2001	1	1754	0	2326

TRINITY_DN10790_c0_g1_i2	Glyma.17G072000.7	100	159	3	248	2001	1	1754	0	2326

TRINITY_DN15893_c0_g4_i5	Glyma.09G225700.2	96.18320611	151	13	733	2468	344	2091	0	2215

TRINITY_DN15893_c0_g4_i5	Glyma.09G225700.4	95.89041096	151	13	733	2468	344	2091	0	2215

TRINITY_DN15893_c0_g4_i5	Glyma.09G225700.3	100	151	13	733	2468	344	2091	0	2215

TRINITY_DN15893_c0_g4_i8	Glyma.09G225700.2	98.61111111	151	13	1168	2903	344	2091	0	2215

TRINITY_DN15893_c0_g4_i8	Glyma.09G225700.4	100	151	13	1168	2903	344	2091	0	2215

TRINITY_DN15893_c0_g4_i8	Glyma.09G225700.3	100	151	13	1168	2903	344	2091	0	2215

TRINITY_DN16360_c0_g4_i8	Glyma.15G023800.1	100	142	1	229	1982	11	1767	0	2438

TRINITY_DN16326_c0_g1_i1	Glyma.08G162200.1	96.96969697	137	9	563	2313	1740	1	0	2337

TRINITY_DN13709_c0_g1_i2	Glyma.01G019100.1	96.41025641	133	2	485	2235	1756	1	0	2462

TRINITY_DN14238_c0_g2_i2	Glyma.08G060100.1	97.6284585	126	0	189	1945	258	2014	0	2547

TRINITY_DN10472_c0_g1_i3	Glyma.05G101900.1	100	279	16	299	2046	311	2058	0	1594

TRINITY_DN10095_c0_g1_i1	Glyma.04G177700.1	100	258	14	229	1973	87	1834	0	1690

TRINITY_DN15617_c0_g2_i2	Glyma.U026800.1	97.18309859	211	7	388	2137	648	2400	0	2015

TRINITY_DN16132_c0_g1_i5	Glyma.14G098600.2	98.57142857	200	5	1174	2928	432	2183	0	2102

TRINITY_DN16132_c0_g1_i5	Glyma.14G098600.1	95.23809524	200	5	1174	2928	432	2183	0	2102

TRINITY_DN16132_c0_g1_i5	Glyma.17G226100.1	100	199	4	1174	2928	432	2183	0	2108

TRINITY_DN16132_c0_g1_i5	Glyma.17G226100.2	97.92207792	199	4	1174	2928	381	2132	0	2108

TRINITY_DN15500_c0_g1_i2	Glyma.01G015000.1	100	163	3	369	2123	1752	1	0	2307

TRINITY_DN15500_c0_g1_i3	Glyma.01G015000.1	99.45054945	163	3	369	2123	1752	1	0	2307

TRINITY_DN16132_c0_g1_i5	Glyma.04G056200.1	100	162	6	1175	2926	436	2187	0	2294

TRINITY_DN14628_c0_g1_i1	Glyma.05G138600.1	97.42268041	160	3	522	2274	1826	74	0	2318

TRINITY_DN14628_c0_g1_i3	Glyma.05G138600.1	95.23809524	160	3	522	2274	1826	74	0	2318

TRINITY_DN14628_c0_g1_i4	Glyma.05G138600.1	99.21259843	160	3	522	2274	1826	74	0	2318

TRINITY_DN14628_c0_g1_i5	Glyma.05G138600.1	97.73550725	160	3	522	2274	1826	74	0	2318

TRINITY_DN14628_c0_g1_i6	Glyma.05G138600.1	99.48453608	160	3	522	2274	1826	74	0	2318

TRINITY_DN14628_c0_g1_i2	Glyma.05G138600.1	100	160	4	522	2262	1826	74	0	2241

TRINITY_DN16058_c2_g2_i4	Glyma.16G027400.1	99.86091794	155	14	65	1809	1499	3240	0	2222

TRINITY_DN14046_c0_g1_i1	Glyma.08G054300.1	97.68786127	154	0	437	2192	1776	21	0	2390

TRINITY_DN14046_c0_g1_i1	Glyma.08G054300.2	97.68786127	154	0	437	2192	1776	21	0	2390

TRINITY_DN2640_c0_g1_i1	Glyma.10G255100.1	96.19047619	145	8	222	1973	1749	1	0	2368

TRINITY_DN2640_c0_g2_i1	Glyma.10G255100.1	96.20253165	145	8	222	1973	1749	1	0	2368

TRINITY_DN14046_c0_g1_i1	Glyma.05G246300.1	100	144	0	437	2192	1764	9	0	2446

TRINITY_DN14573_c2_g1_i1	Glyma.08G014500.1	96.37681159	142	14	234	1986	1836	102	0	2302

TRINITY_DN2640_c0_g1_i1	Glyma.20G136200.1	96.21212121	139	8	222	1973	1749	1	0	2401

TRINITY_DN2640_c0_g2_i1	Glyma.20G136200.1	96.20437956	139	8	222	1973	1749	1	0	2401

TRINITY_DN9449_c0_g1_i1	Glyma.04G236100.4	99.375	137	6	532	2283	1	1752	0	2433

TRINITY_DN9449_c0_g1_i1	Glyma.04G236100.1	99.37694704	137	6	532	2283	1	1752	0	2433

TRINITY_DN9449_c0_g1_i1	Glyma.04G236100.2	100	137	6	532	2283	1	1752	0	2433

TRINITY_DN9449_c0_g1_i1	Glyma.04G236100.3	97.56097561	137	6	532	2283	1	1752	0	2433

TRINITY_DN9449_c0_g1_i1	Glyma.04G236100.5	96.73469388	137	6	532	2283	1	1752	0	2433

TRINITY_DN143_c0_g2_i1	Glyma.05G193900.1	98.88888889	114	2	627	2381	2121	367	0	2599

TRINITY_DN143_c0_g2_i1	Glyma.08G001900.1	98.88888889	101	2	627	2381	2751	997	0	2671

TRINITY_DN13566_c0_g1_i2	Glyma.14G047800.1	96.19047619	356	40	26	1747	1799	67	0	913

TRINITY_DN13979_c0_g2_i1	Glyma.10G183900.1	97.07317073	307	9	74	1823	5	1754	0	1476

TRINITY_DN14989_c0_g1_i1	Glyma.15G090200.1	95.93023256	288	16	88	1832	68	1812	0	1517

TRINITY_DN6769_c0_g1_i2	Glyma.09G054800.1	98.87640449	282	14	252	2002	71	1806	0	1530

TRINITY_DN6769_c0_g1_i2	Glyma.09G054800.3	98.88888889	282	14	252	2002	2	1737	0	1530

TRINITY_DN12717_c0_g1_i2	Glyma.10G159000.2	97.82608696	222	21	3	1748	163	1869	0	1642

TRINITY_DN12717_c0_g1_i2	Glyma.10G159000.1	97.82608696	222	21	3	1748	163	1869	0	1642

TRINITY_DN14728_c0_g2_i1	Glyma.17G033100.1	100	209	1	109	1860	1	1755	0	2063

TRINITY_DN8943_c0_g1_i2	Glyma.20G089300.5	100	205	4	1859	3611	1753	1	0	2080

TRINITY_DN14728_c0_g2_i1	Glyma.07G240300.1	100	201	3	109	1859	1	1754	0	2095

TRINITY_DN13175_c0_g1_i1	Glyma.08G176900.1	97.89473684	177	14	423	2158	1742	1	0	2054

TRINITY_DN16132_c0_g1_i5	Glyma.06G056400.1	96	166	4	1175	2928	436	2186	0	2289

TRINITY_DN16132_c0_g1_i5	Glyma.06G056400.4	96	166	4	1175	2928	436	2186	0	2289

TRINITY_DN16132_c0_g1_i5	Glyma.06G056400.3	96	166	5	1175	2928	436	2180	0	2250

TRINITY_DN39666_c0_g1_i1	Glyma.10G185400.1	100	157	17	1128	2874	1720	1	0	2093

TRINITY_DN6544_c0_g1_i1	Glyma.01G239700.1	98.47715736	152	13	42	1793	1	1737	0	2263

TRINITY_DN16137_c0_g1_i11	Glyma.14G144100.1	97.8021978	140	11	127	1857	7	1761	0	2311

TRINITY_DN14413_c0_g1_i2	Glyma.05G136200.1	96.40522876	131	0	294	2048	1	1755	0	2516

TRINITY_DN14413_c0_g1_i2	Glyma.08G091600.1	98.07692308	130	0	294	2048	1	1755	0	2521

TRINITY_DN11990_c0_g1_i1	Glyma.19G006000.2	97.71689498	130	5	220	1970	1	1751	0	2470

TRINITY_DN11990_c0_g1_i1	Glyma.19G006000.3	97.32142857	130	5	220	1970	1	1751	0	2470

TRINITY_DN15405_c0_g1_i5	Glyma.06G323700.1	97.97979798	114	10	2056	3795	1746	1	0	2455

TRINITY_DN11643_c0_g1_i1	Glyma.05G056100.1	95.12195122	110	0	176	1930	38	1792	0	2632

TRINITY_DN11643_c0_g1_i2	Glyma.05G056100.1	98.55072464	110	0	226	1980	38	1792	0	2632

TRINITY_DN11643_c0_g1_i3	Glyma.05G056100.1	100	110	0	176	1930	38	1792	0	2632

TRINITY_DN11643_c0_g1_i4	Glyma.05G056100.1	98.21958457	110	0	226	1980	38	1792	0	2632

TRINITY_DN8905_c0_g1_i1	Glyma.17G259200.2	100	108	2	117	1865	13	1767	0	2604

TRINITY_DN8905_c0_g1_i2	Glyma.17G259200.2	100	108	2	117	1865	13	1767	0	2604

TRINITY_DN8905_c0_g1_i3	Glyma.17G259200.2	97.81021898	108	2	117	1865	13	1767	0	2604

TRINITY_DN14637_c0_g2_i1	Glyma.10G016000.1	98.13084112	102	2	1	1740	507	2261	0	2579

TRINITY_DN37412_c0_g1_i1	Glyma.03G109000.1	95.08196721	97	1	224	1975	1755	1	0	2684

TRINITY_DN15285_c1_g1_i1	Glyma.04G213800.2	96.66666667	97	5	68	1821	44	1785	0	2614

TRINITY_DN15285_c1_g1_i1	Glyma.04G213800.1	96.7032967	97	5	68	1821	44	1785	0	2614

TRINITY_DN13991_c0_g1_i2	Glyma.09G070900.2	95.08196721	327	11	815	2547	1754	1	0	1291

TRINITY_DN13979_c0_g2_i1	Glyma.20G206300.1	96.69421488	298	7	74	1823	5	1754	0	1537

TRINITY_DN14389_c0_g1_i4	Glyma.11G223900.1	95.89041096	277	22	97	1831	1	1723	0	1382

TRINITY_DN15815_c0_g2_i2	Glyma.12G219400.4	100	261	8	1859	3607	2141	393	0	1729

TRINITY_DN15815_c0_g2_i2	Glyma.12G219400.1	95.69160998	261	8	1859	3607	2141	393	0	1729

TRINITY_DN15815_c0_g2_i4	Glyma.12G219400.4	100	261	8	2420	4168	2141	393	0	1729

TRINITY_DN15815_c0_g2_i4	Glyma.12G219400.1	100	261	8	2420	4168	2141	393	0	1729

TRINITY_DN14503_c0_g1_i1	Glyma.20G244000.1	96.42857143	198	12	376	2127	2010	277	0	2001

TRINITY_DN14503_c0_g1_i1	Glyma.10G294200.1	96.42857143	193	12	376	2127	2019	286	0	2028

TRINITY_DN14090_c0_g1_i2	Glyma.20G111900.1	98.88888889	179	4	153	1902	15	1764	0	2196

TRINITY_DN12593_c0_g3_i3	Glyma.08G175900.1	100	131	1	88	1841	32	1779	0	2475

TRINITY_DN12479_c0_g1_i3	Glyma.05G041000.1	96.36363636	126	8	271	2021	1790	55	0	2405

TRINITY_DN15815_c0_g2_i2	Glyma.13G281900.1	96.03960396	257	6	1859	3607	2141	393	0	1762

TRINITY_DN15815_c0_g2_i4	Glyma.13G281900.1	95.56313993	257	6	2420	4168	2141	393	0	1762

TRINITY_DN16084_c0_g1_i1	Glyma.18G114800.1	100	253	12	882	2616	1	1743	0	1655

TRINITY_DN16084_c0_g1_i2	Glyma.18G114800.1	99.38650307	252	12	255	1989	1	1743	0	1661

TRINITY_DN16084_c0_g1_i3	Glyma.18G114800.1	98.07692308	252	12	267	2001	1	1743	0	1661

TRINITY_DN15042_c0_g2_i1	Glyma.08G193100.2	96.23655914	232	6	118	1867	27	1776	0	1914

TRINITY_DN15042_c0_g2_i1	Glyma.08G193100.1	99	232	6	118	1867	27	1776	0	1914

TRINITY_DN14503_c0_g1_i2	Glyma.18G300100.1	95.83843329	199	10	376	2109	2019	268	0	2006

TRINITY_DN16084_c0_g1_i7	Glyma.18G114800.1	100	194	12	267	2001	1	1743	0	1982

TRINITY_DN15135_c0_g1_i2	Glyma.17G104500.2	96.12903226	162	13	433	2172	1884	145	0	2172

TRINITY_DN13991_c0_g1_i1	Glyma.09G070900.2	100	161	2	676	2424	1752	1	0	2313

TRINITY_DN13991_c0_g1_i3	Glyma.09G070900.2	99.4011976	158	12	676	2411	1752	1	0	2246

TRINITY_DN16433_c0_g2_i2	Glyma.18G036300.1	98.73417722	153	3	145	1894	1	1753	0	2370

TRINITY_DN14909_c0_g1_i2	Glyma.10G021500.2	95.92641261	147	0	134	1886	6	1758	0	2423

TRINITY_DN14909_c0_g1_i2	Glyma.10G021500.1	95.92145015	147	0	134	1886	6	1758	0	2423

TRINITY_DN14909_c0_g1_i3	Glyma.10G021500.2	95.93301435	147	0	134	1886	6	1758	0	2423

TRINITY_DN14909_c0_g1_i3	Glyma.10G021500.1	95.93023256	147	0	134	1886	6	1758	0	2423

TRINITY_DN14909_c0_g1_i2	Glyma.02G152800.2	95.91836735	146	0	134	1886	6	1758	0	2429

TRINITY_DN14909_c0_g1_i2	Glyma.02G152800.1	95.91836735	146	0	134	1886	6	1758	0	2429

TRINITY_DN14909_c0_g1_i3	Glyma.02G152800.2	95.93023256	146	0	134	1886	6	1758	0	2429

TRINITY_DN14909_c0_g1_i3	Glyma.02G152800.1	95.93023256	146	0	134	1886	6	1758	0	2429

TRINITY_DN9983_c0_g1_i1	Glyma.04G183400.1	98.33333333	141	1	404	2156	2181	432	0	2436

TRINITY_DN4713_c0_g1_i1	Glyma.17G115300.2	98.68421053	121	3	77	1817	3	1755	0	2490

TRINITY_DN6008_c0_g1_i1	Glyma.07G263400.1	97.15302491	110	14	65	1763	1	1752	0	2272

TRINITY_DN16005_c1_g2_i1	Glyma.02G033300.1	98.46153846	76	1	217	1966	1	1753	0	2796

TRINITY_DN11586_c0_g2_i1	Glyma.11G227300.1	96.15384615	287	13	255	2000	1748	6	0	1548

TRINITY_DN16084_c0_g1_i1	Glyma.08G304300.1	98.5915493	245	9	882	2616	1	1746	0	1729

TRINITY_DN16084_c0_g1_i2	Glyma.08G304300.1	99.38650307	244	9	255	1989	1	1746	0	1735

TRINITY_DN16084_c0_g1_i3	Glyma.08G304300.1	100	244	9	267	2001	1	1746	0	1735

TRINITY_DN14503_c0_g1_i2	Glyma.08G361700.1	95.83858764	201	13	378	2109	2017	268	0	1980

TRINITY_DN16084_c0_g1_i7	Glyma.08G304300.1	99.05660377	192	9	267	2001	1	1746	0	2023

TRINITY_DN11757_c0_g1_i1	Glyma.19G080400.1	95.4954955	187	4	301	2049	1743	1	0	2122

TRINITY_DN11757_c0_g1_i1	Glyma.16G066400.1	95.92391304	175	4	301	2049	1743	1	0	2189

TRINITY_DN11757_c0_g1_i1	Glyma.16G066400.2	100	172	8	301	2049	1731	1	0	2128

TRINITY_DN16032_c0_g1_i1	Glyma.07G239700.2	98.92473118	159	0	287	2038	1	1752	0	2355

TRINITY_DN16032_c0_g1_i3	Glyma.07G239700.2	100	159	0	236	1987	1	1752	0	2355

TRINITY_DN15403_c0_g4_i1	Glyma.14G018300.3	97.10144928	150	5	165	1904	1	1749	0	2307

TRINITY_DN15403_c0_g4_i1	Glyma.14G018300.2	97.10144928	150	5	165	1904	1	1749	0	2307

TRINITY_DN16326_c0_g1_i1	Glyma.15G265400.1	96.96969697	150	8	563	2313	1734	1	0	2281

TRINITY_DN16326_c0_g1_i1	Glyma.15G265400.2	96.96969697	148	11	563	2313	1728	1	0	2254

TRINITY_DN6579_c0_g1_i1	Glyma.12G089000.1	100	145	1	129	1877	19	1770	0	2412

TRINITY_DN15403_c0_g4_i1	Glyma.02G295100.2	100	142	4	165	1904	1	1749	0	2351

TRINITY_DN15403_c0_g4_i1	Glyma.02G295100.1	99.20634921	142	4	165	1904	1	1749	0	2351

TRINITY_DN6544_c0_g1_i1	Glyma.11G004000.1	98.48484848	142	11	42	1793	1	1731	0	2313

TRINITY_DN14204_c0_g1_i4	Glyma.01G175600.3	100	135	0	145	1896	1	1752	0	2488

TRINITY_DN16032_c0_g1_i4	Glyma.07G239700.2	97.34513274	135	0	338	2089	1	1752	0	2488

TRINITY_DN9566_c0_g1_i2	Glyma.11G103200.2	100	134	0	241	1992	1	1752	0	2494

TRINITY_DN9566_c0_g1_i2	Glyma.11G103200.1	100	134	0	241	1992	1	1752	0	2494

TRINITY_DN11025_c0_g1_i1	Glyma.08G125700.2	96.8503937	129	5	1	1746	261	2006	0	2444

TRINITY_DN5614_c0_g1_i1	Glyma.12G222700.1	96.19565217	119	2	113	1861	1	1749	0	2538

TRINITY_DN14280_c0_g2_i2	Glyma.17G176400.3	97.83163265	117	6	269	2013	1757	13	0	2497

TRINITY_DN38533_c0_g1_i1	Glyma.14G007100.1	97.81021898	111	4	446	2192	1	1750	0	2575

TRINITY_DN38533_c0_g1_i1	Glyma.14G007100.2	97.81021898	111	4	446	2192	1	1750	0	2575

TRINITY_DN16032_c0_g1_i2	Glyma.07G239700.2	100	107	0	287	2038	1	1752	0	2643

TRINITY_DN11_c0_g1_i1	Glyma.08G063600.1	95.58823529	107	2	229	1977	1749	1	0	2604

TRINITY_DN14204_c0_g1_i3	Glyma.01G175600.3	100	102	0	145	1896	1	1752	0	2671

TRINITY_DN5259_c0_g2_i1	Glyma.03G090000.3	96.96969697	94	1	183	1934	17	1767	0	2708

TRINITY_DN15403_c0_g1_i1	Glyma.14G018300.3	95.0617284	94	3	165	1916	1	1749	0	2695

TRINITY_DN15403_c0_g1_i1	Glyma.14G018300.2	96.1038961	94	3	165	1916	1	1749	0	2695

TRINITY_DN14961_c1_g1_i2	Glyma.08G266700.4	100	90	2	1137	2888	1735	2	0	2621

TRINITY_DN14961_c1_g1_i2	Glyma.08G266700.5	100	90	2	1137	2888	1735	2	0	2621

TRINITY_DN15403_c0_g1_i1	Glyma.02G295100.2	95.26066351	90	2	165	1916	1	1749	0	2717

TRINITY_DN15403_c0_g1_i1	Glyma.02G295100.1	98.39034205	90	2	165	1916	1	1749	0	2717

TRINITY_DN16032_c0_g1_i5	Glyma.07G239700.2	100	83	0	338	2089	1	1752	0	2776

TRINITY_DN16032_c0_g1_i7	Glyma.07G239700.2	100	83	0	185	1936	1	1752	0	2776

TRINITY_DN16345_c0_g1_i4	Glyma.07G093700.2	98.4375	80	1	642	2393	3527	1779	0	2772

TRINITY_DN12830_c0_g1_i2	Glyma.13G043000.1	98.75776398	420	45	1	1731	238	1960	2.88E-169	597

TRINITY_DN13134_c0_g1_i2	Glyma.16G026900.2	95.33898305	350	4	330	2078	1749	1	0	1269

TRINITY_DN13134_c0_g1_i2	Glyma.16G026900.3	95.33898305	350	4	330	2078	1749	1	0	1269

TRINITY_DN13134_c0_g1_i2	Glyma.16G026900.1	95.32163743	350	4	330	2078	1749	1	0	1269

TRINITY_DN13134_c0_g1_i1	Glyma.16G026900.2	95.3271028	342	11	330	2062	1749	1	0	1210

TRINITY_DN13134_c0_g1_i1	Glyma.16G026900.3	95.3271028	342	11	330	2062	1749	1	0	1210

TRINITY_DN13134_c0_g1_i1	Glyma.16G026900.1	95.3125	342	11	330	2062	1749	1	0	1210

TRINITY_DN10472_c0_g1_i3	Glyma.17G165000.1	100	280	9	299	2046	161	1905	0	1624

TRINITY_DN14047_c0_g1_i3	Glyma.02G228500.1	96.25	271	20	900	2639	544	2280	0	1570

TRINITY_DN14047_c0_g1_i3	Glyma.02G228500.2	99.28571429	271	20	900	2639	22	1758	0	1570

TRINITY_DN16187_c1_g2_i2	Glyma.07G107100.1	97.32142857	225	5	107	1854	1	1748	0	1949

TRINITY_DN2753_c0_g1_i1	Glyma.03G137400.1	96.82539683	169	14	2	1727	1314	3060	0	2109

TRINITY_DN5938_c0_g1_i1	Glyma.05G080900.1	100	169	28	241	1965	178	1905	0	1980

TRINITY_DN15821_c0_g2_i1	Glyma.10G280000.1	97.02048417	169	60	4	1673	1697	1	0	1424

TRINITY_DN5938_c0_g1_i1	Glyma.05G080900.2	100	168	34	241	1965	178	1896	0	1929

TRINITY_DN11440_c0_g7_i1	Glyma.07G187300.2	99.39393939	167	12	230	1971	1735	6	0	2115

TRINITY_DN22418_c0_g1_i1	Glyma.14G079200.1	97.61904762	155	3	201	1936	1	1748	0	2259

TRINITY_DN3207_c0_g1_i1	Glyma.04G062500.1	99.43181818	149	20	1	1705	219	1968	0	2104

TRINITY_DN4009_c0_g1_i1	Glyma.15G213200.1	96.77419355	138	7	70	1816	1	1720	0	2242

TRINITY_DN13709_c0_g1_i2	Glyma.01G019100.2	98.03921569	133	1	485	2235	1750	1	0	2490

TRINITY_DN3268_c0_g1_i1	Glyma.06G016400.2	100	123	3	329	2079	1749	2	0	2532

TRINITY_DN3268_c0_g1_i1	Glyma.06G016400.1	100	123	3	329	2079	1749	2	0	2532

TRINITY_DN14047_c0_g1_i3	Glyma.11G169900.2	95.84158416	105	1	892	2639	536	2286	0	2632

TRINITY_DN14047_c0_g1_i3	Glyma.11G169900.1	100	105	1	892	2639	536	2286	0	2632

TRINITY_DN14047_c0_g1_i3	Glyma.11G169900.6	100	105	2	892	2639	536	2274	0	2555

TRINITY_DN14344_c0_g1_i1	Glyma.20G202200.3	100	103	5	431	2171	1750	1	0	2591

TRINITY_DN14344_c0_g1_i1	Glyma.20G202200.4	96.36363636	103	5	431	2171	1750	1	0	2591

TRINITY_DN14344_c0_g1_i1	Glyma.20G202200.1	97.87685775	103	5	431	2171	1750	1	0	2591

TRINITY_DN14344_c0_g1_i1	Glyma.20G202200.2	100	103	5	431	2171	1750	1	0	2591

TRINITY_DN14344_c0_g1_i1	Glyma.20G202200.5	99.45355191	103	5	431	2171	1750	1	0	2591

TRINITY_DN14344_c0_g1_i6	Glyma.20G202200.3	98.4375	103	5	431	2171	1750	1	0	2591

TRINITY_DN14344_c0_g1_i6	Glyma.20G202200.4	95.64393939	103	5	431	2171	1750	1	0	2591

TRINITY_DN14344_c0_g1_i6	Glyma.20G202200.1	95.64428312	103	5	431	2171	1750	1	0	2591

TRINITY_DN14344_c0_g1_i6	Glyma.20G202200.2	95.64428312	103	5	431	2171	1750	1	0	2591

TRINITY_DN14344_c0_g1_i6	Glyma.20G202200.5	95.64502876	103	5	431	2171	1750	1	0	2591

TRINITY_DN13991_c0_g1_i1	Glyma.13G111500.4	98.71794872	344	11	678	2424	1738	1	0	1229

TRINITY_DN13991_c0_g1_i3	Glyma.13G111500.4	99.61832061	339	18	678	2411	1738	1	0	1173

TRINITY_DN13544_c0_g1_i3	Glyma.01G062400.1	98.96907216	268	21	24	1764	1818	84	0	1592

TRINITY_DN16396_c2_g1_i3	Glyma.11G138300.1	97.24409449	254	20	516	2250	169	1894	0	1572

TRINITY_DN15795_c0_g1_i1	Glyma.19G109500.2	99.25187032	237	27	1125	2844	1699	1	0	1395

TRINITY_DN15795_c0_g1_i3	Glyma.19G109500.2	96.08695652	237	27	1299	3018	1699	1	0	1395

TRINITY_DN15795_c0_g1_i5	Glyma.19G109500.2	98.5915493	237	27	1165	2884	1699	1	0	1395

TRINITY_DN9901_c0_g1_i1	Glyma.02G034500.1	97.24770642	221	30	263	1981	1722	1	0	1626

TRINITY_DN5817_c0_g1_i1	Glyma.17G240500.1	100	203	30	440	2149	1733	24	0	1591

TRINITY_DN5817_c0_g1_i1	Glyma.17G240500.2	98.48484848	203	30	440	2149	1733	24	0	1591

TRINITY_DN9977_c0_g2_i1	Glyma.07G141900.4	100	166	0	419	2168	2823	1074	0	2313

TRINITY_DN9977_c0_g2_i1	Glyma.07G141900.3	100	166	0	419	2168	2823	1074	0	2313

TRINITY_DN9977_c0_g2_i1	Glyma.07G141900.2	100	166	0	419	2168	2823	1074	0	2313

TRINITY_DN9977_c0_g2_i1	Glyma.07G141900.1	100	166	0	419	2168	2823	1074	0	2313

TRINITY_DN15042_c0_g2_i3	Glyma.08G193100.2	98.13829787	157	0	118	1867	27	1776	0	2362

TRINITY_DN15042_c0_g2_i3	Glyma.08G193100.1	98.13907933	157	0	118	1867	27	1776	0	2362

TRINITY_DN14540_c0_g1_i1	Glyma.19G067400.1	99.36708861	157	10	688	2419	1735	1	0	2148

TRINITY_DN14765_c0_g2_i1	Glyma.08G005200.1	100	157	39	48	1764	1	1713	0	1910

TRINITY_DN16130_c2_g6_i1	Glyma.19G196000.2	96.49122807	153	1	337	2085	456	2205	0	2377

TRINITY_DN16130_c2_g6_i11	Glyma.19G196000.2	98.18181818	153	1	664	2412	456	2205	0	2377

TRINITY_DN14730_c0_g1_i1	Glyma.17G037400.2	98.07692308	144	4	1230	2971	1749	1	0	2375

TRINITY_DN14730_c0_g1_i5	Glyma.17G037400.2	100	144	4	871	2612	1749	1	0	2375

TRINITY_DN6660_c0_g2_i1	Glyma.20G099900.1	95.63758389	141	2	672	2418	420	2169	0	2431

TRINITY_DN14238_c0_g2_i2	Glyma.07G189200.1	97.61904762	128	0	196	1945	1	1750	0	2523

TRINITY_DN14238_c0_g2_i2	Glyma.08G060100.3	97.61904762	126	0	196	1945	1	1750	0	2534

TRINITY_DN14238_c0_g2_i2	Glyma.08G060100.2	97.61904762	126	0	196	1945	1	1750	0	2534

TRINITY_DN9202_c0_g1_i1	Glyma.19G006100.2	97.01492537	121	19	215	1948	14	1741	0	2316

TRINITY_DN9202_c0_g1_i1	Glyma.19G006100.1	97.82608696	121	19	215	1948	14	1741	0	2316

TRINITY_DN12226_c0_g2_i1	Glyma.13G053100.3	98.93048128	115	1	513	2262	1749	1	0	2588

TRINITY_DN12226_c0_g2_i1	Glyma.13G053100.1	98.93617021	115	1	513	2262	1749	1	0	2588

TRINITY_DN13137_c0_g1_i1	Glyma.04G074500.1	96.68246445	108	9	3	1739	23	1753	0	2427

TRINITY_DN16130_c2_g6_i12	Glyma.19G196000.2	96.23188406	105	1	664	2412	456	2205	0	2643

TRINITY_DN16130_c2_g6_i13	Glyma.19G196000.2	98	105	1	664	2412	456	2205	0	2643

TRINITY_DN16130_c2_g6_i2	Glyma.19G196000.2	99.72899729	105	1	337	2085	456	2205	0	2643

TRINITY_DN16130_c2_g6_i3	Glyma.19G196000.2	99.05660377	105	1	337	2085	456	2205	0	2643

TRINITY_DN16005_c1_g2_i1	Glyma.01G032100.1	95.4312115	76	0	217	1966	1	1750	0	2811

TRINITY_DN16005_c1_g2_i1	Glyma.02G033300.2	95.43147208	76	0	217	1966	1	1750	0	2811

TRINITY_DN15778_c0_g1_i1	Glyma.03G152000.2	98.75	405	55	1055	2776	1845	136	6.17E-158	560

TRINITY_DN15778_c0_g1_i1	Glyma.03G152000.4	98.75	405	55	1055	2776	1845	136	6.17E-158	560

TRINITY_DN15710_c0_g1_i1	Glyma.15G042900.1	95.45454545	277	22	477	2212	2210	481	0	1489

TRINITY_DN16056_c0_g2_i1	Glyma.14G012400.2	100	272	6	511	2253	1746	1	0	1664

TRINITY_DN15353_c0_g1_i1	Glyma.03G008800.1	98.93617021	272	17	292	2021	2637	896	0	1555

TRINITY_DN15353_c0_g1_i2	Glyma.03G008800.1	98.7654321	272	17	292	2021	2637	896	0	1555

TRINITY_DN16396_c2_g1_i5	Glyma.11G138300.1	97.25685786	258	19	516	2250	169	1894	0	1561

TRINITY_DN16187_c1_g2_i2	Glyma.09G170600.2	96.68508287	234	2	107	1854	1	1748	0	1921

TRINITY_DN18177_c0_g1_i1	Glyma.17G245500.1	98.33333333	225	10	101	1838	1	1744	0	1881

TRINITY_DN16005_c1_g2_i1	Glyma.08G289100.1	95.50561798	177	0	217	1965	1	1749	0	2250

TRINITY_DN16098_c0_g1_i1	Glyma.05G168500.1	100	165	11	459	2198	1773	28	0	2239

TRINITY_DN13134_c0_g1_i2	Glyma.19G254000.1	95.32258065	145	0	330	2078	1749	1	0	2427

TRINITY_DN13134_c0_g1_i1	Glyma.19G254000.1	96.62447257	144	4	330	2062	1749	1	0	2329

TRINITY_DN14057_c0_g1_i1	Glyma.07G212300.1	100	143	19	197	1935	101	1824	0	2211

TRINITY_DN16360_c0_g4_i8	Glyma.13G350700.1	100	137	0	234	1982	22	1770	0	2471

TRINITY_DN10656_c0_g1_i1	Glyma.10G192200.1	100	137	9	121	1857	1	1743	0	2355

TRINITY_DN37204_c0_g1_i1	Glyma.04G036300.3	97.77777778	136	15	120	1837	34	1772	0	2211

TRINITY_DN16379_c0_g2_i3	Glyma.10G057100.1	98.55072464	128	0	315	2063	1773	25	0	2521

TRINITY_DN5614_c0_g1_i1	Glyma.13G278800.1	96.19651347	128	1	113	1861	1	1746	0	2501

TRINITY_DN2125_c0_g1_i1	Glyma.20G153600.2	95.23809524	125	1	74	1815	1	1749	0	2492

TRINITY_DN16216_c0_g3_i2	Glyma.20G241900.4	95.86206897	125	7	515	2257	1	1734	0	2401

TRINITY_DN16216_c0_g3_i2	Glyma.20G241900.2	98.57142857	125	7	515	2257	1	1734	0	2401

TRINITY_DN16216_c0_g3_i2	Glyma.20G241900.3	100	125	7	515	2257	1	1734	0	2401

TRINITY_DN12226_c0_g2_i1	Glyma.13G053100.2	98.93617021	116	0	514	2262	1749	1	0	2588

TRINITY_DN12226_c0_g2_i3	Glyma.13G053100.2	98.92473118	116	1	658	2405	1749	1	0	2580

TRINITY_DN6303_c0_g1_i1	Glyma.02G094400.2	98.95833333	115	4	191	1933	1746	1	0	2534

TRINITY_DN13395_c0_g1_i1	Glyma.08G044100.1	98.63481229	114	0	92	1840	1	1749	0	2599

TRINITY_DN13395_c0_g1_i4	Glyma.08G044100.1	95.37572254	114	0	82	1830	1	1749	0	2599

TRINITY_DN15764_c0_g1_i8	Glyma.09G135100.1	95.51020408	114	6	1	1742	3	1744	0	2508

TRINITY_DN38533_c0_g1_i1	Glyma.02G305900.2	97.8021978	109	3	446	2192	1	1747	0	2601

TRINITY_DN38533_c0_g1_i1	Glyma.02G305900.3	97.80487805	109	3	446	2192	1	1747	0	2601

TRINITY_DN38533_c0_g1_i1	Glyma.02G305900.1	97.80775717	109	3	446	2192	1	1747	0	2601

TRINITY_DN13395_c0_g1_i1	Glyma.05G237000.2	97.6744186	106	0	92	1840	1	1749	0	2643

TRINITY_DN13395_c0_g1_i1	Glyma.05G237000.1	96.91358025	106	0	92	1840	1	1749	0	2643

TRINITY_DN13395_c0_g1_i4	Glyma.05G237000.2	99.40828402	106	0	82	1830	1	1749	0	2643

TRINITY_DN13395_c0_g1_i4	Glyma.05G237000.1	95.37572254	106	0	82	1830	1	1749	0	2643

TRINITY_DN6303_c0_g1_i1	Glyma.18G287300.1	95	106	2	191	1933	1749	1	0	2604

TRINITY_DN16159_c0_g1_i4	Glyma.02G228100.1	98.91304348	103	3	180	1925	1	1749	0	2639

TRINITY_DN11_c0_g1_i1	Glyma.07G185600.1	100	102	0	229	1977	1749	1	0	2665

TRINITY_DN15634_c2_g1_i2	Glyma.02G159900.1	99.60629921	316	24	359	2090	4321	2590	0	1271

TRINITY_DN15634_c2_g1_i4	Glyma.02G159900.1	100	316	24	359	2090	4321	2590	0	1271

TRINITY_DN12035_c0_g1_i1	Glyma.20G237800.1	98.97260274	284	11	268	2006	1739	1	0	1539

TRINITY_DN12035_c0_g1_i1	Glyma.20G237800.2	98.97260274	284	11	268	2006	1739	1	0	1539

TRINITY_DN12035_c0_g1_i2	Glyma.20G237800.1	98.93617021	284	11	268	2006	1739	1	0	1539

TRINITY_DN12035_c0_g1_i2	Glyma.20G237800.2	100	284	11	268	2006	1739	1	0	1539

TRINITY_DN11672_c0_g1_i1	Glyma.02G060500.1	100	211	5	10	1753	2327	4070	0	2008

TRINITY_DN11672_c0_g1_i1	Glyma.02G060500.8	96.55172414	211	5	10	1753	2327	4070	0	2008

TRINITY_DN11672_c0_g1_i1	Glyma.02G060500.7	97.43589744	211	5	10	1753	2327	4070	0	2008

TRINITY_DN11672_c0_g1_i1	Glyma.02G060500.5	97.92843691	211	5	10	1753	2327	4070	0	2008

TRINITY_DN11672_c0_g1_i1	Glyma.02G060500.6	100	211	5	10	1753	2327	4070	0	2008

TRINITY_DN11672_c0_g1_i1	Glyma.02G060500.2	95.78947368	211	5	10	1753	2327	4070	0	2008

TRINITY_DN11672_c0_g1_i1	Glyma.02G060500.3	100	211	5	10	1753	2327	4070	0	2008

TRINITY_DN11672_c0_g1_i1	Glyma.02G060500.4	96.8	211	5	10	1753	2327	4070	0	2008

TRINITY_DN16415_c1_g1_i2	Glyma.U018900.1	97.84946237	156	5	2621	4368	1755	17	0	2305

TRINITY_DN16415_c1_g1_i9	Glyma.U018900.1	96.57142857	156	5	2527	4274	1755	17	0	2305

TRINITY_DN12017_c0_g1_i2	Glyma.11G228500.1	98.11320755	150	14	327	2057	1744	17	0	2158

TRINITY_DN16135_c0_g1_i2	Glyma.01G051400.1	100	144	16	1468	3204	3115	1400	0	2152

TRINITY_DN16067_c2_g1_i1	Glyma.17G025200.2	100	142	2	321	2067	1	1747	0	2429

TRINITY_DN16067_c2_g1_i2	Glyma.17G025200.2	97.94520548	142	2	321	2067	1	1747	0	2429

TRINITY_DN4106_c0_g1_i1	Glyma.11G232900.2	98.8372093	130	0	69	1816	1	1748	0	2508
[truncated: 43,775,236 more chars]
